# Single-cell RNA sequencing and spatial transcriptomics reveal cancer-associated fibroblasts in glioblastoma with protumoral effects

**DOI:** 10.1172/JCI147087

**Published:** 2023-03-01

**Authors:** Saket Jain, Jonathan W. Rick, Rushikesh S. Joshi, Angad Beniwal, Jordan Spatz, Sabraj Gill, Alexander Chih-Chieh Chang, Nikita Choudhary, Alan T. Nguyen, Sweta Sudhir, Eric J. Chalif, Jia-Shu Chen, Ankush Chandra, Alexander F. Haddad, Harsh Wadhwa, Sumedh S. Shah, Serah Choi, Josie L. Hayes, Lin Wang, Garima Yagnik, Joseph F. Costello, Aaron Diaz, Dieter Henrik Heiland, Manish K. Aghi

**Affiliations:** 1 Department of Neurosurgery, UCSF, San Francisco, California, USA.; 2 Department of Neurosurgery, University of Freiburg, Breisgau, Germany.

**Keywords:** Oncology, Brain cancer, Fibronectin

## Abstract

Cancer-associated fibroblasts (CAFs) were presumed absent in glioblastoma given the lack of brain fibroblasts. Serial trypsinization of glioblastoma specimens yielded cells with CAF morphology and single-cell transcriptomic profiles based on their lack of copy number variations (CNVs) and elevated individual cell CAF probability scores derived from the expression of 9 CAF markers and absence of 5 markers from non-CAF stromal cells sharing features with CAFs. Cells without CNVs and with high CAF probability scores were identified in single-cell RNA-Seq of 12 patient glioblastomas. Pseudotime reconstruction revealed that immature CAFs evolved into subtypes, with mature CAFs expressing actin alpha 2, smooth muscle (*ACTA2*). Spatial transcriptomics from 16 patient glioblastomas confirmed CAF proximity to mesenchymal glioblastoma stem cells (GSCs), endothelial cells, and M2 macrophages. CAFs were chemotactically attracted to GSCs, and CAFs enriched GSCs. We created a resource of inferred crosstalk by mapping expression of receptors to their cognate ligands, identifying PDGF and TGF-β as mediators of GSC effects on CAFs and osteopontin and HGF as mediators of CAF-induced GSC enrichment. CAFs induced M2 macrophage polarization by producing the extra domain A (EDA) fibronectin variant that binds macrophage TLR4. Supplementing GSC-derived xenografts with CAFs enhanced in vivo tumor growth. These findings are among the first to identify glioblastoma CAFs and their GSC interactions, making them an intriguing target.

## Graphical abstract

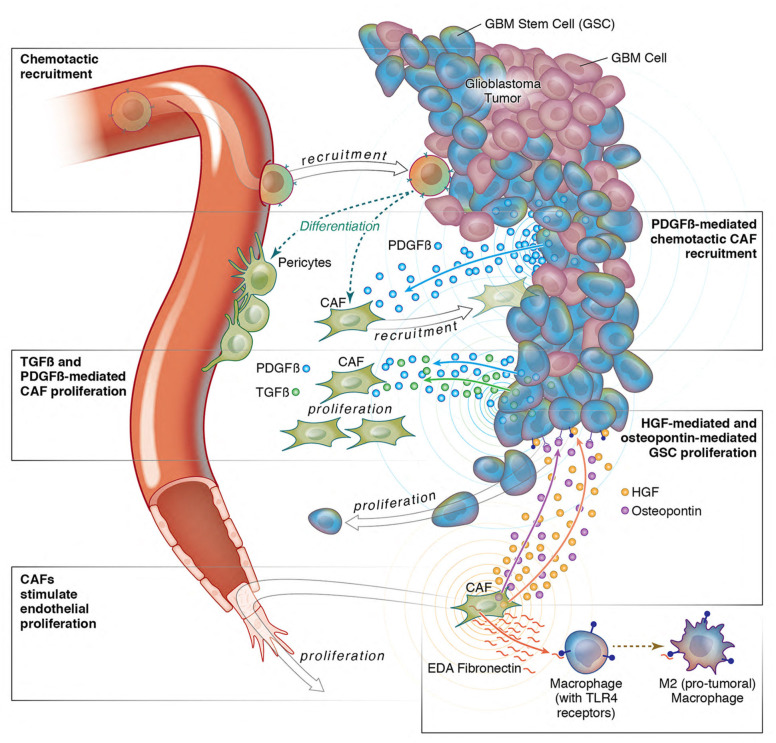

## Introduction

Glioblastoma (GBM) is an aggressive brain cancer with a poor prognosis (1). Current therapies have failed in large part because they treat GBM cells in isolation and fail to account for the understanding that GBM is an organ with complex interplay between tumor cells and their microenvironment (2). In terms of the cellular makeup of the GBM microenvironment, while numerous studies have investigated endothelial and immune cells (2), little attention has been paid to whether cancer-associated fibroblasts (CAFs), a cell type described as crucial in the stroma of carcinomas (3), exist in GBM. While many have presumed that GBMs lack CAFs based on the lack of fibroblasts in the central nervous system (4), some studies have identified cells expressing CAF markers in GBM (5–7). However, these studies fail to comprehensively profile these cells and their effects on GBM and its microenvironment. More importantly, the reliance of these studies on cell-surface markers without comprehensive gene expression profiling raises the possibility that the identified cells could be other cells in the microenvironment, such as pericytes, cells in capillary walls that share overlapping cell-surface markers with fibroblasts (8).

To address this knowledge gap, we used a serial trypsinization method described as isolating CAFs in other cancers (9) and analyzed the resulting cells transcriptomically to verify that they were CAFs based on their gene expression profile. We used single-cell trajectory analysis to define the lineage of these cells. We then identified these cells using single-cell RNA-Seq (scRNA-Seq) of patient GBM specimens. We determined the effects of these cells on GBM cells and the microenvironment in culture and in vivo.

## Results

### Identifying CAFs in GBM by serial trypsinization.

To determine whether a CAF-like population exists in GBM, we performed serial trypsinization (9) on dissociated newly diagnosed GBM patient samples for 5 weeks to remove less adherent tumor cells, resulting in retention of cells resistant to trypsinization that have been confirmed to be CAFs in other cancers (9). Within 5 weeks, cells emerged with the large spindle-shaped morphology that has been described for CAFs and fibroblasts (10).

**Figure 1:**
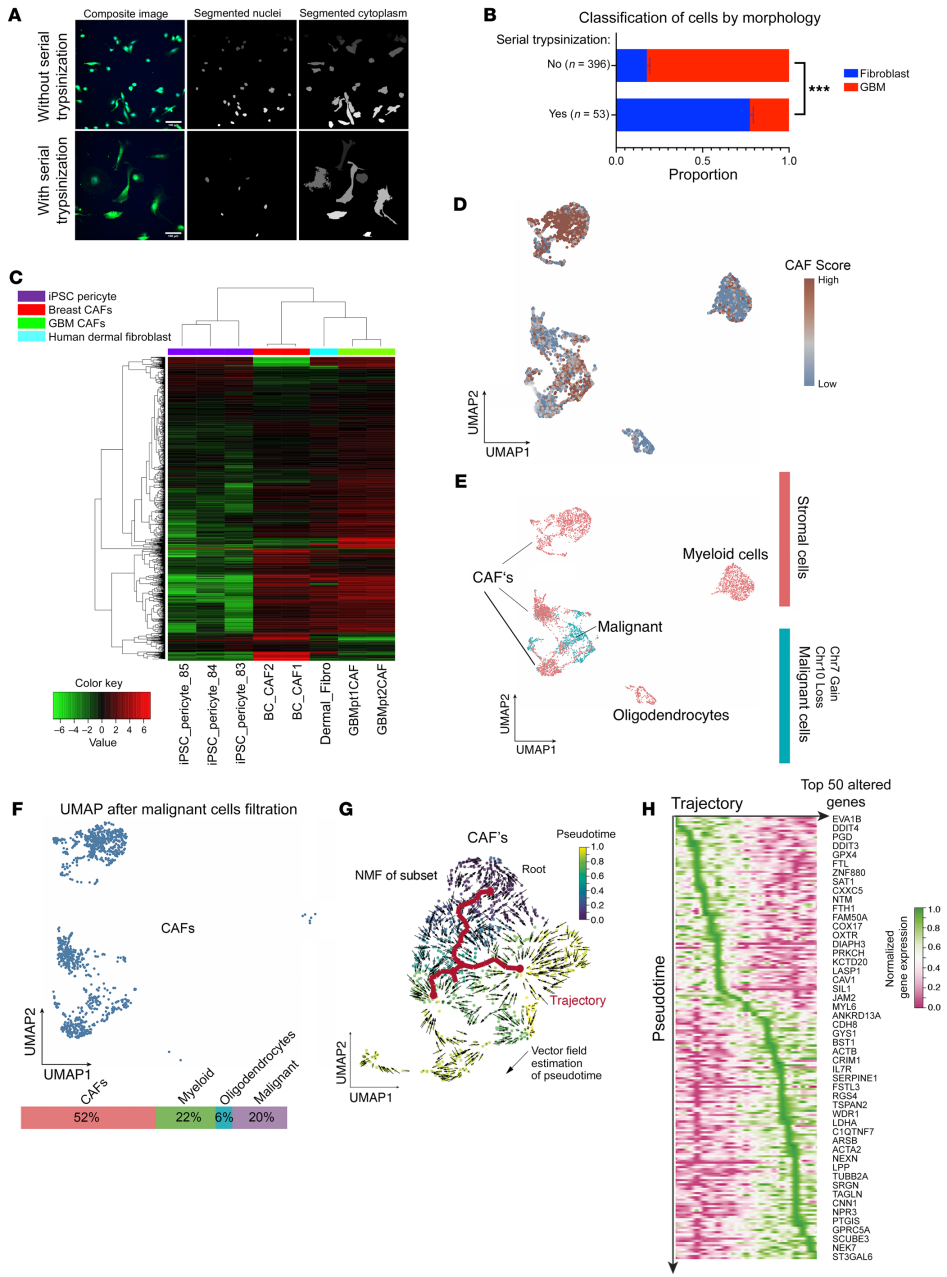
Identification of CAFs in GBM by serial trypsinization. (**A**) Segmented images of cells from patient GBM with or without serial trypsinization. Shown are GBMpt3CAF. Scale bars: 100 μm. (**B**) Using VAMPIRE analysis, we trained a machine-learning logistic regression classifier utilizing breast CAF data (366 1997T cells and 499 2124T cells) and GBM data from GBM6 (803 cells), GBM43 (350 cells), and U251 (685 cells). For testing, 159 cells with serial trypsinization and 1,187 cells without serial trypsinization were assessed, revealing that 77% of GBMpt3CAF cells from serial trypsinization of GBM exhibited fibroblast morphology, defined using 1997T and 2124T, compared with just 23% of these cells exhibiting GBM morphology, defined using GBM6, GBM43 and U251. In contrast, only 18% of cells from this patient not undergoing serial trypsinization had fibroblast morphology. *P* < 0.001, Fisher’s exact test. (**C**) Serially trypsinized cells from patient GBMs (GBMpt1CAF and GBMpt2CAF) exhibited transcriptomic profiles similar to those of breast CAFs and dermal fibroblasts but distinct from brain pericytes, as assessed by bulk RNA-Seq. Heatmap is based on log_2_ (fold change) and significant *P* adjusted values. (**D**–**G**) Results from scRNA-Seq of 4,385 serially trypsinized cells from GBMpt4CAF with UMAP showing (**D**) CAF probability scores based on CAF marker expression and stromal marker absences; (**E**) CNV revealing tumor cells with CNV alterations (cyan) and stromal cells without CNV alterations (red); and (**F**) cells deemed CAFs (navy) after tumor cells with CNV changes were removed from cells with high CAF probability scores. CAFs were in cluster containing mostly CAFs (top left) or cluster (lower left) associated with tumor cells but distinct from them based on CNV. (**G** and **H**) Pseudotime reconstruction of scRNA-Seq data using a minimum spanning tree (MST) approach revealed that early CAFs evolved into 2 subtypes (**G**), with heatmaps showing temporal gene expression during this process (**H**). ***P < 0.001.

We quantified the morphology of these cells by developing a modified visually aided morpho-phenotyping recognition (VAMPIRE) analysis (11) to classify and compare irregular cellular and nuclear shapes. By pairing nuclear and cytoplasm data sets by cell, we generated a 16–data point profile for each cell. We then designed a machine-learning logistic regression classifier utilizing data from 2 breast CAF lines (Supplemental Figure 1; supplemental material available online with this article; https://doi.org/10.1172/JCI147087DS1) and 3 GBM cell lines (Supplemental Figure 2) to achieve a nominal accuracy of 91% in distinguishing GBM cells from CAFs. Our classifier identified 77% of the cells from serial trypsinization of patient GBMs as exhibiting CAF morphology. In contrast, when patient GBM samples were cultured without serial trypsinization, the classifier found GBM cells predominated at 82%, reducing the population of cells with CAF morphology to 18% (*P* < 0.001; Figure 1, A and B), supporting our hypothesis that serial trypsinization created a CAF-enriched cell population. Expanding from a 2-class to a 3-class weighted regression (Supplemental Methods) by adding cultured human astrocyte data to the breast CAF and GBM cell line data produced similar results, with serial trypsinization leading to CAF enrichment (*P* < 0.001; Supplemental Figure 3).

We then performed bulk RNA-Seq to analyze the gene expression of these CAF-like cells we had identified in patient GBMs. Bulk RNA-Seq revealed that these CAF-like cells in GBM exhibited a transcriptomic profile (Supplemental Table 1) similar to that of breast CAFs (12), but different from that of pericytes (13), a cell type whose morphology and surface-marker expression overlap with CAFs (Figure 1C). Comparison of these CAF-like cells to normal fibroblasts from 8 tissues revealed that these cells most resembled dermal fibroblasts (14, 15) (Figure 1C and Supplemental Figure 4). Together, these findings support the hypothesis that these cells were GBM CAFs.

### scRNA-Seq of cells isolated from serial trypsinization of GBMs.

To rigorously assess the transcriptomic profile and purity of these cells isolated from patient GBM by serial trypsinization, we carried out scRNA-Seq on 4,385 of these cells. Markers expressed by cells sharing some lineage with CAFs, but not expressed by CAFs, were absent from most cells isolated from GBM by serial trypsinization, including *EPCAM*, an epithelial cell marker expressed by 0.07% of the cells, *SMTN*, a smooth muscle cell marker expressed by 4% of the cells, and *PECAM1*, an endothelial marker expressed by 10.7% of the cells. As with other cancers (16), cell-surface markers for CAFs were expressed by these cells isolated by serial trypsinization of patient GBMs, but not uniformly (Supplemental Figure 5).

We therefore used a previously described negative selection strategy (17) on these 4,385 serially trypsinized cells to exclude non-CAF stromal cells expressing cell-surface markers, defining them as epithelial cells (*EPCAM*), endothelial cells (*PECAM1*), pericytes (*CSPG4*), or immune cells (*PTPRC*). Among the remaining 75.2% of cells, transcripts for cytoplasmic CAF markers established in other cancers were robustly expressed, including actin alpha 2 (*ACTA2*) (18) and *COL1A1* (19), expressed in 73.8% and 96.5% of cells, respectively. Additionally, the following previously reported CAF-associated cell-surface markers were expressed in this population: *FAP* (10) (5.6%), *TNC* (20) (37.3%), *PDGFRA* (10) (8.9%), *PDGFRB* (18) (12.7%), *PDPN* (21) (19.8%), and *S100A4* (10) (17.2%) (Supplemental Table 2). Overall, 86.5% of the cells not expressing epithelial, endothelial, pericyte, or immune cell-surface markers or 65.1% of the total 4,385 cells expressed at least 1 of 9 CAF markers (*ACTA2*, *FAP*, *PDGFRA*, *PDGFRB*, *PDPN*, *S100A4*, *TNC*, *VIM*, and *COL1A1*) (Supplemental Table 2). We then calculated CAF probability scores for individual cells isolated by serial trypsinization of patient GBM based on the absence of 5 non-CAF markers (the 4 listed above, *PTPRC*, *EPCAM*, *PECAM1*, and *CSPG4*, plus *RGS5*, a second pericyte marker) and their degree of expression of the 9 CAF markers listed above (Supplemental Figure 6, A–C, and Figure 1D). We then used CopyKAT (22) to infer copy number alterations at 5 Mb resolution by averaging large chromosomal regions (1 Mbp). Copy number variation (CNV) analysis revealed that, within the cells isolated by serial trypsinization, most cells with high CAF probability scores lacked chromosomal alterations, while some cells with variable CAF probability scores exhibited the gain of chromosome 7 and loss of chromosome 10 that is a hallmark of GBM (23) (Figure 1E, Supplemental Figure 6, D and E, and Supplemental Figure 7). Filtering these cells exhibiting gain of chromosome 7 and loss of chromosome 10 out of the cells with high CAF probability scores revealed that 52% of the cells isolated by serial trypsinization of patient GBMs were CAFs, 22% were myeloid, 20% were malignant, and 6% were oligodendrocytes (Figure 1F). Further evidence supporting serially trypsinized cells being CAFs was obtained via 3 approaches. First, mitochondrial single nucleotide variant (mito-SNVs) analysis revealed 6 mitochondrial genotypes in cells cultured by serial trypsinization, with cells with high CAF probability scores having a mitochondrial genotype distinct from that of neoplastic cells with CNV alterations (Supplemental Figure 8), meaning that cells with high CAF probability scores had a lineage distinct from that of tumor cells. Second, we identified cells with high astrocyte probability scores based on their expression of 10 astrocyte markers (Supplemental Methods) and found no overlap between cells with high CAF and high astrocyte probability scores (Supplemental Figure 9). Third, we identified the most differentially expressed genes between cells with high CAF probability scores versus those without, which included genes expressed by CAFs in other cancers: *CCDC80* (24), *BGN* (25), *COL4A2* (25), *COL5A1* (25), *NR2F2* (17), *COL3A1* (25), *INHBA* (25), *STC2* (26), and *LOXL2* (25) (Supplemental Figure 10).

To understand the lineage and differentiation of these cells identified as CAFs after serial trypsinization of patient GBMs, we studied pseudotime reconstruction of reembedded CAF scRNA-Seq data using the Monocle 3 (27) approach, revealing that an earlier CAF population evolved into 2 CAF subtypes (Figure 1G). We computed the gene expression along calculated pseudotime trajectories, revealing distinct expression profiles from early (*EVA1B*, *DDIT4*) to late-stage (*ACTA2*, *SRGN*) GBM CAFs (Figure 1H). These findings are consistent with breast cancer studies (28) and suggest that *ACTA2*-expressing CAFs represent a more differentiated CAF subtype across cancers. We also found gene expression patterns within serially trypsinized cells from patient GBMs consistent with 3 CAF subtypes (steady state–like, mechanoresponsive, and immunomodulatory) conserved across multiple cancer types and species (29) (Supplemental Figure 11 and Supplemental Methods).

### Identifying CAFs in patient GBMs using scRNA-Seq.

**Figure 2:**
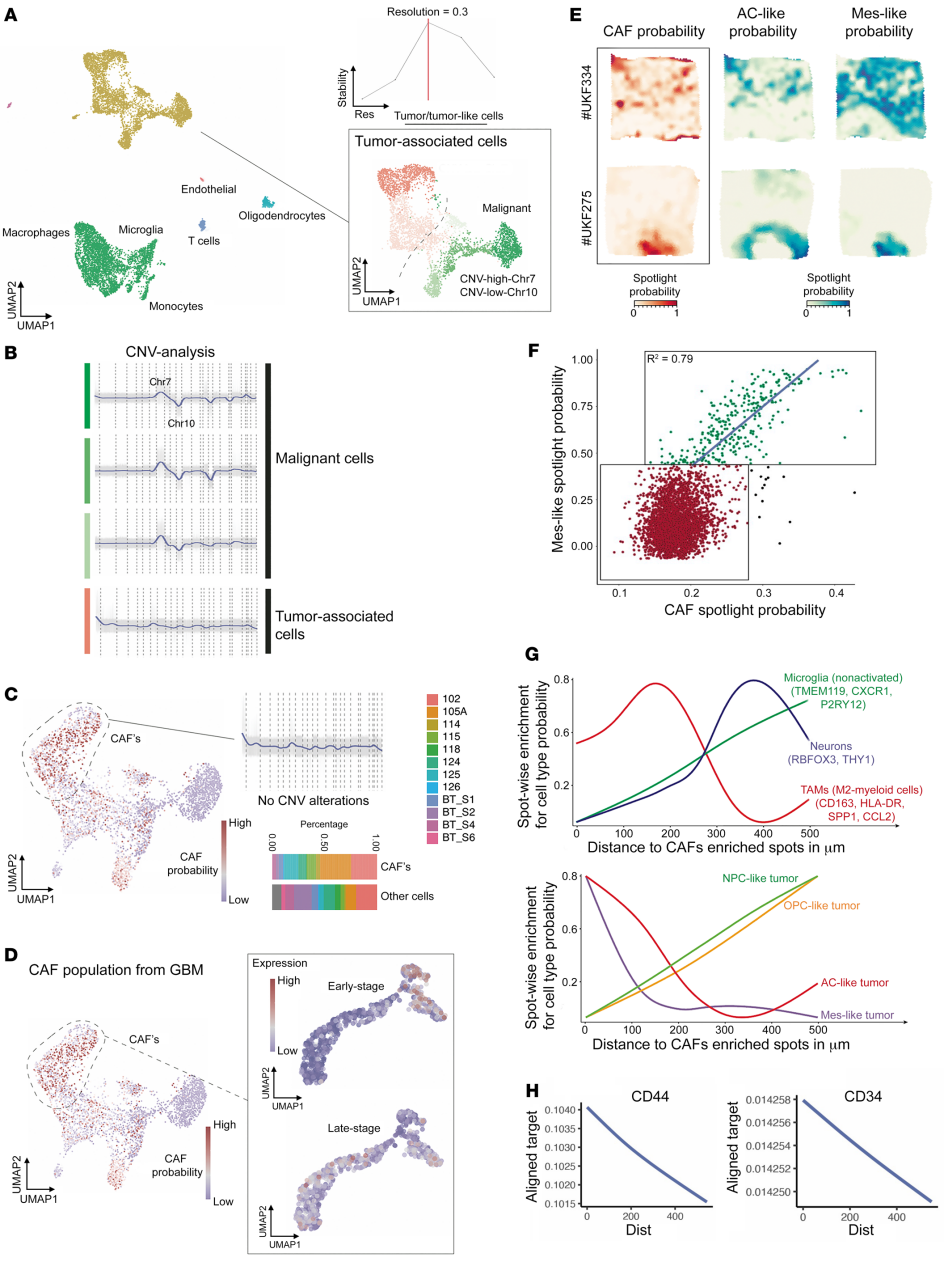
Identification of CAFs in GBM by scRNA-Seq of patient GBMs. (**A**–**D**) scRNA-Seq results from 12 patient GBMs (30, 31) were analyzed using mutual nearest neighbor horizontal integration followed by SNN clustering. (**A**) Optimal number of clusters was determined by the cluster stability score (upper right) resulting in 18 robust cell clusters. While most stromal cells clustered away from tumor cells, some stromal cells clustered close to tumor cells. (**B**) Green cells were tumor cells based on CNV analysis, while red cells were stromal. (**C**) CAF probability scores based on exclusive gene signatures and defined exclusion criteria were computed (left side). CAFs exhibited no CNV alterations (upper right) and were identified in each of the 12 patients (lower right). (**D**) Presence of early versus late-stage CAF subtypes was evaluated in cells with high CAF probability scores, with late-stage CAFs predominating over early stage CAFs in these 12 patients. (**E**–**H**) Deconvolution of spatially resolved transcriptomics was performed. (**E**) Surface plots obtained from 6 × 6 mm tissue samples revealing that CAFs (left) spatially correlated with the MES and astrocyte-like (AC-like) GBM cell signatures (30). Two examples of low overlap (top) and high overlap (bottom) are demonstrated. (**F**) Spatial correlation between CAFs and mes-GBM cells was significant (*P* < 0.001, Pearson’s *R^2^* = 0.79). (**G** and **H**) Line diagrams show the spatial relationship between CAFs and other cell types or states (tumor subtypes). The *x* axis represents the relative distance to CAFs. The *y* axis shows the cell type/state probability of a particular gene set or spotlight probability. The spatial distance of CAFs to different cell types or states was computed based on ranked cell-type probability. If high cell probability values are displayed at a short distance (dist) from CAFs, the likelihood of a spatial relationship is high, as occurred for (**G**) mes- and AC-GBM cells and M2 TAMs and (**H**) CD44^+^ GSCs and CD34^+^ endothelial cells.

To determine whether CAFs could be identified in scRNA-Seq of patient GBMs, we analyzed scRNA-Seq results from 12 patient GBMs (30, 31). Using mutual nearest neighbor horizontal integration followed by shared nearest neighbor (SNN) clustering, we found that the optimal number of clusters was determined by the cluster stability score, resulting in 18 robust cell clusters (Figure 2A) seen in each individual patient (Supplemental Figure 12).

The uniform manifold approximation and projection (UMAP) plot revealed closely grouped clusters of tumor and stromal cells (Figure 2A), distinguished by CNV analysis (Figure 2, B and C, and Supplemental Figure 13), revealing tumor cells exhibiting gain of chromosome 7 and loss of chromosome 10 and no chromosomal aberrations in tumor-associated stromal cells (Figure 2B). We then calculated CAF probability scores for individual cells in these clusters (Figure 2C), revealing that CAFs were in each of the 12 patient GBMs (Figure 2C). Notably, clusters housing cells with high CAF probability scores did not exhibit CNV (Figure 2C) and did not express macrophage marker *AIF1*, oligodendrocyte marker *OLIG1*, T cell marker *CD3D*, or endothelial marker *PECAM1* (Supplemental Figure 14).

We then determined whether these CAFs we identified using patient GBM scRNA-Seq harbored the same early and fully differentiated subtypes seen in scRNA-Seq of cells isolated by serial trypsinization of patient GBMs. Extracting the pseudotime-dependent genes with an early stage versus those with a late stage in the cells we had identified as CAFs in patient GBMs revealed that, while cells isolated by serial trypsinization harbored a mix of early and fully differentiated CAF subtypes, patient GBMs harbored mostly the fully differentiated CAF subtype (Figure 2D).

To determine what cell types these CAFs were in closest spatial proximity to, we integrated scRNA-Seq and previously described spatially resolved transcriptomics from 16 patients (32) to spatially localize CAFs (Figure 2, E–G) using seeded nonnegative matrix factorization (NMF) regression (33). Of the 4 malignant cell states in GBM (30), CAFs were most spatially correlated with the mesenchymal-like (MES-like) signature (Figure 2, E–G). CAFs were also in close proximity to M2 protumoral macrophages and were more distant from microglia and neuronal gene signatures (Figure 2G). CAFs were also noted to be in close proximity to cells expressing glioblastoma stem cell (GSC) marker CD44 (Figure 2H) and were enriched in the perivascular niche where GSCs reside (34) based on their proximity to endothelial cells expressing *CD34* (Figure 2H) or *CDH5* (Supplemental Figure 15A). CAFs also existed at both close and remote distances from pericytes, consistent with these 2 cell types being distinct not just in gene expression (Figure 1C), but in spatial localization too (Supplemental Figure 15B), and were remote from epithelial cells (Supplemental Figure 15C).

### CAFs induce protumoral effects on GSCs.

**Figure 3:**
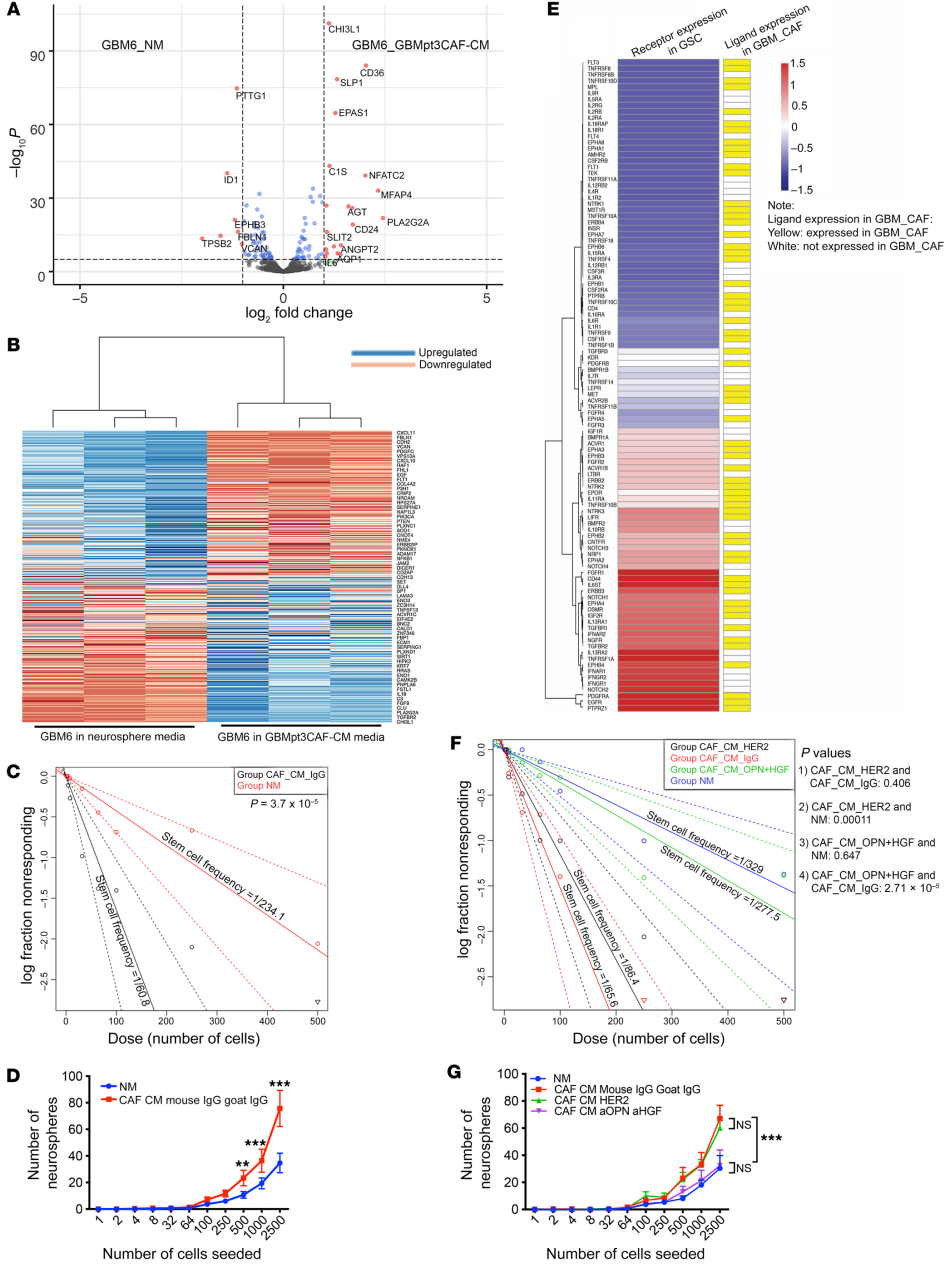
CAFs induce protumoral effects on GSCs. Multiplex transcriptomic analysis using the NanoString nCounter platform revealed cancer progression genes upregulated by GBMpt3CAF CM in GBM6 GSCs. (**A**) Volcano plot showing significantly (*P* < 0.05) up- (right of rightmost vertical dashed line) and downregulated genes (left of leftmost vertical dashed line). (**B**) Heatmap showing significantly (*P* < 0.05) up- and downregulated genes. (**C**) Limiting dilution sphere-formation assay represented by Poisson’s distribution shows increased GSC frequency with GBM6 cells in CAF_CM (*P* = 3.7 × 10^–5^). (**D**) Limiting dilution sphere-formation assay showing that CAF_CM increases neurosphere formation (2,500 cells: *P* < 0.0001; 1,000 cells: *P* = 0.001; 500 cells: *P* < 0.0067). (**E**) Receptor expressions in GBMpt1CAFs and GBMpt2CAFs (Supplemental Table 3) were mapped to their cognate ligands/agonists expressed by GBM6 neurospheres (37) based on a database of 491 receptor-ligand interactions (67). Shown are cognate pairs coexpressed by GBM CAFs and GSCs for which FPKM of the ligand is greater than 0.05 and read counts of the receptor are greater than 10 (174 CAF ligands with receptors expressed by GSCs). (**F**) Limiting dilution sphere-formation assay represented by Poisson’s distribution shows that the increased GSC frequency in CAF_CM is mitigated by combining anti-HGF and anti-OPN (*P* values on graph). GSC frequency was not mitigated by HER2 antibody in CAF_CM. (**G**) Limiting dilution sphere-formation assay showing that induction of neurosphere formation by CAF_CM is mitigated by combining anti-HGF and anti-OPN (2,500 cells: *P* < 0.0001; 1,000 cells: *P* = 0.009; 500 cells: *P* = 0.04). Sphere-formation was not mitigated by anti-HER2 in CAF_CM (*P* = 0.7–0.8). ANOVA with post hoc Tukey’s test. For limiting dilution sphere-forming assays, log-fraction plots of the limiting dilution model fitted to the data are shown. The slope of the line is the log-active cell fraction. Dotted line shows 95% CI. Data value with zero negative response at a particular dose is represented by a downward pointing triangle. **P < 0.01; ***P < 0.001.

Because GBM CAFs resided in the perivascular niche close to tumor-initiating GSCs, we analyzed the effects of GBM CAFs on these GSCs. This was done by taking GSC-containing neurospheres derived from GBM6 cells and culturing them in conditioned media (CM) from GBMpt3CAFs (CAF_CM) for 72 hours. These cells were then transcriptomically assessed and compared with GBM6 neurospheres in control neurosphere media (NM) using the NanoString nCounter platform and a 770-gene multiplex PanCancer progression panel (nanostring.com) to analyze expression of cancer progression genes. The analysis revealed that the GBM CAF secretome upregulated cancer progression pathways, including HIF-1α, EMT, and cell proliferation in GSCs (Figure 3, A and B, and Supplemental Figures 16 and 17). A mechanism for GBM CAF activation of the HIF-1α pathway was identified when we found that GBMpt5CAF CM increased production of ROS, which drive HIF-1α (35) by GSCs derived from GBM43 (*P* < 0.05; Supplemental Figure 18)

We then analyzed consequences of these transcriptomic changes by determining whether CAF_CM induced changes in GSC-enriched GBM neurospheres. To do so, we carried out limiting dilution neurosphere-formation assays (36), revealing increased GBM6-derived GSC frequency in GBMpt5CAF CM (1/60.8) compared with NM (1/234.1) (*P* = 3.7 × 10^–5^; Figure 3C). GBMpt5CAF CM also increased the yield of GBM6 neurospheres at different dilutions (2,500 cells, *P* < 0.0001; 1,000 cells, *P* = 0.0001; 500 cells, *P* = 0.0006) (Figure 3D). Culturing GSC-containing neurospheres derived from luciferase-expressing GBM6 cells in CM from GBMpt4CAFs for 72 hours led to increased bioluminescence compared with growing these cells in NM (*P* < 0.001; Supplemental Figure 19). Consistent with these results, incubating GSC-containing neurospheres from DBTRG-05MG GBM cells in GBMpt1CAF CM for 24 hours increased the expression of GSC genes *Nanog* (6.7-fold, *P* = 0.009), *Sox2* (5.0-fold, *P* < 0.001), and *Oct4* (3.0-fold, *P* = 0.005) (Supplemental Figure 20).

To identify mediators of CAF effects on GSCs, we created a resource of inferred crosstalk by mapping the expression of GSC receptors to that of their cognate ligands/agonists expressed by CAF cells, using RNA-Seq results from GBM CAFs (Figure 1C) and GBM6-derived neurospheres (37) (Figure 3E and Supplemental Table 3). In identifying candidates from this GSC-CAF receptor-ligand analysis (Supplemental Table 3) to investigate as mediators of CAF-mediated GSC enrichment, we chose osteopontin (OPN) and its receptor CD44 and hepatocyte growth factor (HGF) and its receptor c-Met because OPN and HGF are expressed by mechanoresponsive and immunomodulatory CAF subtypes (29), respectively, which we identified in cells isolated in serial trypsinization of patient GBMs (Supplemental Figure 11), and because both enrich GSCs (38, 39). We conducted a neurosphere formation assay in the presence of anti-OPN and/or anti-HGF neutralizing antibodies. GBMpt5CAF CM increased the total area of GBM6 neurospheres (*P* < 0.001), which accounts for the number and size of neurospheres, effects mitigated by anti-HGF (*P* < 0.001) or anti-OPN (*P* < 0.001; Supplemental Figure 21). Similarly, GBMpt5CAF CM increased the frequency of GBM6 and GBM43 neurosphere formation in a limiting dilution assay in a manner reduced by combining anti-HGF and anti-OPN antibodies (GBM6: *P* = 2.71 × 10^–5^, Figure 3F; GBM43, *P* = 3.79 × 10^–9^, Supplemental Figure 22A). Combining anti-HGF and anti-OPN antibodies also reduced GBM6 and GBM43 sphere formation compared with CAF_CM with IgG control antibodies (GBM6: *P* < 0.001, Figure 3G; GBM43: *P* < 0.01, Supplemental Figure 22B). CAF_CM also increased GBM neurosphere diameter in a manner reduced by combining anti-HGF and anti-OPN antibodies in GBM6 (*P* < 0.001; Supplemental Figure 23A) and GBM43 (*P* < 0.001; Supplemental Figure 23B and Supplemental Figure 24) neurospheres grown in GBMpt5CAF CM. These results suggest that the increased neurosphere formation induced by CAFs is mediated through the OPN-CD44 and HGF-cMET axes. We then determined whether CAFs chemotactically attracted GSCs. We performed a chemotaxis assay comparing the migration of neurospheres derived from GBM6 cells toward control media or GBMpt1CAF CM and found no difference in chemotaxis (*P* = 0.1; Supplemental Figure 25).

### GSCs drive CAF chemotaxis and proliferation via PDGF and TGF-β pathways.

**Figure 4:**
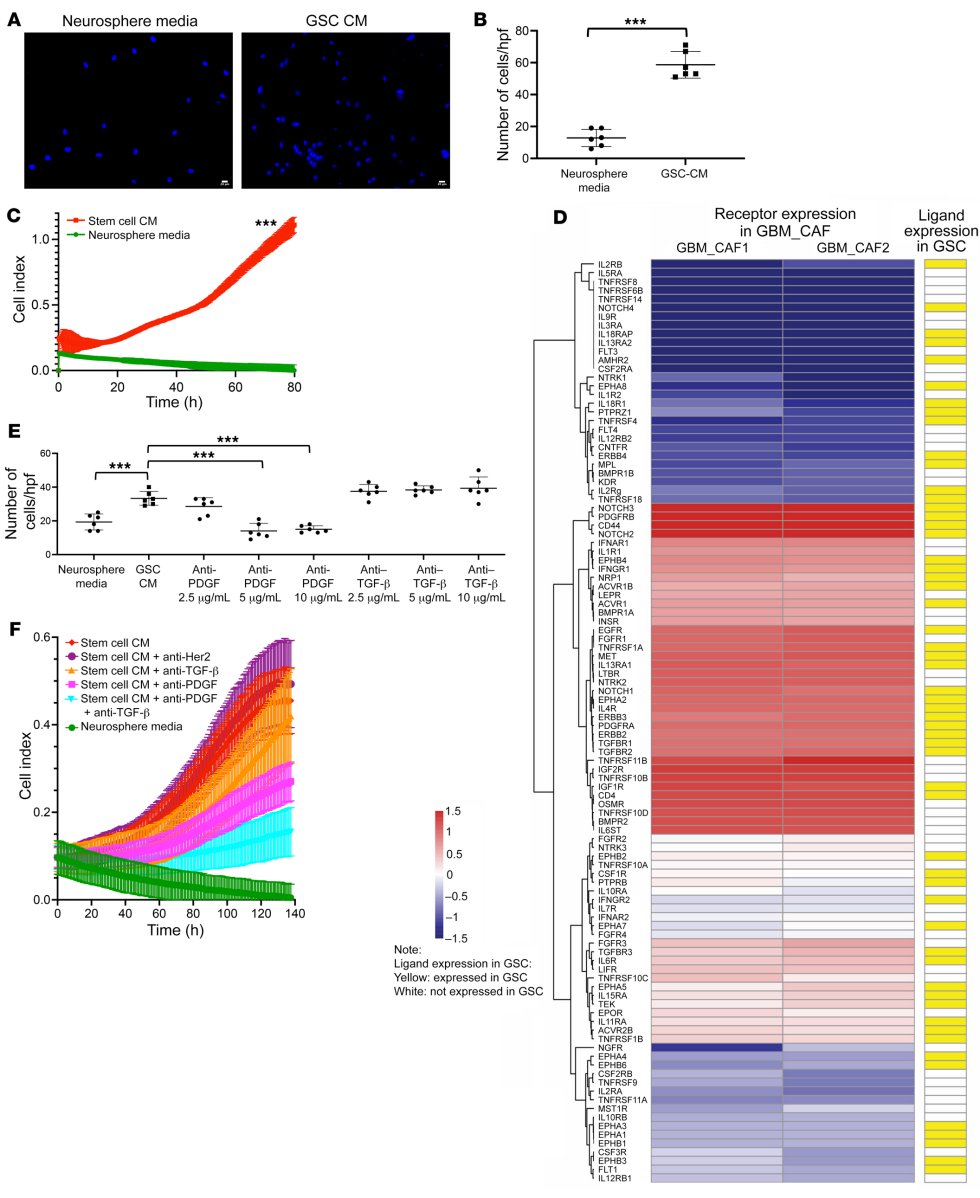
GSCs mediate CAF invasion and proliferation via PDGF and TGF-β pathways. Compared with NM, CM from GBM6 stem cell–enriched neurospheres (**A** and **B**) attracted more GBMpt1CAFs in chemotaxis assays (*n* = 6/group; *P* < 0.001, *t* test) and (**C**) stimulated GBMpt5CAF proliferation (*P* < 0.001 at all time points; *n* = 5/group; *t* test). Scale bars: 20 μm. (**D**) We mapped the expression of receptors expressed by GBMpt1CAFs and GBMpt2CAFs (Supplemental Table 2) to that of their cognate ligands/agonists expressed by GBM6 neurospheres (37) based on a database of 491 known receptor-ligand interactions (67). Shown are cognate pairs coexpressed by GBM CAFs and GSCs for which FPKM of the ligand is greater than 0.05 and read counts of the receptor are greater than 10, which represented 189 GSC ligands with receptors expressed by CAFs. (**E**) Chemotaxis of GBMpt1CAFs toward GBM6 neurosphere CM was abrogated by neutralizing antibodies against PDGF (*P* < 0.001 at 5 and 10 μg/mL), but not TGF-β. TGF-β–neutralizing antibodies did not abrogate invasion at 2.5–10 μg/mL (*P* = 0.3–0.7). PDGF-neutralizing antibodies reduced the number of invading cells at 5 and 10 μg/mL (*P* < 0.001; *n* = 6/group). ANOVA with post hoc Tukey’s test. (**F**) PDGF-neutralizing antibodies minimally reduced and TGF-β antibodies did not alter GBM43 GSC CM-induced GBMpt5CAF proliferation (PDGF: *P* = 0.1–0.5 from 0–119 hours, *P* = 0.02–0.047 from 120–140 hours; TGF-β: *P* = 0.2–0.6 from 0–140 hours), while combining these antibodies reduced GBM43 GSC CM-induced GBMpt5CAF proliferation (*P* = 0.01–0.04 from 105–119 hours, *P* = 0.007–0.009 from 120–140 hours, both antibodies versus no antibodies in GBM43 GSC CM; *P* = 0.02–0.04 from 120–140 hours, both antibodies versus anti-PDGF; *P* = 0.01–0.04 from 100–119 hours and *P* = 0.006–0.009 from 120–140 hours, both antibodies versus anti–TGF-β). HER2 antibodies exerted no effect (*P* = 0.6–0.8 from 0–140 hours) on GBM43 GSC CM-induced GBMpt5CAF proliferation. *n* = 5/group; ANOVA with post hoc Tukey’s test. ***P < 0.001.

Based on our spatial transcriptomics results (Figure 2, E–H), we hypothesized that GSCs may recruit CAFs to the perivascular niche of GBM. To ascertain whether CAFs were attracted to GSCs, we assessed the trans-Matrigel chemotactic response of CAFs to GSC CM (Figure 4A). We found that GSC CM attracted GBMpt1CAFs 5 times more than NM (*P* < 0.001; Figure 4B).

We also determined whether GSCs promote CAF proliferation. We found that, compared with their lack of growth in NM, CAFs grew more in GSC CM derived from GBM43 cells (*P* < 0.001 from 0–80 hours; Figure 4C) or GBM6 (*P* < 0.001 from 0–95 hours, *P* < 0.01 from 96–179 hours, *P* < 0.05 from 180–230 hours; Supplemental Figures 26 and 27 and Supplemental Table 4).

Then, to investigate potential mediators of these GSC effects on CAFs, we created the converse of our map between CAF ligands/agonists and GSC receptors (Figure 3E) by mapping the expression of receptors expressed by CAFs to that of their cognate ligands/agonists expressed by GSCs, using the RNA-Seq results described above (Figure 4D and Supplemental Table 3). Using this resource, to investigate mediators enabling GSCs to recruit CAFs and stimulate their proliferation, we focused on PDGF and TGF-β, since both appeared in our GSC CAF ligand-receptor analysis (Figure 4D and Supplemental Table 3) and both have receptor signaling in the mechanoresponsive CAF subtype (29) we identified in our cells cultured via serial trypsinization of patient GBMs (Supplemental Figure 10). Varying concentrations of neutralizing antibodies to TGF-β or PDGF were placed in GSC CM before the Boyden chamber and CAFs were applied. TGF-β neutralizing antibodies did not inhibit invasion at 2.5 to 10 μg/mL (*P* = 0.3–0.7; Figure 4E). A neutralizing antibody targeting PDGF-B in the PDGF-BB homodimer and the PDGF-AB heterodimer whose receptors *PDGFRA* and *PDGFRB* were preferentially expressed in the CAFs identified in our cells isolated from patient GBMs by serial trypsinization (Supplemental Figure 28) reduced the number of invading cells at 5 and 10 μg/mL (*P* < 0.001; Figure 4E). In terms of mediators of GSC-induced CAF proliferation, a neutralizing antibody against PDGFB minimally reduced and an antibody against TGF-β did not alter GBM43 GSC CM–induced CAF proliferation (PDGFB: *P* = 0.1–0.5 from 0–119 hours, *P* = 0.02–0.047 from 120–140 hours; TGF-β: *P* = 0.2–0.6 from 0–140 hours), while combining these antibodies reduced GBM43 GSC CM–induced CAF proliferation (*P* = 0.01–0.04 from 105–119 hours, *P* = 0.007–0.009 from 120–140 hours, both antibodies versus no antibodies in GBM43 GSC CM; *P* = 0.02–0.04 from 120–140 hours, both antibodies versus anti-PDGFB; *P* = 0.01-0.04 from 100–119 hours and *P* = 0.006–0.009 from 120–140 hours, both antibodies versus anti–TGF-β) (Figure 4F). Similarly, while antibodies against PDGFB or TGF-β did not affect GBM6 neurosphere CM-induced CAF proliferation (0–139 hours: *P* = 0.7–0.9, PDGF, *P* = 0.5–0.9, TGF-β), combining antibodies against PDGFB and TGF-β reduced GBM6 neurosphere-induced CAF proliferation (50–139 hours: *P* = 0.02–0.04, PDGFB+TGF-β versus TGF-β; 79–139 hours: *P* = 0.03–0.04, PDGFB+TGF-β versus PDGFB; 60–139 hours: *P* = 0.03–0.048, PDGFB+TGF-β versus GSC CM) (Supplemental Figure 29).

### CAFs fail to induce protumoral effects on nonstem GBM cells.

We then analyzed to determine whether GBM CAFs exerted protumoral effects on nonstem adherent GBM cells that were similar to the protumoral effects they had on GSCs. Adding GBMpt1CAF CM to nonstem adherent DBTRG-05MG cells did not change MES gene expression (*P* = 0.8; Supplemental Figure 30A). GBMpt1CAF CM also did not change morphology assessed by shape factor (40) (*P* = 0.06–0.8; Supplemental Figure 30B), Matrigel chamber invasion (*P* = 0.5; Supplemental Figure 30C), or proliferation (*P* = 0.3–0.9; Supplemental Figure 30D) of non–stem-adherent GBM6 cells. These results show that the protumoral effects of GBM CAFs are specific to GSCs.

### Effects of GBM CAFs on stroma in culture.

**Figure 5:**
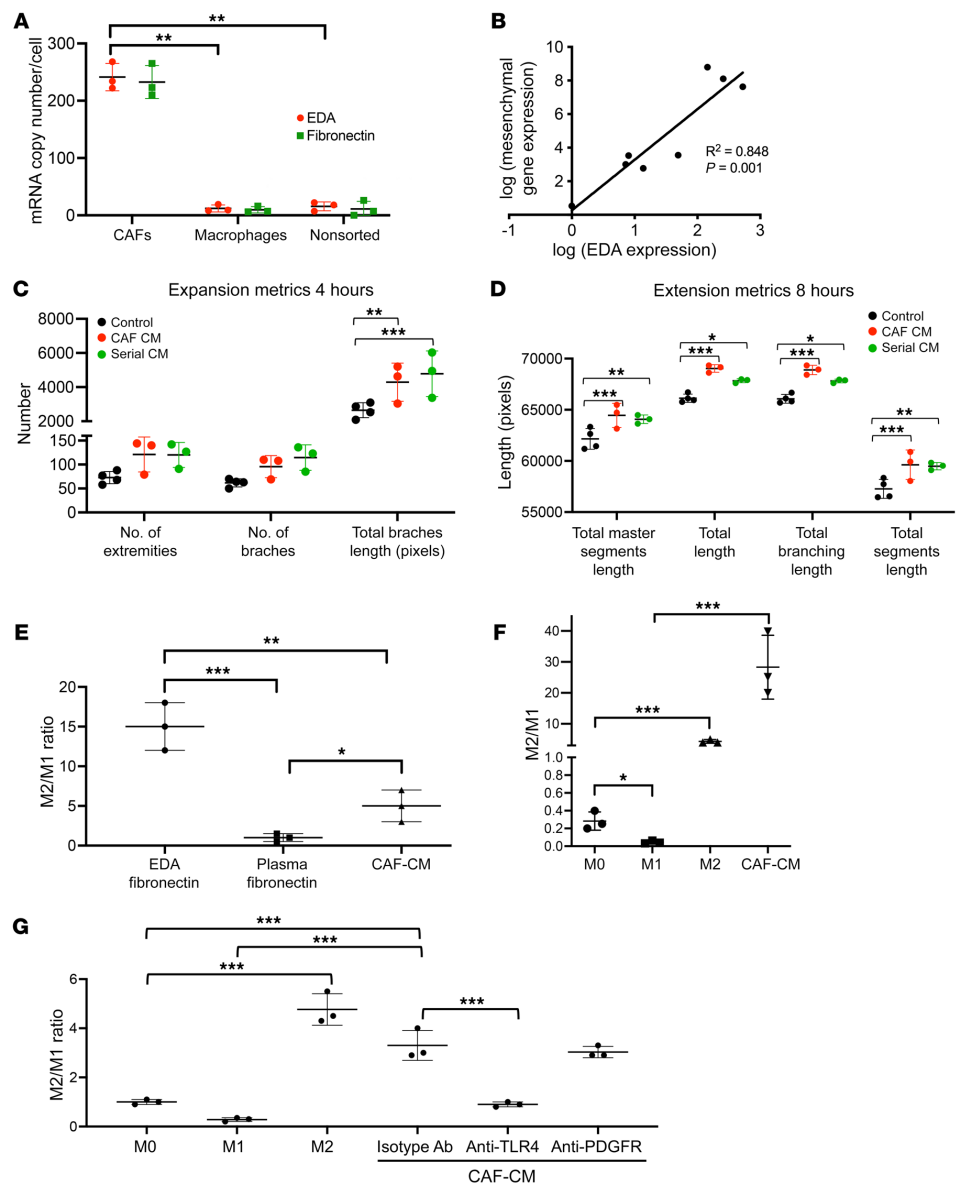
Effect of GBM CAFs on the tumor microenvironment in culture assays. (**A**) qPCR revealed elevated expression of total and EDA splice variant of FN in CAF-like cells isolated by serial trypsinization of patient GBMs relative to (a) CD11b^+^ TAMs (*P* = 0.008) and (b) a tumor cell–enriched population obtained by flow sorting a freshly resected GBM to eliminate CD11b^+^, CD31^+^, and CD3^+^ cells (*P* = 0.007; *n* = 3/group). Ct values were normalized to GAPDH. (**B**) EDA expression correlated with aggregate expression of 5 MES genes (Supplemental Table 6) as assessed by qPCR of newly diagnosed GBM patient specimens (*n* = 8; *P* = 0.0012). GBMpt4CAF CM increased (**C**) total branch length (*P* = 0.003) and (**D**) total master segment length (*P* < 0.001), total length (*P* < 0.001), total branching length (*P* < 0.001), and total segment length (*P* < 0.001). Serial CM from GBM cells grown in CAF CM did not increase these metrics compared with that of HUVECs in CAF CM (*P* = 0.1-0.9; *n* = 6/group). (**E**–**G**) GBMpt2CAF_CM caused M2 macrophage polarization based on ratio of qPCR gene expression of 3 M2 genes (*ARG1*, *TGFB1*, and *MMP9*) to 3 M1 genes (*NOS2*, *CXCL10*, and *IL1B*). (**E**) CAF_CM and CAF-produced EDA caused more M2 polarization of cultured macrophages derived from circulating human monocytes than plasma FN lacking the EDA splice variant (*n* = 3/group; *P* = 0.04, CAF_CM versus plasma FN; *P* < 0.001, EDA versus plasma FN; *P* = 0.003, CAF_CM versus EDA). (**F**) CAF_CM drove more M2 polarization of THP-1 immortalized monocytes differentiated into macrophages followed by incubation in CAF_CM than a cytokine-positive control that drives M2 polarization (*n* = 3/group; *P* < 0.001). (**G**) Effects of CAF CM on M2 polarization of cultured macrophages derived from human monocytes isolated from peripheral blood were reduced by a blocking antibody against EDA receptor TLR4 (*n* = 3/group; *P* < 0.001). All *P* values were generated by ANOVA with post hoc Tukey’s test statistical analysis. *P < 0.05; **P < 0.01; ***P < 0.001.

Because our RNA-Seq analysis revealed that fibronectin (FN; *FN1* gene) was differentially expressed in GBM (log_2_[fold change] = 5.3; *P* = 6.9 × 10^–22^) CAFs relative to pericytes (Supplemental Table 1) and because FN is the most abundant GBM ECM protein (41), we further analyzed CAF *FN1* expression. First, using the GlioVis databank (http://gliovis.bioinfo.cnio.es/), we found that GBM had higher *FN1* expression than nontumor brain samples (*P* < 0.001, Supplemental Figure 31A). GBM also had higher *FN1* expression than low-grade gliomas (*P* < 0.001, Supplemental Figure 31B). Because FN lacking splice variants is not part of cancer pathogenesis (42), we then analyzed expression of total FN and its extra domain A (EDA) splice variant in GBM CAFs, tumor-associated macrophages (TAMs), and tumor cells. Quantitative PCR (qPCR) revealed 32-fold more total FN and 16-fold elevation of the EDA splice variant in CAFs relative to TAMs (*P* = 0.002–0.004; Figure 5A) and tumor cells (*P* = 0.002; Figure 5A), suggesting that EDA is a more specific GBM CAF biomarker than the cell-surface receptors described for other CAFs (Supplemental Figure 4 and Supplemental Table 2). Transcriptomic analysis also revealed a positive correlation between patient GBM expression of EDA and aggregate expression of MES subtype genes *CHI3LI*, *TIMP1*, and *SPOCD1* that confer a worse prognosis (*P* = 0.0012; Figure 5B) (43).

We then investigated angiogenic effects of CAFs because of our finding that CAF_CM activated HIF-1α signaling, which drives angiogenic factor VEGF (44), in GSCs (Figure 3C). Consistent with these pathways activated by CAF_CM in GSCs, we found that, while GBMpt5CAFs secreted less VEGF than neurospheres from GBM6 or GBM43 cells, VEGF secretion by GBM6 and GBM43 cells increased when the cells were grown in GBMpt5CAM CM (*n* = 3/group; *P* = 0.02, GBM6; *P* < 0.001, GBM43; Supplemental Figure 32).

In contrast to these findings suggesting that CAFs exerted angiogenic effects via GBM cells as an intermediary, when we performed functional assays in cultured HUVECs, we found that adding GBMpt4CAF CM to cultured HUVECs without GBM cells increased aspects of the first 2 of the 3 stages of angiogenesis, expansion of the network by tip cells and tubule formation, without affecting the third stage (fusion of the newly formed vessels) (45). Specifically, CAF CM increased total branch length (*P* = 0.003), a measure of HUVEC expansion, at 4 hours and total master segments length (*P* < 0.001), total length (*P* < 0.001), total branching length (*P* < 0.001), and total segment length (*P* < 0.001), measures of HUVEC extension, at 8 hours (Figure 5, C and D, and Supplemental Figures 33 and 34) without affecting mesh fusion at 16 hours (Supplemental Figure 35 and 36). Additionally, a serial CM experiment in which HUVECs were grown in CM taken from GBM6 cells grown in CAF CM did not increase these metrics compared with HUVECs in CAF CM (*P* = 0.1–0.9) (Figure 5, C and D, and Supplemental Figures 33–36), suggesting that CAFs exert direct angiogenic effects on endothelial cells not potentiated by GBM cells.

We then investigated the effects of CAFs on macrophages, which make up 40% of the mass of GBM (46). We found that GBMpt2CAF CM and the EDA splice variant of FN that they produce caused more M2 polarization of cultured macrophages derived from human monocytes isolated from peripheral blood than plasma FN lacking the EDA splice variant (*P* = 0.04, CAF_CM versus plasma FN; *P* < 0.001, EDA versus plasma FN; Figure 5E). Similarly, when THP-1 monocytes were differentiated into macrophages followed by incubation in GBMpt2CAF CM, GBMpt2CAF CM drove more M2 polarization than a cytokine-positive control known to drive M2 polarization (*P* < 0.001; Figure 5F). The M2 polarization GBMpt2CAF CM induced in cultured macrophages derived from circulating human monocytes was reversed by a blocking antibody against TLR4, a receptor for EDA FN (47) (*P* = 0.01; Figure 5G). While CAFs caused M2 macrophage polarization, CAFs did not induce macrophage proliferation (*P* = 0.3–0.9; Supplemental Figure 37A) or chemotaxis (*P* = 0.7; Supplemental Figure 37B).

### Regional variation in CAF localization in GBM.

**Figure 6:**
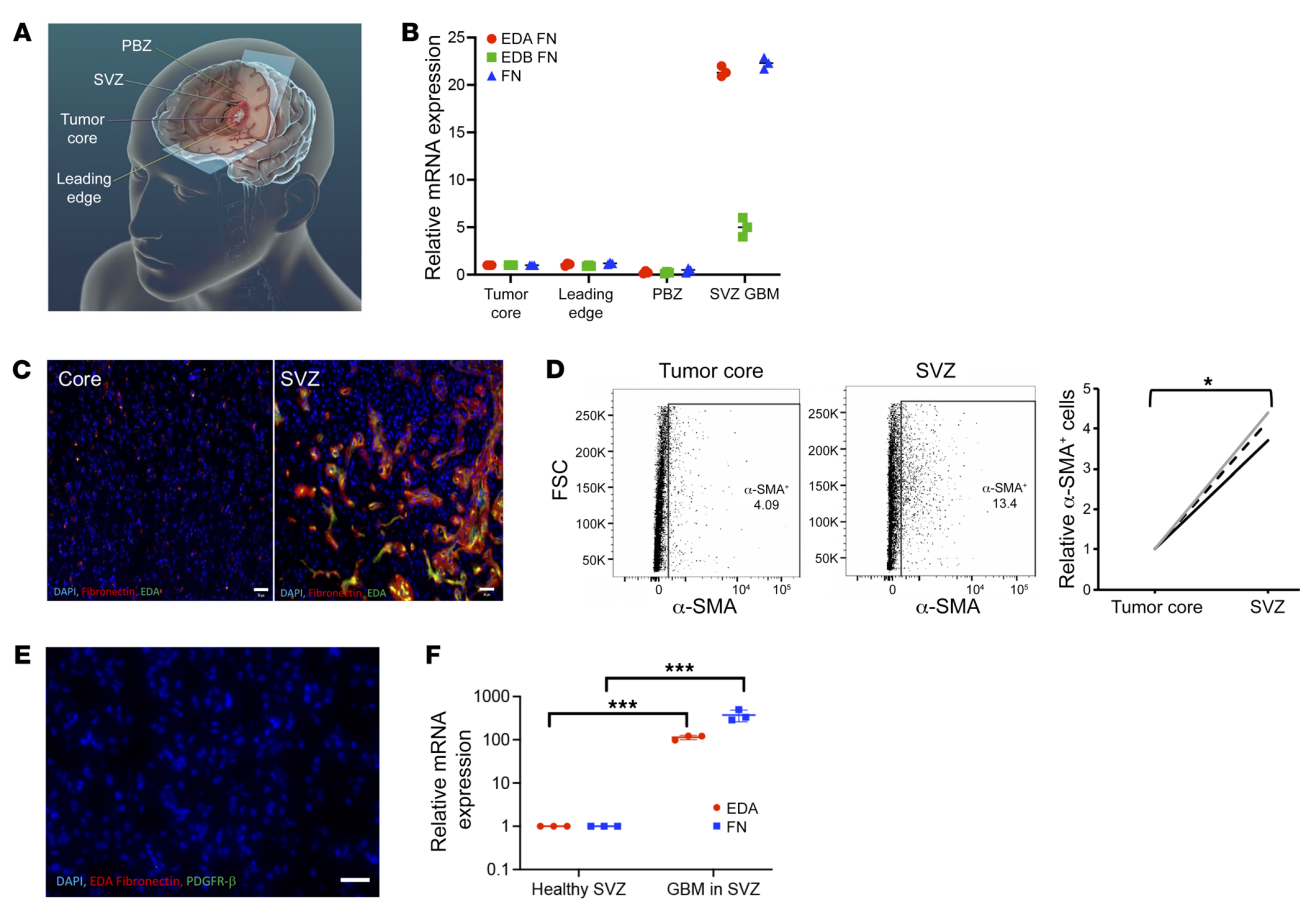
Regional variation of CAF localization in GBM. (**A**) Schematic showing where site-directed biopsies from patient GBMs were taken. (**B**) qPCR revealed that SVZ GBM had 22-fold increased expression of EDA (*P* < 0.001), 22-fold increased total FN expression (*P* < 0.001), and 5-fold increased EDB expression (*P* = 0.02) compared with the tumor core (*n* = 3/group ANOVA with post hoc Tukey’s test). (**C**) Immunofluorescence confirmed elevated EDA (green) and total FN (red) in SVZ GBM compared with the tumor core. Scale bars: 30 μm. (**D**) Flow cytometry for CAF marker α-SMA reveals elevation in the SVZ compared with the tumor core (*n* = 3 paired specimens; *P* = 0.02 paired *t* test). (**E**) Immunofluorescence revealed no PDGFR-α or EDA staining in the SVZ of a GBM patient whose tumor did not involve the SVZ. Original magnification, ×100. Scale bar: 30 μm. (**F**) Total and EDA FN expression by qPCR was elevated in SVZ GBM but virtually undetectable in tumor-free SVZ from epilepsy surgery (*P* < 0.001; *t* test; *n* = 3). *P < 0.05; ***P < 0.001.

To evaluate CAF levels in different tumor regions, we acquired site-directed biopsies from different regions of patient GBMs (48, 49): (a) tumor core; (b) leading edge of tumor enhancement; (c) peritumoral brain zone (PBZ), nonenhancing FLAIR bright regions surrounding the tumor; and (d) subventricular zone (SVZ), the largest germinal zone in the brain found along the lateral walls of the lateral ventricles, which houses the neural stem cells believed to produce GSCs (50) in cases in which tumor is involved this area (Figure 6A). We then performed qPCR for FN and its EDA and EDB splice variants, revealing that samples from SVZ GBM had 22-fold increased expression of EDA (*P* < 0.001), a marker we found to be highly expressed by CAFs, and 22-fold more FN expression (*P* < 0.001), but just 5-fold increased EDB expression (*P* = 0.02) normalized relative to the tumor core (Figure 6B). Immunofluorescence also revealed SVZ GBM to be enriched for EDA FN (Figure 6C). SVZ GBM was also enriched by flow cytometry for cells expressing α-SMA, a marker expressed by most of our cultured CAF cells (Supplemental Table 2), with 4.9% of tumor core cells expressing α-SMA compared with 13.4% of SVZ GBM cells (*P* = 0.02; Figure 6D). No EDA staining occurred in SVZ samples from autopsies of GBM patients whose tumors did not involve the SVZ (Figure 6E and Supplemental Figure 38). No staining for EDA (Supplemental Figure 39) and no detectable EDA mRNA by qPCR (Figure 6F) was observed in SVZ samples from non–tumor-bearing patient specimens from epilepsy surgeries. To determine whether CAF enrichment in tumor-bearing SVZ was related to GSC enrichment in this area, we performed qPCR on biopsies of SVZ-containing GBM versus tumor core and found unchanged expression of GSC markers nestin or CD44 (*P* = 0.1; Supplemental Figure 40A). Similarly, RNA-Seq from regional biopsies (http://cbi.ucsf.edu/apps/shinyproxy/app/GliomaAtlas3D) revealed no correlation between nestin or CD44 expression and distance from the ventricle (*P* = 0.1–0.6; Supplemental Figure 40B).

### Inclusion of CAFs with GSCs induces tumor growth in vivo.

**Figure 7:**
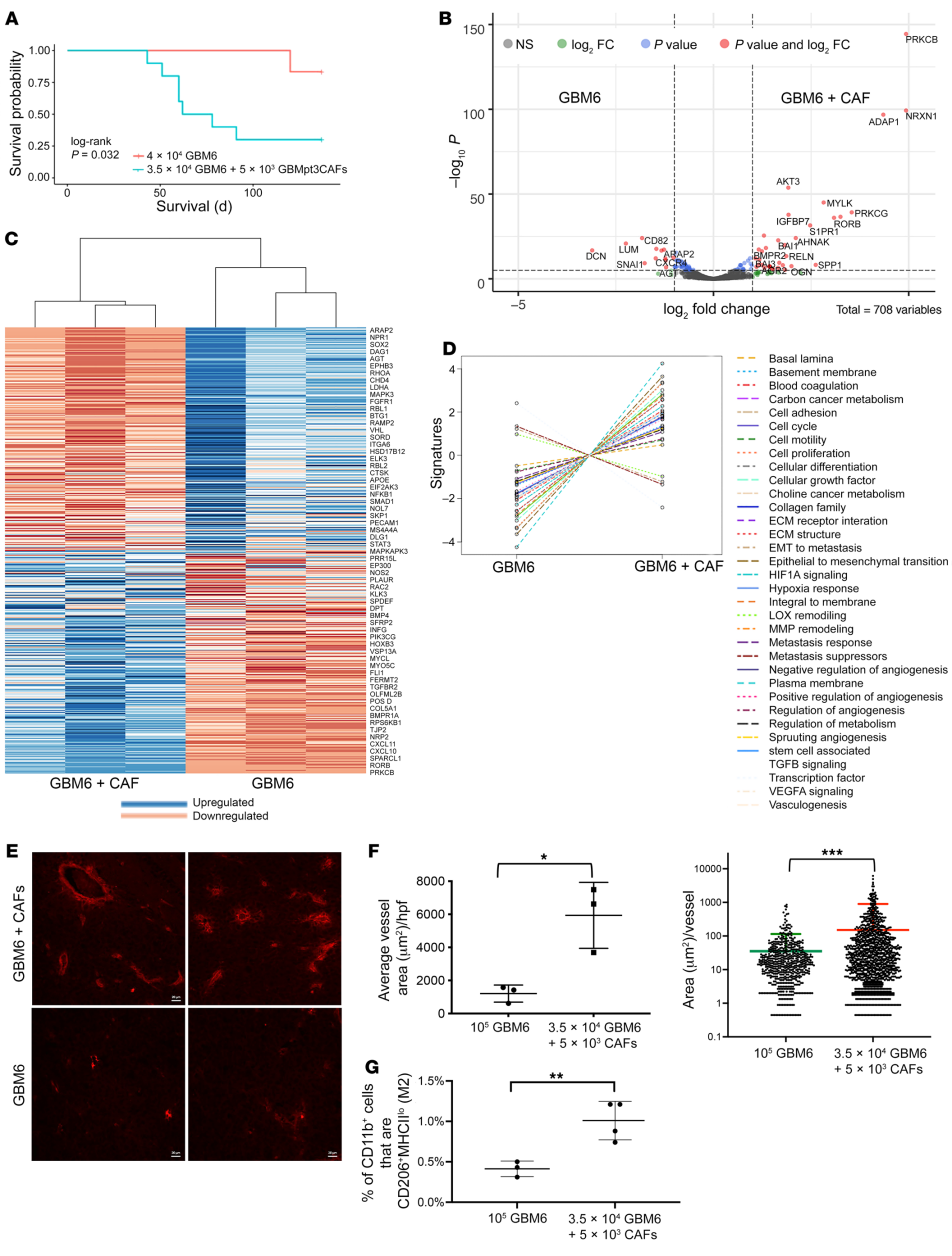
CAFs induce GBM tumor growth intracranially in vivo. (**A**) Kaplan-Meier curve showing intracranial implantation of 3.5 × 10^4^ GBM6 neurospheres with 5 × 10^3^ GBMpt3CAFs reduced survival compared with mice receiving 4.0 × 10^4^ GBM6 neurospheres, a threshold not associated with tumor formation in most mice (*n* = 10/group; *P* = 0.03). Compared with mice receiving 10^5^ GBM6 cells in neurospheres (higher number used to generate tumors), intracranial implantation of 3.5 × 10^4^ GBM6 neurospheres with 5 × 10^3^ CAFs upregulated cancer progression genes as determined by NanoString nCounter multiplex analysis using a PanCancer progression codeset, and as seen by (**B**) volcano plot showing significantly (*P* < 0.05) up- (to the right of rightmost vertical dashed line) and downregulated genes (to the left of leftmost vertical dashed line). (**C**) Heatmap showing significantly (*P* < 0.05) up- and downregulated genes constructed based on the log_2_ (fold change) and significant *P* adjusted value. (**D**) Pathway analysis showing that CAFs upregulated HIF-1 signaling, EMT, and cell proliferation pathways in GBM6 tumors (*P* < 0.003). (**E**) Immunofluorescence images (×20 magnification) showing increased vasculature, labeled via rhodamine B-dextran perfusion in sections from mice with GBM6+CAFs, quantified by (**F**) total vessel area/hpf (*P* = 0.02); *P* = 0.0002) (3 mice/group; 8 fields/mouse; *t* test). (**G**) CAFs increased the percentage of macrophages that were CD206^+^ M2 protumoral macrophages in GBM6 neurosphere-derived tumors (*P* = 0.0096; *t* test). Scale bar: 20 μM. *P < 0.05; **P < 0.01; ***P < 0.001.

To determine whether the protumoral effects of CAFs on GSCs we noted in cultured neurospheres also occurred in vivo, we intracranially implanted 40,000 GBM6 neurosphere cells, below the 100,000 neurosphere–cell threshold needed to establish intracranial GBM6 tumors (51), and 35,000 GBM6 neurosphere cells mixed with 5,000 GBMpt3CAFs into athymic mice (*n* = 10/group). Including CAFs with neurospheres enabled tumor growth to reach end point in most mice, which did not occur without CAFs (*P* = 0.03; Figure 7A), revealing that the tumor-promoting effects of CAFs on GSCs that we noted in culture also occurred in vivo. In fact, adding 5,000 GBMpt3CAFs to 35,000 GBM6 neurosphere cells caused mice with 35,000 GBM6 neurosphere cells to reach end point at the same time point as mice with 100,000 GBM6 neurosphere cells and no CAFs (*P* = 0.4; Supplemental Figure 41), revealing the magnitude of the tumor-promoting effects of CAFs on GSCs in vivo. Moreover, GBM43, a faster growing patient-derived xenograft (PDX) than GBM6, grew faster when 35,000 GBM43-derived neurosphere cells were implanted alongside 5,000 GBMpt5CAFs than when 40,000 GBM43 neurosphere cells were implanted (*P* < 0.05; Supplemental Figure 42). The growth-promoting effect of CAFs on PDX neurospheres was not seen with normal fibroblasts, as implanting 5,000 normal human fibroblasts with 35,000 GBM43-derived neurosphere cells did not alter survival compaared with implanting 40,000 GBM43-derived neurosphere cells (*P* = 0.98; Supplemental Figure 42). This growth-promoting effect of CAFs on PDX neurospheres was due to effects of CAFs on the PDX cells and not from any oncogenic potential of CAFs, as implanting 40,000 GBMpt5CAFs alone did not produce tumors (Supplemental Figure 43). To track CAFs in vivo, GBMpt5CAFs were labeled with GFP before being implanted with GBM43 neurosphere cells. The resulting tumors had sparse green signal by flow cytometry (Supplemental Figure 44), indicating that implanted CAFs were diluted out by proliferating tumor cells as the tumor reached end point, with few green cells identified by microscopy (Supplemental Figure 45).

Analyzing these tumors at end point revealed that the protumoral effects of GBM CAFs on the microenvironment noted in culture also occurred in vivo. Consistent with our findings with cultured GSCs grown in CAF CM, transcriptomic profiling by NanoString nCounter platform of tumors derived from GBM6 neurospheres grown alongside CAFs in vivo compared with GBM6 neurospheres grown without CAFs in vivo revealed upregulated HIF-1, EMT, and cell-proliferation pathway genes (Figure 7, B–D, and Supplemental Figure 46). Immunofluorescence of tumor vasculature labeled via rhodamine B-dextran perfusion revealed that CAFs caused GBM6 neurosphere-derived tumors to exhibit increased total vessel area/high-power field (hpf) (*n* = 3 mice/group; *P* = 0.02) (Figure 7, E and F) due to CAFs increasing the area of individual vessels (*P* = 0.0002; Figure 7F) without altering vessels/hpf (*P* = 0.3; Supplemental Figure 47). Moreover, flow cytometry revealed that CAFs increased the percentage of macrophages that were CD206^+^ M2 protumoral macrophages in GBM6 neurosphere-derived tumors (*P* = 0.0096; Figure 7G).

### Impact of GBM CAFs on patient survival.

To determine whether CAFs affected GBM patient survival similarly to the way in which adding CAFs worsened survival of mice carrying intracranial GSC-derived xenografts, we performed 2 analyses. First, we quantified the percentage of cells that were not epithelial cells, endothelial cells, pericytes, or immune cells, but did express at least 1 of 5 CAF markers in scRNA-Seq of 9 newly diagnosed GBMs (52). On average, 12.3% of cells (range = 0.4%–39.5%) in these tumors lacked non-CAF stromal markers, but expressed at least 1 of 5 CAF markers. Multivariate Cox’s regression accounting for age and sex revealed no correlation between survival and CAF levels identified in this manner (*P* = 0.4; Supplemental Table 5). Second, using The Cancer Genome Atlas (TCGA) data set, we found that survival of newly diagnosed GBM patients worsened when the tumor exhibited high expression of *ACTA2*, one of the most expressed CAF markers by our cultured cells (Supplemental Figure 2), combined with high expression of any of 5 other CAF markers (*FAP*, *PDPN*, *DES*, *THY1*, or *S100A4*) (*P* = 0.0007–0.02; Supplemental Figure 48).

## Discussion

GBMs derive much of their aggressive biology and treatment refractoriness from their microenvironment (2). Unlike with other tumors, it is currently unknown whether CAFs exist in GBM. The main argument for a lack of CAFs in GBM is that, apart from a small amount in blood vessels, there are no fibroblasts in the brain (4). However, because of evidence suggesting that CAFs in other tumors arise from marrow-derived precursors rather than usurping local fibroblasts (53–56), it seems plausible that CAFs could exist in GBM. Indeed, studies have identified cells expressing markers associated with CAFs in GBM (5–7), but gene expression profiling to prove these cells are CAFs and evidence for their role in GBM biology are lacking, a knowledge gap that our current study addresses.

We began by determining whether serial trypsinization of cells from GBM specimens, a method used to generate CAFs in other cancers (9), could isolate CAF-like cells. Trypsin detaches cultured cells from the culture dish through proteolysis of cell-surface integrins, and serial trypsinization takes advantage of the fact that primary tumor cells are less adherent and durable than CAFs. Morphologic analysis using our 3-class weighted classifier revealed 63% of these cells to be CAFs, and a negative selection strategy applied to scRNA-Seq revealed 52% of these cells to be CAFs. The difference between these values and the 79% of cells found to be CAFs when serial trypsinization was used in a murine lineage-tracing study (9) could reflect our analysis occurring in cells derived from human tissue and the stringency of our transcriptomic criteria.

Cells emerging from GBM serial trypsinization did not uniformly express CAF markers. Our pseudo-time reconstruction of scRNA-Seq data suggested early versus fully differentiated subtypes of GBM CAFs. Markers of late differentiation we identified in GBM CAFs such as *ACTA2* have been identified in late-stage differentiated breast CAFs, underpinning that CAFs undergo similar differentiation trajectories across cancer entities (28). We also found transcriptomic evidence within cells isolated by serial trypsinization of patient GBM of 3 CAF subtypes (steady state–like, mechanoresponsive, and immunomodulatory) conserved across multiple cancer types and species (29).

Among the unique proteins expressed by GBM CAFs was the EDA splice variant of FN. The EDA FN splice variant arises at the 11th type III repeat (EDA). FN expressing the EDA domain is termed cellular or oncofetal FN and has pivotal roles in wound healing, embryogenesis, and cancer (42). EDA containing FN is principally produced by fibroblasts, and in malignancy, CAFs are its source (42). In contrast, FN lacking splice variants is called plasma FN and is produced by hepatocytes and is not part of cancer pathogenesis (42). Our demonstration of tumor CAF-produced EDA containing FN promoting M2 macrophage polarization implicates EDA as not just a CAF biomarker, but a mediator of CAF-driven protumoral effects on the microenvironment

Systemic CAFs render the microenvironment more protumoral by recruiting monocytes and promoting their differentiation and polarization into M2 macrophages (57). We found similar effects of GBM CAFs, which drove M2 polarization of macrophages via TLR4, a receptor for CAF-produced EDA FN. The clinical impact of this level of M2 TAM enrichment is difficult to definitively determine, but combined with the angiogenesis changes we described, suggests that CAFs alter the GBM microenvironment through multiple mechanisms that combine with the direct effects we identified of CAFs on GSCs to create the robust effects of CAFs on GBM neurosphere–derived xenograft growth we identified in vivo.

Another effect we found that our CAF population exerted on the GBM microenvironment was on its microvasculature. GBM pathology is defined by 3 findings: proliferation of astrocytic neoplastic cells, tumor cell necrosis, and aberrant hypertrophied and glomeruloid microvasculature (58). Our finding that CAFs shift GBM vasculature to a larger, hypertrophied phenotype suggests that CAFs help establish this defining GBM feature. The unique architecture of GBM microvasculature has been postulated as an explanation for why GBMs are less responsive to antiangiogenic therapies such as bevacizumab. It would be interesting to determine whether CAFs play a role in this resistance by maintaining the unique vasculature of GBM.

Not only did we find an impact of GBM CAFs on the tumor vasculature, but we found using spatial transcriptomics that these cells were enriched in the perivascular niche, tumor regions bordering vessels. The GBM perivascular niche has garnered attention because it houses the GSCs whose recruitment of and nourishment by CAFs we demonstrated. CAF localization to the perivascular niche empowers CAFs to maintain and nourish GSCs, another rare cell type that also resides in the perivascular niche and contributes to GBM therapeutic resistance (34).

Our finding of regional variation in expression of markers associated with these GBM CAFs, with CAF-produced EDA more prevalent in the SVZ of GBM patients, but only when the SVZ contained tumor, will need verification. Patients whose GBMs contact the SVZ have shorter survival than patients with tumors outside the SVZ (59). While a study correlating survival differences between SVZ-involved GBMs and proteomic differences (60) suggested that GSC enrichment in the SVZ of SVZ-involved GBMs caused the poor prognosis of these patients, we did not find GSC enrichment in the SVZ of SVZ-involved GBMs. Further work is needed to confirm GBM CAF enrichment in tumor-bearing SVZ and define its mechanism.

Another area of uncertainty we explored is the lineage of this GBM CAF population we identified. Studies of CAFs in mouse models of other cancers have identified CAFs originating from local or remote sources. Local sources include fibroblasts (61), endothelial cells (62), or vascular mural cells (8) (pericytes on capillaries or smooth muscle cells on larger arteries). Importantly, despite sharing some markers, the cells we identified as GBM CAFs were transcriptomically distinct from pericytes and, while residing in the perivascular niche, were not as close to vessels as pericytes. In some cancers, endothelial PDGF-BB recruits pericytes onto angiogenic vessels by activating PDGFRβ, while tumor cell–derived PDGF-BB attracts pericytes to migrate from vessels through a chemoattractant mechanism with vessel-disassociated pericytes becoming CAFs (8). Further work will be needed to determine whether such a mechanism occurs in GBM, but our results with a PDGF-BB–blocking antibody implicate PDGF-BB in GBM CAF chemotaxis. The remote source of CAFs in the literature is MES stem cells (MSCs), multipotent stem cells in the bone marrow (53–56, 63), that could get to GBM because of breakdown of the blood-brain barrier (BBB) around GBM, a defining feature that allows recruitment of endothelial and myeloid progenitor cells derived from hematopoietic stem cells in the marrow for neovascularization (64) and establishment of TAMs (65), respectively. Such a source of CAFs would also be consistent with the distinct lineages identified by mito-SNV analysis of CAFs compared with the tumor cells and other stromal cells found in our serially trypsinized cultures.

Unfortunately, CAFs were rare enough in tissue-derived scRNA-Seq data that their low unique molecular identifier (UMI)/cell ratio precluded meaningful trajectory analysis and we were only able to apply this technique to cells isolated by serial trypsinization. That analysis yielded insights into early versus fully differentiated CAF subtypes and, along with our mito-SNV analysis, suggested that CAFs did not originate from other stromal cells isolated by serial trypsinization, but did not identify the more upstream origin of these cells. The question of whether GBM CAFs arise from BBB breakdown, allowing the recruitment of MSCs to GBM, which then differentiate into CAFs, or whether CAFs arise from a local cellular source will thus require further investigation.

Because our analysis relied on markers from non-GBM CAFs to identify GBM CAFs, we could have overlooked a population possessing functional properties ascribed to CAFs without expressing canonical CAF markers. The lack of ubiquitous CAF markers in GBM made it impossible to obtain a 100% pure population, which could have affected functional assays. The lack of these ubiquitous markers also limited the rigor of GBM CAF quantification and made it impossible to visualize these cells with immunohistochemistry, a problem that arises in other cancers (66).

Our identification of GBM CAF early versus late-differentiated subtypes from scRNA-Seq do not mean such data are a substitute for traditional lineage-tracing studies involving genetic labeling of a cell followed by tracking its offspring. Unfortunately, studying GBM CAFs in mouse models proved challenging because implanted murine GBMs do not produce CAF-like cells during serial trypsinization, suggesting that these cells are recruited to tumors that naturally form like human GBM and would best be studied in transgenic mice that naturally form GBMs, a potential area of future study.

Further work is needed to improve GBM CAF purification and to determine whether CAF metrics offer prognostic or therapeutic insights for GBM patients, as has been done for CAFs in other cancers (10). Overall, our findings provide compelling evidence that GBM CAFs promote GBM growth, insight that can be exploited for therapeutic benefit.

## Methods

### Cell culture.

GBM6 (Mayo Clinic), GBM43 (Mayo Clinic), DBTRG-05MG (ATCC), U-251 (ATCC), T98-G (ATCC), and LN-229 (ATCC) GBM cells; HUVEC cells (ATCC); THP-1 human monocytes (ATCC); and human astrocytes (ScienCell) were verified using short tandem repeat (STR) profiling, passaged under 6 times, and confirmed mycoplasma free. Breast CAFs were provided by the Breast Cancer Now Tissue Bank (London, United Kingdom). GBM cells were cultured in DMEM/F-12 plus 10% FBS and 1% penicillin/streptomycin at 37°C. HUVECs were grown in EGM-2 media (Lonza, catalog CC-3162). THP-1 cells were grown in complete RPMI with HEPES. Human astrocytes were grown in Gibco Astrocyte Medium (Thermo Fisher).

To generate GSC-containing neurospheres, GBM cells were grown in NM, consisting of DMEM/F12 (Gibco, Thermo Fisher Scientific) supplemented with 20 ng/ml EGF (Peprotech), 20 ng/mL bFGF (Peprotech), and 2% GEM21/neuroplex (Gemini Bio-Products). When comparing CAF_CM to NM, CAF_CM was generated by replacing the media of cultured CAFs with NM for 72 hours, after which media was collected and centrifuged at 300*g* for 5 minutes, followed by filtration through a 40 μm filter.

### Sample dissociation.

Tumor was finely chopped with sterile scalpels. Tumor chunks were suspended in papain at 37°C for 30 minutes and vortexed to assure good mixture. After this incubation, the solution was applied to a 50 μm filter and rinsed with culture media. Cells were centrifuged for 5 minutes at 500*g*. Media was aspirated, and cells were treated with 1 ml of ACK RBC Lysis Buffer (Lonza) for 2 minutes. RBC lysis was halted by adding 5 mL Dulbecco’s PBS (dPBS). The remaining cells were centrifuged for 5 minutes at 500*g*, ACK lysis buffer/dPBS was aspirated, and cells were resuspended in fresh dPBS and counted.

### Serial trypsinization to isolate CAFs.

To isolate and grow GBM CAFs in culture, the serial trypsinization method (9) was used in which dissociated GBM samples from 5 patients were cultured in DMEM/F12 with 10% FBS and 1% P/S. Cells underwent serial trypsinization with 0.25% trypsin-EDTA. Because primary tumor cells are less adherent than CAFs, we trypsinized for 30 seconds and discarded the supernatant, which had weakly adherent GBM cells, after which we trypsinized for 15 minutes to detach CAFs, which were then transferred to a fresh plate. This serial trypsinization yielded cells with fibroblast morphology within 5 weeks (~5 passages), designated GBMpt1CAF, GBMpt2CAF, GBMpt3CAF, GBMpt4CAF, and GBMpt5CAF based on their patient of origin (Supplemental Table 6) and maintained for under 9 passages. Normal murine or human brain did not yield cells when cultured like this.

### Human GBM tissue acquisition.

GBM tissue processed for scRNA-Seq was confirmed to have chromosome 7 gain and chromosome 10 loss through DNA analysis. Site-directed biopsies were obtained as in Supplemental Methods.

### Morphology analysis.

15,000 Cells/well were seeded in Permanox 2-chamber slides (MilliporeSigma, catalog C6682), incubated overnight at 37°C, stained with CytoTracker Green (Thermo Fisher, catalog C2925) supplemented media for 30 minutes, fixed using 4% paraformaldehyde in PBS (Thermo, catalog J19943-K2), and mounted in DAPI. Cells were imaged at 20× on a Zeiss Spinning Disc Confocal Microscope using ZEN Blue 2012 software. Images were segmented into blue and green channels. CellProfiler was used to identify nuclei as primary objects and cytoplasm as secondary objects. Propagation and watershed methods were used, with threshold equal to 0.5. VAMPIRE morphology analysis (11) as reported in Supplemental Methods. We designed a machine-learning binary logistic regression classifier utilizing breast CAF data (366 1997T and 499 2124T cells) and GBM data from GBM6 (803 cells), GBM43 (458 cells), and U-251 (797 cells) for training to achieve 91% nominal accuracy using a 70%/30% train/test split of 2,704 images.

### Neurosphere-formation assays.

For limiting dilution neurosphere-formation assays, GBM6 or GBM43 cells were seeded with increasing dilutions with 8 replicates in 96-well low attachment plates. Cells were allowed to form spheres for 7 days. After 7 days, the fraction of wells lacking neurospheres was quantified as reported in Supplemental Methods.

### NanoString multiplex transcriptomic analysis.

Using the RNeasy Mini Kit (QIAGEN), RNA was extracted from GBM6 neurospheres in NM or CAF_CM and GBM6 xenografts grown with or without CAFs. A bioanalyzer was used to determine quantity and quality of the RNA sample. RNA (150 ng) from each sample was hybridized with the NanoString PanCancer Progression code set for 18 hours, and 30 μL of the reaction was loaded into the nCounter cartridge and run on the nCounter SPRINT Profiler. Raw data were extracted, followed by quality control and alignment using the NanoString software.

### Cell proliferation assay.

GBM CAFs were plated at 1,000 cells per well in 96-well plates in NM or GSC CM. Proliferation was continuously assessed using the xCELLigence RTCA MP instrument (ACEA Biosciences); details are in Supplemental Methods.

### RNA Extraction.

RNA was extracted using RNeasy products (QIAGEN) and protocol applied to whole ex vivo samples or dissociated cells. Extracted RNA was stored at –80°C.

### Bulk RNA-Seq.

GBM CAF RNA libraries were prepared and Illumina HiSeq NGS performed (UCD Core, Davis, California, USA) per standard protocols. GBM CAF RNA-Seq data sets were aligned (BowTie2) and gene exons counted (FeatureCounts) with standard inputs using the Galaxy server (https://usegalaxy.org/). Data sets used are provided in Supplemental Methods. Differential gene expression, heatmap, and sample cluster were performed by iDEP8.1 (http://bioinformatics.sdstate.edu/idep/). For differential gene expression analysis, raw read counts were processed in iDEP using the limma-voom function. Batch effects were addressed by inclusion as a defined factor in the limma-voom function. The receptor-ligand analysis method is described in Supplemental Methods.

### scRNA-Seq.

scRNA-Seq used the chromium Next GEM Single Cell 3′ v3.1 protocol (10x Genomics). CAFs cultured by serial trypsinization were used for scRNA-Seq library preparation using the manufacturer’s protocol. Postlibrary preparation cells were sequenced using Illumina NovaSeq. Raw data were preprocessed using Cell Ranger to obtain matrix and count files, which, along with scRNA-Seq data from 12 patient GBMs (30, 31), were analyzed in R using scRNA-Seq Seurat 10x Genomics workflow. The PercentageFeatureSet function was used to filter out low-quality/dying cells with mitochondrial DNA (mt.percent >20%), and cells with less than 200 or more than 20,000 UMIs were also excluded. Data normalization used LogNormalize, a global-scaling normalization method. Nonlinear dimensional reduction was used to generate UMAPs to visualize data sets. The FindMarkers function was used to identify markers of clustered cells (Supplemental Methods). To identify CAFs, we used a CAF probability score based on negative expression of *PTPRC*, *EPCAM*, *PECAM1*, *CSPG4*, and *RGS5* and their degree of expression of CAF markers *ACTA2*, *FAP*, *PDGFRA*, *PDGFRB*, *VIM*, *PDPN*, *S100A4*, *TNC*, and *COL1A1*:


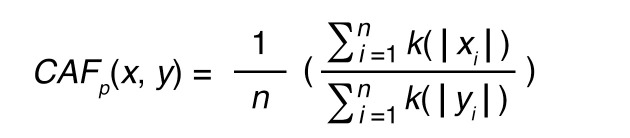


 (Equation 1)

with k defined as a Gaussian kernel:


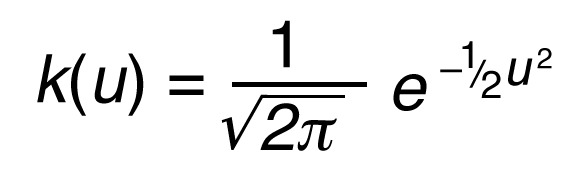


 (Equation 2)

where x indicates the positive marker gene set, y the set of non-CAF genes, p the CAF probability score, n the total number of genes in the score, i the number of the genes used in the equation, u the gene expression value, and e the exponential constant.

### CNV and SNV analysis.

Copy number alterations were estimated with the CopyKat package using the following parameters: rawdata, id.type = “S”, ngene.chr = 5, win.size = 25, and KS.cut = 0.1. BAM files from the cellranger output were used for mito-SNV detection. We used the cellSNP algorithm with the following input parameters: cellSNP -s $BAM -b $BARCODE -O $OUT_DIR -p 22 --minMAF 0.1 --minCOUNT 100 –chrom M. The resulting.vcf file was imported into R using the vcfR package. We removed low-quality SNV annotations and SNVs detected in fewer than 100 cells. The resulting genotype matrix was used for hierarchical clustering. Visualization was performed using the oncoplot tool from vcfR.

### Spatial transcriptomics.

Integrated scRNA-Seq and spatially resolved transcriptomics from 16 newly diagnosed IDH-WT GBMs (32) were analyzed using SPATA2 (https://themilolab.github.io/SPATA2/) including the wrapper functions in the SPATAwrappers package (https://github.com/heilandd/SPATAwrappers). Computation of proximity analysis was performed using the SPATAwrappers:inferJuxtaposition() and visualized by SPATAwrappers:plotJuxtaposition().

### Pseudotime analysis.

We learned trajectory graphs and performed pseudotime analysis using the Monocle 3 (learn-trajectory) function. First, we isolated CAFs by their CAF probability scores. An NMF for dimensional reduction was performed on CAFs (SPATAwrappers:runNMF()). The SPATA object was transformed to a CDS object using the SPATA2:transformSpataToCDS() function. Next, we performed clustering and dimensional reduction (UMAP) in the Monocle package (NMF as dimensional reduction). To infer single-cell–directed differentiation, we estimated single-cell vector fields. In the dimension reduction determined space, the pseudotemporal state of each point can be represented as a vector (*x*). We computed the changes as a vector within vector field *f*, which is composed of the coordinates *x* in the d-dimensional space of all spots, leading to a vector *v* in the same space, i.e., *v* = *f*(*x*). This was performed by the SPATAwrappers:inferVectorFields() function and visualized by the SPATAwrappers:plotVectorFields() function.

### qPCR.

cDNA was created using qScript XLT cDNA Supermix (Quantabio) per the manufacturer’s protocol. qPCR was carried out using Power SYBR Green Master Mix (Applied Biosystems) and primers (Supplemental Table 7) in an Applied Biosystems StepOne Real-Time PCR cycler: 95°C (10 minutes), followed by 40 cycles of 95°C (15 seconds) and 60°C (1 minutes). Ct values were calculated using StepOne software accompanying the real-time cycler.

### Immunofluorescence.

Tissues were fixed in 4% paraformaldehyde for 24 hours, transferred to 30% sucrose for 20 hours, embedded in OCT (Fisher Scientific), and frozen at –80°C; 10 μm thick slices were rinsed with PBS followed by blocking in 5% serum, 2% BSA, and 0.3% Triton X-100 in PBS. Slides were incubated in primary antibodies at 4°C overnight, rinsed with PBS, incubated in secondary antibody for 2 hours, and mounted with DAPI mounting media. Sections were imaged using a Zeiss M1 fluorescent microscope. Images were processed using Fiji’s ImageJ software. Antibodies used are listed in Supplemental Table 8. Measuring vessel area and CAF tracking are described in Supplemental Methods.

### Flow cytometry and FACS.

Samples were prepared via manual mechanical separation and papain digestion. RBC lysis was performed. Samples were resuspended in DMEM, pelleted, and resuspended in FACS buffer with Fc-block (Human Seroblock, Bio-Rad). Samples were repelleted and suspended in fluorophore-conjugated primary antibodies (Supplemental Table 8). After incubation at 4°C, samples were rinsed 3 times in FACS buffer and suspended in FACS buffer for sorting with FACSAria III (BD Biosciences). Living single cells were selected via forward scatter/side scatter isolation.

### Invasion assays.

Invasion assays were completed using Matrigel (Corning) solution on Boyden chamber membranes per the manufacturer’s protocol. Test medium was placed at the bottom of the Boyden chambers, and invading cells were placed on the other surface. After 24 hours, noninvading cells were washed away, and cells per hpf (×40 magnification) were quantified via DAPI staining.

### Angiogenesis assay in culture.

Geltrex LDEV-Free Reduced Growth Factor Basement Membrane Matrix (Thermo Fisher, catalogA1413202) was thawed overnight at 4°C and plated (120 μL) into 48-well tissue culture plates (Corning, catalog 353078), with plates tapped to spread the Geltrex, and incubated at 37°C for 30 minutes. 40,000 HUVEC cells in EGM-2 with hydrocortisone, ascorbic acid, GA-1000, and heparin without growth factors were added to wells, and 100 μL of each condition of medium was added to wells. Plates were tilted in all directions to distribute cells. After 3.5 hours, 100 μL of EGM-2 was added with 1.5 μL of 1 mg/mL calcein-am (Thermo Fisher, catalog C1430). Imaging and image processing are described in Supplemental Methods.

### Macrophage studies.

THP-1 cells and monocytes isolated from peripheral blood (AllCells) run through the MojoSort Human CD14 Selection Kit were treated with 50 ng/μL phorbol myristate acetate (PMA) for 4 days to allow cell adhesion to the plate and differentiation into resting M0 macrophages, which were incubated with 20 ng/mL IFN-*γ* (for M1 polarization) or 20 ng/mL IL-4 (for M2 polarization) or treated under experimental conditions. Assessment of growth on FN, polarization, and proliferation are described in Supplemental Methods.

### Murine intracranial xenografts.

Either 40,000 or 100,000 GBM cells grown as neurospheres or 35,000 GBM cells grown as neurospheres mixed with 5,000 GBM CAFs were implanted stereotactically into the right frontal lobes of athymic mice (6 to 8 weeks, female). Because 40,000 GBM6 neurosphere cells alone did not form tumors, to obtain control tissue, 100,000 GBM6 neurosphere cells were implanted in another cohort.

### Data and code availability.

Sequencing data have been deposited in the National Center for Biotechnology Information Gene Expression Omnibus (GEO GSE132825). Cell morphology analysis script is at https://github.com/alexanderchang1/GBM_CAF_open/tree/7f252ef0f73cff182aee6962fde4765769871f2f (commit ID: 7f252ef0f73cff182aee6962fde4765769871f2f).

### Statistics.

Invasion, cell proliferation, neurosphere formation, and qPCR assays were done with 3 technical and biological replicates. To compare multiple groups, ANOVA (parametric) or Kruskal-Wallis (nonparametric) tests were used for continuous outcome variables, while χ^2^ and Fisher’s exact tests were used for categorical outcome variables. ANOVA or Kruskal-Wallis tests were followed by Tukey’s or pairwise Wilcoxon’s post hoc tests for comparisons between groups, respectively. The nonparametric 2-tailed *t* test was used to compare 2 groups. NanoString data were analyzed using the DESeq2 package in R, which carries out an internal normalization where a geometric mean is calculated for each gene across replicates and counts in each replicate are then divided by the mean, with count outliers removed using Cook’s distance analysis and Wald’s test used to assess significance. Kaplan-Meier analysis was carried out for in vivo survival studies. scRNA-Seq analysis used the Seurat workflow. Horizontal lines are at the median for dot plots. Population bioinformatics is described in Supplemental Methods.

### Study approval.

Animal and human tissue experiments were approved by the UCSF IACUC (AN105170-02) and IRB (11-06160).

## Author contributions

SJ and JWR designed the project, conducted experiments, processed data, interpreted results, and edited the manuscript. RJ, AB, JS, ACCC, NC, ATN, SS, EC, JSC, AC, AFH, HW, SSS, SC, JLH, LW, SG, and GY conducted experiments, processed data, and interpreted results. JFC and AD interpreted results. DHH designed experiments, interpreted results, and wrote and edited the manuscript. MKA procured funding, designed experiments, interpreted results, and wrote and edited the manuscript.

## Supplementary Material

Supplemental data

Supplemental table 1

Supplemental table 2

Supplemental table 3

Supplemental table 4

Supplemental tables 5-8

## Version 1 03/01/2023

Electronic publication

## Version 2 03/09/2023

Added graphical abstract
