# Supplementary material for: Single-cell RNA sequencing and spatial transcriptomics reveal cancer-associated fibroblasts in glioblastoma with protumoral effects
Source: J Clin Invest. 2023 Mar 1;133(5):e147087. doi: 10.1172/JCI147087 (PMC9974099; doi:10.1172/JCI147087)
Supplement: Supplemental data [file jci-133-147087-s010.pdf]

SUPPLEMENTAL METHODS

Additional information on morphology analysis

Morphology analysis was done with the VAMPIRE package (1) with 100 coordinates and 100 shape modes as the settings and corresponding data was fed into our logistic regression. Segmented nuclei and cytoplasm were fed into the VAMPIRE pipeline to calculate their unique morphological features such as circularity. By pairing nuclei and cytoplasm datasets by cell in Python, we generated a 16 data-point profile for each cell.

A multiclass weighted classifier was developed by adding data from 148 human astrocyte cells to the binary logistic regression classifier for training to achieve nominal accuracy of 76% using a 70%/30% train/test split of 3071 cells. For testing, 159 cells with serial trypsinization and 1187 cells without serial trypsinization were assessed. The multiclass weighted classifier was additionally validated using T98-G (283 cells) and LN-229 (121 cells).

Bulk RNA-sequencing additional information

iPSC-Pericyte (GSE117469 and GSM2790558) (2), dermal fibroblast (GSM3124683) (3), and breast CAF (GSE106503) (4) RNA-seq results from GEO and RNA-seq results from seven types of normal human fibroblasts were provided by Susan Thibeault (University of Wisconsin) (5). To infer receptor-ligand interactions between GBM CAFs and GBM6 neurospheres, we compared RNA-seq data from two GBM CAFs and GBM6 neurospheres (6) to a database of 491 receptor-ligand interactions (7). Then we annotated cognate pairs co-expressed by GBM CAFs and GSCs for which the FPKM of

the ligand is >0.05 and read counts of the receptor is >10. This produced 189 GSC ligands with receptors expressed by CAFs and 174 CAF ligands with receptors expressed by GSCs.

scRNA-sequencing additional information

We identified cell types using the following markers: GFAP (glia); RBFOX3/THY1 (neurons); OLIG1 (Oligodendrocytes); PTPRC (immune cells); CD3D (T-cells); TMEM19/CX3CR1/P2RY12 (microglia-non-activated); CD163/HLA-DR/SPP1/CCL2 (M2-myeloid cells); EPCAM (epithelial cells); PECAM1/CD34/CDH5 (endothelial cells); CSPG4/RGS5 (pericytes); CDK4 (NPC-like GBM); EGFR/PDGFRA (OPC-like GBM); and NF1/TME (AC-like GBM). Astrocyte probability score was derived based on degree of expression of ten astrocyte markers from the Azimuth human cortex data (https://azimuth.hubmapconsortium.org/references/human_motorcortex/): *SLC1A2*, *ADGRV1*, *SLC1A3*, *GPC5*, *RNF219.AS1*, *ARHGAP24*, *CST3*, *HPSE2*, *AQP4*, and *COL5A3*. For CAF subtype annotations and comparison with published data (8), we built scores for three subtypes using the following genes: immunomodulatory (IM) - *CXCL12, DCN, STAT1* and *SPON2*; mechanoresponsive (MR) – *COL6A1, POSTN, COL4A1, PDGFRB, MGP,* and *FOSB*; and steady state-like (SSL) - *FAP* and *TIMP2*.

Spatial transcriptomics additional information

Integration of single cell analysis and spatially resolved transcriptomics was performed by the spotlight algorithm through seeded NMF regression (9, 10). Integration was performed by the wrapper function *SPATAwrappers::inferSpotlight()*.

Quantifying Neurosphere Formation

ELDA software (11) was used to perform limiting dilution analysis under the assumption that a single cell can generate a sphere and that inter-well variation follows a Poisson distribution. Per the Poisson distribution, GSC frequency was defined as the reciprocal of the slope when log transformed of the negative fractions was plotted against seeding density. To compare the number of spheres formed, GBM6 or GBM43 cells were seeded at increasing dilutions.in triplicate in 12-well low attachment plates with neurosphere media or CAF_CM. We assessed sphere formation after 7 days for GBM6 cells and 5 days for GBM43 cells. Spheres were imaged under 100x magnification using a Nikon D90 mounted on a Nikon Eclipse TS100 microscope, with 15 high power fields (hpf) from each condition analyzed using ImageJ. The total number of spheres were counted. To determine the effect of CAF_CM on neurosphere luminescence or area, 5,000 or 10,000 GBM6 cells or 1,000 GBM43 cells were seeded in triplicate in a 12-well low attachment plate with either neurosphere media or CAF_CM. For cells expressing luciferase, we assessed bioluminescence after 72 hours. For other cells, we imaged at 72 hours under 100x magnification using a Nikon D90 mounted on a Nikon Eclipse TS100 microscope, with 15 high power fields (hpf) from each condition analyzed using ImageJ. ROI manager function was used to measure the total area and diameter of neurospheres.

Cell Proliferation Assay Details

Proliferation was continuously assessed using the xCELLigence RTCA MP instrument (ACEA Biosciences) to measure impedance as a surrogate for cell count over 120 hours

(12). First, 50 μL of media was added to each well of 96 well E-Plates (ACEA Biosciences) and background impedance was measured and displayed as Cell Index. Dissociated adherent GBM CAF cells were seeded at 1000, 3000, 5000, or 7000 cells/well of the E-Plate in a volume of 100 μL and allowed to passively adhere on the electrode surface. After seeding, the E-Plate was kept at ambient temperature inside a laminar flow hood for 30 minutes and then transferred to the RTCA MP instrument inside a cell culture incubator. Data recording was initiated immediately at 15-minute intervals for the duration of the experiment.

Imaging and Processing Cell Culture Angiogenesis Data

Cells were imaged 30 minutes later at 4-, 8-, 16-, and 24-hours. Cells were imaged at 2.5x on a Zeiss Spinning Disc Confocal microscope using ZEN Blue 2012 (Carl Zeiss) software, with full z-stacks and stitching to capture the entire well. Images were processed to produce a Max Intensity Z-projection that was analyzed using the ImageJ Angiogenesis Analyzer package (http://image.bio.methods.free.fr/ImageJ/?Angiogenesis-Analyzer-for-ImageJ&lang=en#outil_sommaire_0). Default software settings were used with the exception of not suppressing isolated elements. For cell culture assays, we normalized and averaged expansion (Nb extrem. /Nb branches / Tot. branches length), extension (Tot. master segments length / Tot. length / Tot. branching length / Tot. segments length), and fusion (Nb Junctions / Nb master junction / Nb master segments / Nb meshes / Nb pieces / Nb segment) metrics. Each datapoint was normalized to control.

Macrophage Experiments - Additional Details

Macrophages underwent qPCR analysis of expression of three M1 (*NOS2*, *CXCL10*, and *IL1B*) and three M2 genes *(ARG1*, *TGFB1*, and *MMP9*), from which we derived an M2/M1 ratio of the expression of the three M2 markers divided by expression of the three M1 markers (13). To assess macrophage proliferation, cells were plated into black 96-well clear bottom plates and analyzed using CyQuant cell proliferation assay kits (Thermo Scientific, c7026). To culture macrophages on fibronectin, after rinsing six-well plates with saline, non-EDA (R and D systems; #1918-FN-02M) and EDA fibronectin (ScienCell; #8488) suspended in PBS were placed in the wells at 5mg/cm^2. These were stored overnight at 4°C to allow binding. PBS was aspirated. Macrophages in their media were added to the wells, cultured for 48 hours, and then assessed.

Human GBM Tissue Acquisition Details

Site-directed biopsies using BrainLab^{TM} MRI guidance were obtained from: (1) tumor core; (2) tumor leading edge, the outer edge of the tumor enhancement on MRI; (3) peri-tumoral brain zone (PBZ); the FLAIR bright region outside gadolinium enhancement on MRI (14); and (4) SVZ when GBMs invaded this area. Additional SVZ samples were obtained from (1) patients undergoing epilepsy surgery and (2) autopsies of GBM patients without SVZ tumor.

Measuring Vessel Area

Images of rhodamine-perfused vessels taken on a Zeiss Spinning Disk confocal microscope at 20x magnification were analyzed using QPath software. Within a single channel, thresholds were set to exclude background signal and to create boundaries

around rhodamine-positive vascular structures. The same thresholding parameters were used for each high-powered field across experimental groups. Areas of bounded structures were calculated and used as a metric for vessel area.

CAF Tracking

For CAF tracking, 5000 CAFs expressing GFP after lentiviral transduction and 35000 GBM43 cells were stereotactically injected in mice brain. At euthanasia, we carried out rhodamine B-dextran perfusion to stain the blood vessels before harvesting the brain. Images of GFP expressing CAFs along with the rhodamine-perfused vessels were taken on a Zeiss Spinning Disk confocal microscope at 20x magnification DiAna, an ImageJ tool (15), was used to measure the distance between GFP-labeled CAFs and rhodadmine perfused vessels.

Population-based Bioinformatics

Data from the U133A array of the TCGA_GBM dataset was obtained from GlioVis (gliovis.bioinfo.cnio.es) with analyses performed using Gliovis statistical tools. To determine the impact of dual expression of CAF markers on prognosis, TCGA GBM data was downloaded from the Broad GDAC Firehose website (http://gdac.broadinstitute.org/) and analyzed in RStudio (RStudio, Inc., v1.2.1335). Gene expression levels as RSEM scaled estimates were multiplied by 10^6 to convert to transcripts per million (TPM) and $\log_2$-transformed. The transcript abundance of a pair of CAF marker genes was quantified by calculating the geometric mean expression. A threshold for stratifying patients into "high" or "low" expression of a CAF marker gene pair was calculated using the maxstat R

package (v0.7.25) to perform maximally selected rank statistics. After dichotomizing the cohort at the threshold, Kaplan-Meier survival curves were constructed for each stratum using the survminer R package (v0.4.9) and compared via the log-rank test.

REFERENCES FOR SUPPLEMENTAL METHODS

1. Wu P-H, et al. Evolution of cellular morpho-phenotypes in cancer metastasis. *Scientific Reports.* 2015;5(1):18437.
2. Wimmer RA, et al. Human blood vessel organoids as a model of diabetic vasculopathy. *Nature.* 2019;565(7740):505-10.
3. Fleischer JG, et al. Predicting age from the transcriptome of human dermal fibroblasts. *Genome Biol.* 2018;19(1):221.
4. Yan W, et al. Cancer-cell-secreted exosomal miR-105 promotes tumour growth through the MYC-dependent metabolic reprogramming of stromal cells. *Nat Cell Biol.* 2018;20(5):597-609.
5. Foote AG, et al. Tissue specific human fibroblast differential expression based on RNAsequencing analysis. *BMC Genomics.* 2019;20(1):308.
6. Zhou D, et al. Distinctive epigenomes characterize glioma stem cells and their response to differentiation cues. *Genome Biol.* 2018;19(1):43.
7. Graeber TG, and Eisenberg D. Bioinformatic identification of potential autocrine signaling loops in cancers from gene expression profiles. *Nat Genet.* 2001;29(3):295-300.
8. Foster DS, et al. Multiomic analysis reveals conservation of cancer-associated fibroblast phenotypes across species and tissue of origin. *Cancer Cell.* 2022;40(11):1392-406 e7.
9. Elosua-Bayes M, et al. SPOTlight: seeded NMF regression to deconvolute spatial transcriptomics spots with single-cell transcriptomes. *Nucleic Acids Res.* 2021;49(9):e50.
10. Ravi VM, et al. Lineage and Spatial Mapping of Glioblastoma-associated Immunity. *bioRxiv.* 2021:2020.06.01.121467.
11. Hu Y, and Smyth GK. ELDA: extreme limiting dilution analysis for comparing depleted and enriched populations in stem cell and other assays. *J Immunol Methods.* 2009;347(1-2):70-8.
12. Ke N, et al. The xCELLigence system for real-time and label-free monitoring of cell viability. *Methods Mol Biol.* 2011;740:33-43.
13. Castro BA, et al. Macrophage migration inhibitory factor downregulation: a novel mechanism of resistance to anti-angiogenic therapy. *Oncogene.* 2017;36(26):3749-59.
14. Lemee JM, et al. Intratumoral heterogeneity in glioblastoma: don't forget the peritumoral brain zone. *Neuro Oncol.* 2015;17(10):1322-32.
15. Gilles JF, et al. DiAna, an ImageJ tool for object-based 3D co-localization and distance analysis. *Methods.* 2017;115:55-64.

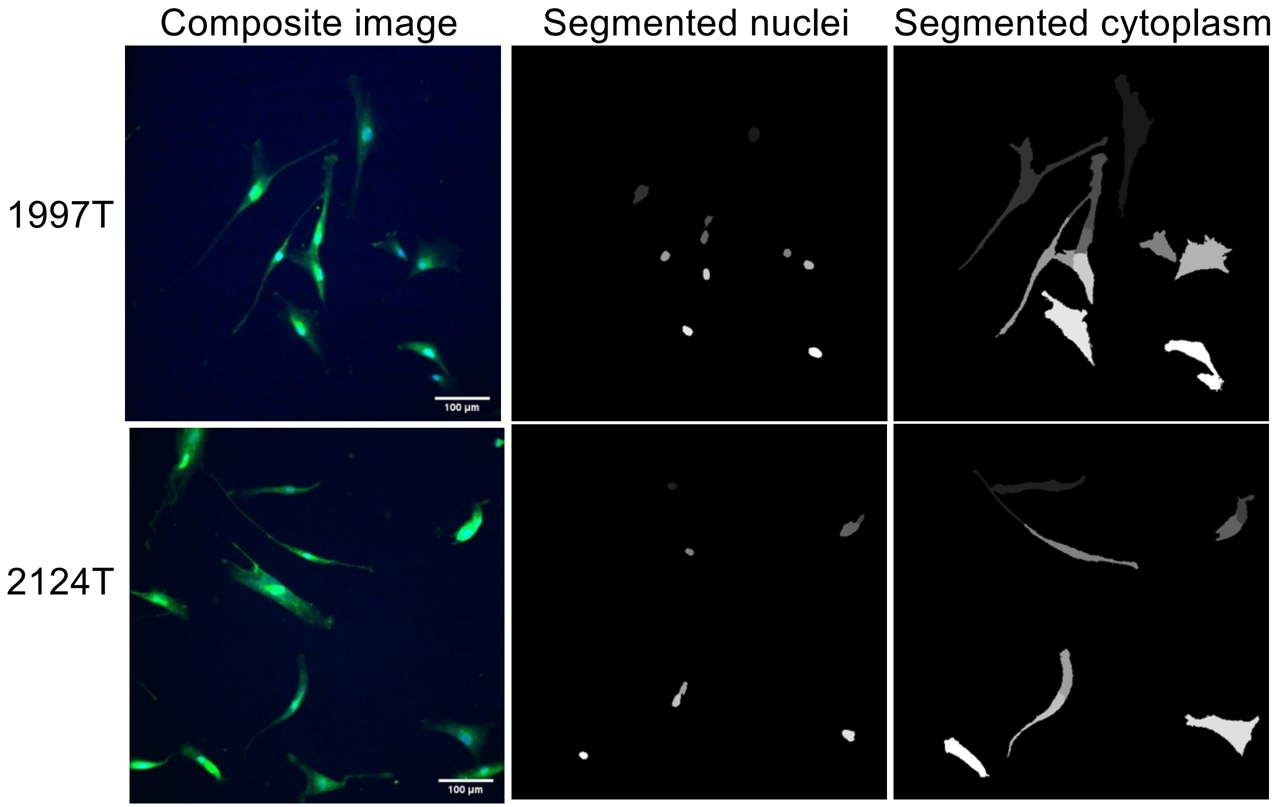

Supplemental Figure 1. Representative segmented images from breast cancer CAFs. Related to Figures 1A-B. Breast cancer CAF cells 1997T and 2124T were imaged (left) using CellTracker (green) and DAPI (blue). Segmentation was performed using ImageJ to split images into blue (nuclei, middle) and green (cytoplasm) channels. Segmented images were analyzed for morphology and results were used to generate a classifier, as described in the Methods section. 10x magnification; scale bar 100 μm.

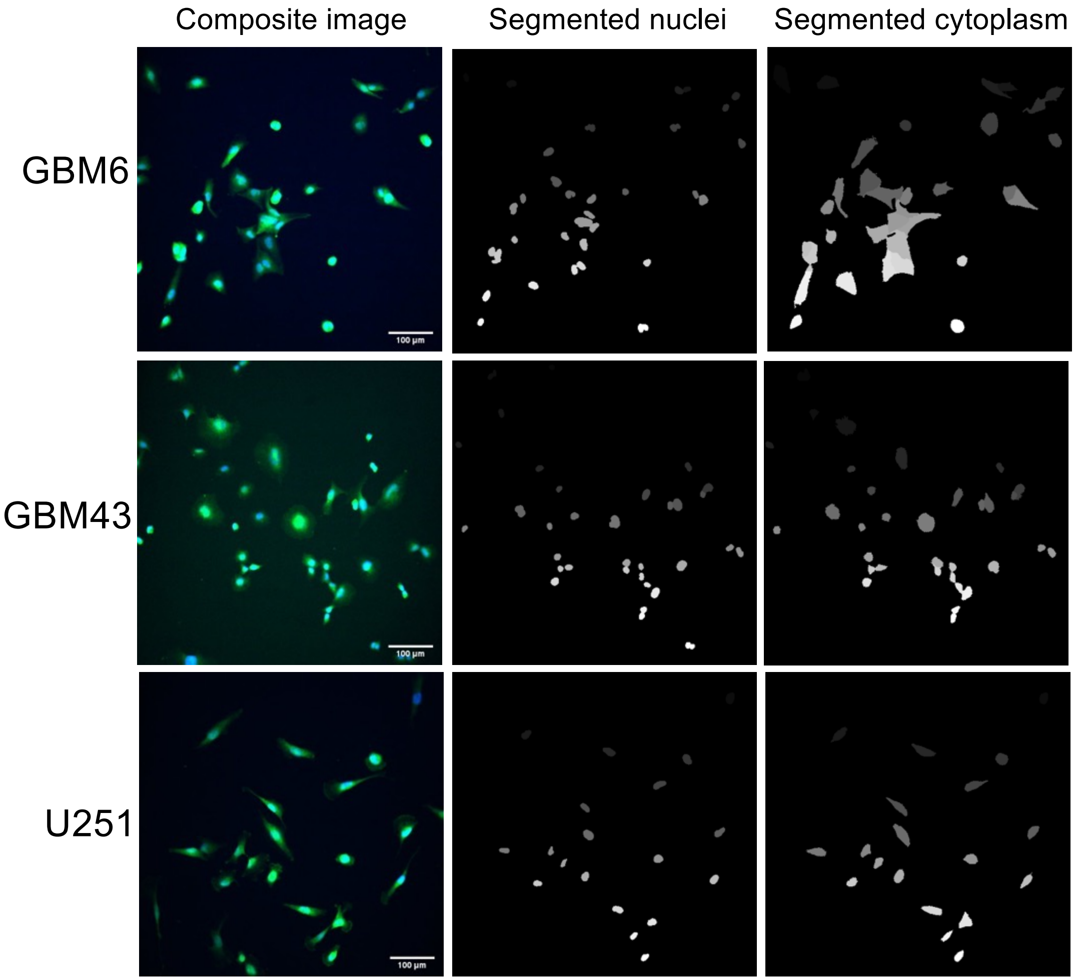

Supplemental Figure 2. Representative segmented images from GBM cells. Related to Figures 1A-B. GBM cells GBM6, GBM43, and U251 were imaged (left) using CellTracker (green) and DAPI (blue). Segmentation was performed using ImageJ to split images into blue (nuclei, middle) and green (cytoplasm, right) channels. Segmented images were analyzed for morphology and results were used to generate a classifier, as described in the Methods section. 10x magnification; scale bar 100 μm.

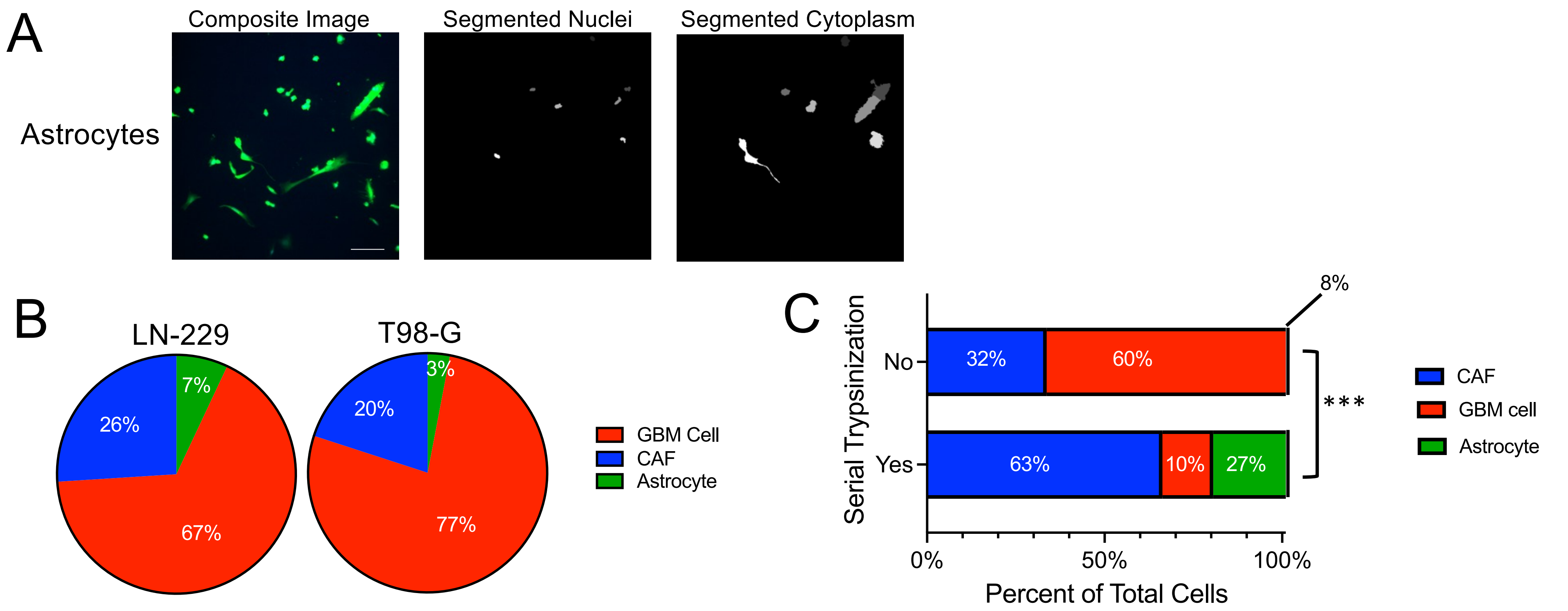

Supplemental Figure 3. Serial trypsinization of cells isolated from GBM enriches cells that are morphologically fibroblasts when classified via a three-class weighted regression. Related to Figures 1A-B. (A) Shown are images of cultured human astrocytes (10x magnification, 100 mm scale bar) incorporated into our binary (CAF/GBM cell) classification to develop a three-class weighted regression to classify cell morphology as fibroblast, GBM tumor cell, or astrocyte. (**B**) When this regression was applied to two GBM tumor cell lines (T98-G and LN-229) that were different than the GBM6, GBM43, and U251 tumor cells used to create the regression, both T98 and LN229 were designated as mostly GBM cells. (**C**) When this regression was applied to cells from the same patient GBM that were processed with or without serial trypsinization, serial trypsinization was found to enrich the GBM CAF population to 63% ($P<0.001$; Chi-squared), close to the level seen when these cells underwent transcriptomic analysis.

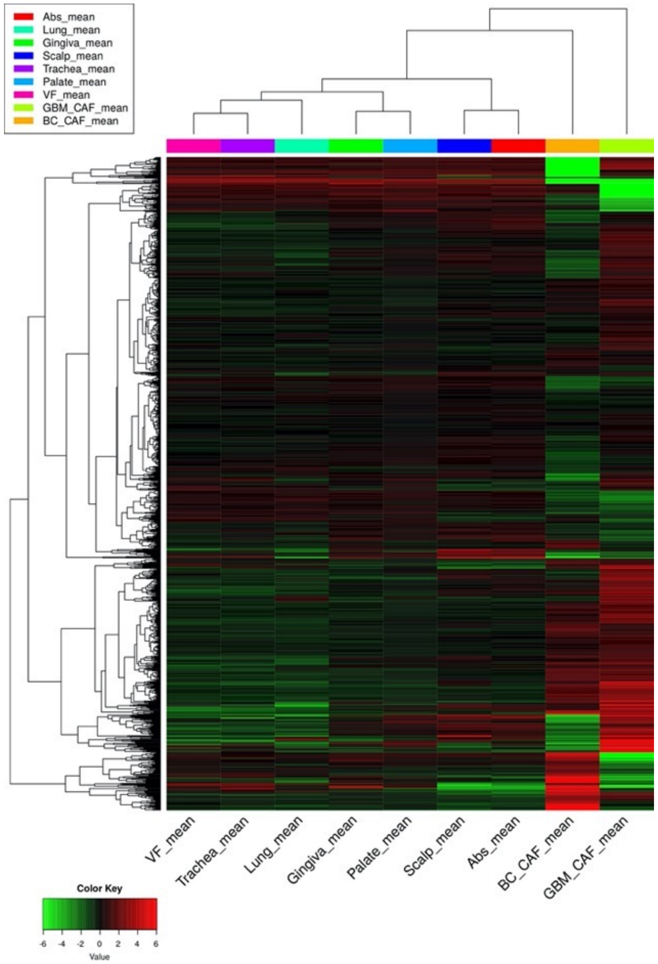

Supplemental Figure 4. Transcriptomic profiling reveals that cells that emerge from serial trypsinization of primary GBMs resemble CAFs from other cancers. Related to Figure 1C. RNA-seq was performed on cells isolated from GBM after serial trypsinization (n=1 case shown here from the two cases shown in **Fig. 1C**) which were then compared to archived RNA-seq results form breast cancer CAFs (n=1 case shown here from the two cases shown in **Fig. 1C**) and normal fibroblasts from seven different tissues (scalp dermis, soft palate, upper gingiva, vocal fold, trachea, lung, and abdomen dermis). Cells isolated from GBM after serial trypsinization more closely resembled breast cancer CAFs than normal fibroblasts. Of the 7 normal fibroblasts shown here, the GBM cells and breast cancer CAFs most closely resembled fibroblasts from abdomen dermis and scalp.

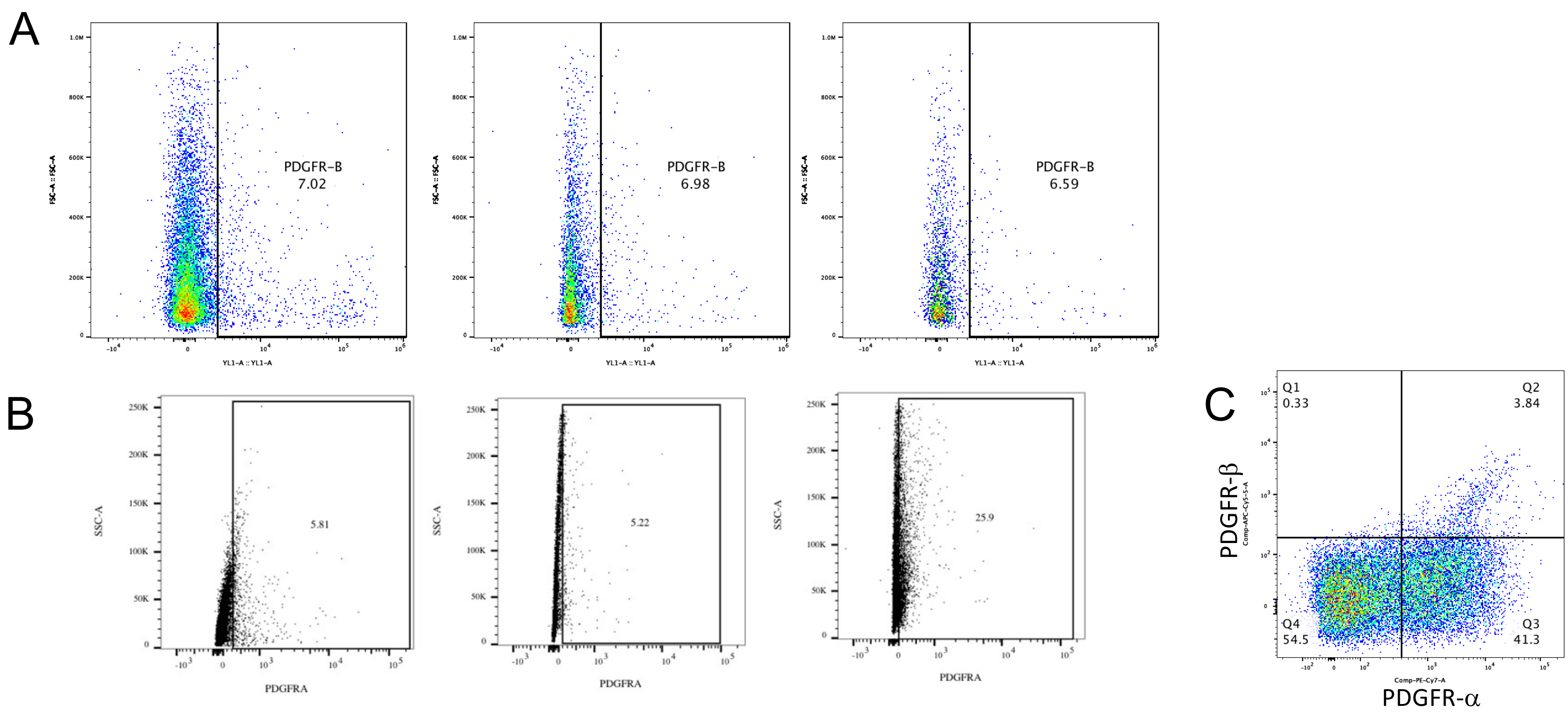

Supplemental Figure 5. Flow cytometry of cells reveals no ubiquitous CAF markers. Related to Figure 1D. (**A**) Flow cytometry of GBMpt1CAF cells isolated from GBM after serial trypsinization reveals expression of CAF marker PDGFR-β in 6.59-7.02% of cells (n=3 cases). (**B**) Flow cytometry of cells isolated from GBM after serial trypsinization reveals expression of CAF marker PDGFR-α in 5.22-25.9% of cells (n=3 cases). (**C**) Co-staining of a case of cells isolated from GBM after serial trypsinization reveals low levels of PDGFR-β alongside high levels of PDGFR-α in this case but with most of the PDGFR-β positivity occurring in PDGFR-α positive cells

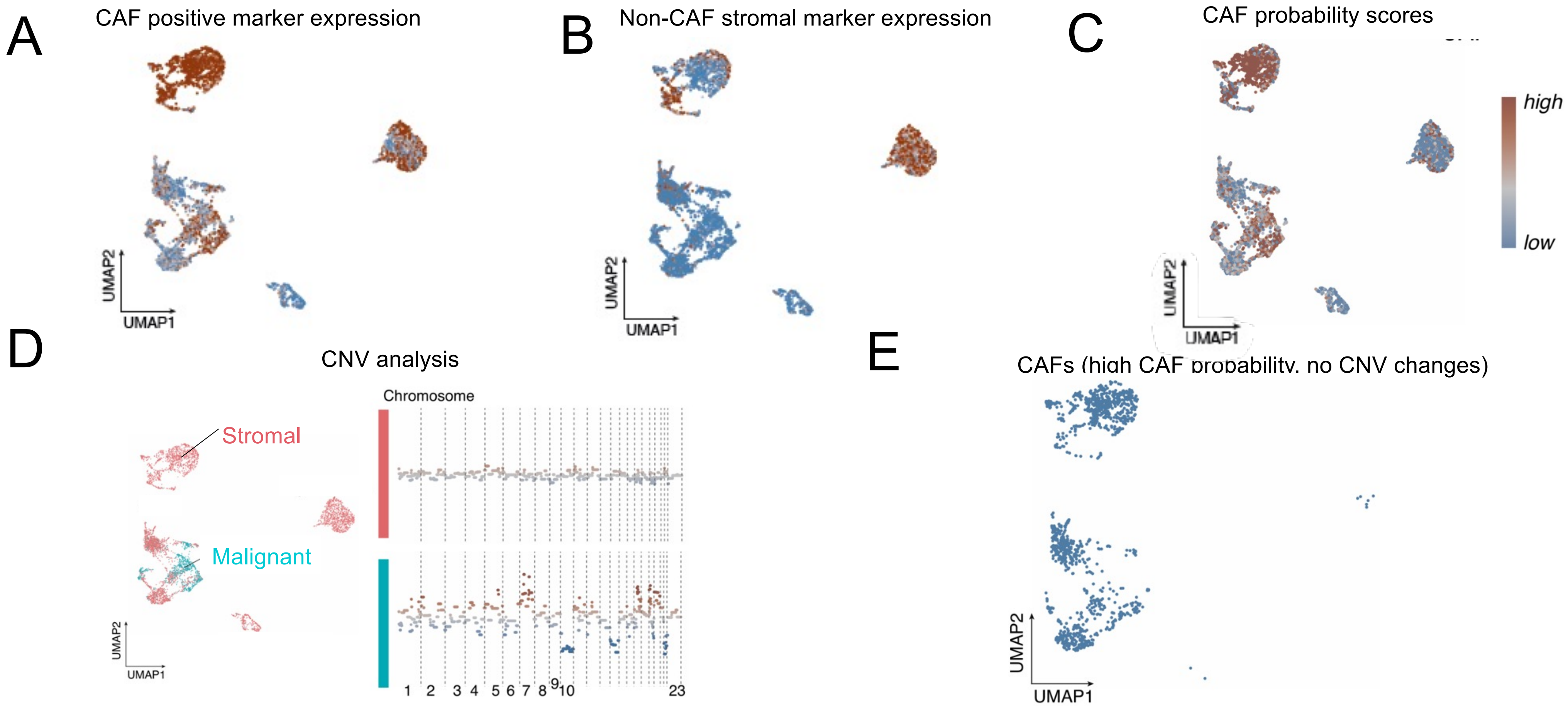

Supplemental Figure 6. Identifying CAFs in scRNA-seq of cells isolated from serial trypsinization of patient GBMs. Related to Figs. 1D-F. UMAP plot of cells isolated by serial trypsinization from patient GBM was overlaid with (**A**) CAF marker expression; (**B**) non-CAF stromal marker expression; (**C**) CAF probability scores derived by subtracting non-CAF stromal marker expression in (B) from CAF marker expression in (A); (**D**) CNV analysis revealing stromal cells (red) without CNV changes and tumor cells (cyan) with CNV changes, including gain of chromosome 7 and loss of chromosome 10 commonly seen in GBM; and (**E**) CAF cells in dark blue identified by taking cells with high CAF probability scores in (C) and removing cells with CNV changes in (D).

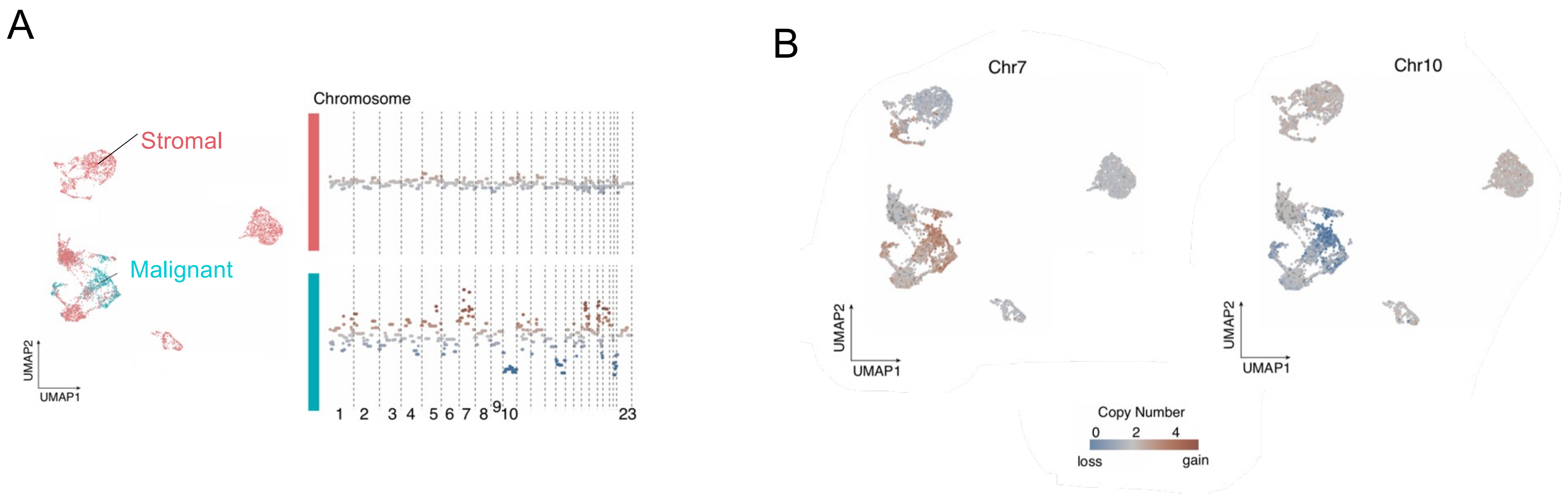

Supplemental Figure 7. Copy-number variation (CNV) analysis of cells isolated from serial trypsinization of patient GBMs. Related to Figures 1D-F. (A) UMAP plot of cells isolated by serial trypsizination from patient GBM was overlaid with CNV analysis which produced chromosome histogram (right), leading to identification of malignant cells exhibiting copy number alterations (cyan) and stromal cells without copy number changes (red). (**B**) Shown are CNV maps for chromosome 7 (left) and choromsome 10 (right), revealing gain of chromosome 7 and loss of chromosome 10 as the specific chromosomal changes in malignant cells.

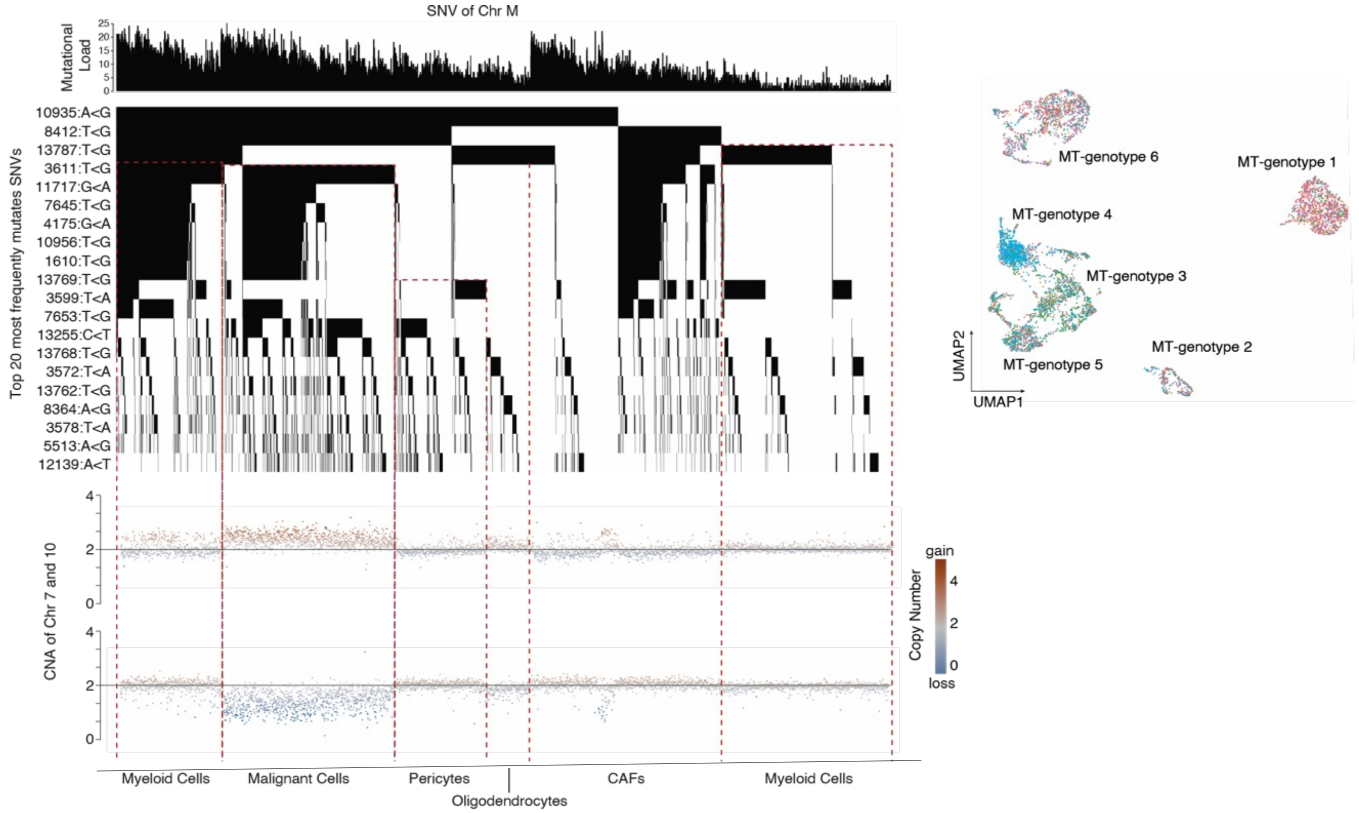

Supplemental Figure 8. Mitochondrial SNV reveals CAFs to have a distinct origin from other cells arising from serial trypsinization of patient GBMs. Related to Figures 1D-F. Shown is a waterfall plot of mitochondrial SNV of cells isolated by serial trypsinization of patient GBM, with mutational load illustrated on top. The 20 most variant SNVs are illustrated in a onco-print in which every column represents a cell with its correspondent mutational profile. This analysis revealed six mitochondrial genotypes (shown in the UMAP plot to the right). Chromosomal gains of Chr 7 and losses of Chr 10 for each cell (bottom left) helped identify tumor cells, with gene expression profile used to characterize remaining cell types as labeled on the bottom. The distinct mitochondrial genotype of each of these cell types revealed that CAFs had a distinct lineage from tumor cells or other stromal cells identified in this analysis.

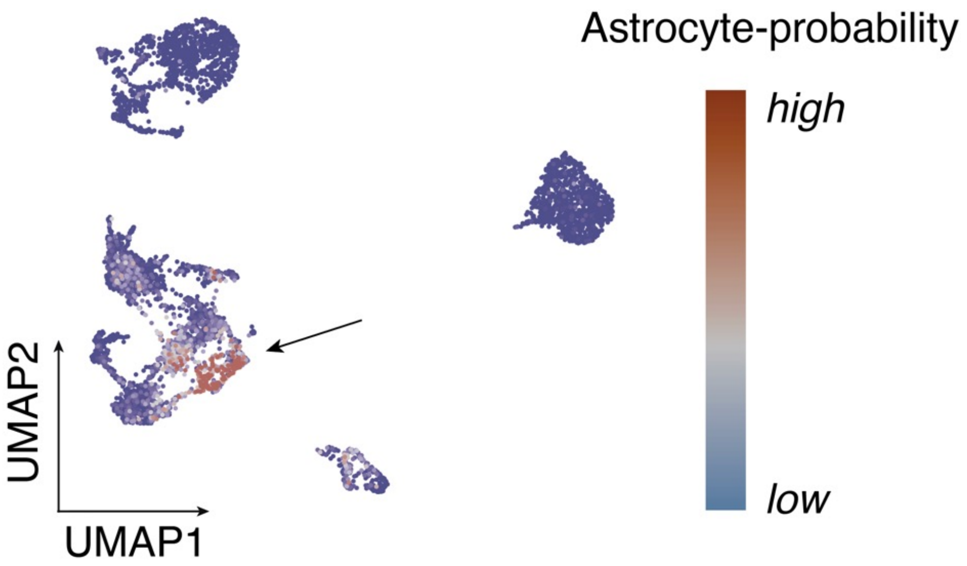

Supplemental Figure 9. Astrocyte-probability scores reveal a very small number of astrocytes amongst cells isolated by serial trypsinization of patient GBM. Related to Figures 1D-F. UMAP plot of cells isolated by serial trypsinization from patient GBM was overlaid with astrocyte probability scores derived by measuring gene expression of 10 astrocyte markers from the Azimuth human cortex data, revealing a very small number of cells amongst those isolated by serial trypsinization that transcriptomically profile as CAFs, with these cells (labeled with arrow) distinct from the CAFs present in the upper cluster of cells.

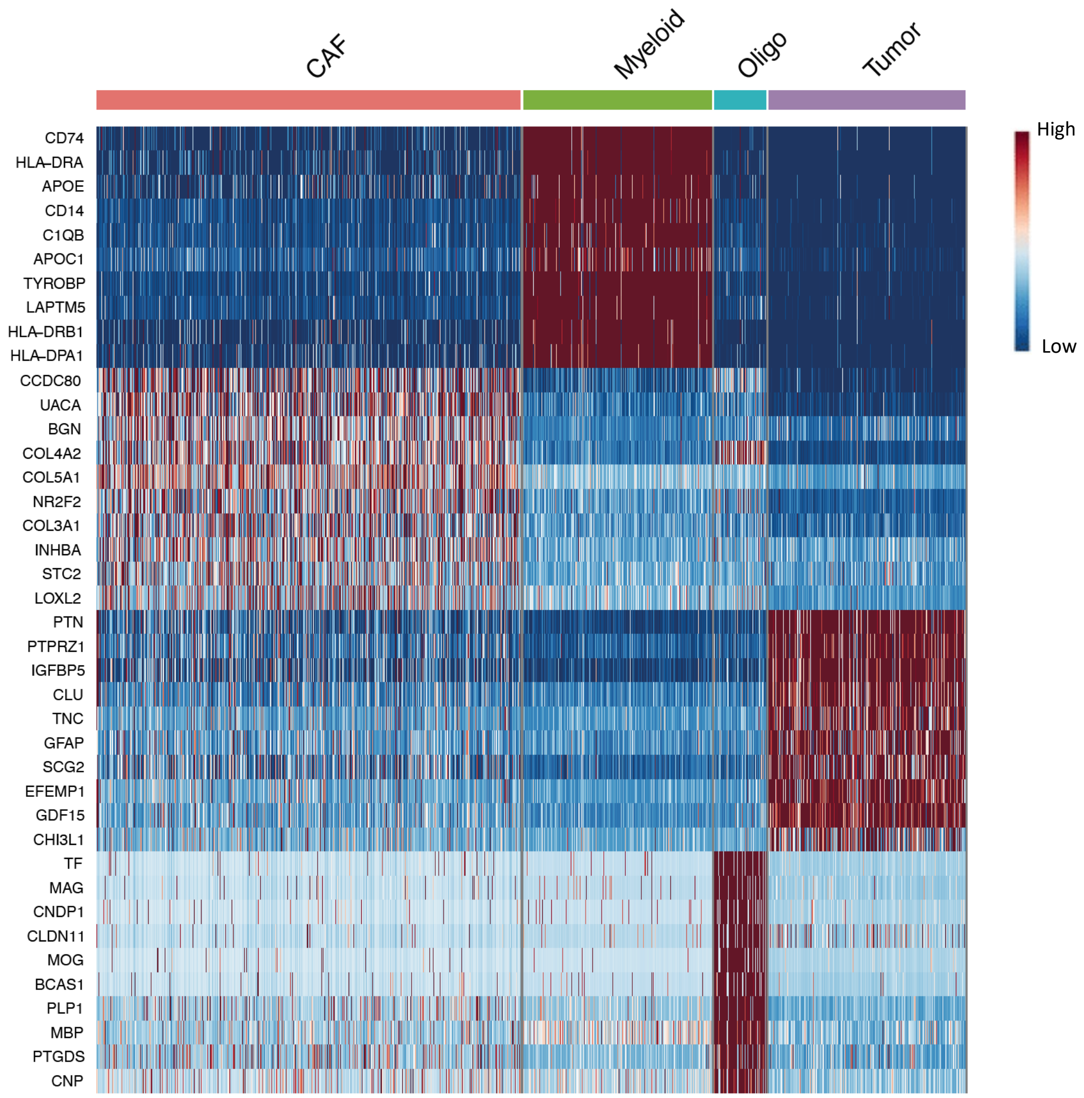

Supplemental Figure 10. Heatmap of differentially expressed genes between cells with high vs. low CAF probability scores after serial trypsinization. Related to Figures 1D-F. Shown is a heatmap indicating the top differentially expressed genes between cells with high vs. low CAF probability scores. The predicted cell identity of cells with low CAF probability scores is shown in the horizontal bar above the heatmap. Genes expressed in cells with high CAF probability scores had proven roles in CAF biology demonstrated in other cancers.

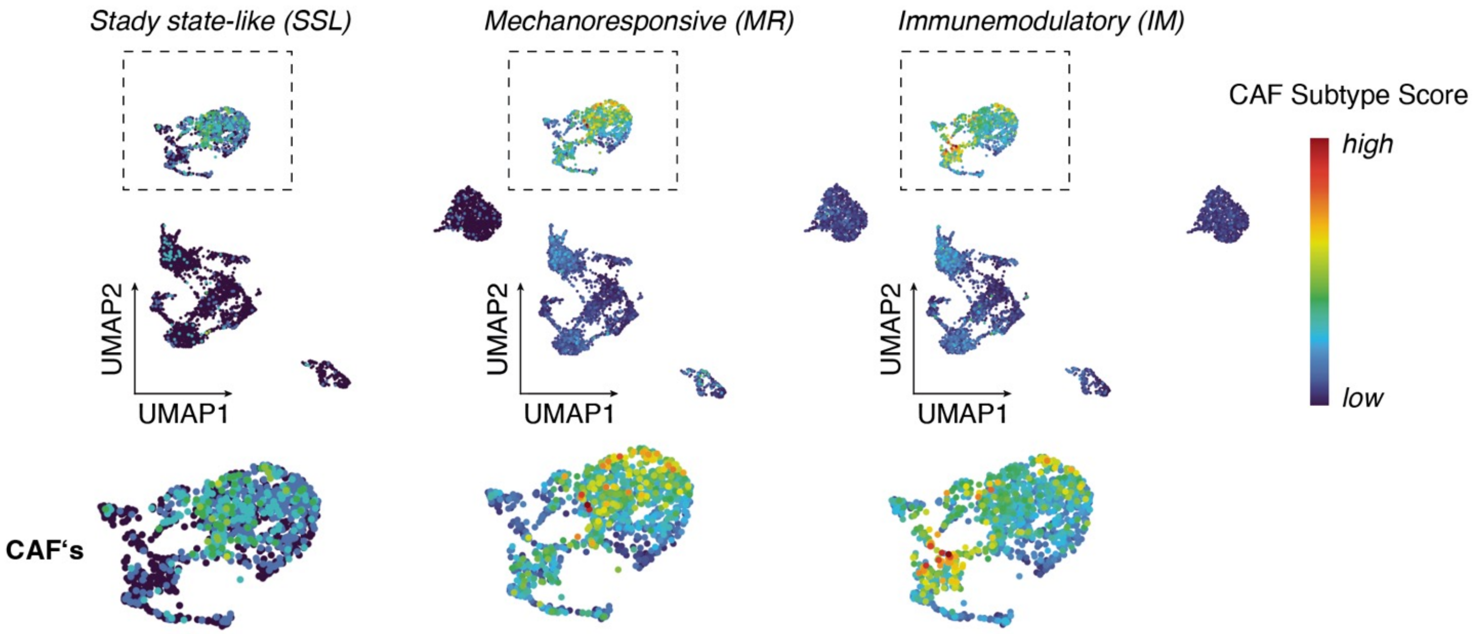

Supplemental Figure 11. Gene-expression analysis identifies 3 CAF subtypes defined across multiple cancer types and species in cells isolated from GBM by serial trypsinization. Related to Figures 1G-H. UMAP plot of cells isolated by serial trypsinization from patient GBM was overlaid with subtype score for three CAF subtypes (steady-state like=SSL, mechanoresponsive=MR, and immunomodulatory=IM) described as being preserved across cancer types and species.

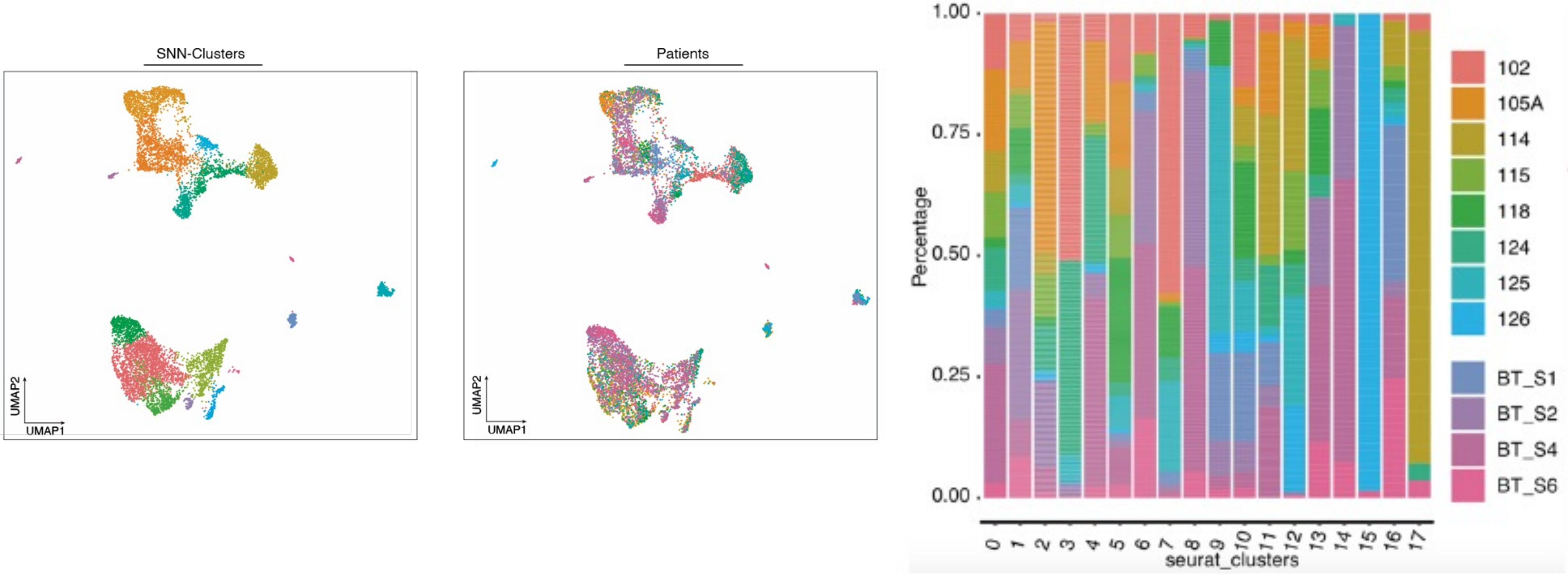

Supplemental Figure 12. Single cell RNA-seq reveals 18 cell clusters in 12 patient GBMs. Related to Figure 2A. Nearest neighbor clustering and a non-dimensional reduction technique UMAP were used to identify and visualize 18 robust cell clusters in 12 patient GBMs. As shown here, all 18 clusters were seen in each of the 12 individual patients.

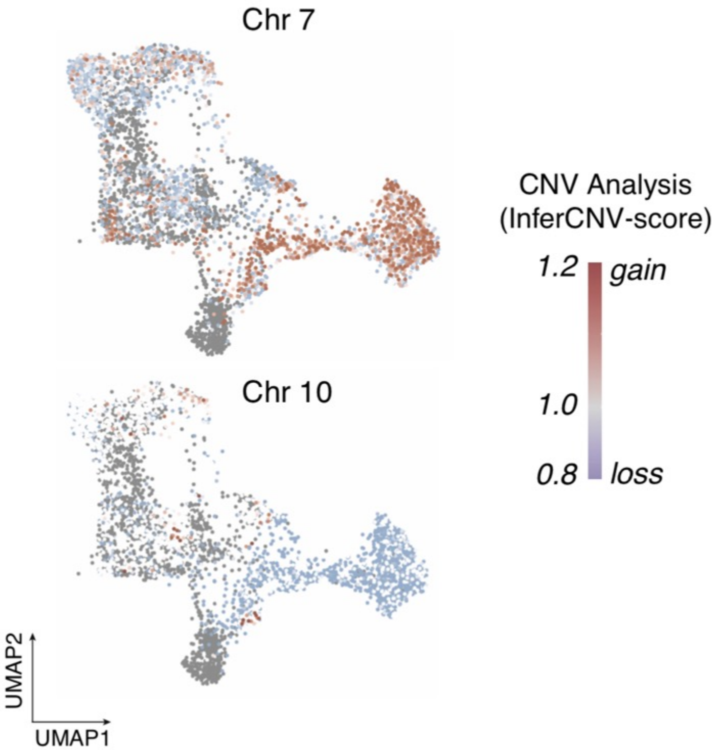

Supplemental Figure 13. CNV analysis in cells from patient GBMs reveals a cluster of cells without copy number variation that corresponded to cells with high CAF probability. Related to Figures 2A-C. Shown are results of CNV analysis from chromosome 7 and Chromosome 10 imposed upon a UMAP plot of cells isolated from 12 GBM patients. Based on CNV analysis, tumor cells were enriched in the right side of the upper cluster, with the remaining cells being stromal and cells with high CAF probability identified outside the tumor cell portion of the upper cluster, as shown in Fig. 1C.

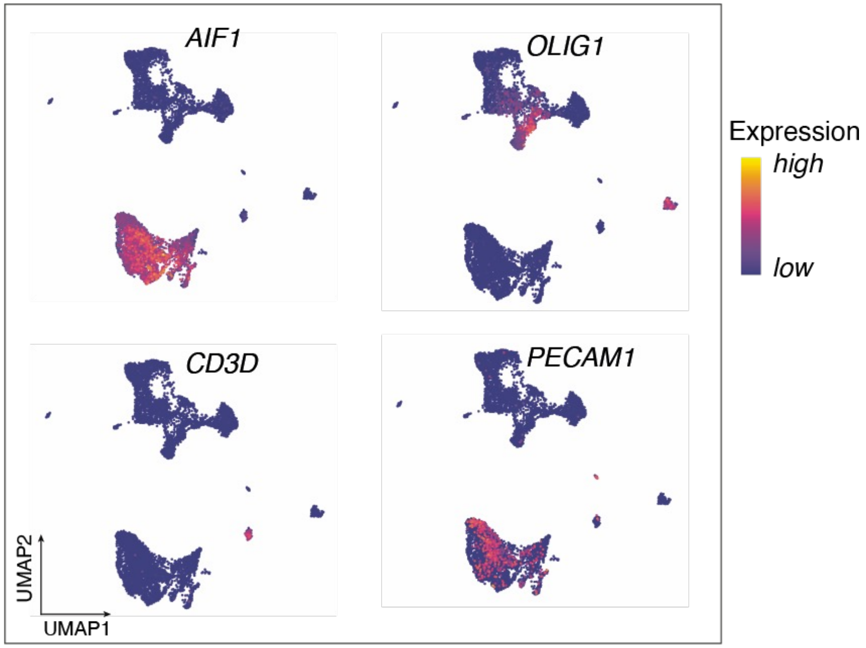

Supplemental Figure 14. Cells with high CAF probability in patient GBMs do not express other stromal cell markers. Related to Figures 2A-C. The cells with high CAF probability to the upper left in Figures 2A and C did not express macrophage marker AIF1, oligodendrocyte marker OLIG1, T-cell marker CD3D, or endothelial marker PECAM1

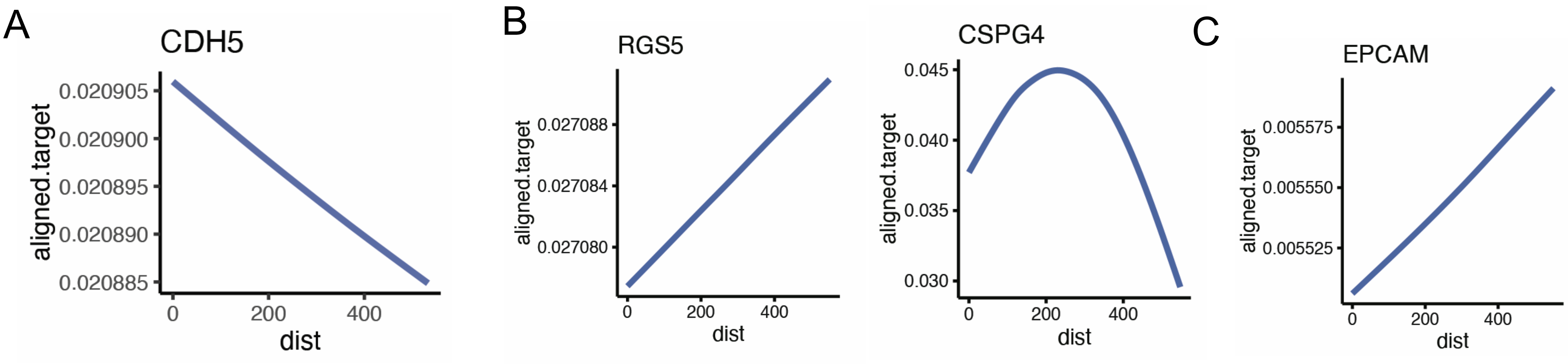

Supplemental Figure 15. Spatial transcriptomics reveals proximity of CAFs to other cells in patient GBMs. Related to Figure 2E-H. Spatial transcriptomics was used to reveal the probability of cell types being within certain distances of GBM CAFs. (**A**) Endothelial cells (expressing CDH5) were in close proximity to CAFs. (**B**) Pericytes (expressing RGS5 or CSPG4) were in variable proximity to CAFs. (**C**) Epithelial cells (EPCAM1) were not in close proximity to CAFs.

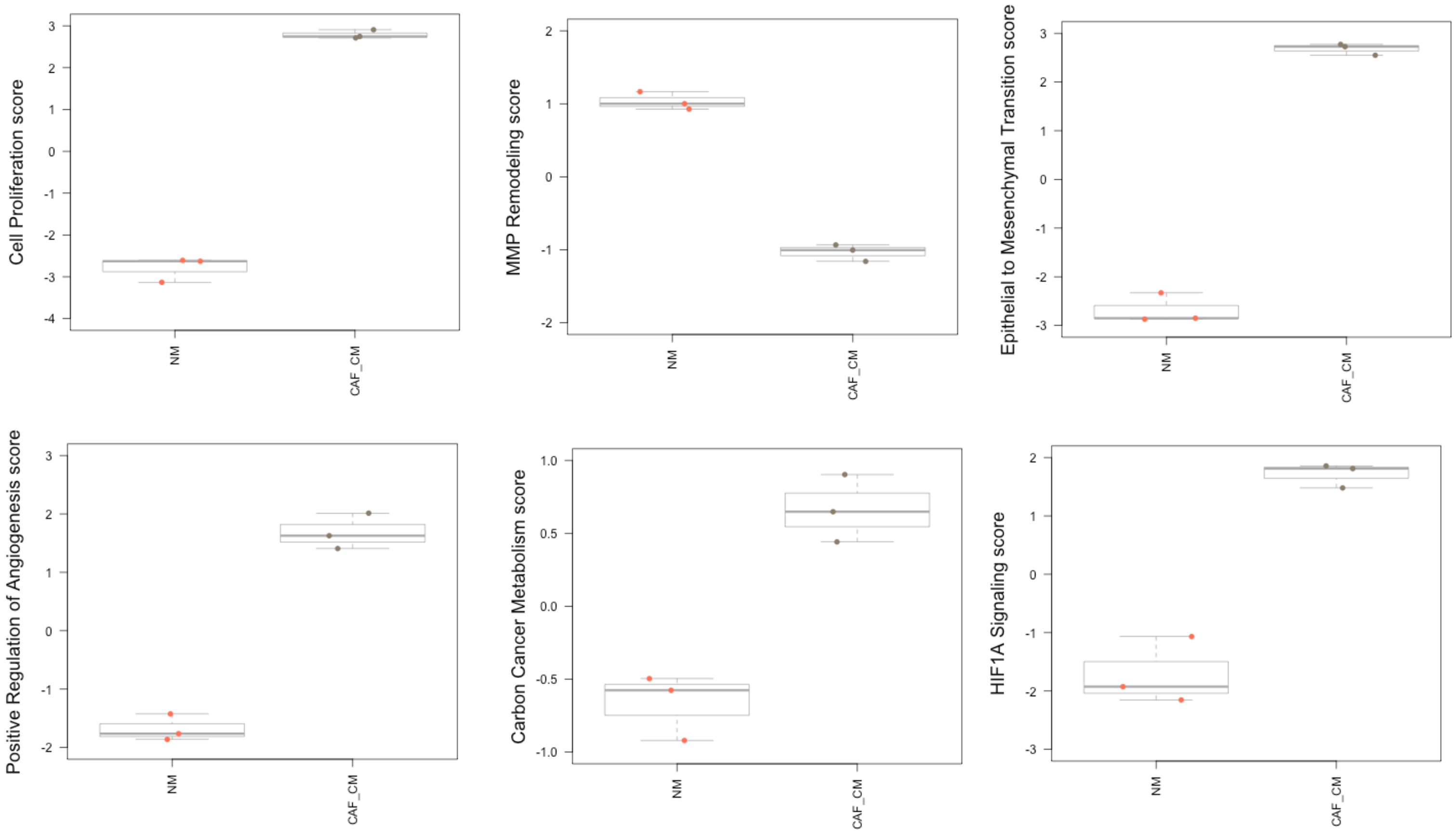

Supplemental Figure 16. Pathways upregulated when GBM stem cells are grown in CAF-conditioned media. Related to Figures 3A-B. Shown are the effects of GBMpt3CAF CM on pathways in cultured GBM6 neurospheres assessed by the nanostring multiplex pathways. Cell proliferation, EMT, positive regulation of angiogenesis, carbon cancer metabolism, and HIF1α signaling were upregulated, while MMP remodeling was downregulated.

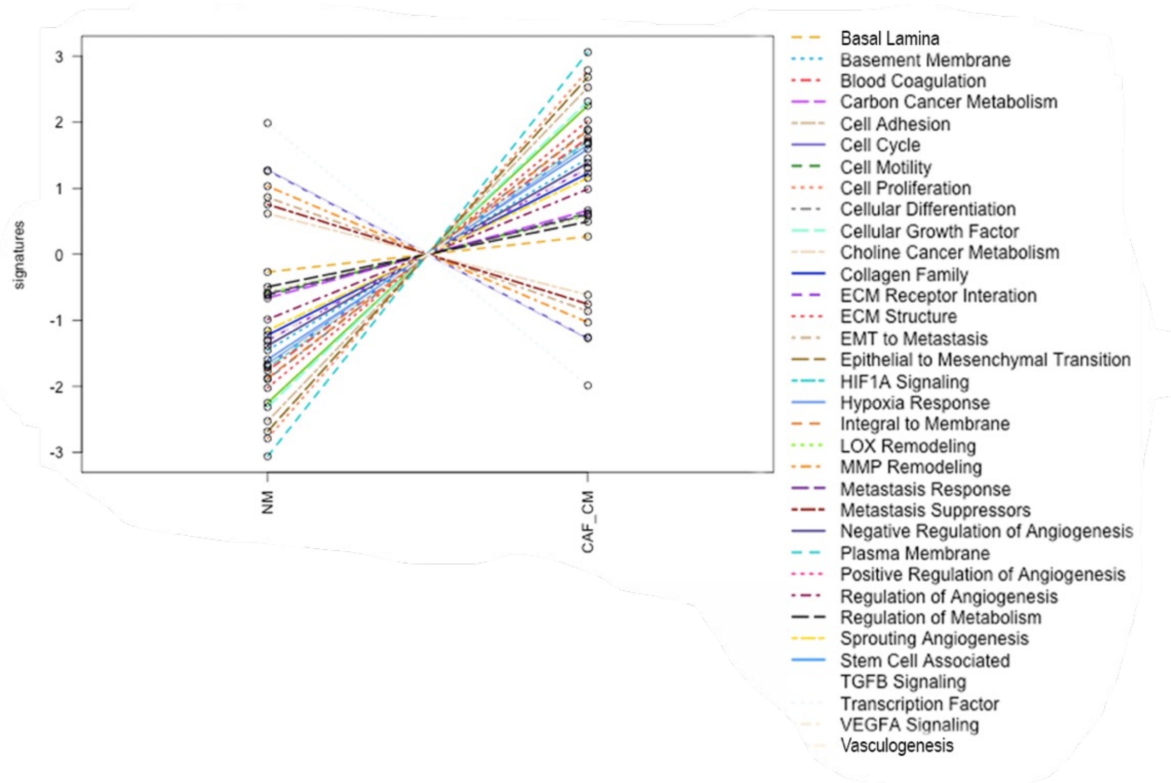

Supplemental Figure 17. Pathways activated in GSCs grown in GBM CAF CM. Related to Figures 3A-B. Multiplex transcriptomic analysis using the NanoString nCounter platform revealed genes in the cancer progression process upregulated by GBMpt3CAF CM in GBM6 stem cells, as seen by pathway analysis showing that CAF CM upregulated pathways associated with HIF-1α, EMT, and cell proliferation in GBM stem cells (P<0.002).

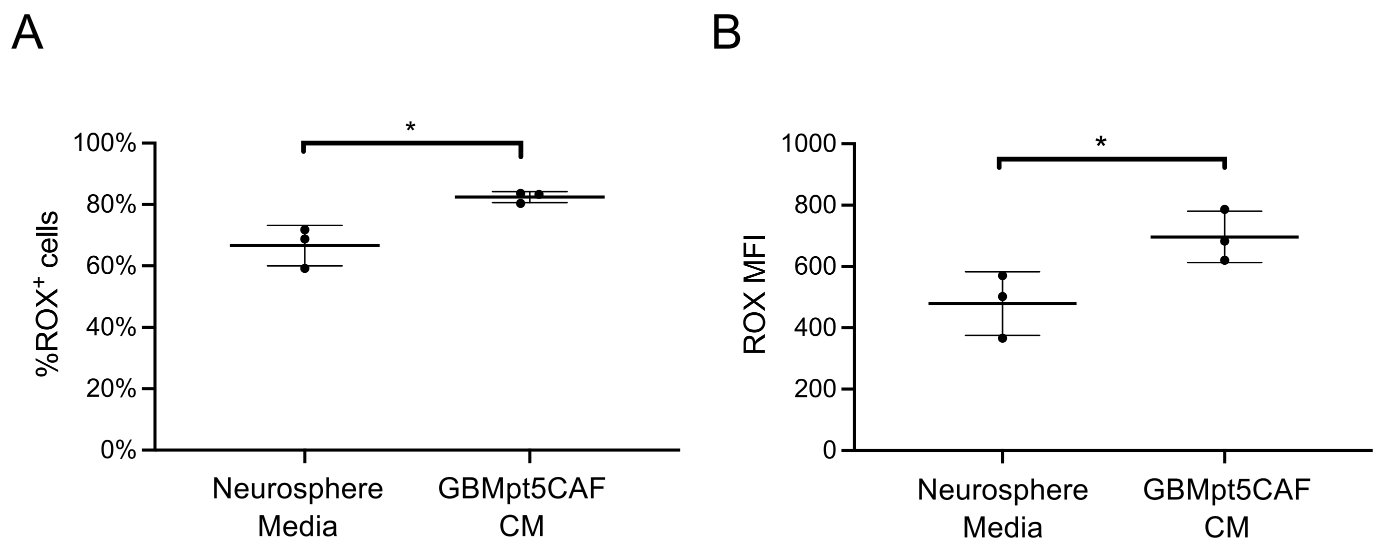

Supplemental Figure 18. CAF-secreted factors enhance ROS production by GBM stem cells. Related to Figures 3A-B. GBM43 neurospheres produced more ROS when grown in CM from GBMpt5CAF cells than when in neurosphere media, as assessed by (**A**) percentage of cells taking up the CellROX^{TM} reagent (n=3/group; P=0.016; t-test) and (**B**) mean fluorescence intensity (MFI) of cells incubated with the CellROX^{TM} reagent (n=3/group; P=0.048; t-test). Shown are scatter dot plots with horizontal lines at mean and vertical lines representing standard deviations).

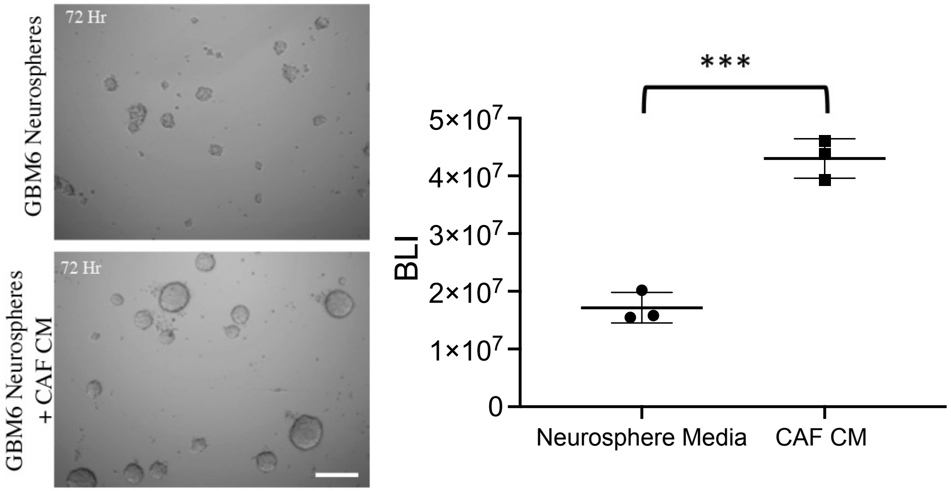

Supplemental Figure 19. CAF conditioned media promotes proliferation of GBM cells growing in neurospheres. Related to Figures 3C-D. Culturing GSC-containing neurospheres derived from luciferase-expressing GBM6 GBM cells in CM from GBMpt4CAFs for 72 hours led to increased bioluminescence (BLI) compared to growing these cells in neurosphere media ($P<0.001$; t-test). Shown are scatter dot plots with horizontal lines at mean and vertical lines representing standard deviations).

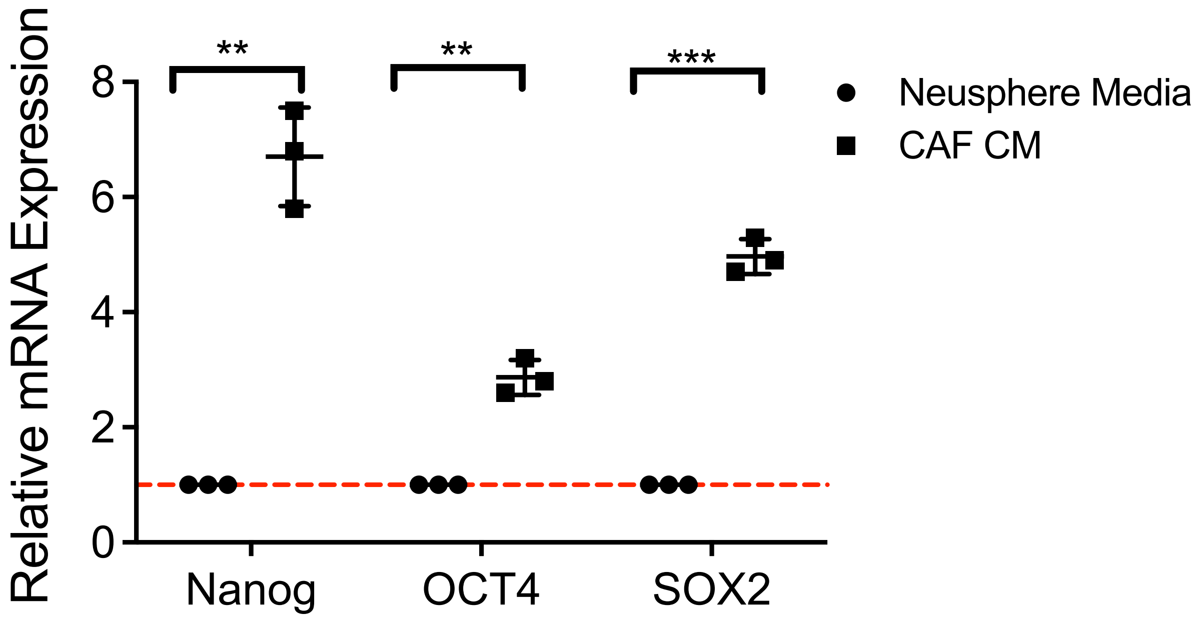

Supplementary Figure 20. CAF conditioned media promotes proliferation of GBM cells growing in neurospheres. Related to Figures 3C-D. Incubating GSC-containing neurospheres derived from DBTRG-05MG GBM cells in GBMpt1CAF CM for 24 hours also increased the expression of GBM stem cell genes Nanog 6.7-fold (P=0.009; t-test), Oct4 3.0-fold (P=0.005; t-test), and Sox2 5.0-fold (P<0.001; t-test). Shown are scatter dot plots with horizontal lines at mean and vertical lines representing standard deviations).

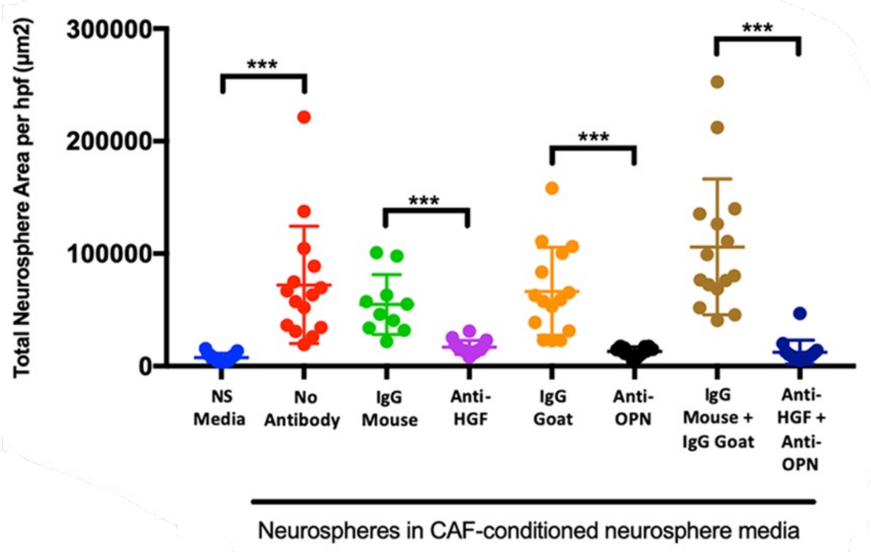

Supplemental Figure 21. GBM CAF effects on GSCs are mitigated by HGF and/or OPN blocking antibodies. Related to Figures 3F-G. 1000 GBM6 cells were seeded in a 12-well plate in triplicate with either neurosphere media or CAF CM with or without antibodies targeting osteopontin (OPN) and/or hepatocyte growth factor (HGF) for 72 hours. GBMpt4CAF CM induced neurosphere formation as measured by the total neurosphere area ($P<0.001$), effects that were mitigated by both anti-HGF ($P<0.001$) and anti-OPN ($P<0.001$), with the combination of anti-HGF and anti-OPN reducing the total neurosphere area more than either antibody alone ($P<0.001$) (n=15 hpf across 3 wells/group; ANOVA with post-hoc Tukey). Shown are scatter dot plots with horizontal lines at mean and vertical lines representing standard deviations).

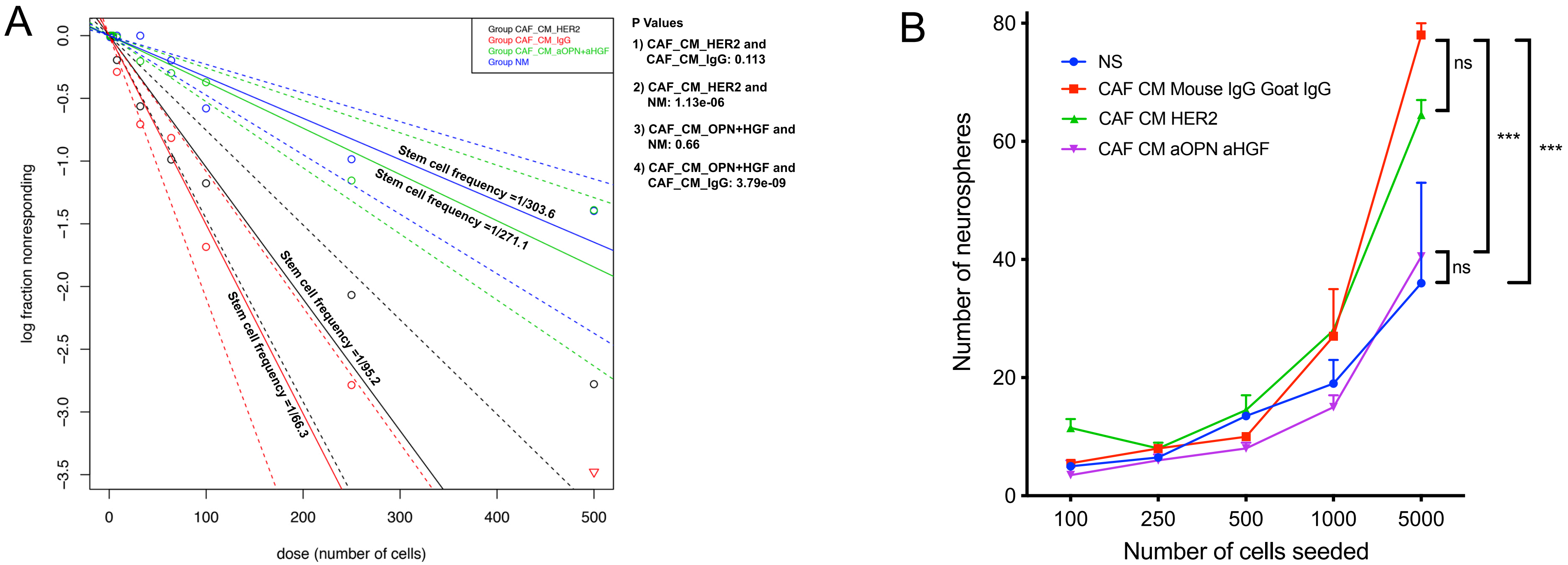

Supplemental Figure 22. CAF conditioned media enriches GBM 43 GSCs in a manner inhibited by antibodies targeting osteopontin and HGF. Related to Figures 3F-G. (A) Limiting dilution sphere formation assay represented by a Poisson distribution shows that the increase in GSC frequency in CAF_CM is mitigated by combining anti-HGF and anti-OPN. GSC frequency was not mitigated when using a HER2 antibody in CAF_CM. Shown is a log-fraction plot of the limiting dilution model fitted to the data. The slope of the line is the log-active cell fraction. The dotted line gives the 95% confidence interval. The data value with zero negative response at a particular dose is represented by a downward pointing triangle. **(B)** Number of GBM43 spheres were calculated, in which neurosphere yield is plotted as a function of number of cells seeded. Growing 5000 cells in conditioned media (CM) from GBMpt5CAFs increased neurosphere yield ($P<0.001$), with the combination of anti-osteopontin and anti-HGF antibodies reversing this effect ($P<0.001$) in a manner not seen with anti-Her2 antibody ($P=0.3$) (n=3/group; ANOVA with post-hoc Tukey).

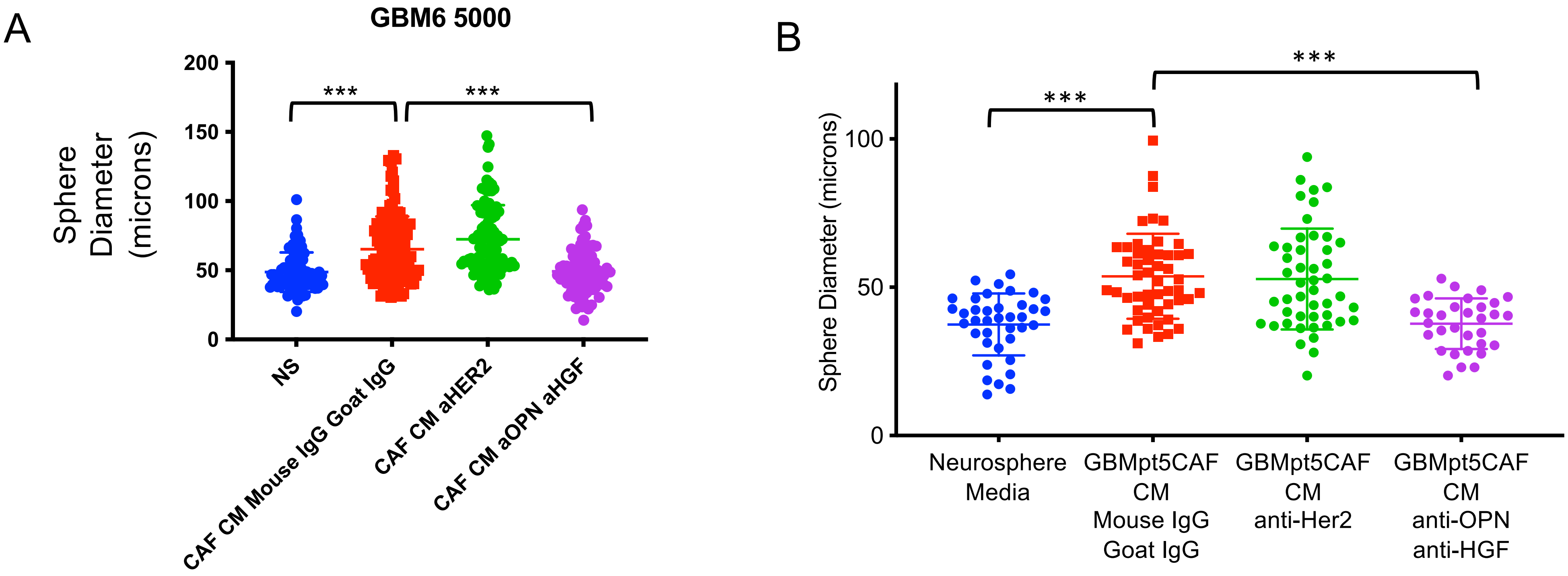

Supplemental Figure 23. Neurosphere diameter is increased by CAF CM in a manner inhibited by antibodies targeting osteopontin and hepatocyte growth factor. Related to Figs. 3F-G. (A) GBM6-derived neurospheres achieved a larger diameter when 5000 cells were grown in CM from GBMpt5CAFcells (P<0.001) in a manner not affected by an antibody targeting Her2 (P=0.1) but reversed when antibodies targeting osteopontin (OPN) and hepatocyte growth factor (HGF) were combined (P<0.001) (ANOVA with post-hoc Tukey). (**B**) Shown are sphere diameters when 1000 GBM43 cells were plated, with GBMpt5CAF CM in the presence of control antibodies increasing sphere diameter (P<0.001) in a manner reversed by adding anti-osteopontin and anti-HGF antibodies (P<0.001) but not affected by adding anti-Her2 antibody (P=0.99) (n=32-51/group; ANOVA with post-hoc Tukey). Shown are scatter dot plots with horizontal lines at mean and vertical lines representing standard deviations.

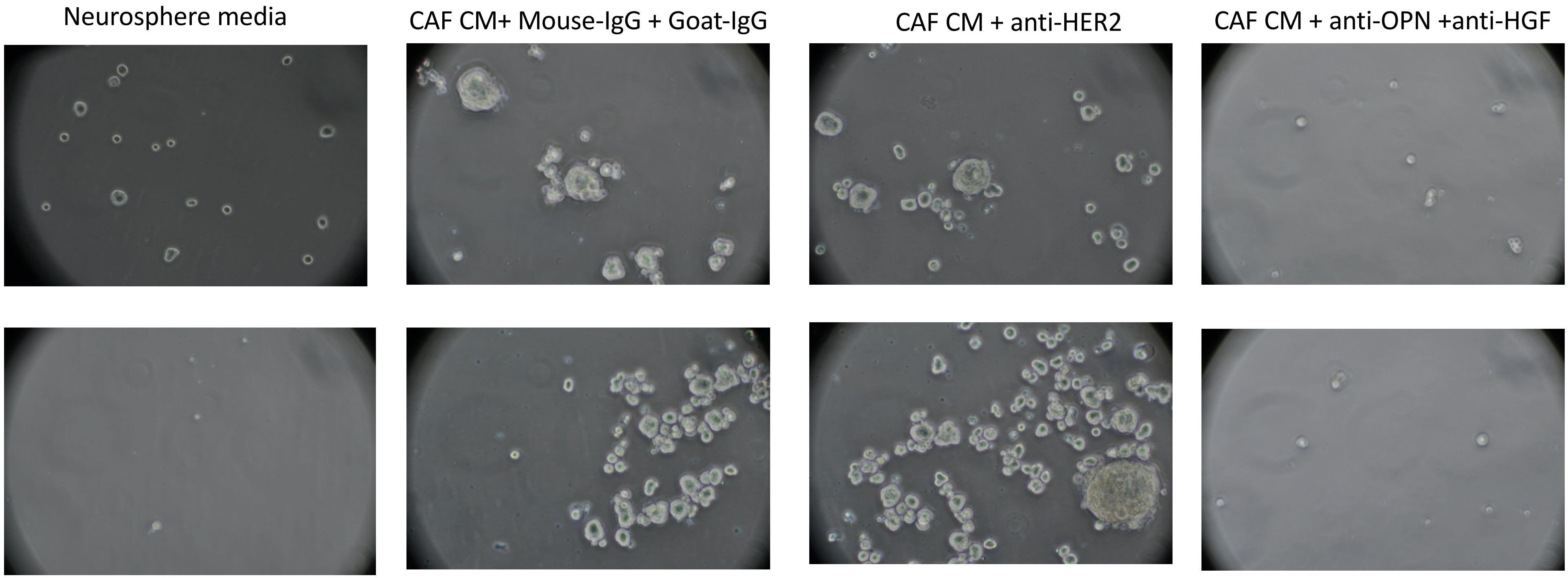

Supplemental Figure 24. Representative images of GBM43 neurospheres. Related to Figures 3C-G. Shown are sphere diameters when 1000 GBM43 cells were plated, with GBMpt5CAF CM in the presence of control antibodies increasing sphere diameter (P<0.001) in a manner reversed by adding anti-osteopontin and anti-HGF antibodies (P<0.001) but not affected by adding anti-Her2 antibody (P=0.99) (n=32-51/group).

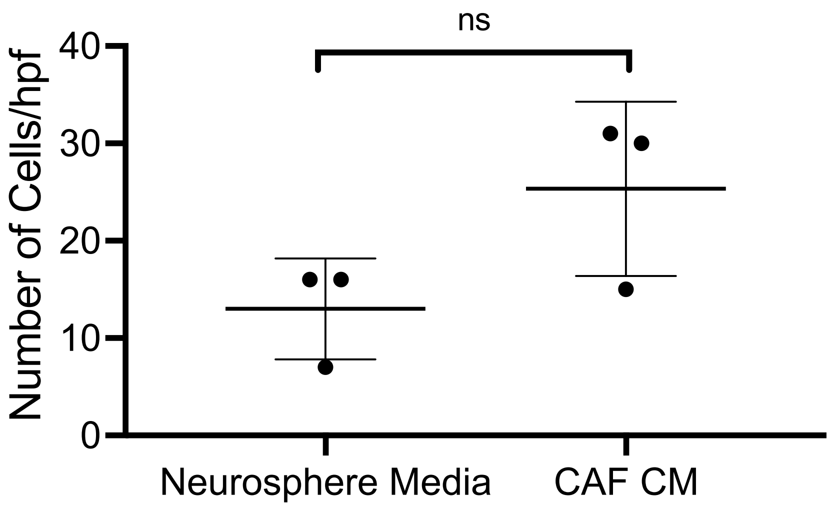

Supplemental Figure 25. Lack of chemotactic attraction of GSCs towards CAFs. Related to Figure 3. Neurospheres derived from GBM6 cells migrated equally towards control media or GBMpt1CAF CM (P=0.1=NS; t-test), indicating lack of chemotactic attraction. Shown are scatter dot plots with horizontal lines at mean and vertical lines representing standard deviations

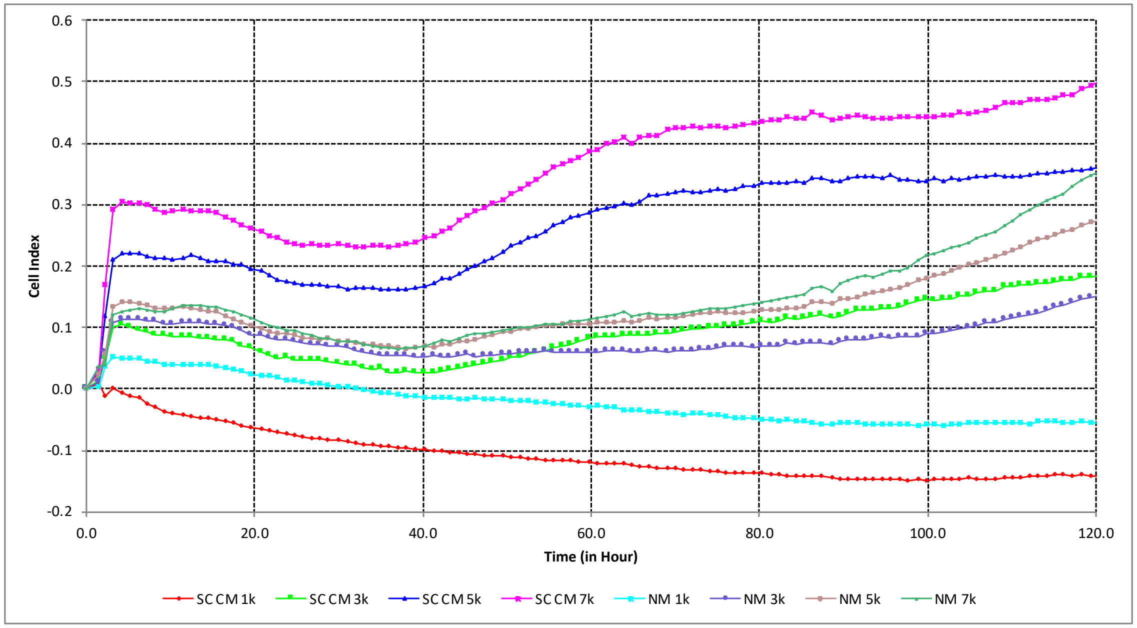

Supplemental Figure 26. Optimization of number of cultured GBM CAFs for proliferation assay. Related to Figures 4C and 4F. Shown is the continuous growth of GBMpt4CAFs plated at varying densities (1000, 3000, 5000, or 7000 cells per well in 96 well plates) in neuropshere media (NM) or GBM6 stem cell conditioned media (SC CM). Proliferation was continuously assessed using the xCELLigence RTCA MP instrument to measure impedance as a surrogate for cell count over 120 hours. Based on these results, 1000 cells per well was chosen for cells involving GBMpt4CAFs grown in GBM6 neurosphere CM in **Supp. Fig. 28.** For experiments involving GBMpt5CAFs grown in GBM43 neurosphere CM in **Figs. 4C and 4F**, 5000 cells per well were chosen based on a similarly performed optimization study.

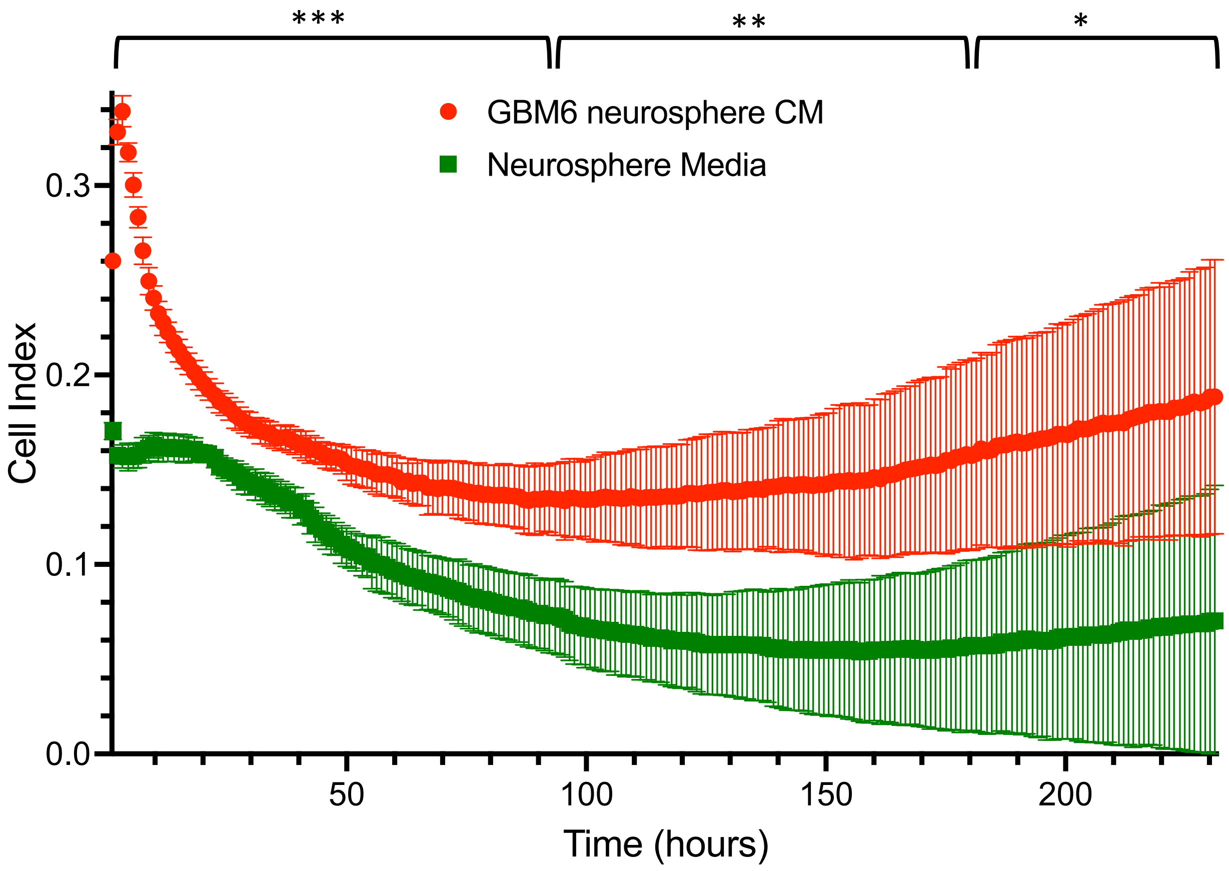

Supplemental Figure 27. Conditioned media from GBM6-derived neurospheres promotes CAF proliferation. Related to Figure 4C. Proliferation of 1000 CAFs/well in a 96-well plate was continuously assessed using the xCELLigence RTCA MP instrument to measure impedance as a surrogate for cell count over 120 hours. CAFs grown in GBMpt5CM derived from GBM6 neurospheres exhibited far greater proliferation than CAFs grown in neurosphere media. n=5/group; t-test. *P<0.05; **P<0.01; ***P<0.001

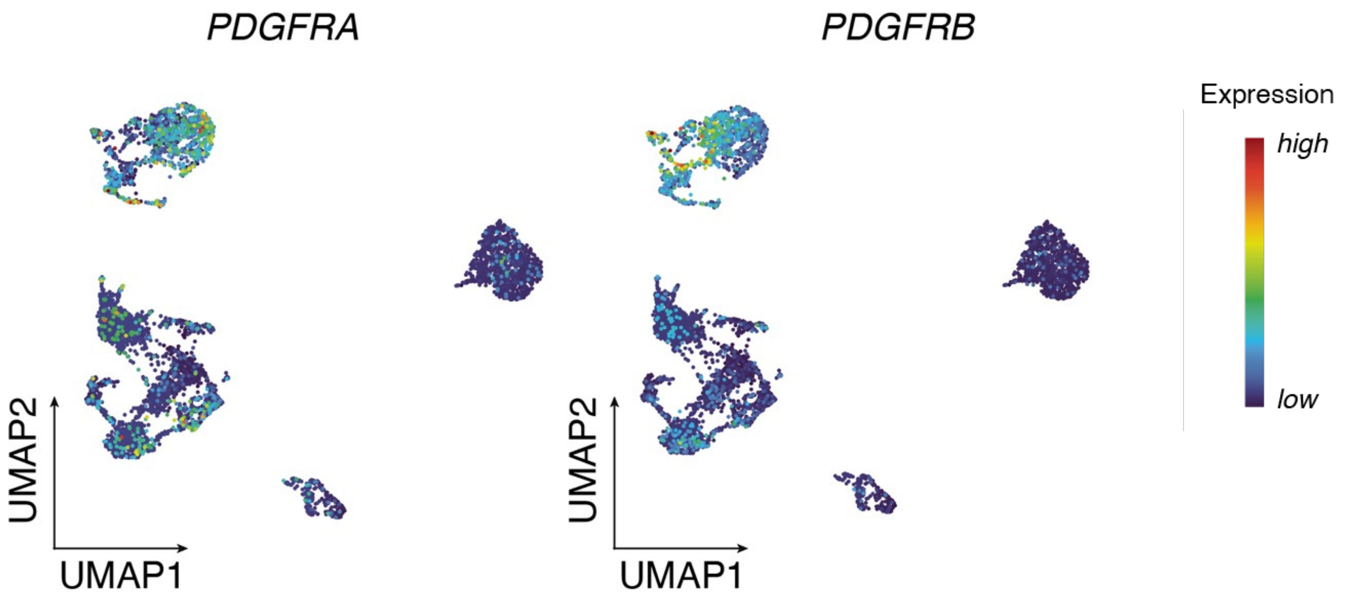

Supplemental Figure 28. PDGFRA and PDGFRB expression is elevated in CAFs compared to other cells isolated by serial trypsinization from patient GBMs. Related to Fig. 4F. Shown are feature plots displaying *PDGFRA* (left) and *PDGFRB* (right) expression in cells isolated by serial trypsinization. Most of the cells expressing *PDGFRA* and *PDGFRB* appear in the upper left cluster where cells with high CAF probability scores were found.

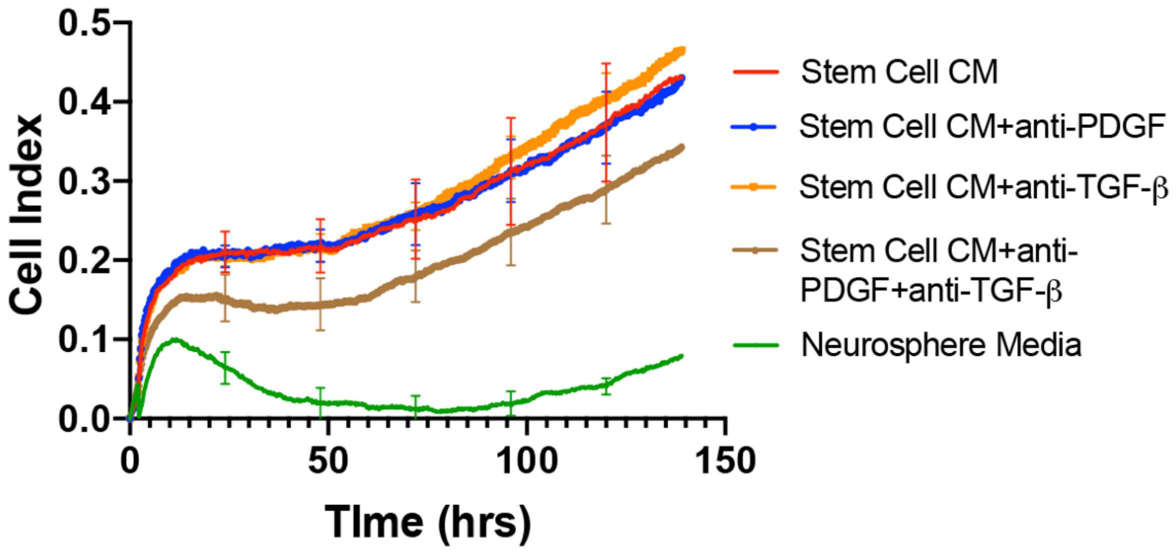

Supplemental Figure 29. Conditioned media from GBM6-derived neurospheres promotes CAF proliferation in a manner mediated by PDGF and TGF-β. Related to Fig. 4F. Proliferation of 1000 CAFs/well in a 96-well plate was continuously assessed using the xCELLigence RTCA MP instrument to measure impedance as a surrogate for cell count over 120 hours. While antibody against PDGF or TGF-β did not affect GBM6 neurosphere CM-induced GBMpt4CAF proliferation (0-139 hours: P=0.7-0.9 PDGF, P=0.5-0.9 TGF-β), combining neutralizing antibodies against PDGF and TGF-β at these concentrations reversed GBM6 neurosphere-induced GBMpt4CAF proliferation (50-139 hours: P=0.02-0.04 PDGF+TGF-β vs. TGF-β; 79-139 hours: P=0.03-0.04 PDGF+TGF-β vs. PDGF; 60-139 hours: P=0.03-0.048 PDGF+TGF-β vs. GSC CM) (n=5/group; ANOVA with post-hoc Tukey).

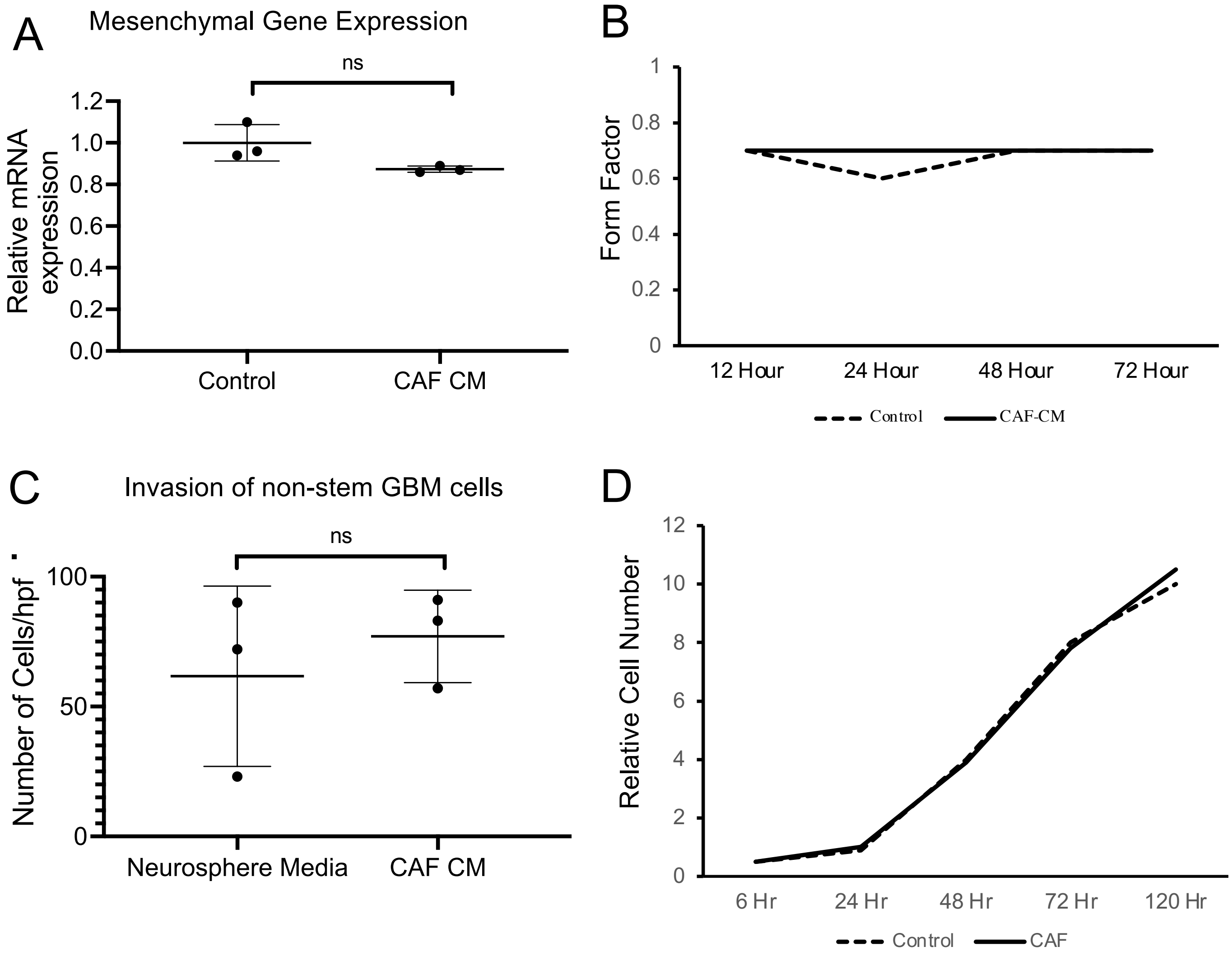

Supplemental Figure 30. Effects of CAF CM on non-stem GBM cells. GBMpt1CAF CM did not alter properties of adherent GBM cells, including (**A**) aggregate expression of five mesenchymal genes (**Supp. Table 6**) as assessed by qPCR in adherent DBTRG-05MG cells (P=0.6); (**B**) morphology of adherent GBM6 cells (P=0.06-0.8); (**C**) invasion of adherent GBM6 cells in matrigel chambers (P=0.5); and (**D**) proliferation (P=0.3-0.9) of GBM6 cells. n=3/group for all panels with t-test used for comparisons. (**A**) and (**C**) are scatter dot plots with horizontal lines at mean and vertical lines representing standard deviations.

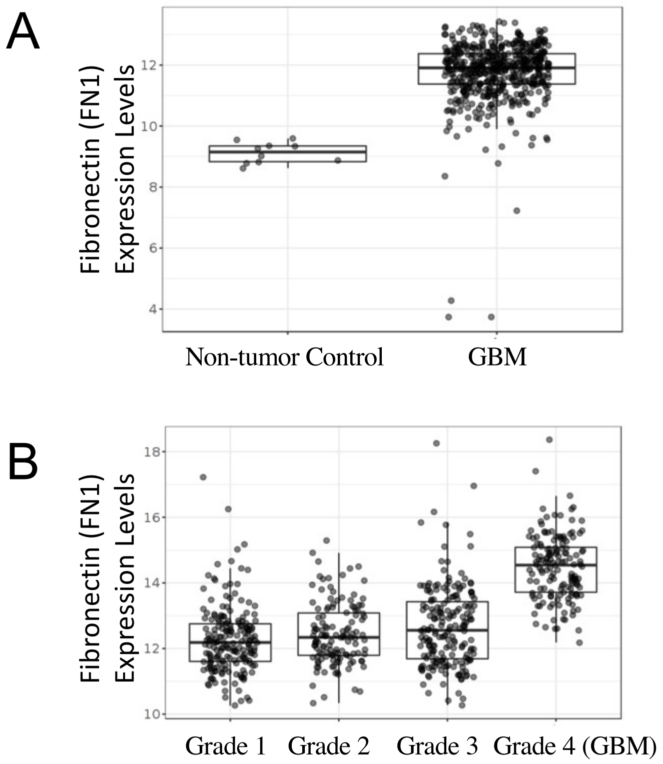

Supplemental Figure 31. Transcriptomic data from GlioVis data set reveal more fibronectin expression in GBM compared to low-grade glioma or normal brain. Related to Figure 5A. Population-based bioinformatic data was obtained from GlioVis (gliovis.bioinfo.cnis.es), and analyzed for fibronectin (*FN1*) expression, revealing that (**A**) GBM had significantly higher expression of *FN1* than non-tumor samples (P<0.001; t-test) and (**B**) GBM also had much higher expression of *FN1* than low grade gliomas (P<0.001; ANOVA with post-hoc Tukey).

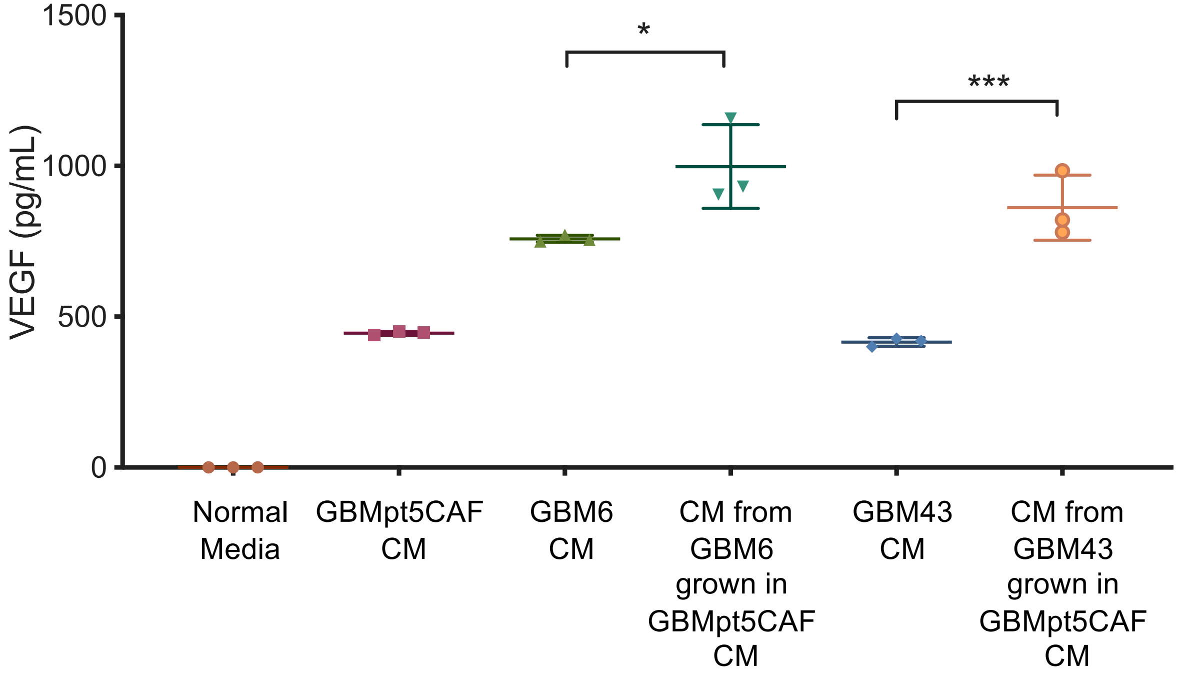

Supplemental Figure 32. CAF conditioned media elevates VEGF production by GBM neurospheres. Related to Figures 5C-D. Shown are VEGF ELISA results, revealing that CM from GBMpt5CAFs elevated GBM6 neurosphere (P=0.02) and GBM43 neurosphere (P<0.001) VEGF production (n=3/group; ANOVA with post-hoc Tukey). Shown are scatter dot plots with horizontal lines at mean and vertical lines representing standard deviations.

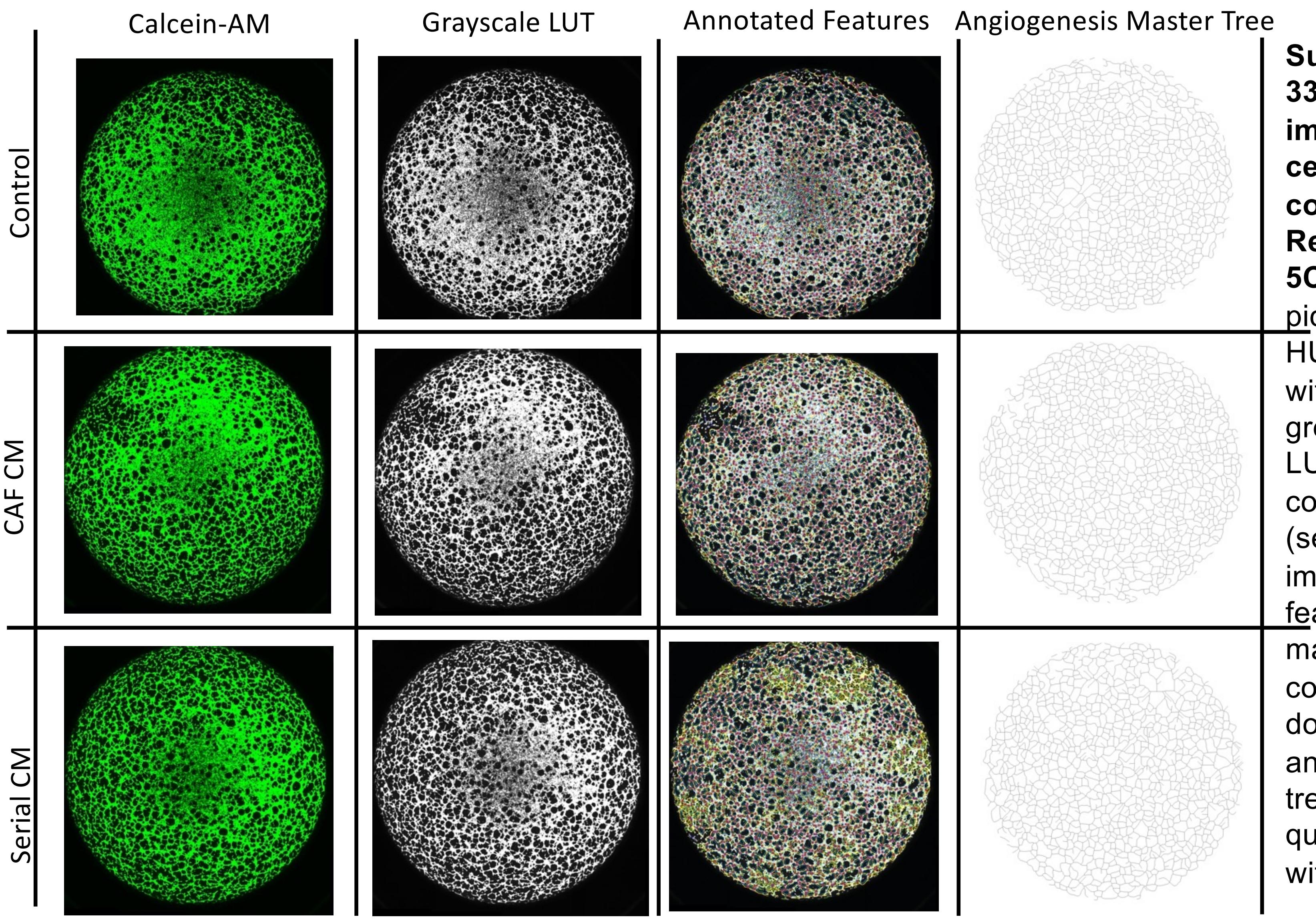

Supplemental Figure 33. Representative images of HUVEC cells in various conditions at 4 hours. Related to Figures 5C-D. Shown are pictures of cultured HUVEC cells labeled with Calcein-AM (left, green); the grayscale LUT (Look Up Tables) colorless image (second column); image with annotated features like nodes and master junctions (third column); and stripped down image of angiogenesis master tree representing quantified metrics without annotations.

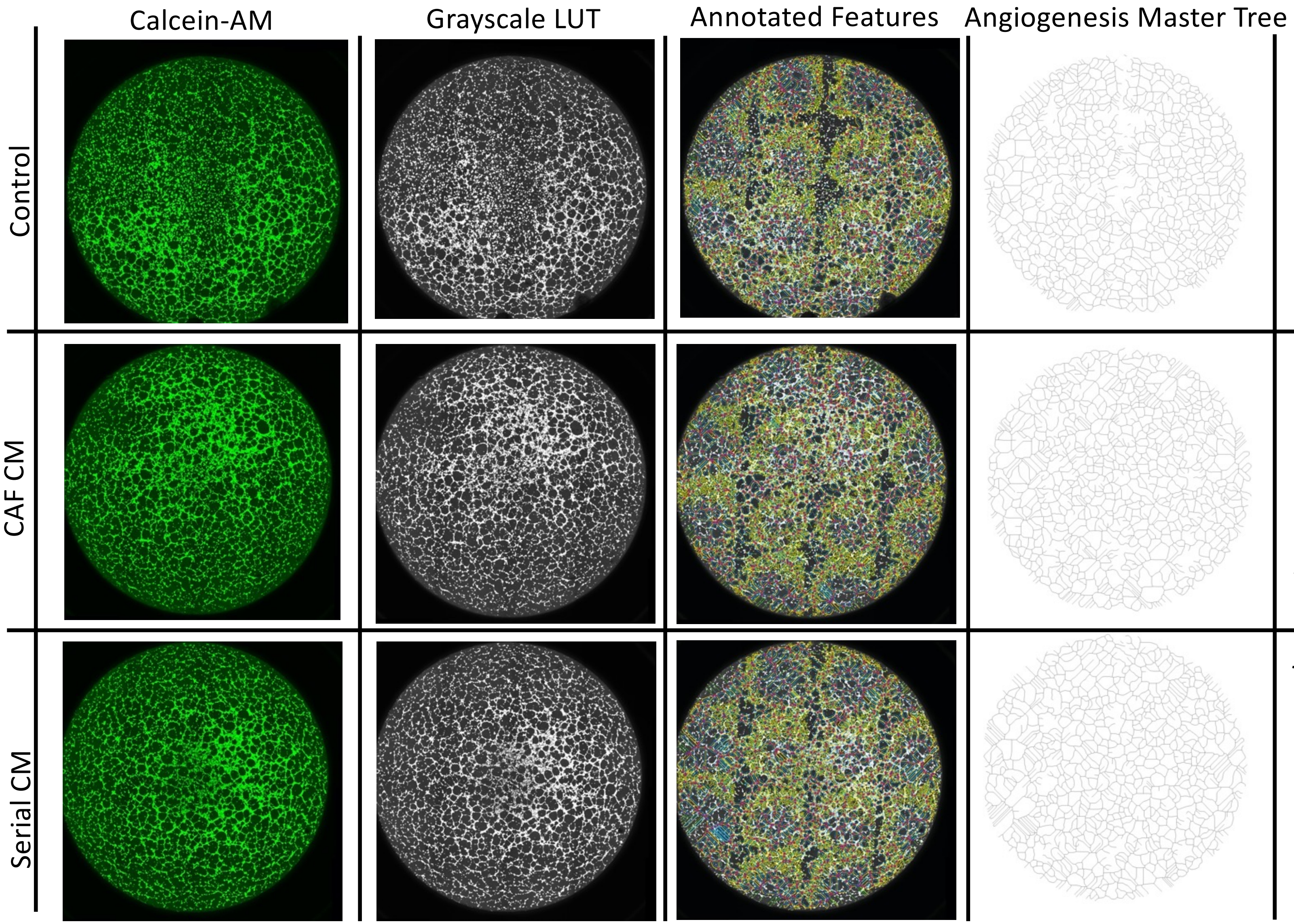

Supplemental Figure 34. Representative images of HUVEC cells in various conditions at 8 hours. Related to Figures 5C-D. Shown are pictures of cultured HUVEC cells labeled with Calcein-AM (left, green); the grayscale LUT (Look Up Tables) colorless image (second column); image with annotated features like nodes and master junctions (third column); and stripped down image of angiogenesis master tree representing quantified metrics without annotations.

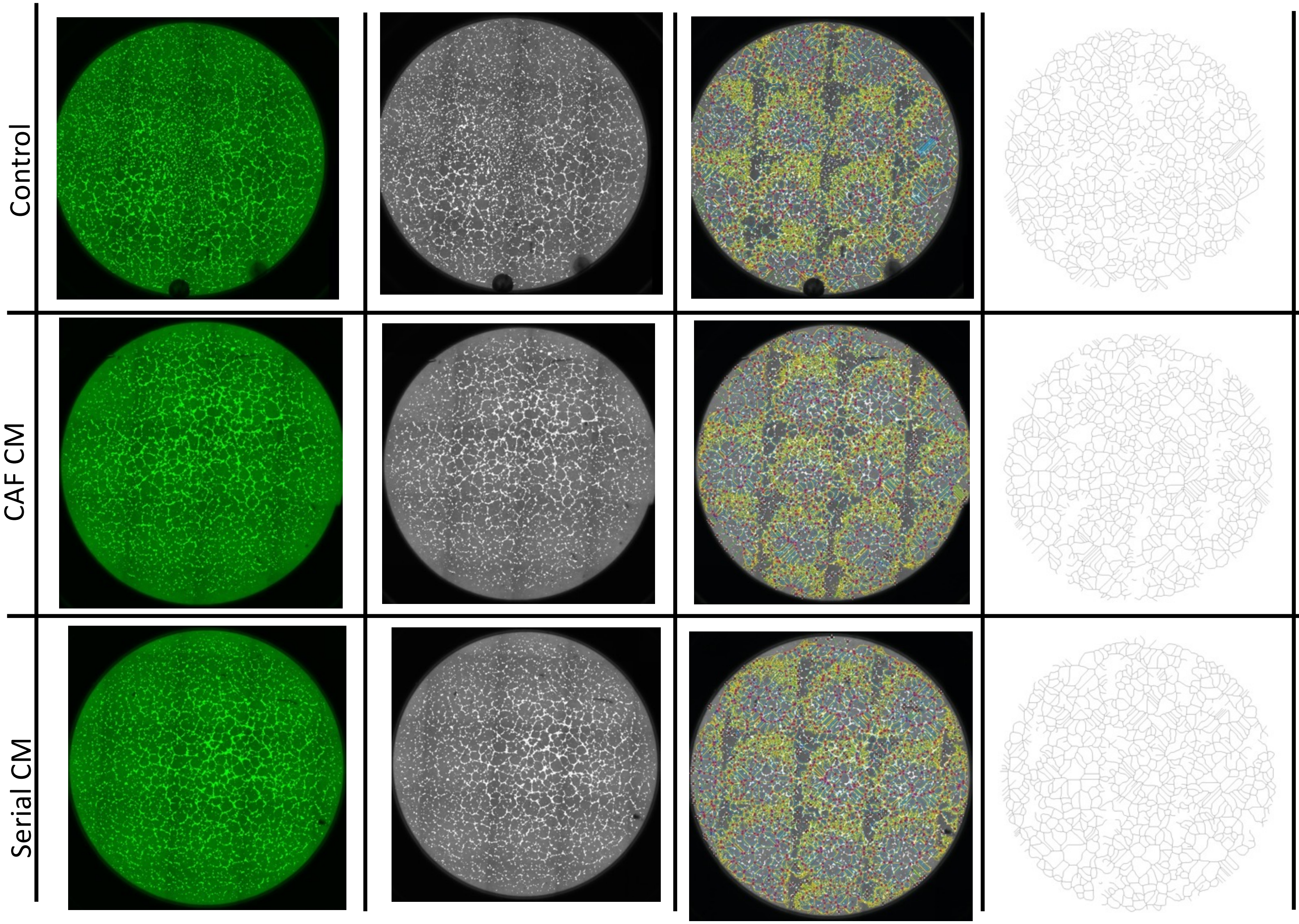

Supplemental Figure 35. Representative images of HUVEC cells in various conditions at 16 hours. Related to Figures 5C-D. Shown are pictures of cultured HUVEC cells labeled with Calcein-AM (left, green); the grayscale LUT (Look Up Tables) colorless image (second column); image with annotated features like nodes and master junctions (third column); and stripped down image of angiogenesis master tree representing quantified metrics without annotations.

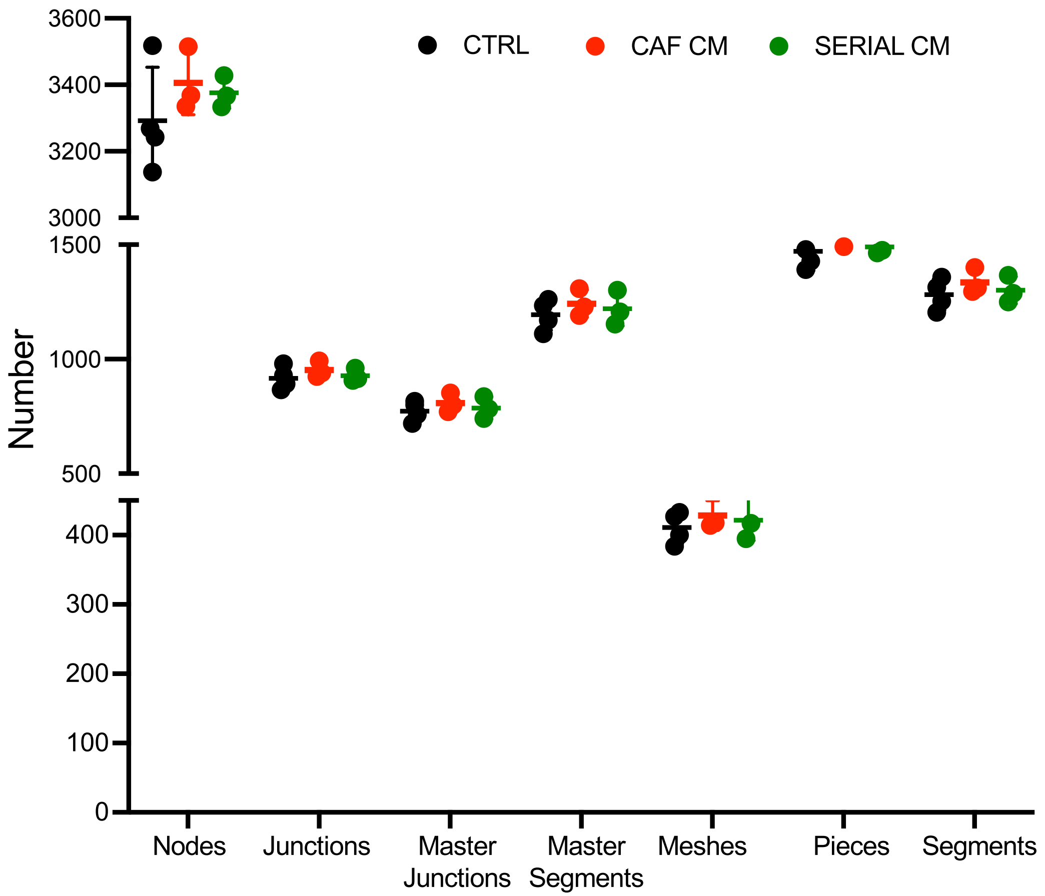

Supplemental Figure 36. GBM CAFs do not alter HUVEC fusion metrics at 16 hours. Related to Figure 5C-D. Shown are results of 7 different HUVEC fusion metrics which reflect the third and final stage of anigogensis (fusion of newly formed vessels). Shown are results when HUVEC cells were grown in control media (CTRL=black), CAF conditioned media (CAF CM=red) and serial CM (green) obtained when CAF CM was applied to GBM6 cells and the resulting CM harvested. Unlike the results seen with earlier stage angiogenesis metrics at 4 and 8 hours, neither CAF CM nor serial CM stimulated late stage angiogenesis (P=0.5-0.9; ANOVA with post-hoc Tukey). Shown are scatter dot plots with horizontal lines at mean and vertical lines representing standard deviations.

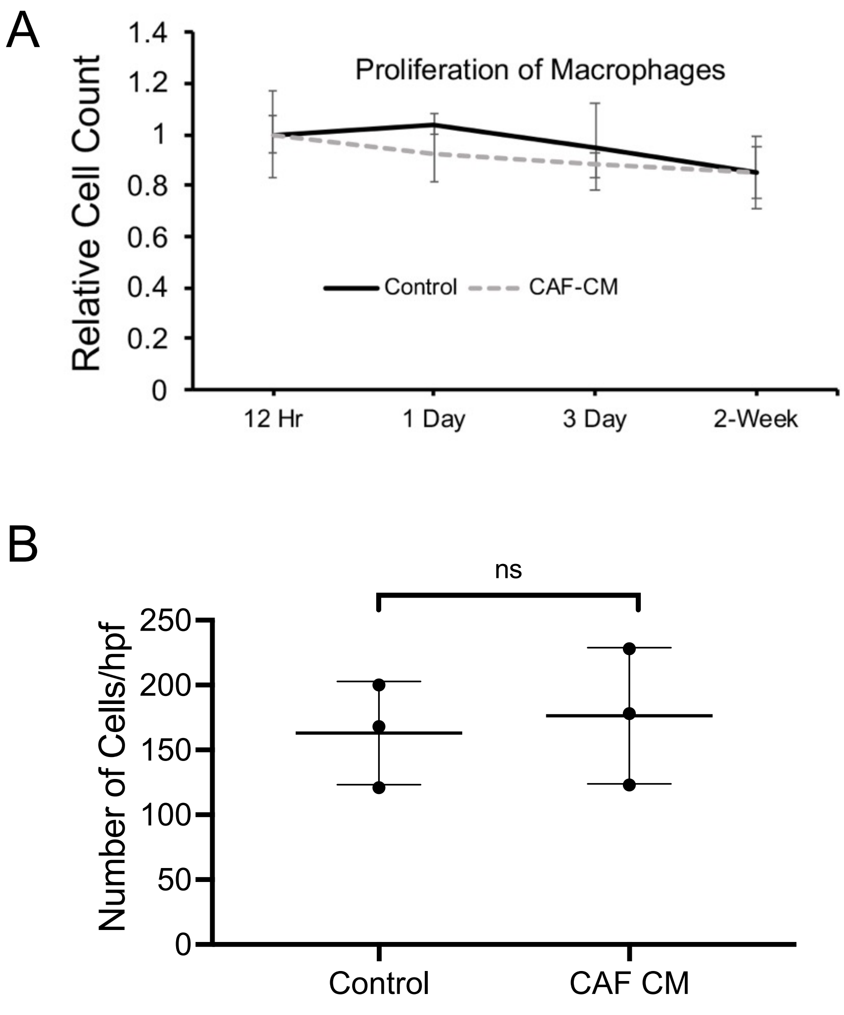

Supplemental Figure 37. Effect of CAFs on macrophage proliferation and invasion. Related to Figures 5E-G. CAFs did not induce macrophage (**A**) proliferation (n=3/group; P=0.3-0.9; t-test) or **(B)** chemotaxis as measured in Matrigel invasion assays performed towards control media or CAF CM (n=3/group; P=0.7; t-test; scatter dot plot with horizontal lines at mean and vertical lines representing standard deviations.).

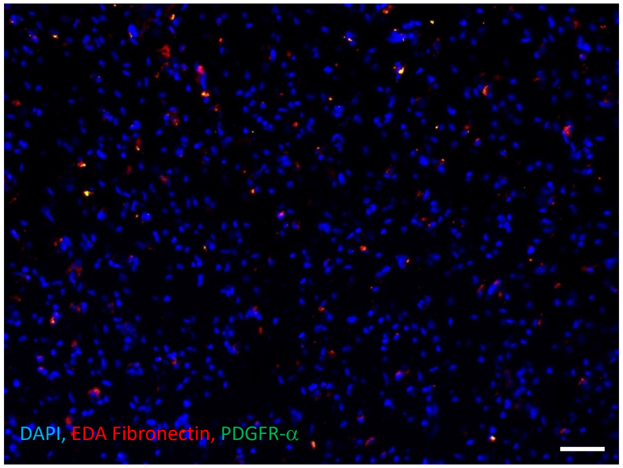

Supplemental Figure 38. Tumor-free SVZ in a GBM patient at autopsy lacks PDGFR-α staining and has sparse EDA staining. Related to Figure 6E. Shown is EDA (red), PDGFR-a (green), and DAPI (blue) staining of tumor-free SVZ taken from a GBM patient at autopsy. No PDGFR-α staining was seen and EDA staining was sparse. 100x magnification, scale bar 30 μm.

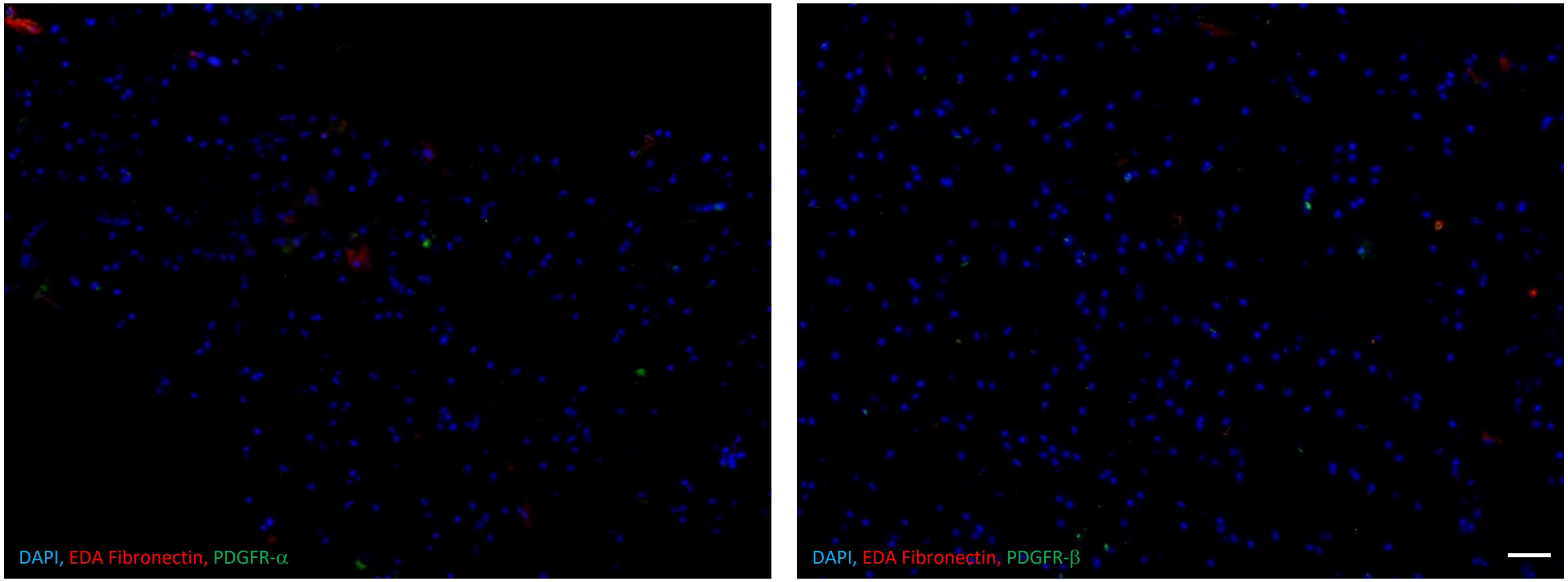

Supplemental Figure 39. SVZ from tumor-free brain lacks PDGFR-α, PDGFR-β and EDA immunopositivity. Related to Figure 6E. Shown is the SVZ from tumor-free brain tissue resected during epilepsy surgery revealing no PDGFR-β or EDA immunopositivity. 100x magnification, scale bar 30 μm.

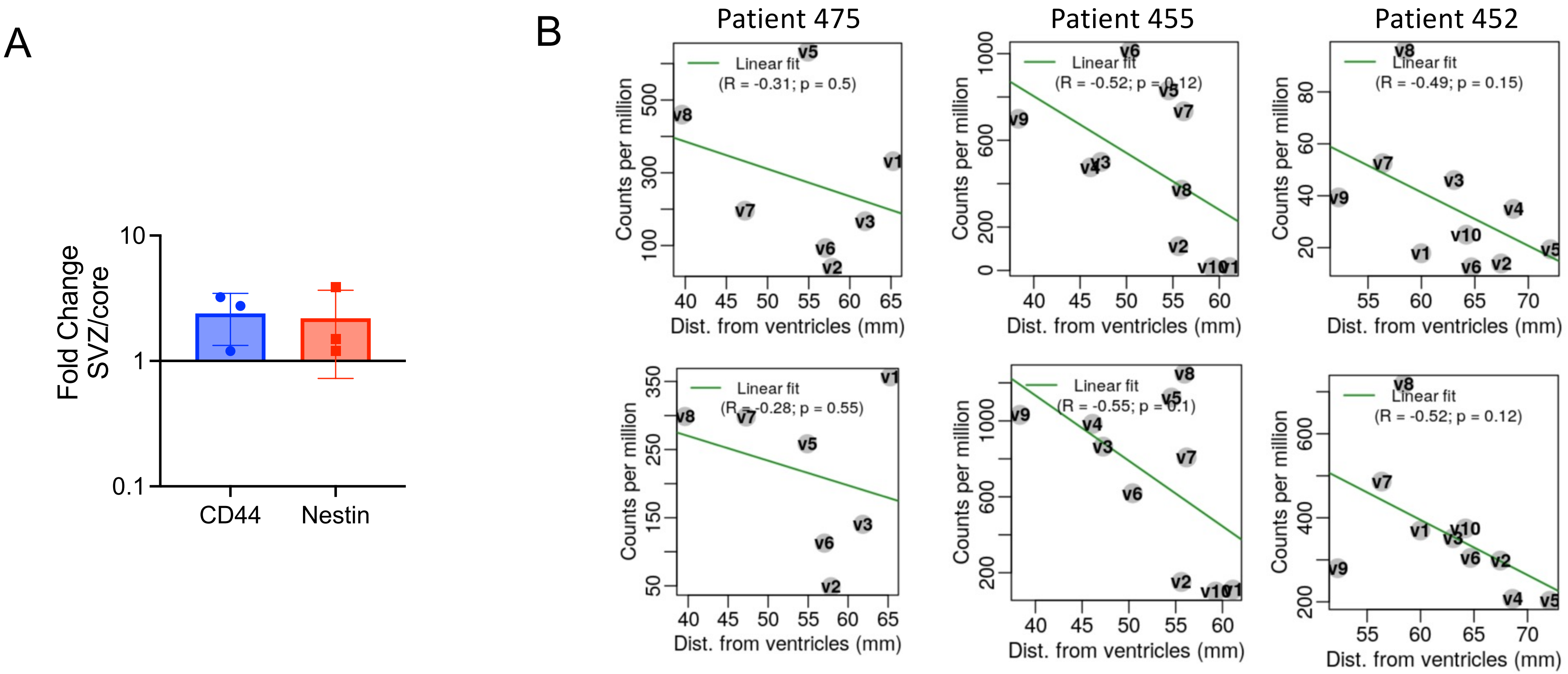

Supplemental Figure 40. Patient GBM-containing SVZ does not express more GBM stem cell markers than tumor core. Related to Figure 6E. (**A**) Shown is fold change in transcription of GSC markers C44 and nestin identified in tumor-bearing SVZ compared to tumor core in matched specimens from three patients (P=0.1; t-test). (**B**) Shown are expression of CD44 and Nestin in site-directed biopsies from three GBM patients as a function of distance from the ventricle. The correlations were not significant (Pearson; P=0.1-0.6).

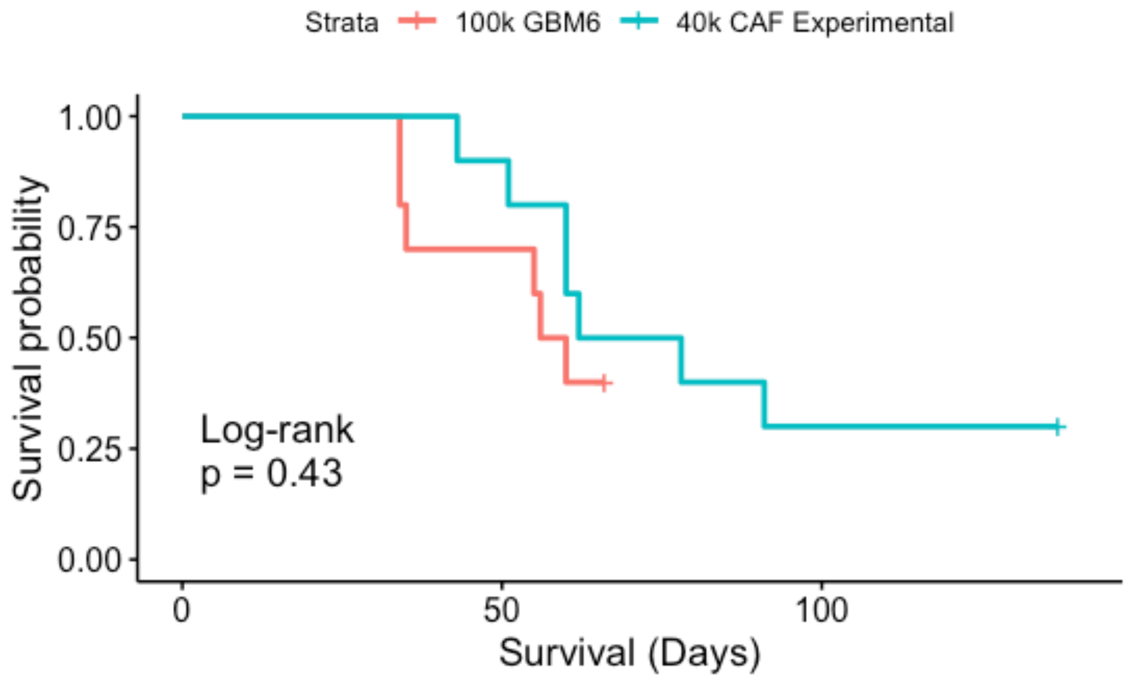

Supplemental Figure 41. Addition of CAFs to GBM6 neurospheres accelerates tumor growth. Related to Figure 7A. Shown are Kaplan-Meier curves revealing that the threshold for intracranial tumor formation in over half of mice with GBM6 neurospheres dropped from 100,000 GBM6 cells in the absence of CAFs (red curve) to 35,000 GBM6 cells when combined with 5,000 GBMpt3CAFs, creating comparable survival curves (n=10/group; P=0.4)

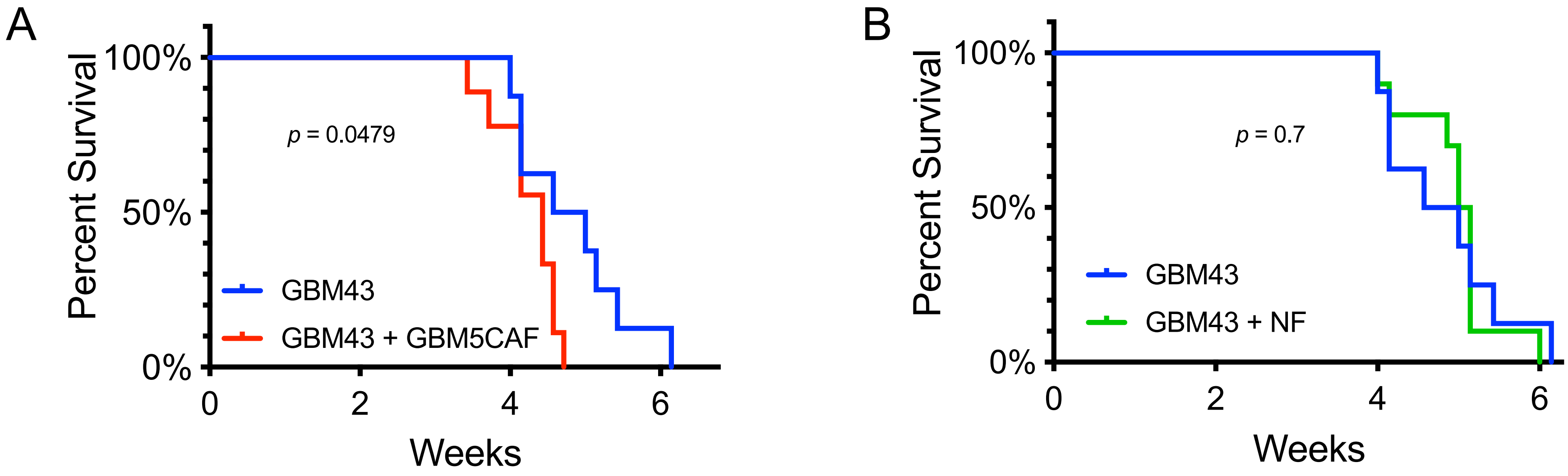

Supplemental Figure 42. CAFs worsen survival of mice with intracranial GBM43 neurosphere-derived xenografts. Related to Figure 7A. Shown are Kaplan-Meier curves illustrating the survival of mice with intracranial xenografts derived from (A) 35,000 GBM43 cells in neurospheres with and without 5,000 GBMpt5CAF cells (n=9/group) and (B) 35,000 GBM43 cells in neurospheres with and without 5,000 normal human fibroblasts (NF) (n=10/group).

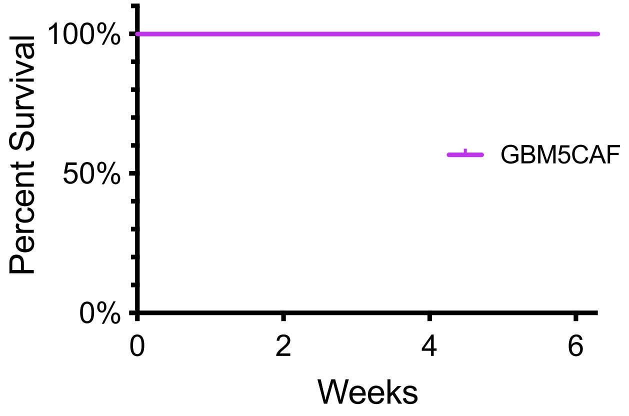

Supplemental Figure 43. Intracranially implanting GBM CAFs leads to no deaths of athymic mice. Related to Figure 7A. Shown is a Kaplan-Meier curve illustrating that no death occurred when 40,000 GBMpt5 CAF cells were implanted intracranially into athymic mice (n=9 mice).

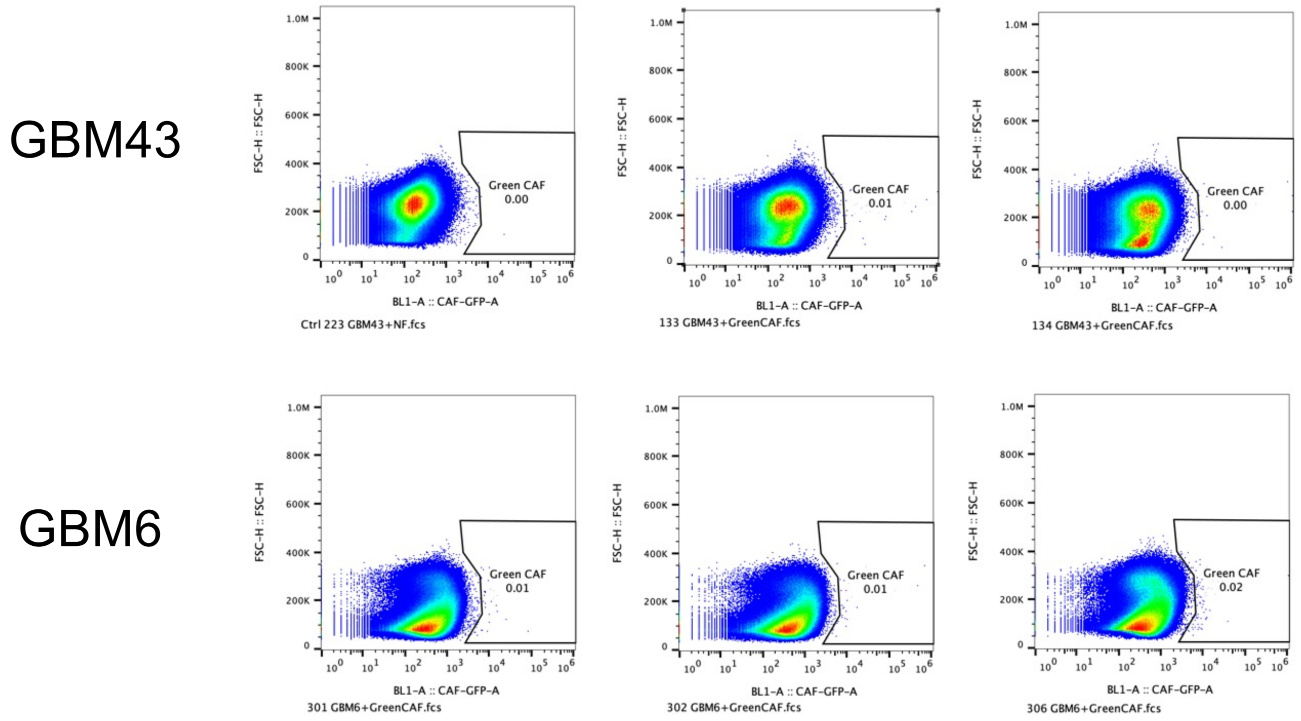

Supplemental Figure 44. CAFs are sparse at endpoint when intracranially co-implanted with GBM neurosphere-derived xenografts. Related to Figure 7A. GFP-labeled GBMpt5CAFs were co-implanted intracranially with 35,000 GBM6 or GBM43 neurospheres. When mice reached endpoint, GFP^{+} cells were undetectable in flow cytometry of single cell suspensions generated from the entire tumor (upper row = GBM43, lower row=GBM6)

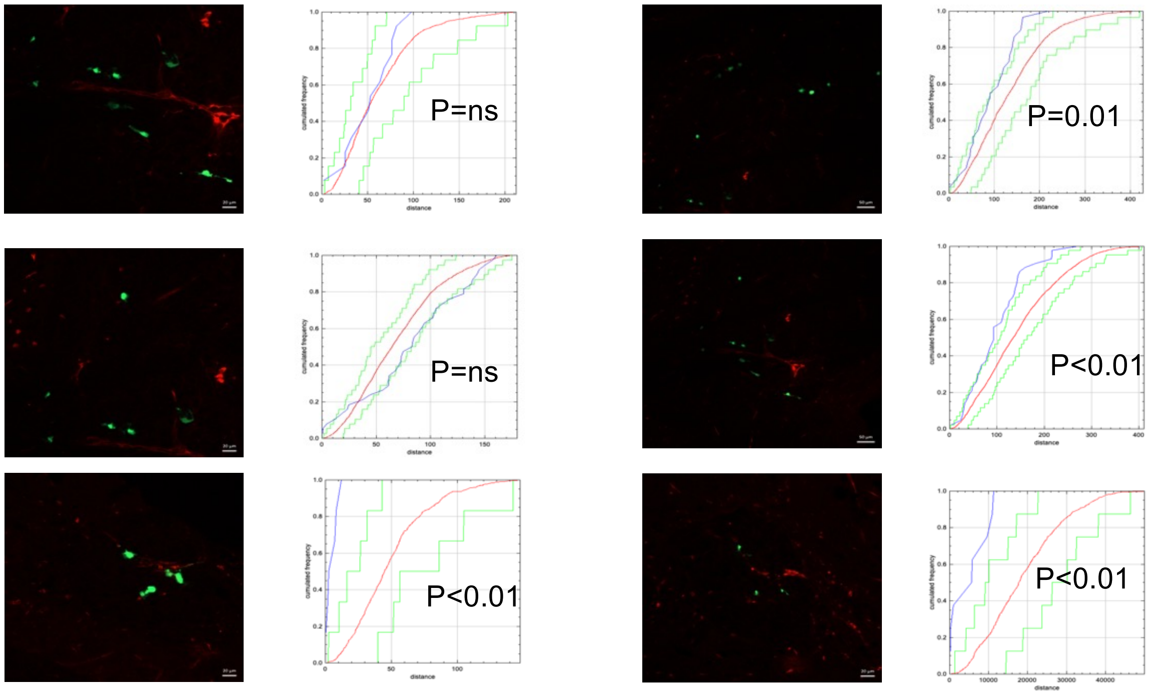

Supplemental Figure 45. CAFs are sparse at endpoint when intracranially co-implanted with GBM neurosphere-derived xenografts. Related to Figure 7A. GFP-labeled GBMpt5CAFs were co-implanted intracranially with 35,000 GBM6 or GBM43 neurospheres. When mice reached endpoint, sparse GFP^{+} cells were visualized in certain high powered fields (shown for GBM6, scale bar 20 μm). Shown are 6 high powered fields. Each field was analyzed using DiAna, an Image J tool, to determine the proximity of GFP+ CAFs to rhodamine perfused vessels. In 4 of the 6 analyze fields, CAFs were closer to vessels than expected by random chance (P<0.05)

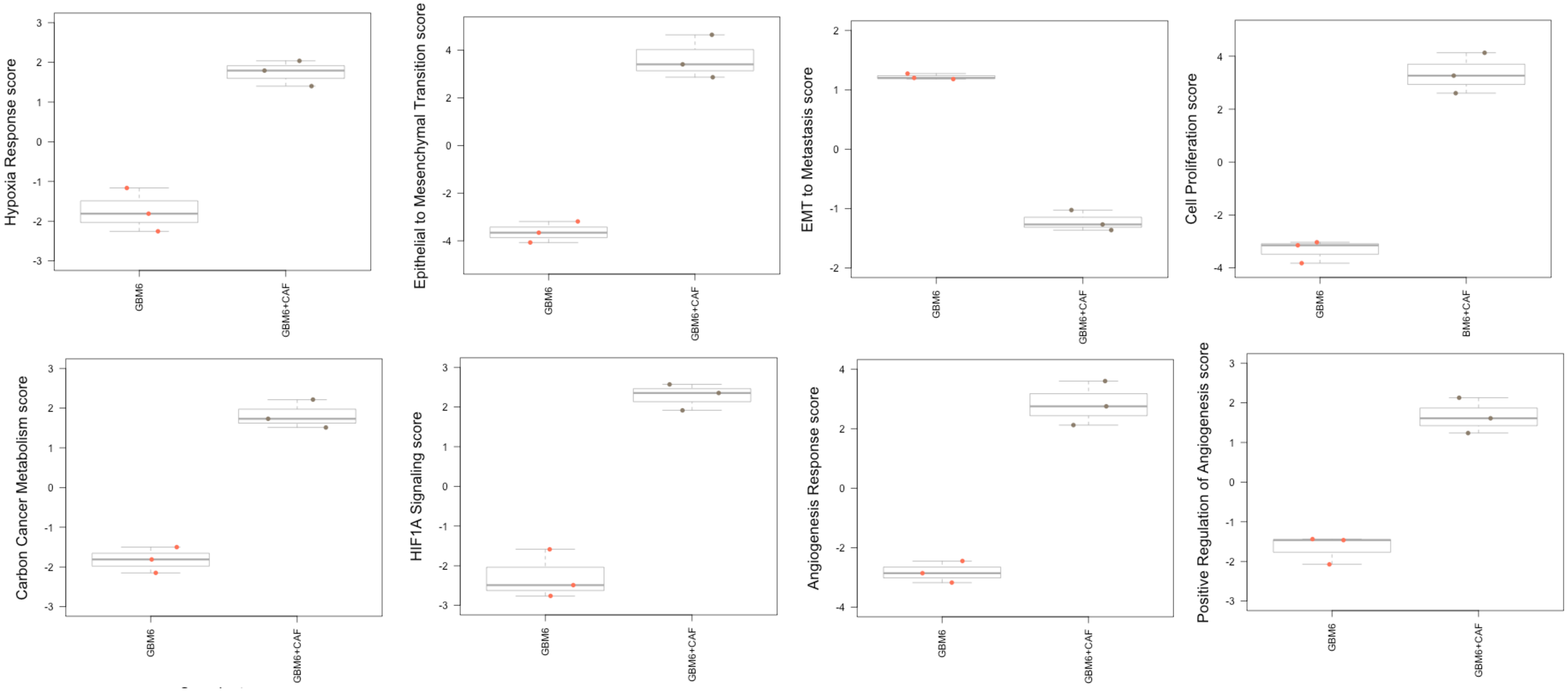

Supplemental Figure 46. Pathways activated by adding GBM CAFs to intracranial GBM6 neurosphere-derived xenografts. Related to Figures 7C-D. Shown are the effects of adding 5,000 GBMpt3CAFs to 35,000 cells in GBM6 neurospheres on gene expression pathways. Adding CAFs increased hypoxia response, EMT, cell proliferation, carbon cancer metabolism, HIF-1α signaling, angiogenesis response, and positive regulation of angiogenesis scores, while decreasing EMT to metastasis score (n=3/group).

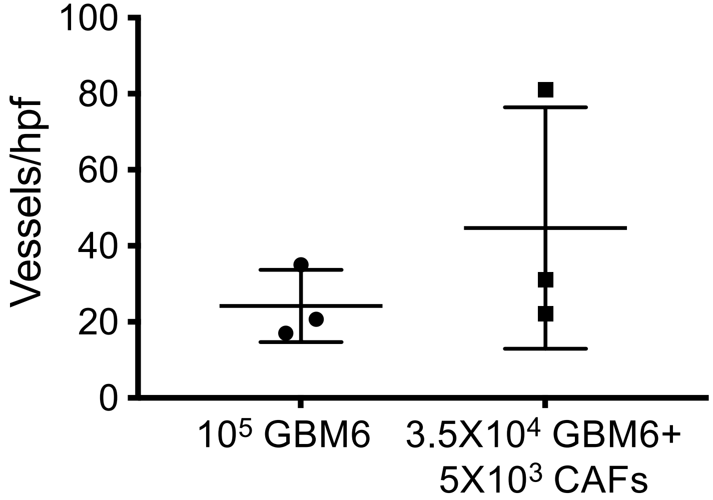

Supplemental Figure 47. Addition of CAFs to GBM6 neurospheres does not alter tumor vessel density *in vivo*. Related to Figures 6E-F. In addition to the increased vessel diameter described in **Figs. 7E-F**, addition of CAFs to GBM6 neurospheres *in vivo* decreased vessel density (P=0.3; t-test), as shown here. The net effect of the former was greater than the latter, leading to increased total vessel surface area, as shown in **Figs. 6E-F**. Shown are scatter dot plots with horizontal lines at mean and vertical lines representing standard deviations).

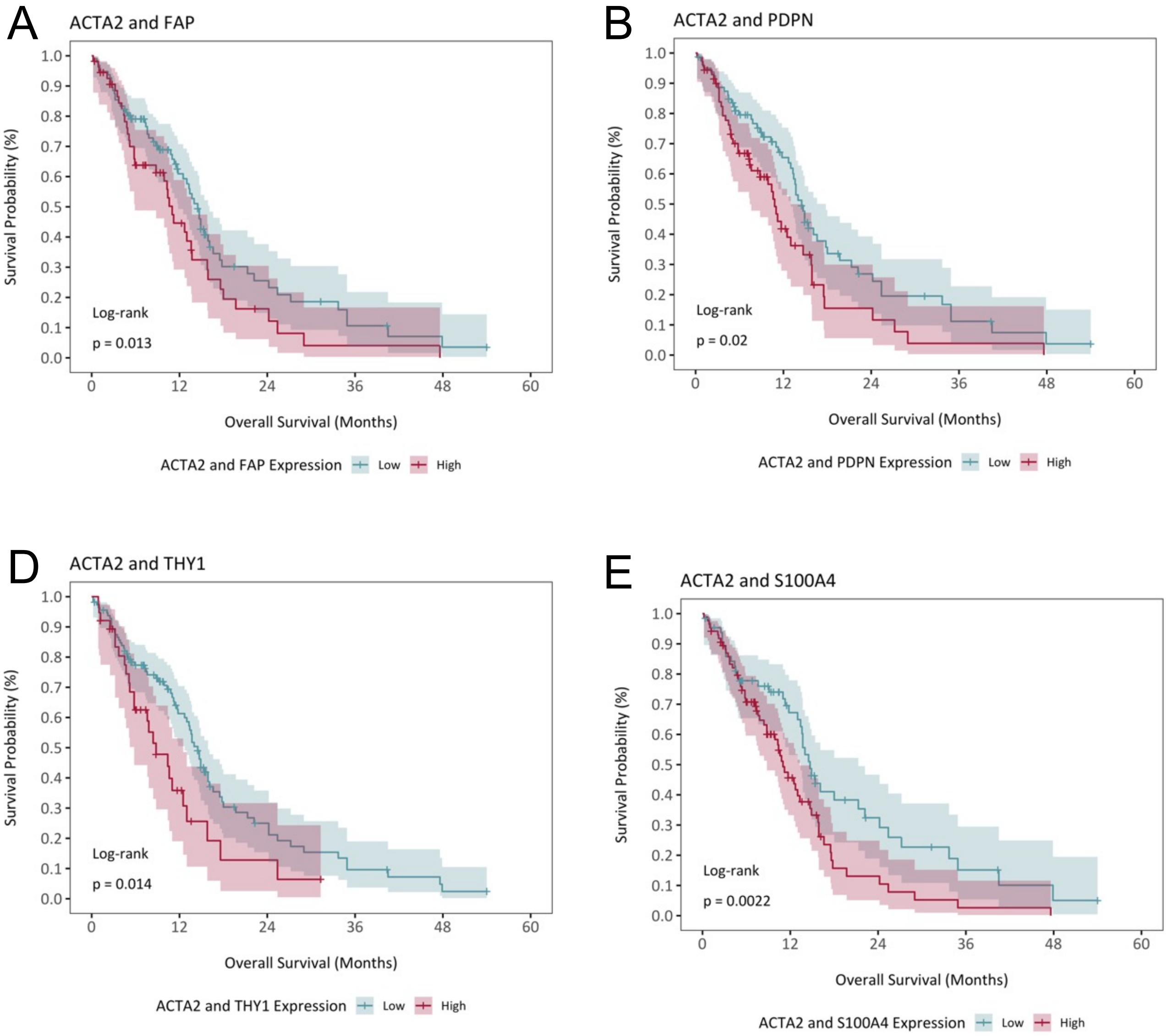

Supplemental Figure 48. Dual expression of CAF markers worsens patient survival in GBM. Related to Figure 7. GBM cases from the TCGA were analyzed with survival compared between groups categorized as high vs. low expression of CAF marker ACTA2 and one other CAF marker – (A) FAP (P=0.013); (B) PDPN (P=0.02); (C) DES (P=0.00073); (D) THY1 (P=0.014); and (E) S100A4 (P=0.0022).
