# Supplementary material for: Single-cell RNA sequencing and spatial transcriptomics reveal cancer-associated fibroblasts in glioblastoma with protumoral effects
Source: J Clin Invest. 2023 Mar 1;133(5):e147087. doi: 10.1172/JCI147087 (PMC9974099; doi:10.1172/JCI147087)
Supplement: Supplemental table 1 [file jci-133-147087-s011.pdf]

Supplemental Table 1: Results of CAF RNA-seq performed on serially trypinsized cells from two patient GBMs as compared to archived RNA-seq data from two breast cancer CAFs and iPSC-derived pericytes.

Row-names	Gene name	BC_CAF-iPSC_pericyte___log2FoldChange	BC_CAF-iPSC_pericyte___padj	GBM_CAF-iPSC_pericyte___log2FoldChange	GBM_CAF-iPSC_pericyte___padj	sum	iPSC_pericyte_83	iPSC_pericyte_84	iPSC_pericyte_85	BC_CAF1	BC_CAF2	GBM_CAF1	GBM_CAF2
ENSG00000122641	INHBA	15.23475208	4.16E-30	13.26893946	2.87E-23	28.50369154	2	2	2	15.27039578	15.252111	13.63010036	12.86013896
ENSG00000163453	IGFBP7	13.19948976	2.76E-23	13.6552506	4.13E-25	26.85474036	3.327711234	2	2	14.17896925	14.1969236	14.95324776	14.24892143
ENSG00000144810	COL8A1	12.36155052	2.88E-21	13.87852371	7.98E-27	26.24007423	2	3.076665209	2	13.3331339	13.3672165	14.60522221	15.08830309
ENSG00000164761	TNFRSF11B	12.76404613	3.65E-19	12.24862457	6.43E-18	25.0126707	2	2	2	12.78826861	12.7942119	11.47523074	12.78817145
ENSG00000103888	CEMIP	11.259841	2.01E-18	13.72078662	3.68E-27	24.98062762	2	2	3.369317972	12.21177394	12.2856726	14.61641343	14.79632422
ENSG00000133816	MICAL2	11.95029075	1.85E-20	12.87016173	8.36E-24	24.82045248	2	2	2	11.96799313	11.9881291	12.78215604	13.00391932
ENSG00000074416	MGLL	11.42509103	6.00E-18	13.36008498	2.09E-24	24.78517601	2	2	2	11.41586652	11.490225	13.08008705	13.63993691
ENSG00000169429	CXCL8	17.22511006	6.05E-23	6.983778924	0.000140535	24.20888898	2	2	2	17.27216181	17.2306876	4.806835018	7.893928381
ENSG00000049540	ELN	10.31101304	1.57E-13	13.27734295	6.08E-22	23.58835599	2	2	2	10.34037819	10.3433038	13.74101055	12.67493222
ENSG00000105887	MTPN	11.37783241	1.04E-18	11.85362949	2.00E-20	23.2314619	2	2	2	11.43320503	11.3789528	11.80202	11.95689865
ENSG00000134531	EMP1	10.75201886	1.21E-13	11.39984752	2.47E-15	22.15186638	2	2	2	10.78928396	10.7740351	10.57227185	11.96193261
ENSG00000205413	SAMD9	13.07258495	1.58E-24	8.876446682	6.18E-12	21.94903164	2	2	2	13.11647091	13.0825676	8.90036762	8.929158763
ENSG00000118503	TNFAIP3	11.56798782	4.62E-15	10.13474606	7.31E-12	21.70273387	3.327711234	2	2	12.52726555	12.5863798	10.03160495	11.74047552
ENSG00000158747	NBL1	10.41629231	6.94E-16	11.20882678	2.04E-18	21.62511909	2	2	2	10.40946091	10.4832045	11.2075668	11.26700563
ENSG00000198542	ITGBL1	11.28123233	1.79E-17	10.22603105	1.13E-14	21.50726338	2	2	2	11.29778296	11.3218808	9.952507193	10.5085588
ENSG00000148516	ZEB1	10.89154343	5.28E-17	10.54762294	3.56E-16	21.43916637	2	2	2	10.94606981	10.8952763	10.7065785	10.43629373
ENSG00000108691	CCL2	10.9971921	4.22E-12	10.43464146	4.84E-11	21.43183356	2	2	2	11.02737923	11.0253156	9.016300808	11.17325195
ENSG00000163531	NFASC	9.219804288	7.50E-11	11.91083054	1.34E-17	21.13063483	2	2	2	9.250166145	9.2611121	11.2693135	12.39414002
ENSG00000117228	GBP1	11.38867332	6.80E-18	9.507845495	7.07E-13	20.89651881	2	2	2	11.4169563	11.4173641	9.261890418	9.77651925
ENSG00000187134	AKR1C1	13.16450662	1.69E-23	7.308886609	5.85E-08	20.47339323	2	2	2	13.1714883	13.2112425	7.071691703	7.618424749
ENSG00000136235	GPNMB	11.06733702	1.54E-16	9.393327834	2.82E-12	20.46066485	2	2	3.369317972	12.0312718	12.0820105	10.70622743	9.973214279
ENSG00000132274	TRIM22	10.38544843	1.12E-19	9.978505426	1.94E-18	20.36395386	2	3.686303414	2	12.2506787	12.2240915	11.97684375	11.66847267
ENSG00000133048	CHI3L1	11.44508223	9.45E-09	8.829573988	1.33E-05	20.27465622	2	2	2	11.44969881	11.4969662	9.802049415	5.390901532
ENSG00000119922	IFIT2	14.56591798	2.56E-28	5.684368489	3.93E-05	20.25028647	2	3.076665209	2	15.55008331	15.5583383	6.363520437	7.019463152
ENSG00000000971	CFH	10.39323397	1.80E-12	9.768149176	3.53E-11	20.16138315	3.327711234	2	2	11.35280306	11.4136982	9.714689414	11.35932363
ENSG00000178860	MSC	10.98784246	1.27E-16	9.084770198	9.47E-12	20.07261266	2	2	2	11.00320274	11.0306754	8.825193025	9.367379581
ENSG00000162783	IER5	9.179123658	2.13E-12	10.65874127	1.60E-16	19.83786493	2	2	2	9.18717761	9.24275568	10.79309453	10.57593535
ENSG00000173210	ABLIM3	10.03820491	9.72E-15	9.759188532	4.18E-14	19.79739345	2	2	2	10.07805004	10.0618152	9.843290512	9.738960951
ENSG00000151846	PABPC3	9.049221252	6.75E-12	10.69040209	2.29E-16	19.73962335	2	2	2	9.093229054	9.07923325	10.53150331	10.88707283
ENSG00000147883	CDKN2B	8.754122749	4.80E-11	10.97417692	6.17E-17	19.72829967	2	2	2	8.784246083	8.80280692	10.75048495	11.21848118
ENSG00000073756	PTGS2	14.20279878	1.26E-20	5.511656368	0.000648354	19.71445514	2	2	2	14.24911738	14.2096019	6.280431262	4.532869193
ENSG00000154678	PDE1C	8.316084246	1.39E-09	11.39424826	2.60E-17	19.71033251	2	2	2	8.365459376	8.35477078	10.98841621	11.75610955
ENSG00000180914	OXTR	5.67763549	3.00E-05	14.02151181	1.21E-27	19.6991473	2	2	2	5.720439317	5.89549381	14.17941331	13.903882
ENSG00000133121	STARD13	10.14445215	1.35E-14	9.542861265	3.97E-13	19.68731342	2	2	2	10.16727262	10.1843212	9.364829867	9.761669384
ENSG00000119138	KLF9	9.138482047	3.06E-12	10.51951401	4.83E-16	19.65799605	2	2	2	9.157231967	9.19229617	10.67475396	10.41281336
ENSG00000140945	CDH13	8.827190941	1.04E-11	10.76292001	4.37E-17	19.59011095	2	3.076665209	2	9.79991149	9.84325683	11.82235644	11.67962039
ENSG00000113739	STC2	8.818404267	6.69E-40	10.5213964	3.56E-57	19.33980067	4.467311984	4.11352228	3.369317972	12.46477506	12.5147103	14.02649715	14.34091864
ENSG00000148677	ANKRD1	8.209103465	1.88E-09	10.94031107	3.46E-16	19.14941454	2	2	2	8.260583448	8.248381	11.27737127	10.57769608
ENSG00000111801	BTN3A3	10.82075211	5.42E-17	8.310620088	1.74E-10	19.1313722	2	2	2	10.81199516	10.8875237	8.354863925	8.354591622
ENSG00000091592	NLRP1	8.254351061	1.43E-09	10.83694694	6.11E-16	19.091298	2	2	2	8.266399315	8.33118782	10.48750758	11.16624784
ENSG00000156804	FBXO32	9.548020166	1.92E-14	9.531478438	1.61E-14	19.0794986	2	3.076665209	3.369317972	11.41978589	11.4048184	11.86528459	10.69530541
ENSG00000213923	CSNK1E	9.630241292	9.03E-14	9.437873222	2.32E-13	19.06811451	2	3.076665209	2	10.62961854	10.6144921	10.47258492	10.3865723
ENSG00000182752	PAPPA	9.560164219	3.83E-23	9.447605902	7.54E-23	19.00777012	2	3.686303414	3.369317972	11.96188328	12.0424814	12.08435998	11.66599202
ENSG00000140853	NLRC5	10.21539356	5.49E-15	8.705728659	3.27E-11	18.92112222	2	2	2	10.19396441	10.2972338	8.612361808	8.867684177
ENSG00000139278	GLIPR1	9.253883196	1.95E-14	9.652343393	9.35E-16	18.90622659	2	3.686303414	2	11.09675311	11.0956404	11.06680613	11.82322502
ENSG00000154175	ABI3BP	9.239273217	9.06E-13	9.666878124	5.46E-14	18.90615134	2	2	3.369317972	10.21165595	10.2525441	10.66635607	10.65083574
ENSG00000135318	NT5E	9.858199049	1.63E-11	9.031998025	7.38E-10	18.89019707	2	5.946638857	2	14.18294647	14.1959195	13.23119452	13.48460951
ENSG00000188219	POTEE	9.590275282	2.32E-12	9.245185667	1.29E-11	18.83546095	2	2	2	9.55186119	9.69266358	8.864319213	9.603790255
ENSG00000146648	EGFR	9.105618931	5.89E-12	9.701555966	1.53E-13	18.8071749	3.327711234	2	2	10.04290398	10.1531049	10.44857879	10.90235372
ENSG00000013588	GPRC5A	7.294522805	4.10E-08	11.50590051	5.93E-19	18.80042332	2	2	2	7.285019958	7.42465798	11.39429296	11.66177264
ENSG00000112837	TBX18	7.732695523	4.40E-09	11.04759459	1.05E-17	18.78029011	2	2	2	7.826439377	7.74329312	10.98195321	11.16550298
ENSG00000133805	AMPD3	10.6549223	1.56E-15	8.098957646	1.99E-09	18.75387995	2	2	2	10.66527283	10.7040796	7.825413909	8.408109518
ENSG00000188375	H3F3C	10.43070934	1.45E-15	8.303199372	2.86E-10	18.73390871	2	2	2	10.46530827	10.4570431	8.195243072	8.485179747
ENSG00000008517	IL32	8.496873615	5.90E-11	10.23416791	1.44E-15	18.73104152	3.327711234	2	2	9.459264257	9.52609769	11.22425456	11.22523682
ENSG00000253159	PCDHGA12	10.14766264	2.97E-13	8.564899689	9.39E-10	18.71256233	2	2	2	10.14850007	10.2089163	8.061837451	9.0005841
ENSG00000183023	SLC8A1	7.900319979	6.87E-09	10.7762413	7.32E-16	18.67656128	2	2	2	7.987681034	7.91175216	10.45095555	11.09046346
ENSG00000154027	AK5	9.310228768	7.18E-12	9.324184924	5.62E-12	18.63441369	2	2	2	9.366644887	9.32403997	9.653364877	8.989564175
ENSG00000163347	CLDN1	9.419440255	6.84E-12	9.165794268	2.28E-11	18.58523452	2	2	2	9.521380925	9.38345569	8.772510235	9.532624709
ENSG00000136869	TLR4	8.317420613	4.15E-09	10.17636352	2.96E-13	18.49378413	2	3.076665209	2	9.338394921	9.29056951	10.49646885	11.62307742
ENSG00000196611	MMP1	13.04516608	3.79E-19	5.441822874	0.000400501	18.48698895	2	3.076665209	3.369317972	14.90812579	14.8999667	8.112172997	5.540219242
ENSG00000136244	IL6	13.9185218	1.08E-26	4.474859558	0.00134027	18.39338136	2	2	2	13.93497504	13.9554865	4.5389195	4.911532223
ENSG00000163739	CXCL1	13.71917198	8.26E-15	4.651016124	0.016089489	18.3701881	2	2	2	13.75359206	13.7383793	3.10571689	5.661099865
ENSG00000167264	DUS2	8.168931648	1.33E-09	10.14807202	2.04E-14	18.31700367	2	2	2	8.164192202	8.26379462	9.904757465	10.4101208
ENSG00000135480	KRT7	6.848888144	2.55E-07	11.45452737	5.71E-19	18.30341551	2	2	2	6.846474326	6.99676009	11.54710573	11.4157153
ENSG00000090339	ICAM1	10.09012651	1.82E-31	8.201913658	2.82E-21	18.29204017	2	4.710564154	4.058998049	13.69222093	13.741006	11.20657426	12.26354895
ENSG00000163661	PTX3	10.31366559	1.87E-15	7.965004277	1.18E-09	18.27866986	2	2	2	10.32606281	10.3626704	7.958717528	8.067092663
ENSG00000144959	NCEH1	9.499772933	1.01E-12	8.740478155	5.76E-11	18.24025109	2	3.076665209	2	10.49739553	10.4864929	9.422103197	9.992781936
ENSG00000172716	SLFN11	8.327530668	3.98E-10	9.893407033	5.27E-14	18.2209377	2	2	2	8.310225403	8.43013848	9.73525344	10.09400257
ENSG00000177409	SAMD9L	11.0274062	2.31E-21	7.123511689	1.55E-09	18.15091789	2	3.686303414	2	12.88366224	12.8667447	8.722467118	9.202055802
ENSG00000154127	UBASH3B	7.743307924	5.10E-09	10.30007287	2.33E-15	18.04338079	2	2	2	7.786523606	7.80517502	10.19848552	10.45226125
ENSG00000137809	ITGA11	8.695281512	3.89E-27	9.318137221	2.14E-31	18.01341873	3.327711234	4.442710129	2	11.82786535	11.8844426	12.66594881	12.26354895
ENSG00000169991	IFFO2	7.854233332	2.91E-09	10.14138067	6.21E-15	17.995614	2	2	2	7.885575888	7.92393105	10.28236597	10.05408645
ENSG00000101347	SAMHD1	10.30086729	1.27E-15	7.619911436	4.97E-09	17.92077873	3.327711234	2	2	11.28088322	11.3017217	8.621327277	8.624257514
ENSG00000145337	PYURF	9.239548032	1.69E-12	8.671729884	3.54E-11	17.91127792	2	2	2	9.332862708	9.21527115	8.666300387	8.756196613
ENSG00000134363	FST	9.734053505	1.83E-13	8.157688725	8.86E-10	17.89174223	3.327711234	2	2	10.71112597	10.739998	9.380756704	8.889499502
ENSG00000135424	ITGA7	4.548829003	0.001525395	13.33023503	6.97E-24	17.87906403	2	2	2	5.022023783	4.53541753	13.17471064	13.51908818
ENSG00000141574	SECTM1	10.84420053	1.12E-16	7.034504393	1.31E-07	17.87870492	2	2	2	10.83696223	10.9094332	6.936150978	7.253757517
ENSG00000150938	CRIM1	7.321373314	2.83E-10	10.55200025	1.91E-20	17.87337356	2	5.303523747	2	11.01044056	11.0396575	14.05584185	14.42690002
ENSG00000054598	FOXC1	5.99760648	7.61E-06	11.83697788	2.61E-20	17.83458436	2	2	2	6.078767801	6.14061114	11.89367234	11.83551901
ENSG00000113578	FGF1	7.157442139	7.49E-08	10.66029989	1.97E-16	17.81774203	2	2	2	7.292639438	7.14977896	10.58798261	10.78560504
ENSG00000184584	TMEM173	9.071734254	2.02E-13	8.682689487	1.90E-12	17.75442374	2	2	4.058998049	10.92446939	11.0102587	10.03141812	10.97629934
ENSG00000178726	THBD	7.508283374	3.43E-08	10.24519032	1.45E-14	17.75347369	2	2	2	7.61176303	7.51759662	9.975607996	10.52491227
ENSG00000135905	DOCK10	9.556562366	3.81E-13	8.19635623	5.85E-10	17.7529186	2	2	2	9.632130776	9.54750898	8.37208139	8.098650814
ENSG00000187688	TRPV2	9.04455143	5.63E-12	8.689380777	3.48E-11	17.73393221	2	2	2	9.03990038	9.12214832	8.801285298	8.653693725
ENSG00000091490	SEL1L3	9.046877699	3.05E-15	8.653119756	3.81E-14	17.69999746	4.00722531	2	2	10.90316293	10.9466189	10.37200212	10.67645725
ENSG00000146859	TMEM140	10.46707858	2.40E-15	7.212115459	8.01E-08	17.67919404	2	2	2	10.49450782	10.5003855	7.475964489	7.043754524
ENSG00000187678	SPRY4	9.18346534	2.39E-11	8.44175099	8.95E-10	17.62521633	3.327711234	2	2	10.1289481	10.2227847	9.808823657	8.938158123
ENSG00000005884	ITGA3	7.996148344	7.04E-15	9.617186836	2.86E-21	17.61333518	5.330594134	2	3.369317972	11.71721007	11.7879618	12.94236789	13.70408423
ENSG00000155816	FMN2	8.740808399	7.13E-11	8.789098831	5.03E-11	17.52990723	2	2	2	8.809693772	8.75042728	8.585635246	9.035944835
ENSG00000174348	PODN	7.820357985	1.21E-08	9.664394538	8.56E-13	17.48475252	2	2	2	7.932663244	7.80780625	9.346924687	9.979766365
ENSG00000240771	ARHGEF25	8.403320644	2.68E-10	9.044581588	7.64E-12	17.44790223	2	2	2	8.532433657	8.35477078	8.941912865	9.209095161
ENSG00000118523	CTGF	6.60887738	9.99E-09	10.83488509	5.42E-22	17.44376247	4.00722531	6.899080544	3.369317972	12.13173051	12.1248442	16.41885044	16.28415028
ENSG00000276547	PCDHGB5	10.4948288	5.50E-16	6.925566537	1.55E-07	17.42039534	2	2	2	10.49945465	10.5503412	6.973973146	7.021346309
ENSG00000197442	MAP3K5	9.68726639	2.01E-13	7.724670244	6.45E-09	17.41193663	2	2	2	9.711725296	9.72921087	7.927733561	7.609689469
ENSG00000102802	MEDAG	9.62706863	1.24E-13	7.777687297	2.91E-09	17.40475593	3.327711234	2	2	10.66527283	10.5710111	8.685899845	8.866637078
ENSG00000118785	SPP1	7.391376779	6.92E-05	10.00144922	3.00E-08	17.392826	2	2	2	7.412876874	7.48837134	10.88560796	7.672153316
ENSG00000176046	NUPR1	8.186907362	1.68E-11	9.205607373	2.15E-14	17.39251474	2	3.686303414	2	10.03156504	10.0323066	11.37343913	10.62660779
ENSG00000023445	BIRC3	10.11986278	5.14E-15	7.234629655	3.48E-08	17.35449244	2	2	3.369317972	11.09484773	11.1262675	8.317350121	8.162331091
ENSG00000162692	VCAM1	8.021905583	1.20E-08	9.268545422	2.76E-11	17.290451	2	3.686303414	2	9.823772619	9.84965517	9.923201804	11.71335694
ENSG00000254598	CSNK2A3	8.817349981	1.84E-11	8.459454335	1.12E-10	17.27680432	2	2	2	8.842507967	8.86981338	8.483898848	8.519561679
ENSG00000111331	OAS3	11.55000833	8.47E-19	5.723742851	2.35E-05	17.27375118	2	2	4.058998049	13.42975069	13.482197	6.791847192	8.196067955
ENSG00000176485	PLA2G16	7.886750452	3.89E-09	9.345923342	1.57E-12	17.23267379	2	2	2	7.954442493	7.91907183	9.192066532	9.548056138
ENSG00000171310	CHST11	9.228783263	2.33E-11	7.935547634	1.10E-08	17.1643309	2	2	2	9.212503151	9.31482024	8.315509958	7.554837457

ENSG00000172985	SH3RF3	8.046563591	3.23E-09	9.100578517	1.35E-11	17.14714211	2	2	2	8.017874397	8.16669309	9.370453824	8.858759519
ENSG00000253873	PCDHGA11	10.18460662	5.33E-15	6.957514663	1.53E-07	17.14212128	2	2	2	10.1837567	10.2472229	6.952030889	7.101862022
ENSG00000104881	PPP1R13L	6.767861479	2.87E-07	10.37292166	5.94E-16	17.14078314	2	3.076665209	2	7.770242853	7.79460185	11.34943455	11.37698864
ENSG00000186340	THBS2	7.669158951	4.79E-23	9.465755718	3.82E-35	17.13491467	4.00722531	3.076665209	4.058998049	10.86502241	10.878791	12.46227734	12.84506654
ENSG00000138166	DUSP5	8.344137776	4.26E-10	8.755473594	4.54E-11	17.09961137	2	2	3.369317972	9.332862708	9.34954609	9.977742731	9.480189131
ENSG00000224831	TMEM183B	8.635649523	5.06E-11	8.422582887	1.42E-10	17.05823241	2	2	2	8.683521494	8.66883353	8.464101787	8.466640307
ENSG00000137959	IFI44L	13.02778908	3.52E-24	3.968812775	0.004363792	16.99660186	2	2	2	13.05661732	13.053069	4.331190017	4.30448772
ENSG00000133106	EPSTI1	11.05812323	8.69E-17	5.923652631	1.66E-05	16.98177586	2	2	2	11.07233814	11.1018181	5.758846871	6.276965131
ENSG00000067798	NAV3	8.907953406	1.25E-26	8.053810183	3.93E-22	16.96176359	4.00722531	2	4.058998049	11.76814309	11.8176385	11.02115097	10.85456511
ENSG00000196954	CASP4	10.00116352	2.98E-14	6.960147065	2.03E-07	16.96131058	2	2	2	10.03042623	10.0356777	7.182183216	6.862343163
ENSG00000152784	PRDM8	10.24244539	1.73E-14	6.698264715	9.16E-07	16.9407101	2	2	2	10.24983013	10.2967656	6.497238296	7.013798886
ENSG00000049130	KITLG	9.093647403	1.14E-10	7.773647619	4.40E-08	16.86729502	2	2	2	9.155143657	9.10513517	8.218405973	7.283877355
ENSG00000121691	CAT	8.521376892	3.04E-10	8.336701666	6.96E-10	16.85807856	2	2	2	8.535647908	8.59001038	8.112172997	8.606804877
ENSG00000006016	CRLF1	10.61783112	6.31E-13	6.213114885	4.72E-05	16.83094601	2	2	2	10.59498781	10.6987733	6.850634117	5.448021784
ENSG00000137842	TMEM62	9.69260104	2.78E-13	7.120023036	1.24E-07	16.81262408	2	2	2	9.726568065	9.72504082	7.368839097	6.977399345
ENSG00000122861	PLAU	9.556955631	4.48E-26	7.237554812	1.60E-15	16.79451044	2	4.11352228	3.369317972	12.36497354	12.4351513	10.37877362	9.716470666
ENSG00000149633	KIAA1755	9.075152744	5.27E-12	7.645921628	7.73E-09	16.72107437	2	2	2	9.086669782	9.13687201	7.633939843	7.763868281
ENSG00000138685	FGF2	9.978536548	4.43E-25	6.697690981	6.16E-12	16.67622753	3.327711234	5.457120953	2	14.07182595	14.0003157	11.00468611	10.46182695
ENSG00000185551	NR2F2	6.661820644	5.31E-14	10.00597837	1.07E-30	16.66779901	2	4.442710129	2	9.490242771	9.51325794	12.71516678	12.95259962
ENSG00000100342	APOL1	9.587776216	1.33E-33	7.021158745	1.31E-18	16.60893496	4.00722531	3.076665209	4.058998049	12.78346024	12.7982697	9.927625703	10.47700137
ENSG00000177425	PAWR	6.513479058	1.03E-06	10.01942027	9.74E-15	16.53289933	2	3.076665209	2	7.454228844	7.60793141	11.10121445	10.91342392
ENSG00000134470	IL15RA	9.921760852	1.40E-13	6.585826992	1.57E-06	16.50758784	2	2	2	9.938102197	9.96965331	6.380048704	6.911910672
ENSG00000131015	ULBP2	8.862840499	1.73E-11	7.582267024	1.06E-08	16.44510752	2	2	2	8.852852814	8.94822691	7.629009275	7.646759583
ENSG00000077238	IL4R	7.864479742	8.27E-12	8.497050272	9.93E-14	16.36153001	2	3.686303414	2	9.669187952	9.77497559	10.42416644	10.27852722
ENSG00000174032	SLC25A30	8.748323978	3.41E-11	7.610829741	9.70E-09	16.35915372	2	2	2	8.826850269	8.74768919	7.705944963	7.62463201
ENSG00000117036	ETV3	7.509993321	1.82E-08	8.844400412	1.89E-11	16.35439373	2	2	2	7.632926745	7.49817903	8.915024678	8.850309106
ENSG00000117226	GBP3	9.620498097	1.54E-23	6.723568803	4.25E-12	16.3440669	2	4.11352228	2	12.03198325	12.0687713	9.049523349	9.266987143
ENSG00000197646	PDCD1LG2	8.362630273	7.05E-09	7.974738178	3.35E-08	16.33736845	2	2	2	8.386974157	8.42500614	7.408510937	8.45344973
ENSG00000147889	CDKN2A	9.104782745	5.23E-12	7.149957604	8.41E-08	16.25474035	2	2	2	9.16555511	9.11685323	7.121690475	7.30412922
ENSG00000136542	GALNT5	9.295470675	5.41E-27	6.884886065	2.15E-15	16.18035674	2	4.442710129	2	12.1230758	12.1403237	9.781089302	9.670110091
ENSG00000155158	TTC39B	7.812198373	1.03E-08	8.36600404	6.65E-10	16.17820241	2	2	2	7.852449262	7.8745862	8.628262104	8.151194533
ENSG00000103647	CORO2B	7.978183834	3.63E-09	8.157712437	1.38E-09	16.13589627	2	2	2	8.006336225	8.04707549	8.381493453	8.000872619
ENSG00000214944	ARHGEF28	7.343638323	8.91E-08	8.778289183	8.81E-11	16.12192751	2	2	2	7.326437704	7.47849652	9.042123971	8.55118937
ENSG00000108771	DHX58	9.486177668	4.08E-13	6.627774725	6.63E-07	16.11395239	2	2	2	9.50179306	9.53882419	6.715756428	6.704867638
ENSG00000111799	COL12A1	7.999418766	1.28E-10	8.1068074	6.38E-11	16.10622617	2	5.457120953	2	11.80507162	11.8920568	11.82656184	12.07566644
ENSG00000156253	RWDD2B	7.594690618	2.18E-08	8.486367699	2.67E-10	16.08105832	2	2	2	7.626911583	7.67287587	8.322856568	8.708487604
ENSG00000105974	CAV1	6.58135894	9.98E-12	9.493125548	1.57E-23	16.07448449	3.327711234	5.457120953	2	10.64271979	10.6460801	13.30271973	13.76627258
ENSG00000180660	MAB21L1	8.156002274	1.16E-09	7.912987405	3.42E-09	16.06898968	2	2	2	8.199071993	8.20512425	8.092249024	7.820122783
ENSG00000184110	EIF3C	10.23844932	1.32E-14	5.826818252	2.21E-05	16.06526757	2	2	2	10.26201025	10.2769623	5.711121288	6.155328188
ENSG00000106366	SERPINE1	7.476436617	9.27E-08	8.541136413	6.87E-10	16.01757303	8.674867122	4.93638933	4.058998049	14.66453201	14.7005231	15.92548369	15.54374762
ENSG00000113389	NPR3	6.100704484	6.07E-06	9.915165446	2.58E-14	16.01586993	2	3.076665209	2	7.165617905	7.09482005	10.7412617	11.05447682
ENSG00000106991	ENG	8.30353082	1.13E-37	7.696301012	9.61E-33	15.99983183	3.327711234	4.710564154	4.058998049	12.12894005	12.1784379	11.39182091	11.68772122
ENSG00000262576	PCDHGA4	10.21892957	4.80E-13	5.780373237	8.13E-05	15.9993028	2	2	2	10.21967182	10.2798081	5.318934974	6.323497254
ENSG00000162496	DHRS3	9.523209818	1.29E-12	6.470521626	2.41E-06	15.99373144	2	2	2	9.574696759	9.53961588	6.779440836	6.298867018
ENSG00000126778	SIX1	6.952096722	1.41E-06	9.02758735	1.58E-10	15.97968407	2	2	2	7.119236776	6.9210099	8.536980328	9.450446099
ENSG00000188158	NHS	7.499838004	2.18E-08	8.418917018	2.19E-10	15.91875502	2	2	2	7.527093191	7.58663873	8.388512422	8.531503464
ENSG00000182253	SYNM	7.880557912	1.17E-08	7.986964212	6.49E-09	15.86752212	2	2	2	7.942383523	7.91907183	8.297599653	7.714671806
ENSG00000100302	RASD2	7.899894061	7.44E-08	7.954413572	5.36E-08	15.85430763	2	2	2	7.913023977	7.98565528	8.453542774	7.34530375
ENSG00000175745	NR2F1	6.342129786	3.56E-08	9.507789595	2.33E-17	15.84991938	2	3.076665209	3.369317972	8.201097719	8.25031674	11.31891342	11.42774592
ENSG00000181588	MEX3D	7.711632835	5.04E-09	8.119528388	6.04E-10	15.83116122	2	3.076665209	2	8.636393836	8.78689171	9.147938916	9.087636837
ENSG00000150527	CTAGE5	9.024923139	2.62E-11	6.771812774	8.44E-07	15.79673591	2	2	2	9.080080551	9.04395769	7.078941352	6.574984961
ENSG00000176692	FOXC2	4.45785955	0.00182751	11.33343421	9.82E-18	15.79129376	2	2	2	4.76150579	4.68029874	11.09165896	11.58959617
ENSG00000185022	MAFF	8.575479738	1.41E-20	7.21379253	5.37E-15	15.78927227	2	4.11352228	3.369317972	11.40294453	11.4332138	9.649231086	10.37906502
ENSG00000128340	RAC2	10.54296157	4.58E-11	5.244770427	0.001958798	15.787732	2	2	2	10.58066597	10.5655798	6.072436333	4.161535617
ENSG00000206190	ATP10A	7.55682658	1.84E-10	8.224195055	2.77E-12	15.78102163	2	3.686303414	2	9.384581425	9.43712594	10.29159999	9.820713969
ENSG00000176641	RNF152	6.140207192	6.33E-06	9.574344448	3.18E-13	15.71455164	2	2	2	6.164317909	6.32089174	9.70112864	9.508660255
ENSG00000101421	CHMP4B	7.798175668	1.32E-14	7.89563063	4.41E-15	15.6938063	2	2	4.523675391	10.28320062	10.2812288	10.13729849	10.58662664
ENSG00000028277	POU2F2	9.427391611	1.45E-10	6.244992575	3.59E-05	15.67238419	2	2	2	9.439666599	9.48394374	5.586442682	6.839042789
ENSG00000157168	NRG1	11.23958285	2.07E-14	4.430834848	0.005079404	15.6704177	2	2	2	11.25015134	11.2862384	5.19095825	3.940519005
ENSG00000003137	CYP26B1	5.997893483	1.63E-05	9.666906369	5.28E-13	15.66479985	2	2	2	6.130704144	6.08962627	9.421533019	9.933764315
ENSG00000196139	AKR1C3	9.819584212	3.83E-14	5.835082837	1.29E-05	15.65466705	2	3.076665209	2	10.72386293	10.8931098	6.900587441	6.845434848
ENSG00000253910	PCDHGB2	10.17762342	6.73E-14	5.427924336	0.00012485	15.60554775	2	2	2	10.18273194	10.2345667	5.22272385	5.867805474
ENSG00000120217	CD274	10.04037135	2.30E-14	5.536606374	4.95E-05	15.57697772	2	2	2	10.07252815	10.0717013	5.815498119	5.529673353
ENSG00000283154	IQCJ-SCHIP1	7.576066127	1.92E-08	7.976990385	2.61E-09	15.55305651	2	2	2	7.523859802	7.73225478	7.989819469	8.060697341
ENSG00000182218	HHIPL1	7.338493294	2.78E-08	8.196059906	3.77E-10	15.5345532	2	3.076665209	2	8.31397419	8.37442884	9.192400798	9.19581595
ENSG00000070371	CLTCL1	6.771629255	5.81E-07	8.748062711	4.53E-11	15.51969197	2	2	2	6.856790872	6.84106088	8.89667997	8.669391068
ENSG00000125538	IL1B	14.32195919	1.23E-26	1.18429571	0.481883257	15.5062549	2	2	2	14.34346783	14.3537526	2.492221014	2.817676617
ENSG00000078098	FAP	9.26323256	7.20E-14	6.235629274	7.90E-07	15.49886183	2	3.686303414	2	11.08938989	11.1114346	8.471282103	7.574209638
ENSG00000134107	BHLHE40	8.425802229	2.13E-25	7.070199799	2.25E-18	15.49600203	2	3.076665209	5.156626401	11.90336716	11.9204473	10.59305515	10.52341813
ENSG00000141052	MYOCD	4.197644785	0.003288418	11.2797101	7.23E-18	15.47735489	2	2	2	4.574143677	4.43005853	11.12828449	11.46843325
ENSG00000251322	SHANK3	7.604786314	2.30E-07	7.87075027	7.16E-08	15.47553658	2	2	2	7.565338955	7.74878077	8.352472895	7.302581437
ENSG00000004799	PDK4	9.905885481	1.21E-09	5.553542327	0.001134029	15.45942781	2	2	2	9.942952235	9.93336537	6.375345632	4.365060851
ENSG00000135454	B4GALNT1	8.12427994	2.70E-09	7.327564663	8.96E-08	15.4518446	2	2	2	8.017874397	8.30907138	7.552954291	7.203223239
ENSG00000121310	ECHDC2	7.214382946	1.26E-07	8.215996542	1.14E-09	15.43037949	2	2	2	7.173892946	7.37584259	8.108635266	8.399447278
ENSG00000164823	OSGIN2	8.281955824	8.81E-19	7.125328613	2.69E-14	15.40728444	3.327711234	3.076665209	3.369317972	10.75935206	10.7163854	9.608982234	9.561707646
ENSG00000106153	CHCHD2	7.613167438	2.82E-15	7.793812376	4.11E-16	15.40697981	2	4.11352228	2	10.07031346	10.0261059	10.25973689	10.19631519
ENSG00000101825	MXRA5	10.9224266	1.23E-16	4.462669624	0.001487806	15.38509622	2	2	2	10.94788047	10.9555451	4.90146639	4.522269241
ENSG00000105376	ICAM5	5.402727663	9.53E-05	9.971712526	3.74E-14	15.37444019	2	2	2	5.37770462	5.71663161	9.998153538	10.00924387
ENSG00000120875	DUSP4	7.484565651	2.89E-08	7.882116774	4.12E-09	15.36668243	3.327711234	2	2	8.553199415	8.4215744	9.084881607	8.64700078
ENSG00000159216	RUNX1	8.433136852	3.45E-18	6.904846874	1.29E-12	15.33798373	2	4.11352228	2	10.85186662	10.8759728	9.230659332	9.444493373
ENSG00000189320	FAM180A	8.038850326	2.26E-09	7.26275343	7.33E-08	15.30160376	2	2	2	8.010962573	8.15847021	7.244115921	7.402777233
ENSG00000108932	SLC16A6	11.64797754	9.99E-16	3.628800204	0.02347325	15.27677774	2	2	2	11.67720334	11.6750476	3.429858444	4.489993931
ENSG00000131979	GCH1	9.216167288	1.28E-11	6.058051462	1.45E-05	15.27421875	2	2	2	9.293531584	9.20931286	5.85994354	6.420873184
ENSG00000233954	UQCRHL	7.359022784	3.76E-08	7.904323544	2.62E-09	15.26334633	3.327711234	2	2	8.401141138	8.32752521	8.740866084	9.051693903
ENSG00000188483	IER5L	7.64479529	4.33E-08	7.59398741	4.84E-08	15.2387827	2	2	2	7.647855834	7.74878077	7.91644678	7.321046127
ENSG00000188522	FAM83G	6.884989229	2.36E-07	8.348320164	1.95E-10	15.23330939	2	3.076665209	2	7.898117066	7.89700047	9.258701797	9.4271968
ENSG00000130513	GDF15	8.427785472	1.90E-08	6.801399962	7.63E-06	15.22918543	2	5.303523747	2	12.05134158	12.1252398	11.18431183	8.965802436
ENSG00000184979	USP18	10.92916137	1.66E-16	4.274930422	0.002609328	15.20409179	2	2	2	10.95600041	10.9608743	4.340871764	4.766376438
ENSG00000138134	STAMBPL1	9.464317437	4.51E-12	5.737698263	4.86E-05	15.2020157	2	2	2	9.516508835	9.48064953	6.148830293	5.513708555
ENSG00000105976	MET	7.224611017	4.53E-14	7.974427665	4.37E-17	15.19903868	2	2	4.874587708	10.13796435	10.1458442	10.74730333	11.01921956
ENSG00000128284	APOL3	7.684612857	2.41E-08	7.505557164	4.76E-08	15.19017002	2	2	3.369317972	8.664561167	8.70880006	8.121330705	8.815461059
ENSG00000137266	SLC22A23	6.400944072	5.70E-08	8.768567553	2.96E-14	15.16951163	3.327711234	3.076665209	2	8.332573384	8.22887947	10.40713106	10.83046661
ENSG00000164161	HHIP	4.734297451	0.000676627	10.42810508	1.21E-15	15.16240253	2	2	2	5.040242897	4.87352701	10.51047766	10.40472054
ENSG00000123096	SSPN	6.874300857	4.70E-07	8.284503889	6.91E-10	15.15880475	2	2	2	6.946463121	6.94988431	8.460220609	8.184349581
ENSG00000261934	PCDHGA9	10.13713138	1.54E-14	5.01972191	0.000282184	15.15685329	2	2	2	10.14060551	10.1959181	5.04460184	5.361470313
ENSG00000093010	COMT	7.681166332	1.30E-09	7.465241816	3.60E-09	15.14640815	5.330594134	2	2	11.18537258	11.2610183	10.72748832	11.24299785
ENSG00000164619	BMPER	7.74583155	2.01E-07	7.393011799	7.04E-07	15.13884335	2	3.076665209	2	8.747293529	8.74768919	7.511762185	8.943630224
ENSG00000214193	SH3D21	6.310659996	4.11E-06	8.827213561	3.64E-11	15.13787356	2	2	2	6.252910529	6.54560135	8.853791658	8.878113577
ENSG00000176928	GCNT4	6.076952483	3.19E-05	9.026969493	1.63E-10	15.10392198	2	2	2	6.096288482	6.26836889	9.445569009	8.544002356
ENSG00000132334	PTPRE	8.638193896	1.58E-10	6.450274621	2.69E-06	15.08846852	2	2	2	8.618316353	8.73668456	6.713894915	6.341697848
ENSG00000179862	CITED4	5.969874025	2.29E-05	9.056174793	2.91E-11	15.02604882	2	2	2	6.024891728	6.14061114	8.7818697	9.349127298
ENSG00000149596	JPH2	4.19614938	0.00341118	10.80012163	2.30E-16	14.99627101	2	2	2	4.299492648	4.68029874	10.80854976	10.8504662
ENSG00000137573	SULF1	7.218065709	1.02E-06	7.739230696	1.27E-07	14.95729641	3.327711234	6.78762695	2	12.46372082	12.5019715	12.99447815	13.01308674
ENSG00000115290	GRB14	9.714462343	1.20E-13	5.242445231	0.000121751	14.95690757	2	2	2	9.789178534	9.70471389	5.4588802	5.361470313
ENSG00000183486	MX2	12.28646142	9.04E-17	2.658180048	0.115813971	14.94464147	2	2	2	12.30065678	12.3271867	3.782262616	2.817676617
ENSG00000147251	DOCK11	7.433108751	3.49E-07	7.501373983	2.42E-07	14.93448273	2	2	2	7.580980221	7.39696604	7.946086551	7.026981076
ENSG00000166401	SERPINB8	8.455540409	5.36E-09	6.471507249	1.14E-05	14.92704766	2	2	3.369317972	9.449924671	9.45402153	6.741568503	7.98359008

ENSG00000008441	NFIX	6.256515292	3.13E-08	8.634499079	6.16E-15	14.89101437	2	5.131603625	2	9.794554993	9.78702715	11.79949881	12.45392616
ENSG00000171812	COL8A2	5.35569622	5.87E-07	9.522169315	3.85E-20	14.87786553	2	4.11352228	2	7.794595599	7.7919464	11.53621767	12.24704688
ENSG00000149571	KIRREL3	5.991284318	1.94E-05	8.880687345	6.20E-11	14.87197166	2	3.076665209	2	6.877205252	7.15805139	9.515898077	10.16237085
ENSG00000241978	AKAP2	11.96533226	5.82E-20	2.903961037	0.048142698	14.8692933	2	2	2	11.96710062	12.0186877	3.411599105	3.660199087
ENSG00000148841	ITPRIP	8.845312785	5.86E-34	5.995410793	3.04E-16	14.84072358	4.815609471	3.686303414	2	12.27263705	12.3259253	9.346924687	9.558793178
ENSG00000197971	MBP	6.515712628	1.06E-06	8.324315508	2.05E-10	14.84002814	2	3.076665209	2	7.565338955	7.50468064	9.311972588	9.33102078
ENSG00000188211	NCR3LG1	8.11500381	1.43E-08	6.719211803	3.44E-06	14.83421561	2	2	2	8.223194971	8.09740995	6.322573937	7.156006254
ENSG00000078401	EDN1	6.649513771	1.06E-06	8.182619018	9.81E-10	14.83213279	2	2	2	6.744602355	6.71776877	8.156706039	8.296681143
ENSG00000253506	NACA2	8.777887517	3.78E-11	6.04972769	8.54E-06	14.82761521	2	2	2	8.756965077	8.87484109	6.118193494	6.199243628
ENSG00000111335	OAS2	11.06584019	2.92E-19	3.696813311	0.005582023	14.7626535	4.00722531	2	2	12.94659359	12.9593671	5.158477451	6.092227206
ENSG00000134899	ERCC5	9.569735008	3.50E-13	5.190094993	0.000153911	14.75983	2	2	2	9.595637371	9.61140668	5.269098128	5.453611367
ENSG00000112769	LAMA4	7.594891898	1.58E-21	7.143787682	2.35E-19	14.73867958	4.467311984	4.11352228	6.073378139	12.50078528	12.5634025	12.5050204	11.47994833
ENSG00000073910	FRY	5.397317071	0.000146384	9.341278642	5.70E-12	14.73859571	2	2	2	5.639097847	5.45984938	9.075851216	9.624999381
ENSG00000006210	CX3CL1	1.599854907	0.390522475	13.11117997	2.83E-19	14.71103488	2	2	2	3.162721474	2.38539787	13.00940021	13.25640611
ENSG00000111110	PPM1H	4.868690713	0.001915398	9.826405338	2.30E-11	14.69509605	2	2	2	4.965943888	5.18011485	9.042123971	10.37741184
ENSG00000076706	MCAM	7.020728799	2.08E-09	7.669660597	4.24E-11	14.6903894	4.00722531	3.076665209	2	9.464333207	9.50192864	10.70130335	9.167185134
ENSG00000102287	GABRE	7.115720141	4.72E-07	7.535195713	7.60E-08	14.65091585	2	2	2	7.186217188	7.17852729	7.258234779	7.862758575
ENSG00000196639	HRH1	6.455199689	2.14E-08	8.158073287	6.04E-13	14.61327298	2	3.076665209	3.369317972	8.354580458	8.32017198	10.04499344	10.0106665
ENSG00000169432	SCN9A	8.442527715	1.33E-10	6.16774534	4.16E-06	14.61027305	3.327711234	2	2	9.476928206	9.40011621	7.121690475	7.266516281
ENSG00000089127	OAS1	12.11453806	7.27E-18	2.489400316	0.123275594	14.60393837	2	2	2	12.14809842	12.1362731	3.584558147	2.885414003
ENSG00000158301	GPRASP2	6.846868502	5.55E-07	7.754509131	9.42E-09	14.60137763	2	2	2	6.936770382	6.90635305	7.718996793	7.889811111
ENSG00000135828	RNASEL	7.179294241	1.33E-07	7.399000589	4.53E-08	14.57829483	2	2	2	7.246306984	7.24216308	7.415399995	7.500519913
ENSG00000051382	PIK3CB	7.257595836	5.25E-08	7.320217753	3.56E-08	14.57781359	3.327711234	2	2	8.182762967	8.34212018	8.369718748	8.281832879
ENSG00000147459	DOCK5	7.146691462	2.90E-19	7.422439378	6.41E-21	14.56913084	3.327711234	3.686303414	4.058998049	10.33669748	10.3437572	10.34082931	10.84569152
ENSG00000167785	ZNF558	6.994663538	1.32E-06	7.573453347	1.24E-07	14.56811689	2	2	2	6.912249745	7.20272083	7.951626215	7.213143202
ENSG00000057657	PRDM1	9.921878738	2.55E-12	4.622334841	0.002135746	14.54421358	2	2	2	9.959800107	9.94834802	5.248671144	4.329022995
ENSG00000184675	AMER1	6.688641392	1.57E-06	7.852149147	1.04E-08	14.54079054	2	2	2	6.622948477	6.90143415	7.738355777	8.050589918
ENSG00000156642	NPTN	7.251733481	3.24E-07	7.276247254	2.69E-07	14.52798073	2	2	5.59507254	11.07923811	11.0764016	10.94185947	11.24662458
ENSG00000253485	PCDHGA5	10.13839488	1.05E-14	4.388851436	0.001648343	14.52724632	2	2	2	10.13690654	10.2019318	4.563936166	4.757365452
ENSG00000168404	MLKL	9.020994699	1.09E-11	5.497460614	6.18E-05	14.51845531	2	2	2	9.073461088	9.04284131	5.54096877	5.740981724
ENSG00000101333	PLCB4	6.886272462	3.76E-07	7.605804823	1.42E-08	14.49207728	2	2	3.369317972	7.90310317	7.89452713	8.787633372	8.40450657
ENSG00000119681	LTBP2	7.078309556	5.84E-07	7.381190925	1.59E-07	14.45950048	2	2	5.59507254	10.86054366	10.9469174	11.14176121	11.26879021
ENSG00000113070	HBEGF	8.052448369	1.37E-08	6.390311855	9.00E-06	14.44276022	2	2	2	8.096156705	8.10384772	6.072436333	6.802276223
ENSG00000006327	TNFRSF12A	7.229321172	1.21E-11	7.211576627	1.18E-11	14.4408978	3.327711234	5.72254323	2	11.4916791	11.5486124	11.46772494	11.53684257
ENSG00000137801	THBS1	6.713056322	2.00E-06	7.708393902	3.21E-08	14.42145022	10.15425007	8.469613413	4.058998049	15.65859347	15.6962604	16.69977347	16.64533661
ENSG00000228672	PROB1	6.725089719	2.25E-06	7.692871881	4.09E-08	14.4179616	2	2	2	6.710878132	6.89154576	8.007404089	7.427130611
ENSG00000164465	DCBLD1	7.359613594	3.17E-14	7.052884194	3.06E-13	14.41249779	2	4.11352228	2	9.809901631	9.77967427	9.45312092	9.525344379
ENSG00000204956	PCDHGA1	10.15456767	7.57E-14	4.251128495	0.003453531	14.40569616	2	2	2	10.15530726	10.2158672	4.230621763	4.810605986
ENSG00000261609	GAN	5.859625691	0.00010005	8.535071444	4.36E-09	14.39469714	2	2	2	6.024891728	5.93454154	9.011762258	7.950369405
ENSG00000179588	ZFPM1	6.49009794	2.28E-06	7.897678118	4.76E-09	14.38777606	2	2	2	6.535745081	6.61935067	7.890311949	8.002780183
ENSG00000006468	ETV1	8.912993781	2.62E-11	5.474163754	7.50E-05	14.38715754	2	2	2	8.979812467	8.92175022	5.755231204	5.475755622
ENSG00000107201	DDX58	9.900908506	4.82E-26	4.465416267	4.44E-06	14.36632477	2	4.93638933	2	13.24904661	13.2653088	7.660277586	8.011333147
ENSG00000262209	PCDHGB3	10.17333512	6.59E-15	4.179832038	0.002761417	14.35316716	2	2	2	10.1755382	10.2330992	4.530483275	4.445805334
ENSG00000099998	GGT5	5.578841382	7.67E-05	8.770990819	9.84E-11	14.3498322	2	2	2	5.829220145	5.60003091	8.599312524	8.994365793
ENSG00000188641	DPYD	8.602660628	8.09E-11	5.737696239	2.43E-05	14.34035687	2	2	3.369317972	9.536701552	9.65735634	6.621594001	6.922026307
ENSG00000106546	AHR	7.481983079	5.78E-39	6.857273907	4.20E-33	14.33925699	4.815609471	5.303523747	5.39244166	12.5470107	12.4794558	11.43822139	12.23269239
ENSG00000152977	ZIC1	3.088849543	0.167558017	11.24250808	5.09E-09	14.33135762	2	2	2	4.358765249	2.2680111	11.30228498	11.23946309
ENSG00000164171	ITGA2	9.931527026	5.05E-40	4.387951093	1.39E-08	14.31947812	3.327711234	4.11352228	5.39244166	14.14727587	14.1454883	8.983840816	8.124285971
ENSG00000078269	SYNJ2	7.516431156	1.65E-14	6.802692513	3.86E-12	14.31912367	2	4.93638933	2	10.87647575	10.8592642	9.939423255	10.34450826
ENSG00000138061	CYP1B1	7.94619651	6.97E-07	6.352168295	9.47E-05	14.29836481	3.327711234	7.232742069	2	13.6491886	13.6246674	12.06368006	12.02371203
ENSG00000157654	PALM2-AKAF	11.9985539	3.26E-20	2.298509911	0.126314768	14.29706381	2	2	2	12.00208679	12.050141	3.235948948	3.100202476
ENSG00000164342	TLR3	8.802754409	3.33E-11	5.479230293	6.44E-05	14.2819847	2	2	2	8.882187604	8.80016655	5.661762729	5.59182244
ENSG00000105711	SCN1B	6.983819774	5.89E-07	7.240886195	1.88E-07	14.22470597	2	2	2	7.012547847	7.09482005	7.497765909	7.08034504
ENSG00000109738	GLRB	7.174963059	1.81E-07	7.026374986	3.03E-07	14.20133804	2	2	2	7.186217188	7.29187696	6.998648055	7.186536869
ENSG00000161281	COX7A1	7.307782327	1.69E-07	6.86146273	9.31E-07	14.16924506	2	2	2	7.363085424	7.37584259	6.69514648	7.142226902
ENSG00000157227	MMP14	7.961989602	9.08E-25	6.200047337	1.43E-15	14.16203694	5.330594134	5.946638857	7.600047325	14.48432121	14.541477	12.96826794	12.49706716
ENSG00000152689	RASGRP3	7.98181187	8.50E-09	6.160312539	1.24E-05	14.14212441	2	2	2	8.040677544	8.02009991	6.48637478	5.999739876
ENSG00000144596	GRIP2	8.593095709	5.46E-10	5.548512162	0.000102519	14.14160787	2	2	2	8.595396228	8.67172529	5.954417431	5.367404784
ENSG00000240764	PCDHGC5	10.1543297	2.21E-14	3.98045929	0.00548702	14.13478899	2	2	2	10.15791688	10.2128923	4.133696865	4.500832793
ENSG00000168874	ATOH8	8.323676331	2.51E-09	5.802623994	5.02E-05	14.12630033	2	2	2	8.264463296	8.46389529	5.60262961	6.192573806
ENSG00000122862	SRGN	5.992311569	1.14E-06	8.106876745	1.69E-11	14.09918831	5.866944937	2	3.369317972	10.28749693	10.3037729	12.30044439	12.50562677
ENSG00000197961	ZNF121	6.304632261	6.38E-06	7.788115495	1.26E-08	14.09274776	2	2	2	6.483440992	6.31350454	7.973574582	7.692387724
ENSG00000169629	RGPD8	10.43410229	1.48E-15	3.633023638	0.01085929	14.06712593	2	2	2	10.47329248	10.4557854	4.133696865	3.972018587
ENSG00000148082	SHC3	9.940464915	6.33E-13	4.124537439	0.005519013	14.06500235	2	2	2	9.891822146	10.0490837	4.742549839	4.062589736
ENSG00000157680	DGKI	6.988831791	1.76E-07	7.067510973	1.13E-07	14.05634276	2	2	3.369317972	8.047448792	7.95036812	8.087224739	8.067092663
ENSG00000105738	SIPA1L3	6.97231752	3.44E-14	7.034573352	1.52E-14	14.00689087	2	4.710564154	2	10.09666793	10.098275	9.943797765	10.34713831
ENSG00000115461	IGFBP5	6.059605911	1.56E-25	7.936491466	6.61E-44	13.99609738	8.107517749	9.470501606	9.541233192	15.2092316	15.2364035	16.78321454	17.35901814
ENSG00000080573	COL5A3	7.456904998	2.84E-08	6.518084443	1.44E-06	13.97498944	2	3.076665209	2	8.397612413	8.5227358	7.667989909	7.39408282
ENSG00000165891	E2F7	7.707391411	8.42E-10	6.266366559	7.99E-07	13.97375797	2	3.686303414	2	9.548682898	9.54829592	8.491524932	7.619668339
ENSG00000185532	PRKG1	7.619601049	6.47E-08	6.353383137	8.31E-06	13.97298419	2	3.076665209	2	8.654247601	8.59001038	7.741100262	6.887339785
ENSG00000035862	TIMP2	6.67793799	1.73E-10	7.267303634	2.61E-12	13.94524162	7.218819781	8.053983481	4.058998049	13.78072469	13.8490983	14.27041633	14.52724307
ENSG00000172123	SLFN12	7.185178603	1.21E-07	6.754695965	6.73E-07	13.93987457	2	2	3.369317972	8.221200057	8.16464176	7.559188434	7.953331607
ENSG00000119508	NR4A3	7.218669792	2.76E-05	6.71820432	9.94E-05	13.93687411	2	2	2	7.250225364	7.31425651	7.479255779	5.459179378
ENSG00000120738	EGR1	7.881109764	1.06E-12	6.038847147	6.99E-08	13.91995691	2	4.710564154	4.058998049	11.4613657	11.5111625	10.33690382	8.28888532
ENSG00000113657	DPYSL3	5.789680994	4.55E-15	8.088992354	8.75E-29	13.87867335	4.467311984	6.228547981	4.058998049	10.98303584	10.9103517	13.19345143	13.29308713
ENSG00000182379	NXPH4	4.258478958	0.002380789	9.619888033	1.55E-13	13.87836699	3.327711234	2	2	5.289065324	5.48637102	10.53940814	10.68063396
ENSG00000116194	ANGPTL1	9.395871856	2.36E-11	4.477090073	0.002725828	13.87296193	2	2	2	9.42153757	9.43967292	4.291799915	5.080008298
ENSG00000154237	LRRK1	6.797632679	7.15E-07	7.045745794	2.35E-07	13.84337847	2	3.076665209	2	7.789219291	7.83385794	8.266822833	7.808173621
ENSG00000123095	BHLHE41	6.537982947	2.34E-06	7.303167547	9.27E-08	13.84115049	2	2	2	6.610808945	6.63721363	7.317051196	7.411419562
ENSG00000254245	PCDHGA3	10.27941858	2.21E-13	3.55310053	0.021103887	13.83251911	2	2	2	10.28797351	10.3323804	3.56816602	4.329022995
ENSG00000251493	FOXD1	7.118376566	1.08E-09	6.712093257	9.11E-09	13.83046982	2	3.686303414	2	8.926937992	9.03050377	8.639092784	8.512214961
ENSG00000124875	CXCL6	11.95237304	5.82E-20	1.876244137	0.224818109	13.82861718	2	2	2	12.01365886	11.9459029	3.011868911	2.885414003
ENSG00000092969	TGFB2	4.264136308	3.86E-06	9.552393745	2.31E-27	13.81653005	4.00722531	2	4.058998049	7.454228844	6.88657603	12.44277645	12.452313
ENSG00000135114	OASL	13.25936858	4.21E-23	0.53672737	0.767692243	13.79609595	2	2	2	13.26565594	13.3067071	2.560105256	2.326754077
ENSG00000162520	SYNC	6.933305946	1.79E-10	6.860120219	2.59E-10	13.79342616	2	4.710564154	2	10.04177408	10.0039039	9.366015681	10.36467412
ENSG00000177606	JUN	7.08028268	9.95E-16	6.659606652	3.79E-14	13.73988933	2	5.595927442	4.058998049	11.39212402	11.4591793	11.07271919	10.93618637
ENSG00000111341	MGP	2.565356494	0.048428417	11.16712765	7.27E-23	13.73248414	2	3.686303414	2	4.694064037	4.63360314	12.94685733	13.08746641
ENSG00000180801	ARSJ	5.239001167	2.60E-05	8.486609031	1.55E-12	13.7256102	2	5.303523747	2	8.907220097	8.98007065	11.81456027	12.47327371
ENSG00000156265	MAP3K7CL	4.215614527	0.000512022	9.50562439	6.46E-17	13.72123892	4.00722531	2	2	6.139181293	6.20590375	11.26407555	11.49256057
ENSG00000167972	ABCA3	3.47444478	0.036622122	10.23382622	4.44E-12	13.708271	2	2	2	4.387512679	3.28037825	10.38053488	10.1391448
ENSG00000115107	STEAP3	6.302534688	8.40E-11	7.400620234	1.22E-14	13.70315492	2	3.076665209	4.058998049	8.799033614	8.74219737	9.730197405	9.982668878
ENSG00000144369	FAM171B	7.265449374	1.09E-06	6.429813421	1.84E-05	13.69526279	2	2	2	7.409376773	7.24216308	6.911998488	5.980509018
ENSG00000188112	C6orf132	4.845725432	0.000881385	8.844103539	1.42E-10	13.68982897	2	2	3.369317972	5.775854763	6.0722229	9.393896061	10.17807184
ENSG00000166446	CDYL2	8.096723313	1.06E-06	5.575808004	0.001131917	13.67253132	2	2	2	8.149581172	8.13770558	6.353989995	4.543391831
ENSG00000137752	CASP1	9.061125551	7.42E-12	4.603104221	0.000949913	13.66422977	2	2	2	9.083378928	9.11260313	4.820739789	4.870334174
ENSG00000167702	KIFC2	6.645885023	1.13E-06	7.002647166	2.39E-07	13.64853219	2	3.076665209	2	7.549526251	7.76782485	8.154648976	7.856442979
ENSG00000116675	DNAJC6	4.746168883	0.001122184	8.902014015	8.65E-11	13.6481829	2	2	2	5.194621444	4.70309173	8.798653052	9.069094714
ENSG00000110852	CLEC2B	9.468026559	6.93E-13	4.158708717	0.003156428	13.62673528	2	2	2	9.521380925	9.48312089	4.5389195	4.400220394
ENSG00000099822	HCN2	5.002601144	0.000506245	8.59782554	3.13E-10	13.60042668	2	2	2	5.242616075	5.14724905	8.853368954	8.386355612
ENSG00000167363	FN3K	5.797329359	3.82E-05	7.780849627	1.28E-08	13.57817899	2	2	2	5.808110275	6.02777321	7.765568476	7.897008641
ENSG00000254221	PCDHGB1	10.14510539	1.15E-13	3.411494743	0.023508946	13.55660013	2	2	2	10.14744996	10.2049293	3.584558147	4.147809227
ENSG00000102575	ACP5	6.46078331	0.002810253	7.094736666	0.000864919	13.55551998	2	2	2	6.529309835	6.57060569	8.059640467	4.292061816
ENSG00000152953	STK32B	4.132508721	0.00623234	9.42122094	1.24E-11	13.55372966	2	2	2	4.470469939	4.43005853	9.003023259	9.800020464
ENSG00000206538	VGLL3	4.682725976	3.90E-12	8.854140034	1.31E-41	13.53686601	3.327711234	5.131603625	3.369317972	8.727753724	8.65574839	12.88907816	12.81060063
ENSG00000153234	NR4A2	8.696347292	8.82E-11	4.835972119	0.000564093	13.53231941	2	2	2	8.716468139	8.75588792	5.158477451	4.927687581
ENSG00000160013	PTGIR	7.395100476	1.84E-07	6.122198649	1.98E-05	13.51729913	2	2	2	7.461007055	7.44845993	6.468821191	5.937276556
ENSG00000198598	MMP17	8.065669592	4.37E-09	5.4424983	0.000118706	13.50816789	2	2	2	8.132699624	8.09310209	5.766051132	5.396716407

ENSG00000074855	ANO8	5.98476352	1.09E-05	7.513693259	1.65E-08	13.49845678	2	2	3.369317972	6.970413838	7.06433609	8.573367846	8.459712934
ENSG00000110107	PRPF19	7.149202388	5.49E-10	6.308324793	5.06E-08	13.45752718	2	3.076665209	3.369317972	9.042162213	9.00665321	8.157391075	8.226587404
ENSG00000136999	NOV	6.798032807	1.01E-06	6.628032515	1.80E-06	13.42606532	2	2	2	6.887305128	6.86146731	6.732402829	6.688368862
ENSG00000087116	ADAMTS2	5.168893171	2.10E-12	8.248290787	2.01E-30	13.41718396	2	5.303523747	4.523675391	9.436230969	9.45988854	12.68338458	12.33617257
ENSG00000102007	PLP2	6.581459187	5.11E-12	6.806513437	7.07E-13	13.38797262	3.327711234	5.457120953	2	10.6209439	10.6719453	10.77189113	10.96411291
ENSG00000100292	HMOX1	8.675230074	2.91E-09	4.707377669	0.002199945	13.38260774	2	3.686303414	2	10.48331504	10.4741226	7.237652294	5.282014932
ENSG00000187479	C11orf96	6.490459016	1.52E-11	6.889357202	5.69E-13	13.37981622	2	4.93638933	2	9.747510575	9.94596123	10.21173728	10.28245849
ENSG00000170624	SGCD	5.289393798	0.000272372	8.089698854	6.62E-09	13.37909265	2	2	2	5.461211713	5.44640346	7.826275727	8.392188801
ENSG00000178951	ZBTB7A	6.275960856	3.69E-06	7.098992757	1.15E-07	13.37495361	2	5.595927442	2	10.18478072	10.3364864	11.17122857	10.99030784
ENSG00000164070	HSPA4L	5.064245722	0.000453925	8.307950406	1.58E-09	13.37219613	2	2	2	5.210797711	5.28958453	8.568740544	8.096865967
ENSG00000235376	RPEL1	6.675651473	2.28E-06	6.69220034	1.97E-06	13.36785181	2	2	2	6.820355514	6.68951263	6.615624322	6.913939481
ENSG00000137033	IL33	14.01558365	1.90E-21	-0.653185163	0.748118922	13.36239849	2	2	2	14.06846635	14.0156896	2.34604626	2.059860178
ENSG00000107984	DKK1	9.247193409	6.19E-13	4.112153131	0.002650534	13.35934654	3.327711234	3.076665209	2	11.1032668	11.1106357	6.564892306	5.269393099
ENSG00000157601	MX1	10.77000438	6.15E-30	2.573677512	0.013923728	13.3436819	2	4.710564154	2	13.87406507	13.887268	5.4588802	6.06327132
ENSG00000198482	ZNF808	7.335447684	2.46E-07	6.007850076	3.01E-05	13.34329776	2	2	2	7.45762193	7.33264456	6.351597515	5.842465082
ENSG00000134954	ETS1	6.7648362	7.17E-10	6.57785291	2.00E-09	13.34268911	2	5.72254323	3.369317972	11.06734964	11.0471929	10.6402526	11.06934348
ENSG00000105889	STEAP1B	8.856319832	6.51E-11	4.481731227	0.001775206	13.33805106	2	2	2	8.912174893	8.87734839	4.530483275	4.927687581
ENSG00000010704	HFE	6.687427044	1.45E-06	6.645562109	1.57E-06	13.33298915	2	3.076665209	2	7.66556918	7.74054144	7.372383768	7.90417044
ENSG00000178776	C5orf46	5.011699957	0.000899661	8.313907304	6.78E-09	13.32560726	2	2	2	5.258265913	5.14724905	7.862014947	8.726491728
ENSG00000134769	DTNA	6.297014054	1.82E-05	7.016520559	1.27E-06	13.31353461	2	2	2	6.436065776	6.35006802	6.717615541	7.379474672
ENSG00000153904	DDAH1	5.566723621	2.92E-06	7.731928235	2.81E-11	13.29865186	3.327711234	5.946638857	2	10.08135305	10.0345549	12.29212498	12.14224092
ENSG00000115267	IFIH1	9.535034437	2.06E-28	3.757544321	3.17E-05	13.29257876	2	4.442710129	2	12.37946895	12.363074	6.683779344	6.617932662
ENSG00000109452	INPP4B	7.135815646	5.33E-06	6.155573789	0.000100443	13.29138943	2	2	2	7.075737784	7.31795292	6.743394671	5.524371359
ENSG00000163565	IFI16	8.491629413	8.19E-10	4.796542071	0.000904021	13.28817148	2	5.72254323	2	12.58957959	12.6104106	9.056150495	8.760712793
ENSG00000183801	OLFML1	4.927831053	0.005313564	8.325688201	5.97E-07	13.25351925	2	2	2	5.128039203	5.13053105	6.994053541	9.060878381
ENSG00000115641	FHL2	5.611328668	2.33E-07	7.625961766	6.87E-13	13.23729043	3.327711234	5.72254323	2	9.879225887	9.93276281	11.90309722	11.92945766
ENSG00000172493	AFF1	6.600194919	4.88E-14	6.637093156	2.66E-14	13.23728808	2	4.710564154	2	9.741955564	9.73406074	9.817517875	9.730640067
ENSG00000112183	RBM24	4.57175992	0.001312777	8.664026258	9.20E-11	13.23578618	2	2	2	4.76150579	4.87352701	8.723392661	8.684919451
ENSG00000117586	TNFSF4	9.414383255	2.77E-09	3.818737604	0.026949319	13.23312086	2	2	2	9.453327884	9.44475345	4.742549839	3.311948089
ENSG00000185950	IRS2	6.198387481	1.45E-10	7.018576531	2.29E-13	13.21696401	2	2	4.874587708	9.081180848	9.16073939	9.907406852	9.966387947
ENSG00000124508	BTN2A2	6.745945437	2.10E-13	6.462112274	1.87E-12	13.20805771	2	4.442710129	2	9.615519136	9.53803206	9.138949083	9.43604749
ENSG00000153944	MSI2	6.135070214	2.95E-07	7.067440856	2.23E-09	13.20251107	2	3.686303414	2	8.074220386	7.93118934	8.781425385	9.05767046
ENSG00000151632	AKR1C2	9.947846482	1.68E-23	3.230727436	0.002564517	13.17857392	2	2	4.523675391	12.41491918	12.4381264	5.598599872	6.003555476
ENSG00000134321	RSAD2	13.39828359	4.52E-17	-0.223249482	0.920567538	13.17503411	2	3.076665209	3.369317972	15.26494408	15.2457575	3.296906311	2.117335275
ENSG00000124785	NRN1	8.412188813	1.68E-07	4.729914713	0.005277336	13.14210353	2	2	2	8.447952961	8.46222608	5.528313928	3.987514168
ENSG00000128567	PODXL	5.418654136	4.50E-07	7.717671677	1.74E-13	13.13632581	6.138360429	3.686303414	3.369317972	10.18119344	10.1515521	11.6770541	12.96674738
ENSG00000180530	NRIP1	7.236459462	1.65E-11	5.889172181	5.61E-08	13.12563164	2	2	4.523675391	9.777686595	9.68838639	8.685899845	8.034828728
ENSG00000253537	PCDHGA7	9.220312596	2.16E-12	3.899408699	0.005658731	13.1197213	2	3.076665209	2	10.18170646	10.244312	5.185595313	4.943664032
ENSG00000226742	HSBP1L1	6.733812829	1.89E-06	6.372621001	6.69E-06	13.10643383	2	2	2	6.809774134	6.81514011	6.597565628	6.320441373
ENSG00000081041	CXCL2	12.14473503	6.54E-17	0.956052317	0.608101226	13.10078734	2	2	2	12.21566187	12.1277429	2.3070652	2.817676617
ENSG00000145242	EPHA5	6.502828051	3.42E-05	6.576689466	2.49E-05	13.07951752	2	2	2	6.522845756	6.65485813	5.982522014	7.12130792
ENSG00000277203	F8A1	8.415558423	2.68E-10	4.64638498	0.000872927	13.0619434	2	2	3.369317972	9.353044608	9.46906021	5.574182174	5.900914815
ENSG00000121350	PYROXD1	7.163240802	2.74E-07	5.850705358	3.41E-05	13.01394616	2	2	2	7.246306984	7.21069602	5.883320383	6.055940621
ENSG00000163814	CDCP1	8.228032737	1.24E-18	4.767534142	6.34E-07	12.99556688	4.00722531	2	4.874587708	11.73741575	11.7703643	8.72385521	7.727417522
ENSG00000065534	MYLK	6.009407532	2.29E-05	6.973271693	5.94E-07	12.98267922	3.327711234	7.051611315	3.369317972	11.57546597	11.6152815	13.16171034	11.5021165
ENSG00000198841	KTI12	7.121659715	5.37E-07	5.857359901	4.71E-05	12.97901962	2	2	2	7.132034771	7.24216308	6.167993056	5.759151667
ENSG00000185567	AHNAK2	5.317952351	1.32E-05	7.623099693	1.27E-10	12.94105204	4.815609471	6.535001748	2	10.54203082	10.6435047	12.32812587	13.30214374
ENSG00000177989	ODF3B	7.562354798	7.17E-08	5.369991598	0.000193253	12.9323464	2	2	2	7.523859802	7.70710533	5.703636713	5.32534152
ENSG00000115415	STAT1	7.404061525	2.97E-14	5.522747535	2.15E-08	12.92680906	7.159102577	8.276714465	4.874587708	14.71318337	14.7019162	13.18543446	12.34846028
ENSG00000154556	SORBS2	4.846844978	0.000497308	8.076640171	1.14E-09	12.92348515	3.327711234	2	2	5.978405189	5.88556448	9.168133494	8.97651941
ENSG00000241399	CD302	6.824274748	1.18E-06	6.090904354	1.64E-05	12.9151791	2	2	2	6.970413838	6.82556437	6.200262696	6.195912571
ENSG00000142552	RCN3	6.216191329	1.10E-07	6.686390069	8.52E-09	12.9025814	4.815609471	6.602424674	2	11.53166763	11.5617274	12.00470931	12.02805033
ENSG00000171617	ENC1	7.273854582	1.65E-13	5.623434517	1.72E-08	12.8972891	2	4.710564154	2	10.46993609	10.2892535	9.012519675	8.406669418
ENSG00000167779	IGFBP6	8.379586388	1.73E-12	4.508419928	0.0002746	12.88800632	2	3.686303414	6.203069917	13.045527	13.0670016	9.083080045	9.297612054
ENSG00000072657	TRHDE	6.678920468	5.03E-06	6.20829868	2.30E-05	12.88721915	2	2	2	6.892328675	6.61334686	6.022174708	6.549111181
ENSG00000172403	SYNPO2	4.328192943	9.81E-07	8.537437011	1.15E-23	12.86562995	4.00722531	5.457120953	5.156626401	9.169698743	9.1566168	14.00882487	12.16264744
ENSG00000139083	ETV6	5.896375837	1.27E-11	6.962031175	5.74E-16	12.85840701	2	4.11352228	3.369317972	8.737556707	8.7802082	9.940418627	9.683551156
ENSG00000174332	GLIS1	5.289472578	0.000218113	7.562651669	4.19E-08	12.85212425	2	2	2	5.474671438	5.43283104	7.716209895	7.513957873
ENSG00000105767	CADM4	5.396728635	0.000195383	7.446667798	1.06E-07	12.84339643	2	2	2	5.540148523	5.56313034	7.686143116	7.297928101
ENSG00000169083	AR	6.494130249	2.20E-06	6.342960969	3.67E-06	12.83709122	2	3.076665209	2	7.507583254	7.52080763	7.207540845	7.509939612
ENSG00000180138	CSNK1A1L	6.758212703	1.78E-06	6.073331379	1.96E-05	12.83154408	2	2	2	6.836083475	6.83591385	6.290458506	6.06327132
ENSG00000074527	NTN4	3.062546602	0.014298818	9.765141439	5.61E-18	12.82768804	2	5.303523747	2	6.750147204	6.88158913	13.4572701	13.47939587
ENSG00000163412	EIF4E3	5.107556054	0.000400716	7.683412054	2.78E-08	12.79096811	2	2	2	5.334065616	5.24368216	7.872900025	7.586981404
ENSG00000141013	GAS8	6.47763442	7.72E-06	6.306200362	1.29E-05	12.78383478	2	2	2	6.61689148	6.51372389	6.173421703	6.600402875
ENSG00000127528	KLF2	5.243336524	7.80E-05	7.50991184	5.04E-09	12.75324836	2	3.686303414	2	7.194375246	7.02868983	9.76049665	8.765214879
ENSG00000176438	SYNE3	4.607304901	0.001569431	8.137916546	3.21E-09	12.74522145	2	2	2	4.670864177	5.00774437	8.124136806	8.242012195
ENSG00000071575	TRIB2	4.312302687	2.48E-05	8.423795092	6.10E-18	12.73609778	2	4.710564154	2	7.510853288	7.38644297	11.82779956	11.15439097
ENSG00000110455	ACCS	6.480258129	3.06E-05	6.240682143	5.82E-05	12.72094027	2	2	2	6.509830055	6.6253296	5.755231204	6.755525668
ENSG00000070882	OSBPL3	6.926050283	2.32E-08	5.792467049	3.76E-06	12.71851733	4.00722531	2	2	8.88471049	8.75861051	8.008923105	7.317985043
ENSG00000162892	IL24	11.5577434	2.95E-09	1.154038378	0.638141963	12.71178178	2	2	2	11.58964598	11.5824281	3.059555873	2.059860178
ENSG00000184470	TXNRD2	5.879927846	2.19E-05	6.82466941	5.41E-07	12.70459726	2	2	3.369317972	6.917187326	6.91614081	7.680435163	7.980689421
ENSG00000128342	LIF	7.356325667	1.25E-09	5.34639163	1.52E-05	12.7027173	6.138360429	3.686303414	2	11.97155766	12.0084303	9.477737996	10.35872838
ENSG00000159208	CIART	6.400646238	7.03E-06	6.295992307	9.41E-06	12.69663854	2	2	2	6.5612028	6.42052719	6.34920106	6.435070131
ENSG00000137878	GCOM1	9.161417346	7.60E-11	3.518252897	0.022469646	12.67967024	2	2	2	9.252121432	9.14105132	3.600766115	4.26688425
ENSG00000001617	SEMA3F	6.804804388	9.44E-10	5.864648191	1.61E-07	12.66945258	2	3.686303414	3.369317972	9.252121432	9.23593348	7.633939843	8.772500938
ENSG00000198133	TMEM229B	5.626469571	0.000104489	7.031495788	6.63E-07	12.65796536	2	2	2	5.829220145	5.69405321	7.241533944	6.944034147
ENSG00000185278	ZBTB37	4.97439965	0.000555287	7.656863959	2.69E-08	12.63126361	2	2	2	5.093559143	5.24368216	7.760166845	7.66011679
ENSG00000138378	STAT4	5.542024556	0.00135052	7.076470944	2.44E-05	12.6184955	2	2	2	5.818703821	5.53799497	7.753839274	6.066922748
ENSG00000041353	RAB27B	8.531848592	1.02E-13	4.080564461	0.000735092	12.61241305	2	3.686303414	2	10.40902283	10.3662449	5.99483933	6.052261256
ENSG00000102524	TNFSF13B	10.05051427	2.38E-11	2.559424727	0.135257068	12.609939	2	2	2	10.12521908	10.0379207	3.709392871	2.782576693
ENSG00000140403	DNAJA4	5.540379592	7.57E-05	7.060859212	2.29E-07	12.6012388	2	3.076665209	2	6.394182228	6.76185814	8.022522757	8.117259579
ENSG00000204941	PSG5	5.245811773	0.000459654	7.336998916	3.69E-07	12.58281069	2	2	2	5.686138801	5.07918227	7.411959578	7.383872668
ENSG00000276910		8.264783965	1.10E-09	4.315107821	0.002615937	12.57989179	2	2	2	8.276040626	8.34212018	4.504874511	4.692661645
ENSG00000134245	WNT2B	4.363223706	0.003871463	8.193886086	6.68E-09	12.55710979	2	2	2	4.846787657	4.40247105	7.984424805	8.456236734
ENSG00000167767	KRT80	2.012384905	0.221153215	10.54444285	5.04E-15	12.55682776	2	2	2	3.162721474	2.86123014	10.4114447	10.72117
ENSG00000163735	CXCL5	13.62562716	6.29E-21	-1.112161687	0.571127612	12.51346547	2	2	2	13.67524369	13.6293234	2.048074405	2.277179783
ENSG00000264364	DYNLL2	6.379367104	1.92E-12	6.111805021	1.44E-11	12.49117213	2	4.442710129	2	9.231457548	9.20133	8.883491454	9.015305099
ENSG00000144036	EXOC6B	5.844987291	1.96E-08	6.644637234	1.10E-10	12.48962453	2	4.710564154	2	8.95000826	8.95417718	9.326055204	10.07214007
ENSG00000106688	SLC1A1	6.799934133	1.30E-09	5.688618705	4.90E-07	12.48855284	2	2	4.874587708	9.771565364	9.72295127	8.963225048	8.233102165
ENSG00000175899	A2M	4.435797002	0.052158739	8.048177417	0.000140835	12.48397442	3.327711234	2	2	6.456560224	2.59489879	9.420391988	8.542691771
ENSG00000183337	BCOR	5.546887283	2.66E-08	6.92024309	1.73E-12	12.46713037	2	4.442710129	2	8.258639605	8.47883193	9.432328044	9.98604778
ENSG00000013619	MAMLD1	5.389289155	0.000113829	7.072675474	1.91E-07	12.46196463	2	2	3.369317972	6.422239259	6.46787796	7.92612656	8.223318958
ENSG00000253731	PCDHGA6	8.346014586	2.24E-11	4.095588627	0.001915611	12.44160321	2	2	4.058998049	10.2136641	10.2764875	5.574182174	6.451924088
ENSG00000136830	FAM129B	6.25240834	5.87E-13	6.18732931	8.72E-13	12.43973765	5.532353208	7.73146271	5.39244166	12.85170056	12.8802457	12.69764229	12.89743356
ENSG00000148926	ADM	6.319606769	8.02E-08	6.109821694	2.08E-07	12.42942846	4.467311984	6.464272742	2	11.44500551	11.4766471	11.10583548	11.38368346
ENSG00000102452	NALCN	4.483303997	0.00209302	7.935433057	7.27E-09	12.41873705	2	2	2	4.647285135	4.83276841	7.918872821	8.047821014
ENSG00000131981	LGALS3	6.298892608	1.05E-21	6.114744884	9.93E-21	12.41363749	3.327711234	4.11352228	5.59507254	10.67003398	10.6372309	10.45318893	10.48674576
ENSG00000128203	ASPHD2	6.619593552	4.23E-06	5.775464118	6.88E-05	12.39505767	2	2	2	6.598566398	6.79936111	6.016144783	5.777095613
ENSG00000213799	ZNF845	6.38489564	8.90E-06	6.000074753	3.05E-05	12.38497039	3.327711234	2	2	7.546342806	7.2537885	7.336538937	6.64749689
ENSG00000184922	FMNL1	7.610863002	7.44E-07	4.772662136	0.002930366	12.38352514	2	2	2	7.593372273	7.73502229	5.427419529	4.365060851
ENSG00000143127	ITGA10	4.047210226	0.007642363	8.333236132	2.80E-09	12.38044636	2	2	2	4.415698455	4.3456612	8.033758684	8.654300638
ENSG00000115339	GALNT3	6.795342121	4.53E-06	5.582791148	0.000203796	12.37813327	2	2	2	6.872128674	6.8715633	6.007052374	5.367404784
ENSG00000112139	MDGA1	6.286129377	2.40E-09	6.09069067	7.07E-09	12.37682005	3.327711234	3.076665209	3.369317972	8.71221318	8.79752133	8.056705941	8.935663927
ENSG00000106246	PTCD1	7.349603154	1.31E-07	5.00289973	0.00049334	12.35250288	2	2	2	7.440576128	7.37938471	5.258920789	5.12973572
ENSG00000261115	TMEM178B	2.374407678	0.164591992	9.973805487	6.45E-12	12.34821316	3.327711234	2	2	4.205751501	3.39976443	10.45249137	11.34219318
ENSG00000171246	NPTX1	6.626231649	0.001337504	5.709121645	0.006337615	12.33535329	2	2	2	6.739036112	6.67805337	6.658838928	3.753786374
ENSG00000162889	MAPKAPK2	6.077907173	4.81E-07	6.255484551	1.92E-07	12.33339172	2	6.311476754	4.058998049	10.96317997	11.0510847	11.07770369	11.28494885

ENSG00000177283	FZD8	7.117599289	2.90E-11	5.212261649	1.70E-06	12.32986094	2	4.11352228	2	9.491898484	9.57325401	7.946879235	7.272853598
ENSG00000162944	RFTN2	6.333454194	3.38E-05	5.994711648	8.72E-05	12.32816584	2	2	2	6.456560224	6.39974839	6.444330878	5.66592325
ENSG00000153246	PLA2R1	6.181537594	6.24E-09	6.136536616	7.32E-09	12.31807421	2	4.11352228	2	8.607665593	8.60672783	8.245719321	8.822498333
ENSG00000104205	SGK3	5.787915971	9.38E-05	6.528422075	7.59E-06	12.31633805	2	2	2	5.987823101	5.83486613	6.382394503	6.815359915
ENSG00000081853	PCDHGA2	8.349722268	4.08E-13	3.962721148	0.001120882	12.31244342	2	3.686303414	2	10.17347624	10.2370092	5.86330637	5.96101835
ENSG00000151967	SCHIP1	7.577610105	4.59E-08	4.732787331	0.001029882	12.31039744	2	2	2	7.530319349	7.72948195	5.002522427	4.911532223
ENSG00000107957	SH3PXD2A	5.812156255	5.96E-05	6.494362698	5.46E-06	12.30651895	4.815609471	7.393647515	2	11.79538311	11.8174753	12.44330319	12.53145936
ENSG00000161956	SENP3	6.612782873	3.45E-11	5.677542088	1.56E-08	12.29032496	4.467311984	2	2	9.037634995	9.14417788	8.177792357	8.154630332
ENSG00000115963	RND3	7.591753554	1.39E-07	4.691555825	0.001786849	12.28330938	8.009475818	6.728495673	2	14.48660668	14.4773038	12.03186006	10.92905675
ENSG00000168062	BATF2	8.902939506	3.36E-11	3.37762757	0.021742881	12.28056708	2	2	2	8.904736303	8.97656697	3.968654369	3.753786374
ENSG00000137449	CPEB2	5.052551984	4.99E-06	7.227998301	1.91E-11	12.28055029	2	5.131603625	2	8.682071821	8.51792306	10.69754037	10.83167443
ENSG00000149346	SLX4IP	4.912909845	0.000645538	7.365799354	9.59E-08	12.2787092	2	3.076665209	2	6.061031724	5.92487824	8.62380783	8.065268319
ENSG00000162745	OLFML2B	3.658670053	0.016459729	8.615778848	6.37E-10	12.2744489	2	3.076665209	2	4.907595604	4.811949	9.986250224	9.103279088
ENSG00000172572	PDE3A	3.993730553	0.010492446	8.277867241	8.63E-09	12.27159779	2	2	2	4.415698455	4.2560182	8.676850263	7.851158781
ENSG00000066697	MSANTD3	6.43182894	4.48E-10	5.828873616	1.76E-08	12.26070256	2	4.93638933	2	9.800579656	9.75398117	8.929534325	9.389783813
ENSG00000258643	BCL2L2-PABI	10.19431583	1.44E-12	2.059801539	0.220786883	12.25411737	2	2	2	10.23853288	10.2123959	2.68698273	3.33635788
ENSG00000170458	CD14	5.274443218	0.000312209	6.978650187	8.54E-07	12.25309341	2	2	2	5.44762523	5.43283104	6.911998488	7.174740504
ENSG00000166396	SERPINB7	8.063666707	7.27E-09	4.176803589	0.004736945	12.2404703	2	2	2	8.160032699	8.06037647	4.261535101	4.683176719
ENSG00000090376	IRAK3	8.264979763	2.98E-09	3.971460605	0.007602279	12.23644037	2	2	3.369317972	9.28496215	9.23983583	5.4588802	4.711446447
ENSG00000177932	ZNF354C	5.956340904	5.96E-05	6.278970348	1.93E-05	12.23531125	2	3.076665209	2	6.970413838	7.01053089	7.705008156	6.746446439
ENSG00000174600	CMKLR1	4.064095652	0.005817392	8.167508143	2.26E-09	12.2316038	2	2	2	4.329433526	4.45712836	8.109343506	8.310608339
ENSG00000242419	PCDHGC4	10.1386649	2.00E-13	2.065675297	0.198633428	12.2043402	2	2	2	10.14007766	10.1994291	2.803598391	3.261853426
ENSG00000131378	RFTN1	5.504963302	1.93E-09	6.696055619	1.30E-13	12.20101892	2	5.303523747	3.369317972	9.437090644	9.45318143	10.78635657	10.45818207
ENSG00000255529	POLR2M	6.743426855	1.01E-11	5.453311745	4.92E-08	12.1967386	2	4.11352228	2	9.234427714	9.12003262	7.888662695	7.916365698
ENSG00000156535	CD109	7.348525958	4.30E-29	4.839693817	2.91E-13	12.18821977	3.327711234	4.11352228	4.523675391	11.03449708	11.0139665	8.544331623	8.512214961
ENSG00000133069	TMCC2	4.430879119	0.00254798	7.754264669	2.01E-08	12.18514379	2	2	2	4.694064037	4.70309173	7.924517766	7.678134128
ENSG00000151414	NEK7	5.433943034	2.71E-05	6.721390202	1.16E-07	12.15533324	2	6.728495673	4.523675391	10.83989045	10.7710044	11.9932919	12.18324251
ENSG00000006459	KDM7A	6.343241413	2.48E-08	5.793326897	3.87E-07	12.13656831	2	4.11352228	2	8.717883675	8.78689171	8.594261972	7.681710751
ENSG00000163734	CXCL3	12.91730977	1.15E-21	-0.781904958	0.675642045	12.13540481	2	2	2	12.96593307	12.9225886	2.267001569	2.117335275
ENSG00000159231	CBR3	6.068929369	3.17E-05	6.066279197	2.90E-05	12.13520857	2	2	2	6.197166296	6.1572134	6.057834398	6.283256815
ENSG00000197808	ZNF461	6.090594234	5.11E-05	6.016336933	5.89E-05	12.10693117	2	2	2	6.24507773	6.14893615	6.380048704	5.821002687
ENSG00000140961	OSGIN1	8.032480815	1.60E-08	4.057818861	0.007388815	12.09029968	2	2	2	8.056427969	8.10384772	4.644324387	4.077147165
ENSG00000083857	FAT1	5.414341658	6.94E-05	6.675394276	5.39E-07	12.08973593	6.366709671	7.60566235	2	11.91575529	11.9163384	13.25854844	13.08889818
ENSG00000136514	RTP4	8.966750681	2.45E-11	3.116318349	0.035697933	12.08306903	2	2	2	9.010169232	8.99861459	3.56816602	3.789585079
ENSG00000197774	EME2	4.722961074	0.001259444	7.353936805	1.33E-07	12.07689788	2	2	2	4.965943888	4.93258395	7.347386207	7.478755384
ENSG00000155629	PIK3AP1	5.642825884	0.00184965	6.40584129	0.000313297	12.04866717	2	2	2	5.639097847	5.90535527	7.143982415	5.294527297
ENSG00000159733	ZFYVE28	5.610629062	5.96E-05	6.416245672	3.07E-06	12.02687473	2	2	3.369317972	6.716553866	6.59518404	7.538302335	7.330190599
ENSG00000221963	APOL6	7.050143105	2.47E-29	4.973034014	3.23E-15	12.02317712	3.327711234	4.93638933	5.59507254	11.73479622	11.7190443	9.856639061	9.421147165
ENSG00000007202	KIAA0100	5.624140788	9.94E-10	6.383756907	2.44E-12	12.00789769	6.366709671	4.442710129	3.369317972	10.67878272	10.7365497	11.17993582	11.70488663
ENSG00000180233	ZNRF2	6.417248447	1.13E-05	5.574599335	0.000155741	11.99184778	2	2	2	6.490083648	6.52655952	5.866661379	5.545463418
ENSG00000169213	RAB3B	4.31754499	5.36E-10	7.648338629	1.31E-29	11.96588362	5.330594134	4.93638933	6.882775709	10.15791688	10.1853333	13.48511211	13.51071303
ENSG00000065320	NTN1	6.561455193	1.47E-05	5.356494604	0.000488129	11.9179498	2	2	2	6.907295206	6.32824132	5.304163849	5.699242824
ENSG00000031081	ARHGAP31	5.639190288	7.67E-14	6.273123309	4.56E-17	11.9123136	2	4.442710129	4.058998049	9.021676912	9.14105132	9.577337433	9.836576475
ENSG00000262406	MMP12	5.869460779	0.046404119	5.991000344	0.039274911	11.86046112	2	2	2	6.01571348	5.96314905	7.05561307	2.172608095
ENSG00000125657	TNFSF9	6.467791727	3.55E-05	5.390463432	0.00067229	11.85825516	2	2	2	6.604700657	6.507263	5.038665118	5.921228818
ENSG00000147119	CHST7	5.32867275	1.15E-05	6.528027858	4.13E-08	11.85670061	2	3.686303414	2	7.157295126	7.26914476	8.306273771	8.472158355
ENSG00000112096	SOD2	8.394878984	1.42E-75	3.44999721	1.85E-13	11.84487619	7.904282243	6.844429755	7.934808822	15.99212855	15.9937102	10.91507154	11.17462754
ENSG00000182890	GLUD2	5.243031407	2.03E-07	6.601777187	2.78E-11	11.84480859	2	4.93638933	2	8.62888906	8.57927024	9.823356991	10.07372892
ENSG00000140379	BCL2A1	8.159361065	1.89E-05	3.682552932	0.075024979	11.841914	2	2	2	8.258639605	8.15020018	4.785723309	2.70970617
ENSG00000188042	ARL4C	5.355503976	0.000225083	6.475041275	5.29E-06	11.83054525	2	5.72254323	2	9.477763976	9.46156046	10.55547888	10.61355952
ENSG00000111424	VDR	6.151853212	2.67E-07	5.6693141	2.29E-06	11.82116731	3.327711234	2	3.369317972	8.083034979	8.02463101	7.749302529	7.388257299
ENSG00000168060	NAALADL1	4.974391091	0.000777117	6.83916125	1.61E-06	11.81355234	2	2	2	5.093559143	5.24368216	6.910373854	6.91798856
ENSG00000185633	NDUFA4L2	0.402374303	0.811969254	11.40814573	2.47E-21	11.81052003	2	3.686303414	2	3.289987342	2.94009902	12.97484098	13.47570932
ENSG00000041982	TNC	6.969284358	7.46E-19	4.829619972	1.29E-09	11.79890433	2	4.710564154	3.369317972	10.50929771	10.2510948	8.181164698	8.336553984
ENSG00000129355	CDKN2D	5.345610914	0.000145175	6.445519183	2.87E-06	11.7911301	2	3.076665209	2	6.328984825	6.47451726	7.559188434	7.36768071
ENSG00000091039	OSBPL8	6.610814149	2.20E-06	5.165665385	0.000283328	11.77647953	3.327711234	6.464272742	2	11.58189337	11.5601677	10.36193351	9.851472775
ENSG00000181754	AMIGO1	3.418024553	0.024824384	8.358316443	1.18E-09	11.776341	2	2	2	3.997744386	3.7864959	8.52588267	8.266036253
ENSG00000136630	HLX	6.608617742	2.54E-06	5.155961167	0.000317146	11.76457891	2	3.076665209	2	7.602596956	7.64959655	6.453284622	5.949196291
ENSG00000112619	PRPH2	5.369075304	0.000416219	6.393057686	1.70E-05	11.76213299	2	2	2	5.552894743	5.49945122	6.712030998	6.215784511
ENSG00000072121	ZFYVE26	6.32981128	2.01E-07	5.415057565	1.06E-05	11.74486885	2	5.303523747	2	9.999333534	10.0596091	9.253905612	8.972144811
ENSG00000168811	IL12A	8.541827338	2.32E-10	3.200113698	0.030786735	11.74194104	2	2	2	8.559529208	8.60672783	3.796405078	3.67940853
ENSG00000172731	LRRC20	5.038066911	0.000702835	6.697128987	3.18E-06	11.7351959	2	2	2	5.210797711	5.24368216	6.681876084	6.866539459
ENSG00000128881	TTBK2	5.318857644	7.78E-07	6.415534404	1.39E-09	11.73439205	2	4.11352228	2	7.691737974	7.81828345	9.08380094	8.543347212
ENSG00000142798	HSPG2	5.113012101	9.45E-11	6.613096789	1.81E-17	11.72610889	6.653098118	6.464272742	8.536706852	12.57305446	12.6628552	13.97758897	14.24620657
ENSG00000149289	ZC3H12C	7.398496028	3.34E-10	4.318685772	0.000425805	11.7171818	2	5.303523747	2	11.13115783	11.0670971	7.971238879	8.103992141
ENSG00000123643	SLC36A1	5.027946002	2.54E-05	6.677937314	9.44E-09	11.70588332	2	4.93638933	2	8.291333947	8.42500614	9.41724949	10.4104801
ENSG00000069667	RORA	6.519337782	4.17E-12	5.181331258	4.92E-08	11.70066904	4.467311984	3.076665209	2	9.437090644	9.36838404	8.362013331	7.72395257
ENSG00000184378	ACTRT3	5.607224019	0.000333419	6.077974753	8.07E-05	11.68519877	2	2	2	5.552894743	5.91514978	5.843010798	6.463051659
ENSG00000154589	LY96	7.494959764	1.28E-07	4.189544496	0.005165222	11.68450426	2	2	2	7.514115926	7.58969985	4.675266036	4.292061816
ENSG00000133657	ATP13A3	6.638815729	0.001939481	5.032820634	0.022450111	11.67163636	2	7.790474954	2	12.84507664	12.8140605	11.11026486	11.3317026
ENSG00000184985	SORCS2	7.000241637	2.39E-07	4.665566867	0.000873364	11.6658085	2	2	3.369317972	8.006336225	8.01555453	5.790986459	5.736403255
ENSG00000249471	ZNF324B	5.787859202	0.000137041	5.874166648	9.77E-05	11.66202585	2	2	2	5.978405189	5.84514933	6.205571486	5.745545708
ENSG00000135801	TAF5L	5.546802302	4.28E-08	6.106829549	1.14E-09	11.65363185	2	4.11352228	2	7.997038782	7.98797735	8.600320517	8.487906181
ENSG00000185634	SHC4	4.828600826	0.000552035	6.807705503	4.41E-07	11.63630633	3.327711234	3.076665209	2	6.699459234	6.77805036	9.231960665	7.764992659
ENSG00000168453	HR	5.541331347	0.000273049	6.075500064	5.15E-05	11.61683141	2	2	2	5.709096143	5.65950892	5.932173402	6.397863414
ENSG00000175600	SUGCT	3.935714364	0.008033366	7.670483561	2.78E-08	11.60619793	2	3.076665209	2	4.846787657	5.3193943	8.843612414	8.479026367
ENSG00000163659	TIPARP	6.356305336	1.68E-10	5.249055358	1.74E-07	11.60536069	2	6.228547981	5.59507254	11.63493198	11.6660124	10.62572272	10.46026599
ENSG00000134259	NGF	2.082487458	0.200921344	9.518357362	1.60E-12	11.60084482	2	2	2	3.162721474	2.94009902	9.51429464	9.589581388
ENSG00000142871	CYR61	4.198399171	0.021458981	7.400862084	1.48E-05	11.59926125	8.653385977	5.131603625	2	11.32078887	11.399377	14.67186756	14.44113699
ENSG00000106819	ASPN	1.777301931	0.335944632	9.817608792	4.92E-11	11.59491072	2	2	2	2.947924294	2.86123014	9.01856474	10.37446816
ENSG00000142583	SLC2A5	6.068297715	0.000247921	5.52293584	0.000909649	11.59123356	2	2	2	6.087554739	6.26070706	6.129409568	4.982847849
ENSG00000161217	PCYT1A	5.013681978	0.000661823	6.558661576	4.50E-06	11.57234355	2	5.72254323	2	9.144656522	9.11366683	10.48546314	10.828989
ENSG00000115232	ITGA4	6.989554019	1.44E-05	4.582246683	0.006305222	11.5718007	2	2	2	7.210554257	6.89154576	5.274160001	4.175132639
ENSG00000173391	OLR1	2.777319919	0.088103631	8.75991591	5.42E-10	11.53723583	2	2	2	3.515101962	3.39680241	8.403026507	9.109931338
ENSG00000151835	SACS	4.978994169	0.000535791	6.556551024	2.65E-06	11.53554519	2	6.464272742	3.369317972	10.00688028	9.88372429	11.74214602	11.25794964
ENSG00000072840	EVC	6.24153683	0.000353647	5.283515407	0.002856714	11.52505224	2	6.464272742	2	11.02652273	11.1590942	10.09769543	10.17870477
ENSG00000197312	DDI2	5.403246559	2.69E-07	6.116398647	3.84E-09	11.51964521	2	4.11352228	2	7.783822874	7.90193448	8.72431761	8.345600992
ENSG00000258102	MAP1LC3B2	6.227748798	2.33E-18	5.291710068	1.18E-13	11.51945887	4.00722531	3.686303414	4.058998049	9.708880707	9.67042697	8.660996377	8.852955181
ENSG00000078018	MAP2	3.57829697	0.005204952	7.902479106	2.95E-11	11.48077608	4.467311984	2	2	6.598566398	5.51241389	10.58976003	10.18249657
ENSG00000115252	PDE1A	2.859811552	0.114510897	8.619998221	4.60E-08	11.47980977	2	2	2	3.566290607	3.45167086	7.625052652	9.257431793
ENSG00000176749	CDK5R1	5.418226877	0.001152927	6.049327564	0.000220783	11.46755444	2	2	2	5.709096143	5.41912973	6.597565628	5.524371359
ENSG00000114270	COL7A1	6.831084582	7.08E-14	4.618574946	6.95E-07	11.44965953	6.89194353	3.686303414	5.772716771	12.65338764	12.6844922	10.44170614	10.47751587
ENSG00000130052	STARD8	5.739298207	0.000121932	5.709484055	0.00012244	11.44878226	2	2	2	5.870535235	5.86549842	5.912830251	5.76365862
ENSG00000106823	ECM2	2.535488634	0.101148097	8.902406661	2.87E-11	11.4378953	2	2	3.369317972	4.034562184	3.86943969	9.691228581	10.07644859
ENSG00000211448	DIO2	4.804403686	0.005436694	6.629671065	6.66E-05	11.43407475	2	2	6.073378139	9.184108452	9.17507637	11.02124505	10.97374872
ENSG00000164105	SAP30	5.399408467	0.000203086	6.013155197	2.66E-05	11.41256366	2	3.076665209	2	6.336376195	6.56439512	6.756113529	7.287011591
ENSG00000175592	FOSL1	6.732406483	9.83E-16	4.677196923	3.92E-08	11.40960341	2	4.11352228	5.59507254	10.92600118	10.9892592	8.766685705	9.043840859
ENSG00000138311	ZNF365	5.39037471	0.000453925	6.015568296	6.84E-05	11.40594301	2	2	2	5.627094009	5.45984938	5.899789217	6.323497254
ENSG00000134775	FHOD3	7.296428699	3.96E-14	4.102524968	4.03E-05	11.39895367	3.327711234	3.686303414	2	9.701744597	9.78435771	6.516588833	6.685996416
ENSG00000130751	NPAS1	3.984923144	0.009315299	7.402720929	2.22E-07	11.38764407	2	2	3.369317972	5.128039203	5.16377554	7.8927823	8.786963584
ENSG00000197044	ZNF441	5.521491071	0.000129797	5.857900152	4.07E-05	11.37939122	2	3.076665209	2	6.470063213	6.66650236	7.111828383	6.640162436
ENSG00000134668	SPOCD1	5.893497214	4.80E-09	5.473435492	5.69E-08	11.36693271	2	4.93638933	2	9.201414645	9.29338856	8.883077364	8.777523662
ENSG00000120899	PTK2B	5.451806831	0.000114314	5.890373966	2.44E-05	11.3421808	2	3.076665209	2	6.442929658	6.56439512	7.001702955	6.847559257
ENSG00000137819	PAQR5	4.987391995	6.56E-06	6.351352782	4.44E-09	11.33874478	3.327711234	3.686303414	2	7.461007055	7.43830714	8.262372054	9.180729263
ENSG00000101160	CTSZ	6.21095136	4.41E-08	5.114649462	8.49E-06	11.32560082	2	5.946638857	6.718950348	11.91945108	11.9583902	10.99110861	10.68108075
ENSG00000130038	CRACR2A	5.48339505	0.000418165	5.789477204	0.000164092	11.27287225	2	2	2	5.552894743	5.70538658	5.646229369	6.141542327
ENSG00000205683	DPF3	6.771568117	2.49E-06	4.499045386	0.002591993	11.2706135	2	2	2	6.846474326	6.85130017	4.799832052	4.711446447

ENSG00000166920	C15orf48	11.5690917	2.32E-08	-0.305551318	0.915553996	11.26354038	2	2	2	11.58790527	11.6068075	2.492221014	2
ENSG00000127824	TUBA4A	4.667757694	2.40E-05	6.588488736	8.15E-10	11.25624643	4.815609471	2	2	7.602596956	7.61995902	9.190394041	9.771772716
ENSG00000065308	TRAM2	4.468777569	0.000742187	6.780669714	9.65E-08	11.24944728	2	6.899080544	7.163363831	10.87457316	10.8896987	12.90512493	13.43049852
ENSG00000196850	PPTC7	5.430571601	7.44E-09	5.809834551	4.70E-10	11.24040615	2	4.442710129	2	8.291333947	8.25031674	8.559959383	8.727068782
ENSG00000172935	MRGPRF	4.355506542	0.000620294	6.883355449	1.59E-08	11.23886199	2	5.131603625	2	7.883054517	7.90193448	9.852414792	10.79623028
ENSG00000168283	BMI1	6.447989679	1.32E-08	4.775339081	3.68E-05	11.22332876	2	4.11352228	2	8.869506572	8.84440862	7.578754018	6.716538017
ENSG00000099860	GADD45B	4.599562783	1.24E-05	6.600671675	1.05E-10	11.20023446	4.00722531	5.303523747	2	8.660150093	8.71862106	11.2093021	9.836041609
ENSG00000125843	AP5S1	4.140193442	0.000887822	7.029744923	2.84E-09	11.16993837	2	3.686303414	2	5.978405189	6.16544343	8.912592153	8.864016001
ENSG00000163874	ZC3H12A	7.229784796	3.12E-17	3.91417973	9.70E-06	11.14396453	4.467311984	4.710564154	2	11.04665822	11.0621467	7.139828829	8.19690135
ENSG00000187583	PLEKHN1	7.048572396	4.58E-06	4.0875162	0.01187557	11.1360886	2	2	2	7.007926688	7.21862736	4.749835737	3.972018587
ENSG00000171951	SCG2	6.890158699	4.27E-07	4.226221142	0.002992839	11.11637984	2	3.076665209	2	7.925329813	7.87959733	5.385935071	5.337485258
ENSG00000077157	PPP1R12B	2.708821257	1.51E-06	8.386220862	3.19E-56	11.09504212	4.815609471	5.131603625	4.058998049	7.288834728	7.30683517	13.04393834	12.82549673
ENSG00000171992	SYNPO	5.059931338	3.98E-07	6.024697421	9.02E-10	11.08462876	2	3.076665209	5.39244166	8.845101141	8.78422202	9.7566591	9.790113704
ENSG00000166592	RRAD	5.935758823	3.92E-07	5.132692664	1.37E-05	11.06845149	2	4.11352228	2	8.369067528	8.32017198	7.953205078	7.0024032
ENSG00000182621	PLCB1	5.139523725	3.67E-06	5.925898384	6.08E-08	11.06542211	2	4.442710129	2	7.982979299	7.91663606	8.215119669	9.103723527
ENSG00000188559	RALGAPA2	4.914510747	5.43E-05	6.124139232	2.61E-07	11.03864998	3.327711234	3.076665209	2	6.766655021	6.88657603	8.100109146	7.910280955
ENSG00000109743	BST1	4.5979203	1.77E-09	6.416496775	9.68E-18	11.01441707	4.815609471	3.686303414	2	8.087422161	8.05816814	9.959986891	9.786796275
ENSG00000152601	MBNL1	5.279630877	0.01073585	5.734295167	0.004914121	11.01392604	2	7.315436877	2	11.04468547	10.9391381	11.53041218	11.35792713
ENSG00000100918	REC8	6.376095146	1.69E-05	4.635221512	0.002359129	11.01131666	2	2	2	6.496695859	6.44101097	4.764298067	4.975095763
ENSG00000160201	U2AF1	8.547838207	5.10E-09	2.447316431	0.139938782	10.99515464	2	2	2	8.663092307	8.51148098	2.911488689	3.539239711
ENSG00000198945	L3MBTL3	6.110562738	5.39E-07	4.877909298	8.21E-05	10.98847204	2	3.686303414	2	7.847284664	8.09525763	6.838725184	6.707209278
ENSG00000138650	PCDH10	5.944544487	2.04E-06	5.040740583	6.79E-05	10.98528507	3.327711234	3.076665209	2	7.753776273	7.90193448	7.240241221	6.580104496
ENSG00000177576	C18orf32	7.235385817	5.81E-10	3.745791518	0.002391435	10.98117733	2	3.076665209	3.369317972	9.162439586	9.05507424	5.825876956	5.611957759
ENSG00000130590	SAMD10	4.471117014	0.00360969	6.50686092	9.12E-06	10.97797793	2	2	2	4.783303513	4.68029874	6.654963382	6.530719415
ENSG00000114529	C3orf52	6.089069341	1.40E-05	4.881478659	0.000611597	10.970548	3.327711234	2	2	7.026323566	7.20671394	5.925754458	6.003555476
ENSG00000132000	PODNL1	7.389895539	6.13E-07	3.557958006	0.025915975	10.94785355	2	2	2	7.454228844	7.4450836	4.271694079	3.660199087
ENSG00000119689	DLST	5.263637208	0.001152409	5.67851475	0.000383457	10.94215196	2	6.04685525	2	9.715981692	9.68338024	10.16515422	10.05867839
ENSG00000163864	NMNAT3	5.601874889	0.000253258	5.328465976	0.000499008	10.93034087	2	2	2	5.662809983	5.81407719	5.565950232	5.408276315
ENSG00000182575	NXPH3	4.678224501	0.013191227	6.25057209	0.000556851	10.92879659	2	2	2	4.927308402	4.89348248	6.983275763	5.191251321
ENSG00000253626	EIF5AL1	6.246091011	2.39E-19	4.6806931	2.27E-11	10.92678411	5.095959458	4.11352228	4.058998049	10.41383446	10.4881343	8.464101787	9.225115665
ENSG00000155980	KIF5A	5.002609039	0.001272804	5.920780405	9.29E-05	10.92338944	2	2	2	5.242616075	5.14724905	6.162543904	5.900914815
ENSG00000198794	SCAMP5	5.298754233	0.000334643	5.621954092	0.000119053	10.92070832	2	3.076665209	2	6.5612028	6.12381559	6.423979439	6.876977108
ENSG00000187815	ZFP69	5.03835279	0.001598865	5.881915897	0.000161735	10.92026869	2	2	2	5.258265913	5.19627118	6.260165241	5.685057129
ENSG00000067066	SP100	6.66859587	1.72E-24	4.248512842	1.36E-10	10.91710871	4.467311984	4.93638933	6.43197674	12.04374227	12.0377259	9.546552631	9.702454181
ENSG00000196878	LAMB3	6.012780149	6.19E-09	4.862284359	3.48E-06	10.87506451	4.815609471	2	2	8.908460392	8.95655043	7.930140711	7.649197382
ENSG00000276497		5.407280655	0.00015131	5.466262816	0.000116623	10.87354347	2	3.076665209	2	6.490083648	6.43421531	6.412547789	6.617932662
ENSG00000140451	PIF1	5.157218495	0.000824293	5.707983961	0.000165601	10.86520246	2	2	2	5.363304456	5.30456641	5.805044075	5.871985934
ENSG00000172613	RAD9A	5.410350889	0.000176723	5.451990966	0.000144706	10.86234186	2	3.076665209	2	6.415275989	6.51372389	6.678062013	6.308152703
ENSG00000184226	PCDH9	7.531614929	2.14E-07	3.326259784	0.035010376	10.85787471	2	2	2	7.559034656	7.61696151	4.053534413	3.560117069
ENSG00000108370	RGS9	2.642628143	0.113003183	8.205551545	1.24E-08	10.84817969	2	2	2	3.227757282	3.50452879	8.542759496	7.884647945
ENSG00000137218	FRS3	5.326381895	0.000623727	5.513297654	0.000346886	10.83967955	2	2	2	5.540148523	5.43283104	5.794513725	5.508347454
ENSG00000185862	EVI2B	6.803323177	0.000344238	4.023798155	0.044848802	10.82712133	2	2	2	6.907295206	6.85130017	5.008609438	3.15612297
ENSG00000151490	PTPRO	5.509235681	0.000456779	5.307647886	0.000715544	10.81688357	2	2	2	5.590471342	5.71663161	5.238348159	5.670730563
ENSG00000133083	DCLK1	3.626557482	0.098953314	7.182976432	0.000326774	10.80953391	2	2	2	4.739373666	2.68924543	7.009312024	7.452476174
ENSG00000169604	ANTXR1	5.662394594	1.09E-08	5.141394561	2.33E-07	10.80378915	4.00722531	7.669933291	6.882775709	12.43151429	12.4430008	11.82747678	12.00074925
ENSG00000121898	CPXM2	10.33316856	2.69E-14	0.465586146	0.800845428	10.79875471	2	2	2	10.34359113	10.3839861	2.526562374	2.326754077
ENSG00000166073	GPR176	5.138532416	9.51E-06	5.658593993	8.09E-07	10.79712641	4.467311984	7.051611315	3.369317972	10.80768086	10.8906298	11.31170051	11.42383583
ENSG00000010818	HIVEP2	5.744685245	1.93E-10	5.050587506	2.58E-08	10.79527275	3.327711234	5.303523747	6.629488387	11.23490719	11.2465449	10.3054184	10.75579885
ENSG00000075223	SEMA3C	6.456987294	0.00021155	4.331481463	0.017146254	10.78846876	2	6.464272742	2	11.34457954	11.2732076	9.287461761	9.088535264
ENSG00000138347	MYPN	7.844585263	1.40E-11	2.942135878	0.02039551	10.78672114	3.327711234	3.076665209	2	9.743346323	9.68338024	5.119635168	4.861951484
ENSG00000104951	IL4I1	9.046143397	0.000978424	1.736889595	0.591567059	10.78303299	2	2	2	9.046675269	9.1189736	3.429858444	2
ENSG00000262045		7.641282711	5.12E-08	3.139146295	0.04071117	10.78042901	2	2	2	7.791909949	7.59275449	3.600766115	3.789585079
ENSG00000185652	NTF3	4.678226271	0.00284647	6.101190341	5.47E-05	10.77941661	2	2	2	4.927308402	4.89348248	6.353989995	6.044874254
ENSG00000058085	LAMC2	4.544613792	3.15E-08	6.223498924	9.55E-15	10.76811272	4.00722531	3.076665209	4.523675391	8.045195239	8.04484669	10.0231736	9.306133598
ENSG00000157625	TAB3	6.014572741	4.08E-14	4.751931877	3.27E-09	10.76650462	3.327711234	4.93638933	2	9.621581886	9.65370449	8.474034275	8.290447859
ENSG00000113361	CDH6	4.106186497	4.45E-11	6.65904277	7.45E-28	10.76522927	4.00722531	4.442710129	4.874587708	8.334420183	8.29413645	11.04040399	10.63554893
ENSG00000117016	RIMS3	2.689912473	0.121631897	8.058535206	9.04E-08	10.74844768	2	2	2	3.462030052	3.33976443	8.550081477	7.460826701
ENSG00000105339	DENND3	4.314502192	7.88E-05	6.422020238	1.12E-09	10.73652243	2	4.11352228	2	6.604700657	6.93551934	8.659546439	9.011520488
ENSG00000077092	RARB	4.408953842	3.98E-05	6.324182854	1.15E-09	10.7331367	2	4.11352228	2	6.79375511	6.94988431	8.915024678	8.569323645
ENSG00000153162	BMP6	3.600613829	0.005178798	7.114696114	3.18E-09	10.71530994	2	3.686303414	2	5.590471342	5.55061739	9.143105199	8.771941776
ENSG00000115993	TRAK2	5.715366676	0.000121908	4.993264582	0.000885779	10.70863126	3.327711234	6.602424674	2	10.75280717	10.8564076	9.622937045	10.43523469
ENSG00000248546	ANP32C	5.236922051	0.000789893	5.467817922	0.000393634	10.70473997	2	2	2	5.501219927	5.30456641	5.665620077	5.566251551
ENSG00000130775	THEMIS2	7.521719479	5.21E-11	3.174431597	0.010215226	10.69615108	2	4.11352228	2	9.932626344	9.90722553	6.092633892	5.108633494
ENSG00000204397	CARD16	8.282130891	6.80E-09	2.412746179	0.13571439	10.69487707	2	2	2	8.369067528	8.28283292	3.44788957	2.981406154
ENSG00000154319	FAM167A	10.45711719	1.87E-11	0.236654862	0.914220926	10.69377205	2	2	2	10.43808429	10.5353368	2.139668783	2.592996859
ENSG00000162104	ADCY9	4.089651108	0.002690684	6.584261618	3.86E-07	10.67391273	3.327711234	6.228547981	2	8.836004565	8.84952549	11.39000052	11.26114065
ENSG00000163131	CTSS	7.028239332	1.76E-12	3.644790896	0.000478057	10.67303023	5.330594134	4.11352228	5.59507254	11.95904309	11.9815895	9.366015681	6.862343163
ENSG00000121966	CXCR4	6.977876896	0.000205988	3.692450517	0.065664664	10.67032741	4.00722531	2	2	8.880924505	8.96835847	6.601598251	3.287118176
ENSG00000228049	POLR2J2	9.434607059	7.76E-12	1.22892022	0.470226835	10.66352728	2	2	2	9.455875035	9.48229757	2.526562374	2.817676617
ENSG00000240891	PLCXD2	4.984350042	0.00141248	5.678353634	0.000203796	10.66270368	2	2	2	5.22679461	5.13053105	5.83618166	5.785984581
ENSG00000198743	SLC5A3	5.072280526	0.000195008	5.589684428	3.19E-05	10.66196495	3.327711234	6.844429755	3.369317972	10.47077592	10.4749506	11.51325565	10.15724173
ENSG00000154188	ANGPT1	6.192472058	1.19E-09	4.466411596	1.75E-05	10.65888365	2	4.11352228	2	8.700804887	8.54970888	6.892381078	6.975457902
ENSG00000180357	ZNF609	5.331380177	0.002435814	5.30724326	0.002454553	10.63862344	2	6.389896633	2	10.08355087	10.1385468	10.14836757	10.02340748
ENSG00000215041	NEURL4	5.415291939	8.41E-08	5.217980383	2.35E-07	10.63327232	2	3.076665209	4.058998049	7.877998516	7.91419618	7.838287442	7.552234734
ENSG00000114251	WNT5A	7.284095001	1.17E-19	3.344094837	6.57E-05	10.62818984	4.815609471	7.393647515	6.203069917	13.75272914	13.7132496	10.06685284	9.470227873
ENSG00000111961	SASH1	5.128805429	3.22E-05	5.49766166	6.84E-06	10.62646709	3.327711234	7.431218269	4.874587708	11.19633074	11.23119	11.43094503	11.71902466
ENSG00000065413	ANKRD44	5.062708401	0.000553262	5.561732193	0.000116602	10.62444059	2	2	3.369317972	6.078767801	6.18985553	6.805896464	6.38329369
ENSG00000162645	GBP2	5.158161374	4.23E-05	5.465387913	1.20E-05	10.62354929	2	5.303523747	2	8.915879855	8.80675844	9.221844492	9.110373733
ENSG00000124942	AHNAK	3.825010446	1.02E-08	6.782750644	1.47E-25	10.60776109	9.174465797	9.608085847	7.777116083	12.83116471	12.8825876	15.47033881	16.09161663
ENSG00000143384	MCL1	5.862753052	1.87E-06	4.738464244	0.000148669	10.6012173	3.327711234	7.761270542	8.206335842	13.28792539	13.2609349	12.25288089	12.04110352
ENSG00000099260	PALMD	2.150635865	0.224142044	8.433407748	1.28E-08	10.58404361	2	2	2	3.289987342	2.86123014	8.822604079	8.018893446
ENSG00000145777	TSLP	5.107930615	0.001199225	5.460767704	0.000443325	10.56869832	2	2	2	5.391962469	5.18011485	5.677130623	5.540219242
ENSG00000103037	SETD6	5.012885554	0.000565281	5.526829163	0.000112508	10.53971472	2	3.076665209	2	6.061031724	6.11534392	6.710164669	6.429408106
ENSG00000121690	DEPDC7	5.546975605	0.000748006	4.990322323	0.002581427	10.53729793	2	2	2	5.614989456	5.76075522	5.502666283	4.77533149
ENSG00000115008	IL1A	9.72218958	4.79E-13	0.81360867	0.639891142	10.53579825	2	2	2	9.759931695	9.75056618	2.592885947	2.466008509
ENSG00000159200	RCAN1	4.563362809	0.03932955	5.971156161	0.005045064	10.53451897	2	7.669933291	2	10.61259734	10.6577951	11.96035313	12.11714577
ENSG00000105516	DBP	3.975078927	0.016635932	6.555492183	2.44E-05	10.53057111	2	2	2	4.358765249	4.2865223	6.972416862	6.209190898
ENSG00000144891	AGTR1	7.204979756	3.73E-07	3.314137975	0.030925245	10.51911773	2	2	2	7.242377933	7.29563107	3.782262616	3.858622973
ENSG00000268043	NBPF12	8.502373814	4.99E-25	2.011430947	0.02816633	10.51380476	3.327711234	4.710564154	2	11.86826293	11.9424648	5.780352619	5.256659864
ENSG00000129473	BCL2L2	5.715984363	0.001807112	4.7965099	0.009956274	10.51249426	2	6.602424674	2	10.67550816	10.7410309	9.517766503	10.02270259
ENSG00000101384	JAG1	1.510630705	0.347244214	8.981894419	1.41E-11	10.49252512	2	5.595927442	2	5.775854763	5.43283104	13.1475794	12.75272692
ENSG00000172780	RAB43	7.793587208	9.95E-16	2.695294881	0.010790371	10.48888209	4.00722531	2	3.369317972	10.22067068	10.3093543	5.445480974	5.171036168
ENSG00000147526	TACC1	4.512495707	1.60E-05	5.972655136	4.82E-09	10.48515084	5.095959458	6.951736442	3.369317972	10.22615207	10.2859546	11.66698686	11.75858124
ENSG00000164099	PRSS12	5.149275579	0.000139918	5.331874574	7.13E-05	10.48115015	2	5.457120953	2	9.006698952	8.99630959	9.061649957	9.294500788
ENSG00000170160	CCDC144A	4.699678514	0.003425349	5.760840738	0.000218364	10.46051925	2	2	2	4.76150579	5.07918227	5.979426183	5.79040861
ENSG00000143643	TTC13	5.108037382	4.02E-07	5.349614473	8.93E-08	10.45765185	2	3.076665209	4.058998049	7.552702688	7.63485389	7.745662833	7.912312056
ENSG00000234444	ZNF736	5.736159256	5.25E-05	4.711433848	0.001067759	10.4475931	3.327711234	2	2	6.79375511	6.76185814	5.83960027	5.772630506
ENSG00000173227	SYT12	3.643909142	0.020787524	6.792288365	3.02E-06	10.43619751	2	2	2	4.105493497	4.02226117	6.860764111	6.876977108
ENSG00000272031	ANKRD34A	4.093117478	0.007427744	6.33901228	1.22E-05	10.43212976	3.327711234	2	2	5.093559143	5.37722731	7.018390248	7.640647
ENSG00000248643	RBM14-RBM	8.582905017	3.95E-10	1.847069631	0.250975661	10.42997465	2	2	2	8.654247601	8.59306436	2.831336763	3.042026731
ENSG00000145945	FAM50B	4.244436116	0.004301255	6.172427606	1.39E-05	10.41686372	2	3.076665209	2	5.552894743	5.18011485	7.125896536	7.271271876
ENSG00000188167	TMPPE	5.081515641	0.001330054	5.334227366	0.000647675	10.41574301	2	2	2	5.22679461	5.30456641	5.413723492	5.571402115
ENSG00000158296	SLC13A3	6.114807307	0.000113586	4.268629392	0.009338878	10.3834367	2	2	2	6.061031724	6.36443784	4.806835018	4.26688425
ENSG00000142627	EPHA2	4.486411981	3.42E-09	5.893203321	2.62E-15	10.3796153	2	5.457120953	4.874587708	9.082280307	8.92660044	10.43748218	10.37446816
ENSG00000110080	ST3GAL4	5.402895735	1.92E-11	4.976310044	6.78E-10	10.37920578	3.327711234	4.93638933	2	8.99156386	9.06500642	8.660513226	8.549232824
ENSG00000169245	CXCL10	12.5294375	1.85E-20	-2.158680165	0.253232241	10.37075733	2	2	2	12.57842157	12.5348018	2.048074405	2.117335275
ENSG00000101307	SIRPB1	7.444122588	1.20E-07	2.921102697	0.058832994	10.36522528	2	2	2	7.467753569	7.53675633	3.600766115	3.496556178

ENSG00000110090	CPT1A	4.636314178	1.70E-14	5.72542173	8.87E-22	10.36173591	4.467311984	5.303523747	6.43197674	10.11933966	10.1196108	11.18909341	11.22237466
ENSG00000039139	DNAH5	4.430875509	0.005775587	5.923726453	0.000121103	10.35460196	2	2	2	4.694064037	4.70309173	5.883320383	6.182510897
ENSG00000117594	HSD11B1	9.863461927	3.60E-05	0.491027451	0.876760915	10.35448938	2	2	2	9.860122857	9.93095363	2.775316226	2
ENSG00000169252	ADRB2	5.516272079	0.000496545	4.83396541	0.002485754	10.35023749	2	2	2	5.709096143	5.6121243	5.196301326	4.878668439
ENSG00000177311	ZBTB38	5.379409309	0.002523465	4.960429568	0.00559508	10.33983888	3.327711234	7.60566235	2	11.42152442	11.445516	11.135169	10.88448866
ENSG00000163249	CCNYL1	4.826120981	1.56E-08	5.501395719	6.99E-11	10.3275167	4.467311984	3.686303414	2	7.990026167	8.07574085	8.450751194	8.913527044
ENSG00000082482	KCNK2	5.970952763	7.33E-07	4.356377122	0.000424437	10.32732989	3.327711234	3.076665209	2	7.872924734	7.84158259	6.157074092	6.418016952
ENSG00000155367	PPM1J	4.469138829	0.00686426	5.857964304	0.000228036	10.32710313	2	2	2	4.443344104	4.97065356	6.063692919	5.888587685
ENSG00000137502	RAB30	4.834325478	0.003061431	5.47430682	0.000633362	10.3086323	2	5.946638857	2	9.20242622	9.13791797	10.13138331	9.387595483
ENSG00000177675	CD163L1	6.029376308	0.000128328	4.258573328	0.008973742	10.28794964	2	2	2	6.155987605	6.12381559	4.281782022	4.784231301
ENSG00000141664	ZCCHC2	5.86505253	2.65E-06	4.38838563	0.000597178	10.25343816	2	4.93638933	2	9.206465443	9.14937381	8.17644121	7.045606259
ENSG00000114200	BCHE	6.490916412	1.26E-05	3.754267178	0.017060366	10.24518359	2	2	2	6.676346669	6.47451726	4.177580368	4.119958326
ENSG00000145390	USP53	4.461244088	0.008799219	5.780617737	0.000440009	10.24186183	2	6.140560021	2	8.957217624	9.03275482	10.14370816	10.45557294
ENSG00000099999	RNF215	4.901678349	3.79E-06	5.33999117	3.56E-07	10.24166952	2	4.11352228	2	7.234487582	7.46188679	7.786089373	7.77730333
ENSG00000170276	HSPB2	6.072701241	0.000102491	4.160038738	0.010418755	10.23273998	2	2	2	6.104969671	6.25300433	4.240999983	4.673629022
ENSG00000149968	MMP3	12.32614326	3.78E-16	-2.105632411	0.304706002	10.22051085	2	2	2	12.34266693	12.3645268	2	2.172608095
ENSG00000198113	TOR4A	5.980050313	1.43E-17	4.236441957	2.38E-09	10.21649227	3.327711234	3.686303414	4.874587708	9.675758322	9.66970392	8.05817395	7.823364542
ENSG00000168386	FILIP1L	4.493304266	6.49E-08	5.715920155	2.61E-12	10.20922442	6.563806279	6.899080544	4.058998049	10.71821594	10.6891726	12.17379072	11.62268983
ENSG00000162775	RBM15	4.863005299	3.91E-06	5.335175921	3.01E-07	10.19818122	2	4.11352228	2	7.281195074	7.35080119	7.717139459	7.837328594
ENSG00000143458	GABPB2	4.399165724	0.000613773	5.798840646	3.16E-06	10.19800637	2	2	4.058998049	6.314087501	6.40670796	7.896069536	7.511280276
ENSG00000212864	RNF208	4.127521263	0.00581032	6.068101059	2.08E-05	10.19562232	2	3.076665209	2	5.093559143	5.43283104	7.007793418	7.184857568
ENSG00000138411	HECW2	3.956502369	0.000319037	6.238038698	3.29E-09	10.19454107	2	4.93638933	2	7.250225364	7.40045674	9.376938504	9.7580035
ENSG00000198431	TXNRD1	6.350192041	6.51E-17	3.836671686	8.19E-07	10.18686373	6.138360429	8.540087354	7.858115942	14.13292277	14.1571423	11.36011126	11.86249151
ENSG00000261150	EPPK1	4.433566182	0.001787884	5.75287232	2.83E-05	10.1864385	2	3.686303414	2	6.456560224	6.18176397	8.028526139	6.990916783
ENSG00000155666	KDM8	4.839337368	0.003057116	5.345974525	0.000860079	10.18531189	2	2	2	5.076005082	5.0259382	5.294231737	5.689801209
ENSG00000166189	HPS6	4.568852222	2.59E-05	5.614510767	1.29E-07	10.18336299	2	4.93638933	2	7.875463856	7.97632944	8.812201585	9.094361437
ENSG00000134243	SORT1	2.920942758	0.010240552	7.261386584	3.61E-12	10.18232934	3.327711234	6.228547981	3.369317972	7.872924734	7.74329312	11.97242248	12.26359867
ENSG00000154553	PDLIM3	3.9941632	0.058745872	6.186388449	0.001802707	10.18055165	2	2	2	4.470469939	4.1930041	7.019897747	4.729989799
ENSG00000106785	TRIM14	6.220604582	1.11E-19	3.955451039	1.38E-08	10.17605562	2	4.710564154	5.39244166	10.52636311	10.5702365	8.256629373	8.338065764
ENSG00000095951	HIVEP1	4.528056225	0.01185213	5.642947911	0.001217827	10.17100414	2	6.389896633	2	9.298270478	9.32495871	10.28440567	10.54697728
ENSG00000160445	ZER1	4.609522631	1.48E-08	5.56085422	4.18E-12	10.17037685	2	5.595927442	5.39244166	9.374743937	9.48723044	10.45806243	10.29613416
ENSG00000120129	DUSP1	4.502317542	0.001807112	5.657132369	5.53E-05	10.15944991	3.327711234	7.572420273	4.058998049	10.6367454	10.6065803	12.17725686	11.21385686
ENSG00000105825	TFPI2	8.70548735	1.00E-07	1.448242356	0.457022495	10.15372971	8.493334308	5.595927442	2	15.78127422	15.7487679	9.001879477	7.802709293
ENSG00000177465	ACOT4	4.273636829	0.009987576	5.873402564	0.000210517	10.14703939	2	2	2	4.443344104	4.68029874	5.744329515	6.205882756
ENSG00000212710	CTAGE1	6.318874479	2.84E-05	3.828102735	0.016148442	10.14697721	2	2	2	6.429169081	6.39974839	4.088436536	4.316807515
ENSG00000165752	STK32C	4.243678226	0.003967676	5.897130032	3.02E-05	10.14080826	3.327711234	2	2	5.433909581	5.3193943	6.910373854	6.955898408
ENSG00000172260	NEGR1	3.978870286	0.025627053	6.160641307	0.000260431	10.13951159	2	2	6.073378139	8.411675819	8.31092742	10.46111737	10.59613413
ENSG00000196535	MYO18A	5.412807314	4.78E-07	4.723238592	1.32E-05	10.13604591	2	5.595927442	3.369317972	9.573133444	9.62191107	8.7818697	9.032680928
ENSG00000169184	MN1	3.691651357	0.001101338	6.443969569	1.91E-09	10.13562093	2	4.93638933	2	7.106324235	7.02417148	9.484031995	10.02716108
ENSG00000183615	FAM167B	4.483544779	0.006679598	5.649276178	0.000399175	10.13282096	2	2	2	4.694064037	4.79082474	5.536762811	5.995914157
ENSG00000260916	CCPG1	6.267787801	3.23E-35	3.864039551	4.64E-14	10.13182735	5.70932728	5.303523747	4.058998049	11.33723019	11.2569323	8.893805266	8.913527044
ENSG00000114698	PLSCR4	6.18499999	2.61E-18	3.946381472	4.53E-08	10.13138146	2	5.131603625	4.523675391	10.40023308	10.2683912	7.991357103	8.226587404
ENSG00000146376	ARHGAP18	5.863527082	4.43E-06	4.267557228	0.001141619	10.13108431	5.095959458	2	2	9.089953145	9.19028095	7.160478198	7.881541153
ENSG00000138356	AOX1	4.848578844	0.001149949	5.281727354	0.000329491	10.1303062	2	5.131603625	2	8.29513208	8.40602977	7.657374802	9.403927624
ENSG00000105894	PTN	5.909938552	1.64E-14	4.215238492	6.86E-08	10.12517704	3.327711234	4.710564154	4.058998049	9.754424396	9.75193315	7.606109069	8.425990514
ENSG00000255150	EID3	5.956108663	0.000110351	4.162285004	0.00911236	10.11839367	2	2	2	6.052081244	6.08962627	4.555645384	4.388595494
ENSG00000129667	RHBDF2	5.892457838	1.60E-09	4.217831575	2.37E-05	10.11028941	2	4.710564154	2	9.012478123	9.01236778	7.506395059	7.20156326
ENSG00000204147	ASAH2B	6.729995082	1.55E-06	3.377641548	0.024591077	10.10763663	2	3.076665209	2	7.662632018	7.82349365	4.785723309	4.434543419
ENSG00000136689	IL1RN	6.145024745	0.018875043	3.961441321	0.144697175	10.10646607	2	2	2	6.29903474	6.19790195	5.130840083	2.225841198
ENSG00000132359	RAP1GAP2	4.11664322	0.014880133	5.987799408	0.000187366	10.10444263	2	2	2	4.574143677	4.2865223	6.370627179	5.768151538
ENSG00000105963	ADAP1	5.027068052	0.000567369	5.071366993	0.000461699	10.09843505	2	2	3.369317972	6.139181293	6.06344184	6.216130818	6.066922748
ENSG00000116260	QSOX1	4.938349612	0.004382954	5.159168772	0.002657411	10.09751838	9.397585735	9.28800521	2	13.65085696	13.7273466	13.71677233	14.08098316
ENSG00000178015	GPR150	4.608276584	0.008438864	5.48742961	0.001245769	10.09570619	2	2	2	4.846787657	4.85329164	6.019162896	5.108633494
ENSG00000198824	CHAMP1	4.687370942	8.04E-05	5.403412324	3.81E-06	10.09078327	2	5.131603625	2	8.192977693	8.2657098	8.83678613	9.033147652
ENSG00000122863	CHST3	3.700540162	0.053839803	6.389190655	0.000350509	10.08973082	2	6.602424674	2	8.693628619	8.71442024	11.38839668	11.37349133
ENSG00000149380	P4HA3	7.125850819	6.51E-09	2.960469145	0.026657325	10.08631996	3.327711234	2	3.369317972	9.052296807	8.99400091	5.333556395	4.654341953
ENSG00000170054	SERPINA9	2.213792519	0.186455215	7.87204008	2.63E-08	10.0858326	2	2	2	3.227757282	3.01487888	8.022522757	7.814703611
ENSG00000164694	FNDC1	7.455820825	1.80E-07	2.603167801	0.099916399	10.05898863	2	2	5.39244166	11.0319387	11.0712094	5.614651724	6.723495198
ENSG00000158258	CLSTN2	-0.686502473	0.723460473	10.74284512	1.61E-15	10.05634265	3.327711234	2	2	2.498691189	2.2680111	11.56082781	11.88625902
ENSG00000117115	PADI2	0.693574828	0.751812007	9.337248726	5.79E-09	10.03082355	2	2	2	2.784612485	2.14022021	9.498161492	9.234480927
ENSG00000118515	SGK1	6.044624108	7.85E-14	3.982196768	1.46E-06	10.02682088	3.327711234	5.303523747	3.369317972	10.13478858	10.1843212	8.394916616	7.762743025
ENSG00000123570	RAB9B	4.678597778	0.005365654	5.335871471	0.001156865	10.01446925	2	2	2	4.984880435	4.83276841	5.201624686	5.740981724
ENSG00000184194	GPR173	5.101320308	8.04E-05	4.91172111	0.000143774	10.01304142	2	3.686303414	2	7.066878356	6.89154576	6.556607032	7.006211772
ENSG00000132718	SYT11	4.383794096	1.21E-17	5.622752907	8.74E-29	10.006547	5.095959458	4.93638933	4.874587708	9.229474035	9.10192275	10.28832008	10.50418883
ENSG00000198517	MAFK	5.167954634	0.001877118	4.83515801	0.003724409	10.00311264	6.96351253	3.076665209	2	10.5295909	10.5609081	10.1025056	10.31659934
ENSG00000179630	LACC1	6.239040206	1.34E-17	3.760620649	4.94E-07	9.999660855	4.467311984	4.11352228	2	9.699596862	9.67763759	7.249266052	7.234405279
ENSG00000196526	AFAP1	3.932163029	0.001734936	6.066460719	4.31E-07	9.998623747	6.73718411	8.077869079	3.369317972	10.88374584	10.9406373	12.74694674	13.29096652
ENSG00000125965	GDF5	4.838777421	0.005488003	5.154287069	0.002690113	9.993064489	2	2	2	4.984880435	5.11361705	4.854926465	5.689801209
ENSG00000111674	ENO2	4.695461659	0.033493947	5.297245343	0.014352343	9.992707002	2	2	7.398165132	10.43808429	10.499165	10.87325587	11.24188786
ENSG00000158869	FCER1G	4.665867641	0.015592762	5.323874194	0.004720857	9.989741835	2	2	2	4.76150579	5.0259382	6.046045499	4.553838275
ENSG00000119318	RAD23B	4.684979733	2.23E-12	5.296831376	1.11E-15	9.981811108	4.00722531	6.228547981	6.322058544	10.44877988	10.4140861	10.98378895	11.09784436
ENSG00000170485	NPAS2	3.908188356	6.42E-05	6.067865182	1.14E-10	9.976053538	2	4.710564154	2	7.012547947	7.1204461	9.167793558	9.218564658
ENSG00000095383	TBC1D2	3.925819185	0.002410798	6.046698472	9.83E-07	9.972517657	2	5.303523747	2	7.596453725	7.6957855	9.734335478	9.756591061
ENSG00000150281	CTF1	5.004475735	0.00103094	4.964904478	0.001062609	9.969380213	2	3.076665209	2	6.197166296	5.9536761	5.696113106	6.323497254
ENSG00000114670	NEK11	5.354853428	0.000792713	4.613133766	0.004247726	9.967987194	2	2	2	5.527288686	5.49945122	4.854926465	4.853519801
ENSG00000163145	C1QTNF7	1.496142873	0.397553222	8.458330535	1.64E-09	9.954473408	2	2	2	2.695456494	2.86123014	8.652274805	8.331250243
ENSG00000107863	ARHGAP21	4.899767137	6.83E-05	5.053510207	3.51E-05	9.953277344	7.031698319	6.464272742	2	11.04524939	11.0446855	11.16664251	11.22951945
ENSG00000214226	C17orf67	5.87864461	0.000176081	4.072488553	0.012349323	9.951133163	2	2	2	5.890757403	6.09824986	4.513461373	4.279527956
ENSG00000186493	C5orf38	6.27871189	0.000142503	3.67115678	0.03552107	9.949868669	2	2	2	6.470063213	6.27599024	4.434275199	3.620995423
ENSG00000103174	NAGPA	4.987177624	5.23E-06	4.952299047	5.66E-06	9.93947667	2	4.11352228	2	7.461007055	7.39696604	7.17948788	7.583161732
ENSG00000111276	CDKN1B	4.101900065	0.004901099	5.836555511	2.99E-05	9.938455576	2	5.595927442	2	8.134820652	8.06698125	9.811872597	9.830413512
ENSG00000131634	TMEM204	5.202928159	0.000376911	4.721050805	0.001326409	9.923978964	2	3.076665209	2	6.18902405	6.34282908	5.711121288	5.913137508
ENSG00000139645	ANKRD52	4.027507384	0.001119359	5.895463286	6.98E-07	9.92297067	7.940205782	6.228547981	3.369317972	10.76552522	10.7571169	12.48827223	12.75329358
ENSG00000198835	GJC2	4.099329107	0.019482624	5.818264329	0.000482998	9.917593436	2	2	2	4.497095146	4.3456612	5.449961224	6.308152703
ENSG00000125740	FOSB	5.93148272	0.000251401	3.981274425	0.018528026	9.912757145	2	3.076665209	2	6.836083475	7.08617588	5.654016955	4.341135913
ENSG00000253846	PCDHGA10	6.700069293	1.62E-09	3.211261844	0.006717774	9.911331137	2	5.131603625	2	10.20056091	10.2688688	6.880813179	6.70252219
ENSG00000145743	FBXL17	4.689708732	2.20E-06	5.221054949	9.64E-08	9.910763681	2	4.710564154	2	7.756533788	7.88956768	8.342266573	8.353844539
ENSG00000151651	ADAM8	5.140717324	0.037143257	4.758850618	0.052981698	9.899567942	2	2	2	5.334065616	5.30456641	5.805044075	2.851942824
ENSG00000091409	ITGA6	5.222553211	7.95E-07	4.663904446	1.18E-05	9.886457657	4.00722531	4.11352228	2	8.360030171	8.42329129	6.952030889	8.387816122
ENSG00000135540	NHSL1	4.418726403	0.000535144	5.457772712	1.14E-05	9.876499116	4.00722531	2	2	6.449761039	6.29111322	7.284811203	7.460826701
ENSG00000151474	FRMD4A	5.386792743	0.001902897	4.486201787	0.011008233	9.87299453	2	6.311476754	2	10.11558569	10.0585048	9.255186154	9.124459205
ENSG00000169583	CLIC3	4.796957066	0.011041916	5.073891144	0.006426257	9.870848211	2	2	2	4.846787657	5.16377554	4.478802948	5.76365862
ENSG00000134070	IRAK2	6.82924899	4.57E-21	3.039971767	6.08E-05	9.869220757	4.00722531	3.686303414	4.058998049	10.28367862	10.2953601	6.387074688	6.711881188
ENSG00000164176	EDIL3	3.75652613	2.04E-07	6.101353265	3.52E-18	9.857879395	5.532353208	7.701025996	6.718950348	10.67550816	10.5932967	13.17282734	12.75010323
ENSG00000169957	ZNF768	4.405285788	0.000517545	5.439178187	1.12E-05	9.844463975	2	5.131603625	2	7.975897842	7.90685167	9.260615815	8.589138522
ENSG00000145040	UCN2	6.086010481	4.52E-06	3.753993897	0.006933647	9.840004378	2	3.686303414	2	7.860161659	8.00642058	5.227950776	6.048572483
ENSG00000157933	SKI	4.36584549	0.012278946	5.469725777	0.001201483	9.835571267	2	7.315436877	3.369317972	10.14797511	10.1385468	11.28913979	11.19823048
ENSG00000198805	PNP	6.739020229	1.34E-10	3.096116242	0.005764691	9.835136471	2	4.93638933	2	10.03894548	10.1185514	6.710164669	6.245088435
ENSG00000081277	PKP1	0.856590506	0.674378575	8.9725828	2.94E-09	9.829173305	2	2	2	2.270786764	2.77779907	8.73537099	9.24096022
ENSG00000143387	CTSK	6.19281176	1.99E-05	3.636095999	0.017626246	9.82890776	3.327711234	6.602424674	2	11.25722675	11.308705	8.589193677	8.872386739
ENSG00000084112	SSH1	4.612004484	3.62E-08	5.198123732	3.61E-10	9.810128216	4.815609471	7.538394143	7.398165132	11.54408973	11.5956213	11.99669313	12.29866876
ENSG00000020633	RUNX3	4.063842474	0.061048542	5.744906714	0.005243018	9.808749188	2	2	2	4.299492648	4.4836996	6.601598251	4.329022995
ENSG00000184156	KCNQ3	4.228891835	0.021105714	5.579141944	0.001502148	9.808033778	2	2	2	4.574143677	4.4836996	5.056402493	6.172377306
ENSG00000205090	TMEM240	3.83869444	0.021503596	5.968331317	0.00014242	9.807025757	2	2	2	4.139692846	4.2865223	6.249924465	5.892708443
ENSG00000117069	ST6GALNAC5	6.461278565	1.49E-05	3.338581844	0.036665023	9.799860409	2	2	2	6.610808945	6.48770488	3.851628042	3.824516949
ENSG00000152223	EPG5	4.973646975	8.87E-08	4.805057692	2.33E-07	9.778704666	3.327711234	6.728495673	5.59507254	10.589972	10.7560955	10.55911238	10.45348223
ENSG00000139197	PEX5	4.906136734	4.16E-05	4.872419878	4.38E-05	9.778556612	2	5.131603625	2	8.395844809	8.49034333	8.329558253	8.487225055

ENSG00000253276	CCDC71L	6.289782742	1.60E-14	3.473714428	4.22E-05	9.76349717	4.815609471	5.838937808	7.285536013	12.53169913	12.5382719	10.07194845	9.266590264
ENSG00000126785	RHOJ	1.918112081	0.08648314	7.835491978	4.47E-16	9.75360406	3.327711234	4.11352228	2	5.058234798	4.83276841	10.17618144	11.03852583
ENSG00000168970	JMJD7-PLA2(	5.417017768	0.001788712	4.335546148	0.014425739	9.752563915	2	2	2	5.514313187	5.62411716	4.971696071	4.147809227
ENSG00000128805	ARHGAP22	5.741075765	2.95E-05	4.003550646	0.004852454	9.744626411	5.330594134	2	2	9.286870873	9.30367823	7.796681167	7.322574236
ENSG00000134594	RAB33A	3.388773887	0.050844567	6.354825786	6.29E-05	9.743599673	2	2	2	3.709785322	4.02226117	6.734240628	6.092227206
ENSG00000125730	C3	6.570393024	4.36E-06	3.166082088	0.039545549	9.736475112	2	4.442710129	2	9.283050899	9.33957954	6.678062013	4.634793542
ENSG00000230667	SETSIP	7.558452893	1.12E-05	2.173201486	0.267968334	9.73165438	2	2	2	7.650823203	7.57741621	2.526562374	3.518055793
ENSG00000174740	PABPC5	4.79754584	0.0040697	4.91807645	0.002942813	9.715622291	2	2	2	4.946755474	5.07918227	5.258920789	4.967301796
ENSG00000117245	KIF17	3.115581199	0.061794627	6.582233802	1.22E-05	9.697815002	2	2	2	3.663521627	3.69849104	6.823267818	6.490500786
ENSG00000134201	GSTM5	2.594998485	0.140051343	7.102579209	3.54E-06	9.697577694	2	2	2	3.162721474	3.50452879	6.802396951	7.462213768
ENSG00000168502	MTCL1	3.682707154	2.58E-05	6.014773788	8.75E-13	9.697480942	2	4.93638933	3.369317972	7.311513918	7.34356593	9.816433958	9.417219134
ENSG00000140511	HAPLN3	4.759620503	1.04E-05	4.933346669	4.21E-06	9.692967171	4.00722531	3.076665209	6.322058544	9.693134408	9.64563775	9.840947095	9.844043851
ENSG00000148426	PROSER2	2.370490653	0.092407051	7.315395622	2.95E-09	9.685886275	4.00722531	2	2	4.497095146	4.56059747	8.852946125	9.495172628
ENSG00000198728	LDB1	5.046869711	1.10E-07	4.63290054	1.17E-06	9.67977025	4.467311984	5.838937808	2	9.635130631	9.76417792	9.262208893	9.316140048
ENSG00000185164	NOMO2	7.571759675	5.14E-46	2.104461046	0.000215968	9.676220721	4.467311984	6.04685525	5.156626401	12.82920138	12.8778999	7.3389565	7.496463996
ENSG00000152503	TRIM36	4.484682994	0.034068642	5.191504983	0.011775054	9.676187976	2	2	2	4.867343068	4.60967607	6.034159474	4.077147165
ENSG00000115828	QPCT	4.912551649	6.85E-11	4.751631685	2.67E-10	9.664183334	3.327711234	4.442710129	3.369317972	8.402902269	8.32017198	8.291371768	8.109313767
ENSG00000010438	PRSS3	3.213684752	0.065966526	6.450171519	4.72E-05	9.663856271	2	2	2	3.840289928	3.65239003	6.121005698	6.864442837
ENSG00000069424	KCNAB2	4.851761512	1.87E-05	4.809477188	2.06E-05	9.6612387	2	4.710564154	2	7.794595599	8.10170499	8.209845962	7.543088047
ENSG00000105499	PLA2G4C	5.475634328	2.75E-08	4.181636009	3.06E-05	9.657270337	2	4.710564154	2	8.530823842	8.66303256	7.260787065	7.388257299
ENSG00000103044	HAS3	3.937408428	0.018276173	5.712081093	0.000300843	9.649489521	2	2	2	4.299492648	4.2865223	5.755231204	5.925257518
ENSG00000197779	ZNF81	4.799881637	2.84E-05	4.849493651	2.10E-05	9.649375288	2	4.11352228	2	7.178012736	7.29563107	7.550870243	6.96179415
ENSG00000074755	ZZEF1	4.404990897	0.001013885	5.24352046	6.32E-05	9.648511357	4.00722531	6.535001748	2	9.526236618	9.52849248	10.34278806	10.38181612
ENSG00000170498	KISS1	0.013475643	0.995777461	9.62855468	3.93E-11	9.642030322	2	2	2	2.498691189	2.14022021	9.852626299	9.442386533
ENSG00000158023	WDR66	4.572789645	0.007975336	5.068302221	0.002694218	9.641091866	2	2	2	4.927308402	4.70309173	4.977914261	5.486701579
ENSG00000144681	STAC	2.083293554	0.242536361	7.552463654	4.71E-07	9.635757208	2	2	2	3.227757282	2.86123014	7.86621124	7.294817518
ENSG00000135362	PRR5L	4.018233434	0.000905022	5.616297898	1.52E-06	9.634531332	2	5.131603625	2	7.59952861	7.54308677	9.0649396	9.228787577
ENSG00000253368	TRNP1	4.027268062	0.00224118	5.605953233	1.01E-05	9.633221295	2	5.303523747	2	7.772969103	7.7127321	9.170171434	9.426131059
ENSG00000004468	CD38	7.473499398	2.20E-06	2.156307426	0.231087488	9.629806825	2	2	2	7.43025113	7.62593544	3.393105702	2.70970617
ENSG00000182095	TNRC18	4.490270688	0.002137885	5.138495153	0.000341963	9.628765841	2	5.595927442	7.600047325	10.74657923	10.8020184	11.46440721	11.37744818
ENSG00000184785	SMIM10	5.031464951	0.000876133	4.592437016	0.002499057	9.623901966	2	3.076665209	2	6.113598934	6.09824986	5.413723492	5.933281326
ENSG00000184260	HIST2H2AC	9.272260933	1.09E-11	0.345701207	0.854663923	9.61796214	2	2	2	9.262828394	9.35135081	2.34604626	2.466008509
ENSG00000151376	ME3	4.364944925	0.000634997	5.252941183	2.52E-05	9.617886108	4.00722531	2	2	6.365568715	6.27599024	7.134272047	7.216434756
ENSG00000186564	FOXD2	3.303747627	0.047844479	6.300060645	3.58E-05	9.603808272	2	2	2	3.754611435	3.86943969	6.490730008	6.29575846
ENSG00000116183	PAPPA2	5.132786454	0.001862035	4.46467139	0.007405859	9.597457844	2	2	2	5.37770462	5.24368216	4.813804156	4.634793542
ENSG00000258659	TRIM34	7.315552352	4.49E-07	2.263490551	0.167292121	9.579042903	2	2	2	7.447418636	7.30311013	3.27687201	3.012034821
ENSG00000120690	ELF1	5.688112584	1.81E-15	3.886175538	9.11E-08	9.574288123	2	5.131603625	5.39244166	10.26249532	10.2158672	8.540660658	8.351600966
ENSG00000149503	INCENP	4.481391235	6.39E-05	5.087187817	3.97E-06	9.568579052	2	4.11352228	2	7.136275664	6.70088158	7.504242605	7.544398272
ENSG00000175315	CST6	2.389392098	0.191457657	7.17727049	5.11E-06	9.566662588	2	2	2	3.162721474	3.28037825	6.62753908	7.672153316
ENSG00000133107	TRPC4	2.56045088	0.121367017	6.991171485	1.52E-06	9.551622364	2	3.076665209	2	3.95996233	3.98554648	8.373261261	7.500519913
ENSG00000115419	GLS	4.05166088	2.04E-07	5.49809559	5.77E-13	9.549756469	5.532353208	7.099070191	4.874587708	10.25715053	10.0479713	11.75011531	11.43146841
ENSG00000131019	ULBP3	5.578252554	0.000993283	3.967817821	0.023750038	9.546070375	2	2	2	5.731694	5.70538658	3.943456545	4.614976609
ENSG00000171262	FAM98B	4.449388618	2.01E-07	5.092840625	1.66E-09	9.542229243	3.327711234	4.442710129	2	7.635924947	7.6318872	8.15052603	8.374617996
ENSG00000138623	SEMA7A	5.395305635	0.003175086	4.141813945	0.027327995	9.53711958	2	2	6.322058544	9.978816937	10.0945086	8.797335126	8.786410001
ENSG00000158560	DYNC1I1	4.907956502	0.003293724	4.623909948	0.005658731	9.53186645	2	2	2	5.003571641	5.21224859	4.881704255	4.845038551
ENSG00000197956	S100A6	5.090320588	3.99E-11	4.437394857	1.02E-08	9.527715445	9.097018025	7.051611315	7.502634626	13.21868427	13.2461576	12.22525492	12.86434166
ENSG00000050165	DKK3	4.699973595	2.33E-08	4.809668242	9.37E-09	9.509641838	6.366709671	8.848535039	9.031722608	13.14167328	13.1776882	13.29176405	13.24675504
ENSG00000164211	STARD4	5.020873136	1.44E-06	4.488296362	1.86E-05	9.509169498	6.366709671	5.457120953	2	10.38783677	10.2788601	9.611484085	9.97345748
ENSG00000130758	MAP3K10	4.219945678	4.70E-06	5.284915715	4.90E-09	9.504861393	3.327711234	5.131603625	2	7.913023977	8.11238697	9.222826582	8.897724096
ENSG00000188372	ZP3	4.89211992	0.00149867	4.608902462	0.002796225	9.501022382	2	3.076665209	2	5.900763176	6.04571775	5.988693817	5.367404784
ENSG00000096433	ITPR3	6.663477447	1.01E-12	2.831125258	0.004649784	9.494602705	3.327711234	4.11352228	6.203069917	11.45458917	11.5791589	7.367655602	7.989373961
ENSG00000067715	SYT1	7.985600531	2.55E-06	1.507607642	0.454406107	9.493208173	3.327711234	2	2	8.985700128	8.98472903	2.457042231	3.789585079
ENSG00000141526	SLC16A3	5.209859184	1.35E-06	4.280251617	9.03E-05	9.490110801	8.87372952	6.04685525	5.930865648	12.77948335	12.8444124	11.6021717	12.11868584
ENSG00000205609	EIF3CL	10.23675981	3.34E-14	-0.7508763	0.685511159	9.485883514	2	2	2	10.26006833	10.2755373	2.183373728	2.225841198
ENSG00000114473	IQCG	4.334295744	0.001101338	5.14231486	7.38E-05	9.476610603	2	3.686303414	2	6.197166296	6.29111322	6.770513376	7.232780814
ENSG00000185745	IFIT1	8.719931752	1.23E-11	0.756471555	0.639891142	9.476403307	7.579279349	4.442710129	3.369317972	14.85650684	14.8459655	6.529346487	7.252154709
ENSG00000226784	PGAM4	11.56603534	4.30E-14	-2.103206978	0.3091698	9.462828359	2	2	2	11.57917009	11.6092606	2	2.172608095
ENSG00000048052	HDAC9	5.160710871	2.33E-07	4.298254194	2.06E-05	9.458965066	2	4.710564154	2	8.327018759	8.24255817	7.406207255	7.469129172
ENSG00000171877	FRMD5	5.609066422	0.000555458	3.847510372	0.022853959	9.456576795	2	2	2	5.870535235	5.6121243	4.340871764	4.09155917
ENSG00000159082	SYNJ1	4.36379392	5.18E-09	5.088899036	5.18E-12	9.452692956	4.00722531	4.710564154	2	7.985332082	8.06917614	8.68304821	8.802925779
ENSG00000168014	C2CD3	4.055916603	0.000672752	5.396209813	3.09E-06	9.452126416	4.00722531	6.140560021	2	8.828161587	8.85716692	10.12141194	10.22744137
ENSG00000157111	TMEM171	5.64379386	7.06E-08	3.784973952	0.000459733	9.428767811	2	4.11352228	2	8.132699624	8.02689124	6.320128742	6.232137896
ENSG00000118473	SGIP1	4.429422363	0.001919616	4.99817985	0.000364719	9.427602212	5.330594134	2	2	7.949630994	8.04484669	8.722004124	8.376090431
ENSG00000100100	PIK3IP1	5.372946357	9.16E-06	4.054522649	0.001071222	9.427469006	2	5.131603625	2	8.848982184	8.95417718	7.782541424	7.395535532
ENSG00000157064	NMNAT2	3.7728109	0.004958412	5.652264968	1.00E-05	9.425075868	3.327711234	2	3.369317972	5.674521485	5.86549842	7.16184437	7.893928381
ENSG00000066923	STAG3	4.305151708	0.016178162	5.11663929	0.003232449	9.421790998	2	2	2	4.739373666	4.43005853	5.586442682	4.935697922
ENSG00000164023	SGMS2	3.260896055	0.008082884	6.160299144	8.54E-08	9.421195198	2	5.131603625	2	6.917187326	6.75096147	9.56215648	9.810132015
ENSG00000168056	LTBP3	4.436701143	0.05663139	4.978095552	0.028692244	9.414796695	2	8.029695761	2	10.83304867	10.9079011	11.30120057	11.51370137
ENSG00000138593	SECISBP2L	4.770018765	0.000875927	4.642948896	0.001171005	9.412967661	2	7.315436877	5.156626401	10.77813214	10.7299752	10.78923234	10.44526431
ENSG00000171428	NAT1	4.871980654	0.001047492	4.538212815	0.002318491	9.410193469	2	3.076665209	2	5.900763176	6.00960265	5.684753614	5.606950214
ENSG00000184995	IFNE	7.169095511	2.93E-06	2.239483032	0.196332247	9.408578543	2	2	2	7.214570819	7.2537885	3.374372158	2.851942824
ENSG00000182685	BRICD5	3.548766083	0.042452833	5.853033332	0.000314836	9.401799415	2	2	2	4.17310023	3.7864959	6.140539117	5.785984581
ENSG00000149201	CCDC81	1.05059529	0.548089791	8.35101951	9.92E-10	9.4016148	2	3.076665209	2	3.162721474	2.86123014	9.214621994	9.470227873
ENSG00000081386	ZNF510	4.575667798	2.66E-06	4.805909766	6.68E-07	9.381577565	3.327711234	4.11352228	2	7.433701013	7.45518898	7.925322387	7.358771532
ENSG00000057019	DCBLD2	5.882240238	1.06E-44	3.489229222	2.37E-16	9.37146946	7.031698319	7.874700259	7.029880454	13.24291444	13.230921	10.95777532	10.72579699
ENSG00000151388	ADAMTS12	4.480102774	0.000767754	4.886709519	0.000199805	9.366812294	2	7.145017454	6.43197674	10.68747873	10.6916678	10.61491551	11.45508498
ENSG00000171100	MTM1	4.70887209	1.36E-05	4.656523614	1.56E-05	9.365395703	2	4.11352228	2	7.132034771	7.19067478	7.171371525	7.04745562
ENSG00000163689	C3orf67	5.559358378	0.001451898	3.804002805	0.035956933	9.363361183	2	2	2	5.627094009	5.77157844	4.530483275	3.735548302
ENSG00000054965	FAM168A	3.993934831	0.055580779	5.369102421	0.007227479	9.363037252	2	7.099070191	2	9.469384409	9.51728265	10.79066808	10.93331374
ENSG00000166033	HTRA1	4.165111451	1.37E-08	5.18434938	7.06E-13	9.349460831	7.667647223	8.806266215	6.629488387	12.07983178	12.1570582	13.27104463	12.99040357
ENSG00000142409	ZNF787	4.507547727	0.00063259	4.840170155	0.000203725	9.347717881	2	5.303523747	2	8.139053376	8.284723	8.516835903	8.567391554
ENSG00000157353	FUK	3.874385028	0.003295139	5.468880876	1.52E-05	9.343265904	2	3.686303414	2	5.959382894	5.65950892	7.367655602	7.314917451
ENSG00000260428	SCX	5.564725446	0.00151176	3.769156546	0.038665093	9.333881992	2	2	2	5.48800675	5.89549381	4.487545924	3.735548302
ENSG00000197106	SLC6A17	3.414022188	0.090465939	5.918811937	0.001434587	9.332834126	2	2	2	3.349643765	4.2865223	6.468821191	5.414021719
ENSG00000081923	ATP8B1	3.000444915	2.48E-05	6.327044162	9.73E-21	9.327489077	6.257048221	4.11352228	5.156626401	8.29513208	8.24255817	11.45119913	11.69592421
ENSG00000101596	SMCHD1	5.33287477	2.00E-05	3.993650253	0.00183886	9.326525023	4.467311984	6.535001748	2	10.54681762	10.5189709	9.447810736	8.899772939
ENSG00000205517	RGL3	4.535724254	0.010654158	4.785207901	0.006279821	9.320932154	2	2	2	4.907595604	4.65713986	5.258920789	4.692661645
ENSG00000129048	ACKR4	6.964812535	1.05E-08	2.345660989	0.083161849	9.310473524	2	3.686303414	2	8.857997553	8.77886778	4.281782022	4.644600858
ENSG00000121281	ADCY7	5.688117803	2.33E-12	3.619583761	1.42E-05	9.307701564	2	4.93638933	3.369317972	9.308641573	9.3166689	7.231159579	7.313381205
ENSG00000182492	BGN	3.832407867	0.00024625	5.474583188	6.09E-08	9.306991055	9.886101849	7.701025996	6.718950348	12.49501766	12.5683571	14.05464728	14.28359817
ENSG00000130024	PHF10	3.977742492	0.000425977	5.322786409	1.15E-06	9.3005289	3.327711234	5.595927442	2	8.195011988	8.14188248	9.699953617	9.271345616
ENSG00000138172	CALHM2	3.877292691	0.001742493	5.422613057	5.41E-06	9.299905748	2	5.131603625	2	7.388195945	7.47187556	9.075488822	8.820877391
ENSG00000078053	AMPH	5.012451268	0.001326022	4.284376767	0.006766036	9.296828035	2	3.686303414	2	6.815074525	6.90143415	5.169385838	6.746446439
ENSG00000163625	WDFY3	4.448472221	0.010175586	4.845854042	0.004483519	9.294326263	2	7.274681896	3.369317972	10.1755382	10.1969221	10.52713712	10.6357026
ENSG00000100393	EP300	4.77325981	0.000140189	4.518825882	0.000318465	9.292085691	2	5.946638857	7.029880454	10.68204987	10.7454982	10.51355748	10.40508119
ENSG00000105643	ARRDC2	5.459323749	0.000179763	3.828108585	0.011275691	9.287432334	2	5.595927442	2	9.376537567	9.50436385	8.086505553	7.512619696
ENSG00000221995	TIAF1	6.444132427	6.79E-06	2.839835025	0.06836276	9.283967452	2	3.076665209	2	7.555872146	7.36873214	3.968654369	4.376876162
ENSG00000118762	PKD2	3.563125295	3.04E-07	5.711844276	2.68E-17	9.274969571	6.009025997	7.431218269	5.930865648	10.17141133	10.1639279	11.83979864	12.66796169
ENSG00000179456	ZBTB18	3.666653424	0.000413591	5.607111147	1.75E-08	9.273764571	2	4.442710129	2	6.658765962	6.40670796	8.522164399	8.335797499
ENSG00000060140	STYK1	4.288815185	0.030317415	4.980456948	0.009741936	9.269272132	2	2	2	4.497095146	4.65713986	4.360042493	5.694529741
ENSG00000178773	CPNE7	5.907971112	3.98E-05	3.358922264	0.027424614	9.266893376	2	5.303523747	2	9.561354269	9.60839132	6.231826305	7.599641096
ENSG00000137203	TFAP2A	3.508384158	0.000968694	5.754190144	1.27E-08	9.262574303	2	4.442710129	2	6.496695859	6.26070706	8.3832514	8.741991736
ENSG00000181619	GPR135	4.14467377	0.007220064	5.105905741	0.000630205	9.250579511	2	3.076665209	2	5.144975053	5.41912973	6.031172603	6.308152703
ENSG00000170373	CST1	3.993161482	0.028289069	5.248902369	0.002615599	9.242063851	2	2	2	4.329433326	4.3456612	4.984105765	5.7500953
ENSG00000198722	UNC13B	3.864817549	0.001319542	5.370158688	3.73E-06	9.234976237	3.327711234	5.72254323	2	8.153770873	8.17690623	9.342112202	9.916412852
ENSG00000083454	P2RX5	6.073705754	0.000934242	3.161106199	0.107213033	9.234811953	2	2	2	6.276156955	6.08095082	4.166733998	3.042026731
ENSG00000185158	LRRC37B	5.21439679	0.000170463	4.013736191	0.004739329	9.228132981	4.00722531	2	2	7.161462517	7.14145882	6.387074688	5.475755622

ENSG00000167978	SRRM2	4.213683134	7.32E-14	5.007891421	2.42E-19	9.221574555	8.257427002	8.929529568	7.342949392	12.5192709	12.5228406	13.29033993	13.33894516
ENSG00000111199	TRPV4	4.826350842	0.000209355	4.391213526	0.000791158	9.217564368	2	3.686303414	2	6.542151749	6.8715633	6.344396176	6.254725615
ENSG00000112425	EPM2A	4.249269982	0.001105783	4.968264008	9.61E-05	9.217533991	2	3.686303414	2	6.104969671	6.22964624	6.804147769	6.905807071
ENSG00000188906	LRRK2	5.421562237	1.59E-05	3.795889199	0.003429913	9.217451436	2	3.686303414	2	7.273514749	7.32163988	5.692336538	5.799216178
ENSG00000137198	GMPR	6.414743772	1.91E-10	2.789903486	0.010112025	9.204647258	2	4.442710129	2	9.219515356	9.23788597	5.427419529	5.945233978
ENSG00000178607	ERN1	4.793814136	5.90E-08	4.40623318	6.65E-07	9.200047316	2	3.686303414	4.523675391	8.04293816	7.97398853	7.865372957	7.343799539
ENSG00000196754	S100A2	4.74410065	0.008829144	4.454237696	0.014029183	9.198338346	2	2	2	4.867343068	5.06165168	4.311629396	5.035976547
ENSG00000187801	ZFP69B	4.897221216	0.001721482	4.296014639	0.006440115	9.193235856	2	3.076665209	2	5.920568914	6.03677338	5.725974948	5.04340913
ENSG00000181773	GPR3	4.69989795	0.013126122	4.492002871	0.017626246	9.191900821	2	2	2	4.804776789	5.04390545	4.155805465	5.171036168
ENSG00000141753	IGFBP4	4.356853012	3.02E-24	4.834898145	4.91E-30	9.191751157	9.579430841	9.566483142	8.699419242	13.66216008	13.7119771	14.35551441	13.94591334
ENSG00000122254	HS3ST2	2.688097229	0.167050868	6.498999846	0.000153586	9.187097075	2	2	2	3.227757282	3.55551834	7.087593115	5.812327524
ENSG00000184374	COLEC10	5.369307178	0.001440368	3.813946263	0.028788809	9.18325344	2	2	2	5.590471342	5.45984938	4.388329244	3.972018587
ENSG00000108846	ABCC3	6.969398152	3.18E-09	2.210397383	0.092310672	9.179795535	2	2	5.156626401	10.25178588	10.2779115	5.673303969	5.571402115
ENSG00000189058	APOD	4.361743477	0.033609824	4.817221442	0.01663167	9.178964919	2	2	2	4.623314311	4.65713986	4.076896059	5.601925227
ENSG00000198752	CDC42BPB	4.714503213	2.56E-11	4.459548247	2.86E-10	9.17405146	4.467311984	6.844429755	6.534109959	10.91709408	10.9155453	10.69965825	10.62320178
ENSG00000159212	CLIC6	3.080716141	0.110520142	6.092501167	0.000488026	9.173217308	2	2	2	3.515101962	3.7864959	5.472156123	6.681239787
ENSG00000187957	DNER	8.059874221	1.31E-14	1.109816135	0.376931525	9.169690355	2	4.11352228	2	10.50274314	10.4477945	3.694366604	4.175132639
ENSG00000166741	NNMT	5.713820895	0.000312208	3.454605337	0.038310303	9.168426232	5.330594134	2	2	9.268635235	9.28774494	7.657374802	5.995914157
ENSG00000156802	ATAD2	3.949460218	5.30E-05	5.216292322	4.03E-08	9.16575254	3.327711234	4.442710129	2	7.210554257	7.04216061	8.697250351	7.949380652
ENSG00000106780	MEGF9	4.871502098	5.10E-18	4.289926009	2.42E-14	9.161428106	4.467311984	5.131603625	4.874587708	9.512436154	9.56938265	9.156530478	8.740277714
ENSG00000105612	DNASE2	4.943154684	2.51E-11	4.19926985	1.78E-08	9.142424534	4.467311984	5.838937808	3.369317972	9.69385389	9.76350037	9.068585983	8.906411656
ENSG00000064726	BTBD1	4.399666174	2.30E-13	4.736437884	1.82E-15	9.136104058	4.467311984	6.228547981	5.156626401	9.805913872	9.78368958	10.19532025	10.06211278
ENSG00000170027	YWHAG	4.25201471	1.92E-14	4.871323451	8.03E-19	9.123338161	6.89194353	7.051611315	8.206335842	11.72109746	11.7227089	12.1422582	12.51497152
ENSG00000128739	SNRPN	5.649836859	4.38E-18	3.473106729	1.82E-07	9.122943587	3.327711234	4.442710129	4.523675391	9.57078529	9.46239569	7.313367756	7.421437279
ENSG00000164266	SPINK1	2.936187977	0.178578057	6.184080836	0.001643664	9.120268813	2	2	2	3.406930997	3.69849104	6.984820384	4.878668439
ENSG00000137501	SYTL2	3.590470427	0.021105714	5.527422273	0.000171403	9.1178927	2	3.686303414	2	5.552894743	5.49945122	7.900986387	6.512090157
ENSG00000096696	DSP	2.476191041	0.003147211	6.630262886	1.18E-17	9.106453927	6.563806279	7.701025996	9.014156722	10.53039672	10.56791	14.72627191	14.67264228
ENSG00000163053	SLC16A14	3.918402359	0.036751137	5.17895821	0.003863831	9.097360569	2	2	2	4.299492648	4.2560182	5.73334482	4.836507146
ENSG00000158406	HIST1H4H	5.97031586	0.001880748	3.12221573	0.127753231	9.09253159	2	2	2	6.104969671	6.06344184	2.911488689	4.188602707
ENSG00000176842	IRX5	6.515388372	1.51E-05	2.574987365	0.121083356	9.090375737	2	2	2	6.496695859	6.70088158	3.374372158	3.287118176
ENSG00000138688	KIAA1109	4.232180426	4.00E-05	4.835912491	1.89E-06	9.068092917	3.327711234	6.899080544	5.39244166	9.972308137	10.0272353	10.7919921	10.38273198
ENSG00000167470	MIDN	4.26033537	0.000594915	4.804576212	8.21E-05	9.064911581	2	6.228547981	4.058998049	9.083378928	9.16587611	9.556194935	9.768412794
ENSG00000025708	TYMP	6.41876226	7.62E-11	2.64612842	0.013130749	9.064890679	2	4.442710129	2	11.02223255	11.0469145	7.561260509	6.97156716
ENSG00000165084	C8orf34	3.560303972	0.031536406	5.502775705	0.000409205	9.063079676	2	2	3.369317972	4.739373666	4.811949	6.010089548	6.947999751
ENSG00000113083	LOX	4.880424182	0.000353266	4.18001283	0.002518254	9.060437012	8.777722034	8.702538795	3.369317972	13.04475171	13.0269828	12.37356765	12.29721225
ENSG00000206560	ANKRD28	4.938183536	0.000613202	4.117673061	0.004914836	9.055856597	4.00722531	6.844429755	2	10.33485359	10.3319235	9.628876603	9.394150543
ENSG00000164308	ERAP2	3.969864118	0.000200919	5.079476025	1.05E-06	9.049340143	4.00722531	3.076665209	6.203069917	8.769305409	8.78955647	9.683639816	10.05638425
ENSG00000131480	AOC2	4.776691808	0.038740151	4.27099541	0.064751221	9.047687218	2	2	2	4.965943888	5.0259382	5.269098128	3.128433649
ENSG00000056736	IL17RB	4.560451638	0.011297503	4.484289849	0.012268798	9.044741488	2	2	2	4.907595604	4.70309173	4.990270811	4.445805334
ENSG00000153993	SEMA3D	4.691606872	0.003501197	4.352610195	0.006975079	9.044217066	2	2	4.058998049	6.804454198	6.54560135	5.289239922	6.963754056
ENSG00000173846	PLK3	5.358424429	0.000880586	3.684280805	0.027686483	9.042705234	2	5.946638857	2	9.677214349	9.70612498	8.252146986	7.773956279
ENSG00000183111	ARHGEF37	2.700789913	0.017734312	6.337430973	1.06E-09	9.038220886	4.467311984	2	2	5.258265913	5.39133044	8.791168836	8.856651551
ENSG00000023171	GRAMD1B	4.4004533	3.31E-05	4.617024456	1.12E-05	9.017477756	2	4.710564154	2	7.504305792	7.55253065	7.857806412	7.613439639
ENSG00000169047	IRS1	4.272685452	0.000900252	4.741108817	0.000182815	9.013794269	2	5.946638857	3.369317972	8.733363582	8.80675844	9.448650487	8.984746522
ENSG00000233822	HIST1H2BN	6.276751247	8.24E-06	2.735817246	0.074484213	9.012568493	3.327711234	2	2	7.352188525	7.24992376	4.076896059	4.133950982
ENSG00000124788	ATXN1	3.796772796	0.090096447	5.215768466	0.014623638	9.012541263	2	7.538394143	2	9.80191506	9.67331554	11.14478699	11.16273299
ENSG00000171051	FPR1	6.281310078	4.22E-05	2.726609731	0.103755371	9.007919809	2	2	2	6.276156955	6.47451726	3.374372158	3.474731315
ENSG00000213160	KLHL23	1.778869165	0.362317951	7.226800848	7.76E-06	9.005670012	2	2	2	3.09461492	2.68924543	7.702194078	6.711881188
ENSG00000136237	RAPGEF5	3.585400667	0.027691078	5.412342745	0.00042866	8.997743411	3.327711234	2	2	4.76150579	4.83276841	6.804147769	6.026239558
ENSG00000132294	EFR3A	5.141287956	0.013783233	3.840496178	0.072675764	8.981784135	2	7.315436877	2	10.86054366	10.8487621	9.661838508	9.446947471
ENSG00000189337	KAZN	3.105431582	0.042682089	5.868211478	3.37E-05	8.97364306	2	5.595927442	2	7.106324235	7.14145882	9.658938866	10.02058586
ENSG00000056586	RC3H2	3.85195384	0.000170463	5.118724625	2.77E-07	8.970678465	4.00722531	6.899080544	4.523675391	9.490242771	9.52849248	10.73013842	10.81195485
ENSG00000163960	UBXN7	3.693180416	0.000170033	5.276578681	2.79E-08	8.969759097	2	5.838937808	5.930865648	8.902248225	8.9350493	10.586203	10.39585643
ENSG00000186470	BTN3A2	5.514567362	1.10E-09	3.451641634	0.000226682	8.966208995	3.327711234	6.728495673	5.930865648	11.31238029	11.3353933	9.185700723	9.346125282
ENSG00000006606	CCL26	4.947124716	0.004419898	4.012780719	0.023554727	8.959905435	2	2	2	5.194621444	5.09650239	4.521997428	4.161535617
ENSG00000249992	TMEM158	7.718838195	1.84E-11	1.240106639	0.363255554	8.958944834	6.366709671	4.442710129	2	12.6386094	12.7873956	6.645228724	5.871985934
ENSG00000188157	AGRN	4.286140565	8.16E-08	4.668578928	3.84E-09	8.954719493	9.2903348	8.414402626	6.80318718	12.74770046	12.7758512	12.87005887	13.37427865
ENSG00000111912	NCOA7	4.7472518	8.06E-22	4.202885665	1.61E-17	8.950137466	6.138360429	7.051611315	6.322058544	11.32427804	11.2159175	10.40063634	10.99438813
ENSG00000113805	CNTN3	7.231325156	1.35E-09	1.715914515	0.211573817	8.947239671	2	2	4.058998049	9.157231967	9.09439916	4.133696865	3.875378318
ENSG00000152409	JMY	3.956103177	0.000155707	4.991100099	1.01E-06	8.947201475	4.00722531	5.595927442	2	8.300810597	8.29037844	9.661114144	8.875513287
ENSG00000241697	TMEFF1	6.635392849	1.09E-05	2.311546548	0.170499056	8.946939397	2	2	2	6.804454198	6.6253296	3.27687201	3.071407821
ENSG00000119917	IFIT3	8.16509734	2.82E-07	0.768823264	0.699688818	8.933920604	2	5.838937808	8.380957451	15.14069964	15.1709914	7.182183216	8.210170621
ENSG00000109321	AREG	9.125028253	4.75E-11	-0.218224377	0.910973565	8.906803876	2	3.076665209	2	10.11665925	10.1201401	2.34604626	2.671842496
ENSG00000135914	HTR2B	1.690223964	0.361833497	7.216223311	2.00E-06	8.906447275	2	2	2	2.947924294	2.77779907	6.932953913	7.56002886
ENSG00000111266	DUSP16	4.857319668	5.62E-05	4.043167396	0.000945348	8.900487064	3.327711234	5.838937808	2	9.257971441	9.24663965	8.517369639	8.369452607
ENSG00000187942	LDLRAD2	3.899565296	0.034437161	5.000204583	0.004698955	8.899769879	2	2	2	4.358765249	4.16043394	4.868377487	5.459179378
ENSG00000008294	SPAG9	4.773769694	3.42E-11	4.114380198	1.36E-08	8.888149892	6.366709671	7.819099893	5.772716771	11.66459642	11.6718015	11.0292199	10.99018765
ENSG00000198053	SIRPA	4.194106351	0.000939403	4.693283818	0.00016843	8.88739017	7.031698319	5.946638857	2	10.08684133	10.1984268	10.40294892	10.8459572
ENSG00000152049	KCNE4	3.20527256	0.00056585	5.677365834	1.34E-10	8.882638394	5.532353208	3.076665209	5.772716771	8.139053376	8.25224987	11.04993501	10.10247774
ENSG00000142611	PRDM16	3.75755215	0.052169147	5.123505645	0.005357536	8.881057795	2	2	2	4.415698455	3.82856379	5.626574484	4.886954834
ENSG00000186174	BCL9L	4.333012226	0.014995326	4.54247202	0.009917482	8.875484247	2	4.710564154	8.236946975	11.03165415	11.100209	11.21128275	11.33681566
ENSG00000159399	HK2	5.060373974	0.000341058	3.80732126	0.008757632	8.867695234	7.096806373	7.790474954	2	11.91235914	11.9600175	10.50913655	10.84236645
ENSG00000160460	SPTBN4	2.69035537	0.127930027	6.176350878	8.09E-05	8.866706248	2	2	2	3.515101962	3.28037825	6.265258498	6.29264319
ENSG00000168679	SLC16A4	5.269751219	2.54E-23	3.595242251	1.85E-11	8.86499347	4.467311984	4.710564154	5.59507254	10.08738902	10.0311812	8.331987532	8.459018364
ENSG00000008086	CDKL5	3.657144508	4.62E-07	5.203413808	1.76E-13	8.860558316	2	4.442710129	5.156626401	7.734324667	7.70710533	9.323615907	9.170158797
ENSG00000048471	SNX29	4.357269646	5.91E-08	4.499147832	1.78E-08	8.856417477	2	4.442710129	5.156626401	8.432517176	8.38855922	8.245719321	8.802925779
ENSG00000115902	SLC1A4	1.876951314	0.363985262	6.974728426	8.35E-05	8.851679739	2	6.602424674	2	7.075737784	6.73446058	12.03768528	11.8900644
ENSG00000180257	ZNF816	4.403750004	0.001160148	4.43236426	0.000997654	8.836114263	2	3.686303414	2	6.321555392	6.29111322	6.560755617	6.06327132
ENSG00000153714	LURAP1L	5.500080598	1.98E-11	3.328905846	8.70E-05	8.828986444	3.327711234	4.93638933	3.369317972	9.279220784	9.35944426	7.508544306	6.753261212
ENSG00000164506	STXBP5	4.943824931	0.008424242	3.882546272	0.042592362	8.826371204	2	6.602424674	2	9.960996031	9.91578828	9.023457772	8.72879856
ENSG00000274290	HIST1H2BE	7.706299672	6.07E-08	1.118303045	0.523326453	8.824602717	2	2	2	7.770242853	7.74878077	2.560105256	2.70970617
ENSG00000134516	DOCK2	4.830339166	0.00023646	3.993386186	0.002779311	8.823725353	4.00722531	2	2	6.682159616	6.8563927	5.798032389	6.13459968
ENSG00000175946	KLHL38	2.012037713	0.265703867	6.809802605	8.10E-06	8.821840319	2	2	2	3.09461492	2.94009902	7.091899617	6.645056213
ENSG00000150556	LYPD6B	4.743744471	0.007283609	4.067666523	0.022954732	8.811410994	2	2	2	4.804776789	5.11361705	4.311629396	4.479073019
ENSG00000027075	PRKCH	1.588461057	0.143712254	7.221267023	5.01E-15	8.809728081	3.327711234	4.442710129	2	5.022023783	4.83276841	9.887932872	10.72261753
ENSG00000239704	CDRT4	7.772291328	6.03E-08	1.028217102	0.564110709	8.80050843	2	2	2	7.711768301	7.92877397	2.68698273	2.509591799
ENSG00000161955	TNFSF13	5.148183554	0.002492025	3.639273535	0.038718339	8.787457089	2	2	2	5.210797711	5.43283104	4.029788235	4.09155917
ENSG00000179772	FOXS1	-1.684535475	0.428119634	10.46953201	2.11E-12	8.784996532	3.327711234	2	2	2.3892346	2	11.78416757	11.04050035
ENSG00000100055	CYTH4	4.132509496	0.054329477	4.652256268	0.026219867	8.784765764	2	2	2	4.470469939	4.43005853	5.47655442	3.875378318
ENSG00000130589	HELZ2	6.012493091	4.63E-10	2.769822063	0.007277007	8.782315155	5.095959458	7.701025996	5.156626401	12.47045468	12.5434121	9.307967678	9.237319199
ENSG00000174004	NRROS	5.693927948	0.003801199	3.083717045	0.14026642	8.777644993	2	2	2	5.627094009	6.00043082	4.144793515	2.918126211
ENSG00000132357	CARD6	5.46942958	1.54E-12	3.306124352	3.44E-05	8.775553931	3.327711234	3.686303414	5.39244166	9.54788723	9.55457603	7.141214687	7.644317657
ENSG00000141905	NFIC	4.228418833	1.18E-13	4.545449851	9.39E-16	8.773868684	7.096806373	6.04685525	7.646393253	11.21699976	11.260058	11.40695016	11.68972084
ENSG00000138604	GLCE	4.82261625	0.000673951	3.944971549	0.006315896	8.7675878	2	5.457120953	2	8.709369553	8.63367258	7.610117916	7.978752403
ENSG00000163635	ATXN7	4.577930579	4.30E-08	4.183105897	6.07E-07	8.761036475	2	5.595927442	4.874587708	9.172798677	9.20032903	8.836358413	8.752233325
ENSG00000198648	STK39	3.964476128	0.003618428	4.793675344	0.000308673	8.758151471	3.327711234	6.140560021	2	8.556367783	8.71301725	9.391568904	9.524680706
ENSG00000068654	POLR1A	3.974597393	0.009844814	4.782326074	0.001419617	8.756923467	3.327711234	6.602424674	2	9.00785664	9.1347778	9.751677657	9.990140122
ENSG00000061337	LZTS1	1.377678148	0.334988875	7.371895952	5.10E-10	8.7495741	2	2	5.156626401	4.965943888	4.79082474	10.49971375	10.81927984
ENSG00000180855	ZNF443	4.723324601	0.011100462	4.015939843	0.032745113	8.739264444	2	2	2	5.022023783	4.87352701	4.667592615	3.956354764
ENSG00000163485	ADORA1	5.63166593	0.000525107	3.104415889	0.072475386	8.736081819	2	2	2	5.720439317	5.81407719	3.648325314	3.698365557
ENSG00000052795	FNIP2	4.127242846	0.044781658	4.602149567	0.022444642	8.729392413	2	7.002537973	2	9.575477782	9.48064953	10.06009163	9.940497614
ENSG00000151276	MAGI1	3.7386577	0.000167805	4.982258644	2.42E-07	8.720916344	3.327711234	5.303523747	2	7.671425626	7.70428369	8.997677834	8.827888359
ENSG00000177575	CD163	1.935682413	0.307741842	6.781936302	2.05E-05	8.717618715	2	2	2	2.784612485	3.15372745	7.139828829	6.509409088
ENSG00000107130	NCS1	4.264739673	0.000798129	4.451372046	0.000410013	8.716111719	2	6.899080544	5.59507254	9.977635701	10.0722485	10.0536666	10.35406616
ENSG00000139625	MAP3K12	4.544606119	1.54E-07	4.161442703	1.66E-06	8.706048823	5.330594134	3.686303414	2	8.485003881	8.40776525	8.085786009	8.051511707
ENSG00000146416	AIG1	4.150213419	0.00590766	4.555734894	0.002143773	8.705948312	2	5.595927442	2	8.14116509	8.14812525	8.438403662	8.644559262
ENSG00000182578	CSF1R	3.244230898	0.078987729	5.457595397	0.001364214	8.701826295	2	2	2	3.79808662	3.74316441	5.85994354	5.300743019

ENSG00000166750	SLFN5	5.645927178	0.001311774	3.051054841	0.102207077	8.696982019	8.609439662	5.131603625	2	12.76808443	12.7631125	10.36682853	9.953377568
ENSG00000174943	KCTD13	4.464865671	5.21E-08	4.230147505	2.48E-07	8.695013176	4.467311984	4.11352228	2	7.872924734	7.97866656	7.899349298	7.459438299
ENSG00000162511	LAPTM5	1.889106343	0.31619153	6.804277836	2.44E-05	8.693384178	3.327711234	3.076665209	2	3.881293667	4.31639476	9.481845891	6.605433158
ENSG00000153029	MR1	3.843200016	2.59E-16	4.844771988	1.16E-25	8.687972003	4.815609471	5.595927442	5.39244166	9.03081742	9.03162973	10.07231173	9.980734518
ENSG00000166016	ABTB2	5.030963174	3.48E-10	3.656702346	7.78E-06	8.687665521	4.00722531	3.686303414	3.369317972	8.192977693	8.28094035	6.977080688	6.804465095
ENSG00000110218	PANX1	4.924683509	3.39E-05	3.762447747	0.00197542	8.687131256	6.653098118	6.602424674	2	10.92262909	10.9213279	9.542625156	9.954856344
ENSG00000134013	LOXL2	3.910757536	4.55E-07	4.776289054	3.92E-10	8.68704659	9.895281535	9.726146444	7.600047325	13.24586113	13.3136663	14.05496899	14.23054703
ENSG00000186765	FSCN2	2.735469455	0.175994512	5.942462043	0.001005841	8.677931498	2	2	2	3.615725078	3.2184422	6.544089217	5.319231103
ENSG00000126882	FAM78A	3.548820592	0.110727349	5.127683283	0.01461872	8.676503875	2	2	2	4.17310023	3.7864959	5.941748584	4.147809227
ENSG00000242221	PSG2	2.391008583	0.206839295	6.281485465	0.000142458	8.672494048	2	2	2	3.349643765	3.08597292	5.925754458	6.723495198
ENSG00000169499	PLEKHA2	3.918321376	1.02E-13	4.751461885	6.52E-20	8.669783261	5.70932728	4.442710129	5.59507254	9.085573664	9.06500642	9.821413244	9.981702022
ENSG00000119900	OGFRL1	5.082680168	1.35E-23	3.586693001	2.39E-12	8.669373168	4.467311984	5.72254323	5.39244166	10.27264434	10.2355442	8.646918883	8.877593894
ENSG00000011454	RABGAP1	3.863894114	0.009737709	4.802950072	0.000925327	8.666844186	4.467311984	7.099070191	2	9.50916971	9.59626647	10.67247936	10.27813351
ENSG00000136040	PLXNC1	3.115211289	0.118797449	5.549694002	0.002463699	8.664905291	2	2	2	3.615725078	3.74316441	6.165271053	4.982847849
ENSG00000182134	TDRKH	3.282596501	0.042407869	5.38155109	0.000354475	8.664147591	2	3.076665209	2	4.598938466	4.4836996	6.162543904	6.669279147
ENSG00000134463	ECHDC3	3.213368236	0.074785353	5.441540968	0.00106239	8.654909204	2	2	2	3.79808662	3.69849104	5.65789504	5.524371359
ENSG00000164309	CMYA5	6.550094872	2.17E-05	2.104505109	0.227259568	8.654599981	2	2	2	6.5612028	6.70653264	2.911488689	3.209956562
ENSG00000121289	CEP89	3.573552177	0.002199247	5.079409191	5.90E-06	8.652961367	2	4.93638933	2	7.030886362	6.8563927	8.416251718	8.426701165
ENSG00000135766	EGLN1	4.21150811	0.000734564	4.438947428	0.000325136	8.650455538	4.00722531	7.35507212	4.058998049	10.163122	10.2094139	10.31312388	10.50671159
ENSG00000170634	ACYP2	4.209434957	8.24E-05	4.436160618	2.75E-05	8.645595574	3.327711234	3.686303414	2	6.682159616	6.71776877	6.768721236	7.05482945
ENSG00000135363	LMO2	3.776712715	0.046133395	4.865065777	0.007316377	8.641778492	2	2	2	4.139692846	4.1930041	4.705557986	5.367404784
ENSG00000177352	CCDC71	3.793507094	0.001214951	4.848091067	2.04E-05	8.641598162	2	4.93638933	2	7.127781374	7.18663697	8.092249024	8.28340307
ENSG00000123700	KCNJ2	4.198925526	0.031426015	4.435206346	0.020987656	8.634131871	2	2	2	4.739373666	4.22485516	5.020706951	4.292061816
ENSG00000196943	NOP9	4.514739579	2.27E-09	4.118787746	5.33E-08	8.633527325	5.095959458	5.457120953	2	9.20242622	9.07159009	8.763089655	8.728222198
ENSG00000151952	TMEM132D	1.39154689	0.556481113	7.241566745	0.000163335	8.633113635	2	2	2	2.868578001	2.59489879	5.582367412	8.068003971
ENSG00000173517	PEAK1	3.762709178	0.001267977	4.866623243	1.75E-05	8.629332421	5.866944937	7.954278496	4.058998049	10.43808429	10.5201748	11.54344182	11.61849721
ENSG00000125686	MED1	4.189417566	0.047220426	4.436335062	0.032697033	8.625752627	2	7.189546432	2	9.759244431	9.79831747	10.00178175	10.04740207
ENSG00000104756	KCTD9	4.611056131	0.002344951	4.012587793	0.00887817	8.623643924	3.327711234	6.602424674	2	9.797905134	9.60612566	8.975319061	9.233668966
ENSG00000047365	ARAP2	4.081212359	0.101317769	4.542129765	0.060381002	8.623342124	2	2	2	4.329433326	4.4836996	5.545162502	3.128433649
ENSG00000095203	EPB41L4B	2.859406534	0.112536896	5.752258743	0.000393732	8.611665277	2	2	2	3.515101962	3.50452879	5.758846871	5.992078266
ENSG00000177508	IRX3	5.031313049	0.001032336	3.57540954	0.024293372	8.606722589	2	3.076665209	2	6.087554739	6.12381559	4.990270811	4.553838275
ENSG00000112343	TRIM38	5.592867965	8.78E-09	3.011134606	0.003301386	8.604002571	4.467311984	6.844429755	4.058998049	11.20493678	11.195718	8.69913345	8.55898913
ENSG00000127399	LRRC61	6.749806174	2.13E-05	1.847242261	0.310818746	8.597048435	2	2	2	6.772115916	6.88158913	2.68698273	3.15612297
ENSG00000137628	DDX60	6.51145857	2.68E-22	2.084283292	0.00396904	8.595741861	4.815609471	5.72254323	3.369317972	11.30956651	11.3218808	7.116063252	6.725806822
ENSG00000171055	FEZ2	4.453093635	4.93E-11	4.142576536	1.03E-09	8.595670171	4.00722531	6.04685525	5.156626401	9.677941812	9.62862374	9.577849253	9.066361132
ENSG00000100558	PLEK2	5.390969793	2.68E-05	3.204445527	0.018019059	8.59541532	2	3.686303414	2	7.261917051	7.26532099	5.04460184	5.349527629
ENSG00000176155	CCDC57	4.795786217	1.21E-07	3.798705265	3.64E-05	8.594491482	2	5.131603625	3.369317972	8.593855195	8.58388295	7.590975897	7.622152309
ENSG00000123689	G0S2	7.332003286	8.87E-21	1.26006099	0.165797053	8.592064276	2	3.686303414	4.874587708	10.79398911	10.8013584	5.096960836	4.729989799
ENSG00000162616	DNAJB4	3.951509478	3.57E-06	4.636696202	3.34E-08	8.588205679	4.00722531	6.78762695	5.59507254	9.845303961	9.6580856	10.63251333	10.211653
ENSG00000138395	CDK15	3.586725007	0.025019077	4.9999964	0.001038388	8.586721406	3.327711234	2	2	4.965943888	4.60967607	6.189586148	5.953147751
ENSG00000127603	MACF1	3.357913025	3.09E-09	5.221786007	3.88E-21	8.579699032	8.16936288	9.687856435	8.865353883	12.36993409	12.4157698	14.27801527	14.23390067
ENSG00000229937	PRPS1L1	3.973668015	0.040174325	4.600007476	0.014521931	8.573675491	2	2	2	4.139692846	4.4836996	4.45225155	5.150533729
ENSG00000169683	LRRC45	3.979876955	0.002187892	4.590077009	0.000303676	8.569953964	3.327711234	3.076665209	2	5.900763176	5.9725602	6.619606851	6.400759785
ENSG00000114268	PFKFB4	4.99585869	0.004382954	3.565287954	0.048832636	8.561146644	2	6.228547981	2	9.576258382	9.64931004	7.864534187	8.463873348
ENSG00000152154	TMEM178A	4.800546312	0.011612142	3.749098355	0.053196051	8.549644666	2	2	2	5.37770462	4.53541753	4.311629396	3.956354764
ENSG00000105971	CAV2	3.231382538	0.00129249	5.309331137	2.87E-08	8.540713675	4.00722531	5.457120953	7.225742779	9.263797825	9.27353851	11.44714298	11.22309073
ENSG00000172137	CALB2	8.605979182	5.56E-10	-0.06745967	0.97269175	8.538519512	2	2	2	8.633396609	8.6601233	2.34604626	2.277179783
ENSG00000126464	PRR12	4.126970846	0.00015212	4.399831248	4.51E-05	8.526802095	5.095959458	6.311476754	2	9.270565666	9.36213202	9.600443341	9.57619232
ENSG00000174529	TMEM81	3.547727235	0.058360787	4.97872866	0.004973982	8.526455895	2	2	2	4.034562184	3.94787299	5.390603804	4.919632516
ENSG00000183087	GAS6	5.494411386	2.52E-06	3.030213822	0.014425801	8.524625208	7.384433967	7.847167909	3.369317972	12.51021088	12.5811024	10.3504468	9.762514036
ENSG00000125898	FAM110A	4.385896319	0.004693545	4.138081795	0.007684751	8.523978114	2	3.076665209	2	5.514313187	5.49945122	5.385935071	5.171036168
ENSG00000134318	ROCK2	3.788277668	0.001047374	4.735616215	2.57E-05	8.523893883	3.327711234	7.274681896	5.39244166	9.787831283	9.89180734	10.76763523	10.8022669
ENSG00000184923	NUTM2A	6.695347911	4.30E-05	1.826909171	0.331750585	8.522257081	2	2	2	6.825617242	6.72335419	2.62493832	3.183290849
ENSG00000128422	KRT17	2.821542772	0.15015685	5.697386356	0.001258335	8.518929128	2	2	2	3.79808662	3.08597292	5.744329515	5.909074772
ENSG00000102554	KLF5	3.016885928	0.011204778	5.495435499	7.70E-07	8.512321427	2	4.93638933	2	6.5612028	6.24526024	8.86850882	8.797990868
ENSG00000172432	GTPBP2	4.367510099	0.001011359	4.143447163	0.00183886	8.510957262	7.159102577	5.595927442	2	10.26055405	10.3455691	10.27985161	9.848823977
ENSG00000099364	FBXL19	4.244683475	0.013556653	4.265152558	0.012599871	8.509836033	5.866944937	2	2	8.442826028	8.38855922	8.50665722	8.362041249
ENSG00000033867	SLC4A7	3.229295081	9.45E-13	5.279245243	6.93E-33	8.508540324	5.866944937	6.464272742	7.029880454	9.6581704	9.73198422	11.86481281	11.6038049
ENSG00000082641	NFE2L1	4.212162052	8.01E-19	4.293358353	1.01E-19	8.505520406	7.667647223	8.916342327	8.486635645	12.62008676	12.6749758	12.7696937	12.68699458
ENSG00000204634	TBC1D8	3.65919245	0.007262014	4.844398467	0.000218044	8.503590916	2	3.686303414	2	5.639097847	5.58783529	6.577231679	6.870723585
ENSG00000110844	PRPF40B	4.292586615	0.001220428	4.206280166	0.001463478	8.49886678	2	3.686303414	2	6.237202172	6.17362678	6.078235975	6.168983563
ENSG00000178104	PDE4DIP	5.008717999	0.000112427	3.487912106	0.009445149	8.496630105	4.00722531	8.376385542	5.59507254	12.0064372	12.0433189	10.56428727	10.44684159
ENSG00000225614	ZNF469	3.275091567	0.146749658	5.205189508	0.01401918	8.480281074	2	7.431218269	2	9.111653326	9.11366683	10.69589097	11.30943053
ENSG00000273173	SNURF	9.480468985	1.65E-10	-1.002012766	0.614177	8.478456219	2	2	2	9.565291356	9.46239569	2.267001569	2.059860178
ENSG00000111052	LIN7A	2.535726363	0.076658392	5.936887369	3.98E-06	8.472613731	2	4.11352228	2	5.178261742	4.98931816	8.838922815	7.520630205
ENSG00000130402	ACTN4	3.032743133	0.001405029	5.432451405	1.58E-09	8.465194538	9.751047882	7.874700259	7.029880454	11.67556519	11.7141436	13.95211632	14.2218472
ENSG00000197506	SLC28A3	3.497936115	0.063878162	4.953053537	0.005506115	8.450989652	2	2	2	4.105493497	3.7864959	4.977914261	5.306932076
ENSG00000135604	STX11	3.52627378	0.000350662	4.922719529	2.31E-07	8.448993309	2	3.076665209	4.523675391	6.554880361	6.39275508	7.958717528	7.692387724
ENSG00000170442	KRT86	1.689483666	0.4198367	6.757311064	9.43E-05	8.44679473	2	2	2	2.868578001	2.86123014	7.395794919	5.905000563
ENSG00000112715	VEGFA	5.149069036	5.54E-07	3.295228685	0.002061106	8.444297721	6.138360429	8.191702463	4.874587708	12.14652252	12.1562841	10.39091191	10.2048333
ENSG00000198855	FICD	3.820079318	0.000637206	4.621494851	2.29E-05	8.441574169	2	4.710564154	2	7.066878356	6.83591385	7.619097297	7.846917442
ENSG00000147437	GNRH1	4.756040247	0.007796496	3.681366835	0.043775504	8.437407081	2	2	2	5.058234798	4.89348248	4.177580368	4.002845082
ENSG00000196159	FAT4	3.707925812	0.054329477	4.727966788	0.010905332	8.4358926	2	6.535001748	2	8.593855195	8.69038176	9.718175864	9.590215873
ENSG00000174945	AMZ1	1.7756677	0.443101353	6.645197601	0.000596608	8.4208653	2	2	2	2.784612485	3.01487888	7.44824109	5.217771644
ENSG00000124813	RUNX2	6.053255828	1.14E-06	2.367114942	0.08323504	8.42037077	2	2	4.874587708	9.020530266	9.02259731	5.815498119	4.990558504
ENSG00000087903	RFX2	3.498830889	0.017592591	4.914373278	0.000468869	8.413204167	2	3.686303414	2	5.44762523	5.45984938	6.292954465	7.150854407
ENSG00000185591	SP1	3.866497077	0.001939227	4.529560624	0.000207819	8.396057701	6.468621203	6.899080544	2	9.946579093	9.94237362	10.71148464	10.49082905
ENSG00000162852	CNST	4.088282043	0.007712736	4.307741101	0.004514133	8.396023143	2	6.535001748	3.369317972	9.135152476	9.11472974	9.623185015	8.994845077
ENSG00000171940	ZNF217	5.077010019	0.001072632	3.315270843	0.040591855	8.392280862	2	7.189546432	4.058998049	10.78591371	10.797723	9.003023259	9.071823126
ENSG00000121879	PIK3CA	4.767898268	0.000473948	3.624074383	0.009668661	8.391972651	3.327711234	6.140560021	2	9.384581425	9.47817393	8.58359791	7.942440336
ENSG00000067840	PDZD4	0.571869157	0.776233807	7.815927214	2.65E-07	8.387796371	2	3.686303414	2	3.615725078	2.59489879	8.885973502	10.09757713
ENSG00000181019	NQO1	5.77326988	3.25E-28	2.607611387	1.54E-06	8.380881267	6.366709671	6.311476754	6.629488387	12.14822967	12.1383651	8.402448739	9.403205664
ENSG00000133794	ARNTL	5.263476455	2.25E-09	3.116434945	0.000679074	8.3799114	3.327711234	4.442710129	2	8.418656444	8.44034872	6.514451546	6.13807518
ENSG00000177453	NIM1K	4.974563799	0.013104298	3.400061511	0.100917556	8.374625309	2	2	2	5.128039203	5.21224859	3.235948948	4.329022995
ENSG00000087095	NLK	4.368440605	1.67E-06	4.001111538	1.25E-05	8.369552142	4.00722531	5.303523747	2	8.486665614	8.4705529	8.367944224	7.81035357
ENSG00000114302	PRKAR2A	3.352450204	0.052698164	5.015726417	0.002179419	8.368176621	2	7.051611315	3.369317972	8.948803189	8.81332035	10.45569736	10.61496321
ENSG00000257365	FNTB	4.798983141	0.001854643	3.567715117	0.024667973	8.366698258	5.532353208	2	2	8.522747711	8.66157866	7.336538937	7.427130611
ENSG00000143515	ATP8B2	4.027789445	1.92E-09	4.331039304	7.96E-11	8.358828749	7.384433967	5.946638857	5.930865648	10.53200702	10.5760362	10.43041464	11.18537541
ENSG00000138641	HERC3	4.582030096	4.78E-06	3.774959631	0.000201497	8.356989728	3.327711234	5.303523747	2	8.535647908	8.4936155	7.564363052	7.859604233
ENSG00000122877	EGR2	5.09847937	0.016034926	3.25498839	0.139084171	8.353467761	2	2	3.369317972	6.197166296	6.14061114	5.284230775	2.782576693
ENSG00000125618	PAX8	4.153025464	0.014151141	4.19773519	0.012393734	8.350760654	2	2	3.369317972	5.144975053	5.43283104	4.799832052	5.727202496
ENSG00000186088	GSAP	6.576324517	8.91E-06	1.768990809	0.295440666	8.345315326	2	2	4.058998049	8.567402595	8.46222608	4.588526421	3.287118176
ENSG00000151690	MFSD6	3.37350894	1.85E-05	4.96301614	7.26E-11	8.33652508	2	4.93638933	4.058998049	7.273514749	7.19470132	8.871434321	8.72128783
ENSG00000213949	ITGA1	4.647910424	0.000421918	3.687424558	0.006183393	8.335334982	2	7.002537973	5.930865648	10.60458486	10.5632459	10.03197853	9.062251023
ENSG00000185262	UBALD2	4.813755985	5.20E-08	3.519099315	9.94E-05	8.3328553	4.467311984	5.595927442	2	9.333786218	9.24178305	8.153962636	7.843728236
ENSG00000130653	PNPLA7	3.859404043	0.039042892	4.466767144	0.013950213	8.326171187	2	2	2	4.237680128	4.22485516	4.799832052	4.654341953
ENSG00000112378	PERP	3.547917517	6.03E-16	4.777411636	1.88E-28	8.325329153	6.816637977	6.844429755	6.534109959	10.24934078	10.2345667	11.09309185	11.76653247
ENSG00000043143	JADE2	5.22490407	1.36E-15	3.095100706	4.17E-06	8.320004776	3.327711234	5.72254323	5.59507254	10.27841188	10.3373973	8.319187939	8.056111835
ENSG00000121577	POPDC2	2.548700716	0.176945672	5.768803074	0.00056294	8.31750379	2	2	2	3.462030052	3.15372745	6.060766632	5.708623051
ENSG00000163395	IGFN1	2.49683765	0.281095622	5.817045798	0.004719809	8.313883449	2	2	2	3.289987342	3.28037825	4.487545924	6.649933445
ENSG00000164463	CREBRF	5.131996233	0.000203981	3.179888796	0.028543748	8.311885029	2	6.228547981	3.369317972	9.918537335	9.83876104	8.224302521	7.597118029
ENSG00000162630	B3GALT2	1.500815324	0.50971472	6.811047416	0.000247567	8.31186274	2	2	2	3.09461492	2.38539787	7.375919751	6.141542327
ENSG00000244405	ETV5	4.054207174	5.65E-16	4.253764943	1.07E-17	8.307972117	4.467311984	5.303523747	5.772716771	9.142549916	9.21427981	9.354710897	9.399228411
ENSG00000166924	NYAP1	3.692786932	0.023196476	4.610800796	0.00331552	8.303587727	2	2	3.369317972	4.783303513	4.98931816	5.9289675	5.459179378
ENSG00000183715	OPCML	5.267094572	0.037231944	3.028416182	0.253721513	8.295510753	2	2	2	5.474671438	5.39133044	4.291799915	2.326754077
ENSG00000130489	SCO2	5.317096923	1.93E-14	2.975742509	3.49E-05	8.292839432	5.095959458	4.442710129	2	9.363030664	9.34230452	7.11465301	6.975457902
ENSG00000000938	FGR	3.275430812	0.20396079	5.015586062	0.037934228	8.291016874	2	2	2	3.921164147	3.65239003	5.99483933	3.33635788

ENSG00000106100	NOD1	3.625809444	0.007885856	4.660603955	0.000397713	8.286413399	3.327711234	3.076665209	2	5.614989456	5.60003091	6.845542321	6.254725615
ENSG00000227124	ZNF717	4.149801872	0.00360402	4.135500475	0.003541393	8.285302347	2	3.686303414	2	6.104969671	6.00960265	6.346800618	5.661099865
ENSG00000101608	MYL12A	3.660120009	7.29E-48	4.625061695	6.35E-77	8.285181703	8.981092835	8.607278467	8.742726175	12.44525263	12.4115594	13.35833349	13.42672912
ENSG00000152242	C18orf25	3.828697786	0.007024937	4.4548156	0.001326915	8.283513385	3.327711234	6.228547981	2	8.651287275	8.50824913	9.37340502	9.00820079
ENSG00000179820	MYADM	3.843302968	0.005370243	4.439856422	0.001021668	8.283159389	3.327711234	7.954278496	8.721235202	11.61113422	11.675768	11.99089185	12.45192034
ENSG00000101236	RNF24	4.830482721	0.001200331	3.450935345	0.025360922	8.281418066	3.327711234	7.467835385	3.369317972	10.81365108	10.797723	9.318112363	9.536251143
ENSG00000221994	ZNF630	3.835074198	0.020143473	4.437392512	0.005795548	8.27246671	3.327711234	2	2	4.825935136	5.18011485	5.80853718	5.243813244
ENSG00000187556	NANOS3	3.732211093	0.056266195	4.539508237	0.016135868	8.27171933	2	2	2	3.881293667	4.3456612	4.580376151	4.975095763
ENSG00000141682	PMAIP1	6.663398997	6.13E-05	1.604927199	0.402280751	8.268326196	2	3.076665209	6.958202216	12.05596597	11.9962262	7.470462265	6.33565646
ENSG00000169826	CSGALNACT2	4.673093807	0.0019028	3.594939886	0.020035793	8.268033693	2	7.538394143	5.156626401	10.8531554	10.8394695	9.7848625	9.758285822
ENSG00000048828	FAM120A	3.75647436	2.99E-06	4.509541666	1.19E-08	8.266016026	5.330594134	7.819099893	6.629488387	10.68132448	10.6309298	11.25865892	11.54324631
ENSG00000176697	BDNF	3.916589394	4.54E-05	4.334788548	4.83E-06	8.251377941	2	5.595927442	5.930865648	9.066811112	8.96128525	9.547075488	9.303427654
ENSG00000179715	PCED1B	3.424951881	0.013666189	4.818276585	0.000275564	8.243228466	2	3.686303414	2	5.289065324	5.49945122	6.835304498	6.559516442
ENSG00000166825	ANPEP	5.966012516	0.000116017	2.275150353	0.181490912	8.241162869	8.468948322	7.315436877	2	13.32873996	13.3982649	9.073675485	10.10492182
ENSG00000185129	PURA	3.516793028	0.021758694	4.721893817	0.001338547	8.238686845	4.00722531	6.899080544	2	8.980991923	8.95536429	10.36549517	9.936261795
ENSG00000114127	XRN1	5.015682045	4.18E-05	3.216914373	0.011903882	8.232596418	4.00722531	7.35507212	4.058998049	11.00087894	10.9761604	9.251341112	9.1401427
ENSG00000243716	NPIPB5	7.290568365	1.62E-31	0.936030687	0.203235525	8.226599052	5.330594134	3.686303414	4.058998049	11.5128316	11.4889959	5.227950776	5.367404784
ENSG00000047188	YTHDC2	4.046718313	9.32E-07	4.174603332	3.54E-07	8.221321646	2	5.131603625	4.058998049	8.020170999	8.06258142	8.275683409	8.050589918
ENSG00000164091	WDR82	3.779453233	2.45E-06	4.441540451	1.89E-08	8.220993684	5.095959458	7.315436877	5.59507254	10.06920484	10.0804323	10.74103322	10.72882541
ENSG00000005469	CROT	4.630939842	1.27E-07	3.587671061	5.74E-05	8.218610904	2	4.442710129	3.369317972	7.77569021	7.8441483	6.875826972	6.707209278
ENSG00000171533	MAP6	2.824771867	0.060296866	5.391002286	9.86E-05	8.215774154	2	5.457120953	2	6.699459234	6.73446058	9.049523349	9.402844548
ENSG00000114859	CLCN2	5.090023711	0.003959538	3.124667276	0.09228972	8.214690988	2	2	2	5.22679461	5.3193943	3.838019203	3.518055793
ENSG00000075643	MOCOS	4.727931341	0.007409183	3.4827898	0.054660707	8.210721141	2	2	3.369317972	5.870535235	5.77157844	5.227950776	3.8919413
ENSG00000115808	STRN	4.09068888	0.027679774	4.11969899	0.025482471	8.21038787	2	6.389896633	2	8.935480545	8.81462916	9.005688562	8.796891925
ENSG00000130997	POLN	4.927933462	0.005269294	3.280538323	0.074166356	8.208471784	2	2	2	5.144975053	5.11361705	3.838019203	3.753786374
ENSG00000106003	LFNG	3.95677742	0.058415094	4.243613723	0.037791254	8.200391143	2	2	2	4.387512679	4.22485516	3.904814898	4.982847849
ENSG00000187372	PCDHB13	4.012586919	0.036823894	4.184454704	0.026879191	8.197041623	2	2	2	4.523237872	4.16043394	4.360042493	4.614976609
ENSG00000188647	PTAR1	3.800557613	0.030593626	4.394853803	0.01045232	8.195411415	2	7.189546432	3.369317972	9.517321994	9.39837151	10.28315082	9.773170377
ENSG00000172061	LRRC15	6.3479258780	3.71E-05	1.846733131	0.293334688	8.19465901	2	2	2	6.490083648	6.39275508	2.987421008	2.885414003
ENSG00000182584	ACTL10	3.597145927	0.059410678	4.59431599	0.011923495	8.191461916	2	2	2	4.139692846	3.90918929	4.778616857	4.895193906
ENSG00000116711	PLA2G4A	7.173589768	1.28E-11	1.016536039	0.427095076	8.190125807	2	4.442710129	2	9.926517581	10.0153322	4.470006665	3.789585079
ENSG00000102466	FGF14	2.151478169	0.27946754	6.03778076	0.000434519	8.189258929	2	2	2	3.349643765	2.77779907	6.010089548	6.27380897
ENSG00000108599	AKAP10	4.292120726	1.89E-10	3.890877478	8.48E-09	8.182998204	3.327711234	5.131603625	5.59507254	9.060130342	9.07923325	8.896269648	8.405948829
ENSG00000185404	SP140L	4.267620293	5.90E-10	3.911205079	1.45E-08	8.178825372	5.095959458	4.11352228	3.369317972	8.293234263	8.36015869	7.812424466	8.122532579
ENSG00000186150	UBL4B	1.390400159	0.494744255	6.786991611	3.36E-05	8.17739177	2	2	2	2.784612485	2.68924543	6.262714117	7.287011591
ENSG00000136925	TSTD2	4.523548691	2.57E-10	3.63744358	4.95E-07	8.160992271	4.467311984	4.710564154	2	8.369067528	8.3920703	7.525623979	7.492396644
ENSG00000173442	EHBP1L1	3.188683664	0.025590506	4.97181313	0.000232764	8.160496794	2	7.189546432	7.225742779	9.764733396	9.79897887	11.27540049	11.79831821
ENSG00000275338		7.379553318	6.79E-08	0.778200119	0.649722124	8.157753437	2	3.686303414	2	9.178978633	9.23104065	3.679182183	2.851942824
ENSG00000142910	TINAGL1	-0.844394841	0.49749462	9.001312748	3.58E-22	8.156917907	3.327711234	3.076665209	4.058998049	2.868578001	3.15372745	11.47790684	12.20053827
ENSG00000167522	ANKRD11	3.917916742	0.006512679	4.228129007	0.002900244	8.146045749	3.327711234	8.639735838	6.322058544	11.22174696	11.260058	11.65071957	11.44336891
ENSG00000138760	SCARB2	4.403237424	2.65E-05	3.738882362	0.000420382	8.142119786	9.128497779	9.125613817	5.59507254	12.99606985	12.9927763	12.31341061	12.34733473
ENSG00000225855	RUSC1-AS1	2.899136394	0.127934297	5.242075933	0.002585025	8.141212327	2	2	2	3.566290607	3.50452879	5.217477917	5.581648407
ENSG00000164124	TMEM144	4.483245877	0.018992676	3.657147793	0.058715617	8.140393671	2	2	2	4.647285135	4.83276841	4.311629396	3.789585079
ENSG00000139318	DUSP6	6.381625656	3.07E-08	1.758349133	0.17846657	8.13997479	4.467311984	2	3.369317972	9.333786218	9.30274583	5.399896186	4.105828626
ENSG00000125650	PSPN	1.595973537	0.397020618	6.541291901	2.24E-05	8.137265438	2	2	2	2.784612485	2.86123014	6.726875343	6.520103598
ENSG00000090539	CHRD	3.328624741	0.044397422	4.807086522	0.002132231	8.135711262	2	3.076665209	2	4.739373666	4.40247105	6.075339068	5.689801209
ENSG00000213145	CRIP1	7.440982399	2.32E-07	0.693761795	0.708536423	8.134744195	2	2	2	7.501020868	7.49817903	2.526562374	2.466008509
ENSG00000079387	SENP1	4.127691222	6.33E-10	4.006752149	1.82E-09	8.13444337	4.00722531	4.11352228	4.523675391	8.087422161	7.94798467	7.962642137	7.835189056
ENSG00000138738	PRDM5	4.041209899	0.002888636	4.089965702	0.002381928	8.1311756	2	3.686303414	2	6.034011955	5.89549381	5.966975997	6.055940621
ENSG00000152804	HHEX	5.217223641	9.33E-05	2.907929202	0.040183283	8.125152842	4.00722531	2	2	7.148924055	7.14977896	5.233158834	4.702084619
ENSG00000171132	PRKCE	4.219219065	0.000106595	3.902050377	0.000349813	8.121269443	2	4.442710129	2	6.941624891	7.16627666	6.788313392	6.714211481
ENSG00000271425	NBPF10	8.416205419	6.39E-75	-0.301634614	0.61592295	8.114570804	5.095959458	5.457120953	5.59507254	13.61227351	13.7312934	4.977914261	5.269393099
ENSG00000168661	ZNF30	3.866284949	0.022954373	4.24160811	0.011002269	8.107893059	2	3.076665209	2	4.887609717	5.18011485	5.725974948	4.911532223
ENSG00000183840	GPR39	6.005900179	0.000406094	2.098758643	0.269325152	8.104658821	2	2	2	5.949776857	6.26836889	2.803598391	3.287118176
ENSG00000068724	TTC7A	3.761417506	0.051722704	4.34300901	0.021313338	8.104426516	2	6.535001748	2	8.705093576	8.68609779	9.163026016	9.374395329
ENSG00000085433	WDR47	4.238951812	0.002112811	3.860631288	0.005396684	8.099583101	2	6.140560021	3.369317972	8.939126212	8.88234995	8.739951687	8.299011699
ENSG00000138792	ENPEP	3.710750922	0.078653566	4.378679339	0.031664471	8.089430261	2	2	2	3.95996233	4.2560182	5.079718116	4.047883918
ENSG00000276966	HIST1H4E	5.490983653	0.004215891	2.588222596	0.21149879	8.079206249	2	2	2	5.742861563	5.52526112	2.831336763	3.717076715
ENSG00000146950	SHROOM2	1.109721726	0.509773777	6.967623362	2.41E-07	8.077345088	3.327711234	3.076665209	2	3.754611435	3.33976443	8.04860508	9.342363956
ENSG00000162729	IGSF8	4.529374396	0.003438359	3.544371864	0.025418276	8.073746261	4.00722531	7.099070191	2	10.10372352	10.2094139	9.047307518	9.296834866
ENSG00000151176	PLBD2	3.263224998	4.32E-10	4.809095612	5.92E-21	8.07232061	7.27616306	7.60566235	6.203069917	10.34771157	10.4188272	11.74812711	12.08565654
ENSG00000154114	TBCEL	4.04947814	0.003951987	4.021205091	0.004075293	8.070683231	3.327711234	6.228547981	2	8.826850269	8.77349359	8.817412208	8.725914443
ENSG00000161013	MGAT4B	4.204949726	0.003504466	3.856702823	0.007768297	8.061652549	6.563806279	7.761270542	2	10.83924025	10.9176784	10.54557014	10.51742604
ENSG00000133321	RARRES3	5.27517315	1.02E-11	2.785326799	0.000607542	8.060499949	4.00722531	4.710564154	2	8.920805049	8.92538942	6.310306306	6.645056213
ENSG00000281429		4.218223822	0.001966935	3.841560988	0.005092678	8.05978481	3.327711234	6.951736442	3.369317972	9.706030497	9.73613428	9.355905054	9.337081571
ENSG00000135272	MDFIC	5.199143103	4.70E-09	2.858504515	0.002185545	8.057647618	3.327711234	6.228547981	4.523675391	10.30549718	10.2232775	7.905886539	7.979721237
ENSG00000135090	TAOK3	4.33986567	0.029985966	3.713869079	0.065273313	8.053734749	2	6.844429755	2	9.590237441	9.57170671	9.011762258	8.904372226
ENSG00000102543	CDADC1	4.675091177	3.61E-05	3.375267487	0.003775209	8.050358664	2	4.11352228	2	7.127781374	7.11195456	5.982522014	5.768151538
ENSG00000130559	CAMSAP1	3.162350605	0.124169408	4.88786415	0.011612184	8.050214755	2	6.78762695	2	8.468280417	8.23475766	10.12998797	10.00615667
ENSG00000177076	ACER2	3.362057223	0.097955385	4.686111842	0.014701036	8.048169064	2	2	2	3.881293667	3.82856379	5.258920789	4.468068808
ENSG00000100307	CBX7	3.360684086	1.71E-05	4.680448885	6.81E-10	8.041132971	2	4.710564154	4.523675391	7.148924055	7.32531744	8.692059118	8.362041249
ENSG00000136158	SPRY2	5.805158017	0.001165874	2.235293719	0.256352368	8.040451735	2	6.464272742	2	10.64569773	10.6701389	7.344982741	6.868633039
ENSG00000171916	LGALS9C	5.410762981	0.002162092	2.624232049	0.16716103	8.034995031	2	2	2	5.602782483	5.52526112	3.10571689	3.580696614
ENSG00000140323	DISP2	2.775635056	0.150440253	5.258632734	0.002666452	8.03426779	2	2	2	3.289987342	3.60476711	5.47655442	5.373314945
ENSG00000275395	FCGBP	4.654042263	0.01717456	3.379777154	0.091924093	8.033819418	2	2	2	4.694064037	5.06165168	3.500668998	4.161535617
ENSG00000166439	RNF169	4.269148573	0.017100337	3.762838571	0.036779004	8.031987144	2	6.228547981	2	8.935480545	8.84568954	8.459109781	8.317521841
ENSG00000143226	FCGR2A	2.33424278	0.299070637	5.690178799	0.004075293	8.02442158	2	2	2	3.289987342	3.08597292	6.442083733	4.711446447
ENSG00000173166	RAPH1	4.522589275	2.40E-07	3.497510596	8.76E-05	8.020099871	2	4.93638933	5.772716771	9.263797825	9.15867957	8.096541671	8.291228494
ENSG00000184860	SDR42E1	4.521947976	0.014871185	3.498028198	0.064654885	8.019976174	2	2	2	4.71689672	4.83276841	4.029788235	3.875378318
ENSG00000043355	ZIC2	-1.313532109	0.596370107	9.332501553	1.74E-08	8.018969444	2	2	2	2	2.2680111	8.702892291	9.820984284
ENSG00000106617	PRKAG2	3.242943566	3.01E-05	4.771340448	2.08E-10	8.014284014	4.467311984	4.442710129	2	7.066878356	6.80989959	8.462439703	8.420292672
ENSG00000185187	SIGIRR	3.370776409	8.93E-05	4.642127463	2.46E-08	8.012903872	4.00722531	4.11352228	2	6.598566398	6.60731796	7.857806412	7.820122783
ENSG00000107159	CA9	4.620082934	0.011889226	3.39184837	0.072085279	8.011931304	2	2	2	4.846787657	4.87352701	3.810410249	3.940519005
ENSG00000120708	TGFBI	4.981548779	5.41E-06	3.027809542	0.008414663	8.009358322	11.88152073	9.680074956	9.735974718	15.79016427	15.8181284	14.62132509	12.08486888
ENSG00000184307	ZDHHC23	5.273937827	0.002252871	2.735021446	0.138306822	8.008959273	2	2	2	5.363304456	5.51241389	3.517841605	3.33635788
ENSG00000110092	CCND1	4.379159459	4.82E-26	3.621990402	2.62E-18	8.001149861	9.90440318	10.22575081	9.990713521	14.40261977	14.4407672	13.14527188	14.04602869
ENSG00000243710	CFAP57	3.860722499	0.072220677	4.139909037	0.048259223	8.000631536	2	2	2	4.415698455	4.02226117	3.824280769	4.895193906
ENSG00000164283	ESM1	7.18619485	1.55E-05	0.812086443	0.692464461	7.998281293	5.866944937	2	2	11.39655036	11.2888305	5.642319755	4.365060851
ENSG00000150991	UBC	5.107471661	2.21E-21	2.886053696	1.59E-07	7.993525357	11.80808675	10.74900803	10.49365885	16.26186415	16.2178222	13.99870688	14.03874037
ENSG00000146757	ZNF92	5.500236622	1.26E-06	2.492826421	0.042401898	7.993063043	2	4.442710129	2	8.406418094	8.185025	5.72966459	5.013446246
ENSG00000241839	PLEKHO2	3.495947156	0.044742745	4.494682434	0.007374689	7.990629591	2	7.099070191	3.369317972	9.029678018	9.10940731	9.98740646	10.13567188
ENSG00000138675	FGF5	4.630532146	1.08E-10	3.355808581	4.48E-06	7.986340727	5.866944937	4.11352228	6.203069917	10.1390214	10.0787993	8.512558892	9.109931338
ENSG00000085831	TTC39A	3.418422052	0.081057036	4.567380847	0.014257344	7.985802899	2	2	2	4.034562184	3.74316441	4.894909044	4.729989799
ENSG00000183671	GPR1	2.7904203	0.054717846	5.189418883	0.00010205	7.979839182	2	2	4.058998049	4.694064037	5.07918227	6.928144991	7.311843322
ENSG00000259305	ZHX1-C8orf76	6.815631771	1.62E-06	1.163404679	0.495779443	7.97903645	2	3.076665209	2	7.683067677	7.96222667	2.937246078	3.209956562
ENSG00000101210	EEF1A2	2.645389014	0.214129738	5.33334526	0.005583444	7.978734273	2	2	2	3.566290607	3.15372745	5.951260634	4.819291477
ENSG00000181788	SIAH2	3.288270443	5.54E-05	4.688505409	2.95E-09	7.976775852	4.815609471	5.946638857	3.369317972	8.219202381	8.28094035	9.903328854	9.314605104
ENSG00000074370	ATP2A3	3.21337828	0.11657228	4.762383412	0.013281894	7.975761692	2	2	2	3.79808662	3.69849104	5.338397457	4.511590831
ENSG00000162825	NBPF20	9.128144727	1.30E-70	-1.168058261	0.051202725	7.960086465	5.095959458	4.710564154	6.073378139	14.28879814	14.4042595	4.406883583	4.316807515
ENSG00000157193	LRP8	4.317119515	0.004529009	3.626925772	0.019007831	7.944045287	3.327711234	2	6.203069917	8.936696791	8.96364685	7.781653072	8.629822748
ENSG00000172986	GXYLT2	2.961273953	0.044744796	4.982083986	0.000281972	7.94335794	2	3.686303414	2	5.110902178	4.83276841	7.121690475	6.525421271
ENSG00000196914	ARHGEF12	3.936133398	2.52E-13	4.007139101	7.09E-14	7.943272499	6.563806279	8.029695761	7.502634626	11.40360438	11.401556	11.30754056	11.62230214
ENSG00000184363	PKP3	3.147521088	0.109887072	4.794448059	0.009113066	7.941969147	2	2	2	3.515101962	3.86943969	5.002522427	5.020995508
ENSG00000126945	HNRNPH2	3.85506626	3.52E-08	4.083886781	4.07E-09	7.938953041	6.563806279	4.93638933	4.874587708	9.363935069	9.46239569	9.40316733	9.846170307
ENSG00000125968	ID1	6.103521504	5.74E-06	1.834589651	0.227161376	7.938111155	5.330594134	2	2	9.660380652	9.64857633	5.390603804	5.621921006
ENSG00000163291	PAQR3	3.990789124	0.004333061	3.945505717	0.004644445	7.936294841	2	5.131603625	2	7.590284224	7.44845993	7.815899769	7.032593922
ENSG00000082438	COBLL1	2.153886206	0.315977132	5.771841878	0.002483378	7.925728084	2	6.844429755	2	7.337530098	7.5014335	11.20060466	10.786851

ENSG00000187118	CMC1	4.502164297	0.001019301	3.421371872	0.015113682	7.923536169	2	3.686303414	2	6.483440992	6.30607931	5.565950232	5.191251321
ENSG00000189195	BTBD8	2.445189841	0.231434543	5.470671262	0.002657411	7.915861103	2	2	2	3.349643765	3.15372745	6.007052374	5.08721802
ENSG00000136379	ABHD17C	4.850537942	3.29E-10	3.055482082	0.000127227	7.906020024	4.00722531	5.303523747	3.369317972	9.137269904	9.10727282	6.983275763	7.646759583
ENSG00000153815	CMIP	4.293605271	0.005775587	3.607879551	0.022516836	7.901484822	6.468621203	3.076665209	2	9.175891964	9.18219179	8.365574792	8.621777169
ENSG00000079819	EPB41L2	3.229695597	0.031289994	4.669673712	0.001065455	7.899369309	2	7.431218269	7.285536013	9.965769838	9.99180603	10.83683561	11.82693096
ENSG00000129566	TEP1	3.877560457	1.62E-13	4.015548432	1.49E-14	7.893108889	5.095959458	5.131603625	6.203069917	9.277301907	9.32403997	9.288400199	9.573627872
ENSG00000273820	USP27X	3.673514098	0.009982485	4.219154605	0.002441241	7.892668703	2	3.686303414	2	5.363304456	5.83486613	6.18690462	6.06327132
ENSG00000152133	GPATCH11	3.134102391	0.004533018	4.756763845	5.97E-06	7.890866236	3.327711234	4.11352228	2	6.01571348	6.08095082	7.975129619	7.123062801
ENSG00000140199	SLC12A6	3.60423027	6.87E-07	4.285237533	2.08E-09	7.889467803	4.467311984	5.838937808	6.718950348	9.476928206	9.42432312	10.39846491	9.796725763
ENSG00000150636	CCDC102B	4.678975799	0.013552546	3.210163922	0.101122291	7.889139721	2	2	2	5.040242897	4.76938658	3.943456545	3.518055793
ENSG00000170417	TMEM182	3.150314916	0.125476823	4.737348422	0.013968105	7.887663339	2	2	2	3.881293667	3.50452879	5.253805068	4.594883674
ENSG00000090554	FLT3LG	6.628415357	1.35E-05	1.257592209	0.490286193	7.886007566	2	2	2	6.604700657	6.80989959	2.62493832	2.746601469
ENSG00000163823	CCR1	2.444086818	0.295066592	5.441695623	0.009007577	7.88578244	2	2	2	3.227757282	3.28037825	6.252491482	4.341135913
ENSG00000100403	ZC3H7B	3.419653484	0.098091497	4.451999595	0.024694885	7.871653079	8.892187472	2	4.874587708	10.7662095	10.8158091	11.45091976	12.11670545
ENSG00000185955	C7orf61	3.596105637	0.08781545	4.273768727	0.03515611	7.869874364	2	2	2	3.997744386	4.05806467	4.04171018	4.951586397
ENSG00000102032	RENBP	4.093671598	0.013079091	3.769493009	0.022726528	7.863164607	2	3.076665209	2	5.194621444	5.28958453	5.212212839	4.644600858
ENSG00000113580	NR3C1	4.844288223	8.66E-05	3.017184654	0.019926216	7.861472876	8.16936288	7.73146271	3.369317972	12.22651752	12.2242146	10.49511466	10.30098708
ENSG00000115414	FN1	2.550145312	9.94E-06	5.308509995	6.92E-22	7.858655307	13.80200664	15.22492386	14.22230326	17.07667487	17.1124152	19.85784669	19.84810262
ENSG00000225830	ERCC6	1.731686895	0.184583015	6.117307888	6.43E-08	7.848994783	6.816637977	7.73146271	3.369317972	8.50645854	8.51792306	12.47218794	13.20189837
ENSG00000165118	C9orf64	4.296723709	9.87E-06	3.547282791	0.000316727	7.8440065	2	3.076665209	4.874587708	7.474468681	7.62295033	6.952030889	6.678855578
ENSG00000125746	EML2	3.650926775	6.01E-05	4.192280808	2.81E-06	7.843207583	5.70932728	4.442710129	2	8.14116509	8.1187584	8.59173005	8.734549554
ENSG00000140545	MFGE8	4.315491043	4.84E-06	3.524109664	0.000234908	7.839600707	10.75779627	9.079034257	7.777116083	13.98613001	14.0263193	13.23375446	13.19646542
ENSG00000101194	SLC17A9	4.434096283	0.016051741	3.40499464	0.070302637	7.839090923	2	6.389896633	2	9.246247604	9.18725282	8.248936733	8.145162126
ENSG00000115540	MOB4	4.174568437	1.02E-13	3.651810213	8.66E-11	7.82637865	4.467311984	4.710564154	5.59507254	8.983347946	8.96128525	8.586652837	8.309067496
ENSG00000122515	ZMIZ2	3.701710124	0.010778206	4.124631016	0.00381062	7.82634114	2	7.315436877	5.39244166	9.733582734	9.73889436	10.05219399	10.2555122
ENSG00000225932	CTAGE4	6.255592151	8.98E-05	1.569969387	0.397192742	7.825561538	2	2	2	6.372775472	6.33555364	2.937246078	2.671842496
ENSG00000181625	SLX1B	8.95786565	3.72E-10	-1.14221716	0.548817763	7.81564849	2	2	2	8.977450658	9.01350799	2.094598369	2.225841198
ENSG00000126803	HSPA2	1.643847027	0.309143132	6.170818812	7.73E-06	7.814665839	2	2	5.39244166	5.406080789	5.34860058	9.803800654	9.725739902
ENSG00000064763	FAR2	3.4828275	0.000167216	4.330632229	1.60E-06	7.813459729	4.815609471	3.686303414	2	7.110641269	6.84618961	7.513903457	8.058864887
ENSG00000178222	RNF212	3.815033538	0.018412016	3.994352657	0.012343956	7.809386194	2	3.076665209	2	4.984880435	5.00774437	5.073924441	5.230851202
ENSG00000263155	MYZAP	5.631459281	0.002669901	2.175042336	0.293035077	7.806501617	2	2	2	5.686138681	5.84514933	2.68698273	3.430065404
ENSG00000160877	NACC1	3.547474472	0.000344238	4.258343239	1.15E-05	7.805817711	5.095959458	8.053983481	6.322058544	10.49161431	10.5189709	10.79210238	11.54111489
ENSG00000100345	MYH9	2.629286489	0.008881252	5.174472469	3.36E-08	7.803758958	8.285626337	10.87022386	11.4884794	13.34084865	13.3502258	15.81354649	15.96311156
ENSG00000128512	DOCK4	5.416093234	2.66E-08	2.386423175	0.023658021	7.802516409	2	4.11352228	3.369317972	8.192977693	8.34574597	5.614651724	5.072762365
ENSG00000134686	PHC2	3.946770212	0.109430874	3.852540258	0.112945603	7.79931047	2	8.395519306	2	10.69648168	10.8013584	10.56221954	10.74315987
ENSG00000107816	LZTS2	3.514772876	0.00938604	4.281818359	0.001131917	7.796591235	2	6.464272742	7.225742779	9.774969254	9.78702715	10.56854263	10.52175615
ENSG00000196932	TMEM26	4.571578677	0.014868925	3.224631572	0.095146591	7.796210249	2	2	2	4.739373666	4.89348248	3.648325314	3.858622973
ENSG00000160218	TRAPPC10	4.299315599	0.000245132	3.495703571	0.003420748	7.79501917	2	5.131603625	6.534109959	9.640365415	9.58633944	8.736288294	8.890530144
ENSG00000033327	GAB2	2.904284387	0.127194913	4.890558998	0.005830103	7.794843384	2	6.311476754	2	7.626911583	7.62891439	9.653364877	9.528327138
ENSG00000151778	SERP2	3.467767398	0.043810684	4.318274834	0.00911236	7.786042232	2	3.076665209	2	4.470469939	4.89348248	5.102662916	5.722580018
ENSG00000058063	ATP11B	4.020211732	0.0142187	3.764431999	0.022137903	7.784643731	4.467311984	7.572420273	2	10.12521908	10.1422001	9.896164504	9.861494087
ENSG00000088387	DOCK9	3.443413842	1.71E-05	4.340351749	2.84E-08	7.783765591	4.00722531	4.93638933	2	7.333842102	7.25764291	8.111466145	8.237969002
ENSG00000038219	BOD1L1	3.847867957	0.015565291	3.929123728	0.012788451	7.776991685	3.327711234	8.077869079	4.058998049	10.41993524	10.4507437	10.61778047	10.4074232
ENSG00000048405	ZNF800	3.876761441	0.024179818	3.891569965	0.022869703	7.768331405	2	5.946638857	2	8.221200057	8.21902896	8.445712697	7.987448576
ENSG00000164574	GALNT10	2.575052524	0.000171546	5.17721411	1.70E-15	7.752266634	6.96351253	8.234834549	6.43197674	9.95740528	10.0010327	12.5158451	12.63521027
ENSG00000158373	HIST1H2BD	5.751358837	1.25E-10	1.994733228	0.04303466	7.746092064	3.327711234	4.710564154	2	9.155143657	9.14937381	5.201624686	5.777095613
ENSG00000107551	RASSF4	2.075188279	0.093856973	5.668794227	2.26E-07	7.743982506	2	4.710564154	2	5.48800675	5.11361705	8.984998983	8.509534124
ENSG00000221823	PPP3R1	4.133460082	8.75E-09	3.609888444	5.96E-07	7.743348526	4.00722531	6.464272742	6.203069917	10.00514223	9.89490419	9.4014335	9.458113344
ENSG00000169933	FRMPD4	4.886213837	0.002602476	2.854294738	0.095264998	7.740508575	2	4.442710129	2	7.501020868	7.74878077	4.220168345	6.397863414
ENSG00000198774	RASSF9	2.150847631	0.28400482	5.588922417	0.001382281	7.739770048	2	2	2	3.289987342	2.86123014	5.766051132	5.689801209
ENSG00000139209	SLC38A4	3.418722624	0.094962877	4.320772398	0.027228112	7.739495022	2	2	2	4.070463721	3.69849104	4.785723309	4.400220394
ENSG00000160584	SIK3	4.086010875	0.028911985	3.651528112	0.0515772	7.737538987	2	6.389896633	2	8.822909147	8.91810182	8.28637003	8.580862167
ENSG00000162390	ACOT11	4.098022861	0.049030905	3.638747112	0.080103167	7.736769972	2	2	2	4.299492648	4.53541753	3.616794016	4.400220394
ENSG00000075391	RASAL2	3.75217178	0.000927691	3.981319419	0.00038106	7.7334912	7.435667355	4.93638933	4.058998049	9.879858319	9.85666073	10.1379928	10.05385647
ENSG00000150551	LYPD1	6.405009125	0.004752131	1.324548647	0.618981618	7.729557773	2	2	2	6.710878132	6.24526024	3.12825509	2.117335275
ENSG00000196189	SEMA4A	2.975077761	0.133011196	4.752426353	0.009778296	7.727504115	2	2	2	3.615725078	3.55551834	5.079718116	4.861951484
ENSG00000165475	CRYL1	3.34802026	0.013546981	4.378989221	0.00080388	7.727009481	2	5.131603625	2	6.872128674	6.93551934	8.044167181	7.762743025
ENSG00000138448	ITGAV	3.51610778	0.00358975	4.207034823	0.000357883	7.723142603	4.467311984	8.747909548	7.285536013	11.17355701	11.145897	12.08377451	11.57027009
ENSG00000040199	PHLPP2	3.79165093	1.71E-08	3.930278737	4.06E-09	7.721929666	4.467311984	5.457120953	3.369317972	8.372666678	8.27524772	8.391426944	8.528858263
ENSG00000080200	CRYBG3	4.1573085	1.48E-13	3.562705974	2.90E-10	7.720014474	5.70932728	5.303523747	4.058998049	9.198375657	9.18725282	8.749069679	8.440841157
ENSG00000130363	RSPH3	3.795298184	5.55E-07	3.923109672	1.91E-07	7.718407856	4.815609471	5.303523747	2	8.241026137	8.17690623	8.294489071	8.375354401
ENSG00000185760	KCNQ5	6.181903684	5.54E-06	1.527028089	0.331063165	7.708931774	2	2	4.058998049	8.178656739	8.03813956	3.44788957	4.228274364
ENSG00000161682	FAM171A2	2.502821509	0.063823695	5.206037609	2.22E-05	7.708859117	2	4.11352228	2	5.022023783	5.11361705	7.985196707	7.124815549
ENSG00000072952	MRVI1	1.35351924	0.527194576	6.354747267	0.000381431	7.708266507	2	3.076665209	7.552162999	7.352188525	7.34718809	12.37080139	12.25951588
ENSG00000158457	TSPAN33	2.494673508	0.255434807	5.209707605	0.00836966	7.704381114	2	2	2	3.023133209	3.50452879	5.832754931	4.720747915
ENSG00000139178	C1RL	4.258534669	4.16E-05	3.445464597	0.00111041	7.703999265	2	6.04685525	5.59507254	9.424141421	9.45821469	8.328342078	8.886919671
ENSG00000184887	BTBD6	3.298285651	0.019232909	4.403488605	0.001143848	7.701774256	4.00722531	6.535001748	2	8.404661252	8.45217004	9.526277199	9.523352442
ENSG00000071242	RPS6KA2	1.674283714	0.128350341	6.024894564	3.25E-10	7.699178278	4.815609471	6.78762695	4.058998049	7.477814553	7.16216989	11.08420293	12.04729732
ENSG00000174738	NR1D2	3.859667609	0.062809849	3.833296534	0.061534934	7.692964143	2	6.951736442	2	9.225498809	9.19732195	9.302094275	9.058129171
ENSG00000211455	STK38L	2.93561061	0.000690859	4.756575115	8.75E-09	7.692185725	6.816637977	7.847167909	5.156626401	9.954406146	9.82583819	11.92035242	11.45925874
ENSG00000058866	DGKG	5.416941938	0.00154394	2.271353055	0.225799741	7.688294993	2	2	2	5.501219927	5.63601114	3.235948948	3.071407821
ENSG00000130592	LSP1	4.003122896	0.074838025	3.683860214	0.098657948	7.68698311	3.327711234	2	2	5.058234798	5.2591458	5.669467138	3.012034821
ENSG00000167549	CORO6	2.253529971	0.067543282	5.433180548	8.58E-07	7.68671052	2	4.11352228	2	4.76150579	4.97065356	7.730090858	7.98359008
ENSG00000055332	EIF2AK2	4.383236353	0.000174604	3.301433038	0.005942742	7.684669392	6.89194353	7.819099893	3.369317972	11.22995516	11.2370588	10.23317759	10.07100411
ENSG00000135605	TEC	3.331780942	0.102509199	4.351550327	0.025215033	7.683331269	2	2	2	3.615725078	4.02226117	4.705557986	4.553838275
ENSG00000197483	ZNF628	3.267239111	0.001032776	4.412598143	4.37E-06	7.679837253	3.327711234	4.442710129	2	6.336376195	6.59518404	7.291067412	7.822284765
ENSG00000158711	ELK4	3.722573332	7.29E-17	3.948551853	4.49E-19	7.671125186	5.095959458	6.04685525	5.930865648	9.423273993	9.35675148	9.665695653	9.562677828
ENSG00000004866	ST7	3.175216663	0.070101138	4.493717652	0.007066912	7.668934315	3.327711234	7.051611315	2	8.695066731	8.71301725	10.19532025	9.807133274
ENSG00000100139	MICALL1	2.486778635	0.100010522	5.177808704	0.000167739	7.66458734	3.327711234	7.761270542	4.523675391	8.83470036	8.82115545	11.62791697	11.37763195
ENSG00000151470	C4orf33	4.497190771	0.000105898	3.16637398	0.008341427	7.663564751	2	4.11352228	2	6.912249745	6.97351258	5.801542492	5.5506886
ENSG00000185304	RGPD2	9.191187738	2.41E-07	-1.531443747	0.505767117	7.659743991	2	2	2	9.217515344	9.23691006	2.225793514	2
ENSG00000275793	RIMBP3	5.655514789	0.000990822	1.987812727	0.299605222	7.643327517	2	2	2	5.839660368	5.73886209	3.172301926	2.817676617
ENSG00000278087		12.82964265	2.59E-20	-5.191149786	0.022002471	7.638492869	2	2	2	12.834431	12.8788387	2	2
ENSG00000196110	ZNF699	3.180030469	0.006978751	4.455588863	7.83E-05	7.635619331	2	4.710564154	2	6.351046291	6.32089174	7.689935911	7.437040198
ENSG00000166823	MESP1	2.390486296	0.240656755	5.244923154	0.003905932	7.63540945	2	2	2	3.289987342	3.15372745	5.707383854	5.04340913
ENSG00000163840	DTX3L	4.88205459	1.01E-12	2.749619062	0.000110145	7.631673652	5.095959458	6.728495673	7.342949392	11.44969881	11.5103551	9.451446131	9.251026218
ENSG00000197256	KANK2	3.119952073	0.003412706	4.511395231	1.03E-05	7.631347304	4.815609471	8.433041974	7.225742779	10.52515083	10.5667454	11.96143224	11.90836987
ENSG00000145623	OSMR	3.747923683	0.006332639	3.88091809	0.004330689	7.628841772	4.467311984	6.04685525	8.978369997	11.33307966	11.3510373	11.46468397	11.48498941
ENSG00000159871	LYPD5	2.012858346	0.350878999	5.614784097	0.002663586	7.627642443	2	2	2	3.162721474	2.86123014	6.205571486	5.08721802
ENSG00000198753	PLXNB3	4.676561152	1.30E-05	2.950892864	0.008495313	7.627454016	2	4.442710129	2	7.580980221	7.42465798	5.812021848	5.888587685
ENSG00000119314	PTBP3	3.79211763	0.001734709	3.833328002	0.001437647	7.625445632	6.009025997	6.728495673	2	9.597176503	9.56082893	9.802049415	9.411845434
ENSG00000179388	EGR3	4.867856792	0.002189164	2.755531516	0.101324269	7.623388308	3.327711234	2	2	6.061031724	5.83486613	4.340871764	3.858622973
ENSG00000112658	SRF	2.953023063	0.086709662	4.668825289	0.003843291	7.621848352	4.00722531	7.35507212	2	8.812346549	8.84055903	10.41158826	10.64580898
ENSG00000029534	ANK1	0.69075686	0.745023679	6.928631153	1.18E-05	7.619388012	2	2	2	2.600425781	2.38539787	7.229857522	6.728114749
ENSG00000180263	FGD6	3.926971903	1.97E-10	3.688394811	2.27E-09	7.615366714	4.467311984	4.710564154	4.058998049	8.207157886	8.04037874	7.8927823	7.889811111
ENSG00000115561	CHMP3	3.840555027	0.004238809	3.772916475	0.004854847	7.613471501	7.384433967	4.442710129	3.369317972	9.802582299	9.80162141	9.289338026	10.07463605
ENSG00000126709	IFI6	7.167089072	6.78E-15	0.445860761	0.705642182	7.612949833	5.532353208	8.124485672	8.699419242	15.06164194	15.122211	8.047866378	8.663373824
ENSG00000137216	TMEM63B	4.292837833	7.99E-05	3.319124694	0.002864384	7.611962527	4.815609471	6.899080544	3.369317972	9.941741253	9.93757622	8.986541761	8.956482366
ENSG00000203867	RBM20	-0.949754075	0.638134514	8.556281998	2.95E-09	7.606527923	2	3.076665209	2	2.3892346	2.2680111	9.060551741	9.918182687
ENSG00000184992	BRI3BP	2.680571936	0.061396429	4.920407824	0.000187114	7.60097976	2	3.686303414	2	4.825935136	4.65713986	6.768721236	6.836905794
ENSG00000173530	TNFRSF10D	2.209350163	0.090904877	5.387704956	4.34E-06	7.597055119	2	6.728495673	6.203069917	8.017874397	8.14604733	11.10494797	11.36841437
ENSG00000117318	ID3	3.120496692	6.24E-05	4.475352413	3.10E-09	7.595849105	8.19931886	6.311476754	6.43197674	10.29936444	10.3782441	11.8938265	11.45604253
ENSG00000132669	RIN2	3.667421312	1.35E-07	3.928120356	1.25E-08	7.595541668	5.866944937	5.303523747	7.163363831	9.877326927	9.92974626	9.919572134	10.372072
ENSG00000156453	PCDH1	0.29836381	0.870417637	7.292692023	3.29E-08	7.591055833	4.467311984	2	2	3.615725078	3.28037825	10.37023041	8.864016001
ENSG00000248592	TMEM110-M	6.995547237	7.30E-06	0.593259835	0.768959881	7.588807072	2	2	2	7.075737784	7.05550676	2.592885947	2.326754077
ENSG00000048392	RRM2B	3.312820157	0.022492512	4.27288224	0.002294707	7.585702397	2	7.315436877	6.882775709	9.858198568	9.76756088	11.18456388	10.18753685
ENSG00000111802	TDP2	4.553110608	0.003256601	3.028670632	0.05973596	7.58178124	3.327711234	6.666836837	2	9.698163261	9.71736428	8.33380681	8.044120856

ENSG00000146833	TRIM4	3.491473863	0.000690666	4.088678657	4.96E-05	7.580152519	3.327711234	3.686303414	6.073378139	8.130575473	8.20911072	8.904453984	8.601149963
ENSG00000265817	FSBP	5.609066294	0.000800798	1.96731063	0.292598927	7.576376924	2	2	2	5.870535235	5.6121243	3.011868911	2.981406154
ENSG00000213199	ASIC3	3.180110954	0.160294059	4.393582479	0.039954567	7.573693433	2	2	2	3.566290607	3.86943969	5.147485955	3.940519005
ENSG00000116539	ASH1L	3.765275161	9.68E-05	3.807176369	7.43E-05	7.57245153	4.00722531	6.899080544	7.398165132	10.4133977	10.3378525	10.46679345	10.36726769
ENSG00000156381	ANKRD9	2.801762768	0.001483636	4.769637588	1.15E-08	7.571400356	5.532353208	4.442710129	2	7.190301983	7.14562489	9.280561171	8.906921063
ENSG00000175556	LONRF3	4.484969351	0.044941351	3.086070495	0.178705011	7.571039846	2	2	2	4.907595604	4.56059747	4.133696865	2.950113106
ENSG00000176148	TCP11L1	3.386774524	0.002505663	4.183163794	0.000124829	7.569938318	4.00722531	5.838937808	2	7.927778434	7.93600799	8.780536345	8.653086557
ENSG00000103404	USP31	3.769281769	0.00128085	3.799889924	0.001089398	7.569171694	3.327711234	5.595927442	2	7.947219213	7.97164382	8.086505553	7.885682058
ENSG00000115844	DLX2	5.739410546	0.001424077	1.82831572	0.367174663	7.567726267	2	2	2	5.890757403	5.84514933	2.62493832	3.183290849
ENSG00000085465	OVGP1	3.973653387	0.061258008	3.590991928	0.089476325	7.564645314	2	2	2	4.139692846	4.4836996	4.350488971	3.600986722
ENSG00000129315	CCNT1	4.002785425	0.062310616	3.56128226	0.096925701	7.564067685	2	7.145017454	2	9.572351151	9.52289845	9.473343311	8.620535396
ENSG00000162139	NEU3	2.731587147	0.024129772	4.826598526	1.90E-05	7.558185673	2	4.710564154	2	5.720439317	6.08095082	8.035995406	7.819040576
ENSG00000124201	ZNFX1	4.552686663	1.50E-05	3.002286521	0.006037433	7.554973184	5.330594134	8.777382925	8.960138144	12.83793404	12.9288719	11.20035539	11.45847708
ENSG00000173918	C1QTNF1	4.710741917	0.003177064	2.838573521	0.090878546	7.549315438	4.00722531	7.232742069	2	10.44536591	10.4770185	8.247007147	8.88330014
ENSG00000158714	SLAMF8	4.828951605	0.055881142	2.717769255	0.303576997	7.546720859	2	2	2	5.040242897	5.04390545	4.017766949	2.374681325
ENSG00000125148	MT2A	6.407060603	1.60E-13	1.139420581	0.26164227	7.546481184	8.285626337	7.572420273	5.39244166	13.8152717	13.9436096	8.944296493	8.220862755
ENSG00000186417	GLDN	3.418726327	0.102212351	4.126967663	0.039942724	7.545693991	2	2	2	4.070463721	3.69849104	4.644324387	4.215171317
ENSG00000185519	FAM131C	3.181160802	0.122984674	4.362248004	0.025584749	7.543408806	2	2	2	3.709785322	3.74316441	4.854926465	4.388595494
ENSG00000158887	MPZ	3.180813036	0.137755718	4.361191917	0.031648409	7.542004954	2	2	2	3.663521627	3.7864959	4.990270811	4.175132639
ENSG00000175564	UCP3	4.163332285	0.039266418	3.373767598	0.097996916	7.537099882	2	2	2	4.17310023	4.72553021	3.917810786	3.807156736
ENSG00000137478	FCHSD2	2.711963179	0.228046677	4.824695066	0.02108503	7.536658245	2	7.274681896	2	8.350935846	8.44373613	10.56738332	10.42424552
ENSG00000115648	MLPH	5.070932377	5.36E-09	2.465633025	0.007862246	7.536565402	2	4.11352228	4.523675391	8.532433657	8.52753255	6.295446113	5.631815919
ENSG00000171873	ADRA1D	4.699497129	0.060057468	2.836764058	0.274683967	7.536261187	2	2	2	4.739373666	5.09650239	4.088436536	2.466008509
ENSG00000178996	SNX18	2.130164232	0.214848439	5.405356275	0.000410013	7.535520507	4.00722531	7.145017454	2	7.862723323	7.77859536	11.33504966	10.75353482
ENSG00000135823	STX6	3.016848168	0.062403761	4.513709512	0.003194404	7.53055768	4.00722531	7.051611315	2	8.610716699	8.61125371	9.935834217	10.2442687
ENSG00000178075	GRAMD1C	4.16961602	0.019121386	3.355993757	0.063125672	7.525609777	2	3.076665209	2	5.144975053	5.45984938	5.032703865	3.987514168
ENSG00000224531	SMIM13	3.433254972	0.075515059	4.09219879	0.028865749	7.525453763	2	6.389896633	2	8.221200057	8.22887947	9.145869306	8.545966004
ENSG00000172216	CEBPB	4.430886458	3.68E-09	3.082184506	6.32E-05	7.513070964	6.009025997	7.701025996	8.408111533	12.04993732	12.1001021	10.80593075	10.64687673
ENSG00000020577	SAMD4A	2.804066637	0.009617767	4.705541026	4.12E-06	7.509607663	7.031698319	7.669933291	4.058998049	9.641111693	9.64637295	11.10796328	11.8719151
ENSG00000137177	KIF13A	3.682379034	3.01E-18	3.825502873	7.88E-20	7.507881907	7.384433967	6.728495673	6.322058544	10.46193323	10.5616878	10.65560768	10.65523878
ENSG00000145246	ATP10D	3.371111502	0.022108229	4.136767086	0.003803056	7.507878588	2	5.838937808	7.502634626	9.610955295	9.66245344	10.54570096	10.23699419
ENSG00000179111	HES7	4.064223735	0.062918791	3.440072984	0.116333358	7.504296719	2	2	2	4.358765249	4.43005853	4.321442857	3.33635788
ENSG00000254852	NPIPA2	11.23534759	4.39E-16	-3.744722507	0.101374362	7.490625088	2	2	2	11.26329852	11.2648535	2	2.059860178
ENSG00000257594	GALNT4	6.27619247	1.35E-05	1.214222082	0.478299157	7.490414552	2	3.076665209	2	7.25413313	7.34718809	3.215043717	2.981406154
ENSG00000077150	NFKB2	4.011487073	0.003482895	3.477957869	0.012368406	7.489444942	4.00722531	7.701025996	4.058998049	10.27889147	10.3005071	9.463959918	10.00258629
ENSG00000142623	PADI1	1.4987618	0.487075206	5.98487083	0.000780302	7.48363263	2	2	2	2.947924294	2.59489879	5.598599872	6.468583424
ENSG00000196460	RFX8	8.209931355	1.73E-08	-0.727281878	0.711605352	7.482649476	2	2	2	8.235106869	8.27524772	2.139668783	2.277179783
ENSG00000184481	FOXO4	2.989536499	0.000119872	4.491543627	1.85E-09	7.481080127	4.467311984	4.442710129	2	6.70517998	6.6952083	8.204552906	8.126037235
ENSG00000176454	LPCAT4	3.309433968	0.00052729	4.17110557	7.22E-06	7.480539538	2	5.72254323	5.156626401	8.001694993	8.22494733	9.148283563	8.766338209
ENSG00000122691	TWIST1	5.288927208	1.99E-09	2.1858045	0.022620058	7.474731708	4.00722531	4.710564154	2	8.874592369	8.96718201	6.239610476	5.470251342
ENSG00000198853	RUSC2	2.842806896	0.017939507	4.628576928	4.36E-05	7.471383824	7.485143553	8.337339476	4.058998049	10.19853443	10.3126002	12.08454008	11.99219313
ENSG00000050327	ARHGEF5	4.739401809	1.55E-08	2.726372975	0.001899142	7.465774784	4.467311984	2	4.874587708	8.469961514	8.5130942	6.459963649	6.590289384
ENSG00000138246	DNAJC13	4.203235238	0.011917779	3.261781339	0.055805422	7.465016578	4.467311984	7.701025996	2	10.4372252	10.4308254	9.580405632	9.407171982
ENSG00000148842	CNNM2	1.811933407	0.183141741	5.650823555	2.04E-06	7.462756962	4.467311984	6.311476754	2	6.965655398	6.77267312	11.02068045	10.19109642
ENSG00000101935	AMMECR1	3.716675033	0.000483867	3.743679224	0.000403865	7.460354258	3.327711234	5.303523747	2	7.725907304	7.5835711	7.51817649	7.830900438
ENSG00000101335	MYL9	2.041738462	4.17E-05	5.416981309	1.17E-30	7.458719771	10.44422623	10.22039562	9.272377397	12.10655496	12.0929736	15.33288762	15.60222779
ENSG00000152332	UHMK1	3.89149463	0.007379211	3.564639218	0.014671069	7.456133848	7.579279349	7.538394143	2	10.8903519	10.7920865	10.43748218	10.59043716
ENSG00000164125	FAM198B	3.812485021	5.28E-05	3.641994132	0.000110991	7.454479153	3.327711234	4.710564154	2	7.307758725	7.16627666	7.258234779	6.860240429
ENSG00000149115	TNKS1BP1	3.277288373	0.000432249	4.17698842	4.12E-06	7.454276793	5.330594134	8.147241016	8.266922072	10.93607039	11.0094017	11.77710915	11.96021567
ENSG00000182326	C1S	5.022304587	7.00E-06	2.429928172	0.043006389	7.452232759	5.532353208	9.514922632	9.099922433	13.78119023	13.8184383	10.77323249	11.54414712
ENSG00000172927	MYEOV	2.391693452	0.324077768	5.055365338	0.021023289	7.44705879	2	2	2	3.406930997	3.01487888	4.088436536	5.876154317
ENSG00000132613	MTSS1L	3.277662631	0.007863092	4.16859713	0.000478334	7.446259761	2	5.946638857	6.718950348	8.954818504	9.07377798	9.785748878	10.00829467
ENSG00000278318	ZNF229	4.078374474	3.96E-08	3.365118452	7.46E-06	7.443492926	5.330594134	4.93638933	2	8.517880242	8.5227358	7.907516231	7.720479276
ENSG00000196834	POTEI	9.590271172	8.18E-12	-2.15516918	0.263504222	7.435101992	2	2	2	9.55186119	9.69266358	2.048074405	2.117335275
ENSG00000100629	CEP128	3.0453365	0.148151369	4.383901602	0.026905364	7.429238103	2	2	2	3.406930997	3.82856379	4.521997428	4.784231301
ENSG00000102221	JADE3	3.300383865	0.034024524	4.12880432	0.006072155	7.429188185	2	5.303523747	2	6.984595707	7.04216061	8.227567998	7.256957802
ENSG00000176273	SLC35G1	3.012182896	0.010924161	4.414206648	8.78E-05	7.426389544	2	4.442710129	2	5.987823101	5.80356929	7.08038692	7.382408159
ENSG00000116514	RNF19B	4.100192263	0.018991992	3.326140156	0.060701932	7.426332419	2	6.04685525	2	8.519504557	8.56222967	7.908330388	7.634508409
ENSG00000108622	ICAM2	3.148558051	0.137320752	4.277606407	0.03302563	7.426164457	2	2	2	3.663521627	3.74316441	4.230621763	4.845038551
ENSG00000181751	C5orf30	3.637647867	3.06E-07	3.78416694	8.27E-08	7.421814807	6.009025997	5.303523747	3.369317972	8.797695539	8.70739158	8.765787532	9.020021944
ENSG00000180777	ANKRD30B	5.045281219	8.78E-05	2.369149985	0.088401773	7.414431204	2	4.11352228	2	7.464384256	7.44169935	4.416072028	5.331426166
ENSG00000173110	HSPA6	5.666112195	0.001534589	1.745133011	0.388182097	7.411245206	2	2	2	5.697663076	5.89549381	3.10571689	2.632958241
ENSG00000173905	GOLIM4	3.666427024	0.001296732	3.744125805	0.000940881	7.410552829	4.467311984	7.73146271	5.156626401	10.1651988	10.0755276	10.00159102	10.371703
ENSG00000254788	CKLF-CMTM1	5.883876011	0.000275437	1.525476148	0.416362316	7.409352159	2	2	2	5.949776857	6.05460702	2.803598391	2.782576693
ENSG00000072110	ACTN1	2.788584999	1.88E-08	4.620747411	1.06E-21	7.40933241	9.689059027	9.785372135	8.585098385	12.20913422	12.2509268	14.08406122	14.03853699
ENSG00000152291	TGOLN2	3.665718382	3.53E-06	3.741767008	1.97E-06	7.40748539	7.096806373	9.338312924	7.896971941	12.05512627	12.0847252	11.97178976	12.30138363
ENSG00000065809	FAM107B	3.228864661	0.002123869	4.178433353	4.08E-05	7.407298013	4.815609471	6.666836837	3.369317972	8.729158235	8.67605209	9.444446837	9.822335095
ENSG00000188266	HYKK	2.63457679	0.122761253	4.772305538	0.002281644	7.406882328	2	3.076665209	2	4.034562184	4.02226117	6.010089548	5.699242824
ENSG00000157657	ZNF618	4.448810538	0.000273699	2.954377145	0.020693942	7.403187683	2	6.602424674	5.156626401	9.844008139	9.88372429	8.559441176	8.176765766
ENSG00000107077	KDM4C	4.03360758	0.021123194	3.361693325	0.05738556	7.395300904	2	6.04685525	2	8.474993083	8.47552601	7.755649985	7.868000534
ENSG00000196369	SRGAP2B	6.831623359	9.16E-12	0.559242485	0.661268816	7.390865844	4.815609471	2	3.369317972	10.11504861	10.0501952	4.487545924	3.753786374
ENSG00000119682	AREL1	3.528922327	7.60E-05	3.861710382	1.17E-05	7.390632709	5.095959458	7.002537973	4.523675391	9.463489617	9.39312469	9.632330087	9.876916845
ENSG00000170469	SPATA24	2.977468897	0.181130407	4.412705086	0.034523766	7.390173983	2	2	2	3.881293667	3.2184422	4.959178705	4.341135913
ENSG00000120322	PCDHB8	4.522783711	0.017920625	2.865134761	0.149193138	7.387918472	2	2	2	4.846787657	4.70309173	3.465698119	3.560117069
ENSG00000114019	AMOTL2	2.290129305	0.076570922	5.097641836	1.38E-05	7.387771141	4.00722531	7.819099893	8.536706852	9.948991943	9.93637437	12.79607961	12.6923973
ENSG00000149177	PTPRJ	2.713266121	1.86E-05	4.671236946	1.65E-14	7.384503067	5.532353208	7.274681896	6.80318718	9.358500114	9.41399842	11.05674387	11.57340217
ENSG00000100580	TMED8	3.37331142	0.000123283	4.008946988	3.28E-06	7.382258408	4.467311984	3.686303414	6.203069917	8.358215887	8.34212018	8.973764228	8.985229012
ENSG00000072364	AFF4	3.619344728	0.016568695	3.75613387	0.01202378	7.375478598	9.667789716	7.051611315	4.058998049	11.95289514	11.9020956	12.0083211	12.11830098
ENSG00000130147	SH3BP4	3.24018063	0.013191227	4.129721985	0.001089398	7.369902615	3.327711234	8.053983481	6.629488387	10.16052179	10.1858391	10.74991732	11.31587024
ENSG00000138028	CGREF1	1.444434559	0.362218166	5.925239184	8.16E-06	7.369673743	2	3.686303414	2	3.615725078	3.90918929	8.055971376	7.429968874
ENSG00000172164	SNTB1	3.307045293	0.000124409	4.058124793	1.45E-06	7.365170086	2	5.457120953	4.874587708	7.708923796	7.94798467	8.648868792	8.495377414
ENSG00000010030	ETV7	4.714492707	0.00020089	2.649701998	0.04875895	7.364194705	2	2	4.874587708	7.617841552	7.77591026	5.626574484	5.807970301
ENSG00000070731	ST6GALNAC2	4.327352479	0.00692683	3.032895919	0.066788326	7.360248398	2	3.076665209	2	5.420062284	5.48637102	4.406883583	4.26688425
ENSG00000123500	COL10A1	8.776980359	5.95E-10	-1.417516949	0.453396071	7.359463409	2	2	2	8.832088406	8.80016655	2.094598369	2.172608095
ENSG00000139304	PTPRQ	3.571713969	0.120948604	3.783434219	0.092291365	7.355148188	2	2	2	3.95996233	4.05806467	3.465698119	4.644600858
ENSG00000130066	SAT1	4.926976378	2.72E-14	2.423220296	0.000354082	7.350196674	8.39321512	7.099070191	7.285536013	12.62471618	12.5906835	10.54740056	9.474014382
ENSG00000160299	PCNT	3.302237014	0.03878829	4.044692574	0.008995102	7.346929588	2	6.602424674	3.369317972	8.319579168	8.4936155	9.182675508	9.10594367
ENSG00000174370	C11orf45	4.079870386	0.101901089	3.266398909	0.192069539	7.346269295	2	2	2	4.105493497	4.65713986	4.360042493	2.817676617
ENSG00000196498	NCOR2	3.062873503	0.029091374	4.282502156	0.001407621	7.345375659	3.327711234	8.451443573	8.434763955	10.90471749	10.9382377	12.13331984	12.14564413
ENSG00000140526	ABHD2	3.520431063	0.00084619	3.822843421	0.000240226	7.343274484	3.327711234	6.389896633	7.163363831	9.755113959	9.75670736	9.960968181	10.14714405
ENSG00000081052	COL4A4	-0.208684324	0.908905358	7.551796196	1.83E-09	7.343111872	2	3.686303414	2	3.023133209	2.59489879	9.385734536	9.401037612
ENSG00000104635	SLC39A14	4.79258068	2.97E-06	2.547651632	0.019771557	7.340232312	5.330594134	8.993716073	8.296287027	12.93963415	12.9555902	10.73381744	10.67645725
ENSG00000179431	FJX1	3.790146787	0.033889281	3.548761401	0.046421006	7.338908188	2	6.04685525	2	8.201097719	8.2657098	7.682340325	8.253273225
ENSG00000138135	CH25H	5.834501132	0.000451072	1.502676835	0.435247515	7.337177968	2	2	2	6.024891728	5.88556448	2.885263063	2.671842496
ENSG00000173276	ZBTB21	4.230065652	0.046591905	3.10601288	0.151419628	7.336078532	2	7.189546432	2	9.79991149	9.83811764	8.832074253	8.560285
ENSG00000134802	SLC43A3	6.224579334	2.37E-10	1.111449232	0.339130027	7.336028566	2	4.710564154	2	9.311457162	9.36838404	4.620674722	4.365060851
ENSG00000136048	DRAM1	3.256129734	3.52E-07	4.076905733	8.68E-11	7.333035467	8.716893051	7.73146271	6.958202216	11.2069543	11.2228348	11.84522962	12.20204378
ENSG00000103269	RHBDL1	2.658055954	0.127502931	4.673947578	0.003551201	7.332003532	2	3.076665209	2	3.997744386	4.0930011	6.013120342	5.481238982
ENSG00000164106	SCRG1	1.372926523	0.484134885	5.954595254	0.000242523	7.327521776	2	3.076665209	2	3.349643765	3.01487888	6.242195894	7.478755384
ENSG00000102024	PLS3	2.760994088	0.000660428	4.561845628	3.85E-09	7.322839716	9.174465797	8.993716073	6.80318718	11.380775	11.4074231	13.20212973	13.18477655
ENSG00000143776	CDC42BPA	3.08634527	1.18E-09	4.227799196	1.88E-17	7.314144466	5.866944937	7.232742069	6.534109959	9.685919789	9.73682479	10.83169479	10.86666148
ENSG00000172650	AGAP5	7.776846802	1.33E-07	-0.463010793	0.818792684	7.313836009	2	2	2	7.821180652	7.83643742	2.3070652	2.172608095
ENSG00000130707	ASS1	2.755359231	0.034922085	4.551961909	0.000201678	7.30732114	5.532353208	6.666836837	2	8.349110081	8.33666436	9.60772968	10.50771945
ENSG00000087510	TFAP2C	1.754986582	0.166572561	5.552206848	4.75E-07	7.30719343	2	4.93638933	2	5.22679461	5.2591458	8.834646275	8.946110698
ENSG00000160685	ZBTB7B	3.137116684	0.169849097	4.169984963	0.055706281	7.307101647	2	7.467835385	2	9.004380786	9.02033034	10.04017303	10.03882852
ENSG00000163888	CAMK2N2	3.975283781	0.050350232	3.326786254	0.102751946	7.302070034	2	2	2	4.387512679	4.2560182	3.917810786	3.735548302
ENSG00000154654	NCAM2	6.329000542	0.000586428	0.969062781	0.666867266	7.298063323	2	2	2	6.306580752	6.53293476	2.831336763	2.277179783

ENSG00000144395	CCDC150	3.011023436	0.148866715	4.285191095	0.029111318	7.296214531	2	2	2	3.566290607	3.65239003	4.461156421	4.683176719
ENSG00000198756	COLGALT2	2.724014486	0.053129029	4.568827205	0.000492089	7.29284169	2	4.442710129	2	5.818703821	5.34860058	6.770513376	7.776188509
ENSG00000112541	PDE10A	0.276588494	0.878583032	7.010675373	4.46E-08	7.287263867	2	3.686303414	2	2.784612485	3.28037825	8.923506363	8.778637453
ENSG00000121743	GJA3	2.819852287	0.177650086	4.466676831	0.021719442	7.286529118	2	2	2	3.615725078	3.33976443	4.727866721	4.729989799
ENSG00000162734	PEA15	2.89806883	2.65E-05	4.386257963	5.04E-11	7.284326793	8.075571721	9.328390744	10.02619908	12.21691381	12.258881	13.53349039	13.89487424
ENSG00000157766	ACAN	-0.893518308	0.552070286	8.176877342	2.60E-12	7.283359033	2	5.131603625	2	3.566290607	3.08597292	11.67280806	11.72040189
ENSG00000143469	SYT14	6.467457802	6.79E-05	0.810403595	0.689153776	7.277861397	2	2	2	6.548530093	6.56439512	2.384001718	2.671842496
ENSG00000182957	SPATA13	4.5774309	2.86E-09	2.699722514	0.000778051	7.277153414	5.095959458	4.11352228	2	8.508095759	8.40776525	6.693258166	6.55171954
ENSG00000269313	MAGIX	3.333646724	0.113679396	3.938947329	0.052055276	7.272594053	2	2	2	3.881293667	3.7864959	4.111244182	4.456980017
ENSG00000166963	MAP1A	3.208800099	1.67E-06	4.063503982	6.50E-10	7.272304081	8.493334308	9.695596167	8.007629717	12.09175474	12.1430612	12.68025986	13.21414468
ENSG00000106327	TFR2	3.669898089	0.100833309	3.600156373	0.101255863	7.270054462	2	2	2	4.470469939	3.55551834	3.904814898	4.147809227
ENSG00000158186	MRAS	2.633843194	4.23E-05	4.627970767	5.85E-14	7.261813961	6.009025997	7.538394143	6.718950348	9.496029474	9.50355257	11.05729453	11.81917129
ENSG00000172059	KLF11	3.202369987	0.002137885	4.058777008	6.10E-05	7.261146995	3.327711234	5.838937808	3.369317972	7.799951945	7.72392024	8.839776604	8.328971229
ENSG00000055208	TAB2	3.638775437	0.059087638	3.619765716	0.057302601	7.258541154	4.815609471	8.671479029	2	10.81265775	10.797723	10.99149283	10.54681386
ENSG00000156925	ZIC3	1.019042799	0.778887556	6.237754085	0.036451497	7.256796883	2	2	2	3.023133209	2	6.092633892	6.546498097
ENSG00000106683	LIMK1	4.42576042	2.63E-29	2.830542578	1.20E-12	7.256302998	6.468621203	6.535001748	5.930865648	10.6671059	10.7478979	8.983840816	9.244188987
ENSG00000113448	PDE4D	5.506463548	7.03E-06	1.749526342	0.204157205	7.25598989	2	4.11352228	2	7.870381136	7.95512324	4.764298067	3.987514168
ENSG00000149639	SOGA1	2.606993285	1.60E-05	4.647993458	1.07E-15	7.254986743	6.009025997	7.35507212	7.552162999	9.649295375	9.72990471	11.41699656	11.98014078
ENSG00000106772	PRUNE2	-3.752916103	0.531143362	11.00549732	0.028438282	7.252581214	2	2	2	2	2	10.26164954	11.53536074
ENSG00000174106	LEMD3	3.564029911	2.40E-05	3.685059989	1.09E-05	7.2490899	2	5.131603625	5.59507254	8.199071993	8.284723	8.362013331	8.362041249
ENSG00000109320	NFKB1	4.19387434	0.059805354	3.052872323	0.17862881	7.246746663	2	7.503546079	2	10.06086293	10.136455	8.62331206	9.240151898
ENSG00000119771	KLHL29	2.513856288	0.010502522	4.730024455	2.45E-07	7.243880743	2	5.303523747	5.59507254	7.202487431	7.38644297	9.087759434	9.795350748
ENSG00000167207	NOD2	3.771481084	0.028728494	3.469198385	0.043887828	7.240679469	3.327711234	2	2	4.867343068	5.04390545	4.984105765	4.341135913
ENSG00000173193	PARP14	5.128672735	1.99E-05	2.11032117	0.108033027	7.238993905	4.467311984	9.00621767	7.552162999	13.01713496	13.0340228	10.20843574	9.78485758
ENSG00000051341	POLQ	3.548274734	0.116163431	3.684300299	0.094939611	7.232575033	2	2	2	4.105493497	3.86943969	3.5515855	4.489993931
ENSG00000197780	TAF13	3.708342731	0.070288814	3.52006932	0.083321465	7.228412051	2	2	6.534109959	8.572106084	8.60521604	8.271259924	8.522888769
ENSG00000121552	CSTA	5.437607043	0.026843757	1.787206801	0.513707623	7.224813844	2	2	2	5.590471342	5.58783529	3.393105702	2.172608095
ENSG00000104154	SLC30A4	2.468611917	0.001318023	4.753766017	4.55E-11	7.222377934	4.467311984	3.686303414	4.523675391	6.586219071	6.23747437	8.852100097	8.421719243
ENSG00000117013	KCNQ4	0.858796749	0.684667709	6.35974563	8.20E-05	7.218542379	2	2	2	2.498691189	2.59489879	6.625560108	6.257923755
ENSG00000171790	SLFNL1	0.272692498	0.911517345	6.944313062	4.65E-05	7.21700556	2	2	2	2.270786764	2.49394783	7.463831776	6.362645675
ENSG00000137962	ARHGAP29	1.801497033	0.20822859	5.414893977	1.61E-05	7.216391009	6.89194353	6.899080544	2	8.102673083	8.08227582	11.34184575	11.96425948
ENSG00000115548	KDM3A	4.319142138	0.00281989	2.892201893	0.05435683	7.211344031	2	7.145017454	4.874587708	10.13319806	10.0858626	8.839349773	8.526871175
ENSG00000254996	ANKHD1-EIF4	7.021681409	4.58E-19	0.186687095	0.862702263	7.208368504	4.00722531	4.11352228	2	10.19039992	10.2123959	3.796405078	3.875378318
ENSG00000076513	ANKRD13A	2.778331682	5.07E-07	4.429043181	1.48E-16	7.207374863	6.366709671	7.35507212	5.930865648	9.427605927	9.44644299	11.06762631	11.09639353
ENSG00000171109	MFN1	3.828013466	0.079373234	3.374537077	0.121142926	7.202550543	2	7.274681896	2	9.506715015	9.50274083	9.030205459	9.077717044
ENSG00000132688	NES	1.927496978	0.188460431	5.273343721	4.74E-05	7.200840699	4.815609471	8.004992147	4.058998049	8.814994458	8.23280093	11.62445289	12.10559801
ENSG00000282637		2.120012726	0.108674252	5.076619508	1.73E-05	7.196632234	2	4.710564154	2	5.289065324	5.39133044	7.761068522	8.485179747
ENSG00000163235	TGFA	6.168884709	9.95E-05	1.023739846	0.591655943	7.192624555	2	2	3.369317972	7.261917051	7.12888795	3.296906311	2.632958241
ENSG00000154864	PIEZO2	0.901266606	0.661500335	6.290655063	0.000109903	7.191921669	2	3.076665209	2	3.289987342	2.49394783	7.117472117	7.489678691
ENSG00000153253	SCN3A	5.354282511	0.011041916	1.835252001	0.434458206	7.189534512	2	2	2	5.433909581	5.58783529	2.42098416	3.311948089
ENSG00000173120	KDM2A	3.791443751	0.000619313	3.396428122	0.002339673	7.187871873	4.00722531	7.901717006	7.691296561	10.99183871	11.0615956	10.63841368	10.62753531
ENSG00000009413	REV3L	3.773255152	0.00024102	3.413563906	0.000966346	7.186819058	4.00722531	7.145017454	5.156626401	9.793212756	9.66027117	9.488666496	9.24176809
ENSG00000197142	ACSL5	5.491250308	1.83E-05	1.689943535	0.243517965	7.181193843	2	2	4.058998049	7.497728447	7.33629421	3.917810786	4.077147165
ENSG00000181652	ATG9B	4.150350771	0.042255933	3.030015804	0.14563462	7.180366575	2	2	2	4.670864177	4.22485516	3.584558147	3.660199087
ENSG00000131725	WDR44	3.57931774	7.67E-05	3.59774275	6.46E-05	7.177060491	2	5.595927442	5.39244166	8.30834735	8.49850989	8.544331623	8.294346816
ENSG00000250254	PTTG2	3.471550796	0.104383657	3.704968003	0.075016235	7.176518799	2	2	2	4.034562184	3.82856379	3.904814898	4.292061816
ENSG00000140465	CYP1A1	4.114055548	0.078971311	3.061049818	0.19694971	7.175105367	2	2	2	4.17310023	4.65713986	2.962551659	4.105828626
ENSG00000181195	PENK	6.933700553	2.18E-06	0.236614424	0.904062298	7.170314977	2	5.595927442	2	10.9250823	10.8900091	4.894909044	3.924507493
ENSG00000174282	ZBTB4	2.752031432	0.063911628	4.416030162	0.001529662	7.168061594	3.327711234	8.981105197	7.934808822	10.68892298	10.7523441	12.47675767	12.28192772
ENSG00000115904	SOS1	3.53496833	0.011191826	3.631375125	0.008618924	7.166343456	6.138360429	7.315436877	2	9.771565364	9.75670736	10.11013328	9.557495966
ENSG00000173757	STAT5B	2.839686165	0.040073481	4.32450111	0.000934116	7.164187274	2	7.099070191	5.930865648	8.882187604	8.8250571	10.23852871	10.41728984
ENSG00000006607	FARP2	1.822550436	0.211727163	5.340695751	3.07E-05	7.163246188	4.00722531	6.389896633	2	6.851641821	6.86652414	10.0366402	10.57561499
ENSG00000154429	CCSAP	2.991703724	0.000698097	4.169997409	9.51E-07	7.161701134	4.815609471	5.131603625	2	7.423326516	7.15805139	8.746340327	8.087908463
ENSG00000151914	DST	3.237516714	8.99E-05	3.92333883	1.27E-06	7.160855543	10.26462289	8.916342327	7.818184403	12.55227704	12.5779266	13.18885183	13.30984437
ENSG00000167971	CASKIN1	2.387612241	0.273627021	4.767484298	0.015667908	7.155096539	2	2	2	2.947924294	3.45167086	5.125248504	4.836507146
ENSG00000181284	TMEM102	3.083146923	0.01291029	4.06523846	0.000637466	7.148385382	2	4.11352228	2	5.565529336	5.63601114	6.475428841	6.569847194
ENSG00000160741	CRTC2	3.57687149	0.075654914	3.561297305	0.072951675	7.138168795	2	6.666836837	2	8.630393142	8.65866647	8.678282924	8.578306014
ENSG00000173821	RNF213	4.446528568	1.68E-17	2.68872402	4.84E-07	7.135252588	9.410448395	8.717821467	8.007629717	13.22864867	13.2860975	11.59105918	11.40547739
ENSG00000182175	RGMA	2.555463744	0.551538856	4.579378793	0.251602132	7.134842537	2	2	2	3.997744386	2	4.778616857	4.870334174
ENSG00000179954	SSC5D	2.770940849	0.033290369	4.363195214	0.00037493	7.134136063	5.532353208	6.666836837	2	8.378048624	8.33848526	9.457021219	10.29943592
ENSG00000125817	CENPB	3.024221293	0.054799589	4.109421294	0.006373412	7.133642586	8.042902253	7.669933291	2	10.19904132	10.3751428	11.35802693	11.38688232
ENSG00000114480	GBE1	3.420396981	0.024746731	3.712341746	0.013403649	7.132738727	7.579279349	7.901717006	2	10.56816382	10.5644133	10.75818265	10.95046432
ENSG00000272333	KMT2B	3.353699635	0.121326135	3.778020727	0.072863536	7.131720362	2	7.099070191	2	8.793673851	8.91810182	9.373110172	9.176932811
ENSG00000167202	TBC1D2B	3.130476712	0.192379762	3.997448234	0.082542158	7.127924947	2	7.847167909	2	9.354865403	9.41658653	10.52342208	9.910074228
ENSG00000198399	ITSN2	3.276583718	0.103576285	3.850911914	0.048101168	7.127495632	2	6.602424674	2	8.270263574	8.2960118	8.916644086	8.783084035
ENSG00000111252	SH2B3	4.266270714	0.00023647	2.85899777	0.018167791	7.125268484	6.468621203	8.469613413	4.523675391	11.50034187	11.5421087	9.88710712	10.31583232
ENSG00000114023	FAM162A	3.269492314	0.00621802	3.854939322	0.000948646	7.124431637	6.366709671	4.710564154	2	8.30834735	8.3920703	8.923506363	8.937160962
ENSG00000112078	KCTD20	1.649689172	0.102464016	5.474288926	6.80E-10	7.123978098	7.709880939	9.018611864	6.073378139	9.72797373	9.65735634	13.61012915	13.40498581
ENSG00000116199	FAM20B	2.635524718	0.000170716	4.478100077	2.51E-11	7.113624794	6.468621203	6.728495673	4.523675391	8.801706047	8.74219737	10.47327283	10.72623001
ENSG00000169981	ZNF35	3.49348253	0.015288584	3.619880014	0.011100946	7.113362544	2	5.131603625	2	7.127781374	6.93551934	6.838725184	7.418582165
ENSG00000077264	PAK3	4.766228498	0.014403895	2.330410594	0.264448359	7.096639092	2	2	2	4.984880435	4.98931816	3.393105702	2.950113106
ENSG00000139289	PHLDA1	5.558719374	2.16E-44	1.532842195	0.000319758	7.091561569	7.53297911	7.467835385	8.236946975	13.31874609	13.3075788	9.462297671	9.107717328
ENSG00000172058	SERF1A	8.278040659	7.83E-09	-1.18744399	0.532750733	7.09059667	2	2	2	8.304583895	8.34030387	2.183373728	2.117335275
ENSG00000168961	LGALS9	5.207392534	3.85E-05	1.882503368	0.179033043	7.089895902	2	4.93638933	2	8.529212228	8.5227358	5.677130623	4.886954834
ENSG00000141497	ZMYND15	3.046463071	0.161240809	4.043029878	0.049620411	7.089492949	2	2	2	3.566290607	3.69849104	4.122514202	4.594883674
ENSG00000109063	MYH3	4.01120481	0.049833933	3.070546395	0.138210579	7.081751205	2	2	2	4.329433326	4.37434574	3.600766115	3.698365557
ENSG00000172183	ISG20	5.599378006	4.78E-05	1.480808025	0.347140092	7.080186032	2	6.311476754	3.369317972	10.40067384	10.4444166	6.231826305	6.498634707
ENSG00000100097	LGALS1	3.867594037	3.83E-06	3.201605637	0.000161199	7.069199674	10.55263183	8.57407404	8.585098385	13.39748587	13.4440161	12.33389445	13.08096347
ENSG00000115009	CCL20	7.578465761	5.71E-06	-0.511052057	0.820068239	7.067413705	2	2	2	7.68596357	7.58049694	2.094598369	2.374681325
ENSG00000249884	RNF103-CHM	9.779844158	4.18E-11	-2.712987522	0.194894738	7.066856637	2	2	2	9.80923777	9.81606952	2	2.117335275
ENSG00000152495	CAMK4	4.180617221	0.046457783	2.885804541	0.179747748	7.066421762	2	2	2	4.387512679	4.58534546	3.767980144	3.236138336
ENSG00000196233	LCOR	2.78006534	0.040340218	4.286163549	0.000804245	7.066228889	4.467311984	6.535001748	2	8.056427969	7.93841129	9.451725397	9.52733357
ENSG00000168298	HIST1H1E	4.319232539	0.031747935	2.744561561	0.188337529	7.0637941	2	2	2	4.670864177	4.53541753	3.27687201	3.580696614
ENSG00000053438	NNAT	4.304670729	0.042473193	2.757015011	0.209143226	7.06168574	2	2	2	4.670864177	4.50979031	3.738983888	3.071407821
ENSG00000174136	RGMB	4.529982179	8.15E-11	2.52414992	0.000524836	7.054132099	6.009025997	7.928237109	6.534109959	11.58441754	11.553128	9.647281667	9.485655697
ENSG00000111328	CDK2AP1	3.967486498	3.85E-33	3.082175817	1.55E-20	7.049662315	7.096806373	6.844429755	6.958202216	10.91122844	10.862748	9.880069077	10.11906036
ENSG00000149308	NPAT	3.619797894	0.051517521	3.428069921	0.063487602	7.047867815	2	6.228547981	2	8.264463296	8.23084153	8.07857076	8.038552764
ENSG00000127329	PTPRB	4.398178356	3.84E-05	2.648500109	0.018649379	7.046678466	3.327711234	3.076665209	4.058998049	7.326437704	7.31795292	6.075339068	5.0581604
ENSG00000063127	SLC6A16	2.080764065	0.328511143	4.964901421	0.008306252	7.045665486	2	2	2	2.947924294	3.15372745	5.357600875	4.935697922
ENSG00000131018	SYNE1	3.108843219	0.003101637	3.934744306	0.000115824	7.043587525	5.330594134	8.414402626	8.825624954	11.18332463	11.2399842	12.20539422	11.84578422
ENSG00000143450	OAZ3	3.71269421	0.080939257	3.328678999	0.115085799	7.041373208	2	2	2	4.237680128	3.98554648	3.694366604	3.956354764
ENSG00000115756	HPCAL1	4.020607589	0.001424077	3.017034999	0.020100516	7.037642589	6.96351253	6.464272742	2	10.09449002	10.1587842	9.041010784	9.211159041
ENSG00000102271	KLHL4	3.879625073	0.062735523	3.153677427	0.132250448	7.0333025	2	2	2	4.299492648	4.1930041	3.600766115	3.807156736
ENSG00000137403		12.22300053	2.68E-18	-5.191150116	0.022468449	7.031850415	2	2	2	12.16350377	12.3326784	2	2
ENSG00000082781	ITGB5	3.612813598	1.10E-06	3.417975451	4.18E-06	7.03078905	8.696033109	8.777382925	6.629488387	11.87367064	11.9476934	11.71192115	11.72112623
ENSG00000170214	ADRA1B	0.313586387	0.885376787	6.716363218	1.49E-05	7.029949604	2	3.076665209	2	2.784612485	2.59489879	7.05119671	8.192729549
ENSG00000110987	BCL7A	3.318966262	0.000595251	3.710809158	9.25E-05	7.02977542	3.327711234	4.442710129	2	6.542151749	6.507263	7.045287119	6.741885308
ENSG00000083093	PALB2	3.843314315	0.008808181	3.177735533	0.032897253	7.021049849	2	5.303523747	2	7.565338955	7.53039799	7.054142452	6.728114749
ENSG00000120451	SNX19	3.236269806	0.082560548	3.782791706	0.036346775	7.019061513	5.330594134	8.607278467	2	10.33439225	10.4338094	10.89385283	10.96545847
ENSG00000031691	CENPQ	3.701626997	0.011105632	3.314119944	0.02391519	7.015746941	2	3.076665209	3.369317972	5.839660368	5.49945122	5.661762729	4.886954834
ENSG00000204381	LAYN	3.093650003	0.021356951	3.920490942	0.002561312	7.014140945	6.653098118	7.099070191	2	9.319871109	9.42346554	10.21749687	10.1734218
ENSG00000029153	ARNTL2	3.709477397	0.000536632	3.299600178	0.002254154	7.009077574	5.866944937	3.076665209	6.629488387	9.305820479	9.43201844	9.232285816	8.63228931
ENSG00000163430	FSTL1	2.602805657	2.48E-07	4.406115134	2.14E-19	7.008920792	11.20805444	12.08448117	12.35768921	14.55571806	14.5688082	16.09506566	16.59301128
ENSG00000130720	FIBCD1	2.95495031	0.080067967	4.05342139	0.011575145	7.0083717	2	3.686303414	2	4.946755474	4.95174433	6.430795201	5.20457242
ENSG00000184384	MAML2	3.259035589	0.008961459	3.749098087	0.002132217	7.008133677	6.468621203	6.728495673	2	9.271529914	9.21030762	9.702302705	9.754328277
ENSG00000050405	LIMA1	3.096326461	2.74E-06	3.910200332	1.58E-09	7.006526794	7.159102577	8.356994601	9.031722608	11.43385118	11.4700176	12.44498745	12.0593766
ENSG00000146535	GNA12	2.712172898	0.000860731	4.293117396	3.45E-08	7.005290293	6.257048221	8.717821467	7.777116083	10.56816382	10.6316726	12.20311522	12.15392944

ENSG00000118369	USP35	2.88787372	0.1083703	4.117193529	0.016000913	7.005067249	2	5.946638857	2	7.173892946	7.32163988	8.336833852	8.568035872
ENSG00000107249	GLIS3	3.112243425	0.02449614	3.891652318	0.00367982	7.003895743	3.327711234	5.131603625	7.646393253	9.420668575	9.36392108	9.554115566	10.5978704
ENSG00000180008	SOCS4	3.525681562	0.004196673	3.477936183	0.004611432	7.003617745	3.327711234	6.78762695	4.058998049	9.029678018	8.88733423	9.089196199	8.711989853
ENSG00000136828	RALGPS1	2.100184869	0.167050868	4.903131145	0.000285449	7.003316014	2	3.686303414	2	4.237680128	4.2865223	6.959905775	6.577546999
ENSG00000107263	RAPGEF1	2.858499539	0.063038247	4.143687183	0.004524025	7.002186723	4.00722531	7.669933291	3.369317972	9.021676912	9.12214832	10.42459331	10.27478258
ENSG00000180776	ZDHHC20	3.268715852	0.00011428	3.731851735	7.67E-06	7.000567586	4.467311984	7.099070191	5.772716771	9.404936476	9.35405368	9.851780083	9.829070247
ENSG00000104419	NDRG1	4.174265519	0.006207698	2.822079567	0.074468007	6.996345086	8.138771685	7.051611315	2	11.24377823	11.2751103	9.646549955	10.13436737
ENSG00000134326	CMPK2	9.307518351	4.93E-21	-2.315769499	0.048314828	6.991748852	2	4.442710129	2	12.08902254	12.1387571	2.526562374	2.326754077
ENSG00000123575	FAM199X	2.950947649	0.112649338	4.040763335	0.02294093	6.991710984	3.327711234	7.315436877	2	8.801706047	8.66303256	9.987213818	9.623140479
ENSG00000182742	HOXB4	7.352935807	7.99E-06	-0.361708631	0.872376417	6.991227176	2	2	2	7.405868159	7.42122542	2.384001718	2.117335275
ENSG00000183668	PSG9	1.497853592	0.527022694	5.492304838	0.00562232	6.990158431	2	2	2	2.868578001	2.68924543	4.749835737	6.185873005
ENSG00000187098	MITF	2.518537148	0.003498455	4.469766418	3.49E-08	6.988303566	4.00722531	4.710564154	2	6.147608921	6.30607931	7.975906509	8.254874792
ENSG00000063601	MTMR1	2.927543491	0.151113687	4.059263922	0.036333082	6.986807413	2	6.602424674	2	7.942383523	7.93600799	8.951423836	9.157371148
ENSG00000144827	ABHD10	3.126198895	0.002731351	3.859808163	0.000142658	6.986007058	4.467311984	5.72254323	2	7.68596357	7.72392024	8.676850263	8.126037235
ENSG00000039560	RAI14	3.844135864	1.65E-06	3.141054069	0.00011376	6.985189933	7.031698319	8.540087354	9.541233192	12.52827438	12.5324175	11.80202	11.85327028
ENSG00000134242	PTPN22	5.01168299	0.055895663	1.969540872	0.488789509	6.981223863	2	2	2	5.258265913	5.14724905	3.517841605	2.172608095
ENSG00000135862	LAMC1	2.345118471	0.010398313	4.631907945	5.53E-08	6.977026416	10.18451821	11.29895829	8.561105505	12.72235977	12.7690318	14.9059816	15.14789346
ENSG00000100647	SUSD6	3.818082363	0.038583311	3.158834485	0.090151257	6.976916848	2	8.414402626	4.874587708	10.71715466	10.776052	10.14163246	10.03557113
ENSG00000170525	PFKFB3	3.7704302	0.000130097	3.200219432	0.001343175	6.970649631	6.563806279	6.228547981	8.884815469	11.45882823	11.5018493	10.7250644	11.07615369
ENSG00000109762	SNX25	3.247202842	0.051734578	3.718587895	0.022521058	6.965790737	3.327711234	6.78762695	2	8.504819462	8.54498542	9.01024623	8.973604485
ENSG00000145949	MYLK4	3.244565412	0.134476647	3.719726475	0.075349452	6.964291887	2	2	2	3.840289928	3.69849104	3.993419637	4.241259475
ENSG00000151012	SLC7A11	5.502337118	6.82E-08	1.461265756	0.207497621	6.963602874	5.095959458	7.145017454	8.677268317	13.08794963	13.066109	8.947864566	9.138408485
ENSG00000161921	CXCL16	2.702773711	3.17E-05	4.260471183	9.13E-12	6.963244894	3.327711234	5.303523747	5.156626401	7.307758725	7.46188679	8.831215892	9.00677572
ENSG00000162545	CAMK2N1	2.822195125	0.001659602	4.13421516	1.58E-06	6.956410284	5.330594134	6.464272742	7.934808822	9.694573013	9.77026155	10.700246	11.3162538
ENSG00000204010	IFIT1B	9.663443238	8.84E-11	-2.711962854	0.196453617	6.951480384	2	2	2	9.690973808	9.70259467	2	2.117335275
ENSG00000186635	ARAP1	3.771376435	0.023509926	3.179239271	0.058676271	6.950615706	2	7.901717006	5.156626401	10.21115348	10.3135262	9.771053529	9.568807213
ENSG00000180881	CAPS2	2.89955963	0.188248635	4.044993774	0.051489483	6.944553404	2	2	2	3.615725078	3.45167086	4.588526421	4.133950982
ENSG00000139970	RTN1	0.42852882	0.833848549	6.515220466	1.25E-05	6.943749286	2	3.076665209	2	2.784612485	2.68924543	7.1328795	7.84903967
ENSG00000107796	ACTA2	1.006423158	0.521983587	5.936594945	3.96E-06	6.943018103	8.009475818	9.318399851	4.058998049	9.278261665	9.22120493	14.24028087	14.09820361
ENSG00000125484	GTF3C4	3.22065677	0.059838231	3.722042383	0.025556146	6.942699153	4.00722531	7.35507212	2	9.122382231	9.06060056	9.568866087	9.61099888
ENSG00000187642	PERM1	3.667383715	0.112319087	3.273705736	0.152745431	6.941089451	2	2	2	4.17310023	3.98554648	4.177580368	3.261853426
ENSG00000005238	FAM214B	3.590311237	0.047154938	3.349213213	0.063066737	6.93952445	4.467311984	8.029695761	2	10.10101391	10.1546561	9.740063182	10.02199736
ENSG00000088256	GNA11	2.829133407	0.299070637	4.108806512	0.109843769	6.937939918	2	9.079034257	2	10.29130517	10.3491863	11.6102001	11.58474907
ENSG00000162433	AK4	2.733280611	0.026147826	4.202571659	0.000304705	6.93585227	4.00722531	6.78762695	8.266922072	9.856271709	9.89118718	11.21284883	11.45534619
ENSG00000149657	LSM14B	2.911407748	0.172910901	4.021174525	0.047681033	6.932582274	6.653098118	2	2	7.852449262	8.01099479	9.011762258	9.04984997
ENSG00000213085	CFAP45	3.839333468	0.069491099	3.09092108	0.146121333	6.930254548	2	2	2	4.237680128	4.1930041	3.796405078	3.518055793
ENSG00000171150	SOCS5	3.760373457	0.048272272	3.168314955	0.098094243	6.928688412	4.00722531	8.147241016	2	10.38472097	10.3715902	9.837317946	9.737529735
ENSG00000107317	PTGDS	0.170255443	0.934841508	6.757919971	7.48E-06	6.928175414	2	3.686303414	2	3.227757282	2.68924543	7.613117257	9.139275853
ENSG00000104332	SFRP1	5.324816833	2.53E-09	1.598862978	0.110078372	6.923679811	5.70932728	4.710564154	5.59507254	10.57090794	10.5648023	7.581819208	5.497565112
ENSG00000065600	TMEM206	3.886433455	0.016316848	3.036710244	0.06554535	6.923143699	2	5.595927442	2	7.900612272	7.85436579	7.319501608	6.711881188
ENSG00000123338	NCKAP1L	1.275646484	0.621394099	5.643850455	0.008475154	6.919496939	2	2	2	2.784612485	2.59489879	6.482006365	4.353147976
ENSG00000112561	TFEB	3.146834581	0.013266834	3.770495855	0.002274706	6.917330436	2	4.710564154	2	6.221320894	6.35727082	7.043805932	6.714211481
ENSG00000168016	TRANK1	4.54810141	1.52E-13	2.368813938	0.000230354	6.916915347	5.70932728	7.503546079	6.80318718	11.34480861	11.3855729	9.369567287	8.993406747
ENSG00000008394	MGST1	4.290238632	2.22E-12	2.624208877	3.11E-05	6.914447509	7.384433967	5.72254323	6.43197674	10.93058681	10.8519526	9.033940582	9.406811858
ENSG00000063244	U2AF2	3.409572142	1.93E-08	3.50271027	6.62E-09	6.912282412	7.159102577	5.595927442	7.225742779	10.15739533	10.1807731	10.21979429	10.30292368
ENSG00000173482	PTPRM	2.76955821	0.161589056	4.141818928	0.026558049	6.911377138	2	8.777382925	5.156626401	10.02185637	10.1009593	11.35780343	11.50042979
ENSG00000135837	CEP350	3.577227925	0.011803936	3.331651676	0.019496236	6.908879601	4.00722531	8.077869079	4.523675391	10.19752012	10.2583269	9.942804722	10.02317256
ENSG00000165417	GTF2A1	3.407418064	0.044940119	3.498058642	0.036484987	6.905476706	3.327711234	6.899080544	2	8.89725916	8.67172529	8.920281122	8.835935827
ENSG00000108256	NUFIP2	3.570829643	9.25E-05	3.333740444	0.000271524	6.904570087	7.53297911	8.376385542	5.39244166	11.11674327	11.0725776	11.05976992	10.62382165
ENSG00000125637	PSD4	2.240048484	0.011707809	4.663984205	1.21E-08	6.904032689	3.327711234	4.442710129	3.369317972	5.742861563	5.80356929	7.865372957	8.317521841
ENSG00000203797	DDO	5.923206793	0.009469798	0.979532975	0.719706986	6.902739769	2	2	2	6.069927018	6.00960265	2.937246078	2.117335275
ENSG00000133065	SLC41A1	3.082573895	0.003820004	3.819591558	0.000229589	6.902165453	4.467311984	7.790474954	5.930865648	9.72797373	9.70330142	10.39324012	10.50452546
ENSG00000164930	FZD6	3.6778466	0.112536896	3.222182716	0.162850128	6.900029315	2	7.701025996	2	9.80923777	9.75806852	9.470865405	9.175242287
ENSG00000167114	SLC27A4	2.988478166	0.05824713	3.910722387	0.009837756	6.899200553	3.327711234	6.464272742	2	7.966401501	7.96929529	8.720150661	9.022374596
ENSG00000068366	ACSL4	4.753955195	7.60E-05	2.139511711	0.100111403	6.893466906	4.467311984	8.124485672	9.06622548	12.85250628	12.8630334	10.11402027	10.36930224
ENSG00000148411	NACC2	2.391589617	0.000157675	4.500802437	7.72E-14	6.892392054	5.330594134	6.844429755	7.225742779	9.044420506	8.98240172	11.05398739	11.17229884
ENSG00000259330	INAFM2	1.69392158	0.000341709	5.198085402	1.55E-32	6.892006982	5.866944937	5.72254323	5.156626401	7.190301983	7.28055563	10.78856921	10.61620978
ENSG00000116903	EXOC8	3.649301244	5.22E-07	3.239551458	9.57E-06	6.888852702	3.327711234	4.93638933	5.772716771	8.491639362	8.40429221	8.24055649	7.816873723
ENSG00000143970	ASXL2	3.066552838	1.82E-11	3.820824391	1.85E-17	6.887377229	6.816637977	5.946638857	5.772716771	9.215512556	9.26686067	9.971718548	10.01161415
ENSG00000137693	YAP1	3.486548563	0.000136335	3.400001924	0.000194399	6.886550487	7.53297911	8.414402626	5.39244166	11.048347	11.0193685	10.88447059	11.009855
ENSG00000197102	DYNC1H1	3.221003615	0.004115118	3.656886327	0.00090549	6.877889942	6.563806279	10.46189757	9.589475645	12.75840141	12.8065979	13.11073939	13.31850957
ENSG00000161888	SPC24	3.012579551	0.197226444	3.858294934	0.083094108	6.870874485	2	2	2	3.754611435	3.45167086	3.724264246	4.604965121
ENSG00000181982	CCDC149	3.126727382	0.000167279	3.741782678	4.17E-06	6.86851006	4.467311984	4.93638933	2	7.053486362	7.24992376	7.937338157	7.548321824
ENSG00000100156	SLC16A8	2.275064302	0.297706831	4.580549106	0.019950506	6.855613408	2	2	2	3.227757282	3.08597292	5.002522427	4.624919101
ENSG00000110880	CORO1C	2.440879815	0.045753442	4.413224832	9.96E-05	6.854104647	5.70932728	9.828248506	8.845626171	11.3151886	11.3278511	12.95249272	13.56695364
ENSG00000145723	GIN1	3.720437925	0.02162448	3.132891384	0.055208449	6.853329308	2	3.686303414	2	5.590471342	5.6826301	5.536762811	4.489993931
ENSG00000164849	GPR146	4.727372448	0.002681847	2.125306579	0.213653476	6.852679027	2	3.076665209	2	5.775854763	5.86549842	3.584558147	3.735548302
ENSG00000113319	RASGRF2	4.673604958	0.000556	2.178289618	0.135501339	6.851894576	6.257048221	2	4.058998049	9.463489617	9.50030288	7.134272047	6.903766785
ENSG00000180917	CMTR2	3.864205747	0.034408534	2.98614017	0.10784488	6.850345917	2	6.140560021	2	8.439397922	8.36015869	7.772739333	7.253757517
ENSG00000145365	TIFA	3.361428392	4.80E-05	3.486825349	2.07E-05	6.848253741	3.327711234	4.442710129	3.369317972	6.872128674	6.80463998	6.860764111	7.052989523
ENSG00000203814	HIST2H2BF	6.958915563	8.69E-07	-0.11248077	0.95448384	6.846434792	2	3.076665209	2	7.980622673	7.95986274	2.457042231	2.632958241
ENSG00000157542	KCNJ6	-0.724548903	0.752249153	7.568088807	1.07E-06	6.843539905	2	2	2	2.270786764	2.14022021	7.846806418	7.360260221
ENSG00000162542	TMCO4	2.640238434	0.00087139	4.199848698	2.70E-08	6.840087132	2	4.93638933	4.523675391	6.670510205	6.72335419	8.261097876	8.176765766
ENSG00000213906	LTB4R2	4.371457102	0.013378187	2.465704348	0.185599465	6.83716145	2	3.076665209	2	5.48800675	5.49945122	4.281782022	3.383972333
ENSG00000101639	CEP192	3.540152727	0.001471851	3.296048447	0.003154055	6.836201174	2	5.946638857	4.523675391	8.297027403	8.28283292	8.218405973	7.8574975
ENSG00000166971	AKTIP	2.844274831	0.003720972	3.988274141	2.23E-05	6.832548972	3.327711234	4.93638933	2	6.470063213	6.53293476	7.697491716	7.517964974
ENSG00000173480	ZNF417	4.439550672	0.000613741	2.38913565	0.083245787	6.828686322	2	5.838937808	3.369317972	8.852852814	8.81724322	7.131485609	6.432241896
ENSG00000109458	GAB1	2.041104352	0.237601833	4.786561489	0.002077008	6.827665841	3.327711234	6.728495673	2	7.292639438	7.27676203	9.796563052	10.1753259
ENSG00000153233	PTPRR	4.457289799	0.028243263	2.370140214	0.270737833	6.827430013	2	2	2	4.670864177	4.76938658	2.987421008	3.407202941
ENSG00000146067	FAM193B	2.969153569	0.000902883	3.856769236	8.89E-06	6.825922806	3.327711234	6.228547981	5.156626401	8.211183901	8.21506984	9.317805994	8.823577951
ENSG00000176853	FAM91A1	3.460337303	0.008386015	3.363562987	0.010235235	6.82390029	2	6.951736442	5.930865648	9.383689875	9.34954609	9.369567287	9.16420533
ENSG00000087494	PTHLH	4.431349699	0.000240382	2.389591042	0.063104764	6.820940741	3.327711234	3.686303414	4.058998049	7.638916932	7.66709116	4.350488971	6.38329369
ENSG00000132932	ATP8A2	1.274445776	0.562605288	5.545208786	0.00218059	6.819654562	2	2	2	2.695456494	2.68924543	5.258920789	6.018717758
ENSG00000021300	PLEKHB1	1.012622956	0.641177228	5.805608199	0.000743299	6.818231155	2	2	2	2.695456494	2.49394783	5.805044075	6.044874254
ENSG00000150594	ADRA2A	0.857221333	0.727489842	5.955775681	0.002130827	6.812997014	2	2	2	2.3892346	2.68924543	5.185595313	6.61544141
ENSG00000136802	LRRC8A	3.523261794	0.002887069	3.287501004	0.005604071	6.810762798	7.031698319	7.73146271	3.369317972	10.29652513	10.4389106	10.22028611	10.04323752
ENSG00000138286	FAM149B1	3.233619719	0.001152009	3.577093105	0.000257739	6.810712824	4.00722531	5.457120953	2	7.514115926	7.4450836	7.926930284	7.697107799
ENSG00000198315	ZKSCAN8	3.561212774	0.010243646	3.247311935	0.020011794	6.808524709	4.467311984	7.538394143	3.369317972	9.697445926	9.6919516	9.558789939	9.181571564
ENSG00000205339	IPO7	3.458259319	1.07E-08	3.348657202	2.95E-08	6.806916522	6.009025997	7.572420273	7.691296561	10.69684063	10.689886	10.70388473	10.45330786
ENSG00000114978	MOB1A	3.737792873	2.98E-14	3.067600753	5.88E-10	6.805393626	7.485143553	7.638155693	6.322058544	10.9941771	10.9493026	10.36356703	10.24063603
ENSG00000090857	PDPR	3.498793147	0.005182914	3.305321883	0.008424324	6.80411503	4.467311984	7.503546079	4.058998049	9.58946437	9.70471389	9.482119335	9.428971287
ENSG00000179104	TMTC2	3.248234319	0.011460822	3.55440453	0.00485155	6.802638849	2	4.442710129	2	6.283823264	5.89549381	6.184218099	6.574984961
ENSG00000282214		3.525985164	0.024581743	3.275325487	0.036849285	6.801310652	2	6.951736442	4.058998049	8.960808841	9.09547637	8.906900259	8.643336952
ENSG00000166578	IQCD	1.863568792	0.459530549	4.934330364	0.024930503	6.797899156	2	2	2	3.289987342	2.49394783	4.659878161	5.492143571
ENSG00000162004	CCDC78	2.390511696	0.325100102	4.407196375	0.047258258	6.797708071	2	2	2	3.289987342	3.15372745	5.19095825	3.875378318
ENSG00000129675	ARHGEF6	2.590408802	0.016876187	4.205187545	3.81E-05	6.795596347	2	5.838937808	4.523675391	7.300218887	7.25764291	8.834646275	8.901818877
ENSG00000103257	SLC7A5	2.712990547	6.19E-05	4.077956508	4.63E-10	6.790947054	8.138771685	7.431218269	6.629488387	10.18988999	10.2374972	12.01230258	10.94712477
ENSG00000102218	RP2	3.489169917	9.51E-08	3.30030614	4.50E-07	6.789476057	5.532353208	6.04685525	4.058998049	8.813671111	8.85589616	8.403026507	8.857178832
ENSG00000143473	KCNH1	4.54717839	0.020944825	2.241167181	0.286960302	6.788345571	2	2	2	4.76150579	4.83276841	3.256555576	3.012034821
ENSG00000131781	FMO5	2.645912701	0.252177378	4.141965527	0.053922041	6.787878228	2	2	2	3.615725078	3.08597292	4.675266036	4.201948172
ENSG00000156113	KCNMA1	3.418038313	0.012001607	3.365839001	0.013077994	6.783877314	3.327711234	3.686303414	6.958202216	8.870779703	8.93384535	8.769824912	8.927653397
ENSG00000197632	SERPINB2	11.97408823	1.09E-17	-5.191150361	0.022295807	6.782937869	2	2	2	12.0000521	12.0037045	2	2
ENSG00000163171	CDC42EP3	2.435872328	0.123290993	4.345997728	0.002975696	6.781870057	8.836089686	5.303523747	4.058998049	9.836208668	9.81083239	11.74784286	11.70993099
ENSG00000109381	ELF2	3.247481578	0.004804831	3.534262262	0.001845662	6.78174384	3.327711234	6.140560021	3.369317972	8.013270196	8.09094333	8.513629334	8.132149972
ENSG00000149781	FERMT3	2.97391488	0.089940832	3.806427063	0.023485564	6.780341943	2	3.686303414	2	5.003571641	4.91316568	6.237020416	4.861951484
ENSG00000196865	NHLRC2	2.797403188	2.53E-05	3.976683057	6.49E-10	6.774086246	3.327711234	5.595927442	5.39244166	7.751013478	7.7377845	8.874770504	8.94164277
ENSG00000076641	PAG1	2.816975801	1.22E-05	3.953900508	2.46E-10	6.770876308	4.815609471	4.11352228	5.59507254	7.527093191	7.58663873	8.841908869	8.491306993

ENSG00000196337	CGB7	4.063065477	0.070101138	2.706345289	0.240957826	6.769410766	2	2	2	4.17310023	4.58534546	3.724264246	3.012034821
ENSG00000120756	PLS1	1.187115691	0.524942496	5.582172558	0.000240832	6.769288249	2	3.076665209	2	3.09461492	3.08597292	6.382394503	6.841176624
ENSG00000141337	ARSG	3.847945381	5.10E-07	2.920136689	0.000187368	6.76808207	2	4.93638933	4.874587708	7.90310317	8.14604733	7.113241388	7.130061054
ENSG00000162078	ZG16B	1.014024556	0.665819919	5.753120077	0.002140974	6.767144633	2	2	2	2.784612485	2.38539787	5.472156123	6.199243628
ENSG00000110395	CBL	3.245470639	0.022825231	3.520694458	0.012183874	6.766165097	5.095959458	7.928237109	3.369317972	9.848538434	9.73406074	10.274494	9.823414835
ENSG00000152952	PLOD2	4.642233504	0.000222301	2.119058652	0.119536754	6.761292156	5.330594134	10.16572116	9.212024683	13.87521525	13.8218999	10.98378895	11.60482568
ENSG00000170577	SIX2	-1.313350828	0.590651191	8.073728458	8.10E-07	6.760377631	2	2	2	2	2.2680111	8.249579355	7.979721237
ENSG00000146676	PURB	3.250361919	4.36E-08	3.508925295	2.54E-09	6.759287215	5.866944937	7.315436877	6.073378139	9.80923777	9.78034427	10.02879997	10.0755426
ENSG00000068781	STON1-GTF2	6.617191483	0.000337602	0.141684102	0.954947148	6.758875585	2	2	2	6.699459234	6.70088158	2.139668783	2.551896972
ENSG00000149582	TMEM25	2.192398011	0.231366574	4.565986976	0.006248071	6.758384987	2	5.946638857	2	6.573765157	6.58294721	8.908528807	8.896185552
ENSG00000135932	CAB39	3.602908284	0.016039408	3.155417988	0.036346775	6.758326272	6.468621203	7.819099893	2	10.30314152	10.2807554	9.905980863	9.783748583
ENSG00000185742	C11orf87	2.494629492	0.303181423	4.25763593	0.055927555	6.752265422	2	2	2	3.023133209	3.50452879	3.956110469	4.975095763
ENSG00000155962	CLIC2	2.975080278	0.221769007	3.774079409	0.104457634	6.749159687	2	2	2	3.615725078	3.55551834	4.628601098	3.474731315
ENSG00000127743	IL17B	2.774647091	0.248162973	3.973560306	0.078790818	6.748207396	2	2	2	3.162721474	3.69849104	4.690491514	3.824516949
ENSG00000136153	LMO7	2.532852309	0.039713176	4.213415107	0.000259014	6.746267417	6.138360429	9.357954881	10.22252928	11.85111104	11.8451168	12.95483431	13.93649465
ENSG00000196700	ZNF512B	2.878532033	0.015001375	3.867711618	0.000670543	6.746243651	6.366709671	6.311476754	2	8.551612617	8.62623826	9.45200461	9.681170767
ENSG00000115306	SPTBN1	2.989734532	0.000905307	3.750551898	1.92E-05	6.74028643	7.750913381	10.48441188	9.516493218	12.58764526	12.6467792	13.03616105	13.65407799
ENSG00000079156	OSBPL6	4.465254108	0.003554578	2.271707022	0.165246475	6.736961129	2	3.076665209	4.058998049	6.979883891	6.99214046	5.634468599	3.580696614
ENSG00000066739	ATG2B	3.466700034	0.057486219	3.270199934	0.071384845	6.736899969	2	7.315436877	3.369317972	9.264766505	9.23104065	9.02571051	9.079525723
ENSG00000060237	WNK1	3.097848028	3.12E-22	3.629892467	1.37E-30	6.727740495	8.257427002	8.557180776	8.143093325	11.39544505	11.4429793	12.11712511	11.76273823
ENSG00000047457	CP	2.97629186	0.2361363	3.751091213	0.117977654	6.727383074	2	2	2	3.754611435	3.39680241	4.652122235	3.36036153
ENSG00000171862	PTEN	3.199783026	0.000645674	3.526733398	0.000136151	6.726516424	6.653098118	7.847167909	4.874587708	10.12521908	10.0345549	10.65148032	10.11112449
ENSG00000121904	CSMD2	3.658402043	0.001197467	3.064962093	0.007494621	6.723364136	3.327711234	2	4.523675391	6.610808945	6.67805337	6.351597515	5.745545708
ENSG00000110429	FBXO3	2.902121863	0.052698164	3.820702689	0.007862246	6.722824552	2	6.228547981	3.369317972	7.708923796	7.6318872	8.599312524	8.552492262
ENSG00000119227	PIGZ	3.042423922	0.060267353	3.680206608	0.019086058	6.72263053	2	5.457120953	2	6.872128674	6.95938211	7.245405178	7.779530392
ENSG00000186994	KANK3	1.690389006	0.472807027	5.031838986	0.012952329	6.722227992	2	2	2	2.947924294	2.77779907	5.680947153	4.543391831
ENSG00000136141	LRCH1	3.60863038	0.003020234	3.11309842	0.011549101	6.7217288	2	6.464272742	5.156626401	8.924487942	8.92538942	8.601327805	8.245238611
ENSG00000147164	SNX12	3.025142263	0.117406202	3.693535934	0.047554519	6.718678197	3.327711234	7.572420273	2	9.04103174	9.06939887	9.512957081	9.900385864
ENSG00000270898	GPR75-ASB3	7.882161789	6.95E-08	-1.16446949	0.545860427	6.717692299	2	2	2	7.786523606	8.06478301	2.139668783	2.172608095
ENSG00000176871	WSB2	3.041989234	0.000769315	3.673207505	3.22E-05	6.715196739	5.532353208	8.276714465	7.029880454	10.37891658	10.3514424	10.69895264	11.24037286
ENSG00000155287	SLC25A28	3.913086838	0.002673268	2.794827598	0.037844164	6.707914436	2	4.93638933	6.718950348	9.29542901	9.35044873	8.389095798	8.017950573
ENSG00000240184	PCDHGC3	3.776647898	0.038521438	2.925324421	0.114540794	6.701972319	4.467311984	8.101365653	2	10.35637181	10.4049916	9.216266648	9.792596779
ENSG00000171608	PIK3CD	2.177922735	0.000866952	4.522925131	1.74E-13	6.700847866	5.532353208	5.303523747	6.073378139	7.656739692	7.8208909	9.477737996	10.48025681
ENSG00000139618	BRCA2	5.045888383	6.66E-06	1.654950995	0.187386354	6.700839378	2	4.11352228	2	7.536750104	7.42465798	4.406883583	4.329022995
ENSG00000187987	ZSCAN23	3.561260315	0.037885253	3.138588207	0.067466122	6.699848522	2	3.076665209	2	4.887609717	4.65713986	4.425202323	4.4231929
ENSG00000171827	ZNF570	3.174923644	0.00305872	3.522953655	0.000823875	6.697877299	3.327711234	5.131603625	2	6.993973425	6.95938211	7.519242774	7.076727469
ENSG00000082512	TRAF5	2.531958139	0.238863041	4.165477204	0.038278239	6.697435343	2	6.844429755	2	7.694616532	7.88209639	9.479108595	9.331400327
ENSG00000148541	FAM13C	3.283629631	0.150709752	3.413625362	0.125922464	6.697254993	2	3.076665209	2	4.739373666	4.31639476	5.381251181	3.100202476
ENSG00000205177	C11orf91	5.978747771	0.000264903	0.71698913	0.726496359	6.695736901	2	2	2	5.968925393	6.20590375	2.457042231	2.551896972
ENSG00000165029	ABCA1	4.717553116	8.87E-30	1.977023143	4.95E-06	6.694576259	7.624139768	6.899080544	6.629488387	11.76848464	11.8038653	8.98885284	9.118313725
ENSG00000140464	PML	3.618641366	4.34E-07	3.074961611	2.12E-05	6.693602977	8.493334308	8.414402626	6.43197674	11.59966248	11.6791849	11.04836626	11.14427326
ENSG00000010327	STAB1	1.274445254	0.558024285	5.416869472	0.002481102	6.691314726	2	2	2	2.695456494	2.68924543	5.328699035	5.777095613
ENSG00000160886	LY6K	3.993352998	0.074741742	2.696784584	0.240873009	6.690137582	2	2	2	4.358765249	4.31639476	3.011868911	3.717076715
ENSG00000197496	SLC2A10	2.940715106	0.006444915	3.748610898	0.000334778	6.689326004	4.00722531	7.60566235	6.203069917	9.452477833	9.49050967	10.2641958	10.28774876
ENSG00000073712	FERMT2	2.245825321	1.11E-14	4.441770381	6.36E-56	6.687595703	8.009475818	8.317412869	7.858115942	10.28272246	10.3455691	12.49041464	12.5231767
ENSG00000171388	APLN	1.815555109	0.312172567	4.865662446	0.001918418	6.681217555	3.327711234	2	2	3.515101962	3.39680241	6.043083161	5.85096143
ENSG00000155393	HEATR3	3.398616747	0.038177023	3.282060146	0.043831981	6.680676893	2	5.595927442	2	7.501020868	7.29187696	7.329261848	7.242500282
ENSG00000196605	ZNF846	3.307271811	0.012174173	3.369215573	0.009915802	6.676487383	2	4.11352228	2	5.697663076	5.88556448	6.043083161	5.631815919
ENSG00000123143	PKN1	2.766440651	0.162667495	3.908272456	0.037527109	6.674713107	5.095959458	8.791896851	2	10.08190282	10.054079	11.24438305	11.16848011
ENSG00000180758	GPR157	2.026650048	0.174256431	4.645967717	0.000501618	6.672617765	2	2	4.874587708	5.128039203	5.19627118	7.87872738	7.354296226
ENSG00000216490	IFI30	9.031942134	2.43E-15	-2.36353864	0.083660985	6.668403494	2	4.11352228	2	11.40799569	11.4431908	2.457042231	2.172608095
ENSG00000165731	RET	2.467990535	0.104145312	4.19954176	0.002768615	6.667532295	2	2	4.058998049	4.694064037	4.56059747	6.231826305	6.141542327
ENSG00000169057	MECP2	3.067822962	0.051305942	3.598649426	0.018882709	6.666472389	8.107517749	7.051611315	2	10.09829921	10.1695649	10.61429194	10.7112881
ENSG00000103260	METRN	2.737151656	0.034673548	3.928078106	0.001437419	6.665229762	4.467311984	6.311476754	2	7.694616532	7.84926609	9.163708059	8.699106494
ENSG00000158683	PKD1L1	2.441806182	0.307380329	4.220088375	0.054660707	6.661894557	2	2	2	2.947924294	3.50452879	4.82764224	4.133950982
ENSG00000171206	TRIM8	3.330764778	0.000953251	3.326387523	0.000915639	6.657152301	7.27616306	8.505280662	5.156626401	10.78523872	10.894967	10.69871735	10.9627661
ENSG00000180543	TSPYL5	2.824209316	0.177920442	3.832317682	0.054838286	6.656526999	2	6.728495673	2	7.959237999	7.96694292	8.958123037	8.960904642
ENSG00000110427	KIAA1549L	3.602372588	0.022954373	3.050300876	0.056438674	6.652673465	2	3.686303414	6.80318718	8.872051712	8.87860041	8.319800025	8.338065764
ENSG00000108021	FAM208B	3.211361392	0.017806575	3.44034869	0.010049511	6.651710083	6.366709671	8.213429689	3.369317972	10.19853443	10.2084185	10.63608101	10.19360379
ENSG00000009335	UBE3C	2.904296539	0.025535393	3.742108727	0.002868555	6.646405266	3.327711234	8.053983481	7.502634626	10.14060551	10.1148377	10.87043852	11.0505632
ENSG00000183060	LYSMD4	2.414977476	0.076743239	4.231387166	0.00080315	6.646364642	2	4.93638933	2	5.775854763	5.87556634	7.613117257	7.521960976
ENSG00000130487	KLHDC7B	2.897841727	0.359800435	3.747421414	0.214267271	6.645263141	2	2	2	3.406930997	3.65239003	4.914492341	2.374681325
ENSG00000151532	VTI1A	3.560133845	3.15E-09	3.085093078	3.32E-07	6.645226923	4.815609471	5.595927442	4.058998049	8.365459376	8.44204342	7.829717863	8.034828728
ENSG00000031003	FAM13B	4.564411499	7.51E-08	2.080333391	0.023172228	6.64474489	3.327711234	5.838937808	4.058998049	9.217515344	9.26207178	6.870823472	6.723495198
ENSG00000244165	P2RY11	3.772990929	0.002468582	2.870165375	0.024934862	6.643156304	2	4.710564154	2	7.021746293	6.74548212	6.028179536	6.029985803
ENSG00000139514	SLC7A1	2.205045471	0.277947155	4.436572097	0.017129277	6.641617568	4.815609471	8.655694736	2	9.360314042	9.36392108	11.43892588	11.7207641
ENSG00000116032	GRIN3B	3.733626046	0.111579909	2.902094201	0.219716772	6.635718247	2	2	2	4.105493497	4.16043394	3.904814898	3.042026731
ENSG00000149187	CELF1	3.771086599	0.010057832	2.862911878	0.056113002	6.633998477	2	7.701025996	6.718950348	10.42167359	10.4832045	9.592360305	9.502606422
ENSG00000137463	MGARP	2.287334733	0.095959724	4.34657288	0.000521666	6.633907613	2	4.11352228	2	4.76150579	4.98931816	6.505870593	7.013798886
ENSG00000065613	SLK	3.378085042	3.31E-07	3.254596603	8.58E-07	6.632681644	5.532353208	7.467835385	7.098165388	10.25325094	10.2564019	10.31004663	9.928254593
ENSG00000075426	FOSL2	2.182747727	2.06E-11	4.449342492	3.22E-45	6.632090219	7.485143553	7.819099893	8.143093325	9.966365455	10.0345549	12.29329414	12.23203189
ENSG00000145214	DGKQ	3.036216329	0.019509132	3.594834547	0.004534024	6.631050876	4.467311984	6.389896633	2	8.076429088	8.185025	8.801285298	8.555744353
ENSG00000108582	CPD	2.930468851	0.000719561	3.699658679	1.17E-05	6.63012753	6.138360429	8.834582582	8.35328246	11.06484891	11.075856	11.86743185	11.80885057
ENSG00000174405	LIG4	3.194471367	0.099101291	3.435348976	0.06941259	6.629820344	2	6.311476754	2	7.885575888	7.93600799	8.260460366	8.026414333
ENSG00000072786	STK10	3.358093793	2.52E-05	3.270795573	3.91E-05	6.628889367	6.816637977	6.228547981	4.058998049	9.323594948	9.43031191	9.330617798	9.250624924
ENSG00000185972	CCIN	3.691457431	0.103405461	2.932426618	0.197522413	6.623884049	2	2	2	4.358765249	3.7864959	3.355392153	3.735548302
ENSG00000096968	JAK2	3.983114804	0.009351711	2.637033617	0.097308454	6.620148421	2	6.464272742	3.369317972	8.965583267	8.94225201	7.851893762	7.357281305
ENSG00000091157	WDR7	2.931868387	0.148258746	3.68649736	0.058167426	6.618365747	2	6.535001748	2	7.810605345	7.93841129	8.61485781	8.626114978
ENSG00000230413		11.80880312	3.88E-17	-5.191150432	0.02246952	6.617652689	2	2	2	11.78512278	11.8866222	2	2
ENSG00000127663	KDM4B	2.573404422	0.045544105	4.04223205	0.000846027	6.615636472	3.327711234	7.761270542	6.203069917	9.186155283	9.18523054	10.69471167	10.60007717
ENSG00000103056	SMPD3	0.686380815	0.777377621	5.925474024	0.001442563	6.61185484	2	2	2	2.270786764	2.68924543	6.249924465	5.799216178
ENSG00000139192	TAPBPL	4.203312448	0.000588482	2.406591689	0.062792893	6.609904137	3.327711234	5.72254323	2	8.48998335	8.50338773	6.651077397	6.821857532
ENSG00000065923	SLC9A7	2.824031389	4.92E-09	3.777637803	1.47E-15	6.601669192	6.816637977	7.669933291	6.534109959	9.924066823	9.8787275	10.91253766	10.78933969
ENSG00000197386	HTT	2.941630093	0.027016433	3.657961923	0.004541723	6.599592016	3.327711234	8.053983481	6.43197674	9.768836458	9.88060333	10.39918908	10.66761654
ENSG00000129911	KLF16	2.906850529	0.001810688	3.687445914	4.57E-05	6.594296442	3.327711234	5.457120953	6.534109959	8.36365191	8.43865202	9.002642099	9.32912155
ENSG00000282107		4.790482846	0.000862526	1.80327906	0.256970059	6.593761907	2	3.686303414	2	6.628980152	6.6952083	3.679182183	4.30448772
ENSG00000258484	SPESP1	1.861070434	0.456251283	4.730173802	0.030900875	6.591244236	2	2	2	3.09461492	2.77779907	5.48093935	4.119958326
ENSG00000198924	DCLRE1A	2.707015332	0.037504648	3.883870881	0.001646167	6.590886213	2	4.442710129	2	5.602782483	5.60003091	6.931352719	6.449128733
ENSG00000259030	FPGT-TNNI3	3.936767379	0.087461944	2.651622658	0.260827638	6.588390037	2	2	2	4.205751501	4.37434574	2.937246078	3.717076715
ENSG00000118518	RNF146	3.531845266	0.018380127	3.055784173	0.043002955	6.587629439	4.00722531	6.728495673	2	8.839910123	8.81854848	8.557366484	8.126912071
ENSG00000188611	ASAH2	4.984858058	0.002812393	1.599491673	0.393071188	6.584349732	2	2	3.369317972	6.034011955	6.08962627	3.616794016	2.950113106
ENSG00000115839	RAB3GAP1	3.101889655	0.01740322	3.481017815	0.006513019	6.58290747	6.89194353	8.169643011	3.369317972	10.18529247	10.2014316	10.52938802	10.61215446
ENSG00000082684	SEMA5B	1.867348823	0.662327642	4.71441858	0.219692889	6.581767402	2	2	2	3.515101962	2	5.002522427	4.878668439
ENSG00000081189	MEF2C	3.482524503	0.002014131	3.09794356	0.006424701	6.580468064	2	3.686303414	4.523675391	6.820355514	6.64311914	6.818078339	5.703940561
ENSG00000154529	CNTNAP3B	3.047982349	0.182152175	3.531665905	0.108113626	6.579648254	2	2	2	3.754611435	3.50452879	4.133696865	3.807156736
ENSG00000132109	TRIM21	4.349012075	0.000227241	2.227065528	0.077869736	6.576077603	6.73718411	6.140560021	2	10.19039992	10.1670053	8.041200979	8.104880443
ENSG00000047056	WDR37	3.495827057	0.086812219	3.079249981	0.129694026	6.575122038	2	6.666836837	2	8.554784469	8.57309694	7.937338157	8.339575961
ENSG00000137393	RNF144B	3.041618323	0.056953525	3.532976221	0.023237554	6.574594544	6.257048221	2	3.369317972	7.805288479	7.71553728	8.138086022	8.344097092
ENSG00000221843	C2orf16	1.310980885	0.492404429	5.263297584	0.000926448	6.574278469	2	2	3.369317972	2.947924294	3.33976443	6.533574091	6.081436739
ENSG00000178209	PLEC	2.875426082	1.73E-18	3.693616396	2.94E-30	6.569042478	10.25038534	9.962611189	9.778213818	12.86261997	12.8987213	13.45045629	13.91034311
ENSG00000150093	ITGB1	3.735732169	8.91E-12	2.827256813	3.54E-07	6.562988982	10.68025843	12.130888	11.78368112	15.38856894	15.3839076	14.52891921	14.42496738
ENSG00000114166	KAT2B	2.79762879	0.004686679	3.765167133	7.79E-05	6.562795923	2	5.595927442	4.523675391	7.300218887	7.30683517	8.152588976	8.347103327
ENSG00000204103	MAFB	3.669345828	0.021398494	2.892571815	0.074588837	6.561917642	2	4.93638933	2	7.062428156	6.90635305	6.795372357	5.331426166
ENSG00000068383	INPP5A	2.589486662	0.017388264	3.970246511	0.000118328	6.559733174	2	6.04685525	5.39244166	7.77569021	7.64076905	8.950238388	9.187034567
ENSG00000151468	CCDC3	-1.970914457	0.114510897	8.528194679	1.32E-17	6.557280222	4.467311984	3.076665209	4.058998049	2.947924294	2.59489879	10.99953788	12.6187847
ENSG00000222047	C10orf55	3.974263736	0.062077767	2.582001897	0.240345235	6.556265633	2	2	2	4.237680128	4.40247105	3.235948948	3.430065404
ENSG00000011422	PLAUR	4.572034112	3.24E-05	1.982268736	0.097369201	6.554302848	4.00722531	7.847167909	7.818184403	11.79956717	11.8745935	9.01781049	9.459850232

ENSG00000179922	ZNF784	2.674345306	0.035422866	3.874474966	0.001290688	6.548820271	2	4.442710129	2	5.420062284	5.71663161	6.693258166	6.718860807
ENSG00000160813	PPP1R35	3.447295308	0.000334546	3.098520648	0.00134027	6.545815956	2	4.442710129	3.369317972	6.604700657	6.6952083	6.305369896	6.329589667
ENSG00000005249	PRKAR2B	5.198849104	4.16E-05	1.346771376	0.356359551	6.54562048	3.327711234	3.076665209	2	7.19843704	6.99676009	3.679182183	3.771796761
ENSG00000109927	TECTA	4.595296216	0.020435724	1.949451494	0.364963349	6.54474771	2	2	2	4.670864177	4.98931816	3.082820997	2.885414003
ENSG00000119397	CNTRL	4.109430629	0.00103094	2.434829009	0.064928899	6.544259638	2	4.11352228	2	6.592405944	6.52015598	5.185595313	4.810605986
ENSG00000167925	GHDC	3.316691099	0.002846809	3.227383735	0.003624465	6.544074834	3.327711234	6.04685525	3.369317972	7.999368766	8.08661608	8.063300251	7.839464964
ENSG00000099814	CEP170B	2.927409656	0.059854337	3.615517188	0.016349295	6.542926844	2	7.393647515	7.858115942	9.938102197	9.99006945	10.72275211	10.57385172
ENSG00000144843	ADPRH	2.3555514	0.077127293	4.180936791	0.000676414	6.536488191	2	4.710564154	2	5.44762523	5.64780787	7.08038692	7.464983909
ENSG00000168490	PHYHIP	3.213038073	0.161654871	3.317359814	0.137443915	6.530397887	2	2	2	3.754611435	3.74316441	4.029788235	3.580696614
ENSG00000111886	GABRR2	4.132322139	0.061450217	2.395664499	0.298391409	6.527986638	2	2	2	4.443344104	4.45712836	3.500668998	2.885414003
ENSG00000126461	SCAF1	2.692668961	0.045443019	3.827825096	0.002799381	6.520494057	7.53297911	8.297207175	3.369317972	10.10372352	10.0551867	11.1203133	11.29801859
ENSG00000122547	EEPD1	2.546703445	0.105084134	3.966504052	0.006924386	6.513207496	2	3.686303414	2	4.598938466	4.63360314	6.106889539	5.611957759
ENSG00000139200	PIANP	1.864214691	0.164314918	4.645670045	9.38E-05	6.509884736	2	4.710564154	2	5.22679461	4.98931816	7.510690356	7.945418846
ENSG00000196196	HRCT1	1.935477082	0.397539867	4.571354566	0.023994345	6.506831648	2	2	2	2.784612485	3.15372745	4.834511823	4.80186789
ENSG00000183793	NPIPA5	10.24908125	2.86E-13	-3.743754742	0.103805123	6.505326512	2	2	2	10.28749693	10.2726831	2	2.059860178
ENSG00000156599	ZDHHC5	3.246118577	0.070433128	3.258055917	0.065664664	6.504174494	8.737455672	6.140560021	2	10.56934051	10.6264653	10.5520967	10.66581178
ENSG00000079308	TNS1	2.936134843	0.012711443	3.567302274	0.001852113	6.503437116	9.579430841	9.574900212	5.772716771	11.94460653	11.996947	12.92076824	12.19131528
ENSG00000231094		5.418222878	0.007764718	1.084805896	0.651103372	6.503028775	2	2	2	5.709096143	5.41912973	2.3070652	2.885414003
ENSG00000101752	MIB1	2.859316204	0.241959192	3.637628268	0.120921014	6.496944472	2	7.901717006	2	9.239364442	9.09977716	10.22911045	9.591800868
ENSG00000223802	CERS1	4.311902383	0.008465185	2.184190768	0.211685601	6.496093151	2	3.076665209	2	5.319220934	5.55061739	3.724264246	3.67940853
ENSG00000165059	PRKACG	3.244232234	0.150422971	3.250323815	0.140213693	6.494556049	2	2	2	3.79808662	3.74316441	3.724264246	3.824516949
ENSG00000120549	KIAA1217	2.95965392	0.156702886	3.527912322	0.080230773	6.487566241	2	3.686303414	8.236946975	9.652259787	9.62041512	9.510546344	10.66791712
ENSG00000175395	ZNF25	2.547219261	0.121156062	3.939168201	0.010890162	6.486387463	2	6.464272742	3.369317972	7.562190249	7.51115308	9.249414741	8.463873348
ENSG00000174080	CTSF	3.1720076	0.044825118	3.311940909	0.033432537	6.483948508	7.624139768	5.303523747	2	9.439666599	9.42003014	9.581426916	9.556522292
ENSG00000173040	EVC2	3.530914144	0.000680216	2.94989298	0.005175579	6.480807124	4.00722531	2	4.874587708	7.136275664	7.07311172	6.807643042	6.222348126
ENSG00000278211		6.082413083	0.000191933	0.397961587	0.852566533	6.48037467	2	2	2	6.29903474	6.0722229	2.457042231	2.374681325
ENSG00000123908	AGO2	2.234311756	0.048417421	4.244543405	4.85E-05	6.478855161	7.27616306	6.602424674	3.369317972	8.627383409	8.64695856	10.75059845	10.51258091
ENSG00000163083	INHBB	4.766464661	0.015772075	1.710501748	0.434458206	6.476966409	2	2	2	5.022023783	4.95174433	2.68698273	3.042026731
ENSG00000188707	ZBED6CL	5.646742537	0.000406306	0.829967377	0.670620849	6.476709914	3.327711234	2	2	6.733448311	6.64900057	2.62493832	3.15612297
ENSG00000225967		11.66763464	8.91E-17	-5.191150548	0.022456739	6.476484095	2	2	2	11.68717402	11.7042919	2	2
ENSG00000141068	KSR1	1.683164237	0.334772114	4.792832494	0.001787956	6.475996731	2	5.595927442	2	5.742861563	5.78232107	9.048785116	8.43731913
ENSG00000112787	FBRSL1	2.803029538	0.019331325	3.672512249	0.001455406	6.475541786	2	6.311476754	5.156626401	7.961629787	8.0713677	9.004546892	8.736843548
ENSG00000205129	C4orf47	2.937815303	0.198526732	3.533627547	0.106686868	6.47144285	2	2	2	3.615725078	3.50452879	4.065262523	3.8919413
ENSG00000156313	RPGR	4.260248306	0.000823063	2.207702812	0.104869869	6.467951118	2	4.11352228	2	6.682159616	6.71216164	4.521997428	5.050803619
ENSG00000152127	MGAT5	2.816047145	0.248162973	3.651855693	0.117745175	6.467902838	2	7.901717006	2	9.081180848	9.17405701	9.964103831	9.955102659
ENSG00000091527	CDV3	2.817176336	0.105584609	3.648517957	0.029020898	6.465694293	7.53297911	8.820493869	2	10.51174802	10.5632459	11.18758515	11.52718335
ENSG00000166340	TPP1	3.507600157	0.00677234	2.954123461	0.024616215	6.461723617	8.075571721	8.94259736	4.058998049	11.5005475	11.5214183	10.82922498	11.0770593
ENSG00000038945	MSR1	3.976615816	0.007764718	2.484692149	0.111079115	6.461307965	5.532353208	2	5.930865648	8.993902695	9.0282492	8.291371768	5.876154317
ENSG00000120647	CCDC77	3.702815615	0.004961216	2.758187184	0.041666835	6.461002799	2	4.710564154	2	6.846474326	6.77805036	6.167993056	5.606950214
ENSG00000182158	CREB3L2	3.056512833	0.03505329	3.395395386	0.017054345	6.45190822	7.940205782	9.566483142	4.058998049	11.42737655	11.4826289	11.84623902	11.73918884
ENSG00000260314	MRC1	1.860225968	0.429847898	4.589651478	0.02674158	6.449877445	2	2	2	3.023133209	2.86123014	5.180212365	4.376876162
ENSG00000175445	LPL	1.320572128	0.714968044	5.126937466	0.100920521	6.447509594	3.327711234	2	2	3.79808662	2	7.05119671	3.807156736
ENSG00000138379	MSTN	0.855699976	0.734406956	5.591317167	0.005313728	6.447017143	2	2	2	2.270786764	2.77779907	5.376552034	6.014942101
ENSG00000196116	TDRD7	4.075609328	0.063652316	2.36973438	0.300233814	6.445343708	2	2	7.098165388	9.523810814	9.5600488	7.770054437	7.932467322
ENSG00000205426	KRT81	3.332378729	0.167819097	3.110864196	0.190983933	6.443242924	2	2	2	3.709785322	3.94787299	4.029788235	3.209956562
ENSG00000112893	MAN2A1	2.804021099	0.000760977	3.636995337	6.96E-06	6.441016436	6.366709671	7.874700259	5.59507254	9.703174645	9.72016048	10.28597271	10.758765
ENSG00000119737	GPR75	4.145582195	0.012427993	2.293179636	0.190459822	6.438761832	2	3.076665209	2	5.304221918	5.27444544	3.694366604	3.858622973
ENSG00000158158	CNNM4	4.172749248	0.001230164	2.264871327	0.098726147	6.437620575	2	6.228547981	4.058998049	8.959612762	9.10727282	7.364099281	6.915965441
ENSG00000151229	SLC2A13	3.559644917	0.009351472	2.877944868	0.038778125	6.437589784	4.00722531	6.389896633	2	8.621345041	8.49524881	7.980559086	7.787298144
ENSG00000061938	TNK2	2.487564732	1.56E-05	3.940316829	1.18E-12	6.42788156	5.70932728	5.838937808	4.874587708	7.975897842	7.90193448	9.654578447	9.033147652
ENSG00000128298	BAIAP2L2	3.273125484	0.15309083	3.154043831	0.160572676	6.427169315	2	2	2	3.615725078	3.94787299	3.753554863	3.660199087
ENSG00000152558	TMEM123	3.593452491	2.38E-17	2.832431255	3.20E-11	6.425883746	7.940205782	8.968383114	8.460932916	12.13040239	12.0764293	11.2843655	11.40042151
ENSG00000276349		8.269516572	4.81E-06	-1.846443244	0.426694614	6.423073328	2	2	2	8.287525788	8.34030387	2.183373728	2
ENSG00000101605	MYOM1	1.251527823	0.506916165	5.171232162	0.000918547	6.422759985	2	2	3.369317972	3.162721474	3.08597292	6.430795201	6.014942101
ENSG00000164983	TMEM65	2.829303322	0.145913141	3.589495816	0.054407162	6.418799139	6.009025997	2	2	7.277360022	7.0774796	7.985968197	7.860656447
ENSG00000277075	HIST1H2AE	4.964290496	0.019164101	1.453106966	0.543480551	6.417397462	2	2	2	4.946755474	5.34860058	2.42098416	3.042026731
ENSG00000086015	MAST2	3.011336201	0.020000883	3.405385838	0.007283244	6.416722039	3.327711234	7.819099893	6.073378139	9.569217728	9.69550802	10.14509029	9.896287008
ENSG00000087250	MT3	3.497393865	0.182333448	2.919241808	0.265028723	6.416635673	2	2	2	4.034562184	3.86943969	2.746468522	4.062589736
ENSG00000196712	NF1	3.362256073	4.80E-22	3.0514345	1.48E-18	6.413690573	7.940205782	7.431218269	7.225742779	10.90254063	10.8893881	10.53150331	10.63831254
ENSG00000114554	PLXNA1	3.092285977	9.76E-06	3.319102663	1.64E-06	6.41138864	7.218819781	8.88960062	7.342949392	11.1032668	11.1286372	11.45091976	11.2251347
ENSG00000119280	C1orf198	0.49410387	0.418545189	5.911300893	6.72E-34	6.405404763	8.653385977	9.090820894	7.777116083	9.09649754	9.10513517	14.41570354	14.5942172
ENSG00000205078	SYCE1L	3.081048508	0.179212705	3.324116159	0.134438751	6.405164667	2	2	2	3.566290607	3.74316441	3.796405078	3.858622973
ENSG00000102606	ARHGEF7	2.730244862	0.047405404	3.669207368	0.005421366	6.39945223	3.327711234	8.053983481	6.073378139	9.500148669	9.57248057	10.37921413	10.55918138
ENSG00000196604	POTEF	7.635431549	4.71E-08	-1.243565637	0.480855236	6.391865912	3.327711234	2	2	8.529212228	8.7394436	2.225793514	2.326754077
ENSG00000116717	GADD45A	2.52846033	0.008801191	3.861825749	2.56E-05	6.390286079	7.750913381	4.93638933	6.203069917	9.17383051	9.23886123	10.37465563	10.67780108
ENSG00000115762	PLEKHB2	3.08026489	0.030837796	3.30561267	0.0187503	6.38587756	8.75772933	8.433041974	3.369317972	11.10137001	11.1007456	11.26494987	11.38459814
ENSG00000158825	CDA	4.16565343	0.045577007	2.219835317	0.312041119	6.385488746	2	2	2	4.54891529	4.40247105	3.12825509	3.128433649
ENSG00000151240	DIP2C	2.918666772	0.02326726	3.466413091	0.005685328	6.385079863	5.330594134	7.538394143	3.369317972	9.147810675	9.20332986	9.800076727	9.636102773
ENSG00000137076	TLN1	2.83656522	0.089599878	3.546107998	0.027546367	6.382673218	4.00722531	10.893964	9.907528187	12.68945875	12.786312	13.27312014	13.60376928
ENSG00000065526	SPEN	2.817701673	0.047964022	3.56018276	0.009627368	6.377884433	3.327711234	6.728495673	8.486635645	10.05583453	10.1211983	10.72587282	10.92566722
ENSG00000197714	ZNF460	3.249064996	0.024456892	3.123914594	0.03002915	6.372979591	2	4.710564154	2	6.336376195	6.39275508	6.631488899	5.717942682
ENSG00000129250	KIF1C	2.361305455	0.055403903	4.00908084	0.000487614	6.370386295	5.866944937	8.124485672	9.868959282	11.06067137	11.0934854	12.48011561	12.93103613
ENSG00000138660	AP1AR	3.817276996	7.43E-05	2.551919242	0.010895983	6.369196238	3.327711234	3.076665209	5.156626401	7.629922299	7.52720828	6.653021698	5.980509018
ENSG00000171860	C3AR1	3.535658969	0.035913463	2.831122633	0.096882542	6.366781602	4.467311984	2	2	6.052081244	6.22964624	6.05195199	4.553838275
ENSG00000166173	LARP6	2.692126572	0.00192982	3.668457535	1.21E-05	6.360584108	5.70932728	8.234834549	6.958202216	9.940529254	10.0373603	11.18489989	10.70133804
ENSG00000196659	TTC30B	4.105938697	0.002373085	2.251950145	0.116476087	6.357888843	2	3.076665209	3.369317972	5.949776857	6.1572134	4.369533167	4.456980017
ENSG00000139675	HNRNPA1L2	5.976342497	9.56E-07	0.377529503	0.810974431	6.353872	3.327711234	5.946638857	2	10.47371148	10.4524262	5.169385838	4.911532223
ENSG00000271079	CTAGE15	5.91344464	0.000827265	0.439299218	0.847708847	6.352743857	2	2	2	6.034011955	6.02777321	2.560105256	2.277179783
ENSG00000130779	CLIP1	2.798165715	6.77E-24	3.549349925	1.32E-38	6.34751564	7.975256505	7.790474954	7.600047325	10.58299811	10.5640243	11.34161973	11.30450932
ENSG00000100485	SOS2	3.139707374	0.002436283	3.206225991	0.001829498	6.345933365	4.467311984	7.051611315	4.523675391	8.95841569	8.99284518	9.0649396	9.018137056
ENSG00000198829	SUCNR1	1.860271891	0.551575085	4.482046599	0.109237936	6.34231849	2	2	2	3.023133209	2.86123014	5.553513572	2.70970617
ENSG00000163697	APBB2	3.02584228	0.091688187	3.316369935	0.057771995	6.342212214	3.327711234	8.623598428	4.058998049	10.14692461	10.1153688	10.57085845	10.25331056
ENSG00000118985	ELL2	3.944304117	4.42E-11	2.397233267	0.00010896	6.341537384	7.159102577	8.623598428	8.763901704	12.28187806	12.2569567	10.87408959	10.55788452
ENSG00000075461	CACNG4	0.836004157	0.795137033	5.504430379	0.037980527	6.340434536	2	4.11352228	2	4.387512679	2	7.57465694	7.953331607
ENSG00000099937	SERPIND1	4.928672011	0.010846156	1.409965459	0.521530272	6.33863747	2	2	2	5.258265913	4.98931816	2.592885947	2.885414003
ENSG00000135503	ACVR1B	3.164267245	0.001104484	3.17399027	0.000997654	6.338257515	5.70932728	5.72254323	2	8.166267464	8.29225867	8.117815385	8.353097069
ENSG00000130517	PGPEP1	3.120404645	2.36E-05	3.216496908	1.15E-05	6.336901553	5.532353208	5.946638857	7.502634626	9.623848865	9.66099896	9.739377057	9.736956851
ENSG00000157106	SMG1	3.691729125	0.004232874	2.644737687	0.047077556	6.336466812	5.866944937	8.968383114	5.156626401	11.29090415	11.3191169	10.40164855	10.10403354
ENSG00000113595	TRIM23	3.076948193	0.002281184	3.258711197	0.001075078	6.335659391	5.532353208	5.131603625	2	7.783822874	7.69294162	8.306273771	7.383872668
ENSG00000120051	CFAP58	4.100832317	0.059311619	2.23480162	0.328120774	6.335633937	2	2	2	4.694064037	4.0930011	3.059555873	3.209956562
ENSG00000071967	CYBRD1	2.737813032	2.71E-16	3.596826432	1.04E-27	6.334639464	8.855032352	8.590771778	8.325066185	11.36279078	11.3010215	11.98530831	12.36968045
ENSG00000177169	ULK1	2.51022061	0.019562832	3.822287906	0.000178123	6.332508516	4.467311984	7.901717006	6.534109959	9.378328971	9.35315328	10.85953478	10.45783446
ENSG00000165322	ARHGAP12	3.59871718	0.001801872	2.733366903	0.021176021	6.332084083	6.257048221	6.389896633	2	9.287824288	9.29432703	8.488261545	8.376090431
ENSG00000183287	CCBE1	3.018868067	0.001148305	3.312079144	0.000292817	6.330947211	4.00722531	4.442710129	6.43197674	8.203120604	8.2561084	8.260460366	8.7391339
ENSG00000105146	AURKC	3.148191506	0.182411111	3.18112633	0.167543084	6.329317836	2	2	2	3.615725078	3.7864959	3.956110469	3.45257121
ENSG00000140396	NCOA2	3.542966138	0.002346365	2.778115599	0.019945307	6.321081738	2	6.389896633	6.073378139	9.109497937	9.17914662	8.457441933	8.312916523
ENSG00000174564	IL20RB	2.702385035	0.159914848	3.61434833	0.047321841	6.316733365	2	3.076665209	2	3.663521627	4.40247105	4.612704558	5.005857272
ENSG00000146242	TPBG	4.968673395	5.26E-13	1.345897957	0.081368703	6.314571352	8.285626337	6.311476754	8.110401118	12.72491396	12.7391892	9.259978091	8.962375727
ENSG00000172399	MYOZ2	-0.310504651	0.917283156	6.623134479	0.002070948	6.312629829	2	2	2	2.270786764	2.2680111	4.644324387	7.521960976
ENSG00000133275	CSNK1G2	3.00341222	0.085411097	3.308853728	0.051766586	6.312265949	4.00722531	8.433041974	3.369317972	9.897454779	9.9554848	10.11895226	10.33507589
ENSG00000125633	CCDC93	3.700083876	6.55E-05	2.610404304	0.006426257	6.31048818	4.00722531	7.051611315	6.958202216	10.15426209	10.1592994	9.242652114	8.88019044
ENSG00000186660	ZFP91	3.587205888	4.23E-05	2.721834742	0.002429239	6.30904063	5.532353208	7.901717006	8.380957451	11.25088489	11.2494513	10.39018357	10.38328123
ENSG00000213064	SFT2D2	1.563793955	0.197587957	4.742488391	7.90E-06	6.306282346	4.467311984	5.838937808	2	6.394182228	6.14893615	8.955762164	9.719663687
ENSG00000092607	TBX15	3.398117033	0.1005893	2.907777199	0.159343355	6.305894232	2	6.535001748	2	8.287525788	8.37797443	8.345877055	7.089349481
ENSG00000167105	TMEM92	2.427248941	0.200555174	3.875728698	0.028223199	6.302977639	2	3.076665209	2	3.754611435	3.98554648	4.652122235	5.361470313
ENSG00000154262	ABCA6	6.746784176	1.37E-05	-0.44482059	0.832938355	6.301963586	2	2	2	6.710878132	6.93069905	2.267001569	2.225841198
ENSG00000167216	KATNAL2	2.494072687	0.307672642	3.804905541	0.094717057	6.298978227	2	2	2	2.947924294	3.55551834	4.291799915	4.077147165

ENSG00000139263	LRIG3	3.21462642	0.015276125	3.082852392	0.019964283	6.297478812	4.467311984	6.464272742	2	8.349110081	8.38680047	8.328950294	8.140837768
ENSG00000149591	TAGLN	2.395130635	0.021070779	3.898777465	6.75E-05	6.2939081	11.54780176	10.20962523	8.296287027	12.92821476	12.9521752	14.45351417	14.43323509
ENSG00000126860	EVI2A	4.738217158	0.010240552	1.554036979	0.451083615	6.292254136	2	3.076665209	2	5.720439317	5.93454154	3.709392871	2.70970617
ENSG00000101079	NDRG3	2.880648239	0.032370417	3.411402999	0.009226918	6.292051237	4.467311984	7.274681896	3.369317972	8.767939462	8.81201036	9.12745764	9.484290996
ENSG00000198198	SZT2	3.323927874	2.11E-13	2.965039368	6.27E-11	6.288967242	5.095959458	6.04685525	6.073378139	9.029678018	9.06500642	8.707109313	8.675981178
ENSG00000163349	HIPK1	2.953087218	0.103167922	3.333313672	0.058285878	6.28640089	4.00722531	8.687092496	3.369317972	10.11504861	10.1217271	10.61191991	10.37299407
ENSG00000170006	TMEM154	5.519519833	3.37E-07	0.765031331	0.562234526	6.284551163	2	4.442710129	2	8.291333947	8.36552655	3.753554863	4.175132639
ENSG00000122674	CCZ1	6.901370272	4.48E-14	-0.617541778	0.596177968	6.283828493	4.00722531	3.076665209	3.369317972	9.860122857	9.70471389	3.316666208	2.918126211
ENSG00000259075	POC1B-GALN	7.235914361	1.45E-06	-0.952773198	0.630693854	6.283141163	2	2	2	7.226553841	7.36873214	2.183373728	2.172608095
ENSG00000183580	FBXL7	2.98530754	0.174608987	3.292955993	0.123227604	6.278263532	2	7.051611315	2	8.537252353	8.34574597	8.756774919	8.741420621
ENSG00000109854	HTATIP2	3.648235386	0.006781203	2.628922124	0.058029621	6.27715751	2	4.93638933	6.80318718	9.044420506	9.21030762	8.200570331	8.036691948
ENSG00000113742	CPEB4	3.799205756	9.91E-07	2.475656906	0.002138016	6.274862662	6.563806279	5.131603625	7.502634626	10.47622292	10.3666911	9.243297541	8.952540041
ENSG00000213658	LAT	4.700389565	0.01506708	1.57380015	0.466152464	6.274189716	2	2	2	4.887609717	4.97065356	2.775316226	2.851942824
ENSG00000126746	ZNF384	3.67424972	0.007764718	2.595755622	0.06838385	6.270005342	2	6.666836837	4.523675391	9.031955922	8.96718201	7.995960198	7.869046644
ENSG00000163378	EOGT	3.518892963	0.008000873	2.744406657	0.042845232	6.263299619	2	6.951736442	5.930865648	9.483600848	9.36302682	8.409366736	8.866637078
ENSG00000115525	ST3GAL5	3.410493016	1.75E-06	2.851101467	7.81E-05	6.261594483	3.327711234	5.457120953	4.523675391	8.001694993	7.89204953	7.334117317	7.462213768
ENSG00000026508	CD44	3.767844057	5.48E-70	2.49322201	5.61E-31	6.261066066	10.35389212	10.18234215	10.26770388	14.02560113	14.0380274	12.6485434	12.85918508
ENSG00000162723	SLAMF9	3.993531291	0.232704499	2.266738357	0.515187862	6.260269649	2	2	2	4.387512679	4.2865223	3.767980144	2.059860178
ENSG00000178096	BOLA1	2.501991805	0.060355042	3.755839105	0.002766242	6.257830911	2	4.710564154	2	5.662809983	5.70538658	6.947285242	6.791281771
ENSG00000148154	UGCG	3.234930177	7.84E-06	3.021510255	3.09E-05	6.256440432	7.384433967	7.002537973	5.156626401	9.991161874	9.96965331	9.945583522	9.565261796
ENSG00000188234	AGAP4	5.429660284	3.17E-09	0.822763757	0.457717047	6.252424041	3.327711234	4.11352228	2	8.272191829	8.30721295	4.099885429	4.018014792
ENSG00000213347	MXD3	2.883517588	0.066569578	3.367969244	0.027078632	6.251486833	2	3.686303414	2	4.846787657	4.95174433	5.524070859	5.101530266
ENSG00000135631	RAB11FIP5	2.384887431	0.000408059	3.866127923	2.15E-09	6.251015354	8.009475818	8.169643011	6.322058544	10.06309216	10.0864045	11.43271451	11.66328098
ENSG00000198162	MAN1A2	2.284076047	0.371250007	3.965421594	0.094841246	6.249497641	2	8.147241016	2	8.866956935	8.8250571	10.40957704	10.62040904
ENSG00000155100	OTUD6B	2.995405596	0.009301554	3.253839023	0.004074912	6.249244618	3.327711234	5.303523747	2	7.066878356	6.81514011	7.052670333	7.32867054
ENSG00000127081	ZNF484	3.485599946	0.023045186	2.762629414	0.076431446	6.24822936	2	5.303523747	2	7.178012736	7.21069602	6.649130473	6.326546677
ENSG00000150477	KIAA1328	2.757969598	0.0324034	3.489996132	0.004927027	6.24796573	2	4.442710129	2	5.651002631	5.64780787	6.419417643	6.245088435
ENSG00000170962	PDGFD	2.861159916	0.315977132	3.386792057	0.21749096	6.247951973	2	2	2	3.709785322	3.28037825	2.775316226	4.489993931
ENSG00000187720	THSD4	1.95656353	0.139117554	4.289495866	0.000334043	6.246059396	2	6.464272742	5.39244166	7.43025113	7.30311013	10.00006426	9.248616776
ENSG00000143816	WNT9A	1.27091119	0.609720423	4.97244723	0.017198851	6.24335842	2	2	2	2.3892346	2.94009902	5.206928476	5.12973572
ENSG00000188643	S100A16	2.58201302	7.13E-06	3.660961726	6.11E-11	6.242974746	8.674867122	7.761270542	7.896971941	10.70292919	10.7598372	11.20607774	12.23269239
ENSG00000170113	NIPA1	2.915919346	0.018178424	3.326305576	0.005911789	6.242224922	2	6.389896633	6.322058544	8.584574266	8.69180693	9.242652114	8.818171767
ENSG00000123610	TNFAIP6	8.546681066	6.43E-06	-2.305640583	0.287678243	6.241040483	2	6.311476754	2	13.21893422	13.2489436	3.724264246	2.374681325
ENSG00000143847	PPFIA4	2.994622115	0.00021565	3.246232843	4.88E-05	6.240854959	3.327711234	6.140560021	5.772716771	8.385193462	8.41641145	8.609360894	8.68907177
ENSG00000118432	CNR1	1.154970505	0.697209163	5.084849603	0.04093731	6.239820108	2	2	2	3.023133209	2.14022021	4.927401733	5.545463418
ENSG00000164180	TMEM161B	3.494448889	0.019477382	2.744119814	0.071516817	6.238568703	2	5.131603625	2	7.088925745	6.96881778	6.577231679	5.980509018
ENSG00000176624	MEX3C	3.026719552	0.040445365	3.207232611	0.02745771	6.233952163	6.257048221	7.538394143	2	9.432787137	9.47403846	9.797661995	9.447297716
ENSG00000064205	WISP2	-1.305489008	0.672951696	7.53692807	0.000876588	6.231439061	2	2	2	2.270786764	2	8.334412727	5.98823215
ENSG00000122912	SLC25A16	2.712716093	0.111386568	3.518359019	0.031231601	6.231075112	2	5.595927442	2	6.733448311	6.73446058	7.479255779	7.552234734
ENSG00000161914	ZNF653	2.955754666	0.020812654	3.272608288	0.008995102	6.228362954	2	4.442710129	2	5.839660368	5.8245091	6.084012396	6.172377306
ENSG00000115020	PIKFYVE	3.38581335	2.06E-07	2.841147308	1.62E-05	6.226960659	5.866944937	7.232742069	5.59507254	9.76610238	9.78836002	9.314431634	9.152224182
ENSG00000123094	RASSF8	4.207705001	0.000644627	2.017787724	0.128084739	6.225492725	8.042902253	7.431218269	3.369317972	11.39897907	11.383149	9.443604635	8.92815536
ENSG00000167065	DUSP18	3.395045366	0.000214195	2.830377466	0.002345349	6.225422832	4.00722531	4.11352228	2	6.652857793	6.5890786	6.040114727	6.127623461
ENSG00000163431	LMOD1	0.490577839	0.841484908	5.734656108	0.002880079	6.225233947	8.009475818	3.076665209	2	7.044488801	6.8715633	12.34020583	11.95141501
ENSG00000137831	UACA	1.836287071	0.081673461	4.388624686	3.83E-06	6.224911758	5.532353208	8.762721501	7.734844356	9.650037049	9.70541961	12.37393608	12.05771452
ENSG00000134030	CTIF	1.5673442	0.103494474	4.655824799	4.37E-08	6.223168999	6.009025997	7.638155693	8.742726175	9.467702639	9.32403997	12.21874707	12.69866275
ENSG00000107185	RGP1	1.792894998	0.349647791	4.425981132	0.009967186	6.21887613	2	7.189546432	3.369317972	7.366699513	7.57741621	10.03067056	10.13066486
ENSG00000083168	KAT6A	2.898462613	0.00551247	3.319862158	0.001171726	6.218324772	6.009025997	7.467835385	4.058998049	9.274418797	9.30181283	9.787520004	9.622210129
ENSG00000160223	ICOSLG	4.430703453	0.068175471	1.7867648	0.496500125	6.217468253	2	2	2	4.670864177	4.72553021	3.296906311	2.374681325
ENSG00000050344	NFE2L3	4.414978197	2.54E-08	1.800353449	0.036866149	6.215331646	6.366709671	5.946638857	4.058998049	10.05807153	10.0722485	7.898530056	6.881131115
ENSG00000109610	SOD3	1.71914596	0.352977549	4.492557899	0.006315896	6.211703859	2	5.72254323	2	5.775854763	6.03677338	8.963225048	8.117259579
ENSG00000132563	REEP2	-3.753168213	0.122079915	9.961365229	9.38E-12	6.208197016	2	2	2	2	2	9.888345571	10.09131582
ENSG00000105576	TNPO2	2.933768252	3.48E-20	3.271652633	3.36E-25	6.205420885	7.218819781	6.899080544	7.163363831	9.973493741	9.99815569	10.3869745	10.25511215
ENSG00000146094	DOK3	3.627768937	0.004232874	2.576530499	0.049027138	6.204299436	2	4.442710129	2	6.476767609	6.44777477	5.65789504	5.275717819
ENSG00000150630	VEGFC	5.834104801	6.99E-05	0.369516888	0.84468287	6.203621688	7.904282243	5.946638857	2	12.42470411	12.4639002	7.200910877	6.821857532
ENSG00000162482	AKR7A3	3.571442141	0.12645146	2.628913066	0.266884496	6.200355207	2	2	2	3.921164147	4.0930011	3.082820997	3.600986722
ENSG00000074181	NOTCH3	1.320545039	0.002273448	4.878301729	7.28E-35	6.198846768	8.444143041	9.256956408	8.608698767	10.10155624	10.1664929	13.83562452	13.52227091
ENSG00000204219	TCEA3	2.35911222	0.037885253	3.838491573	0.000306801	6.197603793	2	5.131603625	3.369317972	6.043074888	6.33555364	7.802823992	7.421437279
ENSG00000158163	DZIP1L	2.898440262	0.00251516	3.298067002	0.000443025	6.196507264	4.00722531	4.442710129	2	6.5612028	6.17362678	6.546183077	6.951954485
ENSG00000163491	NEK10	3.61381902	0.009718181	2.57903915	0.072993837	6.19285817	3.327711234	3.076665209	2	5.651002631	5.53799487	4.580376151	4.77533149
ENSG00000163820	FYCO1	1.966165714	0.085906155	4.22350523	4.79E-05	6.189670944	4.00722531	6.78762695	7.777116083	8.707945635	8.83927354	11.02321941	11.02075007
ENSG00000047644	WWC3	2.568275238	0.017140458	3.619285098	0.000439159	6.187560336	3.327711234	6.311476754	7.029880454	8.664561167	8.7297638	9.925616522	9.529981565
ENSG00000267697	LUZP6	11.37782338	6.62E-16	-5.191150709	0.022650204	6.186672676	2	2	2	11.43320503	11.3789528	2	2
ENSG00000131730	CKMT2	2.08072363	0.386456011	4.105733342	0.058167426	6.186456972	2	2	2	2.947924294	3.15372745	4.133696865	4.673629022
ENSG00000172456	FGGY	3.597711774	0.042507581	2.587437628	0.152650368	6.185149402	2	3.076665209	2	4.76150579	4.85329164	3.767980144	4.188602707
ENSG00000165807	PPP1R36	3.27312122	0.163554675	2.910994154	0.210663463	6.184115373	2	2	2	3.615725078	3.94787299	3.648325314	3.430065404
ENSG00000075275	CELSR1	1.689166646	0.388531091	4.487943586	0.009759057	6.177110232	2	2	5.59507254	6.104969671	4.97065356	8.372671446	8.312916523
ENSG00000168389	MFSD2A	4.838241404	2.85E-08	1.335781555	0.176958539	6.174022959	3.327711234	4.710564154	2	8.304583895	8.20112674	4.868377487	4.998228167
ENSG00000086991	NOX4	3.781542615	0.000132612	2.390650889	0.021303089	6.172193504	3.327711234	4.11352228	4.058998049	7.186217188	7.32531744	6.398709156	5.217771644
ENSG00000175294	CATSPER1	2.938233356	0.267665409	3.231918637	0.208181348	6.170151993	2	2	2	3.663521627	3.45167086	3.011868911	4.254128754
ENSG00000166822	TMEM170A	3.755429525	4.83E-05	2.41452044	0.012405533	6.169949965	4.00722531	3.076665209	5.39244166	7.880528731	7.89700047	6.900587441	6.179140936
ENSG00000136167	LCP1	0.052330339	0.984043262	6.117453105	0.001915899	6.169783444	2	3.076665209	2	2.784612485	2.38539787	7.959503304	5.108633494
ENSG00000132207	SLX1A	8.957862302	1.61E-09	-2.79188483	0.184524743	6.165977471	2	2	2	8.977450658	9.01350799	2.094598369	2
ENSG00000042286	AIFM2	3.975571649	8.10E-14	2.189870025	7.42E-05	6.165441674	5.095959458	5.838937808	5.59507254	9.411949708	9.42260746	7.336538937	7.911296863
ENSG00000160469	BRSK1	3.409634008	0.004777904	2.755371609	0.025405862	6.165005617	2	4.442710129	6.203069917	8.235106869	8.30535212	7.631969638	7.622152309
ENSG00000078804	TP53INP2	2.689028545	3.20E-09	3.475563277	6.75E-15	6.164591822	7.579279349	7.874700259	7.098165388	10.1970127	10.2530269	10.65220953	11.2961726
ENSG00000113719	ERGIC1	2.75788183	0.020652733	3.40519288	0.003212389	6.16307471	8.285626337	5.838937808	9.798878489	11.42953799	11.4842747	11.85159294	12.31828471
ENSG00000160703	NLRX1	2.477452083	0.23731318	3.685607493	0.062813847	6.163059576	2	6.602424674	2	7.337530098	7.64076905	8.660996377	8.712572735
ENSG00000022267	FHL1	-2.147887483	0.185646208	8.310923966	2.50E-09	6.163036482	8.009475818	7.315436877	2	5.144975053	5.09650239	15.12381879	15.6380693
ENSG00000123684	LPGAT1	2.572892658	0.002065909	3.587163082	8.27E-06	6.16005574	6.009025997	7.503546079	5.156626401	9.067921572	9.12952895	10.05127285	10.16258417
ENSG00000146826	C7orf43	3.189914467	0.033156281	2.969324128	0.046894072	6.159238595	3.327711234	6.228547981	2	7.93509946	7.96222667	7.612118169	7.842663598
ENSG00000102781	KATNAL1	2.379095017	1.47E-05	3.775130933	9.66E-13	6.15422595	5.095959458	6.535001748	6.629488387	8.578353644	8.54498542	9.996814524	9.898081691
ENSG00000203812	HIST2H2AA3	11.34428912	8.54E-16	-5.19115072	0.022702853	6.153138398	2	2	2	11.3344645	11.4102394	2	2
ENSG00000272196	HIST2H2AA4	11.34428912	8.54E-16	-5.19115072	0.022702853	6.153138398	2	2	2	11.3344645	11.4102394	2	2
ENSG00000198146	ZNF770	3.261943757	8.92E-05	2.880909522	0.000593789	6.142853279	4.467311984	6.602424674	5.39244166	8.990393018	8.90095288	8.96596483	8.018893446
ENSG00000001561	ENPP4	1.050262246	0.592855432	5.09001386	0.001529662	6.140276106	2	3.076665209	2	3.09461492	2.94009902	6.433059984	5.825320785
ENSG00000187566	NHLRC1	2.935248432	0.066092631	3.199598706	0.040098237	6.134847137	2	2	4.058998049	5.003571641	5.07918227	5.008609438	5.502966356
ENSG00000100888	CHD8	2.960590317	0.019088294	3.174218237	0.010836307	6.134808554	5.095959458	8.590771778	5.59507254	10.1568736	10.290665	10.4929453	10.38144961
ENSG00000140575	IQGAP1	3.135228821	5.26E-05	2.998668176	0.0001103	6.133896997	8.042902253	10.21502048	8.845626171	12.43270008	12.4615001	12.22272076	12.39364033
ENSG00000171817	ZNF540	2.973445569	0.29251725	3.157435879	0.249742787	6.130881448	2	2	2	3.406930997	3.74316441	4.281782022	2.746601469
ENSG00000133030	MPRIP	2.355622558	0.066342136	3.770045937	0.00174607	6.125668494	4.815609471	9.079034257	9.06622548	10.84021544	10.8974396	12.26318033	12.29978441
ENSG00000130477	UNC13A	1.358942678	0.196475406	4.765537198	1.49E-07	6.124479876	3.327711234	3.686303414	5.772716771	5.720439317	5.98191036	8.932738969	9.374763636
ENSG00000155111	CDK19	3.028440123	0.03281527	3.095183509	0.027475652	6.123623632	2	6.389896633	4.058998049	8.109160159	7.96929529	8.266822833	7.926450253
ENSG00000104447	TRPS1	3.373578742	5.46E-06	2.749140206	0.000261617	6.122718948	4.815609471	6.844429755	5.39244166	9.237391778	9.30833119	8.702422971	8.603665995
ENSG00000169641	LUZP1	3.123050695	0.017260781	2.999552232	0.02221024	6.122602928	4.00722531	8.806266215	7.552162999	10.84960847	10.884411	10.57483812	10.89518287
ENSG00000146858	ZC3HAV1L	2.424295804	0.010714204	3.698112377	3.87E-05	6.122408181	4.467311984	3.076665209	4.058998049	6.213314212	5.74985018	7.463831776	6.926052784
ENSG00000206455		4.68720374	0.003695722	1.435203289	0.432125179	6.12240703	3.327711234	2	2	5.709096143	5.85535975	3.082820997	3.36036153
ENSG00000159363	ATP13A2	3.190185484	0.036927252	2.930968936	0.054822702	6.12115442	2	7.393647515	5.39244166	9.286870873	9.29432703	8.813940551	9.2234807
ENSG00000165272	AQP3	2.73647328	0.26922137	3.382773976	0.152201542	6.119247256	2	2	2	3.709785322	3.08597292	3.796405078	3.940519005
ENSG00000166225	FRS2	3.102861168	0.054064283	3.014365784	0.058825076	6.117226953	4.815609471	7.431218269	2	9.208480821	9.06390621	9.110931391	8.986675514
ENSG00000115956	PLEK	0.010133594	0.997264364	6.101480583	0.007467316	6.111614177	2	2	2	2.270786764	2.38539787	7.027411703	4.119958326
ENSG00000101224	CDC25B	3.153180195	0.178994785	2.958319296	0.201973614	6.111499491	2	7.572420273	2	9.136211578	9.12952895	8.557885437	9.24096022
ENSG00000159176	CSRP1	2.22481964	0.003921045	3.884673196	9.88E-08	6.109492836	9.567995545	7.874700259	7.934808822	10.90409587	10.8971308	12.85044134	12.19183793
ENSG00000170265	ZNF282	2.904538895	0.21164903	3.203191472	0.156449615	6.107730367	2	7.467835385	2	8.773395507	8.78955647	9.225442227	8.913019964
ENSG00000139370	SLC15A4	3.429601219	0.010028324	2.676907156	0.048890727	6.106508375	2	6.140560021	7.029880454	9.425874714	9.46406471	8.864738722	8.509534124
ENSG00000156795	WDYHV1	2.350909734	0.104695101	3.754209358	0.005570045	6.105119091	2	4.93638933	2	5.85002558	5.65950892	6.964610156	7.211494604
ENSG00000038532	CLEC16A	2.448978171	0.185413931	3.655996781	0.036320638	6.104974952	3.327711234	7.099070191	2	7.97352961	8.08661608	8.927929329	9.472294474

ENSG00000141858	SAMD1	2.538846335	0.000553577	3.563988517	4.86E-07	6.102834852	4.00722531	4.710564154	5.930865648	7.395290829	7.53675633	8.688271909	8.22740336
ENSG00000168826	ZBTB49	3.244200067	0.01448403	2.855146191	0.032443941	6.099346258	2	4.11352228	2	5.797438367	5.67111583	5.454427604	5.300743019
ENSG00000170571	EMB	4.948607569	0.000729237	1.147847756	0.499266159	6.096455325	4.00722531	2	2	6.989292186	6.80463998	3.917810786	3.236138336
ENSG00000198455	ZXDB	2.845407184	0.123195888	3.249417557	0.06987157	6.094824741	2	5.946638857	2	7.127781374	7.28055563	7.624061799	7.576773052
ENSG00000197776	KLHDC1	3.313965611	0.0960601	2.779023133	0.161994098	6.092988743	2	3.076665209	2	4.76150579	4.3456612	4.580376151	3.496556178
ENSG00000139926	FRMD6	4.054876088	6.36E-07	2.036765167	0.019469114	6.091641256	6.138360429	8.451443573	8.561105505	12.08271877	12.1014432	9.813394654	10.30156833
ENSG00000184227	ACOT1	3.875630924	1.86E-05	2.215959653	0.020780792	6.091590577	4.00722531	3.686303414	5.156626401	8.001694993	7.95749494	5.677130623	6.834765628
ENSG00000123411	IKZF4	2.727556021	0.100196808	3.363494898	0.035315165	6.091050919	2	5.457120953	2	6.598566398	6.6253296	7.118879607	7.327148877
ENSG00000100226	GTPBP1	3.059099934	2.47E-05	3.031181975	2.74E-05	6.090281909	8.257427002	7.701025996	6.073378139	10.57833006	10.6877448	10.46845053	10.73199123
ENSG00000048707	VPS13D	2.738825349	0.006353097	3.349209341	0.000597178	6.08803469	4.467311984	6.951736442	7.818184403	9.636628223	9.66172639	10.22503182	10.28853085
ENSG00000103710	RASL12	2.367679863	0.177357792	3.719784818	0.023412451	6.087464681	2	3.686303414	2	4.574143677	4.31639476	6.060766632	5.020995508
ENSG00000082497	SERTAD4	-2.311083228	0.371950735	8.398403621	3.19E-08	6.087320393	2	2	2	2	2.14022021	8.592743352	8.272375674
ENSG00000133704	IPO8	2.968055741	0.000260005	3.118667058	0.000108468	6.086722799	4.815609471	5.946638857	7.285536013	9.239364442	9.26015177	9.379289361	9.419363022
ENSG00000170759	KIF5B	2.849324163	0.001065505	3.233884891	0.000156598	6.083209055	7.975256505	9.71856931	7.342949392	11.58267051	11.5238208	11.90615729	11.96847501
ENSG00000170385	SLC30A1	3.231671785	0.162428131	2.851455366	0.214965079	6.083127151	2	7.467835385	2	9.03990038	9.16894939	9.014789544	8.37166861
ENSG00000170142	UBE2E1	3.460878138	0.000392827	2.620980549	0.008953574	6.081858688	4.00722531	7.189546432	7.225742779	10.12468558	10.1036386	9.295574678	9.26102247
ENSG00000001461	NIPAL3	1.869196158	0.283079066	4.208068764	0.007459944	6.077264922	4.815609471	8.968383114	4.058998049	9.322664889	9.39749836	11.49474119	11.86616355
ENSG00000142192	APP	3.214465921	6.16E-08	2.861961899	1.63E-06	6.076427821	9.473095744	9.424669273	10.70548138	13.21373885	13.1973249	12.5744683	13.08673594
ENSG00000171448	ZBTB26	2.719056788	0.047700201	3.357105265	0.011354296	6.076162053	2	4.710564154	2	5.910700032	5.84514933	6.637393426	6.30506411
ENSG00000001629	ANKIB1	3.099463387	0.016178162	2.974706814	0.021001623	6.074170201	4.00722531	8.337339476	8.65477198	11.02280532	11.0390977	10.8358731	10.97374872
ENSG00000243708	PLA2G4B	5.098579721	0.005893523	0.975515079	0.658188823	6.0740948	2	2	2	5.242616075	5.3193943	2.656294034	2.509591799
ENSG00000184185	KCNJ12	1.054158706	0.394151052	5.019611174	8.64E-07	6.073769881	2	3.076665209	4.523675391	4.070463721	4.45712836	7.742927022	8.100433454
ENSG00000166349	RAG1	2.494032362	0.358758119	3.578896849	0.160899275	6.072929211	2	2	2	2.947924294	3.55551834	4.496236235	3.287118176
ENSG00000213020	ZNF611	3.555595066	1.98E-07	2.512111855	0.000343479	6.067706922	4.467311984	3.686303414	5.39244166	7.88809286	7.99492122	6.939340974	6.907844475
ENSG00000070404	FSTL3	1.911812009	0.019331325	4.155259572	3.39E-08	6.067071582	8.19931886	7.503546079	9.624627483	10.5946026	10.6387096	12.72467338	12.97888139
ENSG00000108406	DHX40	3.328794141	7.15E-05	2.737727709	0.001294981	6.06652185	4.815609471	5.303523747	7.098165388	9.395237275	9.24566964	8.644966335	8.821417907
ENSG00000084731	KIF3C	2.775062318	0.047622861	3.291083531	0.015805954	6.066145849	6.563806279	7.232742069	2	9.078979414	9.12320501	9.6467939	9.580669163
ENSG00000090530	P3H2	2.384890014	0.069351955	3.67971874	0.002915078	6.064608754	6.816637977	6.04685525	2	8.170409056	8.30721295	9.671701874	9.359213231
ENSG00000183621	ZNF438	2.751740555	0.095756389	3.309874715	0.037646364	6.06161527	2	2	5.156626401	6.260701031	6.04571775	6.658838928	6.718860807
ENSG00000147854	UHRF2	3.278585168	0.002216582	2.780186477	0.01057194	6.058771645	6.73718411	7.051611315	3.369317972	9.626112287	9.58863636	9.390112523	8.767460665
ENSG00000136935	GOLGA1	2.957605775	0.025431454	3.098130744	0.017970055	6.055736519	2	6.389896633	4.523675391	8.008651253	8.11451391	8.1339154	8.265241863
ENSG00000186350	RXRA	2.677041884	0.066450453	3.37434884	0.016614757	6.051390724	7.435667355	6.311476754	2	8.966774408	9.10620439	9.671701874	9.788455943
ENSG00000083799	CYLD	3.309087402	0.023540207	2.740283947	0.064089806	6.049371349	9.247966931	6.04685525	4.523675391	11.18818376	11.100209	10.53743599	10.61605402
ENSG00000105939	ZC3HAV1	4.14344369	0.000411397	1.905180222	0.132382182	6.048623912	4.467311984	8.762721501	8.325066185	12.14455022	12.1401932	9.998344725	9.813396275
ENSG00000224712	NPIPA3	11.23582915	1.65E-15	-5.191150797	0.022726528	6.044678354	2	2	2	11.26402542	11.2650929	2	2
ENSG00000115896	PLCL1	2.33302308	0.392606579	3.711301304	0.142587272	6.044324384	2	2	2	3.162721474	3.2184422	4.667592615	3.209956562
ENSG00000163297	ANTXR2	4.875945914	8.44E-06	1.166995108	0.355406319	6.042941023	6.366709671	7.002537973	9.624627483	13.22230432	13.2509995	9.642885818	9.418291476
ENSG00000198915	RASGEF1A	2.270572932	0.385331055	3.771849892	0.117860642	6.042422824	2	2	2	2.695456494	3.50452879	4.230621763	4.09155917
ENSG00000164949	GEM	3.897143001	0.006549526	2.141862256	0.157487659	6.039005258	2	7.189546432	5.930865648	9.945371153	9.9912274	8.698192208	7.521960976
ENSG00000116132	PRRX1	3.367804535	2.47E-29	2.667260335	5.94E-19	6.03506487	8.107517749	8.433041974	7.896971941	11.51710644	11.530009	10.85806066	10.78961595
ENSG00000176399	DMRTA1	4.133423449	0.061854072	1.899365042	0.423484962	6.032788492	2	2	2	4.598938466	4.2865223	3.1504466	2.746601469
ENSG00000176463	SLCO3A1	4.604967402	0.007224622	1.4236499	0.458667613	6.028617301	2	5.595927442	2	8.581467308	8.57464274	6.081127077	4.56420962
ENSG00000165474	GJB2	4.778111699	0.019324738	1.247289795	0.593027095	6.025401495	2	2	2	5.178261742	4.79082474	2.492221014	2.851942824
ENSG00000197696	NMB	4.750507803	1.63E-05	1.274306805	0.310228532	6.024814608	6.009025997	4.11352228	2	9.34848254	9.3808074	6.037140174	5.909074772
ENSG00000103642	LACTB	3.433400421	7.82E-07	2.589118279	0.00026611	6.0225187	4.815609471	6.951736442	6.203069917	9.610193249	9.63159714	8.642032548	8.910481888
ENSG00000172548	NIPAL4	0.689486832	0.790784372	5.332734687	0.008595717	6.022221519	2	2	2	2.498691189	2.49394783	6.016144783	4.664017718
ENSG00000116852	KIF21B	2.273800221	0.374366032	3.747416652	0.113061522	6.021216872	2	2	2	3.09461492	3.2184422	4.547306681	3.580696614
ENSG00000137877	SPTBN5	5.057189104	0.001872533	0.962833754	0.616400319	6.020022858	2	3.686303414	2	6.799114572	6.98285659	2.803598391	3.858622973
ENSG00000100644	HIF1A	2.976305113	0.000580679	3.043013578	0.000395937	6.019318691	8.138771685	10.36815316	10.77492406	13.10522462	13.0935265	13.12849113	13.20267654
ENSG00000185052	SLC24A3	-1.688040961	0.404229173	7.706417123	6.18E-08	6.018376162	2	3.076665209	2	2.141737246	2.2680111	8.545902039	8.854540499
ENSG00000054967	RELT	3.332610944	0.00059294	2.685738321	0.006620286	6.018349265	5.866944937	4.93638933	2	8.080836374	8.15020018	7.442631903	7.517964974
ENSG00000171757	LRRC34	3.204030721	0.075282483	2.811435577	0.11706113	6.015466298	3.327711234	2	2	4.574143677	4.37434574	4.053534413	4.26688425
ENSG00000185697	MYBL1	2.387162618	0.037134263	3.628170294	0.000758226	6.015332912	3.327711234	2	4.523675391	5.391962469	5.49945122	6.625560108	6.592824411
ENSG00000050748	MAPK9	2.888458564	0.000529129	3.125426843	0.00014468	6.013885408	5.866944937	6.78762695	4.058998049	8.767939462	8.81069917	9.035431928	9.013414035
ENSG00000241360	PDXP	4.015898441	0.009415065	1.997257932	0.226994232	6.013156373	2	5.457120953	2	7.870381136	7.86451142	6.028179536	5.807970301
ENSG00000061656	SPAG4	2.78699126	0.129712892	3.225707667	0.070507478	6.012698928	2	2	5.59507254	6.836083475	6.507263	7.241533944	6.953927794
ENSG00000141298	SSH2	2.609175172	0.260754859	3.401909252	0.125372994	6.011084424	2	7.35507212	2	8.406418094	8.34574597	8.977260248	9.325315571
ENSG00000085117	CD82	4.241239554	1.06E-09	1.768448095	0.018944745	6.009687649	7.096806373	7.503546079	5.39244166	11.08829583	11.1490131	8.408216037	8.870820921
ENSG00000174485	DENND4A	3.343727804	2.90E-05	2.664916642	0.001064564	6.008644446	4.467311984	5.457120953	6.882775709	9.162439556	9.19229617	8.591223132	8.412421212
ENSG00000239305	RNF103	3.239543246	0.151721189	2.762815487	0.220786883	6.002358733	2	7.315436877	2	9.028537716	8.88981993	8.586652837	8.383430148
ENSG00000108854	SMURF2	2.797182886	0.001110684	3.205103911	0.000140544	6.002286797	6.138360429	8.376385542	6.629488387	10.17244415	10.1623867	10.35597781	10.76355445
ENSG00000164935	DCSTAMP	2.014562453	0.566415954	3.987168608	0.215756786	6.001731061	2	2	2	3.289987342	2.68924543	5.147485955	2.277179783
ENSG00000164220	F2RL2	4.396514425	3.52E-10	1.605063669	0.037080714	6.001578093	3.327711234	4.11352228	4.874587708	8.272191829	8.30162324	5.528313928	5.670730563
ENSG00000173917	HOXB2	5.439397426	0.001373482	0.562143408	0.789642976	6.001540834	2	2	5.930865648	9.615519136	9.69550802	5.096960836	4.827924991
ENSG00000160781	PAQR6	2.729369702	0.14612275	3.269738387	0.069822896	5.99910809	3.327711234	2	2	4.329433326	3.82856379	4.612704558	4.445805334
ENSG00000119844	AFTPH	3.412519634	0.007180368	2.58617839	0.046628793	5.998698025	4.00722531	6.844429755	3.369317972	8.912174893	8.87358581	8.299462788	7.811442311
ENSG00000116525	TRIM62	2.505587247	0.182039968	3.488904327	0.050274744	5.994491573	2	5.946638857	2	6.809774134	6.94032357	7.668951058	7.984555672
ENSG00000131773	KHDRBS3	2.486726867	0.163957612	3.507709908	0.038301117	5.994436775	2	5.72254323	2	6.70517998	6.57060569	7.6993745	7.558732759
ENSG00000249931	GOLGA8K	7.469271978	2.14E-06	-1.476031839	0.470865845	5.993240139	2	2	2	7.549526251	7.50468064	2.183373728	2.059860178
ENSG00000139438	FAM222A	3.212530354	0.17595559	2.780805188	0.238575668	5.993155542	2	2	2	3.663521627	3.82856379	3.5515855	3.36036153
ENSG00000197208	SLC22A4	5.399654523	4.96E-08	0.592908605	0.630920961	5.992563127	2	3.076665209	4.874587708	8.589222193	8.69038176	3.904814898	4.411752372
ENSG00000138435	CHRNA1	3.740204137	0.199271942	2.251183441	0.454855955	5.991387579	2	2	3.369317972	5.128039203	4.70309173	4.504874511	2.059860178
ENSG00000163359	COL6A3	3.946248851	1.40E-09	2.035519861	0.003115315	5.981768712	11.18203661	11.26034006	9.541233192	14.76074113	14.8187889	13.05031454	12.6865867
ENSG00000011478	QPCTL	2.838047984	3.72E-05	3.143095517	3.65E-06	5.981143501	5.866944937	4.11352228	5.930865648	8.192977693	8.17894021	8.313052751	8.642725409
ENSG00000197969	VPS13A	2.869547716	0.001128625	3.108259432	0.000350141	5.977807148	7.709880939	5.131603625	6.322058544	9.669187952	9.45653888	9.739377057	9.866478759
ENSG00000105855	ITGB8	5.541755181	1.31E-08	0.432151156	0.728068232	5.973906336	2	4.93638933	4.058998049	9.369349625	9.32035914	3.943456545	4.911532223
ENSG00000104321	TRPA1	7.780651506	2.47E-07	-1.811091662	0.300664025	5.969559844	2	5.946638857	2	12.11504048	12.1075961	3.374372158	3.183290849
ENSG00000123977	DAW1	3.011795051	0.302944922	2.953823836	0.301212939	5.965618888	2	2	2	3.663521627	3.55551834	2.560105256	4.161535617
ENSG00000118276	B4GALT6	3.42608518	0.00680255	2.539092811	0.050514234	5.96517799	2	4.442710129	2	6.276156955	6.26836889	5.463319095	5.431121909
ENSG00000126012	KDM5C	2.849582857	0.131914752	3.109087171	0.092258249	5.958670028	2	9.396454291	7.285536013	10.92201514	10.9843247	11.26947193	11.15321065
ENSG00000185722	ANKFY1	3.196177305	6.34E-17	2.759603351	5.56E-13	5.955780656	7.667647223	7.954278496	7.225742779	10.78962055	10.8671698	10.20462959	10.56047708
ENSG00000130940	CASZ1	1.803556345	0.112165694	4.150545955	4.18E-05	5.9541023	3.327711234	4.11352228	2	4.804776789	4.89348248	7.170014349	6.866539459
ENSG00000153207	AHCTF1	3.573591631	0.002523465	2.379539116	0.053196051	5.953130748	5.095959458	7.145017454	3.369317972	9.479434065	9.45905186	8.551645649	7.959237827
ENSG00000107771	CCSER2	2.577159257	1.11E-13	3.373664261	5.42E-23	5.950823518	6.816637977	7.315436877	7.342949392	9.803915852	9.64563775	10.5938145	10.4433342
ENSG00000197324	LRP10	3.030775841	9.75E-09	2.917333826	3.32E-08	5.948109667	10.50545418	10.54135596	9.287079172	13.21837177	13.2696695	13.24378775	13.00831746
ENSG00000119321	FKBP15	3.070096685	0.016495651	2.875426673	0.024934862	5.945523358	4.467311984	7.761270542	4.523675391	9.441381351	9.51808625	9.203388342	9.365527647
ENSG00000180155	LYNX1	1.004099921	0.435671309	4.940850989	3.91E-06	5.94495091	2	6.311476754	5.772716771	6.401247806	6.56439512	10.27275639	10.44298299
ENSG00000180787	ZFP3	2.287993543	0.032097652	3.654196788	0.00025706	5.94219033	3.327711234	4.442710129	2	5.44762523	5.64780787	6.99251878	6.669279147
ENSG00000176994	SMCR8	3.301888984	1.52E-06	2.638366772	0.000157435	5.940255756	7.624139768	6.535001748	5.59507254	10.04459716	10.0955857	9.482666068	9.333296562
ENSG00000118496	FBXO30	3.476106324	0.135689274	2.459430412	0.299147391	5.935536736	2	7.638155693	2	9.541506037	9.49786081	8.711314045	8.278687361
ENSG00000134287	ARF3	2.899097204	0.003712692	3.035499387	0.002170653	5.93459659	6.96351253	9.386925327	6.958202216	11.10921196	11.1863961	10.99972888	11.5222047
ENSG00000122126	OCRL	2.565755622	0.319889927	3.366743365	0.172175696	5.932498987	2	8.356994601	2	9.356683903	9.31389502	10.15747031	10.10714012
ENSG00000142279	WTIP	2.074267255	0.051696866	3.857411459	8.82E-05	5.931678714	2	5.72254323	5.39244166	6.941624891	7.06433609	9.004546892	8.45344973
ENSG00000127418	FGFRL1	2.681969191	0.181657578	3.247072603	0.093525732	5.929041794	4.467311984	8.469613413	2	9.610955295	9.66535801	9.908424552	10.44438731
ENSG00000119927	GPAM	3.190679513	0.119128865	2.736471904	0.18059707	5.927151417	2	6.464272742	2	8.027038952	8.08878133	8.015738956	7.049302614
ENSG00000181381	DDX60L	4.818851628	0.031171927	1.104640878	0.666867266	5.923492506	2	7.73146271	2	10.95719949	10.9469174	7.131485609	7.401331798
ENSG00000170891	CYTL1	-0.313248726	0.911524164	6.236730233	0.001732414	5.923481507	2	2	2	2.141737246	2.38539787	5.399896186	6.899677537
ENSG00000126821	SGPP1	2.344046073	0.302845914	3.578398312	0.094088158	5.922444385	2	7.099070191	2	7.862723323	7.85690889	9.200067611	8.948586914
ENSG00000198121	LPAR1	3.345414805	0.001006517	2.574550067	0.013658585	5.919964872	5.095959458	8.505280662	8.076950877	11.14516677	11.1026219	10.356127	10.35387936
ENSG00000105429	MEGF8	2.279541318	0.096844942	3.638089335	0.004641907	5.917630653	3.327711234	7.874700259	8.076950877	9.607141034	9.74371185	10.92536393	11.12994454
ENSG00000067141	NEO1	2.170277047	0.20811252	3.74604617	0.019679078	5.916323217	2	8.213429689	6.43197674	9.166592134	9.14209426	10.563383	10.86744686
ENSG00000134046	MBD2	3.183282449	0.065440948	2.731607821	0.114871963	5.91489027	2	4.93638933	7.858115942	9.575477782	9.60612566	8.994997676	9.275296471
ENSG00000136021	SCYL2	3.258479898	0.007752174	2.655267129	0.032913606	5.913747027	4.00722531	8.004992147	5.930865648	10.00572181	10.043513	9.567578199	9.265398973
ENSG00000100311	PDGFB	1.276809956	0.610415582	4.636624124	0.028655643	5.913434079	2	2	2	2.868578001	2.49394783	5.108342547	4.594883674
ENSG00000153179	RASSF3	2.418292721	2.77E-07	3.494846124	2.44E-14	5.913138845	6.009025997	7.051611315	7.163363831	9.193296408	9.22514727	10.3869745	10.16599298
ENSG00000073060	SCARB1	1.86662175	0.352079152	4.046334955	0.026558185	5.912956704	3.327711234	7.467835385	2	7.694616532	7.91663606	9.751450819	10.15873961
ENSG00000137941	TTLL7	2.725817057	0.005157524	3.185969928	0.000801746	5.911786986	4.467311984	3.076665209	4.058998049	6.321555392	6.25300433	7.086154752	6.248307987

ENSG00000104267	CA2	1.494607862	0.683257266	4.412645119	0.170346935	5.907252981	2	2	2	2.600425781	2.94009902	5.545162502	2.225841198
ENSG00000068305	MEF2A	2.349257773	0.152743092	3.55593196	0.021881678	5.905189732	7.384433967	8.169643011	2	9.590237441	9.57248057	10.81094636	10.7560816
ENSG00000115594	IL1R1	4.604721595	5.26E-07	1.299970236	0.211023502	5.90469183	5.330594134	7.60566235	8.486635645	12.19878022	12.2084944	8.888451287	8.93066256
ENSG00000204217	BMPR2	2.772322312	0.002123869	3.130273687	0.000412201	5.902595998	7.384433967	9.192779271	6.718950348	10.9554005	10.8890776	11.31638534	11.24249341
ENSG00000162614	NEXN	2.541537893	0.002036933	3.354437516	2.58E-05	5.895975409	7.485143553	7.002537973	4.874587708	9.344822471	9.28397021	10.09608847	10.15080456
ENSG00000125249	RAP2A	2.796585355	0.003671412	3.099131879	0.001066317	5.895717234	6.653098118	6.311476754	3.369317972	8.70652031	8.72559535	9.215280081	8.784747976
ENSG00000127947	PTPN12	4.001090442	2.04E-07	1.893493481	0.022344999	5.894583923	6.653098118	8.451443573	6.629488387	11.48981601	11.5153943	9.646549955	9.106387289
ENSG00000243156	MICAL3	2.300318278	0.109150595	3.592299439	0.007741661	5.892617718	2	7.099070191	6.629488387	8.567402595	8.57772939	9.649231086	10.03603692
ENSG00000243649	CFB	11.07974309	5.21E-15	-5.191150855	0.022950398	5.88859223	2	2	2	11.06401437	11.1521225	2	2
ENSG00000164296	TIGD6	3.07832547	0.04116967	2.809491032	0.061814261	5.887816502	2	5.131603625	2	6.622948477	6.63721363	6.353989995	6.392053169
ENSG00000167700	MFSD3	2.651890779	0.095299137	3.235355547	0.034772335	5.887246326	3.327711234	6.311476754	2	7.348537858	7.62891439	7.800194554	8.294346816
ENSG00000173706	HEG1	1.767576402	0.210659147	4.119549466	0.001171726	5.887125868	4.467311984	9.267380431	8.784770914	10.24443813	10.253992	12.29485156	12.8473242
ENSG00000133818	RRAS2	2.305716272	0.063703589	3.580783744	0.002227172	5.886500015	7.33131396	7.538394143	3.369317972	9.213506984	9.12531607	10.14664362	10.68242029
ENSG00000166707	ZCCHC18	2.595332244	0.294006549	3.287979782	0.16118623	5.883312026	2	2	2	3.227757282	3.45167086	3.930690651	3.660199087
ENSG00000170185	USP38	3.007978735	0.100023481	2.8740986	0.111705957	5.882077335	2	7.638155693	4.058998049	9.159317259	9.13791797	9.092065441	8.93516457
ENSG00000165997	ARL5B	3.169004983	0.005967521	2.70901109	0.020479608	5.878016072	3.327711234	6.844429755	4.874587708	8.81895723	8.74905888	8.582068017	8.02265879
ENSG00000147614	ATP6V0D2	5.216132875	0.009388115	0.661446867	0.785803002	5.877579742	3.327711234	2	4.058998049	7.764774849	7.85944752	4.487545924	2.117335275
ENSG00000073331	ALPK1	2.913539041	3.37E-05	2.963151474	2.10E-05	5.876690516	4.815609471	4.11352228	4.058998049	6.965655398	7.03768431	7.172727426	6.915965441
ENSG00000170832	USP32	3.296694244	0.03895919	2.577986874	0.111493496	5.874681119	2	7.874700259	5.930865648	9.891822146	9.90230962	9.121501891	9.240556116
ENSG00000154229	PRKCA	1.392685249	0.088331632	4.479636238	4.59E-10	5.872321486	5.095959458	7.145017454	6.629488387	7.860161659	7.91175216	10.48764377	11.30189717
ENSG00000131845	ZNF304	2.737293584	0.004826128	3.133450689	0.000982659	5.870744273	3.327711234	5.946638857	4.058998049	7.433701013	7.58663873	7.927733561	7.873223515
ENSG00000137075	RNF38	2.774525904	0.058330657	3.09394138	0.030778033	5.868467283	3.327711234	6.951736442	3.369317972	8.310225403	8.2657098	8.693004369	8.508863136
ENSG00000165244	ZNF367	2.081434466	0.421293524	3.774888307	0.109758105	5.856322773	2	2	2	3.023133209	3.08597292	3.632645806	4.553838275
ENSG00000099812	MISP	1.392695656	0.580922984	4.461242112	0.039231993	5.853937768	2	2	2	2.947924294	2.49394783	4.727866721	4.720747915
ENSG00000147059	SPIN2A	4.631269401	0.020403301	1.218325855	0.593116818	5.849595256	2	2	2	4.76150579	4.97065356	2.592885947	2.746601469
ENSG00000197892	KIF13B	2.816345506	0.030340464	3.032717791	0.017911792	5.849063297	2	4.710564154	6.43197674	7.805288479	8.07355593	8.10721774	8.208518621
ENSG00000104689	TNFRSF10A	4.025529351	4.10E-06	1.822898921	0.053995487	5.848428273	3.327711234	3.076665209	5.156626401	7.697489359	7.84670947	5.684753614	5.641643428
ENSG00000065060	UHRF1BP1	2.229328004	0.026657522	3.618867056	0.000131309	5.84819506	5.095959458	7.145017454	4.523675391	8.321442665	8.15847021	9.743945072	9.484290996
ENSG00000077420	APBB1IP	4.247776626	0.00464393	1.595577343	0.33487913	5.843353969	4.00722531	3.076665209	2	6.722207358	6.79406287	4.971696071	3.287118176
ENSG00000124882	EREG	6.349544106	1.76E-30	-0.507425928	0.462478098	5.842118178	5.532353208	4.11352228	4.874587708	11.06039244	11.0379776	4.461156421	4.489993931
ENSG00000162757	C1orf74	2.824351946	0.040592042	3.015224656	0.025868544	5.839576602	2	4.11352228	2	5.304221918	5.39133044	5.606648123	5.431121909
ENSG00000183530	PRR14L	2.659643464	0.019452444	3.17946183	0.004097253	5.839105294	4.00722531	7.761270542	6.322058544	9.265734736	9.39662469	9.937031556	9.756873659
ENSG00000263464	PPIAL4C	7.901226884	5.51E-05	-2.06401995	0.405294552	5.837206934	2	2	2	7.939959586	7.96222667	2	2.172608095
ENSG00000130988	RGN	-0.72409335	0.778992908	6.559111261	0.000322101	5.835017911	2	2	2	2.270786764	2.14022021	5.919306827	7.124815549
ENSG00000078369	GNB1	2.613953308	0.000485712	3.215577907	1.12E-05	5.829531214	8.285626337	10.39216648	10.11951029	12.45409166	12.4656716	12.99790049	13.12177191
ENSG00000116819	TFAP2E	2.898715294	0.253395005	2.930395393	0.237344892	5.829110687	2	2	2	3.515101962	3.55551834	3.851628042	3.183290849
ENSG00000140548	ZNF710	2.937295545	0.044706135	2.890810076	0.046006971	5.828105621	3.327711234	6.951736442	3.369317972	8.491639362	8.40429221	8.607356811	8.164036804
ENSG00000138642	HERC6	6.454049373	5.44E-06	-0.627129984	0.72430187	5.826919388	3.327711234	6.535001748	2	11.47879537	11.4830405	4.652122235	4.644600858
ENSG00000197134	ZNF257	2.214045646	0.392182361	3.612566179	0.130326951	5.826611824	2	2	2	3.227757282	3.01487888	4.388329244	3.580696614
ENSG00000075702	WDR62	2.61230771	0.199709404	3.214269274	0.099251185	5.826576983	3.327711234	2	2	4.268917409	3.69849104	3.930690651	4.886954834
ENSG00000213999	MEF2B	5.503574515	0.001640812	0.321158918	0.888753558	5.824733434	2	2	2	5.709096143	5.58783529	2.42098416	2.374681325
ENSG00000120328	PCDHB12	2.939474669	0.248121056	2.884474021	0.247980941	5.82394869	2	2	2	3.79808662	3.28037825	3.256555576	3.753786374
ENSG00000197081	IGF2R	2.99987186	8.12E-05	2.823244421	0.000213322	5.823116281	10.02639697	8.077869079	8.434763955	12.09937748	12.1001021	11.82446067	12.01576379
ENSG00000178297	TMPRSS9	3.243893455	0.183184126	2.579037228	0.290945022	5.822930683	2	2	2	3.754611435	3.7864959	3.534812207	3.100202476
ENSG00000062582	MRPS24	4.405278527	9.11E-08	1.416682774	0.123888817	5.8219613	4.815609471	4.710564154	4.058998049	8.75144642	8.71161289	5.06226682	6.33565646
ENSG00000204172	AGAP9	4.365074667	0.001280898	1.456502569	0.336305876	5.821577236	2	5.131603625	2	7.930222906	7.85181819	5.108342547	5.1574003
ENSG00000172379	ARNT2	0.450268134	0.691119414	5.368131248	1.00E-09	5.818399382	4.467311984	6.844429755	4.874587708	6.328984825	6.17362678	10.72933238	11.38843349
ENSG00000146373	RNF217	2.329821478	0.323812042	3.487988312	0.117523606	5.817809791	2	7.35507212	2	8.197043419	7.99722844	9.555675374	8.854012253
ENSG00000169504	CLIC4	2.434116611	0.002123425	3.379900085	9.67E-06	5.814016696	8.696033109	11.00402286	10.21486071	12.72860514	12.6363624	13.61559199	13.64144911
ENSG00000168675	LDLRAD4	0.212583897	0.925360182	5.60116025	0.00097184	5.813744147	2	4.11352228	2	3.840289928	2.38539787	8.189226297	7.64187159
ENSG00000073008	PVR	2.436187983	2.42E-05	3.376555406	1.76E-09	5.812743389	7.159102577	8.540087354	8.35328246	10.55911051	10.5554386	11.203095	11.7390458
ENSG00000128274	A4GALT	2.169811609	0.333515024	3.642594602	0.081866442	5.812406212	2	3.076665209	8.206335842	8.759716509	8.84055903	10.11154794	10.40399898
ENSG00000135677	GNS	3.012561827	3.62E-06	2.796413439	1.82E-05	5.808975266	8.653385977	10.19331737	8.784770914	12.38047484	12.4206128	12.15680513	12.21207488
ENSG00000126456	IRF3	3.141237377	0.051321923	2.667682949	0.09933964	5.808920326	5.095959458	7.572420273	2	9.275380474	9.40272932	8.856747125	8.883817772
ENSG00000158859	ADAMTS4	4.709533829	5.90E-19	1.096846141	0.065488624	5.806379969	5.095959458	6.666836837	6.322058544	10.80535243	10.8420389	7.332904981	7.154291015
ENSG00000173267	SNCG	3.073446104	0.059053709	2.728867184	0.093025295	5.802313287	2	3.686303414	2	4.739373666	5.3193943	4.659878161	4.853519801
ENSG00000184292	TACSTD2	2.938642954	0.232847072	2.85852276	0.237776037	5.797165714	2	2	2	3.709785322	3.39680241	3.411599105	3.600986722
ENSG00000143322	ABL2	3.834974644	2.54E-08	1.961458758	0.007316377	5.796433402	8.042902253	8.876041558	6.882775709	11.95139166	11.9952166	9.880484031	10.29710605
ENSG00000154930	ACSS1	2.367139927	0.046591905	3.428834062	0.002286691	5.795973989	2	4.11352228	3.369317972	5.334065616	5.34860058	6.615624322	5.964937606
ENSG00000198554	WDHD1	3.247707878	0.001493487	2.543171185	0.015108153	5.790879063	3.327711234	4.710564154	2	6.693715713	6.67228943	6.176128386	5.829625997
ENSG00000100201	DDX17	2.711253137	0.000139786	3.079551563	1.13E-05	5.7908047	7.940205782	9.949724236	9.083172337	11.92329082	11.9103827	12.41400745	12.14288977
ENSG00000187498	COL4A1	1.634787047	0.038349318	4.153876789	6.19E-09	5.788663837	11.09308737	12.91590856	11.51057165	13.69244595	13.7010937	16.49368867	15.87054846
ENSG00000163935	SFMBT1	3.118196222	0.029640097	2.670134479	0.064397771	5.788330702	2	4.93638933	2	6.542151749	6.40670796	6.043083161	6.059610627
ENSG00000169258	GPRIN1	3.063531247	0.078250731	2.721497317	0.116627441	5.785028564	2	5.595927442	2	6.902323594	7.21862736	6.975527754	6.454714037
ENSG00000144218	AFF3	1.810346532	0.094524523	3.971292992	4.86E-05	5.781639524	2	5.303523747	5.930865648	6.777556219	6.84106088	8.795135904	9.053535482
ENSG00000159921	GNE	2.746171259	1.96E-11	3.035078946	6.55E-14	5.781250205	6.138360429	5.838937808	6.322058544	8.740345368	8.7935444	8.960872516	9.141009026
ENSG00000166147	FBN1	2.22421739	0.030773929	3.556347823	0.000245067	5.780565213	10.48735708	12.47802568	9.624627483	13.57375396	13.6090065	14.84878204	14.99421534
ENSG00000110046	ATG2A	3.407096321	0.000107002	2.372733448	0.009191319	5.77982977	6.009025997	6.951736442	4.058998049	9.385472425	9.51245165	8.211166197	8.610562542
ENSG00000124635	HIST1H2BJ	5.301595587	2.04E-06	0.477543959	0.735593063	5.779139547	2	4.11352228	2	7.756533788	7.70710533	3.429858444	3.580696614
ENSG00000158201	ABHD3	3.622423252	0.011003457	2.156041648	0.148400754	5.778464901	2	4.442710129	2	6.548530093	6.31350454	5.445480974	4.594883674
ENSG00000133216	EPHB2	5.211800756	2.39E-07	0.566545163	0.651103372	5.778345919	6.257048221	5.303523747	2	10.37802153	10.404557	5.951260634	5.73181021
ENSG00000152583	SPARCL1	2.861160538	0.327927671	2.914807801	0.30681403	5.77596834	2	2	2	3.709785322	3.28037825	4.099885429	2.632958241
ENSG00000162636	FAM102B	2.915113487	0.127032028	2.860181378	0.128290491	5.775294866	2	6.04685525	2	7.494428494	7.23826702	7.542503814	7.052989523
ENSG00000176165	FOXG1	5.97491672	0.056914368	-0.201051524	0.959149058	5.773865196	2	2	2	6.113598934	6.06344184	2.526562374	2
ENSG00000182979	MTA1	2.659250883	0.036049949	3.114568802	0.011726998	5.773819685	3.327711234	7.393647515	5.39244166	8.859280876	8.76132797	9.326359826	9.196649491
ENSG00000114999	TTL	2.44045415	0.037564908	3.332080035	0.002992423	5.772534185	7.384433967	7.099070191	3.369317972	9.074566443	9.15041075	10.04035872	9.958055169
ENSG00000198814	GK	6.866756812	1.61E-06	-1.096499397	0.524822542	5.770257414	2	5.72254323	2	11.00610223	10.9424344	3.517841605	3.660199087
ENSG00000171346	KRT15	3.570888536	0.175625164	2.197497131	0.419359474	5.768385668	2	2	2	3.840289928	4.16043394	2.560105256	3.518055793
ENSG00000165030	NFIL3	3.196756798	0.045765337	2.570519391	0.111954789	5.767276189	4.00722531	6.951736442	2	8.697938662	8.69749358	8.164223608	7.988411589
ENSG00000215472	RPL17-C18or	10.95339604	1.38E-14	-5.191150878	0.023227301	5.762245166	2	2	2	11.05396204	10.9075945	2	2
ENSG00000162923	WDR26	3.03455677	0.001104484	2.724867992	0.003635849	5.759424762	6.138360429	8.88960062	8.978369997	11.44179676	11.5006301	11.13212295	11.19186952
ENSG00000147454	SLC25A37	3.80552222	6.67E-06	1.952306112	0.030734544	5.757828332	5.330594134	8.029695761	7.734844356	11.19759955	11.207224	9.395058233	9.314988993
ENSG00000100364	KIAA0930	2.735092031	0.006672564	3.022721244	0.002294707	5.757813275	7.667647223	7.73146271	4.523675391	9.910512694	9.89118718	10.19064296	10.18417862
TMEM44		2.973032158	0.036596823	2.783158572	0.049531896	5.756190729	2	4.93638933	6.882775709	8.559529208	8.4936155	8.303800769	8.373881215
ENSG00000144355	DLX1	5.522779477	0.005659011	0.230092795	0.930138959	5.752872272	2	2	2	5.742861563	5.58783529	2.183373728	2.551896972
ENSG00000137094	DNAJB5	2.567271209	0.022996195	3.181311626	0.00364531	5.748582835	4.467311984	4.11352228	6.882775709	8.063125866	8.25224987	8.354863925	9.088535264
ENSG00000271147	ARMCX5-GPI	4.155408393	6.14E-05	1.590491059	0.16435224	5.745899452	2	3.686303414	4.058998049	7.048994596	7.06873058	4.933813368	4.400220394
ENSG00000172046	USP19	2.72501915	0.116680459	3.018608495	0.074520332	5.743627645	4.467311984	7.538394143	2	8.792330793	8.83669913	9.249735981	8.946110698
ENSG00000107036	RIC1	2.383495329	0.063345312	3.359203354	0.005993894	5.742698683	3.327711234	7.35507212	7.777116083	9.340234287	9.42346554	10.21848193	10.47768733
ENSG00000003402	CFLAR	2.83145974	2.38E-05	2.910945783	1.24E-05	5.742405524	8.075571721	7.669933291	9.330306409	11.35621574	11.350135	11.20077081	11.63219444
ENSG00000072062	PRKACA	2.312155079	0.004214141	3.429141488	9.16E-06	5.741296567	5.532353208	7.60566235	7.896971941	9.577818317	9.63233954	10.54922865	10.86966979
ENSG00000106733	NMRK1	3.46404664	0.02530452	2.275430503	0.155557022	5.739477143	2	5.303523747	2	7.19843704	7.14562489	6.049001768	6.018717758
ENSG00000085982	USP40	3.21416886	9.14E-05	2.522074019	0.002647154	5.736242879	5.095959458	6.666836837	4.523675391	8.859280876	8.90710092	7.897710349	8.44785952
ENSG00000110756	HPS5	2.084421352	0.093501828	3.649971246	0.001511468	5.734392598	5.70932728	7.73146271	4.058998049	8.595396228	8.6202631	9.783088106	10.46442481
ENSG00000180938	ZNF572	2.7336337	0.27254373	3.000726037	0.212779129	5.734359737	2	2	2	3.406930997	3.45167086	3.738983888	3.45257121
ENSG00000137414	FAM8A1	2.349379819	0.223030005	3.381278064	0.064416773	5.730657883	4.815609471	8.276714465	2	9.16866395	9.1347778	10.43083967	9.872489883
ENSG00000133937	GSC	5.038445434	0.006880061	0.691754231	0.761301217	5.730199665	2	2	2	5.273747807	5.18011485	2.526562374	2.466008509
ENSG00000184402	SS18L1	2.94451219	0.000827513	2.782033699	0.001598036	5.726545889	4.467311984	6.228547981	4.058998049	8.128448191	8.08878133	8.206540078	7.634508409
ENSG00000120306	CYSTM1	2.603717031	0.09775828	3.12091107	0.039779899	5.724628101	5.330594134	7.431218269	2	8.722121967	8.73530306	9.228053138	9.255433104
ENSG00000197694	SPTAN1	2.807911015	0.004062414	2.915644207	0.00262317	5.723555223	6.89194353	10.00059544	9.385998894	12.0444476	12.036184	11.97417317	12.30302946
ENSG00000164741	DLC1	1.938587204	0.298439087	3.784266443	0.026791677	5.722853647	3.327711234	9.00621767	4.874587708	9.476928206	9.42174886	11.47316881	11.07852969
ENSG00000158470	B4GALT5	3.133790741	0.000211037	2.58862914	0.002611125	5.722419881	5.330594134	7.701025996	7.97167868	10.48289882	10.4848496	9.947565107	9.933514329
ENSG00000176532	PRR15	1.274449266	0.656481504	4.446228641	0.070664362	5.720677906	2	2	2	2.695456494	2.68924543	5.367107572	3.474731315
ENSG00000175518	UBQLNL	3.181502112	0.188518397	2.533718569	0.295652928	5.715220681	2	2	2	3.754611435	3.69849104	3.374372158	3.236138336
ENSG00000145901	TNIP1	3.132466063	5.11E-12	2.576989944	1.75E-08	5.709456007	9.097018025	9.225224669	9.344432377	12.32361727	12.3748777	11.19677777	12.21593427
ENSG00000120137	PANK3	1.945919721	0.351874922	3.762469346	0.050135255	5.708389068	4.00722531	8.276714465	2	8.657201865	8.73806474	10.59987492	10.40291596
ENSG00000007933	FMO3	1.586482027	0.370269344	4.118769091	0.008770914	5.705251118	2	4.710564154	2	4.694064037	4.95174433	7.786089373	6.124122661

ENSG00000129657	SEC14L1	1.978139911	0.184717964	3.721841555	0.006750332	5.699981466	3.327711234	7.928237109	8.585098385	9.706743577	9.67187198	11.00991086	11.75207515
ENSG00000111269	CREBL2	2.728266545	0.058804128	2.970717133	0.035621047	5.698983678	7.33131396	6.140560021	2	8.970341944	8.95060997	9.030205459	9.353992291
ENSG00000170396	ZNF804A	7.140845344	3.05E-06	-1.442201161	0.468753938	5.698644183	2	2	2	7.242377933	7.17037177	2.139668783	2.117335275
ENSG00000129538	RNASE1	-0.7313856	0.818399248	6.41697834	0.006572253	5.685592741	2	2	2	2	2.38539787	6.693258166	6.29575846
ENSG00000124225	PMEPA1	1.527130177	0.056262369	4.157930164	6.56E-09	5.685060341	5.095959458	6.951736442	7.398165132	8.370868225	8.13979554	10.91618505	10.83234501
ENSG00000178919	FOXE1	4.405994612	0.001807656	1.276663541	0.425335565	5.682658153	2	4.93638933	2	7.66556918	7.77322015	5.163941955	4.279527956
ENSG00000124208	TMEM189-U	10.87265855	2.05E-14	-5.191150959	0.023163678	5.681507594	2	2	2	10.85861994	10.9442293	2	2
ENSG00000262418		1.389352449	0.620673635	4.291808583	0.077889486	5.681161032	2	2	2	2.695456494	2.77779907	5.180212365	3.539239711
ENSG00000085449	WDFY1	2.913905438	0.053258105	2.763671404	0.064987892	5.677576841	3.327711234	8.777382925	6.629488387	10.41601627	10.395402	10.27922234	10.23232731
ENSG00000172159	FRMD3	4.119131334	0.043612511	1.557800054	0.483865109	5.676931388	2	3.076665209	2	5.128039203	5.39133044	3.767980144	2.592996859
ENSG00000116406	EDEM3	3.348143684	0.006716746	2.319752523	0.06941259	5.667896208	5.095959458	8.505280662	5.59507254	10.51500864	10.5655798	9.80401941	9.156086127
ENSG00000117500	TMED5	3.579668181	0.000178186	2.088063833	0.038440494	5.667732014	5.330594134	8.029695761	6.203069917	10.58416277	10.4528466	9.338190203	8.651871454
ENSG00000095752	IL11	4.09474139	0.000183325	1.566545523	0.193576	5.661286912	7.790810999	6.311476754	4.058998049	10.74623244	10.7874282	7.811554331	8.592942424
ENSG00000049246	PER3	2.255840863	0.007490024	3.402847196	2.10E-05	5.658688059	4.815609471	4.710564154	2	6.268449691	6.39974839	7.317051196	7.558732759
ENSG00000213139	CRYGS	3.049919775	0.245300741	2.606857701	0.318173125	5.656777476	2	2	2	3.95996233	3.2184422	3.584558147	3.071407821
ENSG00000225828	FAM229A	2.541294475	0.12116737	3.113294565	0.047946397	5.65458904	2	3.686303414	2	4.623314311	4.58534546	5.279204176	4.886954834
ENSG00000170471	RALGAPB	2.406662217	0.062355471	3.247828276	0.008609548	5.654490493	4.815609471	8.557180776	5.930865648	9.685196338	9.64710779	10.65694047	10.33356098
ENSG00000282230		2.933899382	0.03884132	2.719490378	0.054838286	5.65338976	4.467311984	9.814097497	9.06622548	11.88757367	11.8120795	11.36330635	11.86550852
ENSG00000047936	ROS1	2.209989299	0.435112068	3.443201777	0.190011047	5.653191075	2	2	2	2.784612485	3.39680241	3.256555576	4.365060851
ENSG00000174705	SH3PXD2B	3.214799262	0.02248445	2.433423726	0.091103426	5.648222989	6.009025997	9.357954881	5.156626401	11.18971483	11.1670574	10.18477494	10.58519509
ENSG00000271605	MILR1	4.544557479	0.00113016	1.103529743	0.493413792	5.648087222	2	2	4.058998049	6.529309835	6.50077304	3.679182183	3.496556178
ENSG00000105509	HAS1	5.844374058	0.000699054	-0.200552797	0.932012683	5.643821262	2	2	2	5.987823101	5.94414054	2.34604626	2.225841198
ENSG00000224470	ATXN1L	2.541358187	0.130186997	3.097609919	0.055730923	5.638968106	6.73718411	8.297207175	2	9.618553696	9.70330142	10.17584339	10.25411154
ENSG00000155380	SLC16A1	3.467886792	0.000211964	2.167459181	0.027439899	5.635345973	5.330594134	8.395519306	7.342949392	10.97595466	10.8983658	9.7324978	9.544459294
ENSG00000198742	SMURF1	2.963774581	6.81E-08	2.670364072	1.28E-06	5.634138653	6.257048221	7.538394143	6.43197674	9.778365132	9.83360578	9.511886139	9.516692718
ENSG00000088899	LZTS3	1.834573145	0.341039924	3.798008134	0.03100898	5.632581279	3.327711234	7.145017454	2	7.487805859	7.45518898	9.03282106	9.709771424
ENSG00000197713	RPE	3.021683493	0.085482239	2.610315281	0.136341225	5.631998773	4.00722531	7.393647515	2	8.941551548	8.91810182	8.5805365	8.461794641
ENSG00000260873		2.19199165	0.005020885	3.439745829	3.70E-06	5.631737479	5.330594134	6.951736442	7.646393253	9.117027752	9.03500236	10.48982111	10.12323121
ENSG00000168916	ZNF608	1.733502694	0.489660234	3.897990894	0.083545957	5.631493587	2	3.076665209	2	3.95996233	2.49394783	5.565950232	4.30448772
ENSG00000103335	PIEZO1	2.717391927	0.058485499	2.9112882	0.038684826	5.628680127	9.435834703	8.834582582	4.058998049	11.28279734	11.3321984	11.37586927	11.61670776
ENSG00000081377	CDC14B	2.434161315	0.117408639	3.193748953	0.032070986	5.627910268	3.327711234	7.145017454	3.369317972	8.087422161	8.16464176	8.701014095	9.035013043
ENSG00000075073	TACR2	2.331786228	0.367465205	3.29543827	0.17543064	5.627224498	2	2	2	3.023133209	3.33976443	4.017766949	3.560117069
ENSG00000178623	GPR35	2.973017037	0.356981191	2.649978549	0.40736792	5.622995585	2	2	2	3.349643765	3.7864959	3.993419637	2.277179783
ENSG00000110367	DDX6	2.893727756	5.66E-18	2.727712449	2.86E-16	5.621440206	8.042902253	7.503546079	8.143093325	10.75073417	10.8131925	10.63140433	10.6018087
ENSG00000078081	LAMP3	2.935635988	0.17616338	2.685448169	0.211125211	5.621084157	2	2	4.058998049	5.110902178	5.06165168	5.598599872	3.311948089
ENSG00000104365	IKBKB	2.896582202	0.150100354	2.722632223	0.170797377	5.619214425	3.327711234	7.790474954	2	9.122382231	9.15558431	8.977260248	8.955497794
ENSG00000175073	VCPIP1	3.008453781	0.025684466	2.609605731	0.054660118	5.618059512	2	6.666836837	6.882775709	9.160358775	9.16485023	8.914619542	8.602408528
ENSG00000164151	ICE1	2.649276038	0.021794347	2.962617153	0.008934398	5.61189319	6.257048221	7.847167909	4.058998049	9.401417034	9.34230452	9.768589685	9.593700566
ENSG00000101972	STAG2	3.366707267	0.114452129	2.242889143	0.304089967	5.60959641	3.327711234	8.414402626	2	10.21617036	10.2188359	9.256785236	8.922624134
ENSG00000165724	ZMYND19	2.449623303	0.150422971	3.157641846	0.052769782	5.607265149	3.327711234	6.602424674	2	7.593372273	7.53675633	8.469076579	8.018893446
ENSG00000166855	CLPX	2.91358492	0.221769007	2.691354644	0.255544364	5.604939564	2	7.669933291	2	9.006698952	8.97890371	8.865577373	8.672989404
ENSG00000155313	USP25	2.301322347	0.27254373	3.30165937	0.096358916	5.602981716	2	7.901717006	3.369317972	8.752828065	8.5575471	9.879446421	9.381010503
ENSG00000120049	KCNIP2	2.815147938	0.129045271	2.787000424	0.125279255	5.602148362	3.327711234	2	2	4.17310023	4.16043394	4.155805465	4.133950982
ENSG00000107331	ABCA2	2.05885584	0.16074342	3.541646658	0.009371668	5.600502498	3.327711234	6.844429755	8.460932916	9.286870873	9.39487575	10.89467473	10.73901752
ENSG00000214753	HNRNPUL2	3.353748635	0.001883357	2.246698126	0.045247931	5.600446761	7.53297911	7.761270542	4.058998049	10.45090952	10.4465287	9.237154294	9.44799795
ENSG00000074054	CLASP1	2.929962661	1.20E-11	2.669971733	6.91E-10	5.599934395	7.579279349	6.602424674	7.285536013	10.09449002	10.0842356	9.948159052	9.702160718
ENSG00000155085	AK9	2.27937321	0.036303182	3.319362898	0.001236986	5.598736109	4.00722531	3.686303414	2	5.210797711	5.36298494	6.234425698	6.267475858
ENSG00000184524	CEND1	2.691435164	0.301726535	2.907140995	0.249167678	5.598576159	2	2	2	3.615725078	3.15372745	3.316666208	3.735548302
ENSG00000170802	FOXN2	3.273723562	2.66E-06	2.323939382	0.001196943	5.597662944	3.327711234	5.303523747	5.772716771	8.270263574	8.20711886	7.358152527	7.253757517
ENSG00000023516	AKAP11	2.661252011	0.000258801	2.934779274	4.45E-05	5.596031285	8.257427002	6.228547981	7.342949392	10.12735113	10.1233123	10.58442118	10.18375829
ENSG00000049656	CLPTM1L	3.448094924	0.001384172	2.146982464	0.057294878	5.595077388	6.257048221	8.213429689	4.874587708	10.49161431	10.4914114	8.956549551	9.401760658
ENSG00000175137	SH3BP5L	2.413317257	0.190541209	3.179306781	0.071210753	5.592624037	2	5.72254323	8.434763955	9.413697701	9.47155148	10.05550524	10.34130817
ENSG00000184206	GOLGA6L4	6.62100781	4.08E-06	-1.030000294	0.569078707	5.591007516	2	2	3.369317972	7.650823203	7.62593544	2.34604626	2.277179783
ENSG00000133063	CHIT1	-3.753087267	0.350187134	9.344072956	0.003068505	5.590985689	2	2	2	2	2	10.35403689	4.522269241
ENSG00000151327	FAM177A1	2.729347674	0.000546124	2.860647245	0.000252904	5.589994918	5.330594134	7.701025996	6.718950348	9.590237441	9.58096568	9.641172698	9.787626348
ENSG00000145675	PIK3R1	2.976007056	0.0296999	2.613292597	0.057302601	5.589299653	2	6.602424674	4.874587708	8.360030171	8.29788471	8.207201861	7.690021882
ENSG00000088836	SLC4A11	2.560743749	0.103124682	3.02808097	0.045706083	5.588824719	2	2	4.523675391	5.022023783	5.45984938	5.586442682	5.772630506
ENSG00000141084	RANBP10	2.931205783	0.106775299	2.65750796	0.140749923	5.588713743	2	7.099070191	3.369317972	8.486665614	8.5323134	8.278834759	8.198566699
ENSG00000006534	ALDH3B1	3.414418268	0.000735288	2.17214124	0.040046791	5.586559508	2	5.72254323	4.874587708	8.017874397	8.20112674	7.039353222	6.741885308
ENSG00000134198	TSPAN2	0.184200165	0.938887874	5.401221162	0.002674594	5.585421326	3.327711234	2	2	2.3892346	2.86123014	6.989444349	5.601925227
ENSG00000169118	CSNK1G1	3.251719729	1.30E-12	2.332255394	5.63E-07	5.583975123	5.866944937	6.78762695	6.322058544	9.58946437	9.58250309	8.845739021	8.476969394
ENSG00000264522	OTUD7B	2.910478405	0.036655526	2.672457496	0.054639343	5.582935901	2	6.844429755	7.029880454	9.167628414	9.29150981	8.947072423	9.040594787
ENSG00000175471	MCTP1	4.841905864	0.025688178	0.740958587	0.774520328	5.582864451	2	2	3.369317972	6.01571348	5.83486613	3.336159116	2.172608095
ENSG00000138119	MYOF	1.892786721	3.82E-05	3.688880545	4.02E-17	5.581667265	9.423197387	9.415325457	10.17589429	11.58461153	11.6216511	13.08110281	13.65771468
ENSG00000182472	CAPN12	3.081791088	0.210222627	2.499783037	0.308943128	5.581574126	2	2	2	3.663521627	3.65239003	3.235948948	3.33635788
ENSG00000010671	BTK	2.733626332	0.337258691	2.846344344	0.303980057	5.579970676	2	2	2	3.406930997	3.45167086	3.993419637	2.746601469
ENSG00000144283	PKP4	1.305403598	0.524942496	4.27378479	0.016877206	5.579188389	4.00722531	7.847167909	2	7.794595599	7.51115308	10.43423541	10.75424271
ENSG00000101096	NFATC2	3.172165154	0.101896165	2.405818139	0.219476281	5.577983293	2	3.076665209	2	4.670864177	4.1930041	4.133696865	3.518055793
ENSG00000165312	OTUD1	2.074448594	0.157322534	3.503336984	0.010210539	5.577785578	2	5.838937808	3.369317972	6.610808945	6.42052719	8.166267073	7.592058619
ENSG00000096717	SIRT1	2.815860417	0.053222658	2.758288345	0.055572955	5.574148762	3.327711234	6.844429755	3.369317972	8.227176542	8.23671175	8.492068113	7.768360547
ENSG00000114346	ECT2	3.291901158	1.42E-10	2.277617201	1.44E-05	5.569518359	5.095959458	5.838937808	5.772716771	8.909699622	8.68609779	7.985196707	7.589522245
ENSG00000099331	MYO9B	2.879869086	0.001137138	2.685649506	0.002488036	5.565518592	6.009025997	8.433041974	8.721235202	10.94938761	11.0071139	10.8686618	10.69560027
ENSG00000206559	ZCWPW2	4.09951821	0.05993045	1.464982238	0.540166698	5.564500448	2	2	2	4.523237872	4.31639476	2.717032197	2.817676617
ENSG00000023287	RB1CC1	2.702032789	0.000856796	2.856626364	0.000366438	5.558659153	6.468621203	8.607278467	7.225742779	10.40551335	10.3936516	10.83982598	10.19652354
ENSG00000171368	TPPP	0.495926608	0.854188222	5.061578326	0.013923728	5.557504933	2	2	2	2.3892346	2.49394783	5.677130623	4.634793542
ENSG00000100221	JOSD1	2.200952461	0.086717068	3.354612336	0.00554415	5.555564797	4.815609471	8.607278467	6.203069917	9.520570052	9.56082893	10.53914534	10.82697164
ENSG00000159403	C1R	3.618032772	1.56E-06	1.934602302	0.015816392	5.552635074	7.940205782	7.874700259	9.658943166	12.32698367	12.3696559	10.38170786	10.90592582
ENSG00000115165	CYTIP	2.897844812	0.330348729	2.653856994	0.366255977	5.551701805	2	2	2	3.406930997	3.65239003	3.917810786	2.509591799
ENSG00000121060	TRIM25	3.613433879	0.001384172	1.938050801	0.106833944	5.55148468	8.928409675	6.04685525	5.930865648	11.23367077	11.289066	9.532099314	9.648340472
ENSG00000166170	BAG5	2.496921937	0.121290859	3.052724042	0.049497365	5.549645979	4.00722531	7.790474954	3.369317972	8.81895723	8.82894823	9.276467981	9.468503442
ENSG00000249751	ECSCR	1.500040599	0.586949095	4.048838889	0.094671932	5.548879488	2	2	2	3.023133209	2.49394783	3.891700877	4.74829783
ENSG00000063660	GPC1	1.653900322	0.238657623	3.892539811	0.00205263	5.546440133	5.095959458	9.532315021	9.809100877	10.7322922	10.805643	12.97729211	13.03308733
ENSG00000013563	DNASE1L1	3.425892114	0.011026701	2.118529155	0.131976284	5.544421268	2	6.389896633	7.029880454	9.497678564	9.57942664	7.95714469	8.481764443
ENSG00000196367	TRRAP	2.727396513	4.03E-15	2.81614	3.01E-16	5.543536513	6.816637977	7.467835385	7.225742779	9.875425464	9.92793329	9.947565107	10.03183938
ENSG00000173230	GOLGB1	2.577932112	NA	2.964064584	NA	5.541996696	4.815609471	10.38739569	5.39244166	11.4550505	11.4421327	11.91327231	11.74582436
ENSG00000170835	CEL	4.163121868	0.084156162	1.378753002	0.603818931	5.541874871	2	2	2	4.139692846	4.74762503	2.987421008	2.421067459
ENSG00000146122	DAAM2	-1.12503576	0.44518671	6.662929251	2.74E-09	5.537893491	3.327711234	3.686303414	2	2.600425781	2.77779907	9.471416419	8.594208165
ENSG00000166548	TK2	2.945557797	0.03177678	2.59025197	0.059960937	5.535809767	6.816637977	7.051611315	2	9.244284335	9.29620212	8.917453109	8.918082759
ENSG00000130558	OLFM1	2.610979635	0.170726471	2.924434427	0.111958462	5.535414063	3.327711234	2	2	4.105493497	3.90918929	4.088436536	4.400220394
ENSG00000062524	LTK	1.692295777	0.524785881	3.840343301	0.104270596	5.532639078	2	2	2	3.09461492	2.59489879	4.122514202	4.30448772
ENSG00000174437	ATP2A2	2.703154829	2.27E-10	2.828553095	2.59E-11	5.531707924	8.797441463	9.799806309	9.301632639	12.05638564	12.0580365	12.09427683	12.26563573
ENSG00000198930	CSAG1	0.68600976	0.812768397	4.84562937	0.035498586	5.531639129	2	2	2	2.270786764	2.68924543	5.553513572	4.292061816
ENSG00000141503	MINK1	2.234302798	0.0437622	3.296404495	0.001696648	5.530707292	5.532353208	8.762721501	6.80318718	9.782429661	9.90722553	10.84163854	10.96570298
ENSG00000214595	EML6	3.011795918	0.238959909	2.51754657	0.323735478	5.529342488	2	2	2	3.663521627	3.55551834	3.534812207	3.012034821
ENSG00000108604	SMARCD2	2.80091221	0.102386938	2.727564534	0.106905881	5.528476745	4.467311984	7.467835385	2	8.716468139	8.92781046	8.863899583	8.633521012
ENSG00000115504	EHBP1	2.997137721	0.00405304	2.530368381	0.017000808	5.527506102	4.00722531	6.951736442	7.342949392	9.622337941	9.58250309	9.507594406	8.641501544
ENSG00000185499	MUC1	1.947067754	0.20711259	3.57924467	0.011950999	5.526312424	2	5.838937808	3.369317972	6.314087501	6.46787796	7.310906894	8.432373803
ENSG00000162595	DIRAS3	6.27082008	0.000120485	-0.745807928	0.725350511	5.525012152	2	2	2	6.401247806	6.33555364	2.183373728	2.225841198
ENSG00000152413	HOMER1	3.164063129	3.57E-05	2.36094193	0.002637744	5.525005059	3.327711234	5.838937808	5.156626401	8.113468729	8.19711812	7.24282551	7.482861322
ENSG00000213445	SIPA1	2.447889533	0.013097086	3.076793723	0.001269607	5.524683256	3.327711234	6.602424674	5.772716771	8.16211395	8.11451391	8.636638396	8.87395086
ENSG00000173744	AGFG1	2.726985891	0.024500134	2.797686515	0.020011794	5.524672406	3.327711234	7.315436877	7.502634626	9.548682898	9.56394525	9.633561473	9.619726259
ENSG00000130449	ZSWIM6	3.20407876	0.010286652	2.318534936	0.071123944	5.522613696	2	6.535001748	5.39244166	8.610716699	8.68036596	7.848504194	7.692387724
ENSG00000173621	LRFN4	2.2220966	0.194855335	3.298720075	0.041382233	5.520816675	2	6.602424674	3.369317972	7.437142666	7.24992376	8.4151065	8.393643421
ENSG00000041515	MYO16	0.68785149	0.790662721	4.831893387	0.018228014	5.519744877	2	2	2	2.3892346	2.59489879	4.778616857	5.269393099
ENSG00000070950	RAD18	1.977772244	0.156896095	3.541644188	0.006021251	5.519416433	3.327711234	5.72254323	2	6.252910529	6.39275508	7.945293431	7.726263462
ENSG00000141384	TAF4B	2.372126109	0.103759715	3.145380685	0.023910744	5.517506794	2	4.710564154	2	5.37770462	5.70538658	6.344396176	6.195912571
ENSG00000164236	ANKRD33B	1.226571436	0.499380069	4.288543146	0.006284419	5.515114581	3.327711234	6.602424674	2	6.358325776	6.40670796	8.964399877	9.706263779

ENSG00000085063	CD59	2.894523491	1.91E-30	2.619480507	2.46E-25	5.514003997	10.81695923	10.72273565	10.64969256	13.60617143	13.6397447	13.14923767	13.52287338
ENSG00000137413	TAF8	2.935440768	2.00E-06	2.578471146	3.35E-05	5.513911915	6.009025997	4.710564154	5.156626401	8.229163214	8.1187584	8.035995406	7.574209638
ENSG00000213859	KCTD11	2.233791027	0.012308962	3.275708455	0.000117075	5.509499482	4.815609471	7.315436877	6.073378139	8.556367783	8.66303256	9.308892875	9.917424452
ENSG00000185010	F8	2.769220555	0.091117194	2.735738088	0.090145733	5.504958643	2	5.303523747	2	6.503277904	6.49425375	6.558682816	6.368575325
ENSG00000196873	CBWD3	7.927594045	2.69E-08	-2.425390044	0.176769831	5.502204001	2	3.076665209	2	8.869506572	8.98356585	2.183373728	2.059860178
ENSG00000127481	UBR4	2.986241918	0.032193991	2.514522851	0.073848527	5.500764768	4.815609471	9.988044813	9.658943166	12.21465954	12.2824838	11.92080638	11.61880819
ENSG00000058404	CAMK2B	2.333025254	0.366470827	3.167661276	0.193719441	5.50068653	2	2	2	3.162721474	3.2184422	3.891700877	3.518055793
ENSG00000163815	CLEC3B	0.858631528	0.757769467	4.641014625	0.042085537	5.499646152	2	2	2	2.498691189	2.59489879	3.956110469	5.436777225
ENSG00000127526	SLC35E1	2.263278319	0.027309548	3.234385761	0.000941358	5.49766408	6.366709671	8.834582582	6.43197674	10.01265873	9.89614107	10.80593075	11.03281937
ENSG00000164620	RELL2	3.217101944	0.02572938	2.278994294	0.124033936	5.496096238	2	4.93638933	2	6.529309835	6.60731796	5.642319755	5.727202496
ENSG00000133789	SWAP70	2.394907539	0.031675145	3.100361006	0.003875548	5.495268545	5.532353208	8.004992147	5.156626401	9.176921588	9.20432875	9.634299801	10.11112449
ENSG00000150712	MTMR12	2.220415018	1.69E-05	3.270168357	5.48E-11	5.490583375	5.095959458	5.72254323	6.43197674	7.966401501	7.99029569	9.012519675	9.020021944
ENSG00000137101	CD72	2.813760808	0.167895393	2.676598198	0.181647286	5.490359007	3.327711234	2	2	3.95996233	4.3456612	4.45225155	3.518055793
ENSG00000140948	ZCCHC14	2.427148232	0.111655472	3.062435017	0.036695546	5.489583249	4.00722531	7.467835385	3.369317972	8.432517176	8.49198035	9.214621994	8.957957965
ENSG00000123384	LRP1	2.474409639	1.43E-06	3.014481284	2.38E-09	5.488890924	11.86992646	11.30403016	10.60345421	13.78321995	13.8597565	14.21286338	14.49704798
ENSG00000033178	UBA6	3.66686899	0.015236142	1.821562934	0.258826994	5.488431925	5.095959458	8.317412869	3.369317972	10.54761388	10.5628565	8.812201585	8.621156416
ENSG00000155304	HSPA13	2.960225964	0.106055103	2.528113709	0.167157761	5.488339674	5.866944937	8.623598428	2	10.21266037	10.1551727	9.961556635	9.51100768
ENSG00000110651	CD81	2.570449212	2.84E-06	2.916949514	7.42E-08	5.487398726	9.233563228	10.51097332	10.40208303	12.67030265	12.7675965	12.83409244	13.26589377
ENSG00000147050	KDM6A	3.382059104	0.007464915	2.102685481	0.111330307	5.484744585	4.815609471	7.274681896	3.369317972	9.372049302	9.2990102	8.1689872	7.962181894
ENSG00000108187	PBLD	2.745242651	0.017140458	2.735991764	0.016815901	5.481234415	2	4.93638933	3.369317972	6.358325776	6.42052719	6.043083161	6.654794245
ENSG00000184060	ADAP2	0.312646556	0.886720338	5.16646026	0.001313772	5.479106815	2	2	2	2.695456494	2.68924543	6.505870593	5.896817464
ENSG00000058056	USP13	2.290625527	9.84E-05	3.188297763	2.16E-08	5.47892329	5.330594134	6.899080544	6.203069917	8.481674661	8.59611189	9.314124483	9.538225362
ENSG00000177363	LRRN4CL	0.564937038	0.666023939	4.910062942	1.62E-06	5.47499998	4.467311984	3.076665209	3.369317972	4.070463721	4.22485516	7.422256313	8.62735196
ENSG00000168769	TET2	2.963479293	0.019862551	2.510613623	0.050724086	5.474092916	6.73718411	5.838937808	2	8.693628619	8.66883353	8.411091064	8.031095054
ENSG00000164713	BRI3	3.010812637	3.54E-11	2.462338596	8.03E-08	5.473151233	7.27616306	8.376385542	7.934808822	10.84993128	11.0071139	10.26530837	10.49286637
ENSG00000051596	THOC3	3.747204424	4.56E-08	1.719646309	0.020018787	5.466850733	4.467311984	4.93638933	3.369317972	7.908072101	7.95512324	6.078235975	5.863612864
ENSG00000134109	EDEM1	3.394792868	1.74E-24	2.070960513	8.66E-10	5.46575338	7.031698319	7.099070191	6.958202216	10.36045596	10.4179663	8.935537205	9.197066081
ENSG00000162704	ARPC5	2.522091804	3.19E-08	2.941562621	6.63E-11	5.463654425	8.468948322	9.114109157	8.076950877	11.18486086	11.0853755	11.40802991	11.68801764
ENSG00000104833	TUBB4A	5.142499084	0.000173358	0.32091598	0.856270394	5.463415064	3.327711234	3.076665209	2	7.012547947	7.07311172	2.803598391	3.33635788
ENSG00000133135	RNF128	5.173308602	0.044742745	0.288439355	0.929171616	5.461747957	2	2	2	5.363304456	5.33407135	2.656294034	2.059860178
ENSG00000143126	CELSR2	0.590709385	0.713873535	4.870684933	0.000158892	5.461394317	2	4.442710129	6.43197674	6.139181293	5.19627118	9.769486116	10.05155464
ENSG00000073417	PDE8A	3.320752865	0.008045241	2.140189903	0.100850653	5.460942768	3.327711234	7.538394143	5.59507254	9.604847628	9.63900396	8.550603056	8.343344553
ENSG00000108239	TBC1D12	2.158997807	0.135211488	3.301322654	0.015400933	5.46032046	2	6.535001748	4.523675391	7.454228844	7.33629421	8.604345457	8.430249176
ENSG00000197046	SIGLEC15	2.936704802	0.184884539	2.522271791	0.253508506	5.458976593	2	3.076665209	2	4.205751501	4.31639476	4.504874511	3.012034821
ENSG00000105329	TGFB1	2.47117129	0.004754286	2.983697162	0.000469751	5.454868452	7.27616306	9.018611864	9.926432745	11.55323749	11.5710504	11.991084	12.15264135
ENSG00000269713	NBPF9	5.787830879	1.43E-28	-0.334827077	0.614217941	5.453003802	6.138360429	6.535001748	7.552162999	12.56776544	12.6222308	6.497238296	6.572418365
ENSG00000101152	DNAJC5	2.281672594	0.027042585	3.169358695	0.001326571	5.451031289	7.867441304	9.246456519	6.073378139	10.46235554	10.5655798	11.26526767	11.52461319
ENSG00000106686	SPATA6L	3.495974669	0.143386081	1.954433183	0.432815399	5.450407852	2	2	2	3.840289928	4.05806467	2.937246078	3.042026731
ENSG00000147324	MFHAS1	2.649072062	0.227801845	2.800351543	0.191758699	5.449423604	2	6.535001748	2	7.641902724	7.5462416	8.348878903	6.678855578
ENSG00000078747	ITCH	2.573812601	2.92E-06	2.874033382	1.25E-07	5.447845982	6.366709671	7.669933291	7.225742779	9.723047898	9.74919792	10.28753805	9.728912497
ENSG00000174197	MGA	2.907059644	0.000344331	2.538695432	0.001976772	5.445755076	6.138360429	7.847167909	5.930865648	9.734282329	9.86046766	9.724430319	9.065448786
ENSG00000169896	ITGAM	0.274454513	0.927650433	5.170231574	0.024765019	5.444686087	2	2	2	2.3892346	2.38539787	6.040114727	3.956354764
ENSG00000099994	SUSD2	0.604850471	0.611488116	4.839308517	2.35E-07	5.444158988	4.00722531	2	4.523675391	4.358765249	4.02226117	8.010440524	8.187707412
ENSG00000142233	NTN5	1.150943712	0.701695664	4.291782511	0.093960346	5.442726223	2	2	2	2.784612485	2.49394783	5.233158834	3.36036153
ENSG00000105219	CNTD2	2.441176317	0.367367297	3.00028854	0.246966535	5.441464857	2	2	2	2.868578001	3.55551834	3.500668998	3.698365557
ENSG00000133138	TBC1D8B	2.659459529	0.152145189	2.781650241	0.125418384	5.44110977	3.327711234	7.145017454	2	8.427334995	8.12087597	8.44402928	8.360554398
ENSG00000115935	WIPF1	2.96104282	0.025780112	2.475491403	0.065354655	5.436534222	3.327711234	7.874700259	8.042706615	10.34771157	10.3278045	10.0527464	9.623450463
ENSG00000221968	FADS3	2.073721174	0.000262222	3.361482086	6.91E-10	5.43520326	7.867441304	6.951736442	8.380957451	9.874156428	9.90538403	10.99733953	11.33141801
ENSG00000274180	NATD1	1.506649228	0.101137093	3.928282252	1.61E-06	5.43493148	5.095959458	7.189546432	5.772716771	7.821180652	7.72670378	10.57470991	9.619415474
ENSG00000162851	TFB2M	2.983436939	0.020848047	2.450372458	0.060842073	5.433809397	2	5.595927442	3.369317972	7.202487431	7.17037177	6.708295923	6.632790505
ENSG00000176383	B3GNT4	2.497388939	0.342054331	2.930208374	0.246132276	5.427597313	2	2	2	3.349643765	3.2184422	3.782262616	3.287118176
ENSG00000151067	CACNA1C	1.67392066	0.004986804	3.751369507	9.98E-12	5.425290167	4.815609471	6.04685525	5.156626401	7.048994596	7.08183429	9.233585684	8.974090715
ENSG00000075292	ZNF638	2.882194767	0.001788082	2.5412719	0.006389948	5.423466667	5.330594134	8.234834549	7.451345288	10.30737895	10.2707772	10.15969431	9.703334212
ENSG00000117868	ESYT2	1.904272384	0.086760878	3.516755737	0.000593789	5.421028121	5.532353208	8.376385542	9.049077186	10.11773201	10.1463641	11.73470634	11.74753181
ENSG00000071127	WDR1	1.49150699	4.72E-05	3.928974218	7.00E-30	5.420481208	10.34051281	9.896993485	9.849281147	11.52359623	11.5434907	13.69695759	14.19857858
ENSG00000170579	DLGAP1	0.28191919	0.945086484	5.138339023	0.122752719	5.420258213	2	2	2	2.695456494	2	4.881704255	5.651404445
ENSG00000100399	CHADL	2.011409107	0.437765112	3.405585524	0.151541851	5.41699463	2	2	2	3.023133209	3.01487888	3.796405078	3.972018587
ENSG00000177051	FBXO46	1.882593899	0.046185884	3.532565859	4.87E-05	5.415159757	4.815609471	5.457120953	2	6.456560224	6.42052719	8.038228667	8.046896864
ENSG00000137462	TLR2	1.597880247	0.562650016	3.815463293	0.12122066	5.41334354	2	2	2	2.947924294	2.68924543	4.636484163	3.560117069
ENSG00000164715	LMTK2	2.264274195	0.213542763	3.147651539	0.069071834	5.411925735	2	4.11352228	7.451345288	8.211183901	8.21705076	8.978035992	9.191222844
ENSG00000181804	SLC9A9	3.812646118	0.007212171	1.599206258	0.299320086	5.411852376	2	4.93638933	2	7.136275664	7.14977896	4.771475226	5.319231103
ENSG00000143067	ZNF697	4.18292092	0.008608975	1.228315036	0.492372773	5.411235956	3.327711234	6.389896633	2	9.081180848	9.06939887	6.730562686	5.319231103
ENSG00000124151	NCOA3	2.670558104	0.0056444	2.739494593	0.004207821	5.410052696	5.095959458	7.954278496	8.076950877	10.12788365	10.2123959	10.30124027	10.17490298
ENSG00000101938	CHRDL1	2.148910113	0.448379821	3.258585084	0.216645944	5.407495196	2	2	2	3.09461492	3.08597292	4.240999983	3.100202476
ENSG00000175662	TOM1L2	1.861094751	0.299070637	3.545303627	0.031372002	5.406398378	2	8.451443573	6.80318718	9.125585405	9.11047337	10.64294545	10.9326885
ENSG00000100346	CACNA1I	4.130992283	0.192660633	1.273345313	0.715242599	5.404337596	2	2	2	4.237680128	4.63360314	3.12825509	2.059860178
ENSG00000156103	MMP16	2.669819916	0.004515748	2.73225631	0.003390575	5.402076226	5.866944937	5.72254323	3.369317972	7.883054517	7.92635454	8.43671169	7.263337153
ENSG00000151553	FAM160B1	2.444538204	0.335980269	2.953856332	0.227974555	5.398394536	2	8.101365653	2	8.971529165	8.94703391	9.543411506	9.382476431
ENSG00000116127	ALMS1	2.311372033	0.327520219	3.086440291	0.170499056	5.397812324	2	7.35507212	2	8.045195239	8.1187584	8.728011488	8.959923085
ENSG00000176208	ATAD5	3.452476183	0.016039408	1.941588122	0.198118401	5.394064305	3.327711234	3.076665209	2	5.540148523	5.34860058	4.122514202	4.201948172
ENSG00000162419	GMEB1	2.284703441	0.000196909	3.10778202	1.58E-07	5.392485462	4.467311984	5.131603625	4.523675391	6.825617242	6.94032357	7.656405908	7.714671806
ENSG00000115183	TANC1	2.133923372	0.075173015	3.253501015	0.003975743	5.387424387	4.815609471	5.303523747	7.934808822	8.817637515	8.74357228	9.907203225	9.879774129
ENSG00000166145	SPINT1	1.665308784	0.300950806	3.721006907	0.009972043	5.386315691	4.467311984	2	2	4.329433326	4.58534546	6.535683256	6.029985803
ENSG00000166479	TMX3	3.054108486	0.057349984	2.327156174	0.153080856	5.381264661	2	8.029695761	7.818184403	10.40814626	10.3509915	9.592613586	9.71618004
ENSG00000133059	DSTYK	2.338574328	0.035431212	3.041203607	0.004478253	5.379777935	4.815609471	7.847167909	5.59507254	9.011324139	8.9648262	9.77953276	9.587040651
ENSG00000008118	CAMK1G	4.390214635	0.049461281	0.989073682	0.699544446	5.379288317	2	2	2	4.71689672	4.60967607	2.775316226	2.374681325
ENSG00000116641	DOCK7	2.618764995	0.075503547	2.75983479	0.0559313	5.378599785	6.816637977	8.623598428	3.369317972	9.966365455	10.0880289	9.856639061	10.42566819
ENSG00000175216	CKAP5	2.619152529	0.01784745	2.759438829	0.011575145	5.378591358	5.330594134	8.903033433	7.451345288	10.48248248	10.4350864	10.7065785	10.48248
ENSG00000138071	ACTR2	2.241337435	0.057431985	3.135089759	0.005293456	5.376427194	5.70932728	9.318399851	9.491321578	11.13672482	11.0815752	11.92508678	12.07492997
ENSG00000205903	ZNF316	2.644182449	0.002887069	2.730157897	0.001920201	5.374340346	4.00722531	6.602424674	6.882775709	8.842507967	8.86350401	8.951818769	8.924637944
ENSG00000138669	PRKG2	6.852127766	4.25E-05	-1.481166354	0.487891953	5.370961412	2	2	2	6.979883891	6.8715633	2.183373728	2.059860178
ENSG00000063438	AHRR	2.716034074	0.087305384	2.654462099	0.090359864	5.370496174	6.009025997	7.761270542	2	9.268635235	9.22613117	9.167113444	9.20537272
ENSG00000198948	MFAP3L	1.615623527	0.03679441	3.754290265	9.32E-08	5.369913792	6.89194353	5.303523747	5.39244166	7.728718552	7.46188679	9.98393497	9.389054739
ENSG00000176678	FOXL1	1.301760167	0.644603834	4.064883623	0.103371184	5.36664379	8.418903792	2	2	8.130575473	8.14396641	10.5006588	11.18464037
ENSG00000127920	GNG11	4.412826109	1.23E-11	0.951807122	0.204070923	5.364633231	7.82963488	6.844429755	8.721235202	12.40440824	12.3454117	8.810895984	9.030812517
ENSG00000158113	LRRC43	2.54282498	0.377368801	2.818692227	0.310621084	5.361517207	2	2	2	2.695456494	3.74316441	3.616794016	3.33635788
ENSG00000081014	AP4E1	2.547839818	6.96E-06	2.8113683	5.09E-07	5.359208118	5.330594134	6.535001748	6.322058544	8.710792067	8.57772939	8.581047186	9.172702781
ENSG00000103222	ABCC1	6.139052554	6.06E-09	-0.780909427	0.544666992	5.358143127	2	5.946638857	6.322058544	11.59119151	11.6549123	4.675266036	5.108633494
ENSG00000133858	ZFC3H1	2.933528219	0.053285833	2.420506489	0.113283635	5.354034708	7.435667355	7.638155693	2	9.897454779	9.84004698	9.426088142	9.287866987
ENSG00000105835	NAMPT	5.415931016	5.39E-67	-0.062038928	0.888380576	5.353892087	7.904282243	8.147241016	7.691296561	13.3498354	13.3065908	8.017249226	7.715835172
ENSG00000135709	KIAA0513	1.864996942	0.001066135	3.488271378	8.61E-11	5.35326832	6.366709671	5.131603625	6.203069917	7.723090567	7.7919464	9.537108665	9.165057331
ENSG00000243896	OR2A7	3.859837642	0.089428115	1.491394362	0.544635534	5.351232004	2	2	2	4.299492648	4.16043394	2.775316226	2.782576693
ENSG00000157551	KCNJ15	6.758835774	2.90E-05	-1.408714414	0.496385053	5.35012136	2	2	2	6.941624891	6.72335419	2.094598369	2.172608095
ENSG00000089902	RCOR1	2.126052346	0.192916453	3.222256071	0.036232528	5.348308417	2	7.669933291	5.930865648	8.600009476	8.54340749	9.457577544	9.837645613
ENSG00000149506	ZP1	3.211653393	0.203854133	2.134262012	0.409012973	5.345915405	2	2	2	3.566290607	3.90918929	2.911488689	3.236138336
ENSG00000104228	TRIM35	2.641820563	0.040404199	2.699941311	0.033924497	5.341761874	5.866944937	6.602424674	2	8.304583895	8.29975519	8.643500187	8.005636809
ENSG00000065970	FOXJ2	1.967305803	0.174753955	3.371913488	0.012370521	5.339219291	2	7.051611315	6.534109959	8.14116509	8.20911072	9.698306977	9.423997212
ENSG00000147234	FRMPD3	1.389352484	0.619039988	3.946579654	0.106137285	5.335932138	2	2	2	2.695456494	2.77779907	3.56816602	4.784231301
ENSG00000108679	LGALS3BP	3.204307339	8.47E-05	2.131536817	0.012057282	5.335844156	11.29732276	8.820493869	10.39531398	13.66921984	13.7104399	12.55282788	12.67974691
ENSG00000179295	PTPN11	2.276154318	1.22E-10	3.057731529	1.37E-18	5.333885847	8.16936288	8.607278467	8.865353883	10.83891504	10.8388264	11.46710344	11.75667488
ENSG00000106560	GIMAP2	3.281427504	0.098785844	2.049186219	0.316737831	5.330613723	3.327711234	2	2	4.415698455	4.65713986	3.968654369	3.128433649
ENSG00000147027	TMEM47	1.931983571	0.310383334	3.398565477	0.054125008	5.330549048	2	7.145017454	9.06622548	9.779043349	9.69834686	11.22351878	11.17916827
ENSG00000185989	RASA3	2.88278534	0.024475005	2.44595384	0.058553075	5.32873918	3.327711234	7.701025996	7.734844356	9.989404766	10.0451865	9.363939866	9.772611475
ENSG00000164164	OTUD4	2.694278278	0.10892118	2.633423721	0.111962102	5.327702	7.096806373	8.414402626	2	10.0321341	9.96199598	9.996814524	9.874314395
ENSG00000102921	N4BP1	3.045738375	0.013831602	2.281378797	0.071976668	5.327117172	4.00722531	8.395519306	7.098165388	10.36678618	10.3800133	9.658938866	9.563647359
ENSG00000121068	TBX2	3.139403656	0.036469923	2.186115358	0.155557022	5.325519014	6.138360429	7.467835385	2	9.445659335	9.51245165	8.91583461	8.012280354
ENSG00000164291	ARSK	1.532336117	0.003324438	3.790421694	3.01E-15	5.322757811	5.866944937	5.303523747	5.772716771	7.144720234	7.05550676	9.497620601	9.129269498

ENSG00000106714	CNTNAP3	2.598455667	0.329996554	2.723873406	0.293334688	5.322329073	2	2	2	3.566290607	3.08597292	3.56816602	3.261853426
ENSG00000164142	FAM160A1	2.936153342	0.256740427	2.384628069	0.357240472	5.32078141	2	2	2	3.406930997	3.69849104	3.035909414	3.383972333
ENSG00000198208	RPS6KL1	2.011728946	0.32587825	3.307893372	0.081233304	5.319622318	2	3.076665209	2	3.406930997	3.74316441	4.868377487	4.175132639
ENSG00000167964	RAB26	3.243214407	0.203115525	2.072546261	0.428771225	5.315760668	2	2	2	3.663521627	3.86943969	2.803598391	3.261853426
ENSG00000142327	RNPEPL1	1.822070188	0.397020048	3.492993963	0.07747944	5.31506415	4.00722531	8.433041974	2	8.630393142	8.81462916	10.33327078	10.4387618
ENSG00000117472	TSPAN1	2.442338589	0.362156704	2.871594719	0.264665124	5.313933308	2	2	2	3.023133209	3.45167086	3.336159116	3.67940853
ENSG00000189420	ZFP92	-1.306003541	0.649972951	6.618768172	0.001615626	5.31276463	2	2	2	2.270786764	2	6.934553331	6.423723772
ENSG00000166128	RAB8B	3.70415574	0.000375915	1.608549897	0.155092367	5.312705637	4.815609471	7.431218269	4.874587708	9.917305676	9.92490661	8.084345843	7.557435494
ENSG00000123080	CDKN2C	1.937891283	0.211843193	3.371092447	0.019079173	5.308983729	2	4.442710129	2	4.927308402	4.85329164	5.737015686	6.543880272
ENSG00000168137	SETD5	2.531668593	0.003344383	2.776069862	0.001089398	5.307738455	6.366709671	8.356994601	8.99637432	10.7774535	10.7784015	11.11547411	10.92163967
ENSG00000114439	BBX	2.50017414	0.029907596	2.806998161	0.01288167	5.307172301	5.70932728	8.337339476	9.553445883	11.04750286	11.0320825	11.30568783	11.38542086
ENSG00000013364	MVP	2.392200974	0.225247916	2.91052693	0.12583058	5.302727904	9.98400217	8.607278467	2	11.23144255	11.2789083	11.47598604	12.01912506
ENSG00000124249	KCNK15	-1.305316762	0.681389915	6.605989017	0.005488246	5.300672254	2	2	2	2.270786764	2	7.308441828	5.581648407
ENSG00000154065	ANKRD29	4.546029466	0.118824374	0.753533914	0.823417076	5.29956338	2	2	2	4.574143677	4.98931816	2.858551861	2.059860178
ENSG00000075539	FRYL	2.442801492	0.071895607	2.855436104	0.030430098	5.298237596	3.327711234	8.077869079	6.718950348	9.453327884	9.38785872	9.703944794	9.948684752
ENSG00000107779	BMPR1A	2.73529087	3.83E-11	2.562658539	5.74E-10	5.297949409	6.009025997	6.04685525	5.772716771	8.545247918	8.673169	8.48662708	8.391460941
ENSG00000066248	NGEF	-0.723936751	0.790629813	6.019714113	0.002211198	5.295777362	2	2	2	2.270786764	2.14022021	5.318934974	6.64749689
ENSG00000203896	LIME1	3.586325322	0.012675026	1.709285689	0.267993165	5.295611011	2	2	4.523675391	6.24507773	6.14893615	4.604690117	4.4231929
ENSG00000100242	SUN2	2.467886049	0.165072736	2.827435083	0.100639252	5.295321132	8.609439662	6.602424674	2	9.767470067	9.82128771	10.09733848	10.20607564
ENSG00000265190	ANXA8	1.690418739	0.521219774	3.604181959	0.125703081	5.294600698	2	2	2	2.947924294	2.77779907	3.943456545	4.119958326
ENSG00000110422	HIPK3	2.474830727	0.003242657	2.819178567	0.00062762	5.294009294	7.940205782	7.467835385	5.39244166	9.750280981	9.69550802	10.24675978	9.859390114
ENSG00000128596	CCDC136	2.636359197	0.159971389	2.655400156	0.148260019	5.291759353	2	5.838937808	2	6.956091174	6.83074839	7.078941352	6.725806822
ENSG00000282077		4.26058699	0.052219697	1.031018608	0.681460704	5.291605598	2	2	2	4.739373666	4.3456612	2.492221014	2.70970617
ENSG00000183684	ALYREF	2.94661578	0.004929756	2.344007469	0.028590946	5.290623249	3.327711234	6.311476754	4.523675391	8.051945366	8.11663771	7.198250335	7.737762724
ENSG00000093183	SEC22C	2.313219991	0.000636648	2.974613282	6.20E-06	5.287833273	5.330594134	7.232742069	6.80318718	8.948803189	8.92660044	9.395638968	9.769813713
ENSG00000137656	BUD13	2.482119852	0.195669275	2.805649075	0.131096155	5.287768926	2	5.946638857	2	6.965655398	6.72891807	7.190239142	7.140495192
ENSG00000142949	PTPRF	2.102374739	0.101674945	3.183894193	0.008569098	5.286268932	4.467311984	8.820493869	8.236946975	10.12521908	10.0809763	11.24067069	11.12086139
ENSG00000155561	NUP205	2.801720956	0.094156907	2.482854504	0.135518423	5.28457546	5.532353208	7.874700259	2	9.304878886	9.35675148	9.055415647	8.972144811
ENSG00000015676	NUDCD3	2.367340976	0.088711396	2.911149186	0.030006203	5.278490162	8.228665467	8.101365653	3.369317972	9.906793906	9.99873154	10.49009305	10.50081829
ENSG00000107968	MAP3K8	2.747445154	0.076258148	2.530893966	0.100093385	5.27833912	3.327711234	6.04685525	2	7.29643414	7.39346687	6.579278019	7.535201607
ENSG00000006118	TMEM132A	3.636291041	0.000253038	1.640583741	0.127435542	5.276874782	5.866944937	9.102512015	8.941672936	12.13013662	12.1533131	10.15815498	10.14152759
ENSG00000095587	TLL2	3.147453096	0.244748361	2.128167559	0.440774103	5.275620655	2	2	2	3.515101962	3.86943969	2.656294034	3.407202941
ENSG00000140563	MCTP2	2.625286019	2.31E-05	2.650180951	1.68E-05	5.27546697	4.00722531	5.131603625	5.772716771	7.580980221	7.64371756	7.684242973	7.588252384
ENSG00000060642	PIGV	3.075754089	0.070252457	2.198748185	0.204625268	5.274502274	2	6.728495673	3.369317972	8.260583448	8.34212018	7.304736313	7.563910189
ENSG00000122008	POLK	3.155792929	0.037404281	2.117230849	0.175071314	5.273023779	2	7.274681896	5.39244166	9.190240253	9.12847688	8.423103999	7.766116162
ENSG00000015568	RGPD5	10.46403953	2.82E-13	-5.191151153	0.023574771	5.272888379	2	2	2	10.50109983	10.4877241	2	2
ENSG00000183054	RGPD6	10.46403953	2.82E-13	-5.191151153	0.023574771	5.272888379	2	2	2	10.50109983	10.4877241	2	2
ENSG00000135842	FAM129A	2.699332003	0.026904691	2.573336137	0.034537867	5.27266814	6.257048221	7.467835385	4.874587708	9.198375657	9.23886123	9.879654003	7.234405279
ENSG00000125845	BMP2	4.932519785	0.00562177	0.338655386	0.880889575	5.27117517	7.940205782	5.131603625	2	11.44179676	11.4338527	7.647656456	5.20457242
ENSG00000183578	TNFAIP8L3	3.477391059	0.002290805	1.792799132	0.14042775	5.270190191	2	4.442710129	5.59507254	7.942383523	7.76511961	6.664632795	5.59182244
ENSG00000198087	CD2AP	2.781812188	0.015572998	2.488250465	0.031551126	5.270062654	6.468621203	7.35507212	3.369317972	9.277301907	9.10192275	9.081998027	8.693802688
ENSG00000164916	FOXK1	1.944395126	0.001370344	3.320414389	8.54E-09	5.264809515	6.468621203	7.60566235	8.007629717	9.351221511	9.47486651	11.0025909	10.52905457
ENSG00000187626	ZKSCAN4	2.458914819	0.129733292	2.80556442	0.074759029	5.264479239	2	5.131603625	2	5.978405189	6.08962627	6.533574091	6.168983563
ENSG00000214022	REPIN1	2.584577143	0.014636321	2.679600202	0.010581937	5.264177345	5.70932728	8.522788974	6.203069917	9.960398193	9.90046182	10.00540087	10.04508992
ENSG00000008277	ADAM22	1.599895999	0.567865457	3.664077464	0.143706589	5.263973464	2	2	2	3.09461492	2.49394783	3.930690651	4.215171317
ENSG00000131504	DIAPH1	2.43044703	0.239601493	2.828836859	0.157060567	5.259283889	4.815609471	8.834582582	2	9.693134408	9.81476202	9.995665807	10.2930197
ENSG00000047346	FAM214A	3.162665874	0.035415609	2.09138067	0.177532791	5.254046544	4.467311984	6.899080544	2	8.668958796	8.69465306	7.523500045	7.72163797
ENSG00000120262	CCDC170	2.012515229	0.30391278	3.241113355	0.073543326	5.253628584	2	2	3.369317972	3.515101962	3.65239003	4.461156421	4.553838275
ENSG00000163449	TMEM169	2.773988867	0.199271942	2.476398348	0.247980941	5.250387215	2	2	3.369317972	4.387512679	3.82856379	4.378961816	3.209956562
ENSG00000169908	TM4SF1	3.159118368	0.002904929	2.090928249	0.058149869	5.250046617	8.313285031	5.303523747	8.742726175	11.15146597	11.1825997	9.815783218	10.34017703
ENSG00000161082	CELF5	2.861150031	0.277466813	2.385603381	0.363109023	5.246753412	2	2	2	3.709785322	3.28037825	3.355392153	3.071407821
ENSG00000145555	MYO10	2.941264334	7.04E-08	2.300750089	3.33E-05	5.242014423	8.285626337	9.338312924	8.076950877	11.57488023	11.655095	11.08348224	10.85996842
ENSG00000128285	MCHR1	2.213376551	0.403701418	3.027019801	0.225372001	5.240396353	2	2	2	3.162721474	3.08597292	3.517841605	3.717076715
ENSG00000213889	PPM1N	2.68947476	0.30391278	2.54966853	0.320161353	5.23914329	2	2	2	3.406930997	3.39680241	3.193831101	3.430065404
ENSG00000179542	SLITRK4	7.992334969	7.44E-08	-2.753271311	0.186976308	5.239063658	2	2	2	8.087422161	7.99261031	2.048074405	2.059860178
ENSG00000147010	SH3KBP1	3.740009227	0.021998462	1.497688814	0.39901599	5.237698041	3.327711234	6.535001748	9.439621147	11.77036178	11.7718811	9.249414741	9.773170377
ENSG00000138030	KHK	1.152227925	0.681613489	4.080808531	0.088401773	5.233036457	2	2	2	2.868578001	2.38539787	4.628601098	4.147809227
ENSG00000151503	NCAPD3	2.301224637	0.185410261	2.931138801	0.078715146	5.232363438	3.327711234	6.666836837	2	7.477814553	7.48179565	7.964208996	8.220862755
ENSG00000111647	UHRF1BP1L	3.28660371	2.48E-05	1.945353204	0.017959609	5.231956915	4.00722531	6.389896633	6.534109959	9.231457548	9.20332986	7.951626215	7.827675587
ENSG00000108439	PNPO	2.945934744	0.002537365	2.285465144	0.02246952	5.231399888	4.00722531	4.11352228	6.322058544	8.008651253	8.01099479	7.117472117	7.571641661
ENSG00000179195	ZNF664	1.591870014	0.005233903	3.637735593	5.88E-12	5.229605607	7.82963488	8.004992147	6.629488387	9.263797825	9.10299435	11.07199274	11.36146232
ENSG00000136867	SLC31A2	6.351005583	2.95E-08	-1.122524023	0.42197184	5.22848156	3.327711234	3.686303414	2	8.7747563	8.80016655	2.911488689	2.421067459
ENSG00000158079	PTPDC1	2.575605227	0.064574411	2.65094195	0.053081508	5.226547177	3.327711234	5.72254323	2	6.902323594	6.88657603	7.16729616	6.735016458
ENSG00000133740	E2F5	1.935510386	0.217054056	3.289494536	0.023768024	5.225004922	4.467311984	2	2	4.694064037	4.68029874	5.966975997	5.825320785
ENSG00000270136	MINOS1-NBI	10.41609146	4.03E-13	-5.191151159	0.023706691	5.224940302	2	2	2	10.4133977	10.4790834	2	2
ENSG00000122367	LDB3	-1.183882387	0.410884798	6.406962954	6.13E-09	5.223080566	4.00722531	2	3.369317972	2.868578001	2.49394783	9.092781861	8.689663984
ENSG00000141255	SPATA22	2.819431653	0.386043706	2.400077308	0.457596529	5.219508961	2	2	2	3.566290607	3.39680241	3.796405078	2.277179783
ENSG00000186130	ZBTB6	2.580528467	0.215761172	2.638601874	0.196222912	5.219130341	2	6.464272742	2	7.447418636	7.47187556	7.637872201	7.383872668
ENSG00000158008	EXTL1	1.037626851	0.487332808	4.180908336	0.000909742	5.218535187	2	4.710564154	3.369317972	4.497095146	4.76938658	6.560755617	8.146888246
ENSG00000179041	RRS1	2.469462047	0.098765799	2.748807325	0.058606472	5.218269372	3.327711234	5.946638857	2	6.951285179	7.02417148	6.958334233	7.5085977
ENSG00000148341	SH3GLB2	1.900459201	0.211593503	3.315028329	0.019191632	5.21548753	7.33131396	7.145017454	2	8.52598359	8.55127993	9.885040669	9.999723616
ENSG00000188199	NUTM2B	6.448043195	0.000360365	-1.234657172	0.587973908	5.213386022	2	2	2	6.604700657	6.46787796	2.225793514	2.059860178
ENSG00000163877	SNIP1	2.910585576	0.000969079	2.299880973	0.010756531	5.210466549	4.815609471	6.728495673	4.523675391	8.639384849	8.49198035	7.844256003	8.076179938
ENSG00000100906	NFKBIA	3.321857081	5.16E-07	1.886952162	0.00682286	5.208809243	8.836089686	7.467835385	8.380957451	11.61835275	11.6488716	9.491385735	10.67585959
ENSG00000116604	MEF2D	2.026386615	2.89E-11	3.181373988	1.19E-26	5.207760603	7.975256505	7.701025996	8.175061096	9.938102197	9.98484711	11.04198142	11.18159118
ENSG00000266074	BAHCC1	2.293224566	0.120787522	2.914372325	0.040105266	5.207596892	6.468621203	7.35507212	2	8.579911316	8.74905888	9.194070966	9.367009385
ENSG00000197555	SIPA1L1	3.723017218	1.23E-05	1.480199426	0.112527071	5.203216645	6.468621203	8.777382925	6.958202216	11.4340665	11.5095472	9.097786864	9.364044387
ENSG00000169679	BUB1	2.598957481	0.076258148	2.604175901	0.070716745	5.203133383	2	4.11352228	2	5.194621444	5.07918227	5.026717879	5.250250853
ENSG00000131389	SLC6A6	2.922701605	2.80E-06	2.275708628	0.000345801	5.198410233	7.096806373	7.979858146	8.805342542	11.00726039	11.0432906	10.53427493	10.20711012
ENSG00000075151	EIF4G3	2.664360981	0.002388547	2.53317796	0.00391914	5.197538941	6.73718411	9.225224669	7.691296561	10.90813165	10.8986743	10.68832675	10.85205426
ENSG00000169248	CXCL11	10.38726814	4.94E-13	-5.191151162	0.023768024	5.196116975	2	2	2	10.4577033	10.3769158	2	2
ENSG00000059804	SLC2A3	2.482273802	5.66E-09	2.713235937	1.35E-10	5.195509739	8.716893051	7.979858146	8.236946975	10.80568529	10.8076163	11.29350887	10.725075
ENSG00000187634	SAMD11	2.805951894	0.074779862	2.389417369	0.128777677	5.195369263	2	3.076665209	4.058998049	5.565529336	5.2591458	5.622611168	4.077147165
ENSG00000058804	NDC1	2.683610608	0.191106627	2.511430305	0.215861	5.195040914	2	6.389896633	2	7.507583254	7.46522407	7.358152527	7.277588383
ENSG00000214940	NPIPA8	10.38455242	4.80E-13	-5.191151179	0.023719047	5.193401241	2	2	2	10.39802725	10.432105	2	2
ENSG00000214967	NPIPA7	10.38455242	4.80E-13	-5.191151179	0.023719047	5.193401241	2	2	2	10.39802725	10.432105	2	2
ENSG00000103966	EHD4	2.363495566	0.039654564	2.829257908	0.011254194	5.192753475	4.467311984	7.467835385	7.934808822	9.554240322	9.52849248	9.270464658	10.48946923
ENSG00000261796	ISY1-RAB43	10.3779691	5.71E-13	-5.191151138	0.023890695	5.18681796	2	2	2	10.34404954	10.4703907	2	2
ENSG00000123892	RAB38	4.200838112	0.003614241	0.982683879	0.556098014	5.183521991	3.327711234	3.076665209	2	6.155987605	6.12381559	3.738983888	3.15612297
ENSG00000140743	CDR2	2.431988598	0.341594707	2.750799257	0.267661148	5.182787856	2	8.101365653	2	8.965583267	8.92781046	9.56215648	8.885886446
ENSG00000123685	BATF3	3.194994601	0.062735523	1.985730096	0.264144129	5.180724697	2	3.686303414	2	5.304221918	4.98931816	4.461156421	3.735548302
ENSG00000163602	RYBP	3.039524859	0.009118461	2.140669425	0.075047762	5.180194284	3.327711234	6.535001748	7.451345288	9.56371781	9.46323044	8.766685705	8.461794641
ENSG00000167632	TRAPPC9	1.672146707	0.26684385	3.507689262	0.010549918	5.179835969	3.327711234	6.78762695	3.369317972	6.86703417	7.23436042	8.769376872	8.957466266
ENSG00000164188	RANBP3L	0.495933931	0.85245466	4.681122192	0.02246952	5.177056124	2	2	2	2.3892346	2.49394783	4.881704255	4.943664032
ENSG00000143756	FBXO28	2.760284548	0.059583449	2.414454496	0.099599264	5.174739044	6.653098118	7.431218269	2	9.228481255	9.26398924	9.050261203	8.7391339
ENSG00000100836	PABPN1	3.096396012	2.86E-07	2.076967362	0.000862769	5.173363374	6.366709671	6.311476754	7.691296561	10.02013627	9.93817677	8.83678613	9.084938196
ENSG00000196154	S100A4	2.40691025	0.159728332	2.763788392	0.095263386	5.170698642	2	6.602424674	7.691296561	9.000896538	9.07814384	8.147770839	10.05316631
ENSG00000166454	ATMIN	2.633959272	0.080939257	2.533094622	0.08995084	5.167053893	5.330594134	8.607278467	4.058998049	9.865241782	9.79434273	9.773513173	9.684739879
ENSG00000160007	ARHGAP35	1.760206001	6.38E-08	3.406560063	2.36E-27	5.166766064	8.75772933	8.522788974	8.884815469	10.4577033	10.4926385	11.89156381	12.31521775
ENSG00000144647	POMGNT2	1.49314763	0.036228406	3.670744415	1.13E-08	5.163892045	5.532353208	5.457120953	3.369317972	6.463327516	6.50077304	8.642521927	8.57382182
ENSG00000095787	WAC	2.63030871	0.000207528	2.533290081	0.000351347	5.163598791	7.53297911	9.00621767	7.342949392	10.781859	10.7963987	10.88653786	10.46788136
ENSG00000160216	AGPAT3	2.45353413	2.17E-20	2.70893288	5.68E-25	5.16246701	8.042902253	8.029695761	7.858115942	10.38070504	10.4528466	10.62473233	10.71768999
ENSG00000166169	POLL	1.996774262	0.214567154	3.165341153	0.035964483	5.162115415	4.00722531	6.666836837	2	7.182120794	7.33993465	8.591223132	8.204380326
ENSG00000144560	VGLL4	2.549689169	6.56E-10	2.611286634	2.05E-10	5.160975803	7.096806373	6.844429755	7.285536013	9.619311339	9.56160864	9.899034515	9.353618642
ENSG00000172738	TMEM217	2.975968555	0.004961216	2.184940552	0.044818092	5.160909106	4.00722531	2	4.874587708	6.640968371	6.49425375	5.818966034	5.825320785
ENSG00000171621	SPSB1	2.502438763	0.12806343	2.658293193	0.098271797	5.160731956	2	6.899080544	8.110401118	9.511620236	9.53327015	9.618963711	9.733801916
ENSG00000095139	ARCN1	2.38912447	0.033038824	2.769554243	0.011351964	5.158678714	9.015175615	10.5327403	6.958202216	11.85384977	11.8503907	12.1907878	12.27242173

ENSG00000028137	TNFRSF1B	2.974356081	2.34E-05	2.183437642	0.002486145	5.157793722	3.327711234	4.93638933	5.39244166	7.565338955	7.56813436	6.937746858	6.6426114
ENSG00000196155	PLEKHG4	1.484333622	0.390209318	3.666821895	0.017730665	5.151155517	6.468621203	3.686303414	2	6.449761039	6.54560135	7.843404862	9.136672183
ENSG00000198435	NRARP	1.939424255	0.479723716	3.209680182	0.204683486	5.149104437	2	2	2	3.162721474	2.77779907	3.878466556	3.600986722
ENSG00000121454	LHX4	0.42763088	0.852752459	4.721431192	0.007480866	5.149062072	2	3.076665209	2	2.695456494	2.77779907	5.141958644	6.27380897
ENSG00000172071	EIF2AK3	2.833493078	0.099708601	2.315329593	0.179625742	5.148822671	3.327711234	6.728495673	2	8.100504225	8.01782901	7.66606569	7.425709383
ENSG00000197063	MAFG	2.916041429	6.36E-17	2.229374755	2.31E-10	5.145416184	7.867441304	7.467835385	8.206335842	10.75591108	10.7635692	10.06648818	10.08367608
ENSG00000102878	HSF4	3.562951209	0.010828316	1.581271309	0.294849354	5.144222518	2	4.710564154	2	6.676346669	6.66069199	5.079718116	4.56420962
ENSG00000213967	ZNF726	4.029219648	0.076034873	1.114809732	0.663448601	5.144029379	2	2	2	4.358765249	4.37434574	2.746468522	2.509591799
ENSG00000148358	GPR107	2.289527739	0.015836831	2.853767986	0.001919305	5.143295725	6.257048221	9.159587375	8.65477198	10.71325662	10.7523441	11.35735632	11.23206261
ENSG00000166289	PLEKHF1	2.665886509	0.12734523	2.475398677	0.152273554	5.141285187	2	5.457120953	2	6.535745081	6.55815769	6.579278019	6.113568999
ENSG00000139572	GPR84	6.2663893	0.000320814	-1.125903753	0.610519493	5.140485548	2	2	2	6.283823264	6.44101097	2.094598369	2.225841198
ENSG00000248905	FMN1	4.001876443	0.024397609	1.138216699	0.568885801	5.140093142	2	2	5.59507254	7.789219291	7.93600799	5.494014735	4.692661645
ENSG00000133878	DUSP26	1.014354529	0.736178	4.120167769	0.107186013	5.134522297	2	2	2	2.784612485	2.38539787	4.990270811	3.539239711
ENSG00000143878	RHOB	1.7918509	0.014995326	3.339404933	1.17E-06	5.131255833	9.128497779	8.234834549	7.163363831	10.1568736	10.1761984	11.8995701	11.49357837
ENSG00000101311	FERMT1	5.01199917	0.010010073	0.11803254	0.963624158	5.130031709	2	2	2	5.304221918	5.09650239	2.42098416	2.277179783
ENSG00000171488	LRRC8C	3.660041145	0.008716756	1.468770963	0.33487913	5.128812108	2	4.442710129	6.322058544	8.657201865	8.58694992	7.013858277	5.631815919
ENSG00000163029	SMC6	2.412115533	0.086021641	2.714400283	0.046914405	5.126515816	4.467311984	7.189546432	3.369317972	8.29513208	8.20911072	8.953397421	7.992257228
ENSG00000186026	ZNF284	3.144609612	0.030874483	1.980661426	0.189987418	5.125271038	2	4.710564154	2	6.34372989	6.18176397	5.413723492	4.927687581
ENSG00000054793	ATP9A	1.68137001	0.325849593	3.443154401	0.02799101	5.124524411	7.975256505	8.029695761	2	9.111653326	9.06830201	10.77323249	10.91228265
ENSG00000179598	PLD6	3.400011944	0.006529818	1.723276994	0.19604595	5.123288939	4.00722531	2	3.369317972	5.860316854	6.1068222	4.301748724	4.673629022
ENSG00000187079	TEAD1	1.928728878	0.004384186	3.19350787	6.53E-07	5.122236747	8.138771685	9.523645035	9.945092785	11.35143572	11.2588566	12.48349989	12.65034854
ENSG00000187474	FPR3	-0.30732972	0.924945325	5.429398606	0.026356479	5.122068886	2	2	2	2.3892346	2.14022021	6.332313483	3.924507493
ENSG00000146433	TMEM181	2.419535852	0.01404794	2.70072789	0.005236775	5.120263742	5.330594134	8.356994601	7.029880454	9.795895983	9.76282251	10.04832124	10.07054948
ENSG00000146426	TIAM2	2.741151165	0.113850795	2.377328202	0.169003976	5.118479367	2	2	5.156626401	6.164317909	6.14061154	5.916072173	5.699242824
ENSG00000281670		2.258728545	0.082560548	2.857655601	0.022614967	5.116384146	7.904282243	7.274681896	3.369317972	9.262828394	9.34230452	9.778642552	10.00995536
ENSG00000171385	KCND3	1.69329442	0.555426444	3.416951758	0.190459822	5.110246178	2	2	2	3.162721474	2.49394783	3.865109708	3.924507493
ENSG00000137869	CYP19A1	3.666870847	0.164311333	1.441851432	0.61179806	5.108722279	2	2	2	4.105493497	4.05806467	3.059555873	2.374681325
ENSG00000123104	ITPR2	2.700600503	0.085360963	2.408019194	0.123018585	5.108619697	2	7.274681896	5.156626401	8.654247601	8.66738547	8.220374168	8.508191835
ENSG00000155254	MARVELD1	2.023135504	0.035422866	3.084017395	0.000695136	5.107152899	8.444143041	9.246456519	6.322058544	10.45176049	10.4423014	11.44763312	11.56164184
ENSG00000130723	PRRC2B	2.121520886	0.010117882	2.984515568	0.000159121	5.106036454	7.27616306	9.461450882	9.516493218	11.13831145	11.193959	12.10838349	11.94342963
ENSG00000114450	GNB4	2.626108812	1.29E-08	2.479767928	7.86E-08	5.10587674	7.096806373	7.979858146	7.098165388	10.10480594	10.0356777	10.06301929	9.772052356
ENSG00000250151	ARPC4-TTLL3	8.86548825	1.42E-09	-3.761123294	0.108737429	5.104364956	2	2	2	8.88471049	8.9229643	2.048074405	2
ENSG00000170848	PSG6	2.27703585	0.415158868	2.824975009	0.287937041	5.102010859	2	2	2	3.406930997	2.86123014	3.316666208	3.640730416
ENSG00000166250	CLMP	4.967995761	0.003618428	0.132796533	0.952165908	5.100792294	2	6.228547981	8.721235202	12.29568877	12.333934	7.834009015	7.09293558
ENSG00000170903	MSANTD4	2.45092095	0.301707894	2.648979628	0.251602132	5.099900578	2	7.393647515	2	8.274117511	8.24255817	8.580025633	8.315988368
ENSG00000170667	RASA4B	7.88103133	5.74E-07	-2.785644032	0.158705253	5.095387298	2	3.076665209	2	8.737556707	9.01236778	2.183373728	2
ENSG00000171943	SRGAP2C	3.966180648	4.81E-17	1.128818009	0.031069705	5.094998657	6.138360429	5.595927442	6.718950348	10.11612257	10.0501952	7.379447089	7.169655242
ENSG00000147124	ZNF41	3.114832526	0.147934794	1.979899455	0.370178383	5.094731981	2	6.844429755	2	8.386974157	8.34212018	7.191577434	7.307219812
ENSG00000167081	PBX3	2.960817349	0.000972978	2.133701338	0.021537163	5.094518687	3.327711234	5.72254323	6.073378139	8.343618883	8.22494733	7.821097076	7.011905845
ENSG00000143178	TBX19	2.365876789	0.189011771	2.72439038	0.117356112	5.090267168	2	4.11352228	2	4.804776789	4.98931816	5.755231204	4.341135913
ENSG00000270629	NBPF14	6.304119002	1.23E-23	-1.215780007	0.089915373	5.088338995	5.866944937	7.790474954	7.451345288	13.49276167	13.5472161	6.016144783	6.155328188
ENSG00000004700	RECQL	3.188873668	0.008031314	1.899136651	0.131991984	5.088010319	3.327711234	7.638155693	6.958202216	9.950196857	9.94476635	8.785419299	8.536120927
ENSG00000068971	PPP2R5B	2.06912673	0.046720281	3.018250869	0.002233581	5.087377599	4.467311984	7.761270542	7.163363831	9.05005082	9.02486072	9.88958296	10.06759085
ENSG00000179119	SPTY2D1	2.58159614	0.014627781	2.500983888	0.017860434	5.082580028	7.624139768	6.728495673	4.058998049	9.276341511	9.27353851	9.239097094	9.149213283
ENSG00000109674	NEIL3	3.215455832	0.259817515	1.863654172	0.528078406	5.079110004	2	2	2	4.034562184	3.39680241	2.457042231	3.311948089
ENSG00000101849	TBL1X	2.059447669	0.431634218	3.018933693	0.221243146	5.078381363	2	8.191702463	2	8.619831492	8.71442024	9.603714182	9.63702421
ENSG00000127585	FBXL16	0.69117069	0.799285627	4.386376926	0.043660941	5.077547615	2	2	2	2.600425781	2.38539787	4.742549839	4.574506939
ENSG00000109667	SLC2A9	1.152241343	0.685765481	3.921752218	0.107708201	5.073993561	2	2	2	2.868578001	2.38539787	4.122514202	4.4231929
ENSG00000123240	OPTN	2.497219703	1.54E-05	2.575958263	7.20E-06	5.073177966	9.579430841	8.124485672	9.344432377	11.61189576	11.6394858	11.56670583	11.83009033
ENSG00000066336	SPI1	-0.727421237	0.812768397	5.797567426	0.011254194	5.07014619	2	2	2	2.141737246	2.2680111	6.670403488	4.279527956
ENSG00000087253	LPCAT2	3.102998467	6.97E-06	1.964246078	0.006425146	5.067244545	5.532353208	6.228547981	4.523675391	8.702235867	8.5339035	7.803699407	7.112501355
ENSG00000144791	LIMD1	2.428541327	0.022341921	2.638383722	0.011703917	5.06692505	7.709880939	5.457120953	4.874587708	8.894758143	8.96128525	9.150005562	9.123144518
ENSG00000105137	SYDE1	1.861628336	0.000309993	3.204886362	7.87E-11	5.066514699	7.624139768	7.790474954	8.677268317	9.896204982	9.99988257	11.04845858	11.49459545
ENSG00000144837	PLA1A	4.289629241	0.044487261	0.776243094	0.756508981	5.065872335	2	2	2	4.623314311	4.53541753	2.492221014	2.551896972
ENSG00000163686	ABHD6	2.463072939	0.101317769	2.595985253	0.077558619	5.059058193	3.327711234	5.946638857	2	7.007926688	6.95464102	7.364099281	6.802276223
ENSG00000237765	FAM200B	2.274589408	0.003554638	2.783764315	0.00024308	5.058353723	6.009025997	5.457120953	3.369317972	7.494428494	7.45518898	8.058907395	7.884647945
ENSG00000110203	FOLR3	4.534593248	0.080939257	0.523103403	0.866209511	5.057696651	2	2	2	4.739373666	4.83276841	2.717032197	2.117335275
ENSG00000122035	RASL11A	0.992303719	0.437031139	4.063517604	0.000132575	5.055821322	4.00722531	3.686303414	2	3.95996233	4.40247105	7.164572841	6.725806822
ENSG00000197461	PDGFA	1.083689289	0.05874363	3.970908093	1.88E-15	5.054597382	5.532353208	4.93638933	5.59507254	6.229283386	6.48112613	9.220861732	9.150074181
ENSG00000130943	PKDREJ	0.272349571	0.922914669	4.771027746	0.024667973	5.043377317	2	2	2	2.270786764	2.49394783	5.212212839	4.729989799
ENSG00000102879	CORO1A	1.497904385	0.584591586	3.543578477	0.145407903	5.041482862	2	2	2	2.868578001	2.68924543	3.738983888	4.201948172
ENSG00000145391	SETD7	1.559182741	0.38549543	3.479496095	0.032370656	5.038678835	3.327711234	9.532315021	6.80318718	9.682298904	9.75056618	11.53694171	11.72134345
ENSG00000180353	HCLS1	0.694990781	0.837724165	4.343404927	0.116087069	5.038395708	2	2	2	2.784612485	2.14022021	5.163941955	3.753786374
ENSG00000117751	PPP1R8	2.538368044	0.011477015	2.49891976	0.012503529	5.037287804	4.00722531	7.274681896	6.322058544	8.905978734	8.86223882	8.784532718	8.903351429
ENSG00000204176	SYT15	1.716690029	0.182549331	3.31875885	0.004768505	5.035448879	5.095959458	3.076665209	2	5.501219927	5.19627118	6.977080688	6.735016458
ENSG00000118680	MYL12B	1.743302761	0.011618622	3.287800669	3.47E-07	5.03110343	11.08909278	9.549500232	9.945092785	12.10059882	12.0761565	13.25401198	13.93132404
ENSG00000142330	CAPN10	2.181425281	0.151541791	2.845802743	0.050274744	5.027228024	4.00722531	6.464272742	2	7.29643414	7.23826702	7.873733947	7.961201206
ENSG00000157224	CLDN12	3.083407972	0.000882392	1.942789982	0.04529991	5.026197954	4.467311984	6.951736442	7.342949392	9.703174645	9.73267673	8.909342392	8.16744219
ENSG00000172350	ABCG4	0.012663238	0.997041173	5.005894059	0.023327067	5.018557296	2	2	2	2.3892346	2.2680111	5.737015686	4.329022995
ENSG00000091986	CCDC80	0.934463112	0.060422825	4.083154975	7.50E-21	5.017618087	10.84091191	10.42046463	10.74328839	11.59119151	11.6307818	14.20024542	15.15950973
ENSG00000104177	MYEF2	1.532554256	0.013676247	3.483149161	9.42E-10	5.015703417	5.866944937	5.303523747	4.523675391	6.699459234	6.84106088	8.490981547	8.852426354
ENSG00000109861	CTSC	3.278431218	0.000217049	1.737230637	0.066158892	5.015661855	5.866944937	7.189546432	8.536706852	10.81431291	10.8474839	9.003404318	9.538828836
ENSG00000067900	ROCK1	2.651962488	2.38E-05	2.363623081	0.000181868	5.015585569	7.624139768	8.414402626	6.718950348	10.4290383	10.3437572	10.24595488	9.936760772
ENSG00000039600	SOX30	2.549348297	0.349396138	2.465404749	0.355912095	5.014753046	2	2	2	3.515101962	3.08597292	3.193831101	3.33635788
ENSG00000178078	STAP2	2.180179278	0.024886643	2.832817996	0.002385567	5.012997274	2	4.11352228	4.523675391	5.808110275	5.65950892	6.275391383	6.418016952
ENSG00000183426	NPIPA1	10.20385195	1.59E-12	-5.191151239	0.024005483	5.012700712	2	2	2	10.22863675	10.2413953	2	2
ENSG00000165434	PGM2L1	2.358822732	7.03E-05	2.653295876	5.60E-06	5.012118609	6.009025997	6.311476754	6.718950348	8.690748087	8.65574839	9.381049994	8.37829627
ENSG00000121680	PEX16	2.64662678	0.000553874	2.362964357	0.002167809	5.009591137	3.327711234	4.710564154	4.874587708	6.634986713	7.0774796	6.514451546	6.681239787
ENSG00000118507	AKAP7	2.94524553	0.010416238	2.06126126	0.082028804	5.00650679	2	3.686303414	4.058998049	5.959382894	5.84514933	4.778616857	5.361470313
ENSG00000132825	PPP1R3D	2.23213361	0.282224272	2.77233745	0.163643498	5.004471061	2	6.311476754	2	6.922108067	7.0150921	7.45939446	7.529919928
ENSG00000125812	GZF1	2.224587234	0.346349702	2.775187292	0.221243146	4.999774526	2	7.232742069	2	7.855024645	7.89452713	8.714110407	8.044120856
ENSG00000177034	MTX3	2.626699417	0.000917782	2.372752351	0.002916752	4.999451768	6.96351253	6.04685525	4.523675391	8.70652031	8.72280968	8.647894167	8.251669878
ENSG00000127124	HIVEP3	3.070347033	0.000111745	1.921371333	0.021235761	4.991718366	4.00722531	4.93638933	4.058998049	7.363085426	7.15392111	6.464399218	5.768151538
ENSG00000189134	NKAPL	3.638215144	0.021706093	1.352128413	0.43754457	4.990343556	2	2	4.058998049	5.786686927	5.56313034	3.56816602	3.956354764
ENSG00000134508	CABLES1	2.813326782	0.067002675	2.176481799	0.161393405	4.989808581	2	7.002537973	5.39244166	8.570539957	8.63663561	7.137053113	8.502136032
ENSG00000179097	HTR1F	3.116992736	0.540598702	1.872578133	0.721716302	4.989570869	2	2	2	3.840289928	3.50452879	3.517841605	2
ENSG00000103121	CMC2	2.17404781	0.075361599	2.811823887	0.016667916	4.985871697	6.138360429	4.710564154	2	7.093295078	7.10341274	7.617106703	7.817957556
ENSG00000151718	WWC2	2.066066269	0.026395472	2.915073008	0.001037164	4.981139277	5.532353208	6.728495673	8.206335842	9.222510184	9.27923788	10.12701838	10.06416949
ENSG00000154305	MIA3	2.84822169	0.004559857	2.132911125	0.038609931	4.981132815	5.532353208	8.777382925	7.691296561	10.66930252	10.6955802	10.10090399	9.824763374
ENSG00000005471	ABCB4	1.428686499	0.497158772	3.552261433	0.055590322	4.980947932	2	3.076665209	2	3.289987342	3.15372745	5.06226682	4.400220394
ENSG00000198363	ASPH	3.082061351	8.87E-17	1.891977357	5.99E-07	4.974038707	9.460782017	8.955547846	9.372276026	12.38482562	12.3131338	10.84695645	11.41839745
ENSG00000152229	PSTPIP2	1.938236235	0.024764604	3.035186799	0.000189522	4.973423033	3.327711234	5.457120953	4.874587708	6.592405944	6.64311914	8.001311979	7.274433587
ENSG00000129028	THAP10	2.630501498	0.098666313	2.342422965	0.13767291	4.972924463	3.327711234	3.076665209	2	4.71689672	4.72553021	4.749835737	4.147809227
ENSG00000197093	GAL3ST4	1.826781321	0.132570607	3.143202082	0.005270568	4.969983403	2	5.595927442	4.058998049	6.087554739	6.35727082	7.487996019	7.5085977
ENSG00000080819	CPOX	2.580309381	0.236852832	2.389553393	0.268850661	4.969862774	2	6.666836837	2	7.614805492	7.70428369	7.832294085	6.994755759
ENSG00000165806	CASP7	3.203971871	0.065184591	1.764200198	0.332600633	4.96817207	5.095959458	7.979858146	2	9.781753035	9.73820484	8.171023934	8.476283084
ENSG00000107404	DVL1	2.105057516	0.217270166	2.85996514	0.078740418	4.965022656	8.16936288	8.029695761	2	9.585592789	9.63382319	10.58899854	10.09198797
		10.15614525	2.22E-12	-5.191151248	0.024112824	4.964993998	2	2	2	10.16675444	10.2079206	2	2
ENSG00000125347	IRF1	3.227885286	4.13E-05	1.73652762	0.038112383	4.964412906	5.70932728	7.051611315	7.552162999	10.08300173	10.1989281	7.867049036	9.16888512
ENSG00000114315	HES1	1.659454898	0.250900803	3.304087056	0.012430294	4.963541954	4.00722531	5.838937808	2	6.503277904	5.92487824	8.269993575	7.208191747
ENSG00000186352	ANKRD37	2.621065768	0.13602062	2.342208114	0.179503712	4.963273882	2	2	5.156626401	6.113598934	5.96314905	5.696113106	5.859408034
ENSG00000151461	UPF2	2.741992269	0.00086982	2.221012126	0.008154016	4.963004394	5.330594134	7.669933291	6.43197674	9.460955886	9.5348592	9.182338982	8.746552532
ENSG00000102034	ELF4	2.39061265	0.136308666	2.571675612	0.100639252	4.962288262	7.867441304	6.464272742	2	8.984524517	9.22514727	9.185029001	9.387230438
ENSG00000172380	GNG12	1.798193227	0.096174103	3.15917945	0.001597541	4.957372677	7.579279349	10.68823825	9.083172337	11.45182709	11.4065554	12.54306469	12.99917931
ENSG00000143669	LYST	3.102674493	0.002868772	1.854345534	0.090367254	4.957020028	4.00722531	7.35507212	6.43197674	9.55977642	9.50030288	8.642521927	7.829826288
ENSG00000108352	RAPGEFL1	1.855113589	0.110962	3.100403753	0.004067454	4.955517342	3.327711234	4.93638933	2	5.674521485	5.43283104	6.750676314	6.711881188
ENSG00000180370	PAK2	2.114853833	0.005935174	2.836011969	0.000128792	4.950865802	6.138360429	8.337339476	7.896971941	9.889311677	9.74714308	10.54713921	10.53004695

ENSG00000165240	ATP7A	2.791309886	0.001200331	2.159128842	0.014597277	4.950438728	4.00722531	6.78762695	6.073378139	8.756965077	8.74494589	8.035995406	8.210995912
ENSG00000186496	ZNF396	0.276561518	0.922182516	4.673717032	0.029347467	4.95027855	2	2	2	2.498691189	2.2680111	5.056402493	4.739172856
ENSG00000178074	C2orf69	2.295935229	0.153238484	2.65254086	0.088390629	4.94847609	3.327711234	6.228547981	2	7.101994243	7.04216061	7.605105115	7.206537478
ENSG00000204186	ZDBF2	2.505529969	4.45E-05	2.442769274	6.58E-05	4.948299243	5.532353208	6.728495673	5.59507254	8.543652346	8.51952909	8.708044758	8.183508901
ENSG00000146587	RBAK	2.306407115	0.342054331	2.641250071	0.261724935	4.947657185	2	7.538394143	2	8.246921218	8.27334517	8.798653052	8.348604099
ENSG00000124613	ZNF391	1.887051431	0.131912603	3.05828406	0.008662669	4.945335491	3.327711234	4.11352228	2	4.927308402	4.89348248	6.231826305	5.651404445
ENSG00000148634	HERC4	3.664594483	6.89E-19	1.277623226	0.004009929	4.94221771	7.384433967	8.169643011	8.296287027	11.65834356	11.6394858	9.416391256	9.10594367
ENSG00000004897	CDC27	3.01657372	0.013491212	1.920333316	0.130402547	4.936907036	6.653098118	8.806266215	4.874587708	10.61867235	10.5573944	9.45116681	9.540196884
ENSG00000152104	PTPN14	2.12386618	0.017335408	2.812779827	0.001057274	4.936646007	9.219014269	6.602424674	8.699419242	10.60343658	10.6697774	11.31960213	11.3294243
ENSG00000116701	NCF2	4.667650948	0.006771419	0.26591026	0.90314443	4.933561209	2	3.076665209	6.322058544	9.383689875	9.41054037	5.843010798	3.807156736
ENSG00000137960	GIPC2	1.497911908	0.596370107	3.434482572	0.173666446	4.93239448	2	2	2	2.868578001	2.68924543	3.517841605	4.215171317
ENSG00000134343	ANO3	0.495892678	0.862168375	4.436180789	0.045408424	4.932073467	2	2	2	2.3892346	2.49394783	5.04460184	4.254128754
ENSG00000178852	EFCAB13	2.659319641	0.203115525	2.272059925	0.274284885	4.931379565	2	2	3.369317972	4.17310023	3.90918929	4.133696865	3.261853426
ENSG00000114120	SLC25A36	2.588699801	0.11131837	2.338675263	0.147069388	4.927375063	2	7.928237109	7.451345288	9.682298904	9.70964668	9.73525344	9.087187414
ENSG00000138180	CEP55	2.143912168	0.300919213	2.783078113	0.157231541	4.926990281	2	3.686303414	2	4.070463721	4.40247105	3.796405078	5.349527629
ENSG00000169926	KLF13	5.18169143	3.30E-10	-0.256392758	0.813974819	4.925298672	4.467311984	5.457120953	2	9.55977642	9.55222419	4.487545924	4.329022995
ENSG00000167548	KMT2D	2.544942105	4.73E-07	2.378645864	2.65E-06	4.923587968	8.564112515	7.73146271	8.978369997	10.97536299	11.0945633	10.99264488	10.73644041
ENSG00000026950	BTN3A1	3.761824852	7.34E-17	1.159497799	0.019298939	4.921322651	7.159102577	6.844429755	7.858115942	11.05983441	11.0701139	8.627767864	8.30675314
ENSG00000103657	HERC1	2.216971659	0.152654576	2.700848001	0.07056968	4.917819661	3.327711234	8.607278467	6.322058544	9.524619869	9.51245165	10.09483737	9.898850157
ENSG00000101191	DIDO1	2.253054856	0.183753738	2.664038309	0.103880499	4.917093166	7.384433967	8.356994601	2	9.548682898	9.66535801	10.00197246	10.03253982
ENSG00000173011	TADA2B	2.064678435	0.007676211	2.851494784	0.000123257	4.916173219	5.095959458	6.951736442	5.39244166	8.074220386	8.11238697	8.848711014	8.89361766
ENSG00000151364	KCTD14	3.442289685	0.17253254	1.47356142	0.584265208	4.915851105	2	2	2	3.615725078	4.16043394	2.68698273	2.851942824
ENSG00000101773	RBBP8	2.921787413	0.176804511	1.991011699	0.366230344	4.912799113	2	6.78762695	2	8.104838684	8.12931531	7.34858644	7.045606259
ENSG00000198771	RCSD1	2.447761313	0.008715311	2.464839645	0.007665374	4.912600958	4.467311984	4.442710129	2	6.229283386	6.11534392	6.016144783	6.344709081
ENSG00000163755	HPS3	3.419150131	0.00186218	1.490910645	0.211435731	4.910060776	3.327711234	6.951736442	7.163363831	9.883647099	9.90599812	8.055971376	7.907228933
ENSG00000184083	FAM120C	2.554921135	0.005891845	2.352944224	0.011512049	4.907865359	5.330594134	5.303523747	2	7.186217188	7.24216308	6.864125031	7.157719456
ENSG00000144749	LRIG1	1.95490387	0.15865445	2.95240892	0.023955844	4.907312789	3.327711234	6.602424674	8.042706615	8.913410938	8.81724322	9.801172998	9.911598039
ENSG00000160710	ADAR	3.039091046	0.000242028	1.867362577	0.03200445	4.906453623	8.816894989	10.94030351	9.149036566	12.9569702	13.0309294	11.66451928	11.96682692
ENSG00000161813	LARP4	2.678620081	0.004298315	2.22729533	0.019679078	4.905915411	4.815609471	7.790474954	7.285536013	9.755803193	9.70330142	9.40489908	9.145332871
ENSG00000163545	NUAK2	1.206523553	0.065393662	3.698516579	1.49E-10	4.905040132	4.00722531	5.457120953	5.772716771	6.408278948	6.35727082	8.828207602	8.80566012
ENSG00000111615	KRR1	3.577367754	1.31E-08	1.325821959	0.054930338	4.903189712	6.816637977	6.602424674	4.874587708	9.822457304	9.85983381	7.705944963	7.513957873
ENSG00000167968	DNASE1L2	2.691939554	0.343022212	2.210320044	0.435298592	4.902259599	2	2	2	3.663521627	3.08597292	3.374372158	2.817676617
ENSG00000154642	C21orf91	3.344810831	0.095959724	1.556806012	0.465241848	4.901616843	2	6.311476754	2	8.093978021	8.01782901	6.693258166	5.816671626
ENSG00000163623	NKX6-1	-3.75316742	0.137326507	8.65435048	5.09E-08	4.90118306	2	2	2	2	2	8.743149543	8.644559262
ENSG00000175155	YPEL2	2.640044764	0.045589294	2.261052308	0.088390629	4.901097072	6.653098118	5.946638857	2	8.360030171	8.33666436	8.324077374	7.512619696
ENSG00000088808	PPP1R13B	1.992910475	0.074800869	2.907190259	0.005874607	4.900100733	5.095959458	3.686303414	2	5.818703821	5.76075522	6.865802559	6.406535141
ENSG00000155760	FZD7	1.415148616	0.373567499	3.482781308	0.014716406	4.897929923	3.327711234	7.145017454	8.784770914	9.034230235	9.00665321	10.90450119	11.23581828
ENSG00000162813	BPNT1	2.322878729	0.00381227	2.570687511	0.001121109	4.89356624	3.327711234	5.72254323	5.930865648	7.491120977	7.67575955	7.862014947	7.796124663
ENSG00000184677	ZBTB40	2.455910803	0.131645633	2.436085252	0.128442152	4.891996055	6.138360429	7.819099893	2	9.060130342	9.06830201	9.309201142	8.720128855
ENSG00000131115	ZNF227	2.768843127	0.05130801	2.1210329	0.140636706	4.889876027	3.327711234	5.72254323	2	7.062428156	7.09482005	6.757921389	6.070564958
ENSG00000102981	PARD6A	0.728535674	0.742254487	4.158365625	0.022192774	4.886901299	3.327711234	2	2	2.947924294	2.77779907	5.684753614	4.77533149
ENSG00000165457	FOLR2	0.684028022	0.834689821	4.201771236	0.115977634	4.885799258	2	2	2	2.141737246	2.77779907	4.713032626	4.26688425
ENSG00000155966	AFF2	2.393556069	0.446900339	2.49052849	0.415168199	4.88408456	2	2	2	3.566290607	2.77779907	2.656294034	3.717076715
ENSG00000091136	LAMB1	2.388907341	0.002939392	2.495174123	0.001719657	4.884081463	8.517314938	10.74528399	9.670202709	12.28510445	12.3186792	12.12734459	12.64314327
ENSG00000226650	KIF4B	1.59892001	0.594260626	3.28482377	0.227519745	4.88374378	2	2	2	3.023133209	2.59489879	3.215043717	4.215171317
ENSG00000138386	NAB1	2.805281096	0.192332182	2.076838505	0.340665827	4.882119601	3.327711234	8.276714465	2	9.561354269	9.48394374	8.957336509	8.623017875
ENSG00000172020	GAP43	2.504840654	0.631147757	2.375988829	0.64532651	4.880829483	2	2	2	3.95996233	2	3.296906311	3.128433649
ENSG00000119899	SLC17A5	2.531419026	6.33E-07	2.349272143	4.03E-06	4.880691169	7.750913381	7.928237109	6.629488387	10.05359404	10.059057	9.947565107	9.798374054
ENSG00000157240	FZD1	2.969110281	0.053978339	1.910946716	0.229961504	4.880056996	4.467311984	6.951736442	2	8.537252353	8.5339035	7.41654498	7.567781104
ENSG00000157450	RNF111	2.499654145	0.070386148	2.379031688	0.082535125	4.878685833	5.70932728	7.874700259	3.369317972	9.133031936	9.06170328	9.08380094	8.864016001
ENSG00000112531	QKI	2.002418905	0.001234563	2.875565897	1.42E-06	4.877984802	7.82963488	9.055168103	7.646393253	10.37174052	10.2664796	11.16842771	11.21354805
ENSG00000130881	LRP3	2.659138349	0.280324246	2.218695452	0.366255977	4.877833801	2	7.819099893	2	8.888486565	8.89106117	8.398397867	8.506848296
ENSG00000042445	RETSAT	2.596174964	0.027389043	2.280500972	0.053903774	4.876675935	4.467311984	6.78762695	8.35328246	9.751662842	9.88434767	9.444727462	9.563647359
ENSG00000183496	MEX3B	1.228887207	0.200963739	3.647441595	1.41E-05	4.876328802	5.095959458	5.595927442	3.369317972	6.229283386	5.99120031	8.871434321	7.894955865
ENSG00000154122	ANKH	1.050445753	0.187221604	3.825473836	2.80E-08	4.875919588	7.33131396	7.274681896	8.922968322	9.069031178	9.11791381	11.70970445	11.99548996
ENSG00000169174	PCSK9	1.026543418	0.649972951	3.846154296	0.049924592	4.872697714	2	5.595927442	2	5.022023783	5.2591458	6.05195199	8.588503563
ENSG00000175595	ERCC4	2.695805489	0.000213156	2.176098037	0.003334494	4.871903526	4.00722531	5.457120953	6.073378139	7.997038782	7.96222667	7.727325329	7.156006254
ENSG00000058668	ATP2B4	1.474238251	0.000674855	3.395336229	6.92E-17	4.86957448	9.081016759	9.726146444	9.849281147	11.05760014	11.06132	12.6962582	13.21458197
ENSG00000115457	IGFBP2	-2.460715785	0.009775319	7.328206961	1.51E-17	4.867491176	7.579279349	7.232742069	7.777116083	5.949776857	3.65239003	14.54529467	15.08748176
ENSG00000182585	EPGN	7.86979128	5.02E-11	-3.004225267	0.043497213	4.865566013	2	3.076665209	3.369317972	9.750280081	9.72921087	2.139668783	2.172608095
ENSG00000118922	KLF12	2.392858002	0.018020475	2.472007239	0.013658003	4.864865242	4.467311984	6.228547981	7.600047325	9.027396512	8.84055903	9.148283563	8.869253401
ENSG00000177485	ZBTB33	2.718798168	0.060978041	2.145940165	0.143233501	4.864738333	2	7.099070191	5.930865648	8.747293529	8.71862106	8.466867678	7.787298144
ENSG00000144445	KANSL1L	2.818425244	0.000151121	2.045929135	0.00760211	4.86435438	5.095959458	5.131603625	3.369317972	7.43025113	7.34718809	6.884127769	6.347714043
ENSG00000180535	BHLHA15	1.93618411	0.508390119	2.926007849	0.28326297	4.862191959	2	2	2	2.868578001	3.08597292	3.082820997	3.908316285
ENSG00000143179	UCK2	2.086702697	0.009976623	2.771360206	0.000378704	4.858062903	6.563806279	7.790474954	5.59507254	8.978632046	9.00894176	9.462851966	9.858600332
ENSG00000154493	C10orf90	2.148907564	0.474821199	2.708112679	0.343881844	4.857020243	2	2	2	3.09461492	3.08597292	2.803598391	3.841670744
ENSG00000184845	DRD1	-0.719010549	0.846636453	5.573362126	0.052942339	4.854351577	2	2	2	2.3892346	2	6.18690462	5.005857272
ENSG00000131323	TRAF3	2.141148845	0.109443181	2.712012956	0.034924845	4.853161801	3.327711234	7.60566235	7.858115942	9.256998085	9.33776001	9.734335478	9.986288828
ENSG00000119042	SATB2	3.298972655	0.006549526	1.550433926	0.235050017	4.849406581	6.009025997	6.311476754	2	8.874592369	8.7882247	7.472665674	6.632790505
ENSG00000011198	ABHD5	2.759273634	0.148420579	2.090042425	0.278555341	4.849316059	2	7.315436877	3.369317972	8.559529208	8.5323134	8.035995406	7.718159092
ENSG00000090905	TNRC6A	2.587158585	0.001576415	2.262059038	0.0062626	4.849217622	7.096806373	8.540087354	6.322058544	10.1651988	10.2193301	10.0366402	9.678488127
		10.04014075	5.00E-12	-5.191151262	0.024397067	4.848989483	2	2	2	10.03270295	10.1100487	2	2
ENSG00000127511	SIN3B	2.279647042	0.010409823	2.56666106	0.0032699	4.846308102	5.330594134	7.874700259	7.896971941	9.669187952	9.68122943	10.00654186	9.913880743
ENSG00000184254	ALDH1A3	2.624610763	0.158647869	2.216774328	0.232107541	4.841385091	2	3.686303414	2	4.623314311	4.68029874	4.720468739	3.753786374
ENSG00000107897	ACBD5	2.66114483	0.063226377	2.179471978	0.130474522	4.840616809	3.327711234	7.790474954	5.156626401	9.133031936	9.06390621	8.640073371	8.602408528
ENSG00000139793	MBNL2	2.733650716	0.07209881	2.106804991	0.170631309	4.840455707	2	7.572420273	6.718950348	9.380118153	9.28585881	8.852523173	8.555744353
ENSG00000067057	PFKP	2.624311401	2.93E-06	2.212709375	9.77E-05	4.837020776	9.2903348	8.762721501	7.858115942	11.33699991	11.392601	10.7229835	11.15342532
ENSG00000122870	BICC1	2.219292907	1.51E-05	2.610580646	2.21E-07	4.829873554	7.384433967	7.761270542	8.296287027	10.07363422	10.0451865	10.00939041	10.78629737
ENSG00000147650	LRP12	2.557362577	0.122925492	2.272207839	0.168397072	4.829570416	2	8.029695761	7.97167868	9.93140666	9.98484711	9.65506359	9.693624218
ENSG00000010404	IDS	2.326556374	1.70E-06	2.502964084	2.06E-07	4.829520458	8.928409675	8.762721501	7.858115942	10.89724025	10.9121868	10.82675092	11.2967558
ENSG00000143013	LMO4	3.710566359	6.96E-06	1.11660835	0.207307325	4.827174709	7.27616306	7.73146271	5.59507254	10.7879368	10.80663	8.716436576	7.455265057
ENSG00000103540	CCP110	2.693317485	0.076989387	2.133106352	0.16480724	4.826423837	2	6.464272742	4.058998049	7.794595599	7.7515168	7.524562403	6.847559257
ENSG00000186111	PIP5K1C	1.711234209	0.168832359	3.11383577	0.006699223	4.825069978	5.70932728	8.557180776	9.658943166	10.35318724	10.4235527	11.45231606	12.06132282
ENSG00000205364	MT1M	3.571145236	0.231366574	1.253401423	0.699127293	4.82454666	2	2	2	3.881293667	4.12711147	3.059555873	2.172608095
ENSG00000100425	BRD1	2.261747741	0.244284615	2.560447902	0.174059362	4.822195644	2	7.790474954	4.058998049	8.62888906	8.4705529	8.940719573	8.74825912
ENSG00000163132	MSX1	1.706284893	0.016446525	3.110446584	2.78E-06	4.816731477	6.366709671	7.274681896	5.59507254	8.310225403	8.21109983	9.285896341	9.946951964
ENSG00000060558	GNA15	1.85766975	0.558508397	2.958562374	0.318708894	4.816232124	2	2	2	2.784612485	3.08597292	4.111244182	2.70970617
ENSG00000187097	ENTPD5	2.603366888	4.78E-07	2.212772867	2.19E-05	4.816139755	5.70932728	5.131603625	5.930865648	8.067573917	8.12931531	7.538302335	7.87218043
ENSG00000070444	MNT	2.323334258	0.182906893	2.492555376	0.142685271	4.815889633	3.327711234	7.73146271	3.369317972	8.508095759	8.60672783	8.490981547	8.927653397
ENSG00000055813	CCDC85A	3.283522776	0.062977541	1.531345992	0.416586577	4.814868768	2	2	4.058998049	5.319220934	5.41912973	3.429858444	4.279527956
ENSG00000270800	RPS10-NUDT	10.00314705	6.10E-12	-5.191151287	0.024411167	4.811995768	2	2	2	10.03383996	10.0362388	2	2
ENSG00000171988	JMJD1C	2.951420754	0.000334613	1.85959874	0.031149835	4.811019494	5.70932728	8.234834549	7.818184403	10.52636311	10.52338	9.4692111	9.405009888
ENSG00000134294	SLC38A2	2.849246014	0.002273448	1.959875466	0.042922706	4.809121481	8.19931886	11.05905903	10.28977157	13.10421017	13.1090105	12.49360536	11.87647387
ENSG00000145819	ARHGAP26	3.593856344	3.83E-12	1.215261815	0.032897253	4.809118159	5.095959458	5.946638857	5.930865648	9.239364442	9.18219179	7.090465544	6.61544141
ENSG00000135686	KLHL36	1.292351984	0.273065021	3.513620738	0.000749648	4.805972722	7.435667355	8.169643011	4.523675391	8.60460802	8.62325378	10.86667347	10.77921995
ENSG00000065054	SLC9A3R2	1.479252429	0.247900443	3.324195243	0.004035407	4.803447672	4.815609471	8.557180776	6.629488387	8.874592369	8.84312657	10.39991289	10.93967577
ENSG00000157368	IL34	-0.191363728	0.902307277	4.99414876	9.20E-06	4.802785032	2	4.442710129	3.369317972	3.79808662	3.2184422	7.552954291	8.54661996
ENSG00000119714	GPR68	6.522618445	9.77E-05	-1.720115929	0.370546992	4.802502516	6.009025997	2	2	10.7993477	10.8703199	3.517841605	3.100202476
ENSG00000093167	LRRFIP2	2.278335641	5.79E-06	2.524073363	3.67E-07	4.802409004	7.579279349	7.701025996	6.43197674	9.624603733	9.57788596	9.834108158	9.857810118
ENSG00000174640	SLCO2A1	2.372155284	0.232847072	2.428018112	0.210772821	4.800173396	2	3.076665209	2	3.921164147	3.74316441	3.891700877	3.858622973
ENSG00000157540	DYRK1A	2.394609959	0.292274211	2.403991123	0.280132344	4.798601083	3.327711234	8.623598428	2	9.405814997	9.50355257	9.454515095	9.475045344
ENSG00000186432	KPNA4	2.869912299	0.000957099	1.928030189	0.032925752	4.797942488	5.866944937	8.234834549	8.511888463	10.79533062	10.7375851	9.911676379	9.738960951
ENSG00000166889	PATL1	2.868687053	0.084412675	1.927363274	0.258077792	4.796050327	2	8.317412869	7.734844356	10.34588171	10.3033068	9.341509511	9.432159874
ENSG00000138639	ARHGAP24	3.601021371	0.001899922	1.19468277	0.355912095	4.795704141	7.940205782	6.04685525	4.058998049	10.31675113	10.3296366	8.09796972	7.753709246
ENSG00000187792	ZNF70	1.448363288	0.099215121	3.345815592	2.06E-05	4.794178879	5.330594134	3.686303414	4.058998049	5.731694	5.8245091	7.802823992	7.357281305
ENSG00000185305	ARL15	2.296679603	0.188069595	2.495207393	0.141446273	4.791886996	2	5.303523747	2	6.213314212	5.85535975	6.339575235	6.117095472
ENSG00000196569	LAMA2	1.4195239	0.286782071	3.367576905	0.004848864	4.787100805	4.467311984	8.671479029	7.734844356	9.12771691	9.17405701	10.64490073	11.43217636

ENSG00000050820	BCAR1	2.47057639	0.000211512	2.316410856	0.000528448	4.786987246	8.042902253	8.213429689	9.61300511	11.21449494	11.3100976	11.03808111	11.17695249
ENSG00000174306	ZHX3	1.531618879	4.80E-05	3.25203491	9.01E-20	4.783653789	7.384433967	7.431218269	7.818184403	9.026254405	9.09977716	10.55911238	10.96521391
ENSG00000115380	EFEMP1	2.998621371	0.029383982	1.784919299	0.214737844	4.78354067	10.24321353	5.303523747	8.35328246	12.02313643	12.0305632	10.75908557	10.86875488
ENSG00000198576	ARC	3.469809768	0.181904381	1.311782873	0.640863647	4.781592641	2	2	2	3.79808662	4.05806467	2.911488689	2.466008509
ENSG00000113269	RNF130	2.865844886	5.98E-05	1.915138735	0.009905697	4.780983621	6.73718411	7.051611315	4.874587708	9.357592295	9.26877178	8.443467705	8.295903454
ENSG00000153560	UBP1	2.846276786	0.079381692	1.934370142	0.244978858	4.780646927	8.138771685	7.790474954	2	10.2360652	10.2044302	9.365126412	9.258230494
ENSG00000257529	RPL36A-HNR	9.971675083	8.02E-12	-5.191151269	0.024561444	4.780523814	2	2	2	9.95800436	10.0479713	2	2
ENSG00000130158	DOCK6	2.43712143	9.89E-10	2.342013856	3.85E-09	4.779135285	6.009025997	6.04685525	6.322058544	8.473317843	8.5130942	8.44402928	8.353097069
ENSG00000112186	CAP2	1.255452465	0.33027738	3.523659916	0.001950899	4.779112381	3.327711234	5.131603625	7.029880454	7.206526483	6.88657603	9.144833386	9.419720027
ENSG00000118482	PHF3	2.551283859	0.146327881	2.223725421	0.203846251	4.77500928	2	7.467835385	8.65477198	10.11987514	10.1411573	10.06192211	9.49042201
ENSG00000138594	TMOD3	2.090231227	0.010985106	2.682969455	0.000733749	4.773200682	6.366709671	8.623598428	8.380957451	10.18733762	10.1222557	10.48122876	10.96997524
ENSG00000197016	ZNF470	2.659936215	0.035079261	2.111457041	0.098726147	4.771393256	3.327711234	3.686303414	6.43197674	7.626911583	7.72113133	7.284811203	6.977399345
ENSG00000104866	PPP1R37	2.301297364	0.118963488	2.468563181	0.087222372	4.769860545	4.00722531	4.11352228	7.691296561	8.543652346	8.5606705	8.743605802	8.691439168
ENSG00000137193	PIM1	1.060097271	0.18784302	3.709734681	8.99E-08	4.769831952	4.815609471	5.72254323	5.39244166	6.394182228	6.32824132	9.40691683	8.280260977
ENSG00000188993	LRRC66	3.642355167	0.214894239	1.127263972	0.726496359	4.769619139	2	2	2	3.881293667	4.22485516	2.987421008	2.172608095
ENSG00000144233	AMMECR1L	2.118831784	0.112817684	2.65038275	0.039226073	4.769214534	3.327711234	6.78762695	7.934808822	9.054539304	8.95773559	9.556714309	9.513016723
ENSG00000196862	RGPD4	9.957208482	8.41E-12	-5.19115129	0.02453583	4.766057192	2	2	2	10.01092762	9.96730153	2	2
ENSG00000115738	ID2	3.856845812	0.002192022	0.908207025	0.531253192	4.765052837	6.89194353	6.389896633	2	9.860122857	9.9170074	6.91847879	7.040043908
ENSG00000104885	DOT1L	1.636004215	0.056298013	3.128688962	7.45E-05	4.764693177	5.532353208	7.928237109	6.882775709	8.671883119	8.74494589	10.254464	10.12804559
ENSG00000144747	TMF1	2.869627795	0.000400793	1.892596788	0.025292481	4.762224583	7.790810999	8.234834549	5.772716771	10.43335299	10.4482162	9.596154868	9.326839168
ENSG00000174099	MSRB3	1.227681112	0.370269344	3.533171386	0.003415182	4.760852498	9.410448395	7.638155693	5.156626401	9.492725629	9.44559847	11.76676501	11.76989677
ENSG00000114353	GNAI2	1.944140792	0.000372061	2.813366071	9.35E-08	4.757506863	10.65885801	10.42046463	9.385998894	12.20067981	12.1847779	12.83730227	13.2553564
ENSG00000008710	PKD1	2.163144042	0.002577478	2.590610002	0.000218902	4.753754045	9.613202746	8.687092496	10.49998282	11.8899302	11.9862429	12.67177419	11.97741506
ENSG00000170100	ZNF778	1.50183249	0.482249983	3.251759843	0.093750012	4.753592333	2	6.228547981	2	6.104969671	6.26836889	7.600074836	8.118139752
ENSG00000182359	KBTBD3	1.788924073	0.337673274	2.96097947	0.08700755	4.749903543	2	3.686303414	2	4.105493497	3.86943969	5.274160001	4.522269241
ENSG00000112877	CEP72	1.938988358	0.360070875	2.810438802	0.156791365	4.74942716	2	3.076665209	2	3.754611435	3.28037825	4.378961816	3.908316285
ENSG00000174839	DENND6A	2.461772544	6.62E-05	2.286907519	0.000211681	4.748680063	4.815609471	5.838937808	4.523675391	7.590284224	7.53039799	7.408510937	7.370638252
ENSG00000148660	CAMK2G	1.525206429	0.115460825	3.223180199	0.000251101	4.748386628	4.815609471	7.60566235	7.398165132	8.501535708	8.5606705	10.15232487	10.28186948
ENSG00000198863	RUNDC1	1.757552263	0.017287716	2.989836208	1.59E-05	4.747388471	5.330594134	7.35507212	6.43197674	8.388752656	8.27714776	9.546029585	9.565907066
ENSG00000176225	RTTN	3.987047516	3.31E-05	0.758465213	0.503061508	4.745512729	5.532353208	5.131603625	2	8.625876185	8.64842725	5.85994354	5.08721802
ENSG00000170677	SOCS6	2.666976517	0.082560548	2.077863144	0.179747748	4.744839662	6.009025997	7.538394143	2	9.087765067	8.9648262	8.553208128	8.325926932
ENSG00000157510	AFAP1L1	0.666686287	0.606448179	4.07734749	0.000113649	4.744033777	3.327711234	4.93638933	6.80318718	6.328984825	6.21386141	9.495184079	9.723717339
ENSG00000172175	MALT1	2.637780564	0.06531578	2.106162001	0.144649282	4.743942565	2	6.844429755	7.098165388	8.959612762	9.03387903	8.480070636	8.461101072
ENSG00000151151	IPMK	2.658120579	0.145039687	2.085699804	0.25649001	4.743820382	2	5.595927442	2	6.693715713	6.65485813	6.344396176	5.876154317
ENSG00000163683	SMIM14	1.595527996	0.002937529	3.146073905	4.18E-10	4.741601901	8.777722034	7.979858146	7.502634626	9.788505066	9.74164918	11.21466007	11.40213888
ENSG00000115365	LANCL1	1.934307186	0.01984582	2.802401205	0.00039686	4.736708391	7.867441304	7.874700259	5.59507254	9.350309099	9.33867006	10.29596165	10.1155387
ENSG00000122335	SERAC1	1.152697247	0.423308674	3.583316511	0.004287245	4.736013759	6.816637977	5.838937808	2	6.907295206	7.00595521	9.193069097	9.491101628
ENSG00000186812	ZNF397	1.591942856	0.493419043	3.143683357	0.139684521	4.735626213	6.563806279	2	2	6.580005553	6.53928196	8.347678913	7.727417522
ENSG00000198846	TOX	1.019291148	0.839558946	3.716181037	0.398978105	4.735472185	2	2	2	3.023133209	2	3.429858444	4.584731282
ENSG00000141564	RPTOR	2.426331014	0.113542889	2.304591782	0.128348113	4.730922796	7.384433967	7.467835385	2	9.258944141	9.23886123	9.027210383	9.222253259
ENSG00000112406	HECA	2.082849161	0.000150081	2.646874148	7.60E-07	4.729723309	7.159102577	6.04685525	6.203069917	8.622857004	8.53549186	9.333045295	8.914540669
ENSG00000118655	DCLRE1B	1.544614728	0.428119634	3.184465291	0.072608488	4.729080019	2	5.72254323	2	5.731694	5.76075522	7.121690475	7.478755384
ENSG00000104695	PPP2CB	2.201526865	0.000290549	2.525133361	2.35E-05	4.726660226	8.285626337	8.213429689	6.718950348	10.0774988	10.0901921	10.39687046	10.41585888
ENSG00000253953	PCDHGB4	4.501297502	0.000655225	0.223670094	0.895844052	4.724967595	3.327711234	4.442710129	7.342949392	10.39138945	10.4372122	5.790986459	6.538630327
ENSG00000124177	CHD6	2.30492783	0.02131845	2.41986879	0.014538236	4.72479662	8.517314938	9.00621767	5.930865648	10.58338644	10.5940591	10.44717886	10.92088325
ENSG00000141294	LRRC46	2.389914625	0.391949897	2.333363493	0.391460007	4.723278118	2	2	2	3.227757282	3.2184422	3.059555873	3.311948089
ENSG00000071794	HLTF	1.46257296	0.318121128	3.260647481	0.014179007	4.723220441	2	6.951736442	6.322058544	7.590284224	7.47518984	9.299924348	9.319205044
ENSG00000269067	ZNF728	4.444206617	0.037659821	0.276625081	0.918154036	4.720831698	2	2	2	4.694064037	4.72553021	2.3070652	2.466008509
ENSG00000175182	FAM131A	1.989529539	0.101224495	2.730093153	0.018317011	4.719622692	2	5.946638857	5.930865648	7.341208691	7.24992376	8.258546141	7.738907626
ENSG00000117758	STX12	2.455303158	0.098944573	2.263676718	0.12483688	4.718979877	7.096806373	8.747909548	3.369317972	10.03724565	10.0124836	9.737316722	9.924737419
ENSG00000015532	XYLT2	2.304372354	0.022979492	2.413307627	0.016137174	4.717679981	4.815609471	7.538394143	8.042706615	9.579376566	9.60234163	9.753037939	9.643457842
ENSG00000185896	LAMP1	2.467794043	0.000177625	2.249353078	0.000679074	4.717147121	10.00110988	8.876041558	10.66102425	12.47045468	12.4902458	12.11174512	12.39826717
ENSG00000169756	LIMS1	2.463767611	1.22E-14	2.250135684	1.78E-12	4.713903294	8.674867122	8.297207175	8.143093325	10.8713966	10.8030078	10.7387464	10.50031203
ENSG00000179869	ABCA13	2.867963601	0.000218272	1.84259875	0.023328609	4.710562352	4.815609471	4.93638933	5.39244166	7.764774849	7.77859536	6.031172603	7.261744957
ENSG00000278461		3.014261933	8.24E-06	1.695242991	0.017946326	4.709504924	4.00722531	5.303523747	4.523675391	7.691737974	7.45182838	6.173421703	6.420873184
ENSG00000132640	BTBD3	1.451749851	0.588240473	3.256740151	0.181428238	4.708490001	2	8.147241016	2	8.010962573	8.0358969	9.657971023	9.952144095
ENSG00000135241	PNPLA8	3.092272547	0.014980237	1.615821967	0.231005457	4.708094515	6.96351253	8.004992147	3.369317972	10.09394503	10.0907323	8.645942939	8.604294318
ENSG00000173372	C1QA	-2.311499221	0.415158868	7.018722266	0.00011303	4.707223045	2	2	2	2	2.14022021	7.399274054	6.690737414
ENSG00000124466	LYPD3	4.168470658	0.001516222	0.53861101	0.736592879	4.707081668	2	4.11352228	2	6.687949235	6.51372389	3.336159116	3.698365557
ENSG00000130311	DDA1	1.93107328	0.011671303	2.775995215	0.000147509	4.707068495	7.940205782	8.213429689	6.322058544	9.599482126	9.63011121	9.978130526	10.815215
ENSG00000182446	NPLOC4	2.196413452	0.003108658	2.508251783	0.000574909	4.704665235	8.631580145	9.338312924	7.225742779	10.82486117	10.8288217	11.11450432	11.16123925
ENSG00000265972	TXNIP	2.43016199	0.000809887	2.2727605	0.001788445	4.70292249	11.39439513	10.22039562	9.658943166	13.00776242	13.0681681	13.22911886	12.42109226
ENSG00000187796	CARD9	0.72685174	0.738608861	3.975287714	0.026249826	4.702139454	3.327711234	2	2	2.784612485	2.94009902	5.385935071	4.836507146
ENSG00000143412	ANXA9	-1.31051372	0.609315815	6.011610918	0.000843969	4.701097198	2	2	2	2.141737246	2.14022021	5.951260634	6.276965131
ENSG00000131037	EPS8L1	2.667086297	0.087812072	2.032813627	0.196453617	4.699899924	3.327711234	2	3.369317972	4.76150579	4.83276841	4.434275199	4.09155917
ENSG00000116685	KIAA2013	2.364688365	0.172285908	2.331022268	0.170945512	4.695710633	8.564112515	8.029695761	2	10.10155624	10.0777096	10.07684508	10.03510519
ENSG00000173262	SLC2A14	7.87244024	1.40E-07	-3.180102844	0.101051926	4.692337396	2	2	3.369317972	8.899755849	8.84568954	2.139668783	2
ENSG00000235098	ANKRD65	2.082628425	0.148587465	2.60903543	0.058149869	4.691663855	2	3.076665209	4.058998049	4.984880435	4.53541753	4.881704255	5.524371359
ENSG00000206140	TMEM191C	3.546620212	0.168573196	1.137300979	0.687792014	4.683921191	2	2	2	3.881293667	4.0930011	2.831336763	2.421067459
ENSG00000153165	RGPD3	9.874894914	1.41E-11	-5.191151308	0.024699035	4.683743607	2	2	2	9.903687576	9.91090151	2	2
ENSG00000108509	CAMTA2	2.266074758	0.006973831	2.416663798	0.003546828	4.682738555	5.532353208	7.701025996	8.007629717	9.580933135	9.72573666	9.866098684	9.741247946
ENSG00000091831	ESR1	1.858549446	0.519421794	2.822232137	0.292236517	4.680781583	2	2	2	2.868578001	3.01487888	3.679182183	3.261853426
ENSG00000270181	BIVM-ERCC5	9.867533259	1.49E-11	-5.191151307	0.024725305	4.676381952	2	2	2	9.9123685	9.88746056	2	2
ENSG00000158427	TMSB15B	3.819206528	0.106744126	0.857035831	0.74998849	4.676242359	2	2	2	4.268917409	4.12711147	2.457042231	2.632958241
ENSG00000120662	MTRF1	2.491149602	0.133069745	2.184276196	0.185851802	4.675425797	2	5.131603625	2	6.043074888	6.08095082	5.696113106	5.85096143
ENSG00000125952	MAX	2.836824107	8.10E-07	1.837694776	0.002094646	4.674518883	8.107517749	6.78762695	6.882775709	10.17502299	10.2094139	9.163367078	9.23204367
ENSG00000079432	CIC	1.967570814	0.238207033	2.703394439	0.089199351	4.670965253	3.327711234	8.169643011	9.61300511	10.4372252	10.4587184	10.98899358	11.35251454
ENSG00000171606	ZNF274	2.377915927	0.177300996	2.293026132	0.186484076	4.670942058	4.00722531	7.099070191	2	8.03159949	8.01099479	8.305655918	7.442672408
ENSG00000196437	ZNF569	2.473383903	0.185725588	2.195620297	0.237878752	4.6690042	2	5.595927442	2	6.522845756	6.46787796	6.550361701	5.821002687
ENSG00000167641	PPP1R14A	-1.96494491	0.05126002	6.633263113	4.09E-15	4.668318203	4.815609471	5.457120953	2	3.09461492	3.45167086	11.30777198	10.91012447
ENSG00000090975	PITPNM2	2.229003061	0.107251275	2.436874049	0.070958342	4.665877111	2	6.602424674	5.156626401	7.677258395	7.65252709	7.913205701	7.823364542
ENSG00000073670	ADAM11	0.858624886	0.761711449	3.806692124	0.108864366	4.66531701	2	2	2	2.498691189	2.59489879	4.378961816	3.972018587
ENSG00000038002	AGA	2.711919859	0.10980137	1.953236653	0.257630995	4.665156512	3.327711234	6.464272742	2	7.683067677	7.70145652	6.437578914	7.331709059
ENSG00000162066	AMDHD2	3.648696788	0.003512781	1.015110784	0.474513246	4.663807572	3.327711234	5.595927442	2	7.697489359	7.95749494	5.31402805	5.337485258
ENSG00000159263	SIM2	0.279018914	0.930366193	4.381707389	0.079386828	4.660726303	2	2	2	2.600425781	2.14022021	4.620674722	4.692661645
ENSG00000175895	PLEKHF2	2.316448096	0.043740293	2.344018533	0.038597696	4.660466629	4.815609471	5.838937808	2	7.026323566	7.1744553	7.3389565	6.883203642
ENSG00000168038	ULK4	2.144167381	0.170194337	2.514449294	0.094858183	4.658616674	2	4.442710129	2	4.907595604	5.21224859	5.196301326	5.576534358
ENSG00000130827	PLXNA3	2.193679761	0.007925031	2.464694479	0.002373092	4.658374239	6.816637977	8.191702463	9.180874355	10.50725261	10.5675219	10.60176359	10.98850403
ENSG00000105063	PPP6R1	2.298179606	0.025564784	2.359971456	0.020815791	4.658151062	5.095959458	8.376385542	7.734844356	9.864602909	9.88372429	9.931435456	9.939999927
ENSG00000139718	SETD1B	2.344107313	0.120721992	2.312917027	0.120253602	4.65702434	7.485143553	6.389896633	2	8.782894215	8.75316018	8.787633372	8.684919451
ENSG00000121797	CCRL2	0.858622531	0.763635326	3.798138503	0.112770502	4.656761034	2	2	2	2.498691189	2.59489879	4.425202323	3.8919413
ENSG00000115760	BIRC6	2.424258428	0.028967364	2.232022951	0.044399174	4.656281379	5.866944937	9.461450882	9.049077186	11.12929738	11.2037318	10.91081207	11.03666497
ENSG00000104312	RIPK2	3.379558245	3.80E-11	1.275480082	0.022326117	4.655038327	8.042902253	7.189546432	6.80318718	10.78995706	10.7847595	8.506119506	8.860338475
ENSG00000129317	PUS7L	1.694608589	0.341002462	2.960354592	0.073190118	4.654963181	3.327711234	6.602424674	2	6.820355514	6.84106088	8.092249024	8.040411184
ENSG00000178105	DDX10	1.982316489	0.091493251	2.671733701	0.017257852	4.65405019	7.159102577	6.728495673	3.369317972	8.312101014	8.42842973	8.720150661	9.325315571
ENSG00000144857	BOC	2.041465191	0.110115082	2.609986731	0.03302563	4.651451921	2	5.72254323	4.058998049	6.664650034	6.37866593	7.021403672	7.130061054
ENSG00000107338	SHB	2.624976303	0.052851591	2.025738468	0.140205611	4.650714771	3.327711234	7.790474954	5.930865648	9.217515344	9.17405701	8.234726135	8.894131604
ENSG00000174151	CYB561D1	2.356590949	0.026235442	2.293302156	0.029927201	4.649893105	3.327711234	6.728495673	5.59507254	8.074220386	8.07355593	8.125537812	7.887748062
ENSG00000110330	BIRC2	3.077421387	0.009582037	1.57171565	0.21425456	4.649137036	4.815609471	8.981105197	7.896971941	11.08446011	11.0544122	9.703475817	9.420076944
ENSG00000112367	FIG4	1.735619492	0.452840011	2.91168602	0.174638772	4.647305512	2	6.844429755	2	6.846474326	7.16216989	8.196576732	8.130406117
ENSG00000134780	DAGLA	1.705395527	0.112733934	2.941228176	0.003132042	4.646623703	2	4.710564154	5.59507254	6.147608921	6.33555364	7.297296608	7.562617572
ENSG00000146411	SLC2A12	-0.269907868	0.845522872	4.914543319	2.05E-06	4.644635452	6.138360429	5.303523747	2	5.040242897	4.98931816	9.60873181	10.33090604
ENSG00000189120	SP6	2.333643931	0.440702227	2.310744596	0.433987431	4.644388527	2	2	2	3.227757282	3.15372745	3.5515855	2.671842496
ENSG00000213516	RBMXL1	2.725522521	0.019173548	1.915673247	0.109758105	4.641195768	4.00722531	7.572420273	5.772716771	9.16555511	9.09547637	7.977459036	8.611187869
ENSG00000256087	ZNF432	2.755480421	0.023372934	1.883162516	0.132614409	4.638642937	2	6.228547981	6.073378139	8.235106869	8.30721295	7.594015257	7.20984412
ENSG00000181894	ZNF329	1.992607967	0.015889499	2.645733562	0.00085525	4.638341529	3.327711234	5.595927442	5.930865648	7.234487582	7.17037177	7.980559086	7.687652155
ENSG00000138399	FASTKD1	1.676997459	0.075317795	2.960424016	0.000709386	4.637421475	7.53297911	10.24169813	9.528916236	11.18792842	11.1077025	12.27036227	12.57426012

ENSG00000104783	KCNN4	0.092420682	0.966177728	4.544785493	0.007602279	4.637206176	5.70932728	2	2	4.470469939	4.2865223	8.768928693	8.272375674
ENSG00000101574	METTL4	2.472055089	0.228871904	2.163034257	0.290390881	4.635089346	2	6.228547981	2	6.984595707	7.24216308	7.019897747	6.587749895
ENSG00000165152	TMEM246	2.238510944	0.340614989	2.395475552	0.295009548	4.633986496	2	7.189546432	2	7.826439377	7.86451142	7.854430723	8.132149972
ENSG00000188277	C15orf62	1.014336527	0.731092219	3.618129302	0.153081117	4.632465829	2	2	2	2.784612485	2.38539787	4.155805465	3.924507493
ENSG00000100410	PHF5A	2.276763331	0.011761813	2.355551093	0.008462275	4.632314424	6.563806279	4.93638933	4.058998049	7.677258395	7.77859536	7.80632247	7.805990373
ENSG00000010318	PHF7	2.100297411	0.17960083	2.530480908	0.092183627	4.630778319	2	4.11352228	2	4.739373666	4.65713986	5.180212365	4.951586397
ENSG00000164733	CTSB	2.832325181	4.02E-09	1.79831437	0.00030473	4.630639551	12.49796133	11.76214832	11.62203638	14.83208287	14.8581549	14.118582	13.42052145
ENSG00000189190	ZNF600	2.747083529	0.04265939	1.88075426	0.176369212	4.627837789	2	6.04685525	4.058998049	7.444001439	7.5014335	6.818078339	6.420873184
ENSG00000163121	NEURL3	4.433857648	0.008153101	0.193885068	0.929171616	4.627742716	2	2	3.369317972	5.348759107	5.72778967	2.62493832	2.671842496
ENSG00000144655	CSRNP1	2.295984228	0.019121386	2.328381143	0.016731819	4.624365371	5.330594134	7.819099893	5.772716771	8.980991923	9.04507321	8.914214292	9.165908829
ENSG00000181904	C5orf24	1.565580316	1.57E-07	3.057486267	2.01E-26	4.623066584	8.042902253	7.847167909	8.042706615	9.601017164	9.44559847	11.11176776	10.90528859
ENSG00000125753	VASP	1.804799693	0.087057774	2.815104861	0.004461713	4.619904554	5.70932728	8.191702463	8.922968322	9.852410253	9.9489441	10.7202043	11.07547412
ENSG00000157426	AASDH	2.13912798	0.050098285	2.479999996	0.019580896	4.619127977	2	5.457120953	4.523675391	6.529309835	6.5890786	7.07314455	6.674075308
ENSG00000145780	FEM1C	2.410474806	0.202020098	2.204301086	0.24003034	4.614775893	4.467311984	7.928237109	2	8.877128559	8.84312657	8.800408416	8.495377414
ENSG00000047621	C12orf4	2.389596536	0.035285398	2.220908737	0.049717407	4.610505273	3.327711234	6.666836837	4.874587708	7.913023977	7.8441483	7.613117257	7.80708241
ENSG00000163156	SCNM1	2.82649748	1.45E-05	1.783254097	0.008792063	4.609751577	5.330594134	4.442710129	6.073378139	8.058664058	8.1187584	6.898949899	7.219718817
ENSG00000175793	SFN	1.778306598	0.560280703	2.83134867	0.320348511	4.609655268	2	2	2	3.023133209	2.77779907	2.937246078	3.8919413
ENSG00000134996	OSTF1	2.744378563	0.078977124	1.863847028	0.244680076	4.608225591	4.00722531	6.666836837	2	8.063125866	7.92393105	7.08038692	7.184857568
ENSG00000115841	RMDN2	3.383164901	0.000657972	1.221282107	0.267803599	4.604447008	2	3.686303414	4.523675391	6.70517998	6.56439512	4.764298067	4.553838275
ENSG00000144674	GOLGA4	2.406675371	1.03E-06	2.196540665	9.02E-06	4.603216036	8.313285031	9.13702746	8.007629717	11.00233175	10.938838	10.85879791	10.65660252
ENSG00000070770	CSNK2A2	1.459765371	0.347773435	3.141527708	0.026364101	4.601293079	7.384433967	6.78762695	2	7.93753157	8.01327646	9.651421039	9.632103066
ENSG00000119684	MLH3	2.695488514	0.093060766	1.904905627	0.244428647	4.600394141	4.815609471	7.189546432	2	8.581467308	8.46556258	8.031518488	7.388257299
ENSG00000156510	HKDC1	2.151756149	0.485453797	2.447322834	0.410308224	4.599078983	2	2	2	3.349643765	2.77779907	2.803598391	3.600986722
ENSG00000197860	SGTB	2.053696164	0.03605895	2.54084682	0.007353946	4.594542984	4.00722531	7.002537973	6.534109959	8.302698479	8.32752521	9.020825125	8.532164007
ENSG00000105967	TFEC	0.689489289	0.823523332	3.903113734	0.122897491	4.592603024	2	2	2	2.498691189	2.49394783	4.764298067	3.496556178
ENSG00000104219	ZDHHC2	2.181343352	0.167549493	2.410331359	0.116655583	4.591674711	4.00722531	7.538394143	3.369317972	8.341783829	8.22100445	8.309975344	8.683730876
ENSG00000124074	ENKD1	2.163759701	0.257710703	2.427498348	0.190345876	4.591258049	2	5.72254323	2	6.463327516	6.17362678	6.637393426	6.514766253
ENSG00000073849	ST6GAL1	0.016502355	0.991315828	4.56823799	3.36E-05	4.584740345	4.00722531	5.838937808	2	4.739373666	4.79082474	9.194070966	8.995324202
ENSG00000166801	FAM111A	2.70970505	0.01749407	1.873234064	0.111040183	4.582939113	3.327711234	7.189546432	6.073378139	8.883449599	8.87484109	8.038228667	8.06618078
ENSG00000275214		11.57769263	6.99E-21	-6.99822544	0.000718107	4.579467186	2	3.686303414	2	13.4043705	13.4189574	2	2
ENSG00000121671	CRY2	1.749419715	0.219370406	2.828735061	0.033929555	4.578154776	5.70932728	6.844429755	2	7.477814553	7.58663873	8.748160468	8.425990514
ENSG00000146232	NFKBIE	2.270999171	0.135425564	2.304760314	0.122835348	4.575759485	2	7.189546432	6.43197674	8.471640654	8.56844956	7.985968197	8.960904642
ENSG00000177464	GPR4	3.608170841	0.054422065	0.962783204	0.64966917	4.570954045	3.327711234	2	2	4.443344104	5.11361705	3.059555873	2.885414003
ENSG00000110446	SLC15A3	-3.753164585	0.151557124	8.323647244	9.48E-07	4.570482659	2	2	2	2	2	8.551124447	8.157201821
ENSG00000155090	KLF10	2.023207184	0.000282479	2.547083008	2.79E-06	4.570290192	8.228665467	7.393647515	7.285536013	9.695291778	9.70330142	10.56712557	9.764764027
ENSG00000139597	N4BP2L1	1.461837607	0.436838812	3.102218533	0.068532826	4.56405614	2	5.303523747	2	5.363304456	5.18011485	7.184873526	6.301968891
ENSG00000101974	ATP11C	1.742432707	0.160411864	2.820799844	0.015161691	4.56323255	4.00722531	7.819099893	6.322058544	8.534041678	8.39031583	9.259659123	9.763920691
ENSG00000197323	TRIM33	2.839267278	0.044631453	1.722595792	0.241877934	4.56186307	3.327711234	8.029695761	5.772716771	9.614759498	9.52369893	8.619836896	8.284971554
ENSG00000182902	SLC25A18	1.150926308	0.693061884	3.407996869	0.179503712	4.558923176	2	2	2	2.784612485	2.49394783	3.917810786	3.858622973
ENSG00000156671	SAMD8	2.466272479	0.000431391	2.091180704	0.003223268	4.557453183	7.709880939	7.901717006	5.930865648	9.85562885	9.85666073	9.547336845	9.415430124
ENSG00000102804	TSC22D1	2.829671901	5.33E-06	1.726467688	0.008092771	4.556139589	10.79750658	9.856140801	9.132850258	12.9227779	12.9054164	11.84225044	11.7806379
ENSG00000187961	KLHL17	1.874392119	0.178652578	2.678461431	0.04234099	4.55285355	4.467311984	6.228547981	2	6.861921612	6.86652414	7.891135869	7.352801367
ENSG00000205571	SMN2	9.739807391	4.22E-11	-5.191151232	0.025435	4.548656159	2	2	2	9.685196338	9.85538952	2	2
ENSG00000172062	SMN1	9.739469131	4.22E-11	-5.191151233	0.025435	4.548317898	2	2	2	9.685196338	9.8547535	2	2
ENSG00000175175	PPM1E	-0.303340129	0.941709668	4.848779903	0.145407903	4.545439774	2	2	2	2.498691189	2	5.333556395	4.720747915
ENSG00000167553	TUBA1C	2.993677919	9.39E-08	1.551470345	0.009202275	4.545148264	8.816894989	8.469613413	8.206335842	11.52096327	11.4772671	9.300854716	10.5556122
ENSG00000119915	ELOVL3	3.332028241	0.280963509	1.212355966	0.716509282	4.544384207	2	2	2	3.663521627	3.98554648	3.035909414	2.172608095
ENSG00000013288	MAN2B2	2.233181757	0.034724134	2.310332443	0.027071029	4.5435142	5.866944937	9.170736405	8.763901704	10.7018566	10.6994819	10.6478287	10.89672248
ENSG00000196975	ANXA4	3.031015746	7.22E-07	1.512398611	0.021023289	4.543414357	7.27616306	5.595927442	6.534109959	9.516508835	9.64710779	8.035250217	8.120777053
ENSG00000126561	STAT5A	2.603073336	0.053580304	1.940147357	0.156889836	4.543220693	2	4.93638933	6.534109959	7.865280446	7.89204953	7.346184975	7.103640702
ENSG00000169715	MT1E	4.702696723	0.008406966	-0.159534351	0.944101725	4.543162373	7.031698319	2	3.369317972	10.14218788	10.1969221	5.899789217	4.766376438
ENSG00000169247	SH3TC2	0.977614717	0.564719306	3.564660514	0.014458835	4.54227523	3.327711234	3.076665209	3.369317972	3.515101962	4.1930041	5.002522427	6.735016458
ENSG00000165355	FBXO33	2.292705991	3.39E-05	2.249221854	4.43E-05	4.541927845	5.330594134	6.666836837	5.930865648	8.321442665	8.35116763	8.392591106	8.186868687
ENSG00000100994	PYGB	1.365729408	0.256781312	3.172249205	0.00344396	4.537978613	8.418903792	9.497318007	5.772716771	9.867156706	9.94057646	11.72642042	11.6863866
ENSG00000259571	BLID	3.116287235	0.233747682	1.421198117	0.609265042	4.537485352	2	2	2	3.754611335	3.60476711	2.717032197	2.782576693
ENSG00000184014	DENND5A	2.470894845	0.000138719	2.06326463	0.001719657	4.534159475	8.855032352	9.680074956	7.934808822	11.45076334	11.4679397	10.91243621	11.18001149
ENSG00000116678	LEPR	1.381987874	0.355101172	3.151001143	0.019679078	4.532989017	4.467311984	6.464272742	2	6.529309835	6.6253296	8.281350892	8.325926932
ENSG00000109846	CRYAB	-0.276020581	0.728428075	4.806918306	2.13E-15	4.530897725	8.257427002	7.393647515	8.865353883	8.036145657	7.99029569	13.43597286	12.59103903
ENSG00000197629	MPEG1	-1.30515308	0.688878059	5.835817726	0.019545711	4.530664646	2	2	2	2.270786764	2	6.275391383	5.5506886
ENSG00000196371	FUT4	2.62609483	0.007362713	1.902868529	0.058813982	4.528963359	3.327711234	6.389896633	5.156626401	7.905589775	8.05595642	7.283556699	7.264927592
ENSG00000013374	NUB1	2.514679366	1.39E-09	2.012650357	1.67E-06	4.527329724	7.940205782	8.147241016	7.163363831	10.28797351	10.3378525	9.795903284	9.829876356
ENSG00000187608	ISG15	5.400089827	9.28E-14	-0.877162753	0.303668356	4.522927074	9.2903348	7.232742069	9.06622548	14.14029863	14.1713234	7.580798201	8.165740501
ENSG00000198914	POU3F3	2.866948486	0.453973535	1.653663415	0.675209517	4.520611901	2	2	2	4.205751501	2.14022021	3.1504466	2.466008509
ENSG00000135966	TGFBRAP1	1.86720493	0.018700929	2.652776259	0.000461516	4.519981189	5.330594134	7.503546079	6.43197674	8.488325436	8.56844956	9.184693023	9.421503729
ENSG00000105664	COMP	3.64416849	0.229538248	0.8757536	0.796725981	4.51992209	2	2	2	4.139692846	3.98554648	2.885263063	2.117335275
ENSG00000066777	ARFGEF1	2.633091534	0.002023832	1.886203102	0.0323856	4.519294636	7.435667355	8.053983481	5.59507254	9.933844998	9.98018912	9.543411506	8.786963584
ENSG00000163346	PBXIP1	2.173395419	0.001407766	2.344629182	0.000481509	4.518024601	8.468948322	8.702538795	6.882775709	10.34496592	10.4049916	10.41345332	10.66746623
ENSG00000166068	SPRED1	1.723638793	0.000146231	2.793810278	1.36E-10	4.517449071	7.33131396	7.503546079	6.534109959	8.966774408	8.81069917	10.08208599	9.814754207
ENSG00000039319	ZFYVE16	2.826510321	3.88E-11	1.690692991	0.000135456	4.517203312	6.816637977	7.669933291	7.163363831	10.10696834	10.0306182	9.108453756	8.748827534
ENSG00000125457	MIF4GD	0.848049931	0.695215075	3.668234398	0.047681033	4.516284329	2	6.04685525	2	5.161714397	5.62411716	8.359038717	7.809264007
ENSG00000164066	INTU	0.824637671	0.553979465	3.690645994	0.001690432	4.515283665	2	5.595927442	4.058998049	5.210797711	5.37722731	8.408216037	7.515294811
ENSG00000137496	IL18BP	2.978467708	0.057973134	1.536185302	0.354201102	4.51465301	4.467311984	6.951736442	2	8.619831492	8.46389529	7.439255893	6.730418989
ENSG00000171823	FBXL14	2.319335158	0.230806091	2.193888372	0.252105582	4.51322353	2	5.838937808	2	6.628980152	6.54560135	6.453284622	6.482320745
ENSG00000115226	FNDC4	1.559509382	0.449705292	2.952845556	0.118891631	4.512354938	3.327711234	7.431218269	2	7.562190249	7.37938471	8.764439224	8.924637944
ENSG00000244617	ASPRV1	1.858525064	0.597963567	2.653665371	0.426580825	4.512190435	2	2	2	2.868578001	3.01487888	3.981090143	2.326754077
ENSG00000158290	CUL4B	3.04918256	0.00515506	1.462155866	0.211603388	4.511338426	5.095959458	8.791896851	7.734844356	10.90533885	10.8529084	9.380756704	9.211159041
ENSG00000177294	FBXO39	3.59715341	0.182411111	0.914153706	0.76219473	4.511307116	2	2	2	4.139692846	3.90918929	2.803598391	2.277179783
ENSG00000166123	GPT2	1.784816194	0.10022545	2.725283379	0.007667651	4.510099574	5.866944937	7.761270542	4.874587708	8.418656444	8.44204342	9.602960031	9.074546388
ENSG00000110274	CEP164	2.420802535	0.006743859	2.086925449	0.021068527	4.507727984	4.00722531	6.78762695	6.203069917	8.451360823	8.40776525	8.275052313	7.90110545
ENSG00000175054	ATR	2.66203026	0.076244071	1.845480495	0.229495532	4.507510754	2	7.274681896	5.772716771	8.652768197	8.86097251	7.985196707	7.922424886
ENSG00000262607		0.346727922	0.883560872	4.157308547	0.024419064	4.504036468	2	2	4.058998049	3.09461492	3.2184422	4.90146639	6.862343163
ENSG00000184898	RBM43	3.330452516	2.07E-07	1.170349373	0.099416364	4.500801889	5.532353208	4.442710129	6.203069917	8.76657222	8.6932307	6.723178551	6.501335855
ENSG00000277224	HIST1H2BF	3.333959738	0.211918038	1.165160935	0.688459235	4.499120674	2	2	2	3.921164147	3.74316441	2.42098416	2.851942824
ENSG00000172915	NBEA	2.201109161	0.028717201	2.297687199	0.020800484	4.49879636	2	5.131603625	5.39244166	6.79375511	6.79406287	6.666558925	7.078537388
ENSG00000100985	MMP9	-3.273856792	0.31862506	7.771405272	0.00125484	4.49754848	2	3.076665209	2	2	2.14022021	9.738690605	4.353147976
ENSG00000120063	GNA13	2.151488964	0.084559691	2.342441124	0.054227008	4.493930089	4.467311984	8.234834549	8.536706852	10.02128323	9.95489142	10.39730548	9.920959478
ENSG00000198168	SVIP	2.623748729	0.193809048	1.867898473	0.362474125	4.491647201	5.866944937	2	2	6.841288255	6.82556437	6.280431262	5.921228818
ENSG00000139044	B4GALNT3	0.279088054	0.93222913	4.209362084	0.106037661	4.488450138	2	2	2	2.600425781	2.14022021	4.166733998	4.793076545
ENSG00000145794	MEGF10	0.863476532	0.78336925	3.621910536	0.172416263	4.485387068	2	2	2	2.784612485	2.2680111	4.017766949	4.077147165
ENSG00000277377		4.417690307	0.037736766	0.06575594	0.980518967	4.483446247	2	2	2	4.739373666	4.63360314	2.3070652	2.374681325
ENSG00000123243	ITIH5	-4.157567537	2.12E-05	8.639601772	3.14E-27	4.482034234	5.095959458	4.11352228	6.203069917	2.784612485	2.38539787	13.48928411	14.11736845
ENSG00000136114	THSD1	3.058979725	0.031675145	1.420138274	0.352050398	4.479117999	2	4.442710129	2	5.900763176	5.91514978	4.547306681	4.365060851
ENSG00000107566	ERLIN1	2.369767068	0.007296304	2.108680534	0.01795557	4.478447602	7.096806373	7.874700259	5.156626401	9.460955886	9.40620612	9.091700798	9.25263028
ENSG00000149634	SPATA25	2.016102074	0.531143362	2.461699069	0.425433438	4.477801143	2	2	2	3.406930997	2.49394783	3.500668998	2.981406154
ENSG00000165626	BEND7	1.124323933	0.516556851	3.353143987	0.026967588	4.477467921	2	4.93638933	2	4.867343068	4.40247105	6.828438696	6.535998174
ENSG00000170248	PDCD6IP	2.288026277	0.00012178	2.189409771	0.000235994	4.477436048	8.797441463	8.981105197	7.451345288	10.84021544	10.8239557	10.6406201	10.82117286
ENSG00000125355	TMEM255A	0.282075773	0.950628899	4.189882878	0.279259581	4.471958651	2	2	2	2.695456494	2	4.461156421	4.532869193
ENSG00000158467	AHCYL2	1.411858859	0.14556217	3.059000066	0.000486858	4.470858925	6.73718411	6.844429755	4.058998049	7.723090567	7.66128317	9.120096958	9.496527079
ENSG00000240344	PPIL3	2.653800486	0.060426137	1.816849579	0.210056903	4.470650065	4.467311984	6.464272742	2	7.797276258	7.8286851	7.182183216	6.782425482
ENSG00000112419	PHACTR2	0.923372171	0.197969988	3.544743656	9.58E-09	4.468115826	8.313285031	9.842262059	9.25752426	10.15216947	10.2247551	12.36480811	13.14363006
ENSG00000104888	SLC17A7	0.495876252	0.866101534	3.97169921	0.087078455	4.467575462	2	2	2	2.3892346	2.49394783	4.360042493	4.279527956
ENSG00000119929	CUTC	2.214721831	0.085392635	2.25116235	0.074947938	4.46588418	4.467311984	6.04685525	2	6.931899482	7.16627666	7.15911073	7.015689447
ENSG00000085514	PILRA	1.697734472	0.351613795	2.767894354	0.102455592	4.465628825	2	3.076665209	4.058998049	4.358765249	4.56059747	6.075339068	4.033026654
ENSG00000183576	SETD3	1.967922546	0.000770044	2.49500032	1.17E-05	4.462922866	6.138360429	7.538394143	7.600047325	9.129845271	9.21824109	9.795243215	9.595281739
ENSG00000181418	DDN	1.012879912	0.73397139	3.449992171	0.179708528	4.462872083	2	2	2	2.695456494	2.49394783	3.56816602	4.201948172
ENSG00000128591	FLNC	1.443149457	0.003480256	3.019069041	6.35E-11	4.462218498	9.497412313	10.59613751	10.30429804	11.60349653	11.6842056	13.06706733	13.35669676
ENSG00000187244	BCAM	0.954622079	0.569254933	3.507549863	0.016033481	4.462171942	7.624139768	6.602424674	2	7.330144653	7.76511961	10.18174778	9.994939827

ENSG00000179627	ZBTB42	2.175234945	0.045106267	2.285351212	0.032219121	4.460586158	2	4.442710129	5.59507254	6.598566398	6.57678965	6.691367378	6.695462882
ENSG00000182240	BACE2	1.747772875	0.025627053	2.710814932	0.000252233	4.458587808	8.468948322	6.464272742	7.225742779	9.314267267	9.37460909	10.15266847	10.43999426
ENSG00000165684	SNAPC4	2.402816313	0.12734523	2.055723441	0.191066494	4.458539754	3.327711234	3.076665209	6.882775709	7.697489359	7.86198169	7.401588825	7.481493974
ENSG00000136854	STXBP1	1.932722512	0.259806896	2.524286948	0.12381131	4.45700946	7.435667355	8.297207175	2	9.231457548	9.30367823	9.705819181	9.992781936
ENSG00000182263	FIGN	2.440084145	0.010021807	2.013139632	0.036087858	4.453223777	4.467311984	5.131603625	2	6.5612028	6.67228943	6.148830293	6.270645889
ENSG00000244486	SCARF2	1.349517772	0.231258748	3.103345825	0.002260394	4.452863597	6.138360429	8.276714665	9.083172337	9.551067273	9.65224116	11.89104907	10.47837298
ENSG00000081320	STK17B	2.244837065	0.000587551	2.206962456	0.000694887	4.451799521	5.532353208	6.951736442	7.398165132	9.057896526	8.98821297	9.07802367	8.887436006
ENSG00000154545	MAGED4	9.642540498	6.47E-11	-5.191151323	0.025401936	4.451389175	2	2	2	9.678668908	9.67331554	2	2
ENSG00000187243	MAGED4B	9.642540498	6.47E-11	-5.191151323	0.025401936	4.451389175	2	2	2	9.678668908	9.67331554	2	2
ENSG00000166311	SMPD1	2.048837934	0.134855166	2.397783063	0.070852767	4.446620997	4.815609471	9.540933215	9.099922433	10.79633594	10.8506773	11.10459281	11.23642639
ENSG00000165935	SMCO2	2.820338664	0.339862734	1.623569323	0.594804601	4.443907988	2	2	2	3.663521627	3.28037825	3.12825509	2.466008509
ENSG00000104856	RELB	2.264191814	0.202526124	2.179698307	0.213719558	4.443890121	8.418903792	6.311476754	2	9.359407363	9.39837151	9.31719306	9.272532009
ENSG00000166816	LDHD	-1.315777443	0.684355643	5.758544897	0.019926216	4.442767454	2	2	2	2	2.2680111	5.832754931	5.937276556
ENSG00000151726	ACSL1	1.97049231	0.0190625	2.465024571	0.002440403	4.435516881	5.532353208	7.315436877	5.39244166	8.258639605	8.36731141	8.892160003	8.708487604
ENSG00000002834	LASP1	1.226145656	0.030568134	3.208995352	4.31E-10	4.435141008	10.89224845	11.12072186	9.819251342	11.90383364	11.9697428	13.9522767	13.8847597
ENSG00000168411	RFWD3	2.098874821	0.14406389	2.334590084	0.094670586	4.433464905	3.327711234	7.503546079	4.874587708	8.166267464	8.34936267	8.50665722	8.480396055
ENSG00000162434	JAK1	2.440051864	0.129911918	1.99253965	0.21842725	4.432591514	4.00722531	10.16572116	8.380957451	11.37674805	11.4162868	10.96091792	10.93880421
ENSG00000188290	HES4	2.172889715	0.22173333	2.258002404	0.194278246	4.430892118	8.19931886	5.838937808	2	9.025111392	9.01464729	9.1990699	9.002968638
ENSG00000170370	EMX2	-0.718700302	0.861382316	5.146520932	0.112361418	4.42782063	2	2	2	2.3892346	2	5.963846589	4.147809227
ENSG00000159899	NPR2	2.534080135	0.103857132	1.893483742	0.230468038	4.427563876	6.468621203	7.701025996	2	9.077877436	9.20532695	8.528003102	8.487906181
ENSG00000180089	TMEM86B	2.5371604	0.142232651	1.890004159	0.279023454	4.427164558	2	3.686303414	2	4.574143677	4.60967607	4.155805465	3.987514168
ENSG00000204442	FAM155A	5.005142756	0.002167981	-0.57851108	0.773343718	4.426631675	2	2	5.59507254	8.873322599	8.82765235	3.981090143	3.496556178
ENSG00000048649	RSF1	2.065181641	0.041980392	2.361070593	0.017309633	4.426252234	8.674867122	8.414402626	5.59507254	10.10534684	10.1089823	10.45430433	10.34788887
ENSG00000213996	TM6SF2	2.834731596	0.156125244	1.58471474	0.445656681	4.419446335	2	3.076665209	2	3.95996233	4.37434574	3.411599105	3.209956562
ENSG00000101255	TRIB3	1.532807668	0.387432577	2.884498959	0.077151749	4.417306626	2	7.467835385	6.629488387	7.917958914	8.15847021	10.05770853	8.067092663
ENSG00000135018	UBQLN1	2.200887476	4.83E-07	2.21564729	3.66E-07	4.416534765	7.940205782	8.834582582	8.922968322	10.8110007	10.829793	10.90806718	10.75834164
ENSG00000196547	MAN2A2	1.641634485	0.177844856	2.774849816	0.014545829	4.416484301	7.709880939	7.701025996	4.058998049	8.799033614	8.79487126	10.20975726	9.564939053
ENSG00000118960	HS1BP3	1.820340569	0.456251283	2.595194584	0.260775363	4.415535153	2	7.315436877	2	7.559034656	7.56191311	8.413960373	8.224954107
ENSG00000162601	MYSM1	2.597769835	0.043022105	1.817650377	0.166932988	4.415420211	3.327711234	6.389896633	7.734844356	9.226493643	9.21824109	8.652274805	8.212645079
ENSG00000137672	TRPC6	0.515189383	0.793804216	3.899750903	0.01477161	4.414940287	2	5.303523747	2	4.54891529	4.37434574	7.067324363	7.97389845
ENSG00000189001	SBSN	3.621642239	0.148210562	0.792855281	0.781073497	4.41449752	2	2	2	4.237680128	3.82856379	2.62493832	2.421067459
ENSG00000162591	MEGF6	-1.924240815	0.438437558	6.336052598	0.00313113	4.411811783	2	7.669933291	2	4.670864177	4.16043394	11.93560775	12.7588774
ENSG00000108510	MED13	2.466552612	0.175086607	1.944287291	0.287569328	4.410839903	2	8.791896851	8.825624954	10.68241243	10.6813023	10.28424887	10.02645802
ENSG00000120337	TNFSF18	5.716547896	0.052953075	-1.305878904	0.706382819	4.410668992	2	2	2	5.720439317	5.96314905	2.267001569	2
ENSG00000130584	ZBTB46	-0.47572303	0.812947514	4.886200295	0.001927913	4.410477265	2	5.72254323	2	3.881293667	4.16043394	8.96165712	9.027536965
ENSG00000181555	SETD2	2.163160337	0.050378597	2.246129777	0.038950505	4.409290114	5.095959458	8.255926468	8.608698767	10.01150489	10.1317374	10.13242893	10.17828285
ENSG00000143199	ADCY10	2.899992654	0.289703301	1.508921226	0.597377354	4.40891388	2	2	2	3.663521627	3.39680241	2.858551861	2.70970617
ENSG00000145244	CORIN	1.008937075	0.456962569	3.399404944	0.003252545	4.408342019	2	3.686303414	4.058998049	4.387512679	4.02226117	5.960710379	6.62538071
ENSG00000107290	SETX	2.59142962	6.32E-13	1.816616787	7.39E-07	4.408046407	8.493334308	8.337339476	9.06622548	11.23069904	11.2496932	10.41445658	10.51792633
ENSG00000156711	MAPK13	3.312713252	4.13E-09	1.092978467	0.080745253	4.405691719	6.73718411	5.595927442	6.203069917	9.473580277	9.47569407	7.575682301	6.91798856
ENSG00000170191	NANP	1.922472875	0.387605511	2.482485845	0.243517965	4.40495872	2	6.602424674	2	6.984595707	6.92586261	7.540404604	7.459438299
ENSG00000011347	SYT7	3.420206639	0.079639272	0.984038486	0.650307428	4.404245125	5.70932728	2	2	7.440576128	7.43830714	5.610655473	4.445805334
ENSG00000131381	RBSN	2.163075179	2.92E-05	2.240070326	1.30E-05	4.403145506	7.750913381	7.60566235	6.43197674	9.474417988	9.55457603	9.609733245	9.573306996
ENSG00000153201	RANBP2	2.5505255	4.10E-17	1.85257965	1.51E-09	4.40310515	8.564112515	9.030900487	8.845626171	11.34067986	11.4004669	10.80385399	10.53186455
ENSG00000155903	RASA2	2.670129547	0.053560884	1.732223801	0.225078882	4.402353348	6.009025997	7.002537973	2	8.601543953	8.68895519	7.79843893	7.63942137
ENSG00000119596	YLPM1	1.801475253	0.070841918	2.600838521	0.005996167	4.402313775	8.313285031	8.147241016	5.39244166	9.531881013	9.53088329	10.27780546	10.37538871
ENSG00000145715	RASA1	2.998669115	0.066585047	1.402598248	0.42122258	4.401267363	4.467311984	8.255926468	3.369317972	9.764048416	9.79963996	8.247007147	8.14947356
ENSG00000222038	POTEJ	9.590269497	1.05E-10	-5.191151262	0.025794745	4.399118235	2	2	2	9.55265467	9.6919516	2	2
ENSG00000102786	INTS6	2.564919756	0.025631643	1.833622892	0.120387966	4.398542649	3.327711234	7.051611315	6.958202216	8.967964567	9.0169232	8.498029643	7.996092644
ENSG00000069966	GNB5	1.814598426	5.20E-05	2.582362478	2.53E-09	4.396960904	6.009025997	6.04685525	6.80318718	8.04293816	8.11238697	8.850830124	8.823038243
ENSG00000243147	MRPL33	1.908069132	0.216950087	2.488601613	0.092611393	4.396670745	7.667647223	3.076665209	5.156626401	8.343618883	8.06917614	8.59173005	8.95894086
ENSG00000143442	POGZ	2.258890333	0.127775345	2.136271071	0.14497505	4.395161403	3.327711234	8.522788974	6.882775709	9.587142668	9.62638965	9.702067969	9.22919499
ENSG00000169018	FEM1B	1.356566964	0.009715897	3.037703048	3.69E-10	4.394270012	7.435667355	8.655694736	8.35328246	9.60637697	9.56472327	11.36982317	11.14470525
ENSG00000141446	ESCO1	2.484318086	0.096071046	1.908865335	0.205129503	4.393183421	5.095959458	6.899080544	2	8.170409056	8.09094333	7.872065621	7.176431616
ENSG00000168890	TMEM150A	1.652156203	0.480384146	2.738377795	0.209907098	4.390533998	2	6.899080544	2	6.912249745	7.05550676	7.938932725	8.148612304
ENSG00000019144	PHLDB1	2.423349925	0.015831771	1.965973334	0.053853036	4.389323259	6.73718411	8.655694736	9.868959282	11.2965993	11.3514882	10.78724204	10.9440257
ENSG00000101187	SLCO4A1	3.521307695	0.071965527	0.86679076	0.695304679	4.388098455	3.327711234	2	2	4.623314311	4.85329164	3.172301926	2.632958241
ENSG00000198815	FOXJ3	2.367358191	0.032621666	2.019992523	0.070331611	4.387350713	5.866944937	8.147241016	5.156626401	9.30110636	9.34230452	9.083440537	8.861915706
ENSG00000185104	FAF1	1.811294217	0.001702654	2.574440111	3.35E-06	4.385734328	5.532353208	5.595927442	6.718950348	7.68596357	7.8720741	8.476781207	8.589773203
ENSG00000239474	KLHL41	1.274446705	0.688362385	3.110738614	0.272520906	4.385185319	2	2	2	2.695456494	2.68924543	4.144793515	2.981406154
ENSG00000173404	INSM1	1.282428516	0.837050918	3.101871805	0.584589142	4.384300321	2	2	2	3.162721474	2	4.425202323	2
ENSG00000063854	HAGH	2.266267375	0.157563342	2.116002352	0.181344746	4.382269727	7.790810999	6.389896633	2	8.918344554	8.89972012	8.699603841	8.818171767
ENSG00000174840	PDE12	1.873960932	0.113862445	2.507545811	0.026981937	4.381506743	4.00722531	7.638155693	6.073378139	8.461536372	8.34393422	9.018941717	9.043840859
ENSG00000001084	GCLC	3.361800744	0.039063396	1.017548336	0.574400364	4.379349081	2	6.666836837	8.236946975	10.39094584	10.4196875	8.209185391	7.934467448
ENSG00000196422	PPP1R26	2.032134984	0.232496722	2.34348691	0.155092367	4.375621894	2	7.701025996	5.59507254	8.349110081	8.5130942	8.920281122	8.537437492
ENSG00000153922	CHD1	2.654369115	0.102115752	1.720119292	0.301852744	4.374488407	2	7.701025996	5.930865648	9.142549916	9.09655277	8.456328963	7.875307424
ENSG00000123358	NR4A1	1.96337904	0.03398501	2.407252194	0.00729012	4.370631235	4.815609471	7.051611315	5.39244166	7.883054517	8.12510181	8.709446788	8.126037235
ENSG00000196391	ZNF774	0.859970152	0.615680633	3.507727115	0.016000913	4.367697267	2	4.442710129	2	4.299492648	3.65239003	6.375345632	6.267475858
ENSG00000196890	HIST3H2BB	3.62007234	0.148005951	0.746366936	0.794186359	4.366439275	2	2	2	4.034562184	4.05806467	2.384001718	2.632958241
ENSG00000184916	JAG2	0.621609719	0.644603834	3.742013931	0.000583068	4.363623651	3.327711234	4.11352228	2	4.034562184	3.74316441	6.726875343	6.514766253
ENSG00000108819	PPP1R9B	1.678881628	0.088518928	2.683944692	0.00361429	4.36282632	9.435834703	8.433041974	6.534109959	10.2141657	10.2394476	11.1725846	11.28426283
ENSG00000131016	AKAP12	0.664237014	0.343511582	3.696623728	2.46E-10	4.360860742	9.940323746	8.655694736	8.631919287	9.84659862	9.89984536	12.73924589	13.04543027
ENSG00000070476	ZXDC	1.801786951	0.223962299	2.558426795	0.068912293	4.360213746	3.327711234	7.538394143	4.874587708	7.961629787	8.0314011	8.995380861	8.440137439
ENSG00000117408	IPO13	2.023644768	0.341002462	2.335900207	0.256784608	4.359544976	4.00722531	8.376385542	2	8.937912013	8.80016655	9.221844492	9.137974605
ENSG00000141568	FOXK2	1.6479922	0.262397112	2.70842538	0.048844314	4.356417581	3.327711234	8.101365653	8.266922072	9.244284335	9.26686067	10.45137457	10.15574229
ENSG00000010072	SPRTN	2.010406268	0.364510704	2.34560461	0.273901976	4.356010878	2	6.602424674	2	6.946463121	7.12888795	7.483632518	7.239267729
ENSG00000089057	SLC23A2	1.792501542	0.490463824	2.561648318	0.297700044	4.354149861	2	7.928237109	2	8.136938566	8.14604733	8.71690136	9.066361132
ENSG00000058091	CDK14	2.952484641	9.83E-05	1.401613647	0.086347448	4.354098288	5.095959458	7.35507212	7.029880454	9.641111693	9.74233706	7.98287976	8.309067496
ENSG00000176903	PNMA1	1.628799547	2.31E-06	2.724263008	2.47E-16	4.353062555	7.975256505	7.35507212	7.777116083	9.302993856	9.34230452	10.46111737	10.36411774
ENSG00000222009	BTBD19	1.873716398	0.241482575	2.479335739	0.104869869	4.353052137	2	4.93638933	7.029880454	7.584088227	7.55253065	7.855275386	8.417435289
ENSG00000254122	PCDHGB7	3.606487754	1.99E-06	0.746343465	0.399833462	4.352831218	4.815609471	7.051611315	7.163363831	10.19039992	10.2554384	7.38764416	7.398436576
ENSG00000175573	C11orf68	1.710714624	0.184030119	2.641406072	0.029385896	4.352120696	4.00722531	7.701025996	8.236946975	9.115954467	9.16690126	10.05752505	10.07735401
ENSG00000099904	ZDHHC8	1.843113001	0.090381529	2.508014996	0.015805954	4.351127997	7.904282243	7.572420273	4.523675391	9.010169232	9.10406516	9.71701465	9.720533286
ENSG00000136193	SCRN1	1.863457192	0.044940119	2.487633011	0.005240878	4.351090203	8.855032352	10.3138733	7.734844356	11.18537258	11.2225883	11.82580793	11.82901558
ENSG00000196083	IL1RAP	2.715530384	2.09E-08	1.634636166	0.001191054	4.350166549	5.330594134	6.140560021	5.59507254	8.439397922	8.3220138	7.237652294	7.398436576
ENSG00000185046	ANKS1B	1.153728358	0.725192089	3.196326471	0.271421671	4.350054828	2	2	2	2.947924294	2.2680111	3.584558147	3.875378318
ENSG00000060491	OGFR	2.947678434	1.44E-12	1.402311585	0.001432681	4.349990019	8.257427002	7.790474954	7.225742779	10.70756787	10.7810819	9.174577117	9.236508834
ENSG00000167447	SMG8	2.297016138	0.177474856	2.051667378	0.225372001	4.348683516	2	7.790474954	5.59507254	8.788294101	8.75042728	8.571313099	8.480396055
ENSG00000034152	MAP2K3	1.76890476	0.004683603	2.579484739	1.65E-05	4.348389498	8.816894989	7.701025996	7.896971941	9.927741409	10.0227123	10.25109847	11.1725107
ENSG00000197622	CDC42SE1	2.364962281	1.27E-10	1.983314407	8.68E-08	4.348276688	8.009475818	7.669933291	8.460932916	10.40111447	10.4384862	10.04443806	10.0346391
ENSG00000110888	CAPRIN2	2.217955595	0.084982806	2.127203552	0.094541013	4.345159147	3.327711234	6.78762695	7.734844356	8.941551548	9.00091592	8.970259716	8.786963584
ENSG00000143367	TUFT1	0.983915534	0.248121056	3.361035634	4.77E-06	4.344951168	4.00722531	5.595927442	6.43197674	6.573765157	6.56439512	8.802599622	8.984263871
ENSG00000165521	EML5	4.340921089	0.013143309	0.00378445	0.989969048	4.344705539	2	3.076665209	2	5.210797711	5.6826301	2.68698273	2.466008509
ENSG00000180423	HARBI1	2.059256814	0.21101766	2.284441371	0.152011997	4.343698185	2	4.710564154	2	5.161714397	5.33407135	5.684753614	5.177806074
ENSG00000104218	CSPP1	2.18380629	0.319942561	2.159163129	0.317458054	4.342969419	2	6.602424674	2	7.277360022	7.13728069	7.223329552	7.145684098
ENSG00000111981	ULBP1	0.803523706	0.423328228	3.538783967	2.39E-05	4.342307673	4.467311984	5.457120953	4.058998049	5.363304456	5.70538658	8.618842453	7.51663051
ENSG00000101986	ABCD1	2.600146421	0.000203189	1.74097549	0.017043752	4.341121911	5.532353208	6.228547981	7.451345288	9.161399541	9.16690126	8.351275892	8.273956186
ENSG00000065357	DGKA	1.390903188	0.492446389	2.947229176	0.110137106	4.338132364	4.467311984	8.029695761	2	7.805288479	8.07574085	9.45312092	9.521357751
ENSG00000022567	SLC45A4	1.601157292	0.120909818	2.736424961	0.004198848	4.337582253	2	4.710564154	5.156626401	5.818703821	5.93454154	7.180836177	6.695462882
ENSG00000074590	NUAK1	1.693170838	0.335781482	2.644368944	0.109372081	4.337539782	4.00722531	4.11352228	8.460932916	8.689305661	8.63367258	9.853683372	9.306905794
ENSG00000128165	ADM2	1.028291973	0.439229935	3.306846439	0.004137928	4.335138412	4.00722531	7.002537973	5.39244166	6.927012081	6.99214046	9.831964324	8.054273543
ENSG00000163681	SLMAP	2.175966264	0.146700672	2.155441676	0.143504721	4.33140794	2	6.899080544	8.585098385	9.621581886	9.50760441	9.736400072	9.324934421
ENSG00000162714	ZNF496	1.522082097	0.232251697	2.809305831	0.017180311	4.331387928	3.327711234	7.431218269	6.43197674	7.915493556	8.04037874	9.278673449	9.22674878
ENSG00000175352	NRIP3	2.136313201	0.013800383	2.194484868	0.010690228	4.330798069	4.467311984	5.838937808	7.029880454	8.122047456	8.25418043	8.327733606	8.161477479
ENSG00000186197	EDARADD	7.904218508	5.69E-07	-3.573834001	0.05519677	4.330384507	2	4.11352228	2	10.23853288	10.2291785	2.267001569	2
ENSG00000111670	GNPTAB	1.336559917	0.001086736	2.992904793	4.99E-15	4.32946471	7.904282243	7.274681896	8.110401118	9.101928616	9.12109086	10.82211411	10.6966318
ENSG00000119729	RHOQ	2.525954475	0.002821664	1.803364532	0.038931255	4.329319007	5.70932728	8.191702463	8.007629717	10.17037776	10.1406356	9.371930177	9.49788026

ENSG00000196776	CD47	2.916737894	0.01213142	1.407027397	0.258826994	4.323765291	9.143983763	7.847167909	5.156626401	11.00000655	11.026728	9.623185015	9.384672535
ENSG00000198205	ZXDA	2.548522148	0.167369545	1.775070364	0.345152391	4.323592511	2	5.595927442	2	6.483440992	6.64900057	5.899789217	5.772630506
ENSG00000073737	DHRS9	2.496810989	0.523459664	1.826619643	0.642308336	4.323430633	2	2	2	3.289987342	3.28037825	3.465698119	2.059860178
ENSG00000197879	MYO1C	1.068694035	0.094901214	3.253641074	8.27E-09	4.322335109	9.015175615	10.32389548	10.44859742	11.09075629	11.1562579	13.19651665	13.4092619
ENSG00000126775	ATG14	2.558481994	0.005070133	1.763441856	0.062126304	4.32192385	5.330594134	7.315436877	5.156626401	8.887228971	8.74494589	8.243785424	7.783974227
ENSG00000085491	SLC25A24	2.449142445	1.72E-10	1.86956576	1.54E-06	4.318708204	7.031698319	6.666836837	6.322058544	9.166592134	9.04395769	8.569255422	8.494021883
ENSG00000129646	QRICH2	2.31980206	0.174904784	1.995742366	0.241998457	4.315544427	3.327711234	2	5.39244166	6.139181293	6.29861567	6.550361701	4.757365452
ENSG00000099204	ABLIM1	0.972236936	0.262982679	3.343160596	8.39E-06	4.315397532	4.467311984	6.844429755	6.43197674	7.210554257	7.12888795	9.390986528	9.617238105
ENSG00000108389	MTMR4	0.962298544	0.698178574	3.352699953	0.123013154	4.314998497	3.327711234	8.717821467	2	8.14116509	8.11663771	10.26324148	10.70778414
ENSG00000157617	C2CD2	1.212672222	0.006321713	3.101483837	2.74E-14	4.314156059	6.89194353	7.73146271	7.398165132	8.652768197	8.51631523	10.6126694	10.29807728
ENSG00000103111	MON1B	1.675898949	0.530264818	2.635524314	0.292216612	4.311423262	2	8.147241016	2	8.180711314	8.30535212	9.048785116	9.327219816
ENSG00000165886	UBTD1	2.057135241	1.06E-07	2.253309153	3.81E-09	4.310444394	6.816637977	6.844429755	6.203069917	8.697938662	8.65720817	8.919473684	8.823038243
ENSG00000131095	GFAP	1.111715244	0.685365166	3.197329005	0.19011257	4.309044248	2	2	6.073378139	6.476767609	3.15372745	7.793159202	7.392628644
ENSG00000122299	ZC3H7A	1.989402518	0.063965155	2.31923775	0.026467216	4.308640268	5.532353208	8.213429689	5.930865648	9.044420506	9.04841459	9.259020976	9.480531399
ENSG00000132646	PCNA	1.940153717	0.272669235	2.364929723	0.164528915	4.30508344	4.467311984	7.35507212	2	7.783822874	7.95986274	8.415679223	8.158913605
ENSG00000115977	AAK1	1.909514811	0.000106004	2.394452083	6.41E-07	4.303966894	8.716893051	7.761270542	7.691296561	10.02357442	10.0306182	10.58734728	10.42921874
ENSG00000049883	PTCD2	1.752834916	0.28829652	2.550535788	0.101181698	4.303370705	3.327711234	6.140560021	2	6.535745081	6.39974839	7.443755487	7.000495137
ENSG00000071054	MAP4K4	3.08411984	6.71E-10	1.216323733	0.025387429	4.300443572	9.473095744	10.64891478	9.757248847	13.10109474	13.1642759	11.21433092	11.31769121
ENSG00000163220	S100A9	0.497864743	0.881014624	3.80176741	0.152741385	4.299632153	2	2	2	2.498691189	2.38539787	4.73522696	3.287118176
ENSG00000156860	FBRS	2.000298597	0.105778838	2.296844324	0.055557322	4.297142921	7.579279349	9.443177293	5.59507254	10.28080824	10.2731592	10.54962008	10.59534423
ENSG00000167323	STIM1	1.632227523	0.269395698	2.663305225	0.053894075	4.295532748	7.159102577	8.451443573	3.369317972	9.060130342	8.95060997	10.10960242	9.949426738
ENSG00000178878	APOLD1	0.036865314	0.977083699	4.258551747	6.69E-06	4.295417061	6.138360429	4.442710129	6.073378139	5.697663076	5.7929843	8.539610093	10.54059012
ENSG00000138496	PARP9	3.824040474	3.99E-05	0.46998313	0.681431908	4.294023605	8.19931886	5.946638857	8.978369997	11.97066735	11.9540913	8.466314923	8.761839632
ENSG00000128641	MYO1B	2.080657803	3.30E-09	2.212195986	2.45E-10	4.292853789	10.09970294	9.770792087	9.61300511	11.92880215	11.9068589	12.28418861	11.76849593
ENSG00000183246	RIMBP3C	5.695499803	0.00189134	-1.403130722	0.536670773	4.292369082	2	2	2	5.900763176	5.74985018	2.094598369	2.172608095
ENSG00000166529	ZSCAN21	2.066959841	0.260797132	2.222389	0.21477409	4.289348842	2	5.303523747	2	5.602782483	6.01871653	6.231826305	5.651404445
ENSG00000250312	ZNF718	2.505865235	0.052932111	1.783159471	0.177027586	4.289024706	2	4.710564154	3.369317972	6.052081244	5.85535975	5.673303969	4.77533149
ENSG00000101336	HCK	0.269719039	0.937710599	4.018566073	0.146799193	4.288285111	2	2	2	2.141737246	2.59489879	4.834511823	3.640730416
ENSG00000281742		2.154036419	0.163051497	2.133941819	0.159212693	4.287978237	3.327711234	7.60566235	4.523675391	8.378048624	8.34212018	7.924517766	8.662770717
ENSG00000135317	SNX14	2.81673845	0.028112563	1.467200721	0.280137585	4.283939171	5.095959458	8.376385542	5.156626401	9.897454779	9.82063646	8.621823729	8.409548182
ENSG00000127951	FGL2	3.486121733	0.001087557	0.793803328	0.522353469	4.279925061	2	3.686303414	4.058998049	6.470063213	6.35727082	3.917810786	4.175132639
ENSG00000170684	ZNF296	2.750171206	0.162619057	1.529601617	0.455138882	4.279772823	3.327711234	2	2	4.139692846	4.0930011	3.27687201	3.287118176
ENSG00000188672	RHCE	1.937762106	0.385331055	2.339180744	0.272104511	4.27694285	2	3.076665209	2	3.615725078	3.45167086	4.166733998	3.33635788
ENSG00000136848	DAB2IP	1.002022132	0.483401635	3.273169572	0.00856295	4.275191704	8.631580145	7.638155693	4.058998049	8.633396609	8.69038176	11.07062965	10.75622296
ENSG00000151422	FER	1.725001812	6.79E-05	2.546927946	1.07E-09	4.271929758	6.89194353	7.145017454	6.534109959	8.550024073	8.61576544	9.159610958	9.603790255
ENSG00000133687	TMTC1	1.629651486	0.237913826	2.642168315	0.040936541	4.271819801	6.366709671	5.838937808	2	7.144720234	7.12888795	7.47706242	8.575745325
ENSG00000233493	TMEM238	2.594781454	0.366381671	1.676078547	0.567684646	4.270860002	2	2	2	3.162721474	3.50452879	2.962551659	2.746601469
ENSG00000166266	CUL5	2.306410389	0.076743239	1.964182416	0.131858917	4.270592805	7.53297911	7.790474954	3.369317972	9.434510081	9.36034073	9.232935896	8.858759519
ENSG00000139364	TMEM132B	4.761199703	2.65E-12	-0.492299789	0.564054374	4.268899914	5.095959458	4.11352228	3.369317972	8.792330793	8.83798691	3.943456545	4.002845082
ENSG00000128872	TMOD2	2.499068622	0.005710292	1.769440179	0.057699308	4.268508802	4.815609471	6.04685525	3.369317972	7.587189552	7.48837134	6.913621294	6.739599323
ENSG00000197070	ARRDC1	1.462176587	0.461825521	2.805644991	0.122968358	4.267821578	2	7.099070191	3.369317972	7.075737784	7.05992818	8.505043475	8.25407423
ENSG00000181027	FKRP	2.043181932	0.016750741	2.223989605	0.008135238	4.267171537	5.532353208	7.467835385	5.59507254	8.47833774	8.56378716	8.751793877	8.647610514
ENSG00000104826	LHB	2.005881962	0.548198478	2.260740213	0.484174515	4.266622174	2	2	2	2.3892346	3.45167086	3.27687201	3.012034821
ENSG00000135312	HTR1B	1.499055968	0.671487898	2.767283975	0.394823109	4.266339943	2	2	2	2.947924294	2.59489879	4.029788235	2.466008509
ENSG00000128564	VGF	2.973845441	0.309469647	1.292362137	0.67791597	4.266207578	2	2	2	3.462030052	3.69849104	2.962551659	2.374681325
ENSG00000172667	ZMAT3	0.682097526	0.212680082	3.583487469	8.65E-15	4.265584995	7.624139768	8.276714465	8.699419242	9.010169232	8.86476809	12.03680136	11.58959617
ENSG00000100065	CARD10	-0.182483795	0.895265695	4.444174254	1.30E-05	4.261690459	2	5.595927442	5.39244166	4.670864177	4.98931816	8.729394257	9.643763491
ENSG00000235034	C19orf81	1.486820593	0.441376957	2.773712462	0.114946876	4.260533054	2	3.686303414	2	3.754611435	3.7864959	5.085488617	4.400220394
ENSG00000184271	POU6F1	1.705646082	0.050999384	2.553652235	0.001937731	4.259298317	4.815609471	5.838937808	4.058998049	6.628980152	6.80989959	7.909144085	7.05850228
ENSG00000109466	KLHL2	2.486742583	0.181399395	1.772080648	0.348797592	4.25882323	3.327711234	7.002537973	2	7.93753157	8.01327646	7.514972902	6.988993457
ENSG00000166004	CEP295	2.271068987	0.318138811	1.980521362	0.381442321	4.251590349	2	6.844429755	2	7.584088227	7.48179565	7.552954291	6.866539459
ENSG00000132740	IGHMBP2	1.794369412	0.215468947	2.455804753	0.075024979	4.250174165	2	5.72254323	6.882775709	7.510853288	7.64959655	8.368535975	8.081604954
ENSG00000070214	SLC44A1	3.623737086	0.001296825	0.624389161	0.640695034	4.248126246	5.866944937	6.844429755	9.242516611	11.62402619	11.6150937	8.82044307	8.417435289
ENSG00000075711	DLG1	1.480835121	0.001328581	2.7667544	2.08E-10	4.247589521	7.384433967	7.901717006	6.882775709	9.021676912	8.82245717	10.16787644	10.24002969
ENSG00000130433	CACNG6	5.236575891	0.007634516	-0.989333288	0.683334854	4.247242603	2	2	2	5.44762523	5.36298494	2.225793514	2.117335275
ENSG00000173702	MUC13	4.690417399	0.213876745	-0.443285963	0.921262078	4.247131435	2	2	2	5.076005082	4.74762503	2.457042231	2
ENSG00000197363	ZNF517	1.300309689	0.498453223	2.946110092	0.089458136	4.246419781	2	5.457120953	2	5.178261742	5.36298494	7.000176314	6.600402875
ENSG00000169760	NLGN1	2.596386719	0.08193596	1.649747011	0.282680363	4.24613373	2	3.686303414	3.369317972	5.22679461	5.13053105	3.782262616	4.793076545
ENSG00000226763	SRRM5	2.442900379	0.403894818	1.798474787	0.540942209	4.241375166	2	2	2	3.09461492	3.39680241	3.035909414	2.782576693
ENSG00000164631	ZNF12	1.793735085	0.277947155	2.447348759	0.119536754	4.241083844	6.563806279	7.790474954	2	8.534041678	8.46722793	9.404322061	8.838074258
ENSG00000129493	HEATR5A	1.799658024	0.001434462	2.441052085	7.53E-06	4.240710108	6.366709671	7.669933291	6.718950348	8.822909147	8.81201036	9.504905576	9.401760658
ENSG00000124839	RAB17	1.727686389	0.415598099	2.512579706	0.205097324	4.240266095	2	3.076665209	2	3.406930997	3.39680241	3.981090143	3.8919413
ENSG00000174010	KLHL15	2.461268624	0.063911628	1.777335398	0.189290362	4.238604022	2	4.93638933	6.43197674	7.759286043	7.55253065	7.02890981	6.955898408
ENSG00000144040	SFXN5	2.914706941	0.007907751	1.321134151	0.264665124	4.235841092	6.009025997	4.93638933	2	7.818544084	7.7866208	6.275391383	6.245088435
ENSG00000116690	PRG4	1.012885833	0.737721421	3.222218571	0.221458956	4.235104404	2	2	2	2.695456494	2.49394783	3.584558147	3.908316285
ENSG00000146410	MTFR2	3.471550595	0.167941338	0.763141351	0.789642976	4.234691946	2	2	2	4.034562184	3.82856379	2.526562374	2.509591799
ENSG00000018408	WWTR1	2.061608369	0.004263331	2.172585968	0.002338722	4.234194336	8.042902253	7.701025996	9.452720984	10.69144695	10.6189937	10.73805965	10.79416656
ENSG00000173542	MOB1B	2.319263892	0.01380515	1.914299681	0.045052759	4.233563573	4.815609471	7.274681896	7.734844356	9.408447353	9.24566964	9.032447693	8.813832186
ENSG00000164543	STK17A	1.960271648	0.000131113	2.272614269	6.38E-06	4.232885917	7.031698319	7.002537973	8.076950877	9.384581425	9.38785872	9.719104163	9.674305191
ENSG00000244414	CFHR1	7.995311529	2.68E-07	-3.763576298	0.118337719	4.231735231	2	2	2	8.013270196	8.07355593	2.048074405	2
ENSG00000160746	ANO10	2.233570972	0.042924135	1.997998317	0.070335607	4.231569289	7.031698319	8.57407404	5.156626401	9.702459798	9.75874862	9.416105065	9.572665028
ENSG00000076924	XAB2	1.999499984	0.038085116	2.231987533	0.018144183	4.231487517	6.138360429	7.60566235	4.874587708	8.58612524	8.6021877	8.934738262	8.704976832
ENSG00000269955	C7orf55-LUC	9.422318555	2.88E-10	-5.191151298	0.026199962	4.231167256	2	2	2	9.436230969	9.47734778	2	2
ENSG00000165105	RASEF	4.485977378	0.067479075	-0.256198508	0.933434132	4.22977887	2	2	2	5.040242897	4.37434574	2.42098416	2.117335275
ENSG00000161791	FMNL3	2.364711015	8.27E-06	1.864811103	0.000554619	4.229522118	7.27616306	7.572420273	8.486635645	10.1842688	10.2409086	9.883799376	9.523684623
ENSG00000196821	C6orf106	2.157694542	0.003538216	2.071418461	0.005132356	4.229113003	7.750913381	8.862353853	9.809100877	11.14621855	11.2257892	11.04956604	11.14955624
ENSG00000064607	SUGP2	1.900077013	0.103634704	2.326859029	0.038665093	4.226936042	5.095959458	8.57407404	8.699419242	9.968745466	10.0306182	10.4078509	10.44263169
ENSG00000203485	INF2	1.579431484	0.001761901	2.644721783	3.51E-08	4.224153267	9.423197387	8.234834549	8.960138144	10.44877988	10.5852675	11.52052886	11.64071835
ENSG00000184178	SCFD2	2.169803856	0.364510704	2.051259341	0.384577928	4.221063197	2	7.274681896	2	7.910550164	7.81305436	7.50961773	7.949380652
ENSG00000006831	ADIPOR2	1.907191553	0.29492176	2.312306727	0.186045154	4.219498281	2	8.623598428	8.408111533	9.802582299	9.86489641	10.14871212	10.32138402
ENSG00000154040	CABYR	1.164517985	0.517016221	3.054556038	0.054985297	4.219074023	2	5.131603625	2	4.907595604	4.811949	6.67615119	6.514766253
ENSG00000165804	ZNF219	1.514826708	0.026715109	2.702664407	2.18E-05	4.217491115	6.138360429	6.951736442	5.156626401	7.802622679	7.70992146	9.002260838	8.853483814
ENSG00000251192	ZNF674	2.424508787	0.033910747	1.792861367	0.123992425	4.217370154	2	4.11352228	5.39244166	6.503277904	6.60731796	6.213498222	5.646532192
ENSG00000132694	ARHGEF11	2.428987444	0.095066141	1.787805708	0.225799741	4.216793152	6.009025997	4.11352228	8.65477198	9.723047898	9.73475225	8.991160222	9.185355845
ENSG00000136874	STX17	2.755266564	0.003631933	1.461400591	0.146527599	4.216667155	4.815609471	7.315436877	5.39244166	8.95000826	8.99976571	7.811554331	7.574209638
ENSG00000162607	USP1	1.723819894	0.488639525	2.492381404	0.289726676	4.216201298	2	7.393647515	2	7.568480804	7.51759662	8.60986148	7.902127837
ENSG00000160551	TAOK1	1.981734107	4.48E-07	2.2343267	8.49E-09	4.216060808	8.797441463	8.88960062	9.516493218	11.08856943	11.0615956	11.48303928	11.15267381
ENSG00000198887	SMC5	2.016401093	0.003842733	2.198327397	0.001374482	4.21472849	5.70932728	7.145017454	5.59507254	8.386974157	8.26953254	8.553208128	8.463873348
ENSG00000204060	FOXO6	0.86351266	0.790662721	3.350298923	0.228061414	4.213811583	2	2	2	2.784612485	2.2680111	3.851628042	3.841670744
ENSG00000135063	FAM189A2	-0.732281651	0.854305977	4.938402635	0.117842585	4.206120984	2	2	2	2	2.38539787	5.422868615	4.784231301
ENSG00000175048	ZDHHC14	2.258896471	0.313691383	1.946621284	0.382824967	4.205517755	2	6.728495673	2	7.444001439	7.36873214	7.349785676	6.804465095
ENSG00000198182	ZNF607	1.843347093	0.20811252	2.359476359	0.092298578	4.202823452	2	5.457120953	3.369317972	5.959382894	5.9725602	6.807643042	5.984375752
ENSG00000076984	MAP2K7	2.078348622	0.099215121	2.123745318	0.086209421	4.202093939	4.467311984	8.077869079	5.772716771	8.88597028	8.93143442	9.058352796	8.841275974
ENSG00000105722	ERF	1.921447173	0.005467646	2.280094957	0.000727777	4.20154213	7.624139768	6.04685525	7.646393253	9.131970496	9.17099461	9.203056613	9.759696604
ENSG00000182325	FBXL6	2.221894978	0.297505663	1.97543189	0.350123893	4.197326868	2	6.311476754	2	6.993973425	6.9210099	6.457740741	6.942047249
ENSG00000183718	TRIM52	2.450570516	0.046457783	1.74242721	0.165909543	4.192997726	4.467311984	5.946638857	2	7.230526165	7.18258783	6.57313027	6.471341372
ENSG00000143079	CTTNBP2NL	1.659356313	0.00410097	2.530733215	4.48E-06	4.190089528	8.75772933	7.572420273	7.552162999	9.748895993	9.68767229	10.60465476	10.56887086
ENSG00000077235	GTF3C1	1.947630487	5.71E-05	2.239260134	2.56E-06	4.186890621	8.107517749	8.968383114	7.97167868	10.3445078	10.385308	10.51703111	10.78255483
ENSG00000107438	PDLIM1	1.396113426	0.004683802	2.790360728	1.50E-09	4.186474154	7.904282243	8.702538795	7.777116083	9.558196845	9.60839132	10.76179094	11.1573911
ENSG00000197121	PGAP1	2.428428662	0.013540843	1.755817595	0.082334289	4.184246258	4.815609471	6.228547981	3.369317972	7.751013478	7.41433569	6.924930115	6.949978473
ENSG00000178764	ZHX2	1.592487124	0.301084135	2.591227043	0.071431314	4.183714167	6.009025997	7.189546432	2	7.725907304	7.6986238	8.443467705	8.913019964
ENSG00000104983	CCDC61	1.203030837	0.481130174	2.979531236	0.050033331	4.182562073	2	4.710564154	2	4.268917409	4.72553021	6.022174708	6.175763085
ENSG00000139977	NAA30	2.191281588	0.097979988	1.991240376	0.12941073	4.182521963	4.00722531	7.572420273	4.874587708	8.483340231	8.41813449	8.395497408	8.095972716
ENSG00000184575	XPOT	1.701279734	0.243498812	2.479192547	0.072675764	4.18047228	4.467311984	9.443177293	8.677268317	10.26055405	10.2330992	11.36137528	10.57961443
ENSG00000160714	UBE2Q1	2.314980905	0.031200591	1.865308766	0.08678054	4.180289671	7.485143553	8.57407404	5.156626401	9.930185944	9.93817677	9.26856367	9.676099363
ENSG00000153064	BANK1	0.661560295	0.669226282	3.518715193	0.005828245	4.180275488	4.467311984	3.076665209	2	3.663521627	4.22485516	6.013120342	6.81752904
ENSG00000092929	UNC13D	1.819414369	0.375778872	2.360815804	0.227601689	4.180230173	3.327711234	2	3.369317972	4.105493497	4.16043394	5.227950776	3.311948089

ENSG00000259112	NDUFC2-KCT	9.370376944	4.33E-10	-5.191151263	0.026510255	4.179225681	2	2	2	9.354865403	9.45402153	2	2
ENSG00000003393	ALS2	1.660283156	0.131737146	2.507894616	0.015985371	4.168177772	4.815609471	8.169643011	7.552162999	9.014783325	9.05507424	9.670022651	10.05936592
ENSG00000150637	CD226	2.495120064	0.437785917	1.669623792	0.608860386	4.164743856	2	2	2	3.09461492	3.45167086	3.215043717	2.374681325
ENSG00000067225	PKM	2.17050867	8.51E-07	1.993360956	6.72E-06	4.163869625	12.54538064	12.24604664	12.48269311	14.57461638	14.6246954	13.84967977	14.83181639
ENSG00000127325	BEST3	3.472129694	0.172072167	0.691075062	0.811558361	4.163204755	2	2	2	4.105493497	3.74316441	2.526562374	2.466008509
ENSG00000116663	FBXO6	3.338388334	1.47E-08	0.824667618	0.222125563	4.163055952	5.532353208	4.442710129	5.772716771	8.535647908	8.48213028	6.197600957	5.925257518
ENSG00000109756	RAPGEF2	2.217262731	0.000108426	1.945254781	0.000758118	4.162517512	7.031698319	6.464272742	7.896971941	9.435370781	9.41227044	9.072949511	9.230009473
ENSG00000197837	HIST4H4	1.144078058	0.731338839	3.015333907	0.309952764	4.159411965	2	2	2	2.270786764	2.94009902	3.584558147	3.640730416
ENSG00000156787	TBC1D31	3.480709218	0.001532124	0.67694343	0.59982426	4.157652648	4.815609471	5.946638857	2	8.300810597	8.34212018	5.812021848	5.402507939
ENSG00000265763	ZNF488	-0.723819587	0.800057969	4.876795264	0.02251104	4.152975676	2	2	2	2.270786764	2.14022021	5.014670875	5.150533729
ENSG00000099330	OCEL1	1.711589419	0.137305271	2.440488883	0.025468435	4.152078302	3.327711234	6.389896633	4.523675391	6.936770382	6.93551934	7.585896024	7.704159082
ENSG00000123609	NMI	3.49158149	0.020971449	0.656442448	0.709826973	4.148023938	2	4.710564154	7.098165388	9.178978633	9.18523054	6.329884754	6.479583725
ENSG00000021645	NRXN3	-0.366149336	0.535148173	4.51400768	2.25E-23	4.147858345	5.70932728	5.303523747	5.930865648	5.334065616	5.28958453	9.948950598	10.13414984
ENSG00000164112	TMEM155	6.969837767	0.00017384	-2.822347402	0.246641741	4.147490365	2	2	2	6.979883891	7.0991228	2.094598369	2
ENSG00000141040	ZNF287	1.666865963	0.253975538	2.479425666	0.071647452	4.146291629	3.327711234	5.595927442	2	5.910700032	5.92487824	6.510167449	6.847559257
ENSG00000171714	ANO5	1.151019437	0.756818484	2.995075656	0.368954964	4.146095092	2	2	2	2.784612485	2.49394783	4.251304078	2.374681325
ENSG00000177459	ERICH5	0.856943564	0.794276136	3.287313909	0.242584119	4.144257473	2	2	2	2.3892346	2.68924543	3.193831101	4.228274364
ENSG00000145860	RNF145	1.971264372	8.52E-08	2.171908112	2.50E-09	4.143172485	8.540903468	8.077869079	7.777116083	10.11558569	10.1285838	10.23220252	10.40670299
ENSG00000173575	CHD2	2.409241404	0.005150692	1.733037327	0.050915017	4.142278731	6.468621203	8.94259736	8.784770914	10.80867762	10.810899	10.23788114	10.0252855
ENSG00000175229	GAL3ST3	-2.311823937	0.458287513	6.452201368	0.002554195	4.140377432	2	2	2	2	2.14022021	5.747972572	7.051147246
ENSG00000214655	ZSWIM8	2.349034657	0.01537677	1.790612794	0.070728218	4.139647452	5.532353208	8.557180776	7.934808822	10.14902484	10.1143063	9.589063591	9.562354507
ENSG00000136147	PHF11	3.027548457	0.014409744	1.111032089	0.414485452	4.138580546	4.00722531	4.93638933	7.552162999	9.203437087	9.26686067	7.402744819	7.285445324
ENSG00000226174	TEX22	1.008440995	0.747712189	3.129527967	0.255172892	4.137968961	2	2	2	2.3892346	2.77779907	3.738983888	3.640730416
ENSG00000164080	RAD54L2	1.896561271	0.383636346	2.239670481	0.285451923	4.136231752	2	4.442710129	8.742726175	9.074566443	9.13791797	9.287461761	9.591484008
ENSG00000112697	TMEM30A	1.84002324	0.003801199	2.294696364	0.000205536	4.134719604	8.777722034	10.3486505	9.478569125	11.5358871	11.4945185	12.14662045	11.76751454
ENSG00000196358	NTNG2	2.806529512	0.011761813	1.32289909	0.268600882	4.129428602	4.815609471	5.838937808	2	7.549526251	7.61094772	6.320128742	5.957088417
ENSG00000072163	LIMS2	-0.112649604	0.944373487	4.241975349	0.000620166	4.129325745	8.107517749	6.899080544	3.369317972	6.998639523	6.94032357	11.46703437	11.06489955
ENSG00000170871	KIAA0232	1.4816217	0.011067153	2.645215264	1.29E-06	4.126836964	7.384433967	6.602424674	8.042706615	8.89725916	8.91200057	10.04351195	10.07938913
ENSG00000131386	GALNT15	2.600697687	0.046904744	1.523933258	0.26431896	4.124630945	2	3.076665209	4.874587708	5.949776857	5.93454154	5.227950776	4.644600858
ENSG00000010610	CD4	-4.00803025	0.013021922	8.130483429	8.22E-12	4.122453179	2	4.93638933	2	2.141737246	2.2680111	11.41191034	11.4680018
ENSG00000153094	BCL2L11	2.251452502	0.091310742	1.870710802	0.160681517	4.122163304	3.327711234	4.93638933	4.874587708	6.676346669	6.52015598	6.998648055	4.522269241
ENSG00000162194	LBHD1	4.046151037	0.009852067	0.073492717	0.970602478	4.119643754	2	5.457120953	2	7.952038749	7.83643742	4.240999983	4.241259475
ENSG00000122728	TAF1L	1.979918942	0.000706847	2.133846003	0.000205791	4.113764945	5.330594134	4.710564154	5.39244166	6.998639523	6.99676009	7.254397864	7.030725399
ENSG00000100024	UPB1	0.012690705	0.997041173	4.100351053	0.078044162	4.113041758	2	2	2	2.3892346	2.2680111	4.378961816	4.468068808
ENSG00000101473	ACOT8	1.457307622	0.292205343	2.652357323	0.037378894	4.109664944	4.815609471	6.311476754	2	6.640968371	6.58294721	7.792277365	7.754841567
ENSG00000196968	FUT11	1.364406184	0.491651383	2.744359437	0.130332141	4.108765622	8.609439662	5.838937808	2	8.616799622	8.52912793	9.88234985	10.00353926
ENSG00000142875	PRKACB	1.376934383	0.278205251	2.731203277	0.018970092	4.10813766	6.96351253	8.029695761	4.058998049	8.504819462	8.35116763	9.976966829	9.537896513
ENSG00000165071	TMEM71	2.691942469	0.359851119	1.413429579	0.644563698	4.105372048	2	2	2	3.663521627	3.08597292	2.885263063	2.592996859
ENSG00000204899	MZT1	1.915875965	0.279417377	2.1890011	0.201181835	4.104877064	3.327711234	6.535001748	2	7.053486362	6.90635305	7.41654498	7.05482945
ENSG00000152818	UTRN	1.46745059	0.015653107	2.637381922	3.42E-06	4.104832512	7.485143553	8.993716073	8.460932916	9.889311677	9.91517834	10.9525553	11.17653005
ENSG00000163126	ANKRD23	3.611539498	0.077966393	0.491051841	0.839388001	4.102591339	2	3.076665209	2	5.003571641	4.60967607	3.035909414	2.421067459
ENSG00000180626	ZNF594	2.91648701	4.19E-05	1.186065888	0.127844617	4.102552898	4.815609471	4.11352228	4.523675391	7.169761359	7.14977896	5.699879814	5.313094696
ENSG00000072134	EPN2	1.744714861	0.354286428	2.354943866	0.189186264	4.099658727	5.866944937	8.414402626	2	8.852852814	8.73253609	9.465896789	9.330261388
ENSG00000122786	CALD1	0.499761288	0.390679629	3.599119735	3.67E-14	4.098881023	11.72557631	12.86962125	12.5344401	12.97335982	12.9289475	16.21963215	15.857818
ENSG00000115159	GPD2	2.774277959	0.018963331	1.323712586	0.294912398	4.097990546	3.327711234	7.393647515	6.80318718	9.369349625	9.28207914	7.713417604	8.049667541
ENSG00000114771	AADAC	6.752797127	0.001424077	-2.65802783	0.315949328	4.094769296	2	2	2	6.83085985	6.83074839	2	2.117335275
ENSG00000116984	MTR	1.786425337	0.003540321	2.3074622	0.000100034	4.093887537	6.816637977	8.101365653	6.882775709	9.16555511	9.18523054	9.817084406	9.558144718
ENSG00000228623	ZNF883	0.329463551	0.867562397	3.764163669	0.014619621	4.09362722	4.467311984	2	2	3.566290607	3.39680241	5.886629224	6.704867638
ENSG00000165632	TAF3	2.039626962	0.132711491	2.053493331	0.123279471	4.093120292	2	6.602424674	5.930865648	7.737119576	7.72113133	7.872900025	7.599641096
ENSG00000170776	AKAP13	1.725405029	9.76E-11	2.36723813	1.48E-19	4.092643159	8.855032352	8.57407404	8.585098385	10.33991861	10.4389106	10.95669348	11.10085297
ENSG00000173402	DAG1	0.753824512	0.271511354	3.337280599	9.54E-09	4.091105111	8.517314938	9.936721131	9.491321578	10.16156243	10.19993	12.38000169	13.0607906
ENSG00000109323	MANBA	2.879294249	4.98E-07	1.211006168	0.051887684	4.090300416	6.009025997	7.538394143	7.398165132	9.974678372	9.98193763	8.409366736	8.229848462
ENSG00000122420	PTGFR	2.924914678	0.142927959	1.165018797	0.586886119	4.089933475	4.00722531	8.169643011	2	9.564504798	9.57015775	8.018757917	7.602159758
ENSG00000105605	CACNG7	4.936744913	0.036966061	-0.848515493	0.766427996	4.08822942	2	2	2	5.040242897	5.22805098	2.3070652	2.059860178
ENSG00000149311	ATM	2.113430366	0.01977227	1.974333269	0.029695601	4.087763635	6.366709671	8.557180776	9.06622548	10.50766186	10.4397591	10.38887163	10.27990439
ENSG00000141429	GALNT1	0.666664562	0.136159792	3.418854663	3.54E-19	4.085519226	8.39321512	9.203675681	8.677268317	9.489414201	9.44390794	12.08526024	12.32863534
ENSG00000189143	CLDN4	0.977507391	0.671105744	3.107853434	0.118374111	4.085360825	2	2	3.369317972	3.162721474	2.77779907	4.652122235	4.09155917
ENSG00000107581	EIF3A	1.746526271	0.000161536	2.336443624	2.07E-07	4.082969895	8.946185348	9.923599762	9.935792933	11.39699225	11.4287336	11.95937142	12.04371163
ENSG00000106692	FKTN	1.650807606	0.003349323	2.430259052	6.26E-06	4.081066658	5.70932728	7.145017454	6.629488387	8.252792309	8.22887947	9.006068919	9.018608509
ENSG00000270757	HSPE1-MOB4	9.269271308	8.12E-10	-5.191151265	0.026848553	4.078120043	2	2	2	9.316137634	9.29338856	2	2
ENSG00000172201	ID4	-0.072187182	0.973612043	4.147475756	0.016029651	4.075288574	2	3.686303414	7.285536013	6.043074888	5.43283104	9.989523821	9.774287532
ENSG00000198380	GFPT1	1.98936392	8.34E-05	2.080646279	3.32E-05	4.0700102	8.836089686	9.763446433	8.65477198	11.17123391	11.146157	11.27469035	11.22462398
ENSG00000133800	LYVE1	-2.311756458	0.448255827	6.378816374	0.002048422	4.067059917	2	2	2	2	2.14022021	5.938563912	6.860240429
ENSG00000151458	ANKRD50	2.008734087	1.11E-05	2.057895365	5.79E-06	4.066629452	6.89194353	7.467835385	6.80318718	9.108419034	9.04730166	9.363346227	8.843938654
ENSG00000121964	GTDC1	1.222257799	0.415495349	2.842918484	0.035360505	4.065176283	4.00722531	7.002537973	3.369317972	6.856790872	6.89649843	8.505581591	8.421006134
ENSG00000152661	GJA1	1.156704498	0.049729255	2.907205982	4.64E-08	4.06391048	10.87842776	10.71894309	9.647595056	11.69886939	11.6279928	13.13511385	13.64610971
ENSG00000167778	SPRYD3	1.170201333	0.471597639	2.893633039	0.046605398	4.063834371	3.327711234	7.60566235	8.884815469	8.926937992	9.01919552	10.64892515	10.72839317
ENSG00000110876	SELPLG	2.420599233	0.199759863	1.642716176	0.393178386	4.063315409	2	5.595927442	2	6.276156955	6.59518404	5.880003936	5.524371359
ENSG00000123124	WWP1	2.187132244	7.60E-05	1.875248301	0.000778051	4.062380545	5.70932728	6.602424674	5.39244166	8.109160159	8.19108423	7.913205701	7.770601445
ENSG00000135205	CCDC146	2.438721787	0.063840574	1.623350349	0.229804148	4.062072136	4.467311984	3.076665209	2	5.44762523	5.47317115	5.008609438	4.411752372
ENSG00000166140	ZFYVE19	1.942865952	0.238681925	2.118791892	0.186976308	4.061657844	4.00722531	6.602424674	2	6.965655398	7.31795292	7.614115653	6.959831579
ENSG00000115295	CLIP4	1.745813976	0.223356242	2.315758843	0.091236017	4.061572818	3.327711234	8.191702463	6.882775709	8.828161587	8.88234995	9.415532511	9.4271968
ENSG00000121741	ZMYM2	2.162701664	0.112962085	1.892457134	0.164128485	4.055158798	4.00722531	8.522788974	8.380957451	10.07529173	10.0187431	10.09215279	9.376235922
ENSG00000100503	NIN	1.882194432	0.002012733	2.172430516	0.000273878	4.054624948	6.366709671	7.901717006	7.818184403	9.423273993	9.32862783	9.646061941	9.68444279
ENSG00000136378	ADAMTS7	-0.752028641	0.463646059	4.806407171	8.21E-09	4.05437853	8.009475818	7.979858146	5.39244166	6.755670824	6.84106088	12.14260421	12.45240024
ENSG00000117475	BLZF1	2.86888614	1.20E-13	1.18375915	0.004265603	4.05264529	7.384433967	7.393647515	6.629488387	9.974086178	10.0717013	8.429356794	8.267623722
ENSG00000227051	C14orf132	1.753544367	0.218891141	2.298942736	0.092079873	4.052487104	3.327711234	8.191702463	7.098165388	8.894758143	8.95891978	9.553595256	9.378808813
ENSG00000108825	PTGES3L-AA	6.326026666	0.005941499	-2.274386791	0.414555096	4.051639876	2	2	2	6.422239259	6.42052719	2.139668783	2
ENSG00000255072	PIGY	9.239537298	1.08E-09	-5.191151214	0.02716304	4.048386084	2	2	2	9.332862708	9.21527115	2	2
ENSG00000165868	HSPA12A	1.55729252	0.394622747	2.487103237	0.145662728	4.044395757	3.327711234	6.602424674	2	6.722207358	6.67228943	7.792277365	7.379474672
ENSG00000076928	ARHGEF1	2.186103139	0.000615007	1.85787644	0.0040438	4.043979579	7.485143553	6.535001748	5.772716771	8.863124011	8.95180002	8.48225943	8.677176152
ENSG00000182903	ZNF721	2.274647798	0.177613024	1.768386197	0.297432059	4.043033995	2	7.099070191	4.523675391	7.985332082	7.98332947	7.781653072	7.117791742
ENSG00000179071	CCDC89	2.675165863	0.167382231	1.36611685	0.500751055	4.041282713	2	3.686303414	2	4.739373666	4.63360314	3.1504466	4.062589736
ENSG00000107165	TYRP1	4.375095859	0.084532433	-0.334805001	0.914469974	4.040290858	2	2	2	4.523237872	4.76938658	2.094598369	2.421067459
ENSG00000106392	C1GALT1	2.123533601	0.02714508	1.915053984	0.046875269	4.038587584	4.467311984	7.315436877	6.073378139	8.522747711	8.40429221	8.245074976	8.272375674
ENSG00000115355	CCDC88A	2.080989142	0.061157352	1.956721723	0.076411855	4.037710866	6.89194353	8.806266215	5.59507254	9.768836458	9.72712735	9.647281667	9.60127444
ENSG00000149231	CCDC82	2.343185699	0.001927216	1.694319613	0.029895597	4.037505312	5.330594134	7.232742069	6.073378139	8.778830996	8.70032852	8.351874517	7.791718153
ENSG00000097033	SH3GLB1	1.934965142	0.088857833	2.102450294	0.057993418	4.037415436	9.602033055	9.523645035	6.203069917	10.99680327	10.9537642	11.23167307	11.04767876
ENSG00000175984	DENND2C	1.931559669	0.161064485	2.100651691	0.115858711	4.032211361	4.467311984	2	3.369317972	4.825935136	5.24368216	5.032703865	5.349527629
ENSG00000126705	AHDC1	0.917210481	0.159815785	3.114892014	3.70E-08	4.032102495	7.53297911	7.274681896	6.073378139	7.90310317	8.0713677	9.915730882	10.38529333
ENSG00000177192	PUS1	1.960827681	0.361980007	2.070503377	0.324040096	4.031331058	2	6.311476754	2	6.750147204	6.66069199	6.865802559	6.755525668
ENSG00000162383	SLC1A7	-2.311645111	0.43323806	6.342038716	0.001216799	4.030393605	2	2	2	2	2.14022021	6.446574529	6.426568739
ENSG00000074935	TUBE1	2.363379723	0.299422571	1.667010298	0.470583967	4.03039002	2	6.464272742	2	7.190301983	7.29563107	7.186216802	5.475755622
ENSG00000197361	FBXL22	0.015496265	0.997041173	4.014055787	0.129312271	4.029552052	2	2	2	2.498691189	2.14022021	4.675266036	3.940519005
ENSG00000164040	PGRMC2	2.00784454	0.000106536	2.020905742	8.82E-05	4.028750282	7.624139768	8.903033433	8.65477198	10.49078653	10.4918206	10.53493405	10.47305071
ENSG00000173641	HSPB7	0.243515554	0.898893943	3.78402272	0.01182329	4.027538273	7.975256505	7.189546432	2	7.218576229	7.38291816	10.71031802	10.90286456
ENSG00000188827	SLX4	1.811335489	0.002335798	2.215627473	0.000125997	4.026962962	5.532353208	4.93638933	6.203069917	7.388195945	7.28433928	7.841701071	7.614687532
ENSG00000108448	TRIM16L	3.582916858	8.58E-09	0.443095681	0.562406198	4.026012539	4.00722531	4.710564154	5.156626401	7.966401501	8.14188248	5.102662916	5.050803619
ENSG00000149054	ZNF215	2.650648555	0.102405989	1.374349305	0.420838949	4.024997861	2	4.93638933	2	6.078767801	5.9536761	4.920961476	4.80186789
ENSG00000150457	LATS2	0.939491323	0.173748267	3.085400466	2.87E-07	4.024891789	7.384433967	8.862353853	7.691296561	9.018234236	9.11579188	11.26701432	11.1410292
ENSG00000187627	RGPD1	9.214372382	1.18E-09	-5.191151245	0.027118898	4.023221137	2	2	2	9.236404433	9.26398924	2	2
ENSG00000197608	ZNF841	2.494272658	0.000952027	1.526878193	0.053856478	4.02115085	5.330594134	6.535001748	4.874587708	8.166267464	8.23475766	7.567458938	6.847559257
ENSG00000073614	KDM5A	2.22178311	0.098102627	1.799228277	0.181719899	4.021011387	4.467311984	9.079034257	8.721235202	10.54920508	10.5883315	10.12946437	10.16556732
ENSG00000256977	LIMS3	9.211192851	1.22E-09	-5.191151235	0.027169109	4.020041616	2	2	2	9.277301907	9.21626181	2	2
ENSG00000130783	CCDC62	2.860715546	0.407755293	1.159216542	0.751485356	4.019932088	2	2	2	3.663521627	3.33976443	3.035909414	2.117335275
ENSG00000196449	YRDC	2.083505786	0.044893175	1.935558165	0.061325919	4.019063951	4.467311984	7.145017454	5.156626401	8.03159949	8.16669309	7.993660487	7.915353355
ENSG00000142347	MYO1F	0.691793707	0.760287901	3.326687697	0.082542158	4.018481404	4.00722531	2	2	3.162721474	3.50452879	5.976323695	4.4231929

ENSG00000051825	MPHOSPH9	1.98491133	0.389955811	2.032910796	0.366696857	4.017822125	2	6.844429755	2	7.363085426	7.13728069	7.215456797	7.382408159
ENSG00000176170	SPHK1	2.155072207	0.245027882	1.861493913	0.313731738	4.01656612	8.816894989	6.899080544	2	9.693134408	9.72990471	9.735024004	9.015305099
ENSG00000182759	MAFA	-0.307357422	0.924039274	4.321943988	0.079932646	4.014586566	2	2	2	2.3892346	2.14022021	4.946551782	4.161535617
ENSG00000174899	PQLC2L	4.362046499	0.054872798	-0.347533605	0.902389555	4.014512893	2	2	2	4.670864177	4.60967607	2.34604626	2.172608095
ENSG00000215717	TMEM167B	2.00532718	0.079057979	2.002770183	0.075111972	4.008097363	4.815609471	8.433041974	7.342949392	9.425008328	9.51406378	9.599435435	9.3226454
ENSG00000122477	LRRC39	2.689468836	0.348815215	1.318510434	0.662784961	4.00797927	2	2	2	3.406930997	3.39680241	2.592885947	2.817676617
ENSG00000198633	ZNF534	1.709404053	0.310604101	2.298555335	0.149193138	4.007959388	2	4.11352228	2	4.470469939	4.2560182	4.977914261	4.729989799
ENSG00000184619	KRBA2	2.747136273	0.100138144	1.259285049	0.478214219	4.006421322	3.327711234	3.076665209	2	4.783303513	4.85329164	3.968654369	3.261853426
ENSG00000127863	TNFRSF19	4.256767635	0.004847695	-0.253111148	0.89539314	4.003656487	2	5.303523747	2	7.990026167	7.90439517	4.088436536	3.620995423
ENSG00000088888	MAVS	1.430525863	0.095909493	2.572245664	0.001180736	4.002771527	7.159102577	9.514922632	8.742726175	10.18273194	10.1949133	11.29537729	11.35960277
ENSG00000152359	POC5	2.393091584	0.185239825	1.608786305	0.382493233	4.001877889	2	5.303523747	2	6.113598934	6.14893615	5.646229369	5.115701921
ENSG00000121039	RDH10	2.992569752	0.022108229	1.008761815	0.484862967	4.001331566	3.327711234	7.73146271	7.896971941	10.23408802	10.2153718	7.957931324	8.501461594
ENSG00000121621	KIF18A	2.268716152	0.159857485	1.731764474	0.285691035	4.000480626	3.327711234	3.686303414	2	4.946755474	4.811949	4.927401733	3.660199087
ENSG00000240038	AMY2B	4.536107985	0.013985031	-0.538029497	0.813331018	3.998078488	2	3.076665209	2	5.818703821	5.44640346	2.592885947	2.225841198
ENSG00000120616	EPC1	2.592761511	9.45E-09	1.404026771	0.00315168	3.996788282	5.532353208	6.389896633	6.322058544	8.68062069	8.65428713	7.5848779	7.402777233
ENSG00000248333	CDK11B	3.056332831	0.00492344	0.938907334	0.441681135	3.995240165	7.790810999	4.710564154	5.59507254	9.620825434	9.61591793	7.464938976	7.583161732
ENSG00000081177	EXD2	1.540845865	0.167659551	2.452291107	0.019238354	3.993136971	5.330594134	7.73146271	5.156626401	8.178656739	8.03813956	9.075851216	8.944622926
ENSG00000167840	ZNF232	1.879526622	0.097789727	2.113373991	0.05457653	3.992900614	4.00722531	5.131603625	2	5.978405189	5.85535975	6.310306306	5.941260754
ENSG00000071051	NCK2	1.642103102	0.00017253	2.350431392	2.50E-08	3.992534494	6.816637977	6.899080544	7.098165388	8.553199415	8.54656162	9.512153949	8.93466504
ENSG00000172939	OXSR1	1.609675243	4.52E-07	2.382680987	1.55E-14	3.992356231	8.564112515	8.191702463	8.677268317	10.11987514	10.0451865	10.73312834	10.96521391
ENSG00000164440	TXLNB	4.196290716	0.066039055	-0.204719335	0.94282113	3.991571381	2	2	2	4.358765249	4.63360314	2.34604626	2.225841198
ENSG00000176919	C8G	2.214057564	0.464304361	1.777420576	0.553713666	3.99147814	2	2	2	3.227757282	3.01487888	2.987421008	2.817676617
ENSG00000173960	UBXN2A	1.783208833	0.003013039	2.206816159	0.000157435	3.990024992	7.096806373	5.838937808	6.43197674	8.239055745	8.2960118	8.947864566	8.367233194
ENSG00000146463	ZMYM4	2.273414827	0.088904811	1.715221885	0.204625268	3.988636712	4.00722531	8.639735838	8.076950877	10.10588754	10.0771644	9.5209639	9.550666404
ENSG00000064999	ANKS1A	1.317161766	0.54318775	2.670749155	0.179837111	3.987910922	4.00722531	8.213429689	2	8.001694993	8.0314011	9.523622995	9.167610318
ENSG00000215193	PEX26	1.203580496	0.254759301	2.784194833	0.003409966	3.987775329	7.867441304	4.93638933	7.398165132	8.315844936	8.36909406	9.729736885	10.07282122
ENSG00000068831	RASGRP2	-1.632406642	0.315223626	5.619836101	1.43E-05	3.987429459	3.327711234	2	4.058998049	2.270786764	2.77779907	7.631969638	8.538752857
ENSG00000163785	RYK	1.951523651	4.16E-08	2.035367283	8.06E-09	3.986890934	6.96351253	7.315436877	6.80318718	9.014783325	8.94225201	9.119393978	9.002492046
ENSG00000197372	ZNF675	2.40445282	0.049351523	1.582077108	0.209143226	3.986529928	2	4.93638933	3.369317972	6.01571348	6.1068222	5.47655442	5.108633494
ENSG00000162927	PUS10	1.9912333	0.065284486	1.995155413	0.06000113	3.986388714	4.00722531	3.076665209	4.058998049	5.348759107	5.36298494	5.60262961	5.065479856
ENSG00000165097	KDM1B	1.546189058	0.30511035	2.436463072	0.084387372	3.98265213	2	5.131603625	6.718950348	7.140504127	6.94032357	8.164905085	7.609689469
ENSG00000173013	CCDC96	2.091222849	0.244964234	1.891370552	0.28796405	3.982593401	2	2	4.523675391	4.825935136	4.89348248	4.416072028	4.919632516
ENSG00000198729	PPP1R14C	1.279569437	0.714145675	2.701921349	0.396311091	3.981490787	2	2	2	3.023133209	2.2680111	3.663836244	3.100202476
ENSG00000167523	SPATA33	1.612857579	0.237679353	2.368449245	0.066070408	3.981306823	3.327711234	5.303523747	2	5.627094009	5.62411716	6.202919533	6.451924088
ENSG00000121361	KCNJ8	2.565164756	0.117408064	1.415067735	0.40849289	3.980232491	3.327711234	6.140560021	2	7.337530098	7.15805139	6.550361701	5.581648407
ENSG00000132356	PRKAA1	2.020577336	0.052853599	1.95928951	0.058034497	3.979866846	8.313285031	8.147241016	5.156626401	9.76541805	9.70330142	9.643130384	9.703627437
ENSG00000175787	ZNF169	1.25756401	0.524970355	2.721888807	0.125049462	3.979452816	2	3.686303414	2	3.840289928	3.33976443	4.834511823	4.634793542
ENSG00000137975	CLCA2	0.497871438	0.882991782	3.480268575	0.205101914	3.978140013	2	2	2	2.498691189	2.38539787	3.215043717	4.4231929
ENSG00000143248	RGS5	-3.412758367	0.016504635	7.390698486	1.47E-08	3.977940119	8.892187472	7.954278496	3.369317972	4.825935136	4.63360314	15.40760851	15.18675872
ENSG00000159259	CHAF1B	1.207146053	0.349641507	2.770024042	0.016350264	3.977170094	4.00722531	3.686303414	2	4.54891529	4.16043394	5.751606453	5.713290392
ENSG00000019995	ZRANB1	2.229422518	0.120981575	1.744488457	0.229217512	3.973910975	6.468621203	8.255926468	3.369317972	9.262828394	9.2990102	8.99844268	8.569323645
ENSG00000182329	KIAA2012	1.59688831	0.649878555	2.376948523	0.474346524	3.973836834	2	2	2	2.868578001	2.77779907	3.724264246	2.421067459
ENSG00000099381	SETD1A	1.777838787	0.433814242	2.195725069	0.312608952	3.973563856	3.327711234	8.276714465	2	8.442826028	8.56222967	8.77875662	9.044303988
ENSG00000162402	USP24	2.051045699	0.168292291	1.92134335	0.191124222	3.972389049	3.327711234	8.557180776	7.098165388	9.44052423	9.51808625	9.35261874	9.349127298
ENSG00000117152	RGS4	0.084829216	0.88968542	3.886555059	4.15E-19	3.971384275	9.143983763	9.148351514	9.839340503	9.496854254	9.49867529	12.93004339	13.58122643
ENSG00000124762	CDKN1A	0.569826231	0.643838836	3.401369374	0.000826214	3.971195605	12.98008639	10.95004268	10.2528023	12.42805917	12.4626484	15.75086129	14.56374084
ENSG00000186834	HEXIM1	2.529237251	0.031442498	1.44153827	0.243017249	3.970775521	8.696033109	7.051611315	4.874587708	10.04290398	10.135408	8.937532627	9.075453001
ENSG00000164400	CSF2	9.161904005	1.72E-09	-5.19115121	0.027439899	3.970752795	2	2	2	9.161399541	9.23397834	2	2
ENSG00000089094	KDM2B	2.115554878	0.142046401	1.854205551	0.19591446	3.96976043	4.467311984	7.315436877	3.369317972	7.987681034	8.14396641	7.6993745	7.914340301
ENSG00000142687	KIAA0319L	1.72671269	0.174638808	2.24259072	0.065343113	3.96930341	5.532353208	9.018611864	6.534109959	9.480268385	9.50517467	9.929431577	10.07916314
ENSG00000140534	TICRR	1.910529589	0.145397751	2.058130233	0.106931842	3.968659821	2	5.131603625	3.369317972	5.651002631	5.86549842	5.751606453	6.033722345
ENSG00000099810	MTAP	1.985796858	0.005771922	1.981722623	0.005607289	3.96751948	5.095959458	7.099070191	6.80318718	8.519504557	8.50663048	8.625789205	8.381965189
ENSG00000157107	FCHO2	2.173046876	0.123897637	1.79311895	0.205383605	3.966165825	7.485143553	4.93638933	3.369317972	8.354580458	8.24644267	8.15052603	7.662532142
ENSG00000124789	NUP153	1.95411257	0.06013597	2.009947571	0.049513463	3.964060142	6.653098118	8.671479029	5.930865648	9.452477833	9.5678312	9.604970226	9.527664835
ENSG00000184743	ATL3	1.423062936	0.21977808	2.540257985	0.018389876	3.963320921	5.866944937	8.487557258	9.413059136	9.893075744	9.96494592	10.91456512	11.16177291
ENSG00000030304	MUSK	1.014404791	0.754153092	2.948282904	0.302076863	3.962687695	2	2	2	2.784612485	2.38539787	3.355392153	3.753786374
ENSG00000165617	DACT1	1.818696993	0.308260189	2.138166469	0.214486601	3.956863462	7.485143553	8.337339476	2	9.172798677	9.22317745	9.959790553	8.870820921
ENSG00000172057	ORMDL3	1.601385503	0.373813246	2.351542826	0.166636971	3.952928329	9.48530526	5.595927442	4.058998049	9.6094308	9.61140668	10.25702296	10.45156308
ENSG00000181074	OR52N4	1.275726885	0.714005062	2.676305764	0.399502541	3.95203265	2	2	2	2.784612485	2.59489879	3.904814898	2.592996859
ENSG00000117411	B4GALT2	1.321824368	0.254759301	2.626233451	0.013611554	3.948057819	6.138360429	9.13702746	6.958202216	9.315202754	9.27828955	10.56931498	10.62180609
ENSG00000107186	MPDZ	1.76598435	0.140821478	2.180372103	0.058715617	3.946356453	5.095959458	8.451443573	6.322058544	8.99156386	9.05839257	9.401144325	9.471950246
ENSG00000116830	TTF2	1.408824307	0.539839197	2.534984585	0.236787259	3.943808893	2	6.602424674	2	6.260701031	6.63721363	7.504242605	7.597118029
ENSG00000138835	RGS3	2.277342002	0.111902276	1.664866853	0.25288003	3.942208855	9.159305284	6.228547981	4.874587708	10.08245238	10.059057	9.377526577	9.540196884
ENSG00000115568	ZNF142	1.566927434	0.335922911	2.374913579	0.121465318	3.941841013	2	7.431218269	6.073378139	7.910550164	7.84158259	8.898729828	8.407389648
ENSG00000163166	IWS1	1.758235374	0.343732241	2.183377004	0.221346372	3.941612378	6.138360429	8.433041974	2	8.772033429	8.95417718	9.183011957	9.385769334
ENSG00000055609	KMT2C	1.912748759	0.016043704	2.026419798	0.009779302	3.939168556	8.228665467	8.777382925	6.534109959	9.978816937	10.0579523	10.22208805	10.03626976
ENSG00000184486	POU3F2	3.339399653	0.282118771	0.599627194	0.864701858	3.939026846	2	2	2	4.470469939	2.68924543	2.560105256	2.374681325
ENSG00000178226	PRSS36	1.594829058	0.61206249	2.343554694	0.428528777	3.938383752	2	2	2	2.695456494	2.94009902	3.336159116	3.042026731
ENSG00000165630	PRPF18	3.307194222	0.000161834	0.631082667	0.542075443	3.938276889	2	5.131603625	4.874587708	7.656739692	7.57432889	4.990270811	5.18454436
ENSG00000005889	ZFX	1.973565339	5.31E-09	1.960256032	5.84E-09	3.933821371	7.940205782	7.572420273	7.285536013	9.571568433	9.58019637	9.578360891	9.546749232
ENSG00000159110	IFNAR2	3.76166134	1.44E-09	0.17190437	0.835180221	3.933565709	5.330594134	5.595927442	4.058998049	8.81895723	8.73392024	5.096960836	5.524371359
ENSG00000186832	KRT16	-0.303158672	0.946210441	4.23523163	0.258826994	3.932072958	2	2	2	2.498691189	2	4.848153631	4.133950982
ENSG00000115363	EVA1A	2.409501782	0.004330368	1.519411116	0.085343919	3.928912898	7.904282243	5.838937808	6.322058544	9.313331174	9.35675148	8.091532339	8.741420621
ENSG00000179532	DNHD1	2.462163631	0.051696866	1.464457394	0.266306871	3.926621025	2	6.228547981	5.930865648	7.942383523	7.88956768	7.21150425	6.610445962
ENSG00000114573	ATP6V1A	2.291182427	0.042589119	1.635014077	0.156803554	3.926196504	5.330594134	8.820493869	8.825624954	10.5602946	10.5974847	9.882142656	9.966632301
ENSG00000188015	S100A3	0.554604805	0.744767011	3.367909491	0.016078695	3.922514296	3.327711234	3.686303414	2	3.615725078	3.50452879	5.201624686	6.338680316
ENSG00000274166		7.064429395	1.11E-06	-3.143713684	0.094537496	3.920715711	2	2	3.369317972	8.124184191	8.02236724	2.094598369	2.059860178
ENSG00000256235	SMIM3	2.764872092	2.42E-05	1.154696701	0.106137285	3.919568793	8.367060731	6.666836837	7.225742779	10.3495391	10.3093543	8.800846924	8.652479133
ENSG00000198513	ATL1	2.285945541	0.270109437	1.633344623	0.437547935	3.919290164	2	6.140560021	2	6.777556219	6.9210099	6.098353065	6.344709081
ENSG00000164241	C5orf63	2.139886124	0.203854133	1.778732054	0.289422154	3.918618178	3.327711234	2	3.369317972	4.105493497	4.58534546	4.209638631	3.940519005
ENSG00000111843	TMEM14C	1.985839664	0.000759047	1.931189404	0.001031466	3.917029068	7.82963488	8.356994601	6.80318718	9.741259681	9.80689202	9.77752902	9.660173108
ENSG00000113615	SEC24A	2.23980086	0.219632896	1.676009717	0.3644148	3.915810577	2	8.94259736	8.380957451	10.33669748	10.335119	9.797442274	9.750643668
ENSG00000168172	HOOK3	1.603339496	5.11E-05	2.31240017	1.58E-09	3.915739666	8.444143041	8.639735838	7.777116083	9.927741409	9.93517152	10.6333753	10.64305971
ENSG00000112659	CUL9	1.862196999	0.223543123	2.052911929	0.167672785	3.915108929	7.384433967	6.535001748	2	8.262524677	8.28661061	8.51523351	8.408829029
ENSG00000196396	PTPN1	1.751800426	0.334567146	2.16033079	0.215861	3.912131216	8.138771685	8.655694736	2	9.56056556	9.60006644	9.769486116	10.17617136
ENSG00000242220	TCP10L	1.690450301	0.613127293	2.220811585	0.484665142	3.911261886	2	2	2	2.947924294	2.77779907	3.517841605	2.592996859
ENSG00000132185	FCRLA	3.010996438	0.281735266	0.899615761	0.769071028	3.910612199	2	2	2	3.566290607	3.65239003	2.68698273	2.421067459
ENSG00000197948	FCHSD1	1.908303732	0.015099487	1.999254338	0.009971111	3.907558069	5.095959458	6.140560021	7.285536013	8.279879181	8.29975519	8.350677018	8.408109518
ENSG00000111737	RAB35	1.872837425	0.067435031	2.034606382	0.042390554	3.907443807	8.517314938	5.457120953	7.646393253	9.424141421	9.59930725	9.660389416	9.690077045
ENSG00000153827	TRIP12	2.256792155	1.24E-07	1.650453288	0.000157435	3.907245443	9.2903348	9.695596167	10.28245311	12.05260425	12.0764293	11.46059623	11.45726032
ENSG00000171509	RXFP1	1.690445815	0.59518254	2.215299096	0.464147051	3.905744912	2	2	2	2.947924294	2.77779907	3.355392153	2.851942824
ENSG00000186814	ZSCAN30	1.50564585	0.479224001	2.398700354	0.228768931	3.904346205	3.327711234	7.60566235	2	7.626911583	7.5462416	8.522164399	8.408109518
ENSG00000122565	CBX3	2.255883912	0.00010208	1.646183374	0.005906677	3.902067285	7.031698319	8.433041974	7.451345288	10.04290398	9.97727021	9.491657377	9.309990451
ENSG00000101665	SMAD7	2.248879609	0.217503328	1.65309165	0.370178383	3.901971259	6.009025997	8.433041974	2	9.36754703	9.29526488	8.780980934	8.699106494
ENSG00000065243	PKN2	2.248992834	0.09785965	1.65234966	0.230540721	3.901342494	4.815609471	8.414402626	5.39244166	9.359407363	9.29432703	8.964399877	8.462487876
ENSG00000135324	MRAP2	-2.308732211	0.442830958	6.204309841	0.00226213	3.89557763	2	2	2	2.141737246	2	6.433059984	6.165581817
ENSG00000134504	KCTD1	1.914459697	0.309774396	1.980472064	0.281992691	3.894931761	2	6.844429755	3.369317972	7.230526165	7.30683517	7.600074836	7.006211772
ENSG00000237172	B3GNT9	1.59403503	0.194136557	2.300528882	0.047681033	3.894563913	7.709880939	7.874700259	4.058998049	8.838609445	8.83669913	9.450887435	9.622520313
ENSG00000115598	IL1RL2	3.114061087	0.26497168	0.779109435	0.802048583	3.893170522	2	2	2	3.462030052	3.86943969	2.656294034	2.374681325
ENSG00000167074	TEF	1.369573053	0.379217697	2.521855017	0.079263128	3.89142807	6.138360429	7.189546432	2	7.398825229	7.65837038	8.475133675	8.840742848
ENSG00000183624	HMCES	1.683027878	0.277913797	2.208061904	0.135257068	3.891089782	2	7.145017454	7.029880454	8.157948442	8.17894021	8.536980328	8.823577951
ENSG00000123473	STIL	1.865041603	0.049361415	2.025503674	0.02901671	3.890545277	4.815609471	4.93638933	2	6.113598934	6.0722229	6.221381643	6.267475858
ENSG00000135269	TES	0.83789391	0.565892094	3.051741934	0.016163983	3.889635843	8.517314938	8.451443573	4.058998049	8.744518279	8.78154738	10.85690136	11.06843302
ENSG00000176658	MYO1D	1.116777974	0.44238177	2.771699121	0.03297173	3.888477094	4.467311984	7.761270542	9.116480286	9.148860528	9.15455108	10.58365688	10.98754108
ENSG00000103042	SLC38A7	2.241072812	0.195613485	1.647038766	0.348235366	3.888111578	2	7.189546432	8.408111533	9.576258382	9.54829592	8.936734789	9.00677572
ENSG00000151348	EXT2	2.398865362	0.078490009	1.487782223	0.29074063	3.886647585	10.00958825	9.267380431	5.156626401	11.51507238	11.5302082	10.39396692	10.80363534
ENSG00000278558	TMEM191B	2.609393652	0.222533677	1.271382925	0.5723508	3.880776577	2	2	3.369317972	3.881293667	4.12711147	2.803598391	3.407202941

ENSG00000197302	ZNF720	1.849724522	0.158007309	2.030067467	0.110621519	3.879791989	5.532353208	2	5.59507254	6.652857793	6.77805036	6.91847879	6.862343163
ENSG00000214517	PPME1	0.468905141	0.706963346	3.408396091	0.000758118	3.877301233	9.635285994	9.616264339	6.43197674	9.596407142	9.57402703	12.33048163	12.68428561
ENSG00000162129	CLPB	1.322351636	0.601636131	2.553792643	0.274904665	3.87614428	2	7.393647515	2	7.210554257	7.09050444	8.417967842	8.299011699
ENSG00000134256	CD101	1.384547394	0.695516458	2.49112559	0.446711602	3.875672983	2	2	2	2.270786764	3.08597292	3.059555873	3.474731315
ENSG00000135452	TSPAN31	2.144614413	0.002796453	1.730705067	0.018121893	3.87531948	5.70932728	7.274681896	5.772716771	8.598473365	8.54024645	8.020265032	8.28888532
ENSG00000112276	BVES	2.851114639	0.005610729	1.020504935	0.368897909	3.871619574	4.467311984	6.666836837	7.691296561	9.601017164	9.56860713	8.127636775	7.302581437
ENSG00000113721	PDGFRB	0.546736728	0.430113744	3.324563343	5.04E-09	3.871300071	9.497412313	10.79296235	9.767769415	10.66893665	10.6923799	13.70188182	13.15825212
ENSG00000064989	CALCRL	0.55361321	0.689610102	3.317509787	0.003344821	3.871122997	4.00722531	4.442710129	2	4.598938466	3.86943969	7.078941352	6.362645675
ENSG00000188763	FZD9	0.227802372	0.902061374	3.643219219	0.010541206	3.871021591	2	4.710564154	2	3.79808662	3.74316441	6.433059984	6.994755759
ENSG00000197226	TBC1D9B	1.631750367	1.00E-07	2.235869475	8.25E-14	3.867619842	9.159305284	9.497318007	9.031722608	10.85926146	10.8881455	11.34463044	11.59798046
ENSG00000130810	PPAN	2.972178563	0.010826309	0.893913026	0.49395309	3.866091588	2	5.303523747	6.203069917	8.157948442	8.08010078	5.988693817	6.215784511
ENSG00000187605	TET3	0.914114326	0.590107068	2.951480803	0.047464741	3.865595129	6.138360429	7.538394143	2	7.25803034	7.4074129	9.364236594	9.324934421
ENSG00000075407	ZNF37A	1.934716663	0.026360838	1.930549605	0.025734513	3.865266268	5.866944937	7.874700259	6.073378139	8.832088406	8.82245717	9.004546892	8.615557586
ENSG00000151748	SAV1	2.224509528	0.190049203	1.640643202	0.340182675	3.86515273	2	7.572420273	8.236946975	9.56371781	9.56860713	9.009866973	8.961885532
ENSG00000120526	NUDCD1	2.512768606	0.000367264	1.351815671	0.07180717	3.864584277	5.330594134	5.946638857	7.163363831	8.737556707	8.84440862	7.81936672	7.451079708
ENSG00000178691	SUZ12	2.167011133	0.000449271	1.697099415	0.007198919	3.864110548	5.70932728	6.951736442	7.398165132	9.023967474	8.91810182	8.632209947	8.368713182
ENSG00000109511	ANXA10	4.416628852	0.044311542	-0.557392851	0.836438942	3.859236001	2	2	2	4.574143677	4.79082474	2.183373728	2.277179783
ENSG00000128016	ZFP36	1.594506055	0.237182775	2.263433052	0.077151749	3.857939107	7.485143553	7.761270542	3.369317972	8.657201865	8.65136017	9.473343311	9.143604888
ENSG00000168610	STAT3	1.431155922	0.004873329	2.426557809	4.48E-07	3.857713731	8.716893051	9.532315021	9.897982195	10.8713966	10.9036026	11.76485583	11.98936987
ENSG00000185838	GNB1L	1.913479453	0.298900033	1.942553013	0.279801848	3.856032466	2	4.93638933	2	5.258265913	5.39133044	5.740677235	4.819291477
ENSG00000223953	C1QTNF5	6.604761574	8.17E-05	-2.75116766	0.219885804	3.853593915	2	2	2	6.592405944	6.77805036	2.048074405	2.059860178
ENSG00000170558	CDH2	3.01980793	5.21E-06	0.833420424	0.268600882	3.853228354	9.521325812	8.791896851	7.818184403	11.87684221	11.8819476	10.01997556	9.283950453
ENSG00000166780	C16orf45	3.951720011	0.002208346	-0.099070654	0.953066893	3.852649357	6.96351253	6.464272742	2	10.0321341	10.0836929	6.315225883	5.821002687
ENSG00000235162	C12orf75	1.548254703	0.00601843	2.304381855	1.84E-05	3.852636557	7.579279349	8.702538795	8.922968322	10.0321341	10.0645682	10.92315021	10.67046947
ENSG00000078043	PIAS2	1.965224316	0.131051356	1.88722739	0.141662309	3.852451706	3.327711234	7.35507212	5.59507254	8.205140657	8.06478301	8.087943566	8.031095054
ENSG00000113916	BCL6	2.004211213	0.288045778	1.846222942	0.321893604	3.850434155	8.998234871	7.189546432	2	9.786482773	9.74988221	9.851568452	9.322263543
ENSG00000156504	FAM122B	2.161312927	0.053389543	1.688260638	0.135544734	3.849573566	6.816637977	5.303523747	3.369317972	7.939959586	7.71553728	7.272216802	7.459438299
ENSG00000113600	C9	4.06595011	0.109507808	-0.216859972	0.944157185	3.849090139	2	2	2	4.598938466	4.16043394	2.139668783	2.421067459
ENSG00000167077	MEI1	2.139668156	0.199554781	1.709381387	0.305685651	3.849049543	2	4.11352228	2	4.647285135	4.79082474	4.188345803	4.522269241
ENSG00000183479	TREX2	0.493769106	0.883547191	3.35521693	0.22200216	3.848986036	2	2	2	2.270786764	2.59489879	4.076896059	3.580696614
	WTH3DI	9.038786388	3.86E-09	-5.191151161	0.028077653	3.847635226	2	2	2	9.091045942	9.06060056	2	2
ENSG00000100014	SPECC1L	1.913376227	0.184360992	1.933352071	0.171451798	3.846728298	4.815609471	8.981105197	6.073378139	9.60255057	9.48723044	9.58830174	9.543149124
ENSG00000130725	UBE2M	1.789832368	0.019984214	2.055790145	0.006279543	3.845622513	7.096806373	8.234834549	6.203069917	9.138327453	9.24954577	9.385734536	9.529320022
ENSG00000082126	MPP4	1.796421316	0.454376509	2.049010497	0.375401268	3.845431813	5.532353208	2	2	5.614989456	5.7929843	4.177580368	6.711881188
ENSG00000135722	FBXL8	2.433680645	0.184268122	1.411164752	0.457954601	3.844845397	2	5.457120953	2	6.34372989	6.29861567	5.381251181	5.361470313
ENSG00000246922	UBAP1L	1.177500794	0.557663675	2.667102872	0.141298013	3.844603666	2	4.11352228	2	3.840289928	4.02226117	5.684753614	4.316807515
ENSG00000037749	MFAP3	2.051857837	0.386972978	1.792112626	0.446711602	3.843970463	2	8.862353853	3.369317972	9.398771806	9.30087923	9.121501891	9.063165393
ENSG00000008283	CYB561	2.031022707	0.265448614	1.812458965	0.31713805	3.843481672	8.609439662	6.78762695	2	9.345738359	9.45149977	8.79689555	9.486337564
ENSG00000160392	C19orf47	1.543142711	0.067215792	2.299811026	0.003805056	3.842953737	5.532353208	5.838937808	3.369317972	6.79375511	6.65485813	7.34858644	7.563910189
ENSG00000104517	UBR5	2.21635571	0.017319385	1.62403783	0.089230416	3.840393541	6.257048221	8.590771778	9.031722608	10.5602946	10.5648023	10.13399593	9.789837542
ENSG00000004139	SARM1	2.298685981	0.302610403	1.540862191	0.496738835	3.839548171	2	6.666836837	2	7.497728447	7.26148706	6.643273885	6.657218517
ENSG00000066422	ZBTB11	1.866358618	0.228635685	1.971880222	0.192814682	3.83823884	7.096806373	7.232742069	2	8.446246007	8.41468635	8.82044307	8.178454506
ENSG00000106665	CLIP2	2.111796345	0.155215303	1.725447896	0.247204345	3.837244241	7.867441304	9.377333006	4.058998049	10.350452	10.3581897	10.0231736	9.913373788
ENSG00000188396	TCTEX1D4	1.384548723	0.694728637	2.452649284	0.453575633	3.837198007	2	2	2	2.270786764	3.08597292	3.355392153	3.15612297
ENSG00000228454		9.025510646	4.27E-09	-5.191151148	0.02816633	3.834359498	2	2	2	9.087765067	9.03724641	2	2
ENSG00000233312		9.025510646	4.27E-09	-5.191151148	0.02816633	3.834359498	2	2	2	9.087765067	9.03724641	2	2
ENSG00000198894	CIPC	1.707915088	0.363034555	2.126400785	0.239257685	3.834315873	2	7.315436877	4.058998049	7.632926745	7.4949172	8.047866378	7.90110545
ENSG00000177119	ANO6	1.547512202	7.89E-07	2.286208696	6.02E-14	3.833720898	8.946185348	8.395519306	8.536706852	10.19345574	10.1629006	10.91497027	10.91494442
ENSG00000158286	RNF207	1.881140986	0.000674334	1.952576054	0.000352745	3.83371704	5.532353208	5.838937808	4.874587708	7.281195074	7.28811304	7.229857522	7.469129172
ENSG00000169180	XPO6	1.434132286	0.220372027	2.399435599	0.028282877	3.833567885	7.384433967	9.988044813	7.225742779	10.22465921	10.2262311	11.06141783	11.30508916
ENSG00000176490	DIRAS1	1.125023859	0.4962262	2.707194619	0.068373122	3.832218478	3.327711234	6.844429755	3.369317972	6.628980152	6.53293476	8.51523351	7.583161732
ENSG00000138459	SLC35A5	2.198547622	0.013143309	1.631467984	0.072675764	3.830015606	4.815609471	7.393647515	7.285536013	9.048926513	9.03612482	8.579514585	8.374617996
ENSG00000115137	DNAJC27	1.905929406	0.131015998	1.923228319	0.120415896	3.829157725	4.00722531	5.457120953	2	6.328984825	6.01871653	6.419417643	5.937276556
ENSG00000085382	HACE1	1.620032112	0.264978658	2.208528355	0.110369822	3.828560466	4.00722531	6.899080544	3.369317972	7.136275664	7.21069602	8.019511671	7.415721389
ENSG00000173511	VEGFB	1.56870199	0.414528109	2.259457346	0.213653476	3.828159335	8.963744667	7.099070191	2	9.244284335	9.3304589	9.937430448	10.01208774
ENSG00000179941	BBS10	1.994280328	0.400992689	1.833105681	0.435161964	3.827386009	2	7.002537973	2	7.300218887	7.53039799	7.590975897	6.836905794
ENSG00000145014	TMEM44	1.925354088	0.00020695	1.900092628	0.000238991	3.825446716	6.816637977	7.954278496	8.007629717	9.623093601	9.5678312	9.428360321	9.700104804
ENSG00000165458	INPPL1	1.702496812	3.75E-09	2.12080255	8.36E-14	3.823299362	8.910412251	9.415325457	9.227351203	10.86342445	10.9343299	11.35227943	11.28053306
ENSG00000135596	MICAL1	1.362374518	0.311059736	2.460917333	0.048008107	3.823291851	4.00722531	8.234834549	8.042706615	8.883449599	9.00321357	9.982196088	10.08952187
ENSG00000012124	CD22	0.68948932	0.841054398	3.132766088	0.280017687	3.822255408	2	2	2	2.498691189	2.49394783	4.166733998	2.981406154
ENSG00000114648	KLHL18	1.608646446	0.158698358	2.206079153	0.042028392	3.814725599	4.00722531	7.002537973	7.552162999	8.365459376	8.36195019	9.070769397	8.829501448
ENSG00000047230	CTPS2	0.94352578	0.610039424	2.870386237	0.078715146	3.813912017	3.327711234	7.538394143	3.369317972	7.106324235	6.94032357	8.739036711	9.079977539
ENSG00000269897	COMMD3-BN	9.004531104	4.87E-09	-5.191151143	0.028266186	3.813379961	2	2	2	9.035366048	9.04841459	2	2
ENSG00000122591	FAM126A	1.938297099	0.006763681	1.873016965	0.008764582	3.811314064	8.285626337	8.57407404	6.629488387	9.992332092	9.95489142	9.90251187	9.91489412
ENSG00000250565	ATP6V1E2	0.927847624	0.635885449	2.881626026	0.091607485	3.80947365	2	3.686303414	2	3.515101962	3.28037825	4.875056259	4.886954834
ENSG00000144134	RABL2A	2.588700803	0.000460886	1.218952708	0.125850973	3.807653511	5.095959458	4.710564154	3.369317972	6.815074525	7.07311172	5.65789504	5.646532192
ENSG00000141519	CCDC40	1.681361362	0.338195259	2.126066254	0.205129503	3.807427616	2	4.710564154	2	5.003571641	4.811949	5.565950232	4.982847849
ENSG00000275074	NUDT18	1.343575016	0.566735645	2.463155327	0.259170136	3.806730343	2	6.728495673	2	6.503277904	6.53928196	7.560224843	7.65163107
ENSG00000143324	XPR1	1.757619468	0.283681035	2.048160658	0.195441403	3.805780126	4.00722531	5.72254323	8.960138144	9.246247604	9.33957954	9.522559945	9.640091423
ENSG00000118200	CAMSAP2	1.997623074	2.24E-11	1.807731834	1.51E-09	3.805354908	8.228665467	8.356994601	8.175061096	10.2793709	10.2054283	10.20661666	9.882366758
	FAM196B	8.995063304	5.87E-09	-5.191151069	0.028636604	3.803912236	2	2	2	8.957217624	9.10406516	2	2
ENSG00000124181	PLCG1	1.932837716	0.186609	1.87035574	0.194491628	3.803193457	4.815609471	6.951736442	9.465702942	10.05527473	10.0977376	10.02260976	10.00615667
ENSG00000138050	THUMPD2	1.90848151	0.362456823	1.894699132	0.357657197	3.803180642	2	6.04685525	2	6.321555392	6.46787796	6.544089217	6.202567011
ENSG00000179454	KLHL28	1.689393596	0.131720426	2.113029519	0.049732463	3.802423115	4.815609471	7.002537973	4.523675391	7.596453725	7.51437845	8.333200639	7.488317792
ENSG00000159256	MORC3	2.200611086	0.009001216	1.598880122	0.064916066	3.799491208	5.330594134	7.002537973	7.734844356	9.205456697	9.09870316	8.830786521	8.2175813
ENSG00000143353	LYPLAL1	2.128483569	0.090021545	1.669475095	0.186478723	3.797958665	5.866944937	4.442710129	2	6.960881212	6.53928196	6.495072127	6.13459968
ENSG00000164535	DAGLB	1.01390443	0.592369314	2.781812144	0.099466732	3.795716574	2	5.595927442	7.97167868	7.571615825	7.70428369	9.246842239	9.514354532
ENSG00000083312	TNPO1	2.108539217	1.81E-12	1.686955404	2.29E-08	3.795494622	9.015175615	9.34816733	9.180874355	11.32822224	11.2545233	11.04540869	10.67256802
ENSG00000147679	UTP23	2.399687087	0.072707408	1.394831785	0.316040074	3.794518872	4.467311984	7.051611315	3.369317972	8.136938566	8.11451391	7.268416942	7.0024032
ENSG00000164707	SLC13A4	-1.060697213	0.571859046	4.854083778	0.001582235	3.793386565	4.815609471	2	2	3.227757282	2.59489879	8.144319434	7.425709383
ENSG00000219607	PPP1R3G	-0.003595342	0.998368875	3.795712391	0.030515328	3.792117048	2	5.838937808	2	4.329433326	4.63360314	7.988280194	8.067092663
ENSG00000159433	STARD9	1.447375651	0.188774353	2.341563013	0.023193105	3.788938663	3.327711234	6.464272742	6.073378139	7.110641269	7.2537885	8.392009143	7.627107456
ENSG00000138592	USP8	2.20264868	0.000763767	1.585566246	0.019165507	3.788214926	6.96351253	8.234834549	6.718950348	9.715981692	9.60536965	9.160294617	8.927151259
ENSG00000151006	PRSS53	1.526883649	0.399455299	2.260807349	0.184116925	3.787690998	2	4.93638933	2	5.194621444	4.74762503	5.801542492	5.475755622
ENSG00000100030	MAPK1	1.440512384	0.112649338	2.345768729	0.005734903	3.786281113	7.33131396	9.756063186	9.589475645	10.66233506	10.6610729	11.50392713	11.62416212
ENSG00000186951	PPARA	1.260541306	0.234303473	2.524516938	0.009322992	3.785058243	5.330594134	8.077869079	8.35328246	8.942762689	9.01122668	10.29518375	10.17151518
ENSG00000049860	HEXB	2.761326217	2.71E-10	1.023674638	0.032436902	3.785000855	7.867441304	8.834582582	8.825624954	11.330306	11.3221109	9.665454884	9.518360533
ENSG00000134852	CLOCK	1.295737468	0.397539867	2.488516697	0.077151749	3.784254165	7.27616306	8.540087354	3.369317972	8.740345368	8.79619691	9.989139077	9.917677242
ENSG00000123200	ZC3H13	1.932699263	0.060764772	1.851273064	0.070288397	3.783972326	5.70932728	8.451443573	8.825624954	10.02013627	10.1280575	9.993749244	9.994220888
ENSG00000164647	STEAP1	7.578431545	1.56E-05	-3.794939964	0.060834366	3.783491582	4.467311984	2	2	10.13319806	10.1057785	2.267001569	2
ENSG00000100418	DESI1	1.858491574	2.47E-05	1.923720159	1.10E-05	3.782211733	7.867441304	8.876041558	8.65477198	10.37846912	10.3800133	10.50362495	10.38236571
ENSG00000178917	ZNF852	1.877470295	0.177037027	1.902587534	0.161125537	3.78005783	4.00722531	4.11352228	2	5.040242897	5.3193943	5.699879814	4.456980017
ENSG00000167395	ZNF646	1.76167294	0.301934025	2.01717627	0.221484241	3.77884921	8.107517749	6.951736442	2	8.78154108	8.8250571	9.135128763	8.974090715
ENSG00000021826	CPS1	1.726301402	0.003288934	2.048824268	0.00033279	3.77512567	5.866944937	4.93638933	5.156626401	6.89733479	7.05550676	7.215456797	7.364717093
ENSG00000180447	GAS1	2.245774907	0.172055101	1.526230519	0.363325139	3.772005426	7.624139768	5.72254323	2	8.562683721	8.62325378	7.558151279	8.148612304
ENSG00000262180	OCLM	2.643918238	0.387577122	1.125949141	0.727622186	3.769867379	2	2	2	3.406930997	3.33976443	2.858551861	2.374681325
ENSG00000166762	CATSPER2	1.049615791	0.641058771	2.71928983	0.17152891	3.768905621	2	3.076665209	2	3.023133209	3.01487888	4.065262523	4.119958326
ENSG00000040531	CTNS	2.002882436	0.083653941	1.765433365	0.125782712	3.768315801	4.815609471	7.274681896	4.523675391	7.954442493	8.17486938	8.025527571	7.615934347
ENSG00000173546	CSPG4	0.114594227	0.942177035	3.652464792	0.002886376	3.767059019	10.14658276	7.467835385	6.43197674	8.923261356	9.0259911	12.84765862	12.04897144
ENSG00000179833	SERTAD2	1.98942365	8.94E-06	1.777183396	7.98E-05	3.766607046	7.940205782	7.874700259	8.677268317	10.15530726	10.2044302	9.732267925	10.17172715
ENSG00000196628	TCF4	2.533805805	0.001130382	1.231976761	0.140213693	3.765782565	8.493334308	8.88960062	6.629488387	10.80967368	10.8020184	9.422388201	9.590215873
ENSG00000177697	CD151	1.935090659	4.27E-07	1.828991123	1.81E-06	3.764081782	11.22635546	10.43440824	10.63827116	12.7143154	12.7616713	12.60099151	12.66265504
ENSG00000121057	AKAP1	1.105579359	0.405447051	2.655996407	0.026005702	3.761575766	7.27616306	4.710564154	4.058998049	7.097651217	7.08617588	8.683523874	8.541379994
ENSG00000174125	TLR1	0.486172233	0.785737459	3.275291813	0.024188368	3.761464046	2	4.11352228	2	3.462030052	3.50452879	5.661762729	5.825320785
ENSG00000114841	DNAH1	2.300830975	0.000123202	1.46037762	0.019857693	3.761208595	5.330594134	4.93638933	4.523675391	7.053486362	7.1744553	6.287958221	6.326546677
ENSG00000010292	NCAPD2	1.704838617	0.304685989	2.056362404	0.197633952	3.761201021	4.467311984	7.928237109	3.369317972	8.201097719	8.19912382	8.486081846	8.606177646
ENSG00000169862	CTNND2	2.603263373	0.471298431	1.156518261	0.760366765	3.759781634	2	2	2	3.95996233	2.2680111	2.62493832	2.671842496

ENSG00000167701	GPT	-0.723710535	0.808916091	4.482845591	0.049413588	3.759135056	2	2	2	2.270786764	2.14022021	4.690491514	4.793076545
ENSG00000137098	SPAG8	0.497858951	0.879726254	3.260275465	0.222125563	3.758134415	2	2	2	2.498691189	2.38539787	3.584558147	3.956354764
ENSG00000127946	HIP1	1.539801765	0.112267143	2.216561539	0.016024153	3.756363304	7.867441304	8.029695761	5.39244166	8.980991923	9.03612482	9.946772799	9.355859082
ENSG00000133961	NUMB	2.252234963	1.86E-18	1.504017304	7.86E-09	3.756252267	8.892187472	8.88960062	9.212024683	11.2267272	11.2638957	10.55027224	10.44543965
ENSG00000169032	MAP2K1	1.667982611	0.020106114	2.087839776	0.002656384	3.755822387	9.460782017	7.60566235	8.460932916	10.36090904	10.3572918	10.75739214	10.79801646
PPP1R12C		1.879381024	0.225247916	1.873297113	0.21938176	3.752678137	3.327711234	8.623598428	8.784770914	9.967555951	10.0396007	10.0182796	9.977343134
ENSG00000165476	REEP3	1.880660042	8.28E-09	1.87150613	8.64E-09	3.752166172	8.468948322	9.067150531	9.014156722	10.80035023	10.6966453	10.71055142	10.76944897
ENSG00000132467	UTP3	1.93971207	0.288829199	1.811515413	0.314949963	3.751227482	8.39321512	6.311476754	2	9.028537716	9.04284131	8.505043475	9.2234807
ENSG00000198909	MAP3K3	1.434152385	0.430098143	2.316179479	0.171763347	3.750331864	2	8.124485672	8.296287027	8.998569022	9.10834046	9.994516174	9.865692849
ENSG00000214827	MTCP1	3.428925614	0.022553657	0.320890204	0.861870179	3.749815818	2	4.11352228	2	5.775854763	5.98191036	3.584558147	3.128433649
ENSG00000117362	APH1A	2.113223079	0.010063091	1.635331615	0.05114311	3.748554694	8.418903792	6.389896633	8.631919287	10.17399201	10.2039308	9.5642243	9.848028389
ENSG00000161681	SHANK1	2.635063569	0.108024028	1.112900592	0.525881687	3.747964161	5.095959458	2	7.029880454	8.31771326	8.37087451	6.499401217	7.140495192
ENSG00000139737	SLAIN1	2.018537754	0.611381475	1.727864402	NA	3.746402156	2	2	2	3.566290607	2.14022021	3.1504466	2.551896972
ENSG00000102699	PARP4	3.177328631	1.87E-05	0.567799909	0.519247447	3.745128539	7.435667355	7.099070191	8.99637432	11.24574219	11.2496932	8.569770117	8.726491728
ENSG00000205143	ARID3C	0.49371813	0.88968542	3.250862887	0.266977444	3.744581018	2	2	2	2.270786764	2.59489879	4.220168345	3.128433649
ENSG00000183340	JRKL	2.019141983	0.140555028	1.725308409	0.207119046	3.744450392	6.468621203	6.535001748	2	7.947219213	7.86703672	7.840848421	7.357281305
ENSG00000139985	ADAM21	0.858593391	0.789422857	2.884980633	0.299323387	3.743574024	2	2	2	2.498691189	2.59489879	3.534812207	3.518055793
ENSG00000101577	LPIN2	1.658962035	0.085333416	2.084170059	0.024699035	3.743132094	7.33131396	6.78762695	9.180874355	9.78107609	9.79633147	10.0712216	10.34074271
ENSG00000165376	CLDN2	0.689488183	0.831318691	3.053522525	0.26116563	3.743010708	2	2	2	2.498691189	2.49394783	3.694366604	3.580696614
ENSG00000177189	RPS6KA3	2.466136234	0.000285776	1.276169153	0.079091424	3.742305387	6.257048221	8.053983481	8.042706615	10.11451133	10.0955857	8.961264871	8.881227752
ENSG00000196458	ZNF605	1.958779011	0.466801761	1.781676375	0.501916413	3.740455386	2	8.255926468	2	8.642369674	8.62175922	8.827777334	7.95628774
ENSG00000127483	HP1BP3	1.800178115	0.007382667	1.939494076	0.003409966	3.739672191	7.82963488	9.497318007	9.242516611	10.82879697	10.7980539	10.71020131	11.15995768
ENSG00000175970	UNC119B	1.549561175	0.454376509	2.189638428	0.263585743	3.739199602	5.095959458	8.57407404	2	8.657201865	8.65574839	9.379582949	9.197482551
ENSG00000187105	HEATR4	1.014440637	0.768549381	2.724111321	0.376042598	3.738551958	2	2	2	2.784612485	2.38539787	3.796405078	2.918126211
ENSG00000178188	SH2B1	1.580223507	0.169907773	2.157615809	0.048821317	3.737839317	4.467311984	8.004992147	7.552162999	8.829471715	8.85589616	9.444166158	9.387230438
ENSG00000172869	DMXL1	1.512620296	0.374656805	2.223616413	0.166829592	3.736236709	2	7.819099893	7.552162999	8.575233246	8.64254348	9.439386236	9.177777331
ENSG00000153933	DGKE	2.285123542	0.003922264	1.451094162	0.079481787	3.736217704	4.815609471	4.442710129	6.322058544	7.620871236	7.52080763	6.730562686	6.791281771
ENSG00000157214	STEAP2	4.7391478	2.42E-10	-1.005787395	0.250703287	3.733360405	4.00722531	5.457120953	5.59507254	9.777686595	9.74782835	4.720468739	3.789585079
ENSG00000100003	SEC14L2	2.397497345	0.12454222	1.333805739	0.41298492	3.731303084	2	5.72254323	7.342949392	8.496596021	8.53707846	7.17948788	7.712342256
ENSG00000241322	CDRT1	3.974453865	0.103494474	-0.243155574	0.935446753	3.73129829	2	2	2	4.268917409	4.37434574	2.183373728	2.374681325
ENSG00000197951	ZNF71	1.483526004	0.536732782	2.246924464	0.323301531	3.730450468	2	6.951736442	2	6.755670824	6.98285659	7.815031728	7.386797235
ENSG00000131080	EDA2R	1.887929307	0.181069609	1.841768587	0.184486831	3.729697894	6.816637977	6.140560021	2	7.794595599	7.78127547	7.954782214	7.493753702
ENSG00000156136	DCK	1.541469513	0.249182594	2.18811344	0.084474028	3.729582953	6.257048221	6.228547981	2	7.169761359	7.18258783	8.039715588	7.532563185
ENSG00000165323	FAT3	0.922394504	0.461270807	2.80672631	0.009915802	3.729120814	4.00722531	3.686303414	5.59507254	5.433909581	5.48637102	7.803699407	6.323497254
ENSG00000163605	PPP4R2	2.320180052	0.007747348	1.408820019	0.122622819	3.729000071	8.631580145	6.464272742	6.80318718	9.968150831	9.88434767	9.086680922	8.951552775
ENSG00000197355	UAP1L1	1.770925407	0.18204098	1.953856671	0.129621144	3.724782079	7.435667355	7.60566235	3.369317972	8.689305661	8.75588792	8.556847345	9.184095521
ENSG00000147133	TAF1	1.967161568	0.000112837	1.753806639	0.000619607	3.720968208	7.384433967	6.228547981	6.629488387	8.744518279	8.7394436	8.610361892	8.446458576
ENSG00000112146	FBXO9	2.091831983	0.003639034	1.628657631	0.027126817	3.720489613	8.009475818	6.844429755	6.43197674	9.283050899	9.34864288	8.380906995	9.213631806
ENSG00000170921	TANC2	1.917391453	0.00063387	1.800230824	0.001356792	3.717622277	7.624139768	7.051611315	8.408111533	9.675758322	9.70259467	9.691228581	9.443440338
ENSG00000188783	PRELP	-1.086671818	0.266515549	4.801576717	2.64E-09	3.714904899	4.00722531	4.11352228	5.930865648	3.997744386	4.0930011	9.199402547	9.81909102
ENSG00000081818	PCDHB4	2.373737522	0.304276217	1.340113903	0.574460139	3.713851425	2	3.076665209	2	4.105493497	3.50452879	3.500668998	2.746601469
ENSG00000144357	UBR3	1.748715457	0.326774611	1.965053587	0.257062588	3.713769043	2	8.451443573	7.398165132	9.142549916	9.20532695	9.443042894	9.333296562
ENSG00000205423	CNEP1R1	1.913173316	0.009852067	1.799979832	0.015286757	3.713153147	5.095959458	6.666836837	5.156626401	7.72026832	7.7127321	7.692773977	7.512619696
ENSG00000064115	TM7SF3	1.836578556	0.068005105	1.876483139	0.058167426	3.713061695	5.330594134	8.356994601	7.858115942	9.484432763	9.44644299	9.457299408	9.551644037
ENSG00000137145	DENND4C	2.078286051	0.010174598	1.633162686	0.047681033	3.711448736	8.009475818	8.077869079	5.930865648	9.689531608	9.66898051	9.470865405	8.9569744
ENSG00000164327	RICTOR	3.234725717	3.55E-05	0.475667395	0.614612732	3.710393112	6.89194353	9.04308532	7.777116083	11.41826296	11.375853	8.91056191	8.332767588
ENSG00000102265	TIMP1	3.888523253	2.36E-18	-0.180553879	0.755865023	3.707969374	12.59833413	11.82700743	11.61331269	15.9367421	15.9921748	12.01740554	11.76576038
ENSG00000143514	TP53BP2	1.527643867	0.375347947	2.17934904	0.18139849	3.706992907	6.009025997	9.640525262	4.523675391	9.713145491	9.73682479	10.19415235	10.53433941
ENSG00000198952	SMG5	1.863739276	0.056662136	1.842773415	0.056553913	3.706512691	6.257048221	8.732943944	9.083172337	10.26733709	10.3236737	10.48587226	10.02762959
ENSG00000136448	NMT1	1.840016501	0.129733292	1.865588719	0.117956298	3.70560522	5.532353208	9.461450882	8.742726175	10.41121192	10.4901834	10.51623025	10.43611728
ENSG00000267508	ZNF285	1.735073995	0.361413515	1.968494242	0.284195618	3.703568238	2	4.93638933	2	5.304221918	5.00774437	5.798032389	4.766376438
ENSG00000101298	SNPH	1.840318881	0.510757053	1.861506566	0.49597846	3.701825446	2	2	8.434763955	8.658676732	8.70880006	8.90526987	8.472158355
ENSG00000177733	HNRNPA0	1.361340924	0.299725694	2.339273983	0.054822741	3.700614906	9.423197387	7.819099893	5.39244166	9.645581273	9.68051177	10.39846491	10.84263274
ENSG00000133641	C12orf29	1.821788504	0.292677052	1.878365578	0.266744705	3.700154082	2	6.78762695	4.058998049	7.218576229	7.18663697	7.535143176	6.913939481
ENSG00000149930	TAOK2	1.616757055	0.013230416	2.08314964	0.000947011	3.699906665	9.048471759	7.819099893	7.552162999	9.905552176	9.88994604	10.33251275	10.39185883
ENSG00000106829	TLE4	2.155983083	0.061614336	1.541996537	0.190242338	3.69797962	5.70932728	9.386925327	8.721235202	10.69000523	10.7526855	9.791055745	10.37022608
ENSG00000104967	NOVA2	2.33872267	0.514344567	1.358883852	NA	3.697606522	2	2	2	3.663521627	2.49394783	3.035909414	2.326754077
ENSG00000104643	MTMR9	1.79615764	0.016930176	1.900539541	0.010456421	3.696697181	5.532353208	7.572420273	6.629488387	8.561107327	8.60672783	8.700544163	8.674186858
ENSG00000112079	STK38	1.15297535	0.016667868	2.540488138	9.78E-09	3.693463488	7.667647223	8.655694736	8.007629717	9.281137113	9.36213202	10.81616151	10.58264661
ENSG00000186976	EFCAB6	1.49903845	0.649972951	2.194409952	NA	3.693448402	2	2	2	2.947924294	2.59489879	3.336159116	2.851942824
ENSG00000100241	SBF1	1.156366766	0.42327046	2.537018043	0.052381201	3.693384809	5.095959458	9.549500232	9.61300511	10.18170646	10.1772163	11.69314688	11.4067386
ENSG00000072803	FBXW11	1.480642584	0.001734936	2.211223514	1.02E-06	3.691866098	8.340423424	7.538394143	8.561105505	9.667723798	9.66608324	10.51369123	10.26488154
ENSG00000163001	CFAP36	2.423002447	0.001762985	1.26879576	0.124086277	3.691798207	4.815609471	7.051611315	6.073378139	8.619831492	8.65136017	7.508544306	7.489678691
ENSG00000108797	CNTNAP1	1.214402292	0.077982082	2.473789388	8.37E-05	3.68819168	7.904282243	8.191702463	6.629488387	8.873322599	8.97305476	9.835606948	10.45208674
ENSG00000066629	EML1	2.729435543	0.173153636	0.956782814	0.661209052	3.686218357	4.00722531	8.124485672	2	9.306761457	9.35225233	7.718996793	7.422862719
ENSG00000101190	TCFL5	1.526260006	0.379713551	2.159789054	0.189077353	3.68604906	2	7.051611315	4.523675391	7.222570549	7.19871665	8.018757917	7.613439639
ENSG00000166949	SMAD3	1.735980908	0.002281839	1.947918984	0.000492889	3.683899891	8.39321512	9.424669273	8.236946975	10.48206602	10.5585666	10.53044605	10.90923487
ENSG00000196365	LONP1	1.619628388	0.100882434	2.062842234	0.029695601	3.682470622	5.866944937	8.747909548	8.175061096	9.59332559	9.65954302	9.999109217	10.13393228
ENSG00000257446	ZNF878	3.049542221	0.304185458	0.632569292	0.849613217	3.682111513	2	2	2	3.921164147	3.28037825	2.656294034	2.277179783
ENSG00000145936	KCNMB1	-3.753147707	0.220358877	7.434961295	0.000837372	3.681813588	2	2	2	2	2	7.900986387	6.92404095
ENSG00000180044	C3orf80	7.447651245	6.02E-06	-3.765866893	0.128150716	3.681784352	2	2	2	7.507583254	7.50468064	2.048074405	2
ENSG00000113716	HMGXB3	1.787325627	0.194794952	1.893984078	0.1588785	3.681309704	6.257048221	6.311476754	9.658943166	10.11128345	10.0874876	10.15284024	10.25671168
ENSG00000158125	XDH	2.900428658	0.097131191	0.777498999	0.690278517	3.677927657	2	2	4.058998049	5.128039203	4.91316568	3.296906311	3.496556178
ENSG00000089159	PXN	2.142418081	0.017649875	1.533800942	0.098356149	3.676219023	7.384433967	9.267380431	9.990713521	11.33238675	11.395009	10.43804609	11.01733363
ENSG00000130787	HIP1R	2.155472997	0.140659286	1.517396707	0.307557173	3.672869704	5.532353208	6.951736442	2	7.855024645	8.05152279	7.328045423	7.334741191
ENSG00000174780	SRP72	1.771216099	0.129861227	1.900379863	0.096243075	3.671595962	8.653385977	8.687092496	5.156626401	9.919767943	9.89428535	10.12526871	9.940248792
ENSG00000166233	ARIH1	1.821153328	2.75E-07	1.845662927	1.64E-07	3.666816255	8.19931886	8.147241016	8.325066185	10.00803782	10.0529704	10.29829283	9.76223254
ENSG00000113441	LNPEP	1.630284279	0.196836305	2.036162154	0.093560552	3.666446433	8.816894989	7.232742069	4.874587708	9.342071312	9.31111579	9.597165068	9.852266467
ENSG00000115556	PLCD4	1.496206818	0.448944973	2.168359625	0.244375349	3.664566443	2	5.457120953	2	5.304221918	5.5755357	6.221381643	5.896817464
ENSG00000073711	PPP2R3A	2.060652454	0.182837442	1.602644941	0.303025886	3.663297394	2	7.393647515	6.80318718	8.615281294	8.55284928	8.05302937	8.206864727
ENSG00000180537	RNF182	-0.028813158	0.98413673	3.691899043	0.001006265	3.663085885	3.327711234	5.131603625	2	4.205751501	3.98554648	7.272216802	7.644317657
ENSG00000167460	TPM4	1.206034897	0.004834581	2.456909105	8.26E-10	3.662944002	12.64261488	11.84277663	11.90062666	13.37147027	13.3921961	14.59460754	14.66928705
ENSG00000215251	FASTKD5	1.862225852	0.200007734	1.798302171	0.209322464	3.660528022	5.532353208	7.819099893	3.369317972	8.381625466	8.39906692	8.125537812	8.504157459
ENSG00000172264	MACROD2	0.689488151	0.832844524	2.970138428	0.281038046	3.659626579	2	2	2	2.498691189	2.49394783	3.584558147	3.580696614
ENSG00000119820	YIPF4	1.622515454	0.065635381	2.036946638	0.016735344	3.659462092	5.866944937	7.847167909	8.325066185	9.272493518	9.26686067	9.876121046	9.457070207
ENSG00000171503	ETFDH	1.508135773	0.323937413	2.150785495	0.137340944	3.658921267	2	7.099070191	6.534109959	7.668500375	7.83127384	8.344674564	8.424568162
ENSG00000177374	HIC1	0.769789225	0.633071268	2.885857858	0.038931255	3.655647083	6.816637977	7.099070191	2	7.035434774	7.26148706	9.04915428	9.403927624
ENSG00000196743	GM2A	0.837689681	0.467543151	2.816272135	0.004738564	3.653961815	5.532353208	8.57407404	7.029880454	8.343618883	8.40255255	10.51235311	10.14174401
ENSG00000172469	MANEA	1.80175155	0.007359418	1.852070346	0.005357536	3.653821896	4.815609471	5.838937808	6.629488387	7.742693204	7.5835711	7.799317009	7.62463201
ENSG00000198466	ZNF587	2.665358069	3.56E-08	0.987008852	0.06346313	3.652366921	7.33131396	6.464272742	7.451345288	9.764733396	9.74919792	8.296356225	7.856442979
ENSG00000114331	ACAP2	2.139193	1.25E-07	1.512471335	0.000268289	3.651664335	7.218819781	7.761270542	7.858115942	9.78445764	9.73129138	9.347224935	8.885886446
ENSG00000162413	KLHL21	2.116078209	0.265702905	1.535566072	0.426645605	3.651644281	9.410448395	8.213429689	2	10.44065847	10.4716357	9.95467636	9.796450865
ENSG00000139651	ZNF740	1.850650111	0.012834002	1.798707399	0.015373036	3.64935751	5.330594134	7.315436877	7.225742779	8.598473365	8.76809931	8.704768045	8.560285
ENSG00000158480	SPATA2	1.690286678	0.221271682	1.957722473	0.143141086	3.648009151	6.138360429	6.666836837	2	7.530319349	7.4949172	7.774526492	7.775072826
ENSG00000023892	DEF6	1.957355793	0.303980957	1.690643961	0.369090445	3.647999754	2	3.686303414	2	3.95996233	4.2560182	4.029788235	3.789585079
ENSG00000018510	AGPS	2.260856807	0.017769551	1.387112855	0.163224785	3.647969662	7.667647223	8.687092496	5.772716771	10.12041043	9.98077219	9.234884382	9.129269498
ENSG00000080561	MID2	2.654866322	0.073563931	0.989946442	0.538569152	3.644812764	6.653098118	7.431218269	2	9.18513223	9.09655277	7.528804029	7.467748741
ENSG00000198585	NUDT16	1.467258145	0.218891141	2.177468171	0.053220172	3.644726316	4.00722531	7.538394143	7.451345288	8.427334995	8.40429221	8.889688585	9.31767336
ENSG00000120279	MYCT1	3.219183929	0.096972333	0.425506341	0.852507508	3.64469027	3.327711234	2	2	4.443344104	4.53541753	2.831336763	2.632958241
ENSG00000102572	STK24	2.351724385	7.58E-05	1.291756847	0.040597375	3.643481232	7.27616306	8.747909548	7.734844356	10.37936391	10.4205473	9.330921459	9.358468538
ENSG00000145730	PAM	1.82970223	1.73E-09	1.811736057	2.29E-09	3.641438287	10.43795805	10.64091981	10.5677762	12.38192656	12.3758755	12.07985022	12.59614149
ENSG00000123106	CCDC91	2.380127198	0.014133546	1.260842454	0.220251675	3.640969652	6.009025997	6.535001748	3.369317972	8.14748175	8.09955907	6.931352719	7.119550903
ENSG00000149782	PLCB3	1.549283729	0.108536572	2.090825845	0.023427954	3.640109574	7.218819781	7.701025996	4.874587708	8.568972129	8.51148098	9.033940582	9.119193255
ENSG00000110906	KCTD10	1.250499998	0.057132864	2.3895414	7.98E-05	3.640041398	9.473095744	8.469613413	8.007629717	10.05303338	9.99815569	10.9216389	11.36776693
ENSG00000187123	LYPD6	4.838985764	0.019426473	-1.199808284	0.630693854	3.63917748	2	2	2	5.022023783	5.07918227	2.183373728	2.117335275
ENSG00000117528	ABCD3	1.715816763	0.233103112	1.922797554	0.169025837	3.638614317	4.00722531	7.819099893	4.874587708	8.27796118	8.09094333	8.454100442	8.326688609
ENSG00000178409	BEND3	1.525993675	0.174897897	2.111241589	0.047215475	3.637235264	2	4.11352228	5.156626401	5.602782483	5.47317115	6.025180246	6.13807518

ENSG00000082701	GSK3B	1.263642183	0.484301277	2.372755616	0.153698217	3.636397798	9.710019321	7.431218269	3.369317972	9.6388717	9.68695783	10.69648025	10.83689669
ENSG00000222014	RAB6C	8.82662083	1.71E-08	-5.19115099	0.029583545	3.63546984	2	2	2	8.902248225	8.82765235	2	2
ENSG00000137802	MAPKBP1	1.818732797	0.105302052	1.813423473	0.100794035	3.632156271	7.940205782	5.946638857	4.874587708	8.514626115	8.65574839	8.714575941	8.435201779
ENSG00000173457	PPP1R14B	1.90504113	0.000144567	1.725298382	0.000614839	3.630339512	7.031698319	8.101365653	7.451345288	9.44052423	9.5427783	9.099926561	9.500245276
ENSG00000151466	SCLT1	2.3318898	0.003882515	1.298012847	0.128417272	3.629902647	3.327711234	5.457120953	4.874587708	7.026323566	6.97351258	6.181526565	5.821002687
ENSG00000124795	DEK	2.038818495	0.007885856	1.587730044	0.04279221	3.626548539	7.485143553	8.981105197	7.163363831	10.17913955	10.1014956	9.871536104	9.485996671
ENSG00000147065	MSN	1.324220322	0.215468947	2.30221188	0.02080403	3.626432202	8.946185348	11.98850994	11.18913795	12.4744381	12.5033925	13.13119617	13.73835164
ENSG00000008256	CYTH3	0.324063107	0.850996577	3.301787113	0.016654612	3.62585022	9.33149386	7.954278496	4.058998049	8.570539957	8.55598286	11.55739329	11.49721959
ENSG00000130475	FCHO1	-0.286492525	0.864318577	3.909727128	0.001909905	3.623234604	2	4.11352228	3.369317972	3.349643765	3.2184422	6.046045499	7.255358547
ENSG00000089916	GPATCH2L	1.657301695	0.159990977	1.963673113	0.084642081	3.620974808	5.095959458	8.356994601	8.699419242	9.678668908	9.64342987	10.16089043	9.74096227
ENSG00000198862	LTN1	1.968779097	0.006540456	1.652042769	0.024545065	3.620821866	7.159102577	8.213429689	6.534109959	9.439666599	9.41917001	9.440512349	8.693212171
ENSG00000198689	SLC9A6	1.5218079	0.374672148	2.09884885	0.197276691	3.620656749	2	7.73146271	6.073378139	8.049698831	8.06258142	8.517369639	8.727068782
ENSG00000131023	LATS1	1.546168424	0.05186708	2.074281655	0.006426257	3.620450079	7.624139768	7.901717006	5.772716771	8.883449599	8.89230135	9.558530648	9.249420371
ENSG00000144026	ZNF514	1.974743127	0.210990211	1.645208262	0.296894493	3.619951389	2	6.666836837	4.523675391	7.391747748	7.24992376	7.238947338	6.718860807
ENSG00000141034	GID4	2.042083539	0.226514714	1.577806122	0.35456714	3.619889661	2	6.535001748	7.97167868	8.85414072	8.86223882	8.60534994	8.157201821
ENSG00000003400	CASP10	1.519550726	0.116925716	2.100067596	0.022260314	3.619618322	3.327711234	3.686303414	4.874587708	5.406080789	5.3193943	5.912830251	5.88031069
ENSG00000145545	SRD5A1	1.408415344	0.415078879	2.210817026	0.171451798	3.619232371	7.667647223	5.72254323	2	7.781117077	7.82349365	8.695364791	8.484497333
ENSG00000179046	TRIML2	3.148890295	0.298769516	0.469759838	0.89230957	3.618650134	2	2	2	3.709785322	3.69849104	2.656294034	2.172608095
ENSG00000263141		2.487016298	0.002227636	1.129720929	0.198274928	3.616737227	6.653098118	5.946638857	4.523675391	8.406418094	8.31463236	7.407359556	6.520103598
ENSG00000154760	SLFN13	1.737220298	0.044788115	1.879172045	0.026212112	3.616392344	4.00722531	3.686303414	4.874587708	5.720439317	5.76075522	5.805044075	5.937276556
ENSG00000205238	SPDYE2	3.937391889	0.098417871	-0.321416484	0.912368677	3.615975405	2	2	2	4.299492648	4.2865223	2.3070652	2.225841198
ENSG00000168792	ABHD15	1.436859392	0.049492187	2.178432884	0.001567678	3.615292276	4.467311984	6.311476754	6.43197674	7.352188525	7.36158648	8.131128273	8.035760639
ENSG00000135740	SLC9A5	1.149423383	0.496953626	2.462986632	0.109152121	3.612410014	4.00722531	6.535001748	2	6.306580752	6.35727082	7.512833218	7.68646583
ENSG00000162267	ITIH3	-2.308256067	0.472536264	5.920309442	0.009193782	3.612053375	2	2	2	2.141737246	2	6.437578914	5.481238982
ENSG00000171793	CTPS1	0.435262512	0.760337788	3.176026944	0.006625005	3.611289456	5.330594134	8.557180776	9.385998894	8.951212326	8.90955283	11.97242248	11.26382758
ENSG00000158483	FAM86C1	2.429177637	0.007711612	1.181554901	0.226109663	3.610732539	3.327711234	4.93638933	4.058998049	6.522845756	6.44101097	4.996409624	5.586744392
ENSG00000144136	SLC20A1	3.159733509	0.001783365	0.449217874	0.712785235	3.608951382	8.418903792	9.656475478	6.43197674	11.81320634	11.8618308	8.804350194	9.405370462
ENSG00000065361	ERBB3	-1.930947148	0.033219225	5.538848946	4.09E-13	3.607901798	4.815609471	3.686303414	5.39244166	3.566290607	3.01487888	9.756885121	10.29516161
ENSG00000198610	AKR1C4	8.798497025	6.32E-08	-5.191150239	0.032903971	3.607346786	2	2	2	8.567402595	9.06500642	2	2
ENSG00000125954	CHURC1-FNT	8.797582656	2.20E-08	-5.191150929	0.029960917	3.606431726	2	2	2	8.772033429	8.8984863	2	2
ENSG00000164185	ZNF474	2.334272268	0.460540532	1.271854422	NA	3.60612669	2	2	2	3.289987342	3.08597292	2.885263063	2.466008509
ENSG00000198467	TPM2	1.443154361	0.011803936	2.162852611	7.38E-05	3.606006973	11.78357814	10.40638494	11.12511995	12.63421185	12.6707361	13.44241619	13.29782891
ENSG00000177330	CASKIN2	0.693507541	0.697239244	2.9094224	0.057634332	3.602929941	6.468621203	7.761270542	2	7.281195074	7.45518898	9.698306977	9.405009888
ENSG00000130695	CEP85	0.87580611	0.35418186	2.725201425	0.00089394	3.601007535	4.815609471	6.228547981	4.058998049	6.268449691	6.08962627	8.054501123	7.885682058
ENSG00000128617	OPN1SW	-3.753148157	0.2198682	7.351342842	0.000951235	3.598194685	2	2	2	2	2	6.937746858	7.768360547
ENSG00000181072	CHRM2	2.557206839	0.058610905	1.040138536	0.476093865	3.597345375	2	5.303523747	6.629488387	7.944803394	8.08010078	6.508020621	6.567271433
ENSG00000142207	URB1	1.435864604	0.191858509	2.16035339	0.037170481	3.596217993	4.815609471	8.053983481	6.958202216	8.485003881	8.60067114	9.393314624	9.118313725
ENSG00000101463	SYNDIG1	-1.27004269	0.581333492	4.86590097	0.006504795	3.59585828	2	3.076665209	2	2.3892346	2.14022021	5.630526941	6.212491471
ENSG00000164050	PLXNB1	0.797991071	0.552458672	2.796722062	0.017084929	3.594713133	8.418903792	6.228547981	4.874587708	8.022463952	7.96929529	9.729736885	10.18942242
ENSG00000182405	PGBD4	1.16067629	0.540424469	2.432237172	0.160349824	3.592913461	2	5.131603625	2	4.76150579	4.93258395	6.197600957	5.768151538
ENSG00000171729	TMEM51	2.988601398	0.027435278	0.603198919	0.700098898	3.591800317	6.563806279	6.951736442	2	9.104095335	9.18016239	6.931352719	6.654794245
ENSG00000119862	LGALSL	-0.060544662	0.976835952	3.652094703	0.025542054	3.591550041	2	5.457120953	2	4.070463721	4.1930041	7.414254101	7.589522245
ENSG00000112137	PHACTR1	2.719878773	0.149717516	0.871460752	0.674724562	3.591339525	2	2	4.523675391	5.474671438	5.37722731	4.301748724	3.311948089
ENSG00000143768	LEFTY2	2.784455792	0.479814084	0.806315915	0.850336885	3.590771707	2	2	2	4.139692846	2.14022021	2.746468522	2.277179783
ENSG00000085832	EPS15	1.661896915	3.46E-07	1.928557735	1.92E-09	3.59045465	8.107517749	7.790474954	7.552162999	9.545497589	9.40967456	9.728815405	9.759978595
ENSG00000165671	NSD1	1.418222696	0.349265407	2.172103282	0.127258741	3.590325978	9.699577239	9.34816733	4.523675391	10.38605714	10.3813388	11.12215672	11.14934098
ENSG00000132405	TBC1D14	1.89672453	0.172055101	1.692225082	0.22004974	3.588949612	5.330594134	7.538394143	3.369317972	8.160032699	8.16669309	7.966556098	7.957271773
ENSG00000142303	ADAMTS10	0.521443173	0.84416062	3.06744605	0.170183679	3.588889223	2	7.189546432	2	6.069927018	6.29111322	8.515767839	8.797441501
ENSG00000182827	ACBD3	1.713018645	0.232251697	1.875200015	0.179198641	3.58821866	4.467311984	9.357954881	7.934808822	9.985884116	9.95192082	10.17110242	10.08884856
ENSG00000188092	GPR89B	3.60855708	0.002803895	-0.020950021	0.989200675	3.587607059	2	5.838937808	4.058998049	8.126317766	8.17282964	4.894909044	4.553838275
ENSG00000080493	SLC4A4	3.30322085	0.013507859	0.283442403	0.86358453	3.586663253	3.327711234	5.946638857	3.369317972	7.97352961	7.88209639	5.654016955	4.062589736
ENSG00000170340	B3GNT2	2.165574599	0.002318605	1.420056838	0.055057455	3.585631437	5.70932728	7.572420273	7.552162999	9.350309099	9.25726695	8.692059118	8.425990514
ENSG00000101882	NKAP	1.739945689	0.10022545	1.844828809	0.074545495	3.584774498	4.467311984	6.728495673	4.523675391	7.300218887	7.40393901	7.674704537	7.196571833
ENSG00000065883	CDK13	1.598528014	0.026712193	1.985956285	0.004489235	3.584484298	8.313285031	8.077869079	6.43197674	9.382797773	9.41486164	9.910864109	9.645596027
ENSG00000116922	C1orf109	1.423906438	0.251883859	2.15891793	0.064830738	3.582824368	3.327711234	6.78762695	5.156626401	7.144720234	7.02417148	8.038228667	7.523290521
ENSG00000116698	SMG7	2.035976112	0.280396747	1.546737187	0.416362316	3.582713299	2	8.505280662	9.180874355	10.31908469	10.3319235	9.770158073	9.902941748
ENSG00000153487	ING1	1.75759886	0.254248233	1.820529461	0.227580218	3.578128322	4.00722531	7.232742069	3.369317972	7.587189552	7.60187978	7.852739912	7.428550441
ENSG00000135956	TMEM127	1.609653759	0.101754679	1.968177296	0.037955813	3.577831055	9.590776211	8.968383114	6.718950348	10.41033668	10.4786707	10.7701007	10.83422097
ENSG00000117000	RLF	1.677345608	0.355050514	1.899847329	0.279798466	3.577192937	2	6.666836837	8.35328246	8.772033429	8.86476809	9.24845059	8.795792144
ENSG00000155592	ZKSCAN2	1.649013296	0.391405869	1.926744449	0.298395371	3.575757744	3.327711234	6.899080544	2	7.119236776	6.99676009	7.379447089	7.277588383
ENSG00000171044	XKR6	2.247500462	0.181478081	1.328182096	0.444850169	3.575682558	2	4.442710129	2	5.161714397	5.13053105	4.604690117	4.077147165
ENSG00000183773	AIFM3	2.083656679	0.51121293	1.491414887	NA	3.575071566	2	2	2	3.227757282	2.86123014	2.775316226	2.782576693
ENSG00000175832	ETV4	1.81056548	0.195586526	1.763992303	0.199377759	3.574557783	6.138360429	3.076665209	3.369317972	6.535745081	6.53293476	6.508020621	6.471341372
ENSG00000133985	TTC9	-3.112005397	0.0683337	6.68578516	3.78E-06	3.573779762	2	5.457120953	2	2.498691189	2.49394783	9.931034903	10.93343876
ENSG00000087088	BAX	1.568217745	0.000812241	2.004849116	1.05E-05	3.573066861	8.285626337	7.274681896	8.175061096	9.50179306	9.5348592	10.00444935	9.899618215
ENSG00000168300	PCMTD1	1.956511459	7.94E-06	1.614124309	0.000274788	3.570635768	7.709880939	7.467835385	7.342949392	9.52946469	9.36034073	9.40720485	8.727645605
ENSG00000111321	LTBR	1.864222396	0.00491763	1.706081977	0.010511932	3.570304372	8.653385977	8.147241016	9.778213818	10.8531554	10.9026799	10.67176032	10.76734655
ENSG00000176022	B3GALT6	1.152490167	0.599989824	2.416914567	0.23044932	3.569404733	2	8.487557258	4.523675391	8.130575473	8.15434112	9.184693023	9.578432478
ENSG00000130227	XPO7	1.716068347	0.348810364	1.852887971	0.298467142	3.568956319	8.777722034	7.928237109	2	9.510803856	9.5631668	9.74508482	9.597808016
ENSG00000124313	IQSEC2	1.742383027	0.09775828	1.826469724	0.076006128	3.568852751	4.467311984	6.78762695	7.691296561	8.476666381	8.57154947	8.609360894	8.606804877
ENSG00000080845	DLGAP4	0.917484935	0.020792238	2.650779505	1.53E-13	3.56826444	9.657036304	9.883504052	9.25752426	10.52434207	10.5542639	12.03167326	12.47443408
ENSG00000172318	B3GALT1	2.646995536	0.41861794	0.921035823	0.79387618	3.568031359	2	2	2	3.709785322	2.94009902	2.267001569	2.817676617
ENSG00000161638	ITGA5	3.137959036	5.38E-09	0.429444685	0.509227321	3.567403721	10.65885801	11.24986486	12.02204252	14.51866036	14.5886674	11.71646058	11.96652151
ENSG00000102349	KLF8	0.430925973	0.738128035	3.134702412	0.002597654	3.565628385	3.327711234	3.076665209	5.39244166	4.54891529	4.70309173	7.199581219	7.034460028
ENSG00000163872	YEATS2	1.848510128	0.000223501	1.716911308	0.000631256	3.565421435	8.564112515	8.101365653	8.511883463	10.23161272	10.2467381	10.51569609	9.537567589
ENSG00000185658	BRWD1	1.860261666	0.000587551	1.704396993	0.001715093	3.564658658	8.009475818	8.395519306	7.163363831	9.80724435	9.78235238	9.867355293	9.369229141
ENSG00000158555	GDPD5	0.100689587	0.968286211	3.462555513	0.094151017	3.5632451	2	6.728495673	2	5.273747807	5.44640346	8.65615757	8.524881346
ENSG00000116584	ARHGEF2	1.687504616	0.20047706	1.874934596	0.143348036	3.562439212	5.532353208	9.640525262	8.076950877	10.2141657	10.2213052	10.76696209	9.919445528
ENSG00000126804	ZBTB1	2.226823746	2.38E-07	1.335522894	0.003033425	3.56234664	7.218819781	8.124485672	7.691296561	9.929575199	9.95370391	9.213963607	8.877074023
ENSG00000150760	DOCK1	1.791687544	0.02516631	1.770604013	0.026185977	3.562291557	6.653098118	8.791896851	7.691296561	9.722342833	9.77900397	9.576313249	9.868833922
ENSG00000047634	SCML1	1.988743476	0.264475702	1.572879709	0.379145564	3.561623184	4.00722531	7.051611315	2	7.574744049	7.61696151	7.401588825	6.942047249
ENSG00000270617	URGCP-MRP	8.750868743	2.79E-08	-5.191150933	0.03010975	3.559717811	2	2	2	8.804373539	8.77618319	2	2
ENSG00000169507	SLC38A11	-3.753134638	0.262397112	7.312837345	0.004028686	3.559702707	2	2	2	2	2	7.98365249	6.298867018
ENSG00000152749	GPR180	2.05487971	0.077163483	1.503129971	0.203570906	3.55800968	6.009025997	7.954278496	4.874587708	8.893506006	8.83412012	8.742236593	7.714671806
ENSG00000137200	CMTR1	2.551144078	4.99E-06	1.005355732	0.100850653	3.55649981	8.540903468	7.901717006	7.029880454	10.48414712	10.4827929	8.90036762	8.989083133
ENSG00000121903	ZSCAN20	1.962307352	0.362261257	1.591563843	0.460972389	3.553871195	2	6.140560021	2	6.687949235	6.37156942	6.482006365	5.812327524
ENSG00000185245	GP1BA	-1.310505255	0.645571902	4.863660437	0.023508946	3.553155182	2	2	2	2.141737246	2.14022021	4.990270811	5.150533729
ENSG00000188486	H2AFX	1.507510665	0.159032929	2.045467834	0.044582925	3.552978499	4.815609471	6.140560021	7.777116083	8.221200057	8.16053033	8.656642182	8.786410001
ENSG00000126001	CEP250	1.894275223	0.253228122	1.658252604	0.314385216	3.552527827	7.867441304	7.572420273	2	9.018234236	9.0282492	8.684949926	8.886403151
ENSG00000109189	USP46	1.981382358	0.001547707	1.570032761	0.014055845	3.551415119	5.70932728	6.666836837	5.156626401	7.971157485	7.89452713	7.541454591	7.517964974
ENSG00000183891	TTC32	1.84417828	0.153618856	1.706702241	0.179539773	3.550880521	3.327711234	4.11352228	2	4.867343068	4.87352701	4.68289886	4.819291477
ENSG00000069275	NUCKS1	1.142590798	0.036736378	2.407080914	1.61E-06	3.549671711	9.143983763	10.15453315	9.116480286	10.71325662	10.6873877	11.84730078	12.06982099
ENSG00000165661	QSOX2	1.441691014	0.306881895	2.107831747	0.113226946	3.549522761	4.467311984	5.303523747	8.143093325	8.151677543	8.32935768	8.788075779	9.00820079
ENSG00000120784	ZFP30	1.768868496	0.38484122	1.780638023	0.370592016	3.549506519	3.327711234	2	6.958202216	7.330144653	7.03319408	7.470462265	6.868633039
ENSG00000092148	HECTD1	1.472197762	0.132505099	2.076664377	0.025734513	3.54886214	7.53297911	10.13758676	8.561105505	10.62321188	10.5902432	11.27988996	11.13658383
ENSG00000110047	EHD1	0.798758374	0.243302388	2.748880971	3.58E-06	3.547639345	8.444143041	8.88960062	9.839340503	9.896204982	10.0423963	11.59984264	12.17686777
ENSG00000137273	FOXF2	0.852945632	0.626071633	2.694123412	0.080856643	3.547069044	2	5.131603625	7.225742779	6.733448311	6.82036165	8.705705008	8.442950253
ENSG00000173041	ZNF680	2.424471092	0.054569925	1.119638228	0.405601197	3.54410932	2	4.11352228	5.59507254	6.777556219	6.65485813	5.801542492	5.094391891
ENSG00000143952	VPS54	1.87136267	0.064736855	1.670881175	0.098450928	3.542243845	4.467311984	7.503546079	6.43197674	8.415170354	8.46222608	8.278834759	8.20189164
ENSG00000100121	GGTLC2	2.386858132	0.457964922	1.155338379	NA	3.54219651	2	2	2	2.868578001	3.50452879	2.457042231	2.817676617
ENSG00000126767	ELK1	1.797143361	0.252106496	1.7426535	0.259868009	3.539796862	9.27634997	6.951736442	4.058998049	9.732182526	9.79831747	9.405764176	9.963452459
ENSG00000132953	XPO4	1.788249823	0.290539585	1.749466527	0.291783001	3.53771635	2	7.847167909	6.43197674	8.511364632	8.48377664	8.3832514	8.531503464
ENSG00000173432	SAA1	3.957393374	0.096765227	-0.419764834	0.885335383	3.53762854	2	2	2	4.470469939	4.12711147	2.225793514	2.277179783
ENSG00000034510	TMSB10	1.92401128	0.000256078	1.610916517	0.002580504	3.534927797	12.32193393	11.1798568	11.6816684	13.70168635	13.7498842	13.10104672	13.66933964
ENSG00000183092	BEGAIN	0.188919815	0.939180462	3.345237539	0.088680604	3.534157354	2	2	3.369317972	2.784612485	2.49394783	4.340871764	4.810605986
ENSG00000146574	CCZ1B	5.944292744	1.40E-14	-2.410233427	0.00588506	3.534059317	4.467311984	4.710564154	2	9.856914282	9.70400783	2.62493832	2.851942824
ENSG00000105321	CCDC9	1.483144511	0.046523239	2.049339885	0.003865737	3.532484396	4.467311984	6.228547981	6.534109959	7.43025113	7.39346687	8.040458475	7.889811111

ENSG00000173369	C1QB	-3.753145913	0.228046677	7.284362014	0.001446259	3.531216101	2	2	2	2	2	7.728247761	6.826173076
ENSG00000182853	VMO1	0.272040666	0.940940174	3.258483055	0.283648588	3.530523721	2	2	2	2.270786764	2.49394783	4.340871764	2.851942824
ENSG00000009954	BAZ1B	1.851448643	0.000740256	1.677846031	0.002375091	3.529294674	9.544849385	8.395519306	8.486635645	10.75453239	10.7437816	10.70435357	10.43505811
ENSG00000145284	SCD5	-0.566728648	0.598764326	4.095304118	2.40E-06	3.52857547	5.532353208	6.844429755	8.236946975	6.856790872	6.55815769	11.15202309	11.47669218
ENSG00000126883	NUP214	1.87655858	0.310197758	1.650665691	0.36789886	3.527224272	3.327711234	8.687092496	4.523675391	9.062360705	9.07705362	8.828637742	8.862441066
ENSG00000160193	WDR4	1.954481436	0.33914729	1.570994813	0.443400867	3.525476249	2	5.838937808	2	6.213314212	6.26070706	5.696113106	6.037449234
ENSG00000181450	ZNF678	1.917128513	0.065154219	1.60742141	0.122910117	3.524549923	4.815609471	4.93638933	2	6.213314212	6.05460702	6.072436333	5.576534358
ENSG00000027001	MIPEP	1.876426931	0.101350025	1.647977797	0.147500239	3.524404729	2	5.457120953	4.523675391	6.358325776	6.25300433	6.063692919	6.117095472
ENSG00000074657	ZNF532	1.455802484	0.350662169	2.068261665	0.161518911	3.524064148	4.467311984	9.680074956	7.646393253	9.886796832	9.89366625	10.55872351	10.43999426
ENSG00000003249	DBNDD1	-0.765903048	0.370378063	4.287718464	5.62E-10	3.521815415	4.00722531	4.93638933	5.930865648	4.574143677	4.40247105	8.993080225	9.504290592
ENSG00000003436	TFPI	2.638889693	3.46E-23	0.882741418	0.001957141	3.521631111	8.39321512	8.777382925	8.536706852	11.23638949	11.1853847	9.478834579	9.442386533
ENSG00000162458	FBLIM1	0.663252114	0.053329277	2.857656607	2.16E-21	3.520908721	9.204317094	9.532315021	8.978369997	9.856271709	9.98484711	12.15954314	12.06229494
ENSG00000072518	MARK2	1.700620978	4.21E-05	1.815098758	9.76E-06	3.515719736	6.89194353	7.503546079	6.718950348	8.745906571	8.79619691	8.887212926	8.882264318
ENSG00000169871	TRIM56	1.28907695	0.323747566	2.22559581	0.067085237	3.51467276	9.473095744	8.147241016	5.39244166	9.685919789	9.73613428	10.81051091	10.45679112
ENSG00000101745	ANKRD12	2.220207915	2.33E-12	1.292393592	8.02E-05	3.512601507	8.138771685	8.414402626	8.042706615	10.43894286	10.4002048	9.644841182	9.32912155
ENSG00000186462	NAP1L2	2.818518668	0.352516309	0.693240494	0.836549691	3.511759162	2	2	2	3.462030052	3.50452879	2.68698273	2.277179783
ENSG00000179304	FAM156B	8.700469613	4.03E-08	-5.191150868	0.030595573	3.509318745	2	2	2	8.709369553	8.77079897	2	2
ENSG00000109790	KLHL5	1.689216353	0.002646347	1.818383686	0.001044308	3.507600039	8.586954094	9.488434476	9.935792933	11.13407655	11.1101028	11.05159418	11.42632529
ENSG00000017797	RALBP1	1.578897175	0.013238784	1.925380753	0.001882006	3.504277928	7.53297911	9.102512015	8.266922072	10.02471865	10.0084861	10.22241543	10.48912908
ENSG00000255526	NEDD8-MDP	8.695047998	4.09E-08	-5.191150874	0.030591756	3.503897124	2	2	2	8.736160353	8.73392024	2	2
ENSG00000254470	AP5B1	2.268667918	0.032248782	1.234298901	0.268896089	3.502966819	5.095959458	7.60566235	8.486635645	9.800579656	9.91273599	8.796015995	8.862966236
ENSG00000130224	LRCH2	2.379073767	0.163782083	1.123250904	0.533552261	3.502324671	2	6.389896633	3.369317972	7.292639438	7.31055061	6.332313483	5.812327524
ENSG00000177694	NAALADL2	1.034706808	0.529862889	2.465482259	0.095140371	3.500189067	6.009025997	3.686303414	2	5.709096143	5.62411716	6.537789343	7.388257299
ENSG00000198604	BAZ1A	2.591126045	0.073335148	0.908970036	0.565009493	3.500096081	3.327711234	8.639735838	7.858115942	10.31160396	10.3107463	8.732155826	8.53941009
ENSG00000186591	UBE2H	1.5043178	0.016404216	1.995527797	0.000939774	3.499845596	10.30651143	10.30378101	8.865353883	11.49993054	11.4321483	11.92739794	11.98611928
ENSG00000108587	GOSR1	1.809084169	0.005532065	1.689854471	0.00975996	3.49893864	6.816637977	7.002537973	8.296287027	9.347568393	9.27068036	9.236830239	9.144037078
ENSG00000135913	USP37	2.266103908	0.156314863	1.23279514	0.459659586	3.498899048	2	6.844429755	4.523675391	7.831679003	7.67863747	6.807643042	6.693102083
ENSG00000164828	SUN1	0.762307692	0.186180631	2.735165911	4.20E-08	3.497473603	8.836089686	9.030900487	7.777116083	9.428470756	9.3816907	11.34545729	11.39761508
ENSG00000155850	SLC26A2	1.111860517	0.530264818	2.382410079	0.138711164	3.494270596	4.467311984	8.469613413	4.058998049	8.153770873	8.11238697	9.07476376	9.651383746
ENSG00000015153	YAF2	2.165351173	0.00023626	1.328332053	0.031839501	3.493683226	6.009025997	6.666836837	5.156626401	8.254744042	8.15020018	7.44824109	7.311843322
ENSG00000163558	PRKCI	1.348954037	0.131883676	2.144699751	0.010661152	3.493653788	8.16936288	7.189546432	5.772716771	8.695066731	8.65428713	9.705116572	9.175665104
ENSG00000165055	METTL2B	1.342639847	0.00692683	2.150405926	4.55E-06	3.493045773	6.366709671	6.535001748	5.59507254	7.64488235	7.42122542	8.296978073	8.365751687
ENSG00000169777	TAS2R1	-1.30428344	0.736220139	4.794405526	0.127753231	3.490122086	2	2	2	2.270786764	2	4.406883583	5.436777225
ENSG00000152684	PELO	1.959067364	0.28945592	1.53063052	0.41000811	3.489697883	8.998234871	8.053983481	2	9.923453481	10.0102007	9.544459308	9.537567589
ENSG00000065802	ASB1	1.302293506	0.429843212	2.187143625	0.153259447	3.489437132	3.327711234	8.213429689	9.116480286	9.404936476	9.50598504	10.25526417	10.41514286
ENSG00000136367	ZFHX2	0.012452852	0.992812774	3.476568643	0.000328492	3.489021495	4.00722531	4.93638933	2	4.299492648	4.02226117	7.451596164	7.142226902
ENSG00000136643	RPS6KC1	2.275062609	0.001730605	1.213946302	0.115961049	3.489008911	8.009475818	8.213429689	6.203069917	9.982354854	9.95963168	8.977648172	8.851897333
ENSG00000168899	VAMP5	1.46734928	0.214681289	2.020969105	0.07263329	3.488318386	5.866944937	6.464272742	8.631919287	8.902248225	8.98007065	8.757226889	9.97588724
ENSG00000185220	PGBD2	1.837750374	0.150495614	1.650100078	0.191572761	3.487850452	2	4.710564154	3.369317972	5.348759107	5.34860058	5.348031117	4.990558504
ENSG00000100815	TRIP11	1.735123931	0.163554675	1.752032262	0.150827473	3.487156194	8.107517749	8.451443573	4.523675391	9.461800958	9.49378147	9.707457275	9.244995051
ENSG00000183665	TRMT12	1.500436373	0.317999975	1.986693652	0.165909543	3.487130025	4.467311984	6.389896633	2	6.682159616	6.57678965	7.138441638	7.060335194
ENSG00000196663	TECPR2	1.266336495	0.485504025	2.219609941	0.187718342	3.485946436	2	7.979858146	8.110401118	8.715051214	8.72698617	9.922396	9.358096047
ENSG00000185437	SH3BGR	3.665856255	0.141361699	-0.180655174	0.95264385	3.485201081	2	2	2	3.95996233	4.1930041	2.3070652	2.277179783
ENSG00000089060	SLC8B1	1.402862729	0.331987296	2.082206125	0.126883017	3.485068855	7.27616306	4.710564154	3.369317972	7.206526483	7.45182838	7.977459036	8.02265879
ENSG00000164808	SPIDR	1.41017389	0.24668092	2.073110285	0.071494314	3.483284175	6.138360429	7.145017454	3.369317972	7.484483106	7.69294162	8.151901655	8.331250243
ENSG00000080371	RAB21	1.754901318	0.000799417	1.728370128	0.000915639	3.483271446	7.031698319	7.819099893	8.35328246	9.594867189	9.53882419	9.577849253	9.502606422
ENSG00000157014	TATDN2	2.244725709	0.139433008	1.238162522	0.434687247	3.482888231	3.327711234	6.464272742	8.511888463	9.482768454	9.48147379	8.232777435	8.697340724
ENSG00000138002	IFT172	1.280202627	0.570234127	2.201368533	0.296233541	3.481571161	2	6.311476754	2	6.061031724	6.05460702	6.842137779	7.026981076
ENSG00000177427	MIEF2	0.681480327	0.685840372	2.79992398	0.052695388	3.481404307	2	6.602424674	4.523675391	5.949776857	6.1068222	8.113585664	8.044120856
ENSG00000198160	MIER1	1.990803486	0.007541767	1.48994492	0.051047807	3.480748406	6.009025997	7.35507212	8.110401118	9.392580682	9.3185152	8.956549551	8.75393321
ENSG00000170881	RNF139	1.699171049	0.275154513	1.777484847	0.243286625	3.476655896	3.327711234	8.101365653	5.59507254	8.395844809	8.54024645	8.703830473	8.37166861
ENSG00000119723	COQ6	2.552390503	0.004523149	0.923473776	0.352922604	3.475864279	3.327711234	5.457120953	4.058998049	6.902323594	7.03319408	5.318934974	5.540219242
ENSG00000159374	M1AP	0.015595214	0.997041173	3.459162295	0.226906648	3.474757509	2	2	2	2.498691189	2.14022021	4.065262523	3.771796761
ENSG00000081842	PCDHA6	2.394168084	0.464304361	1.079753107	NA	3.473921191	2	2	2	3.615725078	2.68924543	2.68698273	2.551896972
ENSG00000115310	RTN4	1.640375464	1.92E-08	1.832590331	2.34E-10	3.472965755	11.04852648	11.53726652	11.53541251	13.02801696	13.0336013	13.20295944	13.24264946
ENSG00000185112	FAM43A	1.455054709	0.315574141	2.014302705	0.143374446	3.469357414	2	6.311476754	6.43197674	7.300218887	7.12888795	8.117111293	7.294817518
ENSG00000107372	ZFAND5	1.289201606	0.163887287	2.175750551	0.011666318	3.464952157	10.1311248	10.27307349	7.858115942	11.06429261	11.0173806	11.77722057	12.06143722
ENSG00000076108	BAZ2A	1.844682252	1.05E-08	1.618250956	5.96E-07	3.462933207	9.159305284	9.018611864	8.561105505	10.73753559	10.8089302	10.62906029	10.46165359
ENSG00000070778	PTPN21	1.348254286	0.479038245	2.113999591	0.23691073	3.462253877	2	8.732943944	7.225742779	8.966774408	8.88608977	9.698306977	9.677294239
ENSG00000129003	VPS13C	2.083325284	0.00104027	1.377808501	0.037254341	3.461133784	7.096806373	7.503546079	8.65477198	9.982354854	9.95429779	9.209346464	9.321499526
ENSG00000165219	GAPVD1	1.700001036	0.241525716	1.758304585	0.216349452	3.45830562	4.00722531	8.395519306	9.014156722	9.906793906	9.82388974	10.01280126	9.829339
ENSG00000164187	LMBRD2	1.506454626	0.162158714	1.950672829	0.058080925	3.457127455	7.031698319	4.11352228	6.43197674	7.781117077	7.67287587	8.475683062	7.768360547
ENSG00000133874	RNF122	2.623926435	0.000487609	0.833171918	0.323536602	3.457098354	5.330594134	5.72254323	3.369317972	7.605658789	7.65837038	5.893224226	5.929275
ENSG00000065615	CYB5R4	2.319709965	1.40E-05	1.137068461	0.047012018	3.456778426	5.095959458	5.303523747	5.59507254	7.54315232	7.53992502	6.280431262	6.506723027
ENSG00000198836	OPA1	1.799593132	0.211077851	1.656260976	0.245873893	3.455854107	8.285626337	7.954278496	3.369317972	9.412823969	9.28680218	9.164389779	9.251828472
ENSG00000164855	TMEM184A	1.210637026	0.591930254	2.242861272	0.280327402	3.453498298	2	3.686303414	2	3.709785322	3.39680241	4.813804156	3.560117069
ENSG00000073150	PANX2	2.281327889	0.196009997	1.17126754	0.526432906	3.452595528	4.00722531	6.951736442	2	7.797276258	7.7866208	7.131485609	6.117095472
ENSG00000068650	ATP11A	1.654219603	0.414405236	1.798140637	0.361385021	3.452360239	4.815609471	8.297207175	2	8.43768081	8.52433649	8.62380783	8.624257514
ENSG00000163932	PRKCD	2.183912777	0.079842495	1.268186981	0.328187708	3.452099757	8.107517749	6.535001748	4.058998049	9.15827499	9.14521857	8.327733606	8.157201821
ENSG00000080503	SMARCA2	1.62856316	0.369452745	1.823493334	0.299990634	3.452056494	2	8.469613413	7.098165388	8.937912013	9.01578569	9.161319498	9.180307928
ENSG00000179761	PIPOX	1.217794822	0.526407754	2.232529353	0.203778744	3.450324175	2	3.076665209	3.369317972	3.921164147	3.28037825	4.68289886	4.033026654
ENSG00000188994	ZNF292	1.921311156	0.258819968	1.527429592	0.370755996	3.448740748	6.138360429	9.018611864	3.369317972	9.617795654	9.47652116	9.305806573	8.991966981
ENSG00000169967	MAP3K2	1.945796078	0.023002202	1.501381332	0.085501287	3.44717741	7.790810999	8.848535039	6.43197674	9.960398193	9.91761657	9.625909881	9.355112655
ENSG00000175105	ZNF654	2.157756176	0.154170396	1.28892813	0.410543204	3.446684307	2	6.728495673	7.098165388	8.504819462	8.43013848	7.942911451	7.18317631
ENSG00000109680	TBC1D19	2.506424594	0.061530373	0.939812734	0.519728384	3.446237328	2	5.838937808	4.058998049	7.114945424	7.0150921	5.343222328	5.785984581
ENSG00000170775	GPR37	4.897175552	0.003484322	-1.453817022	0.451026653	3.44335853	2	4.11352228	2	7.300218887	7.21466714	2.831336763	2.172608095
ENSG00000185640	KRT79	4.319709069	0.074800869	-0.877454815	0.759454549	3.442254254	2	2	2	4.739373666	4.45712836	2.094598369	2.277179783
ENSG00000135999	EPC2	2.114988183	0.390019856	1.326465979	0.598500734	3.441454163	2	7.431218269	2	7.982979299	7.94559728	7.249266052	7.133547492
ENSG00000116977	LGALS8	2.726676957	4.73E-06	0.712947531	0.294558331	3.439624488	9.048471759	8.395519306	7.451345288	11.13936824	11.1652631	8.971039232	9.302653594
ENSG00000138757	G3BP2	1.530352294	2.53E-13	1.907424873	2.24E-20	3.437777167	9.460782017	9.514922632	9.541233192	11.07565424	10.9854873	11.37402863	11.44064487
ENSG00000134108	ARL8B	2.670247802	0.137084233	0.766800676	0.699334721	3.437048478	8.138771685	9.04308532	2	10.77881047	10.6952249	9.009108162	8.656119846
ENSG00000108861	DUSP3	1.492272668	0.053811959	1.944443358	0.008947043	3.436716026	9.790941832	9.338312924	7.734844356	10.67841925	10.6657943	11.03528871	11.20632352
ENSG00000107742	SPOCK2	2.214745958	0.498491454	1.221343176	NA	3.436089135	2	2	2	3.289987342	2.94009902	2.885263063	2.421067459
ENSG00000115425	PECR	0.968794954	0.477022398	2.466947033	0.040591855	3.435741987	2	4.710564154	3.369317972	4.76150579	4.40247105	5.83960027	6.011156536
ENSG00000111254	AKAP3	0.272098667	0.936411459	3.159502559	0.259652636	3.431601225	2	2	2	2.270786764	2.49394783	3.865109708	3.539239711
ENSG00000131873	CHSY1	1.248740485	0.219547821	2.181386843	0.021222059	3.430127329	6.468621203	9.28800521	8.042706615	9.580155061	9.60385643	10.4390324	10.59881657
ENSG00000118564	FBXL5	0.821819696	0.536398142	2.605670516	0.026039832	3.427490212	5.330594134	9.067150531	9.099922433	9.371149971	9.36392108	11.3748388	10.8718893
ENSG00000163064	EN1	3.04642094	0.344298341	0.380426941	0.918213588	3.42684788	2	2	2	3.566290607	3.69849104	2.656294034	2.117335275
ENSG00000110911	SLC11A2	2.610345833	0.012491705	0.816205276	0.483694039	3.42655111	5.532353208	8.777382925	7.285536013	10.32141448	10.3491863	8.442343897	8.654300638
ENSG00000100034	PPM1F	1.17726834	0.472493119	2.248141127	0.134649149	3.425409467	4.00722531	9.125613817	6.629488387	8.967964567	8.99168852	10.05752505	10.03183938
ENSG00000168917	SLC35G2	3.251988164	0.053572957	0.172252297	0.93387742	3.42424046	2	4.442710129	7.398165132	9.16866395	9.18624204	5.99483933	6.326546677
ENSG00000160213	CSTB	1.768537516	0.075478678	1.655188279	0.093761478	3.423725795	10.13887448	7.701025996	8.175061096	10.80734846	10.8718924	10.41445658	10.9835621
ENSG00000198677	TTC37	1.761246557	0.047659578	1.661805177	0.06022343	3.423051734	8.009475818	9.74864196	7.552162999	10.52434207	10.5221789	10.47739344	10.36874764
ENSG00000139132	FGD4	0.717587394	0.431518756	2.704430729	0.000501743	3.422018124	5.70932728	7.954278496	7.777116083	8.168339746	8.12510181	9.989716154	10.23658897
ENSG00000108061	SHOC2	1.468991129	0.263164637	1.952013247	0.119669999	3.421004376	4.467311984	8.655694736	8.236946975	9.397888985	9.35764963	9.945186878	9.766168491
ENSG00000173451	THAP2	1.650011816	0.220999164	1.770535357	0.177528888	3.420547173	2	5.303523747	4.874587708	6.113598934	6.1068222	6.750676314	5.385063124
ENSG00000166793	YPEL4	2.043335023	0.211460922	1.375758144	0.40972733	3.419093167	2	3.686303414	4.058998049	5.22679461	4.91316568	5.102662916	3.474731315
ENSG00000198373	WWP2	1.544888061	0.314797955	1.873627623	0.205709574	3.418515684	3.327711234	6.535001748	8.408111533	8.707945635	8.73115061	8.97958623	9.10860334
ENSG00000104093	DMXL2	1.891679514	0.272777026	1.526277904	0.376725096	3.417957418	2	7.638155693	8.076950877	9.174861605	9.15867957	9.090272833	8.447159218
ENSG00000100890	KIAA0391	2.778430346	0.002395404	0.638759899	0.545022271	3.417190245	4.00722531	6.728495673	5.39244166	8.543652346	8.44373613	6.387074688	6.429408106
ENSG00000183495	EP400	1.636761231	0.002559868	1.77878949	0.000883053	3.415550722	7.096806373	7.954278496	8.460932916	9.566077486	9.55457603	9.767692698	9.63241113
ENSG00000121892	PDS5A	1.588313232	0.026020592	1.825962356	0.00889175	3.414275589	7.904282243	9.470501606	8.110401118	10.25909638	10.2602493	10.68167537	10.28422407
ENSG00000168350	DEGS2	0.012819211	0.997163678	3.399352251	0.239702507	3.412171462	2	2	2	2.3892346	2.2680111	4.397636241	3.071407821
ENSG00000105136	ZNF419	1.806635513	0.203704581	1.605535119	0.256352368	3.412170632	2	6.535001748	5.59507254	7.273514749	7.39346687	7.435871964	6.764548117
ENSG00000274600	RIMBP3B	5.695468939	0.030320381	-2.284001069	0.458652814	3.41146787	2	2	2	5.900763176	5.74985018	2.139668783	2
ENSG00000259024	TVP23C-CDRT	8.601227482	8.43E-08	-5.191150712	0.031629908	3.41007677	2	2	2	8.583021623	8.69891174	2	2
ENSG00000102547	CAB39L	1.740821355	0.503519889	1.669096175	0.515079411	3.40991753	2	7.790474954	2	7.939959586	7.96694292	7.623070264	8.103103292

ENSG00000154222	CC2D1B	2.068570494	0.07702728	1.340758554	0.265688503	3.409329048	6.138360429	4.710564154	8.042706615	8.872051712	8.98123666	7.995960198	8.392188801
ENSG00000174243	DDX23	1.742986566	0.037693949	1.665741646	0.045767852	3.408728211	8.367060731	8.191702463	6.073378139	9.555032495	9.59474367	9.404610599	9.586086719
ENSG00000081087	OSTM1	1.679995665	0.001116395	1.72807689	0.000721942	3.408072555	7.82963488	8.540087354	7.398165132	9.72797373	9.6241521	9.558789939	9.873793342
ENSG00000131094	C1QL1	0.919676794	0.649272397	2.485979592	0.166578095	3.405656386	2	4.11352228	2	4.034562184	3.39680241	5.352823931	4.532869193
ENSG00000180596	HIST1H2BC	4.320484731	1.31E-06	-0.915357652	0.384545195	3.40512708	4.00722531	3.686303414	5.156626401	8.408172799	8.42842973	3.215043717	4.062589736
ENSG00000272391	POM121C	2.602506947	2.06E-14	0.802316478	0.03268044	3.404823424	8.228665467	7.669933291	8.175061096	10.60649663	10.6316726	8.704299335	8.94213989
ENSG00000119938	PPP1R3C	0.729984381	0.49620218	2.674793663	0.003705475	3.404778044	9.304185368	7.572420273	6.80318718	8.945181925	9.05949698	11.21786533	10.59534423
ENSG00000110931	CAMKK2	1.24965622	3.70E-05	2.153040276	1.01E-13	3.402696496	7.940205782	7.638155693	7.818184403	9.015934546	9.04730166	9.99643172	9.860179463
ENSG00000144306	SCRN3	1.319082723	0.371698295	2.083496556	0.131743628	3.402579279	3.327711234	6.899080544	4.058998049	6.979883891	6.78340763	7.64960537	7.608437243
ENSG00000088543	C3orf18	1.398159903	0.453666628	1.999258611	0.256697952	3.397418514	2	7.393647515	4.523675391	7.333842102	7.4314987	8.000548654	7.944426693
ENSG00000182308	DCAF4L1	2.387473346	0.446075855	1.009085259	NA	3.396558605	2	2	2	2.947924294	3.45167086	2.560105256	2.632958241
ENSG00000179085	DPM3	1.546425323	0.112649338	1.845853264	0.049503707	3.392278587	5.095959458	3.686303414	6.203069917	6.64692533	6.82036165	6.995586672	7.05482945
ENSG00000164877	MICALL2	1.136889993	0.615473512	2.255043031	0.280004532	3.391933024	8.468948322	2	4.523675391	8.058664058	8.13770558	9.078385428	9.322263543
ENSG00000035403	VCL	0.315607148	0.726269433	3.075088089	1.70E-05	3.390695237	9.720386367	11.66299451	10.68342405	11.19937403	11.2319249	13.64177426	14.24245265
ENSG00000183579	ZNRF3	1.738356523	0.02413324	1.651305209	0.031331843	3.389661732	4.00722531	4.442710129	5.39244166	6.229283386	6.33555364	6.332313483	6.055940621
ENSG00000065325	GLP2R	-0.317990197	0.948248384	3.707175828	0.38169184	3.389185631	2	2	2	2	2.49394783	4.644324387	3.261853426
ENSG00000178966	RMI1	1.595134135	0.452687885	1.791513121	0.38150221	3.386647256	2	5.72254323	2	5.930370745	5.62411716	6.346800618	5.453611367
ENSG00000132436	FIGNL1	1.786897853	0.383862151	1.599502375	0.431616031	3.386400228	2	5.72254323	2	5.930370745	6.00043082	6.016144783	5.524371359
ENSG00000155975	VPS37A	1.953629597	0.281556878	1.432544683	0.435144797	3.38617428	8.631580145	7.099070191	2	9.40053583	9.42945789	9.133040681	8.616180753
ENSG00000135974	C2orf49	1.636786211	0.178578057	1.749312484	0.140060743	3.386098695	7.624139768	5.303523747	4.523675391	8.04293816	7.99722844	8.351275892	7.871136591
ENSG00000184905	TCEAL2	3.362358	0.20486278	0.023426224	0.994111049	3.385784224	2	2	2	3.921164147	3.7864959	2.384001718	2.277179783
ENSG00000075240	GRAMD4	1.529554442	0.524284348	1.853812847	0.422430971	3.383367289	2	6.844429755	2	6.851641821	6.77267312	7.331691625	6.887339785
ENSG00000100852	ARHGAP5	2.011583805	0.18460402	1.370065752	0.375326773	3.381649557	4.00722531	9.367676479	7.858115942	10.26782038	10.2188359	9.867145934	9.283558215
ENSG00000087074	PPP1R15A	2.74943733	0.002429018	0.631007747	0.546187635	3.380445077	7.27616306	9.308339287	10.10298738	12.00455363	12.0735623	9.868819956	9.980250523
ENSG00000159423	ALDH4A1	0.438524799	0.787199307	2.936899527	0.028740291	3.375424326	3.327711234	6.951736442	4.058998049	6.024891728	6.14893615	8.333200639	8.676578788
ENSG00000144468	RHBDD1	1.443370898	0.17057763	1.929823483	0.054562067	3.373194381	4.815609471	7.847167909	7.342949392	8.567402595	8.54656162	9.264754164	8.771382398
ENSG00000219481	NBPF1	4.013015945	6.94E-07	-0.641279242	0.507862588	3.371736703	6.89194353	8.337339476	6.073378139	11.34961059	11.4541495	7.06586564	6.471341372
ENSG00000101493	ZNF516	1.603732389	0.201491557	1.767995429	0.147348187	3.371727819	4.467311984	8.297207175	6.718950348	8.756965077	8.82375772	9.087759434	8.80566012
ENSG00000169962	TAS1R3	0.007224108	0.99798804	3.364485949	0.247980941	3.371710057	2	2	2	2.141737246	2.49394783	4.029788235	3.660199087
ENSG00000142459	EVI5L	0.80142163	0.613329998	2.570040711	0.065337615	3.371462341	6.73718411	8.356994601	3.369317972	7.942383523	8.06698125	9.661355639	9.84988408
ENSG00000100505	TRIM9	2.649567841	0.440574326	0.721164812	0.847276751	3.370732653	2	2	2	3.921164147	2.49394783	2.62493832	2.374681325
ENSG00000139211	AMIGO2	1.576904625	0.073765025	1.793568233	0.036652015	3.370472858	8.653385977	8.522788974	6.534109959	9.701744597	9.76823652	10.34924812	9.399228411
ENSG00000040933	INPP4A	1.336697441	0.317344658	2.032534831	0.106808076	3.369232272	3.327711234	7.315436877	7.502634626	8.093978021	8.2580338	8.855903324	8.877074023
ENSG00000146063	TRIM41	1.746944156	0.305856687	1.618792953	0.337037541	3.365737109	8.107517749	7.189546432	2	8.836004565	8.89972012	8.772063028	8.709655965
ENSG00000162654	GBP4	5.734635802	6.38E-09	-2.370906063	0.028948196	3.363729739	5.532353208	3.686303414	6.43197674	11.2046844	11.1596093	4.065262523	3.012034821
ENSG00000204149	AGAP6	3.095094872	0.027688126	0.267479697	0.877195052	3.362574569	2	4.442710129	6.534109959	8.203120604	8.284723	5.594558848	5.459179378
ENSG00000161800	RACGAP1	1.386926014	0.311279445	1.974854189	0.127639854	3.361780204	5.866944937	6.464272742	2	6.946463121	7.02417148	7.663174541	7.445480288
ENSG00000142599	RERE	1.276897833	0.155953136	2.084623302	0.013226967	3.361521134	7.031698319	9.470501606	8.511888463	9.89995113	9.9465583	10.75863418	10.69795699
ENSG00000188419	CHM	2.038229774	0.007413593	1.323270383	0.094621254	3.361500157	7.27616306	7.538394143	5.39244166	9.036500968	8.98821297	8.380320298	8.224954107
ENSG00000131503	ANKHD1	4.816655689	1.04E-18	-1.45787615	0.016837458	3.358779539	6.138360429	5.131603625	4.874587708	10.14850007	10.1741606	4.350488971	4.09155917
ENSG00000107560	RAB11FIP2	1.890352807	0.118659477	1.468217222	0.229961504	3.358570029	3.327711234	7.189546432	6.43197674	8.132699624	8.23866319	7.968118715	7.543088047
ENSG00000107643	MAPK8	2.126900491	0.178652578	1.231059546	0.453051282	3.357960038	7.27616306	7.60566235	2	8.952415387	8.99976571	8.094396944	8.086110269
ENSG00000097007	ABL1	1.495004305	0.12639307	1.862035464	0.047854089	3.357039769	7.750913381	9.994333773	10.54960077	11.28518643	11.3595809	11.64101736	11.73618212
ENSG00000127870	RNF6	1.509272035	0.440574326	1.847531774	0.324961434	3.356803809	2	7.315436877	9.06622548	9.331938606	9.3808074	9.723042782	9.66228668
ENSG00000146425	DYNLT1	1.865017444	0.004829424	1.490368984	0.027424614	3.355386428	9.143983763	7.572420273	7.97167868	10.24542	10.2326097	9.732038014	9.98821576
ENSG00000140992	PDPK1	1.922345888	3.66E-05	1.429048411	0.002775982	3.351394299	7.790810999	8.213429689	7.098165388	9.690252889	9.68338024	9.258063226	9.13188655
ENSG00000099917	MED15	1.446593211	0.097990086	1.902266969	0.023148373	3.34886018	6.563806279	8.968383114	8.35328246	9.690973808	9.71736428	10.06173917	10.2482943
ENSG00000015285	WAS	0.27207001	0.938309757	3.076788287	0.291783001	3.348858297	2	2	2	2.270786764	2.49394783	3.993419637	3.209956562
ENSG00000166670	MMP10	8.538358019	1.30E-07	-5.191150625	0.032232657	3.347207394	2	2	2	8.634896	8.5227358	2	2
ENSG00000196967	ZNF585A	2.169133567	0.027857303	1.176685786	0.258406162	3.345819353	6.257048221	6.140560021	4.058998049	7.839502934	7.89700047	7.434742221	6.041166521
ENSG00000119986	AVPI1	1.556417066	0.545415898	1.788287041	0.475468809	3.344704107	2	7.638155693	2	7.523859802	7.7127321	7.847655556	7.850099615
ENSG00000111962	UST	1.221101671	0.208574574	2.123494073	0.018761048	3.344595744	6.138360429	8.607278467	7.552162999	8.935480545	9.0020652	9.387195342	10.22519644
ENSG00000171223	JUNB	1.930019485	0.012630673	1.414037493	0.075077708	3.344056978	9.740899611	7.73146271	9.049077186	10.96138842	10.9898386	10.76066432	10.08209816
ENSG00000196653	ZNF502	1.62826827	0.281547017	1.715562842	0.244635807	3.343831112	3.327711234	5.595927442	2	5.959382894	5.7929843	6.013120342	5.905000563
ENSG00000075303	SLC25A40	1.525309076	0.069582907	1.817000766	0.025221004	3.342309841	3.327711234	5.303523747	4.874587708	6.078767801	6.18985553	6.537789343	6.27380897
ENSG00000101442	ACTR5	1.195662785	0.324473681	2.146485146	0.05484371	3.342147931	2	5.131603625	4.058998049	5.210797711	5.37722731	6.132200015	6.215784511
ENSG00000242498	ARPIN	1.091032559	0.017866632	2.250947262	1.06E-07	3.341979821	6.366709671	6.844429755	6.203069917	7.697489359	7.4450836	8.691586261	8.747690481
ENSG00000104361	NIPAL2	1.910704697	0.090432691	1.430547503	0.210610076	3.3412522	5.532353208	7.099070191	8.825624954	9.634381252	9.59930725	8.927126162	9.324934421
ENSG00000197863	ZNF790	1.945995729	0.129843791	1.395038697	0.284984329	3.341034426	5.70932728	4.11352228	2	6.422239259	6.31350454	6.013120342	5.66592325
ENSG00000088247	KHSRP	1.644872607	0.020681631	1.694806541	0.016160038	3.339679149	7.159102577	8.876041558	8.884815469	10.11343618	10.1572376	10.03682636	10.31985465
ENSG00000177873	ZNF619	1.218947667	0.142769855	2.116864693	0.005694583	3.33581236	5.095959458	4.442710129	4.058998049	5.731694	5.60003091	6.726875343	6.257923755
ENSG00000196705	ZNF431	1.748406212	0.044940119	1.586001351	0.068074655	3.334407563	3.327711234	5.838937808	5.39244166	6.882264028	6.82556437	6.78654324	6.602920209
ENSG00000141425	RPRD1A	1.560100195	0.296977641	1.768459259	0.221520727	3.328559455	3.327711234	8.234834549	8.206335842	9.196346103	9.20931286	9.515363796	9.29605726
ENSG00000198783	ZNF830	1.68770209	0.474361527	1.639634987	0.478795283	3.327337077	2	6.78762695	2	6.979883891	6.83591385	6.830158212	6.895576665
ENSG00000256525	POLG2	1.407353188	0.279318358	1.919872459	0.119249183	3.327225647	2	5.131603625	4.058998049	5.420062284	5.53799497	6.280431262	5.529673353
ENSG00000153071	DAB2	1.249928089	0.035146883	2.076419429	0.000188322	3.326347517	10.79260216	10.71514053	10.20715117	11.83668751	11.8476762	11.9157543	13.15993594
ENSG00000012223	LTF	0.01278131	0.9971266	3.311979747	0.221353293	3.324761056	2	2	2	2.3892346	2.2680111	3.648325314	3.972018587
ENSG00000188747	NOXA1	-0.310504433	0.925672996	3.633863867	0.164988406	3.323359434	2	2	2	2.270786764	2.2680111	4.378961816	3.640730416
ENSG00000175066	GK5	1.445366762	0.119221214	1.876266104	0.034894638	3.321632866	5.532353208	6.228547981	7.858115942	8.304583895	8.2618769	8.778311345	8.637209821
ENSG00000178694	NSUN3	1.916432387	0.096878945	1.40065029	0.231333139	3.317082677	2	5.303523747	5.772716771	6.744602355	6.88657603	6.414841377	6.22561877
ENSG00000198429	ZNF69	1.476023984	0.072447018	1.840349916	0.019563833	3.3163739	4.815609471	4.710564154	3.369317972	5.709096143	5.80356929	6.095496313	6.099376274
ENSG00000185201	IFITM2	2.199501432	1.06E-08	1.116615205	0.006238495	3.316116637	10.80726569	10.64492283	10.82615578	12.90548945	13.0088736	11.42170857	12.22106411
ENSG00000108468	CBX1	1.492144699	0.219632896	1.823292509	0.119536754	3.315437209	8.87372952	6.951736442	5.39244166	9.144656522	9.24954577	9.375172844	9.664698405
ENSG00000121957	GPSM2	1.394561466	0.53868734	1.920563646	0.374841596	3.315125112	2	6.311476754	2	6.147608921	6.18176397	6.934553331	6.33565646
ENSG00000172943	PHF8	1.34282615	0.123384912	1.97113327	0.016889842	3.31395942	4.815609471	6.602424674	7.225742779	7.852449262	7.78928606	8.612361808	8.245238611
ENSG00000107798	LIPA	2.1735307	0.001190589	1.140072489	0.110628724	3.313603189	7.750913381	9.148351514	7.934808822	10.5732559	10.6024186	9.881106239	9.141874832
ENSG00000111530	CAND1	1.447522376	0.261766667	1.865428034	0.130735409	3.312950411	9.27634997	10.07368926	5.930865648	10.63412381	10.6435047	11.13029223	10.97726982
ENSG00000147588	PMP2	3.221029076	0.544857088	0.091472225	0.987740261	3.312501301	2	2	2	4.523237872	2	2.457042231	2.225841198
ENSG00000251349	MSANTD3-TI	8.50262631	1.62E-07	-5.191150591	0.032497074	3.31147572	2	2	2	8.584574266	8.50338773	2	2
ENSG00000111679	PTPN6	0.316332522	0.916384376	2.99509129	0.227412477	3.311423812	3.327711234	2	2	2.947924294	2.38539787	4.971696071	3.042026731
ENSG00000169627	BOLA2B	8.50185586	1.65E-07	-5.191150583	0.032532189	3.310705278	2	2	2	8.589222193	8.49688027	2	2
ENSG00000130193	THEM6	0.881715739	0.529996014	2.428253465	0.050406299	3.309969204	6.257048221	5.303523747	2	6.18902405	6.08962627	7.302260672	7.900082338
ENSG00000130962	PRRG1	1.642587211	0.146700672	1.666742037	0.13261638	3.309329248	3.327711234	6.728495673	6.629488387	7.807949361	7.70992146	7.69371876	7.868000534
ENSG00000136152	COG3	1.99785036	0.051682313	1.311291674	0.215092713	3.309142035	4.815609471	7.701025996	6.322058544	8.66162195	8.72420319	8.295111724	7.667350749
ENSG00000119392	GLE1	1.413696196	0.38028167	1.894149091	0.217050348	3.307845287	7.435667355	6.464272742	2	7.728718552	7.94559728	8.122734438	8.483814596
ENSG00000161671	EMC10	1.880939635	0.004752131	1.42682627	0.036771903	3.307765905	9.533135543	8.169643011	8.266922072	10.62811365	10.7079584	9.861061271	10.50031203
ENSG00000178935	ZNF552	1.792208506	0.171506539	1.514652682	0.246643226	3.306861188	5.095959458	2	4.058998049	5.764940652	5.58783529	5.343222328	5.502966356
ENSG00000129116	PALLD	-0.06708444	0.91483416	3.373825505	8.15E-14	3.306741066	10.62615013	11.15761406	10.12770132	10.68059872	10.583733	13.85056693	14.26144901
ENSG00000136682	CBWD2	3.780661339	0.000665039	-0.474658576	0.726349422	3.306002763	6.257048221	5.457120953	2	8.967964567	9.08358266	4.720468739	5.164234344
ENSG00000118160	SLC8A2	2.690456475	0.36407141	0.614958872	0.852211698	3.305415347	2	2	2	3.515101962	3.28037825	2.526562374	2.421067459
ENSG00000136205	TNS3	1.959165154	0.006834427	1.344405988	0.072934705	3.303571142	8.009475818	9.608085847	9.757248847	11.25892943	11.2665282	10.95373885	10.26269416
ENSG00000118308	LRMP	1.27444235	0.725609862	2.028257093	NA	3.302699443	2	2	2	2.695456494	2.68924543	3.429858444	2.466008509
ENSG00000119720	NRDE2	1.701589844	0.111942166	1.599987847	0.130241043	3.301577691	5.532353208	7.051611315	4.058998049	7.584088227	7.79989812	7.586913431	7.605929526
ENSG00000130150	MOSPD2	1.152585811	0.516281765	2.148327779	0.188579572	3.30091359	2	7.928237109	6.629488387	7.994705029	7.97398853	9.005308106	8.931163478
ENSG00000132840	BHMT2	0.685023454	0.492643658	2.615740129	0.002064011	3.300763582	4.815609471	4.442710129	6.073378139	6.043074888	5.70538658	7.187558829	8.139971339
ENSG00000125144	MT1G	8.020928413	1.90E-08	-4.721352482	0.036346499	3.299575931	2	2	3.369317972	8.989221226	9.05063789	2.048074405	2
ENSG00000090615	GOLGA3	1.231472863	9.96E-05	2.065335865	9.68E-12	3.296808728	9.174465797	9.558016677	9.767769415	10.70435807	10.7890936	11.57807074	11.58140223
ENSG00000173991	TCAP	0.01279932	0.997163678	3.283196652	0.240873009	3.295995972	2	2	2	2.3892346	2.2680111	4.133696865	3.36036153
ENSG00000197771	MCMBP	1.820516675	0.034515932	1.474902538	0.090709877	3.295419213	7.579279349	8.255926468	5.772716771	9.344822471	9.3166689	9.059452688	8.912005269
ENSG00000136807	CDK9	1.392309583	0.025474975	1.901649888	0.001438346	3.293959472	6.257048221	7.701025996	7.734844356	8.690748087	8.80675844	9.22151698	9.288258056
ENSG00000135482	ZC3H10	1.290546862	0.56072077	2.002546334	0.339728351	3.293093196	2	6.140560021	2	5.968925393	5.83486613	6.656902456	6.493217192
ENSG00000026751	SLAMF7	-3.725402998	0.02361388	7.015993643	2.07E-07	3.290590645	3.327711234	3.076665209	5.156626401	2.498691189	2.14022021	8.840629888	11.58498783
ENSG00000145107	TM4SF19	3.399630251	0.119911452	-0.109145643	0.967718357	3.290484608	2	2	3.369317972	4.574143677	4.70309173	2.803598391	2.225841198
ENSG00000137814	HAUS2	1.40112673	0.018985894	1.889046189	0.000946606	3.290172919	5.532353208	5.946638857	6.629488387	7.559034656	7.32163988	8.140859753	7.667350749
ENSG00000279389		2.014286207	0.035203087	1.274979328	0.198028433	3.289265534	5.532353208	7.847167909	5.930865648	8.833394974	8.78422202	8.264917037	7.863808491
ENSG00000197296	FITM2	-0.146404889	0.936681124	3.43524688	0.018858407	3.288841991	2	4.93638933	7.029880454	5.731694	5.53799497	8.897090174	9.294500788
ENSG00000121858	TNFSF10	5.904868845	0.00059577	-2.621812825	0.164236009	3.28305602	6.009025997	2	2	10.27601154	10.1613584	3.011868911	2.671842496

ENSG00000271880	AGAP11	5.613045543	0.001118566	-2.330358267	0.255544364	3.282687275	2	2	3.369317972	6.610808945	6.70653264	2.048074405	2.225841198
ENSG00000136275	C7orf69	0.274456092	0.939900699	3.007014159	0.323451531	3.281470251	2	2	2	2.3892346	2.38539787	4.099885429	2.851942824
ENSG00000188554	NBR1	1.545204187	0.010826309	1.735876565	0.003524935	3.281080752	9.081016759	10.38739569	9.227351203	11.25015134	11.2213553	11.39072895	11.4608208
ENSG00000244115	DNAJC25-GN	8.471144183	2.12E-07	-5.191150503	0.032980585	3.27999368	2	2	2	8.576794289	8.44711562	2	2
ENSG00000187555	USP7	1.623281294	0.011059186	1.65453515	0.009052045	3.277816443	8.444143041	8.395519306	6.882775709	9.693134408	9.66390646	9.697600698	9.721691937
ENSG00000023608	SNAPC1	2.397177531	0.074912837	0.879842527	0.547042483	3.277020058	3.327711234	7.232742069	4.874587708	8.330724217	8.32935768	6.989444349	6.685996416
ENSG00000047578	KIAA0556	1.465289188	0.116476863	1.808849819	0.044161511	3.274139007	7.485143553	7.315436877	4.874587708	8.356399318	8.40429221	8.639092784	8.797441501
ENSG00000124120	TTPAL	0.707503143	0.530030976	2.566385533	0.008125446	3.273888676	5.330594134	8.029695761	6.322058544	7.683067677	7.70992146	9.263163896	9.763920691
ENSG00000154803	FLCN	2.406104917	1.05E-05	0.867166721	0.150582065	3.273271638	5.70932728	7.099070191	6.718950348	9.002058889	8.98705259	7.600074836	7.342293759
ENSG00000110497	AMBRA1	1.800556154	0.048401835	1.472568236	0.109372081	3.27312439	7.27616306	7.819099893	5.156626401	8.81895723	8.97656697	8.465761957	8.676578788
ENSG00000166922	SCG5	8.839096306	1.18E-12	-5.566038269	0.007570639	3.273058037	2	3.686303414	2	10.67076507	10.682378	2.048074405	2
ENSG00000240857	RDH14	1.417162562	0.063833268	1.853646762	0.011498073	3.270809324	6.009025997	7.232742069	5.39244166	7.815902688	7.83127384	8.217092349	8.28888532
ENSG00000177565	TBL1XR1	1.015862686	0.062718919	2.253885329	5.51E-06	3.269748015	9.143983763	8.169643011	8.143093325	9.664056897	9.46989111	10.91992417	10.67765183
ENSG00000136295	TTYH3	1.546000906	6.03E-05	1.723375201	5.79E-06	3.269376108	9.975371636	9.256956408	9.849281147	11.23391814	11.2935316	11.27870987	11.58713493
ENSG00000276180	HIST1H4I	-2.311964322	0.478464462	5.580879938	0.017373749	3.268915616	2	2	2	2	2.14022021	5.515547099	5.900914815
ENSG00000142178	SIK1	8.45901032	2.20E-07	-5.191150516	0.03297173	3.267859804	2	2	2	8.458152491	8.54340749	2	2
ENSG00000070759	TESK2	1.023891733	0.617138584	2.241945116	0.229720627	3.265836849	2	5.457120953	2	4.965943888	5.07918227	5.985611216	6.27380897
ENSG00000170325	PRDM10	1.478362602	0.203115525	1.786579432	0.110104573	3.264942034	6.653098118	5.131603625	3.369317972	7.127781374	6.86146731	7.470462265	7.107191496
ENSG00000132478	UNK	1.682988223	0.335558745	1.581601188	0.359818453	3.264589411	2	7.819099893	5.930865648	8.16211395	8.33484115	7.973574582	8.309067496
ENSG00000134864	GGACT	1.183492611	0.280090671	2.080935945	0.038900682	3.264428556	4.467311984	4.710564154	2	5.022023783	5.30456641	5.916072173	6.06327132
ENSG00000106459	NRF1	1.593644647	0.208574574	1.669332153	0.177265	3.262976799	2	5.946638857	5.930865648	6.877205252	6.94511186	7.009312024	6.959831579
ENSG00000257315	ZBED6	2.92017094	0.060865068	0.342020052	0.853341364	3.262190992	2	7.35507212	5.39244166	8.929383887	9.04952667	6.34920106	6.577546999
ENSG00000158615	PPP1R15B	1.411843954	0.043881335	1.848115962	0.006017652	3.259959916	8.16936288	8.487557258	6.718950348	9.384581425	9.36570792	9.949148417	9.654117214
ENSG00000186566	GPATCH8	1.350765896	0.160378969	1.909095568	0.036346775	3.259861464	6.468621203	9.159587375	8.266922072	9.632881324	9.72504082	10.13173193	10.33280292
ENSG00000189339	SLC35E2B	1.940510468	0.015305913	1.318905967	0.110602847	3.259416434	6.257048221	8.004992147	8.536706852	9.734981585	9.85856545	9.198071497	9.161646304
ENSG00000147100	SLC16A2	1.134360936	0.000879733	2.124903058	3.90E-11	3.259263994	7.579279349	7.467835385	7.858115942	8.813671111	8.6932307	9.637249338	9.833364301
ENSG00000029725	RABEP1	1.886770318	0.029070155	1.372434755	0.121056843	3.259205074	6.563806279	8.993716073	7.896971941	10.02242928	10.0033302	9.421247846	9.576512555
ENSG00000134313	KIDINS220	1.582193416	0.000135408	1.6764341	4.39E-05	3.258627516	8.042902253	8.955547846	8.511888463	10.13690654	10.1243681	10.25686316	10.19151462
ENSG00000027697	IFNGR1	1.725201628	0.321721272	1.532674362	0.375035076	3.25787599	8.075571721	8.053983481	2	9.205456697	9.18624204	9.143796722	8.850838709
ENSG00000152377	SPOCK1	2.440537816	8.99E-06	0.817111968	0.181719899	3.257649784	9.602033055	9.672251278	10.78531698	12.54062562	12.5849424	10.80166469	11.06900213
ENSG00000100626	GALNT16	-1.086111438	0.450183579	4.343693455	0.000162517	3.257582017	3.327711234	3.076665209	4.058998049	3.227757282	2.49394783	7.076045862	7.448282715
ENSG00000116039	ATP6V1B1	3.388980517	0.206233223	-0.131556533	0.967718357	3.257423984	2	2	2	3.754611435	3.98554648	2.225793514	2.374681325
ENSG00000116731	PRDM2	1.592527772	0.012350658	1.664297286	0.008211245	3.256825058	6.89194353	7.701025996	8.511888463	9.386362875	9.45570025	9.584486439	9.394877047
ENSG00000197343	ZNF655	1.76613515	0.088075356	1.487867869	0.150474848	3.254003018	5.70932728	8.820493869	8.175061096	9.856271709	9.74645749	9.55749302	9.492799274
ENSG00000100401	RANGAP1	1.381469255	0.011026701	1.872463393	0.000337252	3.253932648	8.963744667	8.762721501	9.907528187	10.62849002	10.7188341	11.06196671	11.25994485
ENSG00000107104	KANK1	1.088881824	0.446977367	2.164580502	0.098016923	3.253462326	3.327711234	6.951736442	7.858115942	7.97352961	8.01099479	9.219878303	8.871343049
ENSG00000142166	IFNAR1	1.972546925	0.02795781	1.273815432	0.170653805	3.246362358	6.138360429	8.717821467	8.408111533	10.12841597	10.0193108	9.423527655	9.334054358
ENSG00000099968	BCL2L13	1.65816204	0.048419679	1.588138066	0.056802649	3.246300106	9.460782017	9.277729677	7.225742779	10.56226593	10.6246011	10.38038819	10.6544806
ENSG00000198018	ENTPD7	2.90665826	1.01E-08	0.335544721	0.590927058	3.242202981	6.89194353	6.899080544	7.934808822	10.22067068	10.1659803	7.481445808	7.795024296
ENSG00000151320	AKAP6	-0.609729714	0.786127952	3.850765817	0.037996695	3.241036102	2	5.72254323	2	4.299492648	3.39680241	8.408216037	7.299480881
ENSG00000067704	IARS2	1.552605022	0.04039121	1.679866054	0.024026933	3.232471076	8.674867122	9.04308532	7.029880454	10.02300196	10.0198783	10.14024701	10.15617086
ENSG00000179241	LDLRAD3	1.721638084	0.31167516	1.509845056	0.371461791	3.23148314	4.467311984	7.002537973	2	7.366699513	7.33629421	7.039353222	7.244113847
ENSG00000152137	HSPB8	0.922563799	0.1808519	2.307568417	0.000173235	3.230132215	8.946185348	7.315436877	8.511888463	9.246247604	9.37815424	10.68452972	10.70163167
ENSG00000163328	GPR155	0.796223173	0.140821478	2.431580462	2.67E-07	3.227803636	6.96351253	7.051611315	5.930865648	7.474468681	7.56502709	9.195072139	9.070914229
ENSG00000110583	NAA40	1.29275132	0.282799717	1.934886631	0.08797541	3.227637951	5.532353208	8.101365653	5.39244166	8.085230238	8.30535212	8.651788724	8.996760622
ENSG00000134250	NOTCH2	1.225266896	0.083598237	1.998535319	0.002527641	3.223802215	9.895281535	11.67086841	11.08782089	12.26720893	12.2949627	13.0065992	13.09977244
ENSG00000180354	MTURN	1.231700612	0.194489031	1.991002584	0.024727678	3.222703195	4.467311984	6.535001748	4.874587708	6.907295206	6.66650236	7.421115854	7.630812678
ENSG00000105464	GRIN2D	0.68764422	0.730082566	2.53503807	0.14438651	3.22268229	2	5.131603625	2	4.647285135	4.22485516	6.075339068	6.113568999
ENSG00000147421	HMBOX1	1.307913202	0.49634303	1.914697592	0.291256234	3.222610795	2	7.232742069	4.058998049	7.035434774	7.16216989	7.824551575	7.547015159
ENSG00000116266	STXBP3	2.165957404	0.051020395	1.056166229	0.369090445	3.222123632	7.579279349	6.311476754	4.058998049	8.776115811	8.61876544	7.667028121	7.54177663
ENSG00000172331	BPGM	1.884353966	1.47E-08	1.337308578	8.53E-05	3.221662544	8.313285031	8.276714465	8.631919287	10.25812379	10.3093543	9.525746748	9.92498893
ENSG00000121486	TRMT1L	2.129193784	0.146475623	1.092045767	0.477862788	3.221239551	4.00722531	7.393647515	4.058998049	8.207157886	8.09094333	7.541454591	6.564691065
ENSG00000105327	BBC3	1.116903749	0.283441752	2.103986076	0.02803302	3.220889825	6.96351253	5.72254323	4.523675391	7.053486362	7.20272083	8.526943275	7.480125329
ENSG00000177494	ZBED2	2.211310859	0.502051288	1.007790487	NA	3.219101345	2	2	2	2.947924294	3.28037825	2.384001718	2.782576693
ENSG00000036257	CUL3	1.615137922	0.170042974	1.603668356	0.165345082	3.218806278	5.866944937	9.079034257	6.958202216	9.523001306	9.5188894	9.548120632	9.469883152
ENSG00000174233	ADCY6	1.015279618	0.374685886	2.202779184	0.034100279	3.218058802	6.563806279	8.213429689	9.735974718	9.663322397	9.74027243	10.8739854	10.89774798
ENSG00000205045	SLFN12L	3.665346315	0.147447249	-0.448419963	0.883149421	3.216926352	2	2	2	3.881293667	4.2560182	2.267001569	2.225841198
ENSG00000214274	ANG	1.852947647	0.14259015	1.361979449	0.287006757	3.214927096	2	4.710564154	5.930865648	6.503277904	6.72891807	6.167993056	6.13807518
ENSG00000061676	NCKAP1	1.840330157	0.00515506	1.37364196	0.042119905	3.213972118	8.836089686	10.33384851	9.212024683	11.47440531	11.4175794	11.21251927	10.70353881
ENSG00000165899	OTOGL	0.497902331	0.89458451	2.715591547	0.390470087	3.213493878	2	2	2	2.498691189	2.38539787	2.746468522	3.875378318
ENSG00000160211	G6PD	1.978325781	0.12078466	1.233484174	0.348797592	3.211809955	10.05124847	7.145017454	6.882775709	10.72351064	10.7740351	9.549426	10.35294498
ENSG00000134278	SPIRE1	1.525711641	0.348069747	1.681773262	0.287145127	3.207484903	3.327711234	8.191702463	9.149036566	9.657432896	9.72643218	9.825944587	9.869095369
ENSG00000103126	AXIN1	1.126977345	0.425073921	2.08029856	0.109843769	3.207275905	7.435667355	7.761270542	3.369317972	8.149581172	8.19108423	9.350523544	8.831112735
ENSG00000168944	CEP120	1.796618131	0.494607899	1.409852552	0.592571552	3.206470683	2	7.847167909	2	7.992367495	8.13351654	8.067679772	7.161139769
ENSG00000136052	SLC41A2	2.773917665	0.001021144	0.428207414	0.673751517	3.202125079	5.532353208	4.11352228	6.43197674	8.291333947	8.26379462	6.242195894	5.722580018
ENSG00000115275	MOGS	1.005622917	0.598708746	2.195779027	0.208181348	3.201401944	3.327711234	6.844429755	9.69246155	9.257971441	9.35044873	10.68654814	10.2622961
ENSG00000135926	TMBIM1	1.729050359	4.18E-06	1.472289804	0.000105356	3.201340163	10.11549941	9.338332924	9.926432745	11.49848994	11.6009408	11.25057591	11.33662661
ENSG00000156587	UBE2L6	2.771235371	1.07E-10	0.429636171	0.400966753	3.200871542	9.448362284	8.451443573	8.978369997	11.7599213	11.785461	9.32239471	9.54478665
ENSG00000235109	ZSCAN31	2.712266924	0.156635009	0.487526146	0.82326635	3.19979307	2	5.72254323	2	6.877205252	6.82036165	4.959178705	4.614976609
ENSG00000182010	RTKN2	0.555327983	0.781636058	2.642223993	0.118459494	3.197551976	2	4.11352228	2	3.754611435	3.2184422	4.952879058	5.319231103
ENSG00000154133	ROBO4	0.828347031	0.71541993	2.366368427	0.241080855	3.194715458	4.00722531	2	2	3.349643765	3.50452879	3.878466556	5.028505473
ENSG00000092964	DPYSL2	0.348082994	0.611410824	2.844214067	2.13E-07	3.192297061	9.33149386	9.540933215	9.344432377	9.80923777	9.70047233	11.41205386	12.77179957
ENSG00000058453	CROCC	1.689883544	0.223753194	1.501954196	0.276627835	3.191837741	2	6.311476754	6.43197674	7.467753569	7.42465798	7.41654498	7.091143645
ENSG00000047849	MAP4	0.85643116	0.075173015	2.335106717	5.48E-08	3.191537876	10.81212061	11.46438781	11.81191322	12.2506787	12.2992933	13.65806653	13.84229602
ENSG00000135924	DNAJB2	1.75862777	0.000301529	1.432123083	0.003816216	3.190750852	7.096806373	7.35507212	7.97167868	9.20040236	9.30367823	9.177956973	8.62735196
ENSG00000181104	F2R	1.726928389	0.200056839	1.463397099	0.277052271	3.190325488	8.777722034	7.60566235	4.523675391	9.520570052	9.44390794	9.664973224	8.575745325
ENSG00000184371	CSF1	1.636827642	0.000370047	1.552157628	0.000743084	3.188985269	9.567995545	8.876041558	9.287079172	10.88532146	10.9118811	10.39788531	11.13690957
ENSG00000118804	STBD1	3.029130793	0.019173548	0.159527005	0.922180661	3.188657798	6.257048221	4.442710129	2	7.852449262	8.05152279	5.233158834	5.217771644
ENSG00000278812		4.239394658	0.000129021	-1.051620405	0.419522977	3.187774253	3.327711234	2	4.523675391	7.106324235	7.29937545	2.831336763	3.042026731
ENSG00000137760	ALKBH8	1.915034605	0.106975585	1.270944327	0.295788824	3.185978932	2	5.72254323	5.772716771	7.017154451	7.03319408	6.464399218	6.344709081
ENSG00000132003	ZSWIM4	1.810474987	0.181669225	1.374746603	0.314561325	3.18522159	4.00722531	5.72254323	7.934808822	8.43768081	8.50176361	7.678527483	8.328971229
ENSG00000169992	NLGN2	1.064428208	0.039785638	2.120657946	8.65E-06	3.185096155	8.981092835	8.469613413	9.589475645	10.13584795	10.1432423	11.06990214	11.30663426
ENSG00000176619	LMNB2	1.29018044	0.079160897	1.893450308	0.006446703	3.183630749	7.218819781	8.590771778	7.098165388	9.005540335	9.17609501	9.444446837	9.9049832
ENSG00000251369	ZNF550	1.3949007	0.214356852	1.787794796	0.096172658	3.182695497	3.327711234	6.228547981	5.156626401	6.755670824	6.57678965	7.403899888	6.574984961
ENSG00000262862		8.373406524	3.93E-07	-5.191150377	0.033995019	3.182256146	2	2	2	8.43768081	8.39557285	2	2
ENSG00000155744	FAM126B	1.731634634	0.085637804	1.448483333	0.15050827	3.180117967	4.467311984	7.315436877	6.203069917	8.134820652	8.09094333	8.057440132	7.570355956
ENSG00000140937	CDH11	1.049783193	0.1047992	2.130254815	0.000309103	3.180038009	9.435834703	10.81799725	9.703463497	11.18460494	11.1490131	12.27301174	12.21860406
ENSG00000124370	MCEE	0.98953587	0.561910399	2.189810476	0.158634895	3.179346346	3.327711234	5.946638857	2	5.697663076	5.45984938	6.828438696	6.590289384
ENSG00000110851	PRDM4	1.771598147	0.160124234	1.405663451	0.267319871	3.177261598	4.00722531	7.60566235	8.076950877	9.037634995	9.10406516	8.666781604	8.748827534
ENSG00000084764	MAPRE3	1.792407453	0.187213731	1.379075319	0.314029219	3.171482772	3.327711234	5.838937808	7.600047325	8.14748175	8.21506984	7.709686122	7.837328594
ENSG00000131791	PRKAB2	0.91606495	0.629529096	2.255249125	0.189186264	3.171314075	2	8.169643011	7.691296561	8.258639605	8.29975519	10.08838597	8.88019044
ENSG00000139505	MTMR6	1.765519368	0.316926582	1.405303912	0.428859931	3.17082328	2	7.979858146	8.236946975	9.35577494	9.20831741	9.061649957	8.776966445
ENSG00000034693	PEX3	2.454654629	0.126821984	0.715214433	0.687732077	3.169869062	4.00722531	6.602424674	2	7.748245381	7.54938955	5.722275846	6.195912571
ENSG00000143337	TOR1AIP1	1.747383287	0.063359512	1.422392221	0.133187948	3.169775508	6.138360429	8.623598428	8.763901704	9.969934001	9.95726349	9.719336144	9.556197588
ENSG00000163513	TGFBR2	2.296062866	0.009222762	0.872647727	0.366230344	3.168710593	7.485143553	9.424669273	10.09465441	11.63155624	11.649055	10.0549539	10.36800786
ENSG00000213214	ARHGEF35	2.795738851	0.170233898	0.372113393	0.87553967	3.167852245	2	2	5.772716771	6.887305128	6.88657603	4.360042493	4.911532223
ENSG00000112874	NUDT12	1.062058495	0.646239362	2.10472761	0.325173109	3.166786105	2	6.311476754	2	5.94010643	5.76075522	7.0047514	6.662054875
ENSG00000196924	FLNA	0.226305148	0.787571227	2.940116882	5.89E-06	3.16642203	12.39170213	13.27674723	14.17615456	13.66999705	13.7025084	16.32484542	16.47101995
ENSG00000196456	ZNF775	1.29018586	0.553674554	1.876047422	0.364727545	3.166233282	2	5.946638857	2	5.602782483	5.8245091	6.442083733	6.066922748
ENSG00000134698	AGO4	1.684221933	0.292538621	1.481432204	0.351097101	3.165654137	6.653098118	7.503546079	2	8.23312837	8.22100445	8.169666431	7.869046644
ENSG00000198663	C6orf89	1.206528573	3.23E-07	1.95844029	1.02E-17	3.164968863	9.031919741	9.067150531	9.049077186	10.23062141	10.2698233	11.0815887	10.91418426
ENSG00000162444	RBP7	0.49580458	0.888389865	2.667841846	NA	3.163646426	2	2	2	2.3892346	2.49394783	3.296906311	3.474731315
ENSG00000104765	BNIP3L	1.169517402	0.004862033	1.992151526	3.87E-07	3.161668929	9.602033055	9.936721131	10.23015729	11.22772118	10.9849062	11.64438086	12.17327288
ENSG00000156471	PTDSS1	1.587010714	0.10807059	1.573405455	0.105765803	3.160416169	8.367060731	8.94259736	6.073378139	9.825086737	9.75670736	9.688861377	9.861494087
ENSG00000105245	NUMBL	0.531084102	0.550985513	2.628638951	0.000420382	3.159723052	8.285626337	6.535001748	6.629488387	7.815902688	7.99029569	9.913907765	10.05224558
ENSG00000242252	BGLAP	3.18149027	0.274207949	-0.021934301	0.994944393	3.159555968	2	2	2	3.754611435	3.69849104	2.457042231	2.172608095
ENSG00000154380	ENAH	0.326926092	0.472437308	2.832107976	7.92E-15	3.159034067	9.949165863	10.3486505	9.553445883	10.38561189	10.2428544	12.7687974	12.86450558

ENSG00000135049	AGTPBP1	1.245402354	0.39960716	1.910616191	0.168562587	3.156018545	2	6.666836837	5.772716771	6.965655398	6.91125523	7.452712791	7.708840864
ENSG00000187239	FNBP1	0.313532654	0.823523332	2.842409233	0.01185928	3.155941887	5.330594134	8.903033433	7.029880454	8.063125866	8.08661608	10.52408619	10.64535113
ENSG00000169710	FASN	0.709820827	0.217903746	2.445873053	9.73E-07	3.15569388	9.761125267	10.62479571	9.868959282	10.83761346	10.8598983	12.15744732	12.91067024
ENSG00000162144	CYB561A3	1.890177232	0.000620266	1.264945766	0.027818382	3.155122998	7.435667355	7.189546432	8.380957451	9.596407142	9.65370449	8.808281231	9.176510366
ENSG00000188229	TUBB4B	0.736145645	0.610764042	2.41855295	0.056285331	3.154698595	10.14658276	9.930175364	6.073378139	10.20157308	10.2707772	11.18632706	12.39785952
ENSG00000164989	CCDC171	2.052677102	0.298875829	1.101544266	0.59080501	3.154221367	2	3.686303414	2	4.139692846	4.22485516	3.724264246	3.236138336
ENSG00000168234	TTC39C	1.850742811	0.001743699	1.298847874	0.033210265	3.149590685	5.095959458	5.131603625	5.39244166	6.946463121	6.93069905	6.564892306	6.232137896
ENSG00000104497	SNX16	1.617924239	0.461620063	1.529456903	0.478337468	3.147381142	2	6.04685525	2	6.237202172	5.99120031	6.290458506	5.727202496
ENSG00000131127	ZNF141	1.376220595	0.27007879	1.769675727	0.136351095	3.145896321	2	4.11352228	5.156626401	5.348759107	5.45984938	6.037140174	5.431121909
ENSG00000023909	GCLM	2.17811235	0.023406655	0.965439718	0.351483224	3.143552068	5.532353208	7.467835385	8.460932916	9.747510575	9.72504082	8.402448739	8.651871454
ENSG00000138771	SHROOM3	-0.502078289	0.488943881	3.644632636	3.80E-10	3.142554347	7.33131396	6.602424674	5.59507254	6.237202172	6.1572134	10.25462407	10.28186948
ENSG00000052749	RRP12	1.529845544	0.080997278	1.611530832	0.060381002	3.141376377	4.815609471	6.602424674	7.285536013	8.029321023	8.04707549	8.28197924	7.934467448
ENSG00000019582	CD74	0.595590002	0.722734802	2.545398029	0.078757712	3.14098803	3.327711234	6.311476754	8.206335842	7.546342806	7.61094772	9.953296359	8.855068552
ENSG00000071537	SEL1L	1.65779824	0.038776418	1.483013292	0.064830738	3.140811532	7.624139768	9.733683991	9.553445883	10.93576629	10.8259041	10.74171855	10.67241823
ENSG00000178385	PLEKHM3	1.070037607	0.329903713	2.070509522	0.040228096	3.140547129	4.815609471	6.464272742	7.858115942	7.852449262	7.9408106	8.931137537	8.838608371
ENSG00000151883	PARP8	2.245778966	0.139563643	0.894667073	0.583522797	3.140446039	7.27616306	7.145017454	2	8.868232317	8.83412012	7.366471135	7.657697387
ENSG00000145934	TENM2	0.602496184	0.762843325	2.537206672	0.140420203	3.139702856	2	4.93638933	2	4.299492648	4.12711147	6.205571486	5.513708555
ENSG00000152061	RABGAP1L	1.205999673	0.030197122	1.929745814	0.000214172	3.135745487	5.330594134	6.228547981	6.629488387	7.359462263	7.27295842	8.019511671	8.032963099
ENSG00000156026	MCU	0.817652958	0.514396922	2.31684913	0.036346775	3.134502087	6.653098118	5.131603625	8.434763955	8.219202381	8.01327646	9.645817872	9.564293351
ENSG00000188171	ZNF626	1.780263211	0.016876187	1.351096154	0.075115142	3.131359365	4.467311984	5.131603625	5.156626401	6.722207358	6.47451726	6.490730008	5.821002687
ENSG00000126391	FRMD8	1.335496608	0.39305959	1.794143135	0.228416028	3.129639742	3.327711234	8.124485672	8.408111533	9.037634995	9.02486072	8.958123037	9.873011408
ENSG00000140262	TCF12	1.865944446	0.020534986	1.262610452	0.128777677	3.128554898	8.367060731	9.479495904	7.285536013	10.51378677	10.4971286	9.975607996	9.829070247
ENSG00000171603	CLSTN1	1.493908468	0.000723989	1.632825692	0.000179948	3.126734161	9.435834703	10.24169813	10.34701993	11.54707907	11.5582157	11.55836616	11.81320514
ENSG00000163884	KLF15	-2.312004462	0.48457279	5.437613979	0.023854618	3.125609517	2	2	2	2	2.14022021	5.545162502	5.631815919
ENSG00000099365	STX1B	2.315991592	0.226093212	0.807337786	0.696969597	3.123329377	2	5.457120953	2	6.221320894	6.18985553	4.5389195	5.080008298
ENSG00000261587	TMEM249	3.936331056	0.119879274	-0.813188228	0.784238084	3.123142827	2	2	2	4.139692846	4.43005853	2.267001569	2.117335275
ENSG00000123983	ACSL3	1.904024408	0.05610284	1.218726581	0.237056099	3.122750989	6.468621203	9.377333006	8.236946975	10.37712591	10.3332939	9.42067733	9.887279953
ENSG00000131507	NDFIP1	1.757273518	9.86E-08	1.364266033	4.68E-05	3.121539551	8.468948322	9.090820894	8.99637432	10.64904058	10.6095994	10.27670249	10.19735668
ENSG00000179546	HTR1D	0.725878283	0.771079266	2.393851486	0.272841778	3.119729769	2	2	3.369317972	2.695456494	3.01487888	4.133696865	3.496556178
ENSG00000172575	RASGRP1	-2.512714196	0.034445258	5.630001759	7.62E-09	3.117287563	5.095959458	3.686303414	2	2.695456494	2.59489879	9.650204809	8.91098986
ENSG00000212719	C17orf51	1.28933874	0.441837321	1.827746458	0.249115502	3.117085198	6.366709671	7.431218269	2	7.555872146	7.81828345	8.593249736	7.722795734
ENSG00000167861	HID1	-2.272313756	0.318802002	5.388292613	0.001147244	3.115978857	2	3.076665209	2	2.141737246	2.14022021	6.670403488	6.175763085
ENSG00000153317	ASAP1	1.215145914	0.344412784	1.899543476	0.115663273	3.11468939	6.366709671	9.956182102	10.28977157	10.83272206	10.7887607	11.3875211	11.59331975
ENSG00000087008	ACOX3	0.694260029	0.52990438	2.419589281	0.01125466	3.11384931	4.00722531	6.844429755	6.958202216	7.097651217	7.07311172	8.757226889	8.804020138
ENSG00000111371	SLC38A1	1.215754325	0.042514667	1.897463897	0.00077242	3.113218222	7.904282243	8.433041974	9.301632639	9.847245515	9.88808233	10.54085267	10.55349898
ENSG00000173064	HECTD4	1.338988539	0.024718288	1.774177207	0.001976772	3.113165747	9.27634997	8.101365653	8.110401118	9.909274163	9.96140527	10.49050086	10.23679159
ENSG00000110172	CHORDC1	1.748694895	0.356811536	1.364257323	0.474542652	3.112952218	2	7.189546432	4.058998049	7.507583254	7.47187556	7.535143176	6.520103598
ENSG00000255112	CHMP1B	2.075936778	0.003866084	1.035444671	0.177968622	3.111381449	9.112843761	7.274681896	7.896971941	10.36136198	10.3568427	9.261890418	9.382110089
ENSG00000167258	CDK12	1.703701439	0.00072502	1.407570492	0.006022874	3.111271931	8.075571721	7.874700259	8.960138144	10.03554381	10.1014956	9.842651775	9.701867195
ENSG00000120334	CENPL	1.448339037	0.267906024	1.662020766	0.188368879	3.110359803	3.327711234	5.946638857	3.369317972	6.229283386	6.05460702	6.442083733	6.245088435
ENSG00000033030	ZCCHC8	1.610344423	0.105038947	1.495251461	0.128390822	3.105595883	4.467311984	6.140560021	7.225742779	7.772969103	7.99953197	8.042684843	7.452476174
ENSG00000130522	JUND	0.982717236	0.067009569	2.122408156	1.40E-05	3.105125392	8.75772933	9.856140801	9.647595056	10.44194384	10.5056622	11.40348951	11.79350523
ENSG00000221890	NPTXR	0.367646493	0.877879848	2.73649753	0.165909543	3.104144023	3.327711234	7.638155693	2	6.516352584	6.51372389	8.723392661	8.933165411
ENSG00000189410	SH2D5	1.397532436	0.319054857	1.705675355	0.206700396	3.103207791	3.327711234	4.11352228	6.718950348	6.699459234	6.81514011	6.907119087	7.191563076
ENSG00000103034	NDRG4	3.087594726	0.127598657	0.015520842	0.994944393	3.103115568	2	6.228547981	2	7.772969103	7.66419009	5.102662916	4.511590831
ENSG00000175305	CCNE2	1.77137633	0.458136023	1.33006145	0.576450932	3.101437779	2	3.076665209	2	3.349643765	3.50452879	3.44788957	2.817676617
ENSG00000162222	TTC9C	1.742105539	0.33524083	1.358228695	0.455315905	3.100334234	4.815609471	7.538394143	2	7.947219213	7.8208909	7.667989909	7.330190599
ENSG00000108557	RAI1	1.65881768	0.410884798	1.441231161	0.47216327	3.100048842	2	7.954278496	9.589475645	10.02529042	10.0923519	9.658455026	10.00568113
ENSG00000101350	KIF3B	1.321676713	0.129712892	1.776313661	0.03302563	3.097990375	8.009475818	9.726146444	7.896971941	10.07252815	10.1772163	10.22094162	10.86574466
ENSG00000082898	XPO1	2.132119181	0.003707011	0.964536394	0.224818109	3.096655575	7.82963488	9.849218119	9.358421373	11.40931048	11.3114888	10.32567245	10.0543164
ENSG00000165733	BMS1	1.814578119	0.003642609	1.28121448	0.047023433	3.095792599	6.653098118	8.124485672	8.110401118	9.565291356	9.58019637	8.860117405	9.204958522
ENSG00000128607	KLHDC10	1.281418267	0.370174297	1.813345371	0.180586512	3.094763638	4.00722531	8.687092496	7.734844356	9.080080551	8.93625225	9.55463569	9.522022954
ENSG00000274611	TBC1D3	8.283594897	7.42E-07	-5.191150183	0.035315165	3.092444713	2	2	2	8.302698479	8.35297033	2	2
ENSG00000068394	GPKOW	1.531667536	0.487909731	1.560652422	0.470583967	3.092319958	3.327711234	7.847167909	2	7.821180652	7.86703672	7.684242973	8.039482273
ENSG00000169313	P2RY12	4.083114711	0.094962877	-0.994016814	0.726496359	3.089097897	2	2	2	4.598938466	4.1930041	2.225793514	2.117335275
ENSG00000105559	PLEKHA4	2.801995215	3.35E-05	0.286699249	0.732333373	3.088694465	8.816894989	7.35507212	9.099922433	11.37876293	11.3862332	8.56564741	9.133628617
ENSG00000114013	CD86	-1.310497508	0.677345605	4.399110958	0.073198426	3.088613449	2	2	2	2.141737246	2.14022021	5.119635168	4.018014792
ENSG00000205572	SERF1B	8.278026389	7.69E-07	-5.191150175	0.035365736	3.086876214	2	2	2	8.304583895	8.34030387	2	2
ENSG00000187630	DHRS4L2	1.941512121	0.177844856	1.144727819	0.442622978	3.08623994	2	4.93638933	6.43197674	7.097651217	7.20671394	6.022174708	6.676467423
ENSG00000277974		8.275165251	7.89E-07	-5.191150164	0.035434326	3.084015088	2	2	2	8.293234263	8.34574597	2	2
ENSG00000278299	TBC1D3C	8.275165251	7.89E-07	-5.191150164	0.035434326	3.084015088	2	2	2	8.293234263	8.34574597	2	2
ENSG00000011405	PIK3C2A	1.695122148	0.045753442	1.388714652	0.104797108	3.0838368	6.366709671	8.687092496	8.325066185	9.783781964	9.75874862	9.621944737	9.292162928
ENSG00000174059	CD34	1.119640448	0.680321745	1.963435452	0.435161964	3.0830759	2	2	3.369317972	3.023133209	3.08597292	2.68698273	4.09155917
ENSG00000115825	PRKD3	2.017413346	0.002635445	1.064437907	0.135544734	3.081851253	8.981092835	7.393647515	7.691296561	10.15164584	10.2360327	9.242329292	9.249822
ENSG00000070159	PTPN3	-2.424045793	0.099653945	5.504713225	7.53E-07	3.080667432	2	3.076665209	4.058998049	2.270786764	2.38539787	8.04490778	7.930464419
ENSG00000156869	FRRS1	2.07026914	0.12909603	1.008562002	0.483213396	3.078831142	2	3.076665209	4.874587708	5.461211713	5.44640346	4.596630906	4.456980017
ENSG00000138380	CARF	1.922716366	0.005958463	1.155803344	0.114203801	3.078519709	5.095959458	4.710564154	4.874587708	6.687949235	6.63721363	6.181526565	5.636738041
ENSG00000282087		8.269521542	8.20E-07	-5.191150151	0.035498586	3.078371391	2	2	2	8.287525788	8.34030387	2	2
ENSG00000141424	SLC39A6	1.817909711	0.119309305	1.260079379	0.290138932	3.07798909	6.138360429	9.842262059	8.922968322	10.77711405	10.7271979	10.16345022	10.22805302
ENSG00000011028	MRC2	1.913487605	6.77E-27	1.164091828	1.22E-10	3.077579433	11.26581873	11.40661699	11.38635888	13.23212001	13.3004149	12.53234403	12.50284941
ENSG00000154727	GABPA	1.819864845	0.239927272	1.257690958	0.426580825	3.077555802	2	6.728495673	7.225742779	8.209172298	8.20711886	7.872065621	7.399884913
ENSG00000275153		8.268573456	8.26E-07	-5.191150148	0.035498785	3.077423308	2	2	2	8.285617933	8.34030387	2	2
	LOC101060[illegible]	8.268573456	8.26E-07	-5.191150148	0.035498785	3.077423308	2	2	2	8.285617933	8.34030387	2	2
ENSG00000143479	DYRK3	2.504819184	0.165298109	0.57071095	0.778729259	3.075530134	8.16936288	5.457120953	2	9.258944141	9.27258642	7.426809156	7.285445324
ENSG00000143162	CREG1	2.201017869	5.60E-05	0.872724827	0.144905041	3.073742697	8.963744667	7.901717006	7.818184403	10.50438458	10.5189709	9.356203439	9.0005841
ENSG00000069869	NEDD4	2.132358276	0.200056839	0.940604686	0.594921579	3.072962962	7.867441304	6.535001748	2	8.846395982	8.90095288	8.032265607	7.261749957
ENSG00000119537	KDSR	1.862210167	2.17E-12	1.209028442	8.59E-06	3.071238609	8.228665467	8.276714465	8.434763955	10.22615207	10.096662	9.561380283	9.461931741
ENSG00000147874	HAUS6	1.795062027	0.100196808	1.275743301	0.251602132	3.070805328	4.00722531	6.04685525	7.285536013	8.091796043	7.99953197	7.675661224	7.382408159
ENSG00000168488	ATXN2L	1.511308239	1.87E-09	1.559127061	4.43E-10	3.070435301	8.716893051	8.671479029	8.884815469	10.23458257	10.2845385	10.37656901	10.23476408
ENSG00000148484	RSU1	1.035327784	0.000888639	2.032293662	4.35E-12	3.067621447	8.609439662	8.834582582	8.904018013	9.829021922	9.80557618	10.63066452	10.97155887
ENSG00000090470	PDCD7	1.359872287	0.16375923	1.705967992	0.068587835	3.065840279	4.815609471	7.002537973	7.502634626	8.145379268	8.14188248	8.751340201	8.158913605
ENSG00000198793	MTOR	1.223706216	0.000107077	1.841882538	1.39E-09	3.065588754	8.042902253	8.57407404	8.325066185	9.562930393	9.54040713	10.14336241	10.19067811
ENSG00000075399	VPS9D1	1.698964941	0.292000423	1.360409629	0.398956117	3.05937457	7.579279349	6.535001748	2	8.254744042	8.23084153	7.88783736	7.930464419
ENSG00000107758	PPP3CB	0.891548837	0.433930421	2.167519725	0.033633667	3.059068562	5.330594134	8.540087354	7.734844356	8.634896	8.54182783	9.764548842	9.942486645
ENSG00000182197	EXT1	1.40258299	0.012710848	1.656465928	0.002554195	3.059048918	10.59831544	10.23108619	9.287079172	11.52136866	11.5517552	11.61737522	11.94454653
ENSG00000068976	PYGM	-0.464372904	0.802617873	3.522924727	0.017580275	3.058551823	2	4.710564154	2	3.09461492	3.50452879	6.615624322	6.622902297
ENSG00000258644	SYNJ2BP-CO[illegible]	8.248820207	9.40E-07	-5.191150112	0.035746075	3.057670095	2	2	2	8.29892024	8.28849576	2	2
ENSG00000167182	SP2	1.136415155	0.231366574	1.920982143	0.03002915	3.057397298	6.89194353	6.844429755	4.523675391	7.517371202	7.54308677	7.912394292	8.60805852
ENSG00000103599	IQCH	1.435999456	0.481906026	1.621306168	0.409695273	3.057305624	2	3.686303414	2	3.79808662	3.65239003	4.017766949	3.67940853
ENSG00000146021	KLHL3	3.337599882	0.003692538	-0.280483606	0.845736528	3.057116276	3.327711234	5.131603625	2	7.097651217	7.14977896	4.005644654	3.807156736
ENSG00000215187	FAM166B	-1.316847075	0.744846249	4.373650241	0.191114239	3.056803166	2	2	2	2	2.2680111	4.785723309	4.500832793
ENSG00000247626	MARS2	1.581667375	0.330982566	1.47437911	0.359439631	3.056046485	2	5.838937808	3.369317972	6.061031724	6.02777321	6.132200015	5.727202496
ENSG00000159228	CBR1	1.744931257	0.000373498	1.310982387	0.009285622	3.055913644	7.485143553	8.487557258	8.631919287	10.04685164	9.98775074	9.589571269	9.581626682
ENSG00000126368	NR1D1	2.499315824	0.085143446	0.555281488	0.734896874	3.054597311	9.081016759	5.303523747	5.39244166	10.13372843	10.2084185	8.247007147	8.236348546
ENSG00000173852	DPY19L1	1.822552495	0.140480229	1.230582365	0.330230765	3.05313486	5.095959458	8.505280662	6.203069917	9.1138055	9.09224234	8.432756021	8.595472797
	NARR	8.244231739	9.99E-07	-5.191150075	0.035936624	3.053081664	2	2	2	8.328872678	8.248381	2	2
ENSG00000135218	CD36	0.151052059	0.954430302	2.90144188	0.185967679	3.052493939	3.327711234	2	3.369317972	3.162721474	2.94009902	5.870008604	2.918126211
ENSG00000109079	TNFAIP1	0.062077921	0.96300973	2.989986037	0.004649784	3.052063959	9.940323746	9.433952963	6.43197674	9.225498809	9.29244949	12.02479168	12.31842831
ENSG00000162817	C1orf115	0.342943731	0.842753717	2.708350396	0.054562067	3.051294127	2	5.595927442	4.058998049	4.907595604	4.811949	6.126613714	7.617180085
ENSG00000162924	REL	1.871703693	0.017393053	1.178624655	0.149212315	3.050328348	4.467311984	6.464272742	5.39244166	7.605658789	7.37229174	6.867478139	6.771278116
ENSG00000266094	RASSF5	0.791701709	0.645184945	2.257872853	0.139643037	3.049574561	3.327711234	5.838937808	2	5.540148523	5.04390545	6.625560108	6.739599323
ENSG00000163701	IL17RE	0.340532129	0.871671212	2.708252683	0.112770502	3.048784812	2	4.11352228	2	3.462030052	3.28037825	4.720468739	5.566251551
ENSG00000116871	MAP7D1	1.090396929	0.035977572	1.958376397	5.08E-05	3.048773325	9.358295023	9.792607274	10.49998282	11.00233175	11.0915972	11.90263765	11.92606907
ENSG00000139218	SCAF11	0.96351038	0.157701036	2.08394333	0.000732621	3.047453709	8.653385977	10.25223258	9.465702942	10.55158859	10.5760362	11.56683474	11.7890907
ENSG00000174579	MSL2	1.450908327	0.453416873	1.595575369	0.393866241	3.046483696	2	4.710564154	7.734844356	7.650823203	7.8208909	8.030023089	7.713507501
ENSG00000184216	IRAK1	1.668925781	0.001995992	1.375625725	0.012347695	3.044551506	9.2903348	9.067150531	10.23015729	11.23614254	11.3278511	10.84981999	11.11767423
ENSG00000166012	TAF1D	1.33857129	0.094280197	1.701974389	0.026587498	3.04054568	7.624139768	6.04685525	5.930865648	8.060896687	8.04707549	8.547470747	8.26365177

ENSG00000218336	TENM3	2.056129334	0.311473389	0.98015118	0.646868487	3.036280513	5.095959458	8.623598428	2	9.225498809	9.18320541	7.861174222	8.372406522
ENSG00000110031	LPXN	4.871090537	0.001952423	-1.835219961	0.289910617	3.035870576	4.00722531	4.11352228	8.434763955	11.78326477	11.8212241	5.737015686	4.522269241
ENSG00000104687	GSR	1.945657821	0.349594603	1.090114584	0.61339599	3.035772405	8.998234871	5.457120953	2	9.484432763	9.44221542	8.681143984	8.544657203
ENSG00000005810	MYCBP2	1.979897114	0.007220064	1.050608998	0.17880563	3.030506112	8.816894989	8.876041558	6.958202216	10.35864221	10.4716357	9.51268942	9.468503442
ENSG00000186318	BACE1	1.49181805	0.000460725	1.538438352	0.000269051	3.030256401	8.797441463	8.916342327	8.042706615	10.0993857	10.144804	10.28315082	10.04439554
ENSG00000177679	SRRM3	0.301026642	0.858274786	2.729177194	0.044174878	3.030203836	5.866944937	3.686303414	2	4.907595604	4.87352701	7.042323222	7.252154709
ENSG00000124343	XG	8.220939248	1.15E-06	-5.191150033	0.036232528	3.029789215	2	2	2	8.291333947	8.240612	2	2
ENSG00000274791	F8A2	8.220466611	1.21E-06	-5.191149991	0.036373066	3.029316619	2	2	2	8.207157886	8.3220138	2	2
ENSG00000277150	F8A3	8.220466611	1.21E-06	-5.191149991	0.036373066	3.029316619	2	2	2	8.207157886	8.3220138	2	2
ENSG00000042832	TG	3.846210474	0.067133789	-0.817590789	0.73782085	3.028619684	2	3.076665209	2	4.965943888	5.07918227	2.139668783	2.551896972
ENSG00000186847	KRT14	-3.229474305	0.156783493	6.2577098	0.000264451	3.028235495	2	2	4.058998049	2.270786764	2	7.44824109	8.72879856
ENSG00000134285	FKBP11	2.670839676	0.000124594	0.357069745	0.674724562	3.027909421	7.667647223	7.099070191	5.59507254	9.640365415	9.66898051	7.319501608	7.417152486
ENSG00000152767	FARP1	0.657285673	0.667164929	2.370106338	0.075077708	3.02739201	6.816637977	9.863030423	6.073378139	9.216514298	9.1617682	10.97536401	10.81059428
ENSG00000213390	ARHGAP19	1.180166259	0.487140735	1.846314164	0.246641741	3.026480423	2	5.946638857	3.369317972	5.786686927	5.73886209	6.353989995	6.423723772
ENSG00000078814	MYH7B	0.429740808	0.867562397	2.596671108	0.220732836	3.026411916	2	3.076665209	2	2.868578001	2.59489879	4.155805465	3.824516949
ENSG00000153237	CCDC148	0.015684726	0.997041173	3.009269496	0.331063165	3.024954222	2	2	2	2.498691189	2.14022021	3.44788957	3.753786374
ENSG00000156172	C8orf37	0.725760001	0.514678379	2.298906217	0.016733631	3.024666218	4.815609471	3.686303414	3.369317972	4.470469939	4.83276841	5.922534244	6.215784511
ENSG00000131089	ARHGEF9	-0.178082364	0.911633612	3.199558121	0.010279412	3.021475757	3.327711234	7.232742069	7.858115942	6.882264028	6.83591385	10.05219399	10.31813217
ENSG00000136059	VILL	1.733217968	0.214218894	1.288001911	0.360920918	3.02121988	3.327711234	4.93638933	2	5.194621444	5.62411716	5.248671144	4.766376438
ENSG00000182667	NTM	2.372084227	0.205285997	0.648800587	0.75370958	3.020884815	6.257048221	2	7.934808822	9.074566443	9.08900105	8.008923105	6.251520369
ENSG00000138463	DIRC2	1.427352618	0.466982135	1.592226903	0.401725514	3.019579521	2	7.701025996	4.523675391	7.671425626	7.70145652	7.732851095	7.955303035
ENSG00000166886	NAB2	1.518505827	0.002152817	1.500463068	0.002335708	3.018968895	7.904282243	7.467835385	8.561105505	9.508351943	9.57634363	9.40316733	9.63702421
ENSG00000126858	RHOT1	1.598548807	0.398338237	1.420157104	0.449551169	3.018705912	6.138360429	8.376385542	2	8.710792067	8.60823803	8.818278819	8.047821014
ENSG00000073146	MOV10L1	1.4834135	0.52563759	1.534255736	0.496731642	3.017669237	2	3.076665209	2	3.349643765	3.15372745	3.355392153	3.209956562
ENSG00000165494	PCF11	2.041286071	0.000236772	0.975246255	0.103004472	3.016532327	7.709880939	7.538394143	6.80318718	9.381905119	9.45821469	8.731695932	7.868000534
ENSG00000139899	CBLN3	1.791792236	0.45900055	1.224674195	0.617024792	3.016466431	2	7.002537973	2	7.178012736	7.26532099	6.52722803	6.806650651
ENSG00000213341	CHUK	1.969665388	6.05E-06	1.046706362	0.023911633	3.01637175	6.653098118	6.602424674	6.882775709	8.70652031	8.57000035	7.953993861	7.463599504
ENSG00000169136	ATF5	2.610689615	3.96E-06	0.405128474	0.550387484	3.01581809	8.797441463	7.431218269	7.777116083	10.71112597	10.7114757	8.443467705	8.589138522
ENSG00000179242	CDH4	4.73959828	9.76E-06	-1.723822982	0.160349824	3.015775299	3.327711234	2	4.523675391	7.745471964	7.63485389	2.746468522	2.551896972
ENSG00000243207	PPAN-P2RY1	8.205535819	1.40E-06	-5.19114992	0.036768558	3.014385899	2	2	2	8.323303759	8.17486938	2	2
ENSG00000159459	UBR1	1.718805332	0.352202552	1.292700591	0.487618515	3.011505923	2	7.73146271	8.763901704	9.455026484	9.46822882	9.130251861	8.940150381
ENSG00000179562	GCC1	1.410737864	0.620468344	1.600010763	0.564099325	3.010748626	2	8.687092496	2	8.48998335	8.54813611	8.829927393	8.571895748
ENSG00000068097	HEATR6	1.187280873	0.476737643	1.822429944	0.245051346	3.009710817	4.00722531	7.874700259	4.058998049	7.590284224	7.6986238	8.301943231	8.237969002
ENSG00000054523	KIF1B	1.239287775	8.10E-07	1.770395413	4.21E-13	3.009683188	8.716893051	8.862353853	8.585098385	9.954406146	9.97141463	10.49403038	10.49048921
ENSG00000105397	TYK2	1.338482146	0.48903311	1.669877245	0.367889318	3.008359391	8.313285031	9.067150531	2	9.429335066	9.54672161	9.810131135	9.82772573
ENSG00000134247	PTGFRN	0.515132849	0.698766691	2.490654101	0.027497225	3.00578695	5.330594134	8.834582582	8.904018013	8.811020771	8.90095288	10.58734728	11.02533189
ENSG00000131398	KCNC3	-3.753139074	0.256437356	6.758319752	0.007323777	3.005180679	2	2	2	2	2	6.950450741	6.711881188
ENSG00000165490	DDIAS	2.060358502	0.307879292	0.943492214	0.658783502	3.003850716	2	3.686303414	2	4.497095146	3.7864959	3.500668998	3.287118176
ENSG00000198353	HOXC4	4.844761182	2.60E-05	-1.844702684	0.155606188	3.000058498	4.467311984	4.11352228	2	8.304583895	8.26762243	3.12825509	2.374681325
ENSG00000133612	AGAP3	1.325540086	0.486515971	1.674084493	0.359872083	2.999624579	8.797441463	8.469613413	2	9.319871109	9.43542545	9.8644215	9.571701541
ENSG00000272325	NUDT3	2.735165094	0.01977227	0.261345905	0.855356794	2.996510999	2	6.04685525	5.59507254	7.939959586	7.92150349	5.390603804	5.713290392
ENSG00000196935	SRGAP1	2.074609006	0.059194088	0.921819938	0.434090596	2.996428944	8.340423424	7.145017454	4.874587708	9.374743937	9.45234084	8.254069715	8.28888532
ENSG00000106351	AGFG2	0.688830115	0.651205609	2.307091323	0.084128005	2.995921438	2	6.04685525	4.523675391	5.540148523	5.6826301	6.865802559	7.386797235
ENSG00000134020	PEBP4	-0.727649172	0.837764017	3.722083119	0.189633354	2.994433947	2	2	2	2.141737246	2.2680111	4.659878161	3.261853426
ENSG00000126353	CCR7	-1.310500242	0.668579277	4.30478236	0.070233601	2.994282119	2	2	2	2.141737246	2.14022021	4.406883583	4.757365452
ENSG00000119403	PHF19	0.265866542	0.885649929	2.727173883	0.066744424	2.993040425	5.532353208	7.232742069	2	6.463327516	6.18985553	8.75224741	8.716646332
ENSG00000120008	WDR11	1.402012553	0.372007399	1.590932431	0.295637434	2.992944984	3.327711234	8.687092496	8.296287027	9.245266304	9.40880824	9.518299895	9.516025051
ENSG00000271321	CTAGE6	5.839248142	0.013180916	-2.84661821	0.320401275	2.992629932	2	2	2	5.978405189	5.94414054	2.094598369	2
ENSG00000174456	C12orf76	1.039912515	0.517016221	1.950113329	0.185017104	2.990025844	2	5.595927442	3.369317972	5.44762523	5.22805098	6.21875862	6.148451724
ENSG00000151693	ASAP2	1.637379061	0.10807059	1.351887694	0.185412187	2.989266755	5.70932728	6.844429755	8.486635645	9.018234236	9.10620439	8.823035893	8.735123394
ENSG00000082196	C1QTNF3	1.987246121	0.259802912	1.001367237	0.587346695	2.988613358	2	3.076665209	3.369317972	4.299492648	4.0930011	3.709392871	3.236138336
ENSG00000166228	PCBD1	0.714424984	0.559383109	2.27396168	0.03472132	2.988386664	4.467311984	7.901717006	6.882775709	7.725907304	7.65252709	9.044347772	9.395603187
ENSG00000102854	MSLN	0.279381352	0.943520433	2.708508551	NA	2.987889903	2	2	2	2.600425781	2.14022021	2.937246078	3.771796761
ENSG00000277443	MARCKS	1.596454329	0.036506808	1.390921256	0.069687163	2.987375585	9.081016759	10.80015962	10.29705308	11.82638973	11.8063345	11.00639811	12.03593134
ENSG00000282185		-0.323181186	0.863478741	3.310292978	0.024438946	2.987111793	2	4.710564154	2	3.406930997	3.39680241	6.351597515	6.482320745
ENSG00000015171	ZMYND11	1.254422611	0.459689613	1.732404009	0.280587322	2.98682662	3.327711234	8.147241016	9.301632639	9.53348965	9.48723044	10.08298767	9.882625765
ENSG00000078304	PPP2R5C	1.104527194	0.27893875	1.8822516	0.047119088	2.986778794	7.624139768	8.94259736	6.203069917	9.125585405	9.0561812	9.951322636	9.772331943
ENSG00000146909	NOM1	1.150050318	0.509715065	1.836126306	0.260870186	2.986176624	3.327711234	5.131603625	8.296287027	8.056427969	7.98565528	8.785862385	8.606804877
ENSG00000168676	KCTD19	0.012816003	0.997163678	2.972891288	0.307389297	2.985707291	2	2	2	2.3892346	2.2680111	3.296906311	3.824516949
ENSG00000111490	TBC1D30	-0.307135341	0.93146871	3.292818809	0.25649001	2.985683468	2	2	2	2.3892346	2.14022021	3.968654369	3.620995423
ENSG00000085377	PREP	1.653046962	0.167653704	1.331125875	0.268158791	2.984172837	4.815609471	8.395519306	6.882775709	8.918344554	9.01805981	8.431623835	8.839676004
ENSG00000091127	PUS7	1.421968731	0.181069609	1.561771204	0.130056692	2.983739935	3.327711234	5.838937808	6.534109959	7.03996889	7.13309042	7.196918221	7.24894374
ENSG00000006451	RALA	2.641968903	2.16E-09	0.341712395	0.524228701	2.983681298	8.696033109	8.101365653	8.805342542	11.23144255	11.1448568	9.189724501	8.532164007
ENSG00000144909	OSBPL11	1.507105566	0.179604546	1.475828495	0.181024624	2.98293406	5.70932728	8.029695761	5.39244166	8.361842177	8.40255255	8.471832957	8.220043091
ENSG00000120868	APAF1	2.056712236	0.11481363	0.925714857	0.504387978	2.982427092	7.384433967	5.946638857	3.369317972	8.449657898	8.16874151	7.603095107	6.657218517
ENSG00000140836	ZFHX3	0.638704827	0.686208373	2.343372984	0.088390629	2.982077812	5.095959458	9.549500232	7.098165388	8.880924505	8.91566441	10.46914042	10.71012106
ENSG00000204160	ZDHHC18	1.326354984	0.343731455	1.655598575	0.218712264	2.98195356	8.564112515	7.145017454	4.058998049	8.780186674	8.79884455	9.202060967	9.024723418
ENSG00000080824	HSP90AA1	1.396408003	9.99E-10	1.58481909	2.44E-12	2.981227093	11.48534568	11.72102722	11.38976845	12.93879979	12.9321918	13.10118024	13.14611335
ENSG00000214013	GANC	1.781680448	0.291638623	1.197745587	0.485369749	2.979426035	7.82963488	6.311476754	2	8.458152491	8.41468635	7.667989909	8.031095054
ENSG00000156639	ZFAND3	1.097543782	0.053991261	1.879930899	0.000395857	2.977474682	9.384607364	9.328390744	8.143093325	10.1220151	10.1741606	10.91689319	10.94017357
ENSG00000100941	PNN	1.892537972	0.001161823	1.083787728	0.078447389	2.976325701	8.257427002	8.791896851	7.285536013	10.15112202	10.0971999	9.352020423	9.286301649
ENSG00000132155	RAF1	1.256474995	0.008781104	1.718431523	0.000188952	2.974906518	8.836089686	8.213429689	9.25752426	10.10642804	10.0390409	10.41072665	10.64687673
ENSG00000101109	STK4	1.650908382	0.178186326	1.321483892	0.28290231	2.972392274	4.815609471	8.687092496	8.143093325	9.55265467	9.54986851	9.306733157	9.135803248
ENSG00000100079	LGALS2	-2.307876711	0.495905043	5.279750861	0.034674478	2.97187415	2	2	2	2.141737246	2	5.390603804	5.497565112
ENSG00000105778	AVL9	1.682661917	0.00326405	1.288692248	0.028067889	2.971354165	8.313285031	7.503546079	6.958202216	9.383689875	9.34048844	9.12500825	8.798540026
ENSG00000261221	ZNF865	0.587797955	0.298074813	2.381944371	6.27E-07	2.969742326	7.579279349	6.78762695	6.629488387	7.539954763	7.7127321	9.222499293	9.562031113
ENSG00000146678	IGFBP1	-2.072956259	0.307942408	5.042484217	0.001703637	2.969527958	2	3.686303414	2	2.270786764	2.2680111	6.134985075	7.370638252
ENSG00000012232	EXTL3	0.973658171	0.271011111	1.995697363	0.013555764	2.969355533	9.544849385	7.638155693	7.858115942	9.580155061	9.58173459	10.44409424	10.73916056
ENSG00000155265	GOLGA7B	0.497949615	0.915839444	2.470592243	NA	2.968541858	2	2	2	2.498691189	2.38539787	3.917810786	2.059860178
ENSG00000240694	PNMA2	-2.634480682	0.01733931	5.603018159	1.73E-08	2.968537477	4.815609471	5.595927442	6.718950348	4.237680128	2.86123014	10.67080105	11.91008607
ENSG00000186212	SOWAHB	-2.307857601	0.496953626	5.275409601	0.03552107	2.967552	2	2	2	2.141737246	2	5.304163849	5.566251551
	ATP5J2-PTCD	8.158179723	1.81E-06	-5.191149865	0.037234619	2.967029858	2	2	2	8.229163214	8.17894021	2	2
ENSG00000148331	ASB6	1.365954596	0.225370868	1.601039251	0.141569199	2.966993846	4.815609471	7.467835385	8.206335842	8.657201865	8.79221631	8.943899495	8.974576781
ENSG00000247077	PGAM5	0.757789377	0.379217697	2.208466303	0.003474236	2.96625568	5.866944937	6.228547981	7.734844356	7.552702688	7.59884443	9.069678103	8.94213989
ENSG00000175213	ZNF408	1.407611178	0.460238382	1.557728687	0.397767254	2.965339864	4.00722531	7.189546432	2	7.127781374	7.17852729	7.385306888	7.208191747
ENSG00000183439	TRIM61	3.212285742	0.088950597	-0.247130029	0.914220926	2.965155713	2	3.686303414	2	5.058234798	5.24368216	3.035909414	2.466008509
ENSG00000128791	TWSG1	1.450916905	0.011158168	1.51410836	0.00747349	2.965025265	8.367060731	9.574900212	9.61300511	10.76244194	10.7069016	10.6229975	10.95404265
ENSG00000165801	ARHGEF40	1.127698759	0.237333582	1.837286557	0.039733569	2.964985316	7.750913381	9.608085847	7.552162999	9.691694368	9.80557618	10.12246481	10.7279608
ENSG00000182541	LIMK2	2.117863655	0.000122541	0.846326309	0.161125537	2.964189963	8.797441463	7.572420273	7.734844356	10.17347624	10.3042388	8.976484087	8.974576781
ENSG00000278326		8.151191053	1.97E-06	-5.191149816	0.037509667	2.960041237	2	2	2	8.248880905	8.14396641	2	2
ENSG00000171155	C1GALT1C1	2.113652168	0.005097116	0.846387752	0.304133236	2.96003992	7.096806373	6.535001748	8.434763955	9.680848002	9.64710779	8.265552582	8.535462193
ENSG00000156049	GNA14	-1.137865377	0.431178842	4.096821087	0.000445233	2.95895571	3.327711234	3.686303414	3.369317972	2.695456494	3.01487888	6.287958221	7.467748741
ENSG00000152492	CCDC50	1.149239406	0.138334654	1.807897097	0.013077994	2.957136503	9.858207046	9.28800521	7.896971941	10.3905021	10.3428503	10.84344883	11.18432523
ENSG00000214814	FER1L6	4.046953687	0.318639797	-1.092861105	0.809485785	2.954092582	2	2	2	4.387512679	4.37434574	2.3070652	2
ENSG00000239836		9.10590246	1.12E-10	-6.152947053	0.005662518	2.952955407	3.327711234	2	2	10.04685164	10.1499977	2	2
ENSG00000053254	FOXN3	0.540246659	0.756618096	2.410972976	0.108572237	2.951219635	4.00722531	8.655694736	9.767769415	9.299216392	9.27068036	11.16621714	11.13157999
ENSG00000164323	CFAP97	0.834980095	0.483350437	2.113693147	0.047119088	2.948673242	7.031698319	5.72254323	8.805342542	8.621345041	8.43695333	9.831105898	9.771493021
ENSG00000067167	TRAM1	1.918718128	0.001679195	1.029874519	0.112494976	2.948592646	9.015175615	9.896993485	10.5916593	11.94324566	11.8270724	11.04910471	10.94662937
ENSG00000163946	FAM208A	1.244455741	0.08383018	1.70255654	0.013108075	2.947012281	7.624139768	9.030900487	7.646393253	9.566077486	9.43967292	10.17093281	9.713270561
ENSG00000117533	VAMP4	1.261219659	0.312962462	1.685656317	0.156803554	2.946875975	4.00722531	5.72254323	7.398165132	7.510853288	7.52080763	8.281350892	7.470508284
ENSG00000129968	ABHD17A	1.335718102	0.012810571	1.610386298	0.002019672	2.9461044	7.096806373	7.431218269	8.296287027	8.989221226	9.03387903	9.267612236	9.301104226
ENSG00000083444	PLOD1	0.993388892	0.040278645	1.950375076	1.27E-05	2.943763968	12.13890018	11.48015755	11.12102291	12.62961284	12.6409809	13.58778272	13.59591238
ENSG00000138316	ADAMTS14	0.128812861	0.949002608	2.812114867	0.093670473	2.940927728	2	6.389896633	4.058998049	5.37770462	5.14724905	6.446574529	8.508191835
ENSG00000157303	SUSD3	2.401179135	0.269864545	0.539672239	0.82433351	2.940851375	2	3.076665209	2	3.997744386	3.69849104	2.885263063	2.671842496
ENSG00000160789	LMNA	1.047226017	0.393187917	1.890279003	0.095521719	2.93750502	11.17828117	10.94355721	7.691296561	11.55759209	11.6302244	12.13170852	12.68814343
ENSG00000069849	ATP1B3	1.794420511	2.14E-06	1.141652159	0.003792422	2.93607267	9.097018025	9.488434476	9.746650996	11.2472134	11.2658107	10.35493302	10.8183324
ENSG00000156052	GNAQ	1.30485017	0.346271967	1.630049107	0.220537149	2.934899277	5.095959458	9.523645035	8.076950877	9.778365132	9.69337522	10.08982211	10.0302037

ENSG00000085719	CPNE3	1.276910497	0.004297518	1.657791213	0.000126297	2.93470171	9.112843761	8.505280662	8.143093325	9.926517587	9.89552277	10.2461159	10.33375043
ENSG00000080031	PTPRH	0.72858269	0.764673299	2.20304881	0.30263573	2.931631499	3.327711234	2	2	2.947924294	2.77779907	3.767980144	3.660199087
ENSG00000116117	PARD3B	-0.6900269	0.749166874	3.621321002	0.047708245	2.931294102	2	5.131603625	8.486635645	6.387081877	6.37866593	10.55599851	10.6947155
ENSG00000163125	RPRD2	1.287897482	0.418287941	1.643181522	0.28010057	2.931079004	3.327711234	8.762721501	8.076950877	9.133031936	9.21030762	9.556714309	9.493138564
ENSG00000139734	DIAPH3	-1.427692635	0.420476172	4.357308364	0.004497948	2.929615729	2	6.535001748	3.369317972	3.921164147	4.0930011	9.272995418	9.500920284
ENSG00000092529	CAPN3	4.378714258	0.001386931	-1.453049573	0.354405417	2.925664685	4.467311984	2	2	6.946463121	6.91614081	2.68698273	2.509591799
ENSG00000172757	CFL1	1.084165028	0.20823704	1.841262599	0.022093349	2.925427626	12.21602152	10.74900803	10.31150679	12.39989575	12.4351513	12.79907327	13.47179816
ENSG00000109171	SLAIN2	1.265291806	0.359143464	1.658199357	0.208570841	2.923491162	8.946185348	8.505280662	4.523675391	9.449924671	9.45234084	9.875912956	9.807406144
ENSG00000244682	FCGR2C	0.429780277	0.874101085	2.493622671	0.267777028	2.923402949	2	3.076665209	2	2.868578001	2.59489879	4.301748724	3.407202941
ENSG00000188779	SKOR1	0.26663352	0.962060833	2.656548799	NA	2.923182319	2	2	2	2	2.68924543	3.256555576	3.496556178
ENSG00000005339	CREBBP	1.427992015	0.015763734	1.494309202	0.010593672	2.922301217	9.143983763	7.73146271	8.380957451	9.925905286	9.96612421	10.07485216	9.946704253
ENSG00000111145	ELK3	1.663884875	0.023888254	1.258291463	0.094431932	2.922176338	7.096806373	9.055168103	8.325066185	10.01150489	10.0356777	9.490026756	9.738960951
ENSG00000118058	KMT2A	1.423154445	0.078125311	1.498058272	0.058395723	2.921212717	8.564112515	9.799806309	7.777116083	10.350452	10.4019472	10.58021248	10.3092961
ENSG00000104812	GYS1	1.224767423	0.347067822	1.694369015	0.170827212	2.919136438	10.32020829	8.255926468	6.882775709	10.35136432	10.3813388	10.10534852	11.3170206
		8.108156379	2.59E-06	-5.191149723	0.038130367	2.917006657	2	2	2	8.126317766	8.18299958	2	2
ENSG00000134758	RNF138	1.997292401	0.023633746	0.918334499	0.332076786	2.9156269	5.095959458	5.72254323	3.369317972	7.012547947	6.83591385	6.118193494	5.641643428
ENSG00000171466	ZNF562	1.678941526	8.29E-06	1.235913944	0.001354312	2.91485547	6.73718411	7.274681896	7.451345288	8.868232317	8.80807321	8.427654172	8.37166861
ENSG00000282750		8.105987317	2.65E-06	-5.191149706	0.038217881	2.914837611	2	2	2	8.113468729	8.19108423	2	2
ENSG00000183779	ZNF703	1.299128413	0.452192995	1.614185269	0.329209308	2.913313682	7.867441304	7.002537973	2	8.176599235	8.22691474	8.72755027	8.261263339
ENSG00000102908	NFAT5	2.063860479	0.044161456	0.847361536	0.444451639	2.911222015	7.218819781	9.367676479	10.36791565	11.57429426	11.4832463	10.41087029	10.21350733
ENSG00000160255	ITGB2	-0.944792845	0.688658118	3.855947898	0.054642188	2.911155053	5.330594134	2	2	3.162721474	3.50452879	8.219062336	5.727202496
ENSG00000115084	SLC35F5	2.061029893	5.36E-07	0.850101691	0.057679184	2.911131584	7.790810999	8.147241016	8.380957451	10.18529247	10.1567216	9.231310145	8.642113606
ENSG00000176407	KCMF1	1.169698718	0.019862551	1.741243301	0.000261128	2.910942019	7.709880939	8.791896851	8.325066185	9.505076228	9.51325794	10.22143306	9.918940524
ENSG00000205835	GMNC	0.010004217	0.997481474	2.900577038	0.348689353	2.910581255	2	2	2	2.270786764	2.38539787	2.911488689	3.972018587
ENSG00000163577	EIF5A2	1.310483156	0.003591958	1.600043453	0.000246393	2.91052661	6.73718411	6.140560021	6.629488387	7.831679003	7.72392024	8.181838221	7.934467448
ENSG00000089692	LAG3	0.497905282	0.895675696	2.411798403	NA	2.909703685	2	2	2	2.498691189	2.38539787	2.746468522	3.600986722
ENSG00000168264	IRF2BP2	1.05826229	0.392721273	1.850750879	0.106982382	2.909013169	6.653098118	9.377333006	6.718950348	9.207473484	9.26494702	10.30850554	9.671609742
ENSG00000180891	CUEDC1	0.541030609	0.725318811	2.367871685	0.073990493	2.908902294	8.75772933	7.467835385	4.058998049	8.221200057	8.2561084	9.967428009	10.13153689
ENSG00000106012	IQCE	1.415943709	0.036071417	1.492538044	0.024788756	2.908481753	7.485143553	6.04685525	6.203069917	8.072008297	8.13141746	8.151901655	8.202721679
ENSG00000124496	TRERF1	0.903041054	0.621621222	2.0052456	0.230071506	2.908286654	5.095959458	7.431218269	2	7.030886362	7.00136498	7.985968197	8.195234077
ENSG00000104722	NEFM	1.912322275	0.385406506	0.994962842	0.665769105	2.907285118	4.467311984	2	8.408111533	8.778830996	8.8250571	8.372671446	7.179807904
ENSG00000182179	UBA7	1.840788536	0.333078915	1.065972507	0.58769378	2.906761043	3.327711234	5.838937808	9.601288346	9.903687576	10.0136237	8.996912583	9.358468538
ENSG00000163161	ERCC3	1.459658133	0.141653394	1.446911344	0.138280206	2.906569477	6.366709671	8.747909548	6.80318718	9.123450746	9.18320541	9.100995221	9.17988647
ENSG00000187908	DMBT1	3.594462417	0.233603426	-0.690089515	0.842587044	2.904372902	2	2	2	3.754611435	4.2560182	2.34604626	2.059860178
ENSG00000148773	MKI67	1.13744563	0.610493708	1.76685207	0.401339976	2.9042977	3.327711234	6.311476754	2	6.34372989	5.6121243	4.434275199	7.453871289
ENSG00000105656	ELL	1.829122755	0.253735228	1.074743324	0.517007889	2.903866079	7.624139768	6.844429755	2	8.451360823	8.56844956	7.733770002	7.800517754
ENSG00000148798	INA	-0.144572707	0.938309757	3.046931005	0.041995639	2.902358298	4.467311984	6.78762695	2	5.37770462	5.43283104	8.725241966	8.16062336
ENSG00000143797	MBOAT2	1.314593438	0.0316272	1.587496711	0.007499883	2.902090149	8.075571721	7.232742069	8.143093325	9.218515697	9.10727282	9.833465342	8.882782322
ENSG00000144589	STK11IP	0.969913071	0.556828814	1.93169378	0.204191294	2.901606852	5.095959458	6.899080544	2	6.693715713	6.61334686	7.378272268	7.770601445
ENSG00000196715	VKORC1L1	0.973460856	0.43698019	1.926391067	0.092679263	2.899851923	5.866944937	7.232742069	9.227351203	9.025111392	9.04060595	10.03085749	9.928254593
ENSG00000163354	DCST2	0.49578662	0.895265695	2.404046802	NA	2.899833422	2	2	2	2.3892346	2.49394783	3.5515855	2.817676617
ENSG00000166436	TRIM66	2.287547961	0.00023626	0.61106281	0.389304796	2.898610771	5.095959458	4.710564154	4.874587708	6.912249745	7.10768997	5.47655442	5.385063124
ENSG00000163069	SGCB	1.543930637	1.41E-08	1.353748536	7.51E-07	2.897679174	9.423197387	9.338312924	9.149036566	10.8774261	10.8161359	10.5076599	10.7930647
ENSG00000172890	NADSYN1	1.334933896	0.030870877	1.560044109	0.009596225	2.894978005	7.218819781	8.077869079	6.629488387	8.799033614	8.72698617	8.900776778	9.067728571
ENSG00000134982	APC	1.216166105	0.124010482	1.678055851	0.02597163	2.894221955	6.96351253	7.874700259	8.960138144	9.363030664	9.35764963	9.742804424	9.892948091
ENSG00000140416	TPM1	0.637968274	0.234421046	2.255751845	1.19E-06	2.893720119	12.62340907	11.50239087	12.26789926	12.82090962	12.858361	14.47704462	14.43668992
ENSG00000154813	DPH3	1.294127454	0.215656888	1.598460937	0.111748606	2.892588391	6.73718411	8.469613413	5.772716771	8.738951712	8.68609779	9.089196199	8.935663927
ENSG00000116786	PLEKHM2	1.465741702	4.71E-07	1.426758341	8.90E-07	2.892500043	9.128497779	9.514922632	9.06622548	10.70542879	10.7271979	10.72194193	10.63154769
ENSG00000054277	OPN3	0.577340645	0.698378828	2.315075569	0.07118462	2.892416214	2	4.710564154	6.073378139	5.552894743	5.49945122	6.897310495	7.405663765
ENSG00000072310	SREBF1	1.006370113	0.357081274	1.882635898	0.061692379	2.889006011	8.075571721	8.124485672	5.59507254	8.660150093	8.60672783	8.733075176	10.0028246
ENSG00000141837	CACNA1A	1.783746604	0.317344658	1.103300299	0.547274868	2.887046903	2	5.303523747	7.646393253	8.054188409	8.08661608	6.981729486	7.728570659
ENSG00000189308	LIN54	1.547403489	0.141109656	1.339231476	0.20113831	2.886634965	4.467311984	7.145017454	5.59507254	7.688853661	7.63485389	7.730090858	7.123062801
ENSG00000130856	ZNF236	1.256620858	0.634315819	1.629448933	0.522581231	2.886069791	2	7.638155693	2	7.409376773	7.24216308	7.905886539	7.442672408
ENSG00000124831	LRRFIP1	1.095593514	0.362915467	1.78686846	0.112712865	2.882461974	9.922475255	8.929529568	6.322058544	10.0950348	10.08857	10.7425177	10.81860316
ENSG00000137275	RIPK1	1.538776737	0.025736029	1.341485744	0.053383916	2.880262481	6.816637977	8.376385542	8.561105505	9.617037214	9.63530531	9.357992454	9.498556374
ENSG00000182132	KCNIP1	-1.265414385	0.719366965	4.144088735	0.164866748	2.87867435	3.327711234	2	2	2.498691189	2	3.235948948	6.102937566
ENSG00000059588	TARBP1	1.472343416	0.325101958	1.404983578	0.341761606	2.877326994	2	6.535001748	6.80318718	7.517371202	7.56191311	7.622078048	7.308762629
ENSG00000119139	TJP2	3.626360193	1.10E-06	-0.749284856	0.394959847	2.877075337	4.467311984	4.11352228	4.874587708	7.852449262	7.86451142	3.600766115	4.161535617
ENSG00000134853	PDGFRA	2.429635281	0.017875395	0.444768554	0.710952496	2.874403835	7.904282243	10.96612994	9.330306409	12.32756329	12.3434862	10.2021419	10.49133865
ENSG00000100614	PPM1A	1.343161685	0.321826745	1.530684477	0.245064763	2.873846162	4.467311984	8.747909548	8.296287027	9.329162745	9.32954365	9.743945072	9.244592076
ENSG00000162836	ACP6	-3.753133088	0.276585553	6.626294153	0.013315484	2.873161065	2	2	2	2	2	6.972416862	6.38621943
ENSG00000185477	GPRIN3	0.303711777	0.911798376	2.568776732	0.257812226	2.872488509	2	3.686303414	2	3.09461492	2.94009902	5.243518884	3.261853426
ENSG00000282110		1.397893884	0.821277934	1.474322103	NA	2.872215987	2	2	2	3.227757282	2	2.831336763	2.70970617
ENSG00000105866	SP4	0.975100895	0.54007403	1.896468801	0.195422681	2.871569696	2	5.457120953	3.369317972	4.907595604	5.39133044	6.106889539	5.913137508
ENSG00000078140	UBE2K	1.516872063	0.311071331	1.354604018	0.362520034	2.871476081	9.233563228	8.505280662	4.058998049	9.952603669	9.76282251	9.724430319	9.673706637
ENSG00000101966	XIAP	1.512994155	0.07809295	1.358347504	0.112145055	2.871341659	9.204317094	7.538394143	7.225742779	9.851120801	9.67835667	9.767692698	9.44062848
ENSG00000105298	CACTIN	1.561499709	0.219703759	1.307066029	0.303728756	2.868565739	3.327711234	7.35507212	6.882775709	8.111316053	8.15640714	7.880388025	7.88671543
ENSG00000119950	MXI1	1.196041064	0.011144863	1.672271152	0.000207352	2.868312215	7.27616306	7.538394143	8.175061096	8.857997553	8.91810182	9.527072508	9.17354978
ENSG00000087448	KLHL42	0.973783029	0.001343782	1.893085046	3.08E-11	2.866868074	7.790810999	7.954278496	7.646393253	8.75144642	8.79221631	9.675532774	9.694509651
ENSG00000136231	IGF2BP3	1.304893142	0.527512895	1.560767111	0.432736935	2.865660253	4.00722531	7.701025996	2	7.530319349	7.51437845	7.801071566	7.744618542
ENSG00000118412	CASP8AP2	1.485455148	0.357081274	1.379748605	0.385870817	2.865203753	7.33131396	6.844429755	2	8.083034979	7.89700047	8.21050623	7.474637727
ENSG00000114867	EIF4G1	1.331037302	2.18E-05	1.533997148	6.63E-07	2.86503445	10.70657014	11.05905903	11.23155829	12.29106025	12.3962337	12.417014	12.66702221
ENSG00000146007	ZMAT2	1.421701693	4.45E-05	1.442809771	2.97E-05	2.864511464	7.667647223	7.638155693	8.042706615	9.207473484	9.17914662	9.05761907	9.355859082
ENSG00000274641	HIST1H2BO	6.619714578	2.54E-05	-3.759275672	0.072505978	2.860438906	3.327711234	2	2	7.688853661	7.5835711	2.094598369	2
ENSG00000099219	ERMP1	0.849719255	0.575968182	2.010169922	0.143374446	2.859889177	2	6.311476754	6.534109959	6.542151749	6.81514011	8.033012338	7.55613706
ENSG00000164136	IL15	1.918708666	0.284626966	0.940727841	0.617051806	2.859436507	6.138360429	2	3.369317972	6.516352584	6.60126376	5.85994354	5.379200993
ENSG00000131149	GSE1	1.252808981	0.437181027	1.606125414	0.29637811	2.858934395	4.467311984	9.018611864	5.930865648	8.88471049	8.8984863	9.358290408	9.116112551
ENSG00000164122	ASB5	-1.272311879	0.588479045	4.12969454	0.027228112	2.85738266	2	3.076665209	2	2.270786764	2.2680111	5.590506472	4.870334174
ENSG00000180398	MCFD2	1.746793893	6.74E-05	1.11049032	0.015545758	2.857284213	10.09970294	10.87363932	11.06240811	12.48060115	12.4782175	11.74926356	11.93227541
ENSG00000118046	STK11	0.945916076	0.217903135	1.911308249	0.006424889	2.857224324	8.444143041	8.124485672	6.534109959	8.795015659	8.89354047	9.757111106	9.850148984
ENSG00000104320	NBN	2.310456695	0.001663588	0.546659127	0.518789265	2.857115822	8.468948322	8.747909548	6.718950348	10.508071	10.5173643	8.878929913	8.626733602
ENSG00000126218	F10	-0.343823244	0.863551136	3.199959587	0.046571766	2.856136343	4.00722531	6.951736442	2	5.348759107	5.24368216	8.674459329	8.725336927
ENSG00000206340		8.047083063	3.97E-06	-5.191149543	0.039270477	2.85593352	2	2	2	8.115618198	8.07355593	2	2
ENSG00000166272	WBP1L	0.811022465	0.064313177	2.044444216	2.11E-07	2.85546668	8.517314938	9.170736405	8.380957451	9.498502402	9.59245646	10.7588599	10.79141033
ENSG00000116580	GON4L	1.708051563	5.57E-08	1.146611631	0.000397696	2.854663193	8.313285031	7.790474954	8.007629717	9.72797373	9.74782835	9.152756493	9.20620076
ENSG00000122512	PMS2	2.191834163	0.105206276	0.662568289	0.65836129	2.854402452	6.653098118	6.04685525	2	7.956842238	7.93841129	6.615624322	6.289521179
ENSG00000139223	ANP32D	2.389904445	0.458595738	0.463608915	NA	2.853513361	2	2	2	3.227757282	3.2184422	2.3070652	2.551896972
ENSG00000159202	UBE2Z	1.390626654	0.033756605	1.461636056	0.023809617	2.85226271	9.317904228	9.632483534	10.79563557	11.4400825	11.4577141	11.45029098	11.58618106
ENSG00000271303	SRXN1	4.647453335	0.013410886	-1.796957671	0.381806697	2.850495664	3.327711234	7.790474954	2	10.8767926	10.8890776	4.628601098	4.729989799
ENSG00000124215	CDH26	2.015355299	0.596379651	0.834379539	NA	2.849734838	2	2	2	3.349643765	2.59489879	2.831336763	2.172608095
ENSG00000158109	TPRG1L	0.883794463	0.14612275	1.965736024	0.000339822	2.849530488	9.204317094	8.297207175	7.896971941	9.4022977	9.49378147	10.44409424	10.60416654
ENSG00000143398	PIP5K1A	0.979355878	0.056923475	1.866622143	8.15E-05	2.845978021	8.998234871	8.777382925	8.042706615	9.62912467	9.64121862	10.22388773	10.76411688
ENSG00000106266	SNX8	2.152674458	0.055618402	0.69253238	0.576450932	2.845206838	4.467311984	7.002537973	8.076950877	9.222510184	9.27353851	7.761068522	7.846917442
ENSG00000120694	HSPH1	2.249233505	1.61E-05	0.595796776	0.31653454	2.845030281	9.233563228	8.762721501	8.076950877	10.99037529	11.0213536	9.624671944	9.031747025
ENSG00000119669	IRF2BPL	1.75163113	0.101896165	1.093381555	0.322038974	2.845012685	7.867441304	7.393647515	10.08627303	10.67332098	10.7170855	10.10570349	9.969317474
ENSG00000129933	MAU2	1.466601571	0.02248445	1.377252536	0.032067896	2.843854107	7.33131396	8.655694736	7.451345288	9.372948073	9.44306193	9.47746372	9.142307541
ENSG00000234284	ZNF879	1.507954291	0.483180449	1.335810784	0.530234743	2.843765074	2	5.838937808	2	5.808110275	5.8245091	5.805044075	5.492143571
ENSG00000151929	BAG3	1.003702538	0.311446617	1.83982764	0.044684078	2.843530178	9.751047882	7.928237109	7.451345288	9.695291778	9.76891184	10.39889946	10.7152199
ENSG00000187288	CIDEC	2.329169315	0.517280172	0.513942789	NA	2.843112104	2	2	2	2.695456494	3.55551834	2.183373728	2.671842496
ENSG00000160678	S100A1	-0.727654445	0.838100556	3.570689841	0.212471855	2.843035396	2	2	2	2.141737246	2.2680111	4.487545924	3.287118176
ENSG00000100813	ACIN1	1.545296846	3.14E-09	1.297535621	7.98E-07	2.842832466	9.27634997	9.298208073	8.978369997	10.72913699	10.7382749	10.56480374	10.40381853
ENSG00000282607		0.472225567	0.801541753	2.370294611	0.133814471	2.842520177	3.327711234	2	4.874587708	4.139692846	4.16043394	4.888321757	6.338680316
ENSG00000149428	HYOU1	1.648959525	0.041269777	1.19313316	0.147829677	2.842092685	8.107517749	10.07368926	9.301632639	10.98685701	11.0432906	10.03235202	10.9463816
ENSG00000101306	MYLK2	0.015720062	0.997041173	2.825459542	0.376931525	2.841179603	2	2	2	2.498691189	2.14022021	3.663836244	3.287118176
ENSG00000198961	PJA2	1.031169668	0.519532942	1.807097244	0.223166355	2.838266911	5.095959458	10.50658023	9.926432745	10.72562309	10.6923799	11.54455506	11.41991513
ENSG00000183281	PLGLB1	5.470641317	0.048431027	-2.633351554	0.413811187	2.837289762	2	2	2	5.602782483	5.63601114	2	2.117335275
ENSG00000111880	RNGTT	1.240267006	0.441837321	1.596244278	0.298915693	2.836511284	7.031698319	7.232742069	2	7.872924734	7.68723697	8.121330705	8.145162126

ENSG00000136824	SMC2	1.55258824	0.127668185	1.28224159	0.209153239	2.834829829	4.00722531	6.951736442	6.629488387	7.810605345	7.85181819	7.710619897	7.405663765
ENSG00000198720	ANKRD13B	0.308209421	0.82459995	2.525119688	0.024879166	2.833329109	2	5.72254323	5.772716771	5.433909581	5.5755357	7.628021136	7.612190665
ENSG00000130508	PXDN	0.824567426	0.033290369	2.008323781	1.18E-08	2.832891207	11.43272766	12.10496808	11.63358652	12.56462203	12.5888679	13.62095662	13.8868593
ENSG00000168575	SLC20A2	0.075762959	0.947432314	2.756708697	0.002325611	2.832471656	7.867441304	8.191702463	10.10298738	9.208480821	9.09870316	11.37785453	12.16903209
ENSG00000136531	SCN2A	2.325581883	0.252142759	0.506671096	0.823417076	2.832252979	4.00722531	2	2	4.54891529	4.50979031	3.534812207	2.851942824
ENSG00000168781	PPIP5K1	1.19921197	0.385295588	1.632808293	0.213016766	2.832020263	7.218819781	4.442710129	4.058998049	7.136275664	7.06433609	7.354572674	7.674548617
	TNFAIP8L2-S	8.02130996	4.78E-06	-5.191149458	0.039834475	2.830160502	2	2	2	8.045195239	8.09310209	2	2
ENSG00000107611	CUBN	2.655294295	0.113756797	0.173841927	0.932122597	2.829136221	3.327711234	2	3.369317972	4.647285135	4.93258395	2.831336763	3.236138336
ENSG00000085662	AKR1B1	3.839895367	7.45E-05	-1.010764034	0.361796964	2.829131333	10.37373127	10.10308486	7.225742779	13.55263966	13.6081105	8.612861354	8.862441066
ENSG00000188021	UBQLN2	1.115475623	0.568958143	1.713503637	0.355608544	2.82897926	8.836089686	8.876041558	2	9.430198858	9.33684939	9.968013835	9.990620812
ENSG00000175820	CCDC168	2.079153501	0.534782548	0.749273083	NA	2.828426584	2	2	2	2.784612485	3.28037825	2.560105256	2.466008509
ENSG00000159214	CCDC24	1.044628963	0.612008031	1.781997866	0.354312588	2.826626829	2	5.303523747	2	4.694064037	5.07918227	5.489669427	5.621921006
ENSG00000109686	SH3D19	1.002502974	0.020115865	1.823078833	5.77E-06	2.825581807	7.940205782	8.505280662	8.784770914	9.459264257	9.43116542	10.40063634	10.10913368
ENSG00000079435	LIPE	0.78447177	0.691448868	2.040495998	0.248858956	2.824967767	2	4.442710129	2	4.205751501	3.60476711	4.965450964	4.943664032
ENSG00000173678	SPDYE2B	3.937386335	0.120832449	-1.11303236	0.702841297	2.824353975	2	2	2	4.299492648	4.2865223	2.094598369	2.225841198
ENSG00000169914	OTUD3	1.066907905	0.342227985	1.754556322	0.094172652	2.821464227	4.815609471	7.099070191	4.874587708	7.101994243	7.03768431	7.439255893	7.991296779
ENSG00000213853	EMP2	1.472604686	0.104022412	1.34687985	0.133542516	2.819484537	6.257048221	8.255926468	8.721235202	9.562142546	9.48805095	9.154129994	9.61099888
ENSG00000075568	TMEM131	1.848850241	0.069445337	0.969989336	0.364791599	2.818839577	7.096806373	9.181799937	6.629488387	9.963384909	9.89675912	9.080915197	9.031747025
ENSG00000140553	UNC45A	1.146996467	0.403335693	1.669361475	0.197276691	2.816357942	5.532353208	9.599860728	7.646393253	9.528658349	9.58173459	10.09430085	10.05638425
ENSG00000117479	SLC19A2	2.035774245	0.114685272	0.780272858	0.575511775	2.816047103	6.366709671	5.838937808	2	7.52061915	7.5462416	6.202919533	6.429408106
ENSG00000081026	MAGI3	0.701616206	0.674035246	2.113061856	0.152092183	2.814678061	2	4.93638933	6.718950348	6.229283386	6.14061114	7.733770002	7.331709059
ENSG00000186666	BCDIN3D	1.555128814	0.365734008	1.255422738	0.466314777	2.810551552	2	5.946638857	3.369317972	6.096288482	6.12381559	6.192262702	5.343519008
ENSG00000170734	POLH	0.392747294	0.708629645	2.416413322	0.005377605	2.809160617	5.866944937	7.232742069	8.434763955	7.842101511	7.97866656	9.975996365	9.856492132
ENSG00000203747	FCGR3A	-3.75310509	0.347620176	6.561632913	0.043929255	2.808527823	2	2	2	2	2	7.406207255	4.943664032
ENSG00000172348	RCAN2	-1.714574544	0.197249313	4.522488304	6.66E-05	2.807913759	2	5.303523747	4.058998049	3.406930997	2.94009902	8.015738956	9.035013043
ENSG00000177054	ZDHHC13	2.009608423	0.203194975	0.798202706	0.638087371	2.807811129	4.00722531	6.389896633	2	6.970413838	7.09482005	5.951260634	5.803599879
ENSG00000105613	MAST1	0.183876679	0.947256262	2.621302847	0.264448359	2.805179526	2	2	3.369317972	2.3892346	2.86123014	4.478802948	3.33635788
ENSG00000128829	EIF2AK4	1.128386994	0.38727917	1.676757001	0.172175696	2.805143995	5.330594134	8.505280662	9.372276026	9.600249849	9.58480612	10.13729849	10.13979504
ENSG00000158473	CD1D	3.045259312	0.301996555	-0.241216246	0.944101725	2.804043067	2	2	2	3.406930997	3.82856379	2.183373728	2.374681325
ENSG00000176783	RUFY1	1.300577444	0.460626621	1.500587689	0.37615226	2.801165133	7.975256505	7.73146271	2	8.534041678	8.59915299	8.789844052	8.73970592
ENSG00000100266	PACSIN2	1.185240983	0.403737922	1.615912333	0.231087488	2.801153316	7.485143553	9.00621767	4.523675391	9.060130342	9.09439916	9.498161492	9.512347353
ENSG00000119787	ATL2	1.81447857	0.113324339	0.985008399	0.41126962	2.799486968	4.00722531	7.315436877	6.80318718	8.485003881	8.22494733	7.934143724	7.010010317
ENSG00000115464	USP34	1.790023304	0.119919599	1.009093757	0.399869187	2.799117061	6.366709671	9.828248506	9.725218844	11.01965231	11.0665479	10.30896804	10.21823539
ENSG00000168159	RNF187	0.934878691	0.133409766	1.864113711	0.001063556	2.798992403	8.696033109	9.405920729	10.11951029	10.42037002	10.4827929	11.48720025	11.26243498
ENSG00000187193	MT1X	3.6615396	0.000101566	-0.862683757	0.430112177	2.798855844	6.366709671	6.228547981	3.369317972	9.422406043	9.34683476	5.371837532	4.604965121
ENSG00000060749	QSER1	1.153712923	0.119776467	1.644186545	0.019679078	2.797899468	7.750913381	8.213429689	6.322058544	8.847689662	8.69465306	9.279302964	9.240151898
ENSG00000139668	WDFY2	2.001133626	0.287567214	0.79592993	0.691793928	2.797063555	3.327711234	8.077869079	3.369317972	8.519504557	8.61125371	7.444878196	7.313381205
ENSG00000176095	IP6K1	1.065961396	0.478780358	1.730712906	0.218585328	2.796674302	6.89194353	9.923599762	5.930865648	9.682298904	9.60914575	10.2683239	10.34713831
ENSG00000124557	BTN1A1	3.305309898	0.256719105	-0.510113158	0.881429417	2.79519674	2	2	2	3.95996233	3.65239003	2.34604626	2.117335275
ENSG00000130382	MLLT1	1.192985989	0.117455955	1.600556673	0.028078154	2.793542663	8.39321512	9.386925327	7.502634626	9.763363111	9.87371334	10.1071225	10.33507589
ENSG00000116497	S100PBP	1.129905457	0.47627622	1.663198898	0.2660773	2.793104355	6.653098118	8.395519306	3.369317972	8.310225403	8.36731141	9.138255231	8.532164007
ENSG00000137494	ANKRD42	1.473998306	0.395630265	1.31882697	0.443133621	2.792825277	2	6.04685525	7.734844356	8.033874364	7.94559728	7.949254677	7.7169976
ENSG00000158411	MITD1	1.750203409	0.007222848	1.041435024	0.126314768	2.791638433	5.532353208	4.710564154	4.874587708	6.622948477	6.75096147	6.126613714	5.892708443
ENSG00000145362	ANK2	-0.848018358	0.499737617	3.63935418	0.000559099	2.791335821	3.327711234	6.228547981	6.882775709	5.614989456	4.89348248	9.397089785	9.845904672
ENSG00000132623	ANKEF1	0.719244279	0.762684238	2.069811962	0.329610478	2.789056242	2	3.686303414	2	3.289987342	3.2184422	4.652122235	3.474731315
ENSG00000184661	CDCA2	2.805923552	0.010849998	-0.01978254	0.988654005	2.786141012	3.327711234	5.595927442	3.369317972	7.161462517	7.17852729	4.281782022	4.810605986
ENSG00000175414	ARL10	-0.362929574	0.85249814	3.148349855	0.047119088	2.785420281	8.777722034	5.595927442	3.369317972	7.030886362	7.00595521	10.57214341	10.39984297
ENSG00000157999	ANKRD61	-0.313903303	0.931247849	3.098989004	0.298214368	2.785085701	2	2	2	2.141737246	2.38539787	3.679182183	3.660199087
ENSG00000176915	ANKLE2	1.351211071	5.93E-05	1.433873987	1.66E-05	2.785085059	9.371511179	9.28800521	8.742726175	10.52838133	10.4873138	10.61914866	10.56112449
ENSG00000119685	TTLL5	1.215139655	0.174428066	1.564350031	0.067386662	2.779489685	4.467311984	6.78762695	6.718950348	7.491120977	7.4949172	7.683291963	7.972925696
ENSG00000237190	CDKN2AIPNL	0.878605881	0.564421351	1.899346125	0.172231671	2.777952006	2	6.464272742	5.156626401	6.283823264	6.22964624	7.146744843	7.325625608
ENSG00000132471	WBP2	1.031872246	8.80E-05	1.745761235	4.21E-12	2.777633482	9.497412313	9.632483534	9.61300511	10.57755058	10.6438729	11.16817282	11.46385329
ENSG00000135315	CEP162	1.522224911	0.387998502	1.254646785	0.475883236	2.776871696	3.327711234	6.228547981	2	6.379946408	6.26836889	6.151583464	5.98823215
ENSG00000112365	ZBTB24	1.336626944	0.381296261	1.440091386	0.332115168	2.77671833	3.327711234	7.002537973	4.058998049	6.979883891	6.99676009	7.183528998	6.987067564
ENSG00000185621	LMLN	1.932493928	0.003085869	0.842076037	0.23478853	2.774569966	5.095959458	6.535001748	5.930865648	8.027038952	7.66998641	6.583362013	6.983208048
ENSG00000148700	ADD3	1.747515187	0.014968435	1.025874811	0.172755691	2.773389999	9.371511179	9.856140801	7.934808822	11.02051291	10.9793733	10.23187735	10.32710477
ENSG00000149948	HMGA2	2.564335009	3.00E-05	0.208826321	0.788857412	2.77316133	6.816637977	8.255926468	8.143093325	10.44750059	10.3857483	8.354863925	7.736616914
ENSG00000148356	LRSAM1	1.104322747	0.57816817	1.667132923	0.374392974	2.77145567	4.815609471	7.874700259	2	7.461007055	7.64666005	8.070592084	8.147750532
ENSG00000183647	ZNF530	1.14522636	0.421750953	1.625243748	0.225861304	2.770470107	2	4.93638933	3.369317972	5.003571641	4.811949	5.427419529	5.23734678
ENSG00000251201	TMED7-TICA	8.922970381	3.03E-10	-6.15294715	0.005714661	2.770023231	2	2	3.369317972	9.911750163	9.92248069	2	2
ENSG00000136026	CKAP4	1.748271279	1.18E-05	1.021679778	0.015233955	2.769951057	11.67849893	12.43359305	12.12382753	13.8324849	13.885323	12.89278527	13.33854399
ENSG00000174137	FAM53A	1.090947744	0.564770025	1.677954217	0.347715703	2.768901961	2	4.710564154	2	4.443344104	4.37434574	4.996409624	4.80186789
ENSG00000062370	ZNF112	2.011641434	0.104928256	0.757183	0.572430102	2.768824434	4.467311984	5.595927442	2	6.529309835	6.507263	5.056402493	5.59182244
ENSG00000159714	ZDHHC1	1.488202593	0.313991908	1.280096897	0.384020069	2.768299491	6.468621203	6.666836837	2	7.337530098	7.56813436	7.541454591	6.895576665
ENSG00000198042	MAK16	1.949981094	0.224835356	0.817845983	0.633808927	2.767827077	2	7.35507212	5.772716771	8.126317766	8.10812366	7.024410815	6.994755759
ENSG00000152484	USP12	1.671765098	0.356532724	1.095260672	0.553726324	2.76702577	2	7.901717006	8.511888463	9.339314897	9.29150981	8.832932104	8.648219991
ENSG00000163873	GRIK3	2.867387832	0.607619146	-0.101338069	0.986936055	2.766049763	2	2	2	4.237680128	2	2.384001718	2.225841198
ENSG00000140521	POLG	0.766696349	0.416762166	1.99880831	0.017463001	2.765504658	8.631580145	6.311476754	7.552162999	8.621345041	8.4504872	9.908424552	9.59812349
ENSG00000169592	INO80E	1.462706723	0.000263663	1.301602073	0.001237837	2.764308796	7.218819781	6.78762695	6.534109959	8.325162455	8.28283292	8.083625221	8.204380326
ENSG00000163807	KIAA1143	1.257307223	0.002222495	1.50618855	0.000167667	2.763495773	6.816637977	7.274681896	7.646393253	8.509731121	8.52593541	8.824330559	8.701457496
ENSG00000166432	ZMAT1	1.296378455	0.351836711	1.464015248	0.275776462	2.760393703	4.00722531	4.442710129	2	4.965943888	4.811949	5.454427604	4.445805334
ENSG00000166685	COG1	1.28743921	0.38727917	1.471270643	0.306126091	2.758709854	8.257427002	7.847167909	3.369317972	8.702235867	8.85462427	9.073312544	8.842874172
ENSG00000060069	CTDP1	2.025637517	0.113077307	0.732236112	0.598176643	2.757873629	3.327711234	6.464272742	7.552162999	8.47833774	8.6202631	7.289818338	7.269688419
ENSG00000153936	HS2ST1	1.965688948	0.037719519	0.78796485	0.44254216	2.753653798	6.366709671	6.728495673	8.677268317	9.595637371	9.5561418	8.558404203	8.239587639
ENSG00000184304	PRKD1	1.133883377	0.539294232	1.619100855	0.357994057	2.752984232	8.468948322	7.638155693	2	8.666028533	8.64548836	9.01441148	9.249822
ENSG00000127990	SGCE	1.433984388	0.010605581	1.318937742	0.019302872	2.752922129	8.39321512	8.124485672	7.029880454	9.357592295	9.40533771	9.202392925	9.329501596
ENSG00000133773	CCDC59	1.636040299	0.143639472	1.113411858	0.329610478	2.749452157	6.468621203	6.78762695	3.369317972	7.703217906	7.7377845	7.325609491	7.083953563
ENSG00000166046	TCP11L2	1.392181144	0.621742885	1.356024708	0.627083786	2.748205852	8.257427002	2	2	8.047448792	8.08227582	8.14016682	7.909264332
ENSG00000147799	ARHGAP39	1.869292484	0.157705645	0.878594434	0.529438052	2.747886918	2	6.04685525	4.874587708	6.755670824	6.89154576	5.883320383	5.884455123
ENSG00000152284	TCF7L1	0.788308943	0.455839475	1.959527919	0.037602746	2.747836862	5.330594134	7.73146271	5.930865648	7.440576128	7.5462416	8.660513226	8.634751663
ENSG00000141582	CBX4	1.155647655	0.325307017	1.591790899	0.153932236	2.747438554	8.285626337	7.274681896	4.874587708	8.583021623	8.42671895	9.200067611	8.619914108
ENSG00000107140	TESK1	1.098174494	0.437912435	1.648892318	0.215287817	2.747066812	4.467311984	7.901717006	8.960138144	9.043291801	9.09332116	9.714689414	9.509666762
ENSG00000004660	CAMKK1	1.630854221	0.292091049	1.115970017	0.477543645	2.746824238	3.327711234	5.595927442	2	5.978405189	5.77157844	5.436478516	5.373314945
ENSG00000140400	MAN2C1	0.947657014	0.521741285	1.798936732	0.185049898	2.746593746	4.00722531	8.395519306	6.534109959	8.115618198	8.18906731	8.967137431	9.022374596
ENSG00000157978	LDLRAP1	-0.489452467	0.805509199	3.235407096	0.049334117	2.745954629	7.485143553	8.487557258	2	7.012547947	7.03768431	10.35791611	10.99030784
ENSG00000066855	MTFR1	1.32415491	0.20606912	1.420864021	0.163643498	2.74501893	4.00722531	6.464272742	7.029880454	7.565338955	7.57741621	7.846806418	7.459438299
ENSG00000145414	NAF1	1.040943586	0.251528741	1.70387763	0.0439318	2.744821216	4.815609471	4.93638933	6.629488387	6.529309835	6.80463998	7.232460462	7.39408282
ENSG00000204946	ZNF783	1.32849268	0.337673274	1.414459558	0.294688747	2.742952239	5.095959458	3.686303414	7.342949392	7.337530098	7.4314987	7.447120996	7.491038307
ENSG00000186891	TNFRSF18	2.079149751	0.540424469	0.661664827	NA	2.740814579	2	2	2	2.784612485	3.28037825	2.592885947	2.374681325
ENSG00000179832	MROH1	1.713337853	0.091677988	1.027447214	0.328380953	2.740785067	7.031698319	5.457120953	8.325066185	9.088859521	9.0020652	8.364981826	8.368713182
ENSG00000197535	MYO5A	0.697839044	0.667312826	2.042031404	0.157060567	2.739870449	4.00722531	8.806266215	9.116480286	9.062360705	9.13582529	10.12456825	10.69427292
ENSG00000165732	DDX21	1.271314794	0.076622525	1.468463096	0.035337738	2.73977789	7.940205782	9.687856435	9.516493218	10.51174802	10.4856715	10.72136295	10.66896863
ENSG00000046889	PREX2	-1.269416704	0.637611669	4.008638154	0.06941864	2.73922145	2	2	3.369317972	2.3892346	2.14022021	5.815498119	3.956354764
ENSG00000047932	GOPC	1.653517548	0.137415848	1.085565799	0.342050085	2.739083347	8.340423424	8.777382925	5.39244166	9.746123826	9.67547818	9.163708059	9.129269498
ENSG00000117298	ECE1	0.611853258	0.114890958	2.124435107	3.85E-10	2.736288365	10.1311248	10.74900803	10.23774519	10.96675639	11.0555197	12.42250978	12.6161781
ENSG00000152778	IFIT5	2.360511616	0.00037188	0.374881416	0.637246531	2.735393032	6.816637977	8.297207175	8.631919287	10.43765481	10.4541068	8.385592001	8.554444396
ENSG00000157077	ZFYVE9	1.359350186	0.039642635	1.375324082	0.035171121	2.734674269	6.816637977	6.728495673	8.110401118	8.697938662	8.68752719	8.726627392	8.690255954
ENSG00000105668	UPK1A	-0.687348191	0.780246302	3.420751441	0.083002579	2.73340325	2	2	3.369317972	2.3892346	2.38539787	4.301748724	4.943664032
ENSG00000184602	SNN	-0.202395157	0.906452235	2.935454527	0.030273608	2.733059369	4.467311984	7.274681896	3.369317972	5.829220145	5.77157844	8.852523173	8.834865421
ENSG00000232653	GOLGA8N	7.923222943	9.74E-06	-5.191149092	0.042128548	2.732073851	2	2	2	8.006336225	7.93841129	2	2
ENSG00000144339	TMEFF2	2.11075326	0.313991908	0.620277279	0.787507734	2.73103054	2	3.686303414	2	4.623314311	3.65239003	3.082820997	3.311948089
ENSG00000188786	MTF1	1.813673055	0.191729965	0.91592059	0.529629876	2.729593645	4.815609471	6.602424674	8.99637432	9.505895854	9.5300868	8.763539652	8.476283084
ENSG00000180182	MED14	1.171890363	0.433017846	1.555658549	0.273747396	2.727548913	4.00722531	8.791896851	7.285536013	8.875861021	8.80807321	9.325140951	9.113466702
ENSG00000128159	TUBGCP6	0.964174018	0.63904641	1.762763648	0.358092441	2.726937665	2	6.602424674	8.922968322	8.562683721	8.55441692	9.332135462	9.368859419
ENSG00000183723	CMTM4	-0.400865562	0.486993352	3.122661312	1.37E-11	2.72179575	8.042902253	6.951736442	7.398165132	7.161462517	7.10341274	10.72240494	10.50587116
ENSG00000206127	GOLGA8O	7.912392298	1.05E-05	-5.191149049	0.042390794	2.721243249	2	2	2	7.994705029	7.92877397	2	2
ENSG00000065518	NDUFB4	1.870897843	0.002715259	0.84999279	0.207460653	2.720890634	8.418903792	7.638155693	6.80318718	9.584041242	9.65077635	8.480070636	8.718968947

ENSG00000278567		7.911711469	1.14E-05	-5.191148959	0.042837614	2.720562511	2	2	2	7.872924734	8.04484669	2	2
ENSG00000150347	ARID5B	0.644441967	0.311485333	2.075203831	0.000179121	2.719645798	9.247966931	10.3778065	10.60345421	10.84151467	10.8161359	12.37308854	12.13214626
ENSG00000196652	ZKSCAN5	1.432712851	0.223670516	1.286883826	0.271141542	2.719596677	4.467311984	7.928237109	6.629488387	8.30646685	8.37442884	8.364981826	8.006587762
ENSG00000006837	CDKL3	2.534170854	0.125092709	0.184862267	0.926455594	2.719033121	2	4.11352228	2	5.110902178	5.00774437	3.336159116	3.236138336
ENSG00000130340	SNX9	2.277146905	4.55E-07	0.440341668	0.406781563	2.717488573	8.910412251	9.497318007	9.999667074	11.77852445	11.8337041	10.02035217	9.925491821
ENSG00000155034	FBXL18	1.17438553	0.010955982	1.543052184	0.000501994	2.717437715	7.435667355	6.78762695	6.80318718	8.215198712	8.15227214	8.348878903	8.723602991
ENSG00000013810	TACC3	1.442976149	0.298925954	1.273863148	0.354405417	2.716839297	4.467311984	5.838937808	2	6.147608921	6.1572134	5.669467138	6.257923755
ENSG00000116473	RAP1A	1.617517759	6.67E-05	1.098823591	0.008939862	2.71634135	6.96351253	6.899080544	7.600047325	8.78154108	8.75316018	8.317350121	8.18854565
ENSG00000154845	PPP4R1	1.269502587	0.008153101	1.446571412	0.002075724	2.716073999	8.468948322	8.702538795	7.734844356	9.665524776	9.58096568	9.588555735	9.984600648
ENSG00000131943	C19orf12	1.521052903	0.021456873	1.194789446	0.075777971	2.715842349	7.33131396	8.732943944	7.451345288	9.481935579	9.52849248	9.071859866	9.282773417
ENSG00000157637	SLC38A10	1.275514678	0.000285776	1.440251073	3.13E-05	2.715765751	9.689059027	9.574900212	9.014156722	10.69791695	10.7571169	10.85119671	10.93206299
ENSG00000162409	PRKAA2	-0.453130259	0.769854407	3.167111114	0.012869116	2.713980855	2	6.535001748	5.39244166	5.161714397	5.13053105	8.682572389	8.53018147
ENSG00000131446	MGAT1	0.648162013	0.234880504	2.065133075	1.44E-05	2.713295088	10.88765625	10.80015962	9.935792933	11.25625289	11.2397407	12.37463584	12.90408303
ENSG00000148396	SEC16A	1.032292346	0.001737311	1.678848743	8.59E-08	2.711141089	9.635285994	10.01303782	9.452720984	10.73054016	10.7747077	11.47783828	11.31375888
ENSG00000106034	CPED1	0.37120571	0.808679443	2.339793206	0.067094792	2.710998916	4.815609471	8.834582582	6.718950348	7.966401501	8.01782901	9.681736371	10.16578017
ENSG00000184007	PTP4A2	1.230214989	0.013448518	1.48072325	0.002239989	2.710938239	9.317904228	9.785372135	10.36791565	11.10109884	11.1257404	11.16204184	11.54037635
ENSG00000135299	ANKRD6	-0.923545272	0.558941971	3.634376952	0.007051903	2.710831679	3.327711234	6.602424674	3.369317972	4.623314311	4.43005853	8.337438499	9.190804563
ENSG00000124459	ZNF45	1.326929285	0.39383571	1.383212314	0.362782971	2.710141599	6.96351253	6.951736442	2	7.68016596	7.6957855	7.814163164	7.66855289
ENSG00000136449	MYCBPAP	1.27173153	0.547559684	1.43593974	0.482681185	2.707671271	2	3.686303414	2	3.663521627	3.55551834	3.917810786	3.496556178
ENSG00000141252	VPS53	1.189571563	0.000120617	1.516241998	4.74E-07	2.705813562	8.340423424	8.101365653	8.408111533	9.436230969	9.48723044	9.651907244	9.910582344
ENSG00000119396	RAB14	1.472365635	0.145397751	1.232427883	0.223084039	2.704793518	6.653098118	9.479495904	9.272377397	10.42471067	10.3051703	10.16889595	10.08390135
ENSG00000153066	TXNDC11	1.248975624	0.44518671	1.455213756	0.356290523	2.70418938	8.963744667	7.638155693	3.369317972	9.138327453	9.09977716	9.085961465	9.528327138
ENSG00000156298	TSPAN7	-0.31396534	0.93407822	3.017927837	0.337194476	2.703962497	2	2	2	2.141737246	2.38539787	3.082820997	4.002845082
ENSG00000158715	SLC45A3	1.505109494	0.486454906	1.198558861	0.579500907	2.703668355	2	5.72254323	2	5.552894743	5.83486613	5.737015686	5.013446246
ENSG00000167996	FTH1	3.645269536	1.35E-06	-0.941740737	0.274614244	2.703528799	14.02224794	12.20053628	12.26418878	16.70361438	16.7763081	12.29446237	11.99973474
ENSG00000198715	GLMP	2.003086876	0.032193991	0.699505671	0.49591259	2.702592547	6.009025997	8.57407404	8.65477198	10.14797511	10.1190812	8.810024926	8.864540598
ENSG00000197937	ZNF347	0.749686183	0.393232855	1.94918618	0.01243418	2.698872362	5.095959458	6.951736442	5.59507254	6.892328675	6.80989959	8.161494476	7.869046644
ENSG00000117222	RBBP5	1.359031525	0.040747488	1.339720137	0.041517795	2.698751662	7.384433967	6.844429755	5.930865648	8.239055745	8.09310209	8.438967212	7.78508305
ENSG00000165168	CYBB	-2.307535798	0.518188676	5.004968725	0.063842507	2.697432926	2	2	2	2.141737246	2	5.436478516	4.911532223
ENSG00000160321	ZNF208	3.825406485	0.09741056	-1.128591071	0.666867266	2.696815414	2	2	3.369317972	4.965943888	5.04390545	2.492221014	2.059860178
ENSG00000135636	DYSF	0.526742625	0.57541332	2.169932554	0.006709168	2.696675179	4.00722531	5.946638857	6.322058544	6.213314212	6.21386141	7.994427465	7.581886257
ENSG00000198019	FCGR1B	2.391122996	0.505231655	0.303858541	NA	2.694981537	2	2	2	3.349643765	3.08597292	2.62493832	2.117335275
ENSG00000127415	IDUA	1.448205846	0.082711278	1.243668653	0.135257068	2.691874499	7.218819781	5.303523747	7.398165132	8.262524677	8.32752521	7.953205078	8.221681954
ENSG00000011258	MBTD1	1.649366555	0.011067153	1.042455474	0.122835348	2.691822029	4.815609471	6.04685525	6.203069917	7.450827758	7.33264456	7.012344451	6.559516442
ENSG00000117602	RCAN3	0.078997334	0.972415697	2.612704728	0.169351211	2.691702062	3.327711234	7.274681896	2	5.775854763	6.02777321	8.480618146	8.226587404
ENSG00000132275	RRP8	0.658538731	0.624897619	2.032532519	0.087371458	2.69107125	4.00722531	7.274681896	5.156626401	6.739036112	6.77805036	8.247650629	7.934467448
ENSG00000110237	ARHGEF17	0.367453575	0.745038229	2.32335018	0.013586383	2.690803755	7.53297911	9.616264339	10.42884603	9.922226014	10.031744	11.84735385	12.00753405
ENSG00000168310	IRF2	1.735573809	0.160777328	0.953568785	0.45939756	2.689142594	8.313285031	6.728495673	4.523675391	8.896009193	8.95655043	8.318575593	7.966097995
ENSG00000197782	ZNF780A	2.204626412	0.015668605	0.48249955	0.646493605	2.687125963	4.00722531	6.464272742	6.073378139	8.056427969	7.90193448	6.651077397	5.863612864
ENSG00000235194	PPP1R3E	0.940454805	0.688364462	1.745953082	0.424370697	2.686407887	2	6.228547981	2	5.709096143	5.6121243	6.453284622	6.380362005
ENSG00000117505	DR1	0.902390521	0.03191533	1.783457641	4.24E-06	2.685848162	8.009475818	8.147241016	8.742726175	9.256998085	9.19229617	10.21585362	9.977585641
ENSG00000128272	ATF4	1.257935558	0.254759301	1.426745434	0.182742647	2.684680992	11.59645304	10.35843478	8.677268317	11.90523218	11.905324	12.52778173	11.4079987
ENSG00000173226	IQCB1	0.977973036	0.522431564	1.706661253	0.227974555	2.684634288	4.00722531	6.844429755	3.369317972	6.503277904	6.51372389	7.382965824	7.017577533
ENSG00000133703	KRAS	0.935335497	0.040893562	1.748344497	3.45E-05	2.683679993	7.624139768	7.232742069	8.143093325	8.68062069	8.56534297	9.403744811	9.457418003
ENSG00000182504	CEP97	1.486511731	0.012898585	1.196968583	0.048314828	2.683480314	6.468621203	6.04685525	5.930865648	7.708923796	7.47849652	7.645704907	6.885273196
ENSG00000085224	ATRX	1.372507745	0.001997826	1.310309429	0.003174989	2.682817175	8.367060731	9.28800521	8.99637432	10.29841863	10.3065663	10.38974639	10.07395575
ENSG00000163818	LZTFL1	1.542985139	0.299070637	1.139137403	0.447404663	2.682122543	6.468621203	6.899080544	2	7.650823203	7.63485389	7.274744493	7.221358049
ENSG00000180113	TDRD6	-0.314150389	0.943613626	2.994915704	0.433184926	2.680765315	2	2	2	2.141737246	2.38539787	4.301748724	2.172608095
ENSG00000049245	VAMP3	1.060260805	0.004700034	1.620134281	5.85E-06	2.680395087	8.910412251	9.497318007	9.624627483	10.49986612	10.3622229	10.98995534	10.99246943
ENSG00000163626	COX18	0.892411519	0.197017026	1.787963263	0.004469801	2.680374782	5.866944937	5.131603625	6.43197674	6.86703417	6.55815769	7.75021102	7.411419562
ENSG00000043514	TRIT1	1.520871805	0.354199679	1.159215915	0.483045217	2.68008772	7.096806373	5.131603625	2	7.307758725	7.34356593	6.812870125	7.119550903
ENSG00000162522	KIAA1522	0.7244839	0.492298378	1.952589319	0.037315852	2.677073219	9.112843761	8.487557258	6.322058544	9.012478123	9.1617682	10.12596884	10.47511327
ENSG00000120733	KDM3B	1.322209443	0.318322589	1.352936174	0.297469212	2.675145617	5.70932728	9.599860728	7.552162999	9.728676049	9.71245786	9.697365195	9.80303401
ENSG00000143369	ECM1	0.967472598	0.152981087	1.706595965	0.006499774	2.674068563	10.7426185	9.608085847	9.636156974	11.0364838	11.0883544	11.3315637	12.15414401
ENSG00000169621	APLF	1.243007645	0.561101819	1.42930552	0.49125968	2.672313166	2	5.595927442	2	5.527288686	5.13053105	5.699879814	5.294527297
ENSG00000123552	USP45	1.606482441	0.226793445	1.065233203	0.434090596	2.671715644	6.257048221	5.595927442	2	6.984595707	6.90635305	6.651077397	6.158754168
ENSG00000164190	NIPBL	1.686982353	0.279279457	0.983993473	0.541021035	2.670975826	4.00722531	9.488434476	8.206335842	10.11182193	10.0907323	9.656275732	9.092123385
ENSG00000178295	GEN1	1.766442103	0.169077527	0.904419629	0.501207541	2.670861732	3.327711234	6.140560021	4.058998049	6.733448311	6.67228943	6.287958221	5.32534152
ENSG00000142634	EFHD2	1.728479323	0.002054399	0.937311808	0.115419908	2.665791131	7.384433967	7.393647515	8.536706852	9.595637371	9.56938265	8.761288263	8.831649431
ENSG00000067369	TP53BP1	1.417411793	0.029261156	1.247866721	0.055892984	2.665278514	7.790810999	9.338312924	9.242516611	10.35546265	10.3514424	10.15866828	10.20979627
ENSG00000095564	BTAF1	1.612292098	0.263419443	1.052978316	0.476093865	2.665270415	4.00722531	8.687092496	8.65477198	9.730079663	9.69763767	9.357098224	8.926648946
ENSG00000187650	VMAC	1.137179203	0.407386256	1.526832416	0.241370875	2.664011619	2	4.11352228	5.59507254	5.540148523	5.47317115	5.751606453	5.97274432
ENSG00000135835	KIAA1614	0.974697615	0.171596185	1.688530319	0.010410997	2.663227934	6.138360429	4.93638933	6.43197674	6.970413838	6.73998187	7.671830666	7.425709383
ENSG00000111731	C2CD5	1.689549335	0.26495819	0.973450133	0.535268829	2.662999467	2	6.951736442	6.073378139	7.677258395	7.64959655	7.172727426	6.723495198
ENSG00000161202	DVL3	0.742359942	0.041992389	1.920289348	5.40E-09	2.66264929	9.189468652	9.77810053	9.330306409	10.16934346	10.2281967	11.46440721	11.27807399
ENSG00000140443	IGF1R	0.779484532	0.149100125	1.882257119	9.95E-05	2.661741651	8.963744667	10.02537381	9.452720984	10.33761854	10.3135262	11.62184936	11.19927059
ENSG00000187398	LUZP2	-3.509010171	0.033794082	6.169764805	4.48E-07	2.660754634	2	4.11352228	3.369317972	2.141737246	2.2680111	8.056705941	9.551969768
ENSG00000162909	CAPN2	0.881955897	0.037940998	1.776511717	5.60E-06	2.658467614	10.35389212	10.65686569	11.11279376	11.60655646	11.6367136	12.31329535	12.69269276
ENSG00000166833	NAV2	2.006471123	0.286042726	0.650372099	0.749694888	2.656843223	2	8.791896851	6.882775709	9.465176303	9.62191107	8.402448739	7.968052066
ENSG00000141367	CLTC	1.210729687	0.041005338	1.446075035	0.012003587	2.656804722	10.93293345	12.0991443	10.9788475	12.65956989	12.6507234	12.76913358	13.00219207
ENSG00000133739	LRRCC1	2.015965816	0.170143299	0.637452859	0.691626719	2.653418674	3.327711234	4.710564154	2	5.552894743	5.41912973	4.705557986	3.735548302
ENSG00000043093	DCUN1D1	1.446082695	0.000365863	1.206230188	0.003365603	2.652312884	7.096806373	7.847167909	7.734844356	9.065699797	8.99400091	8.903637637	8.671790956
ENSG00000152404	CWF19L2	1.783069235	0.175251415	0.868256417	0.530574359	2.651325652	3.327711234	7.315436877	5.772716771	8.067573917	7.86198169	7.296052918	6.811011855
ENSG00000021762	OSBPL5	1.27021009	0.315574141	1.380423196	0.263184603	2.650633287	4.467311984	8.297207175	7.734844356	8.796356221	8.75179438	9.148628127	8.558340758
ENSG00000155097	ATP6V1C1	2.102017892	2.87E-10	0.547963962	0.146477581	2.649981855	8.138771685	8.791896851	8.434763955	10.56777138	10.5879489	9.062381638	8.999151483
ENSG00000117139	KDM5B	1.174790195	0.099215121	1.473908514	0.03159717	2.648698709	8.493334308	10.12043894	10.08627303	10.89536487	10.9234525	11.33027328	11.07445415
ENSG00000166908	PIP4K2C	0.965156699	0.216076248	1.683539008	0.020364713	2.648695707	7.031698319	7.393647515	8.721235202	8.864402784	8.84824797	9.356800024	9.755460113
ENSG00000213648	SULT1A4	7.839314907	1.74E-05	-5.19114876	0.044185492	2.648166147	2	2	2	7.917958914	7.86198169	2	2
ENSG00000261052	SULT1A3	7.839314907	1.74E-05	-5.19114876	0.044185492	2.648166147	2	2	2	7.917958914	7.86198169	2	2
ENSG00000101846	STS	0.385943899	0.849097902	2.261509726	0.186667231	2.647453625	2	7.979858146	8.296287027	7.999368766	7.90193448	9.427508673	10.11200841
ENSG00000136381	IREB2	1.328409677	0.062735523	1.315930785	0.062080537	2.644340461	7.485143553	9.067150531	7.777116083	9.590237441	9.62936766	9.4949131	9.693328953
ENSG00000155961	RAB39B	0.274454648	0.941409766	2.367105464	NA	2.641560113	2	2	2	2.3892346	2.38539787	3.429858444	2.950113106
ENSG00000092295	TGM1	-0.185862332	0.936649074	2.82709693	0.140416036	2.641234598	5.095959458	2	2	3.566290607	3.74316441	5.4091291	6.709547124
ENSG00000143951	WDPCP	0.945955489	0.663228562	1.692671387	0.403003915	2.638626876	2	5.595927442	2	5.22679461	4.91316568	5.740677235	5.768151538
ENSG00000123560	PLP1	0.914715675	0.822767511	1.72269462	0.65155864	2.637410294	4.00722531	2	2	4.237680128	2	3.5515855	4.479073019
ENSG00000125971	DYNLRB1	0.948663349	0.090124275	1.688744277	0.001093475	2.637407626	9.397585735	8.276714465	8.699419242	9.75855684	9.85029345	10.31281645	10.74087591
ENSG00000144834	TAGLN3	5.382677	0.006730413	-2.748740215	0.268972233	2.633936785	2	2	2	5.501219927	5.5755357	2.048074405	2.059860178
ENSG00000108306	FBXL20	1.2615036	0.042589119	1.372180394	0.024545065	2.633683994	8.16936288	6.951736442	8.266922072	9.135152476	9.15144695	9.463959918	9.006300384
ENSG00000137267	TUBB2A	1.126149674	0.400687122	1.507270483	0.238341966	2.633420157	8.946185348	7.145017454	5.156626401	8.983347946	8.84312657	8.792933324	9.664095852
ENSG00000186399	GOLGA8R	7.824182883	2.01E-05	-5.191148647	0.044848802	2.633034235	2	2	2	7.93509946	7.81305436	2	2
ENSG00000189367	KIAA0408	7.824023956	1.94E-05	-5.191148689	0.044648908	2.632875266	2	2	2	7.908072101	7.84158259	2	2
ENSG00000130699	TAF4	2.113665643	0.315369213	0.519153485	0.823280183	2.632819127	2	5.946638857	2	6.496695859	6.48770488	5.264018433	4.729989799
ENSG00000111358	GTF2H3	1.670847243	0.058804128	0.960793281	0.296967694	2.631640524	4.815609471	7.232742069	6.43197674	8.076429088	8.13979554	7.17543541	7.614687532
ENSG00000260097	SPDYE6	2.273109036	0.486515971	0.356263819	NA	2.629372855	2	2	2	3.023133209	3.28037825	2.34604626	2.466008509
ENSG00000175265	GOLGA8A	2.995602965	8.23E-09	-0.368619795	0.562234526	2.626983171	7.904282243	8.862353853	7.646393253	11.24304106	11.2104591	7.959503304	7.805990373
ENSG00000147036	LANCL3	-1.317080176	0.761841469	3.942291544	0.282983706	2.625211368	2	2	2	2	2.2680111	4.301748724	4.292061816
ENSG00000171116	HSFX1	3.148163868	0.280922232	-0.523007381	0.877195052	2.625156487	2	2	2	3.615725078	3.7864959	2.139668783	2.326754077
ENSG00000074047	GLI2	1.507969014	0.41134066	1.116865386	0.545217341	2.6248344	2	8.029695761	6.073378139	8.264463296	8.28661061	8.189226297	7.512619696
ENSG00000058335	RASGRF1	0.271993944	0.944373487	2.351960904	NA	2.623954847	2	2	2	2.270786764	2.49394783	2.68698273	3.580696614
ENSG00000140873	ADAMTS18	-0.302885678	0.955377328	2.926666065	0.542075443	2.623780387	2	2	2	2.498691189	2	3.796405078	3.261853426
ENSG00000186815	TPCN1	0.415328255	0.817831695	2.208198329	0.147401264	2.623526583	4.467311984	7.874700259	9.990713521	9.09649754	9.18421833	10.76830805	11.06922971
ENSG00000108511	HOXB6	5.45365659	0.000530987	-2.830269874	0.111750609	2.623386715	2	4.11352228	2	7.844695415	7.77859536	2.384001718	2.059860178
ENSG00000169169	CPT1C	1.548161696	0.239494508	1.074691805	0.423751061	2.622853501	7.159102577	7.538394143	3.369317972	8.332573384	8.35477078	7.633939843	8.085210331
ENSG00000276802		2.731243082	0.021534651	-0.108592474	0.942222441	2.622650608	6.257048221	9.067150531	5.930865648	10.50397439	10.5542639	7.608114885	7.811442311
ENSG00000215695	RSC1A1	7.813253426	2.13E-05	-5.191148622	0.045052759	2.622104804	2	2	2	7.815902688	7.91175216	2	2
ENSG00000143321	HDGF	1.154053059	0.144865788	1.46697271	0.053383916	2.621025769	8.674867122	10.33384851	10.66665689	11.24008859	11.2904776	11.49263913	11.65852437
ENSG00000197566	ZNF624	0.8275107	0.634493636	1.791986234	0.260698796	2.619496934	2	5.838937808	3.369317972	5.289065324	5.40529704	6.423979439	6.033722345

ENSG00000101654	RNMT	1.43278937	0.041269777	1.18541715	0.094023077	2.61820652	7.031698319	8.848535039	8.296287027	9.648553319	9.68266366	9.402011675	9.437105934
ENSG00000119471	HSDL2	0.850381466	0.6915899	1.767075949	0.370584029	2.617457416	2	8.762721501	5.772716771	8.205140657	8.19711812	9.162002347	9.053075307
ENSG00000125551	PLGLB2	5.470650263	0.033330344	-2.853750004	0.349664874	2.61690026	2	2	2	5.602782483	5.63601114	2.094598369	2
ENSG00000135632	SMYD5	1.243603718	0.585257988	1.371208582	0.536695429	2.6148123	2	7.954278496	3.369317972	7.653784481	7.67863747	7.765568476	7.816873723
ENSG00000155545	MIER3	1.666752732	0.011919917	0.947738912	0.173968415	2.614491644	6.366709671	7.572420273	6.43197674	8.557949361	8.55598286	8.142936557	7.476011586
ENSG00000160293	VAV2	1.457189068	0.412532734	1.155962042	0.516505072	2.61315111	8.009475818	6.311476754	2	8.139053376	8.35836495	8.082904238	7.816873723
ENSG00000204262	COL5A2	1.327112453	0.092649431	1.28480411	0.098958811	2.611916563	11.65437133	13.45660594	12.03516535	13.91902576	13.9406531	13.70141217	14.05244606
ENSG00000146834	MEPCE	1.235805372	0.502698475	1.3756491	0.44235966	2.611454472	2	8.004992147	8.296287027	8.807036108	8.79752133	9.036921734	8.838074258
ENSG00000256374	PPIAL4D	7.800514757	2.26E-05	-5.1911486	0.045228764	2.609366158	2	2	2	7.847284664	7.85690889	2	2
ENSG00000271567	PPIAL4E	7.800514757	2.26E-05	-5.1911486	0.045228764	2.609366158	2	2	2	7.847284664	7.85690889	2	2
ENSG00000275974		7.800514757	2.26E-05	-5.1911486	0.045228764	2.609366158	2	2	2	7.847284664	7.85690889	2	2
ENSG00000133114	GPALPP1	1.06184983	0.060429834	1.547300531	0.003794368	2.60915036	6.468621203	7.189546432	6.43197674	7.842101511	7.74329312	8.53381827	7.950369405
ENSG00000167110	GOLGA2	1.181272242	0.022811457	1.425481202	0.004716327	2.606753443	9.689059027	8.557180776	9.478569125	10.46530827	10.5149509	10.64428999	10.8183324
ENSG00000132819	RBM38	0.129642725	0.88983543	2.476584016	0.00040416	2.606226741	4.467311984	5.595927442	6.203069917	5.614989456	5.78232107	7.637872201	8.216759768
ENSG00000006576	PHTF2	0.723389123	0.685840372	1.882243648	0.242785116	2.605632771	4.467311984	8.955547846	5.156626401	8.180711314	8.31278108	9.52945581	9.250223518
ENSG00000153790	C7orf31	1.100908968	0.34242241	1.504155948	0.170620999	2.605064916	5.095959458	4.710564154	2	5.602782483	5.16377554	5.83618166	5.699242824
ENSG00000127364	TAS2R4	0.855108633	NA	1.749858381	NA	2.604967015	2	2	2	2.270786764	2.77779907	3.296906311	2.326754077
ENSG00000106367	AP1S1	1.033844826	0.299078862	1.569820974	0.093567925	2.6036658	9.174465797	7.874700259	6.534109959	9.250166145	9.27068036	9.75507596	9.830950469
ENSG00000173273	TNKS	1.413252573	0.254264315	1.189321379	0.337150716	2.602573952	5.095959458	8.791896851	8.699419242	9.650037049	9.59092963	9.518566517	9.266590264
ENSG00000263528	IKBKE	2.534468276	0.215468947	0.066297591	0.977728724	2.600765866	3.327711234	7.572420273	2	8.573670512	8.56222967	5.626574484	6.559516442
ENSG00000124222	STX16	1.498094994	0.217082182	1.102165807	0.369236619	2.600260801	8.816894989	9.574900212	5.772716771	10.19853443	10.2335885	9.861691911	9.780694439
ENSG00000062716	VMP1	1.731862494	0.006082629	0.864924034	0.19944136	2.596786528	10.57565445	9.45234302	10.76445573	12.08888579	12.1023812	11.58483423	10.75777696
ENSG00000272414	FAM47E-STB	7.786890439	2.65E-05	-5.191148445	0.046085237	2.595741994	2	2	2	7.739909082	7.93118934	2	2
ENSG00000168481	LGI3	-0.310502485	0.931034311	2.904513777	0.323536602	2.594011292	2	2	2	2.270786764	2.2680111	3.679182183	3.383972333
ENSG00000172888	ZNF621	1.121226836	0.562222129	1.469413257	0.430383523	2.590640092	2	7.761270542	8.845626171	8.914645924	8.95180002	9.388946358	9.159937761
ENSG00000127666	TICAM1	0.916365612	0.628420441	1.673802696	0.343240742	2.590168308	9.064836028	5.72254323	2	8.592312514	8.48870446	9.059452688	9.493138564
ENSG00000146083	RNF44	0.905325365	0.375529496	1.68479109	0.073836453	2.590116454	6.468621203	8.505280662	6.322058544	8.372666678	8.37442884	9.180655169	9.109931338
ENSG00000078142	PIK3C3	1.684925839	0.00948606	0.904859811	0.188307274	2.58978565	7.485143553	8.414402626	6.882775709	9.41544358	9.40880824	8.408216037	8.836470732
ENSG00000261862		7.776944904	2.63E-05	-5.1911485	0.045860622	2.585796404	2	2	2	7.842101511	7.81567127	2	2
ENSG00000145012	LPP	0.145169276	0.722229354	2.440179756	4.77E-15	2.585349032	9.848787621	9.583268459	10.14394518	10.02414665	10.0119132	12.43082064	12.17507145
ENSG00000067596	DHX8	1.281368151	0.273690508	1.30359691	0.25635543	2.584965061	5.532353208	8.981105197	8.561105505	9.52946469	9.56705484	9.409506941	9.715307813
ENSG00000214711	CAPN14	0.010001629	0.997481474	2.574723422	NA	2.58472505	2	2	2	2.270786764	2.38539787	3.5515855	3.071407821
ENSG00000185924	RTN4RL1	-0.723399098	0.841155419	3.307633494	0.259517738	2.584234396	2	2	2	2.270786764	2.14022021	4.188345803	3.311948089
ENSG00000064313	TAF2	1.371088271	0.207448076	1.212827346	0.262333756	2.583915617	5.095959458	7.274681896	8.296287027	8.777474042	8.69749358	8.541710459	8.619914108
ENSG00000197375	SLC22A5	0.909563536	0.705604924	1.673354985	0.456551187	2.582918521	2	6.389896633	2	5.890757403	5.67111583	6.647180917	6.347714043
ENSG00000169155	ZBTB43	1.681598136	0.000326631	0.901126222	0.070466789	2.582724358	7.667647223	7.467835385	6.718950348	9.005540335	8.99861459	8.018757917	8.413855585
ENSG00000123091	RNF11	0.815993495	0.371365629	1.765690331	0.033376067	2.581683826	9.940323746	9.763446433	7.691296561	10.25471453	10.2173523	11.08384263	11.27679362
ENSG00000204446	C9orf170	2.444644759	0.440624206	0.135666309	NA	2.580311068	2	2	2	3.289987342	3.2184422	2.384001718	2.326754077
ENSG00000173221	GLRX	2.388424651	1.42E-06	0.190855488	0.761845241	2.579280139	8.228665467	7.002537973	7.691296561	10.04910261	10.1111143	7.902621621	7.90110545
ENSG00000024422	EHD2	0.290813673	0.721168354	2.288357336	0.000505952	2.579171009	12.05742222	10.67655334	10.7904855	11.58169902	11.6378232	13.19868131	13.92358827
ENSG00000136169	SETDB2	1.961157148	0.190847995	0.609854535	0.710603737	2.571011683	2	6.844429755	6.718950348	8.22518713	8.03365075	7.090465544	6.468583424
ENSG00000151338	MIPOL1	0.922626262	0.693189026	1.647809572	0.451377576	2.570435835	2	6.04685525	2	5.363304456	5.5755357	6.473229652	5.722580018
ENSG00000188026	RILPL1	1.0572236	0.253880504	1.512533031	0.084716522	2.569756631	7.867441304	6.844429755	5.39244166	7.992367495	8.12087597	8.434452636	8.577026237
ENSG00000138658	ZGRF1	0.993995515	0.483854018	1.575195068	0.233466011	2.569190583	3.327711234	4.710564154	2	4.670864177	4.53541753	5.19095825	5.013446246
ENSG00000135744	AGT	-2.30028285	0.211593503	4.868289281	0.002344194	2.568006431	2	2	5.39244166	2.695456494	2.68924543	8.119222541	8.777523662
ENSG00000285437	POLR2J3	4.601493261	2.69E-08	-2.034036654	0.025714394	2.567456607	4.815609471	3.686303414	5.930865648	9.452477833	9.47652116	3.172301926	3.717076715
ENSG00000137965	IFI44	-3.753125373	0.302544659	6.32026359	0.028809061	2.567138217	2	2	2	2	2	6.396389756	6.435070131
ENSG00000153767	GTF2E1	1.666923177	0.228980535	0.898902049	0.534155222	2.565825226	3.327711234	7.467835385	5.59507254	7.925329813	7.93118934	7.377096489	6.944034147
ENSG00000127914	AKAP9	1.745130342	0.010140946	0.820306821	0.260698796	2.565437164	7.384433967	9.170736405	8.65477198	10.34359113	10.290665	9.475542327	9.313068526
ENSG00000186453	FAM228A	-0.103112999	0.968293784	2.668294011	0.204564701	2.565181012	3.327711234	2	2	2.498691189	2.59489879	4.251304078	3.824516949
ENSG00000189319	FAM53B	0.957511745	0.579274794	1.606441988	0.320401275	2.563953733	2	7.431218269	7.225742779	7.608714138	7.7866208	8.589193677	8.036691948
ENSG00000130956	HABP4	0.743630791	0.688362385	1.818964001	0.278961643	2.562594791	8.138771685	7.145017454	2	7.875463856	7.88956768	8.985384832	8.903351429
ENSG00000181885	CLDN7	2.291414727	0.261829517	0.270617475	0.909261712	2.562032202	4.00722531	2	2	4.139692846	4.79082474	3.316666208	2.851942824
ENSG00000132824	SERINC3	1.154034432	9.21E-06	1.405904066	3.59E-08	2.559938498	10.4253394	10.62884366	10.26027233	11.63211941	11.5677554	11.76575458	11.93315092
ENSG00000196220	SRGAP3	1.345042367	0.52794944	1.212943004	0.563495697	2.557985371	2	4.442710129	2	4.54891529	4.12711147	4.720468739	3.539239711
ENSG00000123607	TTC21B	1.516897314	0.219156141	1.039882744	0.409012973	2.556780057	3.327711234	7.099070191	6.629488387	7.872924734	7.80517502	7.570548195	7.142226902
ENSG00000110841	PPFIBP1	1.538255894	0.062284456	1.018067363	0.2306006	2.556323257	7.218819781	9.338312924	9.272377397	10.42037002	10.4062943	9.913096751	9.876656812
ENSG00000196843	ARID5A	1.298725242	0.419943377	1.257299995	0.42731388	2.556025238	8.777722034	7.145017454	3.369317972	8.902248225	8.90218458	8.696779192	9.0086755
ENSG00000163170	BOLA3	1.49909005	0.07045483	1.056459374	0.211464999	2.555549425	6.366709671	6.04685525	4.058998049	7.2696592	7.18663697	6.67615119	6.913939481
ENSG00000179387	ELMOD2	1.086624195	0.532648303	1.468721361	0.377510751	2.555346006	2	7.572420273	6.203069917	7.59952861	7.48508724	8.175765162	7.604674031
ENSG00000084628	NKAIN1	2.975834417	0.562661877	-0.421393043	0.94131204	2.554441374	2	2	2	3.709785322	3.45167086	2	2.466008509
ENSG00000139112	GABARAPL1	1.325307733	6.09E-05	1.228839949	0.00020825	2.554147683	10.27168932	9.74864196	9.703463497	11.22970711	11.2743971	11.16459958	11.14718668
ENSG00000213988	ZNF90	1.624378619	0.392606579	0.92870637	0.635038067	2.553084989	3.327711234	3.076665209	2	4.139692846	3.65239003	3.694366604	3.128433649
ENSG00000155307	SAMSN1	-0.313998298	0.935584164	2.865793269	0.376931525	2.551794971	2	2	2	2.141737246	2.38539787	3.917810786	2.950113106
ENSG00000183153	GJD3	3.301116068	0.006082261	-0.750293682	0.593027095	2.550822387	3.327711234	4.442710129	2	6.463327516	6.49425375	2.911488689	3.430065404
ENSG00000125844	RRBP1	0.91323918	0.001774792	1.63620575	3.24E-09	2.54944493	10.81695923	10.56689847	11.06667478	11.72409419	11.7574918	12.45002848	12.47623726
ENSG00000128245	YWHAH	0.424797486	0.439449514	2.124373735	3.29E-06	2.549171221	8.696033109	9.246456519	9.798878489	9.746817367	9.7285167	11.46031868	11.40430528
ENSG00000124134	KCNS1	-1.303810926	0.768597193	3.852061139	0.305716361	2.548250213	2	2	2	2.270786764	2	4.04171018	4.388595494
ENSG00000013392	RWDD2A	0.84586136	0.082516252	1.702059951	0.000118641	2.54792131	6.009025997	6.844429755	6.629488387	7.423326516	7.31425651	8.166947586	8.252471774
ENSG00000172824	CES4A	1.701061056	0.384580714	0.846831014	0.679226319	2.547892069	2	4.93638933	2	5.289065324	4.95174433	4.04171018	4.692661645
ENSG00000141279	NPEPPS	1.760894541	0.069176139	0.78634686	0.447739474	2.547241401	8.340423424	10.06773758	7.502634626	10.76003928	10.8333485	9.80554977	9.845638987
ENSG00000064393	HIPK2	1.114461615	0.23620818	1.432592354	0.112072333	2.547053969	7.485143553	9.835272298	9.601288346	10.3851665	10.4036876	10.32506285	11.01638973
ENSG00000143319	ISG20L2	0.737044267	0.068175471	1.808398	5.97E-07	2.545442266	7.096806373	7.701025996	7.225742779	8.115618198	8.09310209	9.164389779	9.165057331
ENSG00000087470	DNM1L	1.537643742	0.349323187	1.005855687	0.548363823	2.543499429	5.866944937	9.883504052	5.59507254	9.95740528	10.0102007	9.472242545	9.437105934
ENSG00000121644	DESI2	0.860629953	0.171930246	1.681501387	0.003528509	2.54213134	8.777722034	8.607278467	7.502634626	9.273456479	9.23006009	10.31895264	9.765606869
ENSG00000003987	MTMR7	-1.303813348	0.768464398	3.845444016	0.306126091	2.541630668	2	2	2	2.270786764	2	4.301748724	4.133950982
ENSG00000164674	SYTL3	2.58059919	0.00141337	-0.040851049	0.969279885	2.539748141	4.467311984	5.303523747	3.369317972	6.936770382	7.0991228	4.504874511	4.711446447
ENSG00000178202	KDELC2	0.53954224	0.574668905	1.997651743	0.016599301	2.537193983	8.340423424	9.214490408	6.882775709	9.017084849	8.92660044	10.15764151	10.64763892
ENSG00000101216	GMEB2	0.806738536	0.56521797	1.728468153	0.177968622	2.535206689	7.096806373	7.638155693	3.369317972	7.656739692	7.63485389	8.464101787	8.638437331
ENSG00000169442	CD52	-1.279450278	0.745616581	3.814446666	0.264665347	2.534996389	3.327711234	2	2	2	2.49394783	5.873348082	2.374681325
ENSG00000107281	NPDC1	1.493609048	0.320964241	1.040700652	0.496230402	2.5343097	4.815609471	8.169643011	9.897982195	10.19345574	10.2252472	9.792379418	9.72342817
ENSG00000131848	ZSCAN5A	0.836427041	0.689206562	1.697005253	0.377073141	2.533432294	2	5.131603625	2	4.54891529	4.58534546	5.008609438	5.566251551
ENSG00000177666	PNPLA2	1.078955795	0.225518037	1.453762856	0.087242653	2.532718651	9.922475255	8.717821467	7.777116083	10.13690654	10.1458442	10.13138331	10.81779073
ENSG00000143622	RIT1	1.585418608	0.391687888	0.94708529	0.61896509	2.532503898	8.674867122	8.297207175	2	9.50916971	9.47486651	8.89667997	8.818713298
ENSG00000162733	DDR2	0.254337744	0.741606385	2.278049059	0.000224741	2.532386803	9.820154628	8.747909548	10.38167985	10.02357442	10.0700583	12.17700352	11.94702543
ENSG00000084636	COL16A1	1.179449462	0.095026336	1.352578163	0.048281896	2.532027625	9.174465797	8.522788974	10.09465441	10.56305371	10.5971045	10.41603173	11.025801
ENSG00000110514	MADD	1.47390807	0.257133882	1.057977615	0.423741616	2.531885684	4.467311984	8.505280662	8.236946975	9.235416412	9.36213202	8.68637457	9.063622361
ENSG00000183474	GTF2H2C	5.909137245	4.06E-07	-3.378556965	0.015050726	2.53058028	3.327711234	2	4.058998049	8.429064458	8.41985547	2.094598369	2.277179783
ENSG00000146963	LUC7L2	1.983153886	3.36E-05	0.546578297	0.314407502	2.529732183	7.940205782	6.844429755	7.646393253	9.446513412	9.5372395	7.992893101	8.142569067
ENSG00000108179	PPIF	2.19491778	4.77E-08	0.334323244	0.487891953	2.529241024	8.39321512	8.981105197	8.699419242	10.86534179	10.9331253	8.720150661	9.313452824
ENSG00000169031	COL4A3	-2.665163286	0.252535231	5.193943043	0.005963489	2.528779757	2	2	4.058998049	2	2.38539787	7.218085825	7.171352322
ENSG00000100811	YY1	0.52500514	0.231434376	2.003157498	1.08E-07	2.528162638	9.233563228	9.540933215	8.763901704	9.696010185	9.78502553	11.33497397	11.08417113
ENSG00000048991	R3HDM1	1.145139374	0.428693153	1.380051718	0.320805825	2.525191093	4.00722531	8.433041974	6.882775709	8.485003881	8.42329129	8.374440168	8.943630224
ENSG00000143190	POU2F1	1.468575201	0.077266405	1.055013595	0.212493403	2.523588796	5.866944937	7.928237109	6.629488387	8.540455898	8.50013767	8.191234678	8.028288444
ENSG00000167280	ENGASE	1.091304147	0.517792902	1.432142201	0.372318292	2.523446348	2	6.535001748	7.451345288	7.481152682	7.62891439	8.014983227	7.755972999
ENSG00000147894	C9orf72	2.012818281	0.271053897	0.509362906	0.801100986	2.522181187	4.00722531	7.099070191	2	7.638916932	7.68437616	6.520853931	5.812327524
ENSG00000112357	PEX7	1.571106967	0.190366567	0.950429059	0.442284492	2.521536026	2	4.710564154	5.59507254	6.139181293	6.09824986	5.50697268	5.576534358
ENSG00000131469	RPL27	0.703091552	0.531143362	1.817369016	0.071495019	2.520460568	10.81212061	8.655694736	8.266922072	10.40375541	10.4114935	11.55927357	11.47883521
ENSG00000119013	NDUFB3	1.21038275	0.001829326	1.30802218	0.000603619	2.51840493	6.89194353	6.728495673	7.029880454	8.102673083	8.02009991	8.038228667	8.269209446
ENSG00000185585	OLFML2A	-2.235460683	0.013585291	4.752668595	6.73E-09	2.517207912	5.70932728	7.274681896	5.156626401	4.670864177	3.98554648	10.83029933	11.23835038
ENSG00000175455	CCDC14	1.572189539	0.254887129	0.932910458	0.513742625	2.505099997	4.00722531	8.356994601	8.110401118	9.309580714	9.18421833	8.939525293	8.193564875
ENSG00000278760		7.695995495	4.72E-05	-5.191148055	0.048550418	2.50484744	2	2	2	7.671425626	7.82349365	2	2
ENSG00000185483	ROR1	-0.816466534	0.581462748	3.320506394	0.007450401	2.504039861	3.327711234	5.303523747	2	3.79808662	3.45167086	7.20223931	7.296373648
ENSG00000023572	GLRX2	1.28881391	0.249770351	1.215183407	0.271243655	2.503997318	6.653098118	5.595927442	3.369317972	7.017154451	6.89154576	6.958334233	6.806650651
ENSG00000010310	GIPR	0.236667037	0.915905242	2.265820918	0.226351497	2.502487955	2	5.303523747	2	3.997744386	4.43005853	6.132200015	5.867805474
ENSG00000196151	WDSUB1	1.067123085	0.444480686	1.43523671	0.278276141	2.502359795	3.327711234	4.11352228	6.534109959	6.18083559	6.3857277	6.651077397	6.62538071
ENSG00000125945	ZNF436	0.74086527	0.717844348	1.761163083	0.347741804	2.502028354	8.444143041	5.838937808	2	7.877998516	7.74878077	8.737663157	8.901818877

ENSG00000189060	H1F0	1.431559757	0.048733067	1.069711277	0.147317101	2.501271034	10.74769552	9.514922632	11.37608156	12.13716306	12.186169	11.66216814	11.92650878
ENSG00000156304	SCAF4	1.293957564	0.482463711	1.206412065	0.506531437	2.500369628	7.579279349	8.487557258	2	8.785596684	8.83153649	8.676850263	8.765776654
ENSG00000187790	FANCM	1.629638197	0.076551676	0.870217543	0.365505781	2.49985574	3.327711234	4.442710129	5.156626401	6.052081244	5.78232107	5.385935071	5.050803619
ENSG00000153339	TRAPPC8	1.495933512	0.207531949	1.003241164	0.408534991	2.499174677	8.042902253	7.874700259	4.523675391	8.908460392	8.93143442	8.63860224	8.191057445
ENSG00000181192	DHTKD1	1.0337798	0.25067706	1.464634602	0.085942697	2.498414401	5.70932728	6.535001748	7.777116083	7.917958914	7.93360068	7.943705881	8.667588528
ENSG00000065457	ADAT1	1.189132353	0.179882373	1.309187903	0.128338636	2.498320256	5.532353208	7.847167909	6.80318718	8.219202381	8.17078703	8.322856568	8.304435067
ENSG00000104626	ERI1	0.746256918	0.29276914	1.750095194	0.005691812	2.496352112	5.095959458	6.464272742	5.59507254	6.628980152	6.51372389	7.652523812	7.429968874
ENSG00000130821	SLC6A8	2.001015341	0.198333509	0.494824257	0.775687249	2.495839597	4.467311984	8.820493869	5.39244166	9.349396108	9.47072153	8.050081351	7.778417291
ENSG00000116106	EPHA4	3.684371466	0.020403301	-1.188599506	0.503356521	2.49577196	2	5.303523747	2	7.481152682	7.27676203	3.27687201	3.236138336
ENSG00000145632	PLK2	1.872170586	0.413198702	0.623213664	0.799962733	2.495384249	10.11549941	6.464272742	2	10.50970639	10.5040407	9.810566697	8.37093032
ENSG00000124664	SPDEF	2.215432512	0.511909846	0.278914973	NA	2.494347485	2	2	2	3.349643765	2.86123014	2.3070652	2.466008509
ENSG00000166813	KIF7	1.066525759	0.040530546	1.427119586	0.004135001	2.493645345	7.031698319	6.535001748	7.646393253	8.15586117	8.18299958	8.52906215	8.522223965
ENSG00000173548	SNX33	0.434060427	0.808247156	2.059537274	0.179215774	2.493597701	4.467311984	8.077869079	10.05225195	9.206465443	9.28774494	10.75705321	10.9686339
ENSG00000135074	ADAM19	1.304036262	0.046986259	1.189527545	0.069532318	2.493563808	9.667789716	9.540933215	10.86587029	11.44329506	11.4685634	11.5630268	11.07999859
ENSG00000186480	INSIG1	2.166083015	0.017108279	0.325758971	0.762594103	2.491841986	8.418903792	10.75272247	10.31867969	12.28593974	12.2856726	9.739148276	10.92189172
ENSG00000108175	ZMIZ1	1.200454181	0.244599958	1.290918513	0.199257862	2.491372695	7.904282243	10.62073637	10.44204362	11.24843827	11.2703488	10.99331648	11.63534878
ENSG00000174373	RALGAPA1	1.260683513	0.341930387	1.229694764	0.346574255	2.490378277	3.327711234	7.051611315	7.398165132	8.008651253	7.84158259	8.025527571	7.755972999
ENSG00000008324	SS18L2	1.613313993	0.022108229	0.875650652	0.238915711	2.488964645	5.70932728	5.457120953	4.058998049	6.761173376	6.76185814	6.170709933	5.937276556
ENSG00000124766	SOX4	3.098312539	2.36E-05	-0.610264177	0.47862258	2.488048362	8.737455672	7.503546079	7.163363831	11.04016618	11.0485841	7.808940772	6.730418989
ENSG00000185386	MAPK11	-3.753121909	0.311453673	6.240599322	0.035923719	2.487477412	2	2	2	2	2	6.492902701	6.168983563
ENSG00000153046	CDYL	0.843555674	0.016138255	1.64369842	3.93E-07	2.487254094	7.485143553	7.60566235	7.600047325	8.48998335	8.2960118	9.080915197	9.289821273
ENSG00000146872	TLK2	1.679598658	0.163759565	0.806919066	0.525667607	2.486517724	7.709880939	8.337339476	4.523675391	9.18717761	9.20931286	8.324077374	8.343344553
ENSG00000104904	OAZ1	1.069526125	0.002717218	1.415969572	4.04E-05	2.485495697	11.69698977	11.05003091	11.39656351	12.46319341	12.4818262	12.68103423	12.94449676
ENSG00000213096	ZNF254	1.884778024	0.001311774	0.600601609	0.360717166	2.485379633	5.330594134	5.72254323	6.43197674	7.753776273	7.64666005	6.656902456	6.222348126
ENSG00000179855	GIPC3	-0.904009055	0.633283628	3.389106825	0.03824337	2.48509777	4.00722531	2	6.718950348	4.329433326	4.79082474	8.770720575	8.456236734
ENSG00000095015	MAP3K1	0.372792669	0.832844524	2.109713268	0.154214134	2.482505937	2	7.051611315	5.930865648	6.604700657	6.18176397	8.141552353	8.051511707
ENSG00000005700	IBTK	1.746478042	0.000552485	0.735830003	0.181128882	2.482308045	7.867441304	9.00621767	8.825624954	10.39846869	10.3733676	9.21856602	9.524680706
ENSG00000099942	CRKL	0.910901593	0.551800049	1.569740805	0.273134268	2.480642399	4.815609471	9.433952963	9.735974718	9.856914282	10.0187431	10.61778047	10.57545478
ENSG00000234616	JRK	0.584544044	0.620673635	1.894815599	0.067784655	2.479359643	7.031698319	7.189546432	4.058998049	7.106324235	7.26532099	8.620333861	8.312147538
ENSG00000121895	TMEM156	3.220678467	0.000172096	-0.74200312	0.45939756	2.478675347	4.467311984	4.442710129	4.874587708	7.54315232	7.6318872	3.429858444	4.4231929
ENSG00000134644	PUM1	1.217741635	0.011872202	1.259477679	0.008608574	2.477219314	8.737455672	9.566483142	8.608698767	10.24099634	10.2683912	10.30973854	10.28285103
ENSG00000091513	TF	0.009998476	0.997481474	2.465808835	NA	2.475807311	2	2	2	2.270786764	2.38539787	3.393105702	3.128433649
ENSG00000136122	BORA	2.127473214	0.263300675	0.347848986	0.872577887	2.4753222	2	5.303523747	2	5.808110275	5.94414054	4.271694079	4.365060851
ENSG00000127980	PEX1	1.252104972	0.4623472	1.223057643	0.463451338	2.475162614	5.095959458	7.051611315	2	7.017154451	7.07311172	7.278527746	6.697819825
ENSG00000168564	CDKN2AIP	1.240394697	0.285861007	1.232472249	0.279466521	2.472866946	4.00722531	7.051611315	7.285536013	7.797276258	7.95274764	8.122032742	7.562617572
ENSG00000166747	AP1G1	1.016028867	0.000801566	1.456445392	5.93E-07	2.472474259	9.189468652	9.591588447	9.636156974	10.48206602	10.5161581	10.8420647	11.02908049
ENSG00000070366	SMG6	1.507760942	0.209654079	0.964260603	0.434758717	2.472021545	4.815609471	8.451443573	8.236946975	9.271529914	9.36392108	8.753154049	8.804567006
ENSG00000128965	CHAC1	0.697611415	0.745616581	1.772721154	0.365400061	2.470332569	2	6.140560021	8.076950877	7.467753569	7.56813436	9.352020423	6.786860422
ENSG00000167555	ZNF528	1.148438995	0.313233304	1.321623977	0.230545768	2.470062972	7.53297911	5.131603625	5.156626401	7.517371202	7.51437845	7.836577593	7.51663051
ENSG00000079263	SP140	3.74034959	0.024807493	-1.270348852	0.500318204	2.470000738	3.327711234	2	3.369317972	5.697663076	5.84514933	2.68698273	2.225841198
ENSG00000197381	ADARB1	-1.001646519	0.424331051	3.470721552	0.001279761	2.469075033	7.867441304	8.255926468	4.523675391	6.61689148	6.55189318	10.90419513	11.08709598
ENSG00000101000	PROCR	2.899772903	0.031612654	-0.431391148	0.787507734	2.468381755	7.904282243	5.838937808	3.369317972	9.539105795	9.51083771	6.159811591	6.359671687
ENSG00000076201	PTPN23	0.941661613	0.102251384	1.52640918	0.004583447	2.468070793	8.107517749	9.067150531	7.97167868	9.403177828	9.42088976	9.836890387	10.13567188
ENSG00000163848	ZNF148	1.040250431	0.262504755	1.427427064	0.106512505	2.467677495	9.015175615	8.297207175	6.534109959	9.375641031	9.21922971	9.817951213	9.537567589
ENSG00000132535	DLG4	1.436259864	0.399184147	1.03132221	0.548805363	2.467582074	9.081016759	6.728495673	3.369317972	9.18103275	9.20931286	8.832503242	8.753366804
ENSG00000116044	NFE2L2	1.627523515	0.007030767	0.839950964	0.190829323	2.46747448	9.128497779	9.308339287	10.40208303	11.34824023	11.3526149	10.76314172	10.33393985
ENSG00000198265	HELZ	0.884874463	0.140909508	1.580406765	0.004461713	2.465281228	8.586954094	9.214490408	7.858115942	9.532685556	9.55143939	10.31542752	10.14908313
ENSG00000167123	CERCAM	1.477743315	0.041225744	0.986422399	0.185611962	2.464165714	11.26581873	10.45279253	9.439621147	12.035819	12.0534707	11.74539597	11.33302995
ENSG00000109819	PPARGC1A	2.644938647	0.391746117	-0.180950602	NA	2.463988045	2	2	2	3.515101962	3.2184422	2.3070652	2.277179783
ENSG00000161011	SQSTM1	3.412778277	1.13E-09	-0.94917777	0.132407854	2.463600507	12.02014453	11.73423878	10.54349105	14.94887343	14.9920283	10.5812339	10.64244805
ENSG00000102886	GDPD3	0.173692358	0.942525992	2.289528528	0.259652636	2.463220886	2	4.11352228	2	3.663521627	2.68924543	5.096960836	4.456980017
ENSG00000135763	URB2	0.703467952	0.564402109	1.759447454	0.109209308	2.462915406	3.327711234	6.728495673	6.073378139	6.744602355	6.48112613	7.701254831	7.58443608
ENSG00000138035	PNPT1	2.902007495	8.64E-17	-0.441070221	0.284420805	2.460937274	7.27616306	7.60566235	7.342949392	10.29082969	10.3023742	6.805896464	7.162846889
ENSG00000093000	NUP50	1.316692809	0.007453224	1.144175514	0.021448254	2.460868323	7.159102577	7.819099893	8.296287027	9.147810675	9.11260313	8.878514511	9.035479014
ENSG00000114021	NIT2	1.525891421	0.012730716	0.934756455	0.143321997	2.460647876	6.257048221	5.946638857	7.285536013	8.049698831	8.11238697	7.438128798	7.561323797
ENSG00000124198	ARFGEF2	1.242417649	0.35327495	1.217027963	0.35551459	2.459445612	5.330594134	9.558016677	8.978369997	10.02471865	9.96317668	10.0116652	9.92498893
ENSG00000096401	CDC5L	1.52867965	0.341899875	0.930119219	0.573981168	2.458798869	9.081016759	7.874700259	3.369317972	9.571568433	9.52609769	8.98885284	8.91909319
ENSG00000186866	POFUT2	1.010762326	0.00854774	1.447639954	8.09E-05	2.458402279	8.816894989	9.443177293	8.904018013	10.08355087	10.1041739	10.65111557	10.39640069
ENSG00000131558	EXOC4	0.706063269	0.05155675	1.752315556	7.77E-08	2.458378824	8.138771685	8.276714465	8.266922072	8.939126212	8.91810182	9.737316722	10.16833188
ENSG00000108784	NAGLU	1.314365332	0.428693153	1.143519785	0.487108958	2.457885117	3.327711234	8.993716073	8.825624954	9.645581273	9.64857633	9.301164706	9.633950463
ENSG00000135334	AKIRIN2	1.194524485	0.108276603	1.263145911	0.082516727	2.457670396	8.517314938	7.819099893	6.629488387	8.952415387	9.10299435	9.22151698	8.962375727
ENSG00000158445	KCNB1	-2.312396552	0.561910399	4.769951586	0.133361322	2.457555034	2	2	2	2	2.14022021	3.679182183	5.66592325
ENSG00000058272	PPP1R12A	0.898698645	0.131061576	1.557896051	0.004816017	2.456594696	8.696033109	9.648522413	8.561105505	10.0132353	9.89180734	10.82632022	10.35742448
ENSG00000130164	LDLR	1.282150194	0.372282912	1.1719926	0.410543204	2.454142795	6.138360429	10.54135596	10.11951029	11.07012316	11.0867303	10.17888295	11.47591991
ENSG00000135002	RFK	1.027422038	0.535148173	1.426650042	0.366590687	2.45407208	7.33131396	7.099070191	2	7.753776273	7.55253065	8.111466145	7.98359008
ENSG00000188283	ZNF383	0.926443987	0.713873535	1.526777102	0.52282666	2.453221088	2	6.728495673	2	6.197166296	6.04571775	7.081831041	6.165581817
ENSG00000142892	PIGK	1.233587955	0.498655693	1.218003713	0.495887113	2.451591668	8.313285031	7.847167909	2	8.799033614	8.68180105	8.690166757	8.761276323
ENSG00000171680	PLEKHG5	-0.128643718	0.943594867	2.578437275	0.084015928	2.449793556	8.19931886	5.457120953	3.369317972	6.772115916	6.68951263	9.273627415	9.500920284
ENSG00000169855	ROBO1	2.872921056	2.92E-05	-0.424320531	0.609295543	2.448600525	7.904282243	9.648522413	9.757248847	12.16972358	12.1875587	8.839776604	8.943133617
ENSG00000021776	AQR	1.448220104	0.301217705	0.999613183	0.482248188	2.447833288	7.159102577	8.57407404	4.058998049	8.951212326	8.90710092	8.398397867	8.566746948
ENSG00000100368	CSF2RB	0.00363709	0.999290064	2.443872465	NA	2.447509555	2	2	2	2	2.59489879	3.648325314	2.70970617
ENSG00000112033	PPARD	0.83804147	0.296072769	1.605577185	0.029202998	2.443618655	8.107517749	7.538394143	9.344432377	9.32080297	9.39662469	10.00521062	10.23212406
ENSG00000141314	RHBDL3	2.083662902	0.546147534	0.359327793	NA	2.442990695	2	2	2	3.227757282	2.86123014	2.526562374	2.277179783
ENSG00000133422	MORC2	1.358122524	0.208280231	1.084587963	0.316802663	2.442710488	5.866944937	6.602424674	8.561105505	8.754208387	8.85462427	8.704299335	8.341838298
ENSG00000088726	TMEM40	-2.312259723	0.531143362	4.754065177	0.096882542	2.441805454	2	2	2	2	2.14022021	4.667592615	5.230851202
ENSG00000111058	ACSS3	0.26977722	0.8692818	2.170780244	0.10548229	2.440557463	6.138360429	4.442710129	2	5.289065324	5.18011485	6.940933331	7.094725293
ENSG00000122642	FKBP9	1.413531148	8.04E-09	1.023387983	4.33E-05	2.436919131	10.22148166	10.03760521	10.36791565	11.58111582	11.6638355	11.16204184	11.30160663
ENSG00000079805	DNM2	0.790971047	0.313530525	1.642070046	0.022211412	2.433041093	8.228665467	10.10889278	9.778213818	10.34221503	10.3795712	11.25306166	11.16592866
ENSG00000177731	FLII	1.180609335	0.105550682	1.250859183	0.079732946	2.431468518	9.992581382	9.672251278	8.266922072	10.60726063	10.7061966	10.45346786	10.95798087
ENSG00000024862	CCDC28A	1.858680979	0.171070032	0.570673943	0.701499184	2.429354922	3.327711234	4.710564154	7.029880454	7.477814553	7.64371756	6.439833081	6.185873005
ENSG00000196576	PLXNB2	0.712798147	0.101674945	1.715830792	1.15E-05	2.428628939	11.0444064	10.44362967	10.23774519	11.29920208	11.3564391	12.37511444	12.28344877
ENSG00000100300	TSPO	0.99977257	0.143386081	1.427795461	0.027684956	2.42756803	10.36714848	8.762721501	9.491321578	10.66417187	10.6998361	11.12688615	11.09136013
ENSG00000120800	UTP20	1.427459356	0.019135943	0.998974479	0.110655463	2.426433834	6.257048221	7.274681896	6.073378139	8.132699624	7.97164382	7.490172831	7.763868281
ENSG00000102871	TRADD	1.363113742	0.466535804	1.062343679	0.570544354	2.425457421	8.257427002	6.228547981	2	8.254744042	8.41641145	7.851893762	8.208518621
ENSG00000069974	RAB27A	1.452270037	0.001624359	0.967903223	0.042824635	2.42017326	6.653098118	6.04685525	6.43197674	7.867833045	7.70710533	7.426809156	7.194904178
ENSG00000128594	LRRC4	1.25288781	0.610659792	1.166837603	0.629414332	2.419725413	3.327711234	2	2	3.289987342	2.94009902	2.937246078	3.209956562
ENSG00000102904	TSNAXIP1	1.638659485	0.366658864	0.780733676	0.682314785	2.419393161	2	3.076665209	3.369317972	3.921164147	3.94787299	3.172301926	3.518055793
ENSG00000175274	TP53I11	-1.835184867	0.074921525	4.252430441	4.67E-06	2.417245574	8.910412251	8.862353853	5.930865648	6.567497652	6.63721363	12.67330171	12.58675327
ENSG00000188215	DCUN1D3	0.926770603	0.757909059	1.490307571	0.603099089	2.417078174	8.716893051	2	2	8.049698831	8.08010078	8.693949001	8.545311752
ENSG00000164743	C8orf48	1.380372518	0.489368203	1.036698521	0.605522215	2.417071039	2	4.93638933	2	4.825935136	4.87352701	4.82764224	4.228274364
ENSG00000100473	COCH	-0.306986923	0.937842924	2.72400912	NA	2.417022197	2	2	2	2.3892346	2.14022021	3.796405078	2.918126211
ENSG00000066455	GOLGA5	1.179926175	2.16E-05	1.235685971	6.95E-06	2.415612147	8.609439662	8.655694736	8.296287027	9.718105191	9.69337522	9.779087725	9.743246098
ENSG00000136560	TANK	1.517132713	0.422347566	0.897363835	0.644306757	2.414496549	8.87372952	7.638155693	2	9.310519243	9.30647181	8.591223132	8.789175794
ENSG00000106211	HSPB1	-0.026639446	0.970008162	2.439734766	4.16E-06	2.41309532	11.59645304	10.59199661	10.38167985	10.91524435	10.9496004	13.44313619	13.34825857
ENSG00000144824	PHLDB2	0.47825396	0.712692238	1.934420458	0.085387145	2.412674417	9.097018025	9.267380431	5.772716771	9.1138055	9.17099461	10.64977738	10.53252493
ENSG00000118873	RAB3GAP2	1.387714128	0.276359671	1.024727998	0.427095076	2.412442126	5.095959458	9.079034257	8.35328246	9.638124262	9.58403885	9.402878503	9.079977539
ENSG00000143147	GPR161	1.090941266	0.39383571	1.32134025	0.282899381	2.412281516	6.138360429	8.862353853	5.930865648	8.705093576	8.73806474	9.088477995	8.796891925
ENSG00000140044	JDP2	1.221298346	0.199255138	1.190278528	0.203829413	2.411576874	6.009025997	7.638155693	7.646393253	8.486665614	8.46556258	9.035431928	7.429968874
ENSG00000178397	FAM220A	0.299683633	0.844391096	2.110543088	0.095340194	2.410226721	3.327711234	6.389896633	7.502634626	6.722207358	6.88158913	8.843186716	8.251669878
ENSG00000111300	NAA25	1.204810003	0.45539264	1.204942268	0.445080176	2.409752271	3.327711234	6.899080544	8.608698767	8.576794289	8.66157866	8.710847458	8.522888769
ENSG00000044115	CTNNA1	0.749868986	0.154731945	1.656530369	0.000499008	2.406399355	11.33491623	11.46212086	10.44204362	11.90243375	11.8880216	12.63449703	12.9506245
ENSG00000109089	CDR2L	1.224139693	0.009891083	1.179284116	0.012796144	2.403423809	8.16936288	8.903033433	8.143093325	9.591782342	9.75056618	9.830891212	9.393423672
ENSG00000164978	NUDT2	0.945497291	0.38669733	1.45779412	0.153789199	2.403291411	6.257048221	7.099070191	4.058998049	7.226553841	7.15805139	7.605105115	7.776188509
ENSG00000151292	CSNK1G3	1.422694697	0.182333448	0.978956788	0.367118564	2.401651485	7.096806373	8.376385542	5.39244166	8.837307593	8.81332035	8.600824249	8.133891722
ENSG00000107443	CCNJ	1.691681187	0.056014362	0.709933359	0.456432004	2.401614545	4.467311984	6.78762695	5.59507254	7.539954763	7.60187978	6.639356243	6.597881141
ENSG00000131759	RARA	1.090017762	0.170346386	1.311535018	0.086940568	2.40155278	8.042902253	7.874700259	6.073378139	8.633396609	8.64401667	8.732155826	8.974090715
ENSG00000123975	CKS2	2.127330793	0.14155575	0.272946428	0.871954133	2.400277221	6.89194353	5.595927442	2	7.883054517	7.87959733	6.157074092	6.018717758

ENSG00000152193	RNF219	1.909097033	0.188525669	0.48942734	0.761935984	2.398524373	5.70932728	6.78762695	2	7.66556918	7.6318872	6.462183138	6.06327132
ENSG00000164970	FAM219A	1.184712402	0.079842495	1.212456576	0.068184653	2.397168978	7.940205782	7.874700259	9.25752426	9.706030497	9.65077635	9.673139654	9.738674821
ENSG00000182606	TRAK1	-0.465060508	0.782975197	2.86181977	0.042053226	2.396759262	9.371511179	8.469613413	4.058998049	7.975897842	7.96458672	10.9714739	11.52776307
ENSG00000164975	SNAPC3	0.728129768	0.060346208	1.668412708	1.96E-06	2.396542476	8.19931886	8.487557258	8.380957451	9.072354885	9.09762836	10.24756422	9.750075969
ENSG00000164209	SLC25A46	1.14513461	0.324734879	1.249755038	0.269883074	2.394889648	8.257427002	8.505280662	5.156626401	9.004380786	9.02146427	9.198737176	9.029877404
ENSG00000180509	KCNE1	2.153028853	0.385151567	0.24144371	0.932122597	2.394472564	2	3.076665209	2	3.566290607	3.7864959	2.937246078	2.326754077
ENSG00000160326	SLC2A6	1.640229233	0.232267301	0.752867475	0.605340888	2.393096708	5.70932728	8.732943944	5.39244166	9.002058889	9.12426093	7.742927022	8.524881346
ENSG00000152475	ZNF837	0.364376059	0.865682896	2.026770574	0.265638215	2.391146633	2	4.93638933	2	4.105493497	3.94787299	5.618636935	5.20457242
ENSG00000233608	TWIST2	3.274516134	2.43E-06	-0.884377645	0.265797026	2.390138489	6.89194353	6.389896633	5.39244166	9.535899247	9.59016561	4.940196634	5.945233978
ENSG00000185219	ZNF445	0.591274265	0.440185202	1.797454004	0.007204583	2.388728269	6.653098118	8.147241016	7.098165388	8.006336225	8.06258142	9.484578004	8.916565785
ENSG00000169946	ZFPM2	1.28501208	0.55785681	1.10331627	0.614923831	2.38832835	2	8.191702463	4.058998049	7.980622673	7.95274764	7.761068522	7.814703611
ENSG00000197122	SRC	1.041587689	0.497513447	1.346217386	0.359814853	2.387805075	4.00722531	9.04308532	8.042706615	9.070139931	9.14105132	9.470314181	9.344245845
ENSG00000108443	RPS6KB1	1.681557781	0.200555174	0.706227225	0.614975927	2.387785006	8.042902253	8.276714465	4.058998049	9.333786218	9.23788597	8.291995768	8.347853908
ENSG00000114796	KLHL24	1.318996074	0.219370406	1.068237413	0.321488956	2.387233487	6.009025997	7.819099893	8.884815469	9.293531584	9.29432703	9.45200461	8.475596448
ENSG00000163564	PYHIN1	4.583262978	0.043170473	-2.196422407	0.407814557	2.386840572	2	2	2	4.670864177	4.97065356	2.094598369	2.059860178
ENSG00000129128	SPCS3	0.764207024	0.038349318	1.622216774	1.58E-06	2.386423798	9.544849385	9.923599762	9.31604076	10.44322807	10.3190701	11.34658404	11.12173937
ENSG00000070495	JMJD6	1.325205189	0.203871564	1.060195126	0.311050695	2.385400315	8.16936288	7.315436877	5.156626401	8.575233246	8.6932307	8.226262694	8.506848296
ENSG00000171444	MCC	0.339923643	0.728428075	2.044136516	0.011903882	2.384060159	4.467311984	6.666836837	5.59507254	6.24507773	6.14061114	7.857806412	7.826599033
ENSG00000065150	IPO5	0.724179564	0.397020048	1.658542585	0.031841861	2.382722148	10.16187685	10.69597594	8.677268317	10.72878599	10.8468444	11.41585194	11.9741374
ENSG00000154451	GBP5	5.370405629	2.29E-05	-2.987965947	0.030448685	2.382439682	5.532353208	3.076665209	4.058998049	9.678668908	9.62713473	3.059555873	2.172608095
ENSG00000160957	RECQL4	0.749063758	0.676328124	1.632437128	0.318708894	2.381500886	3.327711234	5.946638857	2	5.334065616	5.39133044	6.192262702	6.168983563
ENSG00000051523	CYBA	1.986448028	0.003480353	0.394065173	0.620474069	2.380513202	9.358295023	7.538394143	8.763901704	10.66893665	10.739998	9.138949083	9.09971864
ENSG00000124782	RREB1	0.966831602	0.1808519	1.413589647	0.038255118	2.380421249	7.82963488	8.376385542	6.629488387	8.692189072	8.79221631	9.042123971	9.317290185
ENSG00000005007	UPF1	0.973475844	0.001970076	1.406735544	3.14E-06	2.380211388	9.081016759	8.848535039	9.196533591	9.971714969	10.0529704	10.59634277	10.27517721
ENSG00000132481	TRIM47	0.319277679	0.880215531	2.060753695	0.247087402	2.380031374	8.285626337	6.04685525	2	7.318995108	7.28433928	8.842760893	9.173126343
ENSG00000075420	FNDC3B	1.451216499	0.011919917	0.9281119	0.122378239	2.379328399	9.533135543	10.93049815	10.27509734	11.82589752	11.7866286	11.23597623	11.33046897
ENSG00000152076	CCDC74B	2.68559618	0.028202356	-0.308581604	0.835379021	2.377014576	2	4.710564154	3.369317972	6.096288482	6.16544343	3.429858444	3.789585079
ENSG00000183354	KIAA2026	1.358817989	0.01600303	1.01655093	0.078156821	2.37536892	8.493334308	7.572420273	7.398165132	9.214510118	9.27734059	9.123256136	8.651263518
ENSG00000198464	ZNF480	1.692626053	0.347244214	0.680927171	0.722957397	2.373553224	3.327711234	7.60566235	3.369317972	7.821180652	7.81567127	6.984820384	6.662054875
ENSG00000154447	SH3RF1	0.206462846	0.820270883	2.166665198	0.002482931	2.373128044	7.709880939	8.297207175	9.491321578	8.869506572	8.92417737	10.55742653	11.09023921
ENSG00000012174	MBTPS2	1.16763028	0.539874611	1.205479271	0.520317876	2.37310955	2	8.639735838	8.266922072	9.064587625	9.02372946	8.952213594	9.200809988
ENSG00000135899	SP110	2.952432247	0.001150989	-0.579678786	0.586432904	2.372753461	8.16936288	5.457120953	7.451345288	10.26540233	10.3604317	6.497238296	7.09829807
ENSG00000096654	ZNF184	0.868514619	0.728012667	1.502327736	0.523757284	2.370842355	2	6.535001748	2	5.997179931	5.76075522	6.887434761	5.917188835
ENSG00000110944	IL23A	1.162087386	0.591410858	1.208501607	0.566009975	2.370588993	2	3.686303414	2	3.615725078	3.45167086	3.709392871	3.407202941
ENSG00000055917	PUM2	1.39880682	0.299070637	0.971354989	0.477355787	2.370161809	5.330594134	9.523645035	9.31604076	10.32374052	10.2198241	10.03887247	9.625308966
ENSG00000132406	TMEM128	1.147845584	0.170554882	1.222171474	0.133443484	2.370017059	7.031698319	5.457120953	5.39244166	7.341208691	7.18663697	7.514972902	7.137025524
ENSG00000184787	UBE2G2	0.818184408	0.525545049	1.550901796	0.190242338	2.369086205	5.866944937	9.599860728	8.865353883	9.56056556	9.58940119	10.06849268	10.50771945
ENSG00000163637	PRICKLE2	1.561318462	0.380355326	0.807746487	0.664743846	2.369064948	2	7.979858146	8.110401118	8.998569022	9.0282492	8.289498148	8.245238611
ENSG00000282650		-1.310495096	0.691746353	3.679377362	0.166987227	2.368882266	2	2	2	2.141737246	2.14022021	4.240999983	3.924507493
ENSG00000077782	FGFR1	1.328875491	0.143140149	1.039811088	0.256088094	2.368686579	7.790810999	10.04973379	10.06936278	10.92783718	10.956731	10.43593029	10.84329825
ENSG00000126878	AIF1L	-2.311950223	0.069585809	4.680592777	3.31E-05	2.368642554	5.866944937	5.131603625	3.369317972	3.881293667	2.49394783	9.408931763	9.818008038
ENSG00000164010	ERMAP	1.721581315	0.239652463	0.646961026	0.682571547	2.368542341	6.468621203	6.728495673	2	7.59952861	7.83127384	6.928144991	6.368575325
ENSG00000167771	RCOR2	0.66778214	0.61090653	1.700685814	0.144465782	2.368467955	2	4.442710129	4.058998049	4.415698455	4.3456612	5.376552034	5.14363432
ENSG00000172086	KRCC1	0.845979953	0.127962856	1.521592188	0.002976633	2.367572141	7.096806373	7.669933291	6.882775709	8.115618198	8.08227582	9.049892323	8.413855585
ENSG00000170917	NUDT6	0.558337223	0.785383233	1.804546931	0.316598154	2.362884154	2	4.11352228	2	3.566290607	3.45167086	4.478802948	4.376876162
ENSG00000250741	NT5C1B-RDH	7.553581144	0.000114143	-5.191147348	0.053140582	2.362433795	2	2	2	7.608714138	7.61094772	2	2
ENSG00000133318	RTN3	0.866872767	0.138486261	1.495318548	0.005942742	2.362191314	10.22148166	8.981105197	9.358421373	10.48373114	10.4724652	11.09067303	11.11943354
ENSG00000198331	HYLS1	0.860542047	0.588479045	1.498873837	0.311050695	2.359415883	2	5.946638857	4.058998049	5.540148523	5.63601114	6.126613714	6.238627697
ENSG00000080823	MOK	0.131443194	0.929816715	2.22577788	0.063181453	2.357221074	3.327711234	6.666836837	7.285536013	6.449761039	6.74548212	8.850830124	8.424568162
ENSG00000121988	ZRANB3	0.742643237	0.422586005	1.614501665	0.051229661	2.357144902	4.00722531	4.11352228	4.874587708	4.965943888	5.00774437	5.801542492	5.745545708
ENSG00000096070	BRPF3	1.31009197	0.519874318	1.046628698	0.607213739	2.356720668	2	9.090820894	9.413059136	9.965173975	9.99998257	9.877784692	9.543476778
ENSG00000180346	TIGD2	1.12886893	0.625679022	1.227367511	0.587145381	2.356236441	2	6.04685525	2	5.764940652	5.55061739	6.049001768	5.379200993
ENSG00000063180	CA11	-0.72731905	0.719117881	3.082355302	0.073412065	2.355036252	2	5.303523747	2	3.709785322	3.33976443	6.736076089	6.841176624
ENSG00000103266	STUB1	1.268047467	0.215393228	1.086263786	0.287317112	2.354311252	6.468621203	8.862353853	9.330306409	9.91915277	9.88247671	9.881313582	9.538225362
ENSG00000165675	ENOX2	1.042916535	0.695713377	1.309583814	0.61151596	2.352500349	2	7.503546079	2	6.927012081	7.04662306	7.220710071	7.272853598
ENSG00000198646	NCOA6	0.924106431	0.236885537	1.427654858	0.051981925	2.351761288	6.89194353	8.395519306	8.742726175	9.069031178	9.16587611	9.840733868	9.35623215
ENSG00000146054	TRIM7	0.698504224	0.495803512	1.652796091	0.070347683	2.351300316	4.467311984	3.686303414	4.874587708	5.003571641	4.93258395	6.089765781	5.513708555
ENSG00000104774	MAN2B1	1.605804676	0.127670293	0.745021843	0.504623054	2.350826519	6.257048221	9.267380431	7.858115942	9.844656195	9.88932507	8.899548956	9.115231141
ENSG00000165813	CCDC186	0.983612907	0.583878202	1.367042616	0.425523578	2.350655523	2	7.761270542	7.285536013	7.975897842	7.89946959	8.660513226	7.862758575
ENSG00000124226	RNF114	1.671921042	0.000106412	0.675914562	0.151862412	2.347835604	9.533135543	8.57407404	9.212024683	10.79901337	10.8388264	9.887726478	9.76195099
ENSG00000103047	TANGO6	0.622331497	0.760903016	1.724689923	0.351097101	2.34702142	3.327711234	3.076665209	7.646393253	6.766655021	6.77805036	7.657374802	8.013226939
ENSG00000112981	NME5	-0.310501882	0.932714208	2.65656894	NA	2.346067059	2	2	2	2.270786764	2.2680111	3.355392153	3.407202941
ENSG00000185753	CXorf38	0.836915159	0.306087183	1.508940377	0.045614369	2.345855536	6.653098118	6.666836837	8.266922072	8.205140657	8.23866319	8.891336668	8.882264318
ENSG00000205726	ITSN1	0.761685713	0.374329883	1.583722188	0.042742908	2.345407901	6.653098118	8.276714465	8.585098385	8.855427477	8.74905888	9.204383073	9.941741079
ENSG00000179134	SAMD4B	0.864157677	0.16153158	1.481122249	0.00978288	2.345279925	8.696033109	8.929529568	9.963514551	10.14218788	10.1868502	10.76381664	10.79581778
ENSG00000020426	MNAT1	1.425921215	0.154641078	0.919192852	0.370178383	2.345114067	7.435667355	6.602424674	4.523675391	8.083034979	7.91663606	7.489084836	7.520630205
ENSG00000143772	ITPKB	0.198806608	0.905370978	2.146069809	0.112945603	2.344876416	6.468621203	6.602424674	2	6.205262847	6.1572134	8.167627778	7.962181894
ENSG00000104164	BLOC1S6	1.003150477	0.30391278	1.340056142	0.14848394	2.343206619	7.667647223	9.461450882	7.225742779	9.508351943	9.41830936	9.683639816	9.906257642
ENSG00000112701	SENP6	1.40914275	0.354199679	0.933802937	0.546828054	2.342945687	4.00722531	8.276714465	9.149036566	9.6094308	9.62191107	9.415818816	8.806752407
ENSG00000084676	NCOA1	1.003007619	0.466535804	1.339752029	0.30559948	2.342759649	6.73718411	8.762721501	4.874587708	8.512996295	8.6202631	8.897500263	8.904882354
ENSG00000113722	CDX1	-0.307017014	0.936463188	2.6489926	NA	2.341975586	2	2	2	2.3892346	2.14022021	3.393105702	3.36036153
ENSG00000197579	TOPORS	1.027465915	0.555006373	1.31321203	0.434435201	2.340677945	3.327711234	9.055168103	6.882775709	8.765203682	8.8354102	9.092781861	9.075453001
ENSG00000204406	MBD5	1.210827225	0.410137517	1.128172472	0.436457359	2.338999697	4.00722531	6.728495673	8.561105505	8.508095759	8.62474679	8.500731308	8.47078082
ENSG00000165424	ZCCHC24	0.652294456	0.197034134	1.686160209	0.000175366	2.338454665	8.564112515	9.574900212	9.358421373	9.870978946	9.88683852	10.8308362	10.98440704
ENSG00000150403	TMCO3	0.309634389	0.514390461	2.025065989	1.42E-07	2.334700378	9.219014269	8.540087354	8.536706852	9.09649754	9.11685323	10.69069483	10.92918213
ENSG00000136144	RCBTB1	1.321192923	0.531161556	1.01222991	0.635038067	2.333422832	4.467311984	8.213429689	2	8.063125866	8.03365075	7.79843893	7.688837505
ENSG00000198682	PAPSS2	0.612836879	0.040383064	1.720522629	1.37E-10	2.333359508	8.963744667	9.13702746	9.242516611	9.706030497	9.74988221	10.69600884	10.95662832
ENSG00000078808	SDF4	1.23024544	0.054876729	1.102572067	0.085204406	2.332817507	11.31453223	10.22039562	9.888372618	11.81700892	11.8573927	11.68592437	11.73331274
ENSG00000137460	FHDC1	0.718084867	0.649032625	1.614257551	0.261053473	2.332342419	4.00722531	2	5.39244166	5.040242897	4.79082474	5.711121288	5.740981724
ENSG00000143195	ILDR2	0.66509634	0.838427142	1.666535507	0.574861708	2.331631846	3.327711234	4.11352228	2	4.647285135	2	4.764298067	4.522269241
ENSG00000143924	EML4	0.656557467	0.596166767	1.67470297	0.131976284	2.331260437	6.73718411	9.488434476	7.029880454	9.00785664	8.9350493	9.932236229	10.034406
ENSG00000108100	CCNY	0.709297824	0.279279457	1.621069622	0.006021251	2.330367446	8.042902253	7.761270542	9.06622548	9.104095335	9.09762836	9.843290512	10.15531359
ENSG00000163568	AIM2	7.520834048	0.000141304	-5.19114714	0.054407162	2.329686908	2	2	2	7.59952861	7.55566492	2	2
ENSG00000144815	NXPE3	0.837345016	0.495345811	1.491241596	0.189415864	2.328586612	4.815609471	7.790474954	8.325066185	8.379838154	8.40949864	9.139989235	8.937659629
ENSG00000155330	C16orf87	1.26516708	0.000854905	1.063171067	0.005585099	2.328338147	7.218819781	6.899080544	6.80318718	8.252792309	8.18704757	8.10721774	7.929461924
ENSG00000135476	ESPL1	1.299826981	0.443997681	1.028485828	0.543853497	2.328312809	2	5.838937808	3.369317972	5.900763176	5.64780787	5.498346994	5.561082532
ENSG00000069482	GAL	0.391192469	0.88199664	1.936481853	0.389444033	2.327674322	3.327711234	3.686303414	2	3.754611435	3.01487888	5.371837532	2.632958241
ENSG00000162298	SYVN1	1.275279045	0.199759863	1.052382543	0.290417779	2.327661588	6.138360429	8.702538795	8.805342542	9.508351943	9.58863636	9.284015592	9.369598769
ENSG00000005100	DHX33	0.836421048	0.201194682	1.490981899	0.013866293	2.327402947	6.89194353	6.389896633	7.777116083	7.980622673	7.89452713	8.701014095	8.456932644
ENSG00000176018	LYSMD3	0.911569847	0.548376437	1.41548108	0.323969847	2.327050927	3.327711234	7.901717006	7.818184403	8.289431124	8.11238697	8.997677834	8.328971229
ENSG00000133392	MYH11	4.247663782	0.003295139	-1.921559035	0.243248502	2.326104747	2	3.076665209	3.369317972	6.197166296	6.18176397	2.384001718	2.225841198
ENSG00000185271	KLHL33	-2.312308415	0.540667714	4.636420665	0.119124184	2.32411225	2	2	2	2	2.14022021	4.434275199	5.211187127
ENSG00000116191	RALGPS2	0.724352198	0.711973842	1.599143011	0.373931636	2.32349521	8.468948322	6.78762695	2	7.992367495	8.00183183	8.994231002	8.71548362
ENSG00000213047	DENND1B	1.430116838	0.35327495	0.893289162	0.571694567	2.323405999	2	6.844429755	5.772716771	7.344877929	7.1204461	6.882471426	6.530719415
ENSG00000132361	CLUH	1.020675648	0.576726175	1.300492786	0.460189911	2.321168434	3.327711234	6.666836837	9.301632639	8.94033939	8.97539718	9.271414214	9.199147229
ENSG00000164305	CASP3	0.207215705	0.806463377	2.113621463	0.001567325	2.320837168	9.33149386	8.356994601	7.552162999	8.756965077	8.84055903	10.73324321	10.65826756
ENSG00000218823	PAPOLB	1.499555752	0.435748344	0.821187643	0.679613476	2.320743396	2	4.11352228	2	4.070463721	4.2865223	3.796405078	3.560117069
ENSG00000155511	GRIA1	-1.06138023	0.703860951	3.381313373	0.167205719	2.319933143	5.70932728	2	2	4.205751501	2.2680111	6.433059984	7.986484919
ENSG00000157992	KRTCAP3	1.480585034	0.547023674	0.837157974	0.739591934	2.317743008	2	3.076665209	2	3.023133209	3.45167086	3.035909414	2.782576693
ENSG00000070367	EXOC5	1.143132015	0.462285707	1.173995587	0.438543047	2.317127602	4.00722531	9.079034257	8.825624954	9.592554172	9.46739696	9.645573761	9.473326665
ENSG00000164038	SLC9B2	2.086456631	0.104768499	0.22539473	0.883235347	2.311851361	8.564112515	6.140560021	4.874587708	9.373846284	9.37105521	7.64960537	7.408544534
ENSG00000105176	URI1	0.852687418	0.569331332	1.457866831	0.298467142	2.310554249	4.00722531	8.395519306	8.677268317	8.83470036	8.83024294	9.461743163	9.404288469
ENSG00000080345	RIF1	1.255650283	0.015155122	1.053108888	0.043968507	2.308759171	8.737455672	7.761270542	8.721235202	9.695291778	9.72712735	9.704647975	9.284342586
ENSG00000134987	WDR36	1.361799511	0.150716856	0.946100291	0.327012123	2.307899802	5.866944937	8.147241016	6.534109959	8.578353644	8.48213028	8.293866149	7.923432281
ENSG00000187013	C17orf82	1.252890733	0.61318036	1.05465232	0.668986346	2.307543053	2	2	3.369317972	3.289987342	2.94009902	3.172301926	2.851942824
ENSG00000076716	GPC4	0.391889328	0.844353006	1.915471533	0.264665124	2.307360861	8.19931886	6.666836837	2	7.559034656	7.30683517	8.740866084	9.115231141
ENSG00000120889	TNFRSF10B	1.002288575	1.28E-07	1.303258547	2.39E-12	2.305547122	10.46903009	10.44821838	10.57378414	11.47335809	11.5206165	11.79510361	11.80044455

ENSG00000108799	EZH1	0.868406056	0.641177228	1.436744769	0.413358122	2.305150825	8.257427002	7.431218269	2	8.190940526	8.17486938	8.961264871	8.48994763
ENSG00000169246	NPIPB3	5.44931766	6.69E-30	-3.145845775	7.01E-10	2.303471885	6.366709671	6.728495673	5.39244166	11.67428979	11.6634724	3.663836244	3.600986722
ENSG00000089050	RBBP9	1.417786575	0.13915257	0.884969515	0.369236619	2.30275609	7.384433967	7.503546079	4.874587708	8.392303091	8.35836495	7.787860081	7.910280955
ENSG00000141756	FKBP10	0.737654234	0.251050327	1.563476168	0.007494256	2.301130402	12.26464645	11.09754095	12.48269311	12.77192248	12.826278	13.73492812	13.50561406
ENSG00000150394	CDH8	-0.737231445	0.688378388	3.03786246	0.053922041	2.300631014	2	4.710564154	7.098165388	5.022023783	5.21224859	9.079831553	8.274745794
ENSG00000072501	SMC1A	0.627710597	0.223475046	1.669022462	0.000259929	2.296733059	9.031919741	9.00621767	8.143093325	9.345738359	9.46906021	10.24160068	10.62444126
ENSG00000011566	MAP4K3	2.563194483	3.24E-06	-0.267335605	0.696157407	2.295858878	6.563806279	7.874700259	7.858115942	10.08190282	10.0993493	7.440382108	7.126566171
ENSG00000171867	PRNP	2.066344773	0.016788447	0.228749321	0.827299212	2.295094094	11.50346787	9.981728319	9.242516611	12.63561678	12.6047477	10.99024375	10.5456694
ENSG00000214413	BBIP1	1.360718295	0.36589956	0.933754478	0.541713981	2.294472773	6.89194353	6.389896633	2	7.344877929	7.49164798	6.940933331	7.065820006
ENSG00000111186	WNT5B	0.113639527	0.888172933	2.179731972	0.000343839	2.293371499	7.709880939	8.981105197	9.31604076	8.918344554	8.9398551	11.04947379	10.92000025
ENSG00000155858	LSM11	0.268124543	0.701852817	2.024798334	0.000313211	2.292922877	6.468621203	6.602424674	6.80318718	7.017154451	6.74548212	9.000353022	8.071643455
ENSG00000103479	RBL2	1.215311903	0.329061986	1.074178547	0.384709619	2.28949045	5.330594134	9.00621767	8.845626171	9.562142546	9.6544356	9.518299895	9.417219134
ENSG00000115520	COQ10B	1.458023393	0.157714101	0.830792877	0.437993223	2.288816269	9.081016759	8.414402626	6.073378139	9.835556805	9.66898051	9.149317009	9.112583673
ENSG00000162777	DENND2D	0.243561862	0.865682896	2.045228439	0.077151749	2.288790301	4.467311984	4.11352228	2	4.034562184	4.0930011	5.853194273	5.282014932
ENSG00000130529	TRPM4	0.228462299	0.849223293	2.059994301	0.034146284	2.288456601	5.70932728	7.60566235	8.677268317	7.982979299	8.0087095	9.795903284	9.826110653
ENSG00000074695	LMAN1	1.084201768	0.03803836	1.202088253	0.019063806	2.286290021	10.63713535	10.51097332	9.516493218	11.39677132	11.3707459	11.46655078	11.53569017
ENSG00000141437	SLC25A52	4.582529336	0.174292193	-2.297129444	0.537958482	2.285399893	2	2	2	4.54891529	5.06165168	2.139668783	2
ENSG00000141569	TRIM65	0.540439672	0.55375014	1.741416429	0.029917935	2.281856101	7.790810999	6.899080544	5.59507254	7.539954763	7.55253065	8.929133243	8.498084662
ENSG00000029364	SLC39A9	0.875208041	0.035558155	1.406517692	0.000321467	2.281725733	8.892187472	9.405920729	8.608698767	9.912986572	9.85538952	10.3722972	10.45435372
ENSG00000184967	NOC4L	1.343871037	0.478334777	0.935078014	0.626461244	2.278949051	4.00722531	7.051611315	2	6.931899482	7.00136498	6.346800618	6.769038269
ENSG00000163945	UVSSA	0.535893442	0.720558899	1.742581466	0.188722009	2.278474908	4.467311984	4.93638933	7.818184403	7.062428156	7.01963893	8.264281212	8.180141273
ENSG00000112159	MDN1	0.688468532	0.628451704	1.588984615	0.220081795	2.277453147	4.467311984	7.874700259	8.763901704	8.550024073	8.50338773	9.481298847	9.35623215
ENSG00000108688	CCL7	6.051420788	0.003930403	-3.774546937	0.180505068	2.276873851	2	2	2	6.172600389	6.14893615	2.048074405	2
ENSG00000171492	LRRC8D	2.138935195	0.145483541	0.13778175	0.937449278	2.276716945	3.327711234	7.35507212	8.536706852	9.651519255	9.58403885	7.596037946	7.680519528
ENSG00000245848	CEBPA	-0.688466766	0.803473818	2.964221236	0.201514215	2.27575447	2	3.686303414	2	2.695456494	2.49394783	5.646229369	3.261853426
ENSG00000169895	SYAP1	0.822875375	0.00491763	1.45137291	1.32E-07	2.274248285	8.816894989	8.834582582	8.677268317	9.607904693	9.58633944	10.07267492	10.35928683
ENSG00000152102	FAM168B	0.243885491	0.895675696	2.028763373	0.189944554	2.272648864	4.467311984	10.28850895	9.945092785	9.826399658	9.77228374	11.51966333	11.63765248
ENSG00000164494	PDSS2	0.841496862	0.321145767	1.430780489	0.069800729	2.272277351	5.330594134	7.274681896	6.073378139	7.307758725	7.26914476	7.871230734	7.859604233
ENSG00000174132	FAM174A	1.189166966	0.488975574	1.082958818	0.523941689	2.272125784	2	6.899080544	7.691296561	7.944803394	7.93600799	7.890311949	7.779530392
ENSG00000135776	ABCB10	0.982360736	0.552722287	1.28871532	0.418932978	2.271076057	2	6.389896633	7.285536013	7.307758725	7.27295842	7.644728141	7.532563185
ENSG00000185267	CDNF	1.206204884	0.588327109	1.064718773	0.629414332	2.270923657	2	3.686303414	2	3.515101962	3.60476711	3.724264246	3.15612297
ENSG00000130822	PNCK	-0.718098908	0.895265695	2.988492177	0.525662412	2.270393268	2	2	2	2.3892346	2	3.930690651	3.15612297
ENSG00000103978	TMEM87A	2.02167045	0.06340191	0.247700786	0.84812366	2.269371236	8.313285031	9.018611864	5.59507254	10.21014801	10.2227847	8.513629334	8.392188801
ENSG00000177106	EPS8L2	0.499594327	0.85245466	1.768284915	0.458817894	2.267879242	9.730679447	4.442710129	2	8.621345041	8.7394436	9.566031214	10.24002969
ENSG00000033100	CHPF2	0.958147825	0.013556653	1.308993957	0.000439228	2.267141782	9.689059027	10.27307349	10.43545992	11.11136778	11.1336271	11.40522087	11.53741843
ENSG00000108262	GIT1	0.600867924	0.765831172	1.666009922	0.361988871	2.266877846	8.468948322	8.702538795	2	8.509731121	8.70032852	9.869029072	9.428616564
ENSG00000278742		7.455553301	0.000218272	-5.191146668	0.057347766	2.264406633	2	2	2	7.447418636	7.57741621	2	2
ENSG00000156414	TDRD9	-3.753101612	0.362134662	6.017239333	0.078163215	2.264137721	2	2	2	2	2	5.119635168	6.716538017
ENSG00000120265	PCMT1	1.087765772	0.43457188	1.175115594	0.383915641	2.262881366	8.75772933	8.607278467	4.523675391	9.231457548	9.20532695	9.449489749	9.144901069
ENSG00000131263	RLIM	1.266869209	0.187284818	0.994256456	0.303223054	2.261125665	9.820154628	8.762721501	7.163363831	10.225157	10.1908874	9.950927567	9.921967897
ENSG00000007047	MARK4	1.013033425	0.368308689	1.246752992	0.250438486	2.259786418	6.009025997	8.607278467	9.212024683	9.437090644	9.46739696	9.796563052	9.563647359
ENSG00000169398	PTK2	0.783383229	0.434884889	1.474313461	0.109780104	2.257696691	7.384433967	9.616264339	9.935792933	10.12788365	10.0928914	10.60364979	10.97180235
ENSG00000258588	TRIM6-TRIM	7.448061742	0.000231352	-5.191146595	0.057776246	2.256915147	2	2	2	7.580980221	7.4280824	2	2
ENSG00000090060	PAPOLA	1.515736122	0.048471712	0.740924767	0.363979257	2.256660889	8.138771685	10.17682307	9.503962296	11.03591644	10.9808314	10.32506285	10.14066157
ENSG00000109118	PHF12	0.945266424	0.312962462	1.311387657	0.140295302	2.256654081	8.009475818	8.395519306	5.930865648	8.713632895	8.70880006	9.026460641	9.120511546
ENSG00000090238	YPEL3	0.541251753	0.707528996	1.714817322	0.178997619	2.256069075	4.00722531	7.847167909	8.110401118	7.971157485	7.98332947	9.348425302	8.89156005
ENSG00000166326	TRIM44	0.697623481	0.092286577	1.557874507	3.15E-05	2.255497987	9.646202137	10.35843478	9.839340503	10.66893665	10.6891726	11.41370331	11.65200579
ENSG00000112763	BTN2A1	1.746408691	0.00247249	0.508651381	0.435186101	2.255060072	9.689059027	8.276714465	9.31604076	10.95689981	10.9276923	9.815783218	9.592751029
ENSG00000163964	PIGX	0.654371904	0.515257886	1.600453023	0.075349452	2.254824926	7.27616306	7.393647515	4.874587708	7.454228844	7.58969985	8.448514034	8.46040717
ENSG00000007384	RHBDF1	1.300844946	0.002487197	0.953653233	0.031386333	2.254498179	8.107517749	8.747909548	8.408111533	9.727271068	9.7560263	9.092423696	9.647426237
ENSG00000178789	CD300LB	-1.303268636	0.814508396	3.556201101	0.462938093	2.252932465	2	2	2	2.270786764	2	4.778616857	2.172608095
ENSG00000183735	TBK1	1.777920034	0.001515995	0.472442827	0.459674606	2.250362861	7.750913381	6.899080544	8.236946975	9.474417988	9.47569407	8.325906648	8.020777347
ENSG00000157483	MYO1E	0.652357625	0.293988413	1.597070096	0.004002525	2.249427721	9.189468652	8.297207175	9.439621147	9.690252889	9.70188757	10.92345228	10.28029762
ENSG00000187535	IFT140	0.970824938	0.634148538	1.278218088	0.515353465	2.249043027	2	7.954278496	4.874587708	7.514115926	7.49164798	7.786974998	7.821204179
ENSG00000117118	SDHB	1.41034914	0.198081092	0.8376604	0.459893021	2.24800954	8.981092835	7.145017454	6.073378139	9.314267267	9.26207178	8.550081477	8.872386739
ENSG00000198081	ZBTB14	0.939101304	0.531266648	1.308818796	0.360920918	2.2479201	6.257048221	6.602424674	2	6.809774134	6.77805036	7.293562319	6.994755759
ENSG00000128513	POT1	1.34709315	0.112649338	0.900401855	0.299350397	2.247495005	6.96351253	6.140560021	5.156626401	7.52061915	7.61395775	7.538302335	6.564691065
ENSG00000167676	PLIN4	-0.727708903	0.844549702	2.973506641	0.332960127	2.245797738	2	2	2	2.141737246	2.2680111	3.865109708	3.236138336
ENSG00000214026	MRPL23	2.589643211	0.014438702	-0.344475398	0.787237277	2.245167813	4.00722531	4.11352228	6.43197674	7.68016596	7.81567127	4.888321757	5.072762365
ENSG00000137936	BCAR3	1.090387397	0.061614336	1.153316643	0.04392157	2.24370404	7.53297911	8.732943944	7.896971941	9.228481255	9.23983583	9.470589819	9.098827153
ENSG00000102053	ZC3H12B	-0.167771671	0.91483416	2.410374805	0.053383916	2.242603134	2	4.11352228	5.39244166	4.105493497	4.37434574	6.098353065	6.887339785
ENSG00000104324	CPQ	0.468101597	0.643120989	1.773344691	0.042824635	2.241446288	9.204317094	7.761270542	6.882775709	8.758341449	8.70032852	9.830032147	10.19985319
ENSG00000065989	PDE4A	0.43308049	0.756699054	1.80792994	0.135544734	2.24101043	8.87372952	7.928237109	4.874587708	8.395844809	8.36015869	9.658696966	9.821254547
ENSG00000163104	SMARCAD1	1.749134507	0.190391386	0.490115373	0.738292369	2.23924988	4.00722531	8.276714465	6.80318718	8.977450658	8.8597051	7.774526492	7.572926221
ENSG00000092439	TRPM7	1.643741281	0.067435031	0.593530551	0.544635534	2.237271832	7.579279349	7.638155693	9.589475645	10.24198055	10.2089163	9.257743834	9.098827153
ENSG00000152926	ZNF117	1.561713645	0.022695801	0.67543045	0.361214879	2.237144095	4.815609471	6.04685525	6.43197674	7.450827758	7.36873214	6.7543034	6.317379004
ENSG00000065000	AP3D1	0.997358352	0.019549332	1.239760841	0.002731113	2.237119192	9.423197387	10.27823701	9.757248847	10.83076085	10.8903195	11.03789512	11.16486422
ENSG00000151743	AMN1	1.538281276	0.485453797	0.696023767	0.763713936	2.234305043	2	5.946638857	2	5.94010643	5.9536761	5.085488617	5.263040529
ENSG00000117394	SLC2A1	1.154690535	0.100527553	1.078057185	0.121709811	2.23274772	10.88304938	10.30378101	9.25752426	11.44457809	11.4469936	11.03463637	11.64071835
ENSG00000154124	OTULIN	0.874070998	0.345449366	1.358288077	0.118414814	2.232359075	6.468621203	7.274681896	8.677268317	8.625876185	8.61876544	9.267294952	8.916059773
ENSG00000174236	REP15	3.009791982	0.305279886	-0.77786129	0.813732645	2.231930693	2	2	2	3.406930997	3.7864959	2.225793514	2.172608095
ENSG00000104936	DMPK	-0.19762642	0.710155984	2.429553625	3.63E-09	2.231927205	9.204317094	8.337339476	9.083172337	8.737556707	8.70315789	11.20690519	11.45473662
ENSG00000187210	GCNT1	1.008738042	0.609550266	1.219823761	0.52282666	2.228561803	2	7.431218269	4.523675391	7.101994243	6.96881778	6.85908071	7.544398272
ENSG00000107651	SEC23IP	1.240125569	0.307710975	0.988210598	0.418322864	2.228336167	6.366709671	9.558016677	7.398165132	9.616278374	9.63159714	9.202392925	9.526339317
ENSG00000171121	KCNMB3	3.110063055	0.05126002	-0.881877375	0.62358047	2.22818568	2	4.93638933	2	6.415275989	6.48770488	3.12825509	3.287118176
ENSG00000184719	RNLS	1.261817373	0.574255931	0.962664209	0.672113452	2.224481582	2	5.946638857	2	5.627094009	5.74985018	5.323825264	5.502966356
ENSG00000120254	MTHFD1L	0.534564434	0.64508255	1.689648955	0.099251185	2.224213389	9.435834703	7.790474954	6.534109959	8.965583267	8.86981338	10.1449176	9.984600648
ENSG00000115170	ACVR1	1.690804058	0.047187606	0.53155749	0.572841244	2.222361549	7.940205782	6.464272742	8.805342542	9.658907527	9.72504082	8.624798858	8.45344973
ENSG00000174576	NPAS4	-0.723374178	0.844052269	2.945493218	0.331905931	2.22211904	2	2	2	2.270786764	2.14022021	3.632645806	3.496556178
ENSG00000068878	PSME4	1.375046689	4.40E-06	0.846783891	0.006873339	2.22183058	8.892187472	9.00621767	9.344432377	10.41296081	10.505257	9.839880645	10.02129178
ENSG00000081019	RSBN1	1.126538129	0.528648153	1.093250027	0.533161677	2.219788156	7.975256505	7.002537973	2	8.149581172	8.05374131	8.317962987	7.769481431
ENSG00000172830	SSH3	0.363983274	0.688378388	1.855305416	0.0150633	2.219288691	5.866944937	6.311476754	7.734844356	7.25803034	7.16216989	8.69913345	8.654300638
ENSG00000184557	SOCS3	1.17466905	0.361180507	1.044243214	0.4140361	2.218912264	6.89194353	8.255926468	4.523675391	8.383410567	8.38503958	8.575932171	7.842663598
ENSG00000213401	MAGEA12	-2.306780167	0.562496388	4.525257085	0.156315531	2.218476917	2	2	2	2.141737246	2	5.323825264	3.8919413
ENSG00000197586	ENTPD6	0.882987688	0.638882507	1.33525392	0.455061132	2.218241607	7.790810999	8.376385542	2	8.458152491	8.36195019	8.912592153	8.802378289
ENSG00000119326	CTNNAL1	-0.208747818	0.833633118	2.424964205	0.001792503	2.216216387	6.73718411	8.791896851	7.552162999	7.762033056	7.72948195	10.0867686	10.58423994
ENSG00000231924	PSG1	0.694332923	0.759550096	1.520729472	0.467385744	2.215062395	2	2	5.156626401	4.237680128	4.45712836	4.875056259	5.20457242
ENSG00000110079	MS4A4A	-2.312353283	0.551211363	4.527326948	0.142012462	2.214973664	2	2	2	2	2.14022021	5.185595313	4.215171317
ENSG00000143373	ZNF687	0.629963618	0.795427588	1.584287128	0.47448492	2.214250746	4.815609471	9.125613817	2	8.235106869	8.2561084	9.167453541	9.213631806
ENSG00000163995	ABLIM2	-2.312355123	0.551575085	4.526286499	0.142685271	2.213931376	2	2	2	2	2.14022021	5.19095825	4.201948172
ENSG00000176401	EID2B	1.01369802	0.623226161	1.197938258	0.547523809	2.211636278	2	4.710564154	2	4.17310023	4.4836996	4.111244182	4.784231301
ENSG00000147166	ITGB1BP2	-1.310494048	0.697008225	3.520945257	0.197561623	2.21045121	2	2	2	2.141737246	2.14022021	3.891700877	4.047883918
ENSG00000171931	FBXW10	3.272401786	0.30635441	-1.062825412	0.764659891	2.209576374	2	2	2	3.515101962	4.02226117	2.048074405	2.277179783
ENSG00000102385	DRP2	0.674714874	0.765893437	1.534509789	0.462478098	2.209224663	2	5.595927442	2	4.946755474	4.72553021	5.367107572	5.812327524
ENSG00000070269	TMEM260	1.610925986	0.391746117	0.598244476	0.765896408	2.209170461	4.467311984	7.467835385	2	7.565338955	7.73225478	6.631488899	6.70252219
ENSG00000173868	PHOSPHO1	-1.269611831	0.617831455	3.477712143	0.096104184	2.208100312	3.327711234	2	2	2.3892346	2.14022021	4.965450964	4.388595494
ENSG00000069493	CLEC2D	4.450966765	3.76E-05	-2.243056047	0.05509797	2.207910718	2	5.946638857	5.59507254	9.595637371	9.54119795	3.753554863	3.183290849
ENSG00000177463	NR2C2	1.036848313	0.326820527	1.170590951	0.25494197	2.207439263	9.544849385	7.099070191	7.502634626	9.512436154	9.48312089	9.743260791	9.508995835
ENSG00000104375	STK3	0.80380305	0.397404116	1.403441869	0.110621519	2.207244919	6.009025997	7.73146271	5.772716771	7.523859802	7.65545169	8.313667445	8.033896215
ENSG00000106397	PLOD3	1.339447667	0.477431438	0.867412905	0.651899269	2.206860572	9.975371636	10.64091981	3.369317972	11.06734964	11.1380772	10.60452918	10.65917495
ENSG00000118197	DDX59	1.140580059	0.236944877	1.06507941	0.26431896	2.20565947	4.815609471	7.274681896	6.073378139	7.555872146	7.45854177	7.566427714	7.288576158
ENSG00000111540	RAB5B	0.474040238	0.535332813	1.73137772	0.008995102	2.205417959	9.097018025	10.27307349	10.8211137	10.68386176	10.7139326	11.81499449	12.08131906
ENSG00000076067	RBMS2	0.881829005	0.012336386	1.323420019	7.80E-05	2.205249025	9.87686338	9.770792087	9.372276026	10.55119161	10.5871833	10.81692048	11.18011685
ENSG00000216588	IGSF23	-2.312322002	0.544079031	4.516778239	0.133193465	2.204456237	2	2	2	2	2.14022021	4.555645384	4.959465495
ENSG00000138768	USO1	1.375035242	0.001224561	0.828559793	0.063908241	2.203595035	8.737455672	9.470501606	9.647595056	10.72491929	10.6866731	10.20346921	10.1173006
ENSG00000204611	ZNF616	1.100075151	0.479960202	1.102409508	0.470407499	2.202484659	3.327711234	4.93638933	7.398165132	7.242377933	7.09050444	7.534088584	6.683620062
ENSG00000086289	EPDR1	0.546703924	0.35926735	1.654063983	0.001488322	2.200767907	7.790810999	7.051611315	8.143093325	8.188900477	8.32017198	9.098500449	9.569772634

ENSG00000198730	CTR9	1.250822554	0.261480902	0.949770045	0.397984664	2.200592599	6.009025997	9.079034257	7.552162999	9.302993856	9.27923788	8.800846924	9.161219358
ENSG00000198796	ALPK2	0.414499648	0.85527092	1.785334928	0.366670893	2.199834576	2	5.457120953	8.35328246	7.409376773	7.31795292	7.778984728	9.276085345
ENSG00000164162	ANAPC10	1.268298292	0.346404872	0.931143315	0.493153856	2.199441607	5.330594134	6.04685525	2	6.205262847	6.58294721	6.176128386	5.992078266
ENSG00000167566	NCKAP5L	0.580074427	0.675598697	1.619316503	0.190484764	2.19939093	5.095959458	8.687092496	9.083172337	8.904736303	8.97305476	9.987021151	9.957809357
ENSG00000143164	DCAF6	1.080334628	2.34E-05	1.117150934	1.01E-05	2.197485562	8.855032352	8.903033433	8.825624954	9.979407193	9.89304688	9.877992513	10.06325576
ENSG00000104432	IL7	3.470607319	0.449678944	-1.274101357	0.797193705	2.196505962	2	2	2	3.921164147	3.94787299	2	2.277179783
ENSG00000108515	ENO3	0.756748919	0.60852038	1.438819195	0.287769738	2.195568115	4.00722531	4.442710129	2	4.415698455	4.45712836	5.4091291	4.468068808
ENSG00000144152	FBLN7	0.893354197	0.717276749	1.301977508	0.580976488	2.195331705	2	6.535001748	2	5.808110275	6.00043082	6.428526856	6.141542327
ENSG00000085871	MGST2	1.42691189	0.183576111	0.767809654	0.49246368	2.194721544	7.159102577	5.457120953	4.523675391	7.412876874	7.57432889	6.510167449	7.133547492
ENSG00000102038	SMARCA1	1.059633281	0.180631866	1.133552576	0.1415706	2.193185857	8.009475818	10.019219	9.426401271	10.45855028	10.3940894	10.46042363	10.53927643
ENSG00000103342	GSPT1	0.950964451	0.07257246	1.240510775	0.01474777	2.191475226	9.781071289	9.616264339	8.65477198	10.40199532	10.3532448	10.58937934	10.73987554
ENSG00000116005	PCYOX1	-0.112585724	0.883684699	2.303712203	6.33E-05	2.191126478	8.836089686	10.12617756	8.960138144	9.361220151	9.28774494	11.7437434	11.72401992
ENSG00000136810	TXN	2.21967697	8.92E-05	-0.030095589	0.968502972	2.189581381	9.820154628	8.395519306	9.049077186	11.40931048	11.4175794	9.088477995	9.250223518
ENSG00000165115	KIF27	2.140021933	0.108187743	0.049334939	0.974984507	2.189356873	2	4.442710129	4.058998049	5.627094009	5.6826301	4.271694079	3.383972333
ENSG00000140829	DHX38	1.259424096	0.453919948	0.929855064	0.58225381	2.189279159	3.327711234	7.73146271	9.132850258	9.275380474	9.27448997	8.766685705	9.10860334
ENSG00000111897	SERINC1	1.225885033	0.184268122	0.962961978	0.299579278	2.188847011	9.262228249	11.59012602	11.60159843	12.39271275	12.3511729	12.12283589	12.09580137
ENSG00000109501	WFS1	-0.013752453	0.990899695	2.200707821	0.019084503	2.186955368	9.689059027	8.806266215	6.882775709	8.804373539	8.88234995	10.78181003	11.27343957
ENSG00000122484	RPAP2	0.665199535	0.680957539	1.520458532	0.301260958	2.185658067	6.563806279	6.78762695	2	6.89733479	6.6253296	7.807195765	7.355789538
ENSG00000147576	ADHFE1	1.756592586	0.340147427	0.428327528	0.833553126	2.184920114	2	4.442710129	2	4.71689672	4.70309173	3.810410249	3.539239711
ENSG00000128595	CALU	0.522318521	0.305223019	1.662540458	0.000174595	2.184858979	12.48433893	13.3410766	12.55584475	13.36578153	13.3766069	14.37571671	14.6349142
ENSG00000176014	TUBB6	0.203316404	0.803640574	1.980347806	0.002293883	2.183664211	10.98115017	10.08551947	9.658943166	10.52393752	10.5810438	11.76350665	12.73131499
ENSG00000125885	MCM8	0.783400726	0.463670664	1.400240071	0.153651094	2.183640797	5.330594134	6.04685525	3.369317972	6.252910529	5.7929843	6.611630769	6.6426114
ENSG00000158458	NRG2	1.533939055	0.536685094	0.64688277	0.804608418	2.180821826	2	2	3.369317972	3.162721474	3.39680241	3.059555873	2.551896972
ENSG00000172785	CBWD1	3.153188618	0.015021195	-0.973593346	0.501722792	2.179595272	6.73718411	6.389896633	2	9.031955922	9.16279627	4.965450964	5.28828468
ENSG00000139547	RDH16	2.690951034	0.412898781	-0.511450235	NA	2.179500799	2	2	2	3.566290607	3.2184422	2.34604626	2.117335275
ENSG00000147224	PRPS1	0.843802214	0.322921865	1.335117654	0.095474849	2.178919868	9.397585735	7.503546079	8.296287027	9.404936476	9.47652116	9.51429464	10.25250913
ENSG00000181722	ZBTB20	2.06918468	0.292000423	0.108286792	0.96272717	2.177471472	2	6.951736442	9.196533591	9.933844998	9.9554848	7.805448645	8.172535245
ENSG00000137807	KIF23	0.787010275	0.633108815	1.388702188	0.366387369	2.175712463	4.467311984	6.389896633	2	5.949776857	5.9536761	6.414841377	6.620419619
ENSG00000100600	LGMN	1.372869502	0.027892519	0.802353879	0.219885804	2.175223381	10.07567912	8.57407404	9.681375057	10.90533885	10.965595	10.48532674	10.23739929
ENSG00000105698	USF2	1.123391612	0.44980498	1.051015419	0.472698621	2.174407032	4.815609471	8.077869079	9.589475645	9.632130776	9.54672161	9.576569363	9.457070207
ENSG00000239713	APOBEC3G	1.881235425	0.124042603	0.292619756	0.836490254	2.173855181	3.327711234	6.844429755	7.225742779	8.394075037	8.31092742	6.7543034	6.843307307
ENSG00000144488	ESPNL	1.325321978	0.583890106	0.845337276	0.731701381	2.170659254	2	2	6.203069917	5.968925393	5.90535527	5.688550058	5.269393099
ENSG00000152402	GUCY1A2	-0.306792081	0.947190051	2.474906812	NA	2.168114731	2	2	2	2.3892346	2.14022021	3.904814898	2.117335275
ENSG00000137857	DUOX1	-1.310492654	0.702149071	3.476628704	0.21425456	2.16613605	2	2	2	2.141737246	2.14022021	4.199031499	3.620995423
ENSG00000100243	CYB5R3	0.177926023	0.833090562	1.986578946	0.00275976	2.164504969	12.54683767	11.6107392	10.87563042	11.97939826	12.0529163	13.58455511	14.0297935
ENSG00000101413	RPRD1B	0.779605691	0.618091142	1.384471553	0.343161384	2.164077244	7.940205782	8.276714465	3.369317972	8.327018759	8.33484115	8.974541854	8.88330014
ENSG00000125107	CNOT1	1.195998688	0.024264667	0.967802786	0.072427994	2.163801474	9.371511179	10.44821838	9.528916236	11.06873707	11.0541353	10.89498282	10.76972906
ENSG00000135338	LCA5	0.792671252	0.593368805	1.370352353	0.322128031	2.163023606	2	6.140560021	6.073378139	6.29903474	6.32824132	7.1328795	6.541257688
ENSG00000116096	SPR	1.306511771	0.451325678	0.856155112	0.627647664	2.162666883	2	7.393647515	5.59507254	7.444001439	7.5014335	7.130090369	6.932071508
ENSG00000131473	ACLY	1.014753101	0.453973535	1.147629836	0.379839399	2.162382937	7.27616306	11.20718648	11.17734896	11.64445391	11.690457	11.48501894	12.05897558
ENSG00000205208	C4orf46	1.187485266	0.561800205	0.974829048	0.635400451	2.162314314	3.327711234	7.002537973	2	6.693715713	6.72335419	6.442083733	6.564691065
ENSG00000102384	CENPI	0.439859469	0.843094589	1.721323467	0.374613381	2.161182936	2	4.93638933	2	3.881293667	4.2560182	4.764298067	5.448021784
ENSG00000143771	CNIH4	1.123941149	0.05248835	1.036586233	0.072440685	2.160527382	8.674867122	7.572420273	7.734844356	9.175891964	9.19932737	9.302713655	8.866113243
ENSG00000170561	IRX2	3.002047866	0.175108303	-0.841904842	0.730630046	2.160143023	5.866944937	2	2	7.355829978	7.03319408	4.350488971	2.918126211
ENSG00000176171	BNIP3	1.417001985	0.034893697	0.742974259	0.294688747	2.159976244	10.38028415	9.159587375	8.941672936	11.0584384	11.0477495	10.15935238	10.57256798
ENSG00000125868	DSTN	0.213268889	0.652839859	1.946352273	2.41E-07	2.159621162	11.18578229	10.62884366	10.40882047	11.02280532	10.9599875	12.79040486	12.65194484
ENSG00000182831	C16orf72	0.957091233	0.118250471	1.200001186	0.041683697	2.157092419	9.204317094	8.356994601	7.858115942	9.530270581	9.53167935	9.896369694	9.637331225
ENSG00000148175	STOM	0.721167	0.112132289	1.434681298	0.00053967	2.155848298	10.29268328	10.27307349	10.84614969	11.22124799	11.2074731	12.19425512	11.59853243
ENSG00000171552	BCL2L1	0.348999595	0.628892599	1.806260295	0.002801737	2.15525989	9.635285994	8.671479029	9.926432745	9.81982307	9.87308535	10.83148019	11.65306896
ENSG00000100697	DICER1	1.023339536	0.192776283	1.128320242	0.139340009	2.151659778	7.53297911	9.405920729	8.460932916	9.672111811	9.69692814	9.995474265	9.548056138
ENSG00000229809	ZNF688	1.175930997	0.114471804	0.975505162	0.189949684	2.151436159	6.468621203	4.93638933	5.39244166	6.750147204	6.9210099	6.693258166	6.595354992
ENSG00000218891	ZNF579	0.230873267	0.909988721	1.918362864	0.260698796	2.149236131	2	7.099070191	8.206335842	7.437142666	7.37938471	9.155502188	8.984263871
ENSG00000174238	PITPNA	0.828709484	0.042415488	1.320466184	0.000568123	2.149175668	8.737455672	8.806266215	9.426401271	9.842062221	9.84709924	10.23949951	10.42371165
ENSG00000049239	H6PD	1.155266461	0.02537741	0.993481693	0.056299816	2.148748154	8.586954094	9.424669273	9.714382178	10.44536591	10.4869034	10.14853986	10.44614079
ENSG00000153048	CARHSP1	0.525811014	0.762607571	1.622720581	0.297427124	2.148531595	8.87372952	7.315436877	3.369317972	8.197043419	8.30348889	9.114816239	9.531303742
ENSG00000116350	SRSF4	0.933273804	0.575357254	1.215154416	0.448059647	2.14842822	9.781071289	8.981105197	4.058998049	9.832946403	9.75806852	10.10942542	10.04323752
ENSG00000197548	ATG7	1.702141255	0.241411697	0.446278469	0.781965112	2.148419724	3.327711234	8.191702463	7.858115942	9.110576034	9.20732128	7.821097076	8.008487789
ENSG00000100596	SPTLC2	1.312285848	2.16E-05	0.835498776	0.009547143	2.147784624	8.928409675	8.732943944	9.272377397	10.30219818	10.2873694	9.798760102	9.840581661
ENSG00000185811	IKZF1	-1.310489421	0.712294566	3.457239919	0.238534046	2.146750498	2	2	2	2.141737246	2.14022021	4.388329244	3.236138336
ENSG00000169221	TBC1D10B	0.751418883	0.016164965	1.394133701	1.58E-06	2.145552584	8.367060731	8.505280662	8.825624954	9.290680758	9.35044873	9.952309835	9.968341625
ENSG00000152642	GPD1L	-0.617600419	0.714145675	2.761944318	0.05471533	2.144343898	3.327711234	6.78762695	3.369317972	4.984880435	4.87352701	8.088662035	8.165740501
ENSG00000078070	MCCC1	0.544060145	0.832844524	1.599817227	0.489336405	2.143877373	2	8.433041974	3.369317972	7.433701013	7.45182838	8.075674526	8.797441501
ENSG00000158042	MRPL17	0.940534771	0.013480111	1.203323984	0.001053111	2.143858755	8.418903792	8.639735838	8.960138144	9.617037214	9.62713473	9.664250432	10.07418255
ENSG00000197415	VEPH1	3.862299748	0.018782812	-1.720028368	0.333565204	2.14227138	6.468621203	2	3.369317972	8.769305409	8.7882247	3.810410249	3.600986722
ENSG00000055130	CUL1	1.409680826	0.353082226	0.727374985	0.646493605	2.137055811	8.946185348	9.148351514	4.058998049	9.894328254	9.88559363	9.2270746	9.195398998
ENSG00000171631	P2RY6	-1.825784293	0.309957701	3.962743357	0.008764582	2.136959064	2	2	4.523675391	2.498691189	2.49394783	6.453284622	6.671679221
ENSG00000005175	RPAP3	1.504681019	0.111579909	0.631212177	0.532750733	2.135893196	5.330594134	7.847167909	6.534109959	8.427334995	8.3920703	7.596037946	7.503217537
ENSG00000196782	MAML3	1.549166801	0.000756769	0.584800361	0.248420606	2.133967161	6.89194353	6.140560021	6.534109959	8.085230238	8.00412803	6.977080688	7.216434756
ENSG00000205542	TMSB4X	1.545809455	0.230169878	0.587589585	0.673347742	2.13339904	13.38954194	10.2574711	10.37481416	13.64291503	13.6712421	12.50279851	12.87228671
ENSG00000167034	NKX3-1	2.554818388	0.310002526	-0.421514527	0.883235347	2.133303861	7.579279349	2	2	8.534041678	8.52593541	5.598599872	5.713290392
ENSG00000158006	PAFAH2	1.09851974	0.164155144	1.034625514	0.183338933	2.133145254	4.815609471	6.602424674	6.80318718	7.288834728	7.4450836	7.335328635	7.277588383
ENSG00000166965	RCCD1	0.129305505	0.930223809	2.003586846	0.09467842	2.132892352	3.327711234	5.838937808	7.163363831	6.221320894	6.27599024	8.110758945	8.021718375
ENSG00000185798	WDR53	1.014669265	0.686884062	1.117790682	0.649707664	2.132459947	2	6.78762695	2	6.155987605	6.36443784	6.419417643	6.301968891
ENSG00000094975	SUCO	2.178283292	4.83E-06	-0.046604406	0.942222441	2.131678886	7.904282243	8.777382925	7.97167868	10.45897359	10.437637	8.450751194	7.989373961
ENSG00000159658	EFCAB14	0.885505418	0.598361649	1.243089391	0.438092003	2.128594809	4.815609471	9.497318007	10.60345421	10.4615108	10.4753644	10.72979303	10.91456428
ENSG00000104611	SH2D4A	1.100412822	0.552383336	1.027402343	0.575949683	2.127815164	7.82963488	5.595927442	2	7.593372273	7.62295033	7.595026956	7.476011586
ENSG00000139629	GALNT6	0.825125251	0.535581235	1.301734213	0.299116044	2.126859464	4.815609471	6.951736442	5.39244166	6.766655021	6.8715633	8.000548654	5.785984581
ENSG00000167491	GATAD2A	0.787996563	0.211852193	1.338231584	0.023059327	2.126228147	8.928409675	9.566483142	8.143093325	9.787157185	9.77564777	10.24063129	10.41335128
ENSG00000164692	COL1A2	1.235715598	0.003820004	0.889991914	0.043497213	2.125707512	15.2112892	16.03971608	15.34601607	16.7869716	16.843947	16.52762301	16.41003914
ENSG00000105717	PBX4	0.343496344	0.886013357	1.781386183	0.37934489	2.124882526	2	3.686303414	2	2.947924294	3.15372745	3.838019203	4.09155917
ENSG00000169410	PTPN9	0.996895769	0.079429462	1.126042535	0.041645457	2.122938305	8.39321512	9.04308532	7.858115942	9.44822005	9.56860713	9.42949507	9.820443605
ENSG00000169718	DUS1L	0.661835494	0.162215294	1.460580477	0.000597178	2.122415971	7.159102577	7.847167909	7.098165388	8.027038952	8.12510181	8.897500263	8.834865421
ENSG00000112144	ICK	0.576350975	0.577039334	1.544706479	0.094399166	2.121057453	7.82963488	6.951736442	5.156626401	7.481152682	7.66128317	8.431623835	8.617426278
ENSG00000159579	RSPRY1	1.255664223	0.128350341	0.865018298	0.303980057	2.120682522	6.009025997	7.847167909	8.175061096	8.80037045	8.88608977	8.47458408	8.439433377
ENSG00000172794	RAB37	-1.303609501	0.785196727	3.423661538	0.407675561	2.120052037	2	2	2	2.270786764	2	3.753554863	4.033026654
ENSG00000167962	ZNF598	0.569490076	0.594985066	1.55055171	0.104869869	2.120041787	8.946185348	7.145017454	6.534109959	8.444537031	8.51631523	9.584486439	9.310375569
ENSG00000173218	VANGL1	0.826358247	0.659761643	1.293526872	0.466865178	2.119885118	8.257427002	7.874700259	2	8.266399315	8.36909406	8.768480374	8.793039018
ENSG00000178922	HYI	7.310913843	0.000544227	-5.191145552	0.064663947	2.119768291	2	2	2	7.277360022	7.46188679	2	2
ENSG00000198707	CEP290	1.538513348	0.010550428	0.581054632	0.377325083	2.11956798	6.563806279	6.389896633	7.398165132	8.413424144	8.27524772	7.70407074	7.026981076
ENSG00000157827	FMNL2	1.601873532	5.08E-05	0.516317938	0.244087001	2.11819147	8.075571721	8.862353853	8.825624954	10.2136641	10.2384727	9.194070966	9.096149382
ENSG00000118193	KIF14	1.721529087	0.383405777	0.394856696	0.855899236	2.116385783	2	4.442710129	3.369317972	5.058234798	4.91316568	2.656294034	4.574506939
ENSG00000162396	PARS2	0.622459673	0.80586146	1.491978265	0.519967781	2.114437938	2	6.464272742	2	5.709096143	5.45984938	6.583362013	6.199243628
ENSG00000105227	PRX	0.050800721	0.970008162	2.063092197	0.059117014	2.113892918	5.095959458	3.686303414	6.322058544	5.44762523	5.39133044	7.774526492	6.674075308
ENSG00000103932	RPAP1	0.693426144	0.557986927	1.420235183	0.191114239	2.113661327	4.467311984	7.701025996	6.882775709	7.494428494	7.59275449	8.425380879	8.070734444
ENSG00000248167	TRIM39-RPP	7.304108672	0.000536632	-5.191145622	0.064342105	2.11296305	2	2	2	7.366699513	7.36516374	2	2
ENSG00000111676	ATN1	0.589393105	0.299726211	1.523514912	0.002547818	2.112908017	9.710019321	10.83211025	10.02619908	10.87266806	10.8474839	11.76749432	11.81761433
ENSG00000175548	ALG10B	0.974855736	0.52971614	1.136945908	0.446952646	2.111801643	2	4.93638933	6.322058544	6.096288482	6.18985553	6.577231679	5.949196291
ENSG00000183475	ASB7	1.191054147	0.056624958	0.918483126	0.145949245	2.109537273	6.89194353	7.274681896	5.772716771	7.956842238	7.95274764	7.759264603	7.610940609
ENSG00000163376	KBTBD8	2.129030575	0.307499144	-0.022709082	0.992767357	2.106321493	2	5.838937808	2	6.456560224	6.34282908	4.652122235	4.254128754
ENSG00000126790	L3HYPDH	1.780330451	0.329205175	0.325694062	0.874266884	2.106024513	2	7.572420273	8.631919287	9.371149971	9.39049411	7.805448645	8.065268319
ENSG00000143819	EPHX1	1.362412843	0.137222257	0.742887558	0.437636581	2.105300401	7.031698319	9.479495904	8.561105505	10.00514223	10.051861	9.133040681	9.645901223
ENSG00000156467	UQCRB	1.026367173	0.253648116	1.077103351	0.220279485	2.103470523	9.344956677	7.431218269	7.552162999	9.432787137	9.38785872	9.405475868	9.514354532
ENSG00000119408	NEK6	1.293232827	0.02572938	0.809185808	0.179357289	2.102418635	8.696033109	8.234834549	7.398165132	9.531881013	9.44136842	8.749978316	9.224298414
ENSG00000167671	UBXN6	1.06148861	0.100196808	1.040081423	0.102066442	2.101570033	10.06758145	9.377333006	8.536706852	10.47454911	10.5546556	10.3964353	10.58503594
ENSG00000111261	MANSC1	0.095188805	0.947256262	2.006367875	0.094088158	2.101556681	7.709880939	6.140560021	4.058998049	6.676346669	6.75642009	8.181164698	8.903861917
ENSG00000261794	GOLGA8H	7.289289823	0.000584335	-5.191145507	0.064982565	2.098144316	2	2	2	7.348537858	7.35440525	2	2
ENSG00000184208	C22orf46	1.367525311	0.478780358	0.730503765	0.715215591	2.098029076	2	6.666836837	8.561105505	8.570539957	8.77214691	7.702194078	8.322876198

ENSG00000032444	PNPLA6	0.742322965	0.499134136	1.355206025	0.181428238	2.09752899	9.613202746	8.732943944	6.629488387	9.466861018	9.5427783	10.02204569	10.20317517
ENSG00000196814	MVB12B	0.472514452	0.786259522	1.624572995	0.291093097	2.097087448	6.009025997	7.189546432	2	6.542151749	6.70088158	7.677572696	7.805990373
ENSG00000033170	FUT8	2.111948023	0.224881984	-0.015629172	0.993931149	2.096318851	7.624139768	8.451443573	2	9.633631483	9.59321927	7.475964489	7.544398272
ENSG00000035687	ADSS	1.740770814	0.074138279	0.355001497	0.749691042	2.095772311	8.75772933	7.669933291	5.930865648	9.587916984	9.58096568	8.068408401	8.340330468
ENSG00000148498	PARD3	0.969397607	0.570297901	1.126108591	0.497145211	2.095506198	5.330594134	8.968383114	4.523675391	8.52598359	8.50824913	8.728472558	8.613686471
ENSG00000144229	THSD7B	-1.310484826	0.725904626	3.405260271	0.276194973	2.094775445	2	2	2	2.141737246	2.14022021	2.831336763	4.489993931
ENSG00000119431	HDHD3	0.529363649	0.799141442	1.565366821	0.400363179	2.09473047	3.327711234	6.602424674	2	5.720439317	5.72778967	6.908747388	6.463051659
ENSG00000170242	USP47	0.47034009	0.630948994	1.6242741	0.057634332	2.094614191	7.790810999	10.17682307	9.681375057	10.01035013	9.96612421	11.26733166	10.99857641
ENSG00000188610	FAM72B	3.975605547	0.032131199	-1.8835802	0.369040004	2.092025347	2	3.076665209	2	5.144975053	5.13053105	2.139668783	2.225841198
ENSG00000198523	PLN	-0.302794136	0.958996611	2.394498538	NA	2.091704402	2	2	2	2.498691189	2	3.584558147	2.746601469
ENSG00000114861	FOXP1	1.247134941	0.046544775	0.844206088	0.189633354	2.091341029	8.564112515	7.701025996	9.212024683	9.863963753	9.83811764	9.489210754	9.410049744
ENSG00000117525	F3	1.257342908	0.283345486	0.83378505	0.485029668	2.091127959	7.82963488	8.655694736	10.81097638	10.90440671	10.9195043	10.75048495	10.17045486
ENSG00000144476	ACKR3	-1.705607266	0.312710697	3.796040524	0.007955752	2.090433258	2	4.442710129	2	2.498691189	2.68924543	6.685680098	6.506723027
ENSG00000063587	ZNF275	0.599152545	0.684522822	1.490321074	0.263504222	2.089473619	4.467311984	8.356994601	8.865353883	8.658676732	8.7017439	9.510546344	9.617549359
ENSG00000163923	RPL39L	0.658298662	0.523956356	1.430538929	0.125265786	2.088837591	4.467311984	5.457120953	6.882775709	6.529309835	6.56439512	7.196918221	7.383872668
ENSG00000102109	PCSK1N	-1.478187369	0.479762551	3.566701523	0.040078938	2.088514154	2	3.686303414	2	2.498691189	2.2680111	5.376552034	5.581648407
ENSG00000205356	TECPR1	0.357912054	0.759044086	1.729908263	0.083021331	2.087820317	5.330594134	7.638155693	8.35328246	7.947219213	7.8745862	9.284329221	9.25263028
ENSG00000176771	NCKAP5	0.708357214	0.569536355	1.375695775	0.229859615	2.084052988	5.330594134	4.710564154	2	5.110902178	5.24368216	5.912830251	5.646532192
ENSG00000116962	NID1	1.273360454	0.052328744	0.809913956	0.232791888	2.08327441	9.992581382	11.43463583	11.43667516	12.34427175	12.3883446	11.73900314	12.05104696
ENSG00000100578	KIAA0586	1.598995805	0.110752836	0.483987996	0.66285442	2.082983801	7.218819781	5.72254323	4.523675391	7.794595599	7.74329312	6.759726988	6.605433158
ENSG00000114349	GNAT1	3.97418412	0.311367551	-1.892859025	0.658045217	2.081325096	2	2	2	4.237680128	4.40247105	2.183373728	2
ENSG00000142784	WDTC1	0.204889618	0.864559929	1.876227703	0.053436739	2.081117321	6.73718411	8.806266215	9.670207709	8.980991923	9.09008229	10.59432051	10.79911455
ENSG00000188177	ZC3H6	0.285820131	0.852386951	1.793123873	0.16325414	2.078944004	4.467311984	4.93638933	7.646393253	6.610808945	6.70653264	8.393172835	7.805990373
ENSG00000079950	STX7	0.81817604	0.211593503	1.260594199	0.040591855	2.078770239	7.53297911	7.572420273	8.763901704	8.896009193	8.86223882	9.443323792	9.181571564
ENSG00000159708	LRRC36	-0.723328282	0.850026272	2.801721455	NA	2.078393173	2	2	2	2.270786764	2.14022021	3.767980144	3.100202476
ENSG00000153832	FBXO36	0.219314878	0.892703066	1.858793286	0.165125488	2.078108164	4.815609471	6.04685525	2	5.242616075	5.28958453	7.07314455	6.463051659
ENSG00000091656	ZFHX4	0.761280191	0.673950159	1.315811245	0.43784645	2.077091436	3.327711234	7.669933291	9.358421373	8.956018563	8.90710092	9.17593001	9.735236833
ENSG00000129925	TMEM8A	1.011536175	0.000651291	1.064534128	0.000282431	2.076070303	8.928409675	8.955547846	9.180874355	9.963981511	10.0928914	10.21815365	9.933014227
ENSG00000132510	KDM6B	1.063331231	0.04914446	1.012512125	0.058936535	2.075843355	8.075571721	9.055168103	8.110401118	9.524619869	9.58096568	9.641417554	9.348752388
ENSG00000147316	MCPH1	0.99471337	0.03160337	1.081041314	0.017106546	2.075754684	7.031698319	6.899080544	6.203069917	7.805288479	7.65545169	7.832294085	7.80161394
ENSG00000127720	METTL25	1.685673245	0.412705524	0.388512835	0.86358453	2.074186081	2	5.457120953	2	5.674521485	5.55061739	4.563936166	4.388595494
ENSG00000149577	SIDT2	0.262045899	0.865152618	1.811856615	0.158126095	2.073902515	5.095959458	9.433952963	8.434763955	8.765203682	8.72141482	10.32932464	10.23800673
ENSG00000215375	MYL5	0.291595036	0.869037712	1.781711608	0.230812916	2.073306644	6.366709671	3.076665209	3.369317972	5.304221918	5.33407135	6.645228724	6.777976866
ENSG00000132952	USPL1	1.25192505	0.438223548	0.82094495	0.617362697	2.07287	5.330594134	7.979858146	3.369317972	7.875463856	7.90685167	7.610117916	7.311843322
ENSG00000145349	CAMK2D	0.105868856	0.841743726	1.966529087	7.45E-07	2.072397944	9.27634997	8.639735838	8.460932916	8.95841569	8.92053511	10.86761565	10.71157971
ENSG00000075785	RAB7A	1.191584289	3.95E-05	0.880697515	0.003096188	2.072281804	11.01943573	10.70750522	10.92346452	12.07293465	12.0829612	11.56367292	11.94591045
ENSG00000088280	ASAP3	0.137132493	0.942460221	1.935037258	0.22807891	2.072169751	4.00722531	8.029695761	4.058998049	6.722207358	6.79936111	8.503966641	8.537437492
ENSG00000165879	FRAT1	1.649309323	0.393187917	0.420733766	0.842253868	2.070043089	2	4.93638933	2	5.093559143	5.07918227	3.956110469	4.175132639
ENSG00000116237	ICMT	0.217117669	0.564310859	1.852325354	8.02E-10	2.069443023	9.497412313	9.159587375	9.703463497	9.664791023	9.69834686	11.23953894	11.38121091
ENSG00000030419	IKZF2	1.652441248	0.144835476	0.416910017	0.740394508	2.069351265	7.031698319	4.11352228	7.285536013	8.270263574	8.25418043	7.264607048	6.802276223
ENSG00000125388	GRK4	0.078559941	0.963296195	1.990331221	0.163471043	2.068891161	5.70932728	3.686303414	2	4.523237872	4.63360314	6.512311087	6.055940621
ENSG00000187775	DNAH17	0.95353929	0.634148538	1.115076707	0.565593677	2.068615997	2	4.710564154	2	4.299492648	4.2865223	4.388329244	4.456980017
ENSG00000140299	BNIP2	1.072717989	0.017649875	0.995650734	0.027841943	2.068368723	9.262228249	8.671479029	8.325066185	9.899327447	9.84197374	9.865050672	9.719663687
ENSG00000130045	NXNL2	1.348833847	0.468732877	0.719504684	0.707381508	2.068338532	3.327711234	2	3.369317972	3.709785322	3.82856379	3.374372158	3.33635788
ENSG00000142556	ZNF614	0.864472727	0.208583912	1.20383812	0.064420602	2.068310847	5.70932728	6.78762695	5.59507254	6.936770382	7.05550676	7.464938976	7.174740504
ENSG00000126458	RRAS	0.125842831	0.857281815	1.941775241	0.000274788	2.067618071	9.624286623	8.317412869	9.301632639	9.260887574	9.33776001	11.1676629	11.04825611
ENSG00000105483	CARD8	1.162410655	0.194213675	0.904980874	0.314431097	2.067391529	4.815609471	6.728495673	7.029880454	7.647855834	7.53675633	7.621085148	6.994755759
ENSG00000111364	DDX55	1.297176716	0.268205982	0.769863359	0.524378192	2.067040075	3.327711234	6.602424674	6.629488387	7.402350992	7.31055061	6.99251878	6.681239787
ENSG00000111834	RSPH4A	2.687966558	0.392020561	-0.621070025	NA	2.066896533	2	2	2	3.227757282	3.55551834	2.267001569	2.172608095
ENSG00000167635	ZNF146	1.018763598	0.065760624	1.045770613	0.05485499	2.064534211	8.340423424	9.443177293	8.677268317	9.941741253	9.88808233	10.12876594	9.727471273
ENSG00000102181	CD99L2	0.618665536	0.065340768	1.44445115	1.90E-06	2.063116686	9.031919741	9.34816733	8.922968322	9.720225568	9.74577157	10.42331231	10.67735328
ENSG00000205209	SCGB2B2	2.646475826	0.406678307	-0.58751271	NA	2.058963116	2	2	2	3.663521627	3.01487888	2.225793514	2.225841198
ENSG00000157593	SLC35B2	1.160213427	0.174135326	0.898464736	0.295507516	2.058678163	10.44422623	8.862353853	8.486635645	10.67696444	10.6920239	10.41660409	10.4302822
ENSG00000134574	DDB2	-0.46952849	0.751812007	2.527512477	0.044161511	2.057983987	4.815609471	6.602424674	8.784770914	7.097651217	7.0991228	9.939024914	10.16811941
ENSG00000133639	BTG1	2.057505175	4.32E-06	-0.000586523	0.999263508	2.056918652	9.317904228	9.883504052	9.116480286	11.52844449	11.5359724	9.755754661	9.139275853
ENSG00000125834	STK35	0.826380415	0.259012717	1.228198194	0.075285273	2.054578609	8.493334308	7.928237109	6.718950348	8.631895657	8.77079897	9.027959735	9.17143135
ENSG00000139508	SLC46A3	0.234320866	0.892887789	1.819801372	0.209657689	2.054122238	6.96351253	6.311476754	2	6.321555392	6.36443784	8.037484631	7.704159082
ENSG00000136068	FLNB	0.789900989	0.231292431	1.260700946	0.041745906	2.050601935	9.730679447	10.87704672	11.18128933	11.49126529	11.5214183	11.78261435	12.14769299
ENSG00000139687	RB1	0.782164367	0.610603213	1.268152464	0.37875047	2.050316831	4.00722531	8.639735838	8.65477198	8.829471715	8.90095288	9.435155466	9.255433104
ENSG00000078124	ACER3	0.937784627	0.084457614	1.109467848	0.034537867	2.047252475	6.89414963	7.790474954	6.80318718	8.213192703	8.13351654	8.362013331	8.324402372
ENSG00000167967	E4F1	0.82642216	0.716973839	1.220110514	0.575342928	2.046532674	4.00722531	8.191702463	2	7.487805859	7.53992502	8.032265607	7.754841567
ENSG00000144677	CTDSPL	0.221245882	0.833848549	1.825136933	0.033221064	2.046382815	7.33131396	8.848535039	6.629488387	8.074220386	8.19711812	9.752357958	9.701279971
ENSG00000165914	TTC7B	0.597345931	0.738885038	1.448602268	0.376394793	2.045948199	7.667647223	7.232742069	2	7.497728447	7.46522407	8.337438499	8.299011699
ENSG00000182141	ZNF708	1.375307317	0.061315658	0.670203979	0.387886116	2.045511296	4.467311984	5.457120953	5.59507254	6.592405944	6.48770488	6.123812431	5.571402115
ENSG00000068024	HDAC4	0.92948396	0.351836711	1.115045024	0.246860689	2.044528983	8.107517749	6.389896633	5.772716771	8.065351606	7.96222667	8.189896068	8.205208936
ENSG00000204962	PCDHA8	2.394791035	0.502627905	-0.351846149	NA	2.042944886	2	2	2	3.663521627	2.59489879	2.34604626	2.172608095
ENSG00000170606	HSPA4	0.521783279	0.243498812	1.518407118	0.000112402	2.040190397	10.32020829	9.672251278	10.37481416	10.67405041	10.6723063	11.48009892	11.8343144
ENSG00000117425	PTCH2	1.007616749	0.61882206	1.032529331	0.602505282	2.04014608	2	4.442710129	2	3.840289928	4.31639476	4.155805465	4.077147165
ENSG00000198040	ZNF84	0.976125375	0.579599436	1.063817107	0.536100907	2.039942482	6.366709671	8.848535039	3.369317972	8.50645854	8.48542112	8.888863837	8.193564875
ENSG00000118260	CREB1	1.005470001	0.370206288	1.032536461	0.34763435	2.038006463	8.928409675	7.099070191	6.073378139	8.857997553	8.82115545	9.009866973	8.707318295
ENSG00000178809	TRIM73	3.146338608	0.298074813	-1.109995635	0.738292369	2.036342974	2	2	2	3.349643765	3.98554648	2.094598369	2.225841198
ENSG00000105287	PRKD2	1.64553101	0.180388625	0.388006168	0.778107221	2.033537178	5.330594134	7.874700259	9.180874355	9.772927884	9.79765578	8.563064723	8.5102048
ENSG00000158623	COPG2	0.529217728	0.788534399	1.502396899	0.397192742	2.031614627	4.815609471	7.393647515	2	6.522845756	6.63721363	7.438128798	7.605929526
ENSG00000113273	ARSB	-0.04275236	0.96053419	2.07355431	0.002204294	2.03080195	8.981092835	8.213429689	9.926432745	9.161399541	9.16382362	11.01246883	11.49043785
ENSG00000148344	PTGES	2.472976517	0.016358013	-0.443783557	0.713381003	2.0291926	9.317904228	6.728495673	8.99637432	11.11566977	11.2164127	7.241533944	8.859812348
ENSG00000100625	SIX4	0.892093948	0.201147319	1.136893421	0.089227181	2.028987369	6.366709671	7.002537973	7.896971941	8.098332103	8.08878133	8.10153367	8.536120927
ENSG00000187860	CCDC157	1.541048918	0.465537342	0.485539685	0.83120048	2.026588603	2	5.457120953	2	5.406080789	5.55061739	4.301748724	4.77533149
ENSG00000183044	ABAT	-0.133894872	0.959837734	2.160401778	0.351928367	2.026506906	2	6.464272742	2	5.210797711	4.53541753	6.040114727	7.632045641
ENSG00000116741	RGS2	3.094439944	0.115966618	-1.067995374	0.620242104	2.02644457	2	6.04685525	2	7.507583254	7.58049694	3.993419637	3.640730416
ENSG00000129534	MIS18BP1	1.424095042	0.406678307	0.602313496	0.738341738	2.026408538	7.53297911	5.946638857	2	7.805288479	7.72392024	7.277267764	6.562106074
ENSG00000141232	TOB1	0.692896367	0.278800879	1.33332274	0.02331086	2.026219107	8.696033109	7.35507212	7.896971941	8.782894215	8.75042728	9.262527297	9.529981565
ENSG00000125414	MYH2	-0.727730262	0.847740162	2.752531592	NA	2.02480133	2	2	2	2.141737246	2.2680111	3.600766115	3.261853426
ENSG00000156140	ADAMTS3	-0.542821492	0.79462063	2.567120897	0.143469849	2.024299404	2	4.93638933	2	3.615725078	3.15372745	5.856572853	5.992078266
ENSG00000168710	AHCYL1	0.650427441	0.65319082	1.373441676	0.302366413	2.023869118	6.138360429	10.35843478	10.32581712	10.4457931	10.4431479	11.20533263	11.12524595
ENSG00000102531	FNDC3A	1.682117034	0.036619822	0.339069233	0.715181868	2.021186267	8.75772933	10.51971956	8.784770914	11.29209249	11.2860026	9.872996531	10.02223248
ENSG00000171469	ZNF561	0.829315169	0.710155984	1.188861007	0.576686766	2.018176176	5.532353208	8.929529568	2	8.29513208	8.3183278	8.902003555	8.374617996
ENSG00000124212	PTGIS	-2.967908405	1.71E-05	4.982329361	3.42E-14	2.014420956	8.998234871	7.538394143	9.132850258	5.639097847	6.00043082	13.22463176	14.02033476
ENSG00000140320	BAHD1	-0.024035888	0.992479093	2.038109097	0.326076859	2.014073209	8.716893051	4.710564154	2	7.161462517	7.24992376	9.327881973	9.143172568
ENSG00000056558	TRAF1	0.714654931	0.737269626	1.298986754	0.51714034	2.013641686	9.174465797	6.899080544	2	8.622857004	8.52113334	9.050629989	9.247008243
ENSG00000214402	LCNL1	-3.753100702	0.367367297	5.7656261	0.097288553	2.012525398	2	2	2	2	2	5.436478516	6.238627697
ENSG00000167106	FAM102A	0.993174344	0.153699077	1.019262224	0.134226978	2.012436569	7.096806373	8.169643011	8.65477198	9.073461088	9.10727282	9.379876477	8.793590064
ENSG00000120925	RNF170	1.441182039	0.282237791	0.570850055	0.690155642	2.012032094	5.70932728	8.101365653	4.523675391	8.302698479	8.2790453	7.611118389	7.236027917
ENSG00000134184	GSTM1	1.879597568	0.044020715	0.130781314	0.909359225	2.010378882	3.327711234	5.303523747	5.930865648	6.917187326	7.00595521	5.079718116	5.524371359
ENSG00000130762	ARHGEF16	-3.651647412	0.050971196	5.661862532	1.57E-05	2.01021512	2	4.11352228	2	2.141737246	2.14022021	8.342868948	7.685278529
ENSG00000113658	SMAD5	0.823612406	0.183172174	1.184655633	0.043076236	2.008268039	7.975256505	9.13702746	8.143093325	9.37922384	9.2990102	9.880691463	9.49042201
ENSG00000203965	EFCAB7	1.412224052	0.238722314	0.594761866	0.641715954	2.006985918	2	4.93638933	4.523675391	5.48800675	5.55061739	4.977914261	4.584731282
ENSG00000125398	SOX9	2.492994734	0.084338909	-0.488039148	0.767692243	2.004955585	2	6.844429755	6.882775709	8.78964092	8.68752719	5.740677235	5.949196291
ENSG00000145911	N4BP3	0.932948161	0.596379651	1.070943515	0.527954208	2.003891675	4.00722531	3.076665209	2	3.997744386	3.69849104	4.240999983	3.600986722
ENSG00000103569	AQP9	-2.306085432	0.608215887	4.308584281	0.248121319	2.00249885	2	2	2	2.141737246	2	5.362362054	2.851942824
ENSG00000181274	FRAT2	1.068447869	0.474064658	0.93276735	0.527954208	2.001215219	3.327711234	6.728495673	4.058998049	6.496695859	6.507263	6.439833081	6.301968891
ENSG00000120278	PLEKHG1	0.059358047	0.977133743	1.941358321	0.263352567	2.000716367	4.00722531	3.076665209	4.058998049	4.470469939	2.49394783	5.801542492	4.654341953
ENSG00000157954	WIPI2	1.138662932	0.005531762	0.861177185	0.040634846	1.999840117	8.674867122	9.357954881	9.491321578	10.29699874	10.4028177	10.0143146	10.13197271
ENSG00000124145	SDC4	1.389759114	0.045746811	0.609097571	0.415216121	1.998856685	10.46286899	9.994333773	10.69449487	11.77954155	11.8176385	10.02373723	11.60270478
ENSG00000167604	NFKBID	1.059044179	0.41446225	0.939768417	0.46274242	1.998812596	4.00722531	4.710564154	2	4.804776789	4.93258395	4.588526421	4.927687581
ENSG00000013297	CLDN11	0.36503847	0.747163598	1.633195902	0.094043218	1.998234372	9.233563228	7.232742069	6.882775709	8.509731121	8.5668971	9.983548733	9.584176963
ENSG00000171522	PTGER4	-0.17050714	0.8177838	2.168222171	0.000117699	1.997715031	6.257048221	5.303523747	6.322058544	5.731694	5.94414054	7.909957323	8.26365177
ENSG00000123066	MED13L	0.975460253	0.325172387	1.020877253	0.292072257	1.996337506	7.218819781	9.835272298	9.491321578	10.1698607	10.2039308	10.34849843	10.10580955

ENSG00000124444	ZNF576	1.009877852	0.432702068	0.98626107	0.434435201	1.996138922	5.866944937	7.051611315	3.369317972	7.169761359	6.93551934	6.975527754	7.091143645
ENSG00000198900	TOP1	1.892943283	5.06E-05	0.10316678	0.864675088	1.996110063	8.042902253	9.125613817	8.608698767	10.51744927	10.5783495	8.854214238	8.675981178
ENSG00000180573	HIST1H2AC	2.423975732	0.069296116	-0.427940406	0.78199021	1.996035325	8.228665467	6.04685525	4.058998049	9.387252775	9.37105521	6.305369896	6.811011855
ENSG00000100084	HIRA	2.159059129	0.217270166	-0.163562477	0.936599654	1.995496652	2	6.535001748	8.236946975	9.243301698	9.10406516	6.831875681	6.949978473
ENSG00000025800	KPNA6	0.592841996	0.529125418	1.401132362	0.098395659	1.993974358	8.517314938	10.74155032	9.478569125	10.42557724	10.4844385	11.16101747	11.35606301
ENSG00000151692	RNF144A	2.57479024	0.07368721	-0.580918544	0.72430187	1.993871695	3.327711234	3.686303414	3.369317972	5.48800675	5.6121243	3.5515855	2.551896972
ENSG00000140525	FANCI	1.357596551	0.495803512	0.635527999	0.760366765	1.99312455	3.327711234	6.844429755	2	6.777556219	6.67228943	6.069527745	6.007361012
ENSG00000273542	HIST1H4K	4.113472272	0.110962	-2.120377681	0.467466101	1.993094591	2	2	2	4.070463721	4.72553021	2.048074405	2.117335275
ENSG00000186063	AIDA	2.402091618	2.47E-06	-0.409142676	0.50032005	1.992948942	7.624139768	8.834582582	8.763901704	10.91771013	10.8737771	8.164223608	8.038552764
ENSG00000179938	GOLGA8J	7.183212894	0.001052791	-5.191144637	0.070759925	1.992068258	2	2	2	7.242377933	7.2537885	2	2
ENSG00000106483	SFRP4	-0.317065049	0.908151255	2.308499981	0.3091698	1.991434932	4.00722531	2	2	3.09461492	2.49394783	5.056402493	3.67940853
ENSG00000087995	METTL2A	0.955415107	0.085357884	1.035720315	0.054916044	1.991135422	6.653098118	5.946638857	6.958202216	7.620871236	7.32531744	7.514972902	7.602159758
ENSG00000213903	LTB4R	1.60284347	0.244378928	0.386931947	0.800472699	1.989775417	4.467311984	6.78762695	3.369317972	7.071314871	7.18258783	6.265258498	5.586744392
ENSG00000142961	MOB3C	1.16733765	0.531143362	0.822401268	0.664743846	1.989738918	2	6.728495673	8.266922072	8.168339746	8.35477078	7.982106617	7.864857644
ENSG00000159461	AMFR	1.161429693	0.450144124	0.826479648	0.594338258	1.987909341	5.095959458	9.695596167	10.23774519	10.54602092	10.6286993	10.21239669	10.2943831
ENSG00000167291	TBC1D16	0.554275226	0.386935633	1.43284042	0.011978839	1.987115645	8.313285031	8.88960062	9.670202709	9.591782342	9.6360458	10.56931498	10.40454019
ENSG00000182087	TMEM259	1.113588749	0.167448969	0.872890905	0.282010983	1.986479654	8.468948322	9.77810053	10.53119344	10.90782161	10.9516838	10.80647677	10.56274175
ENSG00000144597	EAF1	1.678974475	7.14E-07	0.304608198	0.446952646	1.983582673	7.790810999	8.376385542	7.97167868	9.746817367	9.73820484	8.424811996	8.331250243
ENSG00000033627	ATP6V0A1	0.705848517	0.361961292	1.277567849	0.074535792	1.983416365	7.218819781	9.055168103	8.561105505	9.118100239	9.21527115	9.641907141	9.824224109
ENSG00000126070	AGO3	0.263460219	0.895265695	1.718757087	0.311175193	1.982217305	3.327711234	8.077869079	4.523675391	6.887305128	6.96410766	8.620830655	8.018893446
ENSG00000159884	CCDC107	1.903588051	0.000157844	0.078213856	0.906201712	1.981801906	6.468621203	5.595927442	5.930865648	7.865280446	7.85690889	6.004008792	6.185873005
ENSG00000155849	ELMO1	-0.949250998	0.705707601	2.929538429	0.158640059	1.980287431	2	2	3.369317972	2.3892346	2.2680111	4.166733998	4.341135913
ENSG00000118454	ANKRD13C	0.962395522	0.410440873	1.016959244	0.370886214	1.979354765	5.095959458	8.414402626	7.398165132	8.425603456	8.48870446	8.525352075	8.497408326
ENSG00000236334	PPIAL4G	8.131637665	2.47E-08	-6.152947346	0.006279821	1.978690319	2	2	3.369317972	9.118100239	9.14209426	2	2
ENSG00000143106	PSMA5	1.715082549	0.000164731	0.263419042	0.630693854	1.978501591	8.107517749	7.60566235	8.631919287	9.837511512	9.89490419	8.362607519	8.485861839
ENSG00000160563	MED27	0.966173185	0.452168923	1.011813425	0.418932978	1.97798661	3.327711234	6.899080544	6.882775709	7.318995108	7.28811304	7.192914486	7.488317792
ENSG00000164134	NAA15	1.087526297	0.384241406	0.888888282	0.476444675	1.976414579	8.285626337	8.88960062	5.156626401	9.248208205	9.10620439	9.065304654	8.893103533
ENSG00000196550	FAM72A	4.228078017	0.116554196	-2.251841313	0.44435556	1.976236704	2	3.076665209	2	5.461211713	5.2591458	2	2.277179783
ENSG00000069943	PIGB	1.801684997	0.175359664	0.174251143	0.91151969	1.975936139	7.435667355	5.72254323	3.369317972	8.054188409	8.0358969	6.247352872	6.649933445
ENSG00000197724	PHF2	0.695739744	0.117053593	1.278262607	0.00175921	1.974002351	8.609439662	8.053983481	7.777116083	8.875861021	8.87609528	9.513224692	9.393060099
ENSG00000059122	FLYWCH1	0.621251494	0.408406753	1.352075789	0.047025411	1.973327283	9.435834703	8.101365653	8.110401118	9.331938606	9.28585881	10.27575641	9.7489399
ENSG00000197217	ENTPD4	0.590165445	0.181562967	1.382535764	0.000480809	1.972701209	9.590776211	9.377333006	8.742726175	9.889939704	9.84709924	10.66659669	10.65022737
ENSG00000108176	DNAJC12	1.527874574	0.42279064	0.444534421	0.830158879	1.972408995	2	4.442710129	2	4.443344104	4.58534546	3.584558147	3.789585079
ENSG00000108091	CCDC6	0.805953489	0.015213259	1.165326286	0.000221542	1.971279775	8.257427002	8.147241016	8.266922072	9.021676912	9.01805981	9.538686959	9.194147421
ENSG00000183726	TMEM50A	1.543837906	0.020447291	0.424907773	0.571127612	1.968745679	10.72212986	9.664384941	9.196533591	11.5430919	11.5505775	10.17296681	10.64840071
ENSG00000198642	KLHL9	0.825875243	0.038004723	1.142049101	0.00262317	1.967924344	8.107517749	8.848535039	8.699419242	9.433648866	9.38609912	9.752584654	9.695984164
ENSG00000119285	HEATR1	1.220800339	0.486454906	0.74690313	0.678808079	1.967703469	6.366709671	8.993716073	3.369317972	8.883449599	8.83798691	8.408791501	8.374617996
ENSG00000167325	RRM1	0.909580293	0.629335817	1.057573967	0.564110709	1.967154261	8.340423424	8.053983481	2	8.532433657	8.52113334	8.597294425	8.745413684
ENSG00000044446	PHKA2	0.46178078	0.801844483	1.505103633	0.356564979	1.966884413	6.009025997	7.467835385	2	6.772115916	6.84618961	7.939729349	7.702986259
ENSG00000276276		7.157683347	0.00128827	-5.191144223	0.073251024	1.966539123	2	2	2	7.337530098	7.0991228	2	2
ENSG00000228284		-3.753094472	0.381747878	5.718950646	0.112356605	1.965856174	2	2	2	2	2	6.341987719	5.094391891
ENSG00000163527	STT3B	1.58782863	0.226367389	0.378020398	0.796658198	1.965849028	6.563806279	10.49332014	10.71094342	11.66752959	11.6300386	10.64709726	10.20151514
ENSG00000181852	RNF41	0.117366349	0.840490657	1.847778853	2.89E-05	1.965145203	8.816894989	8.077869079	9.083172337	8.793673851	8.8597051	10.51903133	10.57753611
ENSG00000111877	MCM9	0.867795267	0.519874318	1.097029585	0.393412229	1.964824852	5.70932728	5.838937808	2	6.104969671	6.01871653	6.146071859	6.400759785
ENSG00000131462	TUBG1	0.82391365	0.542787593	1.140854813	0.376931525	1.964768463	8.340423424	8.395519306	4.523675391	8.639384849	8.65866647	8.728933481	9.164631393
ENSG00000153012	LGI2	-1.303522209	0.792847991	3.268045143	0.44874768	1.964522934	2	2	2	2.270786764	2	4.088436536	3.407202941
ENSG00000008952	SEC62	1.263753667	0.000289255	0.699954298	0.058364332	1.963707965	9.957954117	9.599860728	10.27509734	11.22224576	11.2324146	10.73312834	10.59281363
ENSG00000107854	TNKS2	1.219327687	0.138060742	0.743923919	0.379855077	1.963251606	7.485143553	9.540933215	9.099922433	10.19294689	10.1158997	9.920984758	9.394150543
ENSG00000061936	SFSWAP	0.810995383	0.588403528	1.150583449	0.420944803	1.961578832	4.00722531	8.607278467	7.225742779	8.341783829	8.33301564	8.748160468	8.594208165
ENSG00000178401	DNAJC22	1.256708848	0.241959192	0.704386343	0.527535357	1.961095191	4.815609471	3.686303414	6.203069917	6.328984825	6.43421531	5.787450547	5.933281326
ENSG00000138709	LARP1B	0.301656791	0.804733172	1.6589647	0.106711001	1.960621491	7.53297911	6.228547981	4.523675391	6.931899482	6.76185814	8.228872122	8.120777053
ENSG00000172315	TP53RK	1.159928615	0.200892735	0.799585857	0.38602169	1.959514472	6.468621203	8.124485672	6.073378139	8.319579168	8.34936267	7.952415862	8.005636809
ENSG00000198959	TGM2	2.20590557	0.075674649	-0.246848989	0.866860327	1.95905658	11.46391134	9.328390744	8.007629717	12.46477506	12.4950495	10.54975054	9.215689211
ENSG00000175782	SLC35E3	0.916315489	0.53071634	1.042734412	0.461118553	1.959049901	4.00722531	6.464272742	8.076950877	7.852449262	7.85436579	7.301021256	8.436613692
ENSG00000197056	ZMYM1	1.099523608	0.355390531	0.858813637	0.472358061	1.958337245	3.327711234	6.311476754	6.203069917	6.892328675	6.73998187	6.975527754	6.052261256
ENSG00000101888	NXT2	1.328329736	0.499226274	0.626616888	0.760440198	1.954946624	3.327711234	6.728495673	2	6.640968371	6.53928196	5.966975997	5.884455123
ENSG00000165282	PIGO	0.805863993	0.669214488	1.14790757	0.523553954	1.953771563	2	7.099070191	8.206335842	7.930222906	8.01327646	8.275683409	8.342591622
ENSG00000103994	ZNF106	0.394527257	0.439157025	1.55790027	0.000319142	1.952427527	9.90440318	9.664384941	9.287079172	10.04007758	10.0277996	10.7983745	11.50464287
ENSG00000116688	MFN2	0.688286267	0.56825652	1.263252969	0.260527422	1.951539236	7.159102577	9.277729677	10.51254821	10.19090967	10.2462532	10.7395472	10.84303208
ENSG00000086062	B4GALT1	1.289695298	0.054670527	0.661768212	0.350940523	1.95146351	9.556468883	10.76380876	11.11279376	11.88411046	11.9216624	10.98658636	11.5167917
ENSG00000139233	LLPH	0.991728033	0.359332006	0.959003985	0.367118564	1.950732018	7.904282243	7.051611315	4.874587708	7.908072101	8.15434112	7.940525532	8.065268319
ENSG00000182952	HMGN4	0.957413978	0.000598253	0.992084101	0.000320664	1.949498079	8.75772933	8.487557258	8.536706852	9.569217728	9.52209753	9.508400079	9.648949641
ENSG00000155918	RAET1L	7.139715022	0.001475449	-5.191143944	0.075016235	1.948571078	2	2	2	7.044488801	7.35080119	2	2
ENSG00000174827	PDZK1	2.068138782	0.300919213	-0.120175854	0.958888695	1.947962928	2	2	4.058998049	4.268917409	4.40247105	2.885263063	2.950113106
ENSG00000171365	CLCN5	1.36186197	0.39960716	0.58568699	0.731331093	1.94754896	4.00722531	7.189546432	3.369317972	7.066878376	7.26532099	6.717615541	6.048572483
ENSG00000117620	SLC35A3	0.997748243	0.065697131	0.949021507	0.077151749	1.94676975	6.563806279	7.145017454	7.734844356	8.279879181	8.11451391	8.23926289	8.057947787
ENSG00000035681	NSMAF	1.484870452	0.022233888	0.461620545	0.523553954	1.946490997	8.16936288	7.928237109	6.534109959	9.18513223	9.16073939	8.103667822	8.214292363
ENSG00000084733	RAB10	0.818999057	0.156553121	1.126456341	0.040371077	1.945455399	8.777722034	9.506147172	10.06083273	10.37936391	10.3310092	10.73289857	10.58742133
ENSG00000243667	WDR92	1.203640099	0.032193991	0.741495279	0.202759883	1.945135378	5.330594134	6.140560021	5.59507254	6.917187326	6.88158913	6.497238296	6.412287469
ENSG00000101782	RIOK3	1.108915401	0.079057979	0.836136236	0.19081938	1.945051637	8.009475818	9.45234302	8.561105505	9.895579677	9.91822548	9.638721848	9.632103066
ENSG00000188626	GOLGA8M	7.135864817	0.001421933	-5.191144077	0.074313018	1.94472074	2	2	2	7.093295078	7.30311013	2	2
ENSG00000189079	ARID2	0.856583248	0.151636445	1.087275979	0.057300582	1.943859227	7.975256505	7.954278496	6.80318718	8.589222193	8.45217004	8.926322547	8.550537483
ENSG00000166200	COPS2	1.41352353	0.18647032	0.529580402	0.646525284	1.943103933	6.468621203	9.599860728	8.699419242	10.16623608	10.1248958	9.35261874	9.173973093
ENSG00000108828	VAT1	0.50873371	0.183786544	1.433190187	2.19E-05	1.941923896	10.92399098	11.23400805	10.66102425	11.45373986	11.4826289	12.20981764	12.5533032
ENSG00000012822	CALCOCO1	1.070995946	0.017885637	0.869858499	0.057854079	1.940854445	9.204317094	9.357954881	10.03493575	10.59575793	10.6909553	10.59077471	10.27951104
ENSG00000164403	SHROOM1	0.747042284	0.533299715	1.192917244	0.290333557	1.939959528	7.485143553	5.72254323	4.523675391	7.062428156	7.18258783	7.746573618	7.342293759
ENSG00000198046	ZNF667	0.634669213	0.704230942	1.303510691	0.397528734	1.938179904	6.138360429	7.051611315	2	6.716553866	6.73998187	7.53303322	7.20156326
ENSG00000102081	FMR1	1.464137511	0.009798993	0.471612016	0.455371809	1.935749526	7.031698319	8.101365653	8.408111533	9.433648866	9.37903917	8.352472895	8.489267468
ENSG00000198252	STYX	0.616957886	0.362270839	1.318679664	0.032017989	1.935637551	6.257048221	7.503546079	6.43197674	7.510853288	7.41778467	8.235375117	8.068003971
ENSG00000157741	UBN2	1.592560436	0.019814274	0.342926909	0.664743846	1.935487345	7.82963488	7.73146271	6.629488387	9.098672425	9.03162973	8.256629373	7.22299542
ENSG00000135547	HEY2	-4.140665176	0.065574814	6.075882984	4.15E-06	1.935127808	3.327711234	3.076665209	2	2.141737246	2	7.908330388	8.002780183
ENSG00000162378	ZYG11B	0.289297311	0.56778367	1.644152988	8.27E-05	1.933450299	8.540903468	8.191702463	9.049077186	8.899755849	8.93384535	10.39469335	10.12125705
ENSG00000131626	PPFIA1	1.086203592	0.06221272	0.845445916	0.15099294	1.931649508	8.816894989	9.479495904	8.143093325	10.05135008	9.94775169	9.706989438	9.812580902
ENSG00000161091	MFSD12	1.125340386	0.407755293	0.803652279	0.558480691	1.928992665	9.521325812	5.838937808	9.926432745	10.26878646	10.3666911	10.29565054	9.622520313
ENSG00000177700	POLR2L	0.684915144	0.477619809	1.243264183	0.162230124	1.928179327	9.98400217	8.376385542	8.042706615	9.676486519	9.81279854	9.812090132	10.66776684
ENSG00000023902	PLEKHO1	1.178515208	0.434235922	0.749169306	0.626611066	1.927684514	4.00722531	8.876041558	8.460932916	9.250166145	9.33957954	9.060185483	8.649438172
ENSG00000120159	CAAP1	0.625445844	0.528827603	1.302200348	0.148716503	1.927646192	5.70932728	7.393647515	5.39244166	7.144720234	7.01053089	7.57260403	7.889811111
ENSG00000116095	PLEKHA3	1.002506444	0.597757024	0.924203044	0.624146383	1.926709487	2	7.274681896	8.434763955	8.392303091	8.36552655	8.474034275	8.105768199
ENSG00000163762	TM4SF18	-1.310490463	0.711621313	3.23494391	0.2712184	1.924453448	2	2	2	2.141737246	2.14022021	3.411599105	4.047883918
ENSG00000126822	PLEKHG3	-1.29854033	0.47271232	3.222582404	0.045860622	1.924042074	4.467311984	8.276714465	3.369317972	5.651002631	5.58783529	9.835606948	10.19067811
ENSG00000128346	C22orf23	-2.398062482	0.152438771	4.31846721	0.002694218	1.920404728	2	4.93638933	2	2.498691189	2.59489879	7.87872738	7.331709059
ENSG00000144043	TEX261	0.806489411	0.129712892	1.113895105	0.028077653	1.920384516	9.613202746	8.862353853	8.699419242	9.879225887	9.95251543	9.950137104	10.45173765
ENSG00000168398	BDKRB2	5.27218442	0.000312209	-3.352415574	0.030925245	1.919768846	4.00722531	6.666836837	2	10.45727963	10.5479824	3.215043717	2.592996859
ENSG00000167005	NUDT21	0.988887806	0.237601833	0.928162853	0.262807198	1.917050659	8.87372952	8.671479029	6.80318718	9.393466757	9.29994502	9.391277746	9.174819346
ENSG00000165138	ANKS6	0.434458782	0.8692818	1.482258435	0.529629876	1.916717217	3.327711234	8.590771778	2	7.580980221	7.38644297	8.457998097	8.575745325
ENSG00000128482	RNF112	0.47893797	0.752212984	1.435786676	0.285392525	1.914724646	4.00722531	4.11352228	2	4.070463721	3.98554648	4.416072028	5.108633494
ENSG00000171604	CXXC5	-0.827870442	0.104928256	2.741504743	7.92E-10	1.913634301	8.836089686	8.916342327	7.97167868	7.920420067	7.71553728	11.19227235	11.52361706
ENSG00000187391	MAGI2	0.455316747	0.776973345	1.457370186	0.308265999	1.912686933	3.327711234	5.595927442	7.600047325	6.877205252	6.76727577	7.446000032	8.083408769
ENSG00000169762	TAPT1	1.352838821	0.387432577	0.559669833	0.735204809	1.912508655	8.285626337	5.303523747	4.058998049	8.27796118	8.240612	7.714348968	7.199901369
ENSG00000160867	FGFR4	-0.524557064	0.760985399	2.436999835	0.087028008	1.912442771	3.327711234	3.686303414	2	3.023133209	2.86123014	4.894909044	5.164234344
ENSG00000148218	ALAD	0.630803541	0.548066193	1.280744959	0.184467558	1.9115485	7.384433967	6.602424674	9.031722608	8.674801527	8.63070345	9.096715824	9.474014382
ENSG00000169439	SDC2	-1.265349062	0.163640595	3.176349494	8.57E-05	1.911000432	10.11549941	9.318399851	7.691296561	8.164192202	8.02236724	12.25879808	12.73497751
ENSG00000005893	LAMP2	1.349915776	9.47E-05	0.560298158	0.137293355	1.910213933	9.624286623	10.28338212	10.04361983	11.38590424	11.3294542	10.47794197	10.65645106
ENSG00000108960	MMD	0.718666891	0.723460473	1.191526712	0.535217469	1.910193603	4.00722531	7.051611315	2	6.490083648	6.22964624	7.210184325	6.280114403
ENSG00000121542	SEC22A	0.844751962	0.531143362	1.065371108	0.410154943	1.91012307	3.327711234	7.232742069	6.43197674	7.169761359	7.20671394	7.562295432	7.224630935
ENSG00000164776	PHKG1	0.923977962	0.687785088	0.985932074	0.660564083	1.909910036	2	2	4.058998049	3.462030052	3.55551834	3.753554863	3.311948089
ENSG00000167232	ZNF91	1.229232395	0.514736001	0.68003481	0.726284264	1.909267205	2	8.213429689	6.718950348	8.422134132	8.14812525	8.123435792	7.247335571

ENSG00000259207	ITGB3	2.603260998	0.348943914	-0.695657657	0.81957996	1.907603341	8.042902253	2	2	9.04103174	9.05396643	6.619606851	4.033026654
ENSG00000147364	FBXO25	0.254962654	0.900708025	1.652209824	0.344818335	1.907172478	7.904282243	5.838937808	2	6.820355514	6.95938211	8.159444235	8.350852333
ENSG00000133731	IMPA1	1.376960153	0.274972023	0.528356153	0.695554779	1.905316306	7.218819781	6.844429755	3.369317972	7.971157485	7.73502229	7.156371901	6.889403418
ENSG00000198382	UVRAG	0.775157547	0.388769665	1.129646397	0.18225392	1.904803945	6.257048221	8.255926468	6.80318718	8.093978021	8.18097132	8.437839892	8.53677936
ENSG00000122574	WIPF3	-2.983882974	0.213019329	4.888341312	0.014755667	1.904458338	2	4.11352228	2	2.3892346	2	7.747483829	6.371531035
ENSG00000149658	YTHDF1	0.645889421	0.395163317	1.257406922	0.071096291	1.903296343	8.19931886	9.159587375	7.451345288	9.013631184	9.15558431	9.755980824	9.629636184
ENSG00000074266	EED	1.39183833	0.112243988	0.510679182	0.590583035	1.902517512	6.009025997	6.728495673	4.523675391	7.288834728	7.45518898	6.763331418	6.232137896
ENSG00000204116	CHIC1	1.146306395	0.47537973	0.755853802	0.643465101	1.902160198	2	7.002537973	6.073378139	7.261917051	7.06433609	6.833591108	6.741885308
ENSG00000174292	TNK1	0.442652377	0.840490657	1.45890352	0.451396209	1.901555897	2	4.11352228	2	3.515101962	3.33976443	4.301748724	3.972018587
ENSG00000139436	GIT2	1.479890151	0.03929762	0.421583223	0.599804676	1.901473375	8.107517749	8.234834549	6.43197674	9.287824288	9.23006009	8.201234858	8.220862755
ENSG00000106591	MRPL32	0.934484227	0.115211747	0.965949127	0.09634677	1.900433354	6.816637977	8.004992147	7.163363831	8.347282003	8.35656898	8.514698983	8.237969002
ENSG00000067208	EVI5	0.541599525	0.105778838	1.358821482	5.09E-06	1.900421057	7.975256505	7.874700259	8.007629717	8.496596021	8.47221251	9.177956973	9.402844548
ENSG00000089048	ESF1	1.532364861	0.023843846	0.367426197	0.635038067	1.899791058	7.384433967	7.954278496	6.203069917	8.808365552	8.93143442	7.79139499	7.647978997
ENSG00000181472	ZBTB2	1.474471667	0.023668321	0.425033011	0.560994904	1.899504678	7.624139768	7.232742069	5.930865648	8.568972129	8.51631523	7.481445808	7.537835213
ENSG00000117010	ZNF684	0.301503568	0.896917828	1.597398189	0.428364332	1.898901757	2	5.131603625	2	4.205751501	4.05806467	5.54934408	4.793076545
ENSG00000162772	ATF3	1.146381957	0.182039968	0.751165571	0.392356999	1.897547528	8.653385977	7.189546432	6.80318718	8.912174893	8.90955283	8.815677424	8.146025444
ENSG00000059728	MXD1	1.168038975	0.012450706	0.729195801	0.133193465	1.897234775	6.366709671	7.145017454	7.225742779	8.136938566	8.08010078	7.783429229	7.562617572
ENSG00000082458	DLG3	0.951392095	0.431799068	0.943715856	0.426131057	1.895107951	4.815609471	7.431218269	5.156626401	7.292639438	7.18663697	7.411959578	7.028854452
ENSG00000157764	BRAF	-0.136285158	0.891774766	2.029000629	0.009269015	1.892715471	6.138360429	8.029695761	6.534109959	6.975156635	7.08183429	9.154129994	9.165908829
ENSG00000153214	TMEM87B	1.669242437	0.395924587	0.221821638	0.920635926	1.891064075	9.33149386	7.73146271	2	9.797905134	9.84133177	8.541185654	8.206037069
ENSG00000126759	CFP	2.013763328	NA	-0.124892997	NA	1.888870332	2	2	2	3.227757282	2.77779907	2.42098416	2.172608095
ENSG00000165934	CPSF2	0.716330331	0.228106188	1.172438209	0.035030931	1.888768539	8.009475818	8.639735838	9.287079172	9.471064221	9.42346554	10.02767645	9.762795477
ENSG00000163781	TOPBP1	0.916676303	0.632871056	0.971521755	0.605992978	1.888198059	3.327711234	8.639735838	4.874587708	8.160032699	8.0314011	8.247650629	8.047821014
ENSG00000164292	RHOBTB3	0.768906019	0.378155417	1.117254418	0.175036042	1.886160437	9.247966931	9.079034257	7.398165132	9.583264843	9.50436385	10.27812044	9.36032955
ENSG00000148908	RGS10	1.281865026	0.505521211	0.601590356	0.764974927	1.883455382	7.750913381	4.93638933	2	7.694616532	7.53675633	6.877490957	7.028854452
ENSG00000204352	C9orf129	7.073258558	0.001861972	-5.191143651	0.077151749	1.882114907	2	2	2	7.169761359	7.11195456	2	2
ENSG00000161940	BCL6B	0.897611828	0.725904626	0.981416451	0.692863851	1.879028279	3.327711234	2	2	2.947924294	2.94009902	3.10571689	2.851942824
ENSG00000163704	PRRT3	0.450554277	0.790303792	1.428319054	0.343471869	1.878873332	3.327711234	7.002537973	4.058998049	6.096288482	6.18176397	6.913621294	7.22299542
ENSG00000101150	TPD52L2	0.336044484	0.691501477	1.540480633	0.032896134	1.876525118	10.7426185	9.849218119	8.845626171	10.31300956	10.3782441	11.51506069	11.58004535
ENSG00000118900	UBN1	0.500765632	0.731288333	1.37548542	0.295571175	1.876251051	5.532353208	8.791896851	9.849211147	9.36574218	9.38433737	10.34534549	10.13892798
ENSG00000273802	HIST1H2BG	3.745268006	0.055746187	-1.870021459	0.368897909	1.875246547	6.009025997	2	2	8.078634414	8.08010078	2.987421008	3.45257121
ENSG00000026025	VIM	1.37811376	0.036049949	0.493564062	0.492815255	1.871677822	13.96181853	13.49758156	12.33487604	14.79555473	14.7831527	14.00582069	13.79656997
ENSG00000106701	FSD1L	1.318257443	0.292677052	0.553037321	0.67823435	1.871294764	2	4.93638933	5.59507254	6.078767801	5.86549842	5.180212365	5.361470313
ENSG00000100949	RABGGTA	1.127620498	0.5253082	0.743084337	0.681279661	1.870704835	7.624139768	7.701025996	2	8.126317766	8.27144011	7.47156439	8.103992141
ENSG00000153006	SREK1IP1	0.893284845	0.036051738	0.976842785	0.019287415	1.87012763	7.33131396	7.790474954	7.502634626	8.463225341	8.41985547	8.729854886	8.284187525
ENSG00000145358	DDIT4L	3.938628018	0.082799505	-2.06871339	0.388256033	1.869914628	4.815609471	4.11352228	2	7.632926745	7.60187978	3.316666208	2
ENSG00000061987	MON2	1.012320805	0.085477895	0.857590652	0.144697175	1.869911457	7.867441304	8.981105197	7.896971941	9.352133347	9.36927498	9.231960665	9.181571564
ENSG00000275031		4.513142663	1.55E-14	-2.64647408	1.87E-05	1.866668583	5.70932728	6.844429755	6.203069917	10.77167208	10.8115546	3.648325314	4.341135913
ENSG00000203772	SPRN	1.358325998	0.552657351	0.503713332	0.836549691	1.86203933	2	5.838937808	2	5.786686927	5.55061739	5.318934974	4.329022995
ENSG00000171295	ZNF440	1.234180521	0.304198642	0.627763435	0.616846858	1.861943956	4.00722531	6.951736442	5.156626401	7.119236776	7.03319408	6.736076089	6.199243628
ENSG00000178093	TSSK6	0.669735175	0.759294561	1.192175293	0.561301735	1.861910467	2	4.710564154	2	4.105493497	4.02226117	4.834511823	4.002845082
ENSG00000166262	FAM227B	0.16797141	0.929751797	1.693022808	0.28326297	1.860994218	3.327711234	3.686303414	2	3.162721474	3.45167086	4.644324387	4.105828626
ENSG00000041880	PARP3	1.240763293	0.03191533	0.620188936	0.311844973	1.860952229	7.790810999	6.602424674	7.691296561	8.676258519	8.64254348	7.861174222	8.210995912
ENSG00000124523	SIRT5	0.911756411	0.467848857	0.948519486	0.43787928	1.860275897	3.327711234	6.844429755	5.930865648	6.872128674	6.80989959	6.99251878	6.750993196
ENSG00000120705	ETF1	0.816984245	0.434235922	1.041983247	0.296112508	1.858967492	8.009475818	9.338312924	10.71094342	10.58610178	10.53732	10.71985653	10.84993646
ENSG00000135655	USP15	1.391776388	0.002659741	0.467143382	0.364515211	1.85891977	8.631580145	8.414402626	7.600047325	9.696010185	9.62936766	8.812636523	8.673588256
ENSG00000072415	MPP5	0.981657857	0.167897149	0.876533899	0.214740154	1.858191756	7.27616306	8.317412869	6.718950348	8.637890117	8.50824913	8.416251718	8.522888769
ENSG00000114742	WDR48	1.063363453	0.292532122	0.794205202	0.435384719	1.857568654	8.468948322	9.102512015	6.322058544	9.42153757	9.41227044	9.176605981	9.121828633
ENSG00000215126	CBWD6	4.588257983	8.16E-09	-2.731257175	0.001820976	1.857000808	2	4.710564154	5.156626401	8.729158235	8.82635531	2.717032197	2.782576693
ENSG00000123374	CDK2	0.676552374	0.717263579	1.179439315	0.501916413	1.855991689	2	7.431218269	8.007629717	7.885575888	7.78928606	8.243140215	8.418864688
ENSG00000177963	RIC8A	0.420657521	0.465054573	1.431573445	0.003946525	1.852230966	9.790941832	10.07961649	8.99637432	10.08135305	10.1427213	10.92445873	11.29335049
ENSG00000197601	FAR1	0.368739111	0.800325517	1.483389554	0.242330228	1.852128666	8.16936288	8.590771778	4.523675391	8.297027403	8.15227214	9.364533261	9.29605726
ENSG00000125755	SYMPK	1.201233202	0.153219079	0.649476341	0.459476825	1.850709543	7.750913381	7.979858146	9.61300511	9.854985705	9.93095363	9.341810888	9.34725177
ENSG00000165288	BRWD3	1.081867233	0.062696835	0.768449349	0.194623596	1.850316582	7.579279349	6.78762695	7.858115942	8.537252353	8.51631523	8.48499076	7.887748062
ENSG00000136827	TOR1A	1.291833851	0.000202427	0.554693466	0.142226777	1.846527317	8.963744667	9.102512015	8.585098385	10.18835911	10.1903833	9.267612236	9.621899879
ENSG00000196507	TCEAL3	0.140279331	0.896732205	1.705732919	0.04836641	1.84601225	8.892187472	6.535001748	7.858115942	8.229163214	8.15227214	9.531042494	9.928756347
ENSG00000142208	AKT1	1.268821073	0.412971533	0.5765566	0.723575557	1.845377674	10.27168932	10.23108619	5.156626401	10.90533885	10.9990778	10.10357235	10.40562199
ENSG00000120594	PLXDC2	0.405505548	0.609032994	1.439793986	0.035847057	1.845299534	6.366709671	6.728495673	6.582775709	6.941624891	7.18258783	8.573367846	7.321046127
ENSG00000196876	SCN8A	2.716539415	0.066281011	-0.871937728	0.593116818	1.844601686	2	7.315436877	6.534109959	9.088859521	9.07487069	5.630526941	5.581648407
ENSG00000070614	NDST1	0.563622702	0.546572519	1.279032617	0.1322507	1.84265532	9.913467515	9.785372135	11.61913434	11.22573252	11.2975154	11.64651719	12.24498338
ENSG00000167721	TSR1	1.040336636	0.098946169	0.802305742	0.207181946	1.842642377	8.674867122	8.732943944	7.342949392	9.399654088	9.42518018	9.08740002	9.259427717
ENSG00000167333	TRIM68	0.611503726	0.748108458	1.23101484	0.487308062	1.842518566	2	7.393647515	5.39244166	6.622948477	6.87658492	7.711553069	6.883203642
ENSG00000078674	PCM1	1.020512758	0.147002974	0.821753161	0.245421661	1.842265919	7.750913381	9.396454291	9.031722608	9.886167436	9.90783885	9.876537138	9.496865493
ENSG00000148158	SNX30	0.856229675	0.434435575	0.982663381	0.352922604	1.838893056	8.042902253	7.928237109	5.156626401	8.381625466	8.31092742	8.377971121	8.559637211
ENSG00000172466	ZNF24	0.954516605	0.522605424	0.881998629	0.548117081	1.836515234	5.095959458	9.514922632	9.636156974	10.01553929	9.93035007	10.20180989	9.521357751
ENSG00000155252	PI4K2A	1.313290143	0.24500653	0.522853577	0.667109583	1.83614372	6.73718411	8.777382925	10.01740919	10.33623673	10.3599836	9.342112202	9.750927434
ENSG00000100068	LRP5L	1.65163554	0.437566006	0.184099464	0.93862683	1.835735004	5.095959458	2	2	5.391962469	4.79082474	3.981090143	3.858622973
ENSG00000141458	NPC1	1.585602776	0.117971256	0.249171742	0.832879767	1.834774518	7.904282243	8.147241016	10.30429804	10.77371523	10.8171157	9.534737983	9.389783813
ENSG00000101955	SRPX	1.549342349	0.478179842	0.285076922	0.906201712	1.834419271	3.327711234	9.125613817	3.369317972	9.141495458	9.11260313	7.79139499	7.958255135
ENSG00000184828	ZBTB7C	-1.303410978	0.802441015	3.137816698	0.491297476	1.834405719	2	2	2	2.270786764	2	4.199031499	2.918126211
ENSG00000138495	COX17	1.142188102	0.051185896	0.69196198	0.254317355	1.834150083	6.009025997	6.140560021	5.156626401	6.951285179	6.92586261	6.375345632	6.62538071
ENSG00000105982	RNF32	0.223521848	0.933881218	1.609739051	0.493182797	1.833260899	2	3.686303414	2	2.600425781	3.28037825	4.188345803	3.33635788
ENSG00000266412	NCOA4	1.067192167	0.04337239	0.76570091	0.155705957	1.832893077	9.590776211	10.76012279	10.18377222	11.3151886	11.330827	10.93098358	11.10773798
ENSG00000197841	ZNF181	1.243468323	0.436019907	0.587680084	0.724656426	1.831148406	6.366709671	6.728495673	2	7.19843704	7.21466714	7.02591204	5.909074772
ENSG00000077254	USP33	1.375837802	1.30E-05	0.454481203	0.195994401	1.830319004	8.564112515	8.522788974	8.960138144	10.01783961	10.0977376	9.242329292	9.033614226
ENSG00000097021	ACOT7	1.051644237	0.525295603	0.776008956	0.641507692	1.827653193	4.815609471	6.78762695	9.624627483	9.275380474	9.34864288	8.621823729	9.361816632
ENSG00000137745	MMP13	7.017586499	0.002488209	-5.191143654	0.081042066	1.826443445	2	2	2	7.003290579	7.16627666	2	2
ENSG00000219545	UMAD1	1.02232181	0.538701364	0.802901589	0.632223509	1.8252234	4.815609471	6.666836837	2	6.408278948	6.51372389	6.004008792	6.465820193
ENSG00000061273	HDAC7	0.393558994	0.438437558	1.431177274	0.000991504	1.824736268	9.544849385	9.514922632	8.65477198	9.696728234	9.67763759	10.66370661	10.77657437
ENSG00000109920	FNBP4	1.11092149	0.279628699	0.710802317	0.498871333	1.821723807	8.737455672	9.148351514	6.322058544	9.568433308	9.61215953	9.252303334	9.130578617
ENSG00000054392	HHAT	0.826428048	0.686228546	0.995024488	0.615427844	1.821452536	3.327711234	6.666836837	2	6.078767801	6.03677338	5.979426183	6.420873184
ENSG00000176248	ANAPC2	0.728187609	0.089730458	1.091346087	0.006751128	1.819533696	7.709880939	7.002537973	7.451345288	8.054188409	8.17894021	8.455215133	8.497408326
ENSG00000151491	EPS8	-0.517095951	0.53177344	2.336033001	0.000833175	1.81893705	8.075571721	9.079034257	9.999667074	8.70652031	8.77618319	11.48201426	11.67783059
ENSG00000106624	AEBP1	-0.323160872	0.682951928	2.141384438	0.000909649	1.818223566	10.45046729	11.26034006	12.15421689	11.10489064	11.1547085	13.55226621	13.63154322
ENSG00000124493	GRM4	-0.727756714	0.851528683	2.545746269	NA	1.817989554	2	2	2	2.141737246	2.2680111	3.215043717	3.407202941
ENSG00000049618	ARID1B	0.941019489	0.495694274	0.87529216	0.521153279	1.81631165	5.532353208	9.018611864	9.798878489	9.836208668	9.8787275	9.846692337	9.735523645
ENSG00000089597	GANAB	0.930074645	0.154687626	0.885354353	0.169698897	1.815428998	10.80239439	11.36354124	12.2511264	12.50345529	12.5485341	12.32267771	12.62452591
ENSG00000165271	NOL6	0.80968831	0.514865066	1.005326195	0.398667475	1.815014505	5.330594134	8.540087354	8.784770914	8.934263273	8.97422645	9.007209385	9.277267848
ENSG00000118217	ATF6	0.71037993	0.097618305	1.102962958	0.006105215	1.813342888	8.87372952	9.04308532	9.647595056	9.963384909	9.89861166	10.27796296	10.36578622
ENSG00000259332	ST20-MTHFS	7.004362544	0.002590896	-5.191143003	0.081460409	1.813219541	2	2	2	7.044488801	7.10341274	2	2
ENSG00000138092	CENPO	1.218954499	0.403701418	0.59347817	0.696976163	1.812432669	6.563806279	5.946638857	2	6.912249745	6.89154576	6.173421703	6.420873184
ENSG00000171786	NHLH1	2.060110941	0.313991908	-0.247936522	0.916470603	1.812174419	2	3.686303414	2	4.070463721	4.2865223	2.62493832	2.918126211
ENSG00000164880	INTS1	0.602137651	0.671105744	1.207522821	0.356353281	1.809660472	10.57565445	7.761270542	7.225742779	9.851765671	9.94536391	10.46278097	10.54141057
ENSG00000170004	CHD3	0.514153223	0.544293669	1.292569277	0.090878546	1.8067225	8.855032352	10.52407288	10.45512158	10.59498781	10.6715842	10.9230495	11.77450991
ENSG00000144426	NBEAL1	0.84954597	0.365703512	0.955819972	0.293820393	1.805365942	8.816894989	7.232742069	6.718950348	8.673343061	8.75724985	9.029457273	8.577666268
ENSG00000196961	AP2A1	0.60874537	0.292553183	1.195945937	0.024032158	1.804691306	10.1076228	10.03760521	8.978369997	10.38650225	10.4132224	10.8706474	11.09225624
ENSG00000106086	PLEKHA8	0.947618085	0.037226271	0.855901662	0.059084307	1.803519748	6.96351253	7.701025996	7.502634626	8.370868225	8.35297033	8.439530542	8.8007022
ENSG00000114423	CBLB	1.85259731	0.095126919	-0.05021032	0.97020102	1.802386991	8.631580145	6.78762695	5.59507254	9.389030931	9.3304589	7.743839536	7.159430626
ENSG00000155729	KCTD18	0.388618136	0.833540602	1.412914998	0.384828556	1.801533134	7.579279349	6.666836837	2	7.080147179	6.92586261	7.886185273	8.117259579
ENSG00000103353	UBFD1	0.632787121	0.632327358	1.168045701	0.343161384	1.800832823	6.653098118	10.47544827	9.636156974	10.21917213	10.2340777	10.63251333	10.87787797
ENSG00000113790	EHHADH	-0.12596852	0.934841508	1.925824172	0.12649658	1.799855652	2	5.946638857	5.156626401	4.846787657	5.13053105	6.940933331	6.864442837
ENSG00000183421	RIPK4	-2.654230762	0.18763416	4.453225096	0.010530438	1.798994334	4.815609471	2	2	2.600425781	2.14022021	7.309674888	7.570355956
ENSG00000111790	FGFR1OP2	0.952285579	0.459782955	0.846439653	0.505528206	1.798725233	8.257427002	7.572420273	4.523675391	8.404661252	8.34212018	8.548515606	7.922424886
ENSG00000164649	CDCA7L	0.663722755	0.66611095	1.133013237	0.431244286	1.796735992	4.00722531	5.595927442	2	5.258265913	5.00774437	5.622611168	5.497565112
ENSG00000149451	ADAM33	1.691772579	0.261480902	0.101184304	0.954416752	1.792956883	6.96351253	6.666836837	2	7.818544084	7.99261031	6.221381643	6.495928492
ENSG00000088002	SULT2B1	-1.303475717	0.796759656	3.096171555	0.484010617	1.792695839	2	2	2	2.270786764	2	3.694366604	3.640730416
ENSG00000163219	ARHGAP25	-2.312443603	0.573719152	4.104630145	0.221372771	1.792186542	2	2	2	2	2.14022021	4.727866721	4.047883918

ENSG00000166734	CASC4	1.117142762	0.404897934	0.674978336	0.624569223	1.792121098	6.138360429	10.13758676	10.08627303	10.70507197	10.6668817	10.31450651	10.17236288
ENSG00000178467	P4HTM	0.835469199	0.586697428	0.955241852	0.522645705	1.790711051	7.435667355	8.876041558	4.058998049	8.649804831	8.57000035	8.663891891	8.791384617
ENSG00000243056	EIF4EBP3	3.423983486	0.03057436	-1.633377028	0.345260656	1.790606458	2	2	4.523675391	6.12217689	5.99120031	2.560105256	2.551896972
ENSG00000018610	CXorf56	0.982000291	0.094108586	0.808457617	0.167430714	1.790457908	6.563806279	6.951736442	5.772716771	7.437142666	7.5240115	7.454943454	7.152573734
ENSG00000171456	ASXL1	0.09154634	0.899933851	1.697561314	0.002384213	1.789107654	8.444143041	9.470501606	9.819251342	9.392580682	9.49296422	10.95846335	11.12491757
ENSG00000103381	CPPED1	0.718096036	0.370967351	1.070861701	0.15655242	1.788957737	8.609439662	8.213429689	6.718950348	8.693628619	8.81985255	8.904045868	9.286693143
ENSG00000011275	RNF216	0.177734422	0.711059078	1.610582106	2.28E-05	1.788316527	8.963744667	9.632483534	8.960138144	9.437090644	9.37105521	10.78558135	10.87683825
ENSG00000112282	MED23	1.332867249	0.300898696	0.455089935	0.742539931	1.787957183	5.70932728	9.018611864	6.534109959	9.115954467	9.11366683	8.385007206	8.091498142
ENSG00000141469	SLC14A1	1.106584767	0.581814479	0.680112932	0.738292369	1.786697699	3.327711234	2	3.369317972	3.515101962	3.69849104	3.600766115	3.012034821
ENSG00000138778	CENPE	0.910755563	0.40768846	0.875539234	0.417447017	1.786294797	4.467311984	6.04685525	4.058998049	6.034011955	5.98191036	6.305369896	5.545463418
ENSG00000256229	ZNF486	0.895284273	0.284862917	0.890650422	0.275628117	1.785934695	5.70932728	4.11352228	5.59507254	6.034011955	6.12381559	6.170709933	5.97274432
ENSG00000103855	CD276	1.373925565	0.236772262	0.411915236	0.745570382	1.785840802	11.55650736	10.88722087	8.076950877	12.10263209	12.1465732	10.92697178	11.36748936
ENSG00000131899	LLGL1	1.010608601	0.467936122	0.772715614	0.579833528	1.783324215	8.653385977	4.93638933	9.132850258	9.389919188	9.35225233	8.939525293	9.306905794
ENSG00000178163	ZNF518B	0.690272654	0.743410913	1.092911278	0.586432904	1.783183932	2	6.844429755	8.960138144	8.321442665	8.40081078	8.990775914	8.485179747
ENSG00000182324	KCNJ14	0.763828567	0.734475554	1.019185924	0.638604205	1.783014492	2	4.710564154	2	3.840289928	4.37434574	4.73522696	3.771796761
ENSG00000077984	CST7	-0.314243794	NA	2.096937505	NA	1.782693711	2	2	2	2.141737246	2.38539787	3.648325314	2.059860178
ENSG00000141556	TBCD	1.187051235	0.415814926	0.592309949	0.696686193	1.779361183	4.00722531	8.255926468	6.203069917	8.174538791	8.2561084	7.440382108	7.799420735
ENSG00000131100	ATP6V1E1	1.204763605	0.23034597	0.573544063	0.58833395	1.778307668	10.59268348	9.102512015	7.934808822	10.76928475	10.8430013	10.05624005	10.28872631
ENSG00000146776	ATXN7L1	1.570127391	0.281556878	0.206575743	0.901842972	1.776703134	2	5.72254323	6.629488387	7.153115662	7.22651534	5.801542492	5.968846243
ENSG00000161835	GRASP	-0.436094991	0.85051447	2.210299884	0.260738589	1.774204893	2	5.303523747	2	3.515101962	3.90918929	6.320128742	5.448021784
ENSG00000256294	ZNF225	0.939272193	0.405878812	0.833655425	0.456352327	1.772927618	3.327711234	6.311476754	6.322058544	6.676346669	6.76185814	6.613628927	6.622902297
ENSG00000139437	TCHP	0.675931733	0.695919199	1.095981466	0.501628498	1.771913199	6.653098118	7.393647515	2	7.214570819	7.1204461	7.555035333	7.600900977
ENSG00000205189	ZBTB10	0.307868617	0.896153568	1.463770994	0.480226627	1.771639611	4.467311984	8.213429689	2	7.114945424	6.99676009	8.397818242	7.944426693
ENSG00000170946	DNAJC24	1.190666075	0.196009997	0.580646402	0.548363823	1.771312477	7.709880939	5.946638857	5.772716771	8.006336225	7.81828345	7.493431902	7.117791742
ENSG00000117335	CD46	1.116896131	0.153513211	0.65199915	0.422109347	1.768895281	8.340423424	10.28338212	9.888372618	10.82814175	10.8281739	10.50833129	10.20462614
ENSG00000122203	KIAA1191	0.610587974	0.12465991	1.156764709	0.001542477	1.767352683	9.689059027	10.12617756	10.42220168	10.71786226	10.7188341	11.19786031	11.32619054
ENSG00000125733	TRIP10	0.516524689	0.205222597	1.249961086	0.000591862	1.766485775	8.493334308	8.053983481	8.511888463	8.839910123	8.90341524	9.776191646	9.400314204
ENSG00000126870	WDR60	1.093607353	0.538070994	0.671489612	0.713730905	1.765096965	2	7.503546079	7.858115942	8.190940526	8.19510963	7.839995266	7.712342256
ENSG00000125827	TMX4	1.38302151	0.085117333	0.382050982	0.670218628	1.765072492	9.384607364	9.018611864	7.342949392	10.16882602	10.2109057	8.94032159	9.409690338
ENSG00000137992	DBT	0.620825829	0.525902595	1.144035889	0.204625268	1.764861718	6.138360429	8.124485672	8.175061096	8.409925372	8.28283292	9.272046904	8.297458414
ENSG00000150764	DIXDC1	0.264639182	0.863874307	1.499903342	0.252589462	1.764542524	4.467311984	8.124485672	8.763901704	8.197043419	8.20112674	9.526807454	9.311145499
ENSG00000155428	TRIM74	3.210583301	0.267665409	-1.447786938	0.649857772	1.762796363	2	2	2	3.406930997	4.02226117	2.139668783	2.117335275
ENSG00000013016	EHD3	-0.721653629	0.118548292	2.484356572	6.37E-10	1.762702943	8.564112515	8.806266215	7.896971941	7.739909082	7.79460185	10.87554743	11.02027932
ENSG00000141580	WDR45B	0.508091864	0.137498142	1.254144501	4.02E-05	1.762236365	9.204317094	9.479495904	9.681375057	9.985296505	9.96140527	10.58403908	10.8390337
ENSG00000125741	OPA3	0.286778506	0.766720309	1.474914211	0.072952984	1.761692717	6.563806279	8.297207175	8.763901704	8.408172799	8.43013848	9.354113447	9.806314352
ENSG00000104442	ARMC1	0.752600093	0.69344525	1.008036289	0.582641607	1.760636383	2	8.053983481	8.076950877	8.285617933	8.17690623	8.655672796	8.290447859
ENSG00000110315	RNF141	-0.88909083	0.271602878	2.645844078	0.000180075	1.756753248	6.73718411	8.623598428	8.585098385	7.370304568	7.29937545	10.72829538	10.93843053
ENSG00000166847	DCTN5	0.579669376	0.433038514	1.176988842	0.080856643	1.756658218	10.18451821	8.522788974	9.372276026	10.06197797	10.1153688	10.69341333	10.67541118
ENSG00000158220	ESYT3	-1.317468484	0.795427588	3.07358351	0.489950838	1.756115026	2	2	2	2	2.2680111	3.810410249	3.474731315
ENSG00000116954	RRAGC	1.767001526	0.000538028	-0.011626713	0.986196197	1.755374813	8.418903792	7.274681896	8.236946975	9.875425464	9.71666438	8.120628326	7.944426693
ENSG00000136720	HS6ST1	0.468928341	0.821640487	1.285900093	0.493153856	1.754828434	2	6.728495673	8.511888463	7.756533788	7.77052502	8.373850835	8.740849281
ENSG00000088826	SMOX	3.503687942	0.000676627	-1.748885556	0.114259051	1.754802386	8.19931886	6.728495673	4.874587708	10.63936224	10.6126122	5.758846871	5.191251321
ENSG00000138802	SEC24B	1.100646987	0.215813666	0.64853403	0.480834472	1.749181018	6.96351253	9.277729677	8.325066185	9.555032495	9.58633944	9.245876366	8.985229012
ENSG00000168994	PXDC1	-0.084593405	0.942177035	1.833205535	0.051887684	1.74861213	10.13887448	8.540087354	7.734844356	9.031955922	9.11153864	11.34267419	10.5004808
ENSG00000230797	YY2	0.404071721	0.854219249	1.344103576	0.489342742	1.748175297	2	4.442710129	2	3.79808662	3.50452879	4.504874511	4.201948172
ENSG00000117280	RAB29	0.523644412	0.718375041	1.224443382	0.357283417	1.748087794	8.564112515	4.710564154	8.805342542	8.652768197	8.673169	9.284015592	9.428971287
ENSG00000100280	AP1B1	0.481718746	0.686208373	1.266252281	0.237913947	1.747971027	9.810482605	9.74864196	6.882775709	9.769519169	9.76891184	10.207113	10.82845131
ENSG00000163536	SERPINI1	2.337666297	0.22692986	-0.590148978	0.784860167	1.747517318	2	5.457120953	2	6.113598934	6.32824132	3.838019203	3.620995423
ENSG00000140265	ZSCAN29	1.063806919	0.153169017	0.682172042	0.370890738	1.745978961	6.138360429	7.928237109	7.451345288	8.369067528	8.43865202	8.202562993	7.828751338
ENSG00000213079	SCAF8	1.04791842	0.018176036	0.695769467	0.128956336	1.743687887	7.53297911	8.147241016	8.486635645	9.139384228	9.14833612	8.811331315	8.777523662
ENSG00000126890	CTAG2	-0.7277976	0.857804601	2.470474711	NA	1.74267711	2	2	2	2.141737246	2.2680111	3.632645806	2.782576693
ENSG00000164087	POC1A	0.625574074	0.78979414	1.1166413	0.611923598	1.742215374	2	5.595927442	2	4.497095146	5.04390545	5.201624686	5.250250853
ENSG00000275594		2.898265153	0.334766555	-1.156717004	0.725350511	1.741548149	2	2	2	3.462030052	3.60476711	2.139668783	2.172608095
ENSG00000185189	NRBP2	0.764531662	0.53122754	0.976961841	0.406566361	1.741493503	7.27616306	7.051611315	9.778213818	9.316137634	9.39662469	9.540526122	9.595281739
ENSG00000118513	MYB	2.332284341	0.686502469	-0.591060112	NA	1.741224229	2	2	2	3.09461492	3.28037825	2	2.421067459
ENSG00000185619	PCGF3	0.919994104	0.064504706	0.820482349	0.098450928	1.740476453	9.304185368	8.395519306	8.434763955	9.667723798	9.70471389	9.614979403	9.559117299
ENSG00000100350	FOXRED2	0.609720413	0.521312957	1.129613943	0.195650955	1.739334356	5.095959458	7.35507212	6.629488387	7.300218887	7.16627666	7.511762185	7.942440336
ENSG00000172845	SP3	0.789041043	0.399039732	0.949491019	0.291402385	1.738532062	7.27616306	9.246456519	9.61300511	9.818504147	9.76823652	10.12491852	9.758568089
ENSG00000112902	SEMA5A	-0.124722214	0.895675696	1.862852672	0.012605537	1.738130459	9.710019321	10.00683004	11.25418149	10.36090904	10.3649055	11.74494028	12.77082042
ENSG00000198561	CTNND1	0.47580392	0.621621222	1.261915956	0.144252411	1.737719877	8.836089686	10.56267266	11.19694409	10.96377665	10.9670671	11.63500472	11.85709766
ENSG00000075089	ACTR6	1.461293785	0.047051348	0.276378319	0.744221494	1.737672104	6.563806279	6.389896633	4.874587708	7.574744049	7.4949172	6.635427934	6.092227206
ENSG00000153558	FBXL2	0.230000872	0.923810945	1.506606659	0.475075841	1.736607531	2	7.503546079	3.369317972	6.18083559	6.33555364	7.417689057	7.562617572
ENSG00000133812	SBF2	0.607126667	0.700598482	1.129318381	0.443373306	1.736445049	4.00722531	8.732943944	8.65477198	8.786946023	8.68895519	9.376644377	9.126210259
ENSG00000085185	BCORL1	0.146895616	0.913792484	1.589222878	0.154026394	1.736118495	4.815609471	7.979858146	8.175061096	7.668500375	7.75969386	9.120448319	9.158655025
ENSG00000196757	ZNF700	1.130140196	0.478107313	0.603010284	0.715036782	1.733150479	2	6.899080544	6.322058544	7.161462517	7.19067478	6.874161066	6.423723772
ENSG00000166086	JAM3	0.597375461	0.577257973	1.133358851	0.25426536	1.730734312	6.96351253	9.170736405	9.624627483	9.522191343	9.57788596	9.692174376	10.39167686
ENSG00000135679	MDM2	1.52073751	5.48E-06	0.209918677	0.605467095	1.730656186	9.602033055	9.849218119	9.849281147	11.29399181	11.283169	10.22682784	9.684739879
ENSG00000186017	ZNF566	0.731332716	0.398162916	0.999300884	0.222928155	1.730633601	4.815609471	6.464272742	5.772716771	6.640968371	6.45450701	7.121690475	6.406535141
ENSG00000164576	SAP30L	0.575839456	0.194620623	1.154539342	0.004283896	1.730378799	8.285626337	7.819099893	8.007629717	8.583021623	8.64401667	9.419535622	8.91098986
ENSG00000123636	BAZ2B	1.475147749	0.088652437	0.250241491	0.802048583	1.725389239	6.138360429	8.414402626	8.007629717	9.219515356	9.30925999	8.271892681	7.79392309
ENSG00000113812	ACTR8	1.148191606	0.48962595	0.576879331	0.737950893	1.725070938	8.797441463	6.844429755	3.369317972	8.734762645	8.66593596	8.2308261	8.034828728
ENSG00000185344	ATP6V0A2	0.826886042	0.568597763	0.898042806	0.526566702	1.724928848	5.330594134	8.540087354	5.39244166	8.054188409	8.07574085	8.261097876	7.997049907
ENSG00000122257	RBBP6	1.017467377	0.095026872	0.70712894	0.255721676	1.724596316	8.517314938	8.522788974	9.670202709	9.970527901	10.0678647	9.799857373	9.61630394
ENSG00000066084	DIP2B	1.331288503	0.509240304	0.391357872	0.856534247	1.722646375	2	9.298208073	9.06622548	9.939922873	9.92005068	9.009487618	8.981364568
ENSG00000184635	ZNF93	0.870236725	0.719117881	0.851331203	0.722362352	1.721567928	2	6.228547981	2	5.590471342	5.60003091	5.553513572	5.601925227
ENSG00000141867	BRD4	0.543076889	0.232704499	1.178047086	0.004306487	1.721123975	9.657036304	8.955547846	8.941672936	9.755113959	9.77093593	10.28440567	10.49896112
ENSG00000149679	CABLES2	0.480257466	0.831692971	1.239458972	0.544751469	1.719716438	3.327711234	7.099070191	2	6.18083559	6.06344184	6.752490997	6.938065226
ENSG00000176788	BASP1	1.052477479	0.212500963	0.665100054	0.443633913	1.717577533	11.74855118	9.664384941	10.83618737	12.05344541	12.0166989	11.34538214	11.89841387
ENSG00000110013	SIAE	1.133515142	0.057080264	0.583443363	0.352922604	1.716958505	7.218819781	7.35507212	6.073378139	8.113468729	8.10170499	7.722704302	7.392628644
ENSG00000253767	PCDHGA8	4.577231877	0.003100969	-2.861558149	0.079386828	1.715673729	2	4.11352228	7.098165388	10.14797511	10.2029317	3.483289517	3.33635788
ENSG00000029363	BCLAF1	1.550725747	0.175563893	0.164940305	0.902362275	1.715666052	6.653098118	9.969011754	8.460932916	10.47329248	10.4637324	8.813506006	9.320735104
ENSG00000151917	BEND6	0.262091201	0.923796154	1.453080171	0.550340609	1.715171372	2	6.899080544	2	5.461211713	5.84514933	6.756113529	6.826173076
ENSG00000198231	DDX42	0.821120167	0.185774297	0.893580179	0.139114355	1.714700347	8.631580145	9.687856435	10.0085654	10.33807884	10.4006406	10.31818704	10.55593703
ENSG00000151116	UEVLD	0.708528612	0.60637008	1.006029182	0.441586799	1.714557795	4.467311984	6.535001748	8.266922072	7.890605449	7.75969386	8.192572047	8.042267212
ENSG00000066135	KDM4A	1.677439278	0.000102491	0.03562567	0.950358432	1.713064948	7.709880939	7.669933291	6.80318718	9.103012382	9.12637045	7.510690356	7.478755384
ENSG00000136710	CCDC115	0.79133698	0.552070286	0.921405089	0.476444675	1.712742069	4.00722531	7.901717006	7.285536013	7.954442493	7.79989812	7.967337618	8.045972122
ENSG00000184343	SRPK3	-1.303435209	0.800325517	3.015417766	0.505167085	1.711982558	2	2	2	2.270786764	2	3.810410249	3.383972333
ENSG00000103319	EEF2K	0.175771088	0.887688028	1.535772249	0.131017641	1.711543337	5.095959458	7.051611315	8.110401118	7.398825229	7.35080119	8.723392661	8.703804674
ENSG00000182963	GJC1	0.286610445	0.792847991	1.423592759	0.12513586	1.710203203	7.435667355	7.315436877	9.413059136	8.709369553	8.64989445	9.937829229	9.668007958
ENSG00000146856	AGBL3	0.187771121	0.946524933	1.522326214	0.540120633	1.710097335	2	2	3.369317972	2.695456494	2.59489879	3.584558147	2.885414003
ENSG00000116209	TMEM59	1.526189282	0.000238144	0.182617313	0.720364135	1.708806595	11.08909278	10.20420974	10.51254821	12.19611659	12.1473525	10.80996641	10.85033379
ENSG00000136383	ALPK3	-0.903931393	0.570747269	2.612147445	0.063922883	1.708216053	2	5.72254323	6.718950348	4.867343068	5.00774437	6.68637457	7.690021882
ENSG00000139350	NEDD1	1.154315438	0.200056839	0.553395215	0.560395372	1.707710654	7.159102577	6.464272742	8.631919287	8.852852814	8.82635531	8.208524517	8.281047142
ENSG00000132466	ANKRD17	0.867297121	0.067189131	0.83862424	0.073994474	1.705921361	9.460782017	10.42046463	10.2528023	10.95089319	10.9846155	10.98330608	10.89377011
ENSG00000170145	SIK2	0.900979197	0.007742105	0.804498108	0.018178256	1.705477306	8.493334308	8.791896851	8.884815469	9.643348214	9.612912	9.694536155	9.349127298
ENSG00000263001	GTF2I	2.59837292	0.017562521	-0.89350443	0.460053783	1.70486849	6.468621203	8.702538795	10.01740919	11.57566116	11.6103915	8.018757917	8.215115301
ENSG00000128487	SPECC1	1.821668538	0.119484573	-0.11743758	0.93349971	1.704230958	5.330594134	7.60566235	8.805342542	9.578597652	9.68122943	8.089380147	7.199901369
ENSG00000150401	DCUN1D2	0.233216328	0.821674232	1.470857051	0.086540628	1.704073379	5.330594134	7.099070191	5.59507254	6.535745081	6.42738748	7.967337618	7.327148877
ENSG00000021355	SERPINB1	1.651166782	0.001135193	0.052097607	0.937350796	1.703264388	8.009475818	7.315436877	7.691296561	9.354865403	9.29432703	7.304736313	8.8007022
ENSG00000131373	HACL1	0.66277102	0.436914894	1.040214259	0.190601024	1.702985279	7.218819781	5.457120953	6.073378139	7.030886362	7.09050444	7.218085825	7.612190665
ENSG00000091542	ALKBH5	0.489876863	0.329903713	1.212363833	0.007109557	1.702240695	10.25038534	9.357954881	9.926432745	10.27745223	10.4678974	10.91162437	11.26054287
ENSG00000087586	AURKA	1.186019722	0.128090043	0.514982637	0.534560654	1.701002359	4.00722531	5.303523747	5.772716771	6.24507773	6.34282908	5.618636935	5.713290392
ENSG00000112242	E2F3	1.274518199	0.419141353	0.425626669	0.801603223	1.700144868	3.327711234	7.73146271	8.65477198	8.967964567	8.95536429	8.131128273	8.112850643
ENSG00000160072	ATAD3B	1.239739037	0.110962	0.459544204	0.584645012	1.699283241	7.624139768	7.232742069	5.59507254	8.197043419	8.32935768	7.474865722	7.520630205
ENSG00000166478	ZNF143	0.991173746	0.472579685	0.706764051	0.611923598	1.697937797	7.53297911	7.099070191	3.369317972	7.77569021	7.74878077	7.621085148	7.331709059
ENSG00000135047	CTSL	2.930331982	0.003161093	-1.232557583	0.255070887	1.697774399	11.72557631	9.34816733	9.372276026	13.5295502	13.5511473	9.730427609	8.931664222
ENSG00000196453	ZNF777	0.755849822	0.706288309	0.940490892	0.628768646	1.696340714	8.444143041	6.311476754	2	7.855024645	7.95749494	8.157391075	8.017007082

ENSG00000134369	NAV1	0.231700443	0.626623393	1.464370629	0.000174595	1.696071072	11.12074405	11.49575678	10.91875185	11.42521186	11.4368305	12.34622686	12.92177157
ENSG00000159840	ZYX	-0.065875158	0.927489437	1.758259788	0.001514467	1.69238463	11.75864596	10.49332014	11.02342937	11.08829583	11.1492725	12.69425333	13.15230292
ENSG00000073921	PICALM	0.860836774	0.306865781	0.831491923	0.315344239	1.692328697	9.174465797	10.30378101	11.26536143	11.37226044	11.3168096	11.37902969	11.24903735
ENSG00000153879	CEBPG	0.262254474	0.838100556	1.429815407	0.188337529	1.692069881	6.138360429	9.00621767	7.646393253	8.300810597	8.29225867	9.910457803	8.781973674
ENSG00000175550	DRAP1	1.159436679	0.030199232	0.53249983	0.353010925	1.69193651	8.737455672	8.929529568	9.819251342	10.39227626	10.3962764	9.639212351	9.889343672
ENSG00000174516	PELI3	0.258201495	0.91292494	1.433555793	0.488684721	1.691757288	2	8.169643011	4.523675391	7.075737784	6.86146731	8.362013331	7.834118096
ENSG00000068885	IFT80	1.782638111	0.417782282	-0.094013459	0.96989602	1.688624652	3.327711234	7.847167909	2	8.033874364	8.14604733	6.028179536	6.476841503
ENSG00000197045	GMFB	1.451048163	0.12992063	0.236428046	0.831469687	1.687476208	8.107517749	8.791896851	6.073378139	9.528658349	9.42518018	8.245074976	8.302113262
ENSG00000164048	ZNF589	0.839924885	0.712294566	0.847332407	0.705796484	1.687257292	2	7.467835385	3.369317972	6.841288255	6.77805036	7.194250299	6.30506411
ENSG00000153044	CENPH	0.581457228	0.710155984	1.103808891	0.44445996	1.68526612	4.467311984	3.686303414	2	4.415698455	3.7864959	4.720468739	4.365060851
ENSG00000012211	PRICKLE3	0.960214344	0.431243113	0.72230079	0.554788482	1.682515134	4.467311984	7.35507212	5.39244166	7.101994243	7.28055563	6.763331418	7.138761401
ENSG00000198865	CCDC152	3.218783623	0.310450907	-1.537717382	0.65106688	1.681066242	3.327711234	2	2	4.387512679	4.58534546	2.42098416	2
ENSG00000196455	PIK3R4	0.931343823	0.30261211	0.749277178	0.407381942	1.680621001	7.667647223	8.414402626	6.073378139	8.645348336	8.55284928	8.348279032	8.487906181
ENSG00000179051	RCC2	1.086860776	0.033252373	0.59260517	0.270018177	1.679465947	9.590776211	8.57407404	9.589475645	10.34633939	10.4541068	9.908628006	9.908803154
ENSG00000124275	MTRR	1.288641417	0.400196775	0.390818115	0.81390919	1.679459532	3.327711234	8.414402626	7.451345288	8.744518279	8.71301725	7.854430723	7.830900438
ENSG00000131374	TBC1D5	0.52782196	0.596606509	1.151427229	0.205709574	1.67924919	8.493334308	9.030900487	6.534109959	8.80304041	8.94105405	9.446970496	9.537567589
ENSG00000188070	C11orf95	0.340530758	0.840490657	1.338438289	0.365630894	1.678969047	4.00722531	8.993716073	8.206335842	8.459845423	8.41122995	9.731578082	9.035944835
ENSG00000130818	ZNF426	0.908912266	0.537027555	0.769789892	0.601793533	1.678702158	5.70932728	8.717821467	5.156626401	8.341783829	8.27714776	8.313667445	8.016062975
ENSG00000179632	MAF1	0.536197354	0.33517337	1.142108837	0.024149698	1.678306192	8.367060731	9.34816733	9.465702942	9.683748348	9.65881449	10.29968974	10.24949979
ENSG00000123064	DDX54	0.823283121	0.384414708	0.853640492	0.35551459	1.676923613	6.138360429	8.505280662	8.143093325	8.720710586	8.71442024	8.530120421	8.936662123
ENSG00000112977	DAP	0.192643861	0.857804601	1.484197708	0.093533047	1.676841569	11.30078111	10.71132792	8.845626171	10.7521165	10.8336713	11.87479202	12.26508948
ENSG00000182919	C11orf54	0.128922828	0.863599094	1.547421878	0.00811335	1.676344706	6.816637977	6.464272742	5.39244166	6.372775472	6.56439512	7.888662695	7.808173621
ENSG00000140749	IGSF6	-3.753079805	0.413653291	5.42867747	0.166693658	1.675597666	2	2	2	2	2	6.126613714	4.692661645
ENSG00000014123	UFL1	1.03541828	0.593517792	0.638220399	0.747455247	1.673638679	2	7.874700259	8.677268317	8.839910123	8.71020716	8.614358955	8.108428191
ENSG00000181938	GINS3	1.928720422	0.176419446	-0.256147299	0.877983632	1.672573123	4.00722531	5.838937808	2	6.522845756	6.47451726	4.504874511	4.543391831
ENSG00000161509	GRIN2C	-2.312458114	0.577663983	3.983867454	0.243272299	1.67140934	2	2	2	2	2.14022021	4.425202323	4.228274364
ENSG00000271092	TMEM56-RW	4.076651028	0.025536658	-2.405995245	0.247980941	1.670655783	2	2	3.369317972	5.273747807	5.18011485	2.139668783	2.117335275
ENSG00000163939	PBRM1	0.813091611	0.559250209	0.854973898	0.531253192	1.668065509	5.095959458	8.955547846	9.25752426	9.381011913	9.37992356	9.323921045	9.514020196
ENSG00000087460	GNAS	0.66147735	0.177667207	1.006392488	0.029583545	1.667869839	12.19573173	12.50249943	11.55983287	12.79322888	12.8042115	12.97462236	13.29462079
ENSG00000160305	DIP2A	0.42244107	0.620033622	1.244196597	0.099323089	1.666637668	8.418903792	7.761270542	6.43197674	8.213192703	8.1187584	9.078747095	8.873429675
ENSG00000110697	PITPNM1	0.106952757	0.910881185	1.559624581	0.038741655	1.666577338	7.624139768	8.029695761	9.399592459	8.636393836	8.69180693	9.911270301	10.28108376
ENSG00000047410	TPR	1.279819395	0.089945888	0.385784417	0.643465101	1.665603812	9.556468883	10.43902632	8.536706852	10.96556523	11.0116859	10.20827046	9.976858
ENSG00000162702	ZNF281	1.316406222	0.002491297	0.347520258	0.483213396	1.663926481	9.233563228	8.639735838	9.014156722	10.27601154	10.3014409	9.613232802	8.962375727
ENSG00000103264	FBXO31	0.344236409	0.681389915	1.319514954	0.06883389	1.663751363	8.493334308	8.702538795	6.882775709	8.483340231	8.64548836	9.611983935	9.45254121
ENSG00000106636	YKT6	0.518346363	0.304613133	1.145253811	0.0121807	1.663600174	9.497412313	9.415325457	10.26770388	10.29747219	10.290665	10.79815488	11.0311848
ENSG00000169554	ZEB2	1.001810441	0.29623641	0.659890321	0.499253973	1.661700762	8.75772933	10.76748534	8.763901704	10.75073417	10.7824203	10.46416585	10.3860243
ENSG00000105497	ZNF175	-4.714961636	0.0591539	6.376095851	0.000147017	1.661134214	2	3.076665209	2	2	2	7.358152527	7.438450313
ENSG00000014914	MTMR11	1.003446234	0.60852038	0.657066801	0.740394508	1.660513035	2	6.140560021	8.296287027	7.954442493	8.0358969	7.673747216	7.636966981
ENSG00000176182	MYPOP	0.682971386	0.590107068	0.977329206	0.418666323	1.660300592	5.70932728	5.595927442	8.266922072	7.781117077	7.7127321	8.116406856	7.952344882
ENSG00000177548	RABEP2	1.252688487	0.386166537	0.407599832	0.794186359	1.660288319	5.70932728	8.077869079	4.058998049	8.03841338	8.07792246	7.27600668	7.18317631
ENSG00000122042	UBL3	1.072414376	0.539282524	0.587770778	0.74490284	1.660185153	3.327711234	8.414402626	9.516493218	9.5407064	9.57942664	9.132692373	9.022374596
ENSG00000114544	SLC41A3	0.258391167	0.60106289	1.401704186	0.000584048	1.660095354	7.904282243	7.761270542	7.285536013	7.898117066	7.96458672	8.841056341	9.253832157
ENSG00000198837	DENND4B	0.599957924	0.652929267	1.058637257	0.396684362	1.65859518	4.815609471	8.623598428	7.934808822	8.365459376	8.41985547	8.960480054	8.723602991
ENSG00000180332	KCTD4	0.727687405	0.786927084	0.928640424	0.718605505	1.65632783	2	3.076665209	2	2.868578001	2.86123014	2.803598391	3.100202476
ENSG00000153989	NUS1	1.613860488	0.008858946	0.041541349	0.958081539	1.655401837	7.624139768	7.979858146	9.049077186	10.00919444	9.88434767	8.363201462	8.410985412
ENSG00000113356	POLR3G	0.283033323	0.856469262	1.372315582	0.303728756	1.655348905	4.00722531	5.131603625	2	4.299492648	4.65713986	5.654016955	5.177806074
ENSG00000163110	PDLIM5	-0.400975475	0.770883416	2.055769801	0.080413293	1.654794327	7.750913381	11.19631665	11.58682058	10.49161431	10.4603916	12.98609149	12.87062285
ENSG00000170364	SETMAR	-0.048210558	0.966538801	1.700898449	0.066536768	1.652687891	4.467311984	6.04685525	4.058998049	5.058234798	5.21224859	6.68757835	6.841176624
ENSG00000197619	ZNF615	0.930605078	0.521753772	0.72181086	0.619812492	1.652415937	4.00722531	7.35507212	4.874587708	7.157295126	6.90635305	7.182183216	6.37742435
ENSG00000066279	ASPM	1.050047958	0.563707037	0.602323105	0.747588951	1.652371062	3.327711234	5.595927442	2	5.363304456	5.30456641	4.209638631	5.419744333
ENSG00000145050	MANF	1.210561686	0.244396401	0.439208478	0.695304679	1.649770164	9.435834703	6.951736442	7.691296561	9.653739712	9.5678312	8.416823986	9.175242287
ENSG00000082996	RNF13	1.523204258	0.406678307	0.126397912	0.951751695	1.64960217	7.975256505	8.540087354	2	9.255049401	9.17914662	7.962642137	7.698285408
ENSG00000128335	APOL2	1.606955258	0.025425486	0.041102498	0.964424173	1.648057757	8.836089686	9.591588447	10.60345421	11.44136838	11.4780933	9.638721848	10.11774074
ENSG00000198056	PRIM1	1.874424026	0.40298478	-0.226507331	0.929596218	1.647916696	2	3.686303414	2	3.95996233	4.0930011	3.035909414	2.466008509
ENSG00000248672	LY75-CD302	6.837015545	0.005498029	-5.191141267	0.09315428	1.645874278	2	2	2	6.984595707	6.83591385	2	2
ENSG00000155008	APOOL	0.086827905	0.942525992	1.558541114	0.117467726	1.645369019	5.532353208	7.051611315	8.35328246	7.53353831	7.42122542	9.092423696	8.745413684
ENSG00000164073	MFSD8	1.45080403	0.26540262	0.194542859	0.896638566	1.645346889	7.435667355	7.099070191	3.369317972	8.258639605	8.05595642	6.981729486	6.881131115
ENSG00000056277	ZNF280C	1.279188332	0.123978923	0.366003025	0.690278517	1.645191358	4.00722531	5.131603625	5.39244166	6.29903474	5.93454154	5.47655442	5.065479856
ENSG00000106244	PDAP1	0.48647754	0.489850205	1.158685065	0.067503656	1.645162605	9.579430841	8.590771778	8.175061096	9.376537567	9.40011621	9.862742364	10.22886814
ENSG00000090989	EXOC1	1.401182696	0.001537778	0.242985399	0.642930217	1.644168095	8.138771685	7.572420273	7.285536013	9.00785664	9.17201613	8.069864557	7.81253023
ENSG00000174996	KLC2	0.765435081	0.292274211	0.876682379	0.211608759	1.64211746	9.33149386	8.433041974	7.646393253	9.391694063	9.39049411	9.539738196	9.46262491
ENSG00000188313	PLSCR1	3.552397159	0.002181398	-1.913169018	0.120834165	1.639228141	9.435834703	5.946638857	6.958202216	11.7198617	11.7308771	6.433059984	6.222348126
ENSG00000165175	MID1IP1	0.072099296	0.935263493	1.566971447	0.02674158	1.639070742	6.96351253	7.874700259	8.721235202	8.076429088	8.11025689	9.811219795	9.291382799
ENSG00000135945	REV1	0.74852836	0.632487313	0.890002794	0.557451959	1.638531155	4.00722531	8.376385542	6.073378139	7.772969103	7.93600799	7.934143724	8.055192982
ENSG00000126970	ZC4H2	0.531303922	0.714622915	1.107225595	0.410036586	1.638529518	5.330594134	7.572420273	4.058998049	7.030886362	6.74548212	7.302260672	7.593325136
ENSG00000138080	EMILIN1	1.307326783	0.021422337	0.329362623	0.61033641	1.636689406	11.21173326	10.20420974	10.0085654	11.86363439	11.8983971	10.96297653	10.84383043
ENSG00000116062	MSH6	0.090956424	0.921327192	1.544274286	0.035456159	1.63523071	7.096806373	8.876041558	8.922968322	8.540455898	8.65720817	10.10054783	9.984841938
ENSG00000163428	LRRC58	0.600075182	0.628451704	1.034973928	0.372265366	1.63504911	6.138360429	9.540933215	8.486635645	9.236404433	9.18523054	9.834322366	9.423285228
ENSG00000083520	DIS3	0.921912354	0.36126721	0.712269046	0.483045217	1.6341814	6.366709671	9.055168103	8.608698767	9.366644887	9.24080977	9.174915459	9.014359877
ENSG00000125356	NDUFA1	0.959406731	0.196836305	0.673670303	0.372076473	1.633077034	9.097018025	7.572420273	7.734844356	9.204447245	9.30367823	8.862639958	9.07227736
ENSG00000153140	CETN3	1.270834451	0.354199679	0.362180615	0.808803514	1.633015066	6.009025997	5.72254323	2	6.628980152	6.43421531	5.948096914	5.349527629
ENSG00000113407	TARS	1.224644322	0.00192427	0.408114017	0.353971254	1.632758339	9.966689162	9.558016677	9.165043284	10.82354685	10.8180948	10.10214984	9.904473108
ENSG00000119979	FAM45A	2.078249598	0.230676825	-0.445502583	0.819020478	1.632747015	2	7.232742069	8.296287027	9.394352287	9.27639101	6.814608286	6.89352185
ENSG00000159905	ZNF221	0.897070195	0.34242241	0.73424395	0.435748117	1.631314146	4.00722531	5.72254323	4.874587708	5.920568914	5.85535975	6.004008792	5.408276315
ENSG00000127527	EPS15L1	0.713174283	0.549476534	0.917114158	0.424715914	1.630288441	8.468948322	7.393647515	5.156626401	8.276040626	8.20512425	8.360229299	8.520227711
ENSG00000118007	STAG1	1.368359545	0.425434611	0.259833587	0.891849925	1.628193132	3.327711234	8.717821467	6.073378139	8.726347845	8.73530306	7.49992804	7.763868281
ENSG00000122707	RECK	0.798318628	0.162633024	0.82970276	0.137078393	1.628021388	8.777722034	9.225224669	9.553445883	9.989990706	10.0367996	9.514829317	10.43169893
ENSG00000077458	FAM76B	1.271183012	0.291215116	0.356267888	0.787590496	1.6274509	3.327711234	6.535001748	5.772716771	6.851641821	7.02417148	6.417131325	5.601925227
ENSG00000184863	RBM33	0.804947935	0.293290194	0.822348746	0.271724882	1.627296681	9.423197387	7.761270542	8.175061096	9.443949663	9.41572435	9.443604635	9.450795495
ENSG00000059758	CDK17	1.863858309	0.287529501	-0.237163828	0.906201712	1.626694481	3.327711234	7.669933291	9.757248847	10.32001706	10.3626704	8.436147258	8.047821014
ENSG00000151623	NR3C2	2.237766043	0.21912951	-0.611497525	0.762269886	1.626268518	2	4.93638933	2	5.731694	5.49945122	3.429858444	3.287118176
ENSG00000141968	VAV1	-0.727833888	0.863750456	2.3533402	NA	1.625506312	2	2	2	2.141737246	2.2680111	3.648325314	2.551896972
ENSG00000145002	FAM86B2	3.643594916	0.392859546	-2.018322498	0.658188823	1.625272418	2	2	2	4.070463721	4.05806467	2	2.172608095
ENSG00000103248	MTHFSD	0.679015328	0.7690856	0.945471571	0.673457912	1.624486899	3.327711234	7.60566235	2	6.722207358	6.84106088	7.134272047	6.940057611
ENSG00000120256	LRP11	2.290879515	5.30E-06	-0.666830253	0.243487701	1.624049262	6.89194353	6.228547981	7.342949392	9.119171929	9.12531607	6.387074688	6.059610627
ENSG00000103769	RAB11A	0.813024953	0.171886783	0.809866696	0.16559668	1.622891649	10.13887448	9.608085847	8.805342542	10.45261097	10.4010763	10.34909821	10.49507022
ENSG00000155506	LARP1	0.584608584	0.167456107	1.037951569	0.00811335	1.622560153	10.39976609	11.19358635	10.93284392	11.42564506	11.5004268	11.8938265	11.93845516
ENSG00000182117	NOP10	0.460003357	0.705592343	1.162494387	0.291093097	1.622497744	10.36714848	7.790474954	8.007629717	9.690252889	9.67835667	10.25510417	10.50250454
ENSG00000024048	UBR2	1.11325866	0.211811753	0.508393903	0.590415354	1.621652562	6.89194353	9.125613817	7.896971941	9.309580714	9.41658653	8.890924823	8.624257514
ENSG00000184368	MAP7D2	0.312651914	0.912931516	1.307560401	0.605429695	1.620212314	2	3.076665209	2	2.695456494	2.68924543	2.885263063	3.383972333
ENSG00000177885	GRB2	0.483849626	0.223040801	1.135222266	0.001455406	1.619071892	9.509418609	10.18234215	10.04361983	10.40726916	10.440607	11.00668325	11.13810331
ENSG00000066230	SLC9A3	0.65994633	0.638989421	0.958903786	0.472427727	1.618850116	5.330594134	4.11352228	2	4.783303513	5.0259382	5.233158834	5.108633494
ENSG00000168591	TMUB2	0.635565825	0.685365166	0.982613336	0.507844439	1.618179162	9.371511179	7.979858146	4.523675391	8.828161587	9.00091592	9.341208071	9.177355133
ENSG00000127022	CANX	1.19703719	0.091109722	0.419435358	0.586489016	1.616472548	11.08508709	12.53629536	12.65268348	13.43492421	13.4459705	12.35521156	12.91691141
ENSG00000166130	IKBIP	1.104195523	0.000183037	0.512156221	0.10784488	1.616351744	9.247966931	9.114109157	8.763901704	10.19294689	10.1196108	9.581426916	9.552946519
ENSG00000149131	SERPING1	0.79024395	0.58832515	0.825797951	0.56390268	1.616041901	10.08373159	10.12043894	5.772716771	10.28987826	10.3897054	10.52368776	10.211653
ENSG00000187944	C2orf66	4.484493801	0.16845319	-2.869577966	0.429144636	1.614915836	2	2	2	4.846787657	4.63360314	2.094598369	2
ENSG00000129521	EGLN3	-2.272307319	0.358886773	3.887075281	0.044173979	1.614767962	2	3.076665209	2	2.141737246	2.14022021	5.180212365	4.927687581
ENSG00000128294	TPST2	0.405448059	0.825869978	1.208008421	0.465968326	1.61345648	3.327711234	7.954278496	9.180874355	8.551612617	8.50013767	9.124657997	9.49551136
ENSG00000181045	SLC26A11	1.154385238	0.311127936	0.458184811	0.706713381	1.612570049	4.815609471	7.790474954	8.042706615	8.550024073	8.56222967	7.957931324	7.775072826
ENSG00000078687	TNRC6C	0.334909021	0.876735407	1.277429889	0.501916413	1.61233891	5.095959458	7.874700259	2	6.815074525	6.86652414	7.88535852	7.620910859
ENSG00000173726	TOMM20	0.546546066	0.571724149	1.065753203	0.233224134	1.612299269	10.43166252	9.814097497	8.007629717	10.28606625	10.2535095	10.77166745	10.80349856
ENSG00000135245	HILPDA	0.605664469	0.633945061	1.00573159	0.400315488	1.611396059	4.467311984	7.979858146	7.451345288	7.849869274	7.80253898	8.071319244	8.355338318
ENSG00000183808	RBM12B	0.637575315	0.727420094	0.973184423	0.576686766	1.610759737	7.485143553	7.60566235	2	7.659688863	7.54308677	8.234726135	7.548321824
ENSG00000160613	PCSK7	0.509564468	0.647667448	1.099565519	0.282627158	1.609129987	6.96351253	7.572420273	9.478569125	8.890998469	8.95298909	9.544459308	9.471261544
ENSG00000151892	GFRA1	-0.909223561	0.639879212	2.517321104	0.146527599	1.608097543	4.00722531	3.686303414	8.35328246	5.987823101	6.04571775	8.737205015	9.794249791
ENSG00000143382	ADAMTSL4	-0.588907582	0.482526112	2.195689955	0.002195337	1.606782373	6.89194353	8.414402626	8.408111533	7.450827758	7.51437845	9.664732333	10.64977092

ENSG00000168538	TRAPPC11	0.97372773	0.549783955	0.632783393	0.704091061	1.606511123	3.327711234	8.077869079	8.742726175	8.875861021	8.81462916	8.598808264	8.410985412
ENSG00000204104	TRAF3IP1	1.283137273	0.090973094	0.321597152	0.705349408	1.604734425	7.790810999	6.464272742	6.073378139	8.201097719	8.24644267	7.283556699	7.277588383
ENSG00000110048	OSBP	0.538649209	0.510049398	1.065961154	0.154093392	1.604610363	8.716893051	8.522788974	10.14394518	9.832293064	9.87684923	10.4654111	10.28950788
ENSG00000081665	ZNF506	0.980130288	0.083005001	0.623601875	0.282259948	1.603732163	5.866944937	5.946638857	6.629488387	7.165617905	7.06433609	6.932953913	6.587749895
ENSG00000168813	ZNF507	0.445453963	0.825681195	1.157777407	0.528988964	1.60323137	8.468948322	7.274681896	2	7.932663244	7.77322015	8.692059118	8.409548182
ENSG00000063015	SEZ6	0.318951311	0.925027909	1.282946641	0.676479319	1.601897952	2	3.076665209	2	3.09461492	2.14022021	2.885263063	3.36036153
ENSG00000176055	MBLAC2	0.46497279	0.767183028	1.133719986	0.430383523	1.598692775	2	5.303523747	6.203069917	5.697663076	5.71663161	6.615624322	5.984375752
ENSG00000147145	LPAR4	0.051036888	0.985675142	1.545605011	0.538360258	1.5966419	2	3.076665209	2	2.695456494	2.49394783	2.962551659	3.560117069
ENSG00000163257	DCAF16	0.5975618	0.270310495	0.996454348	0.048281896	1.594016147	7.53297911	7.819099893	8.511888463	8.657201865	8.54024645	9.157900392	8.81165749
ENSG00000159069	FBXW5	0.359202117	0.265297898	1.232024834	9.85E-06	1.591226952	9.90440318	9.479495904	9.577565424	9.966365455	10.0727955	10.86939366	10.91202892
ENSG00000175197	DDIT3	2.025519724	0.03133681	-0.434883932	0.687792014	1.590635792	7.27616306	7.503546079	8.721235202	10.01035013	9.96317668	8.224302521	6.251520369
ENSG00000132388	UBE2G1	0.655842194	0.480535674	0.934026411	0.288509468	1.589868605	8.716893051	9.203675681	6.882775709	9.253098082	9.16587611	9.475267633	9.49788026
ENSG00000174326	SLC16A11	-1.303393354	0.803778942	2.891486596	NA	1.588093242	2	2	2	2.270786764	2	3.663836244	3.383972333
ENSG00000138081	FBXO11	1.60413458	0.023977132	-0.01696545	0.984890159	1.58716913	8.340423424	8.395519306	6.629488387	9.591782342	9.56705484	8.029274808	7.916365698
ENSG00000011451	WIZ	0.21696598	0.876259116	1.370023574	0.242288367	1.586989553	10.09173937	8.671479029	6.534109959	9.226493643	9.30554121	10.35074632	10.47545674
ENSG00000005436	GCFC2	0.32914893	0.590895562	1.255710828	0.016427091	1.584859758	5.532353208	6.389896633	6.073378139	6.429169081	6.31350454	7.204892513	7.324100729
ENSG00000136816	TOR1B	0.303325723	0.743829336	1.27893809	0.11156057	1.582263813	9.646202137	8.029695761	7.858115942	9.060130342	9.04395769	10.08496939	9.956825691
ENSG00000168496	FEN1	0.157505675	0.879872883	1.422331745	0.091732358	1.579837421	4.815609471	6.78762695	6.629488387	6.422239259	6.51372389	7.265878131	8.025476363
ENSG00000224383	PRR29	0.045282321	0.988285735	1.533819392	0.562631262	1.579101714	2	3.076665209	2	2.270786764	2.86123014	3.256555576	3.311948089
ENSG00000102753	KPNA3	1.031479043	0.396224049	0.547518559	0.666185912	1.578997602	8.631580145	9.34816733	5.772716771	9.58946437	9.50841342	9.168813127	8.960904642
ENSG00000139626	ITGB7	2.395407291	0.520119153	-0.817698537	NA	1.577708754	2	2	2	3.709785322	2.49394783	2.267001569	2.117335275
ENSG00000171163	ZNF692	0.078437621	0.94475044	1.498258008	0.107289325	1.576695628	6.563806279	6.311476754	4.058998049	6.155987605	5.99120031	7.700314972	7.124815549
ENSG00000177663	IL17RA	1.12509812	0.005668424	0.451099935	0.308943128	1.576198055	8.107517749	8.101365653	8.784770914	9.458417697	9.49623046	8.885560124	8.726491728
ENSG00000072401	UBE2D1	2.041656551	0.092065517	-0.466008713	0.732916452	1.575647838	3.327711234	7.189546432	6.718950348	8.521127045	8.36552655	6.134985075	5.88031069
ENSG00000204963	PCDHA7	1.031511683	0.686593442	0.5428848	0.836490254	1.574396483	2	3.686303414	2	3.840289928	2.86123014	2.858551861	3.383972333
	C19ORF68	6.763667823	0.007296304	-5.191140549	0.097775866	1.572527274	2	2	2	6.86703417	6.81514011	2	2
ENSG00000135164	DMTF1	1.290761951	0.229716518	0.278269063	0.81770874	1.569031013	8.586954094	9.018611864	5.930865648	9.584041242	9.64048078	8.648381562	8.565456872
ENSG00000129038	LOXL1	-1.042748507	0.1406021	2.61159446	3.49E-05	1.568845953	10.1311248	8.834582582	9.703463497	8.668958796	8.55598286	11.62494827	12.69103003
ENSG00000148840	PPRC1	0.375594481	0.796912783	1.192751377	0.35900228	1.568345858	4.815609471	8.623598428	8.825624954	8.559529208	8.56534297	9.422673149	9.321499526
ENSG00000187624	C17orf97	1.223030104	0.362279345	0.345126821	0.81390919	1.568156925	6.009025997	5.303523747	2	6.172600389	6.42738748	5.357600875	5.606950214
ENSG00000087152	ATXN7L3	0.801371311	0.115157031	0.766626984	0.126902787	1.567998295	9.371511179	9.664384941	8.631919287	10.07473944	10.0971999	10.18343032	9.906002844
ENSG00000143119	CD53	-3.75307254	0.427813453	5.320513139	0.191567177	1.567440599	2	2	2	2	2	6.075339068	4.456980017
ENSG00000144366	GULP1	1.020822872	0.558047538	0.546556523	0.762852238	1.567379395	2	7.538394143	7.398165132	7.982979299	7.81305436	7.396955563	7.473262559
ENSG00000100426	ZBED4	0.309073886	0.882971353	1.256306518	0.49597846	1.565380404	2	7.847167909	8.561105505	7.93509946	8.01555453	9.010625386	8.806206367
ENSG00000241553	ARPC4	0.964088954	0.066974888	0.598861386	0.271400583	1.56295034	8.836089686	9.479495904	9.565556058	10.25617662	10.3209133	9.471140938	10.26984055
ENSG00000041802	LSG1	1.095908181	0.379885407	0.466881519	0.723926237	1.562789699	4.815609471	8.191702463	8.460932916	8.861844103	8.9229643	8.162177243	8.37093032
ENSG00000135387	CAPRIN1	0.981449287	0.036333097	0.580933636	0.235600774	1.562382923	10.21416435	11.16599533	10.50627919	11.68934031	11.6446463	11.30065805	11.23297706
ENSG00000176953	NFATC2IP	0.91172266	0.073765025	0.650196725	0.210659124	1.561919385	8.042902253	7.790474954	8.805342542	9.140440229	9.20831741	8.90322929	8.927151259
ZNF664-FAM101A		6.75243517	0.007655523	-5.191140397	0.098726147	1.561294773	2	2	2	6.772115916	6.88657603	2	2
ENSG00000111450	STX2	0.128420195	0.83752462	1.429972387	0.003580998	1.558392581	8.009475818	8.451443573	9.099922433	8.723531969	8.70598173	9.874038788	10.13088292
ENSG00000107929	LARP4B	0.505761843	0.348682275	1.052587342	0.032404634	1.558349185	8.777722034	9.608085847	8.65477198	9.530270581	9.64342987	10.12018263	10.14455455
ENSG00000213625	LEPROT	0.541514584	0.309909983	1.01485488	0.038648151	1.556369464	9.949163863	9.479495904	10.49998282	10.57051624	10.576422	10.87669185	11.19708549
ENSG00000106299	WASL	0.738975847	0.127670293	0.817110483	0.083336867	1.55608633	8.716893051	8.862353853	8.143093325	9.40405742	9.28113268	9.673379144	9.116112551
ENSG00000167378	IRGQ	1.101504635	0.018749668	0.453086382	0.372076473	1.554591017	9.602033055	8.590771778	9.116480286	10.22863675	10.2769623	9.58550484	9.628710015
ENSG00000104894	CD37	-3.753078007	0.418387377	5.305433271	0.182610051	1.552355264	2	2	2	2	2	5.906324469	4.836507146
ENSG00000156983	BRPF1	0.562716433	0.71541993	0.987426887	0.495779443	1.55014332	7.867441304	7.819099893	3.369317972	7.810605345	7.87959733	8.287622092	8.239587639
ENSG00000203778	FAM229B	0.222195793	0.841373243	1.325329055	0.153932236	1.547524848	7.159102577	5.595927442	4.874587708	6.470063213	6.33555364	7.452712791	7.488317792
ENSG00000185963	BICD2	-0.029761675	0.974752037	1.577000746	0.035746075	1.547239071	7.82963488	8.655694736	9.778213818	8.970341944	8.91444416	10.54098392	10.54206659
ENSG00000067992	PDK3	-0.925825098	0.133293863	2.47273291	4.64E-06	1.546907812	6.96351253	6.78762695	6.322058544	5.808110275	5.87556634	8.651302479	9.515022972
ENSG00000125304	TM9SF2	1.328431105	0.003598276	0.217617475	0.688299482	1.546048581	10.49341471	10.11467741	9.516493218	11.40228438	11.4404381	10.15592861	10.45487637
ENSG00000107959	PITRM1	1.155638531	0.2265035	0.389246848	0.708157776	1.544885378	7.485143553	8.297207175	9.868959282	10.00340207	10.0798882	9.172206498	9.379543083
ENSG00000135900	MRPL44	0.980969658	0.126065169	0.563066174	0.397324398	1.544035832	8.285626337	7.467835385	6.80318718	8.557949361	8.66303256	8.219062336	8.177610383
ENSG00000179918	SEPHS2	0.785183514	0.359261467	0.757725853	0.368129487	1.542909367	9.435834703	8.077869079	7.502634626	9.302050417	9.40098777	9.185700723	9.452192236
ENSG00000105472	CLEC11A	1.083810115	0.336744609	0.458185513	0.702091517	1.541995627	10.21416435	7.431218269	8.110401118	10.15373922	10.1822948	9.571181392	9.518693865
ENSG00000145817	YIPF5	1.527811672	7.90E-06	0.013063092	0.976832019	1.540874764	9.567995545	10.14890644	10.21486071	11.55640578	11.5034733	9.997579827	10.04022229
ENSG00000139613	SMARCC2	1.214634889	0.000674918	0.325667974	0.42448075	1.540302864	9.262228249	8.623598428	8.805342542	10.091763	10.1598144	9.166092673	9.313452824
ENSG00000152465	NMT2	0.402296678	0.624897619	1.13765861	0.120205411	1.539955288	6.009025997	7.572420273	7.858115942	7.797276258	7.67575955	8.401292507	8.520893436
ENSG00000273686		3.02803197	0.000112893	-1.490559479	0.076831332	1.537472492	14.00909496	12.13658528	12.19766108	16.12211306	16.0595297	11.70438214	11.43146841
ENSG00000169994	MYO7B	-2.306377453	0.589234072	3.843806445	0.2779606	1.537428992	2	2	2	2.141737246	2	4.406883583	4.002845082
ENSG00000170837	GPR27	-2.306294028	0.594639663	3.843667276	0.286305677	1.537373247	2	2	2	2.141737246	2	3.709392871	4.594883674
ENSG00000166794	PPIB	2.624040511	2.15E-08	-1.086913849	0.032897253	1.537126662	11.01523161	9.975384048	10.30429804	13.09939874	13.1366789	9.22511553	9.582902386
ENSG00000160951	PTGER1	0.67480488	0.785196727	0.861961693	0.719120052	1.536766573	2	6.140560021	2	5.461211713	5.19627118	5.673303969	5.313094696
ENSG00000166887	VPS39	0.779667697	0.582172352	0.756969232	0.587973908	1.536636929	6.009025997	9.943237334	10.28245311	10.31300956	10.3971503	10.31864645	10.34732599
ENSG00000116017	ARID3A	0.938628264	0.034657972	0.597281455	0.19277085	1.535909719	6.96351253	6.899080544	7.451345288	7.959237999	8.10598728	7.842553218	7.539150216
ENSG00000083750	RRAGB	0.545912091	0.749224767	0.989565837	0.537860646	1.535477929	2	7.051611315	6.629488387	6.820355514	6.83074839	7.20223931	7.307219812
ENSG00000259956	RBM15B	0.357682257	0.723107864	1.176838784	0.187681315	1.534521041	9.521325812	9.910357957	7.502634626	9.603316663	9.69266358	10.36252773	10.56015326
ENSG00000254087	LYN	0.669938087	0.663958104	0.864290813	0.560286335	1.5342289	6.257048221	7.819099893	3.369317972	7.363085426	7.36873214	7.272216802	7.792821043
ENSG00000198768	APCDD1L	-3.753070016	0.432702068	5.284632105	0.200448801	1.531562089	2	2	2	2	2	6.057834398	4.376876162
ENSG00000060718	COL11A1	0.062045834	0.917858995	1.467812658	0.001387224	1.529858492	8.493334308	9.067150531	8.721235202	8.959612762	8.72141482	10.56053732	9.836041609
ENSG00000118689	FOXO3	0.982886009	0.028297569	0.546902502	0.246468033	1.529788512	8.39321512	9.328390744	9.06622548	9.941135381	9.98252	9.535001584	9.521025034
ENSG00000164081	TEX264	0.81612179	0.560680027	0.712256235	0.61097572	1.528378025	4.467311984	8.732943944	7.896971941	8.600009476	8.69180693	8.504505158	8.582138547
ENSG00000048740	CELF2	-1.675075969	0.033290369	3.200383366	8.95E-06	1.525307397	8.009475818	6.535001748	6.43197674	5.662809983	5.51241389	9.915528427	10.67451395
ENSG00000159335	PTMS	-0.108964465	0.858427987	1.633216959	0.000478561	1.524252493	10.34051281	9.599860728	10.47451889	10.06030508	10.0901921	11.50728966	12.06640492
ENSG00000115145	STAM2	0.368938665	0.745829169	1.155285084	0.256540578	1.524223749	9.710019321	7.002537973	8.143093325	9.083378928	9.04395769	9.849026448	9.842180628
ENSG00000054118	THRAP3	0.580642444	0.42279064	0.943353434	0.162472292	1.523995877	9.730679447	9.386925327	10.68342405	10.60955021	10.6290713	10.52089571	11.32970929
ENSG00000113621	TXNDC15	0.964678134	0.085477895	0.557316731	0.339494409	1.521994865	9.957954117	9.125613817	8.763901704	10.30172628	10.3608797	9.891848754	9.958792353
ENSG00000129474	AJUBA	-0.935412269	0.220826448	2.457132537	0.000280937	1.521720268	7.624139768	9.079034257	7.552162999	7.29643414	7.42122542	10.81887028	10.61511909
ENSG00000123297	TSFM	0.669041231	0.748404234	0.852589884	0.675028301	1.521631115	3.327711234	8.101365653	3.369317972	7.222570549	7.33264456	7.433611594	7.481493974
ENSG00000163728	TTC14	1.424467638	0.323982764	0.097074046	0.953777463	1.521541683	4.00722531	7.60566235	8.742726175	9.144656522	9.13791797	8.05229293	7.565201648
ENSG00000107862	GBF1	0.504258577	0.565891391	1.015438168	0.209646083	1.519696746	9.966689162	10.66082477	8.561105505	10.45261097	10.4856715	10.87565151	11.07581394
ENSG00000168827	GFM1	0.841601312	0.527890193	0.675526665	0.611774286	1.517127977	8.444143041	8.004992147	4.523675391	8.532433657	8.55127993	8.281350892	8.468021799
ENSG00000071189	SNX13	1.079685743	0.20811252	0.437307577	0.634083172	1.516993321	6.563806279	7.819099893	8.784770914	9.076774616	9.0282492	8.445151776	8.387086051
ENSG00000101844	ATG4A	0.861666882	0.106568751	0.654927626	0.222928155	1.516594508	6.468621203	6.311476754	6.073378139	7.148924055	7.0991228	7.134272047	6.676467423
ENSG00000116198	CEP104	0.727198684	0.306713831	0.789290831	0.25426536	1.516489515	8.737455672	7.232742069	7.896971941	8.813671111	8.78422202	9.072586388	8.611812925
ENSG00000165915	SLC39A13	0.460323398	0.628146304	1.05502017	0.222891601	1.515343568	10.6695979	9.540933215	8.408111533	10.23260335	10.3245927	10.86845263	10.87644816
ENSG00000066933	MYO9A	0.050370042	0.941473373	1.463826739	0.006147699	1.514196781	8.653385977	7.431218269	7.896971941	8.109160159	8.14188248	9.505174684	9.546749232
ENSG00000127220	ABHD8	0.207251615	0.906926658	1.306877806	0.388725469	1.514129421	8.418903792	6.78762695	3.369317972	7.497728447	7.4450836	8.634179825	8.468712049
ENSG00000114948	ADAM23	-0.04237584	0.981811904	1.554516217	0.308354984	1.512140377	3.327711234	7.099070191	4.058998049	5.839660368	5.67111583	7.094763493	7.446882182
ENSG00000131408	NR1H2	0.714729851	0.312262093	0.791290565	0.250438486	1.506020416	9.460782017	8.124485672	9.703463497	9.886796832	10.0096294	10.04351195	10.00781983
ENSG00000100739	BDKRB1	6.957728462	5.66E-09	-5.452162361	0.000459097	1.5055661	2	3.076665209	4.874587708	10.20914183	10.2074225	2.139668783	2
ENSG00000137673	MMP7	0.380498449	0.932860886	1.124670754	0.794654458	1.505169203	2	2	4.058998049	3.349643765	3.08597292	4.45225155	2
ENSG00000178338	ZNF354B	0.71697161	0.660186751	0.785602946	0.622327372	1.502574556	4.467311984	3.076665209	6.80318718	6.18902405	6.23747437	6.656902456	5.76365862
ENSG00000129245	FXR2	0.692227303	0.726949143	0.809704735	0.677249145	1.501932037	10.28571918	8.004992147	3.369317972	9.620825434	9.7138614	9.73525344	9.833096297
ENSG00000226173		9.031355207	7.09E-25	-7.531023029	2.36E-05	1.500332178	4.00722531	4.93638933	2	12.78877383	12.8690299	2.048074405	2
ENSG00000188566	NDOR1	1.252038139	0.036509535	0.247697023	0.720064769	1.499735162	6.009025997	7.232742069	6.322058544	7.836899669	7.88708154	6.85908071	6.905807071
ENSG00000150990	DHX37	0.796898186	0.486515971	0.702551526	0.536069347	1.499449713	8.16936288	6.728495673	5.156626401	7.877998516	7.99953197	7.782541424	7.909264332
ENSG00000076351	SLC46A1	0.414187895	0.704833394	1.085019226	0.269817196	1.499207121	4.815609471	7.051611315	5.59507254	6.592405944	6.49425375	7.463831776	6.856025745
ENSG00000151702	FLI1	0.980274673	0.185790369	0.518417262	0.502693319	1.498691935	6.73718411	8.297207175	7.163363831	8.503178519	8.56534297	7.844256003	8.280260977
ENSG00000175467	SART1	0.786270634	0.514396922	0.711786367	0.549060202	1.498057001	5.866944937	7.638155693	9.116480286	8.817637515	8.89354047	8.641543003	8.910481888
ENSG00000174206	C12orf66	0.769230005	0.765179552	0.728610609	0.776060045	1.497840614	2	2	6.203069917	5.304221918	5.53799497	5.65789504	5.0581604
ENSG00000161642	ZNF385A	0.606651925	0.506977228	0.890561685	0.300692047	1.49721361	5.70932728	7.669933291	7.896971941	7.885575888	8.03365075	8.204552906	8.277112026
ENSG00000069956	MAPK6	0.886924672	0.447339181	0.609944354	0.60616609	1.496869026	6.89194353	10.17128279	9.565556058	10.32281055	10.2496441	10.12894058	9.881848605
ENSG00000131435	PDLIM4	1.009381612	0.37278455	0.486476779	0.683005276	1.495858391	6.816637977	8.557180776	9.926432745	9.886796832	9.97434539	9.173900195	9.614433792
ENSG00000106771	TMEM245	0.066977524	0.954792088	1.42703289	0.149298501	1.494010414	8.418903792	9.277729677	11.03218278	10.11236021	10.0027561	11.51592898	11.30721324
ENSG00000236236		6.684376293	0.010013464	-5.191139549	0.104453095	1.493236744	2	2	2	6.872128674	6.64900057	2	2
ENSG00000125826	RBCK1	1.19135046	0.299070637	0.301613139	0.811551833	1.492963599	10.84091191	9.067150531	7.734844356	10.91339224	10.9608743	9.847329288	10.22784916
ENSG00000163040	CCDC74A	1.36350568	0.322678131	0.128655245	0.935446753	1.492160925	6.138360429	5.457120953	2	6.592405944	6.57060569	5.680947153	5.115701921

ENSG00000186908	ZDHHC17	0.626810959	0.701399082	0.864952357	0.580654321	1.491763316	4.00722531	7.35507212	9.031722608	8.535647908	8.44034872	8.846588786	8.591041727
ENSG00000162599	NFIA	-1.304498935	0.421581455	2.795585088	0.056539999	1.491086152	2	6.602424674	7.225742779	5.22679461	5.13053105	8.677327975	9.460197359
ENSG00000164144	ARFIP1	0.812716076	0.023841784	0.674852841	0.062801111	1.487568917	8.075571721	7.60566235	7.818184403	8.595396228	8.68323472	8.412813334	8.589138522
ENSG00000197472	ZNF695	2.595814869	0.43323806	-1.108884633	NA	1.486930235	2	2	2	3.289987342	3.39680241	2.094598369	2.225841198
ENSG00000091317	CMTM6	1.005606206	0.346801992	0.480108719	0.670289164	1.485714926	6.89194353	9.806969599	9.491321578	10.17399201	10.1807731	9.696187102	9.61068621
ENSG00000234636	MED14OS	-0.344263529	0.88827382	1.82541239	0.372590042	1.481148861	2	3.686303414	2	2.784612485	2.68924543	3.968654369	4.033026654
ENSG00000127241	MASP1	0.195588784	0.945987763	1.285484574	0.62811405	1.481073358	2	7.669933291	2	6.321555392	6.35006802	7.110413993	7.619668339
ENSG00000145476	CYP4V2	2.19020448	0.001757739	-0.710133785	0.364514502	1.480070694	8.138771685	6.602424674	6.629488387	9.446513412	9.49459826	6.710164669	6.528072775
ENSG00000166181	API5	0.877757332	0.681482862	0.597668722	0.783429907	1.475426053	2	8.820493869	9.746650996	9.618553696	9.67619834	9.396799738	9.340856676
ENSG00000120685	PROSER1	0.805055032	0.667251957	0.669610608	0.72102077	1.474665639	8.19931886	7.60566235	2	8.188900477	8.11238697	8.046387837	7.988411589
ENSG00000057757	PITHD1	0.369681713	0.829611423	1.104469726	0.473241998	1.47415144	8.564112515	7.538394143	3.369317972	7.966401501	7.92393105	8.64301114	8.697929554
ENSG00000144228	SPOPL	0.796430225	0.667396303	0.676273006	0.715242599	1.472703231	8.075571721	7.232742069	2	7.994705029	7.84926609	7.966556098	7.62463201
ENSG00000088325	TPX2	-0.691421764	0.69860994	2.163872253	0.170653805	1.472450489	6.257048221	8.777382925	3.369317972	6.856790872	6.71776877	9.639702688	9.556197588
ENSG00000065978	YBX1	0.338371471	0.574012887	1.128820778	0.03233724	1.467192249	11.55650736	10.73780697	10.47451889	11.35143572	11.3225709	11.95568405	12.27937268
ENSG00000089041	P2RX7	-3.753058783	0.451325678	5.21975834	0.22882416	1.466699556	2	2	2	2	2	6.109723842	3.8919413
ENSG00000120802	TMPO	0.213656314	0.789422857	1.252627734	0.06088768	1.466284048	6.816637977	7.467835385	8.175061096	7.917958914	7.66709116	9.157215598	8.388545823
ENSG00000174799	CEP135	1.356151837	0.02780363	0.10995017	0.885109991	1.466102007	5.095959458	5.72254323	5.930865648	7.106324235	6.68379438	5.783905948	5.670730563
	C10ORF10	6.655558276	0.010887704	-5.191139318	0.106137285	1.464418958	2	2	2	6.804454198	6.66650236	2	2
ENSG00000179412	HNRNPCL4	6.655363271	0.010826309	-5.191139354	0.105933593	1.464223917	2	2	2	6.772115916	6.70088158	2	2
ENSG00000277058	HNRNPCL3	6.655363271	0.010826309	-5.191139354	0.105933593	1.464223917	2	2	2	6.772115916	6.70088158	2	2
ENSG00000167986	DDB1	0.239612301	0.722229354	1.224195326	0.032155546	1.463807627	10.85509492	9.923599762	11.25043551	11.00087894	11.0255982	11.85871828	12.12225468
ENSG00000256222	MTRNR2L3	5.591831631	4.78E-09	-4.128620665	0.000386199	1.463210966	3.327711234	4.11352228	3.369317972	8.812346549	8.72280968	2.267001569	2.059860178
ENSG00000137073	UBAP2	0.416869038	0.771061294	1.045910893	0.426580825	1.462779931	7.096806373	8.968383114	5.156626401	8.145379268	8.30721295	8.736746727	8.956482366
ENSG00000161920	MED11	0.171049298	0.91899196	1.291651781	0.372076473	1.462701079	6.009025997	6.535001748	2	5.94010643	5.90535527	6.700796638	7.232780814
ENSG00000102189	EEA1	0.404893583	0.22951741	1.05704694	0.000380145	1.461940523	7.904282243	7.979858146	8.143093325	8.420396336	8.39906692	9.131995505	8.975062684
ENSG00000100461	RBM23	0.336502472	0.769854407	1.124791274	0.271460899	1.461293746	7.031698319	9.479495904	7.552162999	8.697938662	8.83153649	9.349025111	9.724584499
ENSG00000132326	PER2	0.371507834	0.743918027	1.089700854	0.283648588	1.461208688	5.532353208	3.686303414	6.203069917	5.742861563	5.80356929	6.33715871	6.554323192
ENSG00000157796	WDR19	0.91895177	0.54826185	0.54209662	0.730737838	1.46104839	3.327711234	7.572420273	8.175061096	8.211183901	8.29225867	7.972017867	7.783974227
ENSG00000241973	PI4KA	0.914313599	0.363354318	0.545084021	0.599502335	1.45939762	6.96351253	9.488434476	9.25752426	9.833599446	9.8316678	9.116226322	9.746095808
ENSG00000104299	INTS9	0.727600993	0.483194833	0.730902673	0.471266152	1.458503667	5.866944937	6.666836837	4.058998049	6.622948477	6.57060569	6.437578914	6.746446439
ENSG00000181830	SLC35C1	0.365252948	0.632952844	1.092214246	0.107174752	1.457467194	7.435667355	8.487557258	9.132850258	8.847689662	8.89230135	9.549947816	9.633334927
ENSG00000138769	CDKL2	-0.272308266	0.92422517	1.72943474	0.477216955	1.457126474	2	3.076665209	2	2.498691189	2.49394783	3.632645806	3.128433649
ENSG00000214029	ZNF891	-0.113213865	0.96332191	1.568644749	0.475128388	1.455430883	2	5.72254323	2	4.268917409	4.31639476	6.167993056	5.177806074
ENSG00000139194	RBP5	-2.312508989	0.59184685	3.766473241	0.294086803	1.453964252	2	2	2	2	2.14022021	4.04171018	4.26688425
ENSG00000154265	ABCA5	1.275954619	0.261985421	0.177233642	0.892291608	1.453188261	3.327711234	6.389896633	5.39244166	6.755670824	6.70653264	5.489669427	5.871985934
ENSG00000174989	FBXW8	0.336225709	0.803737808	1.116765599	0.35248148	1.452991308	8.16936288	8.433041974	4.874587708	8.219202381	8.02009991	8.932738969	8.856124078
ENSG00000164932	CTHRC1	2.817461463	0.183470484	-1.366018491	0.540695624	1.451442972	10.01801709	6.844429755	2	11.36888555	11.41801	7.754744914	6.454714037
ENSG00000163191	S100A11	0.976670055	0.053673848	0.474464559	0.376596657	1.451134614	11.76115866	10.88044608	11.2690689	12.30537237	12.3415581	11.46717251	12.10631899
ENSG00000013561	RNF14	0.721743853	0.289703301	0.728135391	0.274614244	1.449879244	8.696033109	7.35507212	8.805342542	9.160358775	9.08900105	9.166773267	9.095255687
ENSG00000077097	TOP2B	0.726971824	0.156616494	0.720822273	0.152087349	1.447794097	8.653385977	9.616264339	8.941672936	9.847245515	9.8667903	9.696894073	9.990140122
ENSG00000270011	ZNF559-ZNF	2.704834238	0.205847326	-1.257083543	0.588778065	1.447750695	2	3.076665209	2	4.070463721	4.0930011	2.267001569	2.277179783
ENSG00000009307	CSDE1	0.595187581	0.252535231	0.851944878	0.083938238	1.447132458	11.27639669	12.26437678	11.50743625	12.34816175	12.3371251	12.62183347	12.57626311
ENSG00000131375	CAPN7	0.664464164	0.410513137	0.780941405	0.315074518	1.445405569	7.159102577	8.862353853	8.434763955	8.986874783	8.9648262	9.426088142	8.654907296
ENSG00000129682	FGF13	3.442674196	0.051696866	-1.997418794	0.289718205	1.445255402	2	4.442710129	2	6.205262847	6.25300433	2.717032197	2.225841198
ENSG00000197746	PSAP	0.699686461	0.312262093	0.745101598	0.270301865	1.444788059	13.57805376	12.02889368	12.68078978	13.59239489	13.6113618	13.66815092	13.62615866
ENSG00000138600	SPPL2A	1.090226872	0.014945429	0.352482535	0.478845029	1.442709407	8.716893051	8.376385542	9.272377397	9.922839878	9.90476967	9.066399259	9.285518343
ENSG00000106609	TMEM248	0.570622247	0.038260026	0.871744277	0.000791829	1.442366524	10.19198706	9.975384048	10.08627303	10.65902291	10.6501178	10.8087678	11.08788243
ENSG00000104368	PLAT	0.933909	0.375381504	0.507954471	0.642633186	1.441863471	9.913467515	9.386925327	7.029880454	10.12041043	10.1401137	9.643130384	9.76644922
ENSG00000180198	RCC1	0.173498941	0.776910404	1.268196754	0.011308372	1.441695696	8.042902253	7.467835385	7.646393253	7.915493556	7.89452713	9.319337188	8.553793978
ENSG00000174325	DIRC1	2.777382728	0.415953852	-1.335735674	NA	1.441647055	2	2	2	3.515101962	3.39680241	2.048074405	2.225841198
ENSG00000159674	SPON2	4.432245561	7.02E-14	-2.992057847	8.05E-07	1.440187714	9.579430841	8.077869079	8.608698767	13.28530217	13.333318	6.189586148	5.73181021
ENSG00000143933	CALM2	0.079029916	0.925549897	1.361058578	0.044255311	1.440088494	12.06557695	10.71514053	10.6093157	11.38412223	11.3595809	12.63532708	12.66976376
ENSG00000151689	INPP1	0.585704816	0.753072123	0.853645868	0.633525288	1.439350684	2	7.669933291	7.691296561	7.638916932	7.72670378	7.934942996	7.958255135
ENSG00000106263	EIF3B	0.659177633	0.335863227	0.779400216	0.239064344	1.438577849	9.27634997	10.75272247	9.935792933	10.72738111	10.8187472	10.70950083	11.05711825
ENSG00000264230	ANXA8L1	0.728621256	0.79405451	0.708340691	0.794934423	1.436961948	2	2	3.369317972	2.947924294	2.77779907	3.10571689	2.551896972
ENSG00000139908	TSSK4	-2.312515245	0.59365851	3.748797708	0.299320086	1.436282463	2	2	2	2	2.14022021	4.311629396	3.956354764
ENSG00000091128	LAMB4	2.389898298	0.472233499	-0.954775133	NA	1.435123165	2	2	2	3.227757282	3.2184422	2.183373728	2.172608095
ENSG00000107890	ANKRD26	1.192835034	0.617138584	0.239975319	0.926858085	1.432810353	3.327711234	8.255926468	2	7.905589775	7.90685167	7.229857522	6.666875073
ENSG00000113712	CSNK1A1	0.827855572	0.026772451	0.603795783	0.114660271	1.431651355	10.31337612	9.664384941	10.26027233	10.91647777	10.9436312	10.74502644	10.66701521
ENSG00000166263	STXBP4	0.869195315	0.687246529	0.561050262	0.798439725	1.430245577	3.327711234	7.099070191	2	6.529309835	6.45450701	6.162543904	6.235386446
ENSG00000064687	ABCA7	0.461555301	0.793086452	0.967668431	0.551426737	1.429223732	2	6.389896633	6.958202216	6.483440992	6.67805337	7.414254101	6.622902297
ENSG00000055211	GINM1	0.873207979	0.192577616	0.54849531	0.426645605	1.421703288	9.219014269	7.954278496	9.413059136	9.851765671	9.8547535	9.685540753	9.355859082
ENSG00000048140	TSPAN17	0.333952478	0.691746353	1.085725779	0.143504721	1.419678257	7.82963488	8.255926468	9.528916236	8.996237745	9.11260313	9.695243935	9.903197088
ENSG00000154640	BTG3	0.861246365	0.052183296	0.55799092	0.222263411	1.419237286	8.855032352	8.522788974	8.076950877	9.397888985	9.35135081	9.256785236	8.863491214
ENSG00000151611	MMAA	1.082634973	0.364486576	0.334003092	0.796772666	1.416638065	6.366709671	6.666836837	3.369317972	7.080147179	7.05992818	6.609629839	6.033722345
ENSG00000146197	SCUBE3	-1.401374117	0.003403655	2.816668851	3.17E-10	1.415294733	10.98545464	10.73405387	9.907528187	9.176921588	9.25437635	13.42909294	13.42090046
ENSG00000104343	UBE2W	0.812306953	0.152981087	0.60172012	0.294411384	1.414027073	7.159102577	8.191702463	7.818184403	8.645348336	8.53866332	8.628756176	8.090601559
ENSG00000235718	MFRP	6.604703856	0.013055734	-5.1911387	0.110027396	1.413565156	2	2	2	6.592405944	6.77805036	2	2
ENSG00000035928	RFC1	0.422075165	0.469673243	0.989968242	0.058838964	1.412043407	8.228665467	8.929529568	9.426401271	9.336553206	9.38345569	9.934236219	9.916159841
ENSG00000157985	AGAP1	-0.354943038	0.765528102	1.766455666	0.081972081	1.411512628	9.081016759	6.78762695	6.718950348	7.659688863	7.59580267	9.624919616	9.822065034
ENSG00000007516	BAIAP3	0.960589052	0.653881546	0.447916702	0.840565911	1.408505754	2	4.11352228	2	3.79808662	3.74316441	3.616794016	3.209956562
ENSG00000068400	GRIPAP1	1.278317088	0.426620169	0.129649256	0.942926896	1.407966344	3.327711234	7.901717006	8.784770914	9.120242823	9.09762836	7.785203203	8.139104389
ENSG00000197021	CXorf40B	0.961075455	0.421979907	0.44687078	0.721847383	1.407946235	7.867441304	6.602424674	4.523675391	7.751013478	7.88209639	7.199581219	7.421437279
ENSG00000184588	PDE4B	3.658029975	0.027591082	-2.251839286	0.192951753	1.406190688	8.009475818	8.540087354	2	11.33538698	11.3820459	5.916072173	5.122735884
ENSG00000048028	USP28	1.374769857	0.361961292	0.031180275	0.985627735	1.405950132	4.815609471	9.055168103	6.203069917	9.069031178	9.10727282	7.817634286	7.701812481
ENSG00000166839	ANKDD1A	0.325350326	0.763884658	1.079851944	0.259132418	1.405202269	5.866944937	3.686303414	5.39244166	5.501219927	5.48637102	6.325014994	6.052261256
ENSG00000102934	PLLP	-2.677433158	0.311541535	4.081321192	0.072160727	1.403888034	2	2	4.058998049	2.3892346	2	6.444330878	5.777095613
ENSG00000075213	SEMA3A	4.434025939	0.002427568	-3.031905249	0.046484399	1.40212069	7.82963488	6.899080544	2	11.25064042	11.2638957	3.878466556	4.388595494
ENSG00000100997	ABHD12	0.974046616	0.023504727	0.426059232	0.358251684	1.400105848	9.689059027	9.102512015	8.845626171	10.24099634	10.2094139	9.746906545	9.608808761
ENSG00000175602	CCDC85B	0.43807655	0.290292991	0.961423588	0.010103248	1.399500137	8.468948322	8.395519306	9.031722608	9.052296807	9.12531607	9.588555735	9.629944775
ENSG00000132470	ITGB4	-0.24792847	0.882573218	1.646719013	0.23817122	1.398790543	2	4.710564154	5.39244166	4.804776789	3.7864959	5.72966459	6.329589667
ENSG00000176076	KCNE5	1.818453729	0.39609411	-0.419947643	0.859331521	1.398506086	2	3.686303414	2	4.237680128	3.69849104	2.775316226	2.632958241
ENSG00000166888	STAT6	1.251382143	0.15370945	0.146509939	0.886854075	1.397892082	10.58135316	10.57531312	8.408111533	11.38946169	11.400249	10.19365153	10.3849277
ENSG00000176720	BOK	0.118171837	0.907693271	1.278553681	0.122162715	1.396725518	9.820154628	8.88960062	7.646393253	9.146760056	9.16690126	10.08514941	10.50738357
ENSG00000139324	TMTC3	0.838286751	0.099683072	0.556713676	0.285220143	1.395000426	9.031919741	8.101365653	8.742726175	9.566077486	9.43372295	9.424097045	8.98571134
ENSG00000095319	NUP188	0.717488889	0.625823305	0.677333671	0.642345888	1.394822561	5.330594134	9.74864196	8.325066185	9.418058443	9.34954609	9.05871952	9.582583566
ENSG00000025772	TOMM34	0.656889175	0.43568023	0.737673219	0.364791599	1.394562393	9.128497779	8.540087354	7.098165388	9.064587625	9.19128891	9.089555166	9.320352741
ENSG00000006747	SCIN	-0.349116	0.895265695	1.742330461	0.444850169	1.393214461	2	2	4.523675391	2.947924294	3.28037825	5.158477451	3.640730416
ENSG00000112578	BYSL	0.35607883	0.818460344	1.035923597	0.457596529	1.392002427	7.750913381	6.666836837	3.369317972	7.035434774	7.19067478	7.747483829	7.81035357
ENSG00000236287	ZBED5	0.567393814	0.613157373	0.824596383	0.439516998	1.391990197	7.53297911	8.414402626	5.59507254	8.235106869	8.05595642	8.700544163	8.025476363
ENSG00000137871	ZNF280D	1.251533933	0.457673604	0.137462375	0.942145445	1.388996307	7.485143553	7.189546432	2	8.058664058	7.94320592	6.959905775	6.866539459
ENSG00000143811	PYCR2	0.483678808	0.738128035	0.905115067	0.503902094	1.388793875	8.609439662	8.469613413	4.523675391	8.43079185	8.52753255	8.902003555	8.892589222
ENSG00000111832	RWDD1	0.491798737	0.606852863	0.895662475	0.311515104	1.387461212	5.70932728	7.979858146	7.734844356	7.900612272	7.93841129	8.327733606	8.307525005
ENSG00000163798	SLC4A1AP	0.839573721	0.39058013	0.546619362	0.583098537	1.386193083	5.70932728	8.101365653	8.110401118	8.509731121	8.45385092	8.164905085	8.220043091
ENSG00000162377	COA7	0.147282294	0.905710932	1.23790996	0.231007098	1.385192254	5.532353208	5.595927442	7.777116083	6.83085985	6.84618961	7.948463298	7.854331622
ENSG00000155846	PPARGC1B	-0.907058336	0.649972951	2.291975461	0.193576	1.384917125	2	4.710564154	2	2.947924294	3.15372745	5.502666283	5.385063124
ENSG00000159348	CYB5R1	1.018129065	0.563160425	0.364768853	0.846218002	1.382897918	9.602033055	8.376385542	3.369317972	9.566077486	9.5427783	8.804787505	8.99962918
ENSG00000089505	CMTM1	0.459261904	0.851584865	0.923120377	0.687191321	1.382382282	2	5.72254323	2	4.846787657	4.68029874	5.371837532	4.935697922
ENSG00000088930	XRN2	1.38655917	0.00265282	-0.004594978	0.993931149	1.381964192	8.468948322	9.34816733	8.536706852	10.21867227	10.2394476	8.779646757	8.908956896
ENSG00000116990	MYCL	1.068673975	0.63692076	0.313032287	0.896941641	1.381706261	4.00722531	2	2	3.663521627	3.50452879	3.12825509	3.128433649
ENSG00000138138	ATAD1	0.777713839	0.131645633	0.603921854	0.246132276	1.381635693	8.87372952	9.308339287	8.296287027	9.645581273	9.67979376	9.661597094	9.294890064
ENSG00000023839	ABCC2	3.708523217	0.018902587	-2.327696826	0.168373716	1.380826391	2	2	4.874587708	6.710878132	6.78340763	2.492221014	2.466008509
ENSG00000170322	NFRKB	0.580937352	0.757563401	0.799136928	0.661209052	1.38007428	7.384433967	8.004992147	2	7.694616532	7.76240929	7.778094182	8.091498142
ENSG00000119661	DNAL1	0.741131022	0.254759301	0.638513901	0.323686171	1.379644923	6.73718411	6.140560021	7.398165132	7.64488235	7.45182838	7.247980242	7.629578661
ENSG00000180979	LRRC57	0.384070624	0.468786454	0.993677881	0.034406558	1.377748505	6.96351253	7.35507212	7.029880454	7.626911583	7.38291816	8.337438499	7.836259222
ENSG00000163320	CGGBP1	0.701783468	0.340527707	0.67505467	0.352263729	1.376838138	8.075571721	9.680074956	9.601288346	10.04910261	9.9102895	10.02786376	9.877696666
ENSG00000184270	HIST2H2AB	5.156998032	0.047659578	-3.780590748	0.238854949	1.376407284	2	2	2	5.334065616	5.33407135	2.048074405	2

ENSG00000138646	HERC5	4.993410556	0.006399324	-3.617901426	0.056674464	1.37550913	7.384433967	3.076665209	2	10.79901337	10.7874282	3.296906311	2.851942824
ENSG00000088367	EPB41L1	0.218500927	0.781202672	1.156329651	0.082638	1.374830577	9.081016759	7.60566235	7.934808822	8.543652346	8.58541725	9.426940628	9.560413054
ENSG00000179021	C3orf38	1.194016373	0.001824265	0.179909193	0.694591795	1.373925566	7.485143553	8.053983481	7.97167868	9.129845271	8.95180002	8.158075786	7.912312056
ENSG00000130528	HRC	-2.306155035	0.603854595	3.679349392	0.324412375	1.373194356	2	2	2	2.141737246	2	4.434275199	3.640730416
ENSG00000147905	ZCCHC7	0.839636411	0.550029831	0.532686065	0.712026005	1.372322477	7.33131396	8.297207175	4.058998049	8.221200057	8.15847021	8.069136662	7.680519528
ENSG00000017260	ATP2C1	1.403532336	0.003430894	-0.031767183	0.959231573	1.371765153	8.981092835	9.574900212	10.08627303	10.99096084	11.0379776	9.558011928	9.609747791
ENSG00000198860	TSEN15	0.017155117	0.974752037	1.354100349	0.00095192	1.371255467	7.096806373	6.535001748	6.80318718	6.907295206	6.75642009	8.189896068	8.087908463
ENSG00000158710	TAGLN2	0.047091251	0.941979106	1.32017456	0.009454427	1.367265811	11.92025849	11.21259083	11.95457585	11.73269717	11.8262615	12.5783741	13.4085426
ENSG00000007237	GAS7	0.49187094	0.715717569	0.874968264	0.489336405	1.366839204	4.00722531	7.431218269	6.073378139	6.882264028	6.92586261	7.586913431	6.879055607
ENSG00000198000	NOL8	1.005251372	0.391746117	0.361145665	0.774275034	1.366397037	5.095959458	8.433041974	7.398165132	8.548433777	8.47387021	7.845956781	7.906210156
ENSG00000198932	GPRASP1	0.423226861	0.820270883	0.942728832	0.582327511	1.365955693	7.667647223	6.899080544	2	7.206526483	7.14977896	7.6993745	7.672153316
ENSG00000107099	DOCK8	-1.685494736	0.547559684	3.051139857	0.200422954	1.36564512	2	3.076665209	2	2.270786764	2.14022021	4.927401733	3.383972333
ENSG00000136738	STAM	0.8045575	0.39960716	0.559438241	0.563920281	1.363995741	7.031698319	7.761270542	9.227351203	9.130908275	9.05949698	8.563064723	9.093466633
ENSG00000114062	UBE3A	0.440406244	0.270653422	0.923065399	0.01114802	1.363471643	9.159305284	9.514922632	8.960138144	9.712435568	9.63233954	10.30804289	9.980492541
ENSG00000133246	PRAM1	-1.317630003	0.811052763	2.679408937	NA	1.361778934	2	2	2	2	2.2680111	3.753554863	2.918126211
ENSG00000140577	CRTC3	-0.066473927	0.972436025	1.428044416	0.3943541	1.36157049	3.327711234	8.376385542	9.516493218	8.447952961	8.38855922	10.01865665	9.775403823
ENSG00000119487	MAPKAP1	0.538496117	0.266338693	0.822240598	0.071322601	1.360736714	8.631580145	9.28800521	9.528916236	9.682298904	9.78101395	9.875912956	10.1419604
ENSG00000001460	STPG1	-0.019137042	0.990169856	1.379863925	0.290178986	1.360726883	3.327711234	5.838937808	7.285536013	6.069927018	6.30607931	7.5848779	7.497817236
ENSG00000170581	STAT2	1.910923717	0.011653721	-0.550643497	0.518870344	1.36028022	9.064836028	10.57111193	11.0793996	12.37330564	12.3590431	9.645573761	10.1326262
ENSG00000078967	UBE2D4	-0.046581936	0.982078574	1.403401411	0.429681573	1.356819475	3.327711234	8.234834549	4.523675391	6.687949235	6.84106088	8.00436125	8.34108458
ENSG00000213030	CGB8	4.227093562	0.222198443	-2.873147706	0.450687102	1.353945856	2	2	2	4.299492648	4.72553021	2.094598369	2
ENSG00000165861	ZFYVE1	0.647958669	0.406678307	0.705658678	0.352303034	1.353617347	6.96351253	8.717821467	8.408111533	8.843805136	8.85843657	9.089196199	8.701457496
ENSG00000197818	SLC9A8	1.289202533	0.479814084	0.06325251	0.974738711	1.352455043	2	7.315436877	8.175061096	8.43768081	8.56378716	6.981729486	7.55613706
ENSG00000119912	IDE	1.079395607	0.321826745	0.271408808	0.821189708	1.350804415	8.696033109	8.639735838	5.772716771	9.29542901	9.20133	8.331380596	8.556393893
ENSG00000184574	LPAR5	-3.753073107	0.429843212	5.103853444	0.215047296	1.350780337	2	2	2	2	2	5.152992171	5.408276315
ENSG00000186532	SMYD4	0.554583697	0.467615355	0.796010886	0.268035588	1.350594583	6.73718411	6.78762695	5.59507254	7.097651217	6.92586261	7.583859056	6.811011855
ENSG00000150667	FSIP1	2.330116428	0.19061882	-0.982692834	0.609511386	1.347423595	2	4.11352228	2	5.022023783	4.70309173	2.746468522	2.671842496
ENSG00000100650	SRSF5	1.070491019	0.066558504	0.276300998	0.674724562	1.346792017	10.12333326	9.664384941	9.31604076	10.79800992	10.8145014	9.415246149	10.43664657
ENSG00000136003	ISCU	0.705866774	0.108937139	0.639944088	0.142663282	1.345810862	8.444143041	9.079034257	8.805342542	9.495204222	9.51406378	9.66376837	9.172702781
ENSG00000119541	VPS4B	0.829602835	0.194826666	0.514919321	0.434435201	1.344522156	8.19931886	9.13702746	7.777116083	9.354865403	9.28018559	9.056158084	8.953033422
ENSG00000149260	CAPN5	0.976454458	0.144865788	0.367810783	0.610962735	1.344265241	8.777722034	8.191702463	7.163363831	9.106258804	9.20332986	8.568225482	8.536120927
ENSG00000171408	PDE7B	1.749236283	0.18647032	-0.405396365	0.785325587	1.343839918	3.327711234	6.602424674	7.342949392	8.199071993	8.185025	6.518722958	5.519049808
ENSG00000102096	PIM2	0.689218553	0.50100135	0.651567895	0.518473647	1.340786448	6.009025997	7.051611315	4.523675391	6.946463121	6.81514011	6.892381078	6.797888492
ENSG00000103168	TAF1C	0.58195562	0.758330669	0.758707361	0.680152864	1.340662981	2	7.979858146	7.451345288	7.762033056	7.71833702	7.862014947	7.964141273
ENSG00000186416	NKRF	0.515584257	0.717276749	0.823598215	0.541656025	1.339182472	3.327711234	7.232742069	7.098165388	7.230526165	7.03319408	7.399274054	7.470508284
ENSG00000132950	ZMYM5	1.047587954	0.338720894	0.291281792	0.807912915	1.338869747	4.815609471	7.393647515	5.772716771	7.437142666	7.40393901	6.952030889	6.362645675
ENSG00000263091		6.529182539	0.016750741	-5.191137785	0.116242229	1.338044753	2	2	2	6.716553866	6.507263	2	2
ENSG00000128563	PRKRIP1	0.708902101	0.432702068	0.629050326	0.481058497	1.337952427	6.257048221	7.051611315	8.35328246	8.192977693	8.15020018	8.035250217	8.148612304
ENSG00000139182	CLSTN3	0.807572614	0.311127936	0.529393998	0.516505072	1.336966612	8.16936288	7.099070191	9.014156722	9.060130342	9.11366683	8.789402187	8.832722224
ENSG00000138698	RAP1GDS1	0.373397181	0.374982962	0.961892657	0.010215226	1.335289838	8.696033109	8.717821467	8.536706852	8.989221226	9.05839257	9.860850997	9.303427654
ENSG00000130711	PRDM12	-0.727834839	NA	2.062642664	NA	1.334807825	2	2	2	2.141737246	2.2680111	3.316666208	2.70970617
ENSG00000121406	ZNF549	0.201396428	0.892276535	1.132121948	0.375035076	1.333518376	4.467311984	7.232742069	4.874587708	6.29903474	6.27599024	7.5848779	6.622902297
ENSG00000174718	KIAA1551	1.59802648	0.216138361	-0.265113279	0.856832448	1.3329132	4.467311984	8.317412869	8.175061096	9.299216392	9.29432703	7.81936672	6.973513842
ENSG00000100439	ABHD4	-0.302406377	0.645053038	1.634518405	0.002808849	1.332112028	9.423197387	8.717821467	9.897982195	9.091045942	9.14937381	11.31477425	10.72478611
ENSG00000198792	TMEM184B	0.204291317	0.755058981	1.127428756	0.041403012	1.331720073	11.24442723	10.20962523	10.26027233	10.83337521	10.8819159	11.56276827	11.96792586
ENSG00000196141	SPATS2L	1.003066049	0.009469798	0.328010493	0.445736555	1.331076542	10.41898845	10.66082477	9.91701143	11.34549558	11.3895304	10.59558475	10.78602048
ENSG00000131849	ZNF132	0.730629218	0.734036455	0.599560837	0.781762153	1.330190055	2	3.686303414	7.225742779	6.449761039	6.49425375	6.589466407	6.055940621
ENSG00000197024	ZNF398	0.311820074	0.863136627	1.017904937	0.52813899	1.329725011	6.653098118	8.57407404	3.369317972	7.662632018	7.67863747	8.448514034	8.277899908
ENSG00000214338	SOGA3	4.704047971	7.47E-06	-3.374993898	0.003522037	1.329054073	3.327711234	5.131603625	2	8.446246007	8.49850989	2.34604626	2.466008509
ENSG00000197385	ZNF860	1.123722657	0.624036401	0.204073846	0.934813535	1.327796503	2	4.11352228	2	4.205751501	3.45167086	2.911488689	3.560117069
ENSG00000123496	IL13RA2	6.518686237	0.017140458	-5.191137724	0.11699998	1.327548512	2	2	2	6.535745081	6.67228943	2	2
ENSG00000010017	RANBP9	0.66743219	0.689816154	0.658822683	0.689674098	1.326254873	9.48530526	9.018611864	4.058998049	9.352133347	9.38433737	9.332135462	9.386865301
ENSG00000102984	ZNF821	1.276527354	0.394463454	0.047459591	0.97742548	1.323986965	4.00722531	5.72254323	2	5.764940652	5.81407719	4.742549839	4.644600858
ENSG00000134186	PRPF38B	0.517293757	0.573772614	0.806683812	0.353054225	1.323977569	8.444143041	8.702538795	6.629488387	8.705093576	8.66738547	9.242652114	8.641501544
ENSG00000112739	PRPF4B	1.027931166	0.443895169	0.295110562	0.839017584	1.323041728	5.532353208	9.461450882	9.413059136	9.925905286	9.91944253	9.133737044	9.251427401
ENSG00000198028	ZNF560	2.64071853	0.619792462	-1.317696067	NA	1.323022463	2	2	2	3.023133209	3.65239003	2.267001569	2
ENSG00000049759	NEDD4L	0.897021324	0.585414444	0.424957284	0.804379534	1.321978608	3.327711234	8.147241016	5.930865648	7.759286043	7.78127547	7.153627862	7.448282715
ENSG00000097096	SYDE2	-0.625910628	0.561312002	1.94297005	0.037743848	1.317059421	3.327711234	5.457120953	5.59507254	4.387512679	4.72553021	7.198250335	6.567271433
ENSG00000128989	ARPP19	0.165278394	0.693142866	1.150624343	0.000727777	1.315902737	10.32700827	9.770792087	9.897982195	10.23309841	10.1280575	11.28232575	11.03596654
ENSG00000107864	CPEB3	1.193248963	0.616474704	0.122408887	0.963128357	1.31565785	2	6.228547981	2	5.997179931	5.78232107	4.971696071	4.878668439
ENSG00000050730	TNIP3	5.278658201	3.43E-08	-3.963986266	0.000493944	1.314671935	4.467311984	2	4.058998049	8.562683721	8.50824913	2.183373728	2.225841198
ENSG00000175931	UBE2O	0.767217738	0.251320848	0.547414142	0.420661534	1.314631879	8.998234871	7.928237109	7.600047325	9.066811112	9.05839257	8.879345195	8.808934503
ENSG00000025293	PHF20	-0.338398805	0.546723851	1.65214476	0.000435758	1.313745954	9.384607364	8.414402626	8.699419242	8.551612617	8.55441692	10.69494761	10.34826401
ENSG00000196502	SULT1A1	0.173054131	0.91483416	1.138493556	0.417064406	1.311547686	2	5.303523747	5.59507254	5.003571641	5.18011485	5.381251181	6.394961217
ENSG00000171634	BPTF	0.549226425	0.249172977	0.760810055	0.09315428	1.31003648	8.981092835	9.792607274	9.798878489	10.09557938	10.1442836	10.43310438	10.22110577
ENSG00000150961	SEC24D	0.684985163	0.568972239	0.620813126	0.604252415	1.305798289	8.107517749	11.47791526	10.46161637	11.22249509	11.2720171	11.24050906	11.12393198
ENSG00000197785	ATAD3A	0.875196511	0.035423229	0.430458309	0.329501828	1.30565482	7.867441304	7.979858146	7.285536013	8.625876185	8.6021877	8.05817395	8.282618188
ENSG00000188629	ZNF177	3.116275225	0.307253487	-1.81071869	0.582427016	1.305556535	2	2	2	3.754611435	3.60476711	2.139668783	2.059860178
ENSG00000136628	EPRS	0.310691232	0.848581742	0.992799923	0.495779443	1.303491155	6.009025997	10.54992047	10.67785662	10.36226743	10.3769158	11.13151297	10.96374572
ENSG00000266967	AARSD1	2.288202724	0.0437622	-0.985071943	0.424233954	1.303130781	4.815609471	4.710564154	2	6.358325776	6.34282908	3.851628042	3.261853426
ENSG00000157350	ST3GAL2	0.59452867	0.561228414	0.707605596	0.476093865	1.302134266	7.27616306	9.04308532	9.91701143	9.697445926	9.68838639	9.750316091	9.858600332
ENSG00000177125	ZBTB34	0.625263369	0.495809834	0.676569455	0.448338304	1.301832825	7.82963488	5.838937808	7.734844356	7.915493556	8.03365075	8.261735106	7.744618542
ENSG00000134884	ARGLU1	0.426615264	0.262230813	0.874912052	0.011674378	1.301527317	8.609439662	9.13702746	8.677268317	9.215512556	9.29994502	9.76229903	9.643457842
ENSG00000104081	BMF	1.206977699	0.066640853	0.094242073	0.906201712	1.301219772	7.709880939	7.099070191	6.322058544	8.300810597	8.37087451	7.525623979	6.89352185
ENSG00000138942	RNF185	0.753552529	0.362156704	0.547631922	0.513597151	1.301184451	7.27616306	9.256956408	8.922968322	9.431062134	9.48147379	9.139295883	9.35623215
ENSG00000140941	MAP1LC3B	0.674155513	0.014015823	0.626349588	0.022878027	1.300505101	10.88304938	10.54564457	10.93284392	11.44991178	11.4855078	11.43709348	11.40286138
ENSG00000037757	MRI1	0.98618286	0.026148248	0.313945787	0.523269622	1.300128647	6.96351253	6.602424674	7.225742779	7.900612272	7.89946959	7.366471135	7.105417192
ENSG00000130717	UCK1	0.410851794	0.832844524	0.887329259	0.621823406	1.298181053	5.330594134	9.00621767	4.058998049	7.985332082	7.9809999	8.586144131	8.30675314
ENSG00000118495	PLAGL1	-0.256252962	0.610659792	1.553473465	0.000161735	1.297220504	8.586954094	9.396454291	9.031722608	8.814994458	8.77079897	10.76043889	10.40454019
ENSG00000188895	MSL1	0.163067369	0.832844524	1.133431982	0.075265997	1.296499352	8.418903792	9.770792087	9.849281147	9.596407142	9.68338024	10.73840307	10.46321306
ENSG00000158315	RHBDL2	1.567223455	0.079559698	-0.270954938	0.793999085	1.296268518	4.467311984	4.11352228	5.772716771	6.172600389	6.53928196	4.667592615	4.729989799
ENSG00000132970	WASF3	0.748993669	0.631428733	0.543059568	0.730619275	1.292053237	3.327711234	8.124485672	7.451345288	8.03841338	7.98565528	7.561260509	8.021718375
ENSG00000139433	GLTP	0.094509813	0.92127192	1.194993879	0.127181705	1.289503692	9.473095744	9.225224669	7.502634626	9.025111392	9.07923325	9.956251889	10.3156405
ENSG00000137558	PI15	3.187522367	0.520929186	-1.898269048	0.714892595	1.289253319	2	2	2	4.358765249	2.59489879	2.183373728	2
ENSG00000188191	PRKAR1B	-0.623322183	0.738744016	1.912289698	0.252976607	1.288967515	7.82963488	7.954278496	2	6.682159616	6.75642009	9.203388342	9.215277965
ENSG00000064932	SBNO2	4.87138545	2.78E-06	-3.582558676	0.001115074	1.288826774	6.257048221	5.303523747	2	10.05078854	10.0529704	2.803598391	2.817676617
ENSG00000160094	ZNF362	-0.115443843	0.853173474	1.403617473	0.003902794	1.28817363	7.218819781	7.819099893	8.143093325	7.677258395	7.64666005	9.39534863	8.884335219
ENSG00000135365	PHF21A	0.706991528	0.444373895	0.578851208	0.529459204	1.285842737	7.218819781	8.955547846	9.528916236	9.506715015	9.59550527	9.537898028	9.301104226
ENSG00000108349	CASC3	0.063208646	0.887804971	1.220828874	0.000290492	1.28403752	9.081016759	9.632483534	9.658943166	9.496029474	9.59321927	10.75343304	10.64198913
ENSG00000153786	ZDHHC7	0.201159824	0.677597403	1.082744095	0.00728342	1.283903919	9.048471759	9.763446433	9.196533591	9.539906319	9.60989979	10.32155264	10.57433282
ENSG00000114416	FXR1	0.616203858	0.437089015	0.666183631	0.385866049	1.282387489	9.358295023	10.26789142	8.460932916	10.15269291	10.1721198	10.34594659	10.06485442
ENSG00000181220	ZNF746	0.608965037	0.628451704	0.673350024	0.583522797	1.282315061	8.107517749	8.255926468	4.874587708	8.274117511	8.27334517	8.459665302	8.203551241
ENSG00000153956	CACNA2D1	1.315333476	0.09651388	-0.036280733	0.969875015	1.279052743	6.653098118	8.414402626	8.536706852	9.404936476	9.36927498	7.765568476	8.285755157
ENSG00000023228	NDUFS1	0.333020735	0.80400762	0.945803147	0.434676981	1.278823882	10.43166252	8.317412869	7.225742779	9.573133444	9.62340548	10.14267068	10.27181939
ENSG00000213689	TREX1	7.431041385	1.07E-06	-6.152947209	0.007313014	1.278094176	3.327711234	2	2	8.347282003	8.51952909	2	2
ENSG00000107362	ABHD17B	0.888628652	0.472233499	0.386905027	0.765722308	1.275533678	7.579279349	6.899080544	4.058998049	7.626911583	7.64666005	7.309674888	6.963754056
ENSG00000180104	EXOC3	0.464319523	0.632226513	0.8108536	0.370531531	1.275173123	6.653098118	8.234834549	9.031722608	8.736160353	8.71722214	8.99461439	9.142307541
ENSG00000114739	ACVR2B	0.587447774	0.835281211	0.687415517	0.80197737	1.274863291	2	7.431218269	2	6.476767609	6.48112613	6.797131715	6.311234697
ENSG00000185482	STAC3	0.270502426	0.910353949	1.003483677	0.640556462	1.273986103	2	4.442710129	2	3.566290607	3.55551834	4.434275199	3.620995423
ENSG00000137563	GGH	0.289102994	0.897634669	0.982428951	0.630693854	1.271531944	8.998234871	6.728495673	2	7.823812411	8.11663771	8.518969664	8.792487762
ENSG00000213024	NUP62	0.585000677	0.041502924	0.686417874	0.013709533	1.271418551	8.716893051	8.806266215	8.960138144	9.381905119	9.43882443	9.425519537	9.592751029
ENSG00000244693	CTAGE8	6.461139415	0.020346777	-5.191137047	0.12122066	1.270002368	2	2	2	6.598566398	6.50077304	2	2
ENSG00000140326	CDAN1	0.85835598	0.579168793	0.41117404	0.79903783	1.26953002	4.467311984	7.790474954	4.523675391	7.315259362	7.32531744	7.131485609	6.590289384
ENSG00000214212	C19orf38	0.161627712	0.954276444	1.106667846	0.669090392	1.268295557	2	3.686303414	2	3.09461492	2.77779907	3.956110469	2.746601469
ENSG00000253797	UTP14C	2.009682769	0.028016568	-0.741648019	0.459674606	1.268034751	6.96351253	8.522788974	6.073378139	9.539105795	9.54672161	6.892381078	6.775747403
ENSG00000166923	GREM1	9.892013099	9.06E-05	-8.626227425	0.002061106	1.265785674	2	2	7.646393253	15.91267495	15.9260404	2	2.117335275
ENSG00000187257	RSBN1L	0.499191645	0.355599712	0.766280887	0.129182035	1.265472532	6.96351253	6.602424674	7.098165388	7.315259362	7.43490694	7.877896341	7.348307474

ENSG00000106608	URGCP	0.122473556	0.755348516	1.142525267	0.000248821	1.264998823	8.468948322	8.929529568	8.608698767	8.74312865	8.87484109	9.776414628	9.870140686
ENSG00000132256	TRIM5	0.714338675	0.056070605	0.55038726	0.145899169	1.264725935	9.590776211	9.608085847	10.16000817	10.52717074	10.5141456	10.26324148	10.44543965
ENSG00000162976	PQLC3	0.12828678	0.936431891	1.135486397	0.41910268	1.263773177	3.327711234	7.761270542	7.163363831	7.026323566	7.12888795	8.203889907	7.909264332
	C8ORF4	6.454709124	0.020858537	-5.191136926	0.122048565	1.263572198	2	2	2	6.622948477	6.46120797	2	2
ENSG00000188811	NHLRC3	0.887478925	0.141958529	0.375875142	0.559807045	1.263354067	6.468621203	6.728495673	7.163363831	7.64488235	7.70428369	7.512833218	6.732719555
ENSG00000172037	LAMB2	0.12525297	0.872778119	1.137554932	0.073580128	1.262807902	11.45462681	12.18137127	12.98461505	12.43032753	12.4993288	13.44378564	13.5095186
ENSG00000118777	ABCG2	0.994336921	0.679435427	0.267847808	0.916676409	1.262184729	2	2	4.058998049	3.566290607	3.55551834	3.374372158	2.817676617
ENSG00000165046	LETM2	0.950913582	0.540002704	0.309563122	0.852566533	1.260476704	2	6.04685525	4.523675391	5.829220145	5.87556634	5.269098128	5.263040529
ENSG00000248871	TNFSF12-TN	6.45062145	0.020985554	-5.191136917	0.12214799	1.259484533	2	2	2	6.490083648	6.5890786	2	2
ENSG00000185900	POMK	0.315173632	0.896905654	0.942240171	0.673457912	1.257413803	7.218819781	2	3.369317972	6.024891728	6.08095082	6.508020621	6.777976866
ENSG00000120709	FAM53C	0.438291171	0.444381183	0.818634548	0.119894702	1.256925718	8.39321512	9.479495904	8.742726175	9.347568393	9.42432312	9.811002128	9.717051741
ENSG00000186230	ZNF749	0.815707374	0.672951696	0.438549691	0.825605649	1.254257065	4.00722531	2	6.322058544	5.764940652	5.78232107	5.744329515	5.020995508
ENSG00000177156	TALDO1	1.428315628	0.084560698	-0.17414904	0.857985777	1.254166588	10.87842776	9.506147172	8.884815469	11.4071185	11.4507864	9.577081455	10.04670882
ENSG00000117724	CENPF	0.902162979	0.479099956	0.351611266	0.794825276	1.253774244	5.70932728	6.389896633	3.369317972	6.761173376	6.1322378	5.376552034	6.374480702
ENSG00000120688	WBP4	0.749823083	0.182445823	0.503108989	0.379197781	1.252932072	6.468621203	7.60566235	7.163363831	7.922877029	7.87709394	7.552954291	7.755972999
ENSG00000142733	MAP3K6	-0.22635506	0.780859156	1.478136525	0.028009055	1.251781464	6.96351253	6.228547981	7.818184403	6.739036112	7.06873058	8.647894167	8.517561736
ENSG00000112992	NNT	0.287057887	0.473839036	0.964128369	0.005328635	1.251186256	8.418903792	8.955547846	8.904018013	9.063474594	9.07049489	9.627394005	9.844575759
ENSG00000178950	GAK	0.907126694	0.405114539	0.344048874	0.767139907	1.251175569	7.667647223	8.414402626	10.31867969	10.07473944	10.2153718	9.581682124	9.590533012
ENSG00000059769	DNAJC25	0.833172492	0.266620128	0.417428792	0.595040124	1.250601284	6.89194353	5.457120953	6.958202216	7.426792977	7.29937545	7.141214687	6.753261212
ENSG00000074800	ENO1	0.381845511	0.62849957	0.86860756	0.227933253	1.250453071	13.50647618	12.46336633	12.12587352	13.1870944	13.2239193	13.18080919	14.06901306
ENSG00000129484	PARP2	0.72513945	0.706120595	0.525041335	0.787590496	1.250180785	6.009025997	7.928237109	2	7.391747748	7.43490694	7.382965824	7.032593922
ENSG00000214087	ARL16	0.071420768	0.981947437	1.178401278	0.677268519	1.249822045	2	4.442710129	2	4.034562184	2.2680111	4.652122235	3.496556178
ENSG00000104331	IMPAD1	0.52689232	0.558026599	0.722033677	0.401569414	1.248925997	9.771132748	11.22333918	9.301632639	10.88783887	10.8525899	10.86928913	11.23743934
ENSG00000107021	TBC1D13	0.454709684	0.596370107	0.79310466	0.321893604	1.247814344	6.89194353	8.414402626	8.904018013	8.700804887	8.77752611	9.05908615	9.091227191
ENSG00000119599	DCAF4	0.306014985	0.682009332	0.936932636	0.153226253	1.242947621	5.866944937	6.78762695	5.59507254	6.567497652	6.42738748	6.950450741	7.24894374
ENSG00000136636	KCTD3	0.565623692	0.479092457	0.676703693	0.377779769	1.242327385	6.89194353	8.255926468	8.721235202	8.729158235	8.66883353	8.632702669	8.966780003
ENSG00000135148	TRAFD1	0.604752538	0.625382427	0.637568344	0.599804676	1.242320882	6.366709671	9.792607274	9.149036566	9.565291356	9.64195608	9.506788283	9.756025697
ENSG00000171160	MORN4	0.294653693	0.820270883	0.947535653	0.411135188	1.242189346	5.532353208	7.538394143	4.874587708	6.766655021	6.73446058	7.607112326	7.119550903
ENSG00000147144	CCDC120	0.045756452	0.972563056	1.193750536	0.292297008	1.239506988	4.467311984	6.78762695	4.523675391	5.910700032	5.62411716	6.879153024	6.847559257
ENSG00000126264	HCST	1.583146848	0.705820174	-0.344491346	0.938709627	1.238655501	2	3.076665209	2	3.023133209	3.55551834	2.831336763	2
ENSG00000128585	MKLN1	1.593595479	1.48E-05	-0.355172744	0.405823254	1.238422735	9.097018025	9.13702746	9.703463497	10.9050282	10.9439303	9.1257085	8.827350262
ENSG00000187808	SOWAHD	-3.753052045	0.464100906	4.990667867	0.267778932	1.237615822	2	2	2	2	2	3.943456545	5.842465082
ENSG00000261852		6.428088805	0.022632059	-5.191136546	0.124325213	1.236952259	2	2	2	6.622948477	6.40670796	2	2
ENSG00000135094	SDS	-3.753041652	0.477619809	4.989068695	0.285220143	1.236027043	2	2	2	2	2	5.948096914	3.45257121
ENSG00000198890	PRMT6	0.491246344	0.580694192	0.744570475	0.373980691	1.235816819	5.095959458	6.899080544	6.80318718	6.946463121	6.94988431	6.845542321	7.474637727
ENSG00000172534	HCFC1	0.382702769	0.497766732	0.851706864	0.095670604	1.234409633	10.09173937	9.102512015	9.287079172	9.898079272	9.97960581	10.29362669	10.51174389
ENSG00000197162	ZNF785	0.534270216	0.7112679	0.699645659	0.614845785	1.233915875	7.27616306	3.686303414	6.073378139	6.809774134	6.77267312	7.349785676	6.397863414
ENSG00000108671	PSMD11	1.070589285	0.410778027	0.162315296	0.911555747	1.23290458	8.963744667	10.13758676	6.203069917	10.17141133	10.2549564	9.132692373	9.467467793
ENSG00000134897	BIVM	1.404561463	0.001667272	-0.172041986	0.751485356	1.232519476	6.653098118	7.467835385	7.029880454	8.509731121	8.45552985	7.046766788	6.81969491
ENSG00000135931	ARMC9	0.774999784	0.468786454	0.457500279	0.677816167	1.232500063	7.159102577	8.147241016	9.788583153	9.504256136	9.57170671	9.32818621	9.109046142
ENSG00000196793	ZNF239	0.864176831	0.732095163	0.367321646	0.889682343	1.231498477	2	6.389896633	2	5.720439317	5.76075522	5.650128416	4.80186789
ENSG00000156973	PDE6D	0.277087405	0.905443737	0.952157694	0.652626737	1.229245099	2	5.838937808	8.805342542	7.659688863	7.68723697	8.222339682	8.44505627
ENSG00000197928	ZNF677	-0.998317719	0.34076194	2.227299702	0.017280596	1.228981984	4.467311984	6.464272742	4.523675391	4.670864177	4.60967607	7.780764172	7.491038307
ENSG00000177239	MAN1B1	0.538537864	0.398531703	0.689184858	0.259166961	1.227722723	10.36053552	9.616264339	9.031722608	10.25422683	10.3604317	10.57522267	10.33147536
ENSG00000108639	SYNGR2	-0.921760439	0.099215121	2.148909569	1.87E-05	1.22714913	8.564112515	9.814097497	9.344432377	8.434240442	8.40255255	11.50163612	11.44626306
ENSG00000071655	MBD3	0.412606487	0.544427162	0.813229475	0.194894738	1.225835962	9.143983763	8.929529568	10.22252928	9.934453939	9.97960581	10.40871423	10.30214935
ENSG00000062282	DGAT2	2.226664638	0.328037547	-1.001644031	0.679226319	1.225020607	2	5.457120953	2	6.006476466	6.19790195	3.981090143	2.592996859
ENSG00000110711	AIP	0.343487372	0.557439662	0.879481937	0.094200705	1.222969309	8.285626337	8.395519306	7.285536013	8.444537031	8.38327653	8.985384832	8.903351429
ENSG00000156162	DPY19L4	0.932702663	0.355475457	0.289899631	0.792195215	1.222602294	5.70932728	8.356994601	7.502634626	8.532433657	8.41468635	8.026277797	7.628343587
ENSG00000112655	PTK7	0.379374176	0.717288969	0.84276378	0.381294572	1.222137956	8.586954094	9.181799937	10.80076732	10.19141925	10.2409086	10.98311289	10.29165501
ENSG00000131724	IL13RA1	0.190993908	0.879872883	1.030687472	0.340611737	1.221681381	7.867441304	9.114109157	10.78012988	9.9332358	9.90292502	10.85056146	10.65311485
ENSG00000181704	YIPF6	0.548785042	0.653114396	0.671885752	0.570388322	1.220670794	9.867565369	8.762721501	6.534109959	9.504256136	9.44136842	9.676966742	9.509331338
ENSG00000197119	SLC25A29	0.011884056	0.996696604	1.208047455	0.555606024	1.219931511	3.327711234	8.981105197	4.058998049	7.514115926	7.46188679	8.628756176	8.702631562
ENSG00000053747	LAMA3	2.092431071	0.357081274	-0.8736922	0.718279305	1.218738871	2	2	6.073378139	6.401247806	6.64900057	3.782262616	4.077147165
ENSG00000125630	POLR1B	0.483880942	0.811736938	0.734529546	0.704080508	1.218410488	6.563806279	8.317412869	2	7.471115032	7.71833702	7.875400346	7.815789075
ENSG00000174652	ZNF266	0.226337215	0.894519627	0.990930822	0.509248144	1.217268037	3.327711234	8.124485672	7.777116083	7.714607209	7.53992502	8.419110791	8.344849238
ENSG00000205659	LIN52	1.057953528	0.484134885	0.158201208	0.925792761	1.216154736	3.327711234	7.145017454	8.143093325	8.22518713	8.20112674	7.373563392	7.285445324
ENSG00000205060	SLC35B4	0.42468515	0.64079571	0.791317758	0.350276926	1.216002908	8.285626337	8.557180776	6.534109959	8.461536372	8.43865202	8.52641307	9.052154518
ENSG00000187026	KRTAP21-2	6.406676273	0.023816364	-5.191136358	0.125696822	1.215539915	2	2	2	6.436065776	6.55815769	2	2
ENSG00000118965	WDR35	0.513544122	0.706615573	0.701785344	0.591567059	1.215329466	3.327711234	6.728495673	6.80318718	6.799114572	6.68379438	6.552446484	7.219718817
ENSG00000185215	TNFAIP2	1.607488718	0.001272822	-0.393426114	0.492233435	1.214062604	9.204317094	9.949724236	9.624627483	11.20241092	11.2557283	8.747705648	9.599699825
ENSG00000117262	GPR89A	2.125124191	0.01309428	-0.911151284	0.327334994	1.213972907	7.096806373	5.303523747	5.156626401	8.157948442	8.22494733	5.217477917	5.361470313
ENSG00000063761	ADCK1	0.132299465	0.94581868	1.081572881	0.535270007	1.213872346	4.00722531	6.464272742	2	5.289065324	5.36298494	6.075339068	6.323497254
ENSG00000124596	OARD1	0.842331764	0.561188023	0.370004329	0.808290096	1.212336093	4.467311984	7.232742069	4.058998049	6.766655021	6.84618961	6.442083733	6.261114822
ENSG00000140939	NOL3	0.510329087	0.625823305	0.699772728	0.484010617	1.210101816	8.009475818	5.457120953	7.552162999	7.823812411	7.85436579	8.084345843	7.964141273
ENSG00000185973	TMLHE	0.510790208	0.785196727	0.698778403	0.698000726	1.20956861	3.327711234	7.60566235	4.058998049	6.744602355	6.67805337	6.850634117	6.934072182
ENSG00000144746	ARL6IP5	0.148820374	0.699491428	1.060461035	0.000697154	1.20928141	10.36053552	9.962611189	10.06936278	10.31581664	10.2588077	11.01180608	11.36127648
ENSG00000144635	DYNC1LI1	0.453441296	0.352000159	0.755263567	0.095600702	1.208704864	8.564112515	8.029695761	7.691296561	8.616799622	8.5575471	8.782757919	8.983298084
ENSG00000166860	ZBTB39	0.743440654	0.547789118	0.464155309	0.715036782	1.207595963	4.00722531	6.602424674	7.451345288	7.242377933	7.36158648	7.157741966	6.89352185
ENSG00000063245	EPN1	0.206286405	0.678236128	1.000474967	0.017257913	1.206761372	10.16946352	10.02537381	9.358421373	10.06698504	10.1301615	10.77032462	11.00215671
ENSG00000214518	KRTAP2-2	6.39723104	0.024565732	-5.191136201	0.126534939	1.206094839	2	2	2	6.573765157	6.39974839	2	2
ENSG00000145868	FBXO38	0.616711396	0.735527035	0.588203517	0.744872588	1.204914913	3.327711234	7.393647515	9.25752426	8.542055007	8.74768919	8.72431761	8.508191835
ENSG00000013573	DDX11	0.395491876	0.817474674	0.809328711	0.610398832	1.204820587	6.89194353	5.946638857	2	6.306580752	6.32824132	6.757921389	6.666875073
ENSG00000164754	RAD21	0.906442989	0.421979907	0.297367984	0.806378761	1.203810974	7.096806373	10.019219	9.981704053	10.46530827	10.3577408	10.04073005	9.524680706
ENSG00000135185	TMEM243	0.935304702	0.108937139	0.267982346	0.678808079	1.203287047	6.563806279	7.002537973	5.930865648	7.52061915	7.46188679	7.018390248	6.6426114
ENSG00000159261	CLDN14	3.013777299	0.340147427	-1.811414342	0.593306846	1.202362957	2	2	2	3.881293667	3.28037825	2.139668783	2.059860178
ENSG00000136002	ARHGEF4	0.319437888	0.809107998	0.881907257	0.460053783	1.201345145	3.327711234	6.535001748	6.534109959	6.260701031	6.40670796	7.007793418	6.728114749
ENSG00000137309	HMGA1	1.457476422	0.02162451	-0.257309585	0.728468477	1.200166836	12.23783517	10.89059638	10.9968459	12.95434551	12.9845144	11.20292911	11.30788843
ENSG00000177614	PGBD5	0.058134433	0.979836893	1.14146624	0.568016472	1.199600673	2	3.076665209	4.874587708	3.566290607	4.02226117	5.31402805	3.430065404
ENSG00000125534	PPDPF	0.317595519	0.528648153	0.881134787	0.048142698	1.198730306	9.848787621	9.687856435	10.53735535	10.39449089	10.3808971	11.00144677	10.89633773
ENSG00000185527	PDE6G	-0.689500438	0.806161404	1.887553383	0.436601068	1.198052945	2	2	3.369317972	2.270786764	2.49394783	3.709392871	3.261853426
ENSG00000166987	MBD6	0.39437071	0.310604101	0.802948057	0.023498087	1.197318767	7.709880939	8.234834549	8.206335842	8.47833774	8.44880239	8.917453109	8.817088095
ENSG00000110324	IL10RA	-3.753061303	0.45109867	4.950042938	0.25649001	1.196981634	2	2	2	2	2	5.532544554	4.624919101
ENSG00000175893	ZDHHC21	0.802867199	0.673891173	0.393379168	0.842165885	1.196246367	2	6.951736442	8.206335842	7.990026167	7.85436579	7.401588825	7.633277551
ENSG00000204071	TCEAL6	6.386627013	0.025128901	-5.191136117	0.127181705	1.195490896	2	2	2	6.529309835	6.42738748	2	2
ENSG00000143753	DEGS1	0.487663813	0.296880612	0.707280041	0.108707597	1.194943853	11.24801458	10.51971956	10.48092713	11.26741277	11.2940008	11.46066561	11.53840508
ENSG00000091009	RBM27	1.532065852	0.322947052	-0.337211671	0.8452659	1.194854181	3.327711234	8.487557258	6.882775709	8.894758143	8.82115545	7.127295835	6.907844475
ENSG00000100889	PCK2	-0.909509019	0.622871051	2.10366349	0.21055748	1.194154471	7.096806373	8.147241016	2	6.252910529	6.29861567	9.49599671	8.884335219
ENSG00000107938	EDRF1	1.071471277	0.124202911	0.122568935	0.882372947	1.194040212	5.532353208	6.951736442	6.80318718	7.574744049	7.6318872	6.924930115	6.389139248
ENSG00000108588	CCDC47	1.046741446	0.118180147	0.146944645	0.850843239	1.193686092	8.855032352	8.834582582	10.14394518	10.44792714	10.4653998	9.445288547	9.667106106
ENSG00000168229	PTGDR	3.498201202	0.410778027	-2.305529472	0.608101226	1.19267173	2	2	2	4.139692846	3.74316441	2.139668783	2
ENSG00000122068	FYTTD1	0.643295307	0.433957193	0.549214404	0.500662365	1.192509711	8.19931886	9.828248506	10.11951029	10.28176567	10.1828016	10.13590886	10.14282564
ENSG00000109111	SUPT6H	0.994403086	0.200056839	0.197560017	0.821567233	1.191963103	9.304185368	9.988044813	11.14543241	11.32775878	11.3528401	10.50900237	10.58041299
ENSG00000131043	AAR2	0.207705645	0.925454021	0.983888918	0.625198138	1.191594563	9.317904228	8.732943944	2	8.598473365	8.75996988	9.352020423	9.545113933
ENSG00000103249	CLCN7	1.852015065	0.044720075	-0.660486039	0.513707623	1.191529025	9.720386367	9.114109157	7.029880454	10.81464371	10.8602152	8.353668905	8.325926932
ENSG00000178502	KLHL11	1.107943816	0.365386306	0.083289212	0.952652139	1.191233028	5.330594134	7.431218269	4.523675391	7.370304568	7.4074129	6.575182432	6.199243628
ENSG00000160233	LRRC3	-1.262213643	0.343731455	2.450841418	0.042225848	1.188627775	3.327711234	6.311476754	4.058998049	4.070463721	4.1930041	7.710619897	7.264927592
ENSG00000236761	CTAGE9	6.379500933	0.025566866	-5.191136046	0.127639854	1.188364887	2	2	2	6.496695859	6.44777477	2	2
ENSG00000140450	ARRDC4	0.676292883	0.392311627	0.508704795	0.523029563	1.184997678	8.39321512	8.862353853	9.809100877	9.874791085	9.7546632	10.07738813	9.037341395
ENSG00000176396	EID2	0.145612922	0.942460221	1.038942724	0.568673191	1.184555647	8.138771685	6.728495673	2	7.2696592	7.06433609	7.813294077	8.245238611
ENSG00000128915	ICE2	0.442601512	0.72366688	0.741005176	0.531002733	1.183606687	4.815609471	7.761270542	8.110401118	7.93509946	7.81567127	8.269993575	8.063441666
ENSG00000128510	CPA4	-0.198874274	0.925672996	1.381966079	0.458652814	1.183091805	5.70932728	2	6.073378139	5.110902178	5.22805098	4.841348851	7.417152486
ENSG00000168517	HEXIM2	1.608810586	0.465557972	-0.42577379	0.858491391	1.183036797	2	5.72254323	2	5.742861563	5.84514933	4.271694079	3.789585079
ENSG00000171045	TSNARE1	0.563391967	0.726445618	0.619359922	0.694591795	1.182751889	3.327711234	3.686303414	6.534109959	5.731694	5.78232107	5.818966034	5.799216178
ENSG00000136950	ARPC5L	0.686902497	0.164314918	0.495501931	0.321838434	1.182404428	7.790810999	8.469613413	7.600047325	8.622857004	8.76268478	8.437839892	8.569323645
ENSG00000077721	UBE2A	0.665370224	0.130840883	0.51702798	0.244791857	1.182398204	9.544849385	8.717821467	9.31604076	9.932626344	9.84581957	9.692647041	9.790113704
ENSG00000133116	KL	-1.3104834	0.740521619	2.49193891	NA	1.18145551	2	2	2	2.141737246	2.14022021	3.632645806	2.817676617

ENSG00000106460	TMEM106B	0.33118287	0.471798515	0.849959936	0.03766925	1.181142807	8.674867122	8.317412869	8.110401118	8.795015659	8.62772819	9.423527655	9.002492046
ENSG00000151789	ZNF385D	1.104154447	0.628529867	0.07684531	0.974738711	1.180999758	4.00722531	2	2	3.709785322	3.50452879	2.937246078	3.071407821
ENSG00000198964	SGMS1	0.354144603	0.639949127	0.826787776	0.229804148	1.180932379	8.042902253	6.899080544	8.511888463	8.312101014	8.29037844	8.685424964	8.847658168
ENSG00000277796		6.37079232	0.028382077	-5.191135276	0.132037535	1.179657044	2	2	2	6.172600389	6.70653264	2	2
ENSG00000133226	SRRM1	0.650664961	0.398806121	0.528569389	0.492624208	1.179234349	9.751047882	8.451443573	8.175061096	9.643348214	9.57865651	9.376644377	9.594965643
ENSG00000095970	TREM2	-3.753036251	0.484606942	4.932028529	0.300671384	1.178992278	2	2	2	2	2	5.912830251	3.311948089
ENSG00000213918	DNASE1	-0.317453789	0.770049657	1.495745194	0.106971668	1.178291406	5.095959458	6.728495673	5.930865648	5.720439317	5.85535975	8.066950774	6.649933445
ENSG00000100934	SEC23A	0.400990749	0.693814531	0.776537474	0.412307433	1.177528223	8.653385977	11.13502303	10.62096771	10.8418393	10.8803543	11.2572217	11.21457718
ENSG00000106993	CDC37L1	0.388413332	0.851528683	0.787357733	0.684823328	1.175771065	2	6.602424674	8.296287027	7.517371202	7.46188679	8.123435792	7.589522245
ENSG00000129353	SLC44A2	0.381112134	0.48510951	0.794557118	0.110465713	1.175669252	11.09707093	10.30378101	10.21486071	10.9418361	11.0082583	11.20209938	11.55296168
ENSG00000140750	ARHGAP17	0.223195816	0.52733186	0.952321044	0.001347185	1.17551686	9.015175615	9.090820894	9.439621147	9.385472425	9.43967292	10.06739966	10.20648952
ENSG00000175582	RAB6A	0.648597485	0.237056501	0.526640176	0.339165925	1.175237661	9.740899611	9.883504052	10.76969939	10.87774275	10.8262286	10.76561488	10.69456799
ENSG00000162735	PEX19	0.58017549	0.406294093	0.594422415	0.382921621	1.174597905	8.418903792	9.433952963	9.972637969	9.987058621	9.98368402	9.865889142	10.1219154
ENSG00000090104	RGS1	-2.312741879	0.674378575	3.487252386	0.473060066	1.174510507	2	2	2	2	2.14022021	4.727866721	2.117335275
ENSG00000157259	GATAD1	0.228276904	0.712244219	0.946091631	0.07587755	1.174368534	8.39321512	8.981105197	8.325066185	8.83470036	8.82375772	9.894727355	9.074092868
ENSG00000115687	PASK	0.936025938	0.598708746	0.237651304	0.901746027	1.173677242	3.327711234	5.595927442	2	5.178261742	5.28958453	4.933813368	4.241259475
ENSG00000100744	GSKIP	0.846414387	0.686262329	0.326729498	0.883002588	1.173143885	2	6.389896633	8.784770914	8.332573384	8.248381	7.942116583	7.595854839
ENSG00000007168	PAFAH1B1	0.295041452	0.820993582	0.877746485	0.453149799	1.172787937	7.867441304	10.51535307	11.2690689	10.78523872	10.6795076	11.16263905	11.4526447
ENSG00000221869	CEBPD	0.366906266	0.530881055	0.804922155	0.128777677	1.171828422	8.107517749	9.018611864	8.408111533	8.887228971	8.97656697	9.621696553	9.058587737
ENSG00000127616	SMARCA4	0.410396067	0.52243871	0.75762372	0.199517168	1.168019786	9.174465797	10.3778065	9.503962296	10.17296029	10.2079206	10.37096889	10.68539259
ENSG00000156030	ELMSAN1	0.254378258	0.67218509	0.913302252	0.080754483	1.16768051	8.313285031	7.819099893	8.825624954	8.593855195	8.64842725	9.023081974	9.489401982
ENSG00000144485	HES6	-0.358795464	0.820253704	1.525429812	0.260131361	1.166634348	4.467311984	5.72254323	2	4.470469939	4.45712836	6.353989995	5.896817464
ENSG00000040275	SPDL1	0.91932035	0.476505377	0.245471105	0.860801801	1.164791455	8.586954094	8.376385542	4.874587708	8.84120963	8.89725143	8.242494717	8.162331091
ENSG00000186132	C2orf76	0.392198987	0.841622412	0.77191035	0.674363565	1.164109337	5.532353208	2	3.369317972	4.739373666	4.50979031	5.002522427	4.903386193
ENSG00000173039	RELA	0.366588791	0.528469036	0.79751928	0.12999296	1.164108071	9.384607364	10.07368926	10.48730704	10.43937195	10.3914606	10.65972326	11.01000224
ENSG00000108001	EBF3	-0.681559707	0.809107998	1.843010066	0.459209794	1.161450359	2	3.686303414	2	2.868578001	2.2680111	4.443291373	3.311948089
ENSG00000147649	MTDH	0.491231989	0.207531949	0.670161882	0.070946784	1.161393871	10.05943807	10.40166101	10.75391088	10.92814295	10.9161551	11.07299152	11.12797955
ENSG00000173409	ARV1	0.539420588	0.78336925	0.621013239	0.744740559	1.160433827	2	6.602424674	8.076950877	7.523859802	7.42122564	7.340163764	7.738907626
ENSG00000102910	LONP2	0.088296307	0.931617375	1.071865002	0.215498263	1.160161309	8.716893051	8.540087354	10.45512158	9.601017164	9.60234163	10.62621766	10.53549289
ENSG00000185803	SLC52A2	0.609850721	0.451559843	0.548922807	0.492084442	1.158773528	9.384607364	9.506147172	7.691296561	9.653739712	9.68624302	9.646549955	9.571380235
ENSG00000102890	ELMO3	-0.100042052	0.961244994	1.258662839	0.488001567	1.158620787	4.00722531	3.076665209	2	2.868578001	3.45167086	4.434275199	3.660199087
ENSG00000100522	GNPNAT1	1.07264829	0.275627669	0.085785265	0.940230037	1.158433555	6.73718411	7.638155693	9.196533591	9.32080297	9.22907886	8.237320313	8.356084628
ENSG00000105649	RAB3A	1.011556307	0.535687826	0.146444276	0.935496579	1.158000582	7.031698319	5.72254323	2	6.941624891	6.9210099	6.109723842	6.099376274
ENSG00000004399	PLXND1	-0.843742838	0.137345288	2.001327315	8.35E-05	1.157584477	9.966689162	9.256956408	8.784770914	8.499891024	8.65866647	11.5508907	11.26163861
ENSG00000151876	FBXO4	0.645412024	0.734810023	0.511751494	0.789905257	1.157163518	3.327711234	5.946638857	2	5.093559143	5.41912973	5.348031117	4.911532223
ENSG00000012048	BRCA1	1.170332113	0.235251572	-0.013544381	0.990611355	1.156787732	3.327711234	5.595927442	4.874587708	6.061031724	5.87556634	4.854926465	4.943664032
ENSG00000151502	VPS26B	-0.352645484	0.174638808	1.509076506	7.66E-12	1.156431022	9.128497779	9.170736405	9.083172337	8.820275739	8.73806474	10.62163296	10.64214212
ENSG00000136451	VEZF1	0.645775755	0.725609862	0.5091375	0.784027326	1.154913255	3.327711234	9.566483142	9.099922433	9.479434065	9.35854723	9.395058233	9.164631393
	CCL3L3	6.345960029	0.030507988	-5.191134925	0.134274864	1.154825104	2	2	2	6.139181293	6.68951263	2	2
ENSG00000052841	TTC17	0.589023168	0.704833394	0.565402969	0.71335944	1.154426137	5.532353208	10.26269067	8.865353883	9.785808044	9.75124983	9.970549666	9.477790979
ENSG00000136436	CALCOCO2	0.679720163	0.329996554	0.474513853	0.503157092	1.154234016	10.19198706	9.828248506	8.608698767	10.37802153	10.3478309	10.24273081	10.06895712
ENSG00000118292	C1orf54	0.801092904	0.536848085	0.352581418	0.796677889	1.153674322	7.27616306	5.303523747	4.058998049	6.892328675	6.90143415	6.401024833	6.528072775
ENSG00000089682	RBM41	1.159527669	0.156709059	-0.006060647	0.995062288	1.153467022	7.031698319	7.847167909	5.930865648	8.338106704	8.23671175	7.427945125	6.793487375
ENSG00000080546	SESN1	0.144001584	0.943253539	1.009117024	0.581437534	1.153118608	2	7.761270542	6.958202216	7.080147179	6.89649843	8.359038717	7.006211772
ENSG00000214530	STARD10	0.030227345	0.982666154	1.122817542	0.340932973	1.153044887	4.815609471	8.191702463	8.007629717	7.52061915	7.71833702	8.879345195	8.494699808
ENSG00000160318	CLDND2	0.728617391	0.790784372	0.42284701	0.879570438	1.151464401	2	3.076665209	2	2.947924294	2.77779907	2.68698273	2.782576693
ENSG00000128699	ORMDL1	0.662936493	0.551800049	0.488234036	0.666867266	1.151170529	7.975256505	9.523645035	6.718950348	9.211498619	9.13163078	9.123606729	8.861915706
ENSG00000164168	TMEM184C	0.528923682	0.446900339	0.621156566	0.353466558	1.150075248	7.435667355	8.297207175	8.978369997	8.874592369	8.90710092	9.002642099	8.961885532
ENSG00000124479	NDP	3.047145665	0.523661392	-1.897326629	0.702841297	1.149819036	2	2	2	3.663521627	3.60476711	2.183373728	2
ENSG00000181004	BBS12	0.720736108	0.670452104	0.428957693	0.804379534	1.149693801	2	5.595927442	6.882775709	6.573765157	6.42738748	6.407949645	6.014942101
ENSG00000121653	MAPK8IP1	0.490442097	0.699694748	0.659100455	0.588785095	1.149542552	3.327711234	6.389896633	6.43197674	6.415275989	6.34282908	6.237020416	6.791281771
ENSG00000130649	CYP2E1	0.44687083	0.77994267	0.701307938	0.64228189	1.148178767	2	4.11352228	4.058998049	4.17310023	3.82856379	4.45225155	3.908316285
ENSG00000177045	SIX5	0.702989977	0.73397139	0.445167031	0.833553126	1.148157008	5.866944937	8.395519306	2	7.691737974	7.80517502	7.628021136	7.352801367
ENSG00000168297	PXK	0.443718202	0.796967396	0.704354423	0.668486483	1.148072625	7.384433967	8.451443573	3.369317972	7.922877029	7.8745862	8.589193677	7.532563185
ENSG00000129292	PHF20L1	0.898744777	0.306048059	0.249111812	0.795874263	1.147856589	8.16936288	7.73146271	9.69246155	9.711725296	9.64416621	9.123606729	8.935663927
ENSG00000138686	BBS7	0.982018923	0.531143362	0.164758624	0.925295809	1.146777547	3.327711234	6.899080544	8.266922072	8.136938566	8.15847021	7.557113378	7.09829807
ENSG00000068079	IFI35	2.555363014	0.238266302	-1.409111794	0.532346835	1.14625122	10.12333326	6.464272742	2	11.18792842	11.2037318	7.05561307	7.451079708
ENSG00000162368	CMPK1	0.429866735	0.623107483	0.716331193	0.382493233	1.146197928	8.816894989	9.159587375	10.57976717	10.23853288	10.0760734	10.61877565	10.24769118
ENSG00000196911	KPNA5	0.44367403	0.811172496	0.701474344	0.690278517	1.145148375	2	6.78762695	4.874587708	6.069927018	5.96314905	6.45105139	6.041166521
ENSG00000009780	FAM76A	0.659856564	0.706615573	0.485200421	0.784357456	1.145056985	2	6.602424674	7.342949392	7.093295078	7.09050444	6.857395343	6.985139097
ENSG00000172817	CYP7B1	0.212015851	0.927391636	0.932843185	0.658380896	1.144859036	2	4.11352228	2	3.227757282	3.33976443	3.694366604	3.807156736
ENSG00000116035	VAX2	0.560041001	0.807722186	0.584741022	0.795268255	1.144782022	2	4.93638933	2	4.329433326	3.98554648	3.904814898	4.4231929
ENSG00000022840	RNF10	0.416942322	0.478107313	0.726754355	0.181447155	1.143696677	9.761125267	10.51535307	9.589475645	10.4457931	10.4188272	10.42288506	11.00215671
ENSG00000197362	ZNF786	0.398418583	0.760990111	0.74505019	0.543096343	1.143468773	4.815609471	6.311476754	3.369317972	5.627094009	5.77157844	6.140539117	5.896817464
ENSG00000243244	STON1	0.384134807	0.847860259	0.757507382	0.686224112	1.141642189	2	8.124485672	7.163363831	7.555872146	7.50468064	8.11499695	7.63942137
ENSG00000198001	IRAK4	0.711978449	0.654474154	0.429571327	0.793476131	1.141549776	4.467311984	5.457120953	8.408111533	7.799951945	7.73502229	7.717139459	7.22299542
ENSG00000100441	KHNYN	0.504755617	0.671131573	0.636303123	0.578462503	1.14105874	9.27634997	9.599860728	6.534109959	9.381905119	9.53167935	9.77507622	9.376603759
ENSG00000130347	RTN4IP1	0.166216354	0.91483416	0.974699122	0.473386442	1.140915475	3.327711234	5.72254323	3.369317972	4.623314311	4.97065356	5.4091291	5.631815919
ENSG00000176125	UFSP1	-0.075266601	0.973704667	1.216068003	0.549060202	1.140801402	2	4.710564154	2	3.663521627	3.39680241	4.620674722	4.365060851
ENSG00000104852	SNRNP70	0.12276665	0.903601303	1.016934303	0.229671798	1.139700954	10.43166252	8.234834549	9.91701143	9.971714969	9.83811764	10.6925865	10.89595288
ENSG00000256223	ZNF10	0.765890814	0.767238548	0.373765404	0.889574473	1.139656218	2	6.666836837	2	5.94010643	5.88556448	5.758846871	5.306932076
ENSG00000133561	GIMAP6	-0.733152585	NA	1.872209133	NA	1.139056548	2	2	2	2	2.38539787	3.336159116	2.421067459
ENSG00000260230	FRRS1L	-2.801422478	0.166629297	3.940160475	0.03232554	1.138737997	2	2	5.59507254	2.695456494	2.49394783	8.139473554	7.373589743
ENSG00000168505	GBX2	-3.753052554	0.464636768	4.891395323	0.278925497	1.138342769	2	2	2	2	2	5.650128416	4.188602707
ENSG00000169306	IL1RAPL1	3.443389262	0.422792655	-2.305804935	0.610398832	1.137584327	2	2	2	3.79808662	4.02226117	2.139668783	2
ENSG00000087053	MTMR2	0.18195346	0.856036852	0.95560691	0.265190311	1.13756037	7.624139768	9.814097497	9.478569125	9.432787137	9.40794139	10.31542752	10.05385647
ENSG00000183814	LIN9	1.028460086	0.669545698	0.108968658	0.967122742	1.137428744	2	5.838937808	2	5.433909581	5.30456641	4.984105765	3.987514168
ENSG00000172840	PDP2	0.652630915	0.441837321	0.484326975	0.569975156	1.136957889	8.39321512	6.602424674	7.029880454	8.211183901	8.16669309	8.017249226	8.029224588
ENSG00000163959	SLC51A	-3.753058285	0.456587162	4.889008004	0.268957669	1.135949719	2	2	2	2	2	4.596630906	5.464725981
ENSG00000137876	RSL24D1	0.665345072	0.484223993	0.469559815	0.626190802	1.134904887	9.159305284	8.297207175	6.882775709	9.053418491	9.03948698	9.14414236	8.485861839
ENSG00000144711	IQSEC1	-0.524042989	0.620673635	1.658540477	0.075265997	1.134497488	9.761125267	8.929529568	7.029880454	8.422134132	8.44542686	10.64526705	10.56015326
ENSG00000223609	HBD	-3.753046562	0.472493119	4.885780587	0.289935311	1.132734025	2	2	2	2	2	3.865109708	5.745545708
ENSG00000182993	C12orf60	0.649701868	0.698007365	0.482729901	0.773825851	1.132431769	3.327711234	4.11352228	2	3.997744386	3.7864959	3.930690651	3.600986722
ENSG00000117151	CTBS	2.007010237	0.000140956	-0.874802202	0.127639854	1.132208035	7.667647223	6.728495673	7.97167868	9.56056556	9.46656462	6.781219718	6.574984961
ENSG00000122729	ACO1	-0.364205092	0.506977228	1.495666997	0.001282361	1.130861904	9.384607364	8.981105197	8.699419242	8.643859774	8.73115061	10.12806717	10.85575293
ENSG00000168273	SMIM4	1.223491477	0.293606106	-0.09417951	0.944101725	1.129311966	6.257048221	4.11352228	4.058998049	6.29144905	6.33555364	4.868377487	5.300743019
ENSG00000177084	POLE	0.201821637	0.85332146	0.927330666	0.325557697	1.129152302	5.095959458	7.572420273	6.718950348	7.003290579	6.97819215	7.799317009	7.589522245
ENSG00000129083	COPB1	0.99160389	0.026999437	0.136787676	0.797729416	1.128391566	10.91047246	10.31889309	10.01740919	11.45776963	11.4495233	10.70165563	10.49167828
ENSG00000186187	ZNRF1	-0.136334662	0.85249814	1.262536412	0.032526136	1.12620175	8.313285031	8.004992147	6.882775709	7.706073672	7.7377845	9.041753004	9.163352825
ENSG00000187866	FAM122A	0.421154344	0.832844524	0.701539516	0.710275609	1.12269386	2	7.503546079	8.206335842	7.783822874	7.68723697	8.061837451	7.96021985
ENSG00000109436	TBC1D9	1.593312096	0.007712736	-0.471224391	0.483213396	1.122087705	8.586954094	7.503546079	7.734844356	9.587916984	9.60006644	7.880388025	7.128314671
ENSG00000108821	COL1A1	-0.05553266	0.878292377	1.177415735	1.22E-05	1.121883075	16.50604222	16.62872997	16.91596224	16.60604717	16.6700869	17.77225622	17.96405002
ENSG00000113732	ATP6V0E1	1.007120342	0.010191064	0.114701849	0.809379135	1.121822191	10.25752168	10.24169813	10.87563042	11.50833956	11.4778868	10.4659642	10.72723989
ENSG00000170088	TMEM192	0.2460735	0.687336686	0.875200914	0.099247094	1.121274414	6.653098118	7.60566235	6.882775709	7.437142666	7.28811304	7.86621124	8.087908463
ENSG00000155827	RNF20	0.72042312	0.021458981	0.400271475	0.22346137	1.120694595	8.313285031	8.004992147	8.076950877	8.786946023	8.90218458	8.520567915	8.53677936
ENSG00000257218	GATC	0.773533602	0.227425425	0.346420599	0.610398832	1.119954201	7.867441304	8.234834549	6.80318718	8.538855014	8.50986596	8.201899078	7.996092644
ENSG00000006715	VPS41	0.469161983	0.726949143	0.648859687	0.616400319	1.118021671	5.70932728	8.623598428	9.465702942	9.005540335	9.09655277	9.035431928	9.401760658
ENSG00000125875	TBC1D20	0.219383541	0.733353042	0.898453203	0.107495597	1.117836744	10.08373159	9.632483534	8.805342542	9.820482079	9.81541592	10.53229574	10.45592109
ENSG00000160785	SLC25A44	0.552797788	0.807307128	0.564434798	0.799962733	1.117232587	9.771132748	7.701025996	2	9.05677832	9.03724641	8.846588786	9.243382473
ENSG00000122490	PQLC1	0.341916036	0.588043549	0.774042542	0.176369212	1.115958578	7.579279349	8.234834549	8.865353883	8.679168098	8.63218877	9.128855427	9.038271684
ENSG00000188368	PRR19	0.765281136	0.727489842	0.347897664	0.879564373	1.1131788	2	4.11352228	2	3.709785322	3.55551834	3.483289517	3.236138336
ENSG00000169239	CA5B	-0.393660247	0.739140058	1.50648611	0.143980561	1.112825863	5.095959458	6.228547981	7.777116083	6.358325776	6.42052719	8.546948034	7.845855157
ENSG00000205268	PDE7A	0.701456821	0.463053095	0.411257407	0.675028301	1.112714227	4.00722531	6.140560021	6.073378139	6.29144905	6.41363412	6.247352872	5.888587685
ENSG00000011376	LARS2	0.695737617	0.730413605	0.416976322	0.840253981	1.112713939	8.737455672	7.145017454	2	8.145379268	8.36552655	7.860333006	8.099542409
ENSG00000168461	RAB31	0.687629817	0.052939325	0.424785026	0.249077972	1.112414843	10.27168932	10.57950211	9.972637969	10.99271605	10.9764528	10.80702258	10.63231802
ENSG00000184160	ADRA2C	-1.232188041	0.604386987	2.343096719	0.261724935	1.110908678	2	3.686303414	2	2.270786764	2.59489879	4.530483275	4.254128754
ENSG00000109736	MFSD10	0.565104143	0.560004695	0.543823767	0.570388322	1.10892791	9.886101849	8.777382925	7.552162999	9.567648462	9.62265847	9.330617798	9.782638733

ENSG00000152380	FAM151B	0.706214314	0.758269479	0.399613243	0.864992366	1.105827557	2	5.303523747	2	4.415698455	4.76938658	4.487545924	4.201948172
ENSG00000109572	CLCN3	0.527413376	0.704195879	0.574941694	0.673640399	1.10235507	5.70932728	8.732943944	9.714382178	9.286870873	9.3166689	9.394767778	9.301879118
ENSG00000130813	C19orf66	1.941426824	0.247157846	-0.839151685	0.638604205	1.102275139	10.37373127	5.303523747	6.629488387	10.85444304	10.8725209	8.094396944	8.107542072
ENSG00000187231	SESTD1	0.422866345	0.84453415	0.678015251	0.740101564	1.100881596	8.313285031	9.181799937	2	8.660150093	8.64695856	9.113404777	8.664579282
ENSG00000000419	DPM1	1.475511389	0.163819362	-0.375704265	0.749808479	1.099807124	8.367060731	6.04685525	6.073378139	8.744518279	8.69038176	7.006273211	6.78907279
ENSG00000170961	HAS2	3.818563938	0.005459329	-2.718923171	0.055260188	1.099640767	6.563806279	8.834582582	9.670202709	12.64894221	12.6252174	7.046766788	3.67940853
ENSG00000138764	CCNG2	1.539674367	0.191786205	-0.443022055	0.732581748	1.096652312	5.330594134	8.004992147	8.960138144	9.587916984	9.56082893	7.690882553	7.532563185
ENSG00000106261	ZKSCAN1	0.213179912	0.73312176	0.883200121	0.102696941	1.096380033	9.317904228	9.806969599	10.40208303	10.16675444	10.0755276	11.01398252	10.52458037
ENSG00000109046	WSB1	0.800023724	0.120735975	0.296161657	0.595909021	1.096185381	9.646202137	9.975384048	10.67785662	10.95239719	10.972354	10.56209021	10.34957621
ENSG00000133433	GSTT2B	6.285527061	0.032987346	-5.191134849	0.135323833	1.094392212	2	2	2	6.379946408	6.3857277	2	2
ENSG00000176834	VSIG10	1.154333199	0.488856774	-0.064278552	0.972248849	1.090054647	2	6.535001748	7.285536013	7.43025113	7.58969985	6.475428841	6.179140936
ENSG00000169598	DFFB	0.559215493	0.508392192	0.530616028	0.521153279	1.08983152	4.467311984	5.72254323	4.874587708	5.565529336	5.76075522	5.696113106	5.581648407
ENSG00000014138	POLA2	1.612032121	0.170202551	-0.52241666	0.684865709	1.089615461	3.327711234	6.602424674	5.39244166	7.303993732	7.08183429	4.996409624	5.361470313
ENSG00000069020	MAST4	1.183144606	0.550957372	-0.094351207	0.966440292	1.088793398	2	7.051611315	8.825624954	8.76109026	8.80938679	7.808940772	7.174740504
ENSG00000111911	HINT3	0.378883407	0.33193825	0.709185207	0.048045799	1.088068614	8.228665467	8.414402626	8.076950877	8.643859774	8.60974666	9.121501891	8.764652886
ENSG00000100883	SRP54	1.058306802	0.001669747	0.028985164	0.94688402	1.087291966	8.517314938	8.732943944	9.116480286	9.853698554	9.86489641	8.794695657	8.877074023
ENSG00000129625	REEP5	1.097955562	0.002123735	-0.01101708	0.981056901	1.086938482	10.46286899	9.792607274	9.999667074	11.21699976	11.1947131	10.06976682	10.12913753
ENSG00000136770	DNAJC1	1.545376082	0.179452048	-0.460795479	0.716145684	1.084580603	9.410448395	8.276714465	6.073378139	10.06420548	9.92611804	8.364981826	7.531242162
ENSG00000155096	AZIN1	0.868543828	0.049761083	0.216007871	0.66704421	1.084551699	9.790941832	9.726146444	10.43545992	10.89848915	10.871578	10.02617706	10.41621675
ENSG00000115827	DCAF17	0.415496067	0.535201232	0.668042874	0.288186427	1.083538941	7.750913381	6.535001748	7.098165388	7.662632018	7.54938955	8.059640467	7.615934347
ENSG00000128815	WDFY4	-3.753037904	0.483854018	4.836149506	0.31009769	1.083111602	2	2	2	2	2	5.769639817	3.539239711
ENSG00000177302	TOP3A	0.489107526	0.71007843	0.593939031	0.641832906	1.083046557	5.095959458	7.503546079	8.677268317	8.178656739	8.185025	8.443467705	8.107542072
ENSG00000174796	THAP6	0.751217359	0.39960716	0.331225818	0.724423786	1.082443177	5.330594134	6.311476754	7.163363831	7.157295126	7.18663697	7.125896536	6.283256815
ENSG00000153113	CAST	0.803328538	0.007872825	0.278649428	0.404998648	1.081977966	10.52923584	10.12617756	10.29705308	11.10597219	11.1469365	10.45945184	10.73371512
ENSG00000148400	NOTCH1	-0.526973024	0.706963346	1.608762051	0.195995925	1.081789027	10.00110988	7.189546432	6.882775709	8.244958867	8.20312688	10.56040784	10.0915399
ENSG00000197894	ADH5	0.575321858	0.148344449	0.503610428	0.203432268	1.078932286	10.11549941	9.558016677	9.452720984	10.32559865	10.2962973	10.23236508	10.24668541
ENSG00000167716	WDR81	2.949186931	0.096199	-1.870553838	0.305382004	1.078633093	4.815609471	7.790474954	2	9.253098082	9.35225233	4.914492341	4.479073019
ENSG00000197283	SYNGAP1	3.826808083	9.25E-05	-2.748504671	0.007503255	1.078303412	5.095959458	6.464272742	3.369317972	9.211498619	9.21229508	3.534812207	3.128433649
ENSG00000163516	ANKZF1	0.416287819	0.811431778	0.661789553	0.689452881	1.078077372	3.327711234	8.337339476	8.608698767	8.374462892	8.27524772	8.624303429	8.5102048
ENSG00000139842	CUL4A	0.555931936	0.494662338	0.521035324	0.516558603	1.07696726	10.01801709	9.377333006	8.110401118	9.883647099	9.95310979	9.787077426	9.974915827
ENSG00000143418	CERS2	1.186806401	1.75E-05	-0.110086832	0.74998849	1.076719568	10.13887448	10.3138733	10.07784268	11.34755455	11.384692	9.888345571	10.23598093
ENSG00000183864	TOB2	0.356721654	0.611774065	0.717345209	0.267661148	1.074066863	9.33149386	8.777382925	7.818184403	9.115954467	9.13163078	9.491928968	9.472294474
ENSG00000131236	CAP1	0.929941166	0.283881375	0.142956637	0.885544215	1.072897803	11.70484245	11.00090285	9.478569125	11.89635181	11.9405179	11.05867027	11.20311249
ENSG00000145592	RPL37	0.428416973	0.715571124	0.644315383	0.565593677	1.072732356	11.31110672	9.114109157	8.845626171	10.61183616	10.6621638	11.06379481	10.60510859
ENSG00000138376	BARD1	0.026003698	0.987001945	1.044337043	0.453525552	1.070340741	5.70932728	6.140560021	2	5.514313187	5.37722731	6.529346487	6.251520369
ENSG00000110917	MLEC	-0.012444626	0.98350331	1.082670239	0.024735408	1.070225613	10.19198706	10.83911531	11.16147894	10.74553862	10.7983847	11.63457429	12.06463678
ENSG00000155906	RMND1	-0.079283928	0.947432314	1.14894686	0.268972233	1.069662933	8.075571721	6.951736442	5.156626401	7.157295126	7.00595521	8.4151065	8.146888246
ENSG00000105784	RUNDC3B	-2.305941104	0.617291379	3.371333613	0.392847555	1.065392509	2	2	2	2.141737246	2	3.930690651	3.789585079
ENSG00000102144	PGK1	0.412534376	0.716492539	0.65255626	0.544753443	1.065090635	12.91048752	11.64711661	10.23774519	12.3799161	12.4031015	12.09610914	13.02096146
ENSG00000148843	PDCD11	0.463596082	0.693529621	0.601348771	0.596070489	1.064944853	6.009025997	8.169643011	9.099922433	8.687861792	8.68752719	8.896269648	8.74825912
ENSG00000136463	TACO1	0.279991015	0.848343006	0.784530242	0.554021903	1.064521258	7.096806373	8.004992147	4.058998049	7.348537858	7.4074129	7.755649985	7.980689421
ENSG00000138696	BMPR1B	3.743685578	0.008473525	-2.680087471	0.079084492	1.063598108	2	4.710564154	2	6.841288255	6.83591385	2.384001718	2.421067459
ENSG00000169019	COMMD8	0.71883224	0.575783958	0.344344166	0.797495402	1.063176406	6.89194353	6.728495673	3.369317972	7.088925745	6.88657603	6.685680098	6.572418365
ENSG00000166974	MAPRE2	-0.043076625	0.961267251	1.106247473	0.136956979	1.063170848	9.87686338	8.414402626	8.325066185	8.971529165	9.05949698	10.00711202	10.29710605
ENSG00000080822	CLDND1	0.791530259	0.115289102	0.271623456	0.620529852	1.063153715	9.219014269	8.981105197	8.206335842	9.706743577	9.59398167	9.246842239	9.012940881
ENSG00000104825	NFKBIB	0.841733084	0.501949395	0.219443847	0.872155485	1.061176932	8.75772933	6.140560021	6.073378139	8.447952961	8.34212018	7.674704537	7.883613091
ENSG00000181634	TNFSF15	2.274944218	0.006236051	-1.21466539	0.172169048	1.060278828	5.330594134	4.93638933	5.39244166	7.384635376	7.35080119	4.644324387	3.539239711
ENSG00000072201	LNX1	-0.686353273	0.774715444	1.746433287	0.423520139	1.060080014	2	5.946638857	2	4.205751501	3.82856379	6.054896192	6.222348126
ENSG00000126351	THRA	-0.175274449	0.855650806	1.235243857	0.122176759	1.059969408	7.27616306	8.124485672	9.301632639	8.325162455	8.2657098	9.759594615	9.626855891
ENSG00000065833	ME1	0.898469613	0.031533344	0.160345423	0.740394508	1.058815037	7.975256505	8.687092496	8.325066185	9.26185831	9.25051318	8.35963413	8.672390305
ENSG00000132549	VPS13B	0.218176471	0.841552857	0.838384585	0.381507263	1.056561057	7.031698319	9.470501606	9.31604076	9.135152476	9.18927228	9.619709545	9.922723748
ENSG00000203879	GDI1	0.025887369	0.94878661	1.029112279	0.001098499	1.054999648	10.98115017	10.60026656	10.56174313	10.7438026	10.760856	11.58483423	11.90525006
ENSG00000143632	ACTA1	2.668055973	0.128993961	-1.613897053	0.376931525	1.05415892	2	5.303523747	4.058998049	6.622948477	6.90143415	3.782262616	2.326754077
ENSG00000203668	CHML	-0.798688733	0.46309526	1.851376473	0.056913062	1.05268774	7.579279349	5.595927442	5.39244166	5.987823101	5.56313034	8.552166664	8.116378869
ENSG00000131188	PRR7	-0.184287874	0.864318577	1.235244229	0.166131291	1.050956355	4.467311984	5.303523747	6.534109959	5.304221918	5.67111583	6.704551153	6.957866334
ENSG00000102870	ZNF629	0.077305014	0.881572243	0.972960291	0.016733148	1.050265305	8.981092835	8.234834549	8.585098385	8.658676732	8.74631819	9.470589819	9.704799739
ENSG00000243444	PALM2	2.958923197	0.000162583	-1.909910494	0.022627193	1.049012702	4.815609471	5.595927442	4.523675391	7.855024645	7.92150349	3.904814898	3.15612297
ENSG00000122482	ZNF644	0.736589359	0.130096658	0.311249551	0.548805363	1.047838911	8.042902253	8.356994601	8.486635645	9.035366048	9.03836713	8.93593651	8.20189164
ENSG00000111445	RFC5	-0.050085257	0.982507576	1.096457513	0.587973908	1.046372255	3.327711234	6.844429755	2	5.258265913	5.53799497	6.475428841	6.474094059
ENSG00000196357	ZNF565	0.187934195	0.880488774	0.856953985	0.427508497	1.044888181	4.00722531	5.946638857	4.523675391	5.178261742	5.37722731	6.210860813	5.486701579
ENSG00000198355	PIM3	0.648521463	0.30880261	0.395842158	0.544751469	1.044363621	8.540903468	8.004992147	9.272377397	9.286870873	9.38697919	8.843186716	9.295279234
ENSG00000113569	NUP155	0.286692032	0.88899497	0.757349417	0.688288292	1.044041449	8.340423424	7.315436877	2	7.635924947	7.61395775	8.171702207	7.998006535
ENSG00000018625	ATP1A2	-1.499673253	0.356981191	2.541713422	0.085269823	1.042040169	4.00722531	3.686303414	4.874587708	3.79808662	2.2680111	5.991769846	6.91798856
ENSG00000132964	CDK8	0.730076069	0.096173427	0.311295071	0.505195354	1.04137114	7.435667355	7.189546432	7.451345288	8.15586117	7.98565528	7.858649102	7.432801564
ENSG00000140859	KIFC3	0.466035741	0.717903042	0.575134688	0.646513979	1.041170428	10.29268328	7.051611315	8.434763955	9.629876784	9.64121862	9.524419769	9.934763826
ENSG00000198668	CALM1	0.394443059	0.507120384	0.646417246	0.244987518	1.040860304	11.62983336	11.30149645	10.61515347	11.64928415	11.6210902	11.53917738	12.16696017
ENSG00000176472	ZNF575	-0.082248565	0.95389048	1.122473112	0.355912095	1.040224547	4.467311984	4.442710129	2	3.997744386	3.94787299	4.977914261	4.927687581
ENSG00000100060	MFNG	-2.312593073	0.61703084	3.352633357	0.397192742	1.040040284	2	2	2	2	2.14022021	3.838019203	3.858622973
ENSG00000179152	TCAIM	-0.039175195	0.957413691	1.078840541	0.070856581	1.039665346	6.89194353	6.666836837	7.896971941	7.222570549	7.18663697	8.268726114	8.333525663
ENSG00000086712	TXLNG	0.717213474	0.347836352	0.322251568	0.68935002	1.039465042	6.96351253	6.78762695	8.236946975	8.237082658	8.11663771	7.992893101	7.552234734
ENSG00000123159	GIPC1	-0.199251919	0.80934297	1.238003	0.072624316	1.038751081	8.444143041	9.763446433	10.17589429	9.414570905	9.44559847	10.776023	10.94340508
ENSG00000125629	INSIG2	0.676805393	0.262504755	0.361636402	0.56576762	1.038441794	9.048471759	8.147241016	7.818184403	9.128781483	9.07923325	8.786305336	8.796891925
ENSG00000198947	DMD	-1.690087007	0.215891254	2.728010258	0.032479705	1.037923251	4.467311984	8.639735838	6.629488387	6.006476466	5.62411716	10.13920704	10.15230914
ENSG00000162385	MAGOH	1.532787592	0.254759301	-0.495026377	0.735074986	1.037761215	2	5.72254323	6.073378139	6.687949235	6.93069905	4.952879058	4.919632516
ENSG00000016402	IL20RA	-2.305744058	0.629279802	3.343053519	0.419331922	1.03730946	2	2	2	2.141737246	2	3.12825509	4.316807515
ENSG00000064651	SLC12A2	-0.286900474	0.608680785	1.324134251	0.00487588	1.037233776	7.750913381	7.954278496	8.236946975	7.739909082	7.68150967	8.877267588	9.632103066
ENSG00000105538	RASIP1	-2.141005764	0.314789729	3.177135768	0.077959309	1.036130003	2	3.076665209	3.369317972	2.270786764	2.2680111	5.747972572	4.30448772
ENSG00000139641	ESYT1	0.166129026	0.802782927	0.869843956	0.12255258	1.035972983	9.829762239	10.54135596	9.465702942	10.15216947	10.2168574	10.57483812	11.14297651
ENSG00000140564	FURIN	0.288205309	0.700066046	0.74686252	0.268928351	1.035067829	10.43166252	10.69982928	9.149036566	10.47329248	10.5706238	10.94899883	11.01106879
ENSG00000143157	POGK	-0.267435407	0.73397139	1.302277072	0.051791194	1.034841665	7.579279349	9.018611864	9.149036566	8.468280417	8.47387021	10.19981619	9.837378403
ENSG00000170049	KCNAB3	-0.998593852	0.650789466	2.033042986	0.312242938	1.034449134	2	5.457120953	2	3.615725078	3.28037825	6.146071859	5.685057129
ENSG00000205476	CCDC85C	0.062450639	0.97026695	0.971383889	0.518789265	1.033834528	8.257427002	7.315436877	3.369317972	7.261917051	7.47187556	8.221684808	8.303661547
ENSG00000145241	CENPC	0.737335375	0.665819919	0.295352372	0.868784979	1.032687747	2	6.951736442	7.029880454	7.062428156	7.21862736	6.723178551	6.707209278
ENSG00000143028	SYPL2	-0.725021169	0.73917724	1.756600624	0.370804133	1.031579455	2	4.93638933	2	3.462030052	3.08597292	5.30910438	5.028505473
ENSG00000139636	LMBR1L	1.105290613	0.227729767	-0.07456722	0.944363944	1.030723393	8.285626337	6.228547981	6.718950348	8.379838154	8.4936155	7.461614824	7.069464998
ENSG00000132600	PRMT7	0.51675037	0.730963227	0.511140876	0.730619275	1.027891246	7.031698319	7.73146271	3.369317972	7.381065997	7.40393901	7.182183216	7.566491953
ENSG00000215421	ZNF407	0.5686966	0.625452298	0.458940139	0.694164121	1.027636738	8.285626337	6.04685525	5.930865648	7.68596357	7.79989812	7.474865722	7.78175402
ENSG00000101343	CRNKL1	0.813860906	0.484988029	0.213246242	0.866182611	1.027107149	5.095959458	7.73146271	8.325066185	8.408172799	8.3183278	7.833151805	7.708840864
ENSG00000198356	ASNA1	0.386143043	0.815821608	0.640815325	0.683411628	1.026958368	4.467311984	9.13702746	9.372276026	9.034230235	9.13163078	9.281189863	9.390148213
ENSG00000167552	TUBA1A	0.090832195	0.912408896	0.935642388	0.16984745	1.026474583	12.00538348	11.37081044	11.11279376	11.66716327	11.6066186	11.6643988	12.99956744
ENSG00000028839	TBPL1	0.23083103	0.803843283	0.79361221	0.333586444	1.02444324	5.866944937	7.572420273	7.896971941	7.629922299	7.50792049	8.024025946	8.215937769
ENSG00000158292	GPR153	0.172262748	0.93751235	0.851972567	0.674981326	1.024235315	4.00722531	7.189546432	2	5.94010643	6.00043082	6.913621294	6.238627697
ENSG00000101193	GID8	0.212815897	0.854355329	0.811271147	0.428528777	1.024087045	9.048471759	9.214490408	6.534109959	8.864402784	8.88981993	9.379582949	9.557820379
ENSG00000154359	LONRF1	-0.214675993	0.756822448	1.237237166	0.032014466	1.022561174	6.009025997	6.311476754	6.958202216	6.29144905	6.24526024	7.914016653	7.380942161
ENSG00000145220	LYAR	0.777686828	0.583250623	0.243726873	0.872485883	1.021413701	7.435667355	6.04685525	3.369317972	7.106324235	7.14562489	6.554528258	6.664466987
ENSG00000181007	ZFP82	0.326092604	0.86184497	0.694844839	0.689153776	1.020937443	7.435667355	6.728495673	2	6.856790872	6.89649843	7.464938976	6.959831579
ENSG00000184939	ZFP90	-0.368934056	0.812768397	1.386547575	0.309101085	1.017613519	4.467311984	8.762721501	8.699419242	7.748245381	7.89204953	9.775522494	9.304974529
ENSG00000196664	TLR7	-1.048995563	0.625524857	2.064716866	0.274873919	1.015721302	3.327711234	2	3.369317972	2.498691189	2.59489879	4.73522696	3.735548302
ENSG00000170144	HNRNPA3	1.073278195	0.009101188	-0.057819775	0.911555747	1.015458421	8.963744667	9.599860728	9.788583153	10.56737883	10.5550472	9.357098224	9.508324596
ENSG00000144230	GPR17	-3.753054793	0.463231524	4.766481437	0.290456069	1.013426644	2	2	2	2	2	4.888321757	5.080008298
ENSG00000160049	DFFA	-0.511979825	0.466937891	1.525252154	0.013281894	1.013272329	8.517314938	8.747909548	7.285536013	7.893113669	7.72670378	9.984514132	9.656845513
ENSG00000079841	RIMS1	-0.47657268	0.849039274	1.489662828	0.50032005	1.013090148	2	2	4.523675391	2.695456494	3.33976443	4.416072028	4.341135913
ENSG00000151612	ZNF827	0.521782981	0.730447087	0.491289602	0.743325344	1.013072582	7.82963488	5.131603625	9.439621147	8.814994458	8.84440862	8.457441933	9.075906094
ENSG00000138767	CNOT6L	0.865882135	0.649796974	0.141301366	0.944972617	1.007183501	8.19931886	8.234834549	2	8.545247918	8.44711562	7.953205078	7.589522245
ENSG00000132004	FBXW9	0.470400488	0.864688463	0.534936422	0.841812696	1.00533691	2	6.844429755	2	5.900763176	5.70538658	5.515547099	6.148451724
ENSG00000154144	TBRG1	1.072404723	0.009121679	-0.068444653	0.894874854	1.00396007	7.904282243	8.191702463	8.721235202	9.382797773	9.36034073	8.266822833	8.215115301
ENSG00000088833	NSFL1C	0.391879512	0.638674782	0.611911371	0.438092003	1.003790883	7.384433967	9.181799937	9.049077186	9.123450746	9.12742405	9.240067514	9.440980262

ENSG00000100867	DHRS2	2.39111736	0.48535932	-1.391306034	NA	0.999811326	2	2	2	3.349643765	3.08597292	2.094598369	2.172608095
ENSG00000132912	DCTN4	0.758615305	0.499380069	0.240884264	0.841697129	0.999499569	9.064836028	10.019219	6.958202216	9.927741409	9.87559569	9.481025247	9.285910049
ENSG00000115446	UNC50	0.787275219	0.498492641	0.212068028	0.866223157	0.999343246	8.586954094	7.979858146	5.39244166	8.537252353	8.66157866	8.193240267	7.85221717
ENSG00000147044	CASK	0.739810008	0.490247308	0.25931473	0.82055968	0.999124738	7.435667355	8.540087354	5.772716771	8.435961651	8.32569041	8.070592084	7.725108479
ENSG00000186862	PDZD7	-1.455601688	0.465603893	2.454510686	0.172295853	0.998908998	2	4.93638933	2	2.784612485	3.01487888	5.630526941	5.984375752
ENSG00000144935	TRPC1	-0.225534374	0.87294376	1.224308878	0.308986373	0.998774504	5.532353208	6.311476754	8.585098385	7.210554257	7.15392111	8.802599622	8.380498741
ENSG00000197620	CXorf40A	1.057486238	0.214681289	-0.063347435	0.949476741	0.994138803	7.750913381	6.389896633	5.772716771	7.855024645	7.96222667	6.791847192	6.841176624
ENSG00000112210	RAB23	0.155173961	0.898417957	0.83714063	0.429336127	0.992314591	7.096806373	9.835272298	8.677268317	9.095408867	9.07268445	10.10392776	9.313068526
ENSG00000158417	EIF5B	0.775800356	0.001397551	0.215239376	0.43458157	0.991039732	9.98400217	9.71856931	9.888372618	10.58687666	10.6877448	10.10570349	10.05247582
ENSG00000115816	CEBPZ	0.913897797	0.524696221	0.076785035	0.962239953	0.990682833	9.304185368	8.702538795	4.874587708	9.380118153	9.41313469	8.741779902	8.366492631
ENSG00000110077	MS4A6A	-3.753048705	0.471586224	4.743349317	0.30371251	0.990300612	2	2	2	2	2	5.390603804	4.365060851
ENSG00000102054	RBBP7	0.505658307	0.282799793	0.484102298	0.295471026	0.989760606	9.081016759	9.396454291	8.511888463	9.569217728	9.52928986	9.448650487	9.603161713
ENSG00000147853	AK3	0.196565549	0.821777402	0.793094323	0.295788824	0.989659873	9.771132748	8.255926468	8.296287027	9.136211578	9.16894939	9.662803762	9.824224109
ENSG00000148180	GSN	-0.341803699	0.222084596	1.329588194	3.17E-08	0.987784495	11.50945836	11.42768197	11.4724895	11.0877485	11.1685936	12.61732815	12.95921509
ENSG00000116212	LRRC42	0.206151623	0.89994774	0.781614332	0.596648352	0.987765955	4.467311984	9.067150531	7.777116083	8.23312837	8.21902896	8.599816608	8.967268538
ENSG00000014641	MDH1	0.454799739	0.252546786	0.532893651	0.164653895	0.98769339	9.128497779	9.608085847	9.767769415	9.978816937	9.97960581	9.937430448	10.16748181
ENSG00000006125	AP2B1	-0.059340782	0.906842758	1.046305156	0.007498635	0.986964374	10.70134604	10.51971956	11.19694409	10.75866452	10.7900919	11.69591971	12.04029115
ENSG00000141219	C17orf80	0.342885744	0.812947514	0.643422318	0.635522997	0.986308062	7.579279349	7.901717006	4.058998049	7.574744049	7.5462416	8.113585664	7.540464021
ENSG00000128191	DGCR8	0.408678617	0.774885822	0.576114862	0.676798727	0.984793479	8.928409675	8.356994601	4.874587708	8.543652346	8.54024645	8.660513226	8.753366804
ENSG00000156858	PRR14	0.358733407	0.35676743	0.624980194	0.082216561	0.9837136	7.709880939	8.169643011	7.858115942	8.217201935	8.35656898	8.588177878	8.514222325
ENSG00000126777	KTN1	1.232746047	0.301721022	-0.249059101	0.852211698	0.983686946	7.940205782	9.583268459	11.32712828	11.44115415	11.4604341	10.03738467	9.9049832
ENSG00000174428	GTF2IRD2B	2.16333317	0.004958412	-1.1859433	0.148125821	0.97738987	6.138360429	6.140560021	4.058998049	7.842101511	7.7866208	4.555645384	4.80186789
ENSG00000138190	EXOC6	0.211431143	0.92098972	0.765865694	0.69597295	0.977296837	7.750913381	5.131603625	2	6.470063213	6.72891807	7.142599214	7.138761401
ENSG00000165102	HGSNAT	0.205670872	0.571578307	0.770397839	0.014292331	0.976068711	10.1770105	10.05576005	9.714382178	10.19904132	10.2039308	10.63902691	10.88021462
ENSG00000186314	PRELID2	-0.145847117	0.861743464	1.120327607	0.097911265	0.97448049	5.532353208	5.838937808	4.874587708	5.128039203	5.56313034	6.597565628	6.487779256
ENSG00000197965	MPZL1	0.562287718	0.213853811	0.412004017	0.4954739	0.974291735	10.70134604	11.30149645	11.57188978	11.79739298	11.796598	11.61238761	11.68058894
ENSG00000167525	PROCA1	-0.437619502	0.801824404	1.409791463	0.350123893	0.972171962	3.327711234	4.11352228	2	3.023133209	3.33976443	4.487545924	4.489993931
ENSG00000139990	DCAF5	0.293798487	0.740549771	0.678148369	0.406804715	0.971946856	8.418903792	9.203675681	10.34701993	9.757180673	9.90230962	10.18712501	10.24124211
ENSG00000141985	SH3GL1	0.251616342	0.685162585	0.719270908	0.192814682	0.97088725	9.497412313	10.63690565	9.888372618	10.31488156	10.3657986	10.692232	10.91329715
ENSG00000006740	ARHGAP44	-2.000977692	0.160968748	2.971857812	0.023498087	0.970880121	5.866944937	4.710564154	2	3.754611435	2.86123014	7.652523812	7.697107799
ENSG00000184056	VPS33B	1.409591725	0.38839201	-0.4405703	0.803056929	0.969021425	5.532353208	7.002537973	2	7.292639438	7.23044321	5.180212365	5.772630506
ENSG00000175746	C15orf54	3.275372785	0.463403238	-2.306869944	0.621054729	0.968502842	2	2	2	3.921164147	3.65239003	2.139668783	2
ENSG00000153107	ANAPC1	1.46230928	0.438477481	-0.494509678	0.806845941	0.967799602	7.384433967	8.717821467	2	9.097585392	9.04730166	7.067324363	7.221358049
ENSG00000103199	ZNF500	0.368055054	0.841373243	0.59875438	0.730981666	0.966809434	2	7.051611315	7.225742779	6.89733479	6.96881778	7.267148095	7.038185015
ENSG00000096746	HNRNPH3	0.640198224	0.283681909	0.326526354	0.600974746	0.966724578	9.848787621	8.94259736	8.742726175	9.936887136	9.85793083	9.753944082	9.395240163
ENSG00000184465	WDR27	-0.232320908	0.866469882	1.198849994	0.309292345	0.966529086	3.327711234	6.535001748	6.534109959	5.829220145	5.80356929	7.447120996	6.856025745
ENSG00000179172	HNRNPCL1	5.693619691	0.000723945	-4.72751968	0.050406299	0.966100011	2	3.076665209	2	6.772115916	6.70088158	2.048074405	2
ENSG00000065154	OAT	0.287525884	0.576906213	0.676414691	0.14670132	0.963940574	10.08373159	10.21502048	9.385998894	10.2136641	10.2399348	10.40756301	10.79568025
ENSG00000151746	BICD1	-0.318323161	0.841373243	1.281139929	0.357318652	0.962816768	4.815609471	6.535001748	8.865353883	7.230526165	7.36158648	8.973764228	8.762965592
ENSG00000151687	ANKAR	0.826485518	0.566726644	0.13592821	0.932067558	0.962413728	4.815609471	4.11352228	2	4.783303513	4.65713986	4.45225155	3.789585079
ENSG00000157916	RER1	0.785540321	0.052158739	0.176086749	0.701998223	0.96162707	10.03472846	9.975384048	9.399592459	10.63487332	10.594821	9.816217077	10.17638265
ENSG00000112246	SIM1	6.152526882	0.045644094	-5.191133054	0.147030332	0.961393828	2	2	2	6.197166296	6.31350454	2	2
ENSG00000130985	UBA1	0.440666976	0.136814806	0.517710141	0.070779599	0.958377117	11.26227543	11.41367288	11.47570169	11.78038858	11.8717671	11.71605378	12.06987785
ENSG00000239732	TLR9	2.215435294	NA	-1.257443073	NA	0.957992221	2	2	2	3.349643765	2.86123014	2.225793514	2.059860178
ENSG00000164031	DNAJB14	0.823251859	0.342311654	0.134148093	0.89140859	0.957399952	7.867441304	7.503546079	9.31604076	9.363935069	9.15970984	8.828207602	8.284971554
ENSG00000127080	IPPK	1.797416607	0.340701465	-0.840604118	0.673761372	0.956812489	4.467311984	7.503546079	2	7.831679003	7.89946959	5.085488617	5.576534358
ENSG00000132382	MYBBP1A	0.630073531	0.498588661	0.326632109	0.734171071	0.956705639	7.031698319	8.395519306	9.31604076	9.100844036	9.19330273	8.702422971	8.977974666
ENSG00000163788	SNRK	0.676046577	0.738698334	0.280070079	0.895191045	0.956116656	8.855032352	7.538394143	2	8.413424144	8.4504872	8.195243072	7.869046644
ENSG00000111077	TNS2	-0.007060232	0.988487935	0.9625769	0.010218512	0.955516669	7.485143553	7.819099893	7.858115942	7.608714138	7.83385794	8.544855285	8.807298241
ENSG00000168795	ZBTB5	-0.302071613	0.845142817	1.257001835	0.352660759	0.954930223	9.015175615	5.946638857	5.772716771	7.423326516	7.42465798	8.985770579	8.93516457
ENSG00000181610	MRPS23	0.662266074	0.563764353	0.286075545	0.812905528	0.948341619	5.330594134	7.538394143	8.434763955	8.184811706	8.26379462	7.908330388	7.79832288
ENSG00000113163	COL4A3BP	0.78690186	0.315977132	0.161120301	0.854072961	0.948022162	6.816637977	8.255926468	8.677268317	8.859280876	8.91322288	8.425380879	8.089704419
ENSG00000108474	PIGL	-0.328546146	0.908151255	1.276095261	0.617024792	0.947549115	2	7.099070191	2	5.461211713	5.14724905	7.094763493	6.471341372
ENSG00000113391	FAM172A	-0.632176171	0.77110538	1.577981272	0.428900076	0.945805101	2	4.93638933	8.206335842	6.069927018	6.26836889	8.138086022	8.479026367
ENSG00000270276	HIST2H4B	6.136220417	0.047200445	-5.19113285	0.148400754	0.945087567	2	2	2	6.205262847	6.27599024	2	2
ENSG00000270882	HIST2H4A	6.136220417	0.047200445	-5.19113285	0.148400754	0.945087567	2	2	2	6.205262847	6.27599024	2	2
ENSG00000197054	ZNF763	1.93776038	0.124543846	-0.993223815	0.459077723	0.944536565	4.467311984	3.076665209	3.369317972	5.48800675	5.11361705	3.316666208	2.885414003
ENSG00000204604	ZNF468	0.39481804	0.641177228	0.54794382	0.498062551	0.942761861	7.975256505	7.35507212	6.203069917	7.683067677	7.78928606	8.173735115	7.529919928
ENSG00000144504	ANKMY1	0.006628273	0.997720641	0.934353219	0.715181868	0.940981492	2	6.728495673	2	5.37770462	5.16377554	6.444330878	5.727202496
ENSG00000106526	ACTR3C	4.722420473	0.104495453	-3.783183945	0.271243655	0.939236529	2	2	2	4.887609717	5.00774437	2.048074405	2
ENSG00000182704	TSKU	1.333894466	0.101156806	-0.395120135	0.66187124	0.93877433	10.4253394	9.267380431	8.35328246	10.88185282	10.9629415	9.168473351	9.232450166
ENSG00000182022	CHST15	-0.357797314	0.896917828	1.295610925	0.603099089	0.937813611	2	3.076665209	8.65477198	6.722207358	6.80463998	7.96967964	8.700282474
ENSG00000137124	ALDH1B1	-1.736201203	0.15408844	2.67070397	0.019811774	0.934502768	9.556468883	9.338312924	5.772716771	7.182120794	7.24604864	11.48917421	11.67267253
ENSG00000176058	TPRN	-0.198993166	0.897826717	1.133271685	0.396880252	0.934278519	7.53297911	7.099070191	3.369317972	6.5612028	6.64900057	8.001311979	7.795024296
ENSG00000137040	RANBP6	0.447299193	0.832297095	0.48576192	0.813013269	0.933061113	2	8.623598428	8.631919287	8.469961514	8.51470562	8.663409709	8.38416207
ENSG00000167658	EEF2	-0.458414385	0.092448935	1.389973675	7.63E-09	0.93155929	13.98403793	13.59338974	13.78504471	13.32578388	13.349718	15.15818234	15.21287184
ENSG00000136709	WDR33	0.504663202	0.348531182	0.426873457	0.426694614	0.931536659	7.667647223	8.671479029	8.631919287	8.896009193	8.89477851	8.800408416	8.835935827
ENSG00000214491	SEC14L6	-3.753044207	0.478107313	4.683926509	0.319695652	0.930882302	2	2	2	2	2	4.240999983	5.373314945
ENSG00000054611	TBC1D22A	0.092945177	0.95203552	0.837260472	0.549261882	0.930205649	5.330594134	7.467835385	9.344432377	8.197043419	8.32935768	8.582578161	9.325696621
ENSG00000160209	PDXK	0.391451624	0.586677368	0.538272345	0.43412023	0.929723969	10.60951384	9.903691196	9.049077186	10.36723728	10.3923374	10.50200781	10.55007877
ENSG00000166579	NDEL1	0.169276525	0.58791184	0.760162197	0.003916559	0.929438721	9.790941832	9.624396729	9.452720984	9.755803193	9.83940415	10.43832796	10.33356098
ENSG00000021574	SPAST	0.403711937	0.553397759	0.525586259	0.421989779	0.929298196	6.009025997	6.899080544	7.398165132	7.292639438	7.23826702	7.52030827	7.234405279
ENSG00000174697	LEP	-3.273501426	0.257133882	4.202783823	0.04664104	0.929282398	2	2	3.369317972	2	2.14022021	4.952879058	5.646532192
ENSG00000171467	ZNF318	0.160372977	0.807381293	0.767802743	0.172094144	0.928175721	8.257427002	8.317412869	9.301632639	8.868232317	8.8597051	9.574262696	9.351374718
ENSG00000135069	PSAT1	-0.843829148	0.330982566	1.770155812	0.0252943	0.926326664	8.107517749	9.514922632	7.691296561	7.880528731	7.7866208	10.72217346	10.05546557
ENSG00000196323	ZBTB44	0.564131808	0.139917893	0.361776331	0.35529554	0.925908139	7.940205782	7.847167909	8.076950877	8.595396228	8.41985547	8.465761957	8.135631371
ENSG00000134900	TPP2	1.279534093	0.019532885	-0.354108996	0.566151826	0.925425096	7.867441304	8.834582582	7.777116083	9.516508835	9.52849248	7.775419242	8.024537783
ENSG00000162994	CLHC1	0.428936699	0.869954492	0.495908778	0.845529489	0.924845477	2	6.228547981	2	5.076005082	5.30456641	5.357600875	5.14363432
ENSG00000148606	POLR3A	0.923141841	0.546911592	0.001096196	0.999379962	0.924238036	4.467311984	8.147241016	9.413059136	9.263797825	9.28868707	8.419110791	8.303661547
ENSG00000141873	SLC39A3	0.261054917	0.804796579	0.661390601	0.491172843	0.922445518	7.904282243	8.213429689	5.772716771	7.810605345	7.95749494	8.21774931	8.340330468
ENSG00000081059	TCF7	1.735139078	0.174638808	-0.812848508	0.547277519	0.922290569	4.00722531	7.002537973	4.523675391	7.366699513	7.61094772	4.933813368	5.23734678
ENSG00000174013	FBXO45	0.190028402	0.845522872	0.732212602	0.392103714	0.922241004	6.138360429	6.311476754	7.691296561	7.161462517	6.96410766	7.936540212	7.145684098
ENSG00000118257	NRP2	1.286851975	0.18345602	-0.365231606	0.731701381	0.921620369	7.904282243	9.943237334	10.57976717	11.10272512	11.1482347	9.622689032	9.319587711
ENSG00000104142	VPS18	0.336098373	0.673891173	0.585371264	0.434090596	0.921469637	7.579279349	8.88960062	9.330306409	9.089953145	9.11579188	9.258063226	9.437458576
ENSG00000136108	CKAP2	0.322096409	0.886772235	0.598836776	0.779280283	0.920933185	2	8.671479029	6.073378139	7.64488235	7.63485389	7.801071566	8.014172904
ENSG00000176393	RNPEP	0.298304148	0.516317205	0.622613483	0.13571439	0.920917631	9.689059027	9.00621767	9.031722608	9.538304826	9.60612566	9.848602345	9.940746393
ENSG00000182718	ANXA2	0.572992579	0.339969687	0.347057397	0.574245941	0.920049975	12.53220074	12.90092286	11.97287769	13.08541585	13.0958833	12.36465981	13.23554421
ENSG00000108551	RASD1	2.279770452	0.154170396	-1.361551386	0.411359472	0.918219065	6.563806279	7.315436877	2	8.655725489	8.66593596	5.646229369	4.468068808
ENSG00000077549	CAPZB	0.124576649	0.78138675	0.793394116	0.033633667	0.917970765	10.85038273	10.60848977	11.07092887	10.93576629	11.0202196	11.36604996	11.88101829
ENSG00000071564	TCF3	0.049191117	0.922182516	0.868381052	0.02966485	0.917572169	9.371511179	9.591588447	8.845626171	9.331013912	9.37992356	10.27544091	10.05844914
ENSG00000177943	MAMDC4	-0.271815082	0.82182886	1.187726095	0.251562038	0.915911014	4.815609471	4.11352228	6.322058544	4.846787657	5.33407135	6.437578914	6.482320745
ENSG00000121764	HCRTR1	-2.312619827	0.625382427	3.227469533	0.431879804	0.914849705	2	2	2	2	2.14022021	3.5515855	3.940519005
ENSG00000168702	LRP1B	-1.221676017	0.650879996	2.136415202	0.390307972	0.914739185	2	2	4.874587708	3.162721474	2.59489879	3.483289517	6.099376274
ENSG00000011426	ANLN	-0.852311962	0.39960716	1.766858142	0.054043826	0.91454618	4.815609471	6.899080544	7.342949392	5.968925393	5.7929843	8.065491673	8.702044649
ENSG00000151366	NDUFC2	1.998035816	1.22E-07	-1.084724888	0.006842904	0.913310928	7.940205782	8.124485672	7.342949392	9.825086737	9.83360578	6.750676314	6.834765628
ENSG00000166510	CCDC68	2.579521469	0.148604754	-1.666536728	0.36789886	0.912984741	2	4.93638933	2	6.006476466	5.86549842	2.937246078	2.671842496
ENSG00000198791	CNOT7	0.563324495	0.382177977	0.347609504	0.599062079	0.910933999	9.128497779	9.277729677	7.934808822	9.481935579	9.42860336	9.284642781	9.19623278
ENSG00000177000	MTHFR	0.217303306	0.858812103	0.692522158	0.527241133	0.909825464	5.866944937	8.806266215	8.35328246	8.339946438	8.33118782	8.948260474	8.647610514
ENSG00000115421	PAPOLG	0.740952464	0.47470898	0.168631681	0.882958039	0.909584146	7.750913381	6.666836837	5.39244166	7.68016596	7.56502709	7.450478673	6.538630327
ENSG00000148384	INPP5E	0.018377555	0.993323827	0.890652875	0.62915305	0.909030429	8.910412251	3.686303414	5.39244166	7.464384256	7.52720828	8.359038717	8.348604099
ENSG00000073803	MAP3K13	0.28406475	0.789639588	0.623017578	0.525386697	0.907082329	5.532353208	7.60566235	7.97167868	7.632926745	7.64076905	7.715279732	8.186029473
ENSG00000121933	TMIGD3	-1.303156549	NA	2.209632429	NA	0.906475879	2	2	2	2.270786764	2	3.374372158	2.817676617
ENSG00000180785	OR51E1	-3.753019292	0.508933699	4.658420497	0.362166499	0.905401205	2	2	2	2	2	5.673303969	3.071407821
ENSG00000153885	KCTD15	-0.762435854	0.705011053	1.667707654	0.366065794	0.905271799	6.366709671	8.433041974	2	6.496695859	6.3857277	9.095643988	8.472158355
ENSG00000101417	PXMP4	-0.490532569	0.699868245	1.393516287	0.219792859	0.902983718	6.138360429	4.710564154	7.777116083	6.078767801	6.37866593	8.100821584	8.025476363
ENSG00000173145	NOC3L	0.778773877	0.561910399	0.123856322	0.933434132	0.902630199	4.467311984	7.393647515	8.408111533	8.27796118	8.17690623	7.6993745	7.459438299
ENSG00000181315	ZNF322	1.923333267	0.401419262	-1.020850463	0.669668955	0.902482805	3.327711234	8.169643011	2	8.556367783	8.52912793	6.167993056	4.998228167

ENSG00000145331	TRMT10A	0.292390824	0.848343006	0.609645708	0.668751914	0.902036532	5.866944937	5.131603625	2	5.22679461	5.34860058	5.4091291	5.727202496
ENSG00000197702	PARVA	-0.273296636	0.617138584	1.175091892	0.011742973	0.901795256	11.53317465	11.00402286	10.53735535	10.78388778	10.8343167	12.12445714	12.37594932
ENSG00000169299	PGM2	0.560930906	0.679562529	0.339875403	0.806377308	0.900806309	5.095959458	8.929529568	8.35328246	8.70652031	8.69749358	8.360824221	8.596104698
ENSG00000233276	GPX1	0.638104076	0.096396156	0.262432478	0.523669368	0.900536554	10.33377634	9.741182361	10.38167985	10.8110007	10.8154823	10.41287971	10.4637325
ENSG00000108381	ASPA	-2.312620553	0.625452298	3.21211454	0.434435201	0.899493987	2	2	2	2	2.14022021	3.865109708	3.620995423
ENSG00000275774	HNRNPCL2	6.090511484	0.052262733	-5.191132222	0.152607456	0.899379261	2	2	2	6.24507773	6.14893615	2	2
ENSG00000180730	SHISA2	0.12113734	0.895301283	0.777710723	0.314028074	0.898848063	5.095959458	4.93638933	4.874587708	4.946755474	5.19627118	5.463319095	5.85096143
ENSG00000164604	GPR85	2.158465299	0.258233759	-1.259768128	0.525988168	0.898697171	2	5.131603625	2	5.808110275	5.65950892	3.193831101	3.012034821
ENSG00000168939	SPRY3	6.089355567	0.053105198	-5.191132041	0.153638105	0.898223526	2	2	2	6.043074888	6.33555364	2	2
ENSG00000085978	ATG16L1	0.167973519	0.925390417	0.72743341	0.657735862	0.895406929	9.048471759	6.844429755	4.058998049	7.93753157	7.95749494	8.639092784	8.344849238
ENSG00000277445		6.086068487	0.052698164	-5.191132177	0.152915343	0.89493631	2	2	2	6.213314212	6.17362678	2	2
ENSG00000112234	FBXL4	0.022881158	0.988321837	0.870956549	0.523326453	0.893837707	7.709880939	7.874700259	4.058998049	7.315259362	7.26532099	8.545902039	7.519298205
ENSG00000176105	YES1	1.020375881	0.45109867	-0.129095132	0.932122597	0.891280749	6.138360429	8.702538795	10.21486071	10.17656808	10.1014956	9.035431928	8.955990164
ENSG00000253729	PRKDC	0.130856207	0.881476754	0.759696252	0.306456109	0.890552459	10.25752168	10.76380876	8.960138144	10.33577583	10.2722069	10.85827134	11.0013221
ENSG00000170540	ARL6IP1	0.921247843	0.013687742	-0.030974428	0.947678709	0.890273414	9.867565369	9.828248506	9.25752426	10.6209439	10.5725591	9.772842777	9.511677672
ENSG00000116785	CFHR3	6.081321087	0.053285833	-5.191132085	0.153445957	0.890189002	2	2	2	6.130704144	6.24526024	2	2
ENSG00000105323	HNRNPUL1	0.258289549	0.614578038	0.630428437	0.173219998	0.888717986	10.09970294	10.96932597	10.33998689	10.78253558	10.7730255	11.05931184	11.23378941
ENSG00000134283	PPHLN1	0.568997041	0.256686981	0.319564774	0.538569152	0.888561816	8.042902253	8.777382925	7.934808822	8.890998469	8.86097251	8.536980328	8.71548362
ENSG00000041357	PSMA4	1.089045304	0.051734578	-0.203152552	0.753039803	0.885892752	9.895281535	10.00059544	8.784770914	10.75659993	10.7278927	9.439949402	9.469883152
ENSG00000142751	GPN2	-0.060073814	0.903601303	0.945689283	0.014292331	0.885615469	8.16936288	7.572420273	7.600047325	7.748245381	7.7377845	8.751793877	8.718388644
ENSG00000107882	SUFU	0.306686545	0.624119761	0.578772212	0.317915266	0.885458757	8.075571721	8.337339476	7.098165388	8.244958867	8.23280093	8.496406213	8.51889534
ENSG00000100299	ARSA	0.760705594	0.462743191	0.123745651	0.914220926	0.884451246	9.810482605	9.540933215	7.163363831	9.896204982	10.0513059	9.280561171	9.401037612
ENSG00000144028	SNRNP200	0.267618875	0.627929301	0.615638492	0.220561335	0.883257367	9.800745303	10.69982928	9.878698603	10.47538626	10.437637	10.73931845	10.86548261
ENSG00000055483	USP36	0.456804579	0.837724165	0.425238463	0.846443289	0.882043042	9.646202137	8.522788974	2	9.044420506	9.08358266	9.081998027	8.98184819
ENSG00000153147	SMARCA5	1.461034258	0.230018039	-0.582050769	0.656825095	0.87898349	6.73718411	10.42046463	10.03493575	11.18281219	11.1680817	9.368088514	8.869253401
ENSG00000100209	HSCB	1.285765749	0.122091309	-0.407201284	0.657203666	0.878564465	6.468621203	6.666836837	4.523675391	7.423326516	7.39346687	5.83618166	5.740981724
ENSG00000089472	HEPH	0.897327111	0.344828673	-0.019938651	0.985591183	0.87738846	7.159102577	8.955547846	9.647595056	9.782429661	9.81672282	8.752700801	9.011994108
ENSG00000138834	MAPK8IP3	0.149487957	0.941569674	0.727848987	0.700873168	0.877336943	3.327711234	9.357954881	10.11951029	9.318005579	9.40098777	10.23155211	9.562677828
ENSG00000177685	CRACR2B	-1.277880903	0.525895193	2.155135907	0.229495532	0.877255004	4.00722531	3.076665209	2	2.947924294	2.2680111	4.530483275	5.080008298
ENSG00000164885	CDK5	1.173767623	0.357945396	-0.298390699	0.832869996	0.875376925	5.866944937	7.051611315	3.369317972	7.144720234	7.28433928	5.853194273	5.768151538
ENSG00000166912	MTMR10	2.061851837	0.196711636	-1.186910784	0.474542652	0.874941053	5.095959458	7.002537973	2	7.72026832	7.86955761	4.513461373	4.959465495
ENSG00000103494	RPGRIP1L	0.776031139	0.449317736	0.098056787	0.932122597	0.874087926	5.095959458	7.431218269	5.930865648	7.234487582	7.26914476	6.637393426	6.554323192
ENSG00000185728	YTHDF3	0.565985618	0.490472675	0.308067801	0.717164009	0.874053418	8.493334308	10.34373336	10.16797309	10.47287335	10.3997688	10.20612015	10.15273873
ENSG00000100036	SLC35E4	0.050659701	0.975506192	0.823303735	0.577037638	0.873963436	3.327711234	7.145017454	7.934808822	7.057964187	7.12888795	7.786974998	7.908246991
ENSG00000138279	ANXA7	1.163196814	0.001734804	-0.290430333	0.494470997	0.872766481	9.509418609	9.203675681	8.784770914	10.33393077	10.3751428	8.818278819	8.993886349
ENSG00000167904	TMEM68	0.273580879	0.726499281	0.597706096	0.406622399	0.871286976	7.485143553	6.602424674	6.534109959	7.222570549	7.18258783	7.904255004	6.990916783
ENSG00000101266	CSNK2A1	0.017037327	0.982192754	0.853062979	0.169861871	0.870100306	9.87686338	8.487557258	9.681375057	9.464333207	9.4921465	10.31204759	10.30948877
ENSG00000278608		-1.051907038	0.54826185	1.921945112	0.23044932	0.870038074	5.532353208	3.076665209	2	3.406930997	3.60476711	5.846413283	6.055940621
ENSG00000184988	TMEM106A	1.122529167	0.215763131	-0.252667569	0.803497966	0.869861598	6.009025997	7.928237109	6.203069917	8.136938566	8.07792246	6.732402829	6.797888492
ENSG00000108556	CHRNE	1.164843359	0.641266479	-0.295981584	0.912467872	0.868861775	2	2	4.058998049	3.754611435	3.60476711	3.1504466	2.466008509
ENSG00000103061	SLC7A6OS	0.581916242	0.75774249	0.286431994	0.884256906	0.868348236	2	5.457120953	7.398165132	6.682159616	6.76185814	6.533574091	6.338680316
ENSG00000140612	SEC11A	0.694762107	0.002963985	0.173078253	0.517968728	0.86784036	9.820154628	9.792607274	9.589475645	10.43593562	10.4274075	9.90822107	9.915400542
ENSG00000015475	BID	0.692624333	0.315574141	0.174894954	0.818501743	0.867519287	8.340423424	8.655694736	7.098165388	8.808365552	8.91566441	8.390261842	8.309838123
ENSG00000169413	RNASE6	-3.753043198	0.479762551	4.619939118	0.329937069	0.86689592	2	2	2	2	2	5.201624686	4.411752372
ENSG00000130270	ATP8B3	0.303113923	0.879863666	0.563319946	0.763737563	0.866433869	5.330594134	2	3.369317972	4.139692846	4.60967607	4.73522696	4.468068808
ENSG00000065911	MTHFD2	0.170554272	0.870694345	0.695207239	0.450551283	0.865761511	9.740899611	9.277729677	7.646393253	9.319871109	9.27353851	10.22666466	9.247812734
ENSG00000196616	ADH1B	-3.753029746	0.496719668	4.618022397	0.352068654	0.864992652	2	2	2	2	2	3.584558147	5.524371359
ENSG00000186567	CEACAM19	0.14689704	0.888172933	0.716469029	0.429217146	0.863366069	7.27616306	5.303523747	7.398165132	7.123515401	6.96410766	7.68709225	7.509939612
ENSG00000196187	TMEM63A	0.902111331	0.263458099	-0.040304867	0.966513683	0.861806463	8.963744667	7.145017454	8.922968322	9.384581425	9.49623046	8.63565547	8.365010363
ENSG00000163251	FZD5	0.696367696	0.777603672	0.165303749	0.948615522	0.861671446	2	2	5.39244166	4.76150579	4.37434574	4.301748724	3.987514168
ENSG00000164951	PDP1	1.137783553	0.309598975	-0.276432394	0.823246972	0.861351159	6.73718411	7.315436877	9.426401271	9.483600848	9.38873772	7.851893762	8.200230127
ENSG00000067955	CBFB	0.750150736	0.586657904	0.105633508	0.944101725	0.855784244	4.815609471	8.469613413	8.865353883	8.868232317	8.90587341	8.293242958	8.204380326
ENSG00000185869	ZNF829	0.289262986	0.741924158	0.566170355	0.487033897	0.855433341	6.009025997	5.131603625	5.156626401	5.797438367	5.69405321	6.315225883	5.606950214
ENSG00000184162	NR2C2AP	0.296627234	0.851528683	0.558524888	0.706382819	0.855152122	2	5.838937808	5.59507254	5.406080789	5.53799497	5.957567336	5.419744333
ENSG00000110435	PDHX	0.810141002	0.530053668	0.044886282	0.974562729	0.855027284	5.095959458	8.124485672	8.825624954	8.790986483	8.80544247	8.146391268	7.929461924
ENSG00000196739	COL27A1	0.414369583	0.832844524	0.44059289	0.818406267	0.854962473	4.00722531	6.602424674	9.69246155	8.612239836	8.78288532	8.620830655	8.824117457
ENSG00000254004	ZNF260	0.428945275	0.703668984	0.425073265	0.701904883	0.85401854	5.866944937	8.487557258	8.236946975	8.386974157	8.27334517	8.671105351	7.875307424
ENSG00000106479	ZNF862	0.484074915	0.711397752	0.365834806	0.781762153	0.849909721	5.095959458	6.666836837	8.266922072	7.723090567	7.62891439	7.935741825	7.052989523
ENSG00000198876	DCAF12	0.045116198	0.984043262	0.804645547	0.702841297	0.849761745	2	9.225224669	9.601288346	8.866956935	8.91200057	9.601198809	9.685927623
ENSG00000164111	ANXA5	1.327146299	0.007634516	-0.477581154	0.383233513	0.849565144	12.53220074	11.53079123	11.86432331	13.36165586	13.3648174	11.31669201	11.76793522
ENSG00000124216	SNAI1	1.457601135	0.092161915	-0.60805415	0.513107667	0.849546984	6.89194353	4.93638933	7.098165388	7.994705029	7.95512324	5.909581027	6.048572483
ENSG00000116906	GNPAT	-0.054544392	0.913792484	0.903966368	0.022591207	0.849421975	8.340423424	8.655694736	7.896971941	8.289431124	8.28283292	9.200732369	9.26976224
ENSG00000166035	LIPC	-3.753045051	0.478107313	4.60227063	0.329251531	0.849225578	2	2	2	2	2	4.894909044	4.793076545
ENSG00000028528	SNX1	0.543793405	0.389803748	0.302797257	0.643384314	0.846590662	10.1770105	9.599860728	8.825624954	10.16675444	10.1893748	9.830461743	10.03813112
ENSG00000136938	ANP32B	0.176496191	0.85461878	0.66970055	0.431645387	0.846196741	9.820154628	9.624396729	7.777116083	9.53348965	9.45234084	9.921992929	10.04416401
ENSG00000198589	LRBA	0.220023917	0.903601303	0.624642391	0.709548861	0.844666308	4.467311984	7.761270542	9.746650996	8.713632895	8.75042728	8.942707845	9.301879118
ENSG00000139974	SLC38A6	1.775907192	0.170726471	-0.931480008	0.492815255	0.844427184	6.138360429	5.457120953	2	7.035434774	6.9210099	4.530483275	4.479073019
ENSG00000111412	C12orf49	0.058810721	0.962673456	0.785016795	0.483687127	0.843827515	9.567995545	6.464272742	8.460932916	8.692189072	8.7045705	9.439386236	9.398504094
ENSG00000145979	TBC1D7	0.95345227	0.483591352	-0.109864133	0.942375924	0.843588137	4.00722531	6.728495673	8.007629717	7.826439377	7.99029569	6.816344356	6.928061817
ENSG00000137942	FNBP1L	0.243594988	0.86389717	0.598431476	0.646493605	0.842026464	4.815609471	8.376385542	6.882775709	7.549526251	7.58049694	8.007404089	7.81253023
ENSG00000115602	IL1RL1	1.050376516	0.706793237	-0.208700024	0.944101725	0.841676493	3.327711234	2	2	3.09461492	2.94009902	2.717032197	2.277179783
ENSG00000123353	ORMDL2	0.69694514	0.461193236	0.143006378	0.89090828	0.839951518	5.70932728	7.847167909	7.552162999	7.997038782	7.96694292	7.039353222	7.751441934
ENSG00000123505	AMD1	0.653863444	0.190053842	0.183977645	0.737229272	0.837841089	8.517314938	9.28800521	8.960138144	9.634381252	9.58403885	8.825624065	9.402122045
ENSG00000106070	GRB10	0.139451949	0.877879848	0.698114301	0.369714606	0.83756625	7.33131396	8.834582582	9.099922433	8.697938662	8.78422202	9.528926528	9.018137056
ENSG00000163703	CRELD1	0.523771572	0.540424469	0.312389379	0.723575557	0.836160951	9.189468652	9.415325457	7.451345288	9.388142127	9.47486651	9.126758239	9.311530309
ENSG00000125810	CD93	-3.240232983	0.241642985	4.074703051	0.080865234	0.834470068	4.00722531	2	2	2	2.2680111	6.750676314	4.935697922
ENSG00000197927	C2orf27A	6.024854329	0.059854337	-5.191131311	0.158650282	0.833723018	2	2	2	6.155987605	6.11534392	2	2
ENSG00000108055	SMC3	0.613629925	0.528710992	0.219942769	0.832583267	0.833572694	6.653098118	8.487557258	9.083172337	8.983347946	8.99861459	8.727088905	8.462487876
ENSG00000157837	SPPL3	0.60113162	0.055580779	0.230012424	0.498291593	0.831144043	8.674867122	8.732943944	8.631919287	9.155143657	9.39224836	8.807408592	9.006300384
ENSG00000204991	SPIRE2	0.768574185	0.518554054	0.062538131	0.962746693	0.831112316	3.327711234	4.11352228	5.59507254	5.258265913	5.33407135	4.813804156	4.532869193
ENSG00000124067	SLC12A4	-0.308948409	0.64165426	1.139991917	0.048141929	0.831043507	10.64259676	9.277729677	10.06936278	9.780398828	9.80096123	11.24663807	11.22431746
ENSG00000004455	AK2	0.33594499	0.588546732	0.494004961	0.401725514	0.829949951	9.509418609	9.067150531	10.22252928	10.0010786	10.0204455	9.97191327	10.34111971
ENSG00000143390	RFX5	0.601621043	0.632030586	0.226184039	0.864675088	0.827805082	9.233563228	9.308339287	6.073378139	9.384581425	9.33957954	8.590716036	9.301491724
ENSG00000151729	SLC25A4	-1.148721657	0.021949338	1.976256754	2.29E-05	0.827535097	8.19931886	8.276714465	9.116480286	7.501020868	7.41433569	10.39163988	10.69986899
ENSG00000164902	PHAX	0.16016597	0.891428469	0.666155953	0.521375485	0.826321923	7.031698319	8.607278467	9.757248847	8.99156386	9.02712059	9.390403917	9.624380013
ENSG00000127311	HELB	-3.75303753	0.487478481	4.578340914	0.345156474	0.825303385	2	2	2	2	2	5.289239922	4.133950982
ENSG00000176261	ZBTB8OS	0.66195109	0.290102247	0.163284069	0.81284055	0.825235159	6.96351253	6.666836837	6.073378139	7.277360022	7.23826702	6.430795201	7.052989523
ENSG00000162885	B3GALNT2	1.274167524	0.03553166	-0.449649254	0.498871333	0.82451827	6.366709671	7.761270542	7.098165388	8.399377854	8.50176361	6.833591108	6.693102083
ENSG00000103489	XYLT1	1.241140879	0.33935585	-0.419215645	0.765333198	0.821925234	5.532353208	8.968383114	8.784770914	9.562930393	9.62936766	8.512558892	7.023227011
ENSG00000144893	MED12L	1.491947248	0.466289371	-0.67080781	0.755643195	0.821139438	2	4.11352228	2	4.299492648	4.02226117	2.911488689	2.746601469
ENSG00000090581	GNPTG	1.456902272	0.000157844	-0.63649638	0.128956336	0.820405892	8.696033109	7.928237109	8.434763955	9.777686595	9.86994125	7.709686122	7.792821043
ENSG00000059145	UNKL	0.497601167	0.819371474	0.322797511	0.883858724	0.820398677	2	6.464272742	8.784770914	7.954442493	7.96458672	7.764669607	7.81035357
ENSG00000127324	TSPAN8	-3.753041074	0.483277973	4.573335546	0.340038826	0.820294472	2	2	2	2	2	4.425202323	5.12973572
ENSG00000170876	TMEM43	0.53320276	0.264473942	0.287043709	0.564270352	0.820246469	9.839306291	9.479495904	10.38851302	10.48414712	10.4753644	10.18963871	10.27773968
ENSG00000157500	APPL1	-0.140283637	0.89458451	0.957485929	0.28290231	0.817202292	6.366709671	8.053983481	8.721235202	7.982979299	7.7377845	9.023833473	8.872386739
ENSG00000126243	LRFN3	-0.184121648	0.930075716	1.000651291	0.595347869	0.816529643	2	7.73146271	8.511888463	7.366699513	7.46855365	8.522696167	8.643336952
ENSG00000134970	TMED7	1.174301886	0.01542766	-0.35892372	0.507672408	0.815378166	9.081016759	10.13189344	9.636156974	10.90440671	10.7993766	9.226421872	9.418291476
ENSG00000159713	TPPP3	-0.633996037	0.776246706	1.446420481	0.466865178	0.812424444	3.327711234	2	3.369317972	2.784612485	2.59489879	4.188345803	3.407202941
ENSG00000113645	WWC1	-0.578770667	0.745616581	1.390428178	0.393412229	0.811657511	3.327711234	4.442710129	7.451345288	5.540148523	5.5755357	6.737909218	7.900082338
ENSG00000078403	MLLT10	0.563691884	0.727489842	0.246867335	0.884612514	0.810559219	3.327711234	7.874700259	8.175061096	8.060896687	7.99722844	7.914016653	7.491038307
ENSG00000179943	FIZ1	-0.216174217	0.910642404	1.025997529	0.544751469	0.809823312	7.33131396	7.467835385	2	6.640968371	6.61935067	7.900986387	7.772838868
ENSG00000158717	RNF166	0.266590644	0.621265967	0.542873113	0.271232177	0.809463757	7.159102577	6.535001748	6.958202216	7.178012736	7.1246732	7.577730839	7.244113847
ENSG00000162688	AGL	0.188936235	0.67705568	0.619438698	0.117356112	0.808374933	7.709880939	7.315436877	7.163363831	7.54315232	7.64959655	8.034504643	8.005636809
ENSG00000103194	USP10	0.670683225	0.286739789	0.136285877	0.846777728	0.806969101	9.358295023	9.514922632	8.206335842	9.772927884	9.8316678	9.139642601	9.39160489
ENSG00000168785	TSPAN5	0.102453402	0.868745346	0.70167226	0.173666446	0.804125662	9.87686338	8.929529568	9.272377397	9.50916971	9.51647861	9.861061271	10.32119294
ENSG00000135469	COQ10A	0.554811035	0.812947514	0.249196039	0.91758425	0.804007074	2	7.431218269	3.369317972	6.503277904	6.50077304	6.159811591	6.261114822
ENSG00000160695	VPS11	0.717707119	0.098800583	0.086214535	0.865952727	0.803921654	9.015175615	8.540087354	8.175061096	9.32080297	9.33867006	8.734453103	8.671191358

ENSG00000124571	XPO5	0.537074534	0.807640508	0.266513584	0.906201712	0.803588118	2	9.608085847	8.784770914	9.232448283	9.18320541	9.117986989	8.735697007
ENSG00000168566	SNRNP48	0.027250279	0.98895591	0.775861933	0.665712192	0.803112211	2	6.602424674	7.691296561	6.658765962	6.75096147	7.484724631	7.376535208
ENSG00000113161	HMGCR	0.657821859	0.642184909	0.145106523	0.92531104	0.802928382	6.257048221	10.30883598	9.849281147	10.19396441	10.2413953	9.19106327	10.08660193
ENSG00000170153	RNF150	-0.515288328	0.710155984	1.317215291	0.291682761	0.801926963	7.159102577	6.666836837	3.369317972	5.900763176	5.9536761	7.944499874	7.380942161
ENSG00000139973	SYT16	-5.525988803	0.016319666	6.327562674	2.26E-05	0.801573871	2	3.686303414	2	2	2	8.037484631	8.249261511
ENSG00000086102	NFX1	0.268601402	0.596790354	0.532570646	0.255070887	0.801172048	8.737455672	9.00621767	8.110401118	8.935480545	8.9398551	9.113757772	9.280809554
ENSG00000118508	RAB32	-0.372588738	0.718561383	1.173755194	0.201042704	0.801166455	9.689059027	8.487557258	7.398165132	8.48998335	8.40429221	9.531835181	10.32653371
ENSG00000166167	BTRC	0.286855192	0.487512694	0.512933018	0.178705011	0.79978821	8.39321512	8.702538795	9.014156722	8.995070693	9.02146427	9.336073933	9.121389737
ENSG00000008869	HEATR5B	0.805403442	0.075112275	-0.005946302	0.991443049	0.799457139	7.867441304	8.433041974	8.076950877	8.95000826	8.94703391	8.387344963	7.855387687
ENSG00000102743	SLC25A15	0.789582192	0.175685887	0.00939077	0.988988478	0.798972961	5.70932728	6.535001748	6.80318718	7.140504127	7.22257674	6.310306306	6.541257688
ENSG00000133112	TPT1	0.54815232	0.396129098	0.250393898	0.713019003	0.798546218	13.2480265	12.32176563	11.95227171	13.16475722	13.159391	12.98073122	12.73798674
ENSG00000170852	KBTBD2	0.67606666	0.24500653	0.122340718	0.852603315	0.798407378	9.556468883	8.57407404	8.486635645	9.665524776	9.59016561	9.145869306	9.00677572
ENSG00000106404	CLDN15	0.336920065	0.800325517	0.459912606	0.718917407	0.796832671	6.468621203	5.457120953	4.523675391	5.930370745	6.08095082	6.765130262	4.935697922
ENSG00000108094	CUL2	0.951809761	0.031800415	-0.155778524	0.764528151	0.796031238	8.340423424	8.124485672	8.960138144	9.508351943	9.40359931	8.413960373	8.301338496
ENSG00000143641	GALNT2	0.330995207	0.599159126	0.462746739	0.441227897	0.793741946	10.70657014	9.876711741	10.48730704	10.73509104	10.7174353	10.32552007	11.24571875
ENSG00000114853	ZBTB47	-0.107273677	0.95872661	0.899071624	0.638141963	0.791797947	5.095959458	7.538394143	11.2124308	9.587916984	9.70541961	10.62175706	10.67884542
ENSG00000197647	ZNF433	0.048819949	0.968309509	0.739149791	0.495902901	0.78796974	4.00722531	6.140560021	4.523675391	5.144975053	5.40529704	5.889930493	5.937276556
ENSG00000266338	NBPF15	2.826620958	1.89E-12	-2.042125544	6.31E-07	0.784495415	9.509418609	8.820493869	8.65477198	11.81237837	11.9057847	7.000176314	7.067643653
ENSG00000132773	TOE1	0.544250285	0.475730142	0.239700084	0.763860964	0.783950369	5.095959458	5.946638857	6.322058544	6.336376195	6.43421531	5.787450547	6.353705253
ENSG00000151923	TIAL1	0.480944899	0.45218119	0.302842651	0.64238228	0.78378755	8.981092835	9.497318007	8.143093325	9.405814997	9.51083771	9.277413594	9.28708453
ENSG00000144224	UBXN4	0.576083489	0.247133389	0.205879857	0.70178553	0.781963347	9.913467515	10.29870826	10.89014791	11.01130667	10.9843247	10.67666663	10.57865558
ENSG00000181513	ACBD4	-0.007765027	0.9947462	0.789450554	0.388123757	0.781685527	5.70932728	4.710564154	6.80318718	5.930370745	5.9725602	6.728720192	6.688368862
ENSG00000161980	POLR3K	0.635042208	0.583878202	0.144760974	0.908703812	0.779803182	4.467311984	6.228547981	7.451345288	7.044488801	7.17037177	6.645228724	6.62538071
ENSG00000006007	GDE1	0.482887973	0.424587993	0.296662533	0.632283385	0.779550506	9.344956677	9.415325457	10.42220168	10.32187999	10.2683912	10.13868678	10.0802927
ENSG00000056972	TRAF3IP2	0.762673903	0.494744255	0.016384721	0.989360801	0.779058624	7.159102577	6.951736442	9.344432377	9.006698952	9.00436103	8.102245405	8.414572237
ENSG00000068697	LAPTM4A	0.838295897	0.25593159	-0.059916177	0.944157185	0.778379719	11.82259453	10.18234215	10.66665689	11.88757367	11.9071656	10.94354834	11.05528124
ENSG00000165113	GKAP1	0.623902657	0.707242233	0.153894679	0.930784885	0.777797336	3.327711234	4.442710129	2	4.237680128	3.94787299	3.891700877	3.600986722
ENSG00000164284	GRPEL2	0.413878932	0.807722186	0.363722849	0.830158879	0.77760178	3.327711234	8.169643011	6.629488387	7.419851705	7.49817903	7.709686122	7.032593922
ENSG00000085840	ORC1	0.917154	0.579813548	-0.14080448	0.938141666	0.77634952	2	4.11352228	3.369317972	4.205751501	3.98554648	3.172301926	3.539239711
ENSG00000089327	FXYD5	1.232159408	0.064313177	-0.457350024	0.527954208	0.774809384	9.189468652	10.00683004	10.60345421	11.29470341	11.2513856	9.143105199	9.929007159
ENSG00000136603	SKIL	0.849416769	0.459780125	-0.074910149	0.953777463	0.77450662	6.96351253	9.896993485	9.165043284	9.960996031	9.94357048	9.471967222	8.399447278
ENSG00000188868	ZNF563	0.187425615	0.83752462	0.58662152	0.466293248	0.774047135	4.467311984	5.131603625	4.874587708	4.927308402	5.13053105	5.4091291	5.367404784
ENSG00000147475	ERLIN2	0.044203307	0.954430302	0.728217684	0.27098548	0.772420992	7.867441304	8.687092496	9.330306409	8.855427477	8.72141482	9.239097094	9.667406786
ENSG00000160818	GPATCH4	0.397662508	0.788243206	0.374427002	0.798199695	0.77208951	8.19931886	7.761270542	4.058998049	7.818544084	7.88209639	7.664138901	7.974870548
ENSG00000211456	SACM1L	0.849545211	0.330348729	-0.077669145	0.938363512	0.771876067	6.816637977	8.732943944	8.960138144	9.294480609	9.25630406	8.401292507	8.310608339
ENSG00000132031	MATN3	-0.285803555	0.865218379	1.05687192	0.475546641	0.771068364	2	5.72254323	4.523675391	4.739373666	4.2865223	5.47655442	5.917188835
ENSG00000196313	POM121	1.571856908	1.15E-05	-0.801139597	0.036686534	0.770717311	9.509418609	8.88960062	8.922968322	10.68711744	10.7104214	8.263645107	8.413138577
ENSG00000244462	RBM12	0.072046434	0.915618176	0.697658473	0.220786883	0.769704907	9.015175615	9.357954881	9.798878489	9.482768454	9.5116449	9.593626268	10.50503024
ENSG00000163214	DHX57	0.506592458	0.765893437	0.262037193	0.882673743	0.768629651	6.009025997	8.981105197	4.523675391	8.115618198	8.14812525	7.86872317	7.913326535
ENSG00000104805	NUCB1	0.105258976	0.865039257	0.662599467	0.202582154	0.767858443	11.87225278	10.80015962	11.38294124	11.50362834	11.5383508	11.93825096	12.20505008
ENSG00000187553	CYP26C1	-1.317823125	NA	2.084595296	NA	0.76677217	2	2	2	2	2.2680111	2.42098416	3.496556178
ENSG00000006756	ARSD	0.344409115	0.832844524	0.421956775	0.789486933	0.76636589	3.327711234	7.928237109	7.225742779	7.454228844	7.36873214	7.260787065	7.68409025
ENSG00000123213	NLN	-0.286283385	0.705469615	1.052568411	0.109470992	0.766285025	7.159102577	8.234834549	6.882775709	7.348537858	7.21862736	8.437839892	8.74825912
ENSG00000213281	NRAS	0.165587793	0.926059063	0.600027601	0.71788604	0.765615395	4.467311984	9.506147172	9.788583153	9.260887574	9.24663965	9.455629465	9.88417883
ENSG00000185049	NELFA	-0.152023692	0.926636636	0.916796689	0.531002733	0.764772997	7.218819781	8.004992147	3.369317972	6.917187326	7.03319408	8.042684843	7.998962529
ENSG00000010810	FYN	0.615393721	0.016642205	0.148936832	0.611272575	0.764330553	9.98400217	9.969011754	10.2528023	10.64829839	10.7226735	10.18913632	10.25190776
ENSG00000122406	RPL5	-0.014555117	0.989000921	0.777628505	0.391227832	0.763073388	10.96815916	10.01303782	12.24925065	11.31401914	11.3818252	11.96059845	12.2987658
ENSG00000063978	RNF4	0.267067696	0.814508396	0.493563436	0.643682096	0.760631132	10.46903009	9.318399851	7.734844356	9.787831283	9.86805151	9.931235194	10.16726922
ENSG00000114126	TFDP2	0.282717026	0.525364769	0.477172796	0.248228307	0.759889822	8.564112515	8.147241016	8.865353883	8.845101141	8.80807321	9.079470158	8.9569744
	PGBD3	5.950976265	0.06938652	-5.191130213	0.165757831	0.759846051	2	2	2	5.987823101	6.14061114	2	2
ENSG00000137185	ZSCAN9	0.217735002	0.838100556	0.542095224	0.576450932	0.759830227	6.89194353	6.464272742	4.523675391	6.573765157	6.37156942	6.956760977	6.595354992
ENSG00000067334	DNTTIP2	0.663881989	0.065602331	0.092408771	0.827527224	0.756290761	9.646202137	9.090820894	9.132850258	9.981765804	9.95963168	9.383101353	9.420790513
ENSG00000204950	LRRC10B	0.729575348	0.803737808	0.026450843	NA	0.756026191	2	3.076665209	2	3.023133209	2.68924543	2.775316226	2.374681325
ENSG00000172336	POP7	-0.108509041	0.931219161	0.864421771	0.431786601	0.75591273	5.330594134	7.099070191	8.325066185	7.292639438	7.23826702	8.083625221	8.344849238
ENSG00000131351	HAUS8	0.659419991	0.714367242	0.096052417	0.960499071	0.755472408	2	5.595927442	3.369317972	4.965943888	5.0259382	4.478802948	4.553838275
ENSG00000277276		5.946400417	0.069869263	-5.19113017	0.165966488	0.755270247	2	2	2	6.006476466	6.11534392	2	2
ENSG00000092847	AGO1	-0.139571875	0.897105982	0.89358936	0.332631208	0.754017485	7.096806373	8.834582582	9.478569125	8.624367384	8.63218877	9.354412203	9.901919938
ENSG00000012983	MAP4K5	0.353933401	0.573001609	0.399742189	0.513742625	0.75367559	8.653385977	9.876711741	9.099922433	9.720931668	9.59016561	9.685065753	9.719953612
ENSG00000113194	FAF2	0.333332237	0.682283472	0.420100791	0.592465838	0.753433028	8.892187472	10.32888058	8.904018013	9.814540128	9.94297217	9.826590762	10.09422624
ENSG00000155366	RHOC	-0.057131599	0.914640194	0.808680632	0.058348141	0.751549033	11.68645261	10.8668003	11.23155829	11.20997529	11.2746349	11.9737357	12.2297432
ENSG00000164683	HEY1	-0.629543348	0.74150751	1.377393305	0.429740549	0.747849957	2	4.442710129	4.523675391	3.709785322	3.45167086	5.880003936	3.753786374
ENSG00000138107	ACTR1A	0.025256241	0.957413691	0.722520133	0.060082802	0.747776374	10.94624409	10.3138733	10.37481416	10.5472158	10.6475497	11.22220981	11.3625769
ENSG00000197279	ZNF165	0.434421618	0.816209881	0.313267318	0.865495099	0.747688936	2	3.686303414	3.369317972	3.462030052	3.50452879	3.336159116	3.474731315
ENSG00000129535	NRL	0.833898886	0.704833394	-0.087372175	0.97020102	0.74652671	2	4.11352228	2	3.615725078	3.74316441	3.12825509	3.100202476
ENSG00000185009	AP3M1	0.030722918	0.917958722	0.715207469	0.00165041	0.745930388	9.435834703	9.298208073	9.330306409	9.398771806	9.37105521	10.06867477	10.0637127
ENSG00000154310	TNIK	1.827411195	0.154010606	-1.083654903	0.415216121	0.743756292	5.532353208	7.274681896	4.058998049	8.001694993	7.94320592	5.707383854	4.4231929
ENSG00000185238	PRMT3	0.119594784	0.940829302	0.62262279	0.675301063	0.742217574	5.330594134	3.686303414	7.398165132	6.155987605	6.44101097	6.748859346	6.824016918
ENSG00000136986	DERL1	0.453663783	0.252999676	0.28779831	0.479315748	0.741462093	10.20680973	9.616264339	9.624627483	10.32001706	10.2669578	10.10747704	10.14908313
ENSG00000183378	OVCH2	-2.312654496	0.63692076	3.053538922	0.477568485	0.740884426	2	2	2	2	2.14022021	3.904814898	3.311948089
ENSG00000083937	CHMP2B	1.080304031	0.610039424	-0.340077117	0.881655142	0.740226915	9.533135543	7.393647515	2	9.358500114	9.27923788	7.910770103	7.917377332
ENSG00000137210	TMEM14B	0.040664041	0.970129688	0.699451551	0.467490985	0.740115592	8.468948322	8.147241016	6.073378139	7.992367495	7.8441483	8.298842011	8.798540026
ENSG00000062725	APPBP2	0.471913563	0.370967351	0.266728981	0.62541695	0.738642544	8.340423424	9.267380431	9.049077186	9.414570905	9.40359931	9.390112523	8.992926984
ENSG00000148925	BTBD10	-0.048702723	0.950599592	0.786600747	0.243287647	0.737898023	8.257427002	7.232742069	8.763901704	8.139053376	8.17690623	9.12745764	8.825734765
ENSG00000148803	FUOM	-0.068902512	0.973095237	0.806287644	0.668910133	0.737385132	2	7.503546079	5.39244166	6.034011955	6.33555364	6.812870125	7.221358049
ENSG00000166435	XRRA1	1.264325494	0.012324317	-0.527008228	0.337799565	0.737317266	7.096806373	7.051611315	6.073378139	8.033874364	8.08227582	6.277913523	6.359671687
ENSG00000187837	HIST1H1C	1.252085452	0.361789525	-0.515315037	0.72430187	0.736770414	7.159102577	4.93638933	8.825624954	8.874592369	8.99861459	6.601598251	7.619668339
ENSG00000134874	DZIP1	0.693639681	0.040664998	0.042969742	0.918154036	0.736609423	8.609439662	9.055168103	8.825624954	9.56056556	9.51003006	9.007589341	8.760149043
ENSG00000176723	ZNF843	-1.451194262	0.548895656	2.187793737	0.329251531	0.736599475	2	4.710564154	2	3.162721474	2.2680111	5.680947153	4.836507146
ENSG00000269743	SLC25A53	-0.623718491	0.70144113	1.360015682	0.350712355	0.736297191	2	3.686303414	4.523675391	3.566290607	3.01487888	4.742549839	4.827924991
ENSG00000121579	NAA50	0.710934866	0.241676601	0.024755818	0.972083517	0.735690684	9.128497779	9.532315021	10.39531398	10.56580756	10.4140861	9.813611959	9.802760311
ENSG00000167380	ZNF226	0.705278586	0.717276749	0.029693219	0.988584182	0.734971805	6.816637977	8.297207175	2	7.860161659	7.86703672	7.382965824	6.996671423
ENSG00000108950	FAM20A	-1.296538697	0.468067566	2.03148493	0.212636396	0.734946233	2	4.93638933	3.369317972	2.947924294	3.28037825	4.749835737	6.245088435
ENSG00000079337	RAPGEF3	-0.675175632	0.706047591	1.409615799	0.38737291	0.734440168	2	5.595927442	3.369317972	3.79808662	4.02226117	5.650128416	5.694529741
ENSG00000136758	YME1L1	0.766372421	0.011999587	-0.03256478	0.933173996	0.733807641	10.9638027	10.54135596	10.50627919	11.44607351	11.4539395	10.63927213	10.66581178
ENSG00000186310	NAP1L3	-0.677658251	0.673764708	1.409891167	0.339618327	0.732232916	6.73718411	6.228547981	2	5.406080789	5.2591458	7.099048677	7.491038307
ENSG00000130479	MAP1S	0.094107393	0.962673456	0.63755838	0.737112257	0.731665773	10.22148166	9.267380431	3.369317972	9.32173423	9.35585278	9.843290512	9.91489412
ENSG00000178568	ERBB4	1.897171808	0.438149869	-1.166234078	0.647618864	0.730937729	2	3.076665209	2	3.462030052	3.55551834	2.34604626	2.225841198
ENSG00000120696	KBTBD7	0.499927771	0.827964997	0.228281734	0.922471927	0.728209505	2	4.710564154	2	3.95996233	3.90918929	3.865109708	3.600986722
ENSG00000141570	CBX8	0.209034854	0.913321769	0.518237765	0.774562861	0.72727262	2	5.457120953	7.098165388	6.139181293	6.1322378	6.639356243	6.182510897
ENSG00000100395	L3MBTL2	0.391586259	0.751627932	0.334853349	0.787590496	0.726439608	7.867441304	8.607278467	5.39244166	8.102673083	8.26379462	7.904255004	8.324402372
ENSG00000153443	UBALD1	0.139357912	0.784445644	0.587065145	0.181113049	0.726423058	7.790810999	8.255926468	8.631919287	8.354580458	8.44880239	8.79381476	8.895672339
ENSG00000136279	DBNL	0.052238189	0.945987763	0.670688704	0.311659052	0.722926893	8.87372952	10.00059544	10.31867969	9.894954101	9.91151326	10.29113189	10.71681868
ENSG00000170873	MTSS1	-0.486431102	0.821446892	1.20828405	0.537860646	0.721852948	7.867441304	4.93638933	2	6.139181293	5.88556448	7.929338774	7.302581437
ENSG00000156876	SASS6	0.69682043	0.535083376	0.024989665	0.984153231	0.721810095	4.467311984	5.457120953	4.058998049	5.565529336	5.30456641	5.243518884	4.292061816
ENSG00000110060	PUS3	0.144755353	0.925454021	0.576270283	0.685289111	0.721025636	4.467311984	7.503546079	4.874587708	6.394182228	6.48112613	7.0614806	6.607941737
ENSG00000126246	IGFLR1	1.877711305	0.412886226	-1.156777166	0.627060885	0.720934139	2	3.686303414	2	3.79808662	4.22485516	2.267001569	2.632958241
ENSG00000241878	PISD	0.530568303	0.682219847	0.190109916	0.889682343	0.720678219	5.330594134	8.522788974	8.845626171	8.703665429	8.70032852	8.421393978	8.30675314
ENSG00000089639	GMIP	0.763540462	0.521751449	-0.043423958	0.97326848	0.720116503	5.866944937	5.72254323	8.206335842	7.762033056	7.8720741	6.932953913	7.12130792
ENSG00000265491	RNF115	-0.052792507	0.961244994	0.772648205	0.422682091	0.719855698	9.031919741	7.099070191	9.61300511	8.817637515	8.88981993	9.584231726	9.7588503
ENSG00000183856	IQGAP3	-0.005465152	0.99798804	0.722433311	0.778850367	0.716968159	2	5.946638857	2	4.237680128	4.83276841	4.301748724	5.740981724
ENSG00000156650	KAT6B	0.372152156	0.704032536	0.344744086	0.722618004	0.716896242	6.73718411	8.671479029	9.049077186	8.795015659	8.82894823	8.782313877	8.787516955
ENSG00000101181	MTG2	0.081296255	0.951240273	0.635405798	0.603099089	0.716702053	8.653385977	6.78762695	5.59507254	7.433701013	7.79460185	8.072046038	8.262059922
ENSG00000110713	NUP98	0.252390497	0.675311646	0.463754551	0.408562547	0.716145047	9.371511179	9.726146444	10.48730704	10.20056091	10.1772163	10.41631794	10.38273198
ENSG00000037637	FBXO42	-0.081679049	0.966682851	0.796978917	0.660328198	0.715299868	4.467311984	6.602424674	9.888372618	8.441112993	8.34030387	9.172206498	9.34725177
ENSG00000109689	STIM2	1.221595148	0.064886519	-0.509159716	0.475128388	0.712435431	6.366709671	7.503546079	7.285536013	8.270263574	8.39732094	7.000176314	6.172377306
ENSG00000087338	GMCL1	0.234219105	0.878292377	0.476682733	0.7369322	0.710901837	4.00722531	7.002537973	8.076950877	7.402350992	7.26532099	7.626042826	7.51663051
ENSG00000129450	SIGLEC9	-2.3053361	0.655168404	3.015558758	0.511065006	0.710222658	2	2	2	2.141737246	2	4.177580368	2.671842496

ENSG00000159140	SON	0.209791795	0.632755739	0.498061208	0.211429442	0.707853002	12.19201182	11.60869109	11.55072361	12.02642465	12.0201065	12.24198271	12.3774204
ENSG00000142669	SH3BGRL3	0.213325757	0.767351465	0.494310673	0.457548655	0.70763643	12.15618594	11.54371287	11.28013451	11.89463174	11.9469477	11.52908949	12.65872837
ENSG00000176340	COX8A	0.065830708	0.959731105	0.640122079	0.591055458	0.705952787	10.71696191	8.505280662	7.896971941	9.596407142	9.67763759	10.04998226	10.35313191
ENSG00000163482	STK36	0.384555724	0.806161404	0.32109905	0.836918007	0.705654774	4.00722531	8.451443573	7.285536013	7.883054517	7.76782485	7.551912644	7.949380652
ENSG00000113211	PCDHB6	0.765917475	0.729728652	-0.060708141	0.979401422	0.705209334	2	4.11352228	2	3.566290607	3.69849104	3.12825509	3.128433649
ENSG00000126562	WNK4	-0.046117571	0.98350331	0.749927954	0.71854685	0.703810383	2	5.946638857	8.536706852	7.057964187	7.21466714	8.028526139	7.796124663
ENSG00000126524	SBDS	0.237945637	0.759076344	0.465716875	0.52135424	0.703662511	10.27168932	8.702538795	10.08627303	10.05135008	10.0869461	10.28267996	10.30968141
ENSG00000084234	APLP2	0.138867788	0.888548519	0.564754808	0.519747981	0.703622596	12.76283692	12.46223249	10.67226762	12.35897559	12.3412176	12.61180942	12.92290535
ENSG00000083642	PDS5B	0.961684702	0.116820694	-0.258548017	0.704939784	0.703136686	8.16936288	8.747909548	7.342949392	9.162439556	9.15351711	8.016494289	7.882577494
ENSG00000186448	ZNF197	0.762802852	0.45354552	-0.060158447	0.958081539	0.702644405	5.866944937	8.004992147	8.460932916	8.52598359	8.57464274	7.918064594	7.535201607
ENSG00000089351	GRAMD1A	0.065293134	0.957587914	0.635887649	0.567222525	0.701180783	10.87842776	8.522788974	8.65477198	9.793884031	9.90906469	10.66105227	10.13327938
ENSG00000139154	AEBP2	0.19165712	0.898282189	0.508418955	0.714850336	0.700076076	5.330594134	6.728495673	8.884815469	7.880528731	7.86451142	8.331380596	8.020777347
ENSG00000105053	VRK3	0.84807364	0.085388044	-0.148377441	0.794186359	0.699696199	7.53297911	6.666836837	7.451345288	8.124184191	8.0358969	7.14536429	7.063994051
ENSG00000196136	SERPINA3	2.900846406	0.361569449	-2.201624459	NA	0.699221947	2	2	2	3.754611435	3.28037825	2.094598369	2.059860178
ENSG00000165410	CFL2	-0.526830228	0.237527811	1.225690773	0.002219876	0.698860545	10.00110988	9.806969599	9.196533591	9.269600773	9.09439916	10.8677203	10.99006746
ENSG00000137507	LRRC32	-1.801641984	0.000721583	2.496108099	1.02E-06	0.694466116	7.867441304	8.903033433	9.149036566	6.922108067	7.0150921	11.25322188	11.19155596
ENSG00000119953	SMNDC1	0.407490713	0.500733183	0.28692416	0.639193832	0.694414873	8.285626337	7.467835385	7.163363831	8.184811706	8.04707549	8.045647998	7.950369405
ENSG00000138095	LRPPRC	0.689403621	0.373352069	0.004536763	0.996064263	0.693940384	8.075571721	9.903691196	9.132850258	9.903687576	9.91517834	9.208355151	9.247812734
ENSG00000170365	SMAD1	1.163739471	0.44522898	-0.471075957	0.770200762	0.692663514	3.327711234	7.761270542	5.772716771	7.731524332	7.66128317	6.146071859	6.092227206
ENSG00000198858	R3HDM4	-0.092017401	0.908769676	0.782991588	0.241623158	0.690974187	9.371511179	9.13702746	7.896971941	8.830780654	8.85207713	9.490026756	9.902175458
ENSG00000153130	SCOC	0.431171138	0.414030475	0.259400849	0.632223509	0.690571987	9.112843761	8.777382925	8.266922072	9.269600773	9.10299435	9.244909847	8.748827534
ENSG00000105723	GSK3A	0.346300297	0.753326748	0.342935954	0.753191906	0.689236251	7.384433967	8.981105197	10.10298738	9.519758723	9.57865651	9.589063591	9.501932203
ENSG00000185630	PBX1	0.291979726	0.76909522	0.396506292	0.681431908	0.688486017	8.716893051	8.655694736	6.534109959	8.575233246	8.51952909	8.477329966	8.806206367
ENSG00000196581	AJAP1	-2.312721121	0.663582024	3.000648273	0.525943482	0.687927152	2	2	2	2	2.14022021	4.230621763	2.466008509
ENSG00000168763	CNNM3	0.191214924	0.798284713	0.495398142	0.464069117	0.686613065	6.468621203	7.35507212	6.629488387	7.066878356	7.0774796	7.697491716	6.940057611
ENSG00000175727	MLXIP	-0.106435288	0.932860886	0.792855718	0.478845029	0.68642043	7.384433967	10.35355093	10.26027233	9.707456306	9.71175558	10.80232183	10.37796311
ENSG00000105088	OLFM2	-1.421783508	0.032828609	2.106590658	0.000537851	0.68480715	5.095959458	6.04685525	6.073378139	4.623314311	4.4836996	8.086505553	7.54177663
ENSG00000164347	GFM2	0.822242668	0.114309143	-0.138557445	0.817289897	0.683685223	7.33131396	7.790474954	8.408111533	8.674801527	8.76132797	7.832294085	7.706501872
ENSG00000158545	ZC3H18	0.185246201	0.86184497	0.495939515	0.609008368	0.681185716	9.497412313	7.790474954	7.451345288	8.731963162	8.71722214	9.172206498	8.878113577
ENSG00000164830	OXR1	-0.058745524	0.919646442	0.739480662	0.120443179	0.680735138	8.138771685	7.638155693	8.296287027	8.022463952	7.94559728	9.019318595	8.475596448
ENSG00000157869	RAB28	0.331510088	0.710348924	0.348229093	0.690278517	0.679739181	7.384433967	6.602424674	5.39244166	6.931899482	7.05550676	7.107581047	6.907844475
ENSG00000090621	PABPC4	-0.134142475	0.863478741	0.813553039	0.214752155	0.679410564	9.097018025	10.20420974	8.845626171	9.381011913	9.3808074	10.21453767	10.42655664
ENSG00000169105	CHST14	-0.213981495	0.857034032	0.89204791	0.389087222	0.678066415	9.219014269	6.535001748	7.734844356	7.966401501	8.06478301	9.089196199	9.131450704
ENSG00000023318	ERP44	1.082973146	0.140291258	-0.406234004	0.609008368	0.676739142	10.27168932	9.377333006	8.486635645	10.64606954	10.6257199	9.041010784	9.257431793
ENSG00000140688	C16orf58	0.864123103	0.030796809	-0.187500877	0.683460163	0.676622227	8.075571721	8.717821467	8.175061096	9.210493388	9.22416269	8.256629373	8.090601559
ENSG00000196074	SYCP2	0.925280289	0.53071634	-0.248706415	0.876525531	0.676573875	4.00722531	3.076665209	4.058998049	4.299492648	4.58534546	3.943456545	3.071407821
ENSG00000169814	BTD	-0.089574366	0.968773308	0.765027889	0.721493405	0.675453522	9.358295023	7.051611315	2	7.997038782	7.91663606	8.59173005	8.984746522
ENSG00000135250	SRPK2	0.236762685	0.745616581	0.437842352	0.523326453	0.674605037	8.042902253	8.607278467	9.503962296	9.089953145	9.06500642	9.256145816	9.298000491
ENSG00000080007	DDX43	-3.753029217	0.499533315	4.427034123	0.376931525	0.674004906	2	2	2	2	2	4.099885429	5.115701921
ENSG00000128000	ZNF780B	1.466298839	0.185150547	-0.79240235	0.492708893	0.673896488	4.00722531	7.145017454	6.882775709	7.987681034	7.92393105	5.951260634	5.59182244
ENSG00000091651	ORC6	0.722352048	0.358652871	-0.048806546	0.956860836	0.673545502	4.815609471	5.131603625	4.874587708	5.639097847	5.5755357	5.068107405	4.74829783
ENSG00000077713	SLC25A43	0.30946912	0.834827076	0.36331945	0.800774229	0.67278857	9.344956677	6.78762695	5.930865648	8.401141138	8.40081078	8.39955642	8.506848296
ENSG00000100372	SLC25A17	0.390579384	0.690529475	0.279042295	0.778789835	0.669621679	6.563806279	7.503546079	8.763901704	8.270263574	8.26762243	7.98365249	8.31522102
ENSG00000133619	KRBA1	-0.139560346	0.929222345	0.808649548	0.56354884	0.669089202	6.96351253	7.60566235	3.369317972	6.598566398	6.71216164	7.544599974	7.608437243
ENSG00000185163	DDX51	-0.222412329	0.744473447	0.891354026	0.129961155	0.668941697	6.257048221	7.274681896	6.43197674	6.664650034	6.39275508	7.52137298	7.702986259
ENSG00000130176	CNN1	-2.587342088	1.24E-07	3.255981994	6.95E-12	0.668639906	9.33149386	8.169643011	9.116480286	6.401247806	6.43421531	12.18290317	12.20354771
ENSG00000108946	PRKAR1A	0.473396567	0.025286919	0.195233305	0.393866241	0.668629872	11.25159303	11.26034006	11.31641863	11.76026481	11.7374476	11.37858912	11.55807875
ENSG00000111907	TPD52L1	-2.80785372	0.056626744	3.476135259	0.011014824	0.668281539	2	6.140560021	4.058998049	2.947924294	3.01487888	8.504505158	7.980689421
ENSG00000171928	TVP23B	1.26126292	0.008469405	-0.595567931	0.247751753	0.665694989	8.586954094	7.874700259	8.784770914	9.749588203	9.6675326	8.062569037	7.647978997
ENSG00000172340	SUCLG2	0.621465982	0.098154133	0.043870756	0.923063983	0.665336738	7.82963488	8.053983481	7.934808822	8.600009476	8.51792306	7.804574291	8.153772149
ENSG00000196182	STK40	-0.108538451	0.955340357	0.773123121	0.668910133	0.66458467	5.330594134	9.943237334	5.772716771	8.36726458	8.40776525	9.321478134	9.198731239
ENSG00000071205	ARHGAP10	-0.378429518	0.74176673	1.040811095	0.315894652	0.662381577	9.174465797	6.844429755	7.029880454	7.739909082	7.70992146	9.136171672	9.117873759
ENSG00000184897	H1FX	0.061003135	0.925672996	0.600957565	0.277409224	0.6619607	9.048471759	10.14890644	10.01740919	9.841412998	9.91151326	10.28769449	10.53186455
ENSG00000204138	PHACTR4	0.439862739	0.346404872	0.221978032	0.649746012	0.66184077	8.674867122	9.067150531	9.491321578	9.517321994	9.58557298	9.189724501	9.467467793
ENSG00000173272	MZT2A	0.635991565	0.239055913	0.023355279	0.97050839	0.659346843	8.674867122	8.077869079	7.646393253	8.865680425	8.7802082	8.376795097	8.038552764
ENSG00000114812	VIPR1	-3.753032715	0.495884604	4.411319177	0.373787431	0.658286463	2	2	2	2	2	4.572179576	4.784231301
ENSG00000126003	PLAGL2	0.173951396	0.939485429	0.484127414	0.827299212	0.65807881	4.467311984	9.298208073	3.369317972	7.920420067	8.00183183	8.290747499	8.243626305
ENSG00000135407	AVIL	1.147763814	0.149004691	-0.490124537	0.564099325	0.657639277	5.330594134	5.72254323	4.058998049	6.221320894	6.35727082	4.799832052	4.80186789
ENSG00000110057	UNC93B1	1.216890579	0.03681088	-0.559451855	0.369090445	0.657438723	8.493334308	7.538394143	8.865353883	9.615519136	9.58019637	7.716209895	7.953331607
ENSG00000117477	CCDC181	-0.687342928	0.818202168	1.3443249	0.615729155	0.656981971	2	3.076665209	2	2.3892346	2.38539787	3.483289517	2.782576693
ENSG00000147654	EBAG9	0.671124345	0.29397165	-0.01471447	0.984191003	0.656409875	5.532353208	6.602424674	6.629488387	6.907295206	7.06873058	6.466611899	6.182510897
ENSG00000084070	SMAP2	0.574032486	0.058669633	0.080469439	0.820851865	0.654501925	8.609439662	8.623598428	8.561105505	9.193296408	9.14313645	8.549559709	8.797990868
ENSG00000078699	CBFA2T2	0.717445664	0.509306243	-0.063398539	0.958174026	0.654047125	9.262228249	8.791896851	6.43197674	9.234427714	9.33867006	8.708512253	8.287321088
ENSG00000137845	ADAM10	0.380986816	0.580787928	0.271718197	0.696976163	0.652705013	9.371511179	10.53705456	10.77492406	10.69863405	10.7502937	10.47451025	10.7443005
ENSG00000169490	TM2D2	0.459646722	0.729210347	0.192338625	0.890313426	0.651985347	10.07567912	8.297207175	6.629488387	9.39612172	9.43287094	8.904453984	9.358096047
ENSG00000274286	ADRA2B	-1.303057274	NA	1.955008429	NA	0.651951155	2	2	2	2.270786764	2	3.374372158	2.466008509
ENSG00000166557	TMED3	0.098887683	0.886208951	0.551950234	0.351453916	0.650837918	11.32815351	10.56689847	10.13584611	10.84605279	10.8765996	11.47344391	11.13386651
ENSG00000198816	ZNF358	-0.167727553	0.912094181	0.8181049	0.542439157	0.650377348	5.532353208	9.648522413	8.845626171	8.568972129	8.64106878	9.63011094	9.536251143
ENSG00000142959	BEST4	0.368676804	0.881476754	0.281032589	0.90832745	0.649709394	2	4.710564154	2	3.709785322	3.94787299	3.336159116	4.105828626
ENSG00000100225	FBXO7	0.192228706	0.491293064	0.457157417	0.068317498	0.649386123	9.98400217	9.936721131	9.735974718	10.06086293	10.1036386	10.29767155	10.39367729
ENSG00000006638	TBXA2R	-0.672448741	0.612543393	1.321420778	0.278961643	0.648972037	7.485143553	4.11352228	5.930865648	5.686138681	5.86549842	7.765568476	7.613439639
ENSG00000140025	EFCAB11	0.720938107	0.328225936	-0.072180271	0.932122597	0.648757837	5.532353208	5.595927442	4.523675391	5.949776857	6.00960265	5.169385838	5.331426166
ENSG00000111711	GOLT1B	0.734388726	0.357059066	-0.086199066	0.92531104	0.64818966	7.867441304	9.672251278	8.960138144	9.793884031	9.69337522	9.18804929	8.613062226
ENSG00000100354	TNRC6B	0.468445227	0.751472272	0.179582185	0.908291211	0.648027413	5.532353208	9.159587375	9.897982195	9.411074916	9.58173459	9.159610958	9.262217379
ENSG00000240682	ISY1	0.831790376	0.046962517	-0.18386986	0.699954446	0.647920516	7.53297911	7.60566235	7.285536013	8.184811706	8.41468635	7.113241388	7.478755384
ENSG00000105011	ASF1B	0.201402563	0.941409766	0.445454879	0.867353663	0.646857442	2	5.303523747	2	4.299492648	4.05806467	3.215043717	5.013446246
ENSG00000180616	SSTR2	0.439292997	0.883067069	0.205720839	0.944462782	0.645013836	2	3.686303414	4.523675391	4.268917409	3.7864959	4.652122235	2.059860178
ENSG00000145332	KLHL8	0.466896889	0.739729032	0.177570652	0.904611962	0.644467541	4.00722531	7.73146271	7.451345288	7.517371202	7.5462416	7.57363085	6.830475749
ENSG00000115109	EPB41L5	0.228223992	0.895400127	0.416046231	0.800593168	0.644270223	7.904282243	8.376385542	3.369317972	7.786523606	7.86955761	7.895248429	8.122532579
ENSG00000149218	ENDOD1	-0.590608988	0.698378828	1.234270431	0.37875047	0.643661443	6.009025997	9.443177293	10.67785662	9.108419034	9.00091592	11.02039807	10.7082226
ENSG00000105953	OGDH	-0.333603187	0.757468139	0.976930842	0.314242081	0.643327655	9.015175615	9.470501606	11.23913884	9.870342608	9.95429779	10.85172587	11.51294866
ENSG00000128311	TST	-0.077009948	0.970008162	0.71953003	0.706265396	0.642520083	8.367060731	7.189546432	2	7.169761359	7.32163988	8.042684843	8.013226939
ENSG00000078061	ARAF	0.185215922	0.878396152	0.457245194	0.681460704	0.642461117	10.1311248	8.433041974	7.502634626	9.288777073	9.28113268	9.351421858	9.734089014
ENSG00000047249	ATP6V1H	0.731288694	0.410778027	-0.089555611	0.92948232	0.641733082	9.590776211	8.234834549	7.552162999	9.451627281	9.42603674	8.52322774	8.718968947
ENSG00000143751	SDE2	0.385301785	0.770265142	0.256361497	0.848992364	0.641663282	6.138360429	7.638155693	9.465702942	8.722121967	8.72837565	8.676372393	8.514222325
ENSG00000118855	MFSD1	1.338847245	0.090297135	-0.698589273	0.399585878	0.640257972	9.473095744	9.497318007	7.600047325	10.35546265	10.4786707	8.247650629	8.526871175
ENSG00000167766	ZNF83	0.250508946	0.822912318	0.388757271	0.715258735	0.639266217	7.384433967	8.053983481	9.714382178	8.948803189	8.99976571	9.366608223	8.80183059
ENSG00000137497	NUMA1	0.764556484	0.074018972	-0.126678652	0.798498793	0.637877832	9.957954117	10.73780697	10.13584611	11.05899696	11.1061	10.23447665	10.15187942
ENSG00000144118	RALB	0.65815223	0.419141353	-0.022869885	0.980129329	0.635282345	7.790810999	9.405920729	9.658943166	9.756492097	9.85856545	8.987312532	9.262217379
ENSG00000120370	GORAB	0.737608778	0.618090751	-0.103277143	0.948615522	0.634331635	3.327711234	7.701025996	7.163363831	7.739909082	7.52720828	6.898949899	6.735016458
ENSG00000177479	ARIH2	0.42940172	0.643876123	0.204891525	0.833193204	0.634293245	7.904282243	10.00683004	8.763901704	9.545497589	9.61366407	9.366904403	9.346500876
ENSG00000171067	C11orf24	-0.465032786	0.412187433	1.098216319	0.031149835	0.633183533	9.751047882	9.308339287	10.38167985	9.397888985	9.43372295	10.80297867	11.12612127
ENSG00000110108	TMEM109	-0.369019317	0.456750419	1.001937238	0.022450111	0.63291792	10.21416435	9.656475478	9.385998894	9.422406043	9.43116542	10.61939729	10.9472486
ENSG00000166716	ZNF592	-0.159860838	0.814508396	0.792373589	0.172021653	0.632512752	9.90440318	8.747909548	8.941672936	9.04103174	9.21427981	10.07358251	10.08142138
ENSG00000101346	POFUT1	-0.093734502	0.934842791	0.725920886	0.472907496	0.632186384	7.667647223	10.34373336	9.725218844	9.484432763	9.55770587	10.15524287	10.49980559
ENSG00000173281	PPP1R3B	-0.274112122	0.832844524	0.906257737	0.432001677	0.632145615	5.532353208	7.002537973	8.608698767	7.292639438	7.27676203	8.620333861	8.243626305
ENSG00000139946	PELI2	0.408936114	0.820262885	0.223102144	0.902593423	0.632038259	4.00722531	7.669933291	4.058998049	6.825617242	6.58294721	6.583362013	6.476841503
ENSG00000067221	STOML1	-0.333755093	0.864010109	0.965409337	0.583360046	0.631654245	7.096806373	7.847167909	2	6.509830055	6.75096147	7.923712697	7.875307424
ENSG00000182700	IGIP	-0.207628746	0.935007649	0.838608868	0.72430187	0.630980123	2	4.442710129	8.904018013	7.29643414	7.07311172	8.367352231	8.044120856
ENSG00000215883	CYB5RL	-0.586558378	0.829400292	1.217266624	0.62887288	0.630708246	2	2	6.43197674	4.299492648	4.60967607	6.231826305	5.88031069
ENSG00000110719	TCIRG1	0.850235308	0.47919324	-0.219575757	0.866382985	0.630659551	8.468948322	8.213429689	10.93284392	10.55277887	10.6618002	9.549165021	9.535921843
ENSG00000159873	CCDC117	0.30079432	0.834110208	0.327368933	0.815505549	0.628163253	5.532353208	9.125613817	9.287079172	8.946390023	9.00894176	9.197405512	8.781418172
ENSG00000145990	GFOD1	-0.137542462	0.946595613	0.765410802	0.690305992	0.62786834	2	6.140560021	3.369317972	4.574143677	4.89348248	5.614651724	5.464725981
ENSG00000163393	SLC22A15	1.716002374	0.261861084	-1.088309073	0.488633039	0.627693301	7.218819781	4.93638933	4.058998049	7.617841552	7.7377845	5.73334482	3.620995423
ENSG00000093217	XYLB	0.326800842	0.898630945	0.30068208	0.906004994	0.627482922	2	5.72254323	2	4.523237872	4.76938658	4.340871764	4.870334174
ENSG00000185088	RPS27L	0.142703709	0.866763421	0.484684871	0.523602241	0.62738858	10.56418906	9.055168103	9.272377397	9.899327447	9.97024066	10.38434358	10.15873961
ENSG00000159322	ADPGK	1.101298396	0.021693999	-0.474522217	0.360306411	0.626776179	8.493334308	9.532315021	8.99637432	10.17605323	10.1644413	8.616851507	8.591675571

ENSG00000072756	TRNT1	0.554107726	0.638803946	0.072107368	0.955191984	0.626215094	4.467311984	7.431218269	7.098165388	7.514115926	7.12888795	7.045287119	6.662054875
ENSG00000147416	ATP6V1B2	0.342690976	0.620548015	0.283011522	0.683637326	0.625702498	10.86913986	9.514922632	10.01740919	10.54562241	10.621987	10.34293862	10.68628308
ENSG00000102158	MAGT1	0.405366028	0.794639251	0.220282841	0.890244223	0.625648869	6.009025997	9.903691196	10.60345421	10.19498122	10.1052438	9.745312661	10.15809785
ENSG00000089177	KIF16B	0.121472673	0.946330866	0.504131598	0.770708651	0.62560427	3.327711234	6.464272742	8.486635645	7.355829978	7.37584259	7.629009275	7.843728236
ENSG00000168724	DNAJC21	0.054615641	0.965988399	0.570827326	0.625700973	0.625442967	6.257048221	9.415325457	8.904018013	8.828161587	8.65866647	9.38982107	9.113025255
ENSG00000184058	TBX1	0.426355313	0.904107254	0.198309074	NA	0.624664387	2	3.076665209	2	2.600425781	2.86123014	3.035909414	2.117335275
ENSG00000141644	MBD1	0.17860786	0.725609862	0.445869422	0.335637906	0.624477282	9.667789716	8.791896851	9.372276026	9.476928206	9.5116449	9.793481551	9.726317257
ENSG00000119707	RBM25	0.758418743	0.082371227	-0.134187154	0.790197999	0.624231589	9.533135543	9.00621767	9.849281147	10.25178588	10.2564019	9.285583053	9.441331959
ENSG00000124574	ABCC10	0.538200022	0.794520103	0.084581124	0.968806022	0.622781146	8.855032352	8.169643011	2	8.483340231	8.53072154	7.959503304	8.152913455
ENSG00000162441	LZIC	0.383633885	0.540633385	0.238930513	0.710603737	0.622564398	8.075571721	6.899080544	7.502634626	7.968781453	7.90439517	7.935741825	7.638194697
ENSG00000116337	AMPD2	0.287346392	0.783350857	0.334864048	0.741073451	0.62221044	10.27872131	9.497318007	7.777116083	9.829676744	9.75806852	9.783975576	9.897056431
	MB21D1	5.812770451	0.089797897	-5.191128106	0.178878036	0.621642346	2	2	2	5.818703821	6.04571775	2	2
ENSG00000143110	C1orf162	-0.991610824	0.747288938	1.612814046	0.570849073	0.621203222	2	3.686303414	2	2.141737246	2.77779907	4.369533167	2.851942824
ENSG00000060138	YBX3	-0.175481614	0.556828814	0.795601632	0.00161096	0.620120019	10.76282024	10.47993704	10.75391088	10.51622947	10.4720505	11.37888285	11.54332823
ENSG00000101199	ARFGAP1	0.080262096	0.930786574	0.539802381	0.509619901	0.620064478	9.174465797	9.170736405	10.74328839	9.901820563	10.0524158	10.50025386	10.37096473
ENSG00000137714	FDX1	0.566631682	0.606376439	0.053311991	0.965290088	0.619943672	5.095959458	7.701025996	7.818184403	7.836899669	7.84926609	7.328045423	7.352801367
ENSG00000036054	TBC1D23	0.946620385	0.517222971	-0.32700503	0.833980846	0.619615355	5.330594134	9.876711741	8.922968322	9.921611889	9.82778401	8.888863837	8.265241863
ENSG00000100991	TRPC4AP	0.477177862	0.131912603	0.142027702	0.684760578	0.619205565	10.25752168	9.785372135	10.10298738	10.52838133	10.5400919	10.16293862	10.23638632
ENSG00000111727	HCFC2	0.051103744	0.943613626	0.567683783	0.365809256	0.618787527	7.975256505	7.35507212	7.342949392	7.762033056	7.49817903	8.522696167	7.628343587
ENSG00000266028	SRGAP2	0.813129394	0.185725588	-0.194938161	0.776974137	0.618191233	8.257427002	9.540933215	9.452720984	9.965769838	10.0345549	9.097429939	8.89156005
ENSG00000197933	ZNF823	0.448309168	0.680957539	0.168672028	0.883235347	0.616981195	4.815609471	5.457120953	3.369317972	5.110902178	5.3193943	5.073924441	4.861951484
ENSG00000142002	DPP9	0.324779296	0.712692238	0.291650235	0.738292369	0.616429531	9.913467515	7.979858146	9.647595056	9.731481912	9.69692814	9.611983935	9.747518555
ENSG00000127804	METTL16	-0.375412501	0.845522872	0.990627978	0.572896533	0.615215477	3.327711234	7.35507212	9.344432377	7.691737974	7.76782485	9.067857443	9.091227191
ENSG00000111206	FOXM1	-0.035906969	0.982223298	0.650248729	0.653855654	0.61434176	4.00722531	7.002537973	5.59507254	5.94010643	6.03677338	5.399896186	7.297928101
ENSG00000116273	PHF13	-0.190946053	0.876278112	0.805142932	0.452613409	0.614196879	8.009475818	7.189546432	9.819251342	8.609191952	8.53866332	9.456464678	9.661984931
ENSG00000177200	CHD9	0.493037439	0.531143362	0.119799193	0.889682343	0.612836632	9.730679447	10.16572116	8.434763955	10.08464853	10.1222557	9.942606032	9.484973508
ENSG00000106948	AKNA	0.41894905	0.812190473	0.191667889	0.915522323	0.61061694	4.815609471	4.442710129	8.236946975	7.214570819	7.32531744	7.398115274	6.590289384
ENSG00000138193	PLCE1	-2.822940798	0.076976946	3.432529709	0.024210868	0.609588911	2	6.04685525	7.163363831	4.205751501	3.08597292	9.229031012	9.739818999
ENSG00000165527	ARF6	-0.303321537	0.56715642	0.911886078	0.051766586	0.608564541	10.02639697	10.55418374	10.89974571	10.22266632	10.2457682	11.18498388	11.66824733
ENSG00000005302	MSL3	0.817945855	0.166721726	-0.209403614	0.749808479	0.608542241	7.53297911	7.503546079	8.561105505	8.699372486	8.80807321	7.626042826	7.847978946
ENSG00000158195	WASF2	-0.411521236	0.701800878	1.019934752	0.294703553	0.608413517	8.631580145	11.13786633	11.1494608	10.25958244	10.2802818	11.67532131	11.72127105
ENSG00000067248	DHX29	0.807474287	0.586183061	-0.199260153	0.90169025	0.608214134	9.521325812	8.806266215	4.874587708	9.483600848	9.43627594	8.389678938	8.53018147
ENSG00000143862	ARL8A	0.366700699	0.606992001	0.2413599	0.738195992	0.608060599	9.966689162	9.443177293	8.434763955	9.81982307	9.73267673	9.618217491	9.685333873
ENSG00000103449	SALL1	-0.865482026	0.601928679	1.473122051	0.340932973	0.607640026	2	6.140560021	6.80318718	5.501219927	4.72553021	7.525623979	7.191563076
ENSG00000129422	MTUS1	-1.747217422	0.300402677	2.353336895	0.133579211	0.606119473	2	5.838937808	3.369317972	3.406930997	3.28037825	7.02591204	6.476841503
ENSG00000164251	F2RL1	1.173668482	0.298019815	-0.568401734	0.631319446	0.605266748	4.815609471	6.464272742	7.858115942	8.027038952	7.97164382	6.109723842	6.485052582
ENSG00000131591	C1orf159	0.21922025	0.910380971	0.384813594	0.836009948	0.604033844	7.579279349	6.389896633	2	6.788375664	6.71216164	6.978631953	6.839042789
ENSG00000120805	ARL1	0.189446954	0.474064658	0.41428372	0.083172408	0.603730674	9.87686338	9.969011754	10.05225195	10.15582956	10.1567216	10.44437493	10.31333667
ENSG00000168621	GDNF	1.837270373	0.361789525	-1.234699506	0.547274868	0.602570867	8.107517749	3.076665209	5.156626401	8.554784469	8.50501001	6.412547789	3.287118176
ENSG00000152443	ZNF776	0.530347231	0.751472272	0.071069699	0.968477418	0.60141693	3.327711234	8.077869079	8.486635645	8.22518713	8.28849576	7.731011524	7.875307424
ENSG00000126653	NSRP1	0.515759029	0.190607653	0.083378073	0.853024401	0.599137102	9.031919741	8.376385542	8.631919287	9.231457548	9.19128891	8.774744193	8.790280628
ENSG00000197816	CCDC180	0.856490318	0.713785889	-0.257825018	0.917242457	0.5986653	4.815609471	2	2	4.17310023	4.1930041	3.584558147	3.183290849
ENSG00000184867	ARMCX2	0.090214716	0.968342042	0.507342978	0.817314365	0.597557695	9.304185368	9.74864196	2	9.104095335	8.99976571	9.577081455	9.34837738
ENSG00000138964	PARVG	-2.305380043	0.65272748	2.901291844	NA	0.595911802	2	2	2	2.141737246	2	3.968654369	2.918126211
ENSG00000116213	WRAP73	0.035702978	0.98413673	0.559810573	0.744740559	0.595531551	6.89194353	7.145017454	2	6.604700657	6.39275508	7.137053113	6.864442837
ENSG00000166689	PLEKHA7	1.134436764	0.64718241	-0.539503272	0.835180221	0.594933492	2	4.710564154	2	4.804776789	3.86943969	2.592885947	3.67940853
ENSG00000108924	HLF	-3.753025965	0.504957686	4.347911412	0.392715269	0.594885447	2	2	2	2	2	4.199031499	4.959465495
ENSG00000177842	ZNF620	-0.77334746	0.51181261	1.367432796	0.199468909	0.594085335	3.327711234	4.710564154	5.156626401	3.754611435	4.16043394	6.146071859	5.331426166
ENSG00000170323	FABP4	-3.274019969	0.353103921	3.864473174	0.176769831	0.590453205	3.327711234	2	2	2	2.14022021	5.86330637	2.918126211
ENSG00000106245	BUD31	1.279279043	0.055604743	-0.688930562	0.327012123	0.590348481	8.75772933	7.790474954	7.163363831	9.324524407	9.31482024	7.347386207	7.407104868
ENSG00000120948	TARDBP	0.737973686	0.453160638	-0.149007239	0.891004263	0.588966447	9.544849385	9.792607274	7.342949392	9.991161874	9.93276281	8.972597003	9.180307928
ENSG00000144579	CTDSP1	0.054622793	0.920437233	0.531945668	0.247703343	0.586568462	9.262228249	10.02537381	10.05225195	9.878593178	9.87935305	10.22388773	10.47442608
ENSG00000164609	SLU7	0.967330404	0.47627622	-0.381197367	0.791764439	0.586133037	9.613202746	9.45234302	5.59507254	9.959201773	9.95963168	8.541185654	8.694392964
ENSG00000152082	MZT2B	0.389739051	0.344601719	0.196109417	0.64966917	0.585848467	8.855032352	8.955547846	9.503962296	9.535096495	9.50030288	9.323005438	9.327600363
ENSG00000107679	PLEKHA1	-0.88111134	0.066039055	1.466108281	0.000983739	0.58499694	8.928409675	7.954278496	8.585098385	7.717440541	7.61696151	10.02598953	9.957809357
ENSG00000130303	BST2	1.87506185	0.031250396	-1.290836001	0.149212315	0.584225849	7.904282243	8.903033433	10.17589429	11.14332431	11.165776	8.306273771	7.633277551
ENSG00000198039	ZNF273	0.511518232	0.667273997	0.071813148	0.955158571	0.58333138	4.00722531	4.710564154	3.369317972	4.76150579	4.40247105	4.281782022	4.188602707
ENSG00000123739	PLA2G12A	-0.881958413	0.405292907	1.464475475	0.136351095	0.582517062	5.330594134	8.077869079	7.934808822	6.658765962	6.70088158	9.03282106	8.926146458
ENSG00000126247	CAPNS1	0.232691374	0.76542366	0.349232324	0.640632886	0.581923698	10.06758145	11.35379164	11.53848775	11.31939084	11.3798372	11.143491	11.72993391
ENSG00000133243	BTBD2	-0.12748489	0.941619909	0.708836412	0.659176557	0.581351522	5.095959458	10.36815316	9.809100877	9.418929011	9.43457445	10.34052773	10.1734218
ENSG00000099992	TBC1D10A	0.254166249	0.891376437	0.327035955	0.855655091	0.581202204	9.081016759	6.464272742	4.058998049	7.855024645	8.13141746	7.821097076	8.284971554
ENSG00000103064	SLC7A6	0.333915797	0.573636123	0.247257103	0.681162733	0.5811729	8.963744667	8.671479029	7.934808822	8.899755849	8.9350493	8.558404203	9.061336073
ENSG00000204335	SP5	-1.310474982	NA	1.890539153	NA	0.58006417	2	2	2	2.141737246	2.14022021	2.3070652	3.407202941
ENSG00000090924	PLEKHG2	-0.787861386	0.330789334	1.366938184	0.069363787	0.579076798	8.928409675	8.004992147	9.926432745	8.420396336	8.33118782	10.43593029	10.5951862
LCMT1		0.473654576	0.806161404	0.105131983	0.958081539	0.578786559	7.624139768	7.901717006	2	7.577865504	7.74603955	7.108998216	7.476011586
ENSG00000158321	AUTS2	0.740931805	0.476344102	-0.162682632	0.886854075	0.578249173	4.467311984	7.002537973	6.80318718	7.265793319	7.07311172	6.471027105	6.113568999
ENSG00000141665	FBXO15	0.189126244	0.950359078	0.388294319	NA	0.577420563	2	3.076665209	2	2.784612485	2.49394783	2.937246078	2.466008509
ENSG00000175356	SCUBE2	1.134152537	0.292494247	-0.556991643	0.622741559	0.577160893	3.327711234	5.131603625	4.058998049	5.48800675	5.30456641	4.04171018	3.924507493
ENSG00000159917	ZNF235	0.877181161	0.661258435	-0.300595956	0.887741643	0.576585205	6.138360429	3.076665209	2	5.686138681	5.40529704	4.563936166	4.479073019
ENSG00000164307	ERAP1	0.842849817	0.323812042	-0.266336103	0.774665597	0.576513714	8.257427002	9.506147172	10.40208303	10.45897359	10.4897738	9.359183899	9.380643788
ENSG00000170222	ADPRM	0.504669156	0.737405845	0.071284027	0.965258777	0.575953183	3.327711234	4.93638933	6.958202216	6.139181293	6.33555364	5.960710379	5.694529741
ENSG00000126062	TMEM115	0.024336174	0.973778114	0.551335334	0.392649764	0.575671507	9.848787621	10.08551947	8.699419242	9.611716938	9.75534491	10.1231663	10.29224003
ENSG00000000457	SCYL3	0.693695718	0.709351398	-0.11844719	0.952589029	0.575248529	5.70932728	7.503546079	2	6.89733479	7.07311172	6.302895343	6.110033886
ENSG00000032219	ARID4A	0.866051575	0.221769007	-0.295466468	0.701056475	0.570585107	7.33131396	6.951736442	8.408111533	8.567402595	8.5227358	7.577730839	7.203223239
ENSG00000215440	NPEPL1	1.04014288	0.024327615	-0.470247838	0.344285984	0.569895042	7.940205782	7.145017454	7.398165132	8.425603456	8.66157866	6.970858896	7.156006254
ENSG00000066557	LRRC40	0.688715983	0.226161058	-0.119765167	0.852959423	0.568950817	6.257048221	6.602424674	7.225742779	7.381065997	7.44169935	6.782996409	6.454714037
ENSG00000141736	ERBB2	0.003098851	0.997041173	0.565529491	0.400315488	0.568628341	9.204317094	10.14890644	8.721235202	9.471064221	9.51003006	9.95290183	10.19419604
ENSG00000197265	GTF2E2	0.089094941	0.962823674	0.47952356	0.795268255	0.568618502	3.327711234	8.029695761	9.439621147	8.439397922	8.39557285	8.934738262	8.660355764
ENSG00000105948	TTC26	0.463794743	0.709997456	0.104107428	0.937449278	0.567902171	4.467311984	6.844429755	4.874587708	6.252910529	6.26836889	5.614651724	6.175763085
ENSG00000100599	RIN3	-0.091464764	0.916568051	0.65852947	0.380792862	0.567064706	8.16936288	6.464272742	7.646393253	7.494428494	7.47849652	8.050818921	8.377561365
ENSG00000139517	LNX2	-0.35865738	0.62100324	0.925075168	0.150484813	0.566417788	5.70932728	6.311476754	6.322058544	5.839660368	5.78232107	7.354572674	6.617932662
ENSG00000198912	C1orf174	-0.335229607	0.714367242	0.901503383	0.273716572	0.566273777	7.750913381	7.73146271	5.772716771	7.021746293	7.00136498	8.271259924	8.17168765
ENSG00000124588	NQO2	-0.043265075	0.970415686	0.608621201	0.565677502	0.565356125	7.940205782	8.297207175	5.59507254	7.45762193	7.78127547	8.086505553	8.432373803
ENSG00000228716	DHFR	1.574295665	0.262309738	-1.01262014	0.482463036	0.561675525	3.327711234	7.638155693	6.80318718	8.254744042	8.32017198	5.238348159	6.189227296
ENSG00000142867	BCL10	0.688522688	0.465992837	-0.128078692	0.902389555	0.560443996	8.998234871	6.728495673	7.858115942	8.84120963	8.80016655	7.966556098	8.059781405
ENSG00000183775	KCTD16	-1.32373524	0.651415542	1.884035983	0.500719988	0.560300743	2	2	8.608698767	5.880681751	5.69405321	9.08127623	8.697340724
ENSG00000140993	TIGD7	1.383448926	0.114419311	-0.823289016	0.366065794	0.56015991	4.815609471	5.131603625	5.156626401	6.351046291	6.28357155	4.742549839	3.807156736
ENSG00000107341	UBE2R2	0.099049751	0.833540602	0.460653339	0.256328984	0.55970309	10.29268328	10.09139839	9.589475645	10.09992864	10.1401137	10.58136152	10.37225646
ENSG00000213901	SLC23A3	1.041495701	0.653324928	-0.482809942	0.842082048	0.558685759	2	2	4.874587708	4.358765249	4.37434574	3.500668998	3.042026731
ENSG00000107262	BAG1	1.269388211	0.193841835	-0.710886391	0.48356437	0.55850182	6.653098118	9.170736405	7.734844356	9.437090644	9.51728265	7.35696023	7.66855289
ENSG00000018869	ZNF582	0.40975959	0.804532535	0.148707015	0.930981294	0.558466606	2	5.457120953	5.59507254	5.406080789	5.34860058	5.606648123	4.456980017
ENSG00000113360	DROSHA	0.190244139	0.895265695	0.367452602	0.788187265	0.557696741	8.737455672	9.148351514	5.39244166	8.583021623	8.66303256	8.729394257	8.865589219
ENSG00000148943	LIN7C	0.611774396	0.697744171	-0.056832455	0.97269175	0.554941941	4.00722531	8.414402626	8.805342542	8.729158235	8.61275919	8.062569037	7.959237827
ENSG00000181016	LSMEM1	1.319856956	0.552095038	-0.764960798	0.738292369	0.554896158	2	2	4.874587708	4.739373666	4.43005853	3.235948948	3.012034821
ENSG00000169359	SLC33A1	0.577512865	0.436535627	-0.02271399	0.977998169	0.554798875	7.975256505	7.002537973	8.65477198	8.551612617	8.62474679	8.021770574	7.970003494
ENSG00000140092	FBLN5	-0.028871809	0.96053419	0.583595048	0.245225364	0.554723238	10.56993315	9.549500232	10.24529339	10.19090967	10.1079152	10.71590919	10.80418236
ENSG00000114857	NKTR	0.551877239	0.549066587	0.001733458	0.998969048	0.553610697	8.16936288	10.23640192	8.825624954	9.908654498	9.88247671	9.413813486	9.279629951
ENSG00000158856	DMTN	-0.182885058	0.911458513	0.736069965	0.613476865	0.553184907	2	4.11352228	4.874587708	3.840289928	4.02226117	4.933813368	4.329022995
ENSG00000165704	HPRT1	0.530899193	0.331331826	0.022080833	0.971808785	0.552980026	7.33131396	6.602424674	6.718950348	7.523859802	7.32531744	6.763331418	7.085754446
ENSG00000085415	SEH1L	0.156729678	0.915012803	0.396143222	0.775777561	0.552872901	4.815609471	8.639735838	7.858115942	7.959237999	7.91663606	7.853585565	8.435907908
ENSG00000162302	RPS6KA4	-0.19404378	0.81054847	0.746547465	0.290479733	0.552503685	7.218819781	8.234834549	8.825624954	8.015574133	8.06917614	8.721540982	9.186615069
ENSG00000123600	METTL8	0.344325292	0.768318166	0.207860119	0.861870179	0.552185411	5.330594134	8.213429689	7.163363831	7.778406195	7.55253065	7.494516225	7.576773052
ENSG00000077152	UBE2T	0.464710236	0.83197366	0.086301493	0.969279885	0.551011729	3.327711234	6.602424674	2	5.890757403	5.39133044	5.445480974	5.177806074
ENSG00000099949	LZTR1	0.578189271	0.714821112	-0.027280425	0.987221496	0.550908846	9.204317094	9.28800521	4.523675391	9.215512556	9.31111579	8.638111529	8.688479312
ENSG00000135838	NPL	-1.39040001	0.570724655	1.93920775	0.403634871	0.54880774	2	5.838937808	2	3.349643765	3.55551834	6.898949899	4.853519801
ENSG00000112964	GHR	-1.571678786	0.303447732	2.120125507	0.137639917	0.548446721	2	5.131603625	5.930865648	3.79808662	3.7864959	6.092633892	7.603417442
ENSG00000161904	LEMD2	-0.029171313	0.974752037	0.577385874	0.476093865	0.548214561	10.03472846	8.234834549	9.165043284	9.298270478	9.28868707	10.17076319	9.558468984

ENSG00000171357	LURAP1	0.480006047	0.641227131	0.064939631	0.953719218	0.544945678	4.815609471	4.93638933	4.058998049	5.37770462	4.72553021	4.572179576	4.870334174
ENSG00000072274	TFRC	0.373316877	0.801833271	0.171429488	0.91083145	0.544746365	6.89194353	11.05905903	10.62675862	10.70863622	10.6759114	10.207113	10.72810493
ENSG00000140367	UBE2Q2	0.528758618	0.371475952	0.015911812	0.981190943	0.54467043	8.107517749	8.376385542	9.242516611	9.215512556	9.14521857	8.733534631	8.60805852
ENSG00000090612	ZNF268	0.273809934	0.899665024	0.270207096	0.900092939	0.54401703	4.467311984	9.203675681	4.058998049	7.975897842	7.99492122	7.938932725	8.023598593
ENSG00000008735	MAPK8IP2	1.762634467	0.435930907	-1.22196406	0.594394464	0.540670407	2	6.04685525	2	6.372775472	6.12381559	3.904814898	3.496556178
ENSG00000114405	C3orf14	1.588040315	0.212730396	-1.047578356	0.424853745	0.540461959	5.866944937	3.686303414	3.369317972	6.252910529	6.1068222	4.122514202	3.660199087
ENSG00000137509	PRCP	0.444470304	0.528239125	0.095580686	0.901587015	0.54005099	10.78274301	9.549500232	9.624627483	10.51093171	10.5791198	9.972107965	10.39258649
ENSG00000132958	TPTE2	3.417477183	0.412304476	-2.881694072	0.511130806	0.53578311	2	2	2	3.921164147	3.86943969	2.094598369	2
ENSG00000134684	YARS	-0.150292897	0.773531565	0.686064919	0.1272864	0.535772022	10.46903009	10.61666558	9.746650996	10.1342586	10.217847	11.06635027	10.95305641
ENSG00000124209	RAB22A	0.21428859	0.512728968	0.321004834	0.29554872	0.535293424	9.097018025	9.308339287	9.478569125	9.55265467	9.47734778	9.564482569	9.676696925
ENSG00000180773	SLC36A4	-0.050617735	0.977133743	0.585837449	0.721968109	0.535219714	3.327711234	7.431218269	8.486635645	7.491120977	7.39696604	7.862855183	8.250064747
ENSG00000114933	INO80D	0.588163966	0.08123451	-0.053524017	0.894789442	0.534639949	8.340423424	8.337339476	8.042706615	8.869506572	8.79487126	8.296978073	8.091498142
ENSG00000004961	HCCS	0.047907451	0.981829576	0.484544516	0.80343258	0.532451967	3.327711234	6.899080544	9.491321578	8.197043419	8.18299958	8.518969664	8.717227336
ENSG00000162873	KLHDC8A	3.425341355	0.548693531	-2.893582639	0.624498271	0.531758716	2	2	2	4.694064037	2	2.094598369	2
ENSG00000165792	METTL17	0.674370657	0.747288938	-0.143426893	0.948326217	0.530943764	5.095959458	8.004992147	2	7.348537858	7.21466714	6.435321218	6.549111181
ENSG00000101945	SUV39H1	0.060868933	0.966162087	0.469384177	0.725041248	0.530253109	3.327711234	5.838937808	4.523675391	4.907595604	5.09650239	4.841348851	5.75463059
ENSG00000109606	DHX15	0.224522547	0.625309666	0.305222215	0.487297152	0.529744761	9.460782017	10.10308486	9.478569125	9.961593621	9.92005068	9.927424911	10.10957632
ENSG00000093144	ECHDC1	0.566803269	0.255069337	-0.037263695	0.948615522	0.529539575	8.777722034	8.469613413	7.934808822	9.066811112	8.92538942	8.175765162	8.593575433
ENSG00000071246	VASH1	-0.090076681	0.965522332	0.619212773	0.752411321	0.529136091	7.031698319	8.101365653	2	6.912249745	7.10341274	8.229523742	6.864442837
ENSG00000085788	DDHD2	-0.263089682	0.666522361	0.792115073	0.142327697	0.529025391	9.048471759	8.077869079	9.180874355	8.540455898	8.61576544	9.703944794	9.543476778
ENSG00000002919	SNX11	0.145580757	0.950922102	0.383146536	0.870919566	0.528727293	9.48530526	6.389896633	2	8.139053376	8.27714776	8.304419417	8.571895748
ENSG00000158828	PINK1	0.823447028	0.368407723	-0.295601233	0.763865232	0.527845795	7.579279349	9.896993485	9.491321578	10.06531795	10.1359316	9.018187664	8.957466266
ENSG00000156050	FAM161B	0.035179293	0.981829576	0.492030307	0.724837675	0.5272096	7.096806373	6.951736442	3.369317972	6.64692533	6.42052719	7.246693284	6.64749689
ENSG00000171813	PWWP2B	-0.200322101	0.905447484	0.726596858	0.632965021	0.526274757	3.327711234	7.274681896	7.896971941	6.841288255	6.91125523	8.098683215	7.370638252
ENSG00000256806	C17orf100	0.315682678	0.853531206	0.209968464	0.902389591	0.525651142	2	6.140560021	4.874587708	5.363304456	5.48637102	5.196301326	5.453611367
ENSG00000109971	HSPA8	-0.02427492	0.945043724	0.549373331	0.054901185	0.525098411	13.57305719	13.45717518	13.13440312	13.36470886	13.3867068	13.98526566	13.91224726
ENSG00000184277	TM2D3	-0.156150533	0.885189733	0.681139646	0.471649635	0.524989113	6.138360429	8.356994601	8.460932916	7.829061569	7.80253898	8.634179825	8.649438172
ENSG00000168438	CDC40	1.042076293	0.305827583	-0.519108036	0.626596436	0.522968257	8.517314938	7.431218269	5.772716771	8.627383409	8.68323472	7.192914486	7.04745562
ENSG00000187513	GJA4	-3.75301868	0.514704475	4.275376839	0.414211825	0.522358159	2	2	2	2	2	4.996409624	3.956354764
ENSG00000140968	IRF8	-3.753012457	0.521753772	4.274127284	0.423211461	0.521114827	2	2	2	2	2	5.147485955	3.580696614
ENSG00000100722	ZC3H14	0.715911054	0.519228435	-0.195016238	0.870966304	0.520894817	8.75772933	9.763446433	6.80318718	9.615519136	9.57479964	8.568225482	8.804020138
ENSG00000080815	PSEN1	0.446225931	0.313361774	0.074576157	0.882758816	0.520802088	10.34051281	9.632483534	9.69246155	10.38025813	10.3568427	10.05256229	9.941741079
ENSG00000156232	WHAMM	0.191056695	0.88968542	0.329369043	0.800584348	0.520425738	5.866944937	6.844429755	8.904018013	7.917958914	7.97632944	8.249579355	7.895982618
ENSG00000197329	PELI1	0.613614272	0.510049398	-0.096171248	0.926209474	0.517443024	6.73718411	7.901717006	5.772716771	7.659688863	7.69294162	7.086154752	6.874895611
ENSG00000083844	ZNF264	0.186591168	0.880334493	0.330656153	0.777382971	0.517247321	6.563806279	7.761270542	9.330306409	8.559529208	8.43013848	8.82044307	8.430957733
ENSG00000105792	CFAP69	1.882285193	0.28497373	-1.365756055	0.445853185	0.516529138	2	5.457120953	3.369317972	5.968925393	6.00043082	3.782262616	2.746601469
ENSG00000138346	DNA2	0.302394651	0.911302045	0.210220908	0.937572507	0.512615559	2	5.72254323	2	4.470469939	4.76938658	5.068107405	3.735548302
ENSG00000198483	ANKRD35	0.100337558	0.957413691	0.411815082	0.820281623	0.51215264	5.866944937	2	5.156626401	5.128039203	5.06165168	4.675266036	5.842465082
ENSG00000127423	AUNIP	-1.13735904	0.478467424	1.649047666	0.258489631	0.511688626	2	4.442710129	3.369317972	3.023133209	2.94009902	4.690491514	5.171036168
ENSG00000125895	TMEM74B	0.055935097	0.97695465	0.455345742	0.802702997	0.511280839	2	4.710564154	3.369317972	3.709785322	3.98554648	4.530483275	3.640730416
ENSG00000188997	KCTD21	0.037948177	0.983064817	0.472154082	0.778107221	0.51010226	8.540903468	7.232742069	3.369317972	7.464384256	7.55566492	8.008923105	7.860656447
ENSG00000274320		4.557005953	1.43E-09	-4.049428319	5.31E-06	0.507577634	4.467311984	3.686303414	4.523675391	8.514626115	8.40429221	2.34604626	2.225841198
ENSG00000135414	GDF11	-0.045325292	0.98350331	0.552011935	0.79391096	0.506686643	7.867441304	9.246456519	2	8.109160159	8.08227582	8.744517886	8.624876935
ENSG00000135953	MFSD9	0.18708487	0.917958722	0.319080795	0.85594901	0.506165665	6.816637977	7.002537973	2	6.580005553	6.49425375	6.732402829	6.592824411
ENSG00000213672	NCKIPSD	-0.22307134	0.92127192	0.727033664	0.728633609	0.503962324	9.448362284	7.954278496	2	8.013270196	8.15847021	8.919069795	9.128396091
ENSG00000086758	HUWE1	0.006466878	0.979997472	0.495012427	0.012586703	0.501479304	11.55070946	11.55651981	11.51057165	11.49601699	11.5944788	11.96871957	12.09663891
ENSG00000135723	FHOD1	-0.269855881	0.719407817	0.771109912	0.252830324	0.501254031	7.709880939	8.848535039	8.536706852	8.076429088	8.27144011	8.659546439	9.604418524
ENSG00000064703	DDX20	0.250560262	0.903601303	0.250565645	0.90231922	0.501125906	2	8.169643011	7.97167868	7.756533788	7.7377845	7.852739912	7.633277551
ENSG00000137955	RABGGTB	0.267875162	0.90624054	0.229811202	0.918425911	0.497686363	9.371511179	7.701025996	2	8.423869837	8.47883193	8.447394152	8.379764957
ENSG00000167895	TMC8	-2.305372515	0.653114396	2.801478826	NA	0.496106312	2	2	2	2.141737246	2	3.767980144	3.100202476
ENSG00000047315	POLR2B	0.49489604	0.506870806	0.000940844	0.999263508	0.495836884	8.075571721	9.616264339	9.577565424	9.715981692	9.75874862	9.306733157	9.183254692
ENSG00000072609	CHFR	-0.013742937	0.990573498	0.509208692	0.625351493	0.495465755	9.2903348	8.191702463	6.629488387	8.402902269	8.38151133	8.880175401	8.939154596
ENSG00000138182	KIF20B	0.215590503	0.937471333	0.279467763	0.919037965	0.495058266	2	6.666836837	2	5.474671438	5.33407135	5.650128416	5.250250853
ENSG00000183397	C19orf71	-3.2611602	0.222140997	3.756160483	0.095497186	0.495000284	2	2	4.058998049	2.270786764	2	6.192262702	5.379200993
ENSG00000142065	ZFP14	0.160028995	0.876399317	0.333876736	0.725625206	0.493905732	5.532353208	6.04685525	6.073378139	6.113598934	6.00043082	6.7543034	5.367404784
ENSG00000072694	FCGR2B	-3.753019347	0.514396922	4.246589535	0.417384315	0.493570189	2	2	2	2	2	4.854926465	4.147809227
ENSG00000122884	P4HA1	0.708014246	0.209654079	-0.215031078	0.727736031	0.492983169	12.17515252	11.22065959	11.34128524	12.38036311	12.3222493	11.13046669	11.67715885
ENSG00000138674	SEC31A	0.071369211	0.931247849	0.419753429	0.573211571	0.49112264	11.55650736	12.49586584	10.86096535	11.8525616	11.878197	12.26512815	12.15992538
ENSG00000134851	TMEM165	1.061274942	0.089945888	-0.572890886	0.380616152	0.488384057	10.91047246	9.763446433	9.658943166	11.2913796	11.2838779	9.56938092	9.742104636
ENSG00000143575	HAX1	-0.053316308	0.966718151	0.541674471	0.64649554	0.488358163	9.751047882	9.246456519	6.534109959	8.941551548	9.0020652	9.573749602	9.552621009
ENSG00000170234	PWWP2A	0.203743946	0.883143303	0.282244147	0.832998352	0.485988093	5.330594134	7.503546079	8.763901704	7.971157485	7.97164382	8.292619498	7.754841567
ENSG00000163738	MTHFD2L	1.26578566	0.190366567	-0.784705442	0.432381295	0.481080218	5.70932728	5.457120953	3.369317972	6.268449691	6.40670796	4.675266036	4.292061816
ENSG00000188986	NELFB	0.047152593	0.956792211	0.43391207	0.581663843	0.481064662	8.138771685	8.169643011	9.61300511	8.875861021	8.84312657	9.201728932	9.283950453
ENSG00000178904	DPY19L3	-0.154814657	0.926810059	0.634270891	0.683005276	0.479456235	4.00722531	8.433041974	8.742726175	7.971157485	7.80253898	8.437839892	8.866637078
ENSG00000131171	SH3BGRL	0.363167157	0.681613489	0.116176724	0.901737951	0.479343881	9.635285994	10.05576005	8.042706615	9.869705991	9.79168681	9.645817872	9.522355441
ENSG00000166164	BRD7	0.658072595	0.54335456	-0.179318203	0.879564373	0.478754392	8.797441463	8.169643011	5.930865648	8.648320862	8.74905888	7.782541424	7.958255135
ENSG00000143502	SUSD4	2.146708035	NA	-1.67157831	NA	0.475129725	2	2	2	2.868578001	3.28037825	2.048074405	2.172608095
ENSG00000157600	TMEM164	0.357308632	0.863599094	0.116466122	0.955852858	0.473774754	8.009475818	8.522788974	2	8.065351606	8.06917614	7.694662924	7.954317658
ENSG00000135870	RC3H1	0.668102033	0.533299715	-0.194518901	0.866671393	0.473583132	9.435834703	8.029695761	6.718950348	9.163478821	9.08575246	8.213803048	8.325926932
ENSG00000183648	NDUFB1	0.630682073	0.522626682	-0.158023864	0.883361661	0.472658208	8.39321512	6.602424674	6.43197674	8.058664058	8.04261444	7.093332266	7.441266415
ENSG00000162337	LRP5	-0.992399322	0.479583688	1.464617861	0.268612714	0.472218538	10.31337612	6.464272742	7.97167868	8.109160159	8.06258142	10.27622952	10.738159
ENSG00000050393	MCUR1	0.025756811	0.970415686	0.446241172	0.471041116	0.471997983	7.33131396	7.819099893	8.585098385	8.130575473	7.91419618	8.427654172	8.454843905
ENSG00000123219	CENPK	0.52621539	0.832844524	-0.054631528	0.983038213	0.471583862	5.095959458	2	2	4.237680128	4.12711147	3.904814898	3.580696614
ENSG00000159164	SV2A	-0.198618799	0.859388318	0.66990954	0.502317168	0.471290741	7.031698319	9.235879652	9.601288346	8.754208387	8.8041253	9.475816969	9.79094187
ENSG00000284770	TBCE	-0.206672183	0.891546154	0.677390394	0.621584245	0.470718211	4.00722531	7.979858146	7.646393253	7.035434774	7.14562489	8.000548654	7.910280955
ENSG00000139722	VPS37B	0.276163281	0.832844524	0.193652363	0.883160796	0.469815644	6.138360429	8.147241016	9.439621147	8.75144642	8.68895519	8.555288804	8.717808107
ENSG00000110076	NRXN2	-2.494587274	0.24868502	2.963059295	0.142448826	0.468472022	2	5.457120953	2	2.947924294	2.38539787	6.106889539	7.287011591
ENSG00000105556	MIER2	0.494932959	0.538867025	-0.027148661	0.97520757	0.467784298	9.410448395	7.761270542	8.143093325	9.128781483	9.08466797	8.676372393	8.496731673
ENSG00000134371	CDC73	0.515606155	0.618837592	-0.048949378	0.966283503	0.466656778	9.886101849	8.029695761	7.691296561	9.407570435	9.37460909	8.933938877	8.719549018
ENSG00000103197	TSC2	-0.004368852	0.997041173	0.470884716	0.587320792	0.466515864	7.82963488	9.916994052	9.272377397	9.219515356	9.25534053	9.6668989	9.752061939
ENSG00000117616	RSRP1	0.620521451	0.463760408	-0.154387581	0.866824598	0.46613387	9.579430841	8.255926468	7.97167868	9.381011913	9.41399842	8.958123037	8.201061123
ENSG00000129351	ILF3	-0.026050793	0.970543625	0.491765087	0.432815399	0.465714294	9.761125267	10.92721483	11.01019887	10.61487847	10.6676062	11.02725374	11.27827087
ENSG00000101265	RASSF2	-1.099073218	0.313653871	1.563904084	0.120494972	0.464830866	4.467311984	4.442710129	5.39244166	4.329433326	3.39680241	6.537789343	5.867805474
ENSG00000162695	SLC30A7	0.957095799	0.033936427	-0.492281157	0.302337918	0.464814642	8.696033109	9.235879652	8.325066185	9.773608662	9.74577157	8.287622092	8.355338318
ENSG00000196866	HIST1H2AD	5.655350111	0.114206862	-5.191125726	0.193537693	0.464224385	2	2	2	5.829220145	5.74985018	2	2
ENSG00000197043	ANXA6	0.595260866	0.562304924	-0.131396613	0.90737308	0.463864253	12.07772327	9.506147172	10.89495479	11.74177109	11.8025467	10.84589444	11.22329526
ENSG00000164366	CCDC127	-0.267947256	0.663217573	0.728343034	0.183892387	0.460395778	8.138771685	7.979858146	7.029880454	7.546342806	7.5240115	8.651788724	8.365751687
ENSG00000177853	ZNF518A	0.548347441	0.779920665	-0.08830035	0.966407561	0.460047091	7.159102577	7.954278496	2	7.536750104	7.62295033	7.433611594	6.251520369
ENSG00000167670	CHAF1A	0.323744655	0.709501472	0.134427913	0.883002588	0.458172568	5.330594134	6.04685525	7.029880454	6.449761039	6.73998187	6.277913523	6.55171954
ENSG00000157578	LCA5L	0.456777446	0.846875777	-0.001124696	0.99945274	0.45565275	2	4.11352228	2	3.406930997	3.45167086	3.215043717	3.100202476
ENSG00000090006	LTBP4	-0.961029126	0.16672399	1.414780339	0.031022179	0.453751213	10.1076228	8.702538795	9.577565424	8.573670512	8.65720817	10.51529534	11.33084866
ENSG00000141027	NCOR1	0.224337312	0.67689906	0.229122074	0.666121968	0.453459386	10.19941743	10.36815316	11.08782089	10.81365108	10.8423598	10.91840948	10.74187559
ENSG00000122778	KIAA1549	1.208045071	0.492183068	-0.755393369	0.675750281	0.452651702	3.327711234	8.124485672	4.874587708	7.860161659	7.97632944	6.184218099	5.842465082
ENSG00000122958	VPS26A	0.714145897	0.138731984	-0.261790378	0.616400319	0.452355519	8.493334308	9.235879652	9.426401271	9.814540128	9.81606952	8.728933481	8.953526633
ENSG00000133028	SCO1	0.035348309	0.946040014	0.415821794	0.360288866	0.451170102	7.750913381	8.395519306	8.585098385	8.330724217	8.31278108	8.707577112	8.688479312
ENSG00000174130	TLR6	0.232326039	0.82001794	0.218165907	0.826511578	0.450491946	4.467311984	5.131603625	4.058998049	4.867343068	4.85329164	4.799832052	4.895193906
ENSG00000161996	WDR90	0.371630407	0.802275213	0.077282386	0.960122226	0.448912794	4.00722531	6.228547981	7.858115942	7.021746293	7.18258783	6.67615119	6.949978473
ENSG00000197857	ZNF44	0.396165672	0.531143362	0.052628058	0.939798462	0.44879373	6.96351253	6.535001748	5.772716771	6.841288255	6.93551934	6.613628927	6.501335855
ENSG00000137601	NEK1	0.446082949	0.821801475	0.001545863	0.999378208	0.447628812	2	7.701025996	8.042706615	7.857595438	7.62593544	7.399274054	7.218077721
ENSG00000168872	DDX19A	0.924886113	0.114918885	-0.477598324	0.438543047	0.447287789	8.836089686	8.169643011	7.552162999	9.263797825	9.12531607	7.688988646	7.921416787
ENSG00000159958	TNFRSF13C	-2.305383504	0.652705192	2.752312442	NA	0.446928938	2	2	2	2.141737246	2	3.429858444	3.45257121
ENSG00000166135	HIF1AN	-0.303943487	0.720184992	0.750860814	0.331246196	0.446917327	8.797441463	9.225224669	10.51254821	9.389919188	9.41227044	10.38463615	10.51542313
ENSG00000122678	POLM	-0.469530625	0.711000931	0.914884548	0.435748117	0.445353923	4.467311984	6.464272742	7.734844356	6.18902405	6.39974839	7.672789259	7.599641096
ENSG00000058262	SEC61A1	0.104146997	0.758250661	0.339996795	0.259132418	0.444143792	12.36881891	12.36244576	12.5724427	12.5306927	12.5526578	12.57786247	12.95272298
ENSG00000008130	NADK	0.308607842	0.715896274	0.135087304	0.879564373	0.443695146	10.16187685	8.505280662	8.805342542	9.607141034	9.69905569	9.435720286	9.525344379
ENSG00000145833	DDX46	0.384107681	0.658522427	0.059490682	0.949302416	0.443598364	8.910412251	9.34816733	7.398165132	9.161399541	9.13163078	8.637620652	8.990045056
ENSG00000113810	SMC4	1.027342969	0.531143362	-0.58387539	0.728705652	0.443467578	3.327711234	8.639735838	8.143093325	8.888486565	8.84312657	7.571576479	6.913939481

ENSG00000108107	RPL28	0.256014654	0.722734802	0.186905236	0.79812555	0.44291989	11.99474663	11.41601719	10.48730704	11.65889637	11.7025256	11.59074223	11.63265648
ENSG00000167601	AXL	-0.17954499	0.64928304	0.620514132	0.072784469	0.440969141	11.59081378	12.01658788	12.05247881	11.70101821	11.7431293	12.33714807	12.68506543
ENSG00000035499	DEPDC1B	-0.815681997	0.717844348	1.255985836	0.543323761	0.44030384	2	3.076665209	3.369317972	2.784612485	2.38539787	3.891700877	3.383972333
ENSG00000185480	PARPBP	0.621515017	0.731956618	-0.181546781	0.925393616	0.439968237	2	5.457120953	3.369317972	5.058234798	4.60967607	4.281782022	4.09155917
ENSG00000119616	FCF1	0.15164245	0.915100867	0.288319839	0.833878439	0.439962288	6.138360429	7.145017454	9.372276026	8.343618883	8.32385328	8.483352582	8.454146986
ENSG00000171502	COL24A1	4.098040732	0.015484621	-3.658411256	0.050033331	0.439629476	2	4.11352228	2	6.490083648	6.48770488	2.048074405	2.225841198
ENSG00000124214	STAU1	0.21797436	0.66172885	0.221460203	0.651770088	0.439434563	10.87842776	10.019219	10.59756881	10.73648843	10.776052	10.67331779	10.8415673
ENSG00000113282	CLINT1	0.155826179	0.833643937	0.28232663	0.686013943	0.438152809	8.836089686	9.664384941	10.30429804	9.852410253	9.89922864	10.06575858	9.935263322
ENSG00000135404	CD63	0.851768641	0.126838044	-0.414006577	0.482146792	0.437762064	13.5666075	12.46789278	12.83381799	13.87862076	13.8845443	12.38044183	12.81904935
ENSG00000189376	C8orf76	1.596256949	0.077375879	-1.158858494	0.208960532	0.437398454	7.33131396	5.595927442	5.39244166	7.829061569	8.02236724	5.48093935	5.101530266
ENSG00000099940	SNAP29	-0.071602366	0.897105982	0.505247226	0.279927595	0.43364486	8.797441463	9.125613817	8.266922072	8.719297823	8.69607402	9.142413344	9.405730946
ENSG00000144895	EIF2A	0.417555453	0.463231524	0.015729636	0.980204682	0.433285089	9.410448395	8.356994601	9.31604076	9.491070865	9.5268964	9.096358634	9.123144518
ENSG00000176986	SEC24C	0.564020013	0.108473389	-0.131324007	0.738341738	0.432696006	10.00110988	9.549500232	10.02619908	10.39714397	10.468729	9.620951746	9.851208114
ENSG00000147443	DOK2	-1.310474018	NA	1.74257277	NA	0.432098752	2	2	2	2.141737246	2.14022021	3.316666208	2.277179783
ENSG00000167550	RHEBL1	2.025768347	0.170091125	-1.594720972	0.290456069	0.431047375	2	4.442710129	3.369317972	5.37770462	5.18011485	2.962551659	2.551896972
ENSG00000136783	NIPSNAP3A	0.650220131	0.568853498	-0.219976	0.856534247	0.430244132	6.009025997	7.315436877	8.865353885	8.503178519	8.46222608	7.435871964	7.79392309
ENSG00000172831	CES2	0.548635003	0.196836305	-0.1197069	0.801853641	0.428928104	9.143983763	8.687092496	8.742726175	9.314267267	9.50922197	8.504505158	8.962865756
ENSG00000125257	ABCC4	1.278618505	0.065445182	-0.849779024	0.23478853	0.428839481	7.940205782	8.590771778	6.882775709	9.192278409	9.28397021	7.263334844	7.010010317
ENSG00000155636	RBM45	0.536246302	0.798939121	-0.108021726	0.961024044	0.428224576	2	7.431218269	4.523675391	6.490083648	6.66650236	5.963846589	5.976631893
ENSG00000158716	DUSP23	-0.214008818	0.912931516	0.641126259	0.723919381	0.427117441	2	7.002537973	7.646393253	6.456560224	6.70653264	7.244115921	7.567781104
ENSG00000144736	SHQ1	0.055629245	0.918975348	0.371427376	0.433240072	0.427056621	7.031698319	7.315436877	7.098165388	7.230526165	7.19871665	7.296052918	7.719319651
ENSG00000005812	FBXL3	0.114730942	0.934796779	0.3121848	0.816103107	0.426915741	6.257048221	8.101365653	9.746650996	8.851563757	8.67892944	9.022330083	8.902329908
ENSG00000068323	TFE3	-0.045164025	0.943520433	0.47177711	0.397331772	0.426613085	10.04301211	9.71095217	10.61515347	10.06476182	10.1833083	10.2814236	10.92805327
ENSG00000204084	INPP5B	0.282747339	0.805210364	0.143413153	0.902554091	0.426160492	5.095959458	7.538394143	7.818184403	7.527093191	7.45854177	7.4096614	7.302581437
ENSG00000104129	DNAJC17	0.611591022	0.707094181	-0.186533355	0.914029999	0.425057667	2	5.303523747	6.43197674	6.01571348	5.9725602	5.119635168	5.390901532
ENSG00000067560	RHOA	-0.531493973	0.413879953	0.955686714	0.111723144	0.424192741	13.24354095	12.11221489	12.12791662	12.06350133	12.0702787	13.36457616	13.72000673
ENSG00000060982	BCAT1	0.484623668	0.410126067	-0.060656194	0.927796772	0.423967475	9.579430841	10.76748534	10.09465441	10.75004251	10.6795076	10.21634679	10.12520267
ENSG00000082212	ME2	-0.005589821	0.997041173	0.429390169	0.710106162	0.423800348	6.257048221	7.538394143	9.083172337	8.093978021	8.02236724	8.641543003	8.315988368
ENSG00000080298	RFX3	0.430323887	0.85603014	-0.006743294	0.998049427	0.423580593	2	7.315436877	3.369317972	6.283823264	6.26836889	5.979426183	5.740981724
ENSG00000117682	DHDDS	-0.169865697	0.82182886	0.592892691	0.372337042	0.423026994	7.940205782	9.256956408	9.330306409	8.762462704	8.83669913	9.427224679	9.676995613
ENSG00000136478	TEX2	-0.796491874	0.258819968	1.218655036	0.066009623	0.422163162	8.19931886	9.192779271	9.859153765	8.411675819	8.47717992	10.48669015	10.40706314
ENSG00000131653	TRAF7	0.05815062	0.891376437	0.36319483	0.314431097	0.42134545	9.931427102	9.34816733	9.636156974	9.723752619	9.70118012	9.98393497	10.04786406
ENSG00000089820	ARHGAP4	-0.105338751	0.959724553	0.526570637	0.793118059	0.421231886	3.327711234	5.72254323	2	4.387512679	4.4836996	5.463319095	4.215171317
ENSG00000164442	CITED2	-0.740184813	0.145213802	1.157965778	0.015354606	0.417780966	12.16759578	11.29386853	11.51370025	10.97299388	10.9635316	12.59272163	13.09279326
ENSG00000055070	SZRD1	0.110964005	0.87974571	0.306461429	0.645978013	0.417425434	10.87379128	9.910357957	11.1930463	10.86406385	10.8617987	10.79364543	11.2814173
ENSG00000100227	POLDIP3	-0.193262414	0.588054914	0.60926883	0.051318907	0.416006416	9.317904228	9.799806309	9.714382178	9.44052423	9.43116542	10.23609884	10.23354621
ENSG00000151304	SRFBP1	0.672006078	0.500544612	-0.257276447	0.807781951	0.414729631	6.96351253	4.93638933	7.285536013	7.322721205	7.33993465	6.520853931	6.341697848
ENSG00000158985	CDC42SE2	0.16112983	0.890185466	0.253462113	0.819999326	0.414591942	6.257048221	8.955547846	7.934808822	8.291333947	8.21902896	8.382079673	8.310608339
ENSG00000132664	POLR3F	0.226318052	0.908151255	0.18763624	0.923213606	0.413954293	2	7.35507212	7.502634626	7.144720234	7.01963893	6.956760977	7.130061054
ENSG00000130939	UBE4B	-0.446981021	0.665847367	0.860753237	0.36864007	0.413772217	8.340423424	9.672251278	7.225742779	8.235106869	8.38327653	9.600191431	9.615680827
ENSG00000169188	APEX2	-0.417885488	0.778992908	0.826262323	0.551011557	0.408376835	8.257427002	6.951736442	4.058998049	6.809774134	6.81514011	8.001311979	8.051511707
ENSG00000005075	POLR2J	0.831128975	0.162897673	-0.423333156	0.497326437	0.407795819	8.540903468	8.101365653	7.225742779	8.811020771	8.9398551	7.535143176	7.736616914
ENSG00000108433	GOSR2	0.213126221	0.858165041	0.194536156	0.8682894	0.407662377	9.397585735	10.19331737	7.285536013	9.59332559	9.59930725	9.557233497	9.59812349
ENSG00000172382	PRSS27	-0.114106222	0.964071163	0.520997366	0.832967479	0.406891144	2	5.131603625	2	4.034562184	3.55551834	4.698044417	3.735548302
ENSG00000164244	PRRC1	-0.016544213	0.984277478	0.423070971	0.586489016	0.406526758	8.517314938	10.26269067	9.945092785	9.740563462	9.72712735	10.10286127	10.23739929
ENSG00000285077	ARHGAP11B	5.596258368	0.124401402	-5.191124779	0.199257862	0.40513359	2	2	2	5.742861563	5.72778967	2	2
ENSG00000240849	TMEM189	0.080489471	0.891774766	0.324585425	0.536695429	0.405074896	9.867565369	9.148351514	10.13584611	9.823772619	9.87684923	10.00976979	10.17299833
ENSG00000180190	TDRP	0.17629205	0.950011373	0.228437564	0.936599654	0.404729614	2	7.189546432	2	6.069927018	5.6121243	6.060766632	5.736403255
ENSG00000119041	GTF3C3	0.214441688	0.711059078	0.189559746	0.740394508	0.404001435	8.257427002	8.590771778	7.646393253	8.321442665	8.54182783	8.57541967	8.225770986
ENSG00000128654	MTX2	0.497158934	0.765943216	-0.093678129	0.957844617	0.403480805	7.82963488	8.505280662	3.369317972	8.054188409	8.22100445	7.726402307	7.370638252
ENSG00000115459	ELMOD3	0.491898806	0.818202168	-0.088960767	0.968233365	0.402938038	2	6.535001748	8.561105505	7.783822874	7.78395061	7.527744791	6.81752904
ENSG00000115042	FAHD2A	0.104866349	0.957853023	0.298003595	0.880432293	0.402869943	2	7.189546432	7.97167868	7.178012736	7.14977896	7.27600668	7.424286753
ENSG00000141258	SGSM2	-0.231899655	0.784771092	0.634643574	0.408028698	0.402743919	9.602033055	7.847167909	8.65477198	8.697938662	8.58694992	9.389237988	9.609434849
ENSG00000095261	PSMD5	0.052562094	0.969963817	0.350106186	0.792798504	0.40266828	6.138360429	9.308339287	9.735974718	9.019382707	9.12320501	9.412953205	9.321117366
ENSG00000159055	MIS18A	0.281576951	0.829527562	0.120894106	0.927960061	0.402471057	3.327711234	6.228547981	5.156626401	5.662809983	5.5755357	5.524070859	5.419744333
ENSG00000070501	POLB	0.131048924	0.945043724	0.271410444	0.886197127	0.402459369	2	7.145017454	5.59507254	6.104969671	6.19790195	6.412547789	6.145001162
ENSG00000155016	CYP2U1	-1.948421925	0.21164903	2.350350764	0.117096798	0.401928839	3.327711234	6.844429755	8.699419242	5.578054239	5.6826301	9.896164504	9.729200569
ENSG00000134001	EIF2S1	0.036417628	0.97026695	0.364506672	0.689674098	0.4009243	10.41898845	10.00059544	8.266922072	9.854342273	9.84901661	10.1988183	10.15788386
ENSG00000170949	ZNF160	0.046840041	0.980534836	0.354070062	0.84516091	0.400910103	4.00722531	8.57407404	9.839340503	8.7747563	8.86350401	9.343316829	8.866637078
ENSG00000090686	USP48	0.139316038	0.785196727	0.260691772	0.585262994	0.40000781	9.27634997	8.903033433	8.408111533	9.014783325	9.07377798	9.207032339	9.121828633
ENSG00000140105	WARS	1.735181766	6.81E-20	-1.335924481	2.85E-12	0.399257285	11.98187803	11.89916567	12.03298648	13.68671956	13.7250429	10.72066788	10.55040485
ENSG00000180667	YOD1	0.752673724	0.528794428	-0.35453126	0.776319002	0.398142464	5.095959458	7.099070191	8.325066185	8.098332103	8.08661608	7.033394818	6.985139097
ENSG00000105856	HBP1	0.893583194	0.095791495	-0.496250216	0.374026188	0.397332978	10.3868074	9.246456519	9.703463497	10.73613921	10.7523441	9.380463355	9.338970358
ENSG00000173334	TRIB1	1.233596475	0.437642291	-0.837219839	0.603961617	0.396376636	3.327711234	7.669933291	8.511888463	8.811020771	8.8041253	7.106162485	6.350712758
ENSG00000122741	DCAF10	-0.372568876	0.499134136	0.767791807	0.12649658	0.395222932	9.544849385	9.876711741	9.25752426	9.246247604	9.18117744	10.69848203	9.887021781
ENSG00000144566	RAB5A	0.314636547	0.761583294	0.079166474	0.942375924	0.393803021	10.25038534	10.19877383	7.896971941	10.1262855	10.0557402	9.870700911	9.842979447
ENSG00000109929	SC5D	0.507420964	0.630303294	-0.115193585	0.919715042	0.392227378	7.27616306	9.506147172	9.926432745	9.83881318	9.7131598	9.103841116	9.210746501
ENSG00000168228	ZCCHC4	0.168438463	0.909988721	0.221517673	0.878629811	0.389956136	3.327711234	6.535001748	6.958202216	6.456560224	6.35006802	6.712030998	6.141542327
ENSG00000129636	ITFG1	1.08655283	0.000528881	-0.696690155	0.033633667	0.389862675	9.87686338	9.506147172	9.330306409	10.6527458	10.689886	8.918261679	8.874992666
ENSG00000103043	VAC14	0.870900054	0.190847995	-0.481365673	0.486954891	0.389534381	9.751047882	8.395519306	8.608698767	9.870978946	9.95192082	8.513629334	8.622397655
ENSG00000146243	IRAK1BP1	-0.460686808	0.598708746	0.846798209	0.285556212	0.386111401	4.815609471	5.303523747	4.523675391	4.497095146	4.60967607	5.54934408	5.85096143
ENSG00000108669	CYTH1	1.157143121	0.577257973	-0.774706838	0.714892595	0.382436284	9.344956677	7.274681896	2	9.170732795	9.26781654	7.506395059	7.094725293
ENSG00000147162	OGT	0.250308661	0.842102843	0.131638517	0.91798278	0.381947177	8.009475818	11.09461697	10.87075861	10.7217479	10.7601769	10.70903366	10.53087342
ENSG00000165568	AKR1E2	-3.753012982	0.522811731	4.134909296	0.440692188	0.381896314	2	2	2	2	2	4.230621763	4.644600858
ENSG00000108312	UBTF	-0.019561082	0.958728989	0.401453196	0.209143226	0.381892114	8.910412251	9.338312924	9.212024683	9.09649754	9.19932737	9.531835181	9.601903806
ENSG00000169884	WNT10B	0.323788626	0.912780559	0.057816098	0.984296564	0.381604724	2	3.686303414	2	3.406930997	2.49394783	3.082820997	2.671842496
ENSG00000090674	MCOLN1	0.869931401	0.462190956	-0.488709593	0.688288292	0.381221808	5.866944937	7.431218269	9.014156722	8.811020771	8.8250571	7.426809156	7.529919928
ENSG00000173269	MMRN2	-0.795478245	0.543644578	1.176369084	0.329610478	0.38089084	3.327711234	4.442710129	4.058998049	3.406930997	3.55551834	5.381251181	4.500832793
ENSG00000113583	C5orf15	0.760159909	0.044804086	-0.379432904	0.345156474	0.380727005	10.32020829	9.821190352	10.28245311	10.97151121	10.8541818	9.563449215	9.964920949
ENSG00000170500	LONRF2	-0.550063596	0.847129001	0.928944661	0.729041892	0.378881066	2	2	5.39244166	4.17310023	2.77779907	4.133696865	5.230851202
ENSG00000166192	SENP8	-0.319481101	0.875544825	0.698032268	0.710952496	0.378551167	3.327711234	5.946638857	2	4.387512679	4.4836996	5.29920634	5.32534152
ENSG00000221887	HMSD	-2.305309923	0.657619829	2.682918479	NA	0.377608557	2	2	2	2.141737246	2	3.215043717	3.560117069
ENSG00000106052	TAX1BP1	0.542143813	0.242184305	-0.167632904	0.739634542	0.374510909	9.931427102	10.82153847	10.55568472	11.0564817	10.9924432	10.30294393	10.33052636
ENSG00000166986	MARS	-0.176045435	0.830362923	0.550451877	0.453090834	0.374406442	10.74769552	10.44362967	9.099922433	10.06309216	10.0934307	10.93288529	10.65962843
ENSG00000177191	B3GNT8	0.748484633	0.679837753	-0.374229653	0.841697129	0.374254981	3.327711234	5.595927442	2	5.040242897	5.09650239	4.281782022	3.972018587
ENSG00000175215	CTDSP2	0.157315925	0.714313079	0.216483673	0.600319397	0.373799599	10.6855593	11.35134391	11.14139273	11.20443196	11.2817502	11.23321719	11.36896909
ENSG00000178381	ZFAND2A	1.057111836	0.405184227	-0.683463692	0.59841801	0.373648145	8.228665467	6.951736442	4.523675391	8.190940526	8.31648126	6.556607032	6.559516442
ENSG00000002549	LAP3	1.68013421	0.089428115	-1.307618354	0.189945688	0.372515856	10.57565445	9.318399851	7.818184403	11.30956651	11.3163477	8.038972319	8.590407604
ENSG00000158050	DUSP2	0.672976564	0.692285797	-0.302025746	0.864452193	0.370950818	3.327711234	5.131603625	2	4.71689672	4.56059747	3.838019203	3.908316285
ENSG00000104859	CLASRP	0.111502162	0.945987763	0.258247961	0.874266884	0.369750123	7.867441304	7.874700259	3.369317972	7.370304568	7.48837134	7.514972902	7.630812678
ENSG00000103942	HOMER2	-1.135905223	0.554188019	1.505294743	0.402484163	0.36938952	4.467311984	3.686303414	2	3.406930997	2.49394783	5.385935071	4.133950982
ENSG00000115652	UXS1	0.776340888	0.300991733	-0.410023098	0.59982426	0.36631779	7.940205782	7.954278496	9.301632639	9.347568393	9.28302499	7.854430723	8.379764957
ENSG00000089876	DHX32	0.643468041	0.262230813	-0.27731805	0.650030799	0.366149991	9.143983763	8.820493869	7.934808822	9.392580682	9.32220073	8.351275892	8.532824248
ENSG00000183696	UPP1	1.266044689	0.379127697	-0.902218522	0.536069347	0.363826167	8.653385977	8.356994601	4.058998049	9.12771691	9.29713876	7.081831041	7.078537388
ENSG00000171855	IFNB1	5.553656509	0.131910476	-5.191124081	0.203845577	0.362532428	2	2	2	5.720439317	5.67111583	2	2
ENSG00000146592	CREB5	-0.692286799	0.706018071	1.05452867	0.544666992	0.362241871	2	7.431218269	7.502634626	6.351046291	6.11534392	7.634923938	8.175075046
ENSG00000149761	NUDT22	0.02636921	0.969963817	0.33551982	0.594804601	0.361889029	7.790810999	8.255926468	7.098165388	7.748245381	7.90193448	7.898530056	8.332009115
ENSG00000092330	TINF2	-0.083206249	0.919060361	0.443207042	0.54430284	0.360000793	9.886101849	9.030900487	8.325066185	9.099758639	9.17507637	9.452841924	9.843245622
ENSG00000168395	ING5	-0.114428817	0.89152261	0.473846762	0.522984627	0.359417945	8.009475818	7.928237109	6.534109959	7.440576128	7.60187978	8.327124877	7.83304634
ENSG00000186020	ZNF529	-0.20706616	0.7690856	0.566385978	0.372265366	0.359319819	6.257048221	7.051611315	7.285536013	6.887305128	6.55189318	7.724554489	7.184857568
ENSG00000018236	CNTN1	-2.305113648	0.671272366	2.664419918	NA	0.35930627	2	2	2	2.141737246	2	3.878466556	2.632958241
ENSG00000168096	ANKS3	-0.223217395	0.89133285	0.581224057	0.698780079	0.358006662	7.159102577	7.874700259	3.369317972	6.79375511	6.82036165	7.652523812	7.523290521
ENSG00000116016	EPAS1	-0.150314401	0.816128663	0.508155904	0.374026188	0.357841503	11.30078111	11.24195824	12.26789926	11.51181191	11.5558697	12.09526031	12.28222225
ENSG00000198142	SOWAHC	-0.032330226	0.988321837	0.389880862	0.853341364	0.357550636	3.327711234	5.457120953	9.301632639	7.867833045	7.72948195	8.307508685	8.118139752
ENSG00000128606	LRRC17	0.144974069	0.954132822	0.21106439	0.933991771	0.356038459	2	4.93638933	8.763901704	7.474468681	7.37938471	6.231826305	8.154630332
ENSG00000125124	BBS2	0.577133677	0.442359404	-0.221605734	0.78167297	0.355527944	8.444143041	7.847167909	9.439621147	9.302050417	9.29056951	8.645454719	8.347853908
ENSG00000182107	TMEM30B	-4.714951099	0.096084829	5.069828543	0.017532061	0.354877444	2	2	3.369317972	2	2	6.315225883	5.945233978

ENSG00000171574	ZNF584	-0.368117364	0.829692566	0.721958874	0.650570904	0.35384151	4.467311984	5.131603625	8.296287027	6.496695859	6.67805337	7.522436904	7.763868281
ENSG00000214063	TSPAN4	0.665183565	0.367097793	-0.313957455	0.686181755	0.35122611	10.27168932	8.655694736	9.990713521	10.4041951	10.4959054	9.275521747	9.651383746
ENSG00000253305	PCDHGB6	2.673746455	2.54E-11	-2.322627031	1.33E-08	0.351119424	7.975256505	7.189546432	7.342949392	10.1568736	10.2084185	5.4091291	5.250250853
ENSG00000102302	FGD1	-0.270392058	0.845986004	0.621020103	0.629551284	0.350628046	8.609439662	8.702538795	5.156626401	7.867833045	7.8745862	8.802599622	8.697929554
ENSG00000113140	SPARC	-0.065144413	0.945987763	0.414536766	0.640863647	0.349392353	17.20663094	15.68411684	15.37962849	16.25522284	16.2674118	16.88548476	16.58038783
ENSG00000105289	TJP3	-2.312716832	0.660533364	2.660540267	NA	0.347823435	2	2	2	2	2.14022021	3.679182183	3.012034821
ENSG00000158882	TOMM40L	0.589226498	0.391687888	-0.242092853	0.738292369	0.347133645	7.485143553	7.669933291	6.203069917	7.862723323	7.81305436	7.078941352	6.97156716
ENSG00000105085	MED26	0.112380464	0.956619854	0.234703581	0.909725832	0.347084045	2	5.72254323	7.818184403	6.693715713	6.6253296	6.797131715	6.757786576
ENSG00000173473	SMARCC1	0.123426302	0.937032541	0.22223712	0.886082448	0.345663421	6.009025997	10.19877383	10.19940021	9.746817367	9.80557618	9.744173094	9.99470022
ENSG00000198074	AKR1B10	2.407764739	0.033569383	-2.062283764	0.080940119	0.345480975	3.327711234	3.076665209	4.523675391	5.639097847	5.84514933	2.68698273	2.592996859
ENSG00000044090	CUL7	-0.40056868	0.387701261	0.745963881	0.079886981	0.345395201	8.816894989	9.277729677	9.330306409	8.734762645	8.790887	10.15695659	9.592751029
ENSG00000139890	REM2	0.35164789	0.892338755	-0.00634432	0.99833637	0.34530357	2	4.710564154	2	4.17310023	3.33976443	3.904814898	3.128433649
ENSG00000109066	TMEM104	-0.470113089	0.841054398	0.814877255	0.712935639	0.344764166	2	8.376385542	10.15199903	8.483340231	8.46556258	9.678637907	9.817737166
ENSG00000064933	PMS1	-0.331265394	0.554864335	0.675575647	0.184184495	0.344310254	6.96351253	6.389896633	6.43197674	6.351046291	6.23747437	7.405054033	7.108963622
ENSG00000136238	RAC1	0.052881929	0.908151255	0.291207553	0.463927375	0.344089482	11.6325805	11.0102427	11.1169142	11.32636749	11.3369882	11.55492576	11.58427143
ENSG00000220201	ZGLP1	1.112468172	0.610039424	-0.768776294	0.728258838	0.343691879	2	4.710564154	2	4.358765249	4.45712836	3.316666208	2.885414003
ENSG00000078246	TULP3	-0.127027878	0.906452235	0.469862921	0.629414332	0.342835043	7.031698319	8.732943944	9.465702942	8.551612617	8.63070345	9.157900392	9.208682031
ENSG00000008282	SYPL1	0.541407413	0.47627622	-0.199393519	0.804937917	0.342013894	8.444143041	9.733683991	10.16000817	10.15948039	10.1380242	9.493286157	9.324553169
ENSG00000086570	FAT2	-0.873554179	0.719674266	1.214937869	0.5943601	0.34138369	2	4.11352228	2	2.868578001	2.59489879	3.411599105	4.329022995
ENSG00000108395	TRIM37	-0.567127415	0.61090653	0.908329073	0.385615654	0.341201658	7.33131396	8.806266215	10.16797309	8.616799622	8.64695856	10.00197246	10.18816565
ENSG00000164104	HMGB2	-0.031902352	0.961244994	0.372654252	0.527923969	0.3407519	7.096806373	6.666836837	6.203069917	6.739036112	6.60126376	7.244115921	6.853913776
ENSG00000184381	PLA2G6	-0.358225706	0.738128035	0.698703989	0.480246049	0.340478282	6.138360429	4.11352228	5.156626401	5.076005082	4.95174433	5.812021848	6.124122661
ENSG00000134871	COL4A2	-0.950016152	0.009719754	1.288787746	0.000263742	0.338771594	14.59904125	14.8709666	14.86603163	13.80219439	13.8658061	16.3592535	15.7151211
ENSG00000212916	MAP10	0.496611675	0.844549702	-0.161624375	0.950047826	0.3349873	2	5.838937808	2	4.804776789	4.98931816	4.271694079	4.4231929
ENSG00000175697	GPR156	-0.599159954	0.807354269	0.934047778	0.686224112	0.334887824	2	2	4.874587708	3.09461492	3.33976443	4.378961816	4.175132639
ENSG00000198818	SFT2D1	1.190548665	0.277947155	-0.856570415	0.441358531	0.33397825	8.285626337	8.376385542	5.39244166	9.027396512	9.01008467	7.138441638	6.860240429
ENSG00000244274	DBNDD2	0.537577342	0.78255046	-0.20454159	0.919589888	0.333035751	7.096806373	4.710564154	2	6.415275989	6.17362678	5.747972572	5.448021784
ENSG00000134202	GSTM3	0.73469941	0.237601833	-0.401963468	0.535268829	0.332735942	9.590776211	8.297207175	8.941672936	9.75855684	9.77093593	8.419110791	8.824656761
ENSG00000101161	PRPF6	0.030666537	0.978813376	0.298991975	0.78199021	0.329658513	8.75772933	10.32888058	7.896971941	9.354865403	9.41227044	9.531835181	9.760542411
ENSG00000101544	ADNP2	0.093728418	0.957587914	0.235562774	0.893536663	0.329291192	4.00722531	8.848535039	9.301632639	8.693628619	8.54656162	8.540135471	8.955005256
ENSG00000127334	DYRK2	-0.651156	0.47726999	0.979451989	0.255860639	0.328295989	9.689059027	7.60566235	8.325066185	8.153770873	8.16053033	9.838172685	9.706848979
ENSG00000162517	PEF1	-0.376728753	0.761228536	0.70446934	0.544666992	0.327740588	10.19198706	9.656475478	6.958202216	9.055659246	9.10085035	9.936433011	10.34300322
ENSG00000197763	TXNRD3	-0.455317158	0.876694325	0.781094657	0.778128936	0.325777499	2	7.538394143	2	5.753943344	5.43283104	6.649130473	6.879055607
ENSG00000183826	BTBD9	-0.264336653	0.79719998	0.587326095	0.534923556	0.322989442	8.777722034	6.464272742	8.266922072	7.839502934	7.86955761	8.512558892	8.857178832
ENSG00000100422	CERK	-0.980822534	0.159032929	1.302578883	0.049414607	0.321756349	8.107517749	9.386925327	9.735974718	8.192977693	8.31648126	10.64282315	10.39531195
ENSG00000185761	ADAMTSL5	-1.163383471	0.36509383	1.485029319	0.226994232	0.321645848	4.815609471	7.002537973	8.536706852	6.283823264	6.37866593	8.707577112	9.103279088
ENSG00000147400	CETN2	0.191259023	0.681917972	0.129658052	0.783874152	0.320917075	8.39321512	8.004992147	7.896971941	8.327018759	8.27524772	8.037484631	8.418864688
ENSG00000164077	MON1A	-0.391601305	0.798773623	0.710683882	0.621538139	0.319082577	3.327711234	6.78762695	7.600047325	6.436065776	6.22964624	7.473766117	7.322574236
ENSG00000125046	SSUH2	-2.26658333	0.496719668	2.583924385	0.378260987	0.317341055	2	3.076665209	2	2.270786764	2	3.724264246	4.215171317
ENSG00000048342	CC2D2A	-0.526428713	0.434235922	0.84254902	0.17668291	0.316120307	6.257048221	7.35507212	7.451345288	6.61689148	6.60126376	7.669911566	8.169990965
ENSG00000124164	VAPB	-0.450168724	0.386935633	0.765766047	0.112309718	0.315597323	9.913467515	8.94259736	9.31604076	8.956018563	9.03612482	10.13382191	10.2749799
ENSG00000148339	SLC25A25	0.105362775	0.960095276	0.210213871	0.922180661	0.315576646	3.327711234	6.389896633	9.670202709	8.31397419	8.37442884	8.487716929	8.407389648
ENSG00000173890	GPR160	-0.949099384	0.739704558	1.264018438	0.631802652	0.314919054	3.327711234	2	2	2.3892346	2.2680111	3.316666208	2.918126211
ENSG00000127463	EMC1	0.135579942	0.748334322	0.17742303	0.666121968	0.313002972	10.48735708	10.09725346	9.954333071	10.30502636	10.3590869	10.20578905	10.52474633
ENSG00000115840	SLC25A12	0.58092257	0.704833394	-0.268690395	0.865952727	0.312232175	4.467311984	6.602424674	8.677268317	8.04293816	8.01099479	7.027411703	7.34530375
ENSG00000224420	ADM5	0.442104509	0.870799344	-0.130902488	0.962322454	0.311202021	2	3.686303414	2	3.162721474	3.01487888	3.035909414	2.551896972
ENSG00000101811	CSTF2	0.444066547	0.841155419	-0.133016263	0.953164556	0.311050284	5.095959458	8.124485672	2	7.178012736	7.14977896	6.448814696	6.748721609
ENSG00000165264	NDUFB6	0.20267941	0.663582024	0.108311867	0.82058396	0.310991278	7.790810999	7.431218269	7.451345288	7.751013478	7.77322015	7.485815918	7.83304634
ENSG00000169740	ZNF32	0.167444393	0.925360182	0.143165409	0.935870665	0.310609802	8.653385977	7.847167909	3.369317972	7.890605449	7.92877397	7.752026287	8.008487789
ENSG00000143786	CNIH3	-3.753002793	0.531143362	4.063478256	0.463438411	0.310475463	2	2	2	2	2	4.894909044	3.620995423
ENSG00000166394	CYB5R2	-0.788680511	0.679907921	1.098833864	0.546009747	0.310153353	4.00722531	7.928237109	3.369317972	5.753943344	5.77157844	7.54878318	7.592058619
ENSG00000143850	PLEKHA6	-2.472504455	0.206338371	2.779670271	0.12649658	0.307165816	2	5.303523747	2	2.695456494	2.59489879	6.623578417	6.350712758
ENSG00000150768	DLAT	0.342651512	0.696339473	-0.035963831	0.969519452	0.306687681	6.96351253	8.557180776	8.941672936	8.696503411	8.72420319	8.275052313	8.394370181
ENSG00000136699	SMPD4	0.57626911	0.474064658	-0.27033677	0.747340082	0.30593234	8.19931886	8.671479029	9.907528187	9.660380652	9.70753469	8.738579005	8.940648015
ENSG00000125447	GGA3	-0.049967095	0.978749512	0.355575727	0.841817241	0.305608632	9.473095744	7.431218269	4.058998049	8.172475401	8.185025	8.730775702	8.410266976
ENSG00000149532	CPSF7	-0.253588145	0.79810609	0.559056811	0.54005296	0.305468666	7.904282243	10.17682307	9.589475645	9.256998085	9.23691006	9.978712022	10.12848246
ENSG00000204970	PCDHA1	1.31715843	0.63692076	-1.0137313	0.721493405	0.30342713	2	2	3.369317972	3.566290607	2.59489879	2.3070652	2.326754077
ENSG00000137575	SDCBP	0.747351224	0.062366663	-0.444074916	0.286844962	0.303276308	10.23600589	10.99464252	10.56174313	11.41302923	11.3444829	10.19181371	10.18921304
ENSG00000163867	ZMYM6	0.929586919	0.083215069	-0.62762161	0.254347507	0.301965309	6.73718411	6.666836837	7.552162999	7.942383523	7.93600799	6.554528258	6.286392397
ENSG00000120686	UFM1	0.697907139	0.008153101	-0.39596759	0.153721088	0.301939548	9.678463567	9.616264339	9.439621147	10.30502636	10.2501278	9.282760396	9.088984267
ENSG00000001036	FUCA2	0.978976395	0.318498522	-0.677681152	0.497375263	0.301295243	11.12465208	9.849218119	8.585098385	11.17819202	11.1831065	9.328794492	9.705385533
ENSG00000109519	GRPEL1	0.402574787	0.322710742	-0.101501492	0.8207453	0.301073295	8.777722034	8.955547846	8.763901704	9.220514323	9.25341153	8.445151776	8.979912728
ENSG00000115364	MRPL19	0.065861987	0.930416944	0.234392513	0.738292369	0.3002545	8.609439662	7.954278496	9.372276026	8.838609445	8.80148734	9.030579407	8.942636839
ENSG00000129071	MBD4	0.371026554	0.396920108	-0.073248655	0.881261401	0.297777899	7.667647223	8.213429689	8.206335842	8.372666678	8.46722793	8.11499695	7.836259222
ENSG00000154978	VOPP1	0.532657661	0.442135948	-0.235524605	0.745524236	0.297133056	9.533135543	8.732943944	10.23774519	10.14060551	10.1695649	9.313509983	9.46366404
ENSG00000149480	MTA2	0.158961698	0.938309757	0.138005201	0.946447056	0.296966899	4.00722531	8.317412869	10.68897008	9.475255213	9.57865651	9.404033465	9.603161713
ENSG00000110328	GALNT18	-1.279411486	0.644633417	1.576326301	0.556063931	0.296914815	7.709880939	2	2	5.022023783	4.98931816	7.940525532	7.405663765
ENSG00000188735	TMEM120B	-0.212749574	0.747695986	0.509480363	0.398667475	0.29673079	8.39321512	7.393647515	7.552162999	7.617841552	7.65252709	8.57182706	8.077990544
ENSG00000056097	ZFR	0.218235773	0.831805706	0.078155638	0.940405661	0.29639141	8.586954094	10.76012279	10.72180585	10.54003162	10.5157558	10.5244845	10.23780428
ENSG00000164306	PRIMPOL	0.191009417	0.934841508	0.101577588	0.966283503	0.292587005	2	7.051611315	3.369317972	5.85002558	5.77157844	5.853194273	5.59182244
ENSG00000134153	EMC7	0.809839434	0.361201385	-0.518364081	0.568052013	0.291475353	11.14403498	9.532315021	9.212024683	11.05732061	11.0114006	9.635775323	9.781528029
ENSG00000134086	VHL	0.716140297	0.361166693	-0.426450434	0.597996964	0.289689863	9.730679447	7.954278496	9.528916236	9.95800436	9.97493083	8.929133243	8.725336927
ENSG00000117748	RPA2	0.195379655	0.892020352	0.094149931	0.947678709	0.289529586	4.815609471	8.540087354	7.502634626	7.834291698	7.75969386	7.700314972	7.695929227
ENSG00000162437	RAVER2	-2.523347567	0.115204374	2.812791749	0.069547827	0.289444182	7.031698319	7.701025996	2	4.329433326	4.72553021	9.907610449	9.241364212
ENSG00000102401	ARMCX3	-0.222727419	0.589615937	0.511671467	0.171340702	0.288944047	9.751047882	10.01303782	10.37481416	9.918537335	9.77026155	10.65645596	10.49540898
ENSG00000082516	GEMIN5	-0.025150469	0.988931662	0.311747426	0.857059231	0.286596957	3.327711234	7.35507212	8.585098385	7.494428494	7.53039799	7.86621124	7.819040576
ENSG00000147394	ZNF185	-1.44889044	0.361913214	1.734753922	0.256088094	0.285863483	7.218819781	6.602424674	2	5.040242897	5.06165168	8.087943566	8.053353518
ENSG00000164252	AGGF1	0.417207795	0.386972978	-0.133190519	0.797784693	0.284017276	7.940205782	8.029695761	7.225742779	8.18685754	8.20112674	7.586913431	7.715835172
ENSG00000221983	UBA52	0.28194718	0.466152501	0.001097915	0.998296152	0.283045096	10.85979178	10.28338212	10.75391088	10.91431859	10.950196	10.57970149	10.72117
ENSG00000138443	ABI2	-0.114947602	0.914640194	0.397429098	0.686301227	0.282481496	10.57565445	8.955547846	8.434763955	9.523810814	9.49541459	10.08622907	9.949921184
ENSG00000219438	FAM19A5	-5.493632287	0.008781104	5.775082705	0.000612377	0.281450418	2	5.838937808	2	2.141737246	2.14022021	9.791276442	10.16514154
ENSG00000014824	SLC30A9	0.26606654	0.628420441	0.015114582	0.979817504	0.281181122	8.910412251	9.640525262	8.699419242	9.439666599	9.38521851	9.130600759	9.195398998
ENSG00000101412	E2F1	0.262198119	0.895675696	0.01857733	0.992804891	0.280775449	4.00722531	6.311476754	2	5.304221918	5.33407135	4.713032626	5.408276315
ENSG00000173175	ADCY5	-2.573709635	0.040691338	2.853263347	0.012415381	0.279553712	4.467311984	5.131603625	2	2.947924294	2.59489879	6.737909218	7.218077721
ENSG00000090932	DLL3	-0.211479066	0.937710599	0.489182567	0.850325768	0.277703501	2	3.686303414	2	2.947924294	2.59489879	3.215043717	3.012034821
ENSG00000082213	C5orf22	0.262785018	0.52833407	0.01412643	0.974934399	0.276911448	8.138771685	8.376385542	8.677268317	8.615281294	8.72698617	8.544855285	8.296681143
ENSG00000117625	RCOR3	0.497717397	0.489413619	-0.221749375	0.769071028	0.275968023	8.228665467	8.590771778	7.029880454	8.579911316	8.59458893	7.979784698	7.767238791
ENSG00000198089	SFI1	0.45442313	0.8426245	-0.179186615	0.938715125	0.275236515	8.367060731	2	5.59507254	7.487805859	7.37584259	6.911998488	6.716538017
ENSG00000111875	ASF1A	0.816556944	0.606992001	-0.541506321	0.736592879	0.275050623	8.39321512	7.431218269	3.369317972	8.262524677	8.2130862	6.978631953	6.836905794
ENSG00000102172	SMS	0.885170732	0.042971153	-0.610740832	0.173383246	0.2744299	9.304185368	8.540087354	8.742726175	9.780398828	9.77026155	8.135306947	8.432373803
ENSG00000100234	TIMP3	-0.70024055	0.172180866	0.973040539	0.045739026	0.272799989	11.34834729	11.47116739	10.57976717	10.46446525	10.5064723	11.88836944	12.38191627
ENSG00000189007	ADAT2	0.407146053	0.767234656	-0.135750206	0.925711712	0.271395847	3.327711234	6.389896633	4.874587708	5.686138681	5.84514933	5.413723492	5.115701921
ENSG00000164466	SFXN1	-0.192671668	0.84124654	0.463650998	0.599737667	0.27097933	8.075571721	9.792607274	10.18377222	9.406692983	9.40011621	10.05293048	10.06028212
ENSG00000116205	TCEANC2	-0.050928545	0.975272867	0.320654386	0.839520202	0.269725841	8.285626337	5.303523747	4.874587708	7.035434774	6.82556437	7.517109417	7.032593922
ENSG00000066322	ELOVL1	0.169251729	0.837999	0.099893994	0.90483434	0.269145723	10.04301211	10.35843478	8.721235202	9.997003493	10.0656679	9.762974342	10.13806041
ENSG00000184203	PPP1R2	0.066073126	0.93880631	0.202839418	0.805557931	0.268912544	8.797441463	7.099070191	8.460932916	8.36726458	8.31463236	8.589193677	8.353844539
ENSG00000125449	ARMC7	-0.452982853	0.806791315	0.721305296	0.68221623	0.268322443	2	7.315436877	6.958202216	6.237202172	6.0722229	7.544599974	6.969617847
ENSG00000136928	GABBR2	3.152133138	0.484134885	-2.884875253	0.537048771	0.267257884	2	2	2	4.070463721	3.2184422	2.094598369	2
ENSG00000124356	STAMBP	0.016671501	0.97361691	0.249722254	0.582427016	0.266393755	9.371511179	8.639735838	9.014156722	9.059013867	9.04284131	9.154473165	9.399952364
ENSG00000118181	RPS25	-0.065555729	0.96006048	0.331674606	0.793834795	0.266118877	11.41687936	9.067150531	8.631919287	10.1868266	10.1923984	10.58976003	10.58153023
ENSG00000013583	HEBP1	-0.299050976	0.636158103	0.564339135	0.336343976	0.265288159	9.667789716	8.487557258	8.960138144	8.825537758	8.81593678	9.513492253	9.826379958
ENSG00000161542	PRPSAP1	-0.617934684	0.248274186	0.883129936	0.080480446	0.265195253	8.910412251	7.928237109	8.65477198	7.908072101	7.96694292	9.594132343	9.230009473
ENSG00000120253	NUP43	-0.226177785	0.761391811	0.490744732	0.475879402	0.264566947	7.33131396	7.60566235	8.561105505	7.617841552	7.78928606	8.670625574	8.096865967
ENSG00000166848	TERF2IP	-0.04866475	0.968106553	0.313151181	0.787590496	0.264486431	11.12854954	9.799806309	8.236946975	10.1096668	10.1127112	10.58772851	10.34582388
ENSG00000127328	RAB3IP	-1.091698095	0.181178512	1.355327483	0.079746219	0.263629388	6.653098118	4.93638933	6.718950348	5.273747807	5.2591458	7.593002849	7.578053054
ENSG00000061794	MRPS35	0.402569088	0.453915688	-0.139826853	0.80714548	0.262742235	7.867441304	8.655694736	7.818184403	8.667494409	8.47883193	8.060373167	8.020777347
ENSG00000135045	C9orf40	-0.513069073	0.77088407	0.77264167	0.647304758	0.259572597	6.816637977	6.140560021	2	5.501219927	5.48637102	7.168655895	6.007361012

ENSG00000120341	SEC16B	2.531520275	0.160878275	-2.272819584	0.220299195	0.258700691	2	4.11352228	2	5.210797711	4.85329164	2.384001718	2.277179783
ENSG00000101557	USP14	0.19342017	0.749436229	0.064542482	0.919580542	0.257962652	10.29268328	9.170736405	9.647595056	9.966365455	9.9684779	9.799418565	9.877696666
ENSG00000107819	SFXN3	-0.137527033	0.89442148	0.394948587	0.677551443	0.257421554	8.609439662	9.225224669	10.63827116	9.60637697	9.61666844	9.84030732	10.39094874
ENSG00000132842	AP3B1	0.459559235	0.346404872	-0.202249687	0.694809381	0.257309547	9.262228249	9.648522413	8.825624954	9.730780958	9.760787	8.878514511	9.270949935
ENSG00000026559	KCNG1	0.006543717	0.997264364	0.250432588	0.90231922	0.256976305	7.53297911	7.431218269	2	6.892328675	6.95464102	7.754744914	6.131115787
ENSG00000103496	STX4	0.621890845	0.6972049	-0.365387503	0.82326635	0.256503342	9.699577239	6.728495673	5.59507254	8.943972814	9.00436103	7.842553218	8.139104389
ENSG00000169764	UGP2	-0.040407387	0.969622978	0.295513021	0.768207195	0.255105634	8.87372952	9.386925327	11.01019887	10.04403298	9.9912274	10.33841489	10.36671232
ENSG00000137522	RNF121	-0.076870303	0.945987763	0.331845763	0.758720739	0.25497546	9.829762239	7.954278496	7.552162999	8.705093576	8.75724985	9.203056613	9.067272902
ENSG00000124104	SNX21	0.199529729	0.908645221	0.054798054	0.974300795	0.254327784	9.344956677	7.467835385	4.523675391	8.385193462	8.29975519	8.00283742	8.373881215
ENSG00000197576	HOXA4	5.444097041	0.152989085	-5.19112223	0.214752155	0.252974811	2	2	2	5.578054239	5.6121243	2	2
ENSG00000130749	ZC3H4	0.104946368	0.932700573	0.148008473	0.90481909	0.252954841	6.257048221	8.213429689	9.272377397	8.468280417	8.47387021	8.598808264	8.423144406
ENSG00000131584	ACAP3	-0.49295913	0.704833394	0.744710054	0.546275271	0.251750925	9.521325812	8.487557258	5.930865648	8.054188409	8.14812525	9.274574892	9.378441538
ENSG00000158092	NCK1	0.643947459	0.09566079	-0.392486426	0.324893899	0.251461032	8.107517749	8.433041974	8.076950877	8.869506572	8.84824797	8.006643981	7.640647
ENSG00000144840	RABL3	0.346167148	0.815788738	-0.095228085	0.950246079	0.250939062	7.218819781	4.710564154	8.65477198	7.920420067	7.92635454	7.560224843	7.417152486
ENSG00000144115	THNSL2	0.19459412	0.853531206	0.055877649	0.958419113	0.250471768	6.009025997	7.503546079	7.896971941	7.471115032	7.57741621	7.856119555	6.695462882
ENSG00000126107	HECTD3	-0.12059712	0.946341641	0.370318808	0.83342673	0.249721687	4.00722531	9.055168103	9.577565424	8.634896	8.67172529	9.117986989	9.162073123
ENSG00000280618		0.295294585	0.789557312	-0.047576572	0.967612474	0.247718013	7.709880939	9.916994052	10.36791565	9.986471488	10.0044775	9.504367208	9.789837542
ENSG00000086065	CHMP5	1.274788156	0.059166153	-1.027419376	0.131477233	0.24736878	10.06758145	8.395519306	9.465702942	10.75935206	10.6994819	8.431623835	8.449958388
ENSG00000119686	FLVCR2	-0.705082321	0.770289193	0.951386928	0.682959333	0.246304607	2	5.595927442	2	3.515101962	3.94787299	5.020706951	5.12973572
ENSG00000117643	MAN1C1	-3.073944738	0.000984598	3.315699931	5.62E-05	0.241755192	5.095959458	4.710564154	5.156626401	3.227757282	2.38539787	7.990588491	8.256474583
ENSG00000132313	MRPL35	-0.048694147	0.975624098	0.290292955	0.850843239	0.241598808	8.963744667	8.395519306	4.523675391	8.098332103	8.13141746	8.440093652	8.459018364
ENSG00000057252	SOAT1	1.217252998	0.120812437	-0.977811135	0.215617166	0.239441864	9.678463567	7.928237109	9.809100877	10.55277887	10.568298	8.513629334	8.230662576
ENSG00000100078	PLA2G3	-3.753003693	0.531143362	3.991383105	0.472178974	0.238379412	2	2	2	2	2	3.663836244	4.793076545
ENSG00000117360	PRPF3	0.395945225	0.446038292	-0.157611675	0.774520328	0.23833355	8.631580145	7.701025996	8.076950877	8.516254096	8.62474679	8.040458475	8.009436866
ENSG00000204174	NPY4R	-2.312727365	0.665128529	2.549864217	NA	0.237136852	2	2	2	2	2.14022021	3.082820997	3.518055793
ENSG00000001497	LAS1L	-0.139063308	0.865194998	0.375201627	0.614795534	0.236138319	7.031698319	8.451443573	7.502634626	7.739909082	7.57741621	7.919680595	8.377561365
ENSG00000197959	DNM3	0.88044281	0.690529475	-0.644857767	0.773343718	0.235585043	2	4.442710129	2	3.997744386	3.98554648	3.1504466	2.885414003
ENSG00000165195	PIGA	0.223188316	0.885649929	0.011876821	0.993931149	0.235065137	4.00722531	7.954278496	6.43197674	7.048994596	7.14145882	6.768721236	7.004308743
ENSG00000174109	C16orf91	-0.154432088	0.895265695	0.386779044	0.721563265	0.232346957	6.009025997	4.93638933	7.342949392	6.252910529	6.26070706	6.697032326	6.847559257
ENSG00000160991	ORAI2	-0.365112075	0.646065476	0.595823027	0.42719519	0.230710953	8.257427002	9.514922632	9.990713521	9.085573664	9.0282492	9.842864719	10.1649286
ENSG00000185825	BCAP31	0.73125249	0.08771873	-0.501155301	0.25275751	0.230097189	10.28571918	10.36815316	10.96062172	11.28423127	11.314036	9.882764149	10.23557543
ENSG00000167695	FAM57A	-0.187076095	0.851584865	0.416758582	0.653326175	0.229682487	7.096806373	8.505280662	9.330306409	8.392303091	8.38503958	8.901185819	9.067272902
ENSG00000122550	KLHL7	0.243521005	0.634071993	-0.014690366	0.978701383	0.22883064	8.340423424	7.979858146	8.721235202	8.553199415	8.66883353	8.541185654	8.145162126
ENSG00000213865	C8orf44	0.231672835	0.895723155	-0.005176996	0.997969492	0.22649584	2	4.11352228	5.772716771	4.887609717	4.79082474	4.727866721	4.543391831
ENSG00000123989	CHPF	-0.383315135	0.579274794	0.608588335	0.350450714	0.2252732	12.67495574	11.56710608	11.59570544	11.64594186	11.6755879	12.93232161	12.30336808
ENSG00000125454	SLC25A19	-0.226539289	0.895062834	0.447909933	0.781250208	0.221370644	4.815609471	6.04685525	2	4.927308402	4.811949	5.610655473	5.313094696
ENSG00000110321	EIF4G2	0.093160006	0.923175097	0.128043827	0.893295691	0.221203832	11.54488817	13.1166165	13.63150382	13.10089132	13.0834501	13.092274	13.16103071
ENSG00000105401	CDC37	0.159106845	0.660816284	0.06073039	0.873992896	0.219837236	10.82178169	10.58784379	10.9788475	10.9293654	10.9941769	10.69565518	11.01473643
ENSG00000281300		0.272858106	0.74150751	-0.053355396	0.951751695	0.219502709	8.855032352	10.1878402	8.742726175	9.677214349	9.71736428	9.08740002	9.611311483
ENSG00000189144	ZNF573	-0.256129893	0.903607792	0.475063712	0.812849666	0.218933819	2	3.686303414	6.203069917	4.984880435	4.2865223	5.665620077	4.853519801
ENSG00000088205	DDX18	0.255611721	0.620673635	-0.039254371	0.944101725	0.216357349	8.855032352	9.470501606	8.585098385	9.288777073	9.27068036	8.985384832	8.98860193
ENSG00000196504	PRPF40A	0.600319555	0.328511143	-0.384220452	0.541584801	0.216099103	9.189468652	9.540933215	10.43545992	10.42427719	10.4106283	9.472517815	9.400675953
ENSG00000153187	HNRNPU	0.372226925	0.423319756	-0.156146007	0.74998849	0.216080918	11.99688029	11.23931304	11.37961153	11.95574745	11.9417163	11.31077711	11.52345097
ENSG00000084073	ZMPSTE24	0.480550405	0.40310156	-0.264608533	0.657720059	0.215941872	8.674867122	9.055168103	9.757248847	9.730780958	9.68767229	8.797335126	9.122267395
ENSG00000179364	PACS2	-0.496102049	0.644633417	0.710089012	0.488684721	0.213986963	10.18451821	8.433041974	7.734844356	8.610716699	8.73115061	9.873413524	9.86726424
ENSG00000119699	TGFB3	-1.349845927	0.395836393	1.56355649	0.307397922	0.213710562	4.467311984	7.189546432	9.465702942	6.846474326	6.88657603	10.05477007	9.327600363
ENSG00000106723	SPIN1	0.008439274	0.993765388	0.203721466	0.833507886	0.21216074	8.107517749	10.27823701	9.798878489	9.704603278	9.61215953	9.994516174	9.697162691
ENSG00000187522	HSPA14	0.276097347	0.899484135	-0.064831352	0.975799274	0.211265996	9.048471759	8.557180776	2	8.509731121	8.52753255	8.319187939	8.030160124
ENSG00000157445	CACNA2D3	1.001545229	0.680089245	-0.791204884	0.744740559	0.210340345	2	2	4.874587708	4.329433326	4.3456612	3.411599105	2.746601469
ENSG00000119402	FBXW2	-0.390109076	0.612785008	0.600269925	0.410154943	0.210160849	10.29961392	8.655694736	10.01740919	9.37922384	9.4657318	10.49389479	10.31947205
ENSG00000242247	ARFGAP3	0.47797379	0.307672642	-0.268193506	0.580066685	0.209780283	9.922475255	10.07961649	9.227351203	10.28320062	10.2506114	9.587285313	9.457765716
ENSG00000091483	FH	-0.649045267	0.536848085	0.857196104	0.394793944	0.208150837	9.657036304	7.467835385	7.646393253	8.015574133	7.94798467	9.291523922	9.635180748
ENSG00000111860	CEP85L	0.391709223	0.813408453	-0.183771725	0.914029999	0.207937498	8.075571721	6.844429755	3.369317972	7.437142666	7.4074129	7.17948788	6.457498602
ENSG00000163539	CLASP2	-0.133490794	0.905447484	0.339418097	0.744205289	0.205927303	6.89194353	8.762721501	9.439621147	8.596935617	8.53707846	9.200400028	8.850309106
ENSG00000171314	PGAM1	1.273560737	0.003434646	-1.067809076	0.015942135	0.205751661	11.8768942	11.30403016	11.2764554	12.76859676	12.8021511	10.09805229	10.72853726
ENSG00000182568	SATB1	0.920447446	0.392191303	-0.715587924	0.507672408	0.204859521	6.653098118	7.60566235	4.874587708	7.691737974	7.64666005	6.370627179	5.745545708
ENSG00000276380	UBE2NL	-3.753004132	0.531143362	3.956244847	0.476306746	0.203240715	2	2	2	2	2	4.155805465	4.445805334
ENSG00000102362	SYTL4	0.760695124	0.525220439	-0.558049914	0.643328101	0.20264521	3.327711234	5.838937808	6.322058544	6.12217689	6.51372389	5.294231737	4.911532223
ENSG00000051620	HEBP2	-0.037507482	0.954566832	0.239962107	0.69597295	0.202454624	8.418903792	8.395519306	9.372276026	8.740345368	8.78555748	9.177281635	8.884335219
ENSG00000196678	ERI2	0.143281824	0.941473373	0.058767787	0.975626005	0.202049611	5.095959458	7.051611315	2	6.034011955	5.93454154	6.043083161	5.75463059
ENSG00000153037	SRP19	0.732947727	0.051734578	-0.532399491	0.16559668	0.200548236	7.750913381	8.276714465	7.858115942	8.756965077	8.67172529	7.511762185	7.424286753
ENSG00000133597	ADCK2	-1.357163776	0.474361527	1.557209677	0.393866241	0.200045901	2	6.228547981	8.236946975	5.797438367	5.58783529	8.596789458	8.419578856
ENSG00000211584	SLC48A1	-1.063214715	0.164591992	1.262818251	0.085639381	0.199603537	6.73718411	8.376385542	7.225742779	6.61689148	6.57678965	9.08127623	8.632905293
ENSG00000197563	PIGN	-0.120212127	0.802441015	0.31880469	0.460415275	0.198592563	7.667647223	7.819099893	7.858115942	7.737119576	7.59275449	7.86872317	8.296681143
ENSG00000116171	SCP2	-0.084683343	0.893473749	0.281711832	0.622327372	0.197028489	9.635285994	9.04308532	10.13584611	9.59332559	9.57557184	9.835820934	10.0543164
ENSG00000149269	PAK1	-0.127222321	0.943520433	0.319518619	0.855882497	0.192296298	8.313285031	8.791896851	3.369317972	7.815902688	7.95036812	8.438967212	8.201061123
ENSG00000172733	PURG	-3.003890167	0.115773136	3.193670324	0.05188662	0.189780157	2	4.442710129	2	2.270786764	2.2680111	6.18690462	5.799216178
ENSG00000169733	RFNG	-0.367144045	0.551369059	0.555981394	0.340755867	0.188837349	9.521325812	8.317412869	9.099922433	8.679168098	8.70598173	9.745540467	9.458460889
ENSG00000198933	TBKBP1	-0.216249597	0.892276535	0.404160465	0.789905257	0.187910869	4.815609471	7.315436877	9.049077186	7.620871236	7.74878077	8.464101787	8.107542072
ENSG00000183691	NOG	0.738148119	0.771089389	-0.550454572	0.83120048	0.187693547	2	2	5.156626401	4.443344104	4.3456612	2.962551659	3.824516949
ENSG00000131669	NINJ1	1.31380199	0.191634596	-1.126147825	0.262168393	0.187654165	11.11289609	8.806266215	9.014156722	11.32961175	11.3882124	9.01441148	8.838074258
ENSG00000129472	RAB2B	0.253788403	0.680957539	-0.066311077	0.919852079	0.187477326	8.998234871	7.954278496	8.677268317	8.851563757	8.85080188	8.752700801	8.276323713
ENSG00000184182	UBE2F	0.395241817	0.634203222	-0.208270778	0.808778437	0.186971039	8.716893051	7.979858146	6.882775709	8.399377854	8.46722793	7.636890116	8.016062975
ENSG00000140459	CYP11A1	1.053106589	0.435021415	-0.867161651	0.519967781	0.185944938	5.70932728	5.131603625	2	5.860316854	5.92487824	4.350488971	4.077147165
ENSG00000145536	ADAMTS16	1.504784136	NA	-1.318858121	NA	0.185926015	2	2	2	3.289987342	2	2.267001569	2
ENSG00000168802	CHTF8	-0.113819961	0.913307146	0.298836189	0.760366765	0.185016228	10.69610297	8.540087354	9.272377397	9.635130631	9.70753469	10.06100716	10.10403354
ENSG00000169446	MMGT1	-0.22720727	0.4795951	0.412001116	0.161146297	0.184793846	8.609439662	8.395519306	8.561105505	8.327018759	8.26379462	8.902820827	8.953526633
ENSG00000175470	PPP2R2D	-0.109462885	0.918662967	0.294045875	0.771060512	0.18458299	9.304185368	8.147241016	6.958202216	8.276040626	8.37974396	8.835930569	8.611187869
ENSG00000198453	ZNF568	-0.226029368	0.855033687	0.410526425	0.723926237	0.184497057	6.96351253	6.728495673	4.058998049	6.237202172	6.08095082	6.848938847	6.683620062
ENSG00000156515	HK1	-0.271967927	0.648950726	0.456326486	0.417520898	0.184358559	10.86913986	10.66871049	11.59570544	10.77948848	10.8775392	11.24446364	11.8127295
ENSG00000104884	ERCC2	-0.986691777	0.224578964	1.170487408	0.134337451	0.183795631	8.910412251	6.951736442	8.608698767	7.464384256	7.32898565	9.455350953	9.598438895
ENSG00000167625	ZNF526	-0.118078139	0.942994458	0.300557579	0.852305768	0.18247944	4.00722531	8.147241016	8.721235202	7.739909082	7.84670947	8.350677018	8.045972122
ENSG00000080839	RBL1	0.234662717	0.810928754	-0.053752286	0.957763507	0.180910431	4.467311984	5.457120953	6.322058544	5.870535235	5.74985018	5.703636713	5.367404784
ENSG00000167987	VPS37C	-0.498392697	0.735274416	0.679163131	0.633185132	0.180770434	5.532353208	8.702538795	9.868959282	8.401141138	8.33848526	9.382222556	9.678488127
ENSG00000131732	ZCCHC9	0.307819285	0.880694572	-0.127610649	0.950246079	0.180208636	8.19931886	7.761270542	2	7.72026832	7.73225478	7.485815918	7.087553083
ENSG00000179010	MRFAP1	-0.179507098	0.750738031	0.358723106	0.495887113	0.179216007	12.20129367	11.34889202	11.55983287	11.56959791	11.5718246	11.84150468	12.33347507
ENSG00000116750	UCHL5	0.077581319	0.943613626	0.101066463	0.927960061	0.178647782	8.39321512	6.844429755	6.203069917	7.423326516	7.61696151	7.754744914	7.302581437
ENSG00000174500	GCSAM	-3.753004463	0.531143362	3.929715656	0.479315748	0.176711193	2	2	2	2	2	4.065262523	4.479073019
ENSG00000174807	CD248	0.040614105	0.95872661	0.136045666	0.860323788	0.176659771	11.90670479	11.14918383	11.90777898	11.73793909	11.7341661	11.03547504	12.34136406
ENSG00000139826	ABHD13	0.314585124	0.882991782	-0.138449871	0.948273373	0.176135253	8.716893051	7.538394143	2	8.004017475	7.95274764	7.69371876	7.354296226
ENSG00000038358	EDC4	0.121530913	0.760985399	0.054255525	0.895713612	0.175786438	8.816894989	8.671479029	8.296287027	8.71221318	8.75588792	8.715971642	8.617426278
ENSG00000125912	NCLN	0.318492111	0.765322807	-0.142750166	0.897608659	0.175741945	10.9638027	9.523645035	8.511888463	10.26201025	10.3936516	9.752131227	9.978555258
ENSG00000181638	ZFP41	0.652521426	0.628255701	-0.477551362	0.725841069	0.174970065	5.095959458	5.595927442	8.076950877	7.341208691	7.64666005	6.503717356	6.308152703
ENSG00000175110	MRPS22	0.465645869	0.671105744	-0.291276648	0.794825276	0.174369222	8.75772933	7.467835385	6.073378139	8.219202381	8.33118782	7.54878318	7.515294811
ENSG00000143457	GOLPH3L	0.089045969	0.945455549	0.084909936	0.948481065	0.173955905	9.081016759	7.638155693	5.930865648	8.205140657	8.09094333	8.292619498	7.980689421
ENSG00000114626	ABTB1	-0.566277698	0.442942747	0.740038173	0.291119632	0.173760475	8.228665467	7.538394143	9.116480286	7.862723323	7.88459112	9.284329221	9.035479014
ENSG00000132676	DAP3	0.696580944	0.071684427	-0.526069333	0.179352679	0.170511611	8.564112515	9.148351514	8.960138144	9.626112287	9.58787113	8.2308261	8.541379994
ENSG00000126067	PSMB2	0.284453727	0.590007772	-0.114039899	0.837844551	0.170413828	10.61508061	9.763446433	10.40882047	10.57520961	10.5990046	9.95546434	10.39149486
ENSG00000143183	TMCO1	0.198478225	0.580892944	-0.028086121	0.943162724	0.170392105	9.848787621	9.591588447	10.03493575	10.10264028	9.95489142	9.713525389	9.891147007
ENSG00000197614	MFAP5	-6.293764667	0.009458344	6.464054075	0.000144114	0.170289408	2	2	4.523675391	2	2	8.858854473	9.204544205
ENSG00000185800	DMWD	-0.232771492	0.495143159	0.402562575	0.20216279	0.169791083	9.509418609	9.328390744	9.681375057	9.255049401	9.30181283	10.02879997	9.781805785
ENSG00000198919	DZIP3	-0.66365869	0.596379651	0.833434142	0.487153954	0.169775452	4.00722531	7.051611315	5.59507254	5.527288686	5.36298494	6.987904673	6.711881188
ENSG00000119414	PPP6C	-0.132259033	0.839051597	0.301094124	0.616273347	0.168835091	8.609439662	9.396454291	9.767769415	9.134092596	9.26781654	9.471967222	9.778469175
ENSG00000166913	YWHAB	0.029253429	0.932390851	0.137995077	0.664743846	0.167248506	11.65707219	11.51118914	11.56587392	11.60502731	11.6113331	11.51539471	11.89353851
ENSG00000244734	HBB	-3.753004582	0.531143362	3.920236544	0.480467544	0.167231962	2	2	2	2	2	3.517841605	4.77533149
ENSG00000166477	LEO1	0.627154582	0.768745599	-0.461226456	0.831750977	0.165928126	9.204317094	7.315436877	2	8.610716699	8.57000035	7.543552275	7.493753702
ENSG00000174574	AKIRIN1	0.248849046	0.660813516	-0.085059258	0.887886595	0.163789788	10.51143645	9.479495904	9.888372618	10.30502636	10.2316302	10.00844152	9.859916395

ENSG00000035664	DAPK2	0.146921773	0.933881218	0.016239749	0.992804891	0.163161522	2	3.686303414	4.058998049	3.663521627	3.50452879	3.393105702	3.600986722
ENSG00000164219	PGGT1B	-0.38930468	0.770303179	0.552167114	0.668751914	0.162862434	4.467311984	7.638155693	8.007629717	6.960881212	6.9210099	7.906701615	7.811442311
ENSG00000160256	FAM207A	-0.25706012	0.869968338	0.418807778	0.779317373	0.161747657	7.096806373	7.572420273	3.369317972	6.610808945	6.53928196	7.030406362	7.402777233
ENSG00000144061	NPHP1	-0.301402955	0.855036819	0.462054644	0.767357706	0.160651688	2	4.442710129	5.59507254	4.329433326	4.40247105	4.749835737	5.230851202
ENSG00000189266	PNRC2	1.061078172	2.12E-05	-0.901125083	0.000363392	0.15995309	9.344956677	9.079034257	9.212024683	10.28128703	10.2559202	8.268091966	8.370191653
ENSG00000167685	ZNF444	-0.415774581	0.445961619	0.575678697	0.263479222	0.159904116	8.75772933	7.73146271	8.266922072	7.857595438	7.93118934	8.917048655	8.830038743
ENSG00000122481	RWDD3	1.279523504	0.09807061	-1.122201444	0.147783571	0.157322061	5.095959458	5.457120953	4.523675391	6.276156955	6.28357155	4.369533167	3.924507493
ENSG00000171475	WIPF2	-0.185978238	0.756699054	0.340746183	0.54493451	0.154767944	9.895281535	8.955547846	9.990713521	9.474417988	9.51406378	9.924409668	10.10714012
ENSG00000122359	ANXA11	-0.845036612	0.107655331	0.999517029	0.048821317	0.154480417	10.71696191	9.672251278	10.23774519	9.425008328	9.43031191	11.05011945	11.45403965
ENSG00000168936	TMEM129	-0.497495127	0.418653107	0.651530748	0.266310577	0.154035621	10.05943807	9.102512015	8.960138144	8.976268302	8.95536429	10.2523815	9.948684752
ENSG00000164338	UTP15	0.323112187	0.8692818	-0.16947804	0.932012683	0.153634146	2	7.099070191	7.777116083	7.136275664	7.30311013	6.768721236	6.721179863
ENSG00000196267	ZNF836	0.501921846	0.584830919	-0.350950647	0.705751145	0.150971199	4.467311984	5.72254323	6.203069917	6.024891728	6.16544343	5.565950232	4.998228167
ENSG00000100285	NEFH	0.954626963	0.638803946	-0.80423798	0.692520331	0.150388983	3.327711234	2	5.59507254	5.178261742	5.13053105	4.177580368	3.100202476
ENSG00000180329	CCDC43	0.173580732	0.890295464	-0.023258728	0.9854015	0.150322004	8.87372952	7.189546432	6.073378139	8.015574133	7.9809999	7.763770178	7.844792088
ENSG00000138744	NAAA	-0.093691901	0.895265695	0.243558866	0.709981742	0.149866965	6.89194353	7.572420273	6.629488387	6.956091174	7.06873058	7.57363085	7.063994051
ENSG00000134453	RBM17	0.263449376	0.761694883	-0.113682288	0.900290106	0.149767088	9.262228249	8.414402626	7.342949392	8.796356221	8.80807321	8.407640344	8.449958388
ENSG00000142544	CTU1	-0.429305922	0.671131573	0.578706802	0.546392953	0.14940088	4.467311984	5.946638857	6.534109959	5.527288686	5.43283104	6.723178551	6.041166521
ENSG00000119421	NDUFA8	-0.367486831	0.52974812	0.516355792	0.350276926	0.148868961	8.674867122	8.395519306	7.552162999	7.885575888	7.95749494	8.687797808	8.894645365
ENSG00000171421	MRPL36	-0.046960713	0.943613626	0.195658162	0.756508981	0.148697449	8.816894989	7.790474954	7.934808822	8.151677543	8.2561084	8.296978073	8.578306014
ENSG00000160360	GPSM1	-0.309862264	0.687785088	0.458019465	0.531674005	0.148157201	10.22876205	9.018611864	8.784770914	9.242318391	9.12003262	9.849450427	10.03696805
ENSG00000148019	CEP78	-1.324193242	0.432204028	1.471684378	0.365364036	0.147491136	7.384433967	7.315436877	2	5.662809983	5.40529704	8.175088797	8.273956186
ENSG00000162600	OMA1	-0.203073784	0.909988721	0.350531029	0.837543189	0.147457245	2	6.311476754	6.534109959	5.818703821	5.55061739	6.453284622	5.913137508
ENSG00000165983	PTER	-0.243882774	0.897905614	0.391057722	0.830862053	0.147174948	6.816637977	5.457120953	2	5.242616075	5.74985018	6.495072127	5.576534358
ENSG00000145907	G3BP1	-0.100208559	0.903052048	0.247307787	0.746005167	0.147099228	9.509418609	10.25223258	11.0793996	10.3559173	10.2835937	10.4654111	10.84343131
ENSG00000112167	SAYSD1	-0.04034197	0.966682851	0.187381742	0.841697129	0.147039773	6.257048221	6.04685525	7.734844356	6.841288255	6.83074839	7.125896536	6.983208048
ENSG00000116729	WLS	0.684697395	0.309576645	-0.539594781	0.42570287	0.145102614	10.78274301	9.328390744	9.888372618	10.82025579	10.7937466	9.4949131	9.673706637
ENSG00000100591	AHSA1	0.218997933	0.711659309	-0.073956398	0.906192676	0.145041536	9.858207046	8.806266215	9.725218844	9.76541805	9.72782219	9.390112523	9.517693638
ENSG00000167657	DAPK3	-0.5182187	0.702916163	0.662815882	0.613365058	0.144597182	9.678463567	6.666836837	10.50627919	9.129845271	9.09439916	10.1379928	10.42282144
ENSG00000165092	ALDH1A1	-2.312736954	0.66900125	2.456900924	NA	0.14416397	2	2	2	2	2.14022021	3.336159116	3.183290849
ENSG00000164197	RNF180	-1.638219227	0.477431438	1.781086424	0.420543923	0.142867197	2	5.595927442	2	3.462030052	2.77779907	6.101204172	5.492143571
ENSG00000148335	NTMT1	0.097148178	0.945043724	0.045199248	0.974393699	0.142347426	8.836089686	7.638155693	5.156626401	7.920420067	7.96929529	7.780764172	7.998962529
ENSG00000181924	COA4	-0.112712638	0.871671212	0.254522634	0.693056959	0.141809996	8.777722034	7.819099893	8.206335842	8.166267464	8.24450173	8.166267073	8.88330014
ENSG00000138029	HADHB	0.842504196	0.326142247	-0.700704906	0.415037148	0.14179929	10.79750658	9.821190352	8.65477198	10.81728741	10.8765996	9.287148813	9.332538367
ENSG00000179151	EDC3	-0.214651712	0.887194753	0.356447452	0.804092949	0.14179574	8.39321512	9.664384941	5.59507254	8.429064458	8.43184521	8.978035992	9.013887033
ENSG00000141522	ARHGDIA	-0.048854471	0.919059559	0.189998088	0.668446136	0.141143616	11.63805915	11.38763081	11.44653121	11.40843409	11.4820112	11.30553333	11.98394814
ENSG00000104964	AES	0.606176984	0.113076042	-0.465694334	0.228436335	0.14048265	11.20436621	10.98835491	11.65641302	11.86666856	11.9609044	10.83416038	10.85443307
ENSG00000134291	TMEM106C	-1.051733303	0.606090823	1.191434973	0.546604763	0.13970167	9.317904228	8.101365653	2	7.144720234	7.29937545	9.472517815	9.399590433
ENSG00000174446	SNAPC5	-0.259148328	0.751436954	0.398086351	0.609008368	0.138938023	7.53297911	6.311476754	6.073378139	6.483440992	6.58294721	7.20223931	7.133547492
ENSG00000165259	HDX	0.992676878	0.554602856	-0.853784441	0.611576383	0.138892437	7.159102577	5.838937808	2	6.998639523	7.0774796	5.233158834	5.367404784
ENSG00000104325	DECR1	1.50997339	0.008952796	-1.371345329	0.018674882	0.13862806	7.709880939	8.590771778	7.342949392	9.511620236	9.46072474	6.484192226	6.808832901
ENSG00000156831	NSMCE2	-0.014728869	0.988861116	0.152158107	0.882006656	0.137429238	7.27616306	7.638155693	5.39244166	6.975156635	7.10768997	7.183528998	7.226264598
ENSG00000196268	ZNF493	0.135484548	0.912378494	0.001261307	0.999263508	0.136745855	6.366709671	5.131603625	4.874587708	5.870535235	5.5755357	6.112552588	4.819291477
ENSG00000169609	C15orf40	-0.480584539	0.483180449	0.615096175	0.345033356	0.134511636	7.384433967	6.389896633	7.029880454	6.693715713	6.31350454	7.849352333	7.242500282
ENSG00000183908	LRRC55	-3.753005002	0.531143362	3.886550564	0.484010617	0.133545562	2	2	2	2	2	4.301748724	4.201948172
ENSG00000123595	RAB9A	0.58969212	0.665129218	-0.456633599	0.737989924	0.133058521	9.410448395	7.274681896	5.930865648	8.758341449	8.84952549	7.672789259	7.864857644
ENSG00000182150	ERCC6L2	0.270108633	0.78979414	-0.139155702	0.893479206	0.130952931	6.563806279	7.503546079	8.763901704	8.190940526	8.10812366	7.580798201	7.897008641
ENSG00000138031	ADCY3	-0.38226598	0.832844524	0.511157275	0.768563703	0.128891295	9.957954117	7.232742069	4.874587708	8.227176542	8.240612	9.206039434	9.025192723
ENSG00000186283	TOR3A	-0.363589973	0.654620374	0.4922311	0.527634025	0.128641127	8.16936288	7.819099893	6.43197674	7.366699513	7.21466714	8.300083299	7.941446131
ENSG00000155755	TMEM237	-0.089267388	0.898841046	0.215086404	0.741726039	0.125819016	8.777722034	8.655694736	8.042706615	8.447952961	8.43184521	8.200570331	9.133628617
ENSG00000054654	SYNE2	-0.661766456	0.431378597	0.786675412	0.326487945	0.124908956	6.96351253	5.838937808	6.322058541	6.229283386	5.22805098	7.380620955	6.983208048
ENSG00000159267	HLCS	-0.69383182	0.690326721	0.818105889	0.62915305	0.124274069	4.00722531	7.572420273	9.553445883	7.700356475	7.56502709	9.155502188	9.098381203
ENSG00000105221	AKT2	-0.213532524	0.686696702	0.337675408	0.498871333	0.124142884	10.56418906	9.785372135	10.63252637	10.13213675	10.1868502	10.57445347	10.83274721
ENSG00000182983	ZNF662	0.067634408	0.966177728	0.05519012	0.972248923	0.122824527	4.00722531	3.686303414	6.534109959	5.258265913	5.48637102	5.536762811	5.171036168
ENSG00000134917	ADAMTS8	-2.312742802	0.671489931	2.434718438	NA	0.121975636	2	2	2	2	2.14022021	3.011868911	3.45257121
ENSG00000184209	SNRNP35	-0.309438561	0.885805368	0.430852016	0.835405544	0.121413456	8.444143041	6.899080544	2	7.044488801	6.94511186	7.093332266	8.15205425
ENSG00000140632	GLYR1	0.172051055	0.87178855	-0.051638902	0.962322454	0.120412153	8.444143041	9.170736405	10.67226762	9.900574544	9.90906469	9.601450544	9.758568089
ENSG00000133110	POSTN	1.124764394	0.189674145	-1.006611135	0.237882611	0.118153258	12.82540395	12.83762542	12.94228516	13.99473163	13.9927475	12.58710778	10.34375594
ENSG00000139800	ZIC5	-5.650660466	0.021326273	5.768595101	0.000779484	0.117934635	4.00722531	2	2	2	2	7.54878318	7.873223515
ENSG00000063241	ISOC2	0.062234787	0.962823674	0.054655989	0.968477418	0.116890776	9.771132748	6.728495673	7.777116083	8.700804887	8.70315789	8.499651249	8.866113243
ENSG00000086232	EIF2AK1	-0.139632416	0.857804601	0.256205743	0.726349422	0.116573327	11.15554094	10.35843478	9.624627483	10.35500785	10.3918991	10.69730485	10.8360945
ENSG00000130766	SESN2	-0.026807827	0.985675142	0.142593536	0.925786782	0.11578571	5.866944937	8.623598428	9.61300511	8.618316353	8.69891174	9.365719319	7.957271773
ENSG00000164061	BSN	-0.588057193	0.749240001	0.703259459	0.690278517	0.115202266	2	3.076665209	4.874587708	3.515101962	3.2184422	4.388329244	4.188602707
ENSG00000164332	UBLCP1	0.364238938	0.548198478	-0.251122869	0.68474076	0.113116069	8.075571721	7.572420273	8.742726175	8.616799622	8.50338773	7.927733561	7.979721237
ENSG00000101247	NDUFAF5	0.104170899	0.960095276	0.00888354	0.996970186	0.113054439	2	5.131603625	7.600047325	6.29903474	6.44101097	6.394066622	6.158754168
ENSG00000221926	TRIM16	0.695012933	0.155535752	-0.582855663	0.233677793	0.11215727	8.075571721	7.538394143	7.934808822	8.469961514	8.61726622	6.970858896	7.55093161
ENSG00000160124	CCDC58	0.925705657	0.212930755	-0.813581975	0.272130443	0.112123683	6.257048221	4.93638933	5.59507254	6.586219071	6.49425375	4.785723309	5.080008298
ENSG00000105229	PIAS4	0.001619009	0.997903682	0.110454042	0.879418834	0.11207305	7.27616306	7.503546079	8.434763955	7.815902688	7.83385794	8.135306947	7.694749691
ENSG00000198680	TUSC1	-0.523961365	0.623796324	0.634836873	0.538227094	0.110875507	5.70932728	7.189546432	8.35328246	6.965655398	6.91125523	8.054501123	8.087908463
ENSG00000153814	JAZF1	-0.51078519	0.716843352	0.621204661	0.650182334	0.110419471	4.815609471	8.356994601	8.805342542	7.605658789	7.52080763	8.622816121	8.733401188
ENSG00000087263	OGFOD1	0.243155379	0.647252339	-0.133170437	0.808262771	0.109984942	9.751047882	8.916342327	9.735974718	9.739866907	9.76891184	9.240714099	9.507317152
ENSG00000156502	SUPV3L1	0.403968942	0.383159098	-0.297355898	0.523553954	0.106613044	7.667647223	7.232742069	7.934808822	8.100504225	7.94320592	7.384136831	7.287011591
ENSG00000130669	PAK4	-0.069464078	0.935745276	0.174666789	0.834220385	0.105202712	8.228665467	8.540087354	9.798878489	8.936696791	8.96953398	9.110223929	9.276085345
ENSG00000101367	MAPRE1	-0.241070389	0.803176349	0.34573858	0.708647078	0.104668191	9.031919741	10.47993704	11.20470823	10.20863848	10.2873694	10.66238005	10.98693891
ENSG00000155189	AGPAT5	-0.227270954	0.897554944	0.33192247	0.845529489	0.104651517	3.327711234	7.145017454	8.408111533	7.084543139	7.17852729	7.635907362	7.719319651
ENSG00000158773	USF1	0.735214758	0.253424754	-0.631272195	0.325173109	0.103942563	8.418903792	8.623598428	7.225742779	8.901002576	8.98007065	7.526684775	7.656486163
ENSG00000154945	ANKRD40	-0.110773417	0.879824258	0.21457261	0.75370958	0.103799193	8.87372952	9.405920729	10.21486071	9.539906319	9.44475345	9.737087614	9.892433725
ENSG00000213886	UBD	-0.230175068	0.490900748	0.332986909	0.291605818	0.102811841	12.10369464	12.08742575	12.30989775	11.97318846	11.9067054	12.28739969	12.69058631
ENSG00000139998	RAB15	-0.905376566	0.497088189	1.007976617	0.435748117	0.102600051	8.257427002	5.838937808	9.69246155	7.799951945	7.66998641	9.795023124	9.443089155
ENSG00000054267	ARID4B	0.663278599	0.106608823	-0.561032867	0.172094144	0.102245732	8.963744667	9.415325457	9.478569125	9.990576409	9.93757622	8.965573751	8.490627472
ENSG00000143420	ENSA	-0.311081418	0.638989421	0.411916465	0.518445971	0.100835047	10.12333326	8.903033433	9.990713521	9.449924671	9.45653888	9.963712247	10.3557463
ENSG00000114378	HYAL1	-2.576747169	0.023043738	2.677500958	0.005400389	0.100753789	4.00722531	3.686303414	4.058998049	2.498691189	2.59489879	6.148830293	6.286392397
ENSG00000118407	FILIP1	-0.134945661	0.95389048	0.235444579	0.918692085	0.100498918	3.327711234	2	3.369317972	2.947924294	2.86123014	3.316666208	2.817676617
ENSG00000107518	ATRNL1	-2.619049532	0.06531578	2.719353566	0.036821565	0.100304034	3.327711234	5.131603625	3.369317972	3.023133209	2.2680111	6.723178551	6.676467423
ENSG00000165169	DYNLT3	-0.047117529	0.926779701	0.147358141	0.758720739	0.100240612	8.228665467	8.639735838	8.408111533	8.427334995	8.35836495	8.824761857	8.298235265
ENSG00000120318	ARAP3	0.428592663	0.807381293	-0.328708492	0.85201813	0.09988417	3.327711234	8.053983481	5.772716771	7.165617905	7.2537885	6.645228724	6.29264319
ENSG00000114744	COMMD2	-0.31713075	0.501509841	0.416627805	0.353451393	0.099497056	8.797441463	8.101365653	8.825624954	8.264463296	8.31463236	9.076575731	8.953033422
ENSG00000106105	GARS	-0.135522495	0.770162624	0.232052925	0.597996964	0.09653043	10.80726569	11.31160468	11.51057165	11.09457533	11.1130312	11.59940158	11.3296143
ENSG00000169379	ARL13B	0.184058571	0.91483416	-0.088085159	0.959372651	0.095973411	7.82963488	5.838937808	3.369317972	6.872128674	6.71216164	6.697032326	6.347714043
ENSG00000196466	ZNF799	0.32154846	0.878583032	-0.226725516	0.913644152	0.094822944	6.366709671	3.686303414	2	5.319220934	5.33407135	5.174809258	4.411752372
ENSG00000197321	SVIL	-0.408748485	0.435404672	0.502584803	0.316308322	0.093835958	9.567995545	9.687856435	10.46808205	9.604082348	9.50841342	10.47890139	10.45051519
ENSG00000129187	DCTD	0.141752681	0.790629813	-0.049052226	0.929877038	0.092700455	10.41260941	9.583268459	10.12770132	10.22615207	10.2128923	9.831105898	10.20379719
ENSG00000171403	KRT9	-3.753005516	0.531143362	3.845397614	0.489327719	0.092392098	2	2	2	2	2	3.355392153	4.757365452
ENSG00000139133	ALG10	0.730302692	0.610603213	-0.638154406	0.655811705	0.092148286	5.095959458	4.93638933	2	5.304221918	5.0259382	4.321442857	3.67940853
ENSG00000160058	BSDC1	0.239466113	0.755240998	-0.147510376	0.851219083	0.091955737	11.20066853	9.608085847	10.36791565	10.7284349	10.8180948	10.38082821	10.39513041
ENSG00000042493	CAPG	0.138321124	0.922673812	-0.046934828	0.97326848	0.091386296	10.05943807	8.522788974	6.43197674	9.061245954	9.18219179	8.904861985	8.972631533
ENSG00000088320	REM1	-3.753005537	0.531143362	3.843727009	0.48942971	0.090721472	2	2	2	2	2	3.5515855	4.673629022
ENSG00000135108	FBXO21	-0.500315198	0.606861222	0.590937067	0.529397088	0.090621869	7.159102577	9.214490408	9.478569125	8.408172799	8.43695333	9.532891421	9.476418815
ENSG00000159166	LAD1	-2.272292985	0.446344169	2.361568347	0.360634167	0.089275362	2	2	3.369317972	2.141737246	2.14022021	2.962551659	4.365060851
ENSG00000167977	KCTD5	0.202930992	0.843549372	-0.116704837	0.910950051	0.086226155	9.567995545	7.35507212	9.426401271	9.221512599	9.31018819	8.971428833	8.926648946
ENSG00000088451	TGDS	0.585439624	0.441837321	-0.499295236	0.508321704	0.086144388	5.866944937	6.389896633	6.43197674	6.815074525	6.81514011	6.192262702	5.217771644
ENSG00000177426	TGIF1	0.715227142	0.649501644	-0.629684135	0.688459235	0.085543007	11.03613077	7.538394143	7.285536013	10.36768823	10.3932137	9.095286533	8.987157359
ENSG00000137055	PLAA	0.313375341	0.594131176	-0.228329459	0.700693286	0.085045882	9.397585735	8.469613413	9.528916236	9.489414201	9.52849248	8.852100097	9.080880746
ENSG00000113205	PCDHB3	0.252871434	0.901083982	-0.168269121	0.93349971	0.084602314	2	4.11352228	3.369317972	3.921164147	3.2184422	3.059555873	3.580696614
ENSG00000116809	ZBTB17	0.224745907	0.780077895	-0.140358189	0.863604833	0.084387717	8.009475818	6.899080544	8.561105505	8.14116509	8.23084153	7.793159202	7.860656447
ENSG00000198538	ZNF28	-0.051772415	0.959782419	0.136008115	0.895080873	0.0842357	6.563806279	7.572420273	8.699419242	7.810605345	7.81567127	8.259184498	7.678134128
ENSG00000084093	REST	0.043534826	0.981829576	0.040471047	0.982913396	0.084005872	10.37373127	9.13702746	4.523675391	9.436230969	9.27923788	9.357396362	9.35623215
ENSG00000106462	EZH2	1.754839289	0.168980059	-1.67232205	0.184671758	0.08251724	5.095959458	7.669933291	4.523675391	8.195011988	8.14604733	4.996409624	4.853519801

ENSG00000169220	RGS14	-0.192835627	0.796574107	0.273048131	0.699954446	0.080212504	6.257048221	5.838937808	6.073378139	5.651002631	6.0722229	6.560755617	6.018717758
ENSG00000181026	AEN	-0.379036406	0.751922716	0.458997684	0.694619921	0.079961278	10.46903009	9.159587375	7.451345288	9.103012382	9.13268055	10.16889595	9.695099637
ENSG00000013503	POLR3B	0.015723098	0.990899695	0.063929416	0.963128357	0.079652514	5.095959458	8.004992147	6.073378139	6.941624891	6.9210099	6.898949899	7.052989523
ENSG00000146386	ABRACL	1.343802342	0.104122789	-1.265648571	0.122892685	0.078153771	7.579279349	6.899080544	5.39244166	8.124184191	8.25995663	5.574182174	5.781546943
ENSG00000109654	TRIM2	1.111586373	0.067047287	-1.033894582	0.087579018	0.077691791	7.384433967	7.572420273	6.203069917	8.321442665	8.21705076	6.151583464	6.222348126
ENSG00000101003	GINS1	-0.119721355	0.956634366	0.195676188	0.930031781	0.075954834	3.327711234	6.535001748	2	4.946755474	5.19627118	5.294231737	5.419744333
ENSG00000196214	ZNF766	-0.835896695	0.538896186	0.911815618	0.491920975	0.075918923	4.00722531	7.467835385	7.858115942	6.415275989	6.26836889	8.32468739	7.68646583
ENSG00000188690	UROS	-0.012446354	0.991325463	0.085741807	0.939676905	0.073295453	8.777722034	8.94259736	6.43197674	8.312101014	8.47717992	8.411091064	8.572538058
ENSG00000198586	TLK1	0.587415981	0.090299556	-0.516934659	0.133943241	0.070481322	9.473095744	9.785372135	9.287079172	10.13796435	10.1004228	8.902412249	9.130578617
ENSG00000179218	CALR	0.47090534	0.488092913	-0.401092241	0.552503225	0.069813099	14.00486055	13.04571907	13.08372738	13.88981009	13.9512905	12.51624569	13.43750115
ENSG00000076242	MLH1	0.164459775	0.928883922	-0.097245773	0.958016332	0.067214001	7.485143553	8.834582582	3.369317972	7.895617537	7.93841129	7.474865722	7.824443511
ENSG00000169813	HNRNPF	-0.316547493	0.511669208	0.382947352	0.408802956	0.066399859	10.6695979	10.51097332	9.819251342	10.08080308	10.0457438	10.73943283	10.7818607
ENSG00000257923	CUX1	-0.120181579	0.80412149	0.185576553	0.687726485	0.065394974	9.992581382	10.68045884	10.09465441	10.14850007	10.1954158	10.38302632	10.56516425
ENSG00000175387	SMAD2	-0.008842393	0.993765388	0.074070093	0.943575121	0.065227699	10.3933013	9.523645035	8.042706615	9.587916984	9.61516703	9.673618595	9.694509651
ENSG00000158604	TMED4	-0.069277019	0.889181201	0.131845981	0.778721241	0.062568961	10.72212986	10.22575081	9.963514551	10.24492915	10.2934839	10.47230966	10.46753608
ENSG00000198934	MAGEE1	-0.470179312	0.821622214	0.530848573	0.79391096	0.060669262	7.53297911	2	5.59507254	5.829220145	5.88556448	6.710164669	6.899677537
ENSG00000116863	ADPRHL2	-0.269339926	0.709351398	0.329566777	0.63788893	0.060226851	9.371511179	8.101365653	8.486635645	8.474993083	8.4936155	8.776082909	9.327219816
ENSG00000198156	NPIPB6	3.412484602	0.002364079	-3.352561433	0.0045879	0.059923168	4.00722531	3.076665209	5.156626401	7.412876874	7.37229174	2.656294034	2.225841198
ENSG00000189057	FAM111B	0.061777076	0.982251788	-0.004232548	0.998980237	0.057544528	2	2	4.058998049	3.227757282	2.77779907	2.831336763	3.128433649
ENSG00000135535	CD164	0.801733694	0.187195342	-0.744753344	0.21592192	0.05698035	10.57565445	11.8974889	11.39316998	12.2479868	12.1255035	10.79309453	10.47751587
ENSG00000153823	PID1	2.844507483	1.38E-11	-2.788501435	4.74E-11	0.056006048	8.444143041	8.451443573	8.865353883	11.43428179	11.4250965	6.210860813	5.486701579
ENSG00000152683	SLC30A6	0.133804407	0.798798132	-0.077891002	0.884519325	0.055913405	9.344956677	8.505280662	8.784770914	9.060130342	9.03948698	8.792933324	8.885369555
ENSG00000125818	PSMF1	-0.301844421	0.550280394	0.356520575	0.467088383	0.054676154	10.16946352	9.930175364	9.301632639	9.551067273	9.53961588	10.03328532	10.35107441
ENSG00000008083	JARID2	0.678435393	0.246800072	-0.62386462	0.282300357	0.054570773	8.418903792	8.234834549	9.344432377	9.39612172	9.44390794	8.038228667	8.216759768
ENSG00000104671	DCTN6	0.572252845	0.585414444	-0.517816635	0.619379532	0.054436209	8.998234871	7.315436877	6.629488387	8.593855195	8.53707846	7.38764416	7.59079099
ENSG00000104660	LEPROTL1	-0.294151256	0.653912395	0.348545652	0.58421129	0.054394396	9.48530526	8.169643011	8.99637432	8.707945635	8.6601233	9.373994535	9.267780572
ENSG00000205358	MT1H	5.244689346	0.254666268	-5.191106557	0.288611054	0.053582789	2	2	2	3.349643765	6.22964624	2	2
ENSG00000109270	LAMTOR3	0.226051414	0.788297603	-0.173338053	0.837335975	0.052713361	7.031698319	8.732943944	8.561105505	8.461536372	8.55598286	8.237320313	7.981656955
ENSG00000180340	FZD2	-0.941041324	0.39960716	0.993614281	0.361984045	0.052572957	9.966689162	8.717821467	6.882775709	8.036145657	8.10812366	9.985285987	9.997333704
ENSG00000256771	ZNF253	0.60617024	0.655168404	-0.553716899	0.682815119	0.052453342	4.00722531	7.274681896	5.59507254	6.841288255	6.73998187	5.976323695	5.343519008
ENSG00000089775	ZBTB25	-0.224623135	0.916883733	0.276393186	0.896486957	0.051770051	2	5.946638857	8.143093325	6.682159616	6.59518404	7.324389982	6.889403418
ENSG00000167981	ZNF597	0.235758526	0.914584038	-0.184395296	0.933063871	0.05136323	4.815609471	7.669933291	2	6.610808945	6.45450701	6.249924465	6.011156536
ENSG00000143612	C1orf43	-0.15964596	0.897978564	0.210815293	0.861704178	0.051169333	12.07166289	10.17682307	9.344432377	10.8123265	10.8532269	11.15913757	11.24491309
ENSG00000164182	NDUFAF2	0.228877906	0.841054398	-0.17892196	0.874995212	0.049955946	4.815609471	7.051611315	7.451345288	6.989292186	7.06873058	6.725028131	6.541257688
ENSG00000173327	MAP3K11	-0.278785305	0.778392611	0.327819439	0.733748666	0.049034134	8.16936288	9.018611864	10.31150679	9.171766106	9.14209426	9.531042494	9.957809357
ENSG00000196923	PDLIM7	-0.488793225	0.373373057	0.537153918	0.314029219	0.048360693	11.89987989	10.8599287	11.47570169	10.96228447	11.0056822	11.80075995	12.19032173
ENSG00000100258	LMF2	0.247878395	0.574576953	-0.199926028	0.652472336	0.047952367	11.01943573	10.43902632	10.35401885	10.86374419	10.9005244	10.36223065	10.50536667
ENSG00000108518	PFN1	-0.296046398	0.735002613	0.343934277	0.687843562	0.047887879	13.52950438	11.87039211	12.15221073	12.40121794	12.4293961	12.75624689	13.30233737
ENSG00000105246	EBI3	-3.236747458	0.116637236	3.283447776	0.088390629	0.046700318	2	5.595927442	2	2.270786764	2.68924543	7.184873526	7.351304958
ENSG00000196867	ZFP28	-0.681603878	0.417942235	0.727482504	0.3708938	0.045878627	7.790810999	5.946638857	7.225742779	6.634986713	6.35727082	8.04860508	7.669754031
ENSG00000138375	SMARCAL1	0.096570903	0.93008201	-0.051458881	0.963377583	0.045112022	9.143983763	7.503546079	6.958202216	8.260583448	8.29037844	8.006643981	8.242819476
ENSG00000186326	RGS9BP	-1.269142941	0.673171288	1.314192505	0.638181881	0.045049564	2	3.076665209	2	2.3892346	2.14022021	3.316666208	2.981406154
ENSG00000100105	PATZ1	-0.192719933	0.887105191	0.23763546	0.85594901	0.044915527	4.815609471	8.101365653	7.858115942	7.273514749	7.31425651	7.840848421	7.576773052
ENSG00000138271	GPR87	5.234806949	0.196836305	-5.191118168	0.237913947	0.043688782	2	2	2	5.161714397	5.6121243	2	2
ENSG00000079739	PGM1	-0.142188312	0.819577124	0.183949838	0.759271438	0.041761526	10.45668146	9.45234302	10.40208303	9.948991943	10.1009593	10.20380084	10.48640496
ENSG00000100612	DHRS7	1.36032436	0.021528722	-1.319098561	0.025554781	0.041225799	8.836089686	9.055168103	10.08627303	10.80601808	10.7686428	8.264281212	7.971952287
ENSG00000156531	PHF6	0.02668943	0.983064817	0.014312686	0.99072569	0.041002116	6.009025997	8.747909548	8.742726175	8.343618883	8.240612	8.539610093	7.965119966
ENSG00000122390	NAA60	-0.245891688	0.748322371	0.286443035	0.702091517	0.040551346	9.886101849	9.506147172	8.529066785	9.155143657	9.11153864	9.465620252	9.836041609
ENSG00000163399	ATP1A1	0.494224993	0.256360353	-0.453699805	0.292481588	0.040525187	11.18203661	11.53295289	11.95457585	12.06794823	12.1009069	11.25282129	11.01284462
ENSG00000213965	NUDT19	-0.271711096	0.770883416	0.309651774	0.73428898	0.037940678	7.53297911	8.57407404	6.534109959	7.484483106	7.56191311	8.047866378	8.139971339
ENSG00000197150	ABCB8	0.055154152	0.98047943	-0.017271214	0.993931149	0.037882938	2	7.761270542	8.960138144	7.971157485	7.94559728	7.529862489	8.173382342
ENSG00000116791	CRYZ	-0.350658531	0.58220518	0.386734344	0.531194792	0.036075813	7.667647223	6.602424674	7.691296561	6.979883891	7.10768997	7.684242973	7.841598174
ENSG00000138735	PDE5A	-2.077168152	0.072123063	2.112488153	0.062801111	0.03532	7.33131396	10.24697497	7.691296561	7.030886362	6.97819215	11.36070623	10.90630802
ENSG00000136826	KLF4	-0.899844595	0.309576645	0.934911781	0.278767895	0.035067186	7.940205782	7.761270542	9.624627483	7.778406195	7.84670947	9.674815254	9.581945714
ENSG00000186575	NF2	-0.921481395	0.053285833	0.955799625	0.041273153	0.03431823	9.771132748	9.540933215	10.32581712	8.930605282	9.06500642	10.58238213	11.10707312
ENSG00000163898	LIPH	-2.968225821	0.197278637	3.001843828	0.167372108	0.033618007	2	2	5.59507254	2.270786764	2.77779907	6.104049656	7.411419562
ENSG00000165533	TTC8	0.920318672	0.629335817	-0.886811352	0.638784658	0.03350732	8.16936288	8.337339476	2	8.607665593	8.57154947	6.812870125	6.826173076
ENSG00000173264	GPR137	-0.607812403	0.072172046	0.639840174	0.051748984	0.032027771	8.797441463	9.055168103	8.742726175	8.178656739	8.36552655	9.385442197	9.62840116
ENSG00000114904	NEK4	0.398671978	0.739013584	-0.367078515	0.758322907	0.031593463	5.70932728	6.228547981	8.236946975	7.626911583	7.44845993	6.940933331	6.635252003
ENSG00000134759	ELP2	0.001695854	0.997481474	0.027815915	0.963863059	0.029511769	9.233563228	9.821190352	8.865353883	9.342988948	9.39224836	9.536845448	9.234886737
ENSG00000188725	SMIM15	0.266076458	0.534490579	-0.236746999	0.57774123	0.029329459	8.39321512	8.747909548	8.110401118	8.780186674	8.64254348	8.257907501	8.172535245
ENSG00000244687	UBE2V1	2.853630014	7.05E-08	-2.824695714	1.20E-07	0.028934299	7.904282243	7.002537973	8.35328246	10.63786749	10.7125292	5.073924441	5.250250853
ENSG00000196950	SLC39A10	-1.387512202	0.01604719	1.415518104	0.012677767	0.028005902	8.609439662	8.820493869	7.691296561	7.182120794	7.00136498	9.515096581	10.13979504
ENSG00000038210	PI4K2B	1.006379072	0.030828564	-0.97962898	0.035010376	0.026750091	6.816637977	7.274681896	7.734844356	8.310225403	8.30535212	6.302895343	6.443521721
ENSG00000113300	CNOT6	0.219908419	0.63056237	-0.193411407	0.672104764	0.026497012	8.609439662	8.469613413	9.165043284	9.022822647	8.96128525	8.596789458	8.568679902
ENSG00000134533	RERG	-3.224023371	0.182596921	3.249411075	0.154661864	0.025387704	2	5.595927442	2	2.141737246	2.77779907	5.790986459	7.927454842
ENSG00000092201	SUPT16H	-0.035021536	0.972180135	0.060210061	0.952633805	0.025188725	10.29961392	10.28338212	8.236946975	9.864602909	9.80886355	9.868192425	9.992781936
ENSG00000070718	AP3M2	0.187464546	0.676328124	-0.162463892	0.716074072	0.025000654	8.468948322	8.717821467	8.845626171	8.88597028	8.85716692	8.749978316	8.258072602
ENSG00000125651	GTF2F1	0.008618337	0.994208627	0.016231548	0.988291799	0.024849884	10.51739401	9.497318007	8.110401118	9.650037049	9.73059821	9.428927807	9.925491821
ENSG00000134986	NREP	-0.920685924	0.233103112	0.944815089	0.211911316	0.024129165	9.317904228	10.09725346	10.84117713	9.300161685	9.29807478	11.5927062	10.53268998
ENSG00000185008	ROBO2	-1.915387485	0.346794529	1.939502735	0.321893604	0.02411525	3.327711234	5.457120953	3.369317972	3.709785322	2.14022021	4.914492341	6.847559257
ENSG00000105419	MEIS3	0.03782926	0.968309509	-0.013764803	0.988584182	0.024064457	9.423197387	9.090820894	7.398165132	8.939126212	8.87232943	8.773404234	8.932164792
ENSG00000171159	C9orf16	-0.411472194	0.616466414	0.435524494	0.587446222	0.0240523	9.128497779	7.572420273	9.287079172	8.392303091	8.46222608	9.428644092	9.085388321
ENSG00000107223	EDF1	0.247038642	0.603540731	-0.223519937	0.635737899	0.023518705	10.83615308	10.07368926	10.5916593	10.7730345	10.7844255	10.14560825	10.45661716
ENSG00000205250	E2F4	-0.09503982	0.941409766	0.113864925	0.930293595	0.018825105	8.928409675	9.656475478	6.43197674	8.658676732	8.84568954	8.927527801	8.996281974
ENSG00000161533	ACOX1	-0.085763735	0.890295464	0.10450308	0.862199682	0.018739345	9.384607364	8.337339476	8.884815469	8.929383887	8.74768919	9.088477995	8.969221026
ENSG00000175854	SWI5	-0.173691914	0.91115308	0.190284861	0.900816595	0.016592947	8.228665467	7.538394143	4.058998049	7.25413313	7.18663697	7.566427714	7.585709303
ENSG00000073578	SDHA	0.128196666	0.871671212	-0.113589152	0.885335383	0.014607514	10.3868074	9.102512015	9.116480286	9.773608662	9.81802855	9.649231086	9.454981667
ENSG00000176024	ZNF613	-0.152765746	0.95872661	0.16674461	0.955794916	0.013978864	2	7.761270542	2	6.164317909	6.01871653	6.569017168	6.199243628
ENSG00000164002	EXO5	0.476387863	0.654150801	-0.462585389	0.661465236	0.013802473	5.70932728	6.535001748	4.058998049	6.268449691	6.18985553	5.381251181	5.337485258
ENSG00000140995	DEF8	-0.271183895	0.691499043	0.280227773	0.677268519	0.009043879	8.313285031	9.13702746	9.69246155	8.902248225	8.86476809	9.53894984	9.314605104
ENSG00000164096	C4orf3	0.247453559	0.800804384	-0.238800439	0.804586396	0.008653119	11.02362764	9.055168103	10.75919294	10.77541564	10.7167355	9.763874267	10.63077694
ENSG00000239789	MRPS17	0.156115041	0.861781723	-0.148134601	0.865271077	0.00798044	5.70932728	4.93638933	6.43197674	6.01571348	5.84514933	5.610655473	5.689801209
ENSG00000261652	C15orf65	0.059447946	0.982666154	-0.051798017	0.9847789	0.007649928	2	2	5.772716771	4.358765249	4.45712836	4.369533167	4.26688425
ENSG00000180694	TMEM64	-0.4232975	0.800895544	0.430174925	0.794249133	0.006877425	3.327711234	8.234834549	7.818184403	7.136275664	7.01053089	8.072046038	7.725108479
ENSG00000166619	BLCAP	-0.458332466	0.633070574	0.46472442	0.623383229	0.006391955	10.36053552	8.147241016	9.778213818	9.219515356	9.24275568	9.799418565	10.43134488
ENSG00000156110	ADK	-0.392679346	0.554575573	0.398472379	0.541021035	0.005793033	8.609439662	7.572420273	7.646393253	7.739909082	7.52080763	8.145010376	8.639663797
ENSG00000183617	MRPL54	0.139716208	0.829668812	-0.133926852	0.833626336	0.005789356	7.709880939	8.234834549	7.225742779	7.949630994	7.91175216	7.422256313	7.868000534
ENSG00000162928	PEX13	-0.110719781	0.92127192	0.115621079	0.917565744	0.004901298	7.867441304	7.002537973	9.358421373	8.350935846	8.23084153	8.595273498	8.431665941
ENSG00000159692	CTBP1	0.227414581	0.785042442	-0.223888236	0.785920862	0.003526345	10.58702944	9.148351514	9.132850258	9.99525349	10.046858	9.585759327	9.558144718
ENSG00000134697	GNL2	0.107636163	0.915320238	-0.104464283	0.917689829	0.003171881	10.34721797	8.903033433	8.325066185	9.571568433	9.54593381	9.230659332	9.456374363
ENSG00000129219	PLD2	-0.409619152	0.686593442	0.412054733	0.680326628	0.002435581	8.075571721	8.029695761	5.772716771	7.25803034	7.17037177	7.922907177	8.110198799
ENSG00000206418	RAB12	-0.12038533	0.807798742	0.122667827	0.800369309	0.002282497	9.410448395	8.876041558	8.699419242	8.924487942	8.88981993	9.263482089	9.023314583
ENSG00000177150	FAM210A	0.09577006	0.853531206	-0.094616071	0.85221514	0.001153988	7.485143553	7.954278496	7.285536013	7.703217906	7.71833702	7.595026956	7.449681889
ENSG00000187741	FANCA	0.48459663	0.739704558	-0.483929471	0.736888343	0.00066716	5.095959458	5.595927442	2	5.194621444	5.41912973	4.369533167	4.574506939
ENSG00000090520	DNAJB11	0.824184447	0.129332125	-0.827672174	0.121602573	-0.003487727	9.931427102	9.981728319	9.031722608	10.5182619	10.5464078	8.594767824	9.133193298
ENSG00000151806	GUF1	-0.021509767	0.98350331	0.017711322	0.986497228	-0.003798445	6.563806279	8.469613413	6.958202216	7.53353831	7.60490877	7.815031728	7.366199662
ENSG00000077147	TM9SF3	0.248699125	0.824376979	-0.25564107	0.815972748	-0.006941945	9.533135543	11.14353618	12.21698408	11.61835275	11.5523437	11.12496121	11.03806084
ENSG00000104415	WISP1	0.306557854	0.879440766	-0.313755922	0.873635383	-0.007198068	7.82963488	6.78762695	2	7.136275664	7.1246732	6.019162896	6.909879006
ENSG00000164751	PEX2	-0.365502806	0.428026621	0.357342936	0.427508497	-0.00815987	8.564112515	8.487557258	7.818184403	7.956842238	7.98565528	8.615356492	8.751666251
ENSG00000159592	GPBP1L1	0.174020261	0.852628537	-0.18249688	0.842165885	-0.008476619	8.586954094	9.672251278	10.60345421	10.06865021	9.96140527	9.71002767	9.610373471
ENSG00000115677	HDLBP	-0.400608993	0.490606244	0.391938251	0.492233435	-0.008670742	12.46905053	13.35763551	13.55612433	12.77838142	12.8186338	13.41539496	13.74726846
ENSG00000083535	PIBF1	0.203374661	0.831652978	-0.213591031	0.818512537	-0.01021637	7.27616306	7.232742069	5.59507254	7.114945424	7.05992818	7.015370516	6.254725615
ENSG00000164163	ABCE1	0.087257416	0.93880631	-0.097800389	0.932122597	-0.010542973	7.27616306	9.549500232	7.734844356	8.639384849	8.62772819	8.441219213	8.459712934
ENSG00000081154	PCNP	-0.114738178	0.887076711	0.103904364	0.895904824	-0.010833813	10.29961392	10.019219	8.742726175	9.751662842	9.67691814	10.03403152	9.823144976
ENSG00000095627	TDRD1	-3.753006835	0.531143362	3.739779159	0.502368069	-0.013227677	2	2	2	2	2	4.73522696	3.100202476
ENSG00000134909	ARHGAP32	0.25841915	0.772399916	-0.272656027	0.757050516	-0.014236878	6.563806279	8.124485672	6.629488387	7.590284224	7.5462416	7.083273719	7.017577533

ENSG00000187240	DYNC2H1	0.747023097	0.622552387	-0.763066631	0.609510346	-0.016043533	5.532353208	8.234834549	4.523675391	7.778406195	7.59580267	6.034159474	6.403650353
ENSG00000247596	TWF2	0.229905916	0.858507416	-0.246811198	0.844695037	-0.016905281	10.02639697	7.790474954	7.342949392	9.115954467	9.12426093	8.679237241	8.614934151
ENSG00000159788	RGS12	0.025756057	0.978514936	-0.043143322	0.964338803	-0.017387265	7.031698319	6.728495673	8.408111533	7.584088227	7.61995902	7.669911566	7.385335693
ENSG00000108829	LRRC59	-0.18812856	0.744523835	0.168125379	0.770703322	-0.020003181	11.58798587	10.55418374	11.22775303	10.98773739	11.0025273	11.20375836	11.48422157
ENSG00000111247	RAD51AP1	0.129754643	0.95872661	-0.151641092	0.952529433	-0.021886449	2	5.457120953	2	4.329433326	4.1930041	3.981090143	4.105828626
ENSG00000157388	CACNA1D	-2.312760619	NA	2.290327653	NA	-0.022432966	2	2	2	2	2.14022021	3.355392153	2.950113106
ENSG00000185418	TARSL2	-0.706561556	0.05996039	0.683960643	0.06220751	-0.022600912	7.750913381	8.029695761	7.734844356	7.110641269	7.21069602	8.389678938	8.652479133
ENSG00000281763		0.968011833	0.184484665	-0.991503157	0.165663949	-0.023491325	7.384433967	7.954278496	9.049077186	9.294480609	9.21229508	7.346184975	7.296373648
ENSG00000080854	IGSF9B	-2.632918738	0.274396109	2.608273723	0.252282956	-0.024645015	2	2	5.156626401	2.784612485	2.14022021	5.944926241	6.151894053
ENSG00000183762	KREMEN1	-1.813818676	0.151077965	1.788647565	0.146121333	-0.02517111	4.815609471	4.93638933	7.691296561	4.71689672	4.87352701	8.261097876	8.126912071
ENSG00000143093	STRIP1	0.125951833	0.832844524	-0.151303335	0.79391096	-0.025351503	8.540903468	8.791896851	9.503962296	9.088859521	9.16485023	8.734912119	8.960904642
ENSG00000278312		5.165535877	0.209831388	-5.191117205	0.243536295	-0.025581327	2	2	2	5.420062284	5.2591458	2	2
ENSG00000111707	SUDS3	-0.167811788	0.831652978	0.141553566	0.855655091	-0.026258222	8.16936288	8.806266215	9.714382178	8.830780654	8.90464485	9.201064633	9.148782641
ENSG00000197006	METTL9	0.393840225	0.316169441	-0.420217078	0.273363547	-0.026376853	10.54098126	10.47544827	9.907528187	10.71680073	10.7410309	9.900262774	9.934514013
ENSG00000178229	ZNF543	0.140462711	0.906603349	-0.167352161	0.886082448	-0.02688945	4.815609471	4.93638933	6.80318718	5.949776857	5.92487824	5.699879814	5.601925227
ENSG00000213463	SYNJ2BP	-0.473686445	0.595799399	0.445396054	0.614013611	-0.028290391	7.435667355	9.079034257	9.528916236	8.545247918	8.34936267	9.365719319	9.358096047
ENSG00000115289	PCGF1	-0.11974355	0.903607792	0.090966367	0.926534356	-0.028777182	6.009025997	6.844429755	5.156626401	6.139181293	6.00960265	6.550361701	5.933281326
ENSG00000186715	MST1L	2.625926345	0.180152411	-2.656164129	0.189944554	-0.030237784	3.327711234	3.076665209	2	4.647285135	4.76938658	2.3070652	2.059860178
ENSG00000114850	SSR3	-0.135197821	0.767206549	0.104060828	0.820771665	-0.031136993	11.98618033	11.72292201	12.41570295	11.95199324	11.9166432	12.0510638	12.28629031
ENSG00000126934	MAP2K2	-0.259423584	0.539543786	0.227592017	0.588973243	-0.031831568	10.5468183	9.962611189	10.11127249	9.922839878	10.0130538	10.28941421	10.6018087
ENSG00000112214	FHL5	-2.31280349	NA	2.280676918	NA	-0.032126572	2	2	2	2	2.14022021	3.724264246	2.225841198
ENSG00000215784	FAM72D	5.157088758	0.211381873	-5.191117084	0.244323998	-0.034028325	2	2	2	5.363304456	5.30456641	2	2
ENSG00000101400	SNTA1	-0.929869578	0.679377579	0.895198777	0.686960124	-0.034670802	2	7.35507212	9.888372618	7.54315232	7.6957855	9.441918755	9.408611581
ENSG00000117691	NENF	0.042761786	0.930551849	-0.077769508	0.871169659	-0.035007722	9.304185368	8.590771778	9.149036566	9.04103174	9.12214832	8.969089651	8.955990164
ENSG00000197037	ZSCAN25	-0.255123004	0.870868026	0.219695388	0.887741643	-0.035427616	4.00722531	7.051611315	8.266922072	7.035434774	6.96410766	7.401588825	7.520630205
ENSG00000108651	UTP6	0.153781289	0.850026272	-0.190546327	0.808588735	-0.036765038	9.031919741	8.101365653	7.451345288	8.481674661	8.50338773	8.043426202	8.25407423
ENSG00000100554	ATP6V1D	0.240566546	0.875544825	-0.278305839	0.851903823	-0.037739293	10.60951384	7.954278496	7.098165388	9.60255057	9.55848727	8.924311548	9.193312432
ENSG00000197451	HNRNPAB	-0.038120033	0.970008162	-0.00130432	0.99924195	-0.039424353	10.78274301	9.225224669	8.805342542	9.847892119	9.81476202	9.723505443	9.999484803
ENSG00000040608	RTN4R	-2.113096077	0.213019329	2.073560684	0.192445639	-0.039535393	3.327711234	4.93638933	2	2.784612485	2.68924543	6.247352872	4.870334174
ENSG00000167136	ENDOG	0.129521566	0.946720822	-0.171400072	0.931410087	-0.041878505	2	6.464272742	7.691296561	6.70517998	6.81514011	6.525106458	6.418016952
ENSG00000129680	MAP7D3	-0.559199719	0.362105114	0.516598619	0.391760019	-0.0426011	8.257427002	7.099070191	8.110401118	7.288834728	7.40393901	8.271892681	8.526208204
ENSG00000100749	VRK1	0.339892743	0.764551483	-0.383369704	0.729041892	-0.043476961	5.866944937	5.838937808	3.369317972	5.742861563	5.70538658	5.008609438	5.12973572
ENSG00000099337	KCNK6	-1.644887264	0.000817299	1.601178555	0.001008927	-0.043708709	9.410448395	8.687092496	8.721235202	7.381065997	7.33264456	10.18174778	10.87774805
ENSG00000079931	MOXD1	1.347517964	0.150716856	-1.393053101	0.129597634	-0.045535138	7.218819781	5.838937808	7.934808822	8.590768181	8.49688027	6.329884754	5.250250853
ENSG00000106049	HIBADH	-0.161514578	0.867570088	0.115975185	0.904611962	-0.045539394	9.204317094	9.267380431	7.398165132	8.615281294	8.74768919	8.630730772	9.224707098
ENSG00000113638	TTC33	0.204126551	0.890526182	-0.251110802	0.861440521	-0.046984251	4.00722531	7.232742069	7.691296561	7.161462517	7.15392111	7.031901364	6.314310122
ENSG00000100938	GMPR2	-0.452442272	0.837958929	0.405388112	0.852566533	-0.04705416	9.448362284	8.993716073	2	8.207157886	8.21705076	8.908121842	9.199563099
ENSG00000007923	DNAJC11	0.590114964	0.304803313	-0.637567816	0.255124481	-0.047452852	8.777722034	8.077869079	7.734844356	8.772033429	8.91688363	7.422256313	7.821204179
ENSG00000128626	MRPS12	-0.282336713	0.568853498	0.234569271	0.635038067	-0.047767441	7.435667355	8.124485672	8.206335842	7.723090567	7.64959655	8.094396944	8.289666801
ENSG00000175224	ATG13	-0.016703666	0.977090174	-0.031829675	0.956622971	-0.048533341	10.72727936	10.23640192	9.735974718	10.22266632	10.3213737	10.28691212	10.22805302
ENSG00000131459	GFPT2	2.066295679	1.75E-08	-2.114839439	7.94E-09	-0.048543761	8.981092835	9.540933215	9.465702942	11.38256115	11.4395901	7.475964489	7.019463152
ENSG00000170604	IRF2BP1	-0.587530217	0.256335494	0.537509834	0.292216612	-0.050020383	8.257427002	8.337339476	9.099922433	8.015574133	8.04930085	9.301474629	8.970683659
ENSG00000039068	CDH1	-2.266386616	0.514865066	2.215885979	0.479004141	-0.050500637	2	2	3.369317972	2.270786764	2	3.256555576	4.077147165
ENSG00000143839	REN	0.532894795	0.86129847	-0.583481377	NA	-0.050586582	2	3.076665209	2	2.600425781	2.94009902	2.492221014	2.326754077
ENSG00000083544	TDRD3	-0.215708993	0.838446668	0.165115452	0.875759484	-0.050593541	6.009025997	7.819099893	6.073378139	6.598566398	6.80989959	7.203566521	6.936070085
ENSG00000139726	DENR	0.048379803	0.953297746	-0.099039498	0.904744852	-0.050659695	7.940205782	9.125613817	9.61300511	9.138327453	9.05063789	8.844037986	9.046617405
ENSG00000197429	IPP	-0.620632979	0.786839864	0.569907744	0.80197737	-0.050725235	2	4.93638933	8.511888463	6.542151749	6.36443784	7.655436363	7.561323797
ZASP		5.139299599	0.216431863	-5.19111649	0.247207591	-0.051816891	2	2	2	5.110902178	5.49945122	2	2
ENSG00000131508	UBE2D2	-0.001591732	0.99707802	-0.050689183	0.900190897	-0.052280915	10.19941743	9.849218119	9.670202709	9.95740528	9.88434767	9.918562269	9.824763374
ENSG00000104863	LIN7B	-0.604037908	0.823403844	0.551374774	0.836490254	-0.052663133	2	6.666836837	2	4.623314311	4.74762503	5.812021848	5.606950214
ENSG00000132837	DMGDH	-3.753007336	0.531143362	3.699693042	0.507814827	-0.053314294	2	2	2	2	2	4.177580368	4.033026654
ENSG00000103490	PYCARD	1.128472467	0.042046869	-1.181974984	0.031574569	-0.053502517	6.009025997	6.311476754	6.80318718	7.484483106	7.50792049	5.50697268	5.072762365
ENSG00000140259	MFAP1	0.099816723	0.812768397	-0.153390516	0.701056475	-0.053573793	8.816894989	8.607278467	9.149036566	8.95000826	8.98589128	8.640073371	8.790280628
ENSG00000133315	MACROD1	-1.333178413	0.378770423	1.27920325	0.385805831	-0.053975163	5.532353208	6.464272742	2	4.299492648	4.4836996	6.950450741	6.533361209
ENSG00000122643	NT5C3A	2.346825624	1.72E-05	-2.401265782	1.16E-05	-0.054440159	7.790810999	8.004992147	6.629488387	9.976453497	9.86489641	5.385935071	5.250250853
ENSG00000109618	SEPSECS	0.455770577	0.825685372	-0.510241723	0.800237157	-0.054471145	7.867441304	5.72254323	2	7.017154451	7.05107174	6.473229652	5.621921006
ENSG00000180304	OAZ2	-0.846487328	0.295027628	0.789785345	0.321533624	-0.056701983	10.73241055	10.04368224	8.784770914	9.240349764	9.19128891	10.62881333	11.03421896
ENSG00000205133	TRIQK	-0.606508367	0.75782421	0.548925663	0.779935476	-0.057582704	8.418903792	7.790474954	2	6.993973425	6.95464102	8.195910056	8.006587762
ENSG00000125037	EMC3	0.653337446	0.357081274	-0.711368549	0.302366413	-0.058031103	10.05943807	8.590771778	9.06622548	10.02414665	10.0164701	8.458554046	8.8444706
ENSG00000181896	ZNF101	0.072479198	0.936390504	-0.131175177	0.883777495	-0.058695978	7.27616306	5.72254323	6.073378139	6.529309835	6.61935067	6.412547789	6.347714043
ENSG00000156011	PSD3	-0.608683441	0.770602177	0.549577902	0.792987538	-0.059105539	9.112843761	8.639735838	2	7.783822874	7.64959655	9.001879477	8.705562554
ENSG00000183454	GRIN2A	3.499811463	0.072123063	-3.561167325	0.069213816	-0.061355862	2	5.72254323	2	7.584088227	7.6378145	2.225793514	2.632958241
ENSG00000103018	CYB5B	0.116503589	0.873933481	-0.178752917	0.798439725	-0.062249328	9.646202137	10.13189344	8.904018013	9.793884031	9.73406074	9.116578627	9.754611319
ENSG00000136878	USP20	-0.513326422	0.845989871	0.450274894	0.863507758	-0.063051528	2	5.303523747	10.03493575	8.013270196	7.98797735	9.104551714	8.784193542
ENSG00000197756	RPL37A	-0.305413301	0.729728652	0.241634951	0.786775559	-0.06377835	12.02014453	10.25223258	10.59756881	10.81431291	10.9173739	11.34169508	11.4806329
ENSG00000169857	AVEN	-0.543240672	0.728541941	0.47892818	0.759498144	-0.064312492	5.095959458	8.053983481	9.767769415	8.040677544	8.10170499	9.075126337	9.09033044
ENSG00000119147	C2orf40	-3.753007475	0.531143362	3.688559532	0.509267049	-0.064447943	2	2	2	2	2	3.679182183	4.4231929
ENSG00000137764	MAP2K5	-0.458516318	0.820634582	0.393897343	0.843924153	-0.064618975	6.257048221	8.029695761	2	6.470063213	6.32824132	7.32316944	7.114266974
ENSG00000108852	MPP2	-0.704735147	0.581619703	0.638581505	0.614736828	-0.066153641	8.737455672	6.728495673	5.59507254	6.941624891	6.87658492	7.947671484	8.454843905
ENSG00000254535	PABPC4L	-0.764698938	0.749679501	0.697931827	0.767692243	-0.066767111	2	4.93638933	2	3.349643765	3.15372745	4.628601098	3.789585079
ENSG00000164941	INTS8	0.13935577	0.91483416	-0.207388302	0.870629355	-0.068032532	7.384433967	6.311476754	9.149036566	8.215198712	8.21705076	8.145700987	7.540464021
ENSG00000124535	WRNIP1	-0.404514607	0.466549268	0.334573672	0.544273144	-0.069940935	9.646202137	8.671479029	9.399592459	8.902248225	8.87609528	9.808387568	9.411127426
ENSG00000146278	PNRC1	0.150142013	0.903601303	-0.220966631	0.853313931	-0.070824618	10.91499272	10.02537381	7.896971941	10.22017133	10.2074225	9.80073459	9.886246986
ENSG00000169570	DTWD2	-0.572296878	0.479814084	0.500615318	0.527954208	-0.07168156	5.095959458	5.595927442	6.629488387	5.348759107	5.40529704	6.358763086	6.392053169
ENSG00000134028	ADAMDEC1	-3.753007591	0.531143362	3.679246468	0.51059361	-0.073761123	2	2	2	2	2	4.792794928	2.671842496
ENSG00000240583	AQP1	-2.57392445	0.015963161	2.498910616	0.009732169	-0.075013834	3.327711234	4.442710129	5.156626401	2.868578001	2.77779907	7.091899617	6.301968891
AP5S1	AP5S1	-0.591292888	0.621621222	0.512578007	0.668751914	-0.078714881	8.257427002	7.099070191	5.156626401	6.761173376	6.72891807	7.557113378	8.042267212
ENSG00000125846	ZNF133	0.076002756	0.965988399	-0.155705956	0.931082222	-0.0797032	3.327711234	8.505280662	8.175061096	7.880528731	7.84670947	7.812424466	7.435628703
ENSG00000162946	DISC1	-0.308759446	0.803672304	0.228982632	0.853313931	-0.079776814	4.467311984	7.189546432	5.930865648	5.900763176	6.06344184	6.173421703	6.755525668
ENSG00000139746	RBM26	0.210812497	0.785196727	-0.290969555	0.695197299	-0.080157058	7.624139768	8.414402626	9.180874355	8.727753724	8.77079897	8.3832514	8.110198799
ENSG00000134901	KDELC1	-0.200735699	0.88827382	0.119665379	0.933175306	-0.08107032	9.233563228	7.467835385	5.772716771	7.90310317	7.93600799	8.407640344	8.039482273
ENSG00000162736	NCSTN	0.303259433	0.760220023	-0.384710651	0.690174836	-0.081451218	11.77862605	10.30378101	9.725218844	11.1637228	11.1856376	10.15661401	10.75777696
ENSG00000164088	PPM1M	-1.037700595	0.602587452	0.956219249	0.628501113	-0.081481346	8.998234871	7.819099893	2	6.851641821	7.0150921	9.012898235	8.764090674
ENSG00000148719	DNAJB12	0.286929743	0.658476741	-0.369110368	0.554801672	-0.082180625	8.468948322	9.7032946	9.491321578	9.596407142	9.60461324	8.797335126	9.085838304
ENSG00000198055	GRK6	-0.438010624	0.813586965	0.355269764	0.847870601	-0.08274086	7.624139768	9.235879652	3.369317972	7.602596956	7.6986238	8.458554046	8.405948829
ENSG00000175505	CLCF1	0.689730479	0.702820767	-0.775036065	0.661877164	-0.085305586	10.19198706	6.464272742	5.39244166	9.418058443	9.46739696	7.794921259	8.166591596
ENSG00000196417	ZNF765	0.221614833	0.898841046	-0.308023976	0.855423548	-0.086409143	4.00722531	5.838937808	8.35328246	7.315259362	7.22257674	6.880813179	6.620419619
ENSG00000133131	MORC4	-1.099311311	0.581310291	1.010371243	0.609366282	-0.088940068	2	8.607278467	8.978369997	7.101994243	7.19470132	9.277728661	9.174819346
ENSG00000143971	ETAA1	-0.25784823	0.722734802	0.16888198	0.818792684	-0.08896625	6.89194353	6.844429755	7.97167868	7.080147179	7.06873058	7.682340325	7.268103222
ENSG00000069329	VPS35	0.12725414	0.723898621	-0.216267428	0.524504404	-0.089013289	10.1076228	10.5284131	10.47451889	10.55079452	10.4683133	10.25622378	10.07327514
ENSG00000014216	CAPN1	0.058958566	0.931034311	-0.149087597	0.81957996	-0.090129031	10.50545418	9.969011754	11.10036136	10.65089438	10.6603451	10.16770645	10.68286652
ENSG00000263513	FAM72C	5.09949797	0.224142044	-5.19111587	0.250703287	-0.0916179	2	2	2	5.406080789	5.14724905	2	2
ENSG00000129696	TTI2	-0.098629479	0.932710628	0.006621213	0.995596508	-0.092008266	4.815609471	7.431218269	7.225742779	6.733448311	6.82556437	6.857395343	6.905807071
ENSG00000164237	CMBL	-0.777166665	0.148518474	0.684913234	0.196709806	-0.092253432	8.285626337	7.315436877	7.858115942	7.066878356	7.14145882	8.725241966	8.326688609
ENSG00000185340	GAS2L1	-0.738784008	0.511176757	0.645891117	0.562211	-0.092892891	9.761125267	8.414402626	6.80318718	8.029321023	8.07355593	9.468935198	9.375131848
ENSG00000164172	MOCS2	-0.91551534	0.02176637	0.820962917	0.038452755	-0.094552423	8.009475818	8.276714465	8.742726175	7.474468681	7.46855365	9.148628127	9.220615045
ENSG00000176978	DPP7	0.248337997	0.55246072	-0.344003024	0.388543168	-0.095665027	9.657036304	9.090820894	9.658943166	9.74820345	9.72155655	9.242974864	9.041986855
ENSG00000163293	NIPAL1	-0.552528815	0.838100556	0.455035109	0.863507947	-0.097493706	2	2	4.523675391	3.162721474	2.86123014	3.059555873	4.018014792
ENSG00000089012	SIRPG	3.688652407	0.324700963	-3.788106481	0.356167588	-0.099454074	2	2	2	3.997744386	4.1930041	2.048074405	2
LOC391322		5.090365617	0.225518037	-5.191115802	0.251137486	-0.100750185	2	2	2	5.304221918	5.24368216	2	2
ENSG00000079332	SAR1A	0.037020759	0.946595613	-0.138302831	0.796658198	-0.101282072	11.52728203	10.72273565	10.86096535	11.10164114	11.1336271	10.76122774	11.1039664
ENSG00000102893	PHKB	-0.337609616	0.734347184	0.235865202	0.815278413	-0.101744414	7.435667355	8.848535039	9.647595056	8.540455898	8.59458893	8.710380719	9.464702422
ENSG00000108848	LUC7L3	0.280348145	0.688878059	-0.38289239	0.568416252	-0.102544245	9.097018025	10.47544827	10.24529339	10.31347779	10.3523439	9.798979623	9.533614641
ENSG00000140650	PMM2	1.229317439	0.457169403	-1.332963721	0.406143499	-0.103646281	4.815609471	9.159587375	5.772716771	8.914645924	9.06280517	6.057834398	6.811011855
ENSG00000173786	CNP	0.536250022	0.166335577	-0.639987179	0.087003979	-0.103737156	11.44840376	10.81088865	10.92346452	11.61683606	11.629295	10.43987727	10.45835585
ENSG00000172936	MYD88	0.667055738	0.524245263	-0.771631147	0.445282505	-0.104575409	10.87842776	8.317412869	9.385998894	10.58648927	10.544437	9.070041959	9.194147421
ENSG00000144214	LYG1	1.128863324	0.636158103	-1.236214608	0.599062079	-0.107351284	2	4.11352228	2	3.95996233	3.7864959	2.831336763	2.326754077

ENSG00000172262	ZNF131	-0.167723303	0.88199664	0.060084184	0.957826958	-0.107639119	6.468621203	7.979858146	8.99637432	7.999368766	7.96458672	8.402448739	7.979721237
ENSG00000102858	MGRN1	-0.164980448	0.837724165	0.057127718	0.944157185	-0.10785273	10.58135316	8.981105197	9.859153765	9.739866907	9.825189	9.850086161	10.14369036
ENSG00000147202	DIAPH2	-0.009902157	0.98668938	-0.09815555	0.86473808	-0.108057707	7.667647223	7.638155693	8.408111533	7.947219213	7.91907183	7.992125306	7.68409025
ENSG00000100106	TRIOBP	-0.393773771	0.282435386	0.285201224	0.441391676	-0.108572547	10.84091191	11.0620559	10.48730704	10.4186301	10.4312521	11.00896231	11.19009178
ENSG00000135506	OS9	0.196765202	0.803469715	-0.305510217	0.684964093	-0.108745015	12.12726019	11.32164258	10.5187901	11.6625764	11.6670996	11.04522364	11.2724516
ENSG00000136100	VPS36	0.032194515	0.984117158	-0.141023285	0.932122597	-0.108828771	4.467311984	9.079034257	8.845626171	8.509731121	8.38151133	8.185201128	8.361298015
ENSG00000142655	PEX14	0.048766082	0.912239853	-0.158729418	0.694164121	-0.109963336	8.340423424	8.147241016	8.236946975	8.31397419	8.26379462	7.8927823	8.25407423
ENSG00000164434	FABP7	5.081056982	0.357361029	-5.191085614	0.364514502	-0.110028632	2	2	2	6.18902405	2	2	2
ENSG00000104853	CLPTM1	0.136944888	0.88199664	-0.247599815	0.776294535	-0.110654927	11.65707219	9.71856931	11.00131064	11.12343458	11.1375544	10.6268361	10.85746696
ENSG00000161021	MAML1	-0.142232871	0.798768209	0.031456365	0.956825769	-0.110776506	9.397585735	10.27307349	9.681375057	9.668456061	9.71736428	10.01733654	9.69686815
ENSG00000100023	PPIL2	-0.146465024	0.84752019	0.034517058	0.965258777	-0.111947967	9.2903348	8.029695761	8.175061096	8.486665614	8.44711562	8.489350161	8.787516955
ENSG00000128383	APOBEC3A	3.546549242	0.37096152	-3.659109335	0.391759399	-0.112560093	2	2	2	3.881293667	4.0930011	2	2.059860178
ENSG00000162408	NOL9	-0.198992558	0.739382058	0.082234266	0.89539314	-0.116758292	7.667647223	7.669933291	8.561105505	7.849869274	7.80517502	8.199240359	8.002780183
ENSG00000160959	LRRC14	-0.421822364	0.780343245	0.304887908	0.841557303	-0.116934456	9.33149386	7.503546079	5.156626401	7.72026832	7.77859536	8.52322774	8.405948829
ENSG00000104723	TUSC3	0.743984443	0.336346335	-0.861453029	0.250810834	-0.117468586	11.06895221	9.583268459	9.589475645	10.99738621	11.0105443	9.271730593	9.525676102
ENSG00000138756	BMP2K	0.823005348	0.691466494	-0.94085223	0.641992641	-0.117846882	8.313285031	5.595927442	2	7.772969103	7.72392024	6.057834398	6.037449234
ENSG00000138750	NUP54	0.395870355	0.727489842	-0.51437404	0.640632886	-0.118503685	5.866944937	7.73146271	8.677268317	8.178656739	8.24644267	7.539353852	7.060335194
ENSG00000188295	ZNF669	-0.056530125	0.93987322	-0.062357442	0.933434132	-0.118887567	5.866944937	5.595927442	5.772716771	5.527288686	5.83486613	5.822425633	5.529673353
ENSG00000105518	TMEM205	0.27273544	0.762344209	-0.392492194	0.651136675	-0.119756754	9.112843761	8.671479029	7.163363831	8.693628619	8.89230135	7.903438544	8.34108458
ENSG00000138326	RPS24	-0.056224848	0.960095276	-0.063679825	0.955859616	-0.119904673	12.1882823	10.04368224	11.11951029	11.08473443	11.1095698	11.09800658	11.08146599
ENSG00000120162	MOB3B	0.294559198	0.886587309	-0.414509753	0.834220385	-0.119950555	2	5.131603625	3.369317972	4.443344104	4.1930041	4.209638631	3.183290849
ENSG00000107745	MICU1	0.16171994	0.838100556	-0.281821586	0.705599521	-0.120101646	8.468948322	8.903033433	9.907528187	9.346653666	9.41658653	8.722467118	9.13188655
ENSG00000122435	TRMT13	0.226016381	0.869626608	-0.347389185	0.792195215	-0.121372804	3.327711234	6.602424674	6.203069917	6.139181293	6.14061114	5.72966459	5.470251342
ENSG00000103671	TRIP4	0.250201204	0.827911815	-0.372890939	0.73312167	-0.122689735	8.517314938	7.60566235	5.772716771	7.877998516	7.97632944	7.200910877	7.425709383
ENSG00000183963	SMTN	0.139738834	0.865062033	-0.263940627	0.731726336	-0.124201794	10.81695923	9.235879652	9.809100877	10.16416077	10.3172245	9.839240397	9.841914256
ENSG00000198300	PEG3	-3.104077876	0.151263538	2.979204955	0.14497505	-0.124872922	5.70932728	2	2	2.3892346	2.68924543	7.281044413	6.681239787
ENSG00000189046	ALKBH2	-0.661534528	0.362647422	0.536247735	0.454637787	-0.125286793	5.095959458	6.140560021	6.322058544	5.461211713	5.21224859	6.599583349	6.298867018
ENSG00000206535	LNP1	-0.692243041	0.764891131	0.566756099	0.804586396	-0.125486943	2	4.710564154	2	3.162721474	3.15372745	3.943456545	4.018014792
ENSG00000164749	HNF4G	5.06526085	0.231765386	-5.19111517	0.254503115	-0.12585432	2	2	2	5.391962469	5.09650239	2	2
ENSG00000128534	LSM8	0.250241119	0.613329998	-0.376290351	0.422595617	-0.126049232	8.19931886	7.431218269	7.691296561	8.093978021	7.99953197	7.44824109	7.417152486
ENSG00000166197	NOLC1	-0.351775852	0.747677329	0.225612128	0.839662804	-0.126163724	9.781071289	9.74864196	7.225742779	8.946390023	8.95655043	9.527337514	9.522687851
ENSG00000110436	SLC1A2	1.22415269	0.262567938	-1.35061094	0.206789707	-0.12645825	4.00722531	4.710564154	5.772716771	6.24507773	5.9725602	4.281782022	3.383972333
ENSG00000204065	TCEAL5	5.064183952	0.231292431	-5.191115294	0.253975361	-0.126931342	2	2	2	5.22679461	5.27444544	2	2
ENSG00000119401	TRIM32	-0.617886692	0.387577122	0.490577215	0.49246368	-0.127309477	9.358295023	8.356994601	9.788583153	8.687861792	8.64401667	10.06283648	9.394150543
ENSG00000102119	EMD	-0.660502642	0.156708581	0.532685955	0.253721513	-0.127816687	9.384607364	8.834582582	8.536706852	8.300810597	8.31463236	9.362752343	9.608182402
ENSG00000254402	LRRC24	5.063203033	0.232506713	-5.191115056	0.255070887	-0.127912023	2	2	2	5.058234798	5.41912973	2	2
ENSG00000139631	CSAD	-0.19896804	0.822767511	0.069869286	0.93862683	-0.129098754	5.70932728	7.503546079	7.098165388	6.664650034	6.86652414	7.130090369	6.920008846
ENSG00000186104	CYP2R1	-0.868671148	0.609720423	0.739294327	0.662784961	-0.129376822	6.563806279	5.131603625	2	4.887609717	4.50979031	6.499401217	5.722580018
ENSG00000109103	UNC119	-0.560922838	0.132777783	0.431278991	0.249078729	-0.129643848	8.564112515	8.687092496	8.143093325	7.852449262	8.01555453	8.921491433	8.912005269
ENSG00000154781	CCDC174	-0.028383074	0.981947437	-0.102869885	0.933434132	-0.131252958	6.96351253	8.469613413	5.772716771	7.395290829	7.52080763	7.551912644	7.199901369
ENSG00000106868	SUSD1	0.078161509	0.972415697	-0.212139513	0.926209474	-0.133978004	9.43585023	8.004992147	2	8.276040626	8.5606705	7.853585565	8.376826085
ENSG00000168116	KIAA1586	-0.111413518	0.865906878	-0.028200703	0.966627129	-0.139614221	7.435667355	6.78762695	6.43197674	6.836083475	6.83591385	7.034886728	6.786860422
ENSG00000148834	GSTO1	1.180001383	0.145540049	-1.319626219	0.094172652	-0.139624835	10.87379128	9.090820894	9.372276026	11.17226686	11.1906866	8.512023373	8.854540499
ENSG00000068028	RASSF1	-0.462387907	0.814176064	0.322546449	0.870457209	-0.139841457	9.771132748	7.73146271	3.369317972	8.006336225	8.10598728	8.815677424	8.849779308
ENSG00000105127	AKAP8	0.056492744	0.976731083	-0.197035528	0.918257682	-0.140542784	9.358295023	7.901717006	3.369317972	8.312101014	8.27524772	7.832294085	8.228218854
ENSG00000228300	C19orf24	0.103553021	0.911517345	-0.244497292	0.779935476	-0.140944272	8.910412251	7.274681896	7.502634626	8.100504225	8.26953254	7.802823992	7.885682058
ENSG00000188428	BLOC1S5	-0.775764479	0.588826811	0.634144483	0.660050259	-0.141619996	8.540903468	6.78762695	4.523675391	6.634986713	6.65485813	8.266822833	7.718159092
ENSG00000077514	POLD3	0.244068877	0.888744026	-0.385763569	0.81669225	-0.141694692	3.327711234	7.051611315	8.175061096	7.444001439	7.36516374	7.100474247	6.403650353
ENSG00000196693	ZNF33B	0.732167398	0.582172352	-0.875271313	0.496317147	-0.143103915	4.467311984	7.790474954	6.073378139	7.391747748	7.44845993	6.17883	5.502966356
ENSG00000152518	ZFP36L2	-0.054832111	0.943520433	-0.088403082	0.909584743	-0.143235193	9.667789716	10.28850895	11.15748401	10.42297598	10.4683133	10.32536768	10.49439247
ENSG00000168806	LCMT2	-0.347810519	0.871671212	0.204451173	0.92531104	-0.143359346	5.866944937	8.191702463	2	6.542151749	6.57678965	6.973973146	7.20156326
ENSG00000137815	RTF1	-0.032809666	0.957978247	-0.110780833	0.855882497	-0.143590498	9.33149386	10.35843478	9.798878489	9.858840283	9.86300004	9.579383624	9.961982472
ENSG00000160961	ZNF333	-0.83138998	0.556481113	0.686038205	0.628179702	-0.145351776	3.327711234	7.35507212	7.029880454	5.959382894	5.80356929	7.49992804	7.150854407
ENSG00000163930	BAP1	-0.402677717	0.821801475	0.256859692	0.886860792	-0.145818025	10.62615013	9.814097497	4.874587708	9.279220784	9.33684939	9.99413276	9.934264157
ENSG00000079112	CDH17	1.191350439	0.691746353	-1.339554992	0.65246542	-0.148204553	2	2	3.369317972	3.462030052	2.59489879	2.384001718	2.117335275
ENSG00000133872	SARAF	0.190770447	0.453919948	-0.339878612	0.14757638	-0.149108165	11.40085503	11.35867467	11.337759	11.58267051	11.5298098	10.90501116	11.13897086
ENSG00000130175	PRKCSH	-0.015770433	0.982399308	-0.133680416	0.84468287	-0.149450849	11.91575472	10.67655334	11.07517045	11.25795672	11.3433499	11.07135679	11.28768964
ENSG00000189298	ZKSCAN3	-0.051563396	0.954030819	-0.098965724	0.911774385	-0.15052912	5.532353208	6.951736442	6.534109959	6.442929658	6.39275508	6.68757835	5.968846243
ENSG00000007080	CCDC124	-0.704147357	0.218319116	0.551781169	0.33664227	-0.152366188	8.444143041	9.298208073	8.236946975	8.022463952	8.07355593	9.154129994	9.41686151
ENSG00000089195	TRMT6	-0.054259863	0.953443586	-0.098450355	0.915858934	-0.152710219	7.579279349	5.946638857	7.600047325	6.998639523	7.29187696	6.895669227	7.296373648
ENSG00000099377	HSD3B7	0.118799896	0.946375129	-0.271594625	0.878225373	-0.152794729	3.327711234	7.145017454	8.536706852	7.419851705	7.68723697	7.14536429	7.211494604
ENSG00000114209	PDCD10	0.724515808	0.09063243	-0.877630512	0.033859643	-0.153114704	8.586954094	7.874700259	8.486635645	9.085573664	9.0282492	7.565395753	7.382408159
ENSG00000113318	MSH3	-0.226755965	0.866211938	0.073611841	0.956916625	-0.153144124	8.540903468	7.572420273	5.156626401	7.467753569	7.36873214	7.538302335	7.869046644
ENSG00000101126	ADNP	-0.388176227	0.262259514	0.234373629	0.510878389	-0.153802599	10.16187685	10.17682307	9.963514551	9.718812329	9.71806384	10.54229576	10.10114286
ENSG00000141646	SMAD4	-0.16174422	0.798465488	0.007556273	0.99072569	-0.154187947	8.418903792	9.497318007	9.358421373	9.067921572	8.94225201	9.314738721	9.019079808
ENSG00000136522	MRPL47	0.170765628	0.857034032	-0.325413179	0.714828755	-0.154647551	8.564112515	6.602424674	8.110401118	8.168339746	8.09094333	7.486906381	7.782864551
ENSG00000124116	WFDC3	2.298319033	0.309909983	-2.453493777	0.278679003	-0.155174743	2	4.11352228	2	4.497095146	5.06165168	2.094598369	2.466008509
ENSG00000171847	FAM90A1	-3.753008627	0.531143362	3.596315706	0.521375485	-0.15669292	2	2	2	2	2	3.648325314	4.329022995
ENSG00000159023	EPB41	-0.411845891	0.775566336	0.254314532	0.862994354	-0.157531358	5.532353208	7.538394143	9.358421373	7.786523606	7.81828345	8.66581901	8.2175813
ENSG00000090861	AARS	-0.549024616	0.434146426	0.391243777	0.580587983	-0.157780839	11.04027452	10.47993704	9.69246155	9.930185944	9.98658999	11.22245533	10.47373855
ENSG00000004487	KDM1A	-0.247815143	0.555167478	0.086833785	0.846443289	-0.160981358	9.219014269	9.785372135	9.859153765	9.376537567	9.42860336	9.67529364	9.793698997
ENSG00000062194	GPBP1	0.365033286	0.302694048	-0.526044964	0.115721683	-0.161011678	9.922475255	9.923599762	9.809100877	10.26878646	10.2316302	9.581682124	9.105499915
ENSG00000100504	PYGL	-0.346029015	0.887450188	0.184317181	0.939787567	-0.161711833	10.07567912	7.431218269	2	8.404661252	8.32752521	8.850830124	8.931163478
ENSG00000110042	DTX4	0.012807243	0.996622494	-0.17466616	0.942414968	-0.161858917	3.327711234	7.232742069	2	5.808110275	5.80356929	5.206928476	5.96101835
ENSG00000176890	TYMS	0.413780593	0.785196727	-0.576516307	0.692374562	-0.162735714	4.00722531	6.78762695	4.058998049	6.024891728	5.96314905	5.136410075	5.04340913
ENSG00000152455	SUV39H2	-0.115440766	0.912408896	-0.048301005	0.963748226	-0.163741771	4.467311984	6.464272742	5.59507254	5.651002631	5.64780787	5.737015686	5.685057129
ENSG00000261456	TUBB8	2.904451352	0.182906893	-3.068766981	0.176029831	-0.164315629	2	3.686303414	2	5.076005082	4.60967607	2.048074405	2.225841198
ENSG00000137824	RMDN3	-0.285236357	0.859119352	0.11949187	0.941500505	-0.165744487	9.423197387	9.055168103	4.874587708	8.409925372	8.42842973	8.724779862	8.908956896
ENSG00000135372	NAT10	-0.015488382	0.983101649	-0.150577523	0.830158879	-0.166065905	7.624139768	8.147241016	8.941672936	8.341783829	8.30162324	8.091532339	8.279474384
ENSG00000102393	GLA	0.915968831	0.261009892	-1.082848018	0.169103768	-0.166879186	9.233563228	8.916342327	7.225742779	9.586367937	9.60688128	7.624061799	7.615934347
ENSG00000001631	KRIT1	1.112915557	0.009363387	-1.281493089	0.002330612	-0.168577532	7.031698319	7.847167909	7.646393253	8.624367384	8.68323472	6.339575235	6.298867018
ENSG00000124251	TP53TG5	-3.753008776	0.531143362	3.584371155	0.52282666	-0.168637621	2	2	2	2	2	4.065262523	3.972018587
ENSG00000134882	UBAC2	-0.639763313	0.293257156	0.470414923	0.443446023	-0.16934839	9.87686338	8.702538795	9.859153765	8.957217624	8.91077722	10.12998797	9.939751019
ENSG00000166024	R3HCC1L	0.716930081	0.731288333	-0.887331173	0.663750141	-0.170401093	7.159102577	8.993716073	2	8.399377854	8.56534297	7.08038692	6.723495198
ENSG00000010322	NISCH	-0.410757721	0.630961153	0.239934587	0.785559516	-0.170823134	11.29385609	9.77810053	9.647595056	10.00165982	10.0733424	10.61790491	10.75225974
ENSG00000164344	KLKB1	5.019901363	0.241359928	-5.191114409	0.258315695	-0.171213046	2	2	2	5.161714397	5.2591458	2	2
ENSG00000140157	NIPA2	0.368795086	0.607262407	-0.540103428	0.427903866	-0.171308342	9.771132748	9.656475478	8.325066185	9.763363111	9.73820484	8.766685705	8.924637944
ENSG00000188785	ZNF548	-0.487132927	0.539576503	0.315570148	0.696257748	-0.171562779	7.750913381	6.899080544	6.203069917	6.529309835	6.70088158	7.655436363	7.076727469
ENSG00000112685	EXOC2	0.070767565	0.925390417	-0.24296372	0.728500949	-0.172196155	8.075571721	8.395519306	9.399592459	8.814994458	8.79619691	8.548515606	8.439433377
ENSG00000005020	SKAP2	1.322376003	0.146554142	-1.496790599	0.091551895	-0.174414596	5.095959458	6.951736442	7.502634626	8.096156705	8.12931531	5.60262961	5.197927245
ENSG00000198732	SMOC1	-0.897966126	0.642184909	0.722898539	0.708681786	-0.175067588	2	6.951736442	4.874587708	5.22679461	4.53541753	6.877490957	5.708623051
ENSG00000119655	NPC2	0.469571185	0.653653157	-0.645079769	0.521361073	-0.175508584	10.73752355	9.170736405	8.296287027	10.21566945	10.2350555	9.082719462	9.149213283
ENSG00000164597	COG5	-0.407417581	0.282232621	0.22971684	0.555749022	-0.177700741	8.777722034	9.00621767	8.805342542	8.540455898	8.38855922	9.286209561	8.880709189
ENSG00000030110	BAK1	0.311070522	0.694579859	-0.489644477	0.5142789	-0.178573955	8.16936288	8.590771778	6.882775709	8.361842177	8.35477078	7.57465694	7.566491953
ENSG00000117676	RPS6KA1	-0.172718641	0.92551187	-0.006050293	0.997764007	-0.178768934	3.327711234	7.503546079	4.874587708	5.880681751	6.21386141	6.587434478	5.708623051
ENSG00000178685	PARP10	-6.445436812	0.00487994	6.266656504	4.62E-05	-0.178780308	2	4.442710129	2	2	2	9.024959988	8.967756908
ENSG00000130338	TULP4	-0.104915675	0.807865742	-0.074460146	0.863495873	-0.179375821	8.910412251	9.488434476	9.099922433	9.126651551	9.04841459	9.051735782	9.18199253
ENSG00000160113	NR2F6	-0.588957269	0.708284536	0.408052604	0.798191812	-0.180904665	9.556468883	10.02537381	5.156626401	8.709369553	8.63070345	9.650204809	9.670710139
ENSG00000121236	TRIM6	-0.697825074	0.682343107	0.514098307	0.764313677	-0.183726767	3.327711234	4.710564154	7.502634626	5.651002631	5.37722731	6.678062013	6.640162436
ENSG00000159885	ZNF222	0.382712088	0.826909189	-0.56657688	0.733788685	-0.183864792	2	6.535001748	6.534109959	6.401247806	6.29111322	5.680947153	5.20457242
ENSG00000168818	STX18	-0.138402314	0.828815955	-0.045520675	0.944101725	-0.183922989	7.579279349	8.356994601	7.502634626	7.734324667	7.7515168	7.600074836	8.035760639
ENSG00000187824	TMEM220	-0.012661715	0.993765388	-0.171668597	0.906716859	-0.184330313	4.467311984	6.666836837	4.058998049	5.674521485	5.45984938	5.096960836	5.694529741
ENSG00000124541	RRP36	-0.059332324	0.960501625	-0.127534968	0.916470603	-0.186867292	9.699577239	8.88960062	6.80318718	8.814994458	8.83669913	8.501810558	8.976033998
ENSG00000100532	CGRRF1	-0.213919403	0.911376403	0.026289302	0.988820823	-0.187630101	2	7.189546432	7.098165388	6.372775472	6.3857277	6.752490997	6.449128733
ENSG00000172465	TCEAL1	-0.533120785	0.377368801	0.343869441	0.574881251	-0.189251344	7.27616306	7.002537973	8.076950877	7.101994243	6.88158913	7.77631144	7.924438973
ENSG00000181929	PRKAG1	-0.409735831	0.273150492	0.220311444	0.569975156	-0.189424388	9.064836028	9.616264339	9.272377397	8.923261356	8.94464494	9.643863831	9.470572512
ENSG00000143486	EIF2D	-0.420515895	0.436019907	0.230238162	0.679605718	-0.190277733	8.367060731	7.761270542	8.763901704	7.978262192	7.88209639	8.498029643	8.644559262

ENSG00000130726	TRIM28	-0.318138124	0.721422916	0.12564137	0.893463941	-0.192496754	10.38028415	11.54799449	9.61300511	10.41208663	10.4248388	10.96542343	10.74871193
ENSG00000115685	PPP1R7	0.245583982	0.713873535	-0.438890974	0.484010617	-0.193306992	9.262228249	8.433041974	8.007629717	8.887228971	8.92538942	8.110758945	8.33882106
ENSG00000181035	SLC25A42	-0.909432098	0.282224272	0.715033388	0.397171615	-0.19439871	8.285626337	6.899080544	6.43197674	6.5612028	6.53293476	8.187215117	8.068914703
ENSG00000142046	TMEM91	0.359423836	0.803441373	-0.55426411	0.686301227	-0.194840274	6.366709671	4.11352228	4.058998049	5.709096143	5.45984938	5.119635168	4.329022995
ENSG00000160679	CHTOP	-0.237742771	0.818399248	0.042260431	0.968580178	-0.195482341	7.579279349	9.916994052	9.031722608	8.892252782	8.90341524	9.366311982	8.955990164
ENSG00000170075	GPR37L1	-0.934829896	0.666836354	0.736927311	0.728110981	-0.197902584	3.327711234	2	4.058998049	2.695456494	2.94009902	3.035909414	4.279527956
ENSG00000205456	TP53TG3D	2.689899487	0.584143941	-2.887860292	NA	-0.197960805	2	2	2	3.462030052	3.33976443	2.094598369	2
ENSG00000172508	CARNS1	-1.877744509	0.239916541	1.679545043	0.27708601	-0.198199466	5.70932728	3.686303414	2	3.289987342	3.08597292	6.249924465	5.722580018
ENSG00000180884	ZNF792	0.844886219	0.634552412	-1.046156816	0.543191241	-0.201270597	3.327711234	4.442710129	7.734844356	7.182120794	7.1246732	5.381251181	5.355511328
ENSG00000106804	C5	0.05731432	0.98350331	-0.260451927	0.926209474	-0.203137607	2	2	6.203069917	4.867343068	4.72553021	4.5389195	4.522269241
ENSG00000109339	MAPK10	-1.79975479	0.19577655	1.596489171	0.239770681	-0.203265618	6.009025997	5.303523747	2	3.663521627	3.7864959	6.247352872	6.983208048
ENSG00000175106	TVP23C	2.293573275	0.0383061	-2.496989556	0.022406102	-0.203416281	6.366709671	4.93638933	8.143093325	9.305820479	9.2754408	4.742549839	4.711446447
ENSG00000161714	PLCD3	-1.192254961	0.362599152	0.988693842	0.450551283	-0.203561119	10.38028415	9.470501606	6.322058544	8.239055745	8.32935768	10.226175	10.64611413
ENSG00000278129	ZNF8	-0.717489598	0.772450402	0.511136166	0.839017584	-0.206353432	8.540903468	3.686303414	2	6.29144905	6.36443784	7.646681011	7.370638252
ENSG00000197019	SERTAD1	0.417988553	0.645473237	-0.625087887	0.469777313	-0.207099334	7.709880939	7.274681896	9.149036566	8.699372486	8.673169	7.710619897	7.603417442
ENSG00000111339	ART4	-3.753009283	0.531143362	3.543772668	0.52769064	-0.209236615	2	2	2	2	2	3.584558147	4.30448772
ENSG00000108039	XPNPEP1	-0.178410127	0.725609862	-0.030901903	0.954650147	-0.20931203	9.384607364	10.03150247	9.413059136	9.393466757	9.53961588	9.406340618	9.797550144
ENSG00000101132	PFDN4	0.359400783	0.806299763	-0.570489539	0.682815119	-0.211088756	7.904282243	6.04685525	4.523675391	7.214570819	7.01963893	6.635427934	5.6562603
ENSG00000111291	GPRC5D	-2.312787877	NA	2.101534343	NA	-0.211253534	2	2	2	2	2.14022021	2.656294034	3.383972333
ENSG00000087269	NOP14	-0.017273546	0.983773789	-0.195530034	0.811305502	-0.21280358	8.19931886	8.053983481	9.528916236	8.7747563	8.69607402	8.531177917	8.587232804
ENSG00000069399	BCL3	-0.217029673	0.829171998	0.003519938	0.99765513	-0.213509735	7.33131396	7.669933291	9.180874355	7.975897842	8.17894021	7.968899389	8.566746948
ENSG00000163444	TMEM183A	0.14796585	0.770265142	-0.361744671	0.43609424	-0.213778821	8.946185348	8.213429689	8.978369997	8.898508045	8.88608977	8.406488267	8.368713182
ENSG00000174903	RAB1B	-0.050492189	0.911631829	-0.163441364	0.694750517	-0.213933553	10.16946352	10.54564457	10.73794767	10.43679547	10.468729	10.16430247	10.49710157
ENSG00000178449	COX14	-0.373011824	0.364106676	0.158041123	0.715368383	-0.214970701	8.009475818	8.337339476	7.818184403	7.778406195	7.64666005	8.14846013	8.319819
ENSG00000169508	GPR183	0.451986539	0.772510709	-0.667748816	0.657862568	-0.215762277	2	5.303523747	4.874587708	5.022023783	5.00774437	4.397636241	3.698365557
ENSG00000244754	N4BP2L2	-0.284859376	0.561250332	0.067541064	0.899650959	-0.217318312	10.03472846	9.308339287	9.725218844	9.466018907	9.40011621	9.995091105	9.536580367
ENSG00000112651	MRPL2	-0.194087928	0.695551109	-0.023571064	0.965258777	-0.217658992	7.667647223	7.701025996	7.163363831	7.178012736	7.5014335	7.491260006	7.539150216
ENSG00000196517	SLC6A9	0.15427454	0.922635322	-0.373485386	0.804586396	-0.219210847	9.886101849	6.78762695	6.629488387	8.636393836	8.83798691	8.464655389	7.922424886
ENSG00000121931	LRIF1	1.010778063	0.21521423	-1.230076257	0.117872694	-0.219298193	8.418903792	7.274681896	7.225742779	8.756965077	8.73530306	7.037865927	5.821002687
ENSG00000103356	EARS2	-0.715844376	0.651333637	0.495853842	0.758228816	-0.219990534	9.064836028	8.451443573	4.058998049	7.454228844	7.60187978	8.750886382	8.696162343
ENSG00000164056	SPRY1	-0.672693996	0.490423307	0.450711093	0.645796163	-0.221982903	4.00722531	6.140560021	5.930865648	5.076005082	4.98931816	6.148830293	5.96101835
ENSG00000170631	ZNF16	-0.686798113	0.424587993	0.463814822	0.592366323	-0.222983291	5.095959458	7.051611315	6.718950348	5.920568914	5.81407719	6.913621294	7.011905845
ENSG00000130312	MRPL34	-0.538931517	0.233859725	0.315316476	0.498844659	-0.223615041	9.081016759	8.433041974	8.325066185	8.134820652	8.10170499	8.972986183	8.953526633
ENSG00000169241	SLC50A1	-0.136979759	0.918959987	-0.086712743	0.948481065	-0.223692502	6.366709671	9.695596167	8.35328246	8.47833774	8.63367258	8.55268749	8.660959882
ENSG00000165487	MICU2	0.565512876	0.113360191	-0.790357671	0.020227058	-0.224844795	7.750913381	7.638155693	7.691296561	8.190940526	8.30348889	6.828438696	7.010010317
ENSG00000112599	GUCA1B	0.543759983	0.786662631	-0.769331037	0.689804433	-0.225571054	3.327711234	2	4.523675391	4.17310023	3.69849104	3.374372158	2.782576693
ENSG00000173898	SPTBN2	0.053083641	0.969053019	-0.279742031	0.83342673	-0.226658391	6.73718411	8.57407404	10.15199903	9.174861605	9.09008229	8.779201757	8.826273465
ENSG00000111229	ARPC3	0.243193657	0.766736242	-0.469982289	0.540654844	-0.226788633	10.83137851	9.34816733	9.465702942	10.24689154	10.334663	9.493286157	9.664095852
ENSG00000129009	ISLR	-1.873353696	0.108536572	1.64613969	0.156194904	-0.227214005	7.27616306	9.386925327	10.58572549	7.68596357	7.84158259	10.46000723	11.77297383
ENSG00000112576	CCND3	0.209151165	0.739140058	-0.437017365	0.454500807	-0.2278662	9.940323746	10.19331737	8.99637432	9.975862031	10.0345549	9.349025111	9.375867991
ENSG00000113648	H2AFY	-0.323209233	0.507503623	0.093821546	0.857692232	-0.229387687	10.00958825	9.930175364	10.70548138	9.95800436	9.91151326	10.2789076	10.41871938
ENSG00000164086	DUSP7	-1.235104683	0.011460822	1.005296744	0.041611142	-0.229807939	9.304185368	8.276714465	8.805342542	7.605658789	7.65837038	9.972302634	9.715307813
ENSG00000119965	C10orf88	-0.251712227	0.908179413	0.021177901	0.992406225	-0.230534327	2	7.099070191	8.585098385	7.206526483	7.20272083	7.775419242	7.083953563
ENSG00000178734	LMO7DN	-2.304491744	NA	2.073147501	NA	-0.231344243	2	2	2	2.141737246	2	2.094598369	3.620995423
ENSG00000154240	CEP112	0.255500264	0.898841046	-0.487606773	0.798598135	-0.232106509	2	6.228547981	7.691296561	6.912249745	6.71216164	5.893224226	6.29264319
ENSG00000164037	SLC9B1	-0.065088061	0.975808161	-0.168287498	0.936648304	-0.233375559	4.00722531	2	3.369317972	3.162721474	3.33976443	3.44788957	2.885414003
ENSG00000138668	HNRNPD	0.064090442	0.930344224	-0.297657877	0.658096973	-0.233567435	10.26462289	9.415325457	9.788583153	9.933844998	9.92065856	8.953791814	9.99637663
ENSG00000096060	FKBP5	-0.207604329	0.720126758	-0.026482445	0.966296927	-0.234086773	6.653098118	7.232742069	6.534109959	6.535745081	6.77267312	6.795372357	6.870723585
ENSG00000169375	SIN3A	-0.083808217	0.936411459	-0.150403035	0.885109991	-0.234211252	7.750913381	9.763446433	10.03493575	9.351221511	9.43031191	9.182002376	9.455329967
ENSG00000165006	UBAP1	0.003456296	0.997041173	-0.238403072	0.765637652	-0.234946776	10.75275473	9.055168103	9.878698603	10.02643329	10.0928914	9.852414792	9.785134696
ENSG00000261992		2.570211224	0.020861927	-2.805997252	0.011198174	-0.235786027	4.00722531	7.099070191	5.156626401	8.454760653	8.53866332	3.5515855	3.753786374
ENSG00000143869	GDF7	-3.75300962	0.531143362	3.516785402	0.530948199	-0.236224218	2	2	2	2	2	4.099885429	3.824516949
ENSG00000223547	ZNF844	0.1068001	0.967125696	-0.344782889	0.893909641	-0.237982788	9.112843761	4.442710129	2	7.688853661	7.70428369	7.593002849	6.813187523
ENSG00000163162	RNF149	1.004383823	0.150830829	-1.242581671	0.064830738	-0.238197848	9.031919741	8.702538795	10.21486071	10.47329248	10.4624805	8.284489899	8.186029473
ENSG00000160208	RRP1B	-0.608056259	0.465935157	0.369851433	0.665677302	-0.238204826	7.33131396	8.433041974	9.287079172	7.952038749	7.96222667	8.97687222	8.868207441
ENSG00000128708	HAT1	-0.124151001	0.765831172	-0.114623698	0.78199021	-0.238774699	8.493334308	8.557180776	8.922968322	8.508095759	8.57927024	8.680667534	8.413855585
ENSG00000266964	FXYD1	-0.682576334	0.844391096	0.443615594	NA	-0.238960741	2	3.076665209	2	2.600425781	2.14022021	3.035909414	2.374681325
ENSG00000077232	DNAJC10	0.048098393	0.945987763	-0.2888739	0.661912042	-0.240775507	9.820154628	10.70750522	11.16943577	10.71219168	10.7184845	10.2683239	10.48230911
ENSG00000154719	MRPL39	0.00963404	0.991315828	-0.251874214	0.757701514	-0.242240174	7.159102577	7.145017454	5.59507254	6.687949235	6.93069905	6.668482487	6.449128733
ENSG00000144199	FAHD2B	0.28701399	0.843262224	-0.530019742	0.697571096	-0.243005751	7.218819781	5.131603625	4.058998049	6.29144905	6.36443784	5.553513572	5.576534358
ENSG00000196072	BLOC1S2	0.402554446	0.643136765	-0.646919484	0.431126874	-0.244365038	9.448362284	7.761270542	7.97167868	8.964391141	9.03275482	7.893604812	8.026414333
ENSG00000163114	PDHA2	2.49907863	0.47708865	-2.743628696	NA	-0.244550067	2	2	2	3.515101962	3.01487888	2.048074405	2.059860178
ENSG00000170275	CRTAP	-0.436085469	0.329070084	0.191107576	0.686807452	-0.244977894	12.05742222	11.2524908	11.87895445	11.31705777	11.3474248	11.97640709	11.94082017
ENSG00000120458	MSANTD2	0.088026536	0.977429905	-0.333130707	0.914220926	-0.245104171	2	8.255926468	2	6.772115916	6.82556437	6.809387508	5.812327524
ENSG00000115112	TFCP2L1	-2.272298728	0.421397646	2.02689433	0.408802956	-0.245404399	3.327711234	2	2	2.141737246	2.14022021	3.724264246	3.45257121
ENSG00000165699	TSC1	0.103088394	0.941409766	-0.349713884	0.794078028	-0.24662549	6.468621203	10.13758676	9.491321578	9.430198858	9.44390794	9.052472506	8.91909319
ENSG00000119650	IFT43	-0.16868943	0.825992773	-0.077948508	0.920635926	-0.246637938	6.89194353	8.169643011	7.098165388	7.43025113	7.26914476	7.402744819	7.477384137
ENSG00000142731	PLK4	0.060189533	0.981829576	-0.308364472	0.903920298	-0.248174939	2	5.595927442	2	4.329433326	4.31639476	4.111244182	3.956354764
ENSG00000103995	CEP152	0.032510219	0.987070736	-0.281992007	0.886770342	-0.249481788	7.159102577	5.303523747	2	6.104969671	5.85535975	6.075339068	5.171036168
ENSG00000006704	GTF2IRD1	0.214170463	0.908850677	-0.464133024	0.792727651	-0.249962561	8.910412251	8.147241016	3.369317972	8.229163214	8.22100445	7.887011553	7.131805326
ENSG00000185024	BRF1	-0.270858864	0.593368805	0.019857579	0.971342629	-0.251001284	7.485143553	8.297207175	8.206335842	7.748245381	7.80780625	8.135306947	7.989373961
ENSG00000106344	RBM28	-0.548783563	0.287337376	0.29731032	0.576448639	-0.251473242	8.285626337	7.393647515	8.206335842	7.477814553	7.45182838	8.223648538	8.365751687
ENSG00000157349	DDX19B	0.607799866	0.356507018	-0.859663141	0.168742422	-0.251863275	8.39321512	7.761270542	7.029880454	8.578353644	8.26953254	6.860764111	7.116030435
ENSG00000131778	CHD1L	0.209072136	0.765491992	-0.461261219	0.476851144	-0.252189083	8.042902253	9.00621767	9.212024683	8.946390023	9.13582529	8.033758684	8.659751394
ENSG00000130714	POMT1	-0.200339662	0.895675696	-0.053302122	0.972083517	-0.253641784	6.468621203	9.821190352	7.098165388	8.29892024	8.42671895	8.586652837	8.428121416
ENSG00000164024	METAP1	-0.002955704	0.997204246	-0.254087282	0.748418682	-0.257042986	9.410448395	8.687092496	7.691296561	8.696503411	8.81332035	8.551124447	8.462487876
ENSG00000131174	COX7B	-0.148225362	0.877236903	-0.109803382	0.908314446	-0.258028745	10.00110988	8.029695761	8.941672936	9.077877436	9.04395769	8.986927198	9.20330054
ENSG00000196505	GDAP2	0.060586962	0.929816715	-0.319688245	0.605467095	-0.259101283	7.159102577	7.701025996	8.296287027	7.770242853	7.92393105	7.644728141	7.285445324
ENSG00000109390	NDUFC1	0.593314518	0.27254373	-0.852761818	0.095706512	-0.2594473	7.867441304	7.099070191	6.958202216	7.913023977	7.97164382	6.442083733	6.62538071
ENSG00000043462	LCP2	-3.75300991	0.531143362	3.49355199	0.534071787	-0.25945792	2	2	2	2	2	4.416072028	3.261853426
ENSG00000077684	JADE1	-1.447264575	0.000515432	1.185162936	0.004574546	-0.262101638	8.517314938	8.101365653	8.110401118	6.907295206	6.76727577	9.678399287	9.125772694
ENSG00000103187	COTL1	-0.031438266	0.962823674	-0.231211623	0.716517864	-0.262649889	10.30651143	9.608085847	10.81097638	10.26394956	10.3149142	9.889995187	10.26666875
ENSG00000171302	CANT1	-0.116940358	0.911517345	-0.1472908	0.886197127	-0.264231159	9.730679447	8.791896851	11.0057616	9.910512694	10.096124	9.891025241	10.05638425
ENSG00000139921	TMX1	0.636548941	0.357278052	-0.900960221	0.169365312	-0.26441128	9.233563228	9.7032946	8.143093325	9.842062221	9.7546632	8.242494717	8.304435067
ENSG00000132005	RFX1	-0.215216665	0.885711022	-0.049397118	0.97326848	-0.264613783	8.313285031	7.232742069	4.523675391	7.114945424	7.17852729	7.648631242	6.862343163
ENSG00000171103	TRMT61B	-0.254571096	0.82798291	-0.01036649	0.993322572	-0.264937586	5.095959458	6.535001748	7.777116083	6.598566398	6.60126376	6.845542321	6.824016918
ENSG00000104738	MCM4	0.50220602	0.76909522	-0.767152561	0.640432051	-0.264946541	7.975256505	8.747909548	3.369317972	8.336264621	8.36015869	7.021403672	7.18149309
ENSG00000132432	SEC61G	0.840272721	0.482249983	-1.106697088	0.33238218	-0.266424367	9.624286623	7.145017454	7.029880454	9.296376788	9.2990102	7.366471135	7.386797235
ENSG00000103591	AAGAB	-0.099901681	0.957170231	-0.16841465	0.929020974	-0.26831633	9.975371636	8.929529568	4.058998049	8.908460392	8.84568954	8.648868792	8.954019676
ENSG00000213762	ZNF134	-0.433201056	0.598164094	0.164876005	0.849628112	-0.268325051	8.138771685	7.503546079	9.242516611	7.987681034	8.09740995	8.691586261	8.574463273
ENSG00000186716	BCR	-0.422862257	0.58752019	0.152757637	0.853263424	-0.270104621	9.90440318	9.298208073	8.206335842	8.799033614	8.94703391	9.258063226	9.61318568
ENSG00000164062	APEH	-0.486505286	0.342227985	0.213843628	0.692464563	-0.272661657	9.317904228	9.664384941	8.721235202	8.813671111	8.80016655	9.338794282	9.649254128
ENSG00000204366	ZBTB12	4.918259755	0.264205073	-5.19111239	0.268308947	-0.272852634	2	2	2	5.144975053	5.09650239	2	2
ENSG00000162341	TPCN2	-0.149570362	0.92127192	-0.12342748	0.935063964	-0.272997842	4.467311984	7.145017454	8.380957451	7.114945424	7.32531744	7.024410815	7.444077031
ENSG00000143303	RRNAD1	-0.389838099	0.850552412	0.116069491	0.956087085	-0.273768608	8.444143041	7.145017454	2	7.03996889	6.92586261	7.57363085	7.372114752
ENSG00000103653	CSK	-0.249590421	0.818844433	-0.024695377	0.982605796	-0.274285798	7.709880939	8.376385542	10.01740919	8.707945635	8.86603107	9.033940582	8.990045056
ENSG00000122122	SASH3	-3.753010097	0.531143362	3.478557533	0.53589394	-0.274452564	2	2	2	2	2	4.388329244	3.287118176
ENSG00000167535	CACNB3	-0.535222119	0.462870065	0.259076474	0.731726336	-0.276145645	8.540903468	7.901717006	9.426401271	8.266399315	8.18097132	9.145869306	8.86086441
ENSG00000085998	POMGNT1	-0.14809326	0.929222345	-0.1288043	0.938226757	-0.27689756	6.138360429	9.814097497	10.9743126	9.788505066	9.83037437	9.63945754	9.996137262
ENSG00000131748	STARD3	0.077710168	0.918662967	-0.355023574	0.605467095	-0.277313406	9.344956677	8.820493869	7.896971941	8.817637515	8.94344897	8.576956626	8.318287967
ENSG00000050426	LETMD1	0.028909185	0.981338611	-0.306490148	0.78794815	-0.277580962	6.563806279	8.191702463	9.330306409	8.361842177	8.53549186	8.311822576	7.8990585
ENSG00000198833	UBE2J1	-0.631823285	0.018342641	0.353588945	0.208031037	-0.27823434	10.03472846	10.40166101	10.23774519	9.575477782	9.63530531	10.58174434	10.5931302
ENSG00000160062	ZBTB8A	0.06366546	0.921327192	-0.342856776	0.548805363	-0.279191316	6.563806279	7.099070191	7.600047325	7.206526483	7.20671394	6.916861442	6.700172923
ENSG00000127337	YEATS4	-0.294045454	0.778918686	0.014299537	0.989360801	-0.279745917	6.366709671	6.04685525	4.058998049	5.461211713	5.58783529	5.776790516	5.842465082
ENSG00000178913	TAF7	0.050843435	0.94878661	-0.331012808	0.656735751	-0.280169373	11.32815351	10.46189757	9.757248847	10.71183653	10.7051385	10.45207267	10.19151462
ENSG00000165650	PDZD8	-0.742302471	0.025103126	0.461880925	0.177835207	-0.280421546	9.423197387	9.079034257	9.553445883	8.695066731	8.54970888	9.898624863	9.736956851
ENSG00000197417	SHPK	0.531164676	0.711436164	-0.813510386	0.550336725	-0.28234571	7.218819781	6.04685525	3.369317972	6.699459234	6.77805036	5.217477917	5.694529741

ENSG00000075975	MKRN2	-0.506629535	0.182413091	0.22241316	0.579833528	-0.284216375	9.128497779	8.671479029	9.099922433	8.394075037	8.54970888	9.32239471	9.058587737
ENSG00000136527	TRA2B	0.195146288	0.899681269	-0.479737451	0.73847616	-0.284591162	10.31337612	9.214490408	6.073378139	9.579376566	9.47155148	8.567710236	9.096596023
ENSG00000124486	USP9X	-0.274949579	0.732066351	-0.010297947	0.990275412	-0.285247525	10.37373127	12.01813185	11.91252756	11.34022038	11.3165787	11.58011589	11.6052181
ENSG00000117614	SYF2	0.826514757	0.35107637	-1.112132153	0.188112133	-0.285617395	9.174465797	7.393647515	7.451345288	9.095408867	9.05839257	7.241533944	7.091143645
ENSG00000170345	FOS	0.801000683	0.531497817	-1.087323117	0.374771224	-0.286322434	7.709880939	6.311476754	5.59507254	7.632926745	7.54308677	6.473229652	4.388595494
ENSG00000104870	FCGRT	-0.708525534	0.01449096	0.421708717	0.162812823	-0.286816817	10.45668146	10.01303782	10.28245311	9.535899247	9.57170671	10.65512272	10.70441818
ENSG00000197380	DACT3	-1.069680083	0.461552688	0.782834549	0.591158809	-0.286845535	5.532353208	8.505280662	5.156626401	6.155987605	6.23747437	8.123435792	7.846917442
ENSG00000138640	FAM13A	0.366463059	0.651205609	-0.653475363	0.388123757	-0.287012304	6.96351253	8.505280662	7.777116083	8.323303759	8.17486938	6.854018691	7.561323797
ENSG00000135373	EHF	4.344324678	0.119484573	-4.631915205	0.096576281	-0.287590527	2	2	5.930865648	8.512996295	8.66303256	2	2.466008509
ENSG00000180929	GPR62	0.312648954	0.920079197	-0.600961736	NA	-0.288312782	2	3.076665209	2	2.695456494	2.68924543	2.526562374	2.277179783
ENSG00000197405	C5AR1	-2.127526704	0.415078879	1.838185114	0.475101652	-0.28934159	2	2	6.629488387	3.289987342	3.65239003	7.552954291	5.475755622
ENSG00000099995	SF3A1	-0.473480464	0.332121646	0.180953862	0.728484312	-0.292526602	10.03472846	9.532315021	10.46808205	9.58946437	9.58480612	10.19698705	10.27951104
ENSG00000105993	DNAJB6	-0.080799685	0.91483416	-0.214222638	0.762852238	-0.295022323	9.761125267	9.328390744	10.66665689	9.972901061	9.92065856	9.970159828	9.639171944
ENSG00000144063	MALL	2.329003389	0.210602238	-2.624472601	0.169780322	-0.295469212	3.327711234	3.076665209	2	4.598938466	4.31639476	2.267001569	2.117335275
ENSG00000099875	MKNK2	-0.434975865	0.506870806	0.139355994	0.842279911	-0.295619871	9.128497779	9.956182102	10.27509734	9.363030664	9.49296422	10.30943039	9.608182402
ENSG00000095066	HOOK2	-0.740354847	0.485315834	0.441378876	0.684334335	-0.298975971	5.095959458	7.274681896	5.59507254	5.731694	5.53799497	6.633459761	6.864442837
ENSG00000105755	ETHE1	1.014608558	0.588479045	-1.313687866	0.466340006	-0.299079309	9.931427102	7.819099893	3.369317972	9.685196338	9.64637295	7.160478198	7.545707309
ENSG00000140943	MBTPS1	-0.236671143	0.758253496	-0.063604667	0.937496618	-0.300275809	9.473095744	10.72651826	11.04521411	10.32930776	10.3121369	10.35149484	10.62242656
ENSG00000101439	CST3	-0.317544985	0.382104545	0.014747639	0.971501984	-0.302797346	12.06760845	11.65309132	11.74160414	11.47105152	11.5564566	11.65521291	12.01499609
ENSG00000184517	ZFP1	-0.579695524	0.280922232	0.276018159	0.621070373	-0.303677365	6.73718411	6.951736442	6.80318718	6.237202172	6.32824132	7.350983915	6.808832901
ENSG00000081148	IMPG2	0.592108463	0.82454488	-0.896362502	0.724892691	-0.304254039	2	2	4.058998049	2.947924294	3.60476711	2.592885947	2.632958241
ENSG00000148468	FAM171A1	-0.958628982	0.391821726	0.654318564	0.564387793	-0.304310418	6.366709671	9.470501606	8.631919287	7.794595599	7.57432889	9.179643937	9.378808813
ENSG00000196363	WDR5	-0.519330302	0.300898696	0.211592223	0.690410177	-0.307738079	8.946185348	8.101365653	8.206335842	7.985332082	7.91907183	8.694421086	8.653693725
ENSG00000188677	PARVB	-0.706777786	0.33594872	0.397773984	0.600319397	-0.309003802	8.816894989	7.928237109	7.163363831	7.507583254	7.35080119	8.538032809	8.497408326
	C16ORF52	6.662861068	3.49E-06	-6.971965631	0.00142934	-0.309104563	2	3.686303414	2	8.521127045	8.4504872	2	2
ENSG00000122176	FMOD	-2.626720108	0.09807061	2.317276872	0.141678745	-0.309443236	4.00722531	8.124485672	9.954333071	6.18083559	6.18985553	10.74946305	11.29947428
ENSG00000163866	SMIM12	-0.579138185	0.611410824	0.269322701	0.82055968	-0.309815484	8.928409675	8.916342327	6.203069917	7.836899669	7.91175216	8.355461064	8.998195614
ENSG00000131620	ANO1	-5.694923974	0.031180503	5.384931636	0.006312157	-0.309992338	2	2	4.058998049	2	2	7.761068522	6.883203642
ENSG00000113758	DBN1	-0.768604753	0.176804511	0.458586172	0.435298592	-0.310018581	12.14083108	11.13786633	11.33422412	10.83272206	10.8436425	11.75696777	12.31632069
ENSG00000143341	HMCN1	-1.155513076	0.359275511	0.845295301	0.506163186	-0.310217775	5.095959458	7.847167909	8.742726175	6.682159616	6.76727577	8.263008721	9.010572782
ENSG00000116128	BCL9	-1.108634351	0.437795312	0.794932144	0.58065443	-0.313702207	4.467311984	7.928237109	8.941672936	6.846474326	6.94511186	8.652274805	8.868207441
ENSG00000151655	ITIH2	-3.753010588	0.531143362	3.439230986	0.540908493	-0.313779602	2	2	2	2	2	3.648325314	4.133950982
ENSG00000164889	SLC4A2	0.292226397	0.632716815	-0.606782476	0.280587322	-0.31455608	9.957954117	10.24169813	10.91875185	10.67405041	10.7672916	9.474992887	10.10714012
ENSG00000103811	CTSH	-2.245863179	0.340614989	1.930768819	0.406108709	-0.315094361	2	6.535001748	2	3.09461492	3.60476711	7.078941352	6.681239787
ENSG00000115053	NCL	-0.2026247	0.804551315	-0.113806381	0.891733131	-0.316431081	12.60813735	11.81817171	10.9743126	11.7358446	11.7585122	11.56380211	12.06480799
ENSG00000066027	PPP2R5A	-0.546959254	0.337437807	0.230348902	0.702388158	-0.316610352	7.867441304	8.395519306	8.434763955	7.751013478	7.68437616	8.840629888	8.008487789
ENSG00000164253	WDR41	-0.912043258	0.015510476	0.594863974	0.125418384	-0.317179284	9.358295023	8.687092496	8.960138144	8.128448191	8.11451391	9.675771867	9.551318233
ENSG00000163563	MNDA	-3.753010643	0.531143362	3.434835446	0.541339971	-0.318175197	2	2	2	2	2	4.291799915	3.383972333
ENSG00000140718	FTO	-0.592341579	0.473769274	0.273867478	0.750952725	-0.3184741	8.39321512	9.962611189	10.26027233	9.160358775	9.13687201	9.809259615	10.18669803
ENSG00000188352	FOCAD	-0.663288356	0.478725082	0.344685142	0.72255273	-0.318603214	9.27634997	8.702538795	6.958202216	7.971157485	7.91419618	8.816978708	9.051693903
ENSG00000109881	CCDC34	-0.026566918	0.984043262	-0.292568167	0.82055968	-0.319135085	4.467311984	6.389896633	4.058998049	5.406080789	5.33407135	5.22272385	5.035976547
ENSG00000154096	THY1	1.429765059	0.329061986	-1.75058436	0.214752155	-0.320819301	11.83218596	10.09725346	6.958202216	12.06377966	12.1177047	8.817412208	9.018137056
ENSG00000166387	PPFIBP2	-3.151962709	9.48E-10	2.831000483	7.94E-09	-0.320962226	6.96351253	7.979858146	7.163363831	4.387512679	4.68029874	10.22568517	10.28813986
ENSG00000130706	ADRM1	0.234740894	0.854286984	-0.557743108	0.635985655	-0.323002214	10.60951384	9.936721131	7.398165132	10.01956245	10.0874876	9.068221759	9.438868282
ENSG00000181038	METTL23	-0.198545844	0.927391636	-0.126193953	0.954172164	-0.324739797	8.75772933	7.002537973	2	7.454228844	7.26532099	7.402744819	7.463599504
ENSG00000178719	GRINA	0.193606804	0.713585581	-0.518453854	0.275620191	-0.32484705	11.57090097	12.00728915	12.48428878	12.24651637	12.2775093	11.42398765	11.66734563
ENSG00000154370	TRIM11	-0.481847093	0.366470827	0.155438733	0.787507734	-0.326408359	8.444143041	7.503546079	7.858115942	7.514115926	7.5014335	8.243140215	8.015118249
ENSG00000100528	CNIH1	0.011953501	0.981947437	-0.338522703	0.456684488	-0.326569202	9.689059027	10.40638494	9.897982195	10.02471865	10.0651181	9.849450427	9.524348754
ENSG00000137770	CTDSPL2	-0.180827354	0.917096582	-0.145945653	0.933175306	-0.326773007	4.815609471	7.232742069	9.516493218	8.164192202	7.95986274	8.171702207	8.024537783
ENSG00000068615	REEP1	-2.266295273	0.523661392	1.939415207	0.549471093	-0.326880066	2	3.076665209	2	2.270786764	2	3.616794016	3.45257121
ENSG00000175348	TMEM9B	0.211873006	0.707193196	-0.539138228	0.290417779	-0.327265222	8.517314938	8.590771778	9.372276026	9.110576034	9.06390621	8.191903518	8.481080411
ENSG00000232838	PET117	1.204012107	0.213514288	-1.532923502	0.101181698	-0.328911395	5.532353208	3.686303414	5.772716771	6.372775472	6.26070706	3.878466556	4.002845082
ENSG00000157514	TSC22D3	-0.801445298	0.519400194	0.472389047	0.711063784	-0.329056251	9.800745303	9.367676479	6.534109959	8.227176542	8.38151133	9.998344725	8.956482366
ENSG00000075618	FSCN1	-1.219378269	0.042276369	0.889851411	0.145971903	-0.329526858	12.26818393	11.34889202	11.21627664	10.46993609	10.4778448	12.1473965	12.9123837
ENSG00000175806	MSRA	0.20145032	0.869968338	-0.531157651	0.637573045	-0.329707332	6.138360429	5.946638857	8.007629717	7.106324235	7.29937545	5.886629224	6.928061817
ENSG00000145293	ENOPH1	-0.56886352	0.658741986	0.237697997	0.8601928	-0.331165522	8.816894989	8.732943944	5.39244166	7.703217906	7.6986238	8.446833885	8.542691771
ENSG00000112799	LY86	-3.75301081	0.531143362	3.421462616	0.543166952	-0.331548194	2	2	2	2	2	4.350488971	3.236138336
ENSG00000065621	GSTO2	1.027162119	0.583738181	-1.360193934	0.450687102	-0.333031815	4.467311984	5.131603625	2	5.22679461	5.28958453	3.943456545	2.509591799
ENSG00000087087	SRRT	-0.214681381	0.666056796	-0.118733646	0.816271471	-0.333415027	9.689059027	9.148351514	9.049077186	9.103012382	9.11579188	8.960480054	9.413638893
ENSG00000132763	MMACHC	-0.724796769	0.694043631	0.391321272	0.836584165	-0.333475497	2	5.946638857	7.451345288	5.639097847	5.64780787	6.601598251	6.784644656
ENSG00000152454	ZNF256	-0.482964696	0.789639588	0.149212982	0.936599654	-0.333751714	2	5.946638857	7.098165388	5.614989456	5.63601114	6.244776687	6.185873005
ENSG00000049769	PPP1R3F	-0.791654401	0.722734802	0.456518602	0.84189993	-0.3351358	8.631580145	5.303523747	2	6.422239259	6.44101097	7.976682981	7.20156326
ENSG00000026652	AGPAT4	-0.016225951	0.994366162	-0.319234495	0.878328838	-0.335460446	2	8.540087354	7.896971941	7.703217906	7.62593544	7.329261848	7.407104868
ENSG00000170037	CNTROB	-0.690427135	0.712692238	0.352016513	0.855655091	-0.338410622	3.327711234	7.393647515	9.478569125	7.52061915	7.54938955	8.554768917	8.568035872
ENSG00000183255	PTTG1IP	-0.561400578	0.26397465	0.222048046	0.68121881	-0.339352532	12.60953239	11.91084881	12.89867565	11.96531395	11.9706237	12.66522651	12.83161361
ENSG00000168653	NDUFS5	-0.374840834	0.429456377	0.0350944	0.947397878	-0.339746434	10.43166252	9.616264339	10.2528023	9.74820345	9.78034427	10.06484606	10.2730054
ENSG00000183570	PCBP3	-2.53387251	0.159913355	2.1936141	0.212256603	-0.34025841	3.327711234	6.464272742	2	3.227757282	3.2184422	7.263334844	7.100081147
ENSG00000138613	APH1B	-0.097602976	0.855515488	-0.243235275	0.619827156	-0.340838251	7.709880939	7.503546079	8.236946975	7.826439377	7.65837038	7.545646914	7.656486163
ENSG00000282019		0.314889248	0.567016969	-0.655966684	0.193744404	-0.341077436	11.27287932	10.67263725	10.9968459	11.30745256	11.3225709	9.86021999	10.70939118
ENSG00000156931	VPS8	0.439489774	0.686884062	-0.780852722	0.443851376	-0.341362949	8.042902253	7.189546432	9.681375057	9.084476714	9.13896318	7.890311949	7.920407983
ENSG00000204314	PRRT1	4.848574462	0.281547017	-5.191110886	0.275561942	-0.342536424	2	2	2	4.965943888	5.14724905	2	2
ENSG00000143196	DPT	1.066229779	0.665819919	-1.409802585	0.555970778	-0.343572806	2	3.686303414	2	3.663521627	3.2184422	2.457042231	2.326754077
ENSG00000023330	ALAS1	0.03383807	0.96176107	-0.378811465	0.554788482	-0.344973395	10.25038534	8.916342327	9.926432745	9.791869268	9.8686817	9.39882884	9.441331959
ENSG00000184900	SUMO3	-0.269288051	0.641252598	-0.07577366	0.902642273	-0.345061712	10.67493801	9.680074956	10.63827116	10.1305433	10.1248958	10.21552475	10.41854076
ENSG00000213339	QTRT1	-0.177225064	0.83752462	-0.167949222	0.842413632	-0.345174286	5.866944937	7.35507212	7.451345288	6.820355514	6.94032357	6.672321934	7.08034504
ENSG00000152315	KCNK13	-2.312813426	NA	1.96738736	NA	-0.345426066	2	2	2	2	2.14022021	3.429858444	2.374681325
ENSG00000137500	CCDC90B	0.137049903	0.824880011	-0.482771905	0.376596657	-0.345722003	7.624139768	8.356994601	8.721235202	8.474993083	8.39906692	7.925322387	7.720479276
ENSG00000115318	LOXL3	-0.845395728	0.128214828	0.497340444	0.386914635	-0.348055284	11.13243651	10.16572116	10.23015729	9.713855064	9.760787	10.83544511	11.28239915
ENSG00000090061	CCNK	1.63949277	0.005747675	-1.989313961	0.000598834	-0.349821191	8.340423424	7.638155693	9.083172337	10.07418693	10.1132431	6.459963649	6.585205928
ENSG00000104450	SPAG1	-0.332171113	0.719366965	-0.019045175	0.984485378	-0.351216288	5.866944937	6.389896633	4.523675391	5.501219927	5.51241389	5.699879814	5.884455123
ENSG00000119559	C19orf25	-0.180412213	0.7329405	-0.171833291	0.741322616	-0.352245504	8.16936288	8.487557258	7.600047325	7.910550164	8.01327646	7.934143724	8.007538088
ENSG00000162687	KCNT2	-2.292481394	0.020855853	1.940176582	0.042725112	-0.352304812	5.70932728	4.93638933	5.156626401	3.79808662	3.08597292	6.287958221	7.647978997
ENSG00000153914	SREK1	0.082394618	0.922706766	-0.437203124	0.564260714	-0.354808505	9.2903348	9.949724236	8.236946975	9.435370781	9.37460909	8.975319061	8.797990868
ENSG00000142864	SERBP1	-0.347526853	0.713335141	-0.007736128	0.993931149	-0.355262981	11.61879218	11.04700893	9.399592459	10.6042022	10.6035548	10.85003188	11.03013303
ENSG00000123485	HJURP	0.134682881	0.930008622	-0.492002843	0.728500949	-0.357319962	4.00722531	6.311476754	4.058998049	5.304221918	5.45984938	4.301748724	5.217771644
ENSG00000136982	DSCC1	-0.38728097	0.824427896	0.028613038	0.987221496	-0.358667932	4.00722531	5.131603625	2	3.997744386	3.90918929	4.604690117	3.841670744
ENSG00000129559	NEDD8	-0.12730055	0.841925584	-0.231538879	0.699954446	-0.358839429	10.59831544	9.549500232	9.798878489	9.940529254	9.91151326	9.674576002	9.956333606
ENSG00000133142	TCEAL4	-0.875346126	0.174064658	0.514688406	0.439219521	-0.36065772	10.54098126	9.102512015	9.878698603	9.076774616	9.09116272	10.31389217	10.60620688
ENSG00000174808	BTC	4.829973619	0.286761062	-5.191110333	0.278346547	-0.361136714	2	2	2	5.210797711	4.85329164	2	2
ENSG00000138433	CIR1	0.291220428	0.662232293	-0.65292133	0.283396195	-0.361700902	9.567995545	8.623598428	8.35328246	9.274418797	9.19330273	8.39955642	8.191057445
ENSG00000132612	VPS4A	-0.652060826	0.133069745	0.286115089	0.53558846	-0.365945736	10.01801709	9.566483142	10.37481416	9.306761457	9.43372295	10.37686315	10.2307005
ENSG00000131437	KIF3A	-0.431843689	0.679228019	0.064281625	0.954416752	-0.367562064	8.009475818	6.140560021	8.460932916	7.484483106	7.29937545	8.195910056	7.477384137
ENSG00000161036	LRWD1	0.006316062	0.995663772	-0.374833258	0.687802707	-0.368517197	8.075571721	7.051611315	5.930865648	7.230526165	7.32163988	6.887434761	6.926052784
ENSG00000107554	DNMBP	0.359179529	0.680321745	-0.72974284	0.364350502	-0.370563311	9.097018025	8.993716073	10.63827116	10.12148041	10.1644413	9.042865619	9.076811853
ENSG00000198171	DDRGK1	0.468342435	0.318114195	-0.839688279	0.05360207	-0.371345844	9.720386367	8.862353853	9.242516611	9.763363111	9.79633147	8.544855285	8.415288533
ENSG00000188130	MAPK12	-0.276521267	0.824387358	-0.095317955	0.940405661	-0.371839222	8.313285031	8.297207175	5.39244166	7.514115926	7.57741621	7.583859056	7.851158781
ENSG00000187109	NAP1L1	0.046752507	0.954376589	-0.419373972	0.570849073	-0.372621464	11.37484003	11.00713613	9.725218844	10.93180718	10.8737771	10.45025691	10.42584592
ENSG00000151498	ACAD8	-0.012714951	0.989340687	-0.36149967	0.676479319	-0.374214621	7.031698319	7.232742069	8.65477198	7.805288479	7.82349365	7.262061517	7.656486163
ENSG00000130349	C6orf203	-0.281215463	0.832852241	-0.093454231	0.944699256	-0.374669694	7.709880939	5.72254323	4.874587708	6.34372989	6.31350454	6.589466407	6.420873184
ENSG00000037042	TUBG2	-0.024159918	0.9732242	-0.351339828	0.588182865	-0.375499746	8.928409675	7.669933291	8.486635645	8.458152491	8.38151133	7.890311949	8.278687361
ENSG00000119535	CSF3R	-3.753011378	0.531143362	3.375955232	0.548441916	-0.377056147	2	2	2	2	2	4.350488971	3.128433649
ENSG00000181544	FANCB	-1.085443007	0.646818108	0.707299006	0.767139907	-0.378144001	2	5.131603625	2	3.406930997	2.94009902	4.675266036	4.175132639
ENSG00000166246	C16orf71	-1.465964689	0.514678379	1.087083002	0.62219346	-0.378881688	2	4.710564154	2	2.947924294	2.59489879	4.369533167	4.400220394
ENSG00000165526	RPUSD4	-0.28898036	0.598988441	-0.089920304	0.878637249	-0.378900664	7.218819781	7.701025996	8.206335842	7.471115032	7.48508724	7.730090858	7.613439639
ENSG00000168288	MMADHC	0.341018218	0.739039557	-0.723078583	0.446930583	-0.382060365	11.03197509	9.256956408	8.904018013	10.42037002	10.3613276	9.343015767	9.320352741
ENSG00000107829	FBXW4	-0.489265943	0.733300911	0.105977197	0.944157185	-0.383288746	5.095959458	8.762721501	9.180874355	7.915493556	8.01782901	8.637620652	8.467331218
ENSG00000162512	SDC3	-0.820574106	0.328511143	0.436623076	0.618028749	-0.383951029	10.27872131	10.96932597	8.904018013	9.44822005	9.47817393	10.54203348	10.87071469

ENSG00000101363	MANBAL	-0.630226369	0.280938145	0.246234635	0.692848923	-0.383991734	9.761125267	9.203675681	8.536706852	8.660150093	8.59306436	9.386026816	9.599699825
ENSG00000168454	TXNDC2	-0.467978772	0.884927669	0.083148957	NA	-0.384829815	2	3.076665209	2	2.270786764	2.59489879	2.62493832	2.592996859
ENSG00000177674	AGTRAP	0.471036861	0.461552688	-0.855961766	0.146023342	-0.384924905	9.635285994	8.337339476	9.242516611	9.524619869	9.72990471	8.397238383	8.22740336
ENSG00000114784	EIF1B	0.161973187	0.842086376	-0.547382956	0.450077395	-0.38540977	9.410448395	7.979858146	8.585098385	8.976268302	8.89230135	8.52588267	7.864857644
ENSG00000167680	SEMA6B	-1.434806159	0.531386353	1.049038569	0.64532651	-0.38576759	2	4.93638933	2	2.784612485	3.01487888	4.920961476	4.062589736
ENSG00000168993	CPLX1	-0.951757503	0.77464352	0.565839125	NA	-0.385918378	2	3.076665209	2	2.270786764	2.38539787	3.172301926	2.277179783
ENSG00000221883	ARIH2OS	0.016977224	0.993765388	-0.404928644	0.832938355	-0.38795142	3.327711234	2	4.874587708	3.709785322	3.90918929	3.465698119	3.580696614
	LOC400927-(	7.696521343	1.01E-12	-8.08496528	2.68E-05	-0.388443937	4.467311984	2	3.369317972	10.62924246	10.6114832	2	2
ENSG00000269190	FBXO17	-0.59840767	0.274205421	0.209305397	0.722120418	-0.389102273	8.468948322	8.213429689	7.502634626	7.474468681	7.58663873	8.525352075	8.094184551
ENSG00000130300	PLVAP	0.312648823	0.918611539	-0.702564565	NA	-0.389915742	2	3.076665209	2	2.695456494	2.68924543	2.34604626	2.421067459
ENSG00000163909	HEYL	-3.817131392	0.089224711	3.426737723	0.062305464	-0.390393669	4.467311984	2	2	2.141737246	2.14022021	6.430795201	5.529673353
ENSG00000115325	DOK1	-0.953509061	0.478310707	0.563101614	0.683905619	-0.390407447	10.09173937	6.228547981	8.408111533	7.978262192	8.07355593	9.35888613	9.680872942
ENSG00000165832	TRUB1	-0.239131436	0.910161622	-0.151618213	0.942375924	-0.390749649	2	7.979858146	8.380957451	7.437142666	7.33264456	7.485815918	7.456657479
ENSG00000107731	UNC5B	-0.166573799	0.932700573	-0.224271105	0.909359225	-0.390844903	5.095959458	8.717821467	10.20715117	8.846395982	9.0020652	9.693119552	6.746446439
ENSG00000213512	GBP7	4.799379942	0.295753807	-5.191109406	0.2826457	-0.391729465	2	2	2	5.242616075	4.74762503	2	2
ENSG00000126216	TUBGCP3	-0.29237324	0.88199664	-0.101714477	0.958899411	-0.394087718	6.138360429	8.820493869	3.369317972	7.226553841	7.16216989	7.417689057	7.342293759
ENSG00000174276	ZNHIT2	-0.420402802	0.793600622	0.024263172	0.988451404	-0.39613963	7.667647223	5.946638857	3.369317972	6.147608921	6.08962627	6.625560108	6.451924088
ENSG00000074410	CA12	-7.177270833	0.004413569	6.780332	0.00027703	-0.396938833	2	5.131603625	2	2	2	10.2041324	10.26647028
ENSG00000069431	ABCC9	2.651333088	0.000213646	-3.050071296	1.90E-05	-0.398738208	7.218819781	6.389896633	7.342949392	9.610955295	9.67979376	4.742549839	3.560117069
ENSG00000104976	SNAPC2	-0.411184583	0.58474982	0.01215568	0.988303878	-0.399028903	9.247966931	8.213429689	7.734844356	8.117764469	8.15020018	8.411091064	8.680755147
ENSG00000102738	MRPS31	0.04882451	0.972268825	-0.447956678	0.732916452	-0.399132167	8.138771685	5.946638857	5.156626401	6.941624891	7.10341274	6.649130473	6.435070131
ENSG00000217128	FNIP1	0.649110465	0.367412771	-1.049329733	0.119894702	-0.400219268	8.009475818	9.640525262	9.180874355	9.769519169	9.70964668	8.155334989	7.950369405
ENSG00000136868	SLC31A1	-0.403211133	0.126527284	-0.000501601	0.998980237	-0.403712735	10.81212061	10.63690565	10.49998282	10.20813496	10.2972338	10.68714125	10.62196123
ENSG00000126814	TRMT5	0.113467258	0.891428469	-0.51741566	0.477751115	-0.403948402	8.493334308	6.899080544	8.007629717	8.03159949	8.05595642	7.536196998	7.30412922
ENSG00000163660	CCNL1	0.509099059	0.507120384	-0.914021907	0.197757479	-0.404922848	10.16946352	8.469613413	9.330306409	10.03327157	9.94357048	8.513094212	8.635366595
ENSG00000129480	DTD2	-0.853764353	0.092249336	0.447679973	0.392537339	-0.406084381	7.667647223	6.78762695	7.098165388	6.436065776	6.35727082	7.599066672	7.713507501
ENSG00000149548	CCDC15	-0.359428662	0.890529219	-0.046850069	0.985627735	-0.406278731	2	5.303523747	2	3.754611435	3.7864959	4.321442857	3.580696614
ENSG00000185252	ZNF74	-0.760161024	0.670378341	0.353783967	0.848992364	-0.406377057	4.467311984	8.124485672	4.058998049	6.006476466	6.02777321	7.389977651	6.690737414
ENSG00000119509	INVS	0.05438548	0.951349347	-0.461378071	0.570028066	-0.40699259	6.563806279	7.538394143	8.408111533	7.723090567	7.75969386	7.139828829	7.32867054
ENSG00000135541	AHI1	0.113969824	0.8692818	-0.522714425	0.383915641	-0.408744601	8.892187472	8.820493869	7.818184403	8.684969711	8.72001862	8.307508685	7.79392309
ENSG00000134324	LPIN1	-0.029593605	0.981338611	-0.380126411	0.738292369	-0.409720016	7.33131396	9.949724236	10.0085654	9.438808458	9.51325794	8.712712904	9.450446099
ENSG00000165023	DIRAS2	-3.753011788	0.531143362	3.343185637	0.552967424	-0.40982615	2	2	2	2	2	3.891700877	3.789585079
ENSG00000119638	NEK9	-0.559096197	0.211593503	0.149047176	0.763378865	-0.410049021	10.75275473	9.969011754	10.16000817	9.704603278	9.84261543	10.52222593	10.43823328
ENSG00000187172	BAGE2	4.776907368	0.298608151	-5.191109433	0.282784279	-0.414202065	2	2	2	5.022023783	4.97065356	2	2
	BAGE3	4.776907368	0.298608151	-5.191109433	0.282784279	-0.414202065	2	2	2	5.022023783	4.97065356	2	2
ENSG00000163466	ARPC2	-0.016043333	0.979923499	-0.400831512	0.464862114	-0.416874845	12.54683767	11.64711661	11.60453593	12.00455363	11.9665083	11.53983427	11.66026272
ENSG00000263002	ZNF234	0.130780385	0.906858652	-0.547873817	0.581597072	-0.417093432	5.70932728	7.979858146	7.163363831	7.391747748	7.32898565	7.027411703	6.283256815
ENSG00000104973	MED25	0.056064753	0.94475044	-0.47328207	0.513742625	-0.417217318	8.075571721	9.566483142	8.561105505	8.94033939	8.92660044	8.252146986	8.55118937
ENSG00000123130	ACOT9	-0.3305405	0.719060419	-0.08688559	0.929627216	-0.417426091	10.19198706	8.124485672	9.330306409	9.088859521	9.14105132	9.177956973	9.516692718
ENSG00000144802	NFKBIZ	1.575569171	0.000562684	-1.993060148	7.93E-06	-0.417490977	8.674867122	9.235879652	9.725218844	10.83010652	10.8554541	7.269684675	7.34530375
ENSG00000272602	ZNF595	-0.088069461	0.918975348	-0.329808738	0.678175142	-0.417878199	5.532353208	6.535001748	6.322058544	6.096288482	6.14061114	6.226613426	5.453611367
ENSG00000130703	OSBPL2	-0.418607639	0.379355575	0.000545772	0.999263508	-0.418061868	9.820154628	9.479495904	8.960138144	8.960808841	9.12847688	9.539475459	9.383208837
ENSG00000154217	PITPNC1	1.219595666	0.513480912	-1.638544022	0.356668405	-0.418948356	8.075571721	7.572420273	2	8.422134132	8.5227358	6.165271053	5.050803619
ENSG00000143333	RGS16	-2.95109697	0.112520621	2.530101149	0.158865702	-0.420995821	4.00722531	5.946638857	2	3.09461492	2.49394783	7.903438544	5.470251342
ENSG00000113368	LMNB1	-0.336411325	0.902572075	-0.084804685	0.974738711	-0.421216009	2	6.311476754	2	4.739373666	4.45712836	4.799832052	4.836507146
ENSG00000175115	PACS1	-0.46145765	0.38975761	0.038960289	0.948687403	-0.422497361	10.21416435	10.48441188	11.2124308	10.2335933	10.2457682	10.67463433	10.80007469
ENSG00000168246	UBTD2	-0.081745029	0.841645125	-0.341503902	0.336902641	-0.423248931	8.493334308	8.077869079	8.460932916	8.264463296	8.26953254	7.979009894	8.042267212
ENSG00000123562	MORF4L2	-0.581917382	0.372864851	0.157670578	0.825446679	-0.424246804	12.78399552	11.83228301	11.44325334	11.55026089	11.5548912	12.2230071	12.35668755
ENSG00000087365	SF3B2	-0.410632528	0.228220774	-0.013883577	0.972127213	-0.424516105	11.21540273	11.22868348	10.75391088	10.6575484	10.6895293	10.99886917	11.13690957
ENSG00000127191	TRAF2	-0.188672857	0.91485625	-0.236242463	0.892291608	-0.42491532	9.33149386	7.002537973	4.523675391	7.910550164	7.81567127	7.894426854	7.735470193
ENSG00000130816	DNMT1	0.037177735	0.942177035	-0.462618249	0.290479733	-0.425440515	9.189468652	8.747909548	9.553445883	9.196346103	9.26686067	8.704299335	8.767460665
ENSG00000067842	ATP2B3	-0.949020201	0.764636496	0.523208041	NA	-0.42581216	2	3.076665209	2	2.3892346	2.2680111	3.059555873	2.421067459
ENSG00000282608	ADORA3	-3.753011995	0.531143362	3.326572216	0.555044393	-0.426439779	2	2	2	2	2	4.311629396	3.100202476
ENSG00000154059	IMPACT	-0.428872594	0.544237044	0.001789586	0.998392474	-0.427083009	8.418903792	9.018611864	7.502634626	8.045195239	8.00412803	8.510415622	8.386355612
ENSG00000223496	EXOSC6	-0.614809495	0.350255547	0.186066726	0.794799105	-0.428742769	8.609439662	7.315436877	8.175061096	7.530319349	7.5014335	8.522164399	8.045046786
ENSG00000117984	CTSD	0.231031255	0.739013584	-0.660426985	0.290456069	-0.429395729	13.1138925	12.07561123	12.00879923	12.71608724	12.7327132	12.08025667	11.53610185
ENSG00000184207	PGP	-0.698538837	0.485453797	0.268935019	0.799962733	-0.429603818	9.262228249	7.051611315	7.600047325	7.54315232	7.65837038	8.39955642	8.694982998
ENSG00000164300	SERINC5	-1.225885445	0.000133455	0.794739261	0.016927551	-0.431146185	9.556468883	9.203675681	9.452720984	8.209172298	8.17894021	10.37745126	9.997094495
ENSG00000088035	ALG6	0.336731388	0.802652992	-0.768002364	0.53460268	-0.431270976	5.866944937	6.602424674	3.369317972	6.104969671	6.14893615	5.328699035	4.861951484
ENSG00000124767	GLO1	-0.311041572	0.723978556	-0.121499654	0.895505527	-0.432541226	11.18951828	10.13758676	9.242516611	10.1096668	10.0798882	10.32445299	10.24184794
ENSG00000188452	CERKL	-3.753012085	0.531143362	3.319361679	0.556010306	-0.433650406	2	2	2	2	2	4.133696865	3.430065404
ENSG00000108424	KPNB1	-0.365434296	0.448739712	-0.068951794	0.897115004	-0.43438609	11.24801458	12.01039539	12.06319515	11.43600295	11.4718853	11.68200198	11.81524183
ENSG00000237440	ZNF737	0.482665512	0.538070994	-0.917548007	0.206491773	-0.434882495	6.468621203	5.595927442	6.958202216	6.907295206	6.86652414	5.825876956	5.243813244
ENSG00000129562	DAD1	-0.781454762	0.204362279	0.346305835	0.596208763	-0.435148928	11.0444064	9.785372135	10.11951029	9.617795654	9.66027117	10.87544335	10.64107086
	BAGE4	4.755655615	0.303181423	-5.191108963	0.284871836	-0.435453347	2	2	2	5.022023783	4.93258395	2	2
	BAGE5	4.755655615	0.303181423	-5.191108963	0.284871836	-0.435453347	2	2	2	5.022023783	4.93258395	2	2
ENSG00000168152	THAP9	-0.399282679	0.732697705	-0.036597239	0.975799274	-0.435879919	4.00722531	5.72254323	5.59507254	4.846787657	5.04390545	5.707383854	4.634793542
ENSG00000160791	CCR5	-3.753012115	0.531143362	3.316981831	0.556282123	-0.436030283	2	2	2	2	2	4.397636241	2.851942824
ENSG00000156469	MTERF3	-0.088321698	0.970209755	-0.348475655	0.883002588	-0.436797353	8.493334308	4.93638933	2	6.936770382	6.96881778	6.940933331	6.415155055
ENSG00000150540	HNMT	-0.008814267	0.997041173	-0.428312676	0.814923166	-0.437126943	2	6.04685525	4.523675391	5.058234798	4.89348248	5.026717879	4.047883918
ENSG00000163629	PTPN13	-0.782776319	0.323240837	0.345199017	0.681431908	-0.437577301	8.737455672	9.488434476	7.600047325	8.063125866	8.00412803	9.030953258	9.25982657
ENSG00000147457	CHMP7	-0.471442728	0.566726644	0.033270424	0.970602478	-0.438172304	10.21416435	8.356994601	9.272377397	8.983347946	9.02372946	9.494370989	9.515691102
ENSG00000131931	THAP1	0.106782665	0.931219161	-0.545002917	0.62909049	-0.438220252	5.532353208	6.228547981	7.818184403	7.021746293	6.8716533	6.743394671	5.727202496
ENSG00000166821	PEX11A	-0.700804628	0.763884658	0.262423149	0.914220926	-0.43838148	2	8.101365653	4.058998049	5.910700032	6.01871653	6.944112782	6.81969491
ENSG00000148248	SURF4	-0.456817869	0.254887129	0.017476406	0.97020102	-0.439341463	12.4934348	12.26697646	12.92005579	12.12173968	12.1362731	12.4719126	12.72309441
ENSG00000130119	GNL3L	0.249124511	0.616043469	-0.688558682	0.119746169	-0.439434171	7.485143553	8.004992147	8.007629717	8.126317766	8.07574085	6.953609309	7.380942161
ENSG00000137710	RDX	-0.3761076	0.470515237	-0.06420754	0.911555747	-0.44031514	9.800745303	10.7784592	10.45512158	10.06253517	9.98949012	10.4114447	10.25910765
ENSG00000150893	FREM2	3.189270163	0.579885898	-3.630743412	0.538029429	-0.441473249	2	2	2	4.497095146	2	2	2.059860178
ENSG00000118705	RPN2	0.492700388	0.491521702	-0.936852603	0.15499647	-0.444152216	12.44893028	12.38655348	11.11279376	12.58338056	12.5997269	10.81040203	11.44713892
ENSG00000174343	CHRNA9	2.085402755	0.549630367	-2.530047934	0.467835004	-0.444645179	2	3.076665209	2	3.663521627	3.60476711	2.225793514	2
ENSG00000272047	GTF2H5	-0.88040055	0.135121389	0.435588432	0.480467544	-0.444812118	8.737455672	7.538394143	7.97167868	7.25413313	7.34356593	8.479522918	8.699106494
ENSG00000204370	SDHD	0.118472909	0.911517345	-0.563774258	0.54895459	-0.445301349	9.624286623	8.414402626	7.342949392	8.931825643	8.80148734	8.255989884	8.127786376
ENSG00000198937	CCDC167	-1.032466979	0.269034612	0.58641793	0.541021035	-0.446049049	5.532353208	7.274681896	5.772716771	5.273747807	5.64780787	6.702675117	7.24894374
ENSG00000163071	SPATA18	-1.810630226	0.36589956	1.362507773	0.498878282	-0.448122453	8.75772933	6.04685525	2	5.674521485	5.64780787	9.069314155	8.28340307
ENSG00000130560	UBAC1	-0.521811316	0.754977416	0.073193703	0.967559124	-0.448617613	5.330594137	6.728495673	9.714382178	7.797276258	7.88956768	8.529591383	8.324402372
ENSG00000130304	SLC27A1	-1.968299204	0.090738947	1.518811452	0.194468033	-0.449487752	9.579430841	8.004992147	6.073378139	6.476767609	6.67805337	9.809259615	10.18984111
ENSG00000146828	SLC12A9	-0.489036958	0.690575637	0.039124964	0.975799274	-0.449911994	7.159102577	6.78762695	9.426401271	7.767511442	7.84670947	8.214461509	8.433081317
ENSG00000181817	LSM10	-0.699910125	0.395581532	0.249140629	0.77791101	-0.450769496	8.855032352	8.777382925	7.029880454	7.641902724	7.83385794	8.535927079	8.80566012
ENSG00000184432	COPB2	-0.068203533	0.8177838	-0.38304489	0.123771525	-0.451248423	11.84171405	11.77133279	11.53541251	11.630805	11.6775673	11.31807122	11.36155524
ENSG00000197497	ZNF665	-0.879137244	0.548487869	0.426486725	0.780515702	-0.452650519	5.866944937	3.076665209	6.958202216	5.210797711	5.13053105	6.508020621	6.235386446
ENSG00000179094	PER1	-1.019970907	0.283678293	0.567049685	0.564587637	-0.452921222	6.468621203	8.433041974	8.922968322	7.165617905	7.34718809	8.626284124	8.992447063
ENSG00000106524	ANKMY2	-0.232083296	0.859987098	-0.222028629	0.863495873	-0.454111924	5.70932728	8.124485672	9.031722608	7.975897842	7.8720741	7.922101208	7.947401109
ENSG00000124126	PREX1	0.30164994	0.851414911	-0.755802734	0.608695439	-0.454152794	4.00722531	7.274681896	8.511888463	7.805288479	7.74329312	6.775876478	6.714211481
ENSG00000104915	STX10	-0.449984679	0.651645795	-0.00536009	0.996190831	-0.455344769	9.317904228	7.572420273	7.285536013	7.920420067	7.90439517	8.268091966	8.428831018
ENSG00000132604	TERF2	-0.382436626	0.448617153	-0.074427984	0.893566028	-0.456864609	8.285626337	8.337339476	9.06622548	8.244958867	8.20711886	8.622816121	8.431665941
ENSG00000278588	HIST1H2BI	3.332290267	0.416320096	-3.789453408	0.385135236	-0.457163141	2	2	2	3.709785322	3.94787299	2.048074405	2
ENSG00000183186	C2CD4C	-2.417001423	0.380422171	1.958170196	0.438543047	-0.458831227	2	3.686303414	2	2.270786764	2.14022021	4.644324387	3.100202476
ENSG00000130283	GDF1	4.731989459	0.310556285	-5.191107995	0.28942322	-0.459118536	2	2	2	4.670864177	5.19627118	2	2
ENSG00000221955	SLC12A8	0.31515015	0.743030025	-0.775047219	0.376725096	-0.459897069	5.532353208	6.951736442	5.772716771	6.658765962	6.43421531	5.873348082	5.072762365
ENSG00000147419	CCDC25	-0.360204808	0.307768638	-0.100127129	0.794947828	-0.460331937	8.674867122	8.522788974	8.941672936	8.291333947	8.42842973	8.563064723	8.672390305
ENSG00000182533	CAV3	-3.753012419	0.531143362	3.292621821	0.560073901	-0.460390598	2	2	2	2	2	3.534812207	4.033026654
ENSG00000105793	GTPBP10	0.4004161	0.613073282	-0.861440078	0.234784916	-0.461023978	7.975256505	6.899080544	6.718950348	7.700356475	7.6957855	6.793610851	6.06327132
ENSG00000088854	C20orf194	-0.507358909	0.419141353	0.045878387	0.94830231	-0.461480522	9.602033055	9.102512015	8.266922072	8.568972129	8.60672783	9.172206498	9.09971864
ENSG00000174804	FZD4	-0.832506003	0.252767379	0.369133192	0.632354165	-0.46337281	8.16936288	9.00621767	9.868959282	8.401141138	8.29975519	9.571952335	9.513351291
ENSG00000142230	SAE1	-0.502129464	0.293107873	0.037464402	0.945470349	-0.464665062	9.33149386	9.910357957	10.14394518	9.279220784	9.38785872	9.676010922	10.0434692
ENSG00000179593	ALOX15B	-2.304510325	NA	1.839071568	NA	-0.465438757	2	2	2	2.141737246	2	3.336159116	2.374681325
ENSG00000070087	PFN2	-0.22401762	0.725681209	-0.241832042	0.699954446	-0.465849663	11.93813426	10.83211025	10.9833682	11.14542979	11.0834766	11.04836626	11.14405721

ENSG00000137106	GRHPR	-0.677157603	0.351836711	0.209944735	0.790974913	-0.467212868	10.31337612	8.929529568	9.031722608	8.879660299	8.91566441	9.610733985	9.930260564
ENSG00000196646	ZNF136	-0.37988894	0.838336958	-0.087559394	0.963967825	-0.467448334	3.327711234	6.464272742	8.677268317	7.030886362	7.02868983	7.398115274	7.224630935
ENSG00000117593	DARS2	-0.489935222	0.762843325	0.022263962	0.989426938	-0.46767126	8.228665467	7.467835385	3.369317972	6.936770382	6.79936111	7.142599214	7.561323797
ENSG00000185650	ZFP36L1	-0.185027	0.747940998	-0.282660775	0.608206076	-0.467687775	12.58421296	11.76398989	11.67888398	11.86539179	11.9037876	11.91539999	11.64644938
ENSG00000164654	MIOS	-0.692195506	0.667332365	0.222633871	0.896455571	-0.469561635	3.327711234	8.101365653	7.777116083	6.503277904	6.91614081	7.846806418	7.334741191
ENSG00000106789	CORO2A	-0.929541459	0.472536264	0.459826948	0.731494326	-0.469714511	5.532353208	5.303523747	8.076950877	5.900763176	6.08095082	7.446000032	7.194904178
ENSG00000155659	VSIG4	-3.753012542	0.531143362	3.28277859	0.5613132	-0.470233952	2	2	2	2	2	3.336159116	4.147809227
ENSG00000122507	BBS9	-0.421924028	0.51002601	-0.050958512	0.942419363	-0.47288254	7.096806373	7.790474954	6.80318718	6.907295206	6.88657603	6.989444349	7.482861322
ENSG00000103024	NME3	-0.748282811	0.517016221	0.275130362	0.821586671	-0.473152449	5.70932728	8.124485672	8.784770914	7.226553841	7.32163988	8.480618146	8.045046786
ENSG00000132376	INPP5K	-0.499913102	0.472081543	0.024911465	0.973993243	-0.475001637	9.460782017	8.213429689	8.380957451	8.239055745	8.35836495	8.607356811	9.003921351
ENSG00000130830	MPP1	0.147431068	0.92573854	-0.622734117	0.668910133	-0.475303048	6.73718411	7.431218269	3.369317972	6.861921612	6.61935067	6.307840213	5.641643428
ENSG00000157379	DHRS1	-0.530381467	0.560175881	0.053914202	0.957558622	-0.476467264	8.609439662	6.728495673	7.342949392	7.333842102	7.17037177	7.539353852	8.065268319
ENSG00000151893	CACUL1	-0.100145265	0.910744811	-0.376728453	0.639943914	-0.476873718	10.6316532	9.656475478	8.845626171	9.833599446	9.75261615	9.567062722	9.468158308
ENSG00000143493	INTS7	-0.431947436	0.745616581	-0.046376079	0.973327949	-0.478323515	8.75772933	7.901717006	5.156626401	7.416368504	7.5014335	8.047866378	7.59079099
ENSG00000140153	WDR20	-0.494254005	0.784685584	0.01549131	0.993715232	-0.478762694	3.327711234	8.639735838	9.083172337	7.807949361	7.83643742	8.304419417	8.344097092
ENSG00000142453	CARM1	-0.219233946	0.628892599	-0.259631295	0.554505321	-0.478865241	9.097018025	9.672251278	9.829330889	9.36754703	9.32862783	9.237802185	9.375131848
ENSG00000196511	TPK1	-0.481094844	0.827605625	0.001487897	0.999379962	-0.479606947	6.96351253	3.686303414	2	5.178261742	5.06165168	5.740677235	5.343519008
ENSG00000011638	TMEM159	0.02503224	0.981947437	-0.50720192	0.605467095	-0.48216968	9.460782017	7.572420273	7.552162999	8.468280417	8.56534297	8.147081218	7.819040576
ENSG00000196557	CACNA1H	-4.446332599	0.001101634	3.96396397	0.002319776	-0.482368629	4.815609471	5.457120953	7.163363831	3.289987342	2.14022021	10.56415812	9.224298414
ENSG00000185013	NT5C1B	-3.753012696	0.531143362	3.270429411	0.562988004	-0.482583285	2	2	2	2	2	3.824280769	3.753786374
ENSG00000197748	CFAP43	-0.270544793	0.930223809	-0.212138476	NA	-0.482683269	2	2	3.369317972	2.600425781	2.38539787	2.560105256	2.466008509
ENSG00000103005	USB1	-0.622056241	0.313384316	0.138069322	0.840370813	-0.483986919	10.14658276	9.018611864	9.439621147	8.99156386	8.99053093	9.415532511	10.01563471
ENSG00000141086	CTRL	3.303288798	0.423427099	-3.789547534	0.387476301	-0.486258735	2	2	2	3.709785322	3.90918929	2.048074405	2
ENSG00000160345	C9orf116	-0.373825054	0.767978648	-0.112498978	0.932122597	-0.486324032	5.095959458	3.076665209	5.156626401	4.415698455	4.2865223	4.628601098	4.500832793
ENSG00000197467	COL13A1	-0.191519993	0.882732465	-0.295501866	0.811305502	-0.487021859	10.01801709	7.431218269	7.818184403	8.673343061	8.74631819	8.3234671	8.8444706
ENSG00000156928	MALSU1	-0.522516611	0.531143362	0.031597085	0.972248923	-0.490919527	7.096806373	7.002537973	8.561105505	7.182120794	7.26532099	7.799317009	7.73202455
ENSG00000010278	CD9	-0.305378411	0.62163201	-0.186655633	0.768414094	-0.492034044	10.39976609	9.540933215	9.778213818	9.6388717	9.65662671	9.320255125	10.1060314
ENSG00000247746	USP51	0.538522384	0.835029603	-1.032345622	0.671068215	-0.493823238	2	5.946638857	2	5.022023783	5.04390545	3.663836244	3.875378318
ENSG00000143793	C1orf35	-0.425337119	0.605487532	-0.06944062	0.938021298	-0.494777739	5.866944937	7.538394143	6.629488387	6.394182228	6.50077304	6.857395343	6.718860807
ENSG00000249459	ZNF286B	1.703577931	0.009229661	-2.19850669	0.000640345	-0.494928759	5.866944937	5.303523747	6.322058544	7.454228844	7.54308677	4.220168345	3.698365557
ENSG00000126698	DNAJC8	-0.512052302	0.085098867	0.016137809	0.965171887	-0.495914494	9.922475255	9.988044813	10.14394518	9.4935523	9.52769466	9.870283133	10.18564881
ENSG00000094963	FMO2	-3.753012883	0.531143362	3.255441713	0.564749006	-0.497571171	2	2	2	2	2	3.968654369	3.560117069
ENSG00000165304	MELK	-0.42804397	0.72338932	-0.070368526	0.956587684	-0.498412496	5.095959458	7.315436877	5.156626401	5.860316854	5.89549381	6.205571486	6.222348126
ENSG00000175906	ARL4D	-1.308546862	0.001161121	0.810087604	0.051735128	-0.498459258	8.313285031	8.487557258	7.734844356	6.89733479	6.98285659	9.094571356	8.947101695
ENSG00000128590	DNAJB9	0.529489348	0.202610543	-1.028694667	0.007054497	-0.499205319	9.602033055	9.386925327	9.399592459	10.07086745	9.90967722	8.720150661	8.104880443
ENSG00000184445	KNTC1	-0.039938889	0.98345284	-0.459357011	0.800241379	-0.4992959	6.563806279	7.393647515	2	6.503277904	6.3857277	6.165271053	5.921228818
ENSG00000101901	ALG13	-0.087670616	0.95389048	-0.412575306	0.775706431	-0.500245922	4.467311984	8.505280662	8.110401118	7.759286043	7.64371756	7.210184325	7.540464021
ENSG00000126091	ST3GAL3	1.094643044	0.409632583	-1.595111975	0.203815171	-0.500468932	3.327711234	7.189546432	6.629488387	7.444001439	7.51115308	5.056402493	4.845038551
ENSG00000198034	RPS4X	-1.008285021	0.09741056	0.50708177	0.428827907	-0.501203251	12.96817362	12.07857394	11.63358652	11.31822478	11.3401725	12.76832102	12.91428517
ENSG00000139644	TMBIM6	-0.183409291	0.704833394	-0.318053632	0.48356437	-0.501462924	13.55579352	13.03584485	12.75140232	12.94621622	12.9937154	12.77798604	12.89097863
ENSG00000120913	PDLIM2	-1.167899459	0.329735621	0.666307116	0.591168456	-0.501592344	10.81212061	9.181799937	7.398165132	8.537252353	8.6021877	10.2787502	10.49946786
ENSG00000104327	CALB1	1.604315142	NA	-2.106159237	NA	-0.501844094	2	2	2	3.349643765	2	2.048074405	2.117335275
ENSG00000136696	IL36B	2.3867222	0.653114396	-2.890379571	NA	-0.50365737	2	2	2	2.868578001	3.50452879	2.094598369	2
ENSG00000268738	HSFX2	3.148095335	0.471298431	-3.65198425	0.426694614	-0.503888915	2	2	2	3.615725078	3.7864959	2	2.059860178
ENSG00000136859	ANGPTL2	0.958441931	0.359313288	-1.463660174	0.136560497	-0.505218242	7.750913381	10.02537381	10.57976717	10.77881047	10.8513151	8.585126182	8.204380326
ENSG00000117877	CD3EAP	-0.420975567	0.696640403	-0.084354597	0.941762599	-0.505330164	4.467311984	6.602424674	7.029880454	5.94010643	6.02777321	6.329884754	6.27380897
ENSG00000143919	CAMKMT	-0.74495469	0.654365619	0.239599592	0.891849925	-0.505355097	4.00722531	5.595927442	2	3.881293667	4.05806467	4.713032626	4.810605986
ENSG00000117410	ATP6V0B	0.523872413	0.446344169	-1.02938217	0.101902753	-0.505509757	10.96815916	10.01303782	9.541233192	10.78692561	10.8560898	9.325140951	9.222662522
ENSG00000156675	RAB11FIP1	-1.46971541	0.001242812	0.964048559	0.039698009	-0.505666851	8.564112515	8.053983481	8.110401118	7.066878356	6.52655952	9.393605372	9.0072509
ENSG00000111725	PRKAB1	-0.540637368	0.503519889	0.034765456	0.969279885	-0.505871912	8.855032352	7.35507212	7.502634626	7.523859802	7.55253065	8.286996197	7.892900165
ENSG00000189171	S100A13	0.372984484	0.628218242	-0.879686532	0.208642569	-0.506702048	10.41898845	9.00621767	9.132850258	10.02070987	10.0596091	8.644477784	8.931664222
ENSG00000147127	RAB41	-3.753013009	0.531143362	3.245340444	0.565942887	-0.507672566	2	2	2	2	2	3.824280769	3.717076715
ENSG00000136937	NCBP1	-0.162628686	0.625452298	-0.345394911	0.25649001	-0.508023597	8.87372952	8.820493869	8.631919287	8.600009476	8.63959257	8.360229299	8.514222325
ENSG00000108469	RECQL5	-0.27876941	0.883143303	-0.229519543	0.902568424	-0.508288954	8.836089686	6.78762695	3.369317972	7.218576229	7.4074129	7.534088584	7.171352322
ENSG00000107789	MINPP1	0.397894023	0.460218392	-0.906631667	0.061688239	-0.508737644	8.107517749	9.13702746	8.742726175	9.140440229	9.10513517	7.799317009	7.866953664
ENSG00000135341	MAP3K7	-0.346529224	0.253811546	-0.162273743	0.61128327	-0.508802966	9.710019321	9.632483534	9.788583153	9.410199593	9.31943747	9.686964814	9.395603187
ENSG00000198369	SPRED2	-0.043555355	0.97688117	-0.465886487	0.738195992	-0.509441842	10.27872131	9.256956408	6.203069917	9.236404433	9.33137356	8.901594745	8.828426256
ENSG00000196275	GTF2IRD2	2.239858855	0.000123128	-2.750189176	3.60E-06	-0.51033032	6.009025997	4.93638933	5.930865648	7.842101511	7.7866208	3.411599105	3.496556178
ENSG00000086061	DNAJA1	0.317525555	0.427142195	-0.834362317	0.019134157	-0.516836762	11.75612888	11.1062775	11.60453593	11.82786535	11.8332199	10.71265031	10.64779131
ENSG00000140350	ANP32A	-0.470714904	0.080529035	-0.046638469	0.88481517	-0.517353373	9.544849385	9.45234302	9.399592459	9.004380786	8.99400091	9.319337188	9.515022972
ENSG00000168803	ADAL	-0.202746277	0.849223293	-0.314714979	0.756352835	-0.517461255	5.095959458	6.602424674	7.451345288	6.548530093	6.39974839	6.361143723	6.37742435
ENSG00000145604	SKP2	-0.843026096	0.230169878	0.324642737	0.667089979	-0.51838336	6.816637977	8.276714465	7.451345288	6.64692533	6.99214046	8.091532339	7.835189056
ENSG00000008988	RPS20	0.063171163	0.947611632	-0.581620626	0.504030629	-0.518449463	11.80565454	9.726146444	10.64969256	11.01936533	11.0524721	10.41459985	10.37059545
ENSG00000158793	NIT1	-0.415679516	0.56929975	-0.103074638	0.896486957	-0.518754153	8.609439662	7.315436877	7.451345288	7.366699513	7.62891439	7.723629692	7.891871215
ENSG00000186642	PDE2A	2.154636385	0.521613346	-2.673457098	0.416362316	-0.518820713	4.00722531	2	2	4.598938466	4.22485516	2.384001718	2
ENSG00000101868	POLA1	-0.719855632	0.699169627	0.201024206	0.91912988	-0.518831426	2	5.595927442	7.342949392	5.48800675	5.48637102	6.295446113	6.394961217
ENSG00000105865	DUS4L	-0.353117369	0.842102843	-0.165840021	0.927015754	-0.51895739	2	6.228547981	4.874587708	4.965943888	4.811949	5.136410075	4.967301796
ENSG00000161249	DMKN	1.411713556	0.336817477	-1.931655142	0.177744904	-0.519941586	4.00722531	3.686303414	2	4.523237872	4.53541753	2.560105256	2.551896972
ENSG00000135502	SLC26A10	-0.021894558	0.994204236	-0.498211204	0.851505238	-0.520105762	2	6.228547981	2	4.927308402	4.65713986	4.065262523	4.673629022
ENSG00000087274	ADD1	-0.661402979	0.144346027	0.140422638	0.784356511	-0.520980341	11.44840376	11.39952642	12.14415806	11.02366403	11.0657237	11.71465816	11.96548264
ENSG00000198700	IPO9	-0.466302111	0.44286374	-0.056268332	0.934216456	-0.522570443	10.64259676	10.21502048	9.426401271	9.685919789	9.74164918	9.951520129	10.27517721
ENSG00000100239	PPP6R2	-0.339710906	0.622552387	-0.184626096	0.795874263	-0.524337002	10.21416435	8.862353853	10.09465441	9.470224559	9.52449896	9.594132343	9.707433942
ENSG00000076685	NT5C2	0.619875524	0.414405236	-1.144497961	0.102021981	-0.524622436	7.82963488	9.566483142	8.922968322	9.585592789	9.52928986	7.711553069	7.905190659
ENSG00000177889	UBE2N	-0.205279718	0.564402109	-0.319371875	0.342242445	-0.524651593	9.710019321	9.74864196	9.399592459	9.425874714	9.42603674	9.162002347	9.449047665
ENSG00000059573	ALDH18A1	-0.760203741	0.420363638	0.235122235	0.817323895	-0.525081507	8.75772933	10.14890644	11.1494608	9.570001722	9.55848727	10.51809824	10.592497
ENSG00000143379	SETDB1	-0.356940962	0.642211794	-0.168947463	0.83342673	-0.525888425	7.975256505	7.393647515	8.941672936	7.770242853	7.99722844	8.104378504	8.041339497
ENSG00000106605	BLVRA	-0.4705541	0.688047343	-0.055775819	0.965290088	-0.526329919	6.89194353	7.638155693	9.553445883	8.045195239	7.97398853	8.313667445	8.517561736
ENSG00000143393	PI4KB	-0.271247402	0.657619829	-0.255724868	0.674724562	-0.52697227	10.11549941	9.994333773	11.04521411	10.19090967	10.1964202	10.14836757	10.26746235
ENSG00000168397	ATG4B	-0.444372317	0.679837753	-0.083905149	0.942222441	-0.528277466	10.37373127	8.791896851	7.896971941	8.94033939	8.94941893	9.3448212	9.259427717
ENSG00000183258	DDX41	-0.215415878	0.719874208	-0.313318145	0.58526661	-0.528734023	10.08373159	9.055168103	9.344432377	9.296376788	9.39224836	9.10206309	9.38027698
ENSG00000167920	TMEM99	-0.559619717	0.790662721	0.030546701	0.988721265	-0.529073016	7.579279349	4.710564154	2	5.720439317	5.63601114	6.189586148	6.270645889
ENSG00000053372	MRTO4	-0.264381851	0.844391096	-0.266448056	0.840299173	-0.530829906	9.247966931	8.395519306	5.772716771	8.017874397	8.21506984	7.915637193	8.295125345
ENSG00000041988	THAP3	-0.314487505	0.884847189	-0.216732526	0.920142601	-0.531220032	8.797441463	7.538394143	2	7.504305792	7.32531744	7.755649985	7.221358049
ENSG00000104131	EIF3J	-0.422834632	0.341899875	-0.108802272	0.823280183	-0.531636904	9.509418609	8.747909548	9.272377397	8.838609445	8.73115061	9.159610958	9.032214052
ENSG00000111863	ADTRP	1.162316178	0.63692076	-1.693955742	0.472178974	-0.531639564	5.70932728	2	2	5.627094009	4.89348248	3.011868911	3.287118176
ENSG00000124160	NCOA5	-0.187429141	0.721693747	-0.344220826	0.483542133	-0.531649967	8.009475818	8.077869079	8.784770914	8.091796043	8.19510963	8.047866378	7.929461924
ENSG00000172586	CHCHD1	-0.396916874	0.665128529	-0.135108393	0.889682343	-0.532025267	8.696033109	7.467835385	6.718950348	7.402350992	7.53358066	7.639834368	7.80708241
ENSG00000154473	BUB3	0.073428397	0.930787681	-0.605660809	0.410371427	-0.532232412	10.38028415	9.849218119	8.677268317	9.904309377	9.8316678	9.04656815	9.326077571
ENSG00000099849	RASSF7	0.311097401	0.851362698	-0.843711819	0.575697563	-0.532614419	5.70932728	6.140560021	2	5.590471342	5.81407719	4.875056259	4.456980017
ENSG00000072954	TMEM38A	-2.560121127	0.021321155	2.026401413	0.059608695	-0.533719714	5.70932728	3.076665209	5.59507254	3.162721474	3.28037825	7.263334844	6.791281771
ENSG00000177556	ATOX1	0.239745556	0.654620374	-0.774646829	0.10141086	-0.534901273	8.540903468	8.356994601	9.212024683	8.964391141	9.00321357	7.850199974	8.101323949
ENSG00000018189	RUFY3	0.062613515	0.956151149	-0.598454477	0.56087963	-0.535840962	6.563806279	9.192779271	8.845626171	8.663092307	8.60521604	8.021018	7.941446131
ENSG00000182670	TTC3	-1.082736415	0.002249586	0.546269475	0.148400754	-0.536466941	11.17451594	11.86009815	11.54461865	10.52353286	10.4222654	12.09105348	12.10897797
ENSG00000125459	MSTO1	0.766343328	0.385890403	-1.302905354	0.113061522	-0.536562026	7.709880939	6.728495673	5.772716771	7.753776273	7.62891439	5.371837532	5.984375752
ENSG00000069248	NUP133	-0.323248605	0.68974372	-0.213389073	0.795568436	-0.536637678	9.384607364	8.777382925	7.600047325	8.468280417	8.41468635	8.52588267	8.574463273
ENSG00000147533	GOLGA7	0.085117712	0.91483416	-0.622487469	0.362015414	-0.537369757	10.44422623	9.13702746	9.212024683	9.786482773	9.84133177	9.185364902	9.031747025
ENSG00000176927	EFCAB5	0.625196449	0.705896131	-1.16377545	0.45324474	-0.538579001	4.00722531	4.442710129	2	4.387512679	4.2560182	3.374372158	2.782576693
ENSG00000103091	WDR59	-0.179933155	0.933928913	-0.360426775	0.864992366	-0.54035993	9.015175615	8.376385542	2	7.985332082	7.96694292	7.737439787	7.8574975
ENSG00000107521	HPS1	-0.274982011	0.7400867	-0.266443695	0.745570382	-0.541425706	9.678463567	8.337339476	10.0085654	9.164517339	9.28113268	8.928330746	9.483608161
ENSG00000119801	YPEL5	-0.203512359	0.678957281	-0.33814267	0.464069117	-0.541655028	11.26227543	10.49775373	10.99236731	10.7658674	10.7271979	10.41960523	10.78241603
ENSG00000185808	PIGP	0.547465598	0.60735938	-1.089923456	0.266749214	-0.542457857	7.82963488	6.389896633	5.39244166	7.477814553	7.33629421	5.711121288	5.96101835
ENSG00000232859	LYRM9	-0.359549707	0.823564926	-0.18335188	0.910950051	-0.542901587	2	5.457120953	5.930865648	4.739373666	4.98931816	5.002522427	5.04340913
ENSG00000220205	VAMP2	-0.747394758	0.29492176	0.203976061	0.794797351	-0.543418697	10.83137851	9.806969599	9.330306409	9.352133347	9.41830936	10.11137118	10.52474633
ENSG00000167562	ZNF701	0.288281909	0.894855735	-0.831847131	0.677551443	-0.543565222	2	5.946638857	8.206335842	7.242377933	7.12888795	6.200262696	6.018717758
ENSG00000166444	ST5	-0.05940787	0.973280525	-0.484269041	0.773865459	-0.54367691	4.467311984	8.671479029	9.829330889	8.719297823	8.76674758	8.115702075	8.504157459
ENSG00000205838	TTC23L	-0.470220855	0.898417957	-0.073536776	NA	-0.543757631	2	2	3.369317972	2.141737246	2.68924543	2.858551861	2.172608095
ENSG00000126947	ARMCX1	0.312282624	0.88983543	-0.856851867	0.679613476	-0.544569244	2	9.018611864	9.287079172	8.837307593	8.93865515	7.851047117	7.608437243

ENSG00000183598	HIST2H3D	2.22040346	0.366576641	-2.765139141	0.274695513	-0.544735681	3.327711234	2	2	3.840289928	3.60476711	2.139668783	2.059860178
ENSG00000119574	ZBTB45	-0.835758564	0.40909689	0.289863466	0.789642976	-0.545895098	6.009025997	8.053983481	8.65477198	7.101994243	7.12888795	8.218405973	8.218402364
ENSG00000033050	ABCF2	-0.526179896	0.320074535	-0.021044968	0.972248923	-0.547224863	9.031919741	9.591588447	10.07784268	9.04780133	9.16073939	9.513759765	9.69539454
ENSG00000173705	SUSD5	-2.988949723	0.167659551	2.439457996	0.259652636	-0.549491727	4.00722531	8.747909548	2	4.387512679	4.58534546	9.346023568	9.888570123
ENSG00000090013	BLVRB	-0.726777038	0.34076194	0.177212571	0.833507886	-0.549564467	8.910412251	7.669933291	7.342949392	7.384635376	7.46522407	8.411091064	8.209344857
ENSG00000140307	GTF2A2	-0.127542247	0.836817752	-0.422375949	0.442025836	-0.549918196	8.737455672	7.790474954	8.143093325	8.151677543	8.13979554	7.648631242	8.036691948
ENSG00000272886	DCP1A	0.176031795	0.664619951	-0.726465044	0.037142991	-0.550433249	9.423197387	9.969011754	9.491321578	9.792541168	9.86426457	8.943502388	8.920102913
ENSG00000118420	UBE3D	-0.838008382	0.748522947	0.28585686	0.917287256	-0.552151523	2	6.464272742	2	4.358765249	4.2865223	5.108342547	5.436777225
ENSG00000169217	CD2BP2	-0.458906254	0.110464035	-0.093606868	0.774164954	-0.552513122	9.740899611	9.741182361	10.05225195	9.419799055	9.36660051	9.803144189	9.708895312
ENSG00000196199	MPHOSPH8	-0.040488267	0.958996611	-0.514265926	0.463376829	-0.554754193	10.13887448	8.762721501	8.960138144	9.333786218	9.42689279	8.890924823	8.930161469
ENSG00000115486	GGCX	-0.752394173	0.255434807	0.196724388	0.788486582	-0.555669784	9.33149386	9.71095217	10.67785662	9.290680758	9.25437635	9.998918132	10.40688307
ENSG00000133103	COG6	-0.577561003	0.545365418	0.02124989	0.984153231	-0.556311113	7.485143553	9.338312924	9.647595056	8.570539957	8.45217004	9.432893972	8.682541321
ENSG00000176946	THAP4	-0.796610869	0.134337633	0.238329579	0.684431323	-0.55828129	9.839306291	9.090820894	8.763901704	8.511364632	8.51470562	9.51135037	9.567841145
ENSG00000074842	MYDGF	0.459085104	0.546357577	-1.017728065	0.142297927	-0.558642961	12.02642475	10.69982928	10.57378414	11.67082229	11.7644504	10.1958205	10.29185004
ENSG00000131165	CHMP1A	-0.540643499	0.113304945	-0.018040217	0.965819335	-0.558683716	9.931427102	10.27823701	10.40882047	9.594867189	9.76350037	10.09537369	10.30156833
ENSG00000168286	THAP11	-0.664409581	0.747940998	0.105637722	0.962309739	-0.558771859	9.048471759	8.433041974	2	7.474468681	7.60793141	8.424811996	8.1631842
ENSG00000164294	GPX8	-0.411764502	0.466152501	-0.147843567	0.805630147	-0.559608068	10.64259676	11.26034006	11.65074017	10.82486117	10.8372174	11.31967864	10.82737534
ENSG00000164985	PSIP1	-0.982323736	0.053105198	0.422389996	0.436296276	-0.55993374	8.107517749	9.067150531	8.536706852	7.723090567	7.59884443	9.2270746	8.846065265
ENSG00000148153	INIP	-0.495582726	0.540424469	-0.064728386	0.942414968	-0.560311112	8.674867122	6.899080544	7.858115942	7.555872146	7.42465798	8.014983227	7.805990373
ENSG00000135040	NAA35	0.482691334	0.465166199	-1.043440822	0.081548025	-0.560749488	7.218819781	8.487557258	7.646393253	8.260583448	8.47221251	6.831875681	6.920008846
ENSG00000119812	FAM98A	-0.309421518	0.844353006	-0.252607028	0.872155485	-0.562028546	11.58798587	8.687092496	7.858115942	9.979997207	9.95667084	9.922798958	10.12037877
ENSG00000134255	CEPT1	-0.238369693	0.866444985	-0.324153776	0.811836432	-0.562523469	9.015175615	8.101365653	5.39244166	7.88809286	7.88209639	8.093681326	7.432801564
ENSG00000069345	DNAJA2	-0.110424631	0.880685896	-0.452231511	0.483213396	-0.562656143	9.460782017	9.298208073	10.5187901	9.766786386	9.74233706	9.473068198	9.353618642
ENSG00000108883	EFTUD2	-0.09124473	0.931237974	-0.47196316	0.624555723	-0.56320789	8.19931886	10.5327403	10.19940021	9.814540128	9.88932507	9.355308099	9.582902386
ENSG00000076344	RGS11	-1.12951116	0.499226274	0.564918274	0.742760586	-0.564592886	6.468621203	4.710564154	2	4.268917409	4.40247105	5.769639817	5.867805474
ENSG00000188917	TRMT2B	-0.185200018	0.866763421	-0.379463232	0.712935639	-0.564663249	8.493334308	7.099070191	6.322058544	7.491120977	7.31425651	6.831875681	7.519298205
ENSG00000121440	PDZRN3	-1.027568175	0.010924161	0.461775773	0.285670744	-0.565792401	8.696033109	9.125613817	9.503962296	8.096156705	8.15847021	9.574006172	9.632719128
ENSG00000103066	PLA2G15	-0.557409656	0.667251957	-0.008450378	0.995181721	-0.565860034	9.397585721	6.899080544	10.46808205	8.967964567	8.97070853	9.711427775	9.286693143
ENSG00000011523	CEP68	-1.456681457	0.128993961	0.890648401	0.366387369	-0.566033056	8.609439662	6.04685525	8.042706615	6.529309835	6.44101097	8.971428833	8.560285
ENSG00000122873	CISD1	0.0310295	0.984020078	-0.598546204	0.67741085	-0.567516704	9.344956677	5.595927442	7.029880454	8.134820652	8.14188248	7.497765909	7.54177663
ENSG00000158435	CNOT11	-0.620183109	0.031572147	0.052347231	0.882077604	-0.567835878	8.836089686	8.717821467	8.960138144	8.188900477	8.2580338	8.82044307	8.955005256
ENSG00000148429	USP6NL	-0.317917381	0.7192138	-0.25019443	0.778789835	-0.568111811	8.87372952	7.002537973	7.777116083	7.742693204	7.79725241	7.842553218	7.830900438
ENSG00000071626	DAZAP1	-0.45589922	0.421603569	-0.112334874	0.85594901	-0.568234094	10.23600589	9.181799937	10.16797309	9.496029474	9.46072474	9.801172998	9.839247834
ENSG00000178988	MRFAP1L1	-0.477158994	0.285057543	-0.091376166	0.854928523	-0.56853516	9.27634997	9.443177293	8.677268317	8.717883675	8.68036596	8.97997353	9.176932811
ENSG00000189091	SF3B3	-0.463282559	0.306087183	-0.106286769	0.833139147	-0.569569328	10.29961392	11.0102427	10.3329194	10.10372352	10.1499977	10.40871423	10.55349898
ENSG00000161970	RPL26	-0.261757328	0.723145763	-0.308586551	0.669799484	-0.570343879	11.87225278	10.76012279	10.47451889	10.90160668	10.9124925	10.89908444	10.82049707
ENSG00000148848	ADAM12	-0.527621407	0.552447214	-0.044945214	0.964355081	-0.572566621	9.90440318	11.73423878	10.19160738	10.30737895	10.341489	10.65609247	10.94166594
ENSG00000134152	KATNBL1	-0.431824705	0.688569122	-0.141406967	0.901623508	-0.573231672	8.797441463	7.051611315	6.629488387	7.481152682	7.29563107	7.950836136	7.334741191
ENSG00000090971	NAT14	-0.596194885	0.338188336	0.022244885	0.974689151	-0.57395	7.904282243	7.60566235	8.805342542	7.593372273	7.61696151	8.149149092	8.274745794
ENSG00000174599	TRAM1L1	0.20669036	0.938887874	-0.781544753	0.759271438	-0.574854393	2	6.228547981	2	5.093559143	4.89348248	4.230621763	4.119958326
ENSG00000104957	CCDC130	-0.129824559	0.939161162	-0.446127603	0.783596985	-0.575952163	9.317904228	7.538394143	4.523675391	7.956842238	8.06698125	7.852739912	7.533882999
ENSG00000108774	RAB5C	0.419406787	0.609297559	-0.996402484	0.179070824	-0.576995697	11.13631304	9.277729677	10.44859742	10.89160678	10.8918703	9.406340618	9.551969768
ENSG00000169372	CRADD	-0.473285668	0.660274906	-0.104213486	0.928829011	-0.577499154	8.075571721	6.389896633	5.772716771	6.670510205	6.58294721	6.920094328	7.043754524
ENSG00000079313	REXO1	-0.249060334	0.881733734	-0.328745469	0.838156295	-0.577805803	5.532353208	8.356994601	10.19160738	8.729158235	8.7882247	8.637620652	8.72128783
ENSG00000154736	ADAMTS5	-1.68048304	0.019792117	1.102482692	0.138594698	-0.578000348	9.509418609	8.806266215	9.116480286	7.577865504	7.4450836	9.210337096	10.87267185
ENSG00000196792	STRN3	-0.166749335	0.795687476	-0.411447473	0.48317362	-0.578196808	8.631580145	9.424669273	8.677268317	8.813671111	8.78422202	8.860117405	8.17168765
ENSG00000156697	UTP14A	0.057620144	0.931706466	-0.637483115	0.263817749	-0.579862972	7.867441304	7.538394143	7.818184403	7.77569021	7.82609171	6.593521701	7.5085977
ENSG00000183011	NAA38	-0.573129708	0.322499342	-0.006834356	0.991913393	-0.579964064	7.624139768	8.077869079	8.784770914	7.626911583	7.72113133	8.254069715	8.209344857
ENSG00000198920	KIAA0753	-0.207053354	0.911517345	-0.373879291	0.834048189	-0.580932644	4.00722531	6.899080544	9.212024683	7.694616532	7.74054144	7.626042826	7.478755384
ENSG00000165609	NUDT5	-0.087885325	0.875335246	-0.49317686	0.298915693	-0.581062186	9.344956677	8.876041558	8.460932916	8.780186674	8.92053511	8.547470747	8.347103327
ENSG00000272968	RBAK-RBAKD	4.607708252	0.33707801	-5.191105638	0.300167308	-0.583397386	2	2	2	4.783303513	4.91316568	2	2
ENSG00000115257	PCSK4	-0.826258821	0.575921504	0.2421454	0.878301109	-0.584113421	2	5.303523747	4.874587708	3.754611435	4.16043394	4.742549839	4.919632516
ENSG00000144848	ATG3	0.214629708	0.8177838	-0.798844229	0.328235173	-0.58421452	9.48530526	8.590771778	7.451345288	8.956018563	8.9374542	7.926930284	7.963161916
ENSG00000226479	TMEM185B	-0.26784716	0.833109956	-0.31773546	0.796772666	-0.58558262	6.96351253	10.02537381	8.511888463	8.726347845	8.74219737	8.511487655	8.839676004
ENSG00000160294	MCM3AP	-0.430802875	0.694540471	-0.156820535	0.892291608	-0.587623409	8.517314938	10.00059544	11.24667977	9.925905286	9.84261543	10.16089043	10.15467031
ENSG00000258289	CHURC1	-0.156335845	0.867371688	-0.43163824	0.612133959	-0.587974085	7.940205782	9.125613817	9.935792933	9.093229054	9.02486072	8.859275573	8.709655965
ENSG00000172725	CORO1B	-0.274850826	0.860190935	-0.313898764	0.836381584	-0.58874959	10.96815916	9.497318007	6.629488387	9.597945453	9.612912	9.364533261	9.74381649
ENSG00000170954	ZNF415	-0.700866648	0.299070637	0.111111273	0.884519325	-0.589755375	7.435667355	6.228547981	7.451345288	6.429169081	6.46787796	7.380620955	7.065820006
ENSG00000064652	SNX24	-0.346768386	0.715717569	-0.243482787	0.800241379	-0.590251173	7.82963488	6.666836837	5.772716771	6.604700657	6.71776877	6.763331418	6.760043946
ENSG00000046647	GEMIN8	-0.329660131	0.839097157	-0.261833768	0.871406162	-0.591493899	4.467311984	6.311476754	8.536706852	6.699459234	7.20671394	6.86244555	7.199901369
ENSG00000163655	GMPS	-0.855966987	0.476162581	0.264437503	0.837824475	-0.591529484	6.73718411	9.479495904	10.13584611	8.562683721	8.41813449	9.629617332	9.577792788
ENSG00000100714	MTHFD1	-0.243431884	0.659761977	-0.352723843	0.501916413	-0.596155726	8.493334308	8.053983481	8.941672936	8.339946438	8.24644267	7.985968197	8.362041249
ENSG00000196586	MYO6	-0.94671452	0.25067706	0.350377906	0.692464563	-0.596336613	10.46903009	9.318399851	8.486635445	8.74312865	8.68036596	9.928428593	10.0700947
ENSG00000171016	PYGO1	-0.623689633	0.285611782	0.027066836	0.968381186	-0.596622796	6.563806279	7.503546079	7.163363831	6.490083648	6.58294721	7.364099281	6.934072182
ENSG00000151135	TMEM263	-0.346761724	0.549118904	-0.250189977	0.671298904	-0.596951701	9.820154628	10.93377402	10.50627919	10.18324441	10.1079152	10.36845651	10.10403354
ENSG00000099194	SCD	-1.294475896	0.116761103	0.696769724	0.420713272	-0.597706172	11.43901855	12.88662532	13.02902175	11.33354142	11.2928274	12.49302884	13.81844695
ENSG00000186777	ZNF732	-0.140686901	0.956087463	-0.460748492	0.853015332	-0.601435393	2	4.710564154	2	3.162721474	3.74316441	3.44788957	3.100202476
ENSG00000172409	CLP1	-0.380791427	0.792847991	-0.222153235	0.881261401	-0.602944661	5.532353208	6.140560021	8.721235202	7.190301983	7.02868983	7.267148095	7.264927592
ENSG00000112200	ZNF451	-0.091354897	0.947190051	-0.511854195	0.69384525	-0.603209091	6.653098118	9.903691196	10.22252928	9.502614552	9.42088976	9.260296988	8.790832728
ENSG00000169131	ZNF354A	-0.641524676	0.471535675	0.038077764	0.969515502	-0.603446912	8.340423424	6.844429755	7.225742779	6.979883891	6.99214046	8.097255872	6.992837548
ENSG00000198218	QRICH1	-0.38646303	0.507060161	-0.217189213	0.717711114	-0.603652243	10.78274301	10.04368224	9.681375057	9.794554993	9.92005068	9.96488668	10.08705154
ENSG00000100664	EIF5	0.238934899	0.528830242	-0.843680275	0.010052906	-0.604745376	11.20066853	11.41367288	11.77848812	11.71721007	11.7275807	10.58937934	10.69220572
ENSG00000174442	ZWILCH	-0.302881186	0.877766587	-0.301874933	0.875414673	-0.604756119	7.709880939	7.099070191	2	6.682159616	6.46787796	6.60762613	6.55171954
ENSG00000117632	STMN1	-1.053974223	0.144179754	0.446131448	0.562234526	-0.607842775	10.06758145	9.256956408	8.561105505	8.447952961	8.30907138	9.421818136	10.2100027
ENSG00000155959	VBP1	-0.330010766	0.350112527	-0.2804866	0.423741616	-0.610497366	8.367060731	8.505280662	8.805342542	8.260583448	8.22297724	8.250863742	8.3297313
ENSG00000067365	METTL22	-0.844323078	0.596631897	0.232884774	0.892291608	-0.611438304	3.327711234	7.928237109	8.236946975	6.548530093	6.86146731	7.786974998	7.735470193
ENSG00000171681	ATF7IP	-0.13875923	0.931483663	-0.4732533	0.755970837	-0.61201253	6.138360429	10.59613751	10.46808205	9.873521491	9.81541592	9.737316722	9.244592076
ENSG00000110934	BIN2	-3.753014319	0.531143362	3.140454489	0.579831678	-0.61255983	2	2	2	2	2	4.340871764	2.509591799
ENSG00000138078	PREPL	-1.044563781	0.115443406	0.431895709	0.543191241	-0.612668072	9.097018025	9.148351514	10.38167985	8.679168098	8.58694992	9.938028579	10.24728895
ENSG00000079134	THOC1	0.045561935	0.937917387	-0.658375081	0.175083229	-0.612813146	6.653098118	6.602424674	6.958202216	6.716553866	6.84618961	6.262714117	5.941260754
ENSG00000171094	ALK	-0.300867655	0.918975348	-0.313933711	0.914220926	-0.614801366	2	3.686303414	2	2.868578001	2.59489879	2.987421008	2.421067459
ENSG00000215186	GOLGA6B	2.275697383	NA	-2.890808174	NA	-0.61511079	2	2	2	3.289987342	3.01487888	2.094598369	2
ENSG00000163749	CCDC158	-3.753014354	0.531143362	3.137666994	0.580041481	-0.61534736	2	2	2	2	2	3.904814898	3.45257121
ENSG00000099866	MADCAM1	-3.753014354	0.531143362	3.137634335	0.580041481	-0.615380019	2	2	2	2	2	3.616794016	3.771796761
ENSG00000116704	SLC35D1	0.36739613	0.736046082	-0.983389439	0.31933214	-0.615993309	8.042902253	7.669933291	9.935792933	9.29163166	9.2582292	8.069136662	7.795024296
ENSG00000250571	GLI4	-0.540873287	0.695743402	-0.0758065	0.959231573	-0.616679787	4.00722531	6.728495673	7.777116083	6.306580752	6.31350454	6.833591108	6.671679221
ENSG00000235750	KIAA0040	-4.596837097	0.067812259	3.979743478	0.026072796	-0.617093619	2	4.11352228	2	2.141737246	2	6.162543904	6.543880272
ENSG00000113312	TTC1	-0.402800155	0.498492641	-0.214504071	0.726968373	-0.617304226	9.761125267	8.820493869	8.784770914	8.773395507	8.81724322	8.838922815	9.112583673
ENSG00000147592	LACTB2	0.298046019	0.885491359	-0.91703026	0.624817493	-0.618984241	2	5.303523747	7.451345288	6.586219071	6.32824132	5.238348159	5.419744333
ENSG00000066654	THUMPD1	-0.15355873	0.652929267	-0.465701477	0.123069403	-0.619260208	9.128497779	9.277729677	9.049077186	9.055659246	8.95417718	8.809589199	8.573180082
ENSG00000119718	EIF2B2	-0.483427235	0.780664977	-0.136127533	0.940230037	-0.619554768	9.602033055	8.702538795	4.058998049	8.219202381	8.13770558	8.603340274	8.435907908
ENSG00000146576	C7orf26	-0.82866958	0.713873535	0.207217801	0.931938712	-0.621451778	9.646202137	6.951736442	2	7.461007055	7.45854177	8.816111316	8.037622656
ENSG00000244509	APOBEC3C	-1.652682766	0.000161722	1.030492447	0.024397067	-0.622190319	10.25752168	9.308339287	9.69246155	8.184811706	8.14396641	10.76235392	10.89582457
ENSG00000141098	GFOD2	-0.462355817	0.571137034	-0.160963313	0.853015332	-0.62331913	7.667647223	7.051611315	8.763901704	7.527093191	7.5587924	7.919680595	7.752576035
ENSG00000079335	CDC14A	1.151836057	0.643838836	-1.775317014	0.453489046	-0.623480957	2	6.464272742	2	6.164317909	5.99120031	3.616794016	3.640730416
ENSG00000198925	ATG9A	-0.235863056	0.924580052	-0.3888638	0.872875203	-0.624726857	11.07704221	10.3729879	2	9.91915277	9.97902226	9.760045703	9.833096297
ENSG00000135930	EIF4E2	-0.616459212	0.224421978	-0.009576999	0.987221496	-0.626036211	10.51143645	9.540933215	10.21486071	9.49932577	9.55457603	9.979293284	10.26904825
ENSG00000108106	UBE2S	-0.068856502	0.946330866	-0.558281911	0.546275271	-0.627138413	7.867441304	9.890264534	8.380957451	8.902248225	8.93264039	8.242494717	8.601149963
ENSG00000143155	TIPRL	-0.079630242	0.892338755	-0.547834415	0.270301865	-0.627464657	9.358295023	9.298208073	8.536706852	9.119171929	8.94225201	8.749978316	8.362784101
ENSG00000102900	NUP93	-0.231503024	0.687937839	-0.396995356	0.46213389	-0.62849838	9.064836028	8.337339476	8.143093325	8.356399318	8.32569041	8.015738956	8.323639487
ENSG00000205765	C5orf51	-0.229423351	0.872289176	-0.399519184	0.767357706	-0.628942534	6.653098118	9.298208073	10.42884603	9.283050899	9.17201613	9.13790818	8.977004658
ENSG00000134222	PSRC1	-0.65543864	0.579599436	0.02550263	0.984296564	-0.629936011	4.00722531	5.303523747	6.718950348	5.093559143	5.18011485	5.769639817	5.740981724
ENSG00000020256	ZFP64	-0.316366794	0.470185896	-0.313588187	0.462949352	-0.629954981	8.586954094	8.124485672	8.380957451	8.172475401	7.93600799	8.218405973	7.885682058
ENSG00000167842	MIS12	-0.245522546	0.798798132	-0.384761482	0.674981326	-0.630284028	7.709880939	8.639735838	6.534109959	7.626911583	7.64666005	7.576706933	7.420010428
ENSG00000105821	DNAJC2	0.623718823	0.424331051	-1.255204448	0.078779916	-0.631485624	8.493334308	7.099070191	7.098165388	8.325162455	8.34212018	6.66270409	6.320441373
ENSG00000100865	CINP	-0.794845561	0.188055699	0.162058285	0.811488554	-0.632787276	8.87372952	7.979858146	7.691296561	7.536750104	7.44169935	8.30132352	8.550537483
ENSG00000152439	ZNF773	-0.597365944	0.657839596	-0.03632003	0.980129329	-0.633685974	4.815609471	6.78762695	8.296287027	6.592405944	6.71776877	7.514972902	6.793487375

ENSG00000077463	SIRT6	-0.176298047	0.9141407	-0.457637205	0.765367121	-0.633935251	8.468948322	8.356994601	4.058998049	7.641902724	7.74329312	7.382965824	7.452476174
ENSG00000139168	ZCRB1	-0.447824897	0.547023674	-0.187298609	0.811488554	-0.635123506	9.204317094	7.901717006	7.896971941	7.939959586	8.11451391	8.206540078	8.362784101
ENSG00000063322	MED29	-0.702671303	0.042012415	0.067318098	0.87167541	-0.635353205	9.533135543	9.842262059	9.344432377	8.912174893	8.87609528	9.526011998	9.781250219
ENSG00000196704	AMZ2	-0.563813816	0.52931101	-0.071750342	0.942331353	-0.635564159	9.886101849	9.04308532	7.818184403	8.561107327	8.61275919	9.313202635	8.789175794
ENSG00000120675	DNAJC15	-0.013034994	0.988750252	-0.622853573	0.43187425	-0.635888567	9.646202137	7.847167909	8.805342542	8.979812467	8.87609528	8.093681326	8.524881346
ENSG00000153132	CLGN	3.896739606	0.059474197	-4.532649953	0.04575727	-0.635910348	2	4.11352228	2	6.29144905	6.23747437	2.139668783	2
ENSG00000102763	VWA8	-0.079360543	0.912239853	-0.560797814	0.362015414	-0.640158357	7.579279349	8.077869079	8.865353883	8.134820652	8.24450173	7.721778317	7.712342256
ENSG00000189045	ANKDD1B	0.79519526	0.764636496	-1.435993395	0.569190163	-0.640798135	2	3.686303414	2	3.349643765	3.2184422	2.526562374	2.225841198
ENSG00000173545	ZNF622	0.163787964	0.870709566	-0.805306437	0.354345163	-0.641518473	10.29961392	8.981105197	8.236946975	9.571568433	9.60989979	8.46742022	8.774177123
ENSG00000005243	COPZ2	-0.397874657	0.644424353	-0.24441318	0.782285213	-0.642287837	10.00958825	8.702538795	8.266922072	8.773395507	8.81985255	8.741779902	9.130142376
ENSG00000183137	CEP57L1	-0.200779158	0.84860975	-0.441595986	0.650306201	-0.642375143	6.468621203	5.595927442	7.451345288	6.529309835	6.45450701	6.635427934	5.759151667
ENSG00000133706	LARS	-0.716962803	0.112779646	0.073946123	0.890497536	-0.643016681	9.567995545	10.3778065	10.34701993	9.4022977	9.45821469	10.15507139	10.27813351
ENSG00000149091	DGKZ	-0.59966218	0.323956816	-0.043488757	0.950328841	-0.643150937	9.048471759	8.317412869	7.818184403	7.783822874	7.99261031	8.371491092	8.506176057
ENSG00000111783	RFX4	-0.264565931	0.954430302	-0.379713394	NA	-0.644279325	2	3.076665209	2	2.868578001	2	2.42098416	2.509591799
ENSG00000162623	TYW3	-0.200143982	0.901917502	-0.445664958	0.770017372	-0.64580894	5.095959458	8.101365653	9.577565424	8.283707551	8.28283292	8.128335751	7.94839122
ENSG00000085644	ZNF213	-0.736683411	0.720923534	0.089920732	0.967776844	-0.646762679	2	7.761270542	8.978369997	7.084543139	7.29937545	8.002074901	8.008487789
ENSG00000158796	DEDD	-0.275835888	0.751627932	-0.372347239	0.659557507	-0.648183127	8.19931886	9.591588447	7.934808822	8.459845423	8.54656162	8.331987532	8.481080411
ENSG00000128789	PSMG2	0.079183001	0.906842758	-0.728389395	0.191278435	-0.649206394	9.048471759	8.004992147	8.110401118	8.565831351	8.51952909	7.754744914	7.738907626
ENSG00000197562	RAB40C	-0.86797583	0.314840892	0.217937121	0.818792684	-0.650038709	6.563806279	8.169643011	8.721235202	7.19843704	7.23436042	8.329558253	8.232289427
ENSG00000033800	PIAS1	-0.204423255	0.725318811	-0.446377864	0.405169521	-0.650801119	9.720386367	9.256956408	8.631919287	9.078979414	9.0561812	8.880175401	8.773059882
ENSG00000155508	CNOT8	-0.098443205	0.749513987	-0.55332789	0.032897253	-0.651771095	9.848787621	9.856140801	9.577565424	9.677941812	9.66390646	9.248772045	9.187873195
ENSG00000116679	IVNS1ABP	-0.719299668	0.004354518	0.066866122	0.83065631	-0.652433546	10.79260216	10.92392403	10.9788475	10.14007766	10.2252472	11.09344985	10.82912339
ENSG00000157036	EXOG	-0.433054609	0.8339564	-0.220803647	0.916320081	-0.653858256	8.517314938	8.147241016	2	7.265793319	7.42122542	7.601082296	7.503217537
ENSG00000042753	AP2S1	-0.228640559	0.716867438	-0.425333629	0.470865845	-0.653974188	11.11682541	10.24169813	10.16797309	10.33023354	10.3657986	9.86021999	10.39476727
ENSG00000165633	VSTM4	0.096833233	0.935203319	-0.751832131	0.47448492	-0.654998899	9.810482605	8.124485672	7.225742779	8.88471049	8.88234995	8.151214006	7.929461924
ENSG00000187189	TSPYL4	-0.679907357	0.506977228	0.024308167	0.983104465	-0.65559919	10.19198706	7.819099893	8.536706852	8.537252353	8.51470562	9.183011957	9.264206697
ENSG00000120725	SIL1	0.110702037	0.88608019	-0.767838136	0.236344937	-0.657136099	10.52923584	9.308339287	9.344432377	9.924679904	9.98542831	8.927527801	9.221024773
ENSG00000213551	DNAJC9	0.072534587	0.96300973	-0.733644664	0.610519493	-0.661110077	4.467311984	8.451443573	6.629488387	7.419851705	7.32898565	6.85908071	6.270645889
ENSG00000163644	PPM1K	0.219184769	0.676973067	-0.882163295	0.051399479	-0.662978526	9.790941832	8.94259736	9.636156974	9.717397705	9.71245786	8.759935742	8.468021799
ENSG00000108823	SGCA	-6.969609488	0.004926903	6.305707469	0.000505952	-0.663902019	2	4.93638933	2	2	2	9.503020408	9.607555771
ENSG00000156170	NDUFAF6	-0.420383412	0.883547191	-0.243761687	0.932122597	-0.664145099	2	6.78762695	2	4.965943888	4.93258395	5.463319095	4.624919101
ENSG00000160570	DEDD2	0.138738393	0.870775814	-0.804406717	0.267163049	-0.665668324	8.468948322	8.522788974	9.849281147	9.303936679	9.15867957	8.502349881	8.061612696
ENSG00000100814	CCNB1IP1	-1.076106611	0.375913231	0.410403987	0.751265003	-0.665702623	9.097018025	8.623598428	5.59507254	7.402350992	7.21466714	8.922700729	8.609311074
ENSG00000010803	SCMH1	-0.277061528	0.879733274	-0.390452834	0.82326635	-0.667514361	4.00722531	8.147241016	9.491321578	8.111316053	8.15640714	7.770949957	8.236348546
ENSG00000132024	CC2D1A	-0.757746568	0.395924587	0.08933408	0.930031445	-0.668412488	8.367060731	7.638155693	9.670202709	8.113468729	7.99261031	9.038038079	8.72879856
ENSG00000243612		4.522200632	0.357169987	-5.191103674	0.309101085	-0.668903042	2	2	2	4.783303513	4.76938658	2	2
ENSG00000161999	JMJD8	0.55359923	0.591037548	-1.222796329	0.19277085	-0.669197099	8.228665467	9.125613817	6.534109959	8.860563059	8.86476809	7.043805932	7.184857568
ENSG00000125814	NAPB	0.12000917	0.946420592	-0.790736576	0.632809239	-0.670727406	3.327711234	8.623598428	7.225742779	7.731524332	7.56813436	6.797131715	6.735016458
ENSG00000134575	ACP2	-0.03312885	0.965522332	-0.643597838	0.329793525	-0.676726688	10.38028415	9.159587375	9.809100877	9.826399658	9.83811764	8.760386723	9.57619232
ENSG00000145882	PCYOX1L	-0.925209778	0.633232538	0.248125467	0.905614752	-0.677084311	8.517314938	7.467835385	2	6.604700657	6.6253296	7.92612656	7.561323797
ENSG00000197050	ZNF420	-0.637484772	0.736035171	-0.040096268	0.984191003	-0.67758104	2	7.315436877	6.882775709	6.024891728	5.87556634	7.021403672	5.736403255
ENSG00000204673	AKT1S1	-0.75201632	0.194034006	0.073894597	0.913418819	-0.678121723	10.63713535	9.540933215	9.935792933	9.318938646	9.40098777	9.908424552	10.41299269
ENSG00000081479	LRP2	0.903630838	0.780723424	-1.582436706	NA	-0.678805868	3.327711234	2	2	3.406930997	2.2680111	2.267001569	2.172608095
ENSG00000102241	HTATSF1	-0.533255052	0.615433095	-0.146526195	0.897819577	-0.679781247	9.159305284	10.22575081	7.552162999	8.80304041	8.84312657	9.126058498	9.283558215
ENSG00000204954	C12orf73	-0.939665952	0.603040858	0.25804034	0.894354862	-0.681625612	7.485143553	5.946638857	2	5.474671438	5.47317115	6.666558925	6.522764885
ENSG00000174720	LARP7	0.571068433	0.595406493	-1.252768244	0.201673674	-0.681699811	9.646202137	8.57407404	6.882775709	9.323594948	9.32954365	7.496683627	7.553536683
ENSG00000137574	TGS1	0.199041584	0.644240767	-0.880848614	0.016663213	-0.68180703	8.444143041	7.979858146	7.896971941	8.372666678	8.26762243	7.287316943	7.234405279
ENSG00000136156	ITM2B	-0.063490797	0.930223809	-0.620145547	0.314835008	-0.683636345	13.36497316	12.09476099	12.43892907	12.67459172	12.6714587	12.21628363	12.00990713
ENSG00000139725	RHOF	1.298616881	0.319708718	-1.982595458	0.111042614	-0.683978577	2	4.710564154	5.39244166	5.786686927	5.6826301	3.256555576	3.100202476
ENSG00000117713	ARID1A	-0.747495615	0.104588075	0.063481634	0.908444479	-0.684013981	10.51143645	10.88044608	9.999667074	9.740563462	9.78969165	10.42715191	10.70632165
ENSG00000106853	PTGR1	0.198460197	0.922914669	-0.884632563	0.635038067	-0.686172366	10.05124847	8.276714465	3.369317972	9.054539304	9.03275482	8.00436125	7.940451241
ENSG00000010539	ZNF200	-0.423210266	0.832296582	-0.263529404	0.895505527	-0.68673967	8.042902253	6.844429755	2	6.548530093	6.6253296	6.793610851	6.685996416
ENSG00000136897	MRPL50	-0.181051149	0.918272566	-0.505789887	0.760322555	-0.686841035	9.204317094	7.701025996	4.058998049	7.980622673	7.8286851	7.53725005	7.636966981
ENSG00000185627	PSMD13	0.097628454	0.942177035	-0.784822686	0.51368513	-0.687194231	10.45046729	10.42046463	7.163363831	9.987645515	10.0546329	9.096001355	9.189967637
ENSG00000003056	M6PR	0.506130278	0.527988504	-1.195215789	0.09771017	-0.689085511	10.87842776	9.648522413	9.196533591	10.59035845	10.5993844	8.841482668	8.959923085
ENSG00000160803	UBQLN4	-0.541871481	0.729601901	-0.147853209	0.92969272	-0.68972469	9.567995545	9.386925327	4.874587708	8.356399318	8.4215744	8.85040655	8.704390872
ENSG00000109472	CPE	-1.552499928	0.010454649	0.861916476	0.174415528	-0.690583452	7.435667355	8.101365653	8.721235202	6.716553866	6.60731796	9.306424362	8.688479312
ENSG00000198590	C3orf35	0.049413734	0.984043262	-0.740485644	0.752160737	-0.69107191	2	4.710564154	2	3.462030052	3.74316441	3.235948948	3.012034821
ENSG00000185885	IFITM1	2.734619264	1.48E-05	-3.42800425	2.95E-08	-0.693384986	11.06489013	11.97107825	12.69464022	14.73480042	14.8444446	8.878929913	8.359066013
ENSG00000163510	CWC22	0.108078671	0.914220207	-0.801476359	0.350702316	-0.693397688	6.468621203	8.414402626	8.408111533	8.160032699	8.06258142	7.418832227	6.990916783
ENSG00000143252	SDHC	-0.513034651	0.664949829	-0.181221083	0.885544215	-0.694255734	10.97683282	8.191702463	9.014156722	9.351221511	9.38345569	9.670982446	9.72342817
ENSG00000143748	NVL	0.49240785	0.768037744	-1.186696552	0.439027681	-0.694288701	8.468948322	7.503546079	3.369317972	7.942383523	8.04037874	6.194934299	6.512090157
ENSG00000169951	ZNF764	-0.513841021	0.570100628	-0.180942344	0.85102559	-0.694783365	6.366709671	7.954278496	6.322058544	6.580005553	6.65485813	6.840432491	7.030725399
ENSG00000258429	PDF	0.922452662	0.277913797	-1.617327019	0.041273153	-0.694874356	5.095959458	6.78762695	5.59507254	6.861921612	6.97351258	4.705557986	4.468068808
ENSG00000179262	RAD23A	-0.38832239	0.516281765	-0.306556222	0.608622814	-0.694878612	10.65345793	9.514922632	9.868959282	9.682298904	9.72712735	9.770158073	9.802212758
ENSG00000171202	TMEM126A	0.18854378	0.812790339	-0.88366746	0.194894738	-0.695123679	7.750913381	6.951736442	6.322058544	7.250225364	7.36516374	6.040114727	6.479583725
ENSG00000186625	KATNA1	-0.575170916	0.762607571	-0.119969386	0.952575081	-0.695140301	7.790810999	7.761270542	2	6.70517998	6.59518404	7.146744843	7.032593922
ENSG00000023041	ZDHHC6	0.014674799	0.989593043	-0.709856172	0.465968326	-0.695181373	9.33149386	8.356994601	6.80318718	8.551612617	8.4705529	7.81936672	7.775072826
ENSG00000100523	DDHD1	-0.86623261	0.442343803	0.167694651	0.893305945	-0.698537959	8.928409675	6.951736442	9.963514551	8.166267464	8.24644267	9.462574845	8.95451255
ENSG00000109220	CHIC2	0.272855103	0.838658392	-0.971937213	0.413080568	-0.699082109	6.468621203	7.701025996	9.624627483	8.765203682	8.76944977	7.441507444	7.633277551
ENSG00000160131	VMA21	-0.384977691	0.699347285	-0.314640192	0.752090127	-0.699617883	7.096806373	8.968383114	9.465702942	8.474993083	8.37797443	8.390261842	8.596104698
ENSG00000039650	PNKP	0.112777733	0.945043724	-0.81309559	0.584265208	-0.700317857	9.064836028	7.503546079	4.523675391	8.006336225	8.10598728	6.882471426	7.376535208
ENSG00000131187	F12	-1.573884004	0.322289819	0.873426203	0.581597072	-0.700457801	4.00722531	4.442710129	2	2.784612485	3.01487888	4.881704255	4.047883918
ENSG00000038274	MAT2B	0.209731134	0.531497817	-0.910221558	0.001387469	-0.700490424	9.33149386	9.74864196	9.491321578	9.75786892	9.73613428	8.679237241	8.589138522
ENSG00000068796	KIF2A	-0.096176004	0.91483416	-0.605494076	0.439526418	-0.70167008	9.613202746	8.234834549	8.076950877	8.75144642	8.69038176	8.387928811	8.024537783
ENSG00000189180	ZNF33A	-0.007768121	0.990196005	-0.694142437	0.183892387	-0.701910558	8.340423424	8.876041558	8.784770914	8.686416476	8.673169	8.356654601	7.529919928
ENSG00000104714	ERICH1	-0.355390101	0.716902068	-0.347574936	0.719914201	-0.702965037	8.138771685	7.051611315	5.930865648	6.917187326	7.00595521	6.894026089	7.041900409
ENSG00000178498	DTX3	-0.566066236	0.560330239	-0.139268549	0.89539314	-0.705334785	9.262228249	6.951736442	8.884815469	7.971157485	8.20512425	8.361418899	8.652479133
ENSG00000148814	LRRC27	-0.373731148	0.667273997	-0.331702542	0.699954446	-0.705433691	5.866944937	6.228547981	7.451345288	6.456560224	6.1572134	6.475428841	6.222348126
ENSG00000183628	DGCR6	3.483451168	0.001734804	-4.189830944	0.0005163	-0.706379776	4.467311984	2	4.874587708	7.214570819	7.36516374	2.267001569	2.225841198
ENSG00000177981	ASB8	-0.904564328	0.342300881	0.197703669	0.851990553	-0.70686066	9.112843761	8.903033433	6.718950348	7.691737974	7.66419009	8.673501845	8.851368118
ENSG00000143742	SRP9	0.311437891	0.339794204	-1.018435596	0.000322111	-0.706997705	9.602033055	9.532315021	9.735974718	9.971714969	9.89552277	8.768928693	8.438024223
ENSG00000117153	KLHL12	-0.761696312	0.208501379	0.05435047	0.939029498	-0.707345842	9.317904228	8.777382925	8.007629717	8.045195239	8.04484669	8.97493051	8.716646332
ENSG00000166598	HSP90B1	0.184182085	0.768318166	-0.893262949	0.095461226	-0.709080864	13.77366519	13.31367773	13.11599373	13.60545465	13.6187793	11.9668157	12.94188924
ENSG00000112062	MAPK14	-0.459810925	0.5253082	-0.250831467	0.735959689	-0.710642392	8.928409675	9.856140801	10.50627919	9.428470756	9.45737703	9.733646623	9.563001078
ENSG00000067064	IDI1	-0.249191734	0.609720423	-0.462582903	0.305744318	-0.711774637	10.31337612	10.3778065	10.37481416	10.14218788	10.0717013	9.429778618	10.24608161
ENSG00000230989	HSBP1	0.05615337	0.916366826	-0.768137428	0.076093247	-0.711984057	10.86447339	10.13758676	10.16797309	10.45981982	10.5113233	9.558011928	9.763920691
ENSG00000258947	TUBB3	2.731258988	0.103095955	-3.443750539	0.033898917	-0.712491551	4.815609471	7.393647515	2	8.737556707	8.73392024	3.235948948	3.36036153
ENSG00000134330	IAH1	-0.855988712	0.354286428	0.143106249	0.89090828	-0.712882463	7.031698319	8.820493869	9.399592459	7.836899669	7.89700047	8.855481238	8.852426354
ENSG00000086189	DIMT1	-0.463414708	0.501025025	-0.25058415	0.72430187	-0.713998858	8.564112515	7.847167909	7.163363831	7.641902724	7.37584259	7.807195765	7.634508409
ENSG00000148672	GLUD1	-0.554142247	0.172055101	-0.159942323	0.72045523	-0.71408457	10.34051281	9.770792087	10.40882047	9.641857585	9.64931004	9.909238196	10.15638509
ENSG00000101290	CDS2	-1.053419015	0.005630034	0.338205045	0.425433438	-0.71521397	9.810482605	10.32888058	10.55568472	9.205456697	9.22613117	10.54687782	10.65402549
ENSG00000145247	OCIAD2	-4.072265994	0.010739295	3.356105552	0.029365887	-0.716160442	6.73718411	4.710564154	2	2.784612485	2.59489879	7.804574291	9.308063313
ENSG00000133055	MYBPH	0.493741669	0.908850677	-1.210616419	0.763520469	-0.71687475	2	4.442710129	2	3.709785322	3.60476711	3.215043717	2
ENSG00000167637	ZNF283	-0.55273791	0.663582024	-0.1696176	0.900290106	-0.72235551	5.330594134	5.72254323	7.934808822	6.24507773	6.30607931	6.90385696	6.320441373
ENSG00000254656	RTL1	-3.753015698	0.531143362	3.030052067	0.594469008	-0.722963631	2	2	2	2	2	3.215043717	3.940519005
ENSG00000183655	KLHL25	-1.033074505	0.509300208	0.30855418	0.853872436	-0.724520325	3.327711234	6.04685525	7.818184403	5.742861563	5.60003091	7.227249878	6.592824411
ENSG00000066405	CLDN18	-3.753015734	0.531143362	3.027155226	0.594837647	-0.725860508	2	2	2	2	2	3.600766115	3.640730416
ENSG00000142173	COL6A2	-0.23343907	0.561212599	-0.492559317	0.18084322	-0.725998387	14.83498075	15.27905033	15.4147785	14.94766741	14.9789182	14.83726528	14.55780273
ENSG00000129451	KLK10	1.048071971	0.713118332	-1.774722936	NA	-0.726650966	3.327711234	2	2	2.868578001	3.15372745	2.267001569	2.117335275
ENSG00000120533	ENY2	0.389468241	0.410440873	-1.118428901	0.00730204	-0.72896066	8.946185348	8.317412869	8.236946975	8.941551548	8.89725143	7.31213785	7.545707309
ENSG00000115484	CCT4	-0.516912688	0.509536538	-0.214574049	0.795143796	-0.731486738	11.46391134	10.57950211	9.703463497	10.23507695	10.2462532	10.62559896	10.45348223
ENSG00000131941	RHPN2	-1.395983159	0.409632583	0.663470545	0.707028482	-0.732512615	6.138360429	7.315436877	2	5.058234798	4.97065356	6.775876478	7.073100803
ENSG00000013441	CLK1	0.227551906	0.759044086	-0.960181772	0.135257068	-0.732629866	8.586954094	8.981105197	9.91701143	9.537503411	9.45653888	8.557885437	8.034828728
ENSG00000114054	PCCB	-1.065878069	0.533389071	0.332987587	0.856270394	-0.732890482	9.27634997	8.623598428	3.369317972	7.363085426	7.37584259	8.617349501	8.865065003
ENSG00000162804	SNED1	-0.56809851	0.852516729	-0.165843844	0.957730283	-0.733942354	2	2	7.818184403	5.614989456	5.86549842	6.223999906	6.011156536
ENSG00000198754	OXCT2	1.403792126	0.527945306	-2.139465314	0.314064542	-0.735673188	2	2	4.523675391	4.237680128	4.37434574	2.42098416	2.421067459

ENSG00000007866	TEAD3	-1.182569814	5.52E-06	0.4462193	0.115808265	-0.736350515	9.829762239	9.608085847	9.478569125	8.425603456	8.51792306	10.06045791	10.11949997
ENSG00000171311	EXOSC1	-0.25209171	0.701991975	-0.485566511	0.42719519	-0.737658221	7.53297911	8.451443573	7.398165132	7.536750104	7.73502229	7.507470083	7.307219812
ENSG00000166938	DIS3L	-0.478369298	0.644658664	-0.260510374	0.807781951	-0.738879672	6.257048221	8.806266215	8.175061096	7.659688863	7.57432889	7.722704302	7.932467322
ENSG00000175591	P2RY2	-0.549092175	0.770226986	-0.191406919	0.921796599	-0.740499094	4.00722531	2	4.058998049	2.947924294	3.45167086	3.465698119	3.407202941
ENSG00000176715	ACSF3	-0.741623488	0.654365619	-0.000964374	0.999439688	-0.742587862	4.00722531	7.467835385	9.180874355	7.093295078	7.44845993	8.066950774	7.945418846
ENSG00000196227	FAM217B	-0.20961905	0.892020352	-0.53360601	0.708830978	-0.74322506	4.467311984	7.145017454	8.486635645	7.322721205	7.14562489	7.113241388	6.70252219
ENSG00000176909	MAMSTR	-1.042388583	0.45032434	0.298624224	0.838351035	-0.743764358	5.095959458	4.710564154	2	3.615725078	3.69849104	4.612704558	4.766376438
ENSG00000164542	KIAA0895	-2.051046402	0.076794225	1.304574033	0.2660773	-0.746472369	3.327711234	6.602424674	5.930865648	4.205751501	3.90918929	7.238947338	6.879055607
ENSG00000113248	PCDHB15	-0.482761088	0.848659281	-0.263829319	0.91758425	-0.746590406	2	5.131603625	2	3.566290607	3.55551834	3.500668998	3.8919413
ENSG00000105849	TWISTNB	-0.472807519	0.735527035	-0.275049971	0.848412544	-0.747857489	4.815609471	8.469613413	8.608698767	7.61176303	7.47849652	7.709686122	7.770601445
ENSG00000164938	TP53INP1	-0.721682378	0.226126239	-0.027389064	0.968966553	-0.749071441	8.340423424	9.583268459	9.344432377	8.480007165	8.45720682	9.270464658	9.033147652
ENSG00000132603	NIP7	-0.240170675	0.492183068	-0.508943782	0.10794541	-0.749114458	8.517314938	8.57407404	8.408111533	8.256693138	8.27714776	7.876232824	8.118139752
ENSG00000172247	C1QTNF4	-0.83569486	0.671489931	0.08650516	0.967162683	-0.749189701	4.00722531	3.076665209	2	2.695456494	2.94009902	3.355392153	3.236138336
ENSG00000131233	GJA9	1.895849372	0.478107313	-2.645114166	0.323165276	-0.749264794	2	2	3.369317972	3.289987342	3.69849104	2.048074405	2.172608095
ENSG00000167543	TP53I13	-0.447114158	0.216533153	-0.304285684	0.408760989	-0.751399842	9.533135543	9.148351514	9.703463497	9.052296807	9.00894176	9.22478876	9.118313725
ENSG00000067182	TNFRSF1A	-0.443807411	0.357081274	-0.308681762	0.527954208	-0.752489173	11.27990552	10.45735223	10.9651998	10.49161431	10.499165	10.42217269	10.81195485
ENSG00000164405	UQCRQ	-0.690048273	0.331987296	-0.062563889	0.938941066	-0.752612162	10.43795805	9.00621767	9.31604076	9.025111392	9.04952667	9.472793033	9.827456676
ENSG00000113734	BNIP1	-0.413104475	0.841034276	-0.339603665	0.867480863	-0.75270814	8.138771685	6.389896633	2	6.509830055	6.58294721	6.631488899	6.602920209
ENSG00000163374	YY1AP1	0.007381116	0.994991731	-0.760288974	0.408562547	-0.752907859	7.867441304	9.00621767	10.18377222	9.316137634	9.32587686	8.502889002	8.614310446
ENSG00000168906	MAT2A	0.297915708	0.392191303	-1.050952283	0.000451663	-0.753036575	10.71177538	10.79296235	11.17734896	11.19073463	11.2191332	9.94340063	9.771213272
ENSG00000139405	RITA1	-0.897382372	0.254887129	0.144282138	0.872268569	-0.753100233	8.674867122	7.051611315	8.65477198	7.395290829	7.42465798	8.108635266	8.699694604
ENSG00000143553	SNAPIN	-0.700203091	0.315751098	-0.054334941	0.945727701	-0.754538031	9.204317094	8.433041974	7.646393253	7.893113669	7.85436579	8.405911884	8.608684933
ENSG00000100568	VTI1B	-1.015884291	0.129065162	0.260397382	0.72665212	-0.755486909	10.34721797	9.030900487	9.099922433	8.592312513	8.64401667	9.984900111	9.779860367
ENSG00000134061	CD180	-3.753016105	0.531143362	2.997444774	0.599074031	-0.755571331	2	2	2	2	2	3.824280769	3.33635788
ENSG00000130803	ZNF317	-0.34059655	0.768745599	-0.415147762	0.713898124	-0.755744312	7.384433967	8.451443573	10.09465441	8.727753724	8.7297638	8.747705648	8.555744353
ENSG00000170017	ALCAM	-0.818554266	0.380566315	0.061354536	0.954328634	-0.757199729	10.19941743	12.56192683	11.91252756	11.03846779	11.0284211	11.67585929	12.11505304
ENSG00000020129	NCDN	-0.709516206	0.247942337	-0.049204948	0.9448613	-0.758721154	9.624286623	9.34816733	10.5187901	9.19431369	9.23104065	9.641172698	10.06667928
ENSG00000186184	POLR1D	-0.697000015	0.141109656	-0.062201313	0.911626073	-0.759201328	9.867565369	9.018611864	9.165043284	8.745906571	8.66448499	9.360076838	9.310760585
ENSG00000265681	RPL17	2.243812984	0.001788016	-3.003222343	1.68E-05	-0.759409359	9.371511179	7.819099893	7.896971941	10.84767009	10.7121781	5.367107572	5.888587685
ENSG00000102030	NAA10	-0.238628141	0.483277973	-0.521244258	0.090068933	-0.759872399	8.696033109	8.671479029	8.805342542	8.43079185	8.54182783	8.085066106	8.322112506
ENSG00000180658	OR2A4	4.430848314	0.379335003	-5.191101477	0.319243495	-0.760253163	2	2	2	4.71689672	4.68029874	2	2
ENSG00000186141	POLR3C	0.188434243	0.878396152	-0.948967804	0.369406917	-0.760533561	9.27634997	8.702538795	6.322058544	8.703665429	8.75179438	7.517109417	7.691205288
	LOC1002870	4.42963167	0.380422171	-5.191101266	0.320161353	-0.761469596	2	2	2	4.523237872	4.85329164	2	2
ENSG00000143228	NUF2	-0.305415355	0.863478741	-0.457459847	0.784860167	-0.762875202	3.327711234	3.686303414	4.058998049	3.79808662	3.15372745	2.911488689	3.789585079
ENSG00000132305	IMMT	-0.624315133	0.119466705	-0.138685612	0.759164528	-0.763000745	10.51143645	9.930175364	9.888372618	9.514066606	9.5188894	9.904553464	10.08929747
ENSG00000100211	CBY1	-0.844188375	0.618625373	0.080715812	0.965981107	-0.763472563	3.327711234	7.60566235	8.845626171	6.946463121	6.98750599	7.863694929	7.876348251
ENSG00000148090	AUH	0.086325479	0.910324107	-0.850604442	0.174826446	-0.764278963	6.653098118	6.728495673	5.59507254	6.401247806	6.60731796	5.511266259	5.736403255
ENSG00000163811	WDR43	-0.205956893	0.546145518	-0.558474945	0.065974684	-0.764431839	8.963744667	9.079034257	8.677268317	8.720710586	8.71442024	8.363795161	8.373881215
ENSG00000172732	MUS81	-0.870252996	0.236297945	0.10543871	0.901615613	-0.764814286	8.928409675	8.077869079	7.225742779	7.359462263	7.41087845	8.449073649	8.230662576
ENSG00000110075	PPP6R3	-0.255308503	0.743393183	-0.51062498	0.482463036	-0.765933482	9.219014269	9.842262059	10.79563557	9.828366803	9.85983387	9.439667847	9.726317257
ENSG00000100297	MCM5	-0.633613974	0.744136523	-0.132717644	0.948456219	-0.766331618	6.257048221	7.901717006	2	6.172600389	6.1068222	6.479817188	6.741885308
ENSG00000275111	ZNF2	-0.751537883	0.318559047	-0.015535465	0.985627735	-0.767073348	5.532353208	7.145017454	6.80318718	5.978405189	5.90535527	6.739740021	6.533361209
ENSG00000146457	WTAP	1.104051464	0.056526916	-1.871312113	0.00053196	-0.767260649	11.09308737	10.07961649	11.35530464	12.0672543	12.010716	8.917857451	9.213631806
ENSG00000178386	ZNF223	1.03095917	0.689206562	-1.79855974	0.457954601	-0.76760057	2	6.666836837	2	6.276156955	6.03677338	3.878466556	3.620995423
ENSG00000040487	PQLC2	0.048525777	0.97419422	-0.816136983	0.543323761	-0.767611206	8.928409675	8.077869079	4.874587708	8.040677544	8.12510181	7.318276922	7.149133029
ENSG00000169599	NFU1	0.098102766	0.948315617	-0.866485877	0.52982455	-0.768383111	8.653385977	7.274681896	4.523675391	7.64488235	7.7377845	6.85908071	6.640162436
ENSG00000146701	MDH2	-0.401839214	0.649861077	-0.366925034	0.677816167	-0.768764247	11.26227543	10.73029099	9.242516611	10.20511011	10.2578459	10.05697448	10.44946654
ENSG00000189184	PCDH18	-0.971954753	0.273930661	0.20287422	0.838475663	-0.769080533	8.910412251	11.05605593	10.91875185	9.585592789	9.60989979	10.95797194	10.55024182
ENSG00000143437	ARNT	-0.514955601	0.473719789	-0.254417888	0.732916452	-0.769373488	8.963744667	9.549500232	10.48730704	9.238378447	9.34321171	9.488938651	9.610060665
ENSG00000163714	U2SURP	0.082294193	0.788320904	-0.853303927	0.00040416	-0.771009734	9.895281535	9.672251278	9.658943166	9.873521491	9.77900397	8.910968188	8.88330014
ENSG00000148737	TCF7L2	0.111250568	0.912378494	-0.884793352	0.300068725	-0.773542784	7.624139768	9.030900487	7.163363831	8.262524677	8.31278108	7.052670333	7.528596482
ENSG00000257137	C12orf80	-3.753016332	0.531143362	2.979279574	0.601502996	-0.773736758	2	2	2	2	2	3.694366604	3.474731315
ENSG00000103160	HSDL1	-0.460196038	0.368923829	-0.314881125	0.54430284	-0.775071763	8.39321512	8.297207175	9.083172337	8.119907552	8.22887947	8.475683062	8.142569067
ENSG00000141562	NARF	-0.803470841	0.528830242	0.028345377	0.984153231	-0.775125464	9.730679447	9.170736405	6.073378139	8.149581172	8.18097132	9.031700669	8.943630224
ENSG00000174891	RSRC1	-0.09600776	0.912378494	-0.679615697	0.362166499	-0.775623457	6.366709671	7.145017454	8.042706615	7.246306984	7.24604864	6.775876478	6.580104496
ENSG00000161203	AP2M1	-0.711537532	0.194855335	-0.064464673	0.920627804	-0.776002205	12.92624669	11.92408613	12.80346116	11.90507685	11.9019417	12.28442381	12.77427944
ENSG00000187742	SECISBP2	0.271740744	0.682283472	-1.049402849	0.067723467	-0.777662105	8.696033109	7.60566235	7.646393253	8.310225403	8.36909406	7.046766788	7.043754524
ENSG00000083123	BCKDHB	-0.578093281	0.372026795	-0.202920521	0.769672064	-0.781013802	7.096806373	7.979858146	7.225742779	6.917187326	6.95938211	6.915242278	7.608437243
ENSG00000227500	SCAMP4	-0.299617992	0.612075895	-0.483490541	0.383684848	-0.783108533	10.3868074	9.71095217	10.76969939	10.00861625	10.0939697	9.63945754	10.06667928
ENSG00000172594	SMPDL3A	0.817711623	0.639687692	-1.600829744	0.322890646	-0.78311812	6.468621203	7.35507212	2	7.311513918	7.11195456	5.102662916	4.793076545
ENSG00000132199	ENOSF1	0.93093729	0.301027058	-1.714233072	0.039100734	-0.783295782	6.73718411	7.393647515	8.825624954	8.76109026	8.91200057	6.305369896	6.209190898
ENSG00000186001	LRCH3	-1.213156515	0.018412016	0.429814514	0.446243463	-0.783342001	9.423197387	8.297207175	8.865353883	7.717440541	7.74603955	9.285896341	9.423285228
ENSG00000174165	ZDHHC24	-0.887370916	0.326333176	0.102972339	0.920980141	-0.784398577	6.816637977	8.297207175	9.083172337	7.426792977	7.49817903	8.244430344	8.606804877
ENSG00000104133	SPG11	-0.182243705	0.751812007	-0.602994111	0.239452646	-0.785237816	10.21416435	9.170736405	9.935792933	9.672111811	9.63233954	9.254546025	9.213219973
ENSG00000157429	ZNF19	-0.706972789	0.715896274	-0.078355275	0.969731551	-0.785328065	2	5.72254323	3.369317972	3.840289928	4.0930011	4.588526421	4.329022995
ENSG00000100412	ACO2	-0.070239013	0.930680662	-0.716289889	0.296655486	-0.786528902	10.26462289	9.079034257	8.884815469	9.429335066	9.52049436	8.702892291	8.95451255
ENSG00000135046	ANXA1	0.148279409	0.838100556	-0.935432899	0.122999873	-0.787153489	12.2287862	11.12072186	11.18521898	11.7618096	11.7467334	10.37347689	10.92000025
ENSG00000170298	LGALS9B	4.403162088	0.386463861	-5.191100782	0.322255484	-0.787938695	2	2	2	4.623314311	4.72553021	2	2
ENSG00000087303	NID2	-1.784652458	0.04311869	0.996697504	0.281004483	-0.787954954	11.06489013	12.86533873	12.38869272	10.48912954	10.5165603	12.24081159	13.87892942
ENSG00000121578	B4GALT4	-0.628803586	0.323982764	-0.160058835	0.819657623	-0.788862425	9.720386367	8.671479029	8.561105505	8.564258394	8.34755546	8.803037464	9.035944835
ENSG00000120093	HOXB3	3.440116023	2.27E-06	-4.229033288	5.01E-08	-0.788917265	6.257048221	4.93638933	4.523675391	8.773395507	8.7017439	2.68698273	2.509591799
ENSG00000170545	SMAGP	-0.377645643	0.720482866	-0.411818067	0.690405965	-0.78946371	8.16936288	5.946638857	8.325066185	7.416368504	7.43490694	7.259511486	7.513957873
ENSG00000136940	PDCL	-0.498403694	0.315955574	-0.291951572	0.568416252	-0.790355266	8.493334308	8.337339476	9.180874355	8.27796118	8.16053033	8.360824221	8.485179747
ENSG00000180479	ZNF571	-1.18262253	0.51002601	0.390717577	0.837824475	-0.791904953	2	6.535001748	4.874587708	4.358765249	4.43005853	6.239610476	5.080008298
ENSG00000129515	SNX6	0.012819438	0.982918686	-0.8071713	0.093525732	-0.794351862	10.47516499	9.756063186	10.75919294	10.42297598	10.3742555	9.549686932	9.613809871
ENSG00000070756	PABPC1	-1.41941659	0.011400228	0.624991304	0.301662931	-0.794425285	12.09176592	13.33984242	12.62546556	11.38211482	11.3356213	13.17422418	13.59831928
ENSG00000173809	TDRD12	-3.753016599	0.531143362	2.95789763	NA	-0.795118969	2	2	2	2	2	4.111244182	2.70970617
ENSG00000151694	ADAM17	0.604207722	0.599217549	-1.400269442	0.17932719	-0.796061721	7.579279349	9.608085847	10.65536953	10.39138945	10.3116735	8.308125745	8.411703491
ENSG00000101040	ZMYND8	0.621322073	0.613329998	-1.417530458	0.205343649	-0.796208385	6.653098118	8.57407404	9.972637969	9.524619869	9.61140668	7.52137298	7.58443608
ENSG00000101928	MOSPD1	0.636176159	0.510282114	-1.432618851	0.101255863	-0.796442692	9.371511179	7.002537973	8.585098385	9.26185831	9.21725179	7.341370018	7.045606259
ENSG00000125779	PANK2	0.204708302	0.91483416	-1.001686296	0.558314161	-0.796977994	4.815609471	7.874700259	10.44859742	9.293531584	9.33502642	8.102956788	8.13650041
ENSG00000126214	KLC1	-0.078543467	0.903601303	-0.719650802	0.17883612	-0.798194269	10.81212061	9.672251278	10.19160738	10.19447291	10.2457682	9.614730018	9.54838268
ENSG00000186767	SPIN4	-0.507340314	0.825685372	-0.291486472	0.900758644	-0.798826787	3.327711234	6.899080544	2	5.040242897	5.04390545	5.50697268	4.895193906
ENSG00000003989	SLC7A2	-2.822325533	0.000804913	2.022984424	0.018492461	-0.799341108	7.53297911	6.602424674	8.325066185	4.946755474	5.0259382	10.20860099	8.706733286
ENSG00000170430	MGMT	-0.911892944	0.636476225	0.11214637	0.957713961	-0.799746574	8.138771685	8.297207175	2	6.580005553	6.9210099	7.564363052	7.927454842
ENSG00000107669	ATE1	-0.713252281	0.467358725	-0.087686419	0.936069485	-0.8009387	7.159102577	9.532315021	8.266922072	8.017874397	7.84670947	8.541185654	8.562873254
ENSG00000140854	KATNB1	-0.401060072	0.816824789	-0.404474633	0.811558361	-0.805534705	8.998234871	7.145017454	4.058998049	7.437142666	7.37938471	6.995586672	7.72395257
ENSG00000011105	TSPAN9	-1.258169275	0.059358025	0.451020717	0.535037046	-0.807148558	10.1076228	8.540087354	9.272377397	8.119907552	8.27144011	9.76634617	10.00971824
ENSG00000075239	ACAT1	-0.452618404	0.839051597	-0.355207014	0.872932235	-0.807825418	9.48530526	8.717821467	2	8.087422161	8.16258751	7.813294077	8.53941009
ENSG00000008300	CELSR3	-0.362106571	0.872289176	-0.44614836	0.837960391	-0.808254931	2	5.595927442	8.296287027	6.456560224	6.6952083	6.747040087	6.202567011
ENSG00000147130	ZMYM3	-0.86215367	0.045918436	0.053690538	0.919423567	-0.808463132	9.509418609	9.726146444	8.922968322	8.491639362	8.64989445	9.378702006	9.577792788
ENSG00000103852	TTC23	-0.390353436	0.618255555	-0.418111507	0.584589142	-0.808464943	7.790810999	8.029695761	9.287079172	8.124184191	8.15227214	8.17644121	8.042267212
ENSG00000125285	SOX21	1.504773636	NA	-2.31353874	NA	-0.808765105	2	2	2	3.289987342	2	2.139668783	2
ENSG00000142686	C1orf216	-0.424151114	0.856794892	-0.387222396	0.866925741	-0.81137351	10.14658276	8.469613413	2	8.50645854	8.56844956	8.446833885	8.691439168
ENSG00000164815	ORC5	0.196939654	0.903601303	-1.00927417	0.478299157	-0.812334516	7.031698319	7.701025996	3.369317972	6.931899482	7.18258783	6.075339068	5.727202496
ENSG00000174876	AMY1B	4.378401314	0.396344882	-5.191099045	0.329252781	-0.812697731	2	2	2	5.003571641	4.1930041	2	2
ENSG00000187733	AMY1C	4.378401314	0.396344882	-5.191099045	0.329252781	-0.812697731	2	2	2	5.003571641	4.1930041	2	2
ENSG00000237763	AMY1A	4.378401314	0.396344882	-5.191099045	0.329252781	-0.812697731	2	2	2	5.003571641	4.1930041	2	2
ENSG00000174871	CNIH2	-1.317562515	0.484134885	0.504847856	0.798479518	-0.812714659	3.327711234	5.946638857	2	3.615725078	3.74316441	5.152992171	5.122735884
ENSG00000113013	HSPA9	0.035813284	0.922635322	-0.849958115	0.002681332	-0.814144831	11.61323974	11.14918383	11.31999735	11.41040523	11.4063384	10.52620926	10.52258738
ENSG00000165502	RPL36AL	-0.756669681	0.175327851	-0.058776214	0.929596218	-0.815445895	10.93293345	9.890264534	10.43545992	9.70102904	9.75056618	10.11225475	10.67571014
ENSG00000141385	AFG3L2	-0.394929577	0.781110464	-0.421431842	0.762852238	-0.816361419	6.563806279	8.623598428	10.37481416	8.821593044	8.87609528	8.717365994	8.921111931
ENSG00000145740	SLC30A5	-0.188498162	0.747712189	-0.628940987	0.226495503	-0.817439149	9.533135543	9.994333773	8.978369997	9.389919188	9.36392108	9.106681409	8.750531435
ENSG00000112679	DUSP22	-1.517154802	0.399795702	0.698289329	0.712878225	-0.818865473	3.327711234	7.431218269	9.681375057	6.877205252	6.9210099	9.204383073	8.935663927
ENSG00000068912	ERLEC1	0.131133123	0.784300093	-0.950665033	0.01565797	-0.81953191	10.48735708	11.22601381	10.7904855	11.02423622	10.9741121	10.02786376	9.804948449
ENSG00000101856	PGRMC1	0.063372714	0.910745594	-0.883821062	0.048909865	-0.820448348	11.23361136	10.33384851	10.62096771	10.85508643	10.8278498	9.914515727	9.879774129
ENSG00000139832	RAB20	-0.69209745	0.687848199	-0.130509183	0.943446144	-0.822606633	2	6.389896633	6.882775709	5.461211713	5.45984938	6.132200015	5.807970301

ENSG00000133895	MEN1	-0.4141856	0.258819968	-0.410037778	0.253726618	-0.824223378	8.910412251	8.732943944	8.408111533	8.283707551	8.29788471	8.382079673	8.20189164
ENSG00000088986	DYNLL1	-0.747653177	0.168573196	-0.078885901	0.901746027	-0.826539078	11.59645304	10.47993704	11.20470823	10.41208663	10.4222654	10.92667044	11.22656376
ENSG00000171843	MLLT3	-1.00193187	0.020898129	0.174727391	0.728258838	-0.827204479	7.904282243	8.469613413	8.677268317	7.437142666	7.36516374	8.69913345	8.403784899
ENSG00000111665	CDCA3	-1.099058047	0.461217823	0.271075497	0.865035836	-0.82798255	3.327711234	5.595927442	3.369317972	3.615725078	3.82856379	4.443291373	5.08721802
ENSG00000123836	PFKFB2	-1.124398403	0.417004646	0.295614931	0.844695037	-0.828783472	5.532353208	5.595927442	8.561105505	6.29903474	6.11534392	7.800194554	7.321046127
ENSG00000144021	CIAO1	-0.556662669	0.535148173	-0.272381801	0.772041492	-0.829044471	8.468948322	9.890264534	10.65536953	9.360314042	9.3808074	9.661597094	9.64498544
ENSG00000123352	SPATS2	0.08295674	0.888897361	-0.912174398	0.055208449	-0.829217658	9.460782017	9.424669273	8.631919287	9.239364442	9.36749255	8.503427922	8.106655408
ENSG00000206073	SERPINB4	4.361726697	0.395913674	-5.19109977	0.326816217	-0.829373073	2	2	2	4.647285135	4.63360314	2	2
ENSG00000163106	HPGDS	-3.753017028	0.531143362	2.923522658	NA	-0.829494371	2	2	2	2	2	4.122514202	2.592996859
ENSG00000111775	COX6A1	-0.153334045	0.888333431	-0.67660597	0.480204433	-0.829940015	10.95065381	8.806266215	9.212024683	9.808573603	9.8342512	9.283701895	9.318056434
ENSG00000156256	USP16	-0.121597233	0.86184497	-0.70848483	0.230307325	-0.830082063	9.848787621	8.806266215	8.742726175	9.115954467	9.09332116	8.469076579	8.575104441
ENSG00000051009	FAM160A2	-0.803891693	0.711497595	-0.026200495	0.990893061	-0.830092188	2	7.35507212	9.426401271	7.377487766	7.35440525	8.085066106	8.17168765
ENSG00000174226	SNX31	-0.689658684	0.830335275	-0.140441925	NA	-0.830100609	2	2	3.369317972	2.270786764	2.49394783	2.717032197	2.326754077
ENSG00000107164	FUBP3	-0.211167459	0.573001609	-0.619987338	0.061569555	-0.831154798	9.358295023	9.424669273	9.735974718	9.347568393	9.2591908	8.737663157	9.042914155
ENSG00000008056	SYN1	-0.366213846	0.86184497	-0.465427973	0.818406267	-0.83164182	4.00722531	6.666836837	2	5.076005082	5.00774437	5.136410075	4.74829783
ENSG00000123416	TUBA1B	0.555260257	0.446354696	-1.387157924	0.033296326	-0.831897667	11.87457535	11.31160468	10.81097638	11.97200261	11.9319504	9.288712876	10.49422299
ENSG00000161277	THAP8	-0.783428623	0.79343476	-0.049023761	0.987416408	-0.832452384	2	8.255926468	2	5.829220145	6.09824986	6.653021698	6.681239787
ENSG00000187609	EXD3	-0.907111839	0.628146304	0.074333621	0.97068214	-0.832778218	7.975256505	6.666836837	2	5.997179931	6.06344184	7.11465301	6.804465095
ENSG00000096093	EFHC1	-0.862562624	0.657619829	0.02643922	0.989740833	-0.836123405	2	8.004992147	8.380957451	6.856790872	6.72335419	7.631969638	7.680519528
ENSG00000105810	CDK6	-0.613054718	0.222830111	-0.223549996	0.681431908	-0.836604713	9.895281535	10.4709455	10.63252637	9.799243015	9.71034999	9.788404752	10.42815449
ENSG00000112249	ASCC3	-0.666142661	0.413198702	-0.171414284	0.847380153	-0.837556945	9.143983763	10.48887288	11.04953186	9.778365132	9.74233706	10.42644164	10.05638425
ENSG00000003509	NDUFAF7	-0.500746205	0.685162585	-0.337368504	0.78794815	-0.838114709	6.009025997	6.728495673	8.784770914	7.25803034	7.09050444	7.341370018	7.330190599
ENSG00000174606	ANGEL2	-0.053196802	0.948155695	-0.786841734	0.266613509	-0.840038536	7.485143553	7.538394143	8.825624954	8.013270196	8.05374131	7.462723726	7.149133029
ENSG00000092841	MYL6	-0.924475435	0.082516252	0.083995036	0.89539314	-0.840480399	13.99047696	12.86619626	13.43923225	12.56285085	12.5963066	13.42632465	13.73277744
ENSG00000108788	MLX	-0.366266811	0.846600015	-0.474284399	0.795688254	-0.84055121	10.73241055	7.60566235	5.39244166	8.973900679	8.96835847	8.999589189	8.714319971
ENSG00000149554	CHEK1	0.074734476	0.95872661	-0.915626936	0.476899959	-0.84089246	8.257427002	7.002537973	4.523675391	7.423326516	7.21862736	6.442083733	6.29264319
ENSG00000124155	PIGT	-0.722037561	0.21973507	-0.119196381	0.858897472	-0.841233943	11.69698977	10.5284131	10.81605395	10.38070504	10.3835453	10.85732303	11.09951659
ENSG00000112320	SOBP	-2.600639488	0.12809176	1.758051981	0.301787266	-0.842587507	2	6.228547981	4.058998049	2.947924294	3.28037825	7.123093858	5.957088417
ENSG00000182208	MOB2	-0.370245189	0.835111495	-0.473034216	0.783596985	-0.843279404	4.00722531	8.053983481	9.385998894	7.847284664	8.0314011	8.115702075	7.501869356
ENSG00000100592	DAAM1	-0.132274982	0.91483416	-0.711760248	0.51563255	-0.844035231	6.563806279	9.34816733	9.358421373	8.750063451	8.72837565	8.203226602	8.128660152
ENSG00000148572	NRBF2	-0.336507198	0.539576503	-0.508095075	0.327879239	-0.844602272	9.262228249	8.255926468	9.049077186	8.540455898	8.61275919	8.431623835	8.382697855
ENSG00000149243	KLHL35	-1.37566174	0.396224049	0.530893528	0.757048961	-0.844768212	2	5.595927442	6.534109959	4.54891529	4.22485516	6.430795201	5.631815919
ENSG00000167397	VKORC1	-0.257793347	0.634741192	-0.587732727	0.23570663	-0.845526074	10.62615013	10.09139839	11.0961933	10.37846912	10.4269797	10.01071781	10.13480234
ENSG00000188706	ZDHHC9	-0.281238257	0.718010015	-0.565042876	0.435161964	-0.846281133	9.699577239	9.842262059	11.12920538	10.09721189	10.0934307	9.765223103	9.858600332
ENSG00000099308	MAST3	-0.731270766	0.587266932	-0.115025835	0.937781729	-0.846296601	7.384433967	8.993716073	5.39244166	7.093295078	7.29187696	8.108635266	7.402777233
ENSG00000078487	ZCWPW1	-0.305189258	0.912501485	-0.541184491	0.839285386	-0.846373749	2	6.389896633	2	4.739373666	4.65713986	4.555645384	4.445805334
ENSG00000087206	UIMC1	-0.461885165	0.57369092	-0.385043515	0.639193832	-0.846928681	8.946185348	7.315436877	7.646393253	7.694616532	7.69009211	7.759264603	7.776188509
ENSG00000127955	GNAI1	0.90491794	0.445130278	-1.751923622	0.10692029	-0.847005682	9.590776211	7.954278496	6.43197674	9.452477833	9.3913715	6.932953913	6.674075308
ENSG00000144034	TPRKB	0.020338142	0.981338611	-0.867481653	0.218585328	-0.847143511	6.468621203	5.303523747	5.772716771	5.987823101	5.86549842	4.959178705	5.263040529
ENSG00000102974	CTCF	-0.589671174	0.696183014	-0.258014716	0.869968927	-0.84768589	5.330594134	9.225224669	9.868959282	8.461536372	8.44034872	8.966355803	8.564165641
ENSG00000241945	PWP2	2.380060642	0.015303223	-3.228098505	0.000876588	-0.848037863	6.366709671	4.11352228	4.523675391	7.587189552	7.59884443	2.911488689	3.042026731
ENSG00000124357	NAGK	0.028049655	0.969963817	-0.876392798	0.155720174	-0.848343143	10.05124847	9.863030423	8.699419242	9.604082348	9.7485133	8.63860224	8.908448207
ENSG00000135775	COG2	-0.810240914	0.394385857	-0.039564334	0.970602478	-0.849805248	6.257048221	8.590771778	8.325066185	7.119236776	7.34718809	7.993660487	7.991296779
ENSG00000147439	BIN3	0.512694328	0.380422171	-1.362681562	0.0088054	-0.849987234	6.89194353	7.901717006	7.97167868	8.117764469	8.22691474	6.257611854	6.437892833
ENSG00000111554	MDM1	-0.253497527	0.895265695	-0.59688528	0.737997178	-0.850382807	7.27616306	6.535001748	2	6.252910529	6.04571775	6.126613714	5.459179378
ENSG00000087258	GNAO1	-1.646812564	0.438555858	0.795900003	0.711952414	-0.850912561	2	4.442710129	3.369317972	3.09461492	2.2680111	4.652122235	3.580696614
ENSG00000215218	UBE2QL1	-0.634066049	0.805573299	-0.217756881	0.934077269	-0.85182293	2	4.442710129	2	3.289987342	2.68924543	3.355392153	3.128433649
ENSG00000175104	TRAF6	-0.214395175	0.913299181	-0.639036511	0.726284264	-0.853431687	9.48530526	7.928237109	3.369317972	8.203120604	8.04484669	7.954782214	7.411419562
ENSG00000132846	ZBED3	-3.430389364	0.00368158	2.576928351	0.019796165	-0.853461013	3.327711234	5.946638857	4.058998049	2.695456494	2.68924543	7.310906894	7.376535208
ENSG00000165511	C10orf25	-4.71493145	0.159913355	3.86095831	0.168877492	-0.85397314	2	3.076665209	2	2	2	5.238348159	4.80186789
ENSG00000157191	NECAP2	-0.608128088	0.349396138	-0.246852991	0.721502431	-0.854981078	10.98974631	9.664384941	10.7325871	9.878593178	10.032869	10.11190139	10.49794712
ENSG00000159882	ZNF230	-0.283145771	0.890436443	-0.572832591	0.766433306	-0.855978362	2	5.838937808	7.646393253	6.113598934	6.22964624	5.991769846	5.803599879
ENSG00000122218	COPA	-0.280481638	0.822518774	-0.576360313	0.620145964	-0.856841951	9.613202746	12.42197616	12.73541118	11.78647256	11.835317	11.44398809	11.58379362
ENSG00000172748	ZNF596	0.032164838	0.99113768	-0.889151603	0.736787432	-0.856986765	2	6.728495673	2	5.22679461	5.36298494	4.757085024	4.188602707
ENSG00000221821	C6orf226	-0.299607266	0.848265166	-0.557922518	0.70356954	-0.857529784	6.816637977	4.11352228	4.058998049	5.348759107	5.30456641	5.147485955	5.04340913
ENSG00000164221	CCDC112	-0.411690879	0.793896601	-0.446247973	0.772041492	-0.857938851	3.327711234	5.72254323	7.285536013	5.949776857	5.62411716	6.019162896	5.453611367
ENSG00000184937	WT1	4.333155878	0.402624908	-5.191099051	0.329804519	-0.857943173	2	2	2	4.623314311	4.60967607	2	2
ENSG00000176371	ZSCAN2	0.148764131	0.926779701	-1.007828347	0.478389629	-0.859064216	3.327711234	7.431218269	7.502634626	7.044488801	7.09482005	5.825876956	6.092227206
ENSG00000117174	ZNHIT6	-0.628005157	0.185239825	-0.231210621	0.6505088	-0.859215778	7.867441304	8.623598428	8.631919287	7.860161659	7.7377845	8.064031095	8.307525005
ENSG00000170456	DENND5B	-0.549889132	0.599957698	-0.309392507	0.774853287	-0.859281638	6.816637977	9.090820894	9.372276026	8.272191829	8.20512425	8.654217494	8.273956186
ENSG00000102471	NDFIP2	-0.717865055	0.412901143	-0.142063649	0.884519325	-0.859928703	7.624139768	9.415325457	9.670202709	8.576794289	8.26953254	8.868927112	9.123144518
ENSG00000203995	ZYG11A	-1.231491347	0.362148669	0.369762653	0.797269964	-0.861728694	5.532353208	3.076665209	4.058998049	3.515101962	3.69849104	4.68289886	5.005857272
ENSG00000153721	CNKSR3	-1.45282579	0.222084596	0.589632032	0.642308336	-0.863193758	5.330594134	6.228547981	8.325066185	5.949776857	5.6121243	7.703132714	7.799420735
ENSG00000123395	ATG101	-0.111301012	0.960285623	-0.751979112	0.722957397	-0.863280124	9.602033055	9.170736405	2	8.731963162	8.69180693	8.061837451	8.095972716
ENSG00000135211	TMEM60	0.08684711	0.863599094	-0.950444913	0.017855055	-0.863597803	7.667647223	7.954278496	8.380957451	8.109160159	8.11663771	7.165935143	7.025105265
ENSG00000149483	TMEM138	-0.533020446	0.431896348	-0.330604232	0.633167305	-0.863624678	9.015175615	7.669933291	8.922968322	8.18685754	8.04707549	8.294489071	8.341838298
ENSG00000182318	ZSCAN22	-0.71392501	0.227604787	-0.150860318	0.818089356	-0.864785327	6.257048221	6.602424674	6.322058544	5.578054239	5.88556448	6.471027105	6.029985803
	SQRDL	7.931448953	2.55E-05	-8.797640877	0.000397994	-0.866191925	2	5.303523747	2	11.5430919	11.5891353	2	2
ENSG00000030582	GRN	0.084565189	0.89152261	-0.952636948	0.055557322	-0.868071759	13.24264217	12.43127714	12.26418878	12.78278409	12.810703	11.66933017	11.84618268
ENSG00000135316	SYNCRIP	-0.024408915	0.957978247	-0.844214721	0.023138941	-0.868623636	10.91499272	10.67655334	10.29705308	10.65570314	10.5990046	9.63331528	9.968585649
ENSG00000240654	C1QTNF9	2.775429691	0.558941971	-3.645545009	NA	-0.870115319	2	2	2	3.289987342	3.60476711	2	2.059860178
ENSG00000118096	IFT46	-0.353280875	0.841155419	-0.517607621	0.758720739	-0.870888496	3.327711234	7.467835385	8.561105505	7.210554257	7.21069602	6.977080688	7.12130792
ENSG00000198799	LRIG2	-0.625671101	0.778610361	-0.246398313	0.915259822	-0.872069413	2	6.311476754	8.978369997	6.960881212	7.04216061	7.538302335	7.18149309
ENSG00000117707	PROX1	1.729358872	0.39960716	-2.6023719	0.193600095	-0.873013028	2	4.11352228	2	4.358765249	4.3456612	2.3070652	2.225841198
ENSG00000001167	NFYA	0.196518321	0.922962824	-1.069884866	0.554691707	-0.873366545	3.327711234	8.395519306	9.849281147	8.922033725	8.91566441	7.988280194	7.258555286
ENSG00000089163	SIRT4	-0.770774745	0.762599354	-0.103624975	0.969279885	-0.874399721	2	5.72254323	2	3.709785322	3.86943969	4.580376151	3.940519005
ENSG00000242802	AP5Z1	0.151358295	0.897065049	-1.025859389	0.301496071	-0.874501094	10.14658276	7.538394143	8.742726175	9.260887574	9.40446877	8.246363378	8.086110269
ENSG00000168259	DNAJC7	0.431926879	0.59184685	-1.307191403	0.066431499	-0.875264524	9.081016759	8.732943944	10.34701993	9.956206375	10.0238444	8.147081218	8.373144057
ENSG00000173889	PHC3	-0.523221719	0.605635278	-0.352201142	0.731545698	-0.875422861	9.031919741	9.405920729	11.08361639	9.672841851	9.54908243	10.12491852	9.332538367
ENSG00000138069	RAB1A	-0.362273235	0.611600602	-0.515207353	0.448615444	-0.877480588	11.99046984	10.71514053	10.76445573	10.93819728	10.9088206	10.74627917	10.79526758
ENSG00000174429	ABRA	-3.753017631	0.531143362	2.875241323	NA	-0.877776308	2	2	2	2	2	3.943456545	2.918126211
ENSG00000018699	TTC27	-0.424746247	0.694658127	-0.454282726	0.669330321	-0.879028973	6.73718411	6.602424674	8.699419242	7.303993732	7.23044321	7.118879607	7.349806994
ENSG00000116161	CACYBP	-0.513470559	0.199526015	-0.366654998	0.364637341	-0.880125557	8.653385977	9.256956408	8.825624954	8.381625466	8.48213028	8.492068113	8.659146771
ENSG00000175463	TBC1D10C	-4.714929056	0.166109341	3.834444079	0.180853	-0.880484976	2	3.076665209	2	2	2	5.343222328	4.584731282
ENSG00000112972	HMGCS1	-0.548976929	0.520760111	-0.332844435	0.702841297	-0.881821364	10.4253394	10.9629268	11.04521411	10.31066614	10.2664796	9.284329221	11.15567751
ENSG00000156976	EIF4A2	-0.898575935	0.021970728	0.016739917	0.972606493	-0.881836018	12.07772327	11.34889202	11.87652621	10.90191807	10.9002162	11.92100809	11.70048584
ENSG00000141198	TOM1L1	0.043629322	0.98350331	-0.925990102	0.632223509	-0.882360779	2	6.04685525	8.042706615	6.872128674	6.79936111	5.747972572	6.06327132
ENSG00000115468	EFHD1	-5.20880374	0.001784151	4.325195715	0.007589468	-0.883608025	2	5.838937808	7.818184403	2.947924294	2.38539787	10.96796381	10.70295227
ENSG00000052850	ALX4	-4.644542973	0.032131199	3.760189132	0.052358157	-0.884353841	2	5.595927442	2	2.270786764	2.14022021	6.855708005	8.277112026
ENSG00000137473	TTC29	-3.753017715	0.531143362	2.868543381	NA	-0.884474334	2	2	2	2	2	3.1504466	3.807156736
ENSG00000152760	TCTEX1D1	-2.16178391	0.34076194	1.276455785	0.56576762	-0.885328125	2	4.442710129	2	2.3892346	2.49394783	3.838019203	4.634793542
ENSG00000146085	MUT	-0.80078433	0.002905845	-0.087459749	0.788768394	-0.888244079	8.609439662	8.777382925	8.721235202	7.947219213	7.88459112	8.594261972	8.645780537
ENSG00000152527	PLEKHH2	-1.702469625	0.193080814	0.813509091	0.553184301	-0.888960533	4.00722531	7.099070191	8.110401118	5.590471342	5.49945122	7.891135869	8.017950573
ENSG00000148459	PDSS1	-0.348869546	0.869037712	-0.541603384	0.78794815	-0.89047293	3.327711234	5.946638857	2	4.497095146	4.31639476	4.065262523	4.4231929
ENSG00000159784	FAM131B	-2.353108564	0.171142026	1.462005178	0.404583432	-0.891103386	4.00722531	3.076665209	7.502634626	4.205751501	3.86943969	7.616110375	7.363232998
ENSG00000160200	CBS	2.06449838	0.095138308	-2.956868203	0.01263727	-0.892369824	7.031698319	5.303523747	3.369317972	7.885575888	7.909304	3.500668998	3.474731315
ENSG00000136492	BRIP1	0.050874581	0.96670681	-0.943921186	0.372909708	-0.893046605	6.009025997	5.131603625	7.600047325	6.710878132	6.57678965	5.843010798	5.555894927
ENSG00000119446	RBM18	-0.350174233	0.471757988	-0.545589037	0.230518351	-0.89576327	9.371511179	8.57407404	8.805342542	8.524366558	8.68323472	8.474034275	8.348604099
ENSG00000168010	ATG16L2	-0.80248032	0.688658118	-0.094194107	0.965819335	-0.896674427	2	7.274681896	8.561105505	6.658765962	6.74548212	7.583859056	7.164551992
ENSG00000111087	GLI1	-1.463685389	0.38727917	0.567007642	0.749352532	-0.896677747	2	5.838937808	4.058998049	3.840289928	3.33976443	5.253805068	5.211187127
ENSG00000172115	CYCS	-0.465498577	0.304264796	-0.431974514	0.333367096	-0.897473092	9.128497779	9.821190352	9.714382178	9.175891964	9.07159009	8.96830908	9.325696621
ENSG00000204965	PCDHA5	1.375664444	0.734590946	-2.273389914	NA	-0.89772547	2	3.076665209	2	3.566290607	2.68924543	2.267001569	2
ENSG00000107937	GTPBP4	-0.269704631	0.617709569	-0.628716929	0.199257862	-0.898421561	10.21416435	9.267380431	9.746650996	9.51325161	9.53247497	9.010625386	9.306519748
ENSG00000015479	MATR3	-0.598687344	0.315977132	-0.300077566	0.632223509	-0.898764911	10.59268348	11.60869109	11.79401137	10.84151467	10.8026781	11.25410279	10.97265421
ENSG00000170260	ZNF212	-0.317940103	0.846875777	-0.58159067	0.707642617	-0.899530773	4.00722531	7.145017454	8.608698767	7.178012736	7.22257674	6.779440836	7.09293558
ENSG00000169692	AGPAT2	-0.476854282	0.303980957	-0.422825724	0.357318652	-0.899680006	10.07567912	9.28800521	9.439621147	9.155143657	9.17813013	9.269197611	9.169734363
ENSG00000163931	TKT	-0.262807629	0.764891131	-0.637000343	0.42991362	-0.899807973	12.54246216	11.12645935	10.87075861	11.44585997	11.4554086	10.87814707	11.25184716

ENSG00000189306	RRP7A	-0.521224982	0.600951592	-0.379137277	0.706382819	-0.900362259	7.218819781	9.433952963	9.577565424	8.559529208	8.53549186	8.713178889	8.662770717
ENSG00000072736	NFATC3	-0.335498078	0.699202151	-0.565903784	0.487891953	-0.901401862	8.564112515	8.147241016	9.888372618	8.676258519	8.78422202	8.571313099	8.431665941
ENSG00000263012		4.289461303	0.413324098	-5.191097924	0.334615401	-0.901636622	2	2	2	4.623314311	4.53541753	2	2
ENSG00000126903	SLC10A3	-0.595105891	0.406922957	-0.306929649	0.681460704	-0.902035539	8.228665467	9.256956408	9.849281147	8.62888906	8.70032852	8.973764228	8.927151259
ENSG00000176435	CLEC14A	0.411845342	0.872658363	-1.313930786	0.57016511	-0.902085444	2	4.93638933	2	3.79808662	4.2560182	3.172301926	2.70970617
ENSG00000188243	COMMD6	-0.609410265	0.543796126	-0.292932725	0.781011291	-0.902342989	9.344956677	6.951736442	8.076950877	7.917958914	7.75424764	8.260460366	8.030160124
ENSG00000122566	HNRNPA2B1	0.129803125	0.859640066	-1.033409471	0.086189352	-0.903606346	12.53953777	11.56288089	11.58978828	12.12053612	12.0843183	10.48450807	11.28720059
ENSG00000170348	TMED10	-0.43056494	0.405040536	-0.473581285	0.34601665	-0.904146225	11.6462383	12.26046845	12.60507648	11.78883165	11.7967636	11.58578877	11.89713248
ENSG00000185000	DGAT1	-0.497127047	0.173242934	-0.409888911	0.258351845	-0.907015959	8.19931886	8.317412869	7.896971941	7.731524332	7.59884443	7.793159202	7.710008939
ENSG00000196352	CD55	0.14904633	0.843023983	-1.056777029	0.090455282	-0.907730699	9.460782017	9.04308532	10.41552659	9.893075744	9.91456814	8.827346937	8.573180082
ENSG00000111726	CMAS	0.076956106	0.932714208	-0.986120532	0.19694971	-0.909164426	9.810482605	8.848535039	7.934808822	9.073461088	9.20032903	8.18049086	7.985520618
ENSG00000105270	CLIP3	-1.243946507	0.000578198	0.334741296	0.415479043	-0.909205211	10.70657014	10.86336859	10.31150679	9.420668575	9.39487575	10.75037144	11.17958994
ENSG00000117523	PRRC2C	-0.368748207	0.599701246	-0.540509066	0.418666323	-0.909257273	12.99617415	11.77499027	11.7548855	11.91590947	11.9470968	11.75034236	11.76961671
ENSG00000143633	C1orf131	-0.42514176	0.657619829	-0.484306584	0.60340423	-0.909448344	6.816637977	6.602424674	4.874587708	5.764940652	6.0722229	5.606648123	6.095806168
ENSG00000248587	GDNF-AS1	-3.753018043	0.531143362	2.842223862	NA	-0.910794181	2	2	2	2	2	4.177580368	2.172608095
ENSG00000101444	AHCY	-1.491125563	0.100898224	0.579714891	0.553494044	-0.911410673	8.19931886	10.71514053	9.972637969	8.488325436	8.46556258	10.45039667	10.61574244
ENSG00000006625	GGCT	-0.436415669	0.75303653	-0.475268398	0.726905737	-0.911684066	4.467311984	7.503546079	8.266922072	7.062428156	6.91125523	6.942523933	6.959831579
ENSG00000131067	GGT7	-0.991296372	0.32983595	0.078176498	0.946354454	-0.913119874	9.128497779	6.78762695	9.358421373	7.734324667	7.8745862	8.60534994	9.079977539
ENSG00000132485	ZRANB2	0.267577005	0.793086452	-1.181043444	0.17880563	-0.913466439	8.468948322	9.45234302	10.7325871	10.15112202	10.0656679	8.792492404	8.534803159
ENSG00000123342	MMP19	-0.343216791	0.599957698	-0.57266796	0.350332806	-0.91588475	8.540903468	9.00621767	8.076950877	8.18685754	8.32385328	7.567458938	8.381232151
ENSG00000141965	FEM1A	0.958753507	0.440574326	-1.874788236	0.099916399	-0.916034729	4.815609471	7.60566235	8.380957451	8.447952961	8.50663048	5.870008604	5.596882677
ENSG00000215712	TMEM242	-0.255475968	0.822912318	-0.660815641	0.524163889	-0.916291609	9.064836028	7.790474954	6.43197674	7.913023977	7.85181819	7.594015257	7.36768071
ENSG00000108773	KAT2A	-0.562527749	0.611381475	-0.353843886	0.754015748	-0.916371634	6.96351253	8.607278467	9.788583153	8.244958867	8.36731141	8.560477404	8.465257491
ENSG00000135249	RINT1	-0.210061135	0.672900335	-0.707298361	0.103932144	-0.917359496	7.750913381	8.317412869	8.408111533	7.952038749	8.01099479	7.656405908	7.310303798
ENSG00000101017	CD40	-0.841773245	0.545297456	-0.075731858	0.961240785	-0.917505103	7.579279349	4.11352228	5.772716771	5.552894743	5.74985018	6.137764768	6.574984961
ENSG00000245680	ZNF585B	-0.171704304	0.931077597	-0.746305578	0.684505069	-0.918009882	4.00722531	6.899080544	9.746650996	8.244958867	8.15640714	7.727325329	7.539150216
ENSG00000123349	PFDN5	-0.739749089	0.203773754	-0.178534888	0.783874152	-0.918283977	11.31794963	10.05576005	10.94216274	10.13160579	10.1148377	10.71171785	10.65326667
ENSG00000107625	DDX50	-0.707737923	0.115125804	-0.212073884	0.668986346	-0.919811807	8.564112515	9.308339287	9.25752426	8.369067528	8.39557285	9.012519675	8.718388644
ENSG00000136319	TTC5	-0.939069025	0.623053677	0.019237992	0.992804891	-0.919831033	2	8.147241016	8.007629717	6.496695859	6.68951263	7.507470083	7.543088047
ENSG00000137364	TPMT	-0.084788095	0.923175097	-0.835473645	0.259652636	-0.92026174	8.493334308	6.78762695	7.934808822	7.781117077	7.83127384	6.902223127	7.226264598
ENSG00000163155	LYSMD1	-0.899142457	0.264205073	-0.026008944	0.977454517	-0.925151401	8.39321512	6.666836837	7.163363831	6.744602355	6.68951263	7.674704537	7.446882182
ENSG00000095794	CREM	0.078155987	0.965486841	-1.004719295	0.5332435	-0.926563307	7.624139768	8.747909548	3.369317972	7.786523606	7.8390123	6.840432491	6.671679221
ENSG00000127586	CHTF18	-1.245844645	0.422347566	0.31901841	0.850046117	-0.926826235	6.366709671	6.228547981	2	4.867343068	4.40247105	6.106889539	6.007361012
ENSG00000211460	TSN	-0.607665902	0.272669235	-0.319435694	0.579833528	-0.927101596	9.858207046	8.848535039	9.859153765	9.027396512	8.94344897	9.183011957	9.35623215
ENSG00000243137	PSG4	-0.689618725	0.809318998	-0.238668087	0.935195648	-0.928286812	2	2	5.156626401	3.462030052	3.28037825	2.775316226	4.215171317
ENSG00000100330	MTMR3	0.89786758	0.258164018	-1.826764319	0.012003587	-0.928896739	8.653385977	9.102512015	7.163363831	9.340234287	9.47403846	6.670403488	6.78907279
ENSG00000197170	PSMD12	-0.053115656	0.96053419	-0.876505282	0.352564766	-0.929620937	10.66423795	9.246456519	8.296287027	9.735680502	9.61140668	8.884319278	8.828963952
ENSG00000182916	TCEAL7	-0.533750205	0.821911287	-0.39608692	0.866925741	-0.929837125	8.836089686	5.303523747	2	6.956091174	6.76185814	6.619606851	7.293259708
ENSG00000113649	TCERG1	-0.560341252	0.338203518	-0.36957139	0.53558846	-0.929912641	8.540903468	9.74864196	9.452720984	8.795015659	8.76268478	8.962049262	8.974090715
ENSG00000166171	DPCD	-0.747925753	0.367406838	-0.182842706	0.840762669	-0.930768458	7.82963488	7.189546432	6.073378139	6.542151749	6.41363412	6.643273885	7.324100729
ENSG00000100593	ISM2	0.035142853	0.983064817	-0.965922648	0.503743146	-0.930779795	4.815609471	4.11352228	2	3.997744386	4.16043394	3.500668998	3.261853426
	FLJ44635	6.687811334	3.17E-09	-7.618732524	0.000102845	-0.93092119	4.00722531	3.076665209	2	9.191259691	9.11153864	2	2
ENSG00000140497	SCAMP2	-0.608625479	0.175108303	-0.322887944	0.490052989	-0.931513424	10.65885801	9.849218119	10.43545992	9.731481912	9.7573881	9.899648775	10.1477907
ENSG00000140479	PCSK6	-3.255253103	0.197230313	2.323465978	0.289491881	-0.931787124	2	2	4.058998049	2.141737246	2.14022021	4.875056259	4.161535617
ENSG00000167434	CA4	4.259065389	0.420476172	-5.191097152	0.337980836	-0.932031763	2	2	2	4.54891529	4.56059747	2	2
ENSG00000131238	PPT1	-0.901741279	0.370378063	-0.030767515	0.978413041	-0.932508794	10.65345793	10.65289571	8.143093325	9.310519243	9.2754408	10.11877641	10.19985319
ENSG00000089063	TMEM230	-0.56149375	0.036303182	-0.37228396	0.176769831	-0.93377771	10.16946352	10.40166101	10.49365885	9.798574229	9.80557618	9.916742729	10.06074001
ENSG00000111642	CHD4	0.938064047	0.156773734	-1.872429896	0.001983235	-0.934365849	9.635285994	11.03485733	9.981704053	11.23934953	11.3276219	8.350077895	8.613062226
ENSG00000243955	GSTA1	-3.753018341	0.531143362	2.81840284	NA	-0.934615501	2	2	2	2	2	3.256555576	3.67940853
ENSG00000145375	SPATA5	-0.755197651	0.301889468	-0.17955702	0.82326635	-0.934754671	6.009025997	7.051611315	7.646393253	6.387081877	6.25300433	6.892381078	6.853913776
ENSG00000148450	MSRB2	0.023547138	0.961717317	-0.959821522	0.014490584	-0.936274383	8.228665467	8.337339476	7.734844356	8.260583448	8.03813956	7.11465301	7.261744957
ENSG00000166451	CENPN	0.871378771	0.654593217	-1.807853543	0.314782677	-0.936474771	3.327711234	8.124485672	4.058998049	7.577865504	7.47849652	4.959178705	5.0581604
ENSG00000196262	PPIA	0.006893371	0.995663772	-0.943532934	0.317937692	-0.936639563	13.42879239	11.43000365	11.4724895	12.47653022	12.4088539	11.23248597	11.71466685
ENSG00000147804	SLC39A4	-1.079156221	0.554764081	0.140968958	0.944101725	-0.938187263	7.435667355	5.595927442	2	5.003571641	5.43283104	6.307840213	6.418016952
ENSG00000115806	GORASP2	-0.912975714	0.070246253	-0.0260772	0.96694554	-0.939052914	11.38790617	10.55418374	10.42220168	9.95860319	9.92914219	10.84536314	10.8107304
ENSG00000112304	ACOT13	-0.712216627	0.242017731	-0.230413521	0.726496359	-0.942630147	7.485143553	8.276714465	7.163363831	6.941624891	7.12888795	7.644728141	7.355789538
ENSG00000006194	ZNF263	-0.797105313	0.267233798	-0.146127865	0.856534247	-0.943233179	9.613202746	7.979858146	9.242516611	8.323303759	8.28094035	9.041010784	8.845533906
ENSG00000176101	SSNA1	-0.819374387	0.068942241	-0.125584727	0.810523076	-0.944959114	9.384607364	8.590771778	8.699419242	8.122047456	8.12299044	8.785862385	8.830575839
ENSG00000197885	NKIRAS1	-0.45643516	0.356981191	-0.488536759	0.30872573	-0.944971919	8.16936288	7.467835385	8.236946975	7.568480804	7.50792049	7.396955563	7.609689469
ENSG00000181827	RFX7	-0.597535707	0.43635695	-0.347679369	0.660564083	-0.945215076	8.928409675	8.317412869	9.935792933	8.657201865	8.58388295	9.053576888	8.656119846
ENSG00000120910	PPP3CC	0.162394592	0.720965573	-1.108131193	0.002976633	-0.945736601	9.128497779	9.640525262	9.839340503	9.718812329	9.73682479	8.376795097	8.551840963
ENSG00000135346	CGA	4.245265296	0.424867908	-5.191096489	0.340621206	-0.945831193	2	2	2	4.739373666	4.31639476	2	2
ENSG00000113761	ZNF346	-0.479184796	0.704032536	-0.466673393	0.707642617	-0.945858189	6.563806279	9.04308532	6.534109959	7.311513918	7.54938955	7.55607473	7.330190599
ENSG00000172954	LCLAT1	-1.231221128	0.000206873	0.284296702	0.452298819	-0.946924426	8.138771685	8.414402626	8.608698767	7.288834728	7.08617588	8.657126631	8.703804674
ENSG00000076382	SPAG5	-0.27188904	0.873236533	-0.675112666	0.667404344	-0.947001706	4.467311984	6.899080544	3.369317972	5.540148523	5.33407135	4.927401733	5.217771644
ENSG00000109919	MTCH2	-0.373815969	0.531143362	-0.573223648	0.306515269	-0.947039616	9.317904228	10.17682307	9.25752426	9.287824288	9.27353851	8.824761857	9.301491724
ENSG00000137338	PGBD1	-0.596942659	0.755058981	-0.352401867	0.856791105	-0.949344526	7.975256505	7.099070191	2	6.503277904	6.41363412	6.843841054	6.525421271
ENSG00000154957	ZNF18	-1.301488248	0.327520219	0.351220096	0.808868142	-0.950268152	3.327711234	6.951736442	7.029880454	5.058234798	5.43283104	6.91847879	6.678855578
ENSG00000079215	SLC1A3	0.251770411	0.875544825	-1.203224152	0.385615654	-0.951453741	4.00722531	7.232742069	7.342949392	7.311513918	6.66069199	6.239610476	4.604965121
ENSG00000182923	CEP63	-0.699336718	0.542375976	-0.254048322	0.835954015	-0.95338504	7.790810999	6.311476754	9.242516611	7.593372273	7.49817903	8.055236437	7.908246991
ENSG00000139567	ACVRL1	-0.603130636	0.709997456	-0.350465232	0.833427256	-0.953595869	8.228665467	7.73146271	3.369317972	6.788375664	6.93069905	6.890734189	7.293259708
ENSG00000154889	MPPE1	0.504189461	0.761525791	-1.458355537	0.330230765	-0.954166076	4.467311984	5.838937808	8.677268317	7.767511442	7.90193448	6.037140174	5.846719511
ENSG00000096872	IFT74	-0.070208941	0.906452235	-0.884714224	0.063233483	-0.954923165	7.750913381	7.145017454	7.552162999	7.546342806	7.29563107	6.414841377	6.830475749
ENSG00000125821	DTD1	-0.333105627	0.687168755	-0.621840679	0.419204582	-0.954946306	7.33131396	7.503546079	8.65477198	7.584088227	7.66419009	6.892381078	7.685278529
ENSG00000087245	MMP2	2.283428878	2.70E-09	-3.238428522	7.49E-18	-0.954999643	13.28952258	13.04041058	12.62979741	15.27638247	15.31259	10.02448838	9.483266623
ENSG00000112759	SLC29A1	-1.966626334	7.67E-05	1.011534546	0.054367856	-0.955091788	9.064836028	7.928237109	8.763901704	6.716553866	6.73998187	9.582192405	9.730352282
ENSG00000133997	MED6	-0.20250219	0.897514654	-0.754787767	0.593195109	-0.957289957	9.435834703	7.145017454	5.59507254	7.994705029	8.00412803	7.538302335	7.372114752
ENSG00000124228	DDX27	-0.205559941	0.868087762	-0.75192653	0.492905469	-0.957486471	6.366709671	9.308339287	8.978369997	8.456457569	8.47883193	8.008163797	7.844792088
ENSG00000169519	METTL15	-0.580748814	0.218893333	-0.377609872	0.431879804	-0.958358686	7.750913381	7.315436877	7.342949392	7.012547947	6.80989959	7.284811203	6.913939481
ENSG00000105808	RASA4	2.02716002	0.116189409	-2.985539079	0.015451866	-0.958379059	7.27616306	5.838937808	3.369317972	8.058664058	8.29037844	3.374372158	3.940519005
ENSG00000162971	TYW5	-0.193148398	0.885491359	-0.76531685	0.518870344	-0.958465248	5.095959458	7.189546432	8.296287027	7.173892946	7.1744553	6.848938847	6.350712758
ENSG00000072042	RDH11	-0.421287761	0.383589636	-0.537467239	0.245497099	-0.958755	11.27990552	10.62073637	10.99236731	10.57129953	10.5644133	10.11543113	10.72478611
ENSG00000237649	KIFC1	4.229413014	0.428189845	-5.191096228	0.341748605	-0.961683214	2	2	2	4.670864177	4.37434574	2	2
ENSG00000178234	GALNT11	-1.273314428	0.008246269	0.311412266	0.569500316	-0.961902162	9.219014269	8.191702463	8.825624954	7.497728447	7.58969985	9.030953258	9.18199253
ENSG00000006432	MAP3K9	-2.006749345	0.136243503	1.042989566	0.451766731	-0.963759778	3.327711234	4.93638933	6.43197674	3.840289928	3.65239003	5.532544554	6.885273196
ENSG00000177105	RHOG	-1.035739137	0.225157865	0.071270443	0.94333501	-0.964468694	10.48735708	8.607278467	8.99637432	8.527598812	8.62175922	9.438259243	9.877176832
ENSG00000109062	SLC9A3R1	-2.516670841	2.20E-06	1.552125614	0.004741368	-0.964545227	9.064836028	8.451443573	9.132850258	6.542151749	6.35006802	9.873621976	10.86770856
ENSG00000173598	NUDT4	1.04488855	0.408736505	-2.011825795	0.083380799	-0.966937245	5.866944937	9.433952963	9.399592459	9.96278806	9.90046182	7.052670333	6.769038269
ENSG00000138741	TRPC3	0.052615046	0.988129802	-1.02040173	NA	-0.967786683	2	2	3.369317972	2.784612485	2.38539787	2.094598369	2.509591799
ENSG00000081913	PHLPP1	-0.779068799	0.119536182	-0.189136776	0.733835313	-0.968205575	8.418903792	7.572420273	7.691296561	7.261917051	7.08617588	7.726402307	7.779530392
ENSG00000102098	SCML2	-0.680484809	0.79686468	-0.28805225	0.915553996	-0.968537058	2	6.140560021	2	4.299492648	4.05806467	4.580376151	4.411752372
ENSG00000118579	MED28	-0.783544033	0.410160907	-0.185189464	0.858897472	-0.968733496	9.839306291	8.317412869	7.646393253	8.033874364	8.2130862	8.747250685	8.687293668
ENSG00000060762	MPC1	-0.551078529	0.673480955	-0.417786053	0.74998849	-0.968864581	8.285626337	6.899080544	4.874587708	6.733448311	6.72335419	6.656902456	7.032593922
ENSG00000050628	PTGER3	-3.753018769	0.531143362	2.784113098	NA	-0.968905671	2	2	2	2	2	3.904814898	2.817676617
ENSG00000146247	PHIP	-0.155588464	0.82307937	-0.814032451	0.167996939	-0.969620914	9.635285994	9.181799937	8.536706852	8.993902695	9.06939887	8.68637457	7.99033569
ENSG00000104691	UBXN8	0.202230594	0.910912588	-1.172812323	0.454394312	-0.970581729	6.366709671	6.728495673	2	6.306580752	6.09824986	4.868377487	5.028505473
ENSG00000084774	CAD	-1.171714305	0.468098579	0.200641015	0.910973565	-0.97107329	4.467311984	9.192779271	9.981704053	7.847284664	7.98332947	9.375172844	9.166334389
ENSG00000108344	PSMD3	-0.386729933	0.415575369	-0.584645633	0.190011047	-0.971375566	11.12854954	10.27307349	10.7904855	10.39404824	10.3760296	10.08784705	10.28128023
ENSG00000124380	SNRNP27	0.088089724	0.876599527	-1.060003043	0.017748375	-0.971913318	8.609439662	7.790474954	8.511888463	8.469961514	8.37974396	7.38764416	7.20156326
ENSG00000146263	MMS22L	-0.863653788	0.548900135	-0.110075421	0.944363944	-0.973729209	5.330594134	7.35507212	4.058998049	5.391962469	5.44640346	6.556607032	5.481238982
ENSG00000181467	RAP2B	-0.520543922	0.177849888	-0.453607577	0.236728689	-0.9741515	9.128497779	8.671479029	9.049077186	8.469961514	8.41468635	8.670145638	8.326688609
ENSG00000113522	RAD50	1.313332074	6.08E-05	-2.287581648	6.20E-13	-0.974249574	7.940205782	8.169643011	8.296287027	9.439666599	9.44813055	6.063692919	5.785984581
ENSG00000173200	PARP15	-0.951404396	0.729390378	-0.023090082	0.993837021	-0.974494478	2	2	4.058998049	2.3892346	2.77779907	3.12825509	2.817676617
ENSG00000143256	PFDN2	-0.124203324	0.878583032	-0.850698724	0.209877866	-0.974902048	9.410448395	7.847167909	8.742726175	8.646835365	8.7017439	7.824551575	8.078894996
ENSG00000111011	RSRC2	-0.152667876	0.73835556	-0.822752251	0.032989268	-0.975420127	10.03472846	9.367676479	9.541233192	9.483600848	9.55926825	8.944693381	8.762402722
ENSG00000095321	CRAT	-0.992565275	0.015303223	0.016177903	0.974668275	-0.976387372	10.25038534	9.506147172	10.17589429	9.012478123	9.03500236	9.937829229	10.11090342
ENSG00000276219		4.213560972	0.431878529	-5.191095877	0.343240742	-0.977534905	2	2	2	4.623314311	4.40247105	2	2

ENSG00000138381	ASNSD1	-0.531450811	0.46101922	-0.448468429	0.530188487	-0.979919241	9.992581382	8.469613413	9.116480286	8.837307593	8.75724985	8.975319061	8.778080665
ENSG00000138867	GUCD1	-0.670311904	0.344773612	-0.309765391	0.679613476	-0.980077295	9.435834703	10.76012279	11.01019887	9.872886274	9.88621621	10.15112163	10.32138402
ENSG00000163017	ACTG2	-4.788328933	4.43E-06	3.808237203	0.000275121	-0.98009173	9.031919741	9.608085847	7.934808822	5.22679461	2.94009902	12.73669842	12.88226211
ENSG00000147677	EIF3H	-0.462542674	0.509773777	-0.518460546	0.445705675	-0.981003219	11.22635546	10.1878402	9.829330889	10.15373922	10.0027561	9.982775948	10.06508265
ENSG00000148399	DPH7	-0.501680551	0.628146304	-0.480194257	0.639193832	-0.981874808	8.228665467	8.376385542	5.930865648	7.450827758	7.27676203	7.460505069	7.310303798
ENSG00000065135	GNAI3	-0.33786649	0.665819919	-0.64579722	0.372471975	-0.98366371	9.975371636	10.00683004	11.37951548	10.32141448	10.2277056	9.968404253	9.968585649
ENSG00000141738	GRB7	-1.910181048	0.484988029	0.926275767	0.732364476	-0.983905282	4.00722531	2	2	2.498691189	2.14022021	3.355392153	3.640730416
ENSG00000147118	ZNF182	-0.349476832	0.863750456	-0.636204604	0.738292369	-0.985681436	2	6.728495673	7.858115942	6.580005553	6.40670796	6.539892359	5.816671626
ENSG00000139372	TDG	-0.405174861	0.42301813	-0.580744317	0.223260545	-0.985919178	8.418903792	9.328390744	8.884815469	8.483340231	8.57309694	8.440093652	8.265241863
ENSG00000124786	SLC35B3	-0.450749114	0.468547999	-0.536315886	0.368689059	-0.987065	8.674867122	8.806266215	9.735974718	8.797695539	8.60370266	8.777865933	8.440841157
ENSG00000106080	FKBP14	-0.982017415	0.000876231	-0.005425955	0.988654005	-0.987443371	9.667789716	10.02537381	10.02619908	9.003220305	8.87609528	9.806423533	10.01019245
ENSG00000136045	PWP1	-0.3446276	0.802652992	-0.643404871	0.618163882	-0.988032471	10.56418906	8.505280662	7.225742779	9.094319372	9.01919552	8.506119506	8.977489744
ENSG00000092140	G2E3	-0.499155588	0.70056887	-0.489111855	0.702388158	-0.988267443	8.777722034	6.844429755	5.772716771	7.226553841	7.1204461	7.502086936	6.775747403
ENSG00000189157	FAM47E	-0.692198405	NA	-0.297892091	NA	-0.990090496	2	3.076665209	2	2.141737246	2.59489879	2.746468522	2.172608095
ENSG00000060656	PTPRU	-1.398671931	0.438437558	0.40685429	0.83510727	-0.991817642	7.485143553	8.053983481	2	5.818703821	5.96314905	7.496683627	7.746896594
ENSG00000115944	COX7A2L	-0.727388631	0.288657299	-0.266017491	0.718208015	-0.993406122	10.93738399	9.470501606	9.954333071	9.488585154	9.56627807	10.06265365	9.90753096
ENSG00000189164	ZNF527	-0.700395514	0.298583304	-0.293225455	0.679226319	-0.993620969	5.70932728	6.535001748	6.80318718	5.578054239	5.92487824	6.287958221	5.980509018
ENSG00000106305	AIMP2	-0.592516099	0.121962743	-0.401257829	0.300265097	-0.993773928	7.790810999	7.638155693	8.110401118	7.29643414	7.24604864	7.407359556	7.507254539
ENSG00000181090	EHMT1	-0.681066656	0.047519069	-0.313772281	0.389671888	-0.994838937	9.624286623	9.114109157	9.301632639	8.624367384	8.7394436	9.123256136	8.966780003
ENSG00000117592	PRDX6	-0.239475942	0.798171474	-0.75592572	0.364514502	-0.995401662	12.19014827	11.0102427	10.31150679	11.13619556	11.1492725	10.26514949	10.91671583
ENSG00000134461	ANKRD16	-0.761361661	0.497204407	-0.234572277	0.845108741	-0.995933938	4.467311984	5.72254323	7.098165388	5.44762523	5.40529704	5.906324469	5.917188835
ENSG00000104946	TBC1D17	-0.651769769	0.11481363	-0.344612634	0.425827846	-0.996382403	9.204317094	9.277729677	9.788583153	8.663092307	8.92175022	8.967528086	9.22674878
ENSG00000169228	RAB24	0.505905412	0.639874207	-1.503423978	0.117817538	-0.997518566	7.27616306	9.256956408	7.029880454	8.71221318	8.75316018	6.850634117	6.671679221
ENSG00000117143	UAP1	-0.347035768	0.715844099	-0.652017568	0.464454793	-0.999053336	11.50945836	9.506147172	10.06936278	10.24689154	10.2962973	9.757337057	10.15166451
ENSG00000143889	HNRNPLL	-0.454001731	0.471675519	-0.545794425	0.366665941	-0.999796155	9.27634997	9.405920729	8.175061096	8.655725489	8.54498542	8.584107514	8.433081317
ENSG00000205795	CYS1	-1.146892964	0.031446236	0.146651379	0.812849666	-1.000241584	6.73718411	6.389896633	5.930865648	5.334065616	5.3193943	6.593521701	6.468583424
ENSG00000049192	ADAMTS6	-6.966291077	0.005644199	5.964844852	0.001443968	-1.001446225	2	4.93638933	2	2	2	9.311664913	9.109488808
ENSG00000124140	SLC12A5	-1.694415975	0.696746436	0.692075095	NA	-1.00237688	2	3.076665209	2	2	2.38539787	3.256555576	2.277179783
ENSG00000133026	MYH10	-1.0751562	0.017562521	0.071417679	0.899395052	-1.003738521	9.689059027	10.36330215	10.22252928	9.052296807	9.05063789	9.846904685	10.47046835
ENSG00000077063	CTTNBP2	0.671947796	0.722734802	-1.676061712	0.332277495	-1.004113916	2	6.228547981	4.058998049	5.614989456	5.64780787	3.981090143	3.33635788
ENSG00000178252	WDR6	-0.851252735	0.101553845	-0.153453706	0.79696914	-1.004706441	10.60951384	11.07398149	9.981704053	9.729378027	9.82128771	10.36712466	10.56854893
ENSG00000173581	CCDC106	-1.173624922	0.085637804	0.168611979	0.832583267	-1.005012943	8.042902253	6.951736442	8.486635645	6.777556219	6.83074839	7.91644678	8.289666801
ENSG00000158161	EYA3	-0.430936556	0.641177228	-0.575457645	0.517359347	-1.006394201	9.533135543	7.431218269	9.031722608	8.542055007	8.40081078	8.517903178	8.114615835
ENSG00000095209	TMEM38B	0.61478547	0.617393398	-1.623248815	0.141216873	-1.008463345	5.70932728	7.467835385	8.721235202	8.339946438	8.39031583	6.60762613	5.601925227
ENSG00000167900	TK1	-1.703695037	0.416993309	0.694324972	0.753823132	-1.009370065	4.467311984	8.004992147	2	5.161714397	4.811949	6.826717129	7.562617572
ENSG00000102882	MAPK3	-1.270453101	0.018021461	0.260401752	0.675928124	-1.010051349	10.40620204	9.624396729	10.77492406	9.061245954	9.09332116	10.36963935	10.80076011
ENSG00000115263	GCG	4.180601725	0.439229935	-5.191095048	0.346533496	-1.010493323	2	2	2	4.415698455	4.56059747	2	2
ENSG00000130177	CDC16	-0.359285126	0.386972978	-0.652036102	0.088880848	-1.011321228	10.16946352	9.994333773	9.491321578	9.468543769	9.63900396	9.240714099	9.289430628
ENSG00000066827	ZFAT	-0.917367075	0.600101312	-0.094170699	0.961240785	-1.011537774	8.87372952	5.72254323	4.058998049	6.548530093	6.64311914	7.564363052	7.199901369
ENSG00000150316	CWC15	-0.383108875	0.688362385	-0.628825453	0.483865109	-1.011934329	9.358295023	8.702538795	7.163363831	8.339946438	8.22887947	7.807195765	8.246044089
ENSG00000134077	THUMPD3	-0.69790279	0.493931013	-0.316128509	0.767357706	-1.014031299	9.886101849	7.761270542	7.934808822	8.207157886	8.15640714	8.512558892	8.604294318
ENSG00000197406	DIO3	-4.714921645	0.185413931	3.700594983	0.227412477	-1.014326662	2	2	3.369317972	2	2	5.427419529	4.047883918
ENSG00000186073	C15orf41	-0.771909234	0.703942894	-0.243109286	0.909865545	-1.015018521	2	7.189546432	4.523675391	5.289065324	5.0259382	5.755231204	5.519049808
ENSG00000112996	MRPS30	-0.772328214	0.454478461	-0.242935465	0.826586773	-1.015263679	9.048471759	7.979858146	6.534109959	7.416368504	7.44169935	8.25278818	7.561323797
ENSG00000134440	NARS	-0.312058155	0.456600383	-0.703601755	0.06253927	-1.015659911	11.18951828	10.69597594	10.56174313	10.5227232	10.53732	10.24788588	10.02270259
ENSG00000135617	PRADC1	-1.489308489	0.42315744	0.47306558	0.812849666	-1.016242908	2	7.954278496	6.322058544	5.514313187	5.27444544	6.877490957	7.55613706
ENSG00000196405	EVL	-0.955602265	0.099823785	-0.061350795	0.930293595	-1.01695306	8.653385977	8.213429689	7.451345288	7.222570549	7.28055563	7.917255914	8.312916523
ENSG00000076321	KLHL20	-0.653300232	0.535619507	-0.364521826	0.73782085	-1.017822059	7.096806373	8.590771778	9.778213818	8.211183901	8.22691474	8.360229299	8.635366595
ENSG00000006047	YBX2	-3.753019391	0.531143362	2.734339999	NA	-1.018679391	2	2	2	2	2	3.235948948	3.600986722
ENSG00000119772	DNMT3A	-1.285555143	0.000994715	0.266627837	0.553713666	-1.018927307	8.75772933	8.848535039	8.143093325	7.285019958	7.42465798	8.933539019	8.835400723
ENSG00000105677	TMEM147	-0.218083986	0.586581206	-0.801759187	0.020991008	-1.019843173	9.567995545	9.608085847	10.09465441	9.56056556	9.55535913	8.888863837	9.062251023
ENSG00000106415	GLCCI1	-0.145434447	0.935309765	-0.874493388	0.589352188	-1.019927835	3.327711234	8.297207175	6.718950348	6.975156635	7.08183429	6.609629839	5.980509018
ENSG00000111666	CHPT1	-0.335904197	0.725609862	-0.686877691	0.439455057	-1.022781887	7.218819781	9.067150531	9.399592459	8.451360823	8.5339035	8.25278818	8.032029379
ENSG00000114902	SPCS1	-0.155654521	0.689206562	-0.867327956	0.007526564	-1.022982477	10.3933013	9.863030423	9.972637969	9.902443169	9.97258765	9.24458753	9.215277965
ENSG00000137727	ARHGAP20	-1.379135718	0.496719668	0.355077963	0.872155485	-1.024057755	2	6.311476754	8.561105505	5.753943344	6.1068222	8.012713664	7.043754524
ENSG00000152620	NADK2	-0.33567604	0.596166767	-0.688667967	0.236074775	-1.024344007	7.53297911	8.639735838	8.434763955	8.004017475	7.89452713	7.80632247	7.36768071
ENSG00000151233	GXYLT1	0.04000535	0.984309875	-1.065317209	0.571694567	-1.02531186	8.444143041	7.928237109	2	7.813256448	7.52080763	6.788313392	6.38621943
ENSG00000138385	SSB	-0.298071409	0.716902068	-0.728020609	0.33116471	-1.026092018	10.5468183	9.298208073	8.978369997	9.479434065	9.47734778	8.848286818	9.22919499
ENSG00000265354	TIMM23	-0.363394063	0.451037592	-0.662721366	0.13467376	-1.026115428	9.358295023	10.06773758	10.0085654	9.446513412	9.52289845	8.97493051	9.373289845
ENSG00000066185	ZMYND12	-1.643982387	0.530264818	0.617123023	0.816030298	-1.026859363	4.00722531	2	2	2.498691189	2.2680111	3.355392153	3.261853426
	C9ORF172	4.164200733	0.443453067	-5.191094556	0.348435042	-1.026893823	2	2	2	4.358765249	4.58534546	2	2
ENSG00000117395	EBNA1BP2	-0.489329829	0.483350437	-0.537659561	0.42790044	-1.02698939	9.410448395	8.376385542	8.296287027	8.297027403	8.30907138	7.88204676	8.551840963
ENSG00000164109	MAD2L1	-0.484360616	0.869037712	-0.543011972	0.849422471	-1.027372588	2	7.35507212	2	5.461211713	5.34860058	5.4091291	5.294527297
ENSG00000013523	ANGEL1	-1.42340446	0.027864804	0.396018716	0.58348958	-1.027385745	7.031698319	8.317412869	8.585098385	6.70517998	6.75642009	8.547470747	8.473534575
ENSG00000197982	C1orf122	-0.741883924	0.003365499	-0.286509314	0.298467142	-1.028393238	9.720386367	9.549500232	9.788583153	8.875861021	9.01919552	9.409219381	9.393787153
ENSG00000131711	MAP1B	-1.300081414	1.21E-06	0.271456028	0.38415025	-1.028625386	14.04768507	14.1025305	13.70724472	12.6623295	12.6644892	14.13023131	14.33147456
ENSG00000253719	ATXN7L3B	-0.545349227	0.601702841	-0.483428727	0.641992641	-1.028777954	8.609439662	10.39692156	11.28380426	9.929575199	9.90722553	9.990100744	9.970048927
ENSG00000169139	UBE2V2	-0.641315379	0.122576344	-0.38799175	0.36482448	-1.029307129	10.04301211	9.770792087	9.31604076	9.212503151	8.98589128	9.422103197	9.28512653
ENSG00000166411	IDH3A	0.318612567	0.698007365	-1.347964944	0.056148162	-1.029352377	9.435834703	8.191702463	7.818184403	8.984524517	8.95655043	7.354572674	7.302581437
ENSG00000215114	UBXN2B	-1.2637439	0.274972023	0.23425562	0.856791105	-1.02948828	8.716893051	7.979858146	5.39244166	6.652857793	6.68379438	8.327733606	7.8990585
ENSG00000006744	ELAC2	-0.583073264	0.128141904	-0.446478064	0.24798411	-1.029551327	9.2903348	9.308339287	9.859153765	8.953617446	8.90341524	9.012898235	9.113908014
ENSG00000078902	TOLLIP	-0.78474762	0.246264529	-0.245429321	0.738292369	-1.030176461	10.64259676	9.170736405	10.50627919	9.476091592	9.43797543	9.993365625	9.994460574
ENSG00000145217	SLC26A1	-2.29935171	0.321145767	1.26867813	0.582452573	-1.03067358	2	2	4.874587708	2.600425781	2.38539787	4.636484163	4.468068808
ENSG00000137947	GTF2B	-0.045147061	0.942460221	-0.985546951	0.051573713	-1.030694011	9.27634997	8.395519306	8.585098385	8.7747563	8.73668456	8.045647998	7.570355956
ENSG00000160691	SHC1	-0.2916018	0.677691017	-0.740252767	0.242732554	-1.031854567	13.23633495	12.21407135	12.01984373	12.28032204	12.3252367	11.59118594	12.07747769
ENSG00000084090	STARD7	-0.562764547	0.080556335	-0.469765291	0.144905041	-1.032529838	11.0567315	10.82507101	10.66102425	10.26346497	10.3268876	10.22290636	10.53664546
ENSG00000197530	MIB2	-0.786602906	0.395924587	-0.246410108	0.805557931	-1.033013014	9.204317094	8.317412869	10.48730704	8.776115811	8.87484109	9.582192405	9.103279088
ENSG00000092010	PSME1	0.395657733	0.372520317	-1.428897717	0.000181365	-1.033239984	10.58135316	9.863030423	10.36791565	10.68422387	10.702313	8.712712904	9.025661876
ENSG00000182872	RBM10	-0.512027161	0.129332125	-0.522102101	0.113430713	-1.034129263	9.384607364	8.929529568	9.031722608	8.610716699	8.62474679	8.550081477	8.663373824
ENSG00000184634	MED12	-0.583817841	0.793896601	-0.450504846	0.840192213	-1.034322687	9.048471759	9.896993485	2	8.394075037	8.35836495	8.479522918	8.536120927
ENSG00000124217	MOCS3	-0.717670031	0.145361929	-0.318336489	0.538306766	-1.03600652	6.73718411	7.051611315	7.502634626	6.436065776	6.43421531	6.713894915	6.915965441
ENSG00000153391	INO80C	-1.187439341	0.254030721	0.149543086	0.900996869	-1.037896255	8.16936288	5.946638857	8.585098385	6.772115916	6.76727577	7.65057884	8.401617719
ENSG00000138231	DBR1	-0.432770584	0.664549192	-0.605328164	0.52471944	-1.038098748	7.940205782	5.838937808	7.934808822	6.922108067	7.22257674	7.013858277	6.804465095
	MFSD7	5.981813646	1.49E-05	-7.019994838	0.001016559	-1.038181192	3.327711234	3.076665209	2	7.807949361	7.91419618	2	2
ENSG00000148296	SURF6	-0.815192985	0.463646059	-0.223295935	0.85266974	-1.03848892	9.886101849	9.235879652	6.882775709	8.343618883	8.2790453	8.756774919	9.025661876
ENSG00000105438	KDELR1	-0.815192346	0.161771312	-0.223711006	0.728068232	-1.038903353	12.7234361	11.54799449	11.82708281	11.3298432	11.2885951	11.79620367	11.99698602
ENSG00000130313	PGLS	-0.549436069	0.429519861	-0.489800131	0.476243419	-1.0392362	10.16946352	9.318399851	8.677268317	8.998569022	8.94464494	9.077661821	8.982814948
ENSG00000134627	PIWIL4	0.013562299	0.994867193	-1.052862191	0.533469206	-1.039299892	6.89194353	5.303523747	2	5.742861563	5.7929843	4.940196634	4.673629022
STRN4		-0.799781446	0.240478094	-0.242290328	0.744693168	-1.042071773	10.04301211	9.936721131	11.27276688	9.725161029	9.78502553	10.23236508	10.38511052
ENSG00000122966	CIT	0.198267028	0.917583206	-1.240349219	0.460189911	-1.042082192	2	6.728495673	7.163363831	6.628980152	6.55189318	4.881704255	5.545463418
	FAM26F	4.14761108	0.447517543	-5.191094031	0.350450714	-1.043482951	2	2	2	4.299492648	4.60967607	2	2
ENSG00000215305	VPS16	-0.362172227	0.504589952	-0.6820602	0.170920391	-1.044232426	8.653385977	8.451443573	7.646393253	7.944803394	7.97164382	7.632955077	7.65527392
ENSG00000125691	RPL23	-1.232762946	0.076121912	0.188510057	0.815972748	-1.044252889	12.39494173	10.85302421	11.16147894	10.43162866	10.3715902	11.78472189	11.85544917
ENSG00000077044	DGKD	-1.561494534	0.006399185	0.516659845	0.4152616	-1.044834689	7.790810999	8.903033433	9.132850258	7.119236776	7.24216308	9.199402547	9.259028753
ENSG00000268916	CSAG3	2.596819353	0.600260828	-3.642853624	NA	-1.046034272	2	2	2	3.406930997	3.28037825	2	2.059860178
ENSG00000075413	MARK3	-0.598099596	0.428026621	-0.448678689	0.553628122	-1.046778286	9.397585735	9.923599762	11.01903267	9.692414568	9.6668081	9.886487495	9.767852046
ENSG00000148229	POLE3	-0.613991752	0.141601992	-0.433642759	0.305685651	-1.04763451	9.771132748	9.102512015	9.180874355	8.81895723	8.72280968	8.892982869	9.005824891
ENSG00000162068	NTN3	-3.753019755	0.531143362	2.705187516	NA	-1.047832239	2	2	2	2	2	3.193831101	3.600986722
ENSG00000091073	DTX2	0.285678018	0.895265695	-1.33476464	0.48296181	-1.049086622	9.384607364	5.72254323	3.369317972	8.139053376	8.27714776	6.595545082	6.662054875
ENSG00000115750	TAF1B	-0.165805705	0.931725033	-0.883506509	0.616621341	-1.049312214	7.485143553	7.35507212	2	6.722207358	6.67228943	5.957567336	6.070564958
ENSG00000196296	ATP2A1	-0.847983713	0.528630833	-0.202008767	0.888119558	-1.049992479	4.00722531	4.710564154	3.369317972	3.289987342	3.7864959	4.251304078	3.735548302
ENSG00000144649	FAM198A	-3.753019798	0.531143362	2.701694638	NA	-1.05132516	2	2	2	2	2	3.943456545	2.551896972
ENSG00000196123	KIAA0895L	-0.604667641	0.758269479	-0.447136853	0.821473491	-1.051804494	8.228665467	7.638155693	2	6.804454198	6.80463998	6.647180917	7.211494604
ENSG00000050130	JKAMP	-0.252360647	0.413845402	-0.803564711	0.002720068	-1.055925358	10.19198706	9.930175364	9.849281147	9.7700774	9.71456265	9.261571873	9.131450704
ENSG00000130254	SAFB2	0.014483507	0.988129802	-1.070905702	0.17572907	-1.056422195	9.667789716	7.669933291	9.149036566	9.026254405	9.08466797	8.089380147	7.866953664
ENSG00000134690	CDCA8	-0.998788202	0.701852817	-0.061954108	0.982373817	-1.06074231	2	5.946638857	2	4.17310023	3.28037825	4.331190017	4.683176719
ENSG00000113552	GNPDA1	-0.352412221	0.291168491	-0.71048918	0.019469114	-1.062901401	9.015175615	9.079034257	9.165043284	8.727753724	8.74768919	8.539084523	8.208518621

ENSG00000076604	TRAF4	-0.810166681	0.339253229	-0.255295834	0.781935426	-1.065462514	10.66423795	8.993716073	8.978369997	8.939126212	9.00665321	9.526277199	9.522687851
ENSG00000183807	FAM162B	-3.753019983	0.531143362	2.686904797	NA	-1.066115186	2	2	2	2	2	4.053534413	2.172608095
ENSG00000105290	APLP1	-1.692367215	0.000150601	0.625353647	0.198968723	-1.067013568	8.696033109	7.928237109	8.585098385	6.79375511	6.76185814	8.816978708	9.255833064
ENSG00000138796	HADH	-1.954646762	0.0205779	0.887014627	0.324714138	-1.067632135	8.540903468	7.954278496	6.203069917	6.096288482	5.83486613	8.667743558	8.793039018
ENSG00000143631	FLG	-5.678913119	0.051473882	4.609797636	0.048277535	-1.069115484	4.00722531	2	2	2	2	5.843010798	7.124815549
ENSG00000100344	PNPLA3	0.450219626	0.748958558	-1.520636012	0.223084039	-1.070416386	4.00722531	7.761270542	7.342949392	7.497728447	7.47518984	5.343222328	5.829625997
ENSG00000025796	SEC63	-0.215568052	0.736896378	-0.856732574	0.125144676	-1.072300626	9.613202746	10.83561703	10.48092713	10.23111715	10.130687	9.652393285	9.425420127
ENSG00000114650	SCAP	-0.723306477	0.472007774	-0.349084178	0.738195992	-1.072390656	11.07300288	9.916994052	8.511888463	9.417187348	9.50598504	9.855795196	9.813124536
ENSG00000254636	ARMS2	-3.753020081	0.531143362	2.679094865	NA	-1.073925216	2	2	2	2	2	2.592885947	3.908316285
ENSG00000139173	TMEM117	-1.0307138	0.249172977	-0.043443591	0.967312407	-1.074157392	7.031698319	6.228547981	8.296287027	6.328984825	6.52655952	7.462723726	7.302581437
C8ORF46	C8ORF46	4.115551581	0.454568815	-5.191093324	0.353041048	-1.075541743	2	2	2	4.443344104	4.43005853	2	2
ENSG00000080802	CNOT4	-0.291036276	0.561250332	-0.784555497	0.079746219	-1.075591772	8.444143041	9.203675681	9.014156722	8.616799622	8.65720817	8.332594213	7.939455664
ENSG00000058600	POLR3E	-0.704409354	0.691987391	-0.371700361	0.839520202	-1.076109715	3.327711234	8.687092496	9.180874355	7.745471964	7.63485389	8.082904238	7.950369405
ENSG00000130935	NOL11	-0.101461579	0.92127192	-0.975199093	0.260131361	-1.076660672	8.777722034	8.077869079	6.534109959	8.022463952	7.90193448	7.192914486	7.019463152
ENSG00000197903	HIST1H2BK	1.985308756	0.000911216	-3.061996054	1.08E-07	-1.076687298	8.418903792	8.234834549	9.503962296	10.78827371	10.8233057	5.610655473	6.070564958
ENSG00000123737	EXOSC9	0.135971339	0.823963146	-1.214230678	0.013699636	-1.078259339	7.904282243	7.538394143	8.561105505	8.190940526	8.18704757	6.875826972	6.862343163
ENSG00000150459	SAP18	-0.379302959	0.572669004	-0.699836155	0.262859223	-1.079139114	11.10500521	9.71856931	10.37481416	10.1390214	10.1206693	9.729967164	9.887021781
ENSG00000124191	TOX2	3.135425563	0.030198621	-4.214677163	0.004583447	-1.0792516	2	4.442710129	4.874587708	7.057964187	7.00136498	2.42098416	2.059860178
ENSG00000173418	NAA20	-0.71582275	0.077472236	-0.364804997	0.391016933	-1.080627747	9.87686338	9.277729677	9.399592459	8.756965077	8.89725143	9.00682933	9.329501596
ENSG00000101474	APMAP	-0.837960835	0.003812454	-0.24436069	0.453575633	-1.082321526	10.58135316	10.32888058	10.79563557	9.715981692	9.77093593	10.35791611	10.31179874
ENSG00000164663	USP49	-0.366608227	0.395924587	-0.716294607	0.068345365	-1.082902834	7.218819781	7.35507212	7.646393253	7.106324235	7.00595521	6.784770914	6.649933445
ENSG00000172878	METAP1D	-1.333634932	0.122761253	0.25034146	0.796537901	-1.083293472	6.009025997	4.710564154	6.43197674	4.825935136	4.4836996	5.893224226	6.261114822
ENSG00000116918	TSNAX	-0.23161751	0.59184685	-0.853405132	0.022521058	-1.085022642	8.946185348	8.791896851	8.845626171	8.630393142	8.63367258	7.718068425	8.266830206
ENSG00000100228	RAB36	-0.067777108	0.954792088	-1.017444033	0.326936342	-1.085221141	7.940205782	5.838937808	6.322058544	6.693715713	7.1204461	5.47655442	6.409414172
ENSG00000138398	PPIG	-0.835046209	0.299078862	-0.250252344	0.775592511	-1.085298553	10.61508061	8.981105197	9.149036566	8.948803189	8.95298909	9.680545441	9.365898224
ENSG00000144524	COPS7B	-0.899273878	0.197832048	-0.188602195	0.810298197	-1.087876073	8.75772933	8.557180776	9.981704053	8.302698479	8.39732094	9.021201511	9.087187414
ENSG00000177854	TMEM187	-1.511734095	0.372864851	0.42356458	0.818512537	-1.088169514	2	6.535001748	7.285536013	5.058234798	4.95174433	7.10332117	6.420873184
ENSG00000168434	COG7	-0.541646215	0.807156873	-0.546691561	0.801957964	-1.088337775	9.544849385	7.901717006	2	7.799951945	7.86451142	7.754744914	7.897008641
ENSG00000113384	GOLPH3	-0.829911318	0.016111406	-0.258601544	0.500300419	-1.088512862	10.86447339	10.48887288	11.10451741	10.04628835	9.976101	10.62868983	10.53186455
ENSG00000235711	ANKRD34C	-3.753020266	0.531143362	2.664244902	NA	-1.088775364	2	2	2	2	2	3.663836244	3.042026731
ENSG00000189042	ZNF567	-0.999306923	0.164064972	-0.090437257	0.914220926	-1.08974418	7.33131396	6.04685525	6.958202216	5.85002558	5.96314905	7.062943761	6.403650353
ENSG00000156735	BAG4	-0.755886478	0.572651771	-0.335536356	0.811551833	-1.091422834	5.532353208	9.055168103	9.31604076	7.880528731	7.95274764	8.404758425	8.25407423
ENSG00000136866	ZFP37	-0.192600727	0.918975348	-0.899117159	0.59841801	-1.091717887	2	6.666836837	6.203069917	5.949776857	5.47317115	5.47655442	4.56420962
ENSG00000186825	C2orf27B	4.09781558	0.459397746	-5.191092741	0.35529554	-1.093277161	2	2	2	4.299492648	4.53541753	2	2
ENSG00000090889	KIF4A	-0.92370137	0.493535102	-0.170060956	0.908291211	-1.093762326	4.00722531	5.457120953	3.369317972	4.105493497	3.60476711	4.199031499	4.683176719
ENSG00000119523	ALG2	-0.429174892	0.463676051	-0.664721244	0.225285675	-1.093896136	10.19941743	9.090820894	9.426401271	9.198375657	9.24178305	8.958909138	9.013414035
ENSG00000254685	FPGT	-0.032255367	0.986960349	-1.06169026	0.544751469	-1.093945627	2	7.35507212	7.225742779	6.727838783	6.66650236	6.022174708	5.349527629
ENSG00000126010	GRPR	-2.510834149	0.304711188	1.416197542	0.5723508	-1.094636607	2	6.844429755	2	3.615725078	3.15372745	6.959905775	6.38621943
ENSG00000175166	PSMD2	-0.122160074	0.815788738	-0.975768286	0.022364812	-1.097928359	12.29444214	11.72102722	11.60159843	11.76318136	11.8025467	10.68536117	11.14037952
ENSG00000135052	GOLM1	-0.191672842	0.776256344	-0.907158598	0.117096798	-1.09883144	10.19198706	11.24195824	11.28380426	10.7993477	10.7910896	9.771724757	10.33696731
ENSG00000123454	DBH	-3.75302041	0.531143362	2.652696705	NA	-1.100323705	2	2	2	2	2	3.059555873	3.640730416
ENSG00000163754	GYG1	-0.371503323	0.566726644	-0.729814426	0.223025633	-1.101317749	10.3868074	9.055168103	9.981704053	9.486925632	9.58250309	9.170850107	9.187873195
ENSG00000031698	SARS	-0.841891493	0.020700446	-0.260311713	0.521153279	-1.102203206	10.75779627	11.273328	11.42675132	10.35364261	10.3264289	10.89918683	10.94091995
ENSG00000064012	CASP8	0.361723117	0.782156761	-1.464349169	0.1981584	-1.102626051	5.70932728	6.535001748	8.631919287	7.751013478	7.93360068	6.049001768	6.113568999
ENSG00000109107	ALDOC	-1.631666292	0.014506629	0.529005985	0.474487232	-1.102660307	9.247966931	8.004992147	9.330306409	7.318995108	7.40393901	9.026085624	9.848823977
ENSG00000065717	TLE2	-2.202525568	0.104055955	1.098151797	0.435639036	-1.104373771	5.330594134	7.099070191	3.369317972	4.17310023	3.98554648	7.380620955	6.590289384
ENSG00000116747	TROVE2	-0.810171124	0.324359661	-0.294591559	0.737997178	-1.104762683	8.107517749	9.28800521	9.945092785	8.534041678	8.4504872	9.332742081	8.576385923
ENSG00000101189	MRGBP	-0.526649021	0.073702888	-0.578131923	0.042592362	-1.104780944	8.540903468	8.590771778	8.742726175	8.080836374	8.12931531	8.007404089	8.100433454
ENSG00000157110	RBPMS	-1.236102294	0.023538321	0.13095559	0.842587044	-1.105146704	11.14017917	9.981728319	10.26027233	9.244284335	9.38609912	10.70435357	10.65205172
ENSG00000276340		3.48264496	0.06001882	-4.58784196	0.012795456	-1.105197	2	6.04685525	3.369317972	8.049698831	8.06258142	2.526562374	2.059860178
ENSG00000152944	MED21	-0.671823575	0.386556156	-0.433764128	0.582952722	-1.105587703	9.27634997	7.503546079	8.65477198	8.036145657	7.92393105	8.290747499	8.13650041
ENSG00000092096	SLC22A17	-1.388560145	0.008760885	0.282655365	0.645132043	-1.105904779	8.910412251	9.34816733	9.963514551	8.109160159	8.08227582	10.00483003	9.446246726
ENSG00000068489	PRR11	-0.99505996	0.148587465	-0.111784064	0.889135814	-1.106844025	6.257048221	6.311476754	6.958202216	5.775854763	5.40529704	6.007052374	6.757786576
ENSG00000137825	ITPKA	1.211040879	0.619674661	-2.320849903	0.311632672	-1.109809025	2	4.442710129	2	4.034562184	4.40247105	2.592885947	2.172608095
ENSG00000154134	ROBO3	-1.112312767	0.594639301	0.002295035	0.999263508	-1.110017732	8.998234871	6.666836837	2	6.640968371	6.56439512	7.388811377	7.931466218
ENSG00000090054	SPTLC1	-0.271876088	0.64079571	-0.838929137	0.103603737	-1.110805225	10.47516499	9.849218119	9.426401271	9.720225568	9.70188757	8.979198827	9.298000491
ENSG00000164850	GPER1	-1.515881534	0.472437308	0.403493614	0.859659709	-1.11238792	2	6.140560021	8.904018013	6.01571348	6.11534392	7.34858644	8.3297313
ENSG00000126106	TMEM53	-0.881957126	0.633758183	-0.230431598	0.908314446	-1.112388724	2	7.572420273	6.203069917	5.742861563	5.5755357	6.104049656	6.420873184
ENSG00000186300	ZNF555	-0.987253966	0.409036401	-0.127031685	0.92548706	-1.114285651	8.313285031	5.946638857	5.930865648	6.18083559	6.30607931	7.385306888	6.666875073
ENSG00000172809	RPL38	0.147168197	0.911517345	-1.262702636	0.255721676	-1.115534439	11.08508709	8.732943944	8.408111533	10.00861625	10.150516	8.73123589	8.62735196
ENSG00000171723	GPHN	-0.718806105	0.76909522	-0.396997115	0.875239371	-1.11580322	3.327711234	7.928237109	2	5.870535235	5.63601114	5.985611216	6.127623461
ENSG00000144659	SLC25A38	-0.261422	0.691119414	-0.857927577	0.137924768	-1.119349577	8.418903792	8.255926468	9.385998894	8.43079185	8.59306436	7.877064822	7.972925696
ENSG00000174173	TRMT10C	-0.274382254	0.489001987	-0.845369057	0.014719532	-1.11975131	8.009475818	8.213429689	8.536706852	8.04293816	7.94798467	7.438128798	7.434215826
ENSG00000181915	ADO	-0.664592586	0.613081998	-0.456440194	0.731701381	-1.121032779	7.975256505	7.145017454	10.28245311	8.474993083	8.38855922	8.909749013	8.303661547
ENSG00000070413	DGCR2	-1.024565366	0.326833114	-0.096642018	0.935870665	-1.121207384	8.107517749	10.09139839	11.0057616	9.111653326	9.16073939	10.06594101	10.05293618
ENSG00000090487	SPG21	-0.379978563	0.790662721	-0.742082637	0.57806806	-1.1220612	11.36826231	8.623598428	8.143093325	9.702459798	9.76350037	9.381049994	9.365156975
ENSG00000164897	TMUB1	-1.011003586	0.013877267	-0.111369997	0.822143119	-1.122373583	9.448362284	9.04308532	8.631919287	8.109160159	8.04930085	8.930736901	9.00677572
ENSG00000101337	TM9SF4	-0.51424953	0.174135326	-0.608248361	0.095497186	-1.122497891	11.0567315	10.61258327	11.23155829	10.49945465	10.4499017	10.28346463	10.47270666
ENSG00000147434	CHRNA6	-3.753020699	0.531143362	2.629570255	NA	-1.123450444	2	2	2	2	2	2.831336763	3.753786374
ENSG00000112335	SNX3	-0.453307125	0.221900832	-0.672384849	0.054916044	-1.125691974	11.40085503	10.81088865	10.94216274	10.62924246	10.612236	10.43381138	10.37004136
ENSG00000066926	FECH	-0.628884337	0.15622962	-0.496992381	0.263184603	-1.125876718	8.855032352	8.820493869	8.325066185	8.03841338	8.09740995	7.934143724	8.421719243
ENSG00000198625	MDM4	-0.135381601	0.891546154	-0.990914893	0.234649837	-1.126296495	7.031698319	8.639735838	9.180874355	8.397612413	8.39732094	7.559188434	7.552234734
ENSG00000180053	NKX2-6	4.064225256	0.466248007	-5.191091905	0.358489009	-1.126866649	2	2	2	4.387512679	4.40247105	2	2
ENSG00000158863	FAM160B2	-1.037349073	0.115878835	-0.08951956	0.909689184	-1.126868633	8.946185348	9.532315021	10.45512158	8.752828065	8.74357228	9.722117015	9.65623967
ENSG00000132938	MTUS2	0.048126111	NA	-1.17731756	NA	-1.129191449	2	3.076665209	2	2.498691189	2.68924543	2.139668783	2.421067459
ENSG00000065328	MCM10	0.596241688	0.712287044	-1.72789612	0.23639043	-1.131654432	4.467311984	3.076665209	4.058998049	4.268917409	4.53541753	2.457042231	3.236138336
ENSG00000079974	RABL2B	-0.290694919	0.816824789	-0.841246714	0.456128443	-1.131941633	8.586954094	5.595927442	7.342949392	7.25803034	7.42122542	6.86244555	6.750993196
ENSG00000099256	PRTFDC1	0.014689275	0.995306348	-1.146930482	0.562234526	-1.132241207	8.653385977	6.535001748	2	7.363085426	7.42122542	6.060766632	6.457498602
ENSG00000221914	PPP2R2A	-0.466955826	0.419187861	-0.665678892	0.223069519	-1.132634718	10.44422623	9.7032946	9.399592459	9.44052423	9.46489849	9.431761894	9.053995509
ENSG00000250317	SMIM20	-0.861004115	0.162308072	-0.271714808	0.685920699	-1.132718924	8.586954094	7.467835385	7.600047325	7.226553841	7.02417148	7.5848779	7.814703611
ENSG00000177830	CHID1	-0.278496529	0.653630592	-0.855157639	0.120415896	-1.133654168	9.521325812	9.672251278	10.6093157	9.717397705	9.76214432	9.156187796	9.176932811
ENSG00000179348	GATA2	0.576961711	0.73580717	-1.71095258	0.265569714	-1.133990868	5.70932728	6.728495673	2	6.394182228	6.23747437	4.065262523	4.479073019
ENSG00000068438	FTSJ1	-0.497724693	0.550603127	-0.636382395	0.428827907	-1.134107088	10.16187685	8.968383114	8.380957451	8.830780654	8.91077722	8.561512889	8.887952157
ENSG00000121022	COPS5	-0.30013257	0.652929267	-0.834525836	0.162167432	-1.134658406	9.98400217	8.94259736	8.805342542	9.010169232	9.08140959	8.469076579	8.562873254
ENSG00000163806	SPDYA	-0.308445516	0.912408896	-0.826984552	0.74998849	-1.135430067	2	4.11352228	2	3.289987342	2.59489879	2.858551861	2.632958241
ENSG00000178802	MPI	-1.324798667	0.296385835	0.189180733	0.89578964	-1.135617935	9.90440318	9.159587375	6.073378139	7.781117077	7.71553728	8.897090174	9.525344379
ENSG00000101310	SEC23B	-0.44579734	0.570041492	-0.690645682	0.352432367	-1.136443022	10.5351205	9.170736405	9.099922433	9.325453269	9.31389502	8.886799903	9.244188987
ENSG00000172354	GNB2	-0.803057664	0.001780256	-0.33350613	0.233678267	-1.136563793	11.47313651	11.66496702	11.85201631	10.85926146	10.8800418	11.28460067	11.39025627
ENSG00000172215	CXCR6	-3.753020892	0.531143362	2.61409108	NA	-1.138929812	2	2	2	2	2	3.374372158	3.33635788
ENSG00000183018	SPNS2	-0.785316419	0.760985399	-0.354349952	0.893566028	-1.139666371	2	2	4.523675391	3.023133209	2.77779907	3.059555873	3.183290849
ENSG00000137103	TMEM8B	-2.187153245	0.006538839	1.046967506	0.222377009	-1.140185738	7.159102577	8.337339476	9.272377397	6.365568715	6.36443784	9.828312481	9.166759824
ENSG00000089248	ERP29	-0.726412325	0.184252877	-0.4152589	0.464454793	-1.141671225	10.22148166	10.04368224	11.08782089	9.829021922	9.77295717	10.06265365	10.15809785
ENSG00000187735	TCEA1	-0.325764207	0.609032994	-0.816283644	0.15331931	-1.142047851	9.810482605	9.04308532	9.301632639	9.209487455	8.97188212	8.98885284	8.092394167
ENSG00000172081	MOB3A	-1.055308018	0.025780112	-0.086853857	0.88223205	-1.142161875	9.895281535	9.591588447	10.54349105	9.009013399	9.01919552	9.872787989	10.07440932
ENSG00000072571	HMMR	0.418119138	0.753072123	-1.560339307	0.184116925	-1.142220169	3.327711234	4.11352228	4.523675391	4.523237872	4.2560182	2.858551861	3.236138336
ENSG00000162929	KIAA1841	-0.113369153	0.930344224	-1.030176514	0.354864291	-1.143545666	8.009475818	6.140560021	5.39244166	6.861921612	6.80463998	6.17883	5.703940561
ENSG00000111605	CPSF6	-0.710023136	0.036085138	-0.433878178	0.216308317	-1.143901314	9.174465797	9.71095217	9.553445883	8.799033614	8.790887	8.99920712	9.135368584
ENSG00000154721	JAM2	-2.779269962	0.000125058	1.63518117	0.030981543	-1.144088793	7.435667355	8.395519306	9.399592459	5.987823101	5.86549842	10.40597858	10.0730482
ENSG00000213462	ERV3-1	-0.615699963	0.758753417	-0.528469189	0.792987538	-1.144169153	2	6.899080544	8.266922072	6.483440992	6.65485813	6.384736493	6.883203642
ENSG00000253831	ETV3L	4.046834707	0.470055465	-5.191091418	0.360419662	-1.144256711	2	2	2	4.387512679	4.37434574	2	2
ENSG00000166471	TMEM41B	-0.107699193	0.749240001	-1.036899714	0.00010592	-1.144598907	9.112843761	9.267380431	9.439621147	9.204447245	9.13791797	8.316736994	8.182667731
ENSG00000236438	FAM157A	4.046438119	0.470185896	-5.191091389	0.360479076	-1.14465327	2	2	2	4.329433326	4.43005853	2	2
ENSG00000198283	OR5B21	4.046239732	0.470185896	-5.191091358	0.360546357	-1.144851626	2	2	2	4.299492648	4.45712836	2	2
ENSG00000187475	HIST1H1T	2.496167913	0.622552387	-3.641286977	NA	-1.145119063	2	2	2	3.227757282	3.33976443	2	2.059860178
ENSG00000100632	ERH	-0.155987479	0.815259329	-0.989849544	0.074729474	-1.145837023	9.657036304	8.607278467	9.788583153	9.224503288	9.33228764	8.413960373	8.490627472
ENSG00000277157	HIST1H4D	2.495013423	0.623809291	-3.641029376	NA	-1.146015953	2	2	2	3.09461492	3.45167086	2	2.059860178
ENSG00000196236	XPNPEP3	-0.654425037	0.27254373	-0.492208669	0.41217935	-1.146633705	7.867441304	7.572420273	8.608698767	7.426792977	7.44169935	7.807195765	7.340786405

ENSG00000159792	PSKH1	-1.024090349	0.088708728	-0.123403334	0.861696561	-1.147493683	10.60392551	9.225224669	10.06083273	9.012478123	9.08358266	9.942208568	9.945960865
ENSG00000158987	RAPGEF6	0.824149344	0.225654811	-1.972827325	0.001286666	-1.148677981	7.579279349	8.053983481	6.629488387	8.471640654	8.23084153	5.853194273	5.436777225
ENSG00000027847	B4GALT7	-0.809951852	0.35543975	-0.339107353	0.715680486	-1.149059205	9.371511179	9.549500232	7.451345288	8.302698479	8.19108423	8.701014095	8.725914443
ENSG00000111602	TIMELESS	-0.507191113	0.734475554	-0.642076828	0.659411517	-1.149267941	4.00722531	7.954278496	8.206335842	7.053486362	7.04662306	6.7543034	7.067643653
ENSG00000197448	GSTK1	-0.321106308	0.714968044	-0.828641851	0.297804511	-1.149748159	10.80726569	8.993716073	9.516493218	9.645581273	9.67619834	9.046937881	9.258230494
ENSG00000205744	DENND1C	-3.753021038	0.531143362	2.602399887	NA	-1.150621152	2	2	2	2	2	3.753554863	2.782576693
ENSG00000187699	C2orf88	0.992527916	0.688047343	-2.14343518	0.347127935	-1.150907263	2	6.04685525	2	5.651002631	5.40529704	3.256555576	3.012034821
ENSG00000100150	DEPDC5	-0.367554978	0.759077898	-0.783438795	0.480467544	-1.150993774	6.563806279	7.73146271	4.874587708	6.470063213	6.47451726	5.941748584	6.202567011
ENSG00000171865	RNASEH1	-0.826204556	0.610039424	-0.325089976	0.849222745	-1.151294533	9.710019321	7.393647515	4.874587708	7.68016596	7.54938955	8.041943102	8.172535245
ENSG00000109917	ZPR1	0.001821929	0.999189606	-1.15327064	0.597939463	-1.151448711	10.45668146	8.717821467	2	9.248208205	9.2611121	7.964208996	8.244432684
ENSG00000169249	ZRSR2	-0.412763623	0.589874122	-0.739491901	0.297771937	-1.152255525	6.816637977	7.002537973	8.175061096	7.035434774	7.07311172	6.895669227	6.562106074
ENSG00000156603	MED19	-0.652145459	0.671489931	-0.501849913	0.744573138	-1.153995372	8.564112515	8.433041974	4.058998049	7.388195945	7.23044321	7.430214384	7.485592137
ENSG00000115514	TXNDC9	0.076419501	0.966538801	-1.230486828	0.443133621	-1.154067328	9.556468883	7.874700259	4.058998049	8.535647908	8.38503958	7.168655895	7.189889619
ENSG00000171148	TADA3	-0.747342758	0.043381631	-0.406810223	0.293835258	-1.154152982	10.51739401	10.04368224	9.926432745	9.469384409	9.41227044	9.671462104	9.88081174
ENSG00000167749	KLK4	-3.753021086	0.531143362	2.598550145	NA	-1.154470941	2	2	2	2	2	4.029788235	2
ENSG00000047597	XK	-3.753021089	0.531143362	2.598372343	NA	-1.154648746	2	2	2	2	2	3.534812207	3.128433649
ENSG00000142168	SOD1	-0.16787414	0.842541719	-0.988260321	0.165667977	-1.156134462	11.62432333	10.27307349	10.17589429	10.67405041	10.6970002	9.672420943	10.03999009
ENSG00000180992	MRPL14	-0.714221574	0.298900033	-0.442153267	0.529629876	-1.156374841	9.613202746	9.367676479	8.143093325	8.458152491	8.46722793	8.537506665	8.903351429
ENSG00000170464	DNAJC18	-1.025453499	0.19054829	-0.131282963	0.885564891	-1.156736462	9.015175615	7.60566235	9.491321578	7.842101511	7.91907183	8.60585192	8.906411656
ENSG00000153531	ADPRHL1	-2.238699462	0.019769712	1.079610291	0.276208632	-1.159089171	5.532353208	4.442710129	6.43197674	3.754611435	3.82856379	6.176128386	7.04745562
ENSG00000082068	WDR70	-0.192375771	0.895675696	-0.967872766	0.450687102	-1.160248538	4.815609471	7.819099893	8.631919287	7.608714138	7.53358066	6.674237832	6.947999751
ENSG00000120693	SMAD9	-0.680608217	0.720923534	-0.479783777	0.80363944	-1.160391994	2	7.35507212	7.777116083	6.456560224	6.25300433	6.840432491	6.185873005
ENSG00000182473	EXOC7	0.299855149	0.804920943	-1.460851325	0.158887679	-1.160996175	8.928409675	10.00059544	11.760164	10.96972999	11.0210702	9.114110681	9.358840932
ENSG00000106006	HOXA6	4.029633139	0.473893575	-5.191090891	0.362261771	-1.161457752	2	2	2	4.443344104	4.2865223	2	2
ENSG00000161677	JOSD2	-0.492820998	0.463053095	-0.668822922	0.292072257	-1.161643919	8.138771685	7.315436877	8.65477198	7.688853661	7.59580267	7.309674888	7.615934347
ENSG00000130764	LRRC47	-1.09320368	0.019638737	-0.069009013	0.906104302	-1.162212693	9.886101849	8.993716073	9.859153765	8.522747711	8.56844956	9.640192857	9.479504351
ENSG00000154767	XPC	-0.068601836	0.928929217	-1.095030209	0.077869736	-1.163632045	8.737455672	8.862353853	9.972637969	9.209487455	9.25726695	8.377971121	8.035760639
	DOPEY1	5.787338782	0.000322116	-6.951186767	0.002485079	-1.163847985	2	3.686303414	2	7.59952861	7.60793141	2	2
ENSG00000196642	RABL6	-1.067807383	0.129332125	-0.096933607	0.908291211	-1.164740989	9.533135543	9.936721131	11.10036136	9.272493518	9.29994502	10.30912217	10.19381254
ENSG00000149743	TRPT1	-0.681622314	0.396175926	-0.483223535	0.55075414	-1.164845849	8.696033109	7.189546432	7.342949392	7.017154451	7.43490694	7.381793866	7.485592137
ENSG00000134248	LAMTOR5	-0.439608041	0.367412771	-0.725821236	0.110555942	-1.165429278	9.678463567	8.903033433	8.978369997	8.756965077	8.82635531	8.403604044	8.606804877
ENSG00000140682	TGFB1I1	-1.721461203	0.000159191	0.55499921	0.275628117	-1.166461993	10.77281601	9.785372135	10.64969256	8.723531969	8.77618319	10.99934685	11.03223581
ENSG00000111653	ING4	-0.269533606	0.810373984	-0.89821696	0.366670893	-1.167750566	9.48530526	8.968383114	6.80318718	8.517880242	8.5339035	8.018003769	7.783974227
ENSG00000237155		8.868271774	3.94E-07	-10.03617909	2.17E-05	-1.167907314	3.327711234	6.389896633	2	13.72712598	13.7531302	2	2
ENSG00000156042	CFAP70	0.705110877	0.648413066	-1.873537101	0.184133611	-1.168426224	4.00722531	4.11352228	2	4.268917409	4.12711147	2.68698273	2.671842496
ENSG00000168301	KCTD6	-1.131126886	0.162604083	-0.037654054	0.968812919	-1.16878094	7.709880939	6.389896633	6.203069917	5.808110275	5.89549381	7.156371901	6.567271433
ENSG00000130921	C12orf65	-1.642240652	0.172958175	0.473136163	0.721484546	-1.169104489	6.009025997	7.051611315	9.227351203	6.408278948	6.49425375	8.407640344	8.616180753
ENSG00000265808	SEC22B	0.098604492	0.887804971	-1.269110638	0.021623829	-1.170056146	10.905938	10.62073637	11.80936937	11.31682426	11.2878885	10.0293614	9.841114847
ENSG00000112796	ENPP5	-3.753021288	0.531143362	2.582403787	NA	-1.170617501	2	2	2	2	2	3.429858444	3.236138336
ENSG00000172113	NME6	-0.771283877	0.042332258	-0.401246888	0.312303334	-1.172530766	8.444143041	8.337339476	8.042706615	7.494428494	7.56191311	8.055971376	7.705330953
ENSG00000161558	TMEM143	-1.038307391	0.600231188	-0.137510473	0.948992109	-1.175817864	2	7.189546432	4.523675391	4.825935136	5.0259382	5.73334482	5.745545708
ENSG00000120727	PAIP2	0.162892612	0.818265844	-1.338872718	0.020011794	-1.175980105	10.40620204	9.308339287	9.272377397	9.921611889	9.92914219	8.310591351	8.548580051
ENSG00000139323	POC1B	-0.601141023	0.52931101	-0.57627557	0.538360258	-1.177416593	8.138771685	6.04685525	8.007629717	7.062428156	7.05992818	7.279786628	6.860240429
ENSG00000130724	CHMP2A	-0.892985259	0.003805818	-0.284446477	0.407829503	-1.177431736	9.646202137	9.835272298	9.344432377	8.74173768	8.73806474	9.286209561	9.399228411
ENSG00000157502	MUM1L1	2.874762525	0.340693523	-4.052563301	0.164968351	-1.177800775	2	2	5.39244166	6.586219071	6.56439512	2.457042231	2
ENSG00000180902	D2HGDH	-0.842005764	0.718118984	-0.338593179	0.890313426	-1.180598943	8.998234871	5.131603625	2	6.772115916	6.6253296	7.071691703	7.294817518
ENSG00000170448	NFXL1	-0.469905016	0.825160194	-0.710841106	0.725592339	-1.180746122	2	7.954278496	5.930865648	6.29144905	6.23747437	6.471027105	5.414021719
ENSG00000143436	MRPL9	-0.24747599	0.525575922	-0.933784237	0.00498916	-1.181260227	8.586954094	8.101365653	8.266922072	8.078634414	8.08661608	7.402744819	7.415721389
ENSG00000166501	PRKCB	-1.54516659	0.585723622	0.362551568	0.90481909	-1.182615021	2	4.710564154	2	2.600425781	2.86123014	4.478802948	2.509591799
ENSG00000182901	RGS7	-4.045406193	0.078250731	2.860868663	0.170814432	-1.18453753	2	2	5.156626401	2.270786764	2.14022021	5.582367412	6.755525668
ENSG00000149100	EIF3M	-0.150033946	0.878396152	-1.035201712	0.205129503	-1.185235658	11.02362764	9.443177293	9.227351203	10.02357442	9.94834802	9.046937881	9.162926384
ENSG00000143761	ARF1	-0.787357312	0.219396158	-0.398410952	0.553274344	-1.185768264	13.70485113	12.52221914	12.496991	12.21628798	12.2605626	12.55201409	12.69877307
ENSG00000114391	RPL24	-1.028670926	0.274935376	-0.157167075	0.884519325	-1.185838001	11.93591186	9.7032946	10.27509734	9.893702135	9.97960581	10.78801637	10.82400773
ENSG00000150787	PTS	-0.223914523	0.713961855	-0.962593332	0.066744424	-1.186507855	7.384433967	8.053983481	8.536706852	7.839502934	7.84926609	7.149501993	7.094725293
ENSG00000151665	PIGF	0.098292346	0.946981783	-1.285788624	0.318708894	-1.187496279	8.16936288	5.838937808	4.874587708	7.123515401	6.98285659	5.843010798	5.626876946
ENSG00000158156	XKR8	-0.196746492	0.911302045	-0.991066311	0.52301169	-1.187812803	8.19931886	9.192779271	4.058998049	8.060896687	8.00183183	7.393470826	7.09829807
ENSG00000119878	CRIPT	-0.153951358	0.865062033	-1.033998291	0.171003885	-1.18794965	8.493334308	7.315436877	6.80318718	7.61176303	7.51437845	6.840432491	6.564691065
ENSG00000106799	TGFBR1	-1.012134609	0.236430115	-0.176837368	0.855445922	-1.188971977	8.75772933	10.59199661	10.89974571	9.40053583	9.26015177	10.10818585	10.21659261
ENSG00000168685	IL7R	-4.714927092	0.174626306	3.525365365	0.236344937	-1.189561727	2	2	3.369317972	2	2	4.771475226	4.720747915
ENSG00000178445	GLDC	-1.244783879	0.571216016	0.054998358	0.981401003	-1.18978552	4.00722531	2	2	2.868578001	2.49394783	2.775316226	3.735548302
ENSG00000152147	GEMIN6	-1.264558124	0.433185867	0.074449815	0.967718357	-1.190108309	8.228665467	4.710564154	4.523675391	5.731694	5.58783529	7.060015953	6.786860422
ENSG00000149084	HSD17B12	-0.060175527	0.908645221	-1.130435371	0.004943283	-1.190610898	10.04301211	9.433952963	10.13584611	9.85562885	9.82323967	8.603340274	8.93066256
ENSG00000197943	PLCG2	-1.007344125	0.725609862	-0.183544572	0.951929397	-1.190888697	2	5.131603625	2	3.615725078	2.68924543	4.350488971	2.70970617
ENSG00000198478	SH3BGRL2	-2.543519618	0.12806343	1.351654715	0.43784645	-1.191864904	7.904282243	7.73146271	9.798878489	4.71689672	5.0259382	8.621327277	8.536120927
ENSG00000100413	POLR3H	-0.717518303	0.293044241	-0.476806375	0.492177662	-1.194324678	9.435834703	8.522788974	7.896971941	8.637890117	8.62175922	8.429356794	9.204544205
ENSG00000185127	C6orf120	-0.161189784	0.7690856	-1.033164346	0.024076465	-1.194354131	9.097018025	8.607278467	9.491321578	8.986874783	8.90095288	8.237320313	7.908246991
ENSG00000099804	CDC34	-1.098214769	0.003251348	-0.096852425	0.834220385	-1.195067193	9.613202746	9.799806309	10.07784268	8.667494409	8.83024294	9.968209057	9.482583303
ENSG00000158234	FAIM	-1.188685845	0.111035703	-0.00691277	0.993931149	-1.195598615	5.532353208	5.946638857	6.882775709	5.22679461	5.0259382	5.982522014	6.418016952
ENSG00000124406	ATP8A1	-0.326281744	0.908151255	-0.870210468	0.737997178	-1.196492213	2	5.838937808	2	3.881293667	4.4836996	4.199031499	3.261853426
ENSG00000164684	ZNF704	-4.681164486	0.120133693	3.48449402	0.244635807	-1.196670467	2	7.189546432	2	2.947924294	2	8.864319213	9.249822
ENSG00000155329	ZCCHC10	-1.125852032	0.392041264	-0.071672076	0.962347915	-1.197524108	8.631580145	7.232742069	4.874587708	6.34372989	6.63128385	7.754744914	7.227896413
ENSG00000124279	FASTKD3	-0.412984984	0.736587839	-0.785155802	0.493181118	-1.198140786	7.435667355	5.595927442	4.874587708	5.94010643	6.03677338	5.798032389	5.470251342
ENSG00000261986		3.992752324	0.480588858	-5.191090144	0.364514502	-1.19833782	2	2	2	4.299492648	4.37434574	2	2
ENSG00000123131	PRDX4	-0.167805768	0.812887832	-1.03078147	0.081818603	-1.198587237	11.98187803	11.00713613	10.69999858	11.16683559	11.1737025	10.29019523	10.3282462
ENSG00000258890	CEP95	-0.230072844	0.72758145	-0.970619971	0.089151012	-1.200692815	8.009475818	7.145017454	7.896971941	7.617841552	7.36516374	7.05561307	6.432241896
ENSG00000081307	UBA5	-0.075360792	0.948180321	-1.125461762	0.26264969	-1.200822554	9.423197387	8.903033433	6.629488387	8.71221318	8.57618689	7.698433415	7.517964974
ENSG00000131467	PSME3	-0.644123202	0.216076248	-0.557707131	0.282374528	-1.201830332	10.59831544	10.70750522	11.46604358	10.35864221	10.3102824	10.18813102	10.62118535
ENSG00000196683	TOMM7	-0.458227466	0.624036401	-0.744900827	0.397192742	-1.203128294	10.85509492	8.806266215	9.426401271	9.486925632	9.51808625	9.259659123	9.174396282
ENSG00000110344	UBE4A	0.003342091	0.997041173	-1.206904309	0.069457906	-1.203562217	8.716893051	10.3138733	10.0085654	9.856914282	9.80557618	8.508269163	8.741420621
ENSG00000184436	THAP7	-1.127729573	0.177620155	-0.080646188	0.935063964	-1.208375761	6.653098118	7.790474954	8.721235202	6.820355514	6.88158913	8.009682014	7.720479276
ENSG00000145725	PPIP5K2	-0.435021466	0.724360006	-0.774457851	0.503743146	-1.209479317	6.468621203	9.71856931	9.099922433	8.606137616	8.42329129	8.263008721	8.096865967
ENSG00000108840	HDAC5	-1.516003589	0.001826988	0.305239027	0.590415354	-1.210764562	10.73752355	9.632483534	10.21486071	8.736160353	8.77349359	10.49619812	10.63370358
ENSG00000128928	IVD	-0.91586399	0.357081274	-0.295565704	0.784238084	-1.211429695	8.418903792	9.405920729	10.88048583	9.055659246	8.95891978	9.434872973	9.792045353
ENSG00000227471	AKR1B15	0.931753887	0.646472565	-2.143240536	0.258155626	-1.211486649	2	3.686303414	3.369317972	3.79808662	3.82856379	2.183373728	2.551896972
ENSG00000085760	MTIF2	-0.340799619	0.778610361	-0.872892019	0.429681573	-1.213691638	6.563806279	7.638155693	9.372276026	8.029321023	7.92393105	7.49992804	7.409982764
ENSG00000105697	HAMP	-3.753021845	0.531143362	2.537792347	NA	-1.215229498	2	2	2	2	2	3.838019203	2.466008509
ENSG00000163746	PLSCR2	3.522153188	0.132111686	-4.737651392	0.093843117	-1.215498205	2	2	3.369317972	4.76150579	4.72553021	2.048074405	2
ENSG00000167642	SPINT2	-2.692463962	0.033287513	1.476863431	0.264448359	-1.215600532	8.87372952	6.951736442	4.874587708	5.319220934	4.95174433	9.024959988	9.272136653
ENSG00000198910	L1CAM	-2.303493203	0.355101172	1.087122409	0.676785453	-1.216370793	2	6.728495673	2	3.754611435	3.08597292	5.916072173	6.559516442
ENSG00000180822	PSMG4	-0.849991972	0.469998704	-0.366789801	0.766202864	-1.216781773	6.89194353	4.93638933	7.97167868	6.229283386	6.23747437	6.66270409	6.725806822
ENSG00000185947	ZNF267	0.228934351	0.873367801	-1.446044454	0.232591517	-1.217110103	6.468621203	5.946638857	8.941672936	8.076429088	7.83385794	6.322573937	6.347714043
ENSG00000138032	PPM1B	-0.213538106	0.805756178	-1.004572637	0.176769831	-1.218110743	7.218819781	7.73146271	8.904018013	8.010962573	7.81305436	7.120285726	7.162846889
ENSG00000120896	SORBS3	-1.304273883	0.000812533	0.085866456	0.862065438	-1.218407427	11.01943573	10.38739569	11.0408834	9.486095154	9.60082524	10.7588599	11.0829319
ENSG00000179912	R3HDM2	-0.287119244	0.313894643	-0.931749324	0.000162448	-1.218868568	9.262228249	9.318399851	9.516493218	9.05677832	9.10834046	8.41853943	8.469401969
ENSG00000151332	MBIP	-0.275493029	0.761078417	-0.943405153	0.240015951	-1.218898182	8.418903792	7.847167909	6.43197674	7.517371202	7.50468064	7.0047514	6.70252219
ENSG00000150907	FOXO1	-1.11039219	0.496139185	-0.110031993	0.951839566	-1.220424184	8.075571721	8.732943944	3.369317972	6.761173376	6.83591385	7.942116583	7.581886257
ENSG00000115207	GTF3C2	-0.747664724	0.584419874	-0.473468838	0.733879184	-1.221133562	6.009025997	9.599860728	9.990713521	8.418656444	8.63959257	8.75451294	8.853483814
ENSG00000070081	NUCB2	-0.083879657	0.895420772	-1.137713491	0.024456784	-1.221593147	11.15935596	10.06176125	10.7904855	10.67441498	10.632786	9.73685847	9.457070207
ENSG00000172301	COPRS	-0.984533662	0.10165634	-0.237692245	0.724423786	-1.222225907	8.716893051	9.415325457	9.69246155	8.31397419	8.39382264	8.590716036	9.466776947
ENSG00000221838	AP4M1	-0.238673657	0.907686866	-0.984903803	0.591055458	-1.22357746	8.075571721	7.60566235	2	7.088925745	7.02417148	6.394066622	6.283256815
ENSG00000124006	OBSL1	-1.744648452	1.68E-06	0.520617436	0.202653423	-1.224031016	10.65345793	10.00683004	10.41552659	8.655725489	8.63663561	10.70294658	11.07343347
ENSG00000025423	HSD17B6	-2.271957134	0.312908582	1.047460096	0.64966917	-1.224497038	2	5.131603625	2	2.695456494	2.59489879	5.04460184	4.329022995
ENSG00000144724	PTPRG	-0.733891142	0.356968286	-0.491677397	0.54430284	-1.225568539	8.16936288	9.869887299	9.714382178	8.720710586	8.67749148	8.604847786	9.210746501
ENSG00000105701	FKBP8	-0.880137726	0.125555633	-0.345709765	0.575949683	-1.22584749	11.75612888	11.45985033	12.62546556	11.1619039	11.1500503	11.55082553	11.81625909
ENSG00000071553	ATP6AP1	-0.466164649	0.279628699	-0.759984737	0.058901421	-1.226149386	12.09176592	11.53295289	11.46280979	11.27800726	11.238766	10.7784742	11.13059894
ENSG00000173535	TNFRSF10C	-2.635772979	0.150647216	1.409381717	0.456223157	-1.226391261	6.96351253	4.442710129	2	3.754611435	3.2184422	7.528804029	6.081436739
ENSG00000175264	CHST1	-1.465913847	0.150126699	0.234915292	0.835954015	-1.230998555	4.815609471	4.710564154	4.874587708	3.515101962	3.7864959	5.404520029	4.434543419

ENSG00000167112	TRUB2	-0.926787122	0.124764984	-0.304438165	0.64383682	-1.231225288	8.777722034	8.848535039	7.600047325	7.668500375	7.53358066	8.138779954	8.286538335
ENSG00000169062	UPF3A	-0.358895314	0.278511335	-0.875134355	0.002981664	-1.234029669	8.716893051	8.791896851	8.434763955	8.345451606	8.2657098	7.790512074	7.804897508
ENSG00000105325	FZR1	-0.759358114	0.289112399	-0.47489108	0.517968728	-1.234249195	9.233563228	8.834582582	10.33998689	8.814994458	8.90218458	9.029083034	9.245800665
ENSG00000189052	CGB5	3.955962672	0.484983469	-5.191090298	0.364514502	-1.235127626	2	2	2	4.299492648	4.31639476	2	2
ENSG00000129103	SUMF2	-0.268284522	0.430332959	-0.967577995	0.000907642	-1.235862517	11.41687936	10.95004268	11.18128933	10.87615882	10.9746976	10.30356295	10.1499441
ENSG00000096092	TMEM14A	-0.596384742	0.30261211	-0.640430354	0.252589462	-1.236815097	7.435667355	7.393647515	6.718950348	6.670510205	6.6253296	6.297933466	6.860240429
ENSG00000100567	PSMA3	1.073845132	0.008831138	-2.312505628	1.51E-09	-1.238660496	8.367060731	9.114109157	8.608698767	9.777686595	9.8316678	6.375345632	6.574984961
ENSG00000065548	ZC3H15	-0.251925311	0.619188542	-0.987075196	0.024026933	-1.239000506	9.975371636	9.090820894	9.714382178	9.442237963	9.32495871	8.681620276	8.628587883
ENSG00000143569	UBAP2L	-0.610243518	0.054358412	-0.630170278	0.043410967	-1.240413796	11.17074085	11.26554928	11.65357938	10.73124123	10.805643	10.72402432	10.77364464
ENSG00000062598	ELMO2	-0.117790785	0.902927943	-1.122848425	0.156984236	-1.24063921	9.27634997	9.318399851	10.95142175	9.954406146	9.96022311	8.996529805	8.918588063
ENSG00000161057	PSMC2	-0.163562982	0.876384005	-1.07813224	0.220472648	-1.241695222	11.09707093	9.988044813	8.784770914	10.09339983	10.0755276	9.10597186	9.240151898
ENSG00000183513	COA5	-0.556485408	0.476505377	-0.685699244	0.359691834	-1.242184653	8.367060731	7.819099893	6.629488387	7.218576229	7.23826702	7.231159579	6.96179415
ENSG00000170584	NUDCD2	-0.861353401	0.247792329	-0.381528184	0.628768646	-1.242881585	8.340423424	6.666836837	7.97167868	7.057964187	6.8715633	7.446000032	7.422862719
ENSG00000171530	TBCA	0.714065427	0.119484573	-1.957436161	1.67E-06	-1.243370735	9.128497779	8.276714465	8.922968322	9.557406408	9.48558803	6.814608286	6.965711302
ENSG00000198258	UBL5	-0.511104089	0.50195138	-0.732565587	0.308715893	-1.243669676	10.1076228	8.505280662	9.06622548	8.883449599	8.86223882	8.563064723	8.738561653
ENSG00000170619	COMMD5	-0.501499429	0.517483563	-0.742427492	0.308005991	-1.243926922	8.87372952	7.315436877	8.865353883	8.056427969	7.96222667	7.742013931	7.803803816
ENSG00000122705	CLTA	-0.411708981	0.534043967	-0.834256808	0.170526163	-1.245965789	11.43901855	10.17128279	10.48092713	10.37578145	10.4062943	9.8349648	10.09355512
ENSG00000142534	RPS11	-1.050142282	0.001272822	-0.196791982	0.608318434	-1.246934263	12.5084683	12.0319538	12.60800691	11.32032301	11.3871132	12.17607424	12.23624376
ENSG00000182871	COL18A1	-2.163111779	4.34E-11	0.914191499	0.009411948	-1.24892028	11.57946871	11.28620006	11.86677216	9.431062134	9.44728702	12.33642988	12.66340894
ENSG00000139410	SDSL	-0.417631209	0.694698997	-0.8326945	0.396311091	-1.250325709	4.467311984	6.535001748	6.882775709	5.920568914	5.85535975	5.390603804	5.611957759
ENSG00000184840	TMED9	-0.812155317	0.45032434	-0.438352168	0.693056959	-1.250507485	12.79016004	9.883504052	12.06958695	11.14910699	11.2355938	11.55082553	11.58148201
ENSG00000149136	SSRP1	-0.758585444	0.227473008	-0.492313634	0.444980407	-1.250899078	10.59268348	10.52407288	9.301632639	9.520570052	9.46656462	9.634053733	9.873532744
ENSG00000196562	SULF2	-2.107320846	8.35E-06	0.855476969	0.094172652	-1.251843877	8.564112515	8.777382925	8.904018013	6.652857793	6.72891807	9.067857443	9.995179393
ENSG00000012660	ELOVL5	-0.818189104	0.00026044	-0.43432858	0.06810855	-1.252517684	10.787681	10.79656548	10.85604368	10.07142123	9.92005068	10.28628591	10.46857168
ENSG00000236443		6.92426161	1.47E-05	-8.177102486	0.000313985	-1.252840876	2	4.710564154	2	9.914221922	9.97024066	2	2
ENSG00000176293	ZNF135	-2.177791623	0.234266721	0.922612291	0.63300769	-1.255179332	2	6.728495673	4.058998049	3.406930997	3.86943969	6.428526856	6.120613345
ENSG00000136840	ST6GALNAC4	-1.072634979	0.007453569	-0.183628146	0.695554779	-1.256263125	9.189468652	8.732943944	8.721235202	7.893113669	7.77859536	8.463547972	8.92815536
ENSG00000198301	SDAD1	0.04320208	0.964905581	-1.300226088	0.108113626	-1.257024008	9.435834703	9.079034257	7.342949392	8.887228971	8.92538942	7.726402307	7.420010428
ENSG00000103152	MPG	-0.134148305	0.861594641	-1.123753562	0.072934705	-1.257901867	10.41260941	9.203675681	9.165043284	9.511620236	9.65150894	8.597799214	8.604294318
ENSG00000177570	SAMD12	-6.314953619	0.018254181	5.055363165	0.014763919	-1.259590454	2	2	4.523675391	2	2	7.550870243	7.783974227
ENSG00000115350	POLE4	-0.631675074	0.66635587	-0.628356846	0.664743846	-1.26003192	8.19931886	7.874700259	4.058998049	6.89733479	6.89154576	6.605619634	7.140495192
ENSG00000125970	RALY	-1.140700254	0.001373482	-0.121985554	0.781250208	-1.262685808	11.0444064	10.59199661	10.88048583	9.707456306	9.71806384	10.45472238	10.95773505
ENSG00000132635	PCED1A	-0.995816471	0.645580573	-0.267691322	0.908686871	-1.263507793	9.957954117	8.376385542	2	7.753776273	7.86451142	8.554248841	8.501461594
ENSG00000052802	MSMO1	-0.589940449	0.40395265	-0.674786311	0.323536602	-1.26472676	10.59268348	11.09168706	11.95227171	10.73718662	10.7317082	10.25846039	10.95736623
ENSG00000125944	HNRNPR	-0.679071953	0.426810018	-0.586089117	0.489050844	-1.265161069	11.18578229	10.72273565	9.301632639	9.922226014	9.92126619	9.564740792	10.35667886
ENSG00000125703	ATG4C	-0.808719727	0.531143362	-0.456761313	0.730288533	-1.26548104	7.096806373	4.11352228	7.225742779	5.94010643	5.8245091	6.514451546	5.8339184
ENSG00000134265	NAPG	-0.902204743	0.2695484	-0.363557123	0.677816167	-1.265761867	9.829762239	7.847167909	8.941672936	8.23312837	8.14812525	8.784976076	8.659751394
ENSG00000008853	RHOBTB2	-1.643593783	0.000799417	0.377695109	0.501916413	-1.265898674	8.631580145	8.862353853	9.658943166	7.504305792	7.48837134	9.581682124	9.399228411
ENSG00000172775	FAM192A	-0.52491216	0.490581765	-0.741048207	0.303875747	-1.265960367	8.87372952	9.377333006	10.44859742	9.213506984	9.18117744	8.79381476	9.150074181
ENSG00000169976	SF3B5	-1.249628785	0.107655331	-0.016603094	0.985856566	-1.266231878	10.71177538	9.102512015	9.196533591	8.550024073	8.70739158	9.676249936	10.01492601
ENSG00000168476	REEP4	-0.981920557	0.642855534	-0.28447624	0.900496697	-1.266396797	9.448362284	7.572420273	2	7.265793319	7.24216308	7.988280194	7.883613091
ENSG00000253304	TMEM200B	-0.649873396	0.398651947	-0.617174701	0.415812209	-1.267048096	9.143983763	7.790474954	7.777116083	7.723090567	7.77052502	7.991357103	7.529919928
ENSG00000180957	PITPNB	-0.800552055	0.018276173	-0.467707399	0.186976308	-1.268259455	10.33377634	10.019219	9.767769415	9.260887574	9.26398924	9.499242667	9.681766233
ENSG00000087157	PGS1	-0.871035955	0.11367876	-0.39735864	0.495466824	-1.268394595	8.042902253	8.777382925	9.242516611	7.905589775	7.909304	8.375618113	8.373144057
ENSG00000146918	NCAPG2	-1.317319372	0.310604101	0.048669962	0.973559952	-1.26864941	4.00722531	7.051611315	7.858115942	5.818703821	5.64780787	6.948868859	7.114266974
ENSG00000094804	CDC6	-0.893111324	0.528693205	-0.377976936	0.798498793	-1.27108826	5.532353208	6.666836837	3.369317972	5.076005082	4.79082474	4.771475226	5.8339184
ENSG00000153902	LGI4	-2.435450369	0.179526399	1.164155868	0.52507775	-1.271294501	4.00722531	4.93638933	2	3.023133209	2.2680111	5.489669427	4.522269241
ENSG00000182552	RWDD4	-0.417995771	0.751812007	-0.85606099	0.486233434	-1.274056761	9.262228249	6.78762695	6.43197674	7.691737974	7.6378145	7.31213785	7.157719456
ENSG00000198951	NAGA	-1.444442656	0.015168155	0.170092649	0.812274339	-1.274350007	9.097018025	10.06773758	8.763901704	8.001694993	7.98797735	9.483758913	9.696573548
ENSG00000132434	LANCL2	-0.612725678	0.702917775	-0.661993304	0.674981326	-1.274718982	4.00722531	8.687092496	8.677268317	7.370304568	7.66419009	7.25183424	7.670954172
ENSG00000187531	SIRT7	-0.801713481	0.374121952	-0.47327325	0.610378131	-1.274986731	8.540903468	6.389896633	8.076950877	7.080147179	7.1744553	7.308441828	7.575491915
ENSG00000077380	DYNC1I2	-0.078164343	0.943520433	-1.197581085	0.197561623	-1.275745428	10.26462289	10.54992047	8.042706615	9.911131561	9.86046766	8.707577112	8.837005439
ENSG00000177879	AP3S1	-0.595367349	0.350878999	-0.6811832	0.270175158	-1.276550548	10.46286899	9.470501606	9.212024683	9.290680758	9.16279627	9.173900195	9.111258115
ENSG00000145348	TBCK	-0.621014685	0.426810018	-0.656106944	0.387074442	-1.277121629	7.709880939	9.018611864	8.960138144	7.966401501	8.15434112	7.461614824	8.435201779
ENSG00000197912	SPG7	-0.893655615	0.105206276	-0.383484141	0.515470896	-1.277139756	10.84091191	9.648522413	10.26770388	9.357592295	9.52049436	9.930233463	9.967609306
ENSG00000243709	LEFTY1	0.637088348	0.832852241	-1.916287705	NA	-1.279199356	3.327711234	2	2	3.023133209	2.59489879	2.183373728	2.172608095
ENSG00000144231	POLR2D	-0.970429022	0.002036692	-0.311235857	0.371207868	-1.281664878	9.174465797	8.88960062	8.677268317	7.990026167	7.94798467	8.619836896	8.619914108
ENSG00000135070	ISCA1	-0.682192379	0.284613367	-0.600352267	0.34281457	-1.282544646	9.710019321	8.414402626	8.784770914	8.43768081	8.35656898	8.471282103	8.485861839
ENSG00000138587	MNS1	-2.27056554	0.313384316	0.987578811	0.670910636	-1.28298673	2	5.131603625	2	2.784612485	2.49394783	4.588526421	4.766376438
ENSG00000167004	PDIA3	0.237438585	0.699202151	-1.520696938	0.00262317	-1.283258353	13.1430848	12.32301536	12.37843207	12.88916971	12.9165825	10.74479855	11.46056057
ENSG00000182220	ATP6AP2	-0.212260129	0.652346084	-1.072729571	0.006872527	-1.2849897	11.9089726	11.13786633	11.34128524	11.30297958	11.2729696	10.44029952	10.41854076
ENSG00000102178	UBL4A	-0.84219375	0.153980097	-0.442886933	0.473146336	-1.285080683	8.586954094	9.799806309	9.61300511	8.551612617	8.62623826	9.135476483	8.817088095
ENSG00000089220	PEBP1	-1.074419213	0.012676892	-0.21108279	0.674981326	-1.285502002	12.37539409	11.95020188	11.6648807	10.9673516	10.9400378	11.51999629	12.0611512
ENSG00000132879	FBXO44	-0.938157072	0.586967447	-0.350715008	0.84812366	-1.288872079	9.657036304	7.538394143	4.058998049	7.536750104	7.40393901	8.126237806	7.966097995
ENSG00000141456	PELP1	-1.39756533	0.004858833	0.107548874	0.861820805	-1.290016456	8.87372952	9.28800521	9.963514551	7.994705029	8.12299044	9.584486439	9.517360075
ENSG00000095485	CWF19L1	-0.47484668	0.565600882	-0.815636972	0.290853649	-1.290483652	8.998234871	7.60566235	7.398165132	7.700356475	7.73502229	7.358152527	7.409982764
ENSG00000198131	ZNF544	-0.639659198	0.48167816	-0.652572391	0.462949352	-1.292231589	8.946185348	7.189546432	9.196533591	8.004017475	8.0713677	7.680435163	8.303661547
ENSG00000099960	SLC7A4	-4.714923371	0.18460402	3.422223338	0.26685414	-1.292700033	2	3.076665209	2	2	2	4.644324387	4.673629022
ENSG00000184924	PTRHD1	-0.874313282	0.358348477	-0.419297372	0.675137901	-1.293610653	8.340423424	7.274681896	6.073378139	6.70517998	6.61334686	6.807643042	7.342293759
ENSG00000082515	MRPL22	-0.257195395	0.734460813	-1.037921361	0.114849347	-1.295116756	7.384433967	8.687092496	7.502634626	7.751013478	7.7377845	7.016881172	6.947999751
ENSG00000111696	NT5DC3	-1.851588158	0.056230678	0.556211282	0.604593035	-1.295376876	6.366709671	8.433041974	9.180874355	6.658765962	6.507263	8.920684672	8.965313405
ENSG00000164265	SCGB3A2	-4.229205342	0.146700672	2.931093823	0.205667088	-1.298111519	4.00722531	2	2	2.141737246	2	4.920961476	5.191251321
ENSG00000151445	VIPAS39	-0.497351743	0.821777402	-0.800862822	0.701757981	-1.298214564	9.497412313	8.414402626	2	7.93509946	8.03365075	7.675661224	7.698285408
ENSG00000171490	RSL1D1	-0.592088588	0.07325688	-0.706578269	0.027268332	-1.298666856	11.04852648	10.50217373	10.81097638	10.22664935	10.1974239	10.12211394	10.07350205
ENSG00000005022	SLC25A5	-1.11220466	0.000728334	-0.18849253	0.629414332	-1.300697189	10.65885801	10.24169813	10.22252928	9.298270478	9.26303083	10.02917428	10.35294498
ENSG00000113594	LIFR	-1.194965702	0.01989306	-0.109672298	0.859331521	-1.304638	6.89194353	7.901717006	7.342949392	6.328984825	6.26836889	7.35218116	7.337766965
ENSG00000136875	PRPF4	-0.603775814	0.163887287	-0.701113654	0.093533047	-1.304889468	8.367060731	8.505280662	9.083172337	8.040677544	8.12931531	8.030023089	7.94839122
ENSG00000173153	ESRRA	-0.610315052	0.491805453	-0.696387182	0.418338064	-1.306702235	9.509418609	8.356994601	7.552162999	8.096156705	8.08878133	8.247007147	7.720479276
ENSG00000277462	ZNF670	-0.243709916	0.857421507	-1.065214007	0.3650389	-1.308923923	4.00722531	4.710564154	6.534109959	5.258265913	5.24368216	4.620674722	4.456980017
ENSG00000163812	ZDHHC3	-0.830420795	0.190607653	-0.479726951	0.465741681	-1.310147746	10.9638027	9.664384941	9.981704053	9.466861018	9.50517467	9.700423741	9.958300939
ENSG00000170633	RNF34	-0.573360651	0.751812007	-0.737374828	0.676516223	-1.310735478	4.00722531	8.124485672	9.926432745	8.111316053	8.19912382	8.032265607	7.955303035
ENSG00000072135	PTPN18	-1.154923281	0.349969585	-0.158500863	0.910302233	-1.313424144	5.70932728	8.414402626	9.344432377	7.238438152	7.37229174	8.090815298	8.456236734
ENSG00000092203	TOX4	-0.977112121	0.06635276	-0.338413585	0.561013956	-1.315525706	9.699577239	10.36815316	10.88048583	9.41544358	9.42689279	10.06648818	10.04717102
ENSG00000101916	TLR8	-3.753023129	0.531143362	2.434989022	NA	-1.318034107	2	2	2	2	2	3.679182183	2.632958241
ENSG00000164114	MAP9	-0.392110266	0.568384743	-0.925943256	0.136351095	-1.318053522	8.285626337	7.503546079	8.884815469	7.971157485	7.90193448	7.540404604	7.274433587
ENSG00000147669	POLR2K	-0.751971497	0.315751098	-0.569520156	0.451396209	-1.321491653	10.11549941	8.981105197	8.486635645	8.595396228	8.63367258	8.889688585	8.694392964
ENSG00000132670	PTPRA	-0.220863738	0.794969986	-1.10072301	0.127918786	-1.321586748	9.646202137	10.69982928	11.46604358	10.54043168	10.5879489	9.613232802	9.758568089
ENSG00000164331	ANKRA2	-0.626260046	0.335559831	-0.695385451	0.269861386	-1.321645497	8.39321512	8.655694736	7.502634626	7.691737974	7.61094772	7.874567387	7.219718817
ENSG00000204237	OXLD1	-0.792549038	0.235847119	-0.529336823	0.437014882	-1.321885861	8.19931886	6.899080544	8.076950877	6.970413838	7.1204461	7.47706242	7.105417192
ENSG00000183765	CHEK2	0.761990294	0.668927862	-2.085063831	0.191114239	-1.323073537	8.816894989	6.464272742	3.369317972	8.213192703	8.31463236	5.545162502	5.513708555
ENSG00000186806	VSIG10L	-0.027965848	0.989663573	-1.297571982	0.491100919	-1.32553783	3.327711234	8.169643011	4.058998049	6.658765962	6.73446058	5.557671049	5.448021784
ENSG00000139697	SBNO1	-0.566935356	0.205078218	-0.759074013	0.07517382	-1.326009369	10.00958825	10.019219	10.62096771	9.652999939	9.70612498	9.68720202	9.258230494
ENSG00000152700	SAR1B	-0.756204641	0.154010606	-0.570300222	0.286633227	-1.326504863	10.58135316	9.591588447	10.60345421	9.598713994	9.54829592	9.721422299	9.795075587
ENSG00000101452	DHX35	-0.89625539	0.565047493	-0.431302369	0.792001716	-1.327557758	3.327711234	7.467835385	8.110401118	6.283823264	6.52015598	6.944112782	6.755525668
ENSG00000172819	RARG	-0.529522998	0.428683492	-0.798079675	0.204147015	-1.327602672	9.219014269	9.930175364	10.65536953	9.477763976	9.56860713	9.220861732	9.292162928
ENSG00000105186	ANKRD27	-0.628122517	0.212797514	-0.70082079	0.15123158	-1.328943307	9.579430841	8.687092496	9.049077186	8.537252353	8.51470562	8.627767864	8.256474583
ENSG00000127580	WDR24	-0.724908677	0.728237469	-0.60446095	0.773321468	-1.329369626	2	7.431218269	8.941672936	7.165617905	7.01053089	7.403899888	6.979338179
ENSG00000204514	ZNF814	-0.93519648	0.200334149	-0.394779781	0.610752069	-1.329976261	6.563806279	7.954278496	7.398165132	6.470063213	6.56439512	7.360534171	6.622902297
ENSG00000168528	SERINC2	-0.157578465	0.88438939	-1.172409374	0.19277085	-1.329987839	10.56993315	8.876041558	8.511888463	9.404936476	9.51083771	8.195243072	8.670591511
ENSG00000185946	RNPC3	0.239382337	0.881720015	-1.569991233	0.245468671	-1.330608897	7.384433967	7.503546079	3.369317972	7.093295078	7.18663697	5.22272385	5.611957759
ENSG00000054179	ENTPD2	-1.269030326	0.690257999	-0.062199576	NA	-1.331229902	2	2	3.369317972	2.3892346	2.14022021	2.68698273	2.421067459
ENSG00000143851	PTPN7	-3.753023295	0.531143362	2.421672991	NA	-1.331350304	2	2	2	2	2	3.796405078	2.326754077
ENSG00000104731	KLHDC4	-1.295929675	0.534439893	-0.036624464	0.987416408	-1.332554139	9.015175615	6.389896633	2	6.408278948	6.39275508	7.618102344	7.620910859
ENSG00000132823	OSER1	-0.397262233	0.790951603	-0.936139736	0.495682443	-1.333401969	10.59268348	8.820493869	6.43197674	9.067921572	9.02033034	8.466314923	8.553143267
ENSG00000235708		3.857403838	0.496953626	-5.191090708	0.364514502	-1.333686869	2	2	2	3.95996233	4.45712836	2	2
	C15ORF52	6.757112528	2.69E-09	-8.090970261	3.49E-05	-1.333857732	4.467311984	2	3.369317972	9.677214349	9.70330142	2	2
ENSG00000010244	ZNF207	-0.29019246	0.619015165	-1.043678015	0.039749923	-1.333870475	11.55939759	10.54992047	10.77492406	10.69648168	10.7797423	9.842864719	10.11906036

ENSG00000172292	CERS6	-1.129786443	0.580580155	-0.204186762	0.927897448	-1.333973204	8.313285031	9.338312924	2	7.366699513	7.0774796	8.263008721	7.998006535
ENSG00000149257	SERPINH1	-1.197349674	0.048926942	-0.137559493	0.850018602	-1.334909167	14.21556964	13.04192929	13.01031565	12.31942734	12.3646385	13.33642019	13.46361928
ENSG00000168995	SIGLEC7	-3.753023353	0.531143362	2.417054371	NA	-1.335968981	2	2	2	2	2	3.500668998	2.918126211
ENSG00000106511	MEOX2	-3.753023356	0.531143362	2.416770992	NA	-1.336252364	2	2	2	2	2	2.560105256	3.698365557
ENSG00000136213	CHST12	-0.96881867	0.686456446	-0.367501968	0.884851957	-1.336320637	10.59268348	7.35507212	2	8.166267464	8.22691474	8.798213877	8.784747976
ENSG00000168427	KLHL30	-6.103015833	0.006359008	4.765727346	0.001345688	-1.337288487	3.327711234	2	5.39244166	2	2.14022021	8.737205015	8.44505627
ENSG00000143552	NUP210L	0.311380123	0.927479541	-1.650689564	NA	-1.339309442	2	2	3.369317972	2.600425781	2.77779907	2.34604626	2.059860178
ENSG00000168374	ARF4	-0.711093699	0.138848227	-0.63010723	0.187257885	-1.341200929	13.00468149	12.17723115	12.27897381	11.84965902	11.8008967	11.74630693	12.05047073
ENSG00000189369	GSPT2	-0.584219674	0.313991908	-0.757985921	0.170908786	-1.342205595	8.444143041	7.73146271	8.904018013	7.829061569	7.87709394	7.623070264	7.740051619
ENSG00000118816	CCNI	-1.489329495	6.29E-05	0.146842973	0.750755237	-1.342486521	11.91800836	11.58596781	12.33487604	10.46657187	10.5157558	12.21344546	12.03038993
ENSG00000173674	EIF1AX	0.201137383	0.592806105	-1.544819259	3.41E-07	-1.343681876	9.189468652	9.608085847	9.301632639	9.650778342	9.50922197	7.95714469	7.741194706
ENSG00000071282	LMCD1	-1.910770364	3.75E-06	0.56549001	0.22056224	-1.345280354	8.75772933	9.114109157	9.491321578	7.341208691	7.19067478	9.471691847	9.919950354
ENSG00000159761	C16orf86	-1.155511044	0.525813654	-0.190008756	0.925215975	-1.3455198	6.653098118	4.442710129	2	4.415698455	4.37434574	5.196301326	5.243813244
ENSG00000169045	HNRNPH1	-0.210304565	0.695936302	-1.135357644	0.01186525	-1.345662209	11.51244431	10.69982928	10.71094342	10.86022322	10.7716784	9.816867623	9.96809756
ENSG00000180879	SSR4	0.060099163	0.930223809	-1.405854382	0.008676361	-1.34575522	11.48534568	10.60438382	10.31867969	10.95779865	10.9454246	9.404033465	9.572665028
ENSG00000151575	TEX9	-0.032780982	0.990154914	-1.313383998	0.576534502	-1.34616498	2	5.457120953	2	4.070463721	4.1930041	3.355392153	3.15612297
ENSG00000101407	TTI1	-0.790665793	0.629335817	-0.556755248	0.736888343	-1.347421041	4.00722531	9.00621767	9.180874355	7.683067677	7.8208909	8.04860508	7.915353355
ENSG00000163159	VPS72	-1.345433715	0.031713694	-0.002754463	0.997599173	-1.348188178	9.761125267	8.255926468	9.132850258	7.831679003	7.85181819	9.154473165	9.183675168
ENSG00000125170	DOK4	-0.84824569	0.02494348	-0.499999872	0.205129503	-1.348245562	9.097018025	9.687856435	9.735974718	8.682071821	8.70598173	8.967137431	9.108160402
ENSG00000132017	DCAF15	-0.545980619	0.427719039	-0.803827796	0.214346841	-1.349808414	7.975256505	7.638155693	8.978369997	7.734324667	7.80517502	7.446000032	7.585709303
ENSG00000164638	SLC29A4	-2.19838324	0.154820857	0.848179658	0.608615553	-1.350203582	5.70932728	8.101365653	3.369317972	4.946755474	4.63360314	7.777203086	7.511280276
ENSG00000147121	KRBOX4	-0.255405013	0.711497595	-1.096616753	0.064293553	-1.352021765	7.031698319	7.002537973	8.042706615	7.114945424	7.2537885	6.216130818	6.520103598
ENSG00000100483	VCPKMT	-0.782080185	0.567927475	-0.570127742	0.681431908	-1.352207927	4.00722531	6.535001748	7.646393253	5.870535235	6.00960265	6.475428841	5.703940561
ENSG00000145022	TCTA	-0.681381135	0.463231524	-0.670834195	0.460415275	-1.35221533	9.957954117	9.267380431	8.236946975	8.579911316	8.69891174	7.795801481	9.184515751
ENSG00000167995	BEST1	-5.447844384	0.060484839	4.095447447	0.078793483	-1.352396937	2	3.686303414	2	2	2	6.134985075	5.6562603
ENSG00000213413	PVRIG	1.199646954	0.594033397	-2.553241732	0.220592048	-1.353594778	2	5.303523747	2	5.110902178	4.93258395	2.592885947	2.632958241
ENSG00000070961	ATP2B1	0.849295143	0.258493876	-2.20534949	0.00097064	-1.356054347	9.820154628	11.631062	11.16147894	11.90445538	11.8959261	8.858011903	8.855068552
ENSG00000113971	NPHP3	0.901872832	0.366585534	-2.258151107	0.011804798	-1.356278275	6.816637977	9.00621767	7.163363831	8.915879855	8.89354047	5.935372196	5.73181021
ENSG00000141380	SS18	-0.351722409	0.588327109	-1.004943761	0.081826473	-1.35666617	10.41898845	10.92721483	9.589475645	10.06420548	10.0623662	9.357098224	9.467122411
ENSG00000198093	ZNF649	-0.720133918	0.59691102	-0.636600467	0.638247848	-1.356734385	8.16936288	6.04685525	4.874587708	6.358325776	6.24526024	6.57313027	6.162172032
ENSG00000155256	ZFYVE27	-0.259602376	0.795427588	-1.097196177	0.205667088	-1.356798553	10.30651143	8.395519306	8.677268317	9.135152476	9.12531607	8.515767839	8.047821014
ENSG00000178307	TMEM11	-1.094985281	3.05E-05	-0.262572416	0.37875047	-1.357557697	9.247966931	9.067150531	9.049077186	8.115618198	7.95986274	8.899958346	8.824117457
ENSG00000144713	RPL32	-1.157783461	0.168223251	-0.202174821	0.833553126	-1.359958282	12.97143233	11.07398149	11.39656351	10.92967084	10.8937291	11.79433308	11.93421333
ENSG00000136819	C9orf78	-0.500868214	0.362508916	-0.859655308	0.09315428	-1.360523522	10.22148166	9.367676479	9.242516611	9.147810675	9.21229508	8.79205135	8.856124078
ENSG00000144182	LIPT1	-0.698661558	0.676600545	-0.663404235	0.687792014	-1.362065793	3.327711234	6.78762695	4.058998049	5.040242897	4.79082474	5.108342547	4.77533149
ENSG00000150753	CCT5	-0.876278972	0.000559668	-0.488974869	0.068678166	-1.365253841	11.13243651	11.19085087	11.2124308	10.33669748	10.2722069	10.51007546	10.85192199
	APITD1-CORT	4.786738953	0.038248139	-6.152940704	0.029610048	-1.366201751	3.327711234	2	2	5.590471342	6.11534392	2	2
ENSG00000148730	EIF4EBP2	-1.005701782	0.113324776	-0.361215687	0.600600207	-1.366917469	10.19941743	10.95971654	11.51370025	9.98823217	9.98077219	10.25141933	10.92466139
ENSG00000173588	CEP83	-0.718490093	0.43340631	-0.648721108	0.47051548	-1.3672112	6.257048221	6.228547981	4.874587708	5.590471342	4.85329164	5.528313928	5.101530266
ENSG00000198825	INPP5F	-1.100725665	0.201926325	-0.267641644	0.781305953	-1.368367309	8.16936288	10.28338212	9.196533591	8.374462892	8.37974396	9.422673149	8.942636839
ENSG00000163904	SENP2	-0.786173651	0.231765386	-0.582662911	0.379991231	-1.368836562	8.609439662	9.308339287	10.08627303	8.684969711	8.67028013	8.851676897	8.906411656
ENSG00000181222	POLR2A	-0.721324321	0.290454869	-0.647694924	0.338044189	-1.369019245	11.15554094	12.70874416	12.17214792	11.42152442	11.4291609	11.40918076	11.58331566
ENSG00000117450	PRDX1	0.221158612	0.693063949	-1.590613978	0.000467379	-1.369455366	12.03890377	11.07694755	11.57488832	11.83750166	11.8354782	9.820764745	10.21041545
ENSG00000198298	ZNF485	-1.22731531	0.624769744	-0.144134019	0.957898398	-1.371449329	2	5.838937808	2	3.663521627	3.45167086	4.596630906	4.077147165
ENSG00000148337	CIZ1	-0.908724213	0.126427717	-0.465406477	0.456352327	-1.37413069	11.25516263	10.20962523	10.09465441	9.734282329	9.69337522	10.1959872	10.11377463
ENSG00000183943	PRKX	-1.317039212	0.385680172	-0.058183581	0.97269175	-1.375222793	4.00722531	7.393647515	8.536706852	6.18083559	6.33555364	7.992125306	6.659638722
ENSG00000127554	GFER	-0.436483398	0.609544685	-0.938771847	0.228844962	-1.375255245	8.586954094	6.78762695	8.460932916	7.700356475	7.70992146	7.279786628	7.145684098
ENSG00000102978	POLR2C	-0.886016599	0.00307497	-0.489529851	0.12122066	-1.37554645	10.04301211	10.00683004	10.44204362	9.253098082	9.33320115	9.679353528	9.696573548
ENSG00000108590	MED31	1.10063578	0.076688241	-2.476579785	1.87E-05	-1.375944005	5.095959458	6.04685525	5.930865648	6.782976084	6.83074839	3.694366604	3.717076715
ENSG00000170312	CDK1	-0.770369935	0.652705192	-0.606385745	0.723776062	-1.37675568	3.327711234	6.311476754	3.369317972	4.670864177	4.16043394	4.350488971	4.766376438
ENSG00000172922	RNASEH2C	-0.505808342	0.680089245	-0.872869338	0.447209151	-1.37867768	9.544849385	8.607278467	6.322058544	8.207157886	8.12087597	7.54878318	8.019835704
ENSG00000102595	UGGT2	-0.538138459	0.471279327	-0.842516768	0.228217127	-1.380655227	8.653385977	9.883504052	8.434763955	8.587674547	8.63663561	8.291371768	8.331250243
ENSG00000134308	YWHAQ	-0.331323461	0.581201394	-1.050065408	0.046784923	-1.381388869	12.90368053	11.83753936	12.01322718	12.00411861	11.9950723	11.18792045	11.36989314
ENSG00000177951	BET1L	-0.97979521	0.07251956	-0.401610275	0.493153856	-1.381405485	9.710019321	10.86336859	10.28245311	9.333786218	9.43967292	9.799418565	10.11001882
ENSG00000165389	SPTSSA	-1.119292087	0.20765518	-0.262341327	0.792037041	-1.381633415	9.015175615	8.557180776	6.718950348	7.288834728	7.27676203	8.061837451	8.18182607
ENSG00000177732	SOX12	-1.169108991	0.304573038	-0.212911558	0.867658115	-1.382020549	7.435667355	9.903691196	10.74860941	8.71221318	8.74905888	9.689335129	9.671309937
ENSG00000150455	TIRAP	-0.34830253	0.881863329	-1.035345443	0.625695912	-1.383647973	8.367060731	5.131603625	2	6.516352584	6.68951263	5.982522014	5.925257518
ENSG00000186687	LYRM7	-1.366298137	0.004556921	-0.017419314	0.976832019	-1.383717451	8.19931886	7.503546079	7.398165132	6.415275989	6.42052719	7.884531292	7.545707309
ENSG00000136732	GYPC	0.032080908	0.981681548	-1.416062718	0.210179769	-1.38398181	10.03472846	9.574900212	6.629488387	9.339314897	9.35585278	8.015738956	7.805990373
ENSG00000250021	C15orf38-AP	6.661749443	1.04E-09	-8.046248376	3.01E-05	-1.384498933	4.467311984	3.076665209	2	9.565291356	9.5364465	2	2
ENSG00000143653	SCCPDH	-0.739918221	0.213670455	-0.646803359	0.274221768	-1.386721579	9.771132748	8.702538795	9.819251342	8.736160353	8.81462916	8.658095041	9.051693903
ENSG00000065427	KARS	-0.567409088	0.518602353	-0.819986079	0.323125242	-1.387395168	11.48534568	10.28850895	9.491321578	10.10480594	10.0733424	9.714922106	9.950662535
ENSG00000100109	TFIP11	-0.222675115	0.825480361	-1.16498424	0.174300946	-1.387659355	9.810482605	8.639735838	7.600047325	8.736160353	8.73530306	7.88535852	7.72163797
ENSG00000145919	BOD1	-0.412434843	0.359833218	-0.977563354	0.016000913	-1.389998197	9.384607364	9.949724236	10.16797309	9.466861018	9.45318143	8.997295259	8.793039018
ENSG00000167377	ZNF23	1.152120315	0.506977228	-2.542587672	0.107289325	-1.390467357	7.384433967	5.838937808	2	7.398825229	7.31795292	4.017766949	4.047883918
ENSG00000116661	FBXO2	-1.097844092	0.585772669	-0.293384722	0.892702877	-1.391228814	7.667647223	4.93638933	2	5.258265913	5.3193943	6.275391383	5.73181021
ENSG00000121864	ZNF639	-0.774832779	0.535148173	-0.618292881	0.623218139	-1.39312566	7.33131396	7.431218269	10.04361983	8.134820652	8.0358969	8.360824221	8.109313767
ENSG00000115541	HSPE1	0.204671961	0.826701316	-1.598596648	0.036351322	-1.393924687	8.998234871	7.393647515	7.552162999	8.370868225	8.37620272	6.855708005	6.320441373
ENSG00000116985	BMP8B	-1.849749257	0.485018665	0.455290059	0.872155485	-1.394459198	2	2	4.874587708	2.868578001	2.38539787	4.388329244	3.236138336
ENSG00000277586	NEFL	0.882350118	0.469356107	-2.27697561	0.037729049	-1.394625492	4.815609471	6.228547981	4.523675391	6.5612028	5.83486613	3.981090143	3.042026731
ENSG00000172663	TMEM134	-1.233917966	0.041949021	-0.162672449	0.820142868	-1.396590415	8.963744667	8.276714465	7.552162999	7.202487431	7.12888795	8.209845962	8.222500688
ENSG00000137168	PPIL1	-0.948615344	0.065184591	-0.44863372	0.410449693	-1.397249065	9.064836028	8.557180776	8.175061096	7.791909949	7.62295033	7.924517766	8.434495305
ENSG00000163882	POLR2H	-0.617911612	0.425471375	-0.780737085	0.291836129	-1.398648697	9.771132748	8.557180776	8.206335842	8.266399315	8.51470562	8.102245405	8.356830552
ENSG00000130244	FAM98C	-1.222438416	0.536668932	-0.17783759	0.935430183	-1.400276006	8.138771685	5.72254323	2	5.639097847	5.6826301	6.842137779	6.418016952
ENSG00000244005	NFS1	-1.084533436	0.154269477	-0.315831952	0.705751145	-1.400365387	7.940205782	7.145017454	8.884815469	6.993973425	7.18663697	7.992893101	7.679327321
ENSG00000188529	SRSF10	-0.541982045	0.087151155	-0.859008322	0.00372404	-1.400990367	9.931427102	10.19331737	10.40208303	9.719519122	9.57325401	9.319337188	9.346500876
ENSG00000149089	APIP	-0.187606438	0.917511462	-1.213563819	0.443133621	-1.401170257	8.564112515	7.002537973	3.369317972	7.178012736	7.31055061	6.170709933	6.341697848
ENSG00000171824	EXOSC10	-0.189222728	0.874589067	-1.213366406	0.228416028	-1.402589134	10.58702944	9.102512015	7.858115942	9.382797773	9.42945789	8.342266573	8.438024223
ENSG00000116815	CD58	0.406033062	0.730567296	-1.809396083	0.074893479	-1.403363021	8.16936288	5.595927442	8.175061096	8.024753265	8.16258751	6.297933466	5.502966356
ENSG00000151148	UBE3B	-0.946776905	0.408383735	-0.457013817	0.702456266	-1.403790722	8.285626337	8.806266215	10.9968459	8.920805049	8.93022744	9.375172844	9.447647875
ENSG00000138780	GSTCD	-1.027438739	0.085059099	-0.376369956	0.555749022	-1.403808695	7.33131396	7.051611315	6.43197674	5.997179931	6.01871653	6.894026089	6.29575846
ENSG00000011304	PTBP1	-0.734396537	0.021914674	-0.672570304	0.036138686	-1.40696684	11.99046984	11.55651981	12.04601058	11.14385097	11.1479752	11.11829161	11.29179109
ENSG00000118707	TGIF2	-1.332572964	0.380936196	-0.074711323	0.966296927	-1.407284287	9.112843761	8.451443573	4.058998049	6.965655398	6.94988431	8.17644121	8.200230127
ENSG00000125520	SLC2A4RG	-1.56405937	1.61E-10	0.15548627	0.604131365	-1.4085731	9.624286623	9.785372135	9.589475645	8.098332103	8.14396641	9.917551697	9.728912497
ENSG00000110768	GTF2H1	-0.346117262	0.549552855	-1.062491844	0.03743516	-1.408609106	9.081016759	9.246456519	10.10298738	9.209487455	9.19732195	8.511487655	8.474909484
ENSG00000198898	CAPZA2	-0.388805723	0.565888439	-1.020156226	0.093459108	-1.408961949	9.839306291	10.08551947	11.10866152	10.09230881	10.0396007	9.561639062	9.302266408
ENSG00000031823	RANBP3	-0.686172248	0.360070875	-0.723068617	0.323118682	-1.409240865	10.62062599	9.203675681	9.25752426	9.15618819	9.18421833	8.96830908	9.281988193
ENSG00000132394	EEFSEC	-1.382070308	0.475887749	-0.02749862	0.989709187	-1.409568927	2	8.469613413	8.65477198	6.716553866	6.56439512	7.886185273	8.032963099
ENSG00000137834	SMAD6	-1.63141754	0.239845221	0.221532425	0.890149172	-1.409885115	6.73718411	4.93638933	8.941672936	6.172600389	6.08962627	7.768261726	8.064355282
ENSG00000172179	PRL	3.778423042	0.507060161	-5.191091037	0.364514502	-1.412667994	2	2	2	4.387512679	3.90918929	2	2
ENSG00000213928	IRF9	2.337281161	0.138263648	-3.75071641	0.01144673	-1.41343525	9.081016759	5.595927442	4.523675391	9.909893561	10.0596091	3.930690651	4.489993931
ENSG00000160917	CPSF4	-0.879546085	0.490472675	-0.534525116	0.683123451	-1.414071201	5.330594134	7.954278496	8.941672936	7.234487582	7.08183429	7.688988646	7.274433587
ENSG00000243927	MRPS6	-0.772125796	0.20465032	-0.6430478	0.290375858	-1.415173595	9.371511179	9.079034257	10.22252928	8.870779703	8.87484109	9.228705128	8.729950594
ENSG00000161298	ZNF382	-2.012945014	0.285993957	0.597456042	0.770772698	-1.415488972	2	5.131603625	7.552162999	4.358765249	4.50979031	7.256956941	6.141542327
ENSG00000186272	ZNF17	-0.636105664	0.750725045	-0.780816649	0.688756148	-1.416922313	2	6.228547981	8.007629717	6.237202172	6.1572134	6.282944614	5.799216178
ENSG00000100196	KDELR3	-1.396370439	2.29E-06	-0.020553286	0.958967393	-1.416923724	11.44215374	11.07100932	11.5927499	9.976453497	10.0044775	11.41320151	11.31068228
ENSG00000105281	SLC1A5	-0.92707703	0.009556581	-0.491065545	0.194623596	-1.418142575	11.13243651	11.07990752	10.9743126	10.07142123	10.2039308	10.85013781	10.23171747
ENSG00000008405	CRY1	-1.433307673	3.66E-07	0.014893458	0.968553627	-1.418414215	8.444143041	8.234834549	8.296287027	6.936770382	6.90635305	8.351874517	8.326688609
ENSG00000261371	PECAM1	-1.067670142	0.558688888	-0.351251475	0.856791105	-1.418921617	7.579279349	3.076665209	5.39244166	5.304221918	5.36298494	6.660772804	4.711446447
ENSG00000102780	DGKH	-0.620610512	0.318938708	-0.798759634	0.180023668	-1.419370145	8.631580145	9.479495904	9.819251342	8.778830996	8.77079897	8.859275573	8.279474384
ENSG00000129204	USP6	2.038085675	0.103636128	-3.457762684	0.003716817	-1.419677009	3.327711234	4.442710129	6.534109959	7.2696592	7.29187696	2.937246078	2.817676617
ENSG00000144029	MRPS5	-0.926735356	0.271482843	-0.493608252	0.573477412	-1.420343609	9.521325812	9.214490408	7.451345288	8.029321023	8.08010078	8.478974993	8.485861839
ENSG00000173950	XXYLT1	-1.318582962	0.113829415	-0.102655292	0.918231554	-1.421238254	10.5468183	8.395519306	9.647595056	8.471640654	8.46389529	9.505174684	9.825841298
ENSG00000187726	DNAJB13	-2.278728721	0.566415954	0.857377365	NA	-1.421351356	2	3.076665209	2	2	2.2680111	3.193831101	2.592996859
ENSG00000243943	ZNF512	-0.721375787	0.739034243	-0.70164277	0.743381156	-1.423018557	9.751047882	8.762721501	2	7.975897842	8.11451391	8.005883472	8.124285971
ENSG00000180318	ALX1	-3.753024452	NA	2.32901662	NA	-1.424007832	2	2	2	2	2	3.393105702	2.950113106
ENSG00000007520	TSR3	-0.719501244	0.48167816	-0.706964579	0.480981934	-1.426465823	7.667647223	8.607278467	10.12770132	8.468280417	8.41468635	8.388512422	8.516894472

ENSG00000153250	RBMS1	-0.324072554	0.626363416	-1.102754054	0.058325459	-1.426826607	10.19941743	9.235879652	10.58572549	9.845303961	9.72155655	9.050629989	8.969708735
ENSG00000239672	NME1	-0.40652937	0.548500399	-1.020583053	0.094571914	-1.427112423	9.304185368	8.993716073	10.09465441	9.078979414	9.1882629	8.099396357	8.855068552
ENSG00000122557	HERPUD2	-1.177713482	0.019477382	-0.249556202	0.66811141	-1.427269684	9.646202137	8.903033433	8.699419242	8.054188409	7.89452713	9.058352796	8.707903068
ENSG00000067191	CACNB1	-1.300005714	0.184030119	-0.127446876	0.911555747	-1.42745259	8.737455672	6.228547981	7.600047325	6.604700567	6.57060569	7.494516625	7.926450253
ENSG00000114354	TFG	-0.678566375	0.013719923	-0.749424836	0.005580838	-1.427991211	11.59363617	11.56499503	11.26536143	10.82288924	10.7877614	10.62175706	10.83956746
ENSG00000055950	MRPL43	-1.186486767	0.005498029	-0.241706463	0.626190802	-1.42819323	9.848787621	9.367676479	8.99637432	8.23312837	8.30721295	9.093139938	9.311530309
ENSG00000072422	RHOBTB1	-1.947510101	7.69E-06	0.519113486	0.288589938	-1.428396615	8.998234871	8.356994601	8.175061096	6.79375511	6.48770488	9.068221759	9.066817089
ENSG00000103495	MAZ	0.308644541	0.754977416	-1.737572729	0.03707375	-1.428928188	10.1076228	9.514922632	7.777116083	9.727271068	9.72712735	7.778984728	7.620910859
ENSG00000187151	ANGPTL5	-0.461622215	NA	-0.968100316	NA	-1.429722531	2	3.076665209	2	2.600425781	2.2680111	2	2.592996859
ENSG00000135378	PRRG4	-0.197249917	0.860437532	-1.232726573	0.188401866	-1.42997649	4.815609471	4.11352228	5.39244166	4.647285135	4.68029874	4.029788235	3.640730416
ENSG00000169189	NSMCE1	-1.285333831	0.164037813	-0.144794174	0.893463707	-1.430128005	8.816894989	6.535001748	7.646393253	6.548530093	6.83074839	7.618102344	7.972925696
ENSG00000204969	PCDHA2	0.921247835	0.755643383	-2.351531034	0.392854305	-1.4302832	2	3.686303414	2	3.79808662	2.68924543	2.094598369	2.326754077
ENSG00000130635	COL5A1	-1.482334768	7.19E-08	0.051939177	0.888119558	-1.43039559	14.59270693	14.92196164	14.44962988	13.14048624	13.231656	14.71263075	14.72864471
ENSG00000130309	COLGALT1	-1.00600095	0.073563931	-0.424715337	0.481274312	-1.430716287	11.55939759	11.29386853	12.38698767	10.79734056	10.8423598	11.61288715	11.15073956
ENSG00000133193	FAM104A	-0.102568997	0.885376787	-1.328475655	0.017716114	-1.431044652	7.975256505	8.862353853	9.301632639	8.693628619	8.72559535	7.53303322	7.470508284
ENSG00000176597	B3GNT5	0.242569915	0.89066231	-1.675116994	0.260870186	-1.432547079	2	6.311476754	5.59507254	5.686138681	5.70538658	4.220168345	3.875378318
	LOC441155	6.629128683	2.58E-09	-8.062274789	3.27E-05	-1.433146106	2	3.076665209	4.523675391	9.50916971	9.55926825	2	2
ENSG00000182400	TRAPPC6B	0.532177133	0.39788284	-1.966817605	0.000275322	-1.434640472	8.963744667	8.234834549	7.691296561	8.902248225	8.93264039	6.595545082	6.338680316
C2ORF82	C2ORF82	3.754287162	0.510049398	-5.191091137	0.364514502	-1.436803975	2	2	2	3.997744386	4.2865223	2	2
ENSG00000181481	RNF135	-1.060973779	0.159914848	-0.3770057	0.644088306	-1.437979479	9.435834703	8.169643011	7.858115942	7.638916932	7.5835711	8.478426859	8.059781405
ENSG00000125247	TMTC4	-0.829745383	0.737269626	-0.608647798	0.807781951	-1.438393181	3.327711234	8.053983481	2	6.006476466	5.48637102	6.197600957	5.713290392
ENSG00000183155	RABIF	-0.774001053	0.727489842	-0.664679952	0.765135376	-1.438681005	9.761125267	7.60566235	2	7.708923796	7.7127321	7.692773977	7.933467731
ENSG00000153574	RPIA	-0.508310456	0.83752462	-0.931078713	0.688544443	-1.439389169	2	4.442710129	8.608698767	6.634986713	6.60731796	6.361143723	6.059610627
ENSG00000146733	PSPH	-1.106136133	0.034068642	-0.333539415	0.562413083	-1.439675548	8.009475818	7.638155693	7.163363831	6.634986713	6.507263	7.55607473	7.036323724
ENSG00000076248	UNG	-0.711430401	0.458940186	-0.729159452	0.435504299	-1.440589853	7.218819781	7.315436877	9.116480286	7.474468681	7.45518898	7.307207714	7.575491915
ENSG00000198551	ZNF627	-0.736992151	0.259012717	-0.703752297	0.272931073	-1.440744448	7.33131396	8.213429689	8.585098385	7.527093191	7.28055563	7.688988646	7.142226902
ENSG00000185730	ZNF696	-1.206417215	0.150100354	-0.236414309	0.801869817	-1.442831524	7.384433967	6.389896633	5.59507254	5.304221918	5.6826301	6.617616961	6.172377306
ENSG00000167100	SAMD14	-1.703804007	0.164180702	0.25929675	0.853657176	-1.444507257	9.174465797	5.595927442	9.180874355	6.998639523	6.95464102	8.88183438	8.928657149
ENSG00000102103	PQBP1	-0.581297924	0.242935896	-0.863633598	0.06554197	-1.444931523	8.696033109	8.101365653	8.922968322	7.956842238	8.10384772	7.561260509	7.925444964
ENSG00000111652	COPS7A	-1.207752034	0.012083417	-0.237246178	0.672805615	-1.444998213	10.52923584	9.849218119	10.70548138	9.161399541	9.24080977	9.862322274	10.41907654
ENSG00000160972	PPP1R16A	-0.431265645	0.298315448	-1.013771771	0.006260318	-1.445037416	8.540903468	8.234834549	8.742726175	8.008651253	8.16464176	7.358152527	7.66011679
ENSG00000135678	CPM	-2.119571761	0.141954442	0.673008447	0.669265476	-1.446563314	6.366709671	4.93638933	7.225742779	4.783303513	4.22485516	7.944499874	4.729989799
ENSG00000099246	RAB18	-0.545024483	0.401480288	-0.90154789	0.13626007	-1.446572373	11.31110672	10.43440824	9.981704053	10.19294689	10.0858626	9.868610809	9.69686815
ENSG00000138468	SENP7	-0.892532448	0.14099447	-0.554276852	0.370202745	-1.446809299	7.031698319	7.669933291	6.718950348	6.442929658	6.25300433	6.90385696	6.400759785
ENSG00000132122	SPATA6	-0.678603675	0.735978187	-0.769194256	0.695554779	-1.447797931	2	7.051611315	4.523675391	5.058234798	5.21224859	5.085488617	5.028505473
ENSG00000164924	YWHAZ	-0.580887586	0.032660886	-0.867471222	0.000762931	-1.448358808	13.26936508	12.87899828	13.07845487	12.54471931	12.4609778	12.16436478	12.26796737
ENSG00000164117	FBXO8	-0.57968563	0.154817843	-0.868708389	0.023427954	-1.448394019	8.716893051	8.732943944	8.408111533	8.03841338	8.07574085	7.992893101	7.512619696
ENSG00000121075	TBX4	-0.422645388	0.878583032	-1.027103859	0.684198796	-1.449749246	3.327711234	3.076665209	2	2.600425781	2.86123014	2.803598391	2.172608095
ENSG00000180834	MAP6D1	-0.031101789	0.989593043	-1.419041335	0.494816556	-1.450143124	2	3.076665209	6.534109959	5.128039203	5.04390545	4.065262523	3.824516949
ENSG00000167720	SRR	-0.290034751	0.611381475	-1.161143055	0.017728145	-1.451177805	7.031698319	7.701025996	6.958202216	7.044488801	6.96410766	6.034159474	6.29575846
ENSG00000126787	DLGAP5	-0.769622348	0.659371222	-0.682174821	0.692464563	-1.451797168	3.327711234	5.595927442	3.369317972	4.070463721	3.82856379	3.193831101	4.543391831
ENSG00000101246	ARFRP1	-0.989362484	0.140578614	-0.46414343	0.513597151	-1.453505915	9.771132748	8.234834549	9.426401271	8.279879181	8.30907138	8.72431761	8.898748881
ENSG00000140386	SCAPER	-1.333650035	0.392352229	-0.119913384	0.945540243	-1.453563419	8.444143041	7.274681896	3.369317972	6.237202172	6.02777321	7.224637511	7.376535208
ENSG00000256683	ZNF350	-0.05048753	0.962823674	-1.404177641	0.120415896	-1.45466517	6.366709671	7.051611315	8.585098385	7.552702688	7.61995902	6.239610476	6.317379004
ENSG00000106399	RPA3	-0.339597249	0.845967999	-1.116215091	0.474892185	-1.45581234	6.563806279	5.946638857	2	5.474671438	5.39133044	4.888321757	4.594883674
ENSG00000103876	FAH	0.211613337	0.882991782	-1.667700467	0.16021677	-1.45608713	8.737455672	7.393647515	5.156626401	7.893113669	7.95274764	5.829320042	6.338680316
ENSG00000093134	VNN3	3.7349147	0.512728968	-5.191091218	0.364514502	-1.456176518	2	2	2	4.299492648	3.94787299	2	2
ENSG00000243480	AMY2A	3.7349147	0.512728968	-5.191091218	0.364514502	-1.456176518	2	2	2	4.299492648	3.94787299	2	2
ENSG00000148671	ADIRF	5.484290174	0.001294782	-6.94073151	0.0032699	-1.456441336	2	3.686303414	2	7.206526483	7.38291816	2	2
ENSG00000114923	SLC4A3	-1.953576601	0.064794697	0.496994794	0.675190643	-1.456581806	8.19931886	6.951736442	5.156626401	5.334065616	5.44640346	7.79139499	7.65527392
ENSG00000196290	NIF3L1	-0.981880931	0.634672806	-0.475018853	0.825446679	-1.456899784	9.189468652	7.538394143	2	7.101994243	6.99676009	7.598057803	7.488317792
ENSG00000165416	SUGT1	-0.503973745	0.555006373	-0.95304876	0.228768931	-1.457022504	9.635285994	8.147241016	7.97167868	8.23312837	8.34755546	7.948463298	7.742336888
ENSG00000197008	ZNF138	-0.202166378	0.912234522	-1.255968314	0.428993571	-1.458134692	6.73718411	6.228547981	2	5.839660368	5.69405321	5.04460184	4.56420962
ENSG00000100028	SNRPD3	-0.517787483	0.303181423	-0.941101226	0.042409585	-1.458888708	10.29268328	9.488434476	10.11127249	9.453327884	9.51647861	8.799092094	9.289039876
ENSG00000145685	LHFPL2	-0.217390226	0.738608861	-1.242864596	0.023412451	-1.460254822	10.82178169	11.2913169	11.88421475	11.20190521	11.1611534	9.780867042	10.45800828
ENSG00000105699	LSR	-0.720705481	0.610159623	-0.739801544	0.591655943	-1.460507025	4.815609471	6.464272742	3.369317972	4.907595604	4.68029874	4.5389195	4.990558504
	DOPEY1	5.654690303	0.001390334	-7.116254018	0.003086571	-1.461563716	2	2	4.058998049	7.656739692	7.61395775	2	2
ENSG00000116030	SUMO1	-0.400867737	0.516230128	-1.061288523	0.053149142	-1.46215626	10.9638027	9.806969599	10.10298738	10.01956245	9.93396767	9.241683432	9.394877047
ENSG00000115524	SF3B1	-0.476136794	0.512666787	-0.986575183	0.136166444	-1.462711977	11.25159303	12.86276311	12.26789926	11.78444742	11.8053473	11.31623198	11.25345031
ENSG00000149923	PPP4C	-0.739347384	0.556147109	-0.723812487	0.559807045	-1.463159871	11.65437133	9.648522413	8.380957451	9.783105971	9.75261615	9.646061941	9.908803154
ENSG00000198929	NOS1AP	-2.326272487	0.374953074	0.862118573	0.751485356	-1.464153914	2	4.93638933	2	2.868578001	2.14022021	4.5389195	4.215171317
ENSG00000105968	H2AFV	-1.49748547	2.86E-09	0.032261687	0.92532897	-1.465223783	10.01801709	9.71095217	9.963514551	8.388752656	8.43865202	9.864840978	9.997333704
ENSG00000109184	DCUN1D4	-0.290397939	0.711436164	-1.17512651	0.083368021	-1.465524449	8.228665467	9.672251278	9.839340503	9.143603603	9.087919	8.25342909	8.226587404
ENSG00000109814	UGDH	-0.949179364	0.182358952	-0.517074894	0.485029668	-1.466254258	11.74347718	11.29641566	10.29705308	10.29273067	10.2722069	10.21173728	11.08495917
ENSG00000164610	RP9	-0.255713334	0.813586965	-1.212553702	0.191291972	-1.468267036	8.042902253	6.140560021	6.203069917	6.79375511	6.8715633	6.05195199	5.781546943
ENSG00000181458	TMEM45A	-0.581751928	0.458819694	-0.886880763	0.228318728	-1.468632691	8.87372952	9.890264534	10.67226762	9.425008328	9.39487575	9.023457772	9.185775709
ENSG00000134253	TRIM45	-1.477220988	0.133069745	0.007867964	0.994638415	-1.469353024	7.031698319	5.303523747	5.39244166	4.623314311	4.95174433	6.468821191	5.717942682
ENSG00000234465	PINLYP	0.353675172	0.898417957	-1.823203018	0.452416189	-1.469527846	8.631580145	3.076665209	2	7.281195074	7.57123495	5.274160001	5.459179378
ENSG00000243678	NME2	2.996174115	2.62E-08	-4.46594169	2.08E-16	-1.469767575	8.257427002	6.899080544	7.646393253	10.6638047	10.6679683	3.632645806	3.807156736
ENSG00000198015	MRPL42	-0.813537967	0.211593503	-0.658880454	0.312110723	-1.472418421	8.418903792	9.298208073	9.897982195	8.538855014	8.49850989	8.740408958	8.5998903
ENSG00000186074	CD300LF	-3.753025058	NA	2.280545605	NA	-1.472479453	2	2	2	2	2	3.56816602	2.592996859
ENSG00000150337	FCGR1A	-3.753025059	NA	2.280455726	NA	-1.472569333	2	2	2	2	2	3.393105702	2.885414003
ENSG00000100379	KCTD17	-0.901036234	0.184561268	-0.573055838	0.410673825	-1.474092072	8.674867122	7.638155693	9.165043284	7.656739692	7.79460185	7.935741825	8.155488004
ENSG00000164520	RAET1E	0.744135918	0.704833394	-2.218980077	0.210711023	-1.474844159	3.327711234	3.076665209	4.523675391	4.358765249	4.2865223	2.139668783	2.950113106
ENSG00000136098	NEK3	-1.132339128	0.514610732	-0.342709505	0.853576247	-1.475048633	7.33131396	6.844429755	2	5.433909581	5.53799497	6.398709156	6.018717758
ENSG00000150756	FAM173B	-1.212035397	0.33586874	-0.26369942	0.850222943	-1.475734816	7.579279349	5.595927442	4.523675391	5.334065616	5.30456641	6.000958775	6.368575325
ENSG00000141428	C18orf21	-0.501521848	0.579491849	-0.974293965	0.243352449	-1.475815813	8.19931886	6.535001748	6.718950348	6.86703417	6.86146731	6.609629839	6.179140936
ENSG00000118898	PPL	-3.359310002	0.069933875	1.882931046	0.267661148	-1.476378956	3.327711234	4.442710129	2	2.270786764	2.2680111	4.291799915	5.661099865
ENSG00000163902	RPN1	-0.336938743	0.464545264	-1.140550693	0.004152549	-1.477489436	13.07503738	12.29271883	12.51583706	12.31382177	12.3448457	11.46661987	11.58427143
ENSG00000007038	PRSS21	-3.75302512	NA	2.275517957	NA	-1.477507164	2	2	2	2	2	3.411599105	2.851942824
ENSG00000136014	USP44	-3.524175504	0.099060337	2.044396167	0.2908151	-1.479779337	4.00722531	3.686303414	2	2.3892346	2	5.338397457	4.711446447
ENSG00000166226	CCT2	-0.495386419	0.434185114	-0.985971139	0.088712251	-1.481357558	11.19324461	9.869887299	10.68897008	10.15895941	10.2133886	9.598679043	9.790665867
ENSG00000197062	ZSCAN26	-0.939520837	0.643088643	-0.541975076	0.795268255	-1.481495912	8.963744667	8.077869079	2	7.053486362	7.12888795	7.775419242	7.105417192
ENSG00000136695	IL36RN	3.709533236	0.51607452	-5.191091323	0.364514502	-1.481558088	2	2	2	3.79808662	4.37434574	2	2
ENSG00000198719	DLL1	4.671220287	0.046826482	-6.1529404	0.031054949	-1.481720113	3.327711234	2	2	5.94010643	5.5755357	2	2
ENSG00000115128	SF3B6	-1.022274183	0.074775117	-0.462745207	0.4484575	-1.48501939	10.22876205	9.079034257	9.287079172	8.567402595	8.64401667	9.236830239	9.080880746
ENSG00000100142	POLR2F	-0.487974879	0.487504654	-0.997945978	0.11985978	-1.485920857	8.998234871	7.979858146	7.691296561	7.849869274	7.85944752	7.155000534	7.531242162
ENSG00000116396	KCNC4	-0.983417547	0.657356622	-0.504081051	0.826586773	-1.487498598	2	6.535001748	9.227351203	6.917187326	6.87658492	7.789628618	6.753261212
ENSG00000100711	ZFYVE21	-0.990488931	0.002467749	-0.499913486	0.148716503	-1.490402417	8.631580145	9.030900487	8.677268317	7.834291698	7.80517502	8.199905499	8.399447278
ENSG00000121940	CLCC1	-0.512170113	0.725856392	-0.978992381	0.473680232	-1.491162494	6.563806279	8.862353853	10.68342405	8.948803189	9.07159009	8.53487306	8.562873254
ENSG00000171566	PLRG1	-0.281395807	0.85537103	-1.211740181	0.367297237	-1.493135989	5.70932728	9.806969599	9.868959282	8.999733249	9.03050377	8.162859687	8.023598593
ENSG00000100365	NCF4	-1.689351372	0.585157799	0.196190376	NA	-1.493160996	2	2	3.369317972	2.141737246	2.2680111	2.775316226	2.509591799
ENSG00000181690	PLAG1	-0.94318561	0.632299403	-0.550935149	0.786091427	-1.494120759	2	8.077869079	7.285536013	6.336376195	6.18176397	7.157741966	5.807970301
ENSG00000161544	CYGB	1.790440909	0.121386739	-3.285133478	0.002227172	-1.494692569	5.330594134	7.901717006	7.163363831	8.873322599	8.9398551	4.742549839	3.236138336
ENSG00000180376	CCDC66	-0.214800383	0.832111917	-1.280732508	0.130070296	-1.495532891	6.009025997	7.669933291	7.398165132	6.989292186	6.97819215	6.405645065	5.331426166
ENSG00000141378	PTRH2	-0.827347491	0.475887749	-0.668735638	0.56354884	-1.49608313	9.810482605	7.35507212	7.451345288	7.870381136	7.86703672	7.852739912	8.178454506
ENSG00000173992	CCS	-1.06872093	0.080136816	-0.427404128	0.513742625	-1.496125058	7.485143553	8.317412869	7.098165388	6.610808945	6.78340763	7.421115854	7.213143202
ENSG00000148985	PGAP2	-1.08788745	0.41446225	-0.408473555	0.773867441	-1.496361005	4.467311984	8.147241016	8.266922072	6.70517998	6.53293476	7.393470826	7.154291015
ENSG00000204519	ZNF551	-1.143274248	0.484587476	-0.354255952	0.840156112	-1.4975302	3.327711234	5.946638857	8.110401118	5.85002558	5.6826301	6.562825444	6.460277802
ENSG00000160446	ZDHHC12	-0.752761006	0.391821726	-0.746948385	0.384717028	-1.499709391	9.678463567	8.557180776	7.600047325	8.004017475	8.21109983	8.046387837	8.184349581
ENSG00000158966	CACHD1	0.614358526	0.762344209	-2.114546499	0.240936153	-1.500187973	2	8.004992147	8.699419242	8.404661252	8.44034872	5.815498119	5.75463059
ENSG00000088682	COQ9	-0.844264512	0.710858197	-0.655956933	0.774684899	-1.500221445	10.00110988	7.503546079	2	7.895617537	7.74603955	8.085066106	7.926450253
ENSG00000106009	BRAT1	-0.882232392	0.172591839	-0.618282231	0.34684373	-1.500514623	7.975256505	8.88960062	9.413059136	7.956842238	8.04484669	8.408791501	8.097758666
ENSG00000100290	BIK	-0.46395321	0.884340692	-1.036757009	NA	-1.500710219	2	2	3.369317972	2.498691189	2.38539787	2.34604626	2.277179783
ENSG00000167487	KLHL26	-0.683026678	0.749679501	-0.818284099	0.695197299	-1.501310777	2	7.467835385	9.180874355	7.398825229	7.23044321	7.009312024	7.342293759
ENSG00000110717	NDUFS8	-0.886082181	0.192239251	-0.615312951	0.37239741	-1.501395132	10.20680973	9.792607274	8.784770914	8.770670063	8.88484424	8.742693141	9.382110089
ENSG00000127125	PPCS	-1.050015005	0.13450058	-0.451884141	0.545019112	-1.501899146	9.174465797	8.124485672	9.798878489	8.143273718	8.14188248	8.787633372	8.677773268

ENSG00000084754	HADHA	-0.496525205	0.192239251	-1.005578357	0.003786604	-1.502103562	11.29385609	11.15761406	11.73357624	10.91339224	10.9261795	10.27622952	10.53615161
ENSG00000140839	CLEC18B	1.705091093	0.505493848	-3.207345203	0.193871288	-1.502254109	2	2	4.058998049	3.663521627	4.40247105	2.225793514	2.059860178
ENSG00000275499		3.688784418	0.518691015	-5.19109141	0.364514502	-1.502306992	2	2	2	4.034562184	4.16043394	2	2
ENSG00000141759	TXNL4A	-1.271934671	0.002781499	-0.230696277	0.644509054	-1.502630949	9.895281535	9.695596167	10.44204362	8.846395982	8.71020716	9.606476037	9.996137262
ENSG00000131013	PPIL4	-0.453831438	0.713282111	-1.050125704	0.352922604	-1.503957142	9.544849385	9.102512015	6.322058544	8.409925372	8.37797443	7.968899389	7.623392692
ENSG00000125166	GOT2	-0.906739182	0.29492176	-0.597219871	0.498114559	-1.503959053	11.13631304	10.3729879	8.941672936	9.486925632	9.51808625	9.735024004	9.882366758
ENSG00000153561	RMND5A	-0.716052345	0.274396109	-0.788300626	0.215146032	-1.504352971	8.836089686	9.741182361	8.536706852	8.369067528	8.48542112	8.611362196	8.047821014
ENSG00000125962	ARMCX5	-0.659193896	0.681796824	-0.845337078	0.584335308	-1.504530975	4.00722531	6.78762695	8.65477198	6.777556219	6.86146731	6.697032326	6.582657467
ENSG00000141447	OSBPL1A	-1.238172128	0.000876231	-0.269517126	0.529270693	-1.507689254	8.816894989	8.540087354	9.165043284	7.497728447	7.76240929	8.5805365	8.600520269
ENSG00000103522	IL21R	-0.27649774	0.881480919	-1.23149971	0.444246609	-1.50799745	4.467311984	2	4.874587708	3.79808662	4.0930011	3.12825509	3.474731315
ENSG00000196177	ACADSB	-1.029774972	0.311246056	-0.478242461	0.656134931	-1.508017434	8.75772933	8.004992147	6.322058544	7.003290579	6.99214046	6.939340974	7.955303035
ENSG00000125734	GPR108	-0.22548368	0.658522427	-1.2831875	0.002463508	-1.50867118	10.20680973	9.338312924	9.788583153	9.544700162	9.64121862	8.435582605	8.645170029
ENSG00000177042	TMEM80	-1.858223319	0.063207012	0.349021993	0.761747773	-1.509201326	6.96351253	8.540087354	9.767769415	7.003290579	7.01053089	9.444727462	8.851897333
ENSG00000105193	RPS16	-1.281089022	0.01776689	-0.229430473	0.717035549	-1.510519496	11.98187803	11.0620559	10.85110517	10.04346859	10.1695649	11.11952254	11.19322749
ENSG00000113369	ARRDC3	0.835550045	0.367367297	-2.346301935	0.004207821	-1.51075189	8.16936288	10.2574711	10.40208303	10.75866452	10.7065491	7.354572674	7.77172059
ENSG00000079482	OPHN1	-1.342147925	0.041012176	-0.169669794	0.827324651	-1.511817718	8.75772933	7.232742069	7.97167868	6.861921612	6.73998187	7.938932725	7.952344882
ENSG00000106603	COA1	-1.098712843	0.172176659	-0.413178828	0.633693854	-1.511891671	9.761125267	8.469613413	7.934808822	7.875463856	7.81828345	8.350077895	8.677773268
ENSG00000142039	CCDC97	-1.037416179	0.268404698	-0.474955777	0.631857848	-1.512371957	6.96351253	8.213429689	9.344432377	7.461007055	7.45518898	7.858649102	8.146888246
ENSG00000167748	KLK1	-3.753025559	NA	2.240376398	NA	-1.512649161	2	2	2	2	2	3.355392153	2.885414003
ENSG00000121390	PSPC1	-0.268697088	0.561781402	-1.244586815	0.001446259	-1.513283903	9.204317094	8.522788974	8.585098385	8.496596021	8.57309694	7.569519177	7.57933192
ENSG00000052126	PLEKHA5	-0.378322239	0.784875307	-1.135866233	0.360441295	-1.514188472	6.009025997	9.225224669	9.798878489	8.616799622	8.66593596	7.912394292	7.875307424
ENSG00000060339	CCAR1	-0.070807413	0.932860886	-1.444113969	0.03239564	-1.514921383	8.564112515	10.07368926	10.16000817	9.656695015	9.72225407	8.433321781	8.213468956
ENSG00000115884	SDC1	-1.108062972	0.125657681	-0.406904112	0.603099089	-1.514967085	9.317904228	8.169643011	7.734844356	7.501020868	7.45182838	8.301943231	8.014172904
ENSG00000109787	KLF3	-0.802655777	0.113360191	-0.712517264	0.156916815	-1.515173041	8.87372952	9.591588447	9.926432745	8.76109026	8.69891174	8.877267588	8.759585072
ENSG00000101871	MID1	-0.466563266	0.535988719	-1.050102332	0.125781284	-1.516665597	9.159305284	8.191702463	9.227351203	8.501535708	8.42671895	7.223329552	8.343344553
ENSG00000154277	UCHL1	1.197562714	0.11571852	-2.714450969	7.98E-05	-1.516888254	12.2287862	10.52407288	10.98787477	12.60218578	12.6580329	8.450751194	8.965802436
ENSG00000068308	OTUD5	-1.206015961	0.145426901	-0.31108441	0.735231663	-1.517100371	10.46903009	9.246456519	11.38635888	9.36574218	9.45570025	10.30927629	10.29555071
ENSG00000057608	GDI2	-0.810529571	0.033905673	-0.707352429	0.065229023	-1.517882001	12.08576437	11.86868155	11.36226358	10.97949959	11.0082583	11.06132633	11.13168895
ENSG00000196724	ZNF418	-0.925398621	0.531161556	-0.593149232	0.694164121	-1.518547853	3.327711234	7.145017454	5.59507254	5.003571641	5.41912973	5.762453498	5.243813244
ENSG00000083290	ULK2	-1.438061174	0.014508855	-0.081431294	0.911833873	-1.519492468	8.107517749	8.747909548	9.491321578	7.412876874	7.53039799	8.789844052	8.828963952
ENSG00000144535	DIS3L2	-0.677730176	0.466156888	-0.84237625	0.34391267	-1.520106426	8.696033109	7.701025996	6.534109959	7.218576229	7.24992376	6.824993504	7.287011591
ENSG00000131747	TOP2A	-0.870834295	0.691466494	-0.649960397	0.768665969	-1.520794692	5.532353208	8.469613413	2	6.542151749	5.87556634	5.963846589	6.824016918
ENSG00000138160	KIF11	-0.826752313	0.629335817	-0.694261571	0.685433873	-1.521013883	4.815609471	7.60566235	3.369317972	5.709096143	5.3193943	5.646229369	5.651404445
ENSG00000117461	PIK3R3	0.438651746	0.616855364	-1.959697256	0.008669082	-1.52104551	6.257048221	7.847167909	7.896971941	7.956842238	7.92150349	5.22272385	5.96101835
ENSG00000089234	BRAP	-0.313432194	0.807472212	-1.208002059	0.283292274	-1.521434253	6.816637977	8.903033433	10.11951029	8.85414072	8.82115545	8.050818921	7.850099615
ENSG00000184678	HIST2H2BE	1.251191287	0.322921865	-2.773344459	0.016029651	-1.522153172	9.829762239	8.469613413	6.629488387	10.05695346	10.0864045	5.085488617	6.725806822
ENSG00000129691	ASH2L	-0.809502457	0.725609862	-0.712720593	0.757049561	-1.522223051	10.5468183	8.540087354	2	8.496596021	8.46889137	8.512023373	8.642113606
ENSG00000113558	SKP1	-0.203023344	0.591297391	-1.31971002	1.88E-05	-1.522733364	11.25872341	11.65110249	11.78885549	11.38768406	11.3734127	10.15695659	10.36819284
ENSG00000213639	PPP1CB	-0.406891588	0.40149941	-1.11598792	0.009399663	-1.522879508	10.71696191	11.32663546	11.5927499	10.91524435	10.7827547	10.24289218	10.03650256
ENSG00000136930	PSMB7	-0.213376017	0.665819919	-1.310253853	0.001254829	-1.523629869	11.22271378	10.60026656	10.48092713	10.54402725	10.6398176	9.361563842	9.626546639
ENSG00000108963	DPH1	0.068801649	0.909699221	-1.59333673	0.000463383	-1.524535081	9.015175615	8.297207175	9.301632639	8.998569022	8.98472903	7.377096489	7.330190599
ENSG00000100083	GGA1	-0.783253417	0.282216889	-0.741732146	0.300571978	-1.524985563	9.48530526	8.57407404	10.26027233	8.782894215	8.84952549	8.837641184	8.877593894
ENSG00000034713	GABARAPL2	-0.715646633	0.515922264	-0.810088591	0.447054694	-1.525735224	11.51244431	8.929529568	9.601288346	9.750280081	9.71456265	9.693591907	9.58130758
ENSG00000160087	UBE2J2	-1.090020813	0.219962273	-0.43575758	0.647499233	-1.525778393	10.72727936	9.067150531	8.805342542	8.70652031	8.73115061	9.100282869	9.594016939
ENSG00000160888	IER2	-0.061436621	0.939941719	-1.466000871	0.024374582	-1.527437492	9.2903348	9.461450882	10.53119344	9.822457304	9.79566886	8.751793877	7.974870548
ENSG00000143774	GUK1	-0.763172071	0.08383018	-0.765000872	0.078125458	-1.528172943	11.38138789	10.58784379	11.34831197	10.36045596	10.4119259	10.34308917	10.42495703
ENSG00000160447	PKN3	-1.882949173	0.229349511	0.354401046	0.840156112	-1.528548127	2	5.838937808	6.718950348	3.997744386	4.31639476	6.146071859	6.102937566
ENSG00000145439	CBR4	-0.737664447	0.096461716	-0.79118843	0.065559433	-1.528852877	7.579279349	7.572420273	7.97167868	6.984595707	7.00595521	7.137053113	6.718860807
ENSG00000029639	TFB1M	-0.4082241	0.818265925	-1.120703993	0.484174515	-1.528928094	8.716893051	4.11352228	6.073378139	6.979883891	7.01053089	6.423979439	6.189227296
ENSG00000130520	LSM4	-1.210506601	0.043308828	-0.31888359	0.635737899	-1.529390191	10.3933013	9.00621767	9.735974718	8.627383409	8.60370266	9.410081891	9.585450415
ENSG00000125319	C17orf53	-0.458279944	0.796259474	-1.07111521	0.509775204	-1.529395154	6.563806279	5.946638857	2	5.194621444	5.44640346	4.350488971	5.122735884
ENSG00000127184	COX7C	-1.095890149	0.172471782	-0.434308518	0.614417433	-1.530198667	10.94624409	9.298208073	9.330306409	9.009013399	8.96835847	9.679115027	9.612561218
ENSG00000120798	NR2C1	-0.40937927	0.550957372	-1.120862181	0.067400343	-1.530241451	7.435667355	8.451443573	8.784770914	8.008651253	7.83643742	7.4096614	7.021346309
ENSG00000133398	MED10	-0.540072041	0.670923419	-0.990279311	0.402364322	-1.530351352	9.781071289	8.639735838	6.322058544	8.268332738	8.30907138	7.896069536	7.792821043
ENSG00000164548	TRA2A	-0.289058478	0.785196727	-1.241436909	0.175908868	-1.530495388	10.30651143	8.213429689	8.511888463	9.084476714	8.99284518	7.8927823	8.276323713
ENSG00000011465	DCN	-1.300564051	0.000196745	-0.230972622	0.57665812	-1.531536673	15.07579791	14.52772935	14.69777459	13.48165944	13.4889246	14.33331165	14.74646283
ENSG00000143499	SMYD2	-1.20383463	0.000526024	-0.329051721	0.400109235	-1.532886352	9.097018025	8.820493869	8.784770914	7.77569021	7.65837038	8.360824221	8.771382398
ENSG00000115073	ACTR1B	-1.724873193	0.006218119	0.191814705	0.802148981	-1.533058487	9.820154628	8.57407404	10.01740919	7.862723323	7.90685167	9.524685263	10.00306286
ENSG00000101361	NOP56	-0.458244792	0.498614729	-1.07491066	0.078357385	-1.533155452	10.49341471	9.125613817	9.577565424	9.401417034	9.37815424	8.741323065	8.813288819
ENSG00000105726	ATP13A1	-0.241074372	0.712294566	-1.293141582	0.018856875	-1.534215953	10.70657014	10.11467741	9.426401271	9.889939704	9.97843848	8.793374109	8.978944023
ENSG00000151287	TEX30	-0.685556728	0.822912318	-0.850474766	0.774493502	-1.536031493	7.82963488	2	2	5.775854763	5.51241389	5.870008604	4.998228167
ENSG00000125977	EIF2S2	-0.664128936	0.182039968	-0.872007296	0.066689274	-1.536136232	11.6462383	10.74528399	10.87563042	10.49120048	10.4749506	10.33765955	10.2112406
ENSG00000085733	CTTN	-0.575420157	0.225627059	-0.961289951	0.030397832	-1.536710109	12.31510994	12.10496808	12.71516972	11.83244654	11.8189434	11.05646846	11.74325939
ENSG00000008382	MPND	-1.099671906	0.528056559	-0.437169822	0.811488554	-1.536841728	7.159102577	6.140560021	2	4.927308402	5.37722731	5.528313928	5.980509018
ENSG00000105185	PDCD5	-0.147994077	0.762607571	-1.389288388	0.000378379	-1.537282465	8.910412251	8.234834549	8.941672936	8.538855014	8.61275919	7.346184975	7.364717093
ENSG00000165280	VCP	-0.78814434	0.069078369	-0.751508703	0.080413293	-1.539653043	13.09907181	12.65418243	12.49382591	11.97939826	11.9900129	11.70033342	12.28374298
ENSG00000172007	RAB33B	-1.406044698	0.04814826	-0.136530118	0.872932235	-1.542574816	6.816637977	8.234834549	8.076950877	6.496695859	6.44777477	8.042684843	7.258555286
ENSG00000105700	KXD1	-0.61331871	0.240254037	-0.929314931	0.058802978	-1.542633641	10.15425007	9.842262059	9.132850258	9.137269904	9.18421833	8.701014095	8.980396837
ENSG00000166428	PLD4	-3.753025943	NA	2.209644739	NA	-1.543381204	2	2	2	2	2	3.465698119	2.671842496
ENSG00000120837	NFYB	-1.305834681	0.335280148	-0.240727325	0.874489793	-1.546562006	8.963744667	8.053983481	4.874587708	6.820355514	6.72891807	8.171023934	7.334741191
ENSG00000126267	COX6B1	-0.771415386	0.189075569	-0.775325282	0.178213485	-1.546740669	10.79750658	9.558016677	10.11127249	9.435370781	9.51325794	9.35261874	9.580349849
ENSG00000076043	REXO2	-0.663142779	0.417114533	-0.88385748	0.25649001	-1.547000258	11.05263482	9.695596167	9.31604076	9.549478128	9.57865651	9.399697583	9.287475811
ENSG00000176731	C8orf59	-0.289203395	0.654150801	-1.257925748	0.023011611	-1.547129143	7.940205782	6.78762695	7.646393253	7.2696592	7.21069602	6.205571486	6.403650353
C4ORF22	C4ORF22	3.64349023	0.524450905	-5.191091598	0.364514502	-1.547601368	2	2	2	4.070463721	4.05806467	2	2
ENSG00000163013	FBXO41	-0.664050218	0.733353042	-0.883593821	0.639142933	-1.547644039	2	6.78762695	7.97167868	6.164317909	6.40670796	6.320128742	5.799216178
ENSG00000167595	PROSER3	-1.504591278	0.284673982	-0.04387781	0.978224387	-1.548469089	5.330594134	5.457120953	8.561105505	5.968925393	5.65950892	7.20886319	7.224630935
ENSG00000175084	DES	-4.343113373	0.000228835	2.793790816	0.017043752	-1.549322557	4.00722531	6.389896633	7.342949392	2.868578001	3.15372745	8.285116882	9.732652955
ENSG00000125485	DDX31	-0.611843019	0.766447439	-0.939274038	0.633808927	-1.551117057	2	7.051611315	8.561105505	6.887305128	6.76185814	6.708295923	6.283256815
ENSG00000136273	HUS1	-0.479326724	0.721229196	-1.073205031	0.384149032	-1.552531755	9.556468883	6.899080544	6.718950348	7.865280446	7.88209639	7.138441638	7.431385914
ENSG00000139910	NOVA1	-2.320477225	0.005058045	0.766312867	0.379558392	-1.554164358	6.138360429	4.93638933	5.156626401	3.840289928	3.33976443	6.028179536	6.365613546
ENSG00000141741	MIEN1	-1.034735058	0.198785891	-0.519481132	0.538306766	-1.55421619	10.07567912	8.213429689	8.960138144	8.283707551	8.23475766	8.530649266	8.973604485
ENSG00000118690	ARMC2	-0.916760786	0.649972951	-0.638191759	0.754844937	-1.554952545	6.563806279	3.686303414	2	4.387512679	4.45712836	4.727866721	4.574506939
ENSG00000132321	IQCA1	-2.976211788	0.367097793	1.419422184	0.655105561	-1.556789604	2	3.686303414	2	2	2.2680111	3.709392871	3.600986722
ENSG00000005156	LIG3	-1.17583695	0.002482135	-0.380992218	0.37369315	-1.556829168	8.444143041	7.790474954	8.236946975	6.946463121	7.09482005	7.797560316	7.799420735
ENSG00000112308	C6orf62	-0.262200101	0.537440375	-1.294785324	0.000280988	-1.556985426	11.34834729	11.72481431	12.03516535	11.50956605	11.424668	10.32567245	10.54091836
ENSG00000161929	SCIMP	-1.476497549	0.433973734	-0.081541656	0.968570087	-1.558039206	3.327711234	4.11352228	2	2.695456494	2.68924543	3.648325314	3.071407821
ENSG00000186684	CYP27C1	-3.538553439	0.002397074	1.980122694	0.05519677	-1.558430745	3.327711234	4.11352228	5.156626401	2.3892346	2.49394783	5.985611216	6.254725615
ENSG00000141499	WRAP53	-0.773911016	0.691746353	-0.786555731	0.683005276	-1.560466748	2	6.78762695	8.110401118	6.449761039	6.08962627	6.192262702	6.341697848
ENSG00000103021	CCDC113	-1.676310401	0.065038902	0.115720952	0.914753421	-1.560589449	4.815609471	6.228547981	5.59507254	4.237680128	4.22485516	6.192262702	5.217771644
ENSG00000175203	DCTN2	-0.944028635	0.000672752	-0.618412546	0.032293519	-1.562441181	10.7426185	10.77115258	10.41552659	9.678668908	9.74577157	9.934835676	10.13219057
ENSG00000151882	CCL28	-1.893647614	0.121412754	0.330317647	0.807192459	-1.563329967	5.095959458	3.076665209	5.156626401	3.462030052	3.08597292	4.705557986	5.1574003
ENSG00000135457	TFCP2	-0.757422699	0.724832375	-0.80631297	0.702841297	-1.56373567	2	8.806266215	9.589475645	7.959237999	7.88459112	7.815899769	7.930464419
ENSG00000111669	TPI1	-1.127406666	0.002169292	-0.437410043	0.278589089	-1.56481671	13.67917225	13.35152472	13.71677814	12.44482528	12.4839871	12.81250915	13.43018847
ENSG00000145191	EIF2B5	-0.804637358	0.201926325	-0.760673233	0.221569388	-1.565310591	8.367060731	9.079034257	9.757248847	8.442826028	8.31092742	8.336833852	8.502136032
ENSG00000070423	RNF126	-1.041649651	0.219156141	-0.524957525	0.554505321	-1.566607176	10.58135316	9.04308532	8.825624954	8.616799622	8.73115061	8.860117405	9.454284815
ENSG00000117009	KMO	-1.689389614	0.594639663	0.119757502	NA	-1.569632112	2	2	3.369317972	2.141737246	2.2680111	2.831336763	2.374681325
ENSG00000109113	RAB34	-0.989161904	0.172780449	-0.580837262	0.439051343	-1.569999166	11.32476023	11.13502303	9.681375057	9.905552176	9.87371334	10.08262707	10.48282173
ENSG00000132170	PPARG	-3.627929422	0.014454932	2.057192496	0.133507552	-1.570736925	2	5.131603625	4.058998049	2.270786764	2.49394783	6.019162896	6.124122661
ENSG00000137171	KLC4	-1.079102505	0.605635767	-0.493149075	0.8207453	-1.572251581	9.460782017	7.954278496	2	7.242377933	7.27295842	7.943705881	7.708840864
ENSG00000182393	IFNL1	3.618567051	0.52739221	-5.191091702	0.364514502	-1.572524651	2	2	2	3.840289928	4.22485516	2	2
ENSG00000106346	USP42	-0.646391046	0.459554196	-0.928964617	0.259642722	-1.575355663	7.384433967	8.862353853	9.439621147	8.078634414	8.21705076	8.035995406	7.685278529
ENSG00000144744	UBA3	-0.46264031	0.066039024	-1.11279563	8.97E-07	-1.57543594	9.740899611	9.532315021	9.636156974	9.203437087	9.14937381	8.548515606	8.515559017
ENSG00000162241	SLC25A45	-0.501689755	0.784300093	-1.074075288	0.525858337	-1.575765043	3.327711234	7.73146271	4.874587708	6.096288482	5.74985018	5.125248504	5.641643428
ENSG00000177432	NAP1L5	-1.099016961	0.2546055	-0.477191524	0.640791054	-1.576208485	7.82963488	5.946638857	8.35328246	6.670510205	6.53928196	6.975527754	7.407104868
ENSG00000170445	HARS	-0.340930011	0.509715065	-1.235474309	0.005486623	-1.57640432	10.34721797	9.71095217	9.926432745	9.689531608	9.6675326	8.42082352	9.084037526
ENSG00000197905	TEAD4	-3.045366003	0.085712737	1.46837253	0.431244286	-1.576993474	8.313285031	6.666836837	2	4.205751501	4.50979031	7.907516231	9.041986855
ENSG00000006453	BAIAP2L1	-2.240292079	0.000642562	0.662773446	0.364514502	-1.577518634	8.540903468	7.761270542	6.882775709	5.720439317	5.73886209	8.681620276	8.372406522

ENSG00000132300	PTCD3	-0.435290779	0.17053846	-1.142801875	4.85E-05	-1.578092653	9.689059027	9.591588447	9.272377397	9.109497937	9.08575246	8.335018389	8.456236734
ENSG00000123268	ATF1	-0.432699073	0.70815223	-1.146098997	0.271141542	-1.57879807	6.816637977	7.189546432	9.227351203	7.614805492	7.83643742	7.107581047	6.955898408
ENSG00000123545	NDUFAF4	-0.622473606	0.459212556	-0.958363308	0.222598244	-1.580836914	7.904282243	6.228547981	7.896971941	6.912249745	6.90635305	6.763331418	6.38329369
ENSG00000172239	PAIP1	0.208741726	0.688378388	-1.790387884	1.65E-05	-1.581646158	9.730679447	9.599860728	8.941672936	9.654479106	9.69052657	7.874567387	7.488317792
ENSG00000183597	TANGO2	-0.028212384	0.989759352	-1.554498541	0.412109699	-1.582710925	10.16187685	3.686303414	6.718950348	8.622857004	8.75316018	7.24282551	7.133547492
ENSG00000160075	SSU72	-0.96190806	0.128533462	-0.622094426	0.338976671	-1.584002485	11.31794963	10.27823701	9.981704053	9.657432896	9.71526357	10.05624005	9.993021861
ENSG00000136240	KDELR2	-0.990308687	0.021365645	-0.593811641	0.186486326	-1.584120328	13.33577055	12.72774099	12.5282657	11.92359756	11.9094643	12.18307138	12.43161868
ENSG00000165506	DNAAF2	-0.995616896	0.371190997	-0.588779591	0.607699942	-1.584396488	8.418903792	6.899080544	5.59507254	6.422239259	6.46787796	6.961475607	6.697819825
ENSG00000134824	FADS2	-1.864043699	0.038357022	0.278389614	0.79369186	-1.585654084	13.04132235	10.81444733	12.64414369	10.5575302	10.5925339	12.01712251	13.1828323
ENSG00000103502	CDIPT	-0.887887772	0.232682693	-0.698227105	0.351780632	-1.586114878	11.59645304	10.03760521	10.27509734	9.840113674	10.0039039	10.05164138	10.17236288
ENSG00000153291	SLC25A27	-0.758270085	0.416637582	-0.82840854	0.353010925	-1.586678625	4.467311984	4.710564154	5.59507254	4.387512679	4.31639476	4.406883583	4.175132639
ENSG00000186638	KIF24	-1.095436761	0.617500397	-0.49370262	0.829885014	-1.589139381	3.327711234	6.78762695	2	4.470469939	4.43005853	4.434275199	5.343519008
ENSG00000101546	RBFA	-1.08429857	0.218893333	-0.507955978	0.583582174	-1.592254548	6.366709671	8.169643011	6.629488387	6.268449691	6.25300433	6.645228724	6.96179415
ENSG00000145741	BTF3	-0.968552646	0.205806676	-0.62502701	0.426712094	-1.593579655	11.98832668	10.13189344	11.05383672	10.27841188	10.2892535	10.56905757	10.6815274
ENSG00000178537	SLC25A20	-0.848717697	0.647476405	-0.74512894	0.687731208	-1.593846637	3.327711234	7.761270542	9.61300511	7.571615825	7.5462416	7.505319234	7.800517754
ENSG00000130005	GAMT	-1.371363454	0.17057763	-0.22318916	0.845529489	-1.594552615	8.981092835	8.862353853	6.322058544	7.157295126	7.05992818	8.275052313	8.192729549
ENSG00000085872	CHERP	-0.821368824	0.142652965	-0.773903291	0.161318642	-1.595272114	9.081016759	8.791896851	9.868959282	8.409925372	8.59153817	8.680667534	8.407389648
ENSG00000160051	IQCC	-1.85580017	0.023144808	0.260385785	0.778780813	-1.595414385	5.095959458	5.595927442	4.523675391	3.515101962	3.82856379	5.449961224	5.349527629
ENSG00000185875	THNSL1	-0.857589202	0.254248233	-0.738251224	0.318663122	-1.595840426	5.532353208	6.535001748	5.930865648	5.258265913	5.3193943	5.669467138	5.0581604
ENSG00000114779	ABHD14B	-0.863713487	0.259714186	-0.733137779	0.337851136	-1.596851266	9.460782017	8.234834549	9.963514551	8.446246007	8.59763324	8.357847151	8.89823658
ENSG00000168556	ING2	-1.084290375	0.098414809	-0.513204273	0.457693718	-1.597494648	8.285626337	7.538394143	6.958202216	6.693715713	6.5890786	6.915242278	7.428550441
ENSG00000139291	TMEM19	-0.790567012	0.443668589	-0.80840849	0.422686222	-1.598975502	9.544849385	6.899080544	8.677268317	7.990026167	7.90439517	7.79139499	8.057030103
ENSG00000132963	POMP	0.408542176	0.501949395	-2.008784389	7.65E-05	-1.600242213	10.16946352	9.267380431	10.49365885	10.46951599	10.4658164	8.04860508	8.087009646
ENSG00000221978	CCNL2	-0.991542303	0.004574811	-0.608812607	0.096019822	-1.60035491	10.80239439	11.14636276	11.40332671	10.1289481	10.1685416	10.66298318	10.38437908
ENSG00000159346	ADIPOR1	-0.974731373	0.009276725	-0.626763721	0.107612604	-1.601495094	11.55650736	11.20447669	11.91489601	10.55555238	10.6694157	10.89014846	11.02814425
ENSG00000011260	UTP18	-0.35636865	0.596166767	-1.24636268	0.033143403	-1.60273133	8.946185348	8.147241016	7.646393253	8.036145657	7.95274764	7.236356087	7.0024032
ENSG00000169251	NMD3	-0.682558965	0.35660897	-0.921119735	0.190829323	-1.6036787	10.22148166	9.785372135	8.585098385	9.031955922	8.97188212	8.977260248	8.517561736
ENSG00000176087	SLC35A4	-1.19061362	0.531143362	-0.4159707	0.837702779	-1.60658432	4.00722531	10.93049815	11.18913795	9.248208205	9.35135081	9.73685847	10.34300322
ENSG00000213699	SLC35F6	-1.072914023	0.095993057	-0.536000703	0.42991362	-1.608914726	10.1076228	9.308339287	10.81605395	9.12451847	9.14521857	9.620703391	9.715016953
ENSG00000161547	SRSF2	-0.510673857	0.396866142	-1.099227674	0.044653042	-1.609901531	10.84565509	10.61258327	10.09465441	10.05303338	10.0306182	8.890924823	9.861231258
ENSG00000163634	THOC7	-0.63158217	0.601636131	-0.979785688	0.39010814	-1.611367858	9.858207046	8.590771778	6.629488387	8.223194971	8.284723	7.727325329	8.074367056
ENSG00000151500	THYN1	-0.499212016	0.561438629	-1.112709714	0.155175621	-1.61192173	7.940205782	8.671479029	6.718950348	7.497728447	7.50468064	7.031901364	6.764548117
ENSG00000152457	DCLRE1C	-0.319364436	0.530705838	-1.293177938	0.00277408	-1.612542375	7.867441304	7.393647515	7.896971941	7.450827758	7.37938471	6.679970309	6.232137896
ENSG00000071894	CPSF1	-1.014369994	0.127598657	-0.598733174	0.38415025	-1.613103168	10.68025843	10.39692156	9.132850258	9.196346103	9.20432875	9.588047701	9.638558632
ENSG00000188878	FBF1	-0.403293552	0.834689821	-1.210594242	0.484697884	-1.613887794	7.384433967	5.946638857	2	5.860316854	5.93454154	5.289239922	5.020995508
ENSG00000141627	DYM	-1.369262251	0.00015596	-0.245000521	0.565942887	-1.614262772	9.751047882	9.591588447	10.16797309	8.444537031	8.54498542	9.772619242	9.429325923
ENSG00000011132	APBA3	-1.018981023	0.321495834	-0.595565021	0.5745905	-1.614546044	7.096806373	7.761270542	9.541233192	7.464384256	7.55566492	7.975129619	7.875307424
ENSG00000148308	GTF3C5	-0.906934479	0.007452299	-0.708208404	0.039648512	-1.615142884	9.189468652	9.030900487	9.589475645	8.379838154	8.3920703	8.566163393	8.598629536
ENSG00000173933	RBM4	0.476986874	0.671489931	-2.093092726	0.029674646	-1.616105851	9.613202746	8.747909548	6.718950348	9.288777073	9.21725179	6.683779344	6.77351449
ENSG00000116489	CAPZA1	-0.53108163	0.216092164	-1.0873797	0.00553577	-1.618461329	11.35168561	11.51338035	12.09904656	11.17175047	11.1497911	10.63300595	10.57785604
ENSG00000166902	MRPL16	-1.140998669	0.146175165	-0.477928167	0.568416252	-1.618926835	9.810482605	8.623598428	8.007629717	7.745471964	8.01099479	8.385592001	8.676578788
ENSG00000072958	AP1M1	-1.179511609	0.016308157	-0.44127264	0.412109699	-1.62078425	10.51739401	9.828248506	10.87075861	9.331013912	9.24954577	9.880069077	10.1574558
ENSG00000172009	THOP1	-1.049925723	0.646239362	-0.572169259	0.808778437	-1.622094982	10.35389212	7.874700259	2	7.927778434	8.01555453	8.066950774	8.741991736
ENSG00000187601	MAGEH1	-1.475539763	0.000257387	-0.146569589	0.768207195	-1.622109352	8.87372952	9.00621767	8.236946975	7.352188525	7.23826702	8.678282924	8.523553267
ENSG00000113593	PPWD1	-0.452728914	0.736046082	-1.170912284	0.337870848	-1.623641198	9.613202746	8.029695761	6.073378139	8.139053376	8.0314011	7.55607473	7.18317631
ENSG00000136908	DPM2	-1.143887217	0.000621727	-0.479820398	0.184008652	-1.623707615	8.998234871	8.806266215	9.287079172	7.900612272	7.91663606	8.427654172	8.688479312
ENSG00000197980	LEKR1	-0.045903241	0.986149479	-1.580136215	0.503376289	-1.626039456	2	4.11352228	2	2.947924294	3.28037825	2.560105256	2.421067459
ENSG00000198026	ZNF335	-0.699916836	0.404128024	-0.927196939	0.246468033	-1.627113775	9.497412313	8.655694736	7.502634626	8.078634414	8.07355593	7.752026287	7.946410318
ENSG00000115266	APC2	-1.383856991	0.019668123	-0.243780579	0.719764201	-1.627637571	6.257048221	6.140560021	6.80318718	5.319220934	4.93258395	6.353989995	5.995914157
ENSG00000173614	NMNAT1	-0.949663814	0.251382472	-0.678120911	0.419378285	-1.627784725	8.963744667	7.274681896	7.451345288	7.165617905	7.18663697	7.511762185	7.366199662
ENSG00000149547	EI24	-1.172465159	0.032927043	-0.456108007	0.445567406	-1.628573165	11.60207034	10.93704246	10.37481416	9.906793906	9.87119971	10.47807907	10.71812545
ENSG00000108798	ABI3	-2.999620341	0.29492176	1.370981987	0.608991096	-1.628638354	2	3.686303414	2	2.141737246	2.14022021	4.122514202	2.918126211
ENSG00000156482	RPL30	-0.528807095	0.554920781	-1.09989818	0.180853	-1.628705275	12.12920672	10.26789142	10.54960077	10.6534857	10.7506356	10.11437312	10.15230914
ENSG00000182199	SHMT2	-1.435263653	1.76E-05	-0.193900156	0.632771785	-1.629163809	11.20066853	10.65686569	11.25791777	9.61323902	9.64710779	10.9086776	10.82495146
ENSG00000185361	TNFAIP8L1	-2.079156997	0.080087979	0.449921476	0.736888343	-1.629235521	5.532353208	6.666836837	8.784770914	5.514313187	5.72778967	7.978234673	8.126037235
ENSG00000185842	DNAH14	-0.561511866	0.536732782	-1.068651791	0.200135445	-1.630163657	4.815609471	5.595927442	5.59507254	4.804776789	4.97065356	4.764298067	4.077147165
ENSG00000213626	LBH	-2.985600854	1.31E-32	1.354928113	1.32E-07	-1.63067274	12.05127578	11.68259927	11.80936937	8.887228971	8.87358581	13.30687429	13.10455459
ENSG00000116898	MRPS15	-0.857248483	0.226279403	-0.774491692	0.270404914	-1.631740174	9.657036304	9.114109157	8.042706615	8.178656739	8.28661061	8.11499695	8.491306993
ENSG00000168256	NKIRAS2	-1.206616729	0.059908089	-0.425231973	0.543799327	-1.631848702	11.50046327	10.14890644	10.34701993	9.569217728	9.61666844	10.29003906	10.44841713
ENSG00000115368	WDR75	-0.359640461	0.497802375	-1.274060212	0.005383097	-1.633700673	9.233563228	8.590771778	9.180874355	8.670421699	8.66738547	8.044167181	7.422862719
ENSG00000150054	MPP7	-4.742671606	4.12E-06	3.108839248	4.41E-05	-1.633832358	4.00722531	4.93638933	5.59507254	2.3892346	2.2680111	7.743839536	8.06618078
ENSG00000184698	OR51M1	-3.753027084	NA	2.118273668	NA	-1.634753416	2	2	2	2	2	2.717032197	3.36036153
ENSG00000273780		4.40134359	0.045693057	-6.036288545	0.005795713	-1.634944954	2	7.002537973	2	9.715273165	9.87810168	2.094598369	2.326754077
ENSG00000168040	FADD	0.230984751	0.80683022	-1.866914004	0.015246955	-1.635929252	9.33149386	7.315436877	8.408111533	8.805705438	8.790887	6.571075185	6.895576665
ENSG00000105355	PLIN3	-0.931847766	0.022017331	-0.70420287	0.090276485	-1.636050636	11.40085503	11.30655942	11.91962127	10.63224832	10.642768	10.62126059	11.07286611
ENSG00000177025	C19orf18	-0.23625302	0.91483416	-1.400102605	0.468311342	-1.636355625	4.00722531	4.11352228	2	3.462030052	3.55551834	3.256555576	2.326754077
ENSG00000172269	DPAGT1	-1.095534861	0.042310832	-0.540845681	0.344232516	-1.636380542	9.848787621	9.549500232	8.699419242	8.388752656	8.32569041	8.738579005	9.057211603
ENSG00000091536	MYO15A	-1.248981788	0.635835342	-0.388235429	0.890313426	-1.637217217	6.138360429	2	2	3.709785322	3.74316441	4.521997428	4.215171317
ENSG00000028116	VRK2	0.486062392	0.417669822	-2.123516636	2.79E-05	-1.637454244	8.777722034	8.356994601	7.552162999	8.807036108	8.78555748	6.332313483	6.165581817
ENSG00000133983	COX16	0.908258014	0.077275225	-2.545911432	4.05E-08	-1.637653418	7.384433967	6.899080544	7.691296561	8.235106869	8.24450173	4.73522696	5.164234344
ENSG00000168924	LETM1	-1.26761458	0.000702835	-0.370414385	0.377920795	-1.638028965	9.790941832	9.599860728	10.17589429	8.643859774	8.58388295	9.284329221	9.695689382
ENSG00000136044	APPL2	-1.57493892	0.025870272	-0.06350371	0.94272587	-1.63844263	10.30651143	8.717821467	9.116480286	7.982979299	7.98565528	9.22478876	9.698340256
ENSG00000104763	ASAH1	-0.183684071	0.826100734	-1.454933106	0.032980835	-1.638617177	11.04027452	9.672251278	11.36918911	10.70364381	10.642031	9.52813224	9.274507165
ENSG00000104897	SF3A2	-1.163909042	5.66E-05	-0.47541993	0.12973302	-1.639328973	9.992581382	9.574900212	9.990713521	8.699372486	8.71161289	9.379289361	9.3981418
ENSG00000076003	MCM6	-1.745902078	0.002944485	0.106152008	0.886197127	-1.63975007	7.485143553	8.671479029	7.734844356	6.456560224	6.28357155	8.341061068	7.98359008
ENSG00000100767	PAPLN	-3.019452803	7.07E-05	1.379151089	0.069529485	-1.640301713	5.532353208	5.72254323	6.203069917	3.615725078	3.15372745	6.651077397	7.531242162
ENSG00000128683	GAD1	2.152475678	NA	-3.793052289	NA	-1.640576611	2	2	2	3.406930997	2.68924543	2.048074405	2
ENSG00000013375	PGM3	-0.467614483	0.337673274	-1.1739301	0.007115471	-1.641544584	10.84091191	11.37322539	11.78108696	10.91431859	10.9155453	10.26324148	10.15574229
ENSG00000168036	CTNNB1	-0.573688045	0.076570922	-1.068714112	0.000343749	-1.642402157	12.47671498	12.06519355	12.56341295	11.7822503	11.8378939	11.25618281	11.37339918
ENSG00000144550	CPNE9	-0.272311451	0.93880631	-1.37034212	NA	-1.642653571	2	2	3.369317972	2.498691189	2.49394783	2.42098416	2.059860178
ENSG00000075218	GTSE1	-1.214188438	0.566306066	-0.42863272	0.849170035	-1.642821158	3.327711234	6.666836837	2	4.17310023	4.3456612	4.644324387	5.14363432
C1QTNF9B-AS1	C1QTNF9B-A	3.546158349	0.533465331	-5.191092003	0.364514502	-1.644933655	2	2	2	3.840289928	4.12711147	2	2
ENSG00000196235	SUPT5H	-0.549656807	0.262050542	-1.097783477	0.014619621	-1.647440284	11.42640922	10.62884366	11.54155646	10.69755827	10.7065491	10.11666451	10.19339501
ENSG00000187051	RPS19BP1	-0.817011244	0.386453724	-0.83084243	0.366751756	-1.647853673	10.499447	8.955547846	8.325066185	8.752828065	8.74357228	8.836358413	8.624876935
ENSG00000138303	ASCC1	-0.950919044	0.001032412	-0.697042141	0.018248047	-1.647961185	8.540903468	8.639735838	8.408111533	7.549526251	7.64959655	7.802823992	7.894955865
ENSG00000079246	XRCC5	-0.71954502	0.020700446	-0.928790617	0.001956856	-1.648335637	11.52136524	11.94371713	11.760164	11.06651655	11.0013784	10.66815974	10.9674134
ENSG00000154258	ABCA9	1.136848049	0.409917847	-2.789465146	0.029770746	-1.652617097	4.00722531	3.076665209	4.523675391	4.825935136	4.89348248	2.3070652	2.671842496
ENSG00000132341	RAN	-0.88339077	0.110591109	-0.769276209	0.163161971	-1.652666979	12.24323744	11.26814685	11.34128524	10.8245327	10.7897592	10.67988853	11.12776105
ENSG00000175826	CTDNEP1	-0.819918827	0.121301732	-0.833882783	0.108401159	-1.653801609	10.51143645	10.32389548	9.452720984	9.298270478	9.39837151	9.194070966	9.464010251
ENSG00000167257	RNF214	-0.758673162	0.72338932	-0.898179109	0.668849935	-1.656852271	9.397585735	7.669933291	2	7.409376773	7.49817903	7.371203179	7.263337153
ENSG00000088179	PTPN4	-0.67020026	0.107207965	-0.986972721	0.011684435	-1.65717298	7.940205782	8.004992147	8.561105505	7.549526251	7.51437845	7.178138321	7.266516281
ENSG00000213190	MLLT11	0.362709659	0.26497168	-2.020085307	1.53E-13	-1.657375648	10.31337612	10.3387994	10.32581712	10.70292919	10.6726672	8.085786009	8.521558854
ENSG00000157800	SLC37A3	-1.165995899	0.002229097	-0.491522291	0.235579015	-1.65751819	9.602033055	10.32888058	10.10298738	8.852852814	8.91688363	9.458689553	9.645596027
ENSG00000078668	VDAC3	-1.13255362	0.002449695	-0.525754295	0.1906313	-1.658307916	10.1076228	10.10308486	10.68897008	9.164517339	9.23104065	9.671222295	9.921211649
ENSG00000185624	P4HB	-0.255405536	0.684986885	-1.40319177	0.007503255	-1.658597306	15.03873922	13.88920025	14.24271598	14.1891151	14.2452844	12.93237165	13.19573601
ENSG00000169964	TMEM42	-1.620193879	0.057338722	-0.03919371	0.970049089	-1.659387589	6.366709671	7.467835385	8.536706852	6.18902405	6.1068222	7.852739912	7.470508284
ENSG00000162073	PAQR4	-1.400612513	0.633071268	-0.259187572	0.935195648	-1.659800085	2	2	8.110401118	5.273747807	5.24368216	6.68757835	5.812327524
ENSG00000198908	BHLHB9	-0.953071876	0.644603834	-0.707044466	0.7339259	-1.660116342	2	5.946638857	8.325066185	6.221320894	5.9536761	6.577231679	6.02248356
ENSG00000177613	CSTF2T	-0.940116861	0.524284348	-0.720048381	0.62682033	-1.660165242	5.095959458	9.159587375	9.681375057	7.898117066	8.02914793	8.278834759	8.077990544
ENSG00000282684		-0.56064183	0.704833394	-1.100631355	0.423483563	-1.661273185	4.815609471	8.655694736	9.116480286	7.818544084	7.79989812	7.249266052	7.311843322
ENSG00000214300	SPDYE3	-1.779759788	0.200017793	0.11793418	0.942222441	-1.661825608	6.563806279	3.076665209	5.39244166	4.17310023	3.94787299	5.31402805	5.980509018
ENSG00000070814	TCOF1	-1.20654221	5.45E-05	-0.45531789	0.163428103	-1.6618601	9.262228249	9.506147172	9.553445883	8.16211395	8.33484115	9.126408411	8.849249315
ENSG00000129467	ADCY4	-3.923771053	0.064138813	2.261508784	0.2596534	-1.662262269	2	5.303523747	2	2.270786764	2.2680111	6.031172603	5.957088417
ENSG00000105383	CD33	-3.753027428	NA	2.090678645	NA	-1.662348783	2	2	2	2	2	3.316666208	2.746601469
ENSG00000140280	LYSMD2	0.364222112	0.867562397	-2.026640189	0.276521038	-1.662418077	8.564112515	6.389896633	2	7.590284224	7.67863747	5.30910438	5.414021719
ENSG00000107175	CREB3	-0.59862801	0.509478857	-1.064724746	0.204926957	-1.663352756	11.00254523	10.08551947	8.825624954	9.665524776	9.59245646	9.010625386	9.306519748
ENSG00000120156	TEK	-0.73527338	0.588183879	-0.928368419	0.477216955	-1.6636418	4.467311984	8.077869079	7.818184403	6.64692533	6.79406287	6.109723842	6.866539459

ENSG00000164896	FASTK	-1.579612167	0.001157054	-0.085267373	0.89140859	-1.66487954	9.112843761	9.733683991	10.23774519	8.18685754	8.21109983	9.706989438	9.653813751
ENSG00000186918	ZNF395	-0.661608553	0.390208914	-1.003416452	0.165231608	-1.665025006	8.910412251	9.648522413	10.49998282	9.076774616	9.2611121	8.444590638	9.138408485
ENSG00000183401	CCDC159	-0.643879931	0.734506972	-1.021307844	0.571333187	-1.665187775	7.384433967	5.72254323	2	5.602782483	5.63601114	4.977914261	5.524371359
ENSG00000177542	SLC25A22	-1.183600466	0.16737365	-0.482143441	0.599074031	-1.665743907	10.29961392	9.102512015	8.434763955	8.323303759	8.30721295	8.465761957	9.403927624
ENSG00000116157	GPX7	-1.419725076	5.57E-07	-0.246570324	0.457548655	-1.6662954	8.737455672	8.955547846	9.149036566	7.565338955	7.5462416	8.688745854	8.734549554
ENSG00000111667	USP5	-0.906122188	0.071713825	-0.76141596	0.130742576	-1.667538149	11.00254523	10.55418374	10.03493575	9.634381252	9.72643218	9.583976969	10.03183938
ENSG00000160117	ANKLE1	-0.577098276	0.672951696	-1.091582506	0.38391229	-1.668680782	3.327711234	4.442710129	4.523675391	3.840289928	3.69849104	3.709392871	3.071407821
ENSG00000086589	RBM22	-0.496515901	0.163759565	-1.172718904	0.00023687	-1.669234804	9.097018025	9.386925327	9.439621147	8.861844103	8.78422202	8.322245777	7.965119966
ENSG00000204923	FBXO48	-0.750522552	0.612898467	-0.918923507	0.519595531	-1.669446059	3.327711234	4.442710129	6.322058544	4.358765249	4.72553021	4.698044417	4.062589736
ENSG00000166261	ZNF202	-0.63444433	0.744523835	-1.035313942	0.575633447	-1.669758271	3.327711234	5.946638857	8.99637432	7.057964187	6.89649843	6.800644007	6.350712758
ENSG00000134955	SLC37A2	-1.167834343	0.524450905	-0.503083062	0.79391096	-1.670917405	7.82963488	3.686303414	4.523675391	5.194621444	5.51241389	6.569017168	4.943664032
ENSG00000147383	NSDHL	-0.798609859	0.276678479	-0.872861557	0.220822202	-1.671471416	8.631580145	8.451443573	9.577565424	8.180711314	8.185025	7.491260006	8.541379994
ENSG00000121067	SPOP	-0.402862358	0.361569449	-1.26921843	0.000964865	-1.672080788	9.992581382	9.256956408	9.528916236	9.221512599	9.22219152	8.455215133	8.265241863
ENSG00000165555	NOXRED1	-0.554290629	0.837802275	-1.121803927	0.655320771	-1.676094556	2	4.710564154	2	3.566290607	2.77779907	3.059555873	2.782576693
ENSG00000136718	IMP4	-0.8271799	0.243498812	-0.851034041	0.220441583	-1.678213941	9.751047882	9.318399851	8.110401118	8.408172799	8.37442884	8.47458408	8.252471774
ENSG00000204070	SYS1	-0.82929883	0.272167028	-0.849117501	0.250993106	-1.678416331	9.790941832	8.687092496	8.110401118	8.178656739	8.248381	8.109343506	8.274745794
ENSG00000160783	PMF1	-0.973629399	0.058967988	-0.706376544	0.176639946	-1.680005943	8.910412251	8.004992147	8.236946975	7.467753569	7.48179565	7.523500045	7.922424886
ENSG00000179163	FUCA1	-0.91088221	0.059073176	-0.769819761	0.110655463	-1.680701971	9.27634997	8.297207175	8.922968322	8.008651253	7.95274764	8.098683215	8.140837768
ENSG00000140830	TXNL4B	-0.061577044	0.96857017	-1.619344579	0.220279485	-1.680921622	7.709880939	8.213429689	4.058998049	7.388195945	7.39346687	6.15433139	5.586744392
ENSG00000138785	INTS12	-0.859407716	0.707528996	-0.822117432	0.717366226	-1.681525148	9.790941832	6.899080544	2	7.552702688	7.54308677	7.70407074	7.453871289
ENSG00000198839	ZNF277	-0.456424362	0.582172352	-1.227926763	0.097270672	-1.684351125	7.667647223	6.535001748	8.296287027	7.144720234	7.27676203	6.621594001	6.29575846
ENSG00000092208	GEMIN2	-1.163895752	0.296880612	-0.521028965	0.655988002	-1.684924717	4.00722531	5.595927442	6.629488387	4.927308402	4.4836996	5.371837532	5.191251321
ENSG00000162231	NXF1	-0.250476754	0.70357284	-1.434540897	0.008847264	-1.685017651	10.29268328	8.993716073	9.746650996	9.534293296	9.50598504	8.315509958	8.376090431
ENSG00000136754	ABI1	-0.302496756	0.713785889	-1.383100844	0.049810851	-1.685597599	10.87379128	9.067150531	10.36098398	9.99525349	9.95667084	8.871434321	8.931664222
ENSG00000142186	SCYL1	-0.875594879	0.099672705	-0.812896239	0.122835348	-1.688491117	11.88613247	10.78935021	11.15347798	10.44280012	10.5080911	10.47574661	10.59834356
ENSG00000085274	MYNN	-0.620357133	0.200056839	-1.071745961	0.01704757	-1.692103095	7.904282243	8.124485672	8.35328246	7.484483106	7.57123495	7.364099281	6.746446439
ENSG00000155363	MOV10	0.088830676	0.823806404	-1.781015875	2.32E-09	-1.692185199	10.46903009	10.61258327	10.68342405	10.6531158	10.705844	8.559959383	9.039201374
ENSG00000089006	SNX5	-1.149570905	0.105639361	-0.542732043	0.470337112	-1.692302948	8.444143041	9.523645035	8.110401118	7.717440541	7.67575955	8.582068017	7.933467731
ENSG00000064666	CNN2	-1.92583233	1.07E-06	0.231867908	0.632223509	-1.693964421	10.9550501	11.23666298	11.68722117	9.368448609	9.44052092	11.32281177	11.75674553
ENSG00000261509	TP53TG3B	3.49665459	0.539543786	-5.19109221	0.364514502	-1.694437619	2	2	2	3.95996233	3.94787299	2	2
ENSG00000104969	SGTA	-1.006142379	0.122841764	-0.688622659	0.300383908	-1.694765038	10.92846914	9.799806309	9.577565424	9.180006057	9.2754408	9.47746372	9.60786912
ENSG00000160410	SHKBP1	-0.805313112	0.087707776	-0.88963815	0.052243716	-1.694951262	10.02639697	9.424669273	9.503962296	8.83470036	8.91566441	8.447394152	9.070459565
ENSG00000100304	TTLL12	-1.72350201	0.003221255	0.028321413	0.970049089	-1.695180597	9.2903348	8.834582582	10.19160738	7.815902688	7.86955761	9.664732333	9.483608161
ENSG00000070610	GBA2	-1.098720387	0.061878981	-0.597117846	0.332654942	-1.695838233	10.15425007	8.876041558	9.31604076	8.503178519	8.40776525	9.012898235	8.890530144
ENSG00000023697	DERA	-0.822996441	0.052698164	-0.877385031	0.03408921	-1.700381471	7.750913381	8.053983481	8.325066185	7.330144653	7.1744553	7.019897747	7.363232998
ENSG00000119048	UBE2B	-0.22311307	0.768318166	-1.478495115	0.018944745	-1.701608185	10.03472846	8.487557258	9.553445883	9.29542901	9.23691006	8.102956788	7.940451241
ENSG00000128694	OSGEPL1	-1.261765594	0.333096085	-0.441502877	0.751814452	-1.70326847	4.00722531	6.04685525	7.398165132	5.273747807	5.14724905	6.320128742	5.486701579
ENSG00000160199	PKNOX1	-0.583609933	0.444231654	-1.119795332	0.109134859	-1.703405264	7.904282243	8.94259736	9.636156974	8.352759303	8.4705529	7.86621124	7.902127837
ENSG00000115239	ASB3	1.604573695	0.105639361	-3.309429154	0.000302537	-1.704855459	5.866944937	5.457120953	7.451345288	7.944803394	8.19912382	4.017766949	3.236138336
ENSG00000100219	XBP1	-0.927139927	0.023535526	-0.779682169	0.059210657	-1.706822096	12.34389158	11.70001862	11.60746745	10.99183871	11.0010911	11.18195716	11.10429958
ENSG00000115282	TTC31	-1.085566035	0.003498455	-0.621711953	0.110225496	-1.707277988	8.910412251	8.337339476	8.922968322	7.605658789	7.73225478	8.127636775	8.125161869
ENSG00000105662	CRTC1	-0.635790466	0.302788797	-1.071513371	0.061577604	-1.707303836	9.740899611	8.522788974	9.031722608	8.501535708	8.60067114	8.292619498	7.927454842
ENSG00000172375	C2CD2L	-1.005923373	0.650131252	-0.702852776	0.755000321	-1.708776149	9.781071289	2	8.65477198	7.728718552	7.77322015	8.28637003	7.764992659
ENSG00000189114	BLOC1S3	-0.785214634	0.507120384	-0.923594453	0.41925397	-1.708809087	6.009025997	8.469613413	9.25752426	7.555872146	7.74054144	7.489084836	7.54177663
ENSG00000055147	FAM114A2	-0.94425923	0.330789334	-0.766679321	0.431024961	-1.710938551	7.624139768	7.232742069	9.385998894	7.440576128	7.5014335	7.644728141	7.645539136
ENSG00000101460	MAP1LC3A	-1.33262446	0.214603975	-0.3794546	0.747588951	-1.712079059	10.72727936	8.777382925	8.007629717	8.336264621	8.2960118	9.135476483	9.375867991
ENSG00000205309	NT5M	-2.253563752	0.25952816	0.540829163	0.8046279	-1.712734589	2	6.228547981	3.369317972	3.162721474	3.33976443	5.951260634	4.522269241
ENSG00000132286	TIMM10B	-0.80307707	0.244724681	-0.909869845	0.173666446	-1.712946915	8.468948322	9.632483534	10.06083273	8.734762645	8.72837565	8.571313099	8.678370137
ENSG00000128944	KNSTRN	-1.374195032	0.02660565	-0.339538951	0.627355322	-1.713733983	8.138771685	7.761270542	6.718950348	6.213314212	6.43421531	7.25183424	7.395535532
ENSG00000167723	TRPV3	0.80838034	0.786688692	-2.523361146	0.345358207	-1.714980806	2	2	7.896971941	7.144720234	7.1204461	4.443291373	3.735548302
ENSG00000143882	ATP6V1C2	-1.562550723	0.533990735	-0.152570063	0.955158571	-1.715120786	2	4.11352228	2	2.600425781	2.38539787	2.937246078	3.183290849
ENSG00000113387	SUB1	-0.603790046	0.34984013	-1.112500619	0.062080537	-1.716290665	10.65885801	9.34816733	9.714382178	9.451627281	9.37638276	8.911780399	8.906921063
ENSG00000275221	HIST1H2AK	3.47228305	0.542787593	-5.191092311	0.364514502	-1.718809261	2	2	2	4.139692846	3.69849104	2	2
ENSG00000007392	LUC7L	-0.384197583	0.728252779	-1.334843017	0.170852952	-1.7190406	9.858207046	8.433041974	7.342949392	8.496596021	8.54656162	7.876232824	7.221358049
ENSG00000168765	GSTM4	-1.430444599	0.006866454	-0.29018578	0.633808927	-1.720630379	7.218819781	7.60566235	8.266922072	6.436065776	6.30607931	7.303499023	7.627107456
ENSG00000162664	ZNF326	-0.238381594	0.68556684	-1.482536287	0.002183465	-1.720917882	8.816894989	8.451443573	7.734844356	8.190940526	8.14396641	6.864125031	7.032593922
ENSG00000090432	MUL1	-0.834000644	0.024480315	-0.887427738	0.01510731	-1.721428382	10.06758145	9.433952963	9.829330889	8.924487942	9.01008467	8.808281231	9.014359877
ENSG00000136891	TEX10	-0.608772049	0.180706341	-1.113790138	0.007855038	-1.722562187	9.219014269	8.590771778	9.413059136	8.485003881	8.52593541	7.950836136	8.061612696
ENSG00000179335	CLK3	-0.841309968	0.047154938	-0.88633467	0.03310095	-1.727644637	9.624286623	9.114109157	9.839340503	8.707945635	8.72698617	8.832932104	8.492665077
ENSG00000170271	FAXDC2	-1.273023646	0.289625434	-0.455235384	0.72430187	-1.72825903	5.330594134	7.790474954	8.825624954	6.687949235	6.6253296	7.512833218	7.383872668
ENSG00000120992	LYPLA1	-0.64617318	0.140124762	-1.082377142	0.007862246	-1.728550322	9.262228249	9.00621767	8.511888463	8.401141138	8.23671175	8.007404089	7.764992659
ENSG00000155066	PROM2	-0.701839933	0.69608789	-1.026980046	0.544850149	-1.72881998	5.095959458	3.076665209	3.369317972	3.921164147	3.2184422	3.796405078	2.851942824
ENSG00000068001	HYAL2	-0.81823896	0.135737919	-0.911462058	0.087275793	-1.729701018	8.777722034	9.308339287	9.935792933	8.592312513	8.61726622	8.581557692	8.440137439
ENSG00000135392	DNAJC14	-0.73349074	0.182358952	-0.996864893	0.056539999	-1.730355633	9.751047882	8.791896851	8.865353883	8.50645854	8.44542686	8.071319244	8.347853908
ENSG00000125149	C16orf70	-0.708356003	0.283441752	-1.022345889	0.100649724	-1.730701892	8.816894989	7.847167909	7.502634626	7.447418636	7.49164798	7.258234779	7.062165783
ENSG00000189129	PLAC9	-3.360469219	6.63E-07	1.629205074	0.017958187	-1.731264145	6.563806279	7.189546432	7.029880454	3.921164147	4.02226117	7.778984728	9.054915125
ENSG00000135829	DHX9	-0.603325679	0.433957193	-1.129454314	0.110981627	-1.732779992	9.657036304	10.94030351	11.36918911	10.225157	10.2084185	9.457299408	9.895260472
ENSG00000127540	UQCR11	-0.571339478	0.181227887	-1.16154079	0.002838737	-1.732880269	10.27872131	9.842262059	9.491321578	9.32080297	9.35585278	8.713644723	8.790832728
ENSG00000162604	TM2D1	-0.524645985	0.627093532	-1.212612674	0.217050348	-1.737258659	8.418903792	6.389896633	9.06622548	7.857595438	7.74603955	7.253116621	6.996671423
ENSG00000131061	ZNF341	-1.187749495	0.585838677	-0.549831424	0.808868217	-1.737580919	8.009475818	4.442710129	2	5.461211713	5.43283104	6.285453595	5.736403255
ENSG00000120053	GOT1	-1.27971996	8.61E-05	-0.457955749	0.199124017	-1.737675708	8.564112515	9.018611864	8.699419242	7.423326516	7.60793141	8.288873067	8.36352657
ENSG00000167186	COQ7	-1.498338725	0.039474306	-0.240213869	0.777011185	-1.738552594	7.031698319	7.189546432	8.408111533	6.043074888	6.39275508	7.123093858	7.701812481
ENSG00000244045	TMEM199	0.199713957	0.738698334	-1.938695275	4.72E-05	-1.738981317	8.653385977	8.029695761	7.646393253	8.415170354	8.31648126	6.132200015	6.426568739
ENSG00000144668	ITGA9	-3.268949188	0.370967351	1.529795759	0.642128837	-1.739153429	2	3.076665209	2	2.141737246	2	3.374372158	3.183290849
ENSG00000106144	CASP2	-1.080647744	0.0034093	-0.658897142	0.086198005	-1.739544886	8.836089686	8.234834549	8.742726175	7.565338955	7.5462416	7.909957323	8.027351693
ENSG00000054219	LY75	-2.433843174	0.123996487	0.693116674	0.667996442	-1.7407265	2	4.11352228	4.058998049	2.498691189	2.49394783	4.053534413	4.329022995
ENSG00000146731	CCT6A	-0.805963363	0.044014587	-0.934907752	0.016521712	-1.740871114	10.29961392	11.00402286	10.96062172	9.955006472	10.0164701	9.900058137	9.813939602
ENSG00000155876	RRAGA	-1.030804312	0.189102784	-0.711938895	0.372465764	-1.742743207	11.86992646	10.1878402	11.87895445	10.43765481	10.4983508	10.43140618	11.07104905
ENSG00000063046	EIF4B	-1.181224725	0.012942716	-0.561561847	0.270007586	-1.742786573	11.23722567	12.27732859	11.73357624	10.66012781	10.6058245	11.22089964	11.28171193
ENSG00000072682	P4HA2	-0.462151904	0.568285409	-1.281306023	0.076411855	-1.743457927	12.76534233	11.35134391	11.19694409	11.46811054	11.5206165	10.54583177	10.79719235
ENSG00000139880	CDH24	-1.050174708	0.599957698	-0.693477864	0.732916452	-1.743652573	8.313285031	6.140560021	2	6.155987605	5.88556448	6.446574529	6.276965131
ENSG00000203710	CR1	-0.678242903	0.774549338	-1.065777093	0.631689024	-1.744019997	2	3.076665209	3.369317972	2.784612485	2.49394783	2.526562374	2.509591799
ENSG00000157216	SSBP3	-0.878512015	0.010660073	-0.866073188	0.011165872	-1.744585203	9.567995545	9.055168103	9.491321578	8.579911316	8.44204342	8.444590638	8.601149963
ENSG00000135473	PAN2	-1.0495667	0.615039451	-0.69627695	0.742149828	-1.745843651	9.460782017	8.053983481	2	7.288834728	7.33629421	7.751118939	7.558732759
ENSG00000106330	MOSPD3	-1.457889951	0.146764584	-0.288171706	0.799963632	-1.746061656	8.910412251	6.311476754	7.818184403	6.610808945	6.60731796	7.362911889	8.047821014
ENSG00000167468	GPX4	-0.776421644	0.280259419	-0.969858885	0.15923574	-1.746280529	12.02224098	10.41578665	11.01019887	10.49657106	10.5609081	10.28189487	10.38857981
ENSG00000213029	SPHAR	3.444489832	0.546436072	-5.191092427	0.364514502	-1.746602595	2	2	2	3.95996233	3.86943969	2	2
ENSG00000059378	PARP12	-7.139314663	0.000113553	5.392448157	4.05E-09	-1.746866506	3.327711234	4.442710129	3.369317972	2	2	8.878929913	8.75393321
ENSG00000115661	STK16	-1.175643234	0.392717095	-0.5714471	0.6908855	-1.747090334	8.107517749	4.93638933	9.083172337	6.820355514	7.14145882	7.450478673	7.695929227
ENSG00000147601	TERF1	-0.66729278	0.115572829	-1.081859076	0.006258366	-1.749151856	8.39321512	8.732943944	9.049077186	8.074220386	8.10384772	7.496683627	7.845855157
ENSG00000176714	CCDC121	-1.68853666	0.309909983	-0.061884741	0.973199511	-1.750421402	4.00722531	4.710564154	2	2.947924294	2.94009902	4.311629396	3.45257121
ENSG00000133316	WDR74	-0.123108401	0.780669035	-1.631865051	1.78E-06	-1.754973452	7.667647223	7.847167909	8.110401118	7.656739892	7.86198169	6.315225883	6.289521179
ENSG00000070018	LRP6	-1.567855721	2.60E-06	-0.187360505	0.646525284	-1.755216226	10.90138923	10.30378101	10.5250051	9.077877436	8.99284518	10.29953459	10.51207876
ENSG00000276043	UHRF1	-0.816974689	0.493227914	-0.939594658	0.413811187	-1.756569347	4.467311984	6.899080544	5.930865648	5.242616075	5.43283104	4.596630906	5.670730563
ENSG00000082153	BZW1	0.881558018	5.06E-05	-2.638888745	8.51E-38	-1.757330727	11.07704221	11.07990752	11.18913795	12.03638639	11.9548335	8.385007206	8.589138522
ENSG00000169230	PRELID1	-0.51133154	0.210210865	-1.246118336	0.000633362	-1.757449877	10.29268328	9.656475478	10.24529339	9.584817224	9.57557184	8.744061916	8.949576212
ENSG00000185298	CCDC137	-1.244707294	0.234625812	-0.513865031	0.646098069	-1.758572325	9.579430841	6.666836837	8.742726175	7.536750104	7.51115308	8.189226297	8.293567867
ENSG00000058729	RIOK2	-0.542847744	0.253506089	-1.215767922	0.004575334	-1.758615666	8.540903468	8.732943944	9.301632639	8.334420183	8.37620272	7.851893762	7.513957873
ENSG00000155368	DBI	-0.809067216	0.137065313	-0.950084653	0.070975318	-1.759151869	10.1770105	9.591588447	10.44859247	9.324524407	9.29056951	8.749524069	9.491441317
ENSG00000138336	TET1	-2.820769075	0.107655331	1.060360439	0.572841244	-1.760408636	4.00722531	8.053983481	3.369317972	4.034562184	4.1930041	7.86872317	7.386797235
ENSG00000243335	KCTD7	-1.456930929	0.432787266	-0.30496971	0.882758816	-1.76190064	3.327711234	7.874700259	9.954333071	7.206526483	7.29937545	8.693476763	7.991296779
ENSG00000179044	EXOC3L1	-2.265410344	0.340614989	0.50332903	0.842603619	-1.762081313	2	4.710564154	2	2.3892346	2.59489879	3.517841605	4.241259475
ENSG00000100983	GSS	-0.727673359	0.573772614	-1.03774661	0.399735181	-1.765419969	10.39976609	10.17128279	6.80318718	9.073461088	9.0169232	8.500191379	8.94213989
ENSG00000173275	ZNF449	-1.261453385	0.451753365	-0.505040778	0.775706431	-1.766494163	3.327711234	7.874700259	8.35328246	6.496695859	6.19790195	7.669911566	6.074197996
ENSG00000115204	MPV17	-1.076987109	0.000427769	-0.689672307	0.030115812	-1.766659417	9.781071289	9.540933215	9.344432377	8.456457569	8.53866332	8.767583318	8.987639044
ENSG00000102225	CDK16	-1.168614058	0.013435581	-0.59862171	0.233839673	-1.767235768	11.43272766	10.55843445	11.2764554	9.953204745	9.98600927	10.27764795	10.75820049
ENSG00000128524	ATP6V1F	-0.834221369	0.00864231	-0.933661072	0.002696789	-1.767882441	10.83137851	10.63288029	10.37481416	9.777686595	9.81017641	9.832607809	9.542821395

ENSG00000089289	IGBP1	-0.883057606	0.282118771	-0.886134988	0.270404914	-1.769192594	9.886101849	9.148351514	7.858115942	8.27796118	8.34212018	8.264917037	8.348604099
ENSG00000101464	PIGU	-0.788337454	0.653305306	-0.981822215	0.56354884	-1.770159669	9.143983763	7.701025996	3.369317972	7.210554257	7.30311013	7.006273211	7.130061054
ENSG00000147684	NDUFB9	-1.085676338	0.128503333	-0.684578009	0.352068654	-1.770254347	10.39976609	9.672251278	8.677268317	8.66162195	8.67461127	8.929935295	9.189967637
ENSG00000125772	GPCPD1	-0.006350983	0.997041173	-1.764073338	0.146403239	-1.770424321	8.042902253	8.806266215	5.156626401	8.045195239	7.86703672	6.653021698	5.703940561
ENSG00000155893	PXYLP1	-2.627399147	0.11276309	0.856078881	0.635038067	-1.771320266	7.750913381	6.602424674	2	4.523237872	4.16043394	7.651551654	7.448282715
ENSG00000090372	STRN4	0.503234931	0.722734802	-2.276067687	0.061919051	-1.772832755	7.667647223	4.11352228	7.285536013	7.370304568	7.53039799	5.185595313	4.4231929
ENSG00000155463	OXA1L	-0.854357095	0.166802524	-0.918773302	0.12650708	-1.773130398	10.76282024	9.770792087	9.491321578	9.269600773	9.26207178	9.210337096	9.193312432
APOBEC3A_B	APOBEC3A_E	3.417070077	0.549630367	-5.191092541	0.364514502	-1.774022464	2	2	2	3.881293667	3.90918929	2	2
ENSG00000115241	PPM1G	-1.177677465	0.068908006	-0.596451758	0.381507263	-1.774129223	9.720386367	9.856140801	11.04521411	9.085573664	9.23983583	9.656518038	9.823954402
ENSG00000107874	CUEDC2	-1.066450857	0.020143473	-0.7077506	0.134444561	-1.774201457	9.839306291	9.13702746	9.014156722	8.272191829	8.36552655	8.541185654	8.796342139
ENSG00000130159	ECSIT	-1.539720937	0.453495317	-0.234597927	0.918342152	-1.774318864	9.497412313	7.73146271	2	6.670510205	6.89154576	8.00283742	8.112850643
ENSG00000153896	ZNF599	-1.444145213	0.391746117	-0.332390111	0.856791105	-1.776535324	2	6.728495673	6.322058544	4.387512679	4.95174433	6.013120342	5.256659864
ENSG00000124383	MPHOSPH10	-0.462618842	0.444641873	-1.31489722	0.013709627	-1.777516062	9.064836028	8.317412869	9.528916236	8.610716699	8.5668971	7.824551575	7.66855289
ENSG00000136159	NUDT15	-1.11502814	0.114654417	-0.663096892	0.363476242	-1.778125032	7.867441304	6.951736442	8.585098385	6.917187326	6.78340763	7.249266052	7.330190599
ENSG00000188315	C3orf62	-1.221862388	0.34076194	-0.556557952	0.681335361	-1.77842034	5.330594134	6.311476754	8.536706852	6.197166296	6.14893615	6.984820384	6.607941737
ENSG00000136631	VPS45	-0.799752177	0.021412208	-0.978927477	0.003559969	-1.778679654	8.855032352	9.415325457	9.049077186	8.31397419	8.36373948	8.162177243	8.162331091
ENSG00000214866	DCDC2C	2.01372804	NA	-3.793256136	NA	-1.779528096	2	2	2	3.227757282	2.77779907	2.048074405	2
ENSG00000054938	CHRDL2	-0.474665523	0.863478741	-1.305465833	0.59982426	-1.780131356	2	4.11352228	2	2.868578001	2.94009902	2.384001718	2.746601469
ENSG00000254986	DPP3	-0.442120541	0.69646163	-1.341220527	0.182472647	-1.783341068	10.37373127	7.60566235	9.165043284	8.986874783	9.02712059	7.815899769	8.367233194
ENSG00000182628	SKA2	-1.405351301	0.006781203	-0.381722421	0.514884694	-1.787073722	8.444143041	8.876041558	9.478569125	7.66556918	7.5462416	8.357250999	8.834865421
ENSG00000124098	FAM210B	-1.170804543	0.157179445	-0.620297409	0.473386442	-1.791101952	9.957954117	9.308339287	11.28013451	9.284006841	9.22514727	9.674097378	9.920202701
ENSG00000132792	CTNNBL1	0.054045573	0.970008162	-1.84536911	0.1204601	-1.791323537	10.19941743	8.540087354	6.534109959	9.110576034	9.18523054	7.111828383	7.424286753
ENSG00000054983	GALC	-0.813797303	0.618562037	-0.97813634	0.536324873	-1.791933643	4.00722531	9.235879652	8.825624954	7.647855834	7.71553728	7.482539577	7.558732759
ENSG00000230124	ACBD6	-1.245699672	0.134484147	-0.547787683	0.536069347	-1.793487355	8.564112515	8.124485672	10.02619908	7.978262192	7.84158259	8.505581591	8.68907177
ENSG00000083720	OXCT1	-1.654833767	0.192239251	-0.1389763	0.926455594	-1.793810068	6.138360429	7.538394143	9.61300511	6.727838783	6.89649843	7.81936672	8.654907296
ENSG00000151657	KIN	-0.313383138	0.501522547	-1.480710014	0.000144114	-1.794093153	7.384433967	7.60566235	7.691296561	7.437142666	7.05992818	6.208218574	6.066922748
ENSG00000105672	ETV2	-2.128048467	0.18838102	0.332250817	0.852228272	-1.795797651	4.00722531	4.93638933	2	2.868578001	2.77779907	3.878466556	4.766376438
ENSG00000166347	CYB5A	-1.443442522	0.011601873	-0.352763341	0.586489016	-1.796205864	8.946185348	7.790474954	8.042706615	6.941624891	6.9210099	7.827137031	8.148612304
ENSG00000144285	SCN1A	3.394729588	0.552383336	-5.191092634	0.364514502	-1.796363046	2	2	2	4.415698455	3.01487888	2	2
ENSG00000167740	CYB5D2	-0.862273979	0.361913214	-0.934191509	0.309378866	-1.796465488	8.16936288	7.790474954	9.849281147	8.006336225	8.07792246	7.795801481	8.128660152
ENSG00000083807	SLC27A5	-2.820952644	0.094280197	1.023609331	0.565006761	-1.797343314	6.366709671	4.11352228	2	2.868578001	3.2184422	5.85994354	6.202567011
ENSG00000162415	ZSWIM5	-1.468499382	0.491724432	-0.329676106	0.888119558	-1.798175489	2	4.710564154	8.143093325	5.128039203	5.49945122	6.499401217	6.257923755
ENSG00000170502	NUDT9	-0.690830579	0.061299815	-1.109018462	0.001305492	-1.799849041	9.031919741	8.903033433	9.149036566	8.390528966	8.29788471	7.679481638	8.148612304
ENSG00000165275	TRMT10B	-0.832699964	0.105432147	-0.967245941	0.050027637	-1.799945906	6.96351253	7.538394143	6.718950348	6.351046291	6.31350454	6.239610476	6.168983563
ENSG00000169087	HSPBAP1	-0.837565425	0.40748464	-0.962602854	0.322244282	-1.800168279	7.27616306	4.93638933	6.629488387	5.686138681	5.83486613	5.755231204	5.529673353
ENSG00000143498	TAF1A	0.0412019	0.98108975	-1.841725037	0.189314051	-1.800523137	3.327711234	7.051611315	5.59507254	6.087554739	6.00960265	4.82764224	3.735548302
ENSG00000100324	TAB1	-1.611230071	0.047807202	-0.189870024	0.844740034	-1.801100095	8.946185348	7.73146271	9.878698603	7.423326516	7.58663873	8.907307568	8.909973736
ENSG00000119778	ATAD2B	-0.942424713	0.592997115	-0.859171093	0.62358047	-1.801595807	5.330594134	5.595927442	9.589475645	7.261917051	7.20671394	7.64179387	6.89352185
ENSG00000235173	HGH1	-0.684259048	0.120319176	-1.117699968	0.00651667	-1.801959016	8.340423424	9.125613817	9.014156722	8.16211395	8.22297724	7.797560316	7.734322559
ENSG00000187713	TMEM203	-1.214164078	0.005219399	-0.593637129	0.200464134	-1.807801207	9.771132748	9.599860728	8.884815469	8.260583448	8.26953254	8.81307133	8.94213989
ENSG00000180611	MB21D2	-1.002166037	0.209002923	-0.806671869	0.311846327	-1.808837906	7.218819781	7.315436877	8.805342542	7.021746293	6.95938211	7.108998216	7.24894374
ENSG00000150433	TMEM218	-1.147782609	0.22267971	-0.661120463	0.496156922	-1.808903073	8.19931886	7.467835385	5.772716771	6.237202172	6.44777477	6.679970309	6.934072182
ENSG00000115286	NDUFS7	-0.565256653	0.328225936	-1.244312109	0.017698699	-1.809568763	8.816894989	8.791896851	7.734844356	7.90310317	8.04261444	7.309674888	7.308762629
ENSG00000186298	PPP1CC	-0.994512735	0.048767966	-0.815141233	0.108876124	-1.809653968	10.77778805	11.1798568	10.08627303	9.787831283	9.73059821	9.881520895	9.99254197
ENSG00000213066	FGFR1OP	-0.202638848	0.917642114	-1.607149943	0.338976671	-1.809788791	3.327711234	7.099070191	9.132850258	7.745471964	7.61696151	6.522981762	6.092227206
ENSG00000171853	TRAPPC12	-0.837503368	0.243555525	-0.972978461	0.160951705	-1.810481828	9.219014269	8.414402626	10.06083273	8.485003881	8.61275919	8.367944224	8.463180778
ENSG00000230778	ANKRD63	-3.753029279	NA	1.942520064	NA	-1.810509214	2	2	2	2	2	3.316666208	2.551896972
ENSG00000065183	WDR3	-0.480735168	0.749500183	-1.331216074	0.328380953	-1.811951243	4.815609471	8.834582582	9.149036566	7.994705029	7.96694292	7.20886319	7.085754446
ENSG00000137285	TUBB2B	0.626752522	0.478306674	-2.439446584	0.00124221	-1.812694061	8.797441463	7.669933291	6.958202216	8.744518279	8.49850989	5.304163849	5.953147751
ENSG00000042088	TDP1	-1.054720866	0.619674661	-0.758931177	0.72430187	-1.813652043	9.423197387	7.393647515	2	6.960881212	7.27676203	7.380620955	7.449681889
ENSG00000205710	C17orf107	-0.387943423	0.833035958	-1.426404567	0.376931525	-1.814347989	3.327711234	5.595927442	3.369317972	4.268917409	4.22485516	3.956110469	2.817676617
ENSG00000167850	CD300C	-3.753029357	NA	1.936281607	NA	-1.816747749	2	2	2	2	2	3.336159116	2.509591799
ENSG00000135297	MTO1	-1.378484603	0.309156734	-0.438427681	0.765406629	-1.816912284	9.304185368	7.761270542	5.156626401	6.809774134	6.90143415	7.875400346	7.661324972
ENSG00000168612	ZSWIM1	-1.365921434	0.421293524	-0.454337938	0.802964883	-1.820259372	5.095959458	5.303523747	9.287079172	6.664650034	6.3857277	7.427945125	7.399884913
ENSG00000016864	GLT8D1	-1.245262453	1.94E-08	-0.575269375	0.014854455	-1.820531827	10.22148166	10.03760521	10.06083273	8.88597028	8.85335127	9.592106979	9.475732243
ENSG00000134779	TPGS2	-1.338374893	0.007266635	-0.483255265	0.377517471	-1.821630158	10.61508061	9.994333773	10.95602907	9.260887574	9.21922971	9.779310259	10.34732599
ENSG00000130643	CALY	-2.165107267	0.363586907	0.343271464	0.896486957	-1.821835803	2	5.457120953	2	3.023133209	2.59489879	4.281782022	4.56420962
ENSG00000079459	FDFT1	-1.18789947	0.018486008	-0.635458807	0.233690382	-1.823358276	12.15999925	11.49353862	11.33422412	10.5227232	10.5229798	10.69695151	11.37275397
ENSG00000260001	TGFBR3L	-0.514941899	0.796317099	-1.309072932	0.470609658	-1.824014831	7.031698319	4.93638933	2	5.194621444	5.41912973	4.927401733	4.228274364
ENSG00000146842	TMEM209	-0.411593042	0.573609291	-1.412455249	0.027000298	-1.82404829	7.159102577	8.687092496	8.042706615	7.781117077	7.60490877	6.748859346	6.695462882
ENSG00000106803	SEC61B	-0.72445178	0.220626804	-1.099943045	0.048384016	-1.824394826	10.75779627	9.656475478	9.757248847	9.36574218	9.48312089	8.981908469	9.120072249
ENSG00000169403	PTAFR	-2.151126908	0.22333671	0.324202717	0.87135418	-1.826924191	2	4.93638933	6.203069917	3.566290607	3.39680241	6.098353065	4.279527956
ENSG00000171169	NAIF1	-1.049166523	0.292000423	-0.778471903	0.436962292	-1.827638426	8.586954094	7.099070191	6.203069917	6.604700657	6.61935067	6.732402829	7.000495137
ENSG00000167969	ECI1	-1.429353167	0.113104573	-0.399015997	0.689674098	-1.828369164	9.2903348	8.88960062	6.882775709	7.326437704	7.19470132	8.350677018	8.191057445
ENSG00000092098	RNF31	0.007444297	0.997041173	-1.835826586	0.188561332	-1.828382289	4.815609471	9.298208073	9.565556058	8.809693772	8.96600458	6.997118176	7.154291015
ENSG00000152219	ARL14EP	-1.221563461	0.117596105	-0.606885809	0.460026214	-1.828449269	9.317904228	7.503546079	8.408111533	7.539954763	7.21862736	8.191903518	7.757103546
ENSG00000160161	CILP2	-1.837861712	0.339484846	0.008464701	0.997302572	-1.829397011	2	4.93638933	7.600047325	4.329433326	4.811949	6.295446113	6.209190898
ENSG00000101940	WDR13	-1.342543079	0.004333387	-0.487120177	0.347421792	-1.829663256	9.048471759	10.02537381	9.589475645	8.31771326	8.24255817	8.960480054	9.277661801
ENSG00000179933	C14orf119	-0.807449987	0.17161955	-1.023492492	0.070716745	-1.830942479	10.787681	9.488434476	10.33998689	9.484432763	9.50355257	9.313509983	9.244188987
ENSG00000133627	ACTR3B	-1.178210712	0.468786454	-0.65333065	0.695048367	-1.831541363	6.366709671	5.838937808	2	4.574143677	4.53541753	5.338397457	4.56420962
ENSG00000082269	FAM135A	-0.87090656	0.346696909	-0.964609556	0.281743965	-1.835516116	7.435667355	7.60566235	5.59507254	6.442929658	6.11534392	6.468821191	5.867805474
ENSG00000117133	RPF1	-0.424422243	0.463796219	-1.411674819	0.004833789	-1.836097062	8.981092835	8.029695761	8.110401118	7.994705029	8.04261444	7.142599214	6.957866334
ENSG00000128581	IFT22	-0.9229347	0.098318255	-0.914520143	0.094766332	-1.837454843	7.667647223	8.762721501	8.380957451	7.398825229	7.47518984	7.240241221	7.625870264
ENSG00000162642	C1orf52	-0.576915297	0.323982764	-1.261414655	0.017661584	-1.838329952	9.48530526	8.317412869	9.06622548	8.50645854	8.40602977	7.903438544	7.650414739
ENSG00000125676	THOC2	-0.5500445	0.275627669	-1.288292303	0.004493423	-1.838336803	9.613202746	10.40166101	10.47451889	9.670650622	9.65954302	9.101707221	8.738561653
ENSG00000136715	SAP130	-0.912909408	0.232251697	-0.925610486	0.217050348	-1.838519894	9.423197387	8.169643011	9.999667074	8.413424144	8.52593541	8.356654601	8.553143267
ENSG00000136888	ATP6V1G1	-0.462434135	0.148344449	-1.376465231	9.97E-07	-1.838899366	11.05263482	10.91068521	11.39316998	10.6795094	10.6610729	9.751223945	9.76757159
ENSG00000156261	CCT8	-1.03424512	0.024637077	-0.805336308	0.085735367	-1.839581428	11.55070946	10.96932597	11.91252756	10.49945465	10.4901834	10.85352355	10.57977418
ENSG00000256053	APOPT1	-0.911483305	0.221454877	-0.9285398	0.203560175	-1.840023105	9.384607364	8.124485672	7.818184403	7.694616532	7.72670378	7.764669607	7.619668339
ENSG00000172590	MRPL52	-0.702207078	0.328545024	-1.138804281	0.090404285	-1.841011359	9.448362284	8.297207175	8.007629717	8.102673083	7.95512324	7.493431902	7.702986259
ENSG00000117859	OSBPL9	-0.711873687	0.160563675	-1.131309045	0.017373749	-1.843182732	10.87379128	10.40638494	9.999667074	9.728676049	9.79367921	9.588047701	9.050772231
ENSG00000118242	MREG	-0.357053667	0.844353006	-1.486456233	0.350123893	-1.8435099	5.866944937	3.686303414	7.97167868	6.268449691	6.51372389	5.899789217	4.434543419
ENSG00000242372	EIF6	-0.621665761	0.392541278	-1.222385348	0.066638754	-1.84405111	9.460782017	10.49775373	11.07092887	9.80724435	9.92248069	9.067128536	9.44519497
ENSG00000224051	CPTP	-1.539097829	9.08E-09	-0.306105539	0.320201587	-1.845203368	9.174465797	9.246456519	9.385998894	7.626911583	7.86198169	8.918665794	9.013414035
ENSG00000130772	MED18	-0.706958615	0.582810301	-1.141095955	0.346063415	-1.84805457	9.567995545	7.145017454	6.629488387	7.674344958	7.69009211	7.389977651	7.112501355
ENSG00000138085	ATRAID	-0.336040531	0.466780097	-1.512253766	9.42E-05	-1.848294297	10.78274301	10.03760521	10.26027233	10.02529042	10.0907323	9.003785277	8.764090674
ENSG00000103150	MLYCD	-1.037113817	0.620052795	-0.814047595	0.698497879	-1.851161412	9.189468652	7.189546432	2	6.882264028	6.95938211	7.118879607	7.156006254
ENSG00000175283	DOLK	-1.123616904	0.310065927	-0.728583383	0.519747981	-1.852200287	7.27616306	8.777382925	10.23774519	8.124184191	8.10812366	8.234076861	8.735123394
ENSG00000221909	FAM200A	-0.841016854	0.653037922	-1.012805232	0.576450932	-1.853822086	7.750913381	6.535001748	2	6.01571348	5.78232107	5.954417431	5.497565112
ENSG00000168067	MAP4K2	-1.000983521	0.239153967	-0.852857501	0.314114017	-1.853841022	8.075571721	7.002537973	9.031722608	7.388195945	7.14977896	7.264607048	7.557435494
ENSG00000141012	GALNS	-0.494248942	0.468067566	-1.359892289	0.024203174	-1.854141231	10.22148166	9.433952963	8.845626171	9.065699797	9.17099461	8.064761569	8.435907908
ENSG00000074582	BCS1L	-1.69898779	0.282799717	-0.155843521	0.932122597	-1.854831312	8.797441463	7.60566235	3.369317972	6.221320894	6.00960265	7.583859056	7.629578661
ENSG00000083812	ZNF324	-1.243644273	0.252882834	-0.61123652	0.591752395	-1.854880793	7.27616306	6.228547981	8.978369997	6.755670824	6.67805337	7.402744819	7.253757517
ENSG00000115233	PSMD14	0.031967913	0.959724553	-1.8871424	8.98E-05	-1.855174487	9.397585735	10.49332014	10.0085654	10.03951164	10.0971999	8.00283742	8.309838123
ENSG00000133313	CNDP2	-0.590511677	0.262050542	-1.264931992	0.008241509	-1.855443669	10.1770105	9.806969599	10.7325871	9.703174645	9.69550802	8.770720575	9.248214811
ENSG00000134910	STT3A	-0.751468199	0.004499435	-1.104750285	1.27E-05	-1.856218484	11.85353634	12.2341388	12.05891816	11.3321557	11.2822233	10.8829182	11.02321901
ENSG00000138162	TACC2	-1.598173952	0.05437861	-0.258072438	0.790020846	-1.85624639	8.228665467	7.315436877	9.413059136	7.101994243	6.88657603	8.242494717	8.376090431
ENSG00000104435	STMN2	3.334135715	0.560525946	-5.191092886	0.364514502	-1.856957171	2	2	2	3.95996233	3.69849104	2	2
ENSG00000267631	CGB1	3.333486812	0.560547313	-5.191092889	0.364514502	-1.857606077	2	2	2	3.881293667	3.7864959	2	2
ENSG00000196531	NACA	-0.733498575	0.01701725	-1.124448786	0.000113299	-1.857947361	12.08776766	11.6550774	11.65357938	11.03478106	11.1254767	10.65645596	10.72420814
ENSG00000126254	RBM42	-1.152010977	0.007139137	-0.706835124	0.113430713	-1.858846101	10.15425007	10.10308486	9.399592459	8.842507967	8.71722214	9.052840727	9.375131848
ENSG00000011243	AKAP8L	-0.827286911	0.365077256	-1.031794375	0.240278931	-1.859081286	10.6316532	8.317412869	10.02619908	9.067921572	9.16279627	8.967137431	8.857178832
ENSG00000160471	COX6B2	-2.71967371	0.053462108	0.859391647	0.53844608	-1.860282063	3.327711234	4.11352228	4.058998049	2.270786764	2.68924543	4.749835737	4.341135913
ENSG00000130702	LAMA5	-3.853562229	3.52E-10	1.992053188	0.001927913	-1.861509041	10.65885801	9.267380431	10.45512158	6.799114572	5.99120031	12.28712585	12.170995
ENSG00000149357	LAMTOR1	-0.76984554	0.077518916	-1.09352983	0.008257259	-1.863375371	10.11549941	10.31889309	9.788583153	9.277301907	9.37372144	8.661962194	9.281988193
ENSG00000176428	VPS37D	-1.854760975	0.163796432	-0.008709311	0.995654398	-1.863470286	4.00722531	5.595927442	7.552162999	4.887609717	4.4836996	6.566956211	6.155328188
ENSG00000172244	C5orf34	-0.082691058	0.959439303	-1.785125131	0.194134168	-1.867816189	5.330594134	5.72254323	2	4.984880435	4.95174433	3.753554863	3.496556178

ENSG00000183549	ACSM5	-1.066539995	0.683688476	-0.803253127	0.758322907	-1.869793122	5.095959458	2	2	2.868578001	3.33976443	3.1504466	3.383972333
ENSG00000213380	COG8	-0.882028892	0.216791462	-0.988367085	0.153226253	-1.870395977	9.720386367	8.213429689	8.721235202	8.15586117	8.14188248	8.248293824	7.805990373
ENSG00000081721	DUSP12	-0.323234139	0.539294232	-1.547424126	0.00046995	-1.870658265	8.075571721	7.467835385	8.35328246	7.714607209	7.64959655	6.417131325	6.569847194
ENSG00000169371	SNUPN	-0.437578912	0.7863872	-1.433249597	0.317845862	-1.870828509	7.709880939	8.806266215	4.058998049	7.447418636	7.32531744	6.372988335	6.476841503
ENSG00000100029	PES1	-0.856364746	0.130846679	-1.016250272	0.063605295	-1.872615018	9.556468883	9.566483142	10.49365885	9.085573664	9.09332116	8.656642182	9.161219358
ENSG00000111640	GAPDH	-0.392590719	0.588718348	-1.480409062	0.01853175	-1.87299978	15.9392534	14.99021973	14.54216152	14.85807235	14.9133335	13.50068265	14.04500215
ENSG00000157212	PAXIP1	-0.767301793	0.633945061	-1.106271385	0.471669087	-1.873573178	4.00722531	6.389896633	8.561105505	6.476767609	6.65485813	6.375345632	6.102937566
ENSG00000103343	ZNF174	-1.375675597	0.02259857	-0.498241116	0.448152433	-1.873916713	6.89194353	7.538394143	8.206335842	6.29903474	6.32089174	7.310906894	6.977399345
ENSG00000108733	PEX12	-0.778350223	0.68311016	-1.096247004	0.546129722	-1.874597227	7.667647223	7.847167909	2	6.387081877	6.49425375	6.401024833	5.821002687
ENSG00000066056	TIE1	-2.119341245	0.430098143	0.244499856	0.932012683	-1.874841389	3.327711234	3.076665209	2	2.270786764	2.2680111	3.534812207	2.277179783
ENSG00000160908	ZNF394	-0.79673613	0.468098579	-1.084585415	0.297503104	-1.881321545	8.009475818	6.844429755	9.565556058	7.77569021	7.75969386	7.430214384	7.539150216
ENSG00000188186	LAMTOR4	-1.081721227	0.109717472	-0.800664525	0.242561306	-1.882385752	8.696033109	8.124485672	9.636156974	7.826439377	7.93600799	8.061837451	8.250064747
ENSG00000167208	SNX20	-3.274145399	0.384580714	1.391574176	0.686186	-1.882571223	3.327711234	2	2	2	2.14022021	3.56816602	2.70970617
ENSG00000070540	WIPI1	-0.15575854	0.878154523	-1.727995187	0.032363747	-1.883753727	11.08909278	10.23108619	8.904018013	10.17037776	10.1777249	8.380906995	8.810568912
ENSG00000239264	TXNDC5	1.05421316	0.236638612	-2.938135257	0.000190879	-1.883922097	9.922475255	11.29641566	9.099922433	11.39964074	11.4928844	7.522436904	7.446882182
ENSG00000169193	CCDC126	-1.045011227	0.050710673	-0.838962517	0.114203801	-1.883973744	6.257048221	6.728495673	6.958202216	5.742861563	5.63601114	6.031172603	5.713290392
ENSG00000004478	FKBP4	-1.43220214	7.83E-05	-0.452418607	0.264775609	-1.884620746	10.62062599	10.019219	10.31150679	8.807036108	9.00894176	10.04184345	9.709771424
ENSG00000149636	DSN1	-0.871960099	0.680832734	-1.012728243	0.623122945	-1.884688342	2	6.728495673	8.865353883	6.739036112	6.73446058	6.830158212	6.329589667
ENSG00000109794	FAM149A	-1.215980497	0.444480686	-0.669010756	0.681460704	-1.884991253	3.327711234	3.686303414	6.203069917	3.921164147	4.05806467	4.749835737	3.987514168
ENSG00000124688	MAD2L1BP	-0.719692421	0.594639663	-1.165901893	0.359547024	-1.885594315	10.25752168	7.099070191	7.600047325	8.27796118	8.32935768	7.964991788	7.755972999
ENSG00000120314	WDR55	-0.969961853	0.119633373	-0.918549985	0.135132661	-1.888511838	9.667789716	8.732943944	8.35328246	8.069792811	8.06478301	8.015738956	8.213468956
ENSG00000138175	ARL3	-1.239095719	0.000196923	-0.650625843	0.064617229	-1.889721562	8.928409675	8.862353853	9.116480286	7.739909082	7.75424764	8.10153367	8.521558854
ENSG00000004534	RBM6	-0.806477441	0.209654079	-1.083807814	0.077031166	-1.890285255	9.751047882	9.549500232	10.79563557	9.374743937	9.29807478	8.870181261	9.230416542
ENSG00000105771	SMG9	-1.395311639	0.018810941	-0.496851551	0.446195114	-1.89216319	8.946185348	8.848535039	10.0085654	7.944803394	8.02463101	9.054312673	8.665783734
ENSG00000119977	TCTN3	-0.913533668	0.037504648	-0.978636098	0.023427954	-1.892169765	10.5468183	9.77810053	10.11951029	9.254074072	9.28868707	8.987312532	9.397416937
ENSG00000068745	IP6K2	-0.647920548	0.322658513	-1.244833036	0.038886992	-1.892753584	11.00254523	9.956182102	9.69246155	9.650037049	9.72504082	9.135128763	9.053075307
ENSG00000173465	SSSCA1	-0.955502843	0.134496548	-0.9398925	0.132964024	-1.895395343	8.468948322	8.029695761	7.029880454	6.989292186	7.06433609	6.986363353	7.096512788
ENSG00000139269	INHBE	-1.116606922	0.340882919	-0.779590624	0.501137387	-1.896197546	4.00722531	4.710564154	4.058998049	3.406930997	3.60476711	3.956110469	3.474731315
ENSG00000094841	UPRT	-0.745168249	0.725625854	-1.151607611	0.569190163	-1.89677586	9.143983763	7.35507212	2	7.084543139	7.31425651	6.603610344	6.990916783
ENSG00000132016	C19orf57	-2.94504289	0.251382472	1.047610924	0.686181755	-1.897431967	2	4.442710129	2	2.3892346	2.14022021	4.580376151	3.36036153
ENSG00000104388	RAB2A	-0.886813196	0.040278645	-1.010802116	0.016731085	-1.897615312	10.40620204	10.92062569	11.28380426	10.05191139	10.0067695	9.945980056	9.864906512
ENSG00000172766	NAA16	-0.526577523	0.743333936	-1.371565114	0.348530091	-1.898142637	4.815609471	5.595927442	8.511888463	6.682159616	6.68951263	6.162543904	5.540219242
ENSG00000075188	NUP37	-0.777194817	0.06885972	-1.121106679	0.005233988	-1.898301496	7.485143553	7.274681896	7.451345288	6.772115916	6.51372389	6.442083733	6.185873005
ENSG00000177946	CENPBD1	-1.084430391	0.200330183	-0.814021348	0.340884972	-1.89845174	8.16936288	6.535001748	8.585098385	6.936770382	6.91614081	7.309674888	7.056667034
ENSG00000065665	SEC61A2	-0.747878712	0.182596921	-1.15063918	0.028720091	-1.898517892	7.975256505	7.73146271	6.882775709	6.975156635	6.77267312	6.525106458	6.454714037
ENSG00000141696	P3H4	-1.669941213	0.000673737	-0.228679604	0.699954446	-1.898620817	11.43901855	11.08286144	10.32581712	9.362125691	9.35044873	10.93158439	10.63739192
ENSG00000104047	DTWD1	-0.00138869	0.998610961	-1.897269266	0.021362205	-1.898657956	8.981092835	7.051611315	8.65477198	8.504819462	8.35656898	6.977080688	6.033722345
ENSG00000188613	NANOS1	-2.831437171	0.110015664	0.932321797	0.616643045	-1.899115374	3.327711234	5.72254323	2	2.868578001	2.59489879	5.404520029	5.256659864
ENSG00000100387	RBX1	-0.493920466	0.421603569	-1.405330405	0.009454429	-1.899250871	9.820154628	8.687092496	9.031722608	8.747293529	8.78689171	7.669911566	8.041339497
ENSG00000166704	ZNF606	-0.985956364	0.524696221	-0.913941865	0.548117081	-1.899898228	3.327711234	7.701025996	7.600047325	5.968925393	6.32824132	6.57313027	5.772630506
ENSG00000213638	ADAT3	-0.58257636	0.735527035	-1.319123538	0.405619867	-1.901699898	6.653098118	5.72254323	2	5.22679461	5.14724905	4.727866721	4.353147976
ENSG00000139620	KANSL2	-0.795248293	0.244358561	-1.108355667	0.087576727	-1.90360396	9.460782017	8.213429689	8.206335842	7.952038749	7.98332947	7.758361797	7.554837457
ENSG00000119777	TMEM214	-1.401362596	0.00362723	-0.502360353	0.346019765	-1.903722949	12.14852894	11.26554928	11.21627664	10.1842688	10.2389602	11.03863895	11.17536771
ENSG00000166341	DCHS1	-0.943679579	0.498655693	-0.960605511	0.481615811	-1.90428509	8.716893051	10.34373336	6.203069917	8.287525788	8.2960118	8.102956788	8.428831018
ENSG00000166402	TUB	-2.144466826	3.26E-09	0.239949868	0.584589142	-1.904516958	8.892187472	9.357954881	8.904018013	6.975156635	6.95464102	9.501402587	9.092571273
ENSG00000136271	DDX56	-0.736825346	0.017838528	-1.170161114	6.48E-05	-1.906986459	9.435834703	9.806969599	9.452720984	8.861844103	8.83412012	8.322856568	8.509534124
ENSG00000114956	DGUOK	-1.070024587	0.174638808	-0.837455896	0.29039103	-1.907480482	10.56993315	9.04308532	8.941672936	8.655725489	8.66738547	8.806098644	8.973118091
ENSG00000175198	PCCA	-0.779731748	0.680833278	-1.127848164	0.531540246	-1.907579912	7.27616306	7.928237109	2	6.147608921	6.48112613	6.084012396	5.900914815
ENSG00000053900	ANAPC4	-0.492183521	0.415575369	-1.415558465	0.007718915	-1.907741986	7.485143553	7.274681896	8.325066185	7.190301983	7.36158648	6.363520437	6.412287469
ENSG00000163520	FBLN2	-3.418532982	1.39E-11	1.510735424	0.004914775	-1.907797558	10.29268328	10.50217373	11.45632039	7.450827758	7.45854177	12.37599757	12.32716121
ENSG00000108219	TSPAN14	-1.773409223	0.006990516	-0.135497237	0.868228993	-1.908906459	8.716893051	9.488434476	10.36791565	7.917958914	7.92635454	9.450887435	9.631178479
ENSG00000138777	PPA2	-0.555544611	0.597540846	-1.355171217	0.153080856	-1.910715828	9.497412313	7.572420273	7.342949392	7.939959586	7.93118934	7.283556699	7.010010317
ENSG00000166224	SGPL1	-1.794137607	2.30E-07	-0.11821586	0.790777785	-1.912353467	10.65885801	10.17128279	10.02619908	8.463225341	8.59001038	10.26657884	10.11575905
ENSG00000221878	PSG7	0.603075583	0.838765858	-2.516796132	0.336805988	-1.913720548	2	2	4.523675391	3.566290607	3.86943969	2.094598369	2.551896972
ENSG00000161243	FBXO27	-0.589699445	0.417837871	-1.325270232	0.042999625	-1.914969677	5.70932728	6.78762695	7.098165388	5.997179931	6.16544343	5.253805068	5.540219242
ENSG00000146414	SHPRH	-0.880006645	0.166330801	-1.035084258	0.091257767	-1.915090904	9.048471759	7.790474954	8.143093325	7.656739692	7.45182838	7.536196998	7.263337153
ENSG00000169750	RAC3	-2.500657914	0.009223953	0.585138567	0.59025161	-1.915519347	7.940205782	6.464272742	5.772716771	4.17310023	5.09650239	7.779874724	7.357281305
ENSG00000148300	REXO4	-1.09268405	0.265307052	-0.823981991	0.405601197	-1.916666042	8.493334308	7.790474954	10.11127249	8.083034979	8.02009991	8.547470747	8.042267212
ENSG00000143401	ANP32E	-0.797447389	0.152575674	-1.119685736	0.034280104	-1.917133125	8.946185348	10.13758676	9.809100877	8.925713487	8.91931898	8.610361892	8.596104698
ENSG00000168214	RBPJ	-0.590515183	0.181720064	-1.328608244	0.000859069	-1.919123427	10.40620204	11.03790484	10.42220168	10.04177408	10.0912724	9.508668537	9.131014727
ENSG00000073605	GSDMB	-1.830044684	0.396964364	-0.090831287	0.9703912	-1.920875971	2	6.04685525	9.031722608	5.959382894	5.77157844	8.11499695	6.556922154
ENSG00000116586	LAMTOR2	-0.618696954	0.474824893	-1.303975027	0.097545528	-1.922671981	7.904282243	8.029695761	9.577565424	7.985332082	8.21506984	7.31213785	7.543088047
ENSG00000124570	SERPINB6	-1.074575172	0.033835146	-0.848266152	0.098450928	-1.922841324	10.55263183	10.50217373	11.4530647	9.796566011	9.86931161	10.00616163	10.10957632
ENSG00000142235	LMTK3	-2.121366872	0.407875143	0.197112577	0.944880505	-1.924254295	2	2	6.322058544	3.349643765	3.2184422	4.875056259	5.171036168
ENSG00000135093	USP30	-1.130211699	0.001506743	-0.794161396	0.029318923	-1.924373095	8.468948322	8.053983481	8.110401118	7.202487431	7.0150921	7.52030827	7.352801367
ENSG00000130844	ZNF331	-1.416840574	0.203718914	-0.507976455	0.674475323	-1.924817029	5.70932728	8.762721501	8.486635645	6.658765962	6.85130017	7.89195932	7.339277475
ENSG00000099139	PCSK5	-1.593256233	0.373200768	-0.331608266	0.866860327	-1.924864498	2	7.431218269	5.772716771	4.825935136	4.83276841	6.118193494	5.799216178
ENSG00000175354	PTPN2	-0.947531645	0.046063656	-0.979592082	0.035498785	-1.927123727	8.075571721	8.147241016	8.742726175	7.344877929	7.48837134	7.138441638	7.599641096
ENSG00000155530	LRGUK	0.636138544	0.887753545	-2.563665455	NA	-1.927526911	2	3.076665209	2	2.947924294	2.68924543	2	2.225841198
ENSG00000125835	SNRPB	-0.813527426	0.322678131	-1.114675047	0.154104664	-1.928202473	11.56228205	10.00683004	9.868959282	9.843359791	9.93095363	9.558011928	9.617549359
ENSG00000102057	KCND1	-2.657562356	0.038689773	0.728421195	0.608329799	-1.92914116	3.327711234	6.140560021	7.225742779	3.997744386	3.90918929	6.566956211	7.263337153
ENSG00000068903	SIRT2	-1.313469899	0.046693782	-0.617051441	0.37875047	-1.93052134	10.20680973	8.820493869	8.99637432	8.246921218	8.10598728	8.809589199	8.922120243
ENSG00000100104	SRRD	-1.203941151	0.005734682	-0.727502136	0.107979874	-1.931443287	7.975256505	7.874700259	8.536706852	7.053486362	6.89154576	7.27600668	7.58443608
ENSG00000178184	PARD6G	-2.664378177	0.000246448	0.731394398	0.370750556	-1.932983778	8.517314938	6.899080544	8.845626171	5.720439317	5.72778967	9.047307518	8.987157359
ENSG00000077585	GPR137B	-0.800891219	0.39960716	-1.133445654	0.207913863	-1.934336873	9.410448395	7.274681896	7.858115942	7.731524332	7.6318872	7.36172352	7.352801367
ENSG00000173456	RNF26	-1.264579801	0.0026249	-0.670356175	0.132316489	-1.934935976	10.02639697	10.27307349	9.426401271	8.686416476	8.70739158	9.170171434	9.394150543
ENSG00000106400	ZNHIT1	-1.20987264	8.45E-05	-0.725385471	0.024667973	-1.935258112	10.24321353	9.962611189	10.40208303	8.902248225	9.10620439	9.378702006	9.591800868
ENSG00000187954	CYHR1	-1.508137045	0.075046884	-0.427647392	0.650098837	-1.935784437	10.75779627	8.916342327	8.978369997	8.325162455	8.32752521	9.457577544	9.334433107
ENSG00000165695	AK8	-2.37126688	0.525394621	0.435132908	0.911626073	-1.936133972	2	3.686303414	2	2	2.38539787	3.27687201	2.782576693
ENSG00000253250	C8orf88	-0.3318592	0.731338839	-1.604558181	0.051506371	-1.936417381	6.73718411	5.131603625	5.39244166	5.590471342	5.63601114	4.5389195	4.456980017
ENSG00000111581	NUP107	-0.554285875	0.461962192	-1.384164641	0.039291523	-1.938450516	8.586954094	7.051611315	8.486635645	7.608714138	7.66419009	6.78654324	6.870723585
ENSG00000204175	GPRIN2	-5.272940451	0.045443019	3.330987016	0.100713788	-1.941953435	2	4.710564154	2	2	2.14022021	6.295446113	6.476841503
ENSG00000018280	SLC11A1	-4.24482746	0.149345693	2.299669348	0.404282769	-1.945158112	2	4.710564154	2	2	2.2680111	6.013120342	4.30448772
ENSG00000089335	ZNF302	-0.434034974	0.537614614	-1.513098076	0.013738586	-1.947133051	9.448362284	8.356994601	8.486635645	8.486665614	8.34755546	7.679481638	6.944034147
ENSG00000103275	UBE2I	-0.902037226	0.183896625	-1.047486668	0.110655039	-1.949523894	11.0110152	9.470501606	10.76969939	9.623093601	9.68910013	9.52123003	9.502269352
ENSG00000174791	RIN1	-0.533806087	0.820701528	-1.418456857	0.506072054	-1.952262944	10.05943807	7.847167909	2	8.174538791	8.28849576	7.107581047	7.581886257
ENSG00000127837	AAMP	-0.82104526	0.145540049	-1.132305779	0.03494019	-1.953351039	10.98115017	9.923599762	10.05225195	9.524619869	9.6360458	9.157900392	9.377706707
ENSG00000176410	DNAJC30	-0.88937043	0.601416718	-1.064226363	0.518736842	-1.953596793	9.667789716	8.234834549	4.058998049	7.697489359	7.66709116	7.172727426	7.78508305
ENSG00000149541	B3GAT3	-0.791011956	0.335922911	-1.164846019	0.132955972	-1.955857975	9.98400217	8.487557258	10.45512158	9.038768132	9.09008229	8.821740064	8.553793978
ENSG00000148288	GBGT1	-1.303343492	0.399649306	-0.653388538	0.685289111	-1.95673203	5.095959458	7.393647515	3.369317972	4.927308402	5.0259382	5.638399518	5.475755622
ENSG00000174744	BRMS1	-0.675775574	0.530106276	-1.281779516	0.19506376	-1.957555089	7.218819781	8.169643011	9.767769415	8.040677544	8.15434112	7.315824427	7.669754031
ENSG00000196670	ZFP62	-1.226416917	0.230742948	-0.732799523	0.487153954	-1.95921644	7.867441304	6.389896633	9.014156722	7.026323566	6.79406287	7.648631242	7.085754446
ENSG00000104613	INTS10	-0.76503763	0.185014751	-1.195009111	0.027565187	-1.960046741	9.579430841	9.71095217	8.561105505	8.633396609	8.58848096	8.321634728	8.036691948
ENSG00000198873	GRK5	-1.871723471	0.022553657	-0.088691394	0.93161544	-1.960414865	8.39321512	7.393647515	9.553445883	6.856790872	6.88158913	8.568740544	8.663373824
ENSG00000122417	ODF2L	-0.596781482	0.416081529	-1.365221429	0.038915612	-1.96200291	7.867441304	7.467835385	8.941672936	7.650823203	7.62593544	6.812870125	6.963754056
ENSG00000179476	C14orf28	-1.253880973	0.34242241	-0.708223712	0.604288014	-1.962104685	4.00722531	7.431218269	7.818184403	6.061031724	5.74985018	6.548273902	6.298867018
ENSG00000214113	LYRM4	-1.365768996	7.59E-05	-0.596789914	0.107088128	-1.96255891	8.367060731	8.671479029	8.865353883	7.292639438	7.31795292	7.912394292	8.192729549
ENSG00000100968	NFATC4	-1.005263412	0.003443909	-0.957327373	0.005335787	-1.962590785	11.30078111	11.03180337	10.65536953	9.968745466	10.0651181	10.08064212	10.05017178
ENSG00000173436	MINOS1	-0.212613779	0.845989871	-1.750320965	0.050410355	-1.962934744	9.27634997	6.899080544	8.143093325	8.244958867	8.15020018	6.426254939	6.926052784
ENSG00000086598	TMED2	-1.012217447	2.18E-05	-0.950805551	6.72E-05	-1.963022999	12.57852557	12.38176406	12.74476087	11.56900979	11.5589968	11.61644135	11.63419556
ENSG00000123992	DNPEP	-0.480229876	0.398651947	-1.483936858	0.002818955	-1.964166734	9.521325812	9.835272298	10.57378414	9.573915313	9.5600488	8.411091064	8.714901913
ENSG00000185261	KIAA0825	0.05272122	0.976237425	-2.017397166	0.167574724	-1.964675947	6.009025997	4.710564154	2	5.040242897	5.00774437	3.500668998	3.36036153
ENSG00000168077	SCARA3	-3.153472278	0.027752249	1.188099738	0.447054694	-1.965372541	4.467311984	9.424669273	9.746650996	6.024891728	5.89549381	9.843290512	10.50603928
ENSG00000114757	PEX5L	0.610599278	0.812967163	-2.576535868	0.255841947	-1.96593659	2	2	5.156626401	4.205751501	4.37434574	2.560105256	2.466008509
ENSG00000171204	TMEM126B	-0.586816629	0.582509943	-1.379614378	0.153073981	-1.966431007	9.384607364	8.004992147	6.80318718	7.947219213	7.75424764	7.186216802	6.963754056
ENSG00000115649	CNPPD1	-1.415707947	0.175994512	-0.551669443	0.623961728	-1.96737739	10.71177538	8.077869079	8.784770914	8.207157886	8.2561084	9.130600759	9.042450579
ENSG00000100603	SNW1	-0.718507627	0.161771312	-1.249073198	0.008693809	-1.967580825	10.58135316	10.03150247	9.565556058	9.353955293	9.45234084	8.998824951	8.744273936

ENSG00000151131	C12orf45	-1.695968875	0.150440253	-0.272034196	0.839792588	-1.968003071	7.27616306	4.11352228	6.80318718	5.003571641	4.97065356	6.462183138	6.099376274
ENSG00000127452	FBXL12	-1.1462805	0.000234475	-0.821864184	0.00991729	-1.968144683	8.696033109	8.981105197	8.585098385	7.656739692	7.62295033	8.035995406	7.876348251
ENSG00000215009	ACSM4	-3.753031254	NA	1.784370277	NA	-1.968660976	2	2	2	2	2	3.296906311	2.374681325
ENSG00000239382	ALKBH6	0.412414357	0.777645763	-2.381867968	0.052961956	-1.969453611	7.975256505	6.78762695	4.058998049	7.311513918	7.44845993	4.521997428	4.998228167
ENSG00000168646	AXIN2	-2.414081402	0.132833397	0.44444572	0.80714548	-1.969635682	7.27616306	6.311476754	2	4.358765249	3.98554648	6.823267818	6.649933445
ENSG00000051128	HOMER3	-0.369714932	0.736493595	-1.600684686	0.091962344	-1.970399618	10.36053552	7.638155693	9.69246155	9.231457548	9.24566964	7.919680595	8.114615835
ENSG00000167130	DOLPP1	-1.236898213	0.002958911	-0.735048593	0.09054815	-1.971946806	7.904282243	8.655694736	8.561105505	7.194375246	7.20671394	7.604100461	7.772838868
ENSG00000137513	NARS2	-1.341770162	0.218538145	-0.630804851	0.582427016	-1.972575013	8.39321512	7.189546432	5.39244166	6.18083559	6.1322378	6.802396951	6.868633039
ENSG00000100353	EIF3D	-1.061939404	0.009987576	-0.91115098	0.028762582	-1.973090384	11.12854954	10.58784379	11.39994907	10.00745917	10.0249756	10.05219399	10.27241252
ENSG00000135736	CCDC102A	-2.186562086	9.68E-11	0.211994081	0.60541273	-1.974568005	8.418903792	8.451443573	8.865353883	6.314087501	6.58294721	8.776974697	8.820877391
ENSG00000139292	LGR5	-8.636823166	0.006111607	6.661981911	0.013890006	-1.974841255	6.563806279	2	2	2	2	10.36904805	12.22132013
ENSG00000178567	EPM2AIP1	-0.398020988	0.58791184	-1.5775621	0.012430339	-1.975583088	8.19931886	9.443177293	9.725218844	8.842507967	8.88359764	7.551912644	7.83304634
ENSG00000197747	S100A10	-1.159039485	0.074921525	-0.81711527	0.218712264	-1.976154755	12.07772327	11.08580931	11.37608156	10.43033406	10.4058602	10.08262707	11.2179681
ENSG00000057149	SERPINB3	3.212872721	0.576613399	-5.191093391	0.364514502	-1.97822067	2	2	2	3.754611435	3.74316441	2	2
ENSG00000142676	RPL11	-0.809746002	0.196949817	-1.168610324	0.049227778	-1.978356326	12.74263541	11.65309132	11.52304554	11.25454706	11.2935316	10.80407274	11.01980841
ENSG00000109016	DHRS7B	-1.202074434	0.00733148	-0.77661153	0.093798515	-1.978685964	8.042902253	8.414402626	8.941672936	7.29643414	7.36158648	7.808940772	7.679327321
ENSG00000124657	OR2B6	3.212167157	0.576690781	-5.191093394	0.364514502	-1.978926237	2	2	2	3.663521627	3.82856379	2	2
ENSG00000181789	COPG1	-0.780587023	0.038727587	-1.200326262	0.000760041	-1.980913285	12.45670208	11.94046379	12.39039575	11.47251976	11.5264191	10.84185164	11.2855366
ENSG00000108961	RANGRF	0.480266899	0.465481438	-2.462844644	8.00E-06	-1.982577745	6.257048221	6.951736442	7.163363831	7.300218887	7.31425651	4.841348851	4.279527956
ENSG00000134640	MTNR1B	-3.75303144	NA	1.769476796	NA	-1.983554643	2	2	2	2	2	2	3.45257121
ENSG00000087077	TRIP6	-1.476012375	0.016330246	-0.508032625	0.454394312	-1.984045001	11.30767305	9.863030423	10.48730704	9.162439556	9.24275568	10.0143146	10.30389101
ENSG00000121769	FABP3	-3.298042147	0.070855582	1.313496339	0.501916413	-1.984545808	8.228665467	6.899080544	2	3.840289928	4.43005853	7.128693779	9.102389799
ENSG00000141959	PFKL	-1.267501046	0.0402643	-0.717352625	0.267777028	-1.984853671	11.90215845	11.0102427	12.3188683	10.55990001	10.5883315	10.74069043	11.42517001
ENSG00000122483	CCDC18	0.158810538	0.884722082	-2.144114391	0.013198781	-1.985303853	4.467311984	4.93638933	6.203069917	5.602782483	5.43283104	3.663836244	3.620995423
ENSG00000129460	NGDN	-0.906034206	0.542476242	-1.081746589	0.453341798	-1.987780795	8.493334308	8.414402626	4.058998049	7.035434774	7.00595521	6.895669227	6.804465095
ENSG00000099341	PSMD8	-0.82996723	0.022676194	-1.157943885	0.000884741	-1.987911115	11.68380625	11.08286144	11.6648807	10.63112186	10.7139326	10.28393523	10.40562199
ENSG00000178814	OPLAH	-3.009742941	0.04127093	1.021064353	0.524825533	-1.988678589	3.327711234	8.124485672	6.073378139	4.070463721	4.31639476	7.608114885	8.141703677
ENSG00000213563	C8orf82	-1.290134976	0.066092631	-0.699127347	0.342120369	-1.989262323	8.609439662	8.356994601	9.868959282	7.842101511	7.81305436	8.330165956	8.485861839
ENSG00000178531	CTXN1	-2.198409884	0.015090069	0.208358998	0.850369177	-1.990050886	7.53297911	7.901717006	9.703463497	6.463327516	6.64900057	8.64301114	9.144469138
ENSG00000197128	ZNF772	-1.092459106	0.182231831	-0.897596521	0.271454037	-1.990055627	8.042902253	7.35507212	6.073378139	6.351046291	6.27599024	6.649130473	6.332626253
ENSG00000072818	ACAP1	-2.736342537	0.025398486	0.745383558	0.575961295	-1.990958978	6.563806279	4.93638933	3.369317972	3.227757282	3.45167086	6.262714117	6.102937566
ENSG00000109670	FBXW7	-1.00719304	0.163278075	-0.984068378	0.165345082	-1.991261418	7.975256505	8.557180776	9.553445883	7.834291698	7.86198169	8.075674526	7.632045641
ENSG00000125450	NUP85	-1.000862645	0.030910658	-0.991082597	0.030536043	-1.991945242	8.87372952	8.147241016	8.076950877	7.474468681	7.37584259	7.373563392	7.495109486
ENSG00000168758	SEMA4C	-0.883692972	0.47314822	-1.108498065	0.347715703	-1.992191037	9.204317094	6.311476754	9.636156974	8.063125866	8.04707549	8.124837479	7.469129172
ENSG00000173611	SCAI	-1.444114499	0.11039739	-0.548114876	0.572896533	-1.992229375	6.653098118	7.002537973	8.585098385	6.276156955	6.26836889	7.422256313	6.771278116
ENSG00000171960	PPIH	-0.38878686	0.790951603	-1.604295043	0.207863251	-1.993081903	7.159102577	6.140560021	3.369317972	5.829220145	5.88556448	4.813804156	4.720747915
ENSG00000100147	CCDC134	-0.892296184	0.639595086	-1.101677267	0.548817763	-1.993973451	7.53297911	5.595927442	2	5.527288686	5.41912973	5.163941955	5.396716407
ENSG00000175087	PDIK1L	-1.148355845	0.203854133	-0.845996768	0.352922604	-1.994352612	5.330594134	7.002537973	7.552162999	5.808110275	5.7929843	6.231826305	5.917188835
ENSG00000167306	MYO5B	-1.479266756	0.429843212	-0.517023685	0.793470518	-1.996290441	3.327711234	3.686303414	4.874587708	3.663521627	2.2680111	4.017766949	3.36036153
ENSG00000010270	STARD3NL	-0.509100469	0.545365418	-1.487630084	0.046421006	-1.996730552	10.55842202	9.030900487	8.960138144	9.244284335	9.17914662	8.100821584	8.372406522
ENSG00000030066	NUP160	-0.882853817	0.175376463	-1.114449466	0.074468007	-1.997303283	9.751047882	8.687092496	10.16797309	8.792330793	8.76404032	8.576444489	8.520893436
ENSG00000109133	TMEM33	-0.765805955	0.159553366	-1.23214525	0.015610198	-1.997951205	9.556468883	9.835272298	10.63252637	9.364838907	9.27258642	8.899548956	8.812201472
ENSG00000181191	PJA1	-1.358119156	0.024706507	-0.639844499	0.321669264	-1.997963655	9.940323746	9.090820894	8.536706852	7.927778434	7.99492122	8.677805528	8.663976679
ENSG00000159720	ATP6V0D1	-1.006531095	0.106307854	-0.991447946	0.106512505	-1.997979041	11.4883819	10.81799725	10.17589429	9.867156706	9.97493083	9.632330087	10.1885847
ENSG00000167645	YIF1B	-1.607826157	0.000819269	-0.390250763	0.479041989	-1.99807692	10.99402524	10.04973379	10.30429804	8.94033939	8.86981338	9.877784692	10.3208107
ENSG00000068354	TBC1D25	-1.473829905	0.080169719	-0.524547096	0.566749731	-1.998377002	7.904282243	8.655694736	9.972637969	7.574744049	7.70992146	8.397238383	8.740849281
ENSG00000142945	KIF2C	-1.200981895	0.579599436	-0.799393626	0.716536102	-2.000375521	3.327711234	6.389896633	2	4.139692846	3.98554648	3.632645806	4.870334174
ENSG00000197872	FAM49A	-1.509076804	0.269781523	-0.493173211	0.736281057	-2.002250015	5.866944937	5.72254323	3.369317972	4.237680128	3.90918929	5.485310992	3.956354764
ENSG00000105983	LMBR1	-1.261044467	0.012401427	-0.741569248	0.159452507	-2.002613715	9.751047882	9.624396729	8.721235202	8.235106869	8.13351654	8.546425131	8.836470732
ENSG00000157315	TMED6	-1.727636454	0.551856542	-0.276348822	0.930293595	-2.003985276	2	2	4.523675391	2.141737246	2.86123014	3.193831101	3.15612297
ENSG00000102805	CLN5	0.250672925	0.81413118	-2.255507857	0.008495313	-2.004834932	7.485143553	8.522788974	9.859153765	9.246247604	9.12952895	6.774090992	6.681239787
ENSG00000168439	STIP1	-1.100515019	0.064248308	-0.905100299	0.130055652	-2.005615318	11.81051487	10.63690565	10.72720655	10.04910261	10.0804323	10.15764151	10.35481313
ENSG00000181061	HIGD1A	-0.379279531	0.138339617	-1.626920767	1.55E-13	-2.006200298	10.27872131	10.27823701	10.28245311	9.979407193	9.81998493	8.748615145	8.57061027
ENSG00000130305	NSUN5	-0.078779429	0.927016036	-1.928094709	0.003735066	-2.006874138	8.928409675	7.701025996	7.451345288	8.098332103	8.10170499	6.442083733	6.151894053
ENSG00000138755	CXCL9	3.182066078	0.580892944	-5.191093519	0.364514502	-2.009027441	2	2	2	3.840289928	3.60476711	2	2
ENSG00000149489	ROM1	-1.111319894	0.543001285	-0.897762871	0.624398897	-2.009082765	2	5.303523747	7.098165388	4.887609717	4.91316568	5.294231737	4.845038551
ENSG00000198721	ECI2	-0.627232681	0.237182775	-1.383775969	0.003956363	-2.01100865	9.790941832	8.848535039	9.344432377	8.716468139	8.7882247	7.754744914	8.21922296
ENSG00000183763	TRAIP	-1.472449467	0.34408635	-0.539348743	0.738292369	-2.01179821	4.00722531	4.710564154	2	3.09461492	3.01487888	3.584558147	3.640730416
ENSG00000125089	SH3TC1	-0.698766396	0.666953311	-1.31327117	0.382062502	-2.012037566	3.327711234	4.442710129	6.80318718	4.825935136	5.04390545	4.698044417	4.077147165
ENSG00000175220	ARHGAP1	-1.531481834	1.13E-07	-0.483019712	0.132806015	-2.014501546	11.63532243	11.11207261	11.34831197	9.851120801	9.85411719	10.9107105	10.88487658
ENSG00000131828	PDHA1	-1.467941458	0.149386626	-0.549512242	0.617024792	-2.0174537	7.624139768	9.34816733	10.49365885	8.087422161	8.15434112	8.908528807	9.141874832
ENSG00000137692	DCUN1D5	-0.471660928	0.556356511	-1.546181213	0.028158563	-2.017842142	8.892187472	8.540087354	10.16797309	8.919575326	8.8960155	7.86621124	7.826599033
ENSG00000178974	FBXO34	-1.241719352	0.112781159	-0.779776816	0.333347859	-2.021496168	8.816894989	8.433041974	10.19940021	8.157948442	8.09740995	8.627767864	8.53941009
ENSG00000104472	CHRAC1	-1.282479556	0.208331946	-0.740011308	0.483045217	-2.022490864	8.19931886	7.315436877	9.839340503	7.467753569	7.65837038	7.871230734	8.295903454
ENSG00000072210	ALDH3A2	-1.548096601	6.14E-06	-0.475277218	0.213131367	-2.023373819	9.262228249	9.181799937	9.735974718	7.949630994	7.80517502	8.861379233	9.012940881
ENSG00000126581	BECN1	-0.363209869	0.535148173	-1.661106577	0.000789378	-2.024316446	10.46286899	9.624396729	9.601288346	9.608667949	9.57557184	8.017249226	8.545311752
ENSG00000183431	SF3A3	-0.820497454	0.112331583	-1.204477417	0.013717681	-2.024974871	10.15425007	9.318399851	9.242516611	8.744518279	8.88733423	8.325906648	8.541379994
ENSG00000106077	ABHD11	-1.31921711	0.299548911	-0.705984112	0.592201451	-2.025201222	5.532353208	8.317412869	5.930865648	5.870535235	5.93454154	5.988693817	6.849680541
ENSG00000128609	NDUFA5	-1.111050976	0.030289936	-0.914488795	0.076831332	-2.025539772	9.27634997	9.102512015	8.206335842	7.920420067	7.74878077	8.071319244	7.988411589
ENSG00000160993	ALKBH4	-1.361948653	0.169717282	-0.663618232	0.523865784	-2.025566885	5.866944937	7.701025996	8.561105505	6.34372989	6.50077304	7.086154752	7.107191496
ENSG00000170909	OSCAR	0.990917799	0.661870947	-3.016653633	0.142957636	-2.025735834	2	4.710564154	2	4.205751501	4.40247105	2.3070652	2.326754077
ENSG00000136942	RPL35	-0.661416994	0.198770347	-1.367240027	0.003541393	-2.028657021	11.83218596	10.79296235	11.5927499	10.81860745	10.7983847	10.12000692	10.08929747
ENSG00000112812	PRSS16	-1.268935106	NA	-0.760475137	NA	-2.029410243	2	3.076665209	2	2.3892346	2.14022021	2.457042231	2.277179783
ENSG00000141376	BCAS3	-1.237405045	0.018249043	-0.793043121	0.142869248	-2.030448166	8.009475818	8.297207175	8.978369997	7.03996889	7.46522407	7.594015257	7.803803816
ENSG00000142528	ZNF473	-1.294700994	0.347668061	-0.736398972	0.605488001	-2.031099966	4.467311984	7.467835385	8.631919287	6.351046291	6.40670796	7.164572841	6.612945848
ENSG00000075624	ACTB	-2.561100716	1.41E-11	0.529340817	0.227054772	-2.031759899	16.68863805	16.31538107	16.69915842	13.99575309	14.0391125	16.69559387	17.42791496
ENSG00000003756	RBM5	-0.641277527	0.370429303	-1.390936088	0.032616532	-2.032213616	9.410448395	10.42046463	10.93751085	9.781753035	9.70964668	9.249735981	8.700282474
ENSG00000170522	ELOVL6	-1.117788119	0.010240552	-0.915758966	0.037527109	-2.033547085	9.473095744	9.04308532	8.677268317	7.949630994	8.04037874	8.009682014	8.359066013
ENSG00000102100	SLC35A2	-1.07130572	0.024397609	-0.962953911	0.043346526	-2.034259631	10.05943807	9.055168103	9.735974718	8.559529208	8.65428713	8.708044758	8.721866969
ENSG00000120820	GLT8D2	-0.315322219	0.756395306	-1.719243	0.045247931	-2.034565219	8.340423424	6.899080544	9.149036566	8.020170999	8.13770558	6.387074688	6.975457902
ENSG00000183734	ASCL2	0.428702331	0.895301283	-2.464206963	NA	-2.035504632	2	2	3.369317972	2.784612485	2.68924543	2.183373728	2.059860178
ENSG00000128917	DLL4	-0.319249302	0.899437889	-1.717342599	0.435253955	-2.036586501	3.327711234	3.076665209	2	2.695456494	2.86123014	2.457042231	2.225841198
ENSG00000148143	ZNF462	-0.600667567	0.274064931	-1.437648543	0.003474236	-2.03831611	8.586954094	9.34816733	8.486635645	8.164192202	8.37442884	7.238947338	7.635738219
ENSG00000203705	TATDN3	-1.083216496	0.007764718	-0.957891001	0.017643691	-2.041107497	7.435667355	7.002537973	7.285536013	6.147608921	6.25300433	6.346800618	6.29264319
ENSG00000182816	KRTAP13-2	3.149850445	0.585258297	-5.191093653	0.364514502	-2.041243208	2	2	2	3.840289928	3.55551834	2	2
ENSG00000221826	PSG3	3.148744888	0.585407002	-5.191093658	0.364514502	-2.042348769	2	2	2	3.709785322	3.69849104	2	2
ENSG00000167565	SERTAD3	-1.42809821	0.019551277	-0.616855707	0.349687494	-2.044953918	9.544849385	8.169643011	9.439621147	7.748245381	7.76240929	8.508806077	8.5998903
ENSG00000147140	NONO	-1.291049643	4.44E-07	-0.756305654	0.004768505	-2.047355297	12.32703082	12.0991443	12.40057196	10.97654609	11.0056822	11.37866256	11.65799488
ENSG00000188107	EYS	-0.664692424	0.700085754	-1.382727378	0.380887317	-2.047419802	2	4.710564154	4.058998049	3.406930997	3.65239003	3.296906311	2.885414003
ENSG00000198939	ZFP2	-2.167560754	0.377617138	0.118927886	0.966296927	-2.048632868	2	5.457120953	2	2.600425781	3.01487888	4.604690117	3.771796761
ENSG00000174611	KY	-1.727915059	0.516230128	-0.32113025	0.912100129	-2.049045309	2	2	6.534109959	3.754611435	3.60476711	4.778616857	4.77533149
ENSG00000156970	BUB1B	-1.071492119	0.552001887	-0.977751614	0.582327511	-2.049243734	4.467311984	5.72254323	2	4.237680128	3.45167086	3.483289517	4.329022995
ENSG00000114098	ARMC8	-0.774435765	0.117793766	-1.275050434	0.005763848	-2.049486199	9.358295023	9.181799937	8.380957451	8.260583448	8.27524772	7.829717863	7.718159092
ENSG00000169635	HIC2	-1.176507782	0.479746549	-0.874612071	0.601817251	-2.051119853	3.327711234	8.717821467	7.225742779	6.503277904	6.42052719	6.872493235	6.620419619
ENSG00000092199	HNRNPC	-0.558270541	0.266356869	-1.493041497	0.000802417	-2.051312038	12.70528324	11.70386119	12.28996399	11.72268474	11.7403773	10.85595215	10.73830212
ENSG00000103365	GGA2	-0.90240295	0.059528826	-1.1499592	0.012605537	-2.05236215	10.65885801	9.856140801	9.907528187	9.265734736	9.31482024	8.857590434	9.210333843
ENSG00000111596	CNOT2	-0.634966291	0.347067822	-1.41750044	0.020707999	-2.05246673	10.41898845	10.51535307	9.099922449	9.504256136	9.5116449	8.765787532	8.695572791
ENSG00000108654	DDX5	-0.655566717	0.054833942	-1.398865024	7.33E-06	-2.054431741	13.38629018	13.41853031	13.71813493	12.86525712	12.8550256	11.86229406	12.33409073
ENSG00000183607	GKN2	-4.714890868	0.267434798	2.66031661	0.501315475	-2.054574258	2	2	3.369317972	2	2	4.68289886	2.885414003
ENSG00000168495	POLR3D	-1.717609067	0.018003832	-0.338580569	0.687802707	-2.056189636	10.96815916	9.267380431	9.767769415	8.499891024	8.45385092	10.07285649	9.57683272
ENSG00000161179	YDJC	-1.041348544	0.105982801	-1.015195056	0.108572393	-2.0565436	7.750913381	8.451443573	7.029880454	6.86703417	6.84106088	6.934553331	6.821857532
ENSG00000109265	KIAA1211	-2.116334345	0.364034842	0.059636281	0.982250031	-2.056698064	4.467311984	6.140560021	2	4.070463721	2.14022021	5.454427604	4.574506939
ENSG00000106682	EIF4H	-1.015186601	0.000770868	-1.041931615	0.000500148	-2.057118215	12.15618594	12.47802568	12.42404098	11.34526663	11.3460677	11.09943318	11.50951469
ENSG00000104356	POP1	-0.992270248	0.452776776	-1.065792066	0.405644179	-2.058062315	4.467311984	7.928237109	8.042706615	6.463327516	6.54560135	6.123812431	6.690737414
ENSG00000166260	COX11	-1.127625251	0.163112784	-0.93309944	0.248020572	-2.060724691	9.064836028	7.145017454	8.076950877	7.322721205	7.04662306	7.415399995	7.34530375
ENSG00000177058	SLC38A9	-0.349687697	0.623572187	-1.711284141	0.004335271	-2.060971838	7.750913381	7.099070191	8.143093325	7.409376773	7.33993465	6.405645065	5.621921006
ENSG00000113068	PFDN1	-0.971378036	0.195462641	-1.089744722	0.133624198	-2.061122758	10.27872131	9.975384048	8.561105505	8.7747563	8.84440862	8.435582605	8.91098986

ENSG00000113575	PPP2CA	-0.665488824	0.254264315	-1.395727582	0.008663163	-2.061216406	11.63532243	11.19631665	10.37481416	10.52191308	10.4662328	9.770381989	9.763076863
ENSG00000160352	ZNF714	-0.975262235	0.226076026	-1.088082269	0.161988102	-2.063344504	5.532353208	6.899080544	7.342949392	5.85002558	5.84514933	5.899789217	5.566251551
ENSG00000143590	EFNA3	-1.587282335	0.454602329	-0.47716361	0.834398737	-2.064445945	7.27616306	3.686303414	2	4.497095146	4.37434574	5.294231737	5.492143571
ENSG00000116752	BCAS2	-0.586929969	0.212730396	-1.478319932	0.000422063	-2.065249901	8.998234871	8.317412869	8.266922072	8.006336225	7.96458672	7.21150425	7.008112295
ENSG00000149557	FEZ1	-2.034280737	9.55E-08	-0.031277067	0.951751695	-2.065557804	9.317904228	9.214490408	9.878698603	7.447418636	7.52720828	9.312280199	9.605988
ENSG00000198755	RPL10A	-1.661080764	0.031352923	-0.405469398	0.643682096	-2.066550162	12.38357115	11.11207261	10.57976717	9.893075744	9.92187357	10.79562693	11.4501129
ENSG00000112592	TBP	-0.79419611	0.705469615	-1.272418549	0.522285759	-2.06661466	9.262228249	7.979858146	2	7.363085426	7.43830714	6.973973146	6.897628558
ENSG00000121152	NCAPH	-0.893962105	0.479054624	-1.173404366	0.324488998	-2.06736647	5.095959458	4.442710129	4.523675391	4.299492648	3.60476711	3.215043717	4.201948172
ENSG00000087237	CETP	-1.990290946	0.491841862	-0.077762142	0.979817504	-2.068053088	2	3.686303414	2	2.3892346	2.14022021	2.656294034	2.981406154
ENSG00000173156	RHOD	-1.890666282	0.000155088	-0.179074568	0.772803831	-2.06974085	8.564112515	8.255926468	9.272377397	6.877205252	6.92586261	8.758581951	8.374617996
ENSG00000100124	ANKRD54	-1.418971662	9.11E-07	-0.650911841	0.033763115	-2.069883503	8.674867122	8.487557258	8.845626171	7.311513918	7.23826702	8.028526139	8.027351693
ENSG00000049167	ERCC8	-0.762166898	0.354286428	-1.309121026	0.086247207	-2.071287924	5.70932728	7.145017454	7.552162999	6.229283386	6.29111322	5.880003936	5.601925227
ENSG00000116221	MRPL37	-1.325856072	7.64E-06	-0.747485644	0.016746626	-2.073341716	10.06758145	10.5327403	10.46808205	9.021676912	9.08249654	9.549165021	9.701279971
ENSG00000136404	TM6SF1	-2.961693707	0.292274211	0.887956545	0.753691694	-2.073737162	2	4.11352228	2	2.141737246	2.2680111	4.261535101	2.70970617
ENSG00000116133	DHCR24	-2.070992053	0.008138998	-0.003053784	0.997910957	-2.074045837	10.85509492	11.67869957	12.47629272	9.772927884	9.72990471	10.95954387	12.34756929
ENSG00000140264	SERF2	-1.216026267	0.024616474	-0.860464805	0.121912821	-2.076491072	13.48990769	12.30162123	12.79448541	11.70155491	11.7573216	11.92764893	12.22668595
C1ORF204	C1ORF204	4.076422354	0.127676681	-6.152937149	0.045223643	-2.076514795	2	2	3.369317972	5.242616075	5.21224859	2	2
ENSG00000127084	FGD3	-3.753032607	NA	1.675958486	NA	-2.077074121	2	2	2	2	2	3.296906311	2.225841198
ENSG00000148835	TAF5	-0.912185141	0.627312688	-1.165594607	0.519747981	-2.077779747	7.485143553	6.464272742	2	5.686138681	5.60003091	5.873348082	4.729989799
ENSG00000074071	MRPS34	-1.516297959	0.011057942	-0.561526914	0.39030005	-2.077824873	10.52923584	9.114109157	9.746650996	8.369067528	8.44034872	9.278673449	9.421147165
ENSG00000180964	TCEAL8	-1.541239587	0.068056382	-0.536616216	0.561193746	-2.077855804	10.91499272	9.090820894	9.132850258	8.545247918	8.35297033	9.352020423	9.53921146
ENSG00000164054	SHISA5	-0.959436447	0.11734453	-1.118874571	0.0593663	-2.078311018	13.0669361	11.78952816	12.18004629	11.45755782	11.5200149	11.12136697	11.51211186
ENSG00000177683	THAP5	-0.927878178	0.062310616	-1.151133898	0.01654823	-2.079012076	8.540903468	8.94259736	9.25752426	8.08961076	7.95749494	8.096541671	7.439859051
ENSG00000250486	FAM218A	-1.607501491	0.502887909	-0.473536614	0.85221514	-2.081038105	2	4.93638933	2	2.868578001	2.77779907	3.44788957	3.407202941
ENSG00000255423	EBLN2	-0.942267394	0.544448	-1.13905094	0.447209151	-2.081318334	6.009025997	5.72254323	2	4.694064037	4.3456612	4.301748724	4.434543419
ENSG00000205269	TMEM170B	-2.576016677	0.18345602	0.493845173	0.82055968	-2.082171504	5.095959458	2	7.502634626	4.329433326	3.45167086	6.503717356	6.791281771
ENSG00000174915	PTDSS2	0.36738411	0.750452376	-2.449982092	0.01054493	-2.082597982	6.468621203	9.181799937	7.646393253	8.565831351	8.5323134	5.747972572	5.892708443
ENSG00000162869	PPP1R21	-0.782631205	0.343443781	-1.300247477	0.091607485	-2.082878682	7.904282243	7.35507212	9.132850258	7.641902724	7.45854177	6.900587441	7.18317631
ENSG00000183283	DAZAP2	-0.989226305	0.008368835	-1.094293246	0.002981509	-2.083519551	12.13696669	11.5394185	12.0674595	10.93880439	10.9602832	10.65463759	11.0127263
ENSG00000087111	PIGS	-1.589815922	6.22E-05	-0.494181501	0.267250193	-2.083997423	11.03613077	10.63690565	11.47570169	9.546294576	9.46072474	10.53756755	10.65129185
ENSG00000134717	BTF3L4	-1.173861854	0.036724367	-0.910173715	0.109945258	-2.084035569	9.533135543	8.655694736	9.897982195	8.31397419	8.25031674	8.658579002	8.417435289
ENSG00000150967	ABCB9	-1.502092645	0.262309738	-0.582234456	0.685116055	-2.084327101	4.00722531	6.899080544	8.007629717	5.501219927	5.70538658	6.656902456	6.254725615
ENSG00000109065	NAT9	-1.377833883	5.90E-06	-0.709982111	0.027377975	-2.087815993	9.219014269	9.214490408	9.287079172	7.831679003	7.92150349	8.722467118	8.321348409
ENSG00000204920	ZNF155	-0.400395083	0.843607025	-1.69070491	0.340409749	-2.091099993	8.19931886	7.315436877	2	6.892328675	6.83591385	5.758846871	5.519049808
ENSG00000066117	SMARCD1	-1.109896217	0.014298064	-0.982413282	0.031125527	-2.092309499	10.09970294	9.632483534	10.5187901	8.979812467	9.06280517	8.969089651	9.308063313
ENSG00000091164	TXNL1	-0.298097527	0.345376097	-1.794255988	7.57E-12	-2.092353516	10.08373159	10.07961649	9.778213818	9.739170016	9.64416621	8.305655918	8.104880443
ENSG00000121766	ZCCHC17	-0.762343363	0.524515475	-1.330022907	0.230984425	-2.092366271	7.624139768	9.159587375	10.78531698	9.003220305	8.9229643	8.308125745	8.489267468
ENSG00000143801	PSEN2	-0.687317921	0.731439684	-1.405395592	0.450551283	-2.092713513	3.327711234	7.393647515	9.981704053	7.93509946	7.96694292	7.045287119	7.425709383
ENSG00000262473		-0.933462667	0.052647422	-1.162633663	0.012352962	-2.09609633	10.32020829	9.640525262	9.358421373	8.926937992	8.87860041	8.659546439	8.692030411
ENSG00000109534	GAR1	-0.600033088	0.484940021	-1.496291752	0.051515981	-2.09632484	7.53297911	6.04685525	7.934808822	6.693715713	6.85130017	5.9289675	5.917188835
ENSG00000066044	ELAVL1	-1.369092423	0.137045092	-0.728332207	0.449734118	-2.09742463	11.56516075	10.78209868	9.06622548	9.372948073	9.48558803	9.859588707	10.2503029
ENSG00000089053	ANAPC5	-1.207990897	0.000112284	-0.88961613	0.005607507	-2.097607027	10.83615308	10.69597594	11.24291424	9.734282329	9.74233706	10.0712216	10.03929326
ENSG00000130958	SLC35D2	-0.172280399	0.924945325	-1.927062991	0.205394441	-2.09934339	3.327711234	7.099070191	8.561105505	7.226553841	7.33264456	5.707383854	5.508347454
ENSG00000235961	PNMA6A	-1.639652215	0.377308259	-0.460154278	0.818792684	-2.099806493	4.00722531	6.389896633	2	3.997744386	3.60476711	4.920961476	4.543391831
ENSG00000112855	HARS2	-0.548037367	0.759077898	-1.552375832	0.336472054	-2.100413199	10.22148166	8.213429689	4.523675391	8.469961514	8.40081078	7.551912644	7.336254871
ENSG00000102760	RGCC	-1.434307064	0.092448935	-0.667325699	0.457819373	-2.101632764	5.70932728	7.701025996	7.552162999	5.978405189	5.72778967	6.768721236	6.371531035
STUB1		-0.76082784	0.292677052	-1.341444289	0.044959455	-2.102272129	9.344956677	9.267380431	10.66665689	9.222510184	9.08249654	8.582068017	8.575104441
ENSG00000181264	TMEM136	-1.056033762	0.23823472	-1.04675281	0.234600074	-2.102786571	9.112843761	8.029695761	7.029880454	7.19843704	7.32531744	7.580798201	6.879055607
ENSG00000117054	ACADM	-0.646657581	0.085171677	-1.456389229	1.70E-05	-2.103046809	8.797441463	8.916342327	8.380957451	8.047448792	8.11663771	7.182183216	7.388257299
ENSG00000110171	TRIM3	-1.82921755	5.58E-06	-0.274608862	0.566108671	-2.103826412	8.892187472	8.213429689	8.922968322	6.989292186	6.82556437	8.509342792	8.350852333
ENSG00000242173	ARHGDIG	-3.981249662	0.222165985	1.876685604	0.508679168	-2.104564058	2	3.686303414	2	2.141737246	2	4.350488971	3.518055793
ENSG00000242689	CNTF	0.083289726	0.976290713	-2.188022253	0.364514502	-2.104732527	5.70932728	2	2	4.623314311	4.0930011	2.987421008	2.817676617
ENSG00000205084	TMEM231	-0.809742842	0.353886558	-1.295976176	0.112305841	-2.105719018	8.540903468	6.899080544	6.882775709	6.83085985	6.91614081	6.334738131	6.476841503
ENSG00000182931	WFDC10B	0.975198196	0.745371929	-3.081618455	NA	-2.10642026	2	3.076665209	2	2.947924294	3.01487888	2.048074405	2.117335275
ENSG00000215021	PHB2	-1.287840505	0.00289076	-0.818717585	0.069605713	-2.10655809	11.21173326	10.70367235	10.30429804	9.504256136	9.50517467	9.879031168	10.05867839
ENSG00000123810	B9D2	-0.253584399	0.833633118	-1.856740859	0.061919051	-2.110325258	4.467311984	5.838937808	6.882775709	5.870535235	5.71663161	4.053534413	4.664017718
ENSG00000127314	RAP1B	0.466421923	0.210210321	-2.578299024	2.69E-16	-2.111877102	10.76282024	10.32389548	10.26027233	10.95839757	10.9039101	8.040458475	7.761616892
ENSG00000104880	ARHGEF18	0.463870764	0.790720929	-2.576151125	0.080743277	-2.112280361	9.521325812	8.053983481	4.058998049	8.873322599	8.85589616	6.004008792	5.812327524
ENSG00000138801	PAPSS1	-1.387634426	0.028439901	-0.72504218	0.278961643	-2.112676607	9.448362284	10.66477302	10.89014791	9.044420506	9.10940731	9.504636417	9.935263322
ENSG00000247315	ZCCHC3	-1.376204999	0.05194085	-0.737066541	0.321260238	-2.11327154	10.1076228	8.702538795	8.677268317	7.930222906	7.99953197	8.715506558	8.465949064
ENSG00000101134	DOK5	-1.754704169	0.370378063	-0.359515339	0.868498866	-2.114219509	2	8.004992147	5.772716771	5.003571641	5.18011485	5.692336538	6.845434848
ENSG00000205220	PSMB10	1.054921317	0.208618256	-3.171075496	1.82E-05	-2.116154179	8.981092835	8.077869079	6.958202216	9.20242622	9.34139676	4.927401733	5.436777225
ENSG00000149600	COMMD7	-1.718294834	4.31E-06	-0.397969727	0.35517853	-2.116264561	10.12333326	9.835272298	10.55568472	8.461536372	8.5227358	9.673139654	9.922471842
ENSG00000135698	MPHOSPH6	-0.18216611	0.834344332	-1.934319378	0.005059275	-2.116485488	8.313285031	8.169643011	6.629488387	7.745471964	7.65837038	5.846413283	6.165581817
ENSG00000162576	MXRA8	-1.176189101	0.063034875	-0.941350049	0.140177518	-2.11753915	12.38520102	11.02260251	11.83960343	10.64271979	10.7121781	10.55326835	11.19937456
ENSG00000158062	UBXN11	-1.349043937	0.351836711	-0.769002611	0.607673137	-2.118046548	3.327711234	7.35507212	7.691296561	5.786686927	5.62411716	6.129409568	6.362645675
ENSG00000142661	MYOM3	0.177060991	0.925027909	-2.295675093	0.140636706	-2.118614102	3.327711234	3.076665209	5.156626401	4.237680128	4.31639476	3.059555873	2.374681325
ENSG00000110011	DNAJC4	-1.322214058	0.029718519	-0.796436742	0.207938624	-2.118650799	9.410448395	8.834582582	7.97167868	7.539954763	7.56191311	8.156706039	7.971952287
ENSG00000154832	CXXC1	-0.913939358	0.508212783	-1.205838075	0.360288866	-2.119777433	10.09970294	7.761270542	6.43197674	7.90310317	8.02009991	7.710619897	7.640647
ENSG00000040633	PHF23	-1.10030544	0.082476467	-1.022478313	0.103484196	-2.122783753	10.9550501	9.558016677	10.04361983	9.170732795	9.24469897	9.153099991	9.406811858
ENSG00000254858	MPV17L2	-1.525100242	0.496398079	-0.599510505	0.800446891	-2.124610747	9.895281535	6.666836837	2	6.861921612	7.06433609	7.840848421	7.8990585
ENSG00000168884	TNIP2	-1.21430188	0.210376805	-0.912053614	0.352068654	-2.126355493	8.19931886	9.090820894	10.64399316	8.454760653	8.46722793	8.810024926	8.708487604
ENSG00000034677	RNF19A	-0.967481232	0.592445571	-1.158976143	0.507424606	-2.126457374	5.095959458	8.916342327	11.27276688	9.005540335	8.99284518	8.977648172	8.618670729
ENSG00000197157	SND1	-1.233975571	0.003720538	-0.892787473	0.041810498	-2.126763044	11.81293891	12.0227539	12.60360903	10.88941003	11.01539	11.11468069	11.45412679
ENSG00000148297	MED22	-1.249475396	0.167187365	-0.877479567	0.340137275	-2.126954964	10.49341471	8.607278467	8.608698767	8.260583448	8.32385328	8.500191379	8.804567006
ENSG00000164484	TMEM200A	-0.241635679	0.841373243	-1.885785413	0.056612408	-2.127421092	8.042902253	10.73780697	10.48092713	9.948991943	9.86300004	7.734688324	8.667588528
ENSG00000161526	SAP30BP	-0.677381007	0.194794952	-1.452407411	0.002155456	-2.129788418	10.36053552	9.599860728	9.465702942	9.129845271	9.24857772	8.234076861	8.589138522
ENSG00000128408	RIBC2	-0.95464234	NA	-1.175904597	NA	-2.130546937	2	3.076665209	2	2.141737246	2.49394783	2.139668783	2.421067459
ENSG00000171443	ZNF524	-1.467514393	0.494956066	-0.66327148	0.768414094	-2.130785873	9.233563228	6.311476754	2	6.456560224	6.36443784	7.399274054	6.936070085
ENSG00000099821	POLRMT	-1.749952438	0.000101449	-0.381087311	0.46434877	-2.13103975	9.751047882	8.834582582	9.358421373	7.632926745	7.62593544	9.154129994	8.782528961
ENSG00000096384	HSP90AB1	-1.222249147	0.015366572	-0.909472281	0.078322326	-2.131721428	14.27259948	13.49148113	13.23355721	12.49274548	12.5359925	12.69454834	12.94852295
ENSG00000162244	RPL29	-1.350051289	0.003642609	-0.783522785	0.109386471	-2.133574074	13.06388636	12.15494759	12.24549183	11.14279746	11.2554874	11.78466647	11.74703401
ENSG00000062485	CS	-1.569986492	1.29E-06	-0.563878443	0.114873438	-2.133864935	11.41687936	10.91068521	11.12511995	9.613999459	9.58557298	10.41187535	10.77042905
ENSG00000103507	BCKDK	-1.484445057	0.016810729	-0.650323288	0.331375959	-2.134768345	10.92846914	10.09725346	9.541233192	8.777474042	8.87358581	9.375172844	9.889085869
ENSG00000213366	GSTM2	-0.111145106	0.907490514	-2.023794363	0.005993894	-2.134939468	6.73718411	8.213429689	8.677268317	7.964017617	7.9809999	5.944926241	6.280114403
ENSG00000049089	COL9A2	-1.897603495	0.314701066	-0.237350424	0.911000084	-2.134953919	2	5.457120953	3.369317972	2.868578001	3.2184422	4.45225155	3.735548302
ENSG00000119231	SENP5	-1.098278025	0.099672705	-1.037220261	0.115817438	-2.135498287	9.992581382	10.19331737	11.37608156	9.587142668	9.53803206	9.743945072	9.492120456
ENSG00000112306	RPS12	-0.604811495	0.391805366	-1.532989412	0.015161888	-2.137800906	11.88152073	10.60848937	10.55568472	10.54322901	10.558176	9.584995729	9.665902758
ENSG00000196150	ZNF250	-1.519515677	0.065184591	-0.621080581	0.482146792	-2.140596258	8.468948322	7.538394143	6.43197674	6.260701031	6.21386141	7.314596615	6.847559257
ENSG00000164414	SLC35A1	-0.840710877	0.689206562	-1.300547699	0.514552506	-2.141258576	9.081016759	8.077869079	2	7.222570549	7.30311013	7.269684675	6.155328188
ENSG00000065029	ZNF76	-1.300075503	0.479762551	-0.841719437	0.654382498	-2.14179494	9.839306291	7.73146271	3.369317972	7.226553841	7.35080119	7.597048228	7.866953664
ENSG00000105254	TBCB	-1.371426893	0.001908478	-0.770442772	0.098635116	-2.141869665	10.47516499	9.916994052	10.48092713	8.878394985	9.01464729	9.169152824	9.843245622
ENSG00000171307	ZDHHC16	-1.241774201	0.025049412	-0.900945411	0.112157708	-2.142719611	9.031919741	9.770792087	10.30429804	8.596935617	8.52113334	8.808281231	8.980880783
ENSG00000166313	APBB1	-1.353908123	0.119684351	-0.790396134	0.380413475	-2.144304256	9.602033055	9.192779271	11.18521898	8.866956935	8.9648262	9.215609012	9.697162691
ENSG00000151640	DPYSL4	-1.24937098	0.3854131	-0.89508524	0.538134884	-2.14445622	9.740899611	8.590771778	5.156626401	7.494428494	7.51115308	7.585896024	8.07345976
ENSG00000035141	FAM136A	-1.302371541	0.318470503	-0.842336971	0.528809949	-2.144708512	10.52332707	9.424669273	6.43197674	8.323303759	8.18704757	8.685424964	8.737416477
ENSG00000117543	DPH5	-1.556251877	0.003880482	-0.588969591	0.313919617	-2.145221468	7.867441304	7.701025996	6.958202216	5.949776857	6.17362678	7.228554289	6.704867638
ENSG00000007376	RPUSD1	-1.274345095	0.103167922	-0.870968929	0.274579713	-2.145314024	9.48530526	8.806266215	7.502634626	7.474468681	7.62891439	8.008923105	7.887748062
ENSG00000166343	MSS51	-1.183391095	0.215393228	-0.962760677	0.305318653	-2.146151773	4.467311984	5.838937808	4.874587708	4.268917409	4.16043394	4.588526421	4.161535617
ENSG00000149150	SLC43A1	-0.858469304	0.64998964	-1.289995795	0.473584699	-2.148465099	6.468621203	7.790474954	2	5.949776857	5.85535975	5.744329515	5.217771644
ENSG00000115758	ODC1	-0.1931383	0.754387465	-1.955509775	6.32E-05	-2.148648075	11.13631304	10.45735223	9.981704053	10.40463465	10.4171049	8.641053292	8.675981178
ENSG00000115866	DARS	-0.575199767	0.146453455	-1.573767851	7.33E-06	-2.148967618	10.29268328	10.15453315	10.79563557	9.896204982	9.83554116	8.958516141	8.783638895
ENSG00000099250	NRP1	0.958570297	0.326219261	-3.107835333	0.000254856	-2.149265036	9.949165863	11.13502303	12.32065573	12.39824131	12.4025576	8.72385521	7.845855157
ENSG00000147536	GINS4	-0.626037056	0.667279239	-1.524142648	0.247374227	-2.150179704	4.467311984	6.04685525	3.369317972	4.299492648	4.74762503	3.500668998	4.119958326
ENSG00000167461	RAB8A	0.088773946	0.88983543	-2.240286826	2.25E-06	-2.151512881	9.189468652	9.981728319	10.18377222	9.928352931	9.93817677	7.371203179	7.845855157
ENSG00000165392	WRN	-0.774610683	0.262822363	-1.378502184	0.031613028	-2.153112867	8.855032352	7.35507212	8.206335842	7.471115032	7.52080763	7.037865927	6.76679494
ENSG00000159111	MRPL10	-1.186228999	0.069674535	-0.966967751	0.140757366	-2.153196751	9.751047882	8.862353853	8.236946975	7.954442493	7.86703672	8.131128273	8.123409541

ENSG00000134480	CCNH	-0.718925031	0.235186197	-1.435721066	0.009577248	-2.154646097	7.579279349	8.557180776	8.865353883	7.66556918	7.77591026	7.0047514	7.040043908
ENSG00000142657	PGD	-0.571217333	0.282839894	-1.583470171	0.000737309	-2.154687504	11.94035323	11.46212086	11.83710798	11.15721618	11.2218486	9.63035768	10.57737611
ENSG00000282291		-1.044444823	0.227473008	-1.110945777	0.187955191	-2.1553906	8.564112515	6.78762695	8.904018013	7.377487766	7.22257674	7.240241221	7.234405279
ENSG00000113119	TMCO6	-1.573620583	0.469993356	-0.583918864	0.799962733	-2.157539447	3.327711234	7.099070191	2	4.237680128	4.37434574	5.258920789	5.020995508
ENSG00000170801	HTRA3	-1.243059234	0.534490579	-0.914742312	0.65200599	-2.157801546	2	8.540087354	6.629488387	6.078767801	6.1572134	6.710164669	6.081436739
ENSG00000105426	PTPRS	-1.232478259	0.021674606	-0.926066976	0.091407	-2.158545235	11.23361136	10.05576005	11.06240811	9.621581886	9.65662671	9.993749244	9.892948091
ENSG00000163521	GLB1L	-1.654111368	0.11657228	-0.50465664	0.664628785	-2.158768009	6.257048221	7.701025996	8.742726175	6.351046291	6.22177541	6.544089217	7.928458732
ENSG00000168209	DDIT4	-0.079418835	0.946595613	-2.079863715	0.030994241	-2.159282551	9.975371636	10.18234215	8.677268317	9.662587523	9.67547818	8.413960373	6.165581817
ENSG00000116251	RPL22	-0.566414944	0.43599311	-1.593151683	0.013079078	-2.159566628	11.37155492	10.42046463	9.798878489	10.1342586	10.0891109	9.029831415	9.148782641
ENSG00000176473	WDR25	-1.056627003	0.237601833	-1.104091482	0.206132822	-2.160718485	8.517314938	7.393647515	6.322058544	6.682159616	6.61935067	6.645228724	6.564691065
ENSG00000121064	SCPEP1	-0.088973988	0.866613986	-2.072256591	1.22E-07	-2.161230579	10.66423795	10.08551947	10.55568472	10.35409783	10.3773587	8.072772466	8.659146771
ENSG00000175764	TTLL11	-0.907893093	0.495809834	-1.254964516	0.320805825	-2.162857609	4.00722531	7.35507212	7.646393253	6.130704144	6.1068222	5.610655473	5.957088417
ENSG00000063176	SPHK2	-1.447991278	0.000620294	-0.714922697	0.112037873	-2.162913975	8.653385977	8.356994601	8.941672936	7.127781374	7.34718809	7.689935911	8.186868687
ENSG00000147862	NFIB	-4.361180572	0.003309136	2.19708251	0.15962782	-2.164098062	6.138360429	7.099070191	8.511888463	4.443344104	2.14022021	9.475816969	9.976372702
ENSG00000163512	AZI2	0.054463863	0.954731956	-2.218968904	0.003108279	-2.164505042	10.83137851	9.04308532	9.301632639	10.02414665	9.9946957	7.633939843	7.871136591
ENSG00000101751	POLI	-1.218655126	0.171886783	-0.946001939	0.291093097	-2.164657065	7.709880939	7.928237109	9.589475645	7.477814553	7.45854177	7.989819469	7.425709383
ENSG00000215271	HOMEZ	-1.327430678	0.13908327	-0.837653451	0.363335037	-2.165084129	6.468621203	8.191702463	8.825624954	6.841288255	6.80463998	7.413107295	7.173047408
ENSG00000172425	TTC36	-1.412364034	0.617411375	-0.752734138	NA	-2.165098172	2	3.686303414	2	2.498691189	2.2680111	2.592885947	2.551896972
ENSG00000185513	L3MBTL1	-1.738756165	0.178468688	-0.427415494	0.765367121	-2.166171659	6.257048221	4.11352228	7.502634626	4.443344104	5.2591458	6.265258498	5.913137508
ENSG00000166575	TMEM135	-0.532039691	0.283678293	-1.635024255	0.000157738	-2.167063946	8.257427002	8.929529568	8.266922072	8.051945366	7.95274764	6.730562686	7.09829807
ENSG00000064309	CDON	-1.555266634	0.261480902	-0.613220349	0.677268519	-2.168486983	3.327711234	5.457120953	6.322058544	4.647285135	3.39680241	5.026717879	4.827924991
ENSG00000122882	ECD	-1.018024708	0.148427006	-1.15249115	0.091654931	-2.170515859	9.247966931	8.255926468	9.849281147	8.16211395	8.32385328	7.877064822	8.31522102
ENSG00000142694	EVA1B	-0.559275813	0.387605511	-1.611769558	0.004649784	-2.171045371	10.45668146	9.192779271	10.41552659	9.556615538	9.58019637	8.417967842	8.623017875
ENSG00000141933	TPGS1	-1.054944753	0.309968682	-1.116114634	0.269817196	-2.171059387	6.138360429	7.503546079	8.805342542	6.766655021	6.89649843	6.875826972	6.664466987
ENSG00000162191	UBXN1	-1.057926716	0.118824374	-1.113533801	0.093533047	-2.171460516	10.49341471	9.405920729	9.014156722	8.738951712	8.71722214	8.568225482	8.770822803
ENSG00000145916	RMND5B	-2.11045593	0.212730396	-0.061225755	0.974934399	-2.171681685	9.790941832	8.57407404	3.369317972	6.554880361	6.77267312	8.771615683	8.568035872
ENSG00000140030	GPR65	-1.293666759	0.379713551	-0.878455955	0.547274868	-2.172122714	4.00722531	3.686303414	4.523675391	3.162721474	3.28037825	3.904814898	2.851942824
ENSG00000181991	MRPS11	-0.680881651	0.487705929	-1.49432213	0.093525732	-2.175203781	9.497412313	8.777382925	7.098165388	8.033874364	8.13561258	7.001702955	7.52727182
ENSG00000164118	CEP44	-1.345880238	0.200330183	-0.829846219	0.442768139	-2.175726457	7.27616306	8.806266215	6.322058544	6.554880361	6.507263	7.406207255	6.514766253
ENSG00000204524	ZNF805	-0.664555185	0.319838395	-1.513398195	0.012183874	-2.17795338	8.340423424	7.874700259	7.029880454	7.173892946	7.21862736	6.609629839	6.102937566
ENSG00000196411	EPHB4	-1.5359722	0.007257564	-0.642205806	0.299826316	-2.178178006	9.761125267	8.981105197	10.34701993	8.300810597	8.24644267	9.224134997	9.090778885
ENSG00000124467	PSG8	3.010841338	0.603581513	-5.191094232	0.364514502	-2.180252894	2	2	2	3.566290607	3.65239003	2	2
ENSG00000103051	COG4	-1.312708597	0.133069745	-0.868260095	0.332318898	-2.180968692	10.82658808	9.599860728	8.585098385	8.649804831	8.64989445	8.967137431	9.204129769
ENSG00000103145	HCFC1R1	-1.994206651	8.69E-06	-0.188420544	0.733788685	-2.182627196	9.304185368	8.356994601	8.65477198	6.841288255	6.89154576	8.645942939	8.62735196
ENSG00000178057	NDUFAF3	-1.131240099	0.005333784	-1.052346434	0.009656574	-2.183586533	10.38028415	9.842262059	9.809100877	8.890998469	8.92781046	8.73399394	9.203715214
ENSG00000166562	SEC11C	0.378295764	0.642216602	-2.564401426	0.000116198	-2.186105662	9.143983763	7.819099893	8.206335842	8.888486565	8.85589616	5.467744375	6.403650353
ENSG00000206562	METTL6	-0.976484232	0.310604101	-1.210496329	0.189811454	-2.186980561	7.096806373	7.928237109	9.372276026	7.501020868	7.45518898	7.452712791	7.013798886
ENSG00000137720	C11orf1	-0.740990951	0.467848857	-1.447401486	0.120494972	-2.188392437	6.009025997	5.303523747	7.398165132	5.818703821	5.76075522	4.946551782	5.32534152
ENSG00000122965	RBM19	-0.570828713	0.733731931	-1.618292414	0.283648588	-2.189121127	10.29268328	9.308339287	4.874587708	8.677714042	8.82635531	7.634923938	7.80161394
ENSG00000274229		0.214527813	0.881720015	-2.403755015	0.036373066	-2.189227202	5.532353208	5.838937808	8.35328246	7.377487766	7.35440525	5.008609438	4.819291477
ENSG00000160194	NDUFV3	-1.999244895	0.002312454	-0.190030931	0.814643023	-2.189275827	10.13887448	8.451443573	9.330306409	7.487805859	7.48837134	9.258701797	9.289430628
ENSG00000155275	TRMT44	-1.943957136	0.248222521	-0.246613369	0.899287217	-2.190570505	4.00722531	6.140560021	9.227351203	5.764940652	6.1322378	7.66606569	7.513957873
ENSG00000141179	PCTP	-0.939198182	0.259291944	-1.251524989	0.113966986	-2.190723172	6.366709671	6.951736442	8.143093325	6.429169081	6.44101097	6.327451929	5.921228818
ENSG00000189056	RELN	1.263108008	0.42279064	-3.454829089	0.01363715	-2.191721081	9.143983763	5.303523747	5.930865648	8.984524517	9.09332116	5.014670875	3.940519005
ENSG00000129055	ANAPC13	-1.203518796	0.036049949	-0.988459454	0.088195247	-2.191978251	11.18578229	10.01303782	10.19160738	9.404936476	9.3166689	9.480751596	9.664095852
ENSG00000100429	HDAC10	-0.161779528	0.88968542	-2.031065003	0.028205751	-2.19284453	7.867441304	5.946638857	8.511888463	7.494428494	7.72670378	5.634468599	5.98823215
ENSG00000006282	SPATA20	-1.329637576	0.053270494	-0.864457185	0.222891601	-2.194094761	9.790941832	9.828248506	11.15347798	9.069031178	9.09547637	9.304879394	9.750075969
ENSG00000182103	FAM181B	-3.756860774	0.095646206	1.559671051	0.467249874	-2.197189723	2	4.442710129	3.369317972	2.3892346	2	4.996409624	4.664017718
ENSG00000124659	TBCC	-0.903545994	0.343732241	-1.29566471	0.151114596	-2.199210703	8.228665467	8.297207175	10.18377222	8.270263574	8.35477078	7.733770002	8.096865967
ENSG00000122386	ZNF205	-1.579350541	0.470709201	-0.620189124	0.78961933	-2.199539665	9.304185368	6.04685525	2	6.172600389	6.48770488	7.446000032	7.052989523
ENSG00000110799	VWF	-1.337647337	0.496719668	-0.864052557	0.663337914	-2.201699894	4.815609471	2	4.058998049	2.600425781	3.50452879	3.215043717	3.560117069
ENSG00000232258	TMEM114	-3.753034173	NA	1.550617918	NA	-2.202416255	2	2	2	2	2	3.316666208	2
ENSG00000164615	CAMLG	-1.394804046	0.000720759	-0.808152988	0.062311706	-2.202957033	9.839306291	9.214490408	9.385998894	8.143273718	8.09740995	8.930336153	8.425990514
ENSG00000163961	RNF168	-1.413858365	0.252244014	-0.789123127	0.538029429	-2.202981491	7.82963488	7.928237109	10.71094342	7.992367495	8.16464176	8.636147016	8.757891838
ENSG00000128254	C22orf24	-2.272289522	NA	0.068762586	NA	-2.203526936	2	3.076665209	2	2.141737246	2.14022021	2.3070652	2.851942824
C4ORF32	C4ORF32	3.946973061	0.156616494	-6.152935894	0.050696253	-2.205962832	2	3.076665209	2	5.144975053	5.07918227	2	2
ENSG00000112237	CCNC	-0.986314438	0.013722176	-1.220837991	0.001608419	-2.20715243	9.613202746	9.114109157	8.884815469	8.22518713	8.29225867	8.102245405	7.949380652
ENSG00000145494	NDUFS6	-1.03437552	0.115157031	-1.172807772	0.065682577	-2.207183292	9.820154628	8.671479029	8.608698767	8.136938566	8.10812366	7.728247761	8.205208936
ENSG00000125648	SLC25A23	-2.797117806	0.000187015	0.589446427	0.498291593	-2.207671379	9.159305284	9.488434476	10.94679967	7.307758725	7.33264456	10.32871658	10.95206949
ENSG00000185085	INTS5	-1.568545663	0.465126545	-0.639656873	0.778107221	-2.208202537	2	8.820493869	10.26027233	7.647855834	7.51437845	8.483352582	8.510875165
ENSG00000188807	TMEM201	-1.547629512	0.34368375	-0.660791586	0.702769774	-2.208421098	4.00722531	7.761270542	9.577565424	6.658765962	7.03768431	7.759264603	7.688837505
ENSG00000172270	BSG	-1.379684693	0.000410517	-0.829604782	0.043393159	-2.209289475	14.26110617	13.45717518	13.73632778	12.47506605	12.4814142	13.03518302	13.02078497
ENSG00000129255	MPDU1	-0.948520699	0.446716354	-1.2626001	0.286667458	-2.211120799	10.905938	8.848535039	7.777116083	8.778830996	8.85207713	8.068408401	8.839676004
ENSG00000205464	ATP6AP1L	-1.38509727	0.281735266	-0.826566376	0.527954208	-2.211663647	4.00722531	6.04685525	5.156626401	4.034562184	4.2560182	5.141958644	3.717076715
ENSG00000167515	TRAPPC2L	-1.027432373	0.001958103	-1.184721503	0.000243834	-2.212153877	8.981092835	8.655694736	8.631919287	7.703217906	7.79460185	7.722704302	7.455265057
ENSG00000168175	MAPK1IP1L	-1.420197724	0.002089486	-0.792517791	0.104511913	-2.212715515	11.4974524	10.76380876	10.68342405	9.618553696	9.60989979	9.997388539	10.44649123
ENSG00000198270	TMEM116	-0.819256458	0.551171197	-1.393506958	0.278176873	-2.212763416	6.009025997	3.686303414	7.029880454	5.319220934	5.36298494	5.119635168	4.479073019
ENSG00000024526	DEPDC1	-0.917973963	0.646818108	-1.296329576	0.49588682	-2.214303538	3.327711234	5.595927442	2	3.840289928	3.60476711	3.12825509	3.753786374
ENSG00000127838	PNKD	-1.362401838	0.000182564	-0.852269228	0.024727678	-2.214671066	9.384607364	9.318399851	8.941672936	7.807949361	7.95274764	8.151901655	8.585960925
ENSG00000177917	ARL6IP6	-1.023225645	0.162619057	-1.191610779	0.092106832	-2.214836424	8.928409675	7.431218269	8.721235202	7.555872146	7.40393901	7.606109069	6.955898408
ENSG00000126088	UROD	-1.017517835	0.311127936	-1.19774905	0.217196921	-2.215266885	11.25159303	9.386925327	8.922968322	9.206465443	9.21922971	8.881419814	9.171855284
ENSG00000275824		2.975761116	0.60852038	-5.191094378	0.364514502	-2.215333262	2	2	2	3.709785322	3.45167086	2	2
ENSG00000175029	CTBP2	-1.309103948	0.053925622	-0.9063733	0.192038633	-2.215477247	10.81695923	9.936721131	9.180874355	8.767939462	8.88857762	9.315659588	9.137106455
ENSG00000007541	PIGQ	-1.362080496	0.250618358	-0.854056733	0.48296181	-2.216137229	7.790810999	8.487557258	10.80588088	8.310225403	8.23671175	8.794695657	8.757891838
ENSG00000065882	TBC1D1	-2.290429903	1.87E-09	0.073217966	0.886475445	-2.217211937	10.43795805	11.04094592	11.18913795	8.645348336	8.64548836	10.83619401	11.14243586
ENSG00000276780		2.973682718	0.608680785	-5.191094386	0.364514502	-2.217411669	2	2	2	3.462030052	3.69849104	2	2
ENSG00000120742	SERP1	-1.302941071	0.00562177	-0.9151278	0.0593663	-2.218068871	10.80239439	11.1990418	11.79914889	10.01841412	10.032869	10.53493405	10.27793661
ENSG00000188385	JAKMIP3	-1.680836372	0.503822785	-0.538918214	0.839017584	-2.219754586	5.095959458	2	2	2.784612485	2.86123014	3.316666208	3.539239711
ENSG00000105479	CCDC114	-1.971754019	0.445453858	-0.250433898	0.928829011	-2.222187917	2	4.11352228	2	2.498691189	2.2680111	3.12825509	2.885414003
ENSG00000105568	PPP2R1A	-1.51544718	0.013543185	-0.708219232	0.282259948	-2.223666412	13.00891643	11.68842912	11.95227171	10.78388778	10.8589471	11.40838965	11.81815607
ENSG00000161513	FDXR	-1.780141894	0.001111835	-0.445244223	0.470583967	-2.225386117	8.107517749	7.002537973	7.600047325	6.069927018	5.74985018	7.296052918	7.085754446
ENSG00000167118	URM1	-1.176792429	0.003796745	-1.049811712	0.010307848	-2.22660414	9.667789716	9.799806309	10.28977157	8.716468139	8.82894823	8.65615757	9.108160402
ENSG00000165802	NSMF	-2.005869944	3.74E-05	-0.221445723	0.712407637	-2.227315667	10.60392551	9.849218119	10.9743126	8.550024073	8.55284928	10.44941809	10.1885847
ENSG00000143036	SLC44A3	-2.100027672	0.198785891	-0.128750671	0.945755737	-2.228778343	5.330594134	6.535001748	2	4.070463721	3.45167086	5.650128416	5.136701757
ENSG00000104824	HNRNPL	-0.997737671	0.07806983	-1.231300657	0.024328003	-2.229038327	10.60951384	11.01334259	11.57188978	10.13319806	10.1143063	9.439667847	10.23456118
ENSG00000166526	ZNF3	-1.076189529	0.065058738	-1.153990213	0.043110722	-2.230179741	9.730679447	8.916342327	8.460932916	8.024753265	8.11025689	8.020265032	7.962181894
ENSG00000010219	DYRK4	-1.16411001	0.024656108	-1.068064396	0.03854522	-2.232174406	8.87372952	8.077869079	7.858115942	7.165617905	7.22257674	7.223329552	7.349806994
ENSG00000106278	PTPRZ1	0.919798563	0.795095011	-3.152347578	0.316133759	-2.232549015	4.00722531	5.72254323	2	6.351046291	2	2.987421008	2.225841198
ENSG00000005206	SPPL2B	-1.239553907	0.000599658	-0.993243478	0.006770897	-2.232797385	9.128497779	9.497318007	9.829330889	8.315844936	8.25031674	8.538032809	8.515559017
ENSG00000131788	PIAS3	-1.479479638	4.06E-06	-0.753659176	0.02758368	-2.233138813	10.67493801	10.2574711	10.67226762	9.09649754	9.04952667	9.605472338	9.962227574
ENSG00000179029	TMEM107	-1.283971544	0.467608963	-0.951543788	0.591655943	-2.235515332	7.579279349	6.899080544	2	5.602782483	5.40529704	5.853194273	5.772630506
ENSG00000146066	HIGD2A	-0.877542401	0.305220054	-1.358628375	0.090878546	-2.236170776	10.60951384	9.018611864	8.904018013	8.880924505	8.84696933	8.108635266	8.621777169
ENSG00000178952	TUFM	-1.445345686	0.001031241	-0.791781021	0.09006775	-2.237126707	11.34164739	11.01952247	10.39531398	9.514881142	9.54514558	10.06192211	10.29067944
ENSG00000090263	MRPS33	-1.116087612	0.13727197	-1.12118299	0.127460764	-2.237270601	8.716893051	7.051611315	8.561105505	7.238438152	7.11195456	6.987904673	7.334741191
ENSG00000009844	VTA1	-0.702279529	0.210105576	-1.535491697	0.002467188	-2.237771226	9.358295023	10.12043894	10.55568472	9.401417034	9.38433737	8.538032809	8.594208165
ENSG00000086544	ITPKC	-1.359983844	0.224142044	-0.878592479	0.443890552	-2.238576323	10.32020829	8.191702463	7.552162999	7.839502934	7.85944752	8.486081846	8.140837768
ENSG00000143603	KCNN3	0.409005538	0.825160194	-2.647728319	0.088351175	-2.238722781	5.70932728	2	5.59507254	5.662809983	5.24368216	2.858551861	3.383972333
ENSG00000180185	FAHD1	-0.980068427	0.018147366	-1.258906888	0.001610393	-2.238975315	9.384607364	8.671479029	9.083172337	8.134820652	8.06698125	7.661243884	7.975841991
ENSG00000145354	CISD2	-0.49047917	0.444744248	-1.748753509	0.001504602	-2.239232678	8.16936288	9.338312924	8.434763955	8.235106869	8.284723	6.879153024	7.162846889
ENSG00000182154	MRPL41	-1.588057746	6.91E-05	-0.651709368	0.132806015	-2.239767115	9.761125267	9.549500232	10.2528023	8.285617933	8.32752521	9.028334265	9.414713898
ENSG00000116754	SRSF11	-0.817823715	0.10052636	-1.422645765	0.002070266	-2.24046948	11.69173081	11.04700893	10.72720655	10.44622016	10.3446634	9.924007159	9.650471437
ENSG00000184205	TSPYL2	-0.211559498	0.709501472	-2.030127664	6.23E-06	-2.241687162	10.03472846	9.74864196	10.69449487	10.00861625	9.99584995	8.031518488	8.347103327
ENSG00000155660	PDIA4	-0.444636972	0.449163778	-1.797227554	0.000305645	-2.241864527	12.21051618	12.12373461	12.3988809	11.79320259	11.8160057	9.765897048	10.91886419
ENSG00000253958	CLDN23	0.449065549	0.838658392	-2.691832659	0.148381845	-2.242767109	2	5.595927442	5.39244166	5.258265913	5.49945122	3.56816602	2.225841198
ENSG00000137166	FOXP4	-1.360343882	0.091805172	-0.883772828	0.285220143	-2.24411671	8.674867122	10.46189757	8.978369997	8.287525788	8.21506984	8.888863837	8.535462193
ENSG00000091844	RGS17	0.913290076	0.756463845	-3.158151206	0.226910457	-2.24486113	2	2	7.225742779	6.548530093	6.5890786	3.694366604	2.551896972
ENSG00000113048	MRPS27	-1.901084501	4.21E-05	-0.343923282	0.529270693	-2.245007782	9.790941832	10.15453315	9.116480286	7.920420067	7.81567127	9.2896505	9.52069224

ENSG00000177595	PIDD1	-2.148390332	0.000432249	-0.096843529	0.901842972	-2.24523386	8.313285031	7.431218269	7.818184403	5.900763176	5.74985018	8.219062336	7.20488131
ENSG00000164675	IQUB	-0.172435443	0.948671972	-2.072847484	0.382824967	-2.245282926	2	4.710564154	2	3.289987342	3.60476711	2.746468522	2.326754077
ENSG00000147509	RGS20	-0.724283143	0.774707448	-1.521369687	0.518400462	-2.24565283	7.435667355	3.076665209	2	5.242616075	5.3193943	5.136410075	3.698365557
ENSG00000167196	FBXO22	-1.348318061	0.03918001	-0.898215753	0.181113049	-2.246533814	9.720386367	8.356994601	9.465702942	8.03841338	7.86198169	8.689219644	8.029224588
ENSG00000124733	MEA1	-1.069733664	0.000423512	-1.177265764	8.02E-05	-2.246999428	10.60951384	10.26269067	10.62675862	9.420668575	9.46239569	9.171189325	9.481899659
ENSG00000111785	RIC8B	-1.150532879	0.256719105	-1.096538557	0.272887212	-2.247071436	9.423197387	9.125613817	6.718950348	7.706073672	7.65545169	7.836577593	7.623392692
ENSG00000146670	CDCA5	-1.410718714	0.209225902	-0.836757696	0.46727486	-2.24747641	4.00722531	5.946638857	6.80318718	4.783303513	4.63360314	4.933813368	5.44241046
ENSG00000279566		0.111479647	0.935007649	-2.35988275	0.031285282	-2.248403103	4.00722531	7.099070191	7.098165388	6.628980152	6.78340763	4.604690117	4.316807515
ENSG00000104979	C19orf53	-1.237939981	0.003686449	-1.013896994	0.019496236	-2.251836975	10.70134604	9.916994052	10.05225195	9.014783325	9.05063789	9.122905457	9.376603759
ENSG00000006652	IFRD1	-0.392406963	0.659880399	-1.860096626	0.014001426	-2.25250359	10.43166252	9.433952963	10.41552659	9.788505066	9.75261615	8.917048655	7.260151003
ENSG00000162521	RBBP4	-1.146900147	1.72E-05	-1.106953526	3.11E-05	-2.253853673	11.15935596	10.72651826	10.9788475	9.834904647	9.80623425	9.863792053	9.857019471
ENSG00000169955	ZNF747	-0.794819935	0.41162884	-1.459179416	0.101692496	-2.25399935	9.081016759	6.844429755	7.691296561	7.409376773	7.35800033	6.615624322	6.853913776
ENSG00000173875	ZNF791	-1.611222687	1.37E-05	-0.643153255	0.109831184	-2.254375942	9.602033055	9.04308532	8.922968322	7.605658789	7.64959655	8.59173005	8.571253152
ENSG00000186193	SAPCD2	-1.762136861	0.256571989	-0.494518798	0.76594204	-2.256655659	5.330594134	3.076665209	3.369317972	3.227757282	2.94009902	4.166733998	3.560117069
ENSG00000124299	PEPD	-1.041253114	0.046045679	-1.215856711	0.016706175	-2.257109825	10.1076228	9.125613817	10.06083273	8.755587391	8.82765235	8.426517973	8.789175794
ENSG00000157778	PSMG3	-1.661810848	0.000636258	-0.596359836	0.265494962	-2.258206683	9.159305284	9.192779271	8.175061096	7.250225364	7.31055061	8.350677018	8.302113262
ENSG00000100296	THOC5	-0.99690658	0.408476816	-1.261410345	0.27417388	-2.258316925	10.41898845	8.469613413	7.285536013	8.404661252	8.19309833	7.919680595	8.154630332
ENSG00000131143	COX4I1	-1.35501981	0.037220596	-0.905044725	0.176711799	-2.260064535	12.56276862	11.17709514	11.34831197	10.46277773	10.5044462	10.82664326	11.03141843
ENSG00000214253	FIS1	-1.330651364	0.047054927	-0.930690492	0.174415528	-2.261341856	10.46903009	8.88960062	9.963514551	8.600009476	8.57618689	8.749978316	9.18619545
ENSG00000196419	XRCC6	-1.204224519	0.003463152	-1.05718026	0.011096104	-2.261404779	12.2324126	11.69616579	11.57188978	10.64941153	10.6715842	10.54700852	11.0276759
ENSG00000114646	CSPG5	-2.76125645	0.240049702	0.499502519	0.847549471	-2.26175393	2	5.595927442	2	2.695456494	2.59489879	5.002522427	4.26688425
ENSG00000006025	OSBPL7	-1.510331624	0.362508675	-0.751588907	0.665769105	-2.261920531	7.031698319	6.844429755	2	5.058234798	4.93258395	5.528313928	5.816671626
ENSG00000136521	NDUFB5	-0.819325113	0.296385835	-1.44326904	0.046743116	-2.262594153	10.24321353	9.00621767	8.511888463	8.637890117	8.62474679	8.017249226	8.013226939
ENSG00000198492	YTHDF2	-1.067228125	0.02109997	-1.197228368	0.008293918	-2.264456494	10.83615308	11.05905903	10.10298738	9.660380652	9.65662671	9.438259243	9.614745652
ENSG00000104064	GABPB1	-0.848373848	0.159914848	-1.417666854	0.011593579	-2.266040702	9.317904228	8.169643011	8.325066185	7.831679003	7.88459112	7.355766946	7.244113847
ENSG00000134809	TIMM10	-1.266065996	0.253395005	-1.000227401	0.367386473	-2.266293398	8.928409675	6.666836837	6.534109959	6.542151749	6.64311914	6.649130473	7.025105265
ENSG00000170855	TRIAP1	-1.315411071	0.001935204	-0.951260472	0.029190238	-2.266671543	9.579430841	8.762721501	9.196533591	7.942383523	7.88209639	8.40533527	8.123409541
ENSG00000203943	SAMD13	-0.954618247	NA	-1.312738577	NA	-2.267356824	2	3.076665209	2	2.141737246	2.49394783	2.34604626	2.172608095
ENSG00000099385	BCL7C	-1.095522159	0.055040826	-1.172004681	0.036112934	-2.26752684	9.473095744	8.317412869	9.083172337	7.905589775	7.98565528	7.612118169	8.090601559
ENSG00000107020	PLGRKT	-0.617146737	0.712294566	-1.650467387	0.273834457	-2.267614124	8.928409675	5.72254323	4.874587708	6.902323594	7.02417148	5.85994354	6.088639344
ENSG00000119760	SUPT7L	-1.071609232	2.41E-05	-1.196421287	1.59E-06	-2.26803052	10.28571918	10.08551947	10.26770388	9.16866395	9.12637045	8.893394126	9.143604888
ENSG00000091140	DLD	-1.10968401	0.041566879	-1.158951777	0.03070475	-2.268635787	9.521325812	10.12043894	10.67226762	9.081180848	9.06939887	8.806972075	9.216922247
ENSG00000100842	EFS	-2.024661756	0.016912578	-0.246026092	0.808868217	-2.270687848	7.624139768	9.405920729	10.01740919	7.348537858	7.28811304	9.151037776	8.987157359
ENSG00000198342	ZNF442	-0.12462535	0.966716761	-2.147352734	0.414376291	-2.271978083	6.96351253	2	2	5.514313187	5.16377554	4.029788235	3.287118176
ENSG00000005483	KMT2E	-1.147279858	2.18E-05	-1.125339535	2.86E-05	-2.272619393	11.17451594	11.03180337	11.46604358	10.11987514	10.059057	10.10907137	10.11421585
ENSG00000140365	COMMD4	-0.709513067	0.370174297	-1.563926135	0.029540521	-2.273439202	9.710019321	8.147241016	8.434763955	8.252792309	8.20512425	7.124495877	7.614687532
ENSG00000214046	SMIM7	-0.932868345	9.38E-05	-1.34220685	5.38E-09	-2.275075195	9.810482605	9.806969599	9.788583153	8.845101141	8.90464485	8.362013331	8.57061027
ENSG00000004142	POLDIP2	-1.639080064	0.015702631	-0.636468649	0.389304796	-2.275548712	11.67050115	10.12617756	10.40208303	9.214510118	9.31574487	10.23382726	10.29340937
ENSG00000189067	LITAF	-2.341892435	0.001497712	0.066313198	0.944363944	-2.275579237	11.32135895	9.357954881	10.16797309	8.227176542	8.13141746	10.49619812	10.64412949
ENSG00000124422	USP22	-1.525446723	7.50E-09	-0.753247842	0.007273542	-2.278694565	12.20498975	12.43011779	12.5724427	10.82584613	10.9454246	11.53219589	11.77339293
ENSG00000140391	TSPAN3	-1.354506888	0.000454418	-0.924781457	0.021329954	-2.279288344	12.45359838	12.03348143	12.54671519	11.02423622	10.9915755	11.10893748	11.70415409
ENSG00000242715	CCDC169	-2.262767666	0.229708761	-0.017991203	0.993459837	-2.280758869	4.467311984	2	4.058998049	2.784612485	2.38539787	4.133696865	3.311948089
ENSG00000132661	NXT1	-1.689938669	0.000161722	-0.591232458	0.228666677	-2.281171127	8.540903468	7.847167909	7.818184403	6.29903474	6.60731796	7.479255779	7.569069104
ENSG00000176102	CSTF3	-0.24509708	0.913189302	-2.03661714	0.283648588	-2.281714219	2	8.834582582	9.196533591	8.128448191	8.2790453	6.514451546	6.406535141
ENSG00000185359	HGS	-0.43566131	0.558317273	-1.84624715	0.003646438	-2.281908459	11.67583792	10.47544827	10.23774519	10.49161431	10.5225794	8.967528086	9.22715677
ENSG00000169714	CNBP	-1.179373957	0.007634516	-1.104069052	0.012705607	-2.283443009	12.74263541	11.89244687	12.08228623	11.09020988	11.1267944	11.30784912	11.0480252
ENSG00000088766	CRLS1	-1.220196657	0.003161248	-1.063632302	0.010607918	-2.283828959	8.716893051	9.308339287	9.385989894	7.966401501	7.95512324	8.300083299	7.902127837
ENSG00000086200	IPO11	-1.005402617	0.561371291	-1.280360574	0.443230611	-2.285763191	4.815609471	7.099070191	10.05225195	7.791909949	7.56502709	7.388811377	7.438450313
ENSG00000176623	RMDN1	-1.379470876	0.07497303	-0.906482068	0.255070887	-2.285952944	10.07567912	8.101365653	9.242516611	7.990026167	7.97632944	8.409941741	8.488586985
ENSG00000170166	HOXD4	4.622411758	0.024200025	-6.908755836	0.007633479	-2.286344079	2	3.686303414	2	6.358325776	6.507263	2	2
ENSG00000005486	RHBDD2	-1.142763801	0.005164205	-1.144675825	0.004822148	-2.287439627	11.10895605	10.50217373	10.31867969	9.520570052	9.56938265	9.548642921	9.537896513
ENSG00000169282	KCNAB1	-2.866026939	0.057963449	0.578470371	0.732581748	-2.287556569	2	5.72254323	6.629488387	3.227757282	3.55551834	6.290458506	6.182510897
ENSG00000198856	OSTC	-1.207263016	0.122079915	-1.08172518	0.163562907	-2.288988195	12.16380252	10.89059638	10.30429804	10.14902484	10.1121791	10.29128794	10.21946626
ENSG00000134597	RBMX2	-1.335131993	0.537027555	-0.954261094	0.665712192	-2.289393087	9.800745303	7.35507212	2	7.136275664	7.17037177	7.610117916	7.434215826
ENSG00000282604		2.901620803	0.617500397	-5.191094686	NA	-2.289473883	2	2	2	3.840289928	3.15372745	2	2
ENSG00000160796	NBEAL2	-2.320581108	0.119776467	0.028915421	0.987221496	-2.291665687	3.327711234	8.433041974	8.175061096	5.501219927	5.56313034	7.809812485	7.745758018
ENSG00000169255	B3GALNT1	-0.53422772	0.341329168	-1.757762809	0.000306618	-2.291990529	7.667647223	8.717821467	8.325066185	7.821180652	7.7377845	6.448814696	6.721179863
ENSG00000183395	PMCH	2.898995389	0.617831455	-5.191094697	NA	-2.292099308	2	2	2	3.566290607	3.50452879	2	2
ENSG00000104818	CGB2	2.898557361	0.617843763	-5.191094699	NA	-2.292537338	2	2	2	3.515101962	3.55551834	2	2
ENSG00000111786	SRSF9	-0.72130009	0.191457657	-1.571477772	0.00166238	-2.292777862	11.04027452	10.12617756	10.55568472	9.971121557	9.82972721	8.676372393	9.35772346
ENSG00000161016	RPL8	-1.746583118	0.008869853	-0.547352454	0.463132585	-2.293935572	14.02224794	12.74089149	12.49857094	11.4920928	11.5216186	12.62892023	12.77734718
ENSG00000157335	CLEC18C	2.895487483	0.618153694	-5.191094712	NA	-2.295607229	2	2	2	3.09461492	3.86943969	2	2
ENSG00000106615	RHEB	-0.387692371	0.563160425	-1.909258143	0.000698186	-2.296950514	10.5468183	9.203675681	9.681375057	9.568433308	9.49541459	8.056705941	7.993217039
ENSG00000137996	RTCA	-0.436392366	0.36394741	-1.862264393	4.91E-06	-2.298656759	9.624286623	9.246456519	8.721235202	8.804373539	8.81854848	7.425672291	7.388257299
ENSG00000242259	C22orf39	-2.029238061	0.20665794	-0.270183223	0.885032716	-2.299421284	9.174465797	8.505280662	3.369317972	6.283823264	6.3857277	8.152588976	7.925444964
ENSG00000165669	FAM204A	-1.102087491	0.02474593	-1.197803088	0.012880352	-2.299890579	9.233563228	8.213429689	8.904018013	7.731524332	7.76782485	7.590975897	7.718159092
ENSG00000178921	PFAS	-1.882318281	0.089428115	-0.418773429	0.736888343	-2.301091709	5.70932728	8.94259736	8.434763955	6.221320894	6.53293476	7.580798201	7.998006535
ENSG00000164039	BDH2	-0.200662073	0.72600899	-2.102873539	3.32E-06	-2.303535612	9.081016759	8.557180776	8.042706615	8.456457569	8.39031583	6.558682816	6.574984961
ENSG00000180035	ZNF48	-2.117473647	0.364034842	-0.186474716	0.944101725	-2.303948363	2	5.457120953	9.926432745	6.449761039	6.23747437	8.437839892	7.975841991
ENSG00000172167	MTBP	-0.655140304	0.713187979	-1.649914328	0.306062327	-2.305054632	2	4.93638933	6.322058544	4.907595604	4.40247105	4.029788235	3.735548302
ENSG00000181523	SGSH	-0.876697944	0.290539585	-1.428754501	0.06486798	-2.305452444	11.41049097	9.506147172	10.17589429	9.682298904	9.73682479	8.99192853	9.312299623
ENSG00000092068	SLC7A8	-1.50688755	0.352079152	-0.800853777	0.634245125	-2.307741327	3.327711234	6.602424674	8.007629717	5.527288686	5.47317115	6.736076089	5.171036168
ENSG00000276045	ORAI1	-1.324521159	4.93E-06	-0.984825126	0.000794685	-2.309346285	8.777722034	8.88960062	9.116480286	7.574744049	7.67575955	8.034504643	7.880504067
ENSG00000114125	RNF7	-0.99127646	0.000114327	-1.318449761	1.21E-07	-2.309726221	10.59268348	10.23640192	10.37481416	9.408447353	9.43116542	9.043977376	9.144037078
ENSG00000081760	AACS	-1.37912481	0.176302078	-0.930629026	0.370342728	-2.309753836	8.285626337	8.623598428	10.62675862	8.16211395	8.248381	8.340457938	8.903351429
ENSG00000076555	ACACB	-0.941847677	0.644285833	-1.370048322	0.480467544	-2.311895999	2	5.131603625	7.734844356	5.514313187	5.49945122	5.141958644	5.101530266
ENSG00000144867	SRPRB	-1.061129909	9.86E-08	-1.25137751	1.53E-10	-2.31250742	10.56418906	10.59613751	10.45512158	9.453327884	9.51406378	9.313817266	9.275690962
ENSG00000109445	ZNF330	-1.091404952	0.626071633	-1.222427621	0.575697563	-2.313832573	10.1076228	7.761270542	2	7.626911583	7.7866208	7.816767288	7.297928101
ENSG00000262327		0.495253758	0.754341134	-2.80927219	0.035591756	-2.314018432	3.327711234	7.901717006	7.285536013	7.697489359	7.42122542	4.496236235	4.522269241
ENSG00000072778	ACADVL	-0.765741747	0.220214001	-1.548822257	0.006729172	-2.314564004	12.74390633	11.45757623	12.68078978	11.61151504	11.6612917	10.75286657	10.95009364
ENSG00000131966	ACTR10	-0.933681522	0.167317726	-1.381272582	0.030563837	-2.314954104	9.097018025	10.22575081	10.63827116	9.26766905	9.11047337	8.885973502	8.593575433
ENSG00000046651	OFD1	-1.104535205	0.210990211	-1.210980993	0.157715974	-2.315516198	7.159102577	7.928237109	9.272377397	7.288834728	7.31055061	7.228554289	7.162846889
ENSG00000204152	TIMM23B	0.738379625	0.184268122	-3.054916354	2.33E-10	-2.31653673	7.82963488	8.414402626	8.677268317	9.061245954	9.09870316	5.056402493	5.708623051
ENSG00000139354	GAS2L3	-1.313177251	0.203115525	-1.003511173	0.332515383	-2.316688424	6.138360429	5.303523747	7.691296561	5.433909581	5.51241389	5.711121288	5.803599879
ENSG00000180592	SKIDA1	-2.122610663	0.150705666	-0.196133708	0.908941926	-2.318744371	3.327711234	6.04685525	4.058998049	3.566290607	3.15372745	4.188345803	5.256659864
ENSG00000159648	TEPP	-4.714896419	0.259738629	2.395429686	0.541103884	-2.319466734	2	3.076665209	2	2	2	3.500668998	4.133950982
ENSG00000044459	CNTLN	-0.501100764	0.749056274	-1.820480322	0.186484076	-2.321581086	8.653385977	7.572420273	4.058998049	7.140504127	7.21466714	6.05195199	5.768151538
ENSG00000073464	CLCN4	-2.546933067	0.304297653	0.225115946	0.935446753	-2.32181712	2	5.131603625	2	2.784612485	2.2680111	4.053534413	4.062589736
ENSG00000183049	CAMK1D	-3.456064464	0.010024408	1.13402239	0.445282505	-2.322042074	6.009025997	6.78762695	9.849281147	5.552894743	4.74762503	9.636512519	9.638558632
ENSG00000110104	CCDC86	-1.2902047	0.040174325	-1.032673905	0.103916882	-2.322878605	9.922475255	8.487557258	9.541233192	8.164192202	8.13979554	8.211825862	8.577666268
ENSG00000205592	MUC19	-0.69218051	NA	-1.630843029	NA	-2.323023539	2	2	3.369317972	2.141737246	2.59489879	2.094598369	2.326754077
ENSG00000106638	TBL2	-1.345717549	0.00058344	-0.977856437	0.015303411	-2.323573987	10.71696191	9.936721131	10.56174313	9.088859521	9.11260313	9.492471997	9.439572619
ENSG00000089847	ANKRD24	-1.702281039	0.515393703	-0.621498504	0.821109617	-2.323779543	2	2	5.930865648	3.162721474	3.39680241	4.166733998	3.841670744
ENSG00000084453	SLCO1A2	-4.13918349	0.143847237	1.815238694	0.433240072	-2.323944795	2	3.076665209	3.369317972	2	2.14022021	3.411599105	4.511590831
ENSG00000100601	ALKBH1	-0.98210296	0.169054392	-1.342381546	0.04761818	-2.324484506	7.709880939	7.051611315	8.536706852	6.89733479	6.95464102	6.800644007	6.317379004
ENSG00000170745	KCNS3	-1.549251646	0.346696909	-0.77832167	0.651103372	-2.327573316	3.327711234	7.051611315	8.076950877	5.753943344	5.49945122	6.981729486	5.217771644
ENSG00000157734	SNX22	-0.043751437	0.96929411	-2.28445244	0.011166291	-2.328203877	4.815609471	5.131603625	6.718950348	5.753943344	5.74985018	3.851628042	3.875378318
ENSG00000204852	TCTN1	-0.806251977	0.09337333	-1.522199296	0.000549691	-2.328451272	9.317904228	8.623598428	9.196533591	8.268332738	8.2790453	7.267148095	7.820122783
ENSG00000112984	KIF20A	-1.87813222	0.346404872	-0.4505043	0.836821477	-2.328636521	4.467311984	6.389896633	2	3.881293667	3.50452879	3.500668998	5.502966356
ENSG00000132463	GRSF1	-1.049428755	0.040810687	-1.279562264	0.010048431	-2.328991019	11.92474824	10.79656548	11.35181254	10.38872576	10.3760296	10.16225622	10.14369036
ENSG00000112081	SRSF3	-0.970041883	0.034923735	-1.360447729	0.001927913	-2.330489613	11.1478805	10.68045884	11.35181254	10.1698607	10.064018	9.363643077	10.02058586
ENSG00000186907	RTN4RL2	-1.334223468	0.576805834	-0.996591445	0.670031027	-2.330814913	2	3.076665209	3.369317972	2.270786764	2.59489879	2.526562374	2.551896972
ENSG00000134014	ELP3	-0.953354461	0.226126239	-1.377977717	0.064751221	-2.331332178	10.02639697	8.147241016	9.031722608	8.327018759	8.31463236	7.910770103	7.893928381
ENSG00000105996	HOXA2	2.859699628	0.622818782	-5.191094861	NA	-2.331395233	2	2	2	3.566290607	3.45167086	2	2
ENSG00000269556	TMEM185A	-0.287996468	0.61090653	-2.043692893	8.40E-06	-2.331689361	8.716893051	8.469613413	9.452720984	8.621345041	8.68180105	6.921708059	6.938065226
ENSG00000059377	TBXAS1	-1.810122381	0.383862151	-0.521865964	0.814923166	-2.331988344	2	3.076665209	5.772716771	3.289987342	3.08597292	4.478802948	3.430065404
ENSG00000167554	ZNF610	-0.680012292	0.72338932	-1.652451174	0.346462637	-2.332463466	7.82963488	6.728495673	2	6.213314212	6.11534392	5.553513572	4.911532223

ENSG00000189186	DCAF8L2	2.858349037	0.622871051	-5.191094867	NA	-2.332745829	2	2	2	3.406930997	3.60476711	2	2
ENSG00000205457	TP53TG3C	2.858349037	0.622871051	-5.191094867	NA	-2.332745829	2	2	2	3.406930997	3.60476711	2	2
ENSG00000204843	DCTN1	-1.526318982	7.80E-05	-0.807587227	0.049586809	-2.333906209	12.38845524	11.752905	11.92668027	10.48788555	10.5593475	10.98908978	11.45586848
ENSG00000174177	CTU2	-1.671168656	0.000201031	-0.665481145	0.169565436	-2.336649802	7.790810999	7.35507212	8.143093325	6.069927018	6.26836889	7.254397864	7.010010317
ENSG00000197568	HHLA3	-2.29886036	0.091749244	-0.03900373	0.981056901	-2.33786409	4.815609471	5.838937808	8.511888463	5.110902178	5.0259382	7.106162485	7.261744957
ENSG00000179222	MAGED1	-1.891845568	1.02E-07	-0.446306585	0.274614244	-2.338152154	13.66850366	13.11877686	13.67337272	11.58558106	11.6507048	12.84207702	13.25435596
ENSG00000154822	PLCL2	-1.402842217	0.336743265	-0.937496201	0.527954208	-2.340338417	3.327711234	7.002537973	7.818184403	5.578054239	5.6121243	6.14330814	5.900914815
ENSG00000166166	TRMT61A	-1.325764294	0.541643419	-1.014846099	0.643682096	-2.340610393	9.858207046	7.431218269	2	7.062428156	7.36516374	7.577730839	7.470508284
ENSG00000080603	SRCAP	0.732592001	0.076242146	-3.074283342	2.18E-17	-2.341691342	9.839306291	9.267380431	9.990713521	10.40683041	10.5056622	6.603610344	6.795689611
ENSG00000162999	DUSP19	-2.017941933	0.064138247	-0.324818287	0.794186359	-2.34276022	4.00722531	5.595927442	6.322058544	3.709785322	4.05806467	5.669467138	4.77533149
ENSG00000135845	PIGC	-1.309772255	0.051823689	-1.033061409	0.127753231	-2.342833664	7.624139768	8.968383114	7.858115942	7.003290579	7.00136498	7.043805932	7.467748741
ENSG00000204815	TTC25	-4.714896876	0.259172295	2.371842652	0.544666992	-2.343054224	2	3.076665209	2	2	2	3.584558147	4.047883918
ENSG00000086730	LAT2	-1.096798019	0.495803512	-1.246960318	0.421398024	-2.343758338	2	5.131603625	5.772716771	4.17310023	3.94787299	4.220168345	3.620995423
ENSG00000125871	MGME1	-1.01947662	0.590423475	-1.324883836	0.466737112	-2.344360456	9.839306291	7.638155693	3.369317972	7.433701013	7.65252709	7.528804029	6.90172361
ENSG00000171208	NETO2	0.070757617	0.957153132	-2.415742104	0.021928563	-2.344984486	5.095959458	7.819099893	7.896971941	7.454228844	7.44169935	5.498346994	4.56420962
ENSG00000204131	NHSL2	-5.041827371	2.82E-11	2.695341458	4.13E-05	-2.346485913	6.653098118	6.602424674	6.629488387	3.162721474	2.2680111	9.371930177	9.166759824
ENSG00000143198	MGST3	-0.875260002	0.03287065	-1.471529449	0.000125997	-2.346789451	10.6695979	9.916994052	10.09465441	9.377433547	9.40446877	8.780980934	8.817088095
ENSG00000167699	GLOD4	-1.30548518	0.003515112	-1.041406181	0.022449102	-2.346891362	9.895281535	8.94259736	9.503962296	8.237082658	8.16464176	8.44290591	8.481080411
ENSG00000103253	HAGHL	-1.913144243	0.253947704	-0.434737665	0.815972748	-2.347881908	3.327711234	6.140560021	8.561105505	5.242616075	5.6121243	7.170014349	6.38329369
ENSG00000171735	CAMTA1	-1.108577622	0.000797451	-1.239908348	0.000123316	-2.34848597	8.737455672	9.102512015	8.784770914	7.839502934	7.74878077	7.523500045	7.796124663
ENSG00000149564	ESAM	-3.553350736	0.191751955	1.204423273	0.682126239	-2.348927463	7.53297911	2	2	3.162721474	3.2184422	7.954782214	5.230851202
ENSG00000138382	METTL5	-1.195804692	0.057338722	-1.155569744	0.062814356	-2.351374436	8.892187472	8.124485672	7.451345288	7.088925745	7.11620658	7.127295835	7.156006254
ENSG00000261236	BOP1	-1.765817308	0.315574141	-0.586349597	0.758401903	-2.352166905	10.05943807	8.968383114	3.369317972	7.273514749	7.32531744	8.432756021	8.483814596
ENSG00000094880	CDC23	-1.544722217	0.002664855	-0.808647298	0.138024336	-2.353369515	9.112843761	8.791896851	9.849281147	7.697489359	7.87709394	8.667743558	8.345600992
ENSG00000131196	NFATC1	-0.537446258	0.603040858	-1.817241866	0.044024163	-2.354688124	6.366709671	7.847167909	8.884815469	7.481152682	7.5240115	6.341987719	6.189227296
ENSG00000188312	CENPP	-2.391385183	0.105206276	0.035740098	0.983983084	-2.355645086	5.866944937	5.303523747	2	3.289987342	3.28037825	4.68289886	5.464725981
ENSG00000122033	MTIF3	-1.093634091	0.277913797	-1.263630306	0.19380488	-2.357264397	9.957954117	8.191702463	7.552162999	7.821180652	7.89700047	7.653495316	7.730874172
ENSG00000179604	CDC42EP4	-1.662896303	0.013325886	-0.694414606	0.34077058	-2.357310909	10.84565509	9.159587375	9.945092785	8.512996295	8.4705529	9.43006211	9.474701772
ENSG00000165516	KLHDC2	-1.577325208	2.50E-05	-0.780342398	0.050340543	-2.357667606	9.966689162	9.405920729	9.25752426	8.022463952	8.00412803	8.809589199	8.791936295
ENSG00000137207	YIPF3	-1.002572688	0.100199697	-1.355829046	0.020018787	-2.358401735	12.24682774	10.87022386	11.73893313	10.68241243	10.761874	10.21716837	10.50889441
ENSG00000143891	GALM	-0.365255376	0.678162682	-1.993707682	0.006618212	-2.358963058	8.928409675	8.929529568	7.163363831	8.136938566	8.23084153	6.791847192	6.371531035
ENSG00000086827	ZW10	-0.708521021	0.643838836	-1.650567202	0.23711641	-2.359088223	9.602033055	8.395519306	4.874587708	7.893113669	7.85690889	6.931352719	6.979338179
ENSG00000089091	DZANK1	-0.487562922	0.596116556	-1.872776187	0.018343763	-2.360339109	4.815609471	5.457120953	5.59507254	4.783303513	5.00774437	4.04171018	3.496556178
ENSG00000138442	WDR12	-1.309718157	0.00730559	-1.050653936	0.03362727	-2.360372094	7.975256505	8.981105197	8.608698767	7.178012736	7.4074129	7.617106703	7.480125329
ENSG00000102901	CENPT	-1.586388482	0.015795913	-0.774368531	0.268917609	-2.360757014	8.631580145	9.067150531	10.11951029	7.826439377	7.85690889	8.437839892	8.822498333
ENSG00000165525	NEMF	-0.581379031	0.292697717	-1.78044759	0.000219832	-2.361826621	10.31337612	9.664384941	9.212024683	9.252121432	9.19330273	7.962642137	8.106655408
ENSG00000189241	TSPYL1	-1.242898683	0.026251815	-1.119507998	0.045942033	-2.362406682	11.03197509	11.06803102	12.09069073	10.26540233	10.2237702	10.18023182	10.53367986
ENSG00000196937	FAM3C	-0.523693312	0.41446225	-1.83878744	0.000872643	-2.362480752	9.358295023	9.192779271	9.963514551	9.097585392	8.94225201	7.148124077	8.133891722
ENSG00000115317	HTRA2	-0.768904034	0.420476172	-1.593941173	0.066662083	-2.362845206	8.586954094	6.899080544	9.212024683	7.748245381	7.75424764	7.153627862	6.704867638
ENSG00000138101	DTNB	-1.927012004	0.236297945	-0.436282743	0.808868142	-2.363294747	3.327711234	7.051611315	4.058998049	4.329433326	3.82856379	5.233158834	5.470251342
ENSG00000154914	USP43	-3.260477536	0.180554595	0.896722638	0.719815802	-2.363754897	2	4.710564154	2	2.270786764	2.2680111	3.517841605	4.692661645
ENSG00000160271	RALGDS	0.378887015	0.822240381	-2.743139454	0.04807007	-2.364252439	4.467311984	8.169643011	9.503962296	8.772033429	8.83927354	5.873348082	5.680297397
ENSG00000196655	TRAPPC4	-1.211055401	0.239632794	-1.154136623	0.257017948	-2.365192024	10.76782679	9.405920729	7.97167868	8.58612524	8.60672783	8.596789458	8.706733286
ENSG00000128973	CLN6	-0.798504988	0.65512513	-1.567601677	0.345156474	-2.366106665	3.327711234	7.002537973	8.904018013	6.965655398	6.84106088	5.773219597	6.479583725
ENSG00000173812	EIF1	-1.464644325	0.001638082	-0.904660897	0.063520955	-2.369305223	13.61880139	12.90092286	12.62979741	11.59003251	11.7051743	12.24770346	12.16749172
ENSG00000140990	NDUFB10	-1.249015401	0.024854983	-1.120870943	0.044632457	-2.369886344	10.72727936	9.656475478	9.714382178	8.894758143	8.86223882	8.86850882	9.131014727
ENSG00000158815	FGF17	-1.996104488	0.484805583	-0.373850616	NA	-2.369955103	2	3.686303414	2	2.270786764	2.2680111	2.911488689	2.466008509
ENSG00000092978	GPATCH2	-0.866295714	0.314797955	-1.505481679	0.059927165	-2.371777393	7.867441304	7.901717006	9.541233192	7.789219291	7.82349365	7.367655602	6.967665897
ENSG00000135917	SLC19A3	-0.277912652	0.929574989	-2.093957699	0.446373473	-2.371870351	2	2	4.058998049	3.162721474	2.49394783	2.42098416	2.172608095
ENSG00000114395	CYB561D2	-0.781741296	0.335570391	-1.590245828	0.032165651	-2.371987124	7.218819781	8.029695761	9.014156722	7.416368504	7.58049694	6.473229652	6.92404095
ENSG00000198457		2.816988068	0.628255701	-5.191095039	NA	-2.374106971	2	2	2	3.289987342	3.65239003	2	2
ENSG00000230048		2.816988068	0.628255701	-5.191095039	NA	-2.374106971	2	2	2	3.289987342	3.65239003	2	2
ENSG00000135437	RDH5	-1.963667222	0.273150492	-0.411518528	0.836906381	-2.37518575	7.485143553	5.838937808	2	4.574143677	4.53541753	5.338397457	6.356691554
ENSG00000111142	METAP2	-1.107829734	0.375381504	-1.267459319	0.294668948	-2.375289052	11.61879218	10.28850895	7.896971941	9.481935579	9.49704586	9.394477264	9.264206697
ENSG00000103528	SYT17	-2.006951012	0.464078443	-0.368596857	0.9017845	-2.37554787	2	5.131603625	2	3.09461492	2.2680111	3.993419637	3.128433649
ENSG00000129347	KRI1	-0.892533283	0.357725763	-1.483067682	0.101181698	-2.375600965	9.247966931	6.78762695	8.296287027	7.565338955	7.5240115	6.91847879	7.019463152
ENSG00000162236	STX5	-1.258619398	3.89E-05	-1.118255254	0.000269416	-2.376874652	10.54098126	10.20962523	10.06083273	8.995070693	9.06830201	9.270148001	9.065448786
ENSG00000160310	PRMT2	-1.542002651	0.028640021	-0.835007287	0.261300134	-2.377009938	11.86526254	10.06176125	11.03653965	9.656695015	9.60158363	10.27085842	10.39367729
ENSG00000064225	ST3GAL6	-0.776786731	0.542758789	-1.600543405	0.171406992	-2.377330137	5.532353208	5.595927442	7.858115942	6.069927018	5.96314905	5.699879814	4.624919101
ENSG00000115694	STK25	-1.856753364	2.35E-05	-0.521281042	0.294091971	-2.378034407	11.75612888	10.87363932	11.01462253	9.349396108	9.48312089	10.65609247	10.83475651
ENSG00000129910	CDH15	-2.839931294	0.278879149	0.461880553	0.8682894	-2.378050741	4.467311984	2	2	2.141737246	2.38539787	3.393105702	3.771796761
ENSG00000115268	RPS15	-1.518581679	0.045000662	-0.859539528	0.278339715	-2.378121207	12.8217977	11.42768197	11.04521411	10.42210785	10.5020111	11.06251538	11.17557911
ENSG00000089280	FUS	-1.138176776	0.115157031	-1.242427765	0.078025727	-2.380604541	11.75864596	10.28338212	10.67226762	9.919767943	9.90107802	9.383101353	10.13371468
ENSG00000124532	MRS2	-1.098632886	0.044976341	-1.282667678	0.016076822	-2.381300563	9.533135543	8.414402626	8.742726175	7.944803394	7.82609171	7.621085148	7.786191022
ENSG00000129518	EAPP	-1.125236709	0.23034597	-1.256491817	0.167672785	-2.381728526	7.667647223	8.968383114	10.11951029	8.14116509	8.11238697	7.968118715	8.026414333
ENSG00000092853	CLSPN	-0.298340196	0.795427588	-2.083461977	0.028515268	-2.381802172	4.00722531	6.04685525	6.073378139	5.363304456	5.39133044	3.616794016	4.133950982
ENSG00000163950	SLBP	-1.051182924	0.123980108	-1.334033596	0.042011267	-2.38521652	10.62062599	9.225224669	9.452720984	8.85414072	8.86097251	8.75224741	8.37829627
ENSG00000091622	PITPNM3	-2.76033306	0.217903135	0.373749869	0.885335383	-2.38658319	8.418903792	3.686303414	2	4.470469939	4.31639476	6.710164669	7.662532142
ENSG00000109332	UBE2D3	-0.396707135	0.530264818	-1.990650103	0.000153586	-2.387357238	12.78523054	11.5838842	11.87409387	11.82063682	11.7367574	10.23350246	10.14239308
ENSG00000171612	SLC25A33	-1.418797749	0.11191982	-0.969552463	0.284322026	-2.388350213	7.82963488	7.051611315	5.59507254	5.786686927	5.69405321	5.951260634	6.344709081
ENSG00000165775	FUNDC2	-0.939485128	0.050150442	-1.44927295	0.001318425	-2.388758079	9.858207046	9.181799937	8.978369997	8.461536372	8.45385092	7.801948045	8.093289637
ENSG00000115761	NOL10	-0.801389379	0.032486453	-1.587656244	4.20E-06	-2.389045622	9.064836028	8.505280662	8.561105505	7.975897842	7.89946959	7.191577434	7.145684098
ENSG00000100897	DCAF11	-1.654492672	0.075396159	-0.735273417	0.459209794	-2.389766089	8.928409675	9.055168103	10.9833682	8.349110081	8.33666436	8.91583461	9.52733357
ENSG00000130811	EIF3G	-0.945135751	0.055248319	-1.445048181	0.001888215	-2.390183932	11.20805444	10.23640192	10.49998282	9.759931695	9.77093593	9.16404896	9.365527647
ENSG00000112339	HBS1L	-1.489994623	0.020769965	-0.900969844	0.179539773	-2.390964467	10.58702944	9.04308532	10.15199903	8.595396228	8.5606705	9.333651532	8.967268538
ENSG00000100284	TOM1	-0.886990838	0.234289292	-1.504192442	0.030748092	-2.391183279	11.39439513	9.608085847	10.95602907	9.898079272	9.98833077	9.335468714	9.324171817
ENSG00000221923	ZNF880	-2.00199728	1.69E-06	-0.389639827	0.414555096	-2.391637107	7.867441304	7.393647515	7.451345288	5.808110275	5.51241389	7.320725255	7.071284046
ENSG00000125743	SNRPD2	-1.698215173	0.012684784	-0.695023133	0.348375224	-2.393238306	11.59363617	10.11467741	10.31867969	9.121312924	9.16279627	9.895138115	10.34976357
ENSG00000090534	THPO	-1.514149752	0.557788547	-0.879231858	0.735231663	-2.39338161	4.467311984	2	2	2.498691189	2.68924543	2.803598391	2.885414003
ENSG00000136518	ACTL6A	-0.760550136	0.577531912	-1.633217922	0.19059348	-2.393768059	9.624286623	8.747909548	5.59507254	7.905589775	8.0358969	7.14536429	7.091143645
ENSG00000163933	RFT1	-1.071573789	0.149609361	-1.322315687	0.064234196	-2.393889476	7.485143553	8.820493869	9.242516611	7.737119576	7.51759662	7.247980242	7.512619696
ENSG00000143799	PARP1	-2.104812025	0.016039408	-0.289180365	0.78196159	-2.39399239	8.075571721	9.7032946	10.5916593	7.728718552	7.67287587	9.404322061	9.583539815
ENSG00000186792	HYAL3	-1.145310131	0.410023637	-1.248914557	0.354839138	-2.394224688	9.384607364	5.838937808	7.163363831	6.979883891	7.1246732	6.320128742	7.392628644
ENSG00000124702	KLHDC3	-1.529057663	0.008765708	-0.865309946	0.158640059	-2.394367609	11.34500123	9.943237334	10.69449487	9.189220094	9.30274583	9.827882244	9.982668878
ENSG00000088876	ZNF343	-1.168952059	0.015631681	-1.226111886	0.009845724	-2.395063945	7.709880939	8.004992147	8.65477198	7.066878356	6.99214046	7.036377096	6.909879006
ENSG00000120333	MRPS14	-1.650476833	0.00097459	-0.745411943	0.165549719	-2.395888775	8.444143041	8.395519306	9.272377397	7.062428156	7.20671394	7.801948045	8.213468956
ENSG00000168216	LMBRD1	-0.996266532	0.093060766	-1.401943262	0.012771461	-2.398209794	9.509418609	9.949724236	8.631919287	8.572106084	8.37442884	8.130430649	8.019835704
ENSG00000149499	EML3	-1.262960871	0.127215703	-1.135338823	0.167152492	-2.398299695	10.59831544	8.834582582	8.941672936	8.408172799	8.48542112	8.643989069	8.499436383
ENSG00000135912	TTLL4	-1.135490385	0.115204374	-1.264480641	0.071132677	-2.399971026	7.53297911	8.762721501	8.904018013	7.337530098	7.46188679	7.637872201	6.78907279
ENSG00000135336	ORC3	-0.818107797	0.114510897	-1.581917597	0.000867841	-2.400025394	8.39321512	9.424669273	9.287079172	8.266399315	8.3220138	7.526684775	7.56002886
ENSG00000133884	DPF2	-1.333195971	2.62E-06	-1.068290536	0.000198102	-2.401486507	10.43166252	10.08551947	10.12770132	8.94033939	8.85080188	9.276783254	9.030812517
ENSG00000185122	HSF1	-1.316295693	0.084119732	-1.085853344	0.154872063	-2.402149037	10.06758145	10.03150247	11.62203638	9.454177434	9.4789996	9.748725972	9.641010317
ENSG00000148120	C9orf3	-2.207939752	0.002472293	-0.194521891	0.83065631	-2.402461643	10.30651143	8.590771778	8.845626171	7.307758725	7.24992376	9.253264914	9.275296471
ENSG00000129194	SOX15	-1.692273371	0.513268686	-0.710400391	0.792987538	-2.402673763	5.70932728	2	2	3.09461492	3.2184422	3.968654369	3.560117069
ENSG00000170266	GLB1	-0.774713475	0.312204876	-1.62798819	0.020041931	-2.402701665	12.04303957	10.54992047	10.56174313	10.44237204	10.480321	9.599435435	9.623450463
ENSG00000156500	FAM122C	-0.921006404	0.185725588	-1.482598634	0.022298533	-2.403605039	5.532353208	6.78762695	6.718950348	5.639097847	5.55061739	5.201624686	4.967301796
ENSG00000128602	SMO	-2.447779376	0.125723688	0.042814771	0.982373817	-2.404964606	8.586954094	9.338312924	3.369317972	6.130704144	6.00043082	8.600320517	8.359810397
ENSG00000171953	ATPAF2	-1.3360803	0.362647422	-1.069861039	0.467461223	-2.405941338	8.696033109	8.376385542	4.058998049	6.733448311	6.64900057	6.770513376	7.107191496
ENSG00000166819	PLIN1	-2.917714303	0.414321642	0.5103855	0.893463941	-2.407328802	2	4.11352228	2	2.3892346	2	3.851628042	2.782576693
ENSG00000179115	FARSA	-1.131057795	0.079842495	-1.276697175	0.042070567	-2.40775497	9.761125267	9.443177293	10.82615578	8.992733751	9.0282492	8.758581951	8.966291302
ENSG00000137713	PPP2R1B	-0.617945508	0.555396013	-1.791555442	0.054407162	-2.40950095	8.075571721	9.849218119	10.79563557	9.343906001	9.32771142	8.151214006	8.195234077
ENSG00000100325	ASCC2	-1.310325259	0.141361699	-1.100083477	0.217009028	-2.410408736	10.36714848	10.40166101	8.175061096	8.567402595	8.72280968	8.788960186	8.91909319
ENSG00000002330	BAD	-1.316928886	0.010257088	-1.093999654	0.035245998	-2.41092854	10.19941743	9.680074956	9.06622548	8.378048624	8.4504872	8.498570381	8.760149043
ENSG00000228474	OST4	-0.950055588	0.255415577	-1.461186493	0.063181453	-2.411242081	11.93368604	10.24697497	10.28245311	10.14060551	10.0678647	9.421247846	9.751494798
TXNRD3NB		2.779646541	0.633071268	-5.191095194	NA	-2.411448653	2	2	2	3.754611435	3.08597292	2	2
ENSG00000125991	ERGIC3	-0.622827783	0.227473008	-1.788743136	8.35E-05	-2.411570919	12.34389158	11.5089946	11.58086679	11.22647859	11.2550054	9.838386291	10.28324346
CCL4L1	CCL4L1	2.77917106	0.633108144	-5.191095196	NA	-2.411924136	2	2	2	3.709785322	3.15372745	2	2

ENSG00000187147	RNF220	-1.614846515	7.06E-11	-0.797787146	0.00214576	-2.412633661	10.15425007	10.34373336	10.40882047	8.654247601	8.74631819	9.400276454	9.615992417
ENSG00000183527	PSMG1	-1.483496717	0.065152211	-0.929834309	0.262970634	-2.413331026	9.613202746	7.60566235	8.585098385	7.348537858	7.37584259	7.726402307	8.063441666
ENSG00000100216	TOMM22	-1.21282245	0.16375923	-1.201348877	0.159642087	-2.414171327	10.26462289	9.962611189	8.143093325	8.425603456	8.58234702	8.16558624	8.800186246
ENSG00000161204	ABCF3	-0.697375118	0.144179754	-1.717773543	5.72E-05	-2.415148662	10.35389212	9.828248506	9.503962296	9.16555511	9.31943747	8.054501123	8.393643421
ENSG00000233013	FAM157B	2.775837411	0.63346707	-5.19109521	NA	-2.415257799	2	2	2	3.349643765	3.55551834	2	2
ENSG00000142156	COL6A1	-1.080159768	0.001097079	-1.335120741	3.45E-05	-2.415280509	15.47506374	15.88236009	16.10171637	14.74860836	14.7761579	14.53459064	14.47994585
ENSG00000142408	CACNG8	1.031173708	0.164591992	-3.447823315	1.75E-07	-2.416649607	7.031698319	6.04685525	7.098165388	7.731524332	7.8441483	4.099885429	3.36036153
ENSG00000103510	KAT8	-1.405839974	0.202605116	-1.011063154	0.365582948	-2.416903127	7.579279349	6.899080544	9.624627483	7.053486362	7.21069602	7.562295432	7.474637727
ARL16	ARL16	-1.689851255	0.059194088	-0.727098403	0.44897681	-2.416949658	10.3933013	8.820493869	8.143093325	7.767511442	7.76240929	8.585635246	8.833258322
ENSG00000109084	TMEM97	-3.994036968	0.000161536	1.576876245	0.17143346	-2.417160723	6.563806279	9.890264534	9.287079172	5.258265913	5.28958453	10.00444935	11.15428371
ENSG00000176142	TMEM39A	-1.061123227	0.025464282	-1.356942512	0.003062764	-2.418065739	10.06758145	10.62479571	9.681375057	9.088859521	9.15867957	8.975707507	8.669391068
ENSG00000168303	MPLKIP	-1.446987185	0.003251505	-0.974791901	0.05485101	-2.421779085	8.631580145	8.356994601	9.372276026	7.373900638	7.46855365	7.857806412	7.911296863
ENSG00000196981	WDR5B	-1.227634633	0.305395568	-1.194966814	0.308265999	-2.422601447	8.313285031	6.464272742	5.39244166	6.006476466	6.08095082	6.363520437	5.713290392
ENSG00000100926	TM9SF1	0.744337927	0.120540527	-3.168215297	2.92E-14	-2.423877371	9.730679447	9.680074956	8.845626171	10.17296029	10.2564019	6.285453595	6.449128733
ENSG00000154928	EPHB1	0.156378889	0.958503495	-2.580359002	0.320401275	-2.423980113	2	2	7.342949392	5.949776857	5.98191036	3.709392871	3.640730416
ENSG00000165338	HECTD2	-1.130992805	0.497739811	-1.293126407	0.423874761	-2.424119212	3.327711234	8.395519306	8.805342642	6.965655398	6.9210099	6.920094328	6.640162436
ENSG00000100380	ST13	-1.073394837	0.000288085	-1.35292882	2.77E-06	-2.426323656	11.71783599	11.21797502	11.41005835	10.41732378	10.3649055	10.05770853	10.16578017
ENSG00000160014	CALM3	-1.507094011	8.30E-07	-0.91924387	0.003997578	-2.426337881	12.27171276	11.92243809	12.25299971	10.58958544	10.7125292	11.02190347	11.42774592
ENSG00000244038	DDOST	-0.53102967	0.196009997	-1.895794324	9.77E-08	-2.426823993	12.49946702	11.86696895	12.46016642	11.73985636	11.8032062	10.25766199	10.54730407
ENSG00000198417	MT1F	-0.457299918	0.864318577	-1.972431597	0.397984664	-2.429731514	5.866944937	3.076665209	2	4.139692846	4.22485516	3.724264246	2.225841198
ENSG00000066427	ATXN3	-0.84829045	0.241359928	-1.582866209	0.017961959	-2.431156659	9.174465797	7.954278496	9.553445883	8.219202381	8.16874151	7.670871435	7.242500282
ENSG00000120158	RCL1	-1.160191438	0.086279573	-1.271025631	0.052104808	-2.431217069	6.653098118	6.602424674	7.777116083	5.920568914	6.0722229	5.737015686	6.037449234
ENSG00000037241	RPL26L1	-1.112851175	0.217853755	-1.318925339	0.129597634	-2.431776514	9.128497779	7.701025996	7.029880454	7.017154451	7.24216308	6.800644007	7.056667034
ENSG00000164008	C1orf50	-0.972882603	0.362218166	-1.459371044	0.146023342	-2.432253647	4.815609471	7.393647515	7.398165132	6.024891728	5.98191036	5.630526941	5.470251342
ENSG00000089199	CHGB	0.72863364	0.815259329	-3.161053607	NA	-2.432419967	2	3.076665209	2	2.947924294	2.77779907	2.094598369	2.059860178
ENSG00000173137	ADCK5	-0.894661572	0.679561713	-1.538575323	0.447991703	-2.433236895	4.815609471	7.979858146	2	5.602782483	5.85535975	5.163941955	5.136701757
ENSG00000071994	PDCD2	-1.345766343	0.085059099	-1.087555679	0.165533397	-2.433322022	10.46286899	9.488434476	8.486635645	8.328872678	8.3920703	8.694893015	8.532824248
ENSG00000169288	MRPL1	-0.4523484	0.819093952	-1.981316235	0.246641741	-2.433664635	7.904282243	7.145017454	2	6.687949235	6.44101097	4.984105765	5.300743019
ENSG00000126261	UBA2	-1.065176416	0.003556198	-1.368805479	0.000108944	-2.433981895	10.64803757	10.31889309	10.06936278	9.364838907	9.24178305	9.171528462	8.812201472
ENSG00000166441	RPL27A	-1.077582217	0.216892178	-1.357541586	0.105389356	-2.435123803	13.20898726	11.54156729	11.25418149	11.19022482	11.2114531	10.88757038	10.95453551
ENSG00000188060	RAB42	-2.535197459	0.009053015	0.098780736	0.935481323	-2.436416724	5.70932728	6.464272742	7.934808822	4.76150579	4.56059747	6.479817188	7.523290521
ENSG00000092108	SCFD1	-0.311607502	0.200056839	-2.125006438	3.30E-25	-2.43661394	9.678463567	9.71095217	9.69246155	9.398771806	9.37016537	7.628021136	7.55613706
ENSG00000092445	TYRO3	-1.4334481	0.466616843	-1.00397755	0.614013611	-2.437425651	8.696033109	8.762721501	2	6.820355514	6.68379438	7.450478673	6.81969491
ENSG00000183066	WBP2NL	-3.274201019	0.39960716	0.835741962	NA	-2.438459057	2	3.076665209	2	2	2.14022021	2.775316226	3.042026731
ENSG00000170791	CHCHD7	-0.927506885	0.219104153	-1.511349067	0.032176413	-2.438855952	8.963744667	7.638155693	7.451345288	7.238438152	7.30683517	6.621594001	6.78907279
ENSG00000100154	TTC28	-1.434657469	0.044804086	-1.005168975	0.169441906	-2.439826444	9.128497779	10.24697497	10.92816184	8.924487942	8.77214691	9.133040681	9.407171982
ENSG00000147912	FBXO10	-0.32217918	0.707966552	-2.118278493	0.002715313	-2.440457673	6.89194353	7.638155693	8.65477198	7.638916932	7.53675633	6.013120342	5.699242824
ENSG00000189043	NDUFA4	-1.408724358	0.036457816	-1.032224967	0.13261638	-2.440949324	11.25516263	9.910357957	9.868959282	9.12771691	9.06390621	9.544459308	9.39196883
ENSG00000011009	LYPLA2	-1.167205954	0.007244134	-1.275329222	0.002785432	-2.442535176	9.940323746	9.386925327	9.099922433	8.332573384	8.38855922	8.129732687	8.368713182
ENSG00000107833	NPM3	-1.401625857	0.500480698	-1.046764268	0.617051806	-2.448390125	3.327711234	3.686303414	8.536706852	5.775854763	5.62411716	6.132200015	5.925257518
ENSG00000125967	NECAB3	-0.968328445	0.249938781	-1.483047119	0.06093067	-2.451375565	9.317904228	7.431218269	9.344432377	7.915493556	8.01555453	7.576706933	7.336254871
ENSG00000112149	CD83	-1.17879237	0.522626682	-1.273926492	0.476851144	-2.452718862	9.410448395	6.899080544	3.369317972	6.951285179	6.89649843	6.706424753	6.947999751
ENSG00000165644	COMTD1	-1.441929243	0.538564148	-1.010838243	0.672486215	-2.452767486	2	3.076665209	7.502636426	4.670864177	4.74762503	4.965450964	5.191251321
ENSG00000071539	TRIP13	-1.57508851	0.033658406	-0.878145479	0.250261337	-2.453233989	5.532353208	6.311476754	6.322058544	4.739373666	4.63360314	4.834511823	5.646532192
ENSG00000206530	CFAP44	0.064394262	0.976726726	-2.517690141	0.165966488	-2.453295879	2	5.303523747	7.777116083	6.573765157	6.44101097	4.45225155	3.924507493
ENSG00000102710	SUPT20H	-0.700168705	0.104334281	-1.753769447	5.48E-06	-2.453938151	10.04301211	10.00683004	9.399592459	9.240349764	9.05507424	8.27442094	7.919398473
ENSG00000083457	ITGAE	-1.732153583	0.204801787	-0.721804029	0.62045962	-2.453957612	8.816894989	7.393647515	4.523675391	5.987823101	6.14893615	6.855708005	7.198237561
ENSG00000165572	KBTBD6	-1.715287526	0.016480221	-0.739159184	0.337319235	-2.45444671	7.33131396	8.57407404	9.031722608	6.744602355	6.84106088	8.015738956	7.408544534
ENSG00000115539	PDCL3	-0.747051005	0.417929197	-1.707457056	0.04003872	-2.454508061	8.963744667	7.431218269	7.225742779	7.250225364	7.46522407	6.028179536	6.750993196
ENSG00000164972	C9orf24	0.180799142	NA	-2.635600556	NA	-2.454801415	2	3.076665209	2	2.141737246	3.01487888	2.048074405	2.172608095
ENSG00000255071	SAA2-SAA4	2.735440379	0.638989421	-5.191095378	NA	-2.455654999	2	2	2	3.615725078	3.2184422	2	2
ENSG00000141448	GATA6	-1.774247105	0.217082182	-0.681643909	0.660486629	-2.455891014	5.095959458	9.461450882	9.963514551	7.481152682	7.37229174	8.644477784	8.344849238
ENSG00000100351	GRAP2	2.734950075	0.638989421	-5.19109538	NA	-2.456145305	2	2	2	3.566290607	3.28037825	2	2
ENSG00000143452	HORMAD1	2.734950075	0.638989421	-5.19109538	NA	-2.456145305	2	2	2	3.566290607	3.28037825	2	2
ENSG00000168028	RPSA	-0.660448224	0.125657681	-1.795976725	2.78E-06	-2.456424949	12.12920672	11.81817171	11.29839064	11.09892761	11.1580635	9.950137104	10.04115072
ENSG00000145248	SLC10A4	-0.654581947	0.834689821	-1.802813409	0.524456316	-2.457395356	4.00722531	2	2	2.947924294	2.38539787	2.183373728	2.509591799
ENSG00000128266	GNAZ	-3.495302408	0.078054688	1.037158048	0.631197419	-2.458144361	7.218819781	3.686303414	2	3.406930997	2.68924543	6.639356243	6.881131115
ENSG00000115657	ABCB6	0.23366888	0.897633629	-2.694035618	0.066291657	-2.460366738	3.327711234	7.538394143	8.65477198	7.810605345	7.92635454	5.147485955	5.04340913
ENSG00000171291	ZNF439	-1.476717063	0.097724298	-0.985806346	0.278635374	-2.462523409	8.777722034	6.464272742	8.266922072	6.761173376	6.58294721	7.190239142	7.107191496
ENSG00000157911	PEX10	-1.438990687	0.010939322	-1.024994481	0.077118013	-2.463985168	9.174465797	7.928237109	8.511888463	7.136275664	7.27676203	7.489084836	7.726263462
ENSG00000145388	METTL14	-1.005462991	0.326315684	-1.459239239	0.131842802	-2.46470223	9.613202746	7.315436877	9.981704053	8.395844809	8.29788471	8.092965353	7.678134128
ENSG00000120833	SOCS2	-1.703404432	0.007905366	-0.764833759	0.26685414	-2.468238191	9.048471759	8.590771778	7.451345288	6.902323594	6.77267312	7.744751472	7.761616892
ENSG00000078177	N4BP2	-0.742726027	0.674378575	-1.72719082	0.282968343	-2.469916847	4.815609471	9.386925327	5.930865648	7.373900638	7.13728069	6.484192226	6.117095472
ENSG00000108010	GLRX3	-0.927438342	0.186621771	-1.543306874	0.01873645	-2.470745216	10.47516499	8.820493869	9.788583153	8.951212326	8.89106117	8.281350892	8.341838298
ENSG00000176973	FAM89B	-0.650222403	0.135425564	-1.820824546	2.32E-06	-2.471046949	9.579430841	8.955547846	8.978369997	8.508095759	8.59915299	7.546693093	7.242500282
ENSG00000141577	CEP131	-1.503146073	0.349375154	-0.968407835	0.554691707	-2.471553908	4.00722531	6.602424674	8.978369997	6.29903474	6.1572134	6.768721236	6.711881188
ENSG00000156171	DRAM2	-1.378331148	0.05355269	-1.097398895	0.127604991	-2.475730043	9.810482605	8.505280662	9.963514551	8.205140657	8.17078703	8.828637742	7.978752403
ENSG00000113240	CLK4	-1.282797327	0.459733797	-1.193154164	0.483761199	-2.475951491	3.327711234	8.255926468	9.272377397	7.080147179	6.96881778	7.402744819	6.748721609
ENSG00000163694	RBM47	0.221322555	0.912449258	-2.698238287	0.099707517	-2.476915732	3.327711234	8.124485672	5.930865648	7.148924055	7.02868983	5.04460184	3.311948089
ENSG00000163479	SSR2	-1.240945569	7.78E-06	-1.236669843	7.69E-06	-2.477615413	12.31339889	11.87380718	11.94533713	10.80101819	10.8343167	10.78391875	10.85917895
ENSG00000090273	NUDC	-1.318668884	2.14E-06	-1.158976386	3.35E-05	-2.47764527	10.81695923	10.57950211	10.78531698	9.449072612	9.38257346	9.382222556	9.745241486
ENSG00000111885	MAN1A1	-0.972724244	0.410778027	-1.505252507	0.174488324	-2.477976751	8.138771685	11.40661699	10.94679967	9.718812329	9.73198422	8.817412208	9.49551136
ENSG00000092931	MFSD11	-0.497911016	0.506773631	-1.980318906	0.001927913	-2.478229923	9.590776211	8.053983481	8.585098385	8.385193462	8.39732094	7.042323222	6.828326017
ENSG00000064490	RFXANK	-0.550785144	0.41863397	-1.92864787	0.001004097	-2.479433014	8.716893051	8.732943944	7.398165132	7.942383523	7.78127547	6.535683256	6.514766253
ENSG00000276429		-0.289992535	0.926059063	-2.190312314	NA	-2.480304848	2	3.686303414	2	2.868578001	2.59489879	2.34604626	2.117335275
ENSG00000274675		5.533347298	3.06E-07	-8.014388639	2.83E-05	-2.481041342	4.00722531	3.686303414	2	8.408172799	8.3920703	2	2
ENSG00000047579	DTNBP1	-1.541893308	0.103759715	-0.940463524	0.334815084	-2.482356831	5.532353208	7.979858146	7.646393253	5.720439317	6.0722229	6.482006365	6.468583424
ENSG00000240230	COX19	-1.191135624	0.481054906	-1.292312467	0.432545818	-2.483448091	4.467311984	8.433041974	10.11127249	7.734324667	7.7919464	8.060373167	7.116030435
ENSG00000205138	SDHAF1	-1.912580883	1.14E-06	-0.572103521	0.187327641	-2.484684404	8.517314938	7.819099893	8.266922072	6.306580752	6.41363412	7.740186014	7.571641661
ENSG00000132589	FLOT2	-1.791795935	0.001903145	-0.693013459	0.273571221	-2.484809394	11.59363617	10.19877383	10.76969399	9.134092596	9.23006009	10.27180772	10.28088727
ENSG00000114503	NCBP2	-1.259509244	0.038369791	-1.225589541	0.042265942	-2.485098785	11.4295719	10.019219	10.59756881	9.56371781	9.52209753	9.542100683	9.61068621
ENSG00000136270	TBRG4	-1.692754155	0.016213051	-0.794770971	0.291201113	-2.487525126	10.41260941	8.590771778	9.809100877	8.033874364	8.17078703	8.980360725	9.003921351
ENSG00000204967	PCDHA4	-0.797552948	0.773531565	-1.691651574	0.510353967	-2.489204521	2	4.93638933	2	3.663521627	2.59489879	2.885263063	2.671842496
ENSG00000172803	SNX32	-3.590444403	0.117068598	1.09847501	0.64228189	-2.491969392	2	5.303523747	2	2.3892346	2.2680111	5.169385838	4.644600858
ENSG00000101391	CDK5RAP1	-1.086453648	0.551434318	-1.405731389	0.424102084	-2.492185037	3.327711234	8.234834549	9.778213818	7.477814553	7.6378145	7.190239142	7.30412922
ENSG00000104408	EIF3E	-1.162283456	0.018126811	-1.330553564	0.005669552	-2.49283702	11.44528213	10.65289571	10.47451889	9.767470067	9.75874862	9.700188698	9.482925003
ENSG00000197223	C1D	-0.743765488	0.261139503	-1.749680458	0.003253036	-2.493445945	8.39321512	7.002537973	8.042706615	7.119236776	7.24992376	6.213498222	6.22561877
ENSG00000138617	PARP16	-1.684196757	0.307444336	-0.809832031	0.64013824	-2.494028788	3.327711234	7.60566235	8.978369997	6.205262847	6.29861567	7.354572674	6.771278116
ENSG00000164904	ALDH7A1	-1.379845873	0.169072016	-1.115312513	0.267163049	-2.495158386	6.366709671	9.125613817	8.460932916	7.057964187	6.98750599	7.17543541	7.378005688
ENSG00000173486	FKBP2	-0.74082676	0.089224711	-1.755905645	7.55E-06	-2.496732405	10.09970294	9.532315021	9.61300511	9.033093526	9.03275482	7.749302529	8.26365177
ENSG00000072195	SPEG	-2.369885796	1.98E-27	-0.126913143	0.64228189	-2.496798939	10.34051281	10.20420974	10.16000817	7.930222906	7.84158259	10.16430247	10.05385647
ENSG00000175376	EIF1AD	-1.373307353	0.084538754	-1.126137762	0.157821042	-2.499445115	8.963744667	8.356994601	10.26027233	8.040677544	8.0713677	8.269359984	8.3297313
ENSG00000136305	CIDEB	2.69135397	0.644717004	-5.191095562	NA	-2.499741591	2	2	2	3.615725078	3.15372745	2	2
ENSG00000084072	PPIE	-1.183533564	0.000170819	-1.319550952	1.92E-05	-2.503084516	9.031919741	9.506147172	9.385998894	8.083034979	8.21902896	8.080016699	7.954317658
ENSG00000084623	EIF3I	-1.258944205	0.016890353	-1.244518511	0.017714167	-2.503462716	12.02014453	11.31412071	10.89495479	10.21466712	10.244312	10.06210503	10.40544175
ENSG00000166448	TMEM130	-3.306543218	0.015891709	0.80250793	0.600600207	-2.504035288	5.095959458	4.11352228	7.600047325	3.406930997	3.69849104	6.484192226	7.55093161
ENSG00000170638	TRABD	-1.214990905	0.023974609	-1.291229392	0.014940374	-2.506220297	9.204317094	8.848535039	9.999667074	8.209172298	8.24255817	8.137391755	8.164036804
ENSG00000026297	RNASET2	-0.604166158	0.39960716	-1.90405453	0.002334223	-2.508220688	9.344956677	7.901717006	9.344432377	8.409925372	8.39031583	7.156371901	7.09293558
ENSG00000114316	USP4	-1.358396914	0.210105576	-1.149886507	0.287769738	-2.508283421	8.418903792	10.01303782	11.4724895	9.091045942	9.11791381	9.270781246	9.351374718
ENSG00000132254	ARFIP2	-1.688715308	4.33E-05	-0.820004426	0.064008656	-2.508715734	10.76782679	9.981728319	10.20715117	8.637890117	8.71582187	9.363049315	9.697162691
ENSG00000120438	TCP1	-1.343045428	0.014834268	-1.167558676	0.035608878	-2.510604104	11.69961208	10.56267266	10.70548138	9.730079663	9.75670736	9.880276569	9.955102659
ENSG00000056998	GYG2	-1.275670648	0.542790331	-1.235176457	0.549961603	-2.510847105	5.095959458	8.101365653	2	5.627094009	5.39133044	5.185595313	5.842465082
ENSG00000149929	HIRIP3	-1.920024056	0.049155716	-0.591005282	0.58225381	-2.511029338	7.975256505	7.669933291	5.39244166	5.363304456	5.71663161	7.027411703	6.538630327
ENSG00000087884	AAMDC	-0.508865694	0.361961292	-2.002402428	2.64E-05	-2.511268122	7.790810799	7.145017454	7.163363831	6.89733479	6.90143415	5.243518884	5.703940561
ENSG00000206452		7.351477013	1.74E-28	-9.863379109	4.03E-19	-2.511902097	8.39321512	6.899080544	7.451345288	15.03289325	15.033612	2.139668783	2
ENSG00000189077	TMEM120A	-1.013818542	0.218893333	-1.500562585	0.053864969	-2.514381127	8.107517749	8.234834549	9.788583153	7.88809286	7.95749494	7.277267764	7.597118029
ENSG00000132383	RPA1	-1.800693141	1.50E-10	-0.713940931	0.018611632	-2.514634072	10.41260941	10.06176125	10.53119344	8.517880242	8.59306436	9.626899466	9.642234599

ENSG00000162755	KLHDC9	-4.714897443	0.260096091	2.194821195	0.579335184	-2.520076248	3.327711234	2	2	2	2	3.663836244	3.753786374
ENSG00000023734	STRAP	-1.585814145	4.55E-19	-0.934286384	2.32E-07	-2.52010053	11.53611196	11.59220064	11.46604358	9.97822644	9.92369416	10.57355555	10.6272262
ENSG00000255302	EID1	-1.345382648	0.010846156	-1.175038423	0.027547688	-2.520421071	12.5114563	11.40897279	11.9030147	10.73544052	10.5971045	10.57624762	11.05929667
ENSG00000025770	NCAPH2	-0.965401479	0.522626682	-1.556370322	0.269833543	-2.521771802	9.262228249	8.213429689	4.523675391	7.261917051	7.40393901	6.7543034	6.769038269
ENSG00000155957	TMBIM4	0.482971231	0.616184132	-3.004908379	0.000143403	-2.521937148	10.65885801	9.214490408	8.561105505	10.24001146	10.2267227	6.541992314	7.008112295
ENSG00000186395	KRT10	-1.874933779	0.000458914	-0.647948757	0.271141542	-2.522882535	8.517314938	7.669933291	8.486635645	6.379946408	6.50077304	7.249266052	7.931466218
ENSG00000179409	GEMIN4	-1.948956231	0.036163998	-0.576433476	0.577440741	-2.525389706	10.4253394	8.655694736	8.076950877	7.437142666	7.53992502	8.758581951	8.921111931
ENSG00000133250	ZNF414	-1.439957176	0.158024792	-1.090019896	0.288238766	-2.529977072	6.138360429	7.572420273	8.865353883	6.554880361	6.48112613	6.759726988	6.944034147
ENSG00000095370	SH2D3C	-4.136307932	0.13805413	1.605422752	0.482371468	-2.53088518	2	3.076665209	3.369317972	2.141737246	2	4.199031499	3.539239711
ENSG00000064961	HMG20B	-1.917185419	0.011775752	-0.614019779	0.468899775	-2.531205197	11.45151864	9.443177293	10.40208303	8.660150093	8.8354102	10.14940096	9.933764315
ENSG00000187800	PEAR1	-4.029083255	0.000130593	1.497766824	0.191871601	-2.53131643	9.48530526	7.928237109	6.073378139	4.54891529	4.70309173	9.944988515	9.866740633
ENSG00000140382	HMG20A	-1.101509743	0.259792504	-1.430309444	0.125991152	-2.531819187	8.107517749	9.856140801	10.75919294	8.80037045	8.87734839	8.501271034	8.526208204
ENSG00000131475	VPS25	-1.716982965	3.83E-11	-0.816160463	0.002803772	-2.533143428	10.29961392	10.11467741	9.972637969	8.501535708	8.35477078	9.239097094	9.402483342
ENSG00000249915	PDCD6	-1.418723484	0.007048618	-1.114702138	0.037743848	-2.533425621	10.98115017	9.994333773	9.954333071	8.957217624	9.00321357	9.201396821	9.358840932
ENSG00000140740	UQCRC2	-1.164271916	0.084747221	-1.369347333	0.036253153	-2.533619249	11.78851328	10.75272247	10.23774519	9.914221922	9.91273599	9.670982446	9.746665075
ENSG00000105875	WDR91	-1.142941207	0.348943914	-1.391322395	0.236277861	-2.534263602	6.816637977	6.602424674	9.287079172	6.941624891	7.05107174	7.039353222	6.406535141
ENSG00000138439	FAM117B	-1.603435067	0.095126919	-0.931159035	0.348561445	-2.534594102	6.009025997	8.376385542	7.163363831	6.12217689	5.78232107	6.835304498	6.317379004
ENSG00000162676	GFI1	-1.149162646	0.64718241	-1.386186799	0.564260714	-2.535349444	2	4.442710129	2	2.784612485	2.77779907	2.62493832	2.746601469
ENSG00000104823	ECH1	-1.064238361	0.065953631	-1.471430642	0.007688776	-2.535669004	10.66423795	9.558016677	9.735974718	8.986874783	9.03724641	8.381493453	8.805113667
ENSG00000163319	MRPS18C	0.736875933	0.336111036	-3.275694411	6.73E-07	-2.538818478	7.384433967	6.844429755	5.772716771	7.433701013	7.61995902	3.724264246	4.105828626
ENSG00000115163	CENPA	-1.034967767	0.594008398	-1.505836243	0.408557169	-2.54080401	3.327711234	4.442710129	2	2.868578001	3.15372745	2.592885947	2.981406154
ENSG00000099817	POLR2E	-1.462215913	6.63E-08	-1.078956419	9.13E-05	-2.541172332	11.28689767	10.93049815	11.19694409	9.691694368	9.68194672	9.929030467	10.19568993
ENSG00000057935	MTA3	-1.638743131	0.045222505	-0.903196302	0.291783001	-2.541939433	8.340423424	9.055168103	10.39531398	7.910550164	7.88209639	8.557366484	8.684325286
ENSG00000166845	C18orf54	-2.010427205	0.249656226	-0.533196495	0.78196159	-2.543623701	6.73718411	7.572420273	2	4.984880435	4.60967607	6.546183077	5.596882677
ENSG00000152217	SETBP1	-2.307417779	0.399970556	-0.236594169	0.939029732	-2.544011949	2	7.467835385	2	4.497095146	3.15372745	5.790986459	5.680297397
ENSG00000152556	PFKM	-1.161165901	0.041111897	-1.385061597	0.012102831	-2.546227498	10.905938	10.36815316	9.757248847	9.30110636	9.22317745	8.710380719	9.308834477
LOC643802		2.644834365	0.650789466	-5.191095755	NA	-2.54626139	2	2	2	3.515101962	3.2184422	2	2
ENSG00000173421	CCDC36	-4.302539298	0.080939257	1.754977963	0.440478859	-2.547561335	2	2	4.874587708	2.141737246	2.14022021	5.113999907	4.810605986
ENSG00000173068	BNC2	-1.906033142	0.192994348	-0.641585281	0.686960124	-2.547618423	4.467311984	9.461450882	8.585098385	6.64692533	6.6952083	7.625052652	8.134761809
ENSG00000079785	DDX1	-0.730365011	0.152640264	-1.817621152	6.70E-05	-2.547986163	11.50346787	10.60848937	10.64399316	10.27505029	10.2286877	9.099213681	9.237724211
ENSG00000267680	ZNF224	0.115567252	0.851541104	-2.663737628	6.59E-09	-2.548170376	8.564112515	7.928237109	8.884815469	8.607665593	8.62921659	6.101204172	5.717942682
ENSG00000025156	HSF2	-1.404530659	0.00103094	-1.145778391	0.00795665	-2.55030905	7.975256505	8.124485672	8.608698767	6.907295206	6.86146731	7.328045423	6.911910672
ENSG00000176531	PHLDB3	-1.342017454	0.047187606	-1.208568546	0.071752409	-2.550586	7.53297911	6.844429755	7.691296561	6.12217689	6.08095082	6.647180917	5.631815919
ENSG00000162065	TBC1D24	-1.021005853	0.165981382	-1.529770818	0.027684956	-2.550776671	9.015175615	7.954278496	9.624627483	7.925329813	8.07792246	7.729169604	7.237648733
ENSG00000087086	FTL	-0.595571128	0.474577536	-1.955843277	0.006881281	-2.551414404	16.60962533	15.07629484	15.31092757	15.14447115	15.3260317	14.24616937	13.38261223
ENSG00000187994	RINL	-2.043352044	0.077472236	-0.50816533	0.691742513	-2.551517375	4.00722531	5.72254323	6.958202216	4.139692846	4.2865223	5.805044075	5.191251321
ENSG00000142937	RPS8	-1.806521396	0.005229301	-0.746404159	0.289477544	-2.552925555	13.4577386	12.25916332	11.94765236	10.89848915	10.9127981	11.96837804	11.96003159
ENSG00000165895	ARHGAP42	-1.860656202	0.02322092	-0.693177086	0.437976776	-2.553833287	8.674867122	8.414402626	6.534109959	6.422239259	6.22964624	7.54878318	7.357281305
ENSG00000137700	SLC37A4	-2.068533792	0.133069745	-0.486992724	0.752096732	-2.555526516	9.174465797	8.276714465	4.523675391	6.321555392	6.14893615	7.747483829	7.787298144
ENSG00000196470	SIAH1	-1.035211346	0.61318036	-1.520450822	0.434760049	-2.555662169	2	7.761270542	9.06622548	6.927012081	7.00595521	6.564892306	6.432241896
ENSG00000116001	TIA1	-0.734038039	0.241411697	-1.821743751	0.001138858	-2.555781791	10.31337612	10.28338212	9.083172337	9.32080297	9.20931286	8.344674564	8.014172904
ENSG00000086848	ALG9	-0.650027665	0.386972978	-1.906734159	0.003824718	-2.556761824	7.790810999	9.415325457	8.486635645	8.020170999	8.13979554	6.953609309	6.746446439
ENSG00000163636	PSMD6	-0.910745534	0.248988278	-1.646048421	0.024540431	-2.556793955	11.25516263	9.785372135	9.624627483	9.567648462	9.45905186	8.669665542	8.890530144
ENSG00000108592	FTSJ3	-0.960510941	0.099701083	-1.596632906	0.003306461	-2.557143846	10.54098126	9.338312924	9.859153765	9.000896538	9.07814384	8.187215117	8.603037398
ENSG00000170779	CDCA4	-1.506535558	0.446883723	-1.050964245	0.599804676	-2.557499803	9.143983763	8.276714465	2	6.777556219	6.67228943	7.010829034	7.307219812
	GRAMD3	5.84523908	0.002088916	-8.403369954	0.000643868	-2.558130875	2	4.93638933	2	9.115954467	9.07268445	2	2
ENSG00000138073	PREB	-1.843309437	3.28E-13	-0.717064659	0.008040575	-2.560374097	10.43795805	10.1432577	10.19160738	8.36726458	8.49198035	9.625662378	9.46608577
ENSG00000055044	NOP58	-0.871078877	0.391678983	-1.689929696	0.070032223	-2.561008573	10.48127392	8.955547846	8.007629717	8.601543953	8.68180105	7.667989909	7.98359008
ENSG00000006283	CACNA1G	-0.195789567	0.946040014	-2.365925276	0.350450714	-2.561714843	5.095959458	2	2	3.921164147	3.33976443	2.803598391	2.277179783
ENSG00000260027	HOXB7	2.225840194	0.17177866	-4.788035147	0.00161096	-2.562194953	6.257048221	6.140560021	2	7.778406195	7.81305436	2.775316226	2.172608095
ENSG00000175426	PCSK1	-0.042376226	0.985464453	-2.520318135	0.209322464	-2.562694362	4.00722531	3.076665209	2	3.162721474	3.28037825	2.139668783	2.466008509
ENSG00000100478	AP4S1	-0.948620416	0.1187722	-1.614094853	0.00411419	-2.562715269	7.435667355	6.602424674	6.718950348	6.221320894	5.86549842	5.243518884	5.611957759
ENSG00000137154	RPS6	-2.117565557	0.000754913	-0.446278858	0.542075443	-2.563844414	13.91124438	12.65219511	12.48588269	11.06512699	11.0362957	12.64097106	12.79530838
ENSG00000164919	COX6C	-1.323853676	0.181146687	-1.241042578	0.204538137	-2.564896254	11.19324461	8.623598428	9.61300511	8.873322599	8.88359764	8.996146925	8.924134755
ENSG00000128283	CDC42EP1	-2.462144322	1.03E-05	-0.104904689	0.886770342	-2.56704901	11.65166541	10.42977533	11.78627063	8.941551548	8.95891978	11.24494713	11.34933218
ENSG00000088832	FKBP1A	-0.911099104	0.046672139	-1.659362251	9.35E-05	-2.570461355	12.78892924	12.15073083	12.82880267	11.71011514	11.7106329	10.63583525	11.23002844
ENSG00000173692	PSMD1	-0.977842178	0.00912284	-1.593006423	7.08E-06	-2.570848601	11.83695787	11.2524908	11.63070764	10.57442844	10.6570657	9.778197243	10.19777306
ENSG00000174684	B4GAT1	-1.932399052	0.028063103	-0.639924708	0.510549382	-2.57232376	8.564112515	9.756063186	11.00131064	8.170409056	8.19309833	9.404322061	9.518360533
ENSG00000110442	COMMD9	-1.260664045	0.01578088	-1.311695765	0.010977411	-2.572359809	9.931427102	9.030900487	9.180874355	8.246921218	8.12299044	7.833151805	8.386355612
ENSG00000197858	GPAA1	-1.523865517	0.023379435	-1.048609463	0.129694026	-2.57247498	11.90443341	11.00713613	10.2528023	9.659644277	9.72295127	10.01544857	10.30040559
ENSG00000137288	UQCC2	-1.251444048	5.20E-05	-1.323561371	1.28E-05	-2.575005419	8.609439662	8.607278467	8.699419242	7.409376773	7.40393901	7.16729616	7.488317792
ENSG00000117385	P3H1	-1.259894582	0.000302512	-1.31686521	0.000139067	-2.576759792	12.79261849	12.0991443	12.47147373	11.24500602	11.2004816	11.17706738	11.15492717
ENSG00000197915	HRNR	-1.713156076	0.536668932	-0.864937987	0.76003079	-2.578094063	2	2	4.523675391	2.784612485	2.2680111	2.68698273	3.042026731
ENSG00000197557	TTC30A	-0.525682284	0.51591099	-2.054473486	0.00290832	-2.58015577	7.159102577	6.899080544	8.325066185	7.044488801	7.11620658	5.328699035	5.88031069
ENSG00000166900	STX3	-0.403990471	0.693142866	-2.176662563	0.011271266	-2.580653034	7.218819781	8.834582582	9.465702942	8.293234263	8.44880239	7.012344451	6.141542327
ENSG00000123136	DDX39A	-0.747397278	0.461018278	-1.835200875	0.04279221	-2.582598153	10.30651143	8.124485672	8.511888463	8.573670512	8.57000035	7.264607048	7.704159082
ENSG00000155868	MED7	-1.354877831	0.187957925	-1.228695089	0.22807891	-2.58357292	9.048471759	6.464272742	7.451345288	6.777556219	6.66069199	6.923319986	6.755525668
ENSG00000013275	PSMC4	-0.86350316	0.009557939	-1.720831861	2.87E-08	-2.584335021	10.96815916	10.67655334	11.02781271	9.991161874	10.0804323	8.950238388	9.384672535
ENSG00000185774	KCNIP4	-3.084074005	0.145965965	0.499547159	0.814954199	-2.584526847	3.327711234	3.686303414	2	2.270786764	2.14022021	3.738983888	3.236138336
ENSG00000163517	HDAC11	-1.74837232	0.207275017	-0.838609577	0.565593677	-2.586981897	9.423197387	4.93638933	8.460932916	6.799114572	6.72891807	7.846806418	7.420010428
ENSG00000118620	ZNF430	-0.98702689	0.329061986	-1.600737849	0.090736393	-2.587764739	8.716893051	7.189546432	6.322058544	6.887305128	6.68379438	6.329884754	6.06327132
ENSG00000104549	SQLE	-1.383773033	3.96E-05	-1.204073959	0.000386271	-2.587846992	11.38790617	11.31412071	11.25043551	9.943557345	9.93336537	9.772172069	10.39603787
ENSG00000150995	ITPR1	-1.620199993	0.003298939	-0.969746244	0.093130425	-2.589946237	8.564112515	9.090820894	9.399592459	7.494428494	7.42465798	8.483898848	7.570355956
ENSG00000221829	FANCG	-1.545302846	0.018712352	-1.045057988	0.120202987	-2.590360834	8.009475818	7.051611315	6.629488387	5.870535235	5.86549842	6.490730008	6.168983563
ENSG00000168904	LRRC28	-1.769551214	0.313384316	-0.821419288	0.658193363	-2.590970502	3.327711234	7.189546432	9.426401271	6.237202172	6.57060569	7.27600668	7.379474672
ENSG00000149313	AASDHPPT	-1.0191591	0.000526024	-1.572086721	2.19E-08	-2.591245821	9.579430841	9.443177293	9.61300511	8.527598812	8.54024645	8.154648976	7.799420735
ENSG00000197798	FAM118B	-1.309153718	0.028899691	-1.282157518	0.030994241	-2.591311236	9.397585735	8.191702463	8.325066185	7.426792977	7.47849652	7.449360315	7.5085977
ENSG00000168306	ACOX2	-1.320073357	0.514104281	-1.271863345	0.521952482	-2.591936702	8.631580145	6.535001748	2	5.94010643	6.22964624	5.849807762	6.37742435
ENSG00000064995	TAF11	-1.485313498	0.008096943	-1.109078045	0.053144626	-2.594391543	9.740899611	8.451443573	9.344432377	7.872924734	7.72392024	8.249579355	8.087009646
ENSG00000117595	IRF6	-1.559325673	0.088235741	-1.036215921	0.257062588	-2.595541594	4.467311984	5.131603625	5.930865648	3.95996233	4.02226117	4.612704558	4.147809227
ENSG00000153982	GDPD1	-1.173957089	0.329606836	-1.422559614	0.218679367	-2.596516702	6.653098118	4.442710129	6.958202216	5.348759107	5.16377554	5.511266259	4.329022995
ENSG00000164053	ATRIP	-1.74446742	0.35107637	-0.85312825	0.664743846	-2.597595671	8.444143041	7.002537973	2	5.674521485	5.64780787	6.633459761	6.350712758
ENSG00000147852	VLDLR	-2.14589275	0.121803593	-0.452699259	0.773865459	-2.598592009	5.866944937	10.03150247	10.71094342	7.706073672	7.75697332	9.499512834	9.306133598
ENSG00000167315	ACAA2	-0.895151351	0.366470827	-1.70450735	0.061919368	-2.5996587	10.67493801	10.45279253	8.143093325	9.20040236	9.24275568	8.385007206	8.45344973
ENSG00000198612	COPS8	-1.208971145	0.022546075	-1.392596627	0.007169426	-2.601567771	10.89224845	9.71095217	10.2528023	9.140440229	9.18117744	9.039153561	8.915553582
ENSG00000132507	EIF5A	-1.170452283	0.095852222	-1.432166163	0.03456438	-2.602618446	13.1801726	11.60869109	12.27160023	11.30085596	11.3548657	10.73519466	11.33634299
ENSG00000110801	PSMD9	-0.426797024	0.27007879	-2.176125681	2.65E-11	-2.602922705	9.497412313	9.181799937	9.06622548	8.76657222	8.90218458	6.931352719	7.280736296
ENSG00000099800	TIMM13	-1.639907209	4.27E-05	-0.964477411	0.02217593	-2.60438462	10.27168932	9.856140801	9.735974718	8.241026137	8.44204342	8.708044758	9.266193276
ENSG00000184117	NIPSNAP1	-1.59298922	0.098030868	-1.01203173	0.305936987	-2.60502095	11.17074085	9.756063186	8.585098385	8.630393142	8.62325378	9.01024623	9.372921162
ENSG00000128463	EMC4	-1.172058568	0.000671618	-1.436448016	1.90E-05	-2.608506584	10.27168932	10.65289571	10.90452077	9.460955886	9.46906021	9.100282869	9.297612054
ENSG00000140319	SRP14	-1.111684397	0.026613498	-1.497065242	0.001875691	-2.60874964	12.39655881	11.30403016	11.77848812	10.77677453	10.7940784	10.34218565	10.45766062
ENSG00000122692	SMU1	-1.139544928	0.035422866	-1.469888805	0.004813198	-2.609433733	10.64803757	10.17682307	9.426401271	8.998569022	9.06939887	8.627767864	8.781973674
ENSG00000179292	TMEM151A	-3.642713847	0.022267907	1.032728626	0.538703409	-2.60998522	3.327711234	6.04685525	3.369317972	2.498691189	2.68924543	6.384736493	4.870334174
ENSG00000166199	ALKBH3	-1.44687535	0.392541278	-1.16352346	0.491522021	-2.61039881	4.467311984	6.899080544	9.735974718	6.993973425	6.91125523	7.010829034	7.417152486
ENSG00000130511	SSBP4	-1.451039229	5.97E-06	-1.159742067	0.000327357	-2.610781296	8.892187472	9.298208073	8.904018013	7.641902724	7.59275449	7.794921259	8.004685229
ENSG00000186222	BLOC1S4	-1.364973807	0.10052636	-1.246545702	0.129711398	-2.611519509	9.435834703	8.540087354	7.285536013	7.359462263	7.29187696	7.381793866	7.499169207
ENSG00000124802	EEF1E1	-1.758670335	0.171238625	-0.853210539	0.527954208	-2.611880874	9.613202746	7.467835385	5.930865648	6.604700657	6.77267312	7.486906381	7.649197382
ENSG00000271601	LIX1L	-1.682859333	0.000127603	-0.933408299	0.044614383	-2.616267632	10.00958825	9.994333773	10.80076732	8.612239836	8.67749148	9.271730593	9.497203828
ENSG00000239306	RBM14	-1.73607159	0.00477741	-0.881478543	0.177968622	-2.617550133	8.696033109	8.916342327	10.03493575	7.734324667	7.50468064	8.501271034	8.428831018
ENSG00000138036	DYNC2LI1	-1.06835703	0.183576111	-1.549586184	0.041032339	-2.617943213	7.031698319	6.666836837	8.35328246	6.476767609	6.52015598	5.99483933	6.088639344
ENSG00000172171	TEFM	-0.865238974	0.496953626	-1.753483537	0.13163848	-2.618722511	8.340423424	5.946638857	5.59507254	6.379946408	6.29861567	5.665620077	5.337485258
ENSG00000204764	RANBP17	-0.079418282	0.968054519	-2.539757575	0.120755432	-2.619175857	5.095959458	6.951736442	2	5.590471342	5.80356929	3.600766115	3.735548302
ENSG00000262814	MRPL12	-0.986216212	0.010979795	-1.633428064	7.73E-06	-2.619644275	9.015175615	9.558016677	9.399592459	8.458152491	8.26953254	7.523500045	7.910280955
ENSG00000157326	DHRS4	-0.548370075	0.552095038	-2.071467958	0.009438355	-2.619838033	7.435667355	6.844429755	8.784770914	7.359462263	7.40045674	5.744329515	6.077821908
ENSG00000136261	BZW2	-1.700552031	0.002045073	-0.920038741	0.114871963	-2.620590772	8.609439662	9.13702746	9.897982195	7.577865504	7.68150967	8.375029261	8.422432
ENSG00000111325	OGFOD2	-0.098276887	0.939485429	-2.523066777	0.014049355	-2.621343664	6.89194353	7.790474954	4.874587708	6.892328675	6.83074839	4.291799915	4.919632516
ENSG00000139163	ETNK1	-0.681545763	0.433017846	-1.940020778	0.011321064	-2.621566541	8.444143041	9.969011754	10.46808205	9.253098082	9.08466797	8.153275969	7.657697387

ENSG00000166188	ZNF319	-1.813418683	0.095496255	-0.809202493	0.483542133	-2.622621176	8.517314938	6.535001748	9.725218844	6.998639523	6.96410766	7.937338157	7.987448576
ENSG00000139684	ESD	-1.15286446	0.001617597	-1.470493779	3.33E-05	-2.623358239	10.69084077	10.20962523	10.07784268	9.195330254	9.21030762	8.745429394	9.017193688
ENSG00000172000	ZNF556	-1.689559495	NA	-0.934455208	NA	-2.624014703	2	2	3.369317972	2.141737246	2.2680111	2.560105256	2.059860178
ENSG00000139537	CCDC65	-4.55116024	0.129548839	1.926937082	0.452105431	-2.624223159	2	4.11352228	2	2	2.14022021	4.487545924	4.468068808
ENSG00000135929	CYP27A1	-1.016436552	0.428291678	-1.609317978	0.179430199	-2.625754529	10.32700827	7.35507212	8.236946975	8.080836374	8.27334517	8.122032742	6.764548117
ENSG00000198246	SLC29A3	-1.701685904	0.426363818	-0.924296957	0.677074052	-2.625982862	8.797441463	5.595927442	2	5.709096143	5.78232107	6.830158212	6.011156536
ENSG00000163832	ELP6	-1.391521201	0.000834812	-1.238942864	0.002969599	-2.630464064	8.468948322	8.607278467	9.132850258	7.330144653	7.45182838	7.344982741	7.714671806
ENSG00000172243	CLEC7A	-2.910230673	0.281095622	0.279452138	0.926160637	-2.630778536	2	3.076665209	4.523675391	2	2.59489879	4.076896059	3.287118176
ENSG00000083635	NUFIP1	-1.310248101	0.476911496	-1.322119671	0.462769314	-2.632367772	8.009475818	7.232742069	2	6.034011955	5.64780787	5.825876956	5.859408034
ENSG00000130429	ARPC1B	1.170717704	0.030750279	-3.803621366	6.75E-15	-2.632903662	9.751047882	9.055168103	10.20715117	10.86086404	10.956731	5.812021848	6.209190898
ENSG00000105048	TNNT1	-2.791980982	0.167654826	0.1586039	0.944157185	-2.633377082	2	4.710564154	3.369317972	2.270786764	2.59489879	4.397636241	3.183290849
ENSG00000104872	PIH1D1	-1.464657731	0.073359589	-1.169373468	0.155406001	-2.634031198	10.54098126	9.125613817	8.631919287	8.14748175	8.284723	8.295734109	8.695572791
ENSG00000168778	TCTN2	-1.321132538	0.544796958	-1.315088558	0.540695624	-2.636221096	2	6.04685525	9.132850258	6.372775472	6.49425375	6.320128742	6.55171954
ENSG00000103549	RNF40	-0.68179176	0.290429859	-1.954624818	0.000553999	-2.636416579	10.36714848	10.73405387	11.7010103	10.33623673	10.3980237	9.077661821	9.12358288
ENSG00000166025	AMOTL1	-1.826509718	0.00023389	-0.811316219	0.131351033	-2.637825937	11.29385609	12.35391197	11.48210469	9.911750163	10.0187431	10.90816893	11.04559838
ENSG00000107902	LHPP	-1.974592171	0.011022574	-0.66563123	0.437688449	-2.640223401	7.53297911	7.874700259	9.344432377	6.456560224	6.63128385	7.873733947	7.760489878
ENSG00000176896	TCEANC	-1.204541873	0.142431446	-1.436028821	0.066886602	-2.640570694	5.095959458	6.311476754	5.930865648	4.804776789	4.79082474	4.888321757	4.241259475
ENSG00000188582	PAQR9	-0.979601134	0.729390378	-1.661422481	0.531906196	-2.641023615	2	2	4.523675391	2.784612485	2.86123014	2.803598391	2.277179783
ENSG00000167775	CD320	-1.728474749	5.26E-05	-0.913289515	0.044041567	-2.641764265	9.839306291	10.13758676	9.31604076	8.080836374	8.10384772	8.670625574	9.095255687
ENSG00000167792	NDUFV1	-1.883932015	0.106436871	-0.758473301	0.544775711	-2.642405317	11.49141174	9.624396729	8.076950877	8.481674661	8.49034333	9.418393009	9.766168491
ENSG00000165028	NIPSNAP3B	-0.040677459	0.984108348	-2.601824053	0.129124437	-2.642501511	6.89194353	5.303523747	2	5.662809983	5.77157844	4.065262523	2.950113106
ENSG00000162694	EXTL2	-1.120129499	0.063823695	-1.522784402	0.008354549	-2.642913901	8.737455672	9.532315021	10.14394518	8.491639362	8.44711562	8.091532339	8.052432906
ENSG00000160323	ADAMTS13	-1.620127722	0.409685056	-1.022997444	0.610287918	-2.643125166	8.19931886	5.838937808	2	5.406080789	5.3193943	6.14330814	5.636738041
ENSG00000100442	FKBP3	-0.365381286	0.708408469	-2.278207921	0.00476195	-2.643589206	10.31337612	8.169643011	9.287079172	9.230466133	9.05063789	6.911998488	7.537835213
ENSG00000143947	RPS27A	-1.591494026	0.022379195	-1.052614599	0.144035339	-2.644108625	12.91500773	11.22868348	11.74958762	10.55198546	10.5550472	11.06935627	11.11271466
ENSG00000119705	SLIRP	-0.520564664	0.417361165	-2.123563626	9.65E-05	-2.64412829	8.75772933	7.467835385	8.143093325	7.641902724	7.7515168	6.121005698	6.179140936
ENSG00000144031	ANKRD53	-1.384688194	0.606861222	-1.259577141	0.635737899	-2.644265335	2	6.535001748	2	4.17310023	3.69849104	3.663836244	4.353147976
ENSG00000135916	ITM2C	-1.833493593	0.079559698	-0.813208684	0.4666278	-2.646702278	13.49066495	11.08580931	11.03218278	10.52353286	10.5361304	11.51552829	11.58004535
ENSG00000131370	SH3BP5	-0.35044294	0.673891173	-2.296590822	0.000751772	-2.647033762	8.981092835	8.029695761	7.225742779	7.947219213	7.86955761	5.912830251	6.13807518
ENSG00000123933	MXD4	-2.105516965	2.39E-07	-0.542337742	0.243516721	-2.647854707	11.99474663	11.1687783	11.37608156	9.457570641	9.45737703	10.87804317	11.14048782
ENSG00000169021	UQCRFS1	-1.353037861	1.46E-05	-1.29507286	3.12E-05	-2.648110721	10.55263183	10.63288029	10.23015729	9.100844036	9.17201613	9.005308106	9.361816632
ENSG00000088812	ATRN	-1.751682897	7.29E-06	-0.897632548	0.031375886	-2.649315446	12.07772327	11.23134822	11.67049834	9.920382853	9.98368402	10.80974856	10.79787914
ENSG00000136143	SUCLA2	-1.435463591	3.53E-05	-1.213918803	0.000505593	-2.649382394	9.579430841	9.806969599	9.165043284	8.174538791	8.06917614	8.288873067	8.392188801
ENSG00000164418	GRIK2	1.094154401	0.686593442	-3.745897463	0.117040598	-2.651743062	2	7.145017454	2	6.622948477	6.74548212	2.62493832	3.128433649
ENSG00000102580	DNAJC3	-1.164267877	0.048513855	-1.487678899	0.008884136	-2.651946776	11.13631304	10.87704672	12.02204252	10.2793709	10.2602493	9.628876603	10.20855713
ENSG00000101997	CCDC22	-1.146499301	0.573001609	-1.507415987	0.440486964	-2.653915288	9.27634997	8.522788974	2	7.226553841	7.26148706	6.798888929	6.983208048
ENSG00000122034	GTF3A	-1.401091958	0.007345799	-1.253461593	0.017178874	-2.654553552	10.5468183	9.566483142	9.528916236	8.573670512	8.56534297	8.69064008	8.740277714
ENSG00000072858	SIDT1	-2.555456211	0.290311321	-0.099517732	0.968964721	-2.654973944	2	3.076665209	3.369317972	2.141737246	2.2680111	3.035909414	2.70970617
ENSG00000185101	ANO9	-3.296402287	0.34076194	0.641386778	0.857444981	-2.65501551	2	2	4.058998049	2.270786764	2	3.27687201	3.45257121
ENSG00000100908	EMC9	-1.815677544	0.140439423	-0.840523447	0.518736842	-2.656200991	8.586954094	7.393647515	4.874587708	5.797438367	5.91514978	6.591495479	6.951954485
ENSG00000128050	PAICS	-2.018958335	5.10E-06	-0.637409796	0.197561623	-2.656368131	11.52136524	10.74528399	10.67226762	9.04780133	8.99630959	10.23447665	10.54305007
ENSG00000171863	RPS7	-1.127041599	0.141109656	-1.531983301	0.035956933	-2.6590249	12.09972935	10.50217373	10.5916593	10.16260233	10.1174913	9.788404752	9.683551156
ENSG00000131779	PEX11B	-1.736000205	1.09E-05	-0.923501813	0.026953886	-2.659502018	9.810482605	9.170736405	9.06622548	7.662632018	7.67863747	8.496947559	8.444354606
ENSG00000162188	GNG3	-1.180843895	0.691648862	-1.478851842	NA	-2.659695737	2	3.686303414	2	2.600425781	2.2680111	2.457042231	2.277179783
ENSG00000254999	BRK1	-1.246820537	0.00326405	-1.413981193	0.000672535	-2.66080173	11.88382844	11.29895829	11.95227171	10.46993609	10.5189709	10.02242176	10.58009362
ENSG00000171791	BCL2	-3.151414997	0.211336072	0.489153135	0.863199064	-2.662261862	2	6.311476754	2	3.09461492	2.38539787	5.598599872	4.975095763
ENSG00000162086	ZNF75A	-0.443137041	0.452334443	-2.219924126	6.05E-06	-2.663061168	7.904282243	8.004992147	8.677268317	7.839502934	7.75424764	6.353989995	5.745545708
ENSG00000178028	DMAP1	-1.360529697	0.089666875	-1.302702256	0.099409613	-2.663231953	9.761125267	7.928237109	8.486635645	7.626911583	7.55566492	7.4096614	7.853274783
ENSG00000109771	LRP2BP	-2.665273284	0.028200875	0.001134234	0.999378208	-2.664139049	5.095959458	3.076665209	5.39244166	3.09461492	2.77779907	4.785723309	4.77533149
ENSG00000169885	CALML6	-1.825849609	0.486921279	-0.838741498	0.757654618	-2.664591106	2	2	5.39244166	2.868578001	2.94009902	3.782262616	3.012034821
ENSG00000166546	BEAN1	-2.495415615	0.206479362	-0.169455254	0.940405661	-2.664870869	5.70932728	3.076665209	2	2.947924294	2.68924543	4.628601098	3.824516949
ENSG00000091640	SPAG7	-1.368037655	0.041735857	-1.300409776	0.051271517	-2.668447431	10.80726569	9.799806309	9.242516611	8.703665429	8.77214691	8.66870487	8.930161469
ENSG00000177646	ACAD9	-1.023946107	0.002766788	-1.645473103	4.02E-07	-2.669419211	9.497412313	9.34816733	9.227351203	8.287525788	8.40429221	7.492346363	7.943433856
ENSG00000121104	FAM117A	-1.499318005	0.001565551	-1.170555059	0.014545829	-2.669873064	7.624139768	7.232742069	7.858115942	6.069927018	6.20590375	6.189586148	6.674075308
ENSG00000152464	RPP38	-1.45418264	0.016061522	-1.215698565	0.04533349	-2.669881206	7.667647223	7.35507212	8.608698767	6.604700657	6.507263	6.879153024	6.685996416
ENSG00000100583	SAMD15	-0.151461032	0.957050083	-2.519037623	0.296825224	-2.670498655	2	5.838937808	2	4.299492648	4.40247105	2.492221014	3.128433649
ENSG00000225697	SLC26A6	-1.123590379	0.02438057	-1.546987375	0.001186636	-2.670577755	10.04301211	9.159587375	9.116480286	8.402902269	8.37797443	7.952415862	7.993217039
ENSG00000118402	ELOVL4	-0.607791358	0.70562308	-2.062866833	0.144465782	-2.670658191	9.2903348	7.051611315	4.874587708	7.501020868	7.37584259	6.353989995	5.641643428
ENSG00000187514	PTMA	-1.117788414	0.000160157	-1.553492864	6.15E-08	-2.671281278	12.84803656	12.60879193	12.81745404	11.66367858	11.6257578	10.96258464	11.42080713
ENSG00000157322	CLEC18A	1.60707571	0.620052795	-4.278823407	0.16716103	-2.671747697	2	2	4.058998049	3.709785322	4.2865223	2.139668783	2
ENSG00000105404	RABAC1	-0.916965935	6.72E-05	-1.755355504	1.12E-15	-2.672321439	11.18203661	10.94030351	11.08361639	10.10372352	10.2069242	9.250699274	9.389783813
ENSG00000113851	CRBN	-1.101261666	0.16737365	-1.57191523	0.037364726	-2.673176895	9.949165863	9.328390744	8.007629717	8.203120604	8.2130862	7.958717528	7.493753702
ENSG00000171766	GATM	-2.133495613	0.271773169	-0.539741233	0.796690808	-2.673236845	3.327711234	5.303523747	2	3.09461492	2.59489879	4.122514202	3.383972333
ENSG00000163348	PYGO2	-2.057662074	0.030750279	-0.617013918	0.560994904	-2.674675992	8.777722034	8.655694736	10.89459479	7.842101511	7.75424764	9.304879394	9.136237781
ENSG00000160633	SAFB	-0.823829629	0.419016636	-1.851373685	0.04446759	-2.675203314	11.35168561	9.148351514	9.359998894	9.460955886	9.54672161	8.619836896	8.336553984
ENSG00000152763	WDR78	-1.104732095	0.621262845	-1.572104225	0.460375683	-2.676836321	2	3.686303414	7.163363831	4.846787657	4.63360314	4.771475226	3.771796761
ENSG00000173818	ENDOV	-1.887932946	0.035043531	-0.789542026	0.412508059	-2.677474971	7.384433967	6.728495673	8.845626171	5.987823101	6.20590375	7.417689057	6.830475749
ENSG00000157557	ETS2	-0.778494503	0.034731167	-1.900266644	1.39E-08	-2.678761147	10.58135316	10.19877383	10.61515347	9.630628506	9.76553205	8.344674564	8.793039018
ENSG00000276600	RAB7B	-6.383706317	0.02747405	3.700763167	0.125991152	-2.68294315	2	4.442710129	2	2	2	6.535683256	6.308152703
ENSG00000197147	LRRC8B	-2.976643232	0.067576467	0.292435438	0.87857982	-2.684207794	2	4.93638933	6.322058544	3.289987342	2.77779907	5.822425633	5.115701921
ENSG00000214367	HAUS3	-1.186130103	0.539576503	-1.499491715	0.422327856	-2.685621818	9.371511179	6.04685525	3.369317972	6.841288255	6.72891807	6.774090992	6.124122661
ENSG00000059691	GATB	-1.208054723	0.450332207	-1.478455479	0.335253459	-2.686510202	9.544849385	6.228547981	5.39244166	6.975156635	6.99676009	6.613628927	6.828326017
ENSG00000163719	MTMR14	-1.143716011	0.061530373	-1.543248341	0.008306252	-2.686964353	9.657036304	8.276714465	9.272377397	8.069792811	8.01099479	7.538302335	7.750306941
ENSG00000109956	B3GAT1	-0.679672253	NA	-2.007887435	NA	-2.687559687	2	2	3.369317972	2.695456494	2	2.267001569	2.059860178
ENSG00000104953	TLE6	-1.015917121	0.506649307	-1.672557516	0.235344728	-2.688474637	3.327711234	4.11352228	4.058998049	3.162721474	3.28037825	3.1504466	2.551896972
ENSG00000112305	SMAP1	-0.405347018	0.531143362	-2.283931628	1.91E-05	-2.689278646	7.667647223	8.916342327	8.175061096	7.966401501	7.9408106	6.081127077	6.189227296
ENSG00000165209	STRBP	-2.513067601	0.113748903	-0.179643173	0.92531104	-2.692710774	7.031698319	7.051611315	2	4.205751501	4.2865223	6.327451929	6.298867018
ENSG00000136243	NUPL2	-0.644162096	0.466937891	-2.049959787	0.007873748	-2.694121883	8.716893051	7.35507212	6.882775709	7.25803034	7.21862736	6.040114727	5.73181021
ENSG00000255221	CARD17	2.49669361	0.671489931	-5.191096372	NA	-2.694402762	2	2	2	3.289987342	3.28037825	2	2
ENSG00000260458	KCNJ18	2.494380499	0.67181002	-5.191096382	NA	-2.696715883	2	2	2	3.023133209	3.50452879	2	2
ENSG00000113946	CLDN16	-0.330168266	0.8694764	-2.367401932	0.16091081	-2.697570199	2	3.686303414	4.058998049	3.227757282	3.33976443	2.3070652	2.551896972
ENSG00000246705	H2AFJ	-2.270478075	0.001819985	-0.427980748	0.61684613	-2.698458823	11.33491623	9.726146444	9.849281147	8.227176542	8.2657098	9.773736569	10.32462856
ENSG00000205629	LCMT1	-1.270145134	6.34E-05	-1.429840715	4.83E-06	-2.699985849	11.57090097	11.72481431	12.10735427	10.60534987	10.4938644	10.48300598	10.29360417
ENSG00000137074	APTX	-1.316596536	0.001277928	-1.38396252	0.000591536	-2.700559056	9.473095744	8.702538795	9.399592459	7.915493556	7.93118934	7.86621124	7.847978946
ENSG00000075142	SRI	-1.061104853	0.118143225	-1.640685473	0.010224534	-2.701790326	10.36714848	9.763446433	8.742726175	8.676258519	8.75452469	8.194575779	8.089704419
ENSG00000197124	ZNF682	-1.138388056	0.587855308	-1.564424519	0.435048513	-2.702812575	2	4.442710129	7.502634626	5.144975053	4.97065356	4.894909044	4.456980017
ENSG00000115946	PNO1	-1.00033789	0.144467139	-1.703465995	0.007523544	-2.703803885	9.613202746	8.276714465	9.809100877	8.29513208	8.45720682	7.633939843	7.736616914
ENSG00000160207	HSF2BP	-1.464562995	0.580207026	-1.240486161	0.637526628	-2.705049156	2	2	5.930865648	3.406930997	3.45167086	3.483289517	3.660199087
ENSG00000266472	MRPS21	-1.65245985	0.000151748	-1.05408672	0.020815791	-2.70654657	10.80239439	9.876711741	10.42220168	8.713632895	8.8250571	9.229031012	9.488040822
ENSG00000184281	TSSC4	-1.877719365	0.000108969	-0.829011922	0.113843563	-2.706731287	10.27168932	9.298208073	9.385998894	7.920420067	7.79989812	8.844037986	8.948586914
ENSG00000103707	MTFMT	-0.913380507	0.089385355	-1.793702475	0.000260301	-2.707082982	8.39321512	7.669933291	8.763901704	7.370304568	7.5014335	6.569017168	6.600402875
ENSG00000128059	PPAT	-1.344522704	0.210236498	-1.36320072	0.194132356	-2.707723424	5.866944937	8.820493869	8.175061096	6.799114572	6.70088158	6.928144991	6.506723027
ENSG00000129244	ATP1B2	-1.917192296	0.201282385	-0.790878288	0.615603284	-2.708070584	3.327711234	5.595927442	4.874587708	3.95996233	2.59489879	4.281782022	4.188602707
ENSG00000197114	ZGPAT	-0.167499847	0.938052303	-2.540675104	0.155898571	-2.708174951	2	7.874700259	8.65477198	7.653784481	7.4949172	4.996409624	5.59182244
ENSG00000140006	WDR89	-1.468634891	0.462772837	-1.239945807	0.531674005	-2.708580698	2	7.145017454	8.825624954	6.321555392	6.11534392	6.645228724	6.199243628
ENSG00000130733	YIPF2	-1.364333532	0.000422235	-1.347033319	0.000461516	-2.711366851	10.18451821	9.821190352	10.55568472	8.85414072	8.86350401	9.009866973	8.728222198
ENSG00000171885	AQP4	-1.9755469	0.503635732	-0.736238562	0.811558361	-2.711785461	2	4.710564154	3.369317972	3.162721474	2	3.517841605	3.042026731
ENSG00000082397	EPB41L3	-1.188354604	0.185185634	-1.523546298	0.076272108	-2.711900902	8.540903468	10.26789142	8.865353883	8.195011988	8.31648126	8.396077967	7.226264598
ENSG00000143222	UFC1	-1.15750876	0.00359668	-1.554421305	4.97E-05	-2.711930065	10.43795805	10.10889278	10.12770132	9.070139391	9.09116272	8.313052751	8.984263871
ENSG00000099864	PALM	-6.191029247	2.32E-11	3.478268537	7.32E-05	-2.712760709	8.444143041	8.255926468	6.958202216	3.349643765	2.2680111	11.50493671	11.45325516
ENSG00000101353	MROH8	-1.928752576	0.253424754	-0.7842146	0.660736901	-2.712967176	4.467311984	6.140560021	2	3.663521627	3.39680241	4.166733998	4.56420962
ENSG00000008513	ST3GAL1	-0.463592125	0.585792769	-2.249594884	0.001633879	-2.713187008	9.699577239	10.64091981	11.63645966	10.37891658	10.4333835	8.69913345	8.55898913
ENSG00000113504	SLC12A7	-0.648809293	0.417929197	-2.065040465	0.003051234	-2.713849759	5.330594134	6.951736442	6.718950348	5.85002558	5.92487824	4.636484163	4.614976609
ENSG00000105696	TMEM59L	-0.992596598	0.280090671	-1.721736235	0.043186443	-2.714332834	7.435667355	6.535001748	8.460932916	6.622948477	6.79936111	6.394066622	5.513708555
ENSG00000077312	SNRPA	-1.510383335	0.17350281	-1.203969365	0.2795154	-2.7143527	10.00110988	9.04308532	6.958202216	7.608714138	7.66128317	7.347386207	8.353097069
ENSG00000076053	RBM7	-1.028807136	0.216615116	-1.685952615	0.030507451	-2.714759751	9.966689162	9.067150531	7.896971941	8.246921218	8.13141746	7.660277586	7.418582165
ENSG00000178665	ZNF713	-1.310774944	0.229253543	-1.404459345	0.180731158	-2.715234289	5.532353208	4.11352228	5.772716771	4.358765249	3.94787299	4.416072028	3.67940853

ENSG00000127952	STYXL1	-1.276951996	0.000482403	-1.441764877	5.35E-05	-2.718716873	8.418903792	8.395519306	8.865353883	7.277360022	7.35440525	7.291067412	7.006211772
ENSG00000183780	SLC35F3	3.655394797	0.061530373	-6.375815045	0.009454427	-2.720420248	2	4.442710129	2	6.456560224	6.37156942	2	2.059860178
ENSG00000160948	VPS28	-1.532671859	2.60E-08	-1.187853066	1.93E-05	-2.720524924	9.886101849	9.863030423	10.19160738	8.420396336	8.50663048	8.697250351	8.906411656
ENSG00000075856	SART3	-1.099774519	0.07662408	-1.621826317	0.00575638	-2.721600835	10.19941743	9.246456519	10.69449487	9.060130342	9.07377798	8.596789458	8.500786839
ENSG00000182791	CCDC87	-0.845544851	0.593517792	-1.880467942	0.19011257	-2.726012793	2	4.710564154	4.523675391	3.663521627	3.45167086	2.885263063	3.042026731
ENSG00000274750	HIST1H3E	-1.235446277	0.478344278	-1.490698374	0.3708938	-2.726144651	6.138360429	2	5.930865648	4.443344104	4.37434574	4.04171018	4.353147976
ENSG00000181666	HKR1	-1.626451258	0.159712355	-1.101278066	0.350712355	-2.727729323	6.89194353	9.13702746	10.37481416	7.756533788	7.79989812	8.344072943	8.245238611
ENSG00000100347	SAMM50	-1.264982956	0.048902337	-1.464195165	0.019274967	-2.729178121	9.940323746	9.192779271	8.434763955	8.029321023	8.09955907	7.737439787	7.989373961
ENSG00000162631	NTNG1	2.014492482	0.519228435	-4.744437094	0.151238336	-2.729944612	2	3.076665209	2	3.754611435	3.39680241	2.048074405	2
ENSG00000105202	FBL	-1.477345348	7.66E-05	-1.257094294	0.000823875	-2.734439641	9.730679447	9.170736405	9.06622548	7.872924734	7.90685167	8.042684843	8.168292282
ENSG00000010379	SLC6A13	-1.402290725	0.649972951	-1.33398239	NA	-2.736273115	2	3.686303414	2	2.141737246	2.59489879	2.384001718	2.421067459
ENSG00000182749	PAQR7	-1.514977072	0.140239643	-1.223385197	0.235763044	-2.738362269	9.886101849	7.572420273	10.51254821	8.217201935	8.28849576	8.521632434	8.560932499
ENSG00000116478	HDAC1	-1.037816453	0.075639549	-1.700839492	0.001834875	-2.738655944	10.56418906	9.377333006	10.30429804	9.103012382	9.1566168	8.199240359	8.703218237
ENSG00000122970	IFT81	-0.709468198	0.648413066	-2.029614387	0.143651762	-2.739082585	4.467311984	7.701025996	9.165043284	7.292639438	7.44169935	6.013120342	6.182510897
ENSG00000149932	TMEM219	-1.259822914	0.007788736	-1.48259412	0.001323898	-2.742417034	11.09707093	10.16572116	11.10866152	9.570001722	9.62041512	9.388071115	9.359585433
ENSG00000178053	MLF1	-1.619840107	0.164250542	-1.124035266	0.341075041	-2.743875373	4.467311984	7.73146271	6.80318718	5.334065616	5.34860058	5.876679847	5.708623051
ENSG00000161381	PLXDC1	-3.326036282	0.003441205	0.581518973	0.633084435	-2.744517309	4.00722531	5.303523747	4.874587708	2.695456494	2.68924543	5.843010798	4.614976609
ENSG00000168958	MFF	-1.393334224	0.04626209	-1.351597989	0.051017141	-2.744932213	10.905938	9.148351514	10.31150679	8.909699622	8.8960155	8.99844268	8.887952157
ENSG00000172421	EFCAB3	2.445126554	0.678957281	-5.191096587	NA	-2.745970033	2	2	2	3.349643765	3.15372745	2	2
ENSG00000114383	TUSC2	-1.525617039	0.000408074	-1.220504723	0.00539006	-2.746121761	9.371511179	9.102512015	9.981704053	7.954442493	8.08010078	8.331380596	8.307525005
ENSG00000130414	NDUFA10	-1.596793132	0.001128625	-1.149438745	0.023237554	-2.746231876	11.11289609	9.988044813	10.55568472	9.013631184	9.05285777	9.421533019	9.531964377
ENSG00000140478	GOLGA6D	2.444527733	0.679016801	-5.191096589	NA	-2.746568856	2	2	2	3.289987342	3.2184422	2	2
ENSG00000100027	YPEL1	-1.544692282	0.078250731	-1.201969897	0.165909543	-2.74666218	5.70932728	4.710564154	6.073378139	4.299492648	4.1930041	4.785723309	4.201948172
ENSG00000143376	SNX27	-1.705306929	7.72E-05	-1.04150704	0.021475934	-2.746813969	10.41898845	10.03760521	10.95142175	8.80304041	8.83669913	9.593120016	9.355112655
ENSG00000244607	CCDC13	2.44332935	0.67918823	-5.191096594	NA	-2.747767244	2	2	2	3.162721474	3.33976443	2	2
ENSG00000147123	NDUFB11	-1.31226377	0.004606222	-1.436315885	0.001618861	-2.748579655	10.72727936	9.856140801	10.6093157	9.140440229	9.1347778	8.790727376	9.208268782
ENSG00000141349	G6PC3	-1.281104061	0.000257308	-1.468030187	2.01E-05	-2.749134248	10.95943304	10.94355721	10.85110517	9.617795654	9.66608324	9.095643988	9.744671657
ENSG00000277789		2.44153019	0.679377579	-5.191096602	NA	-2.749566411	2	2	2	2.947924294	3.50452879	2	2
ENSG00000198746	GPATCH3	-0.980049618	0.029202849	-1.770795086	2.20E-05	-2.750844703	8.418903792	7.928237109	8.763901704	7.527093191	7.34356593	6.69514648	6.64749689
ENSG00000110721	CHKA	-0.682091869	0.055943651	-2.072538672	7.22E-11	-2.754630541	7.940205782	8.077869079	8.143093325	7.398825229	7.37584259	6.173421703	5.917188835
ENSG00000150510	FAM124A	-2.589072218	0.023121834	-0.16562593	0.904524117	-2.754698148	4.815609471	6.602424674	4.058998049	3.515101962	3.50452879	5.4588802	5.436777225
ENSG00000156873	PHKG2	-1.36942671	0.33304377	-1.386463661	0.317269735	-2.755890371	5.532353208	8.395519306	9.981704053	7.471115032	7.5462416	7.268416942	7.68646583
ENSG00000169223	LMAN2	-1.072799535	0.002515173	-1.683256345	6.68E-07	-2.75605588	11.99260982	11.50459547	12.10320639	10.79667089	10.8375393	10.05880891	10.34413215
ENSG00000158850	B4GALT3	-1.170095797	0.00375557	-1.586909881	4.46E-05	-2.757005678	10.27872131	10.00683004	9.491321578	8.748679155	8.84824797	8.492068113	8.273166147
ENSG00000109083	IFT20	-1.267985809	0.039760769	-1.491716215	0.012813892	-2.759702024	9.304185368	8.414402626	9.859153765	7.97352961	8.12087597	7.792277365	7.864857644
ENSG00000134049	IER3IP1	-0.301466294	0.686253129	-2.461428383	3.77E-05	-2.762894677	9.940323746	10.06773758	8.65477198	9.411949708	9.34954609	7.458282995	7.008112295
ENSG00000178896	EXOSC4	-1.351040043	0.028339952	-1.412956388	0.019994373	-2.763996432	8.963744667	8.433041974	7.502634626	7.148924055	7.03768431	7.037865927	7.030725399
ENSG00000165819	METTL3	-1.630582495	0.003661907	-1.134993189	0.049999298	-2.765575685	9.931427102	8.623598428	9.301632639	7.797276258	7.73502229	8.363201462	8.13650041
ENSG00000120509	PDZD11	-1.622688606	0.061601819	-1.143049473	0.196946345	-2.76573808	10.3868074	8.903033433	8.206335842	7.883054517	7.82349365	8.299462788	8.352349212
ENSG00000137054	POLR1E	-2.15193064	6.41E-05	-0.61494156	0.305318653	-2.7668722	8.493334308	7.874700259	7.285536013	5.920568914	5.86549842	7.219398545	7.497817236
ENSG00000087301	TXNDC16	-2.485832536	0.226635372	-0.281790676	0.906201712	-2.767623212	2	8.57407404	5.156626401	4.907595604	4.74762503	6.870823472	6.860240429
ENSG00000103067	ESRP2	-2.178340454	0.18239431	-0.589632131	0.725309613	-2.767972585	4.00722531	3.076665209	3.369317972	2.600425781	2.38539787	3.082820997	3.236138336
ENSG00000185909	KLHDC8B	-2.386750533	2.68E-05	-0.38244151	0.571407617	-2.769192043	9.922475255	9.203675681	8.511888463	6.946463121	7.00595521	9.04915428	8.834865421
ENSG00000144455	SUMF1	-2.064266434	4.41E-07	-0.705835076	0.117409111	-2.77010151	9.922475255	9.461450882	9.878698603	7.656739692	7.7866208	8.687797808	9.363673333
ENSG00000173914	RBM4B	-0.955215117	0.609360413	-1.815702999	0.293236581	-2.770918116	10.04301211	8.762721501	3.369317972	7.994705029	8.04707549	7.039353222	7.305675343
ENSG00000205544	TMEM256	-1.496500894	0.114732583	-1.274568724	0.177686131	-2.771069617	8.963744667	6.899080544	7.098165388	6.379946408	6.64311914	6.635427934	6.81969491
ENSG00000213015	ZNF580	-1.922105601	4.02E-08	-0.850018367	0.023768024	-2.772123968	10.29268328	9.962611189	9.735974718	8.113468729	8.10384772	8.955762164	9.35623215
ENSG00000134375	TIMM17A	-1.252383117	0.019565207	-1.524953306	0.003407034	-2.777336423	10.86447339	9.7032946	10.73794767	9.312394472	9.22907886	8.99461439	9.00677572
ENSG00000204977	TRIM13	-1.195801459	0.129734214	-1.583231116	0.03600172	-2.779032575	10.787681	9.090820894	9.372276026	8.745906571	8.78288532	8.58766971	8.139971339
ENSG00000185515	BRCC3	-1.146149607	0.028178148	-1.63393744	0.001006735	-2.780087047	8.228665467	9.159587375	9.242516611	7.895617537	7.72670378	7.178138321	7.478755384
ENSG00000049768	FOXP3	-1.303391381	0.647700657	-1.479173632	0.590766167	-2.782565013	2	2	4.523675391	2.695456494	2.68924543	2.267001569	2.918126211
ENSG00000166483	WEE1	-2.005488868	1.07E-11	-0.778345209	0.01361034	-2.783834077	9.448362284	9.506147172	9.385998894	7.440576128	7.4949172	8.864319213	8.459712934
ENSG00000125656	CLPP	-1.681753265	0.000354648	-1.10316997	0.024438946	-2.784923235	10.30651143	9.357954881	10.13584611	8.392303091	8.23671175	8.692059118	9.062708281
ENSG00000165124	SVEP1	-1.626227311	0.006210955	-1.159050227	0.057959502	-2.785277538	9.081016759	9.648522413	10.49998282	8.254744042	8.240612	8.708979596	8.710239791
ENSG00000149806	FAU	-1.648659952	0.009564066	-1.138680571	0.083207596	-2.787340524	11.98832668	10.81444733	10.54960077	9.585592789	9.64784224	10.11349084	10.13610645
ENSG00000139579	NABP2	-2.068636208	0.011100643	-0.720515516	0.423520139	-2.789151723	8.981092835	8.101365653	10.2528023	7.307758725	7.36516374	8.587161363	8.735123394
ENSG00000182149	IST1	-1.325006323	0.000658244	-1.465679894	0.000129752	-2.790686217	11.59081378	11.08286144	11.89103474	10.2136641	10.2573647	10.12614381	10.06416949
ENSG00000163795	ZNF513	-1.386915597	0.528732117	-1.406266698	0.513903548	-2.793182296	10.24321353	8.101365653	2	7.552702688	7.62295033	7.501007892	7.634508409
ENSG00000106089	STX1A	-1.47088652	0.423427099	-1.322374525	0.467016155	-2.793261045	9.371511179	6.535001748	3.369317972	6.535745081	6.5890786	6.800644007	6.602920209
ENSG00000048544	MRPS10	-1.493452259	0.013191227	-1.302169748	0.032017989	-2.795622007	9.435834703	9.159587375	10.46161637	8.429064458	8.19309833	8.444590638	8.562873254
ENSG00000255408	PCDHA3	-0.578115245	0.807827682	-2.217525796	0.288238766	-2.795641041	3.327711234	4.442710129	2	3.709785322	2.59489879	2.746468522	2.277179783
ENSG00000109180	OCIAD1	-0.67248825	0.157050937	-2.123699882	3.31E-07	-2.796188132	11.59081378	10.75272247	10.85110517	10.47496775	10.4123582	9.045828404	8.952540041
ENSG00000142621	FHAD1	1.545639723	0.441837321	-4.342347618	0.017198932	-2.796707895	3.327711234	2	6.322058544	6.379946408	6.35006802	2.492221014	2.326754077
ENSG00000173660	UQCRH	-1.173193291	0.016661881	-1.62382569	0.000529876	-2.797018982	10.40620204	9.405920729	9.658943166	8.722121967	8.72001862	8.254710055	8.295903454
ENSG00000111971		2.393509416	NA	-5.191096802	NA	-2.797587386	2	2	2	3.566290607	2.77779907	2	2
ENSG00000128039	SRD5A3	-0.37854259	0.529979238	-2.41928596	8.50E-07	-2.797828551	8.892187472	8.834582582	9.778213818	8.85414072	8.86097251	7.045287119	6.635252003
ENSG00000124102	PI3	2.391647173	NA	-5.191096809	NA	-2.799449637	2	2	2	3.406930997	3.01487888	2	2
ENSG00000173113	TRMT112	-1.11611536	0.197898228	-1.68351778	0.039331005	-2.799633139	10.79260216	8.806266215	9.287079172	8.772033429	8.78689171	8.066221408	8.356084628
ENSG00000186081	KRT5	2.391025901	NA	-5.191096812	NA	-2.800070911	2	2	2	3.349643765	3.08597292	2	2
ENSG00000167195	GOLGA6C	2.390404361	NA	-5.191096814	NA	-2.800692453	2	2	2	3.289987342	3.15372745	2	2
ENSG00000097046	CDC7	-1.287739307	0.395924587	-1.51398348	0.298583938	-2.801722786	4.00722531	7.232742069	4.523675391	4.907595604	4.76938658	4.940196634	4.279527956
ENSG00000135637	CCDC142	-1.258851613	0.030150603	-1.54328255	0.005900513	-2.802134162	8.418903792	8.297207175	7.225742779	6.782976084	6.90635305	6.713894915	6.415155055
ENSG00000175756	AURKAIP1	-1.647923258	0.011477015	-1.154481014	0.085841182	-2.802404272	11.27639669	10.03760521	9.829330889	8.873322599	8.90955283	9.331225056	9.430034933
ENSG00000008226	DLEC1	-2.682186671	0.470185896	-0.124467237	0.974678148	-2.806653909	4.00722531	2	2	2	2.38539787	2.937246078	2.918126211
ENSG00000053371	AKR7A2	-1.915836314	0.009469798	-0.890877431	0.261047618	-2.806713746	10.51739401	8.777382925	9.132850258	7.717440541	7.84926609	8.705236603	8.882264318
ENSG00000132517	SLC52A1	-4.295368089	0.204890893	1.487155305	0.630693854	-2.808212784	2	2	4.058998049	2	2.14022021	4.378961816	3.287118176
ENSG00000128604	IRF5	-2.70196544	0.307069196	-0.10628783	0.9716719	-2.80825327	2	2	5.930865648	2.868578001	2.68924543	4.984105765	3.474731315
ENSG00000185236	RAB11B	-1.788554232	0.000255505	-1.020626731	0.048365141	-2.809180963	11.79588449	10.68823825	11.38976845	9.5407064	9.61140668	10.17195017	10.49286637
ENSG00000172671	ZFAND4	-1.739592812	0.000482793	-1.06971743	0.036844987	-2.809310243	7.096806373	7.002537973	7.600047325	5.709096143	5.47317115	6.428526856	5.976631893
ENSG00000140691	ARMC5	-1.74852013	0.015778095	-1.061783584	0.158766578	-2.810303714	8.564112515	7.538394143	9.372276026	6.922108067	6.98750599	7.517109417	7.72395257
ENSG00000111850	SMIM8	-1.73633672	0.042566554	-1.0743204	0.221353293	-2.81065712	5.532353208	7.467835385	6.322058544	4.927308402	5.19627118	5.725974948	5.601925227
ENSG00000076554	TPD52	-5.696119424	0.097440073	2.884803238	0.348299029	-2.811316186	4.00722531	2	2	2	2	5.125248504	4.951586397
ENSG00000126215	XRCC3	-2.387137916	0.023103998	-0.424858383	0.726496359	-2.811996298	5.095959458	8.053983481	6.80318718	5.003571641	4.76938658	6.577231679	6.815359915
ENSG00000132781	MUTYH	-1.392388392	0.486454906	-1.42093167	0.467550744	-2.813320062	8.042902253	5.457120953	2	5.433909581	5.34860058	5.399896186	5.331426166
	ATP5L2	4.1986317	0.01486761	-7.012000278	0.002624999	-2.813368578	3.327711234	3.076665209	2	5.978405189	6.26836889	2	2
ENSG00000188493	C19orf54	-1.872842394	0.004587427	-0.941691474	0.179051068	-2.814533868	8.367060731	8.806266215	7.163363831	6.456560224	6.43421531	7.447120996	7.234405279
ENSG00000130713	EXOSC2	-1.545021592	0.001154449	-1.269739242	0.008252225	-2.814760834	8.586954094	7.928237109	8.941672936	7.044488801	6.99676009	7.232460462	7.342293759
ENSG00000116649	SRM	-1.700989737	4.24E-05	-1.115484574	0.009830805	-2.81647431	11.09707093	10.92392403	10.32581712	9.061245954	9.18624204	9.476915012	9.905748
ENSG00000091947	TMEM101	-1.683820391	0.001858408	-1.133031403	0.042948413	-2.816851795	9.48530526	8.234834549	9.180874355	7.398825229	7.38644297	7.908330388	7.95628774
ENSG00000099899	TRMT2A	-1.615725765	0.238681925	-1.202504385	0.385194227	-2.818230151	10.72212986	8.57407404	6.534109959	7.939959586	7.84670947	8.394916616	8.201061123
ENSG00000172361	CFAP53	-1.266585231	0.479204668	-1.55370456	0.361514946	-2.82028979	2	3.686303414	5.39244166	3.515101962	3.2184422	3.27687201	3.128433649
ENSG00000119969	HELLS	-1.368220969	0.315594945	-1.454353246	0.271500407	-2.822574215	3.327711234	5.457120953	6.80318718	4.670864177	4.45712836	4.713032626	4.241259475
ENSG00000084652	TXLNA	-1.708759619	0.00731379	-1.114026819	0.092695101	-2.822786438	11.22998797	11.46891107	12.66680507	10.19396441	10.2616895	10.86970719	10.77126859
ENSG00000169727	GPS1	-1.53398694	1.93E-07	-1.288878354	1.42E-05	-2.822865294	11.04852648	10.58367898	10.94216274	9.303936679	9.37815424	9.47746372	9.685630779
ENSG00000144642	RBMS3	-1.301597805	8.19E-06	-1.522154119	1.02E-07	-2.823751924	10.11549941	9.695596167	9.935792933	8.693628619	8.56378716	8.499110916	8.319053686
ENSG00000036672	USP2	-3.586497444	0.131232486	0.760600863	0.764012718	-2.825896581	2	5.303523747	2	2.3892346	2.2680111	5.026717879	4.105828626
ENSG00000049449	RCN1	-0.569253257	0.037200815	-2.258619376	1.15E-20	-2.827872634	12.81818241	12.56720988	12.41904395	12.06975087	12.0139954	10.32993244	10.38126632
ENSG00000197299	BLM	-1.678591066	0.502067192	-1.15163983	0.646513979	-2.830230896	2	5.457120953	2	3.162721474	2.94009902	3.193831101	3.496556178
ENSG00000213741	RPS29	-0.939736459	0.367712724	-1.890544924	0.047947959	-2.830281383	11.47006801	9.256956408	9.287079172	9.408447353	9.5300868	8.631223999	8.415288533
ENSG00000112312	GMNN	-1.230192995	0.246051772	-1.600121881	0.113988623	-2.830314877	8.257427002	7.35507212	5.39244166	6.306580752	6.14893615	5.944926241	5.816671626
ENSG00000182899	RPL35A	-1.368613317	0.054635828	-1.463156328	0.036332662	-2.831769646	12.38030589	10.60438382	11.4530647	10.2522744	10.3291788	10.17482878	10.21885096
ENSG00000196408	NOXO1	0.427506011	0.918975348	-3.261183263	NA	-2.833677252	3.327711234	2	2	2.695456494	2.77779907	2.139668783	2
ENSG00000183161	FANCF	-1.567136812	0.054791031	-1.266645596	0.122000983	-2.833782408	8.19931886	6.311476754	7.552162999	6.034011955	6.02777321	6.595545082	5.964937606
ENSG00000179256	SMCO3	-4.714884954	0.291724472	1.876909989	0.666037299	-2.837974965	2	3.076665209	2	2	2	3.429858444	3.560117069
ENSG00000140463	BBS4	-1.496333213	0.001579876	-1.342422527	0.004656627	-2.83875574	9.143983763	8.820493869	8.110401118	7.238438152	7.32531744	7.487996019	7.375063227
ENSG00000086666	ZFAND6	-0.772040178	0.42301813	-2.066836511	0.01584134	-2.83887669	11.26935334	8.903033433	10.02619908	9.689531608	9.5196921	8.404758425	8.236348546
ENSG00000269343	ZNF587B	-1.453356309	0.361244176	-1.385990349	0.376070469	-2.839346657	4.467311984	6.899080544	9.301632639	6.628980152	6.55189318	6.272864826	6.957866334

ENSG00000178149	DALRD3	-1.755367373	0.254759301	-1.085673002	0.492815255	-2.841040375	4.00722531	7.538394143	9.180874355	6.34372989	6.29861567	7.019897747	6.905807071
ENSG00000198440	ZNF583	-1.428157938	0.466937891	-1.413738675	0.461896088	-2.841896613	2	6.666836837	8.434763955	5.709096143	6.00960265	6.049001768	5.689801209
ENSG00000167103	PIP5KL1	-2.625472152	0.164314918	-0.217613221	0.922180661	-2.843085373	7.384433967	2	5.156626401	3.709785322	3.98554648	5.696113106	6.070564958
ENSG00000087502	ERGIC2	-1.373127721	0.023590666	-1.469970646	0.013945355	-2.843098367	10.16187685	8.862353853	10.12770132	8.488325436	8.43865202	8.508806077	8.211820731
ENSG00000135077	HAVCR2	-3.283783577	0.0122849	0.437812053	0.768665969	-2.845971524	5.095959458	4.710564154	5.59507254	2.784612485	2.94009902	6.320128742	3.875378318
ENSG00000198185	ZNF334	-1.287598273	0.481054906	-1.559342235	0.373139559	-2.846940508	5.330594134	7.145017454	2	4.783303513	4.83276841	4.727866721	4.411752372
ENSG00000144591	GMPPA	-1.322859122	6.53E-06	-1.524522455	1.19E-07	-2.847381577	10.05943807	9.680074956	10.04361983	8.624367384	8.61726622	8.303181855	8.530842618
ENSG00000131269	ABCB7	-1.495433426	0.000142273	-1.353171442	0.000546278	-2.848604869	8.493334308	8.029695761	8.763901704	6.936770382	7.03319408	7.124495877	7.123062801
ENSG00000100726	TELO2	-1.537748665	0.075134033	-1.310959432	0.128768928	-2.848708096	9.344956677	7.145017454	9.165043284	7.288834728	7.33629421	7.578754018	7.488317792
ENSG00000164051	CCDC51	-2.121352662	0.061450217	-0.727491856	0.556696605	-2.848844518	9.2903348	6.464272742	6.958202216	5.949776857	6.18176397	7.262061517	7.544398272
ENSG00000147041	SYTL5	-3.322470231	0.318527649	0.472854102	0.895713612	-2.849616129	2	4.442710129	2	2.3892346	2	3.865109708	3.407202941
ENSG00000091137	SLC26A4	-1.378080213	0.329342401	-1.471898841	0.280146209	-2.849979054	3.327711234	4.93638933	5.59507254	3.754611435	3.82856379	4.199031499	3.012034821
ENSG00000132002	DNAJB1	-1.961071125	1.94E-05	-0.889587149	0.072934705	-2.850658274	11.9425688	10.95327452	11.82959564	9.641857585	9.71876306	10.85384055	10.63508781
ENSG00000120697	ALG5	-0.543308838	0.594639663	-2.30757328	0.008111825	-2.850882118	10.33377634	7.874700259	9.049077186	8.868232317	8.88857762	7.107581047	7.178120748
ENSG00000174695	TMEM167A	-1.458430501	0.002590338	-1.393133705	0.003999086	-2.851564207	10.56418906	11.54371287	11.55983287	9.856914282	9.81737584	9.975607996	9.82503293
ENSG00000072506	HSD17B10	-1.642857519	0.008043844	-1.211128127	0.056459449	-2.853985646	9.98400217	8.791896851	9.767769415	7.922877029	8.01555453	7.912394292	8.757326984
ENSG00000143368	SF3B4	-1.590690912	0.005010769	-1.263385199	0.029032528	-2.854076111	11.30078111	10.67263725	10.16000817	9.206465443	9.19832501	9.122554694	9.843777824
ENSG00000169919	GUSB	-2.026631842	4.84E-14	-0.829392316	0.003408467	-2.856024158	9.720386367	9.497318007	9.746650996	7.596453725	7.70428369	8.946676188	8.707903068
ENSG00000169925	BRD3	-1.559884908	3.61E-06	-1.296705458	0.000135243	-2.856590366	10.31337612	10.42977533	9.888372618	8.622857004	8.73530306	9.082719462	8.783638895
ENSG00000274671		-1.8727386	0.390227632	-0.98436935	0.649493169	-2.85710795	2	3.686303414	3.369317972	2.270786764	2.59489879	2.526562374	2.918126211
ENSG00000141449	GREB1L	1.996700168	0.195586526	-4.854346519	0.000723382	-2.857646351	2	6.535001748	4.874587708	7.25803034	7.36158648	2.457042231	2.374681325
ENSG00000130950	NUTM2F	2.330936339	NA	-5.191097062	NA	-2.860160723	2	2	2	2.947924294	3.39680241	2	2
ENSG00000184792	OSBP2	-3.658561394	8.02E-08	0.798298348	0.292247026	-2.860263046	8.107517749	7.002537973	7.342949392	4.237680128	4.1930041	7.667028121	8.797990868
ENSG00000111361	EIF2B1	-1.609522312	0.003680018	-1.25129718	0.027315593	-2.860819492	10.23600589	9.159587375	10.38167985	8.319579168	8.50986596	8.640073371	8.89361766
ENSG00000168569	TMEM223	-1.790798158	0.270288869	-1.074110395	0.521409207	-2.864908553	9.112843761	8.337339476	3.369317972	6.470063213	6.46120797	7.315824427	6.977399345
ENSG00000127989	MTERF1	-1.158270159	0.237918279	-1.707244474	0.065523007	-2.865514633	8.696033109	6.389896633	7.225742779	6.658765962	6.5890786	6.287958221	5.888587685
ENSG00000107949	BCCIP	-1.073710119	0.000513088	-1.791945454	1.17E-09	-2.865655573	10.02639697	9.741182361	10.17589429	9.010169232	8.82765235	8.110051399	8.305980863
ENSG00000172728	FUT10	-1.636585398	0.000131823	-1.229108657	0.004594153	-2.865694054	7.624139768	8.395519306	7.934808822	6.516352584	6.35727082	6.884127769	6.77351449
ENSG00000159596	TMEM69	-1.428699774	0.112341523	-1.446821659	0.101201855	-2.875521433	10.04301211	8.955547846	7.691296561	7.791909949	7.77322015	7.541454591	7.958255135
ENSG00000054690	PLEKHH1	-2.493628445	0.00938604	-0.382286219	0.723692046	-2.875914664	4.00722531	5.457120953	5.59507254	3.023133209	3.50452879	4.868377487	4.827924991
ENSG00000095303	PTGS1	-0.782840344	0.46185912	-2.093187639	0.026578616	-2.876027983	8.493334308	6.535001748	8.884815469	7.559034656	7.4280824	6.672321934	5.59182244
ENSG00000118162	KPTN	-1.869266624	0.001675231	-1.006952166	0.104207046	-2.876218791	7.384433967	7.099070191	6.322058544	5.194621444	5.30456641	5.751606453	6.286392397
ENSG00000182810	DDX28	-1.597583075	0.000547787	-1.278718494	0.006075791	-2.876301569	8.39321512	7.503546079	7.896971941	6.387081877	6.44777477	6.62753908	6.813187523
ENSG00000104998	IL27RA	-2.382374605	0.207400524	-0.494350545	0.815972748	-2.87672515	8.737455672	6.951736442	2	5.144975053	5.39133044	7.332904981	6.693102083
ENSG00000185303	SFTPA2	-3.274362978	0.456607119	0.397169276	NA	-2.877193702	2	2	3.369317972	2	2.14022021	3.1504466	2.117335275
ENSG00000184154	LRTOMT	-1.941909325	0.007283609	-0.93674479	0.215049853	-2.878654115	5.532353208	6.389896633	6.629488387	4.237680128	4.74762503	5.102662916	5.626876946
ENSG00000106268	NUDT1	-2.238690145	0.164314918	-0.640278309	0.717839521	-2.878968455	8.367060731	5.946638857	3.369317972	5.003571641	4.95174433	6.363520437	6.528072775
ENSG00000101004	NINL	-0.522048186	0.546426658	-2.357065361	0.001300235	-2.879113547	7.709880939	7.538394143	9.180874355	7.700356475	7.9408106	6.189586148	5.905000563
ENSG00000146109	ABT1	-1.642894336	4.76E-05	-1.237528247	0.002625096	-2.880422584	8.609439662	9.214490408	9.439621147	7.481152682	7.53358066	7.850199974	7.958255135
ENSG00000182307	C8orf33	-2.060294166	1.17E-06	-0.821146659	0.075265997	-2.881440825	10.15425007	9.415325457	10.29705308	7.944803394	7.97164382	9.286835797	9.0731854
ENSG00000196465	MYL6B	-1.000167292	0.112165694	-1.881954767	0.001141619	-2.882122059	10.25038534	9.04308532	9.624627483	8.724940594	8.7297638	7.451596164	8.173382342
ENSG00000140374	ETFA	-0.90830538	0.448379821	-1.974905765	0.068863117	-2.883211145	10.98974631	9.377333006	7.734844356	9.011324139	9.02712059	7.882875413	8.042267212
ENSG00000182518	FAM104B	-1.958390638	0.008742661	-0.926577288	0.238534046	-2.884967927	6.563806279	7.051611315	5.39244166	4.647285135	4.79082474	5.4588802	5.794819115
ACRC	ACRC	3.266878519	0.310604101	-6.152931129	0.075017773	-2.886052609	3.327711234	2	2	4.54891529	4.50979031	2	2
ENSG00000196407	THEM5	-4.71488067	0.300402677	1.825880697	0.681431908	-2.888999974	2	3.076665209	2	2	2	3.753554863	3.100202476
ENSG00000067533	RRP15	-1.519848591	0.134992306	-1.36934732	0.174527088	-2.889195911	8.285626337	7.538394143	10.10298738	7.53353831	7.57432889	7.68709225	7.715835172
ENSG00000172346	CSDC2	-3.313112567	0.098913801	0.423502963	0.856270394	-2.889609604	8.816894989	6.389896633	2	4.443344104	4.43005853	6.380048704	8.62735196
ENSG00000133328	HRASLS2	0.55292621	0.832844524	-3.442660783	0.126117296	-2.889734573	2	2	4.874587708	3.95996233	4.02226117	2.267001569	2.225841198
ENSG00000117155	SSX2IP	-1.381858822	0.262822363	-1.508804617	0.208845264	-2.890663439	7.384433967	8.655694736	5.156626401	6.422239259	6.22964624	6.439833081	5.933281326
ENSG00000105443	CYTH2	-0.887013828	0.103494474	-2.004361861	4.61E-05	-2.891375688	10.92846914	9.821190352	10.81605395	9.730780958	9.69763767	8.673022865	8.533484187
DUS2	DUS2	-1.703530555	0.041956419	-1.187910495	0.163887111	-2.891441051	6.138360429	8.124485672	7.777116083	5.753943344	6.09824986	6.109723842	6.681239787
ENSG00000184708	EIF4ENIF1	-1.552376744	0.527194576	-1.340369743	0.581686453	-2.892746486	2	6.899080544	10.94679967	7.870381136	7.95274764	8.224302521	8.009436866
ENSG00000069509	FUNDC1	-1.340440469	0.02968828	-1.55354509	0.009522887	-2.893985559	8.674867122	7.35507212	7.818184403	6.634986713	6.84106088	6.546183077	6.528072775
ENSG00000143156	NME7	-0.635275875	0.236508204	-2.258822944	1.17E-06	-2.894098818	8.285626337	7.701025996	7.285536013	7.157295126	7.23044321	5.590506472	5.708623051
ENSG00000178573	MAF	-4.055114762	5.66E-10	1.158654855	0.095534894	-2.896459907	7.435667355	7.874700259	7.552162999	4.443344104	3.28037825	8.601831186	8.937160962
ENSG00000163006	CCDC138	-1.038489915	0.530264818	-1.857977125	0.223744982	-2.89646704	2	5.946638857	4.874587708	4.17310023	4.12711147	3.600766115	3.518055793
ENSG00000071859	FAM50A	-1.267594533	0.058083878	-1.629664137	0.011313199	-2.89725867	11.16695586	9.648522413	10.11127249	9.135152476	9.24469897	8.916644086	8.741420621
ENSG00000186871	ERCC6L	-1.828039726	0.479583688	-1.069803142	0.684964093	-2.897842868	2	5.72254323	2	2.947924294	3.28037825	3.10571689	3.940519005
ENSG00000110881	ASIC1	-2.822067849	0.154010606	-0.077982186	0.973251353	-2.900050035	4.00722531	9.055168103	3.369317972	4.804776789	4.98931816	7.619097297	7.302581437
ENSG00000178343	SHISA3	-3.917262418	0.211000866	1.01686808	0.747201125	-2.900394338	2	4.442710129	2	2.270786764	2	3.838019203	4.241259475
ENSG00000049323	LTBP1	-2.232952584	0.019076624	-0.668287599	0.530366429	-2.901240184	10.95943304	13.78286584	12.98235842	10.74032429	10.7523441	12.56045519	12.00372897
ENSG00000159593	NAE1	-0.284064734	0.801824404	-2.617375397	0.003770117	-2.901440131	10.14658276	8.077869079	8.042706615	8.83470036	8.8354102	6.739740021	6.33565646
ENSG00000182508	LHFPL1	0.991084307	0.713785889	-3.893577725	0.126947565	-2.902493418	2	3.686303414	2	3.09461492	3.65239003	2.094598369	2.059860178
ENSG00000022976	ZNF839	-1.191892872	0.324504083	-1.713020334	0.134216355	-2.904913206	8.816894989	7.538394143	5.39244166	6.604700657	6.71776877	6.22922222	6.099376274
ENSG00000157020	SEC13	-1.700063959	0.002924743	-1.205998832	0.041029321	-2.906062791	12.55410083	11.50459547	11.32712828	10.16571753	10.245283	10.56905757	10.81765528
ENSG00000169905	TOR1AIP2	-1.568062107	0.001457494	-1.33900646	0.007299395	-2.907068567	11.06081658	11.57970793	12.18594181	10.09829921	10.135408	10.25574405	10.42957331
ENSG00000186787	SPIN2B	-0.891140938	0.305697747	-2.016692222	0.010048431	-2.90783316	6.653098118	4.93638933	6.43197674	5.304221918	5.36298494	4.311629396	4.4231929
ENSG00000188342	GTF2F2	-1.255585176	0.230926542	-1.65398868	0.098757188	-2.909573856	10.56993315	8.916342327	7.818184403	8.285617933	8.29788471	7.87872738	7.919398473
ENSG00000140332	TLE3	-0.957558593	0.091400188	-1.952837437	0.000158283	-2.910396029	9.521325812	9.102512015	9.385998894	8.341783829	8.44880239	7.85696323	6.782425482
ENSG00000177600	RPLP2	-1.233378285	0.05970955	-1.677421517	0.007405859	-2.910799802	12.53660743	11.2524908	11.23155829	10.55871559	10.6016606	10.02279773	10.24406712
ENSG00000162384	C1orf123	-1.680155279	3.10E-11	-1.231056894	1.11E-06	-2.911212173	9.097018025	9.067150531	8.922968322	7.341208691	7.41087845	7.767364534	7.863808491
ENSG00000117862	TXNDC12	0.126603713	0.850047138	-3.038859502	8.79E-10	-2.91225579	9.448362284	9.267380431	10.28977157	9.817184017	9.90967722	6.956760977	6.501335855
ENSG00000125901	MRPS26	-1.84124714	0.053531997	-1.071311069	0.280385375	-2.912558209	9.931427102	8.191702463	7.502634626	7.157295126	7.05107174	7.87872738	7.836259222
ENSG00000157343	ARMC12	-2.209184749	0.361916636	-0.70364882	0.784923737	-2.912833568	2	5.303523747	2	2.695456494	2.77779907	3.648325314	3.407202941
ENSG00000128645	HOXD1	2.276328435	NA	-5.191097289	NA	-2.914768854	2	2	2	3.349643765	2.94009902	2	2
ENSG00000159377	PSMB4	-1.27920546	0.028200875	-1.636480699	0.003631339	-2.915686159	12.32363489	11.03180337	11.38976845	10.38694723	10.4355118	10.02129326	10.08862406
C10ORF35	C10ORF35	2.274983007	NA	-5.191097295	NA	-2.916114288	2	2	2	3.227757282	3.08597292	2	2
ENSG00000146267	FAXC	-0.590465287	0.764636496	-2.327173673	0.178036985	-2.917638959	3.327711234	5.131603625	2	3.881293667	3.39680241	2.656294034	2.782576693
ENSG00000241394		3.234980731	0.324107683	-6.15293023	0.079108348	-2.917949498	2	3.076665209	2	4.443344104	4.56059747	2	2
ENSG00000177301	KCNA2	-3.267594726	0.479080051	0.349492645	NA	-2.918102081	3.327711234	2	2	2.141737246	2	3.1504466	2.059860178
[truncated: 862,822 more chars]
