# Supplementary material for: Single-cell RNA sequencing and spatial transcriptomics reveal cancer-associated fibroblasts in glioblastoma with protumoral effects
Source: J Clin Invest. 2023 Mar 1;133(5):e147087. doi: 10.1172/JCI147087 (PMC9974099; doi:10.1172/JCI147087)
Supplement: Supplemental table 2 [file jci-133-147087-s012.pdf]

	Total # of cells	NPEE cells	CAF MARKERS								NPEE cells expressing any CAF marker
			ACTA2	FAP	PDGFRA	PDGRFB	PDPN	TNC	S100A4	COL1A1	
	2640	1987	1466	111	176	253	393	742	341	1917	1719
% cells of NPEE		100%	73.80%	5.60%	8.90%	12.70%	19.80%	37.3%	17.2%	96.5%	86.5%
% cells of Total		75.3%	55.50%	4.20%	6.70%	9.60%	14.90%	28.1%	12.9%	96.5%	65.1%

Supplemental Table 2. Number of cells isolated by serial trypsinization expressing various stromal markers by scRNA-seq. Shown is the number of cells from scRNA-seq of cells isolated from serial trypsinization of patient GBM that were negative for pericyte, epithelial, enothelial, or immune cell markers (NPEE) and the number of NPEE cells expressing each of 8 CAF markers along with the number of NPEE cells expresing any CAF marker, a metric that gives a sense of what portion of these cells are CAFs.
