# Supplementary material for: Single-cell RNA sequencing and spatial transcriptomics reveal cancer-associated fibroblasts in glioblastoma with protumoral effects
Source: J Clin Invest. 2023 Mar 1;133(5):e147087. doi: 10.1172/JCI147087 (PMC9974099; doi:10.1172/JCI147087)
Supplement: Supplemental table 4 [file jci-133-147087-s014.pdf]

SUPPLEMENTAL TABLE 4: CAF proliferation in response to various conditions.

Cell Index at: 0:00:00

	1	2	3	4	5	6	7	8	9	10	11	12
A								0	0	0	0	0
B								0	0	0	0	0
C								0	0	0	0	0
D								0	0	0	0	0
E								0	0	0	0	0
F								0	0	0	0	0
G								0	0	0	0	0
H								0	0	0	0	0

Cell Index at: 0:00:21

	1	2	3	4	5	6	7	8	9	10	11	12
A								0.0002	0.0004	-0.0015	0.0002	-0.0053
B								-0.0008	-0.0021	-0.0047	-0.0018	-0.0007
C								0.0068	-0.0019	-0.004	0.0002	-0.0021
D								0.0034	0.0065	-0.0045	-0.0015	0.0034
E								0.0105	-0.005	-0.007	-0.0049	-0.0067
F								0.0037	-0.0026	-0.0051	0.0058	-0.0003
G								-0.0052	0.0036	-0.0004	-0.0006	-0.0025
H								-0.0011	-0.0023	0.0003	0.001	0.0013

Cell Index at: 2:00:19

	1	2	3	4	5	6	7	8	9	10	11	12
A								0.0354	0.0381	0.0541	0.0476	0.0409
B								0.021	0.0168	0.0256	0.0273	0.0427
C								0.0082	0.0255	0.0357	0.0312	0.0352
D								0.0192	0.0213	0.0136	0.0122	0.0274
E								0.0018	-0.0117	-0.0149	-0.0026	-0.0132
F								0.034	0.0321	0.0315	0.0302	0.0367
G								0.0175	0.0284	0.0154	0.0209	0.0188
H								0.054	0.0498	0.0592	0.0523	0.0384

Cell Index at: 2:15:18

	1	2	3	4	5	6	7	8	9	10	11	12
A								-0.0056	-0.0034	0.0116	0.0022	0.0057
B								-0.0068	-0.0022	0.0161	0.0131	0.0157
C								0.0445	0.0435	0.059	0.0555	0.0489
D								0.0482	0.0491	0.0496	0.0549	0.0534
E								0.0146	0.0011	0.0089	0.009	0.008
F								0.046	0.0501	0.0516	0.0551	0.0598
G								0.0061	0.0139	0.0098	0.0152	0.0099
H								0.045	0.0367	0.0527	0.0454	0.0335

Cell Index at: 2:30:18

	1	2	3	4	5	6	7	8	9	10	11	12
A								0.0014	-0.0012	0.0147	0.0104	0.0117
B								0.0057	0.005	0.021	0.0186	0.0206
C								0.0551	0.0592	0.0666	0.0762	0.0633
D								0.0775	0.0838	0.0741	0.067	0.0754
E								0.0338	0.0199	0.0195	0.0074	0.0255
F								0.0668	0.066	0.0777	0.0681	0.0803
G								0.0163	0.0198	0.0142	0.0295	0.0138
H								0.0526	0.0487	0.0661	0.0618	0.0451

Cell Index at: 2:45:18

	1	2	3	4	5	6	7	8	9	10	11	12
A								0.0024	0.0064	0.0235	0.018	0.0171
B								0.0088	0.0158	0.0267	0.0278	0.0306
C								0.0652	0.0799	0.0914	0.0945	0.0746
D								0.0953	0.0875	0.0878	0.0823	0.0876
E								0.0515	0.0297	0.0396	0.0228	0.036
F								0.0795	0.0863	0.0934	0.0891	0.0871
G								0.0249	0.0346	0.0224	0.0381	0.0234
H								0.0606	0.0567	0.077	0.0671	0.0545

Cell Index at: 3:00:18

	1	2	3	4	5	6	7	8	9	10	11	12
A								0.009	0.0142	0.0146	0.0251	0.023
B								0.0158	0.0312	0.0406	0.0417	0.04
C								0.0836	0.0812	0.0998	0.1008	0.0919
D								0.1096	0.1074	0.1012	0.1089	0.1007
E								0.0638	0.0435	0.0493	0.0356	0.0423
F								0.0954	0.0974	0.101	0.0959	0.0998
G								0.0362	0.0408	0.0332	0.0486	0.0319
H								0.0698	0.0626	0.083	0.0725	0.0617

Cell Index at: 3:15:19

	1	2	3	4	5	6	7	8	9	10	11	12
A								0.0147	0.0193	0.0142	0.0369	0.0324
B								0.0229	0.0372	0.0439	0.0512	0.0412
C								0.1043	0.0974	0.1092	0.1055	0.0934
D								0.1159	0.117	0.1121	0.1127	0.1097
E								0.0752	0.0535	0.0557	0.0432	0.0509
F								0.0994	0.1058	0.1058	0.1041	0.1065
G								0.0398	0.0475	0.0366	0.0546	0.0354
H								0.0781	0.0687	0.0922	0.0792	0.0731

Cell Index at: 3:30:19

	1	2	3	4	5	6	7	8	9	10	11	12
A								0.0296	0.0255	0.0189	0.0507	0.0398
B								0.0251	0.0486	0.0488	0.0588	0.0526
C								0.1009	0.1017	0.1124	0.1112	0.0969
D								0.1185	0.1291	0.1102	0.1213	0.1053
E								0.0824	0.0678	0.0629	0.0491	0.0536
F								0.1101	0.1078	0.1142	0.1047	0.1153
G								0.0464	0.0576	0.0477	0.0642	0.0478
H								0.0851	0.0731	0.0975	0.083	0.0801

Cell Index at: 3:45:19

	1	2	3	4	5	6	7	8	9	10	11	12
A								0.037	0.0304	0.0254	0.0628	0.0356
B								0.0323	0.0568	0.0535	0.0641	0.0605
C								0.1089	0.1095	0.124	0.1255	0.1
D								0.1257	0.1328	0.121	0.1206	0.1178
E								0.0832	0.0682	0.0766	0.0595	0.0633
F								0.1042	0.1256	0.1163	0.1107	0.1188
G								0.0567	0.0643	0.0564	0.0707	0.0531
H								0.0881	0.0778	0.1022	0.0901	0.0851

Cell Index at: 4:00:20

	1	2	3	4	5	6	7	8	9	10	11	12
A								0.0415	0.0343	0.0322	0.0664	0.04
B								0.0361	0.0619	0.0571	0.0675	0.0683
C								0.1148	0.1123	0.1281	0.1291	0.103
D								0.133	0.1414	0.1333	0.1184	0.1172
E								0.089	0.0761	0.0698	0.062	0.0678
F								0.1127	0.1295	0.1254	0.1215	0.1249
G								0.0591	0.0652	0.0569	0.0811	0.0592
H								0.0869	0.0841	0.1048	0.0914	0.0887

Cell Index at: 4:15:20

	1	2	3	4	5	6	7	8	9	10	11	12
A								0.0456	0.0418	0.0384	0.0733	0.0433
B								0.0428	0.0656	0.0622	0.0747	0.0796
C								0.1227	0.115	0.132	0.1386	0.1086
D								0.1384	0.1478	0.1416	0.1302	0.1235
E								0.0984	0.0823	0.0858	0.0667	0.0718
F								0.108	0.1281	0.1288	0.122	0.1346
G								0.0654	0.07	0.0671	0.0805	0.065

Sample Key

A. Neurosphere media
B. Neurosphere media + anti-PDGF
C. Stem Cell CM
D. Stem Cell CM + anti-PDGF
E. Neurosphere media + anti-TGF-B
F. Stem Cell CM + anti-TGF-B
G. Neurosphere media + Both
H. Stem Cell CM + Both

H								0.0897	0.0879	0.1106	0.0951	0.0905

Cell Index at: 4:30:21

	1	2	3	4	5	6	7	8	9	10	11	12
A								0.0483	0.045	0.0399	0.0746	0.0517
B								0.0435	0.0643	0.0689	0.0813	0.0817
C								0.1234	0.1212	0.1359	0.1419	0.112
D								0.1411	0.1527	0.1399	0.1305	0.133
E								0.0978	0.0808	0.0909	0.0804	0.0681
F								0.12	0.1386	0.1344	0.1217	0.1384
G								0.0675	0.0766	0.0711	0.0877	0.0658
H								0.0947	0.0934	0.1144	0.1002	0.092

Cell Index at: 4:45:20

	1	2	3	4	5	6	7	8	9	10	11	12
A								0.0534	0.0476	0.0436	0.0777	0.0574
B								0.0505	0.0792	0.0733	0.0824	0.0869
C								0.1319	0.1198	0.1392	0.1452	0.1218
D								0.1419	0.1536	0.1421	0.1304	0.1375
E								0.1058	0.0889	0.0964	0.0815	0.0731
F								0.1269	0.1404	0.1381	0.1282	0.1444
G								0.0689	0.0769	0.0753	0.0924	0.0697
H								0.098	0.0916	0.1226	0.1072	0.095

Cell Index at: 5:00:21

	1	2	3	4	5	6	7	8	9	10	11	12
A								0.0551	0.0538	0.0447	0.083	0.065
B								0.0527	0.0806	0.0747	0.0909	0.0933
C								0.1365	0.1227	0.1502	0.153	0.1289
D								0.1514	0.1595	0.1468	0.1314	0.139
E								0.1121	0.0947	0.1045	0.0881	0.081
F								0.1298	0.1457	0.1423	0.1298	0.148
G								0.0714	0.0841	0.0782	0.0987	0.0784
H								0.1041	0.0931	0.1251	0.1064	0.0974

Cell Index at: 5:15:21

	1	2	3	4	5	6	7	8	9	10	11	12
A								0.053	0.0564	0.0481	0.0906	0.0709
B								0.0587	0.085	0.0839	0.0998	0.0941
C								0.1348	0.1277	0.1492	0.1616	0.1377
D								0.1646	0.1621	0.1473	0.141	0.1395
E								0.1203	0.1063	0.102	0.0878	0.0826
F								0.1297	0.1459	0.1505	0.1385	0.1498
G								0.0755	0.0901	0.0818	0.1036	0.0766
H								0.1079	0.1016	0.1274	0.1066	0.0966

Cell Index at: 5:30:21

	1	2	3	4	5	6	7	8	9	10	11	12
A								0.0511	0.0599	0.0567	0.0871	0.0737
B								0.0604	0.0834	0.0839	0.0993	0.1006
C								0.1439	0.1247	0.1574	0.1646	0.1388
D								0.1492	0.1668	0.1525	0.145	0.1455
E								0.1162	0.1111	0.1177	0.0858	0.0894
F								0.1272	0.1543	0.1509	0.1417	0.1549
G								0.0751	0.0995	0.0836	0.1018	0.0854
H								0.1104	0.1008	0.1332	0.1146	0.0974

Cell Index at: 5:45:22

	1	2	3	4	5	6	7	8	9	10	11	12
A								0.0606	0.0616	0.0589	0.0946	0.0783
B								0.0631	0.0858	0.0842	0.0978	0.1015
C								0.1413	0.1257	0.1551	0.1636	0.1415
D								0.1552	0.1717	0.1571	0.1408	0.1495
E								0.1163	0.1065	0.11	0.098	0.0885
F								0.1317	0.1518	0.1591	0.1419	0.1587
G								0.0796	0.0962	0.0862	0.1028	0.083
H								0.1179	0.1034	0.1355	0.1166	0.1021

Cell Index at: 6:00:22

	1	2	3	4	5	6	7	8	9	10	11	12
A								0.0586	0.0677	0.0627	0.0972	0.0844
B								0.069	0.0918	0.0898	0.1068	0.1057
C								0.1457	0.1263	0.1598	0.1724	0.1387
D								0.1531	0.1774	0.1552	0.1542	0.1614
E								0.1233	0.1215	0.1202	0.1046	0.0912
F								0.141	0.1632	0.1613	0.1523	0.1577
G								0.0845	0.1065	0.0907	0.1073	0.0896
H								0.1178	0.1053	0.1371	0.117	0.104

Cell Index at: 6:15:22

	1	2	3	4	5	6	7	8	9	10	11	12
A								0.066	0.0677	0.0654	0.1032	0.0884
B								0.0731	0.0935	0.0951	0.1084	0.1055
C								0.1456	0.1396	0.1537	0.1685	0.1515
D								0.1624	0.1714	0.1549	0.1572	0.1678
E								0.1172	0.1076	0.1255	0.1059	0.0964
F								0.1367	0.1642	0.1613	0.154	0.1618
G								0.08	0.1053	0.0934	0.1116	0.0875
H								0.1166	0.1016	0.1384	0.1229	0.1042

Cell Index at: 6:30:21

	1	2	3	4	5	6	7	8	9	10	11	12
A								0.0667	0.0706	0.0674	0.1021	0.0947
B								0.0787	0.0894	0.0948	0.1146	0.1121
C								0.1547	0.1372	0.1683	0.1738	0.1475
D								0.1629	0.1759	0.1602	0.159	0.1604
E								0.1282	0.1218	0.1233	0.1133	0.0934
F								0.1519	0.16	0.1642	0.1576	0.1687
G								0.082	0.1026	0.0988	0.1137	0.0894
H								0.1235	0.1129	0.1399	0.1232	0.1074

Cell Index at: 6:45:22

	1	2	3	4	5	6	7	8	9	10	11	12
A								0.0695	0.0733	0.0671	0.0969	0.0963
B								0.0836	0.0923	0.0973	0.1163	0.1147
C								0.1612	0.143	0.1719	0.1776	0.1582
D								0.1624	0.1795	0.1583	0.1675	0.1583
E								0.1219	0.1257	0.1209	0.1209	0.0909
F								0.1463	0.1672	0.1692	0.1581	0.1675
G								0.0869	0.1056	0.0987	0.1162	0.091
H								0.1203	0.1099	0.1454	0.1305	0.1065

Cell Index at: 7:00:21

	1	2	3	4	5	6	7	8	9	10	11	12
A								0.0692	0.0775	0.0727	0.0969	0.1027
B								0.0906	0.094	0.1013	0.1186	0.1158
C								0.1604	0.1395	0.1775	0.1719	0.1592
D								0.1624	0.1786	0.1559	0.1664	0.1705
E								0.1217	0.1307	0.1396	0.12	0.1005
F								0.1502	0.169	0.1767	0.1602	0.172
G								0.088	0.1069	0.0994	0.1178	0.0929
H								0.1265	0.1131	0.1481	0.1292	0.1098

Cell Index at: 7:15:21

	1	2	3	4	5	6	7	8	9	10	11	12
A								0.0731	0.0816	0.0747	0.1028	0.1045
B								0.0907	0.1	0.0948	0.118	0.1202
C								0.1607	0.1392	0.1743	0.1794	0.1579
D								0.1686	0.1841	0.1688	0.159	0.1654
E								0.1322	0.1499	0.1337	0.1223	0.1076
F								0.1417	0.1733	0.1733	0.1615	0.178

	1	2	3	4	5	6	7	8	9	10	11	12
G								0.0892	0.1102	0.1038	0.1156	0.0955
H								0.1273	0.1156	0.1496	0.1302	0.1103

Cell Index at: 7:30:22

	1	2	3	4	5	6	7	8	9	10	11	12
A								0.0765	0.0818	0.0749	0.1006	0.1068
B								0.0947	0.102	0.1014	0.1227	0.1282
C								0.1606	0.1458	0.1732	0.184	0.1605
D								0.1668	0.196	0.1686	0.1655	0.1749
E								0.1323	0.1342	0.1373	0.1268	0.1118
F								0.1458	0.1717	0.177	0.1647	0.1776
G								0.0849	0.1079	0.1097	0.1236	0.0962
H								0.13	0.1148	0.1535	0.1324	0.1102

Cell Index at: 7:45:23

	1	2	3	4	5	6	7	8	9	10	11	12
A								0.0742	0.0795	0.0769	0.0983	0.1097
B								0.0971	0.109	0.105	0.1244	0.1217
C								0.1568	0.1419	0.1783	0.1855	0.1686
D								0.1696	0.1802	0.1729	0.1627	0.1796
E								0.1354	0.137	0.1388	0.128	0.1104
F								0.1569	0.1733	0.1821	0.1703	0.1789
G								0.0915	0.1125	0.1069	0.1254	0.1006
H								0.1314	0.117	0.1532	0.1327	0.1123

Cell Index at: 8:00:24

	1	2	3	4	5	6	7	8	9	10	11	12
A								0.0802	0.0866	0.0817	0.1026	0.1061
B								0.1014	0.1039	0.1064	0.1269	0.1217
C								0.1558	0.1488	0.1761	0.186	0.1654
D								0.1715	0.1845	0.1751	0.1617	0.1782
E								0.1401	0.1323	0.1455	0.1359	0.1159
F								0.1508	0.1777	0.1813	0.1745	0.1771
G								0.0942	0.1135	0.1143	0.1302	0.1008
H								0.1333	0.117	0.1584	0.1323	0.1087

Cell Index at: 8:15:24

	1	2	3	4	5	6	7	8	9	10	11	12
A								0.0798	0.0865	0.0828	0.104	0.1099
B								0.0989	0.1091	0.1049	0.1287	0.1183
C								0.1623	0.1533	0.1762	0.1853	0.1632
D								0.1725	0.1846	0.1747	0.163	0.1811
E								0.1411	0.144	0.1487	0.1374	0.1275
F								0.1562	0.1803	0.1768	0.176	0.1771
G								0.096	0.1136	0.1145	0.1288	0.1019
H								0.1341	0.1164	0.1583	0.1384	0.1101

Cell Index at: 8:30:25

	1	2	3	4	5	6	7	8	9	10	11	12
A								0.0794	0.084	0.08	0.1077	0.1142
B								0.1095	0.1099	0.1114	0.1332	0.126
C								0.1655	0.1527	0.1817	0.1787	0.1592
D								0.1724	0.1857	0.1775	0.1753	0.1823
E								0.1546	0.154	0.1493	0.14	0.1246
F								0.1595	0.1733	0.1865	0.1752	0.1774
G								0.0944	0.1165	0.1163	0.1298	0.1011
H								0.1369	0.1193	0.1596	0.1392	0.1136

Cell Index at: 8:45:24

	1	2	3	4	5	6	7	8	9	10	11	12
A								0.0836	0.086	0.0778	0.1079	0.1122
B								0.1107	0.1154	0.1113	0.1306	0.1225
C								0.1685	0.1502	0.1847	0.1811	0.1631
D								0.1786	0.1892	0.1827	0.1681	0.1877
E								0.156	0.1524	0.1437	0.1418	0.118
F								0.1544	0.1818	0.1839	0.1732	0.1826
G								0.0979	0.1138	0.1183	0.132	0.0978
H								0.1391	0.1207	0.1586	0.1402	0.1153

Cell Index at: 9:00:25

	1	2	3	4	5	6	7	8	9	10	11	12
A								0.081	0.0885	0.0802	0.111	0.1134
B								0.1112	0.1035	0.1126	0.1353	0.1269
C								0.1708	0.1534	0.1836	0.1841	0.1707
D								0.1829	0.1908	0.1887	0.1696	0.1852
E								0.1472	0.1492	0.1547	0.1444	0.1299
F								0.1573	0.178	0.1833	0.1705	0.1847
G								0.0939	0.1207	0.1168	0.1318	0.1018
H								0.1388	0.1232	0.1681	0.1392	0.1154

Cell Index at: 9:15:25

	1	2	3	4	5	6	7	8	9	10	11	12
A								0.0883	0.0887	0.0804	0.1089	0.1106
B								0.1145	0.1107	0.1168	0.1344	0.1244
C								0.1629	0.1519	0.1811	0.1867	0.1725
D								0.1749	0.1882	0.182	0.1711	0.1841
E								0.151	0.1512	0.1497	0.1505	0.1314
F								0.1604	0.1789	0.1894	0.173	0.1777
G								0.0896	0.1225	0.1193	0.1363	0.1081
H								0.1399	0.1259	0.1704	0.1427	0.1189

Cell Index at: 9:30:24

	1	2	3	4	5	6	7	8	9	10	11	12
A								0.0844	0.0849	0.0826	0.105	0.1147
B								0.1134	0.1056	0.1138	0.1332	0.1227
C								0.1676	0.1436	0.19	0.1905	0.1697
D								0.1832	0.1931	0.1869	0.1802	0.1855
E								0.1471	0.155	0.1597	0.1453	0.1353
F								0.1725	0.1831	0.1898	0.1768	0.181
G								0.0891	0.1091	0.1206	0.1384	0.1053
H								0.1411	0.1247	0.171	0.1473	0.1158

Cell Index at: 9:45:24

	1	2	3	4	5	6	7	8	9	10	11	12
A								0.0835	0.0866	0.0829	0.1094	0.1148
B								0.1121	0.1045	0.115	0.1383	0.125
C								0.17	0.1473	0.1887	0.1971	0.1741
D								0.1846	0.1807	0.1882	0.1797	0.1776
E								0.1591	0.1571	0.1481	0.1474	0.1386
F								0.1708	0.1835	0.196	0.1754	0.1773
G								0.0912	0.1068	0.1302	0.143	0.1028
H								0.1424	0.1278	0.1709	0.1486	0.1175

Cell Index at: 10:00:24

	1	2	3	4	5	6	7	8	9	10	11	12
A								0.0877	0.088	0.0817	0.1101	0.1106
B								0.1145	0.1076	0.113	0.1399	0.1272
C								0.1753	0.1541	0.1919	0.1885	0.1761
D								0.1863	0.1913	0.1821	0.1839	0.1866
E								0.1492	0.1558	0.163	0.1509	0.1298
F								0.1714	0.1895	0.191	0.1756	0.1803
G								0.0902	0.1084	0.1267	0.1381	0.1119
H								0.1464	0.125	0.1732	0.1511	0.117

Cell Index at: 10:15:25

	1	2	3	4	5	6	7	8	9	10	11	12
A								0.0856	0.0934	0.0838	0.109	0.1138
B								0.1116	0.1098	0.1135	0.1352	0.1276
C								0.1667	0.1566	0.2008	0.1944	0.1663
D								0.1788	0.1954	0.1878	0.1779	0.1822
E								0.1507	0.1624	0.1515	0.1596	0.1325

F								0.1763	0.1839	0.2056	0.1779	0.188
G								0.0946	0.1124	0.1253	0.1409	0.1064
H								0.1443	0.1333	0.1729	0.1516	0.118

Cell Index at: 10:30:26

	1	2	3	4	5	6	7	8	9	10	11	12
A								0.0821	0.091	0.0887	0.1107	0.1161
B								0.1159	0.1074	0.11	0.1354	0.1309
C								0.1735	0.1497	0.1941	0.1945	0.1716
D								0.175	0.1881	0.193	0.1878	0.1832
E								0.1507	0.1624	0.1543	0.155	0.1361
F								0.1687	0.1869	0.1988	0.1843	0.1819
G								0.0978	0.1119	0.1218	0.1442	0.1088
H								0.1473	0.1307	0.1789	0.1545	0.1196

Cell Index at: 10:45:27

	1	2	3	4	5	6	7	8	9	10	11	12
A								0.0799	0.0918	0.0938	0.1149	0.1201
B								0.1127	0.112	0.1105	0.1338	0.1312
C								0.1691	0.1591	0.1973	0.2005	0.172
D								0.1757	0.1909	0.1955	0.1875	0.185
E								0.1424	0.1511	0.1474	0.154	0.1371
F								0.1681	0.1907	0.2051	0.1834	0.1778
G								0.096	0.1094	0.1185	0.1462	0.105
H								0.1486	0.1298	0.1796	0.1527	0.1156

Cell Index at: 11:00:26

	1	2	3	4	5	6	7	8	9	10	11	12
A								0.0799	0.0935	0.0925	0.1069	0.122
B								0.1167	0.1071	0.0984	0.1338	0.1326
C								0.1703	0.1584	0.1932	0.2062	0.1771
D								0.1659	0.1863	0.1962	0.1964	0.1892
E								0.1541	0.1517	0.1673	0.1452	0.1377
F								0.1672	0.1975	0.1893	0.1832	0.1817
G								0.0917	0.1134	0.1223	0.1421	0.1059
H								0.149	0.1327	0.1789	0.1561	0.1197

Cell Index at: 11:15:25

	1	2	3	4	5	6	7	8	9	10	11	12
A								0.0862	0.0943	0.0926	0.1038	0.1185
B								0.1156	0.105	0.1032	0.1336	0.1322
C								0.1695	0.1573	0.1968	0.1988	0.176
D								0.1869	0.1989	0.2016	0.1898	0.1905
E								0.1509	0.1544	0.1622	0.1516	0.1415
F								0.1727	0.1989	0.1848	0.1803	0.1792
G								0.0983	0.1176	0.1229	0.1412	0.1022
H								0.1521	0.1298	0.1826	0.1532	0.1155

Cell Index at: 11:30:25

	1	2	3	4	5	6	7	8	9	10	11	12
A								0.0815	0.0874	0.0969	0.1047	0.1193
B								0.122	0.1072	0.1063	0.1401	0.1274
C								0.1737	0.1636	0.2	0.2011	0.1786
D								0.1944	0.1954	0.2122	0.19	0.19
E								0.155	0.1586	0.1635	0.1583	0.134
F								0.1689	0.1909	0.1965	0.1845	0.1861
G								0.0938	0.1156	0.1232	0.1439	0.1049
H								0.1511	0.1278	0.1849	0.16	0.1171

Cell Index at: 11:45:25

	1	2	3	4	5	6	7	8	9	10	11	12
A								0.0847	0.0906	0.0956	0.1042	0.1227
B								0.1221	0.1063	0.1026	0.1405	0.1327
C								0.1734	0.1694	0.2138	0.2099	0.178
D								0.1821	0.1892	0.2126	0.1823	0.1929
E								0.1558	0.1456	0.161	0.165	0.1333
F								0.1741	0.1863	0.1977	0.1861	0.1952
G								0.0938	0.1155	0.1164	0.1464	0.1105
H								0.1509	0.1301	0.1845	0.1594	0.1231

Cell Index at: 12:00:25

	1	2	3	4	5	6	7	8	9	10	11	12
A								0.0848	0.0868	0.0985	0.1042	0.1196
B								0.1198	0.1066	0.1035	0.135	0.1335
C								0.1713	0.1629	0.207	0.2151	0.1788
D								0.1885	0.2063	0.2066	0.1922	0.1942
E								0.1416	0.1535	0.1575	0.1592	0.1303
F								0.1756	0.1874	0.1997	0.1891	0.1953
G								0.095	0.1209	0.1222	0.1418	0.103
H								0.1529	0.1316	0.1896	0.1595	0.121

Cell Index at: 12:15:25

	1	2	3	4	5	6	7	8	9	10	11	12
A								0.0824	0.0876	0.098	0.1055	0.1194
B								0.1207	0.1039	0.1033	0.1425	0.136
C								0.1742	0.1702	0.2047	0.2127	0.1849
D								0.1853	0.2077	0.1996	0.1914	0.1932
E								0.1613	0.1588	0.1674	0.1648	0.1185
F								0.1706	0.2051	0.2032	0.1921	0.1946
G								0.0944	0.1209	0.1177	0.1474	0.1021
H								0.1543	0.1312	0.1879	0.1621	0.1205

Cell Index at: 12:30:26

	1	2	3	4	5	6	7	8	9	10	11	12
A								0.0853	0.0859	0.0969	0.1055	0.1165
B								0.1244	0.1047	0.1001	0.1372	0.1321
C								0.1734	0.1712	0.2108	0.206	0.1833
D								0.1814	0.2103	0.2009	0.1941	0.191
E								0.1516	0.1532	0.171	0.1547	0.1208
F								0.174	0.1885	0.1923	0.1902	0.1959
G								0.093	0.1207	0.1144	0.1447	0.1033
H								0.1554	0.1305	0.1884	0.1621	0.1243

Cell Index at: 12:45:26

	1	2	3	4	5	6	7	8	9	10	11	12
A								0.0781	0.0881	0.0976	0.1057	0.1202
B								0.1239	0.1019	0.0926	0.1408	0.1345
C								0.175	0.1714	0.2133	0.2155	0.1865
D								0.1845	0.2074	0.2058	0.1933	0.19
E								0.1552	0.1491	0.1628	0.1648	0.1227
F								0.172	0.1901	0.2008	0.1927	0.196
G								0.0952	0.113	0.1154	0.1461	0.0955
H								0.1555	0.1374	0.1901	0.1627	0.1241

Cell Index at: 13:00:26

	1	2	3	4	5	6	7	8	9	10	11	12
A								0.0787	0.0876	0.0932	0.1033	0.1263
B								0.1214	0.0998	0.0948	0.1338	0.1301
C								0.1686	0.1721	0.2091	0.2127	0.1886
D								0.1829	0.212	0.2129	0.2005	0.1968
E								0.1455	0.1472	0.1698	0.1615	0.1134
F								0.1728	0.1862	0.1985	0.1893	0.1902
G								0.1	0.1175	0.1129	0.1444	0.0978
H								0.1583	0.1341	0.1919	0.16	0.1233

Cell Index at: 13:15:25

	1	2	3	4	5	6	7	8	9	10	11	12
A								0.0747	0.0816	0.0957	0.0999	0.1217
B								0.1225	0.1041	0.093	0.1394	0.1357
C								0.1831	0.1741	0.2128	0.2192	0.1871
D								0.1853	0.2035	0.1991	0.1947	0.2015

E								0.1494	0.1541	0.1605	0.1597	0.113
F								0.185	0.1895	0.2035	0.1945	0.1973
G								0.1025	0.118	0.1148	0.1437	0.0956
H								0.1572	0.1385	0.1933	0.1633	0.1159

Cell Index at: 13:30:25

	1	2	3	4	5	6	7	8	9	10	11	12
A								0.0731	0.0849	0.0977	0.0962	0.125
B								0.1154	0.1095	0.0883	0.139	0.1355
C								0.1695	0.1741	0.2142	0.2212	0.19
D								0.1914	0.2077	0.2074	0.202	0.1974
E								0.1467	0.1598	0.1658	0.1612	0.1178
F								0.1797	0.1919	0.2079	0.1946	0.2052
G								0.1043	0.1174	0.1155	0.1422	0.1005
H								0.1561	0.1376	0.1922	0.1619	0.1166

Cell Index at: 13:45:25

	1	2	3	4	5	6	7	8	9	10	11	12
A								0.0729	0.0805	0.096	0.0952	0.1181
B								0.1177	0.1115	0.0793	0.1394	0.1322
C								0.1774	0.1715	0.2142	0.2232	0.1847
D								0.1938	0.2019	0.2121	0.201	0.1972
E								0.1554	0.157	0.1726	0.1599	0.1193
F								0.187	0.1958	0.2056	0.2025	0.2016
G								0.104	0.1176	0.117	0.1409	0.0952
H								0.1557	0.1377	0.192	0.1659	0.1186

Cell Index at: 14:00:25

	1	2	3	4	5	6	7	8	9	10	11	12
A								0.0729	0.0819	0.0927	0.0979	0.1193
B								0.1187	0.1108	0.0843	0.14	0.1335
C								0.1718	0.1759	0.2135	0.2243	0.1882
D								0.1959	0.2106	0.2148	0.2082	0.1953
E								0.1491	0.152	0.1702	0.1627	0.1194
F								0.1804	0.1866	0.1987	0.1913	0.2002
G								0.1016	0.115	0.1146	0.1425	0.0989
H								0.1541	0.1357	0.1959	0.1609	0.1184

Cell Index at: 14:15:26

	1	2	3	4	5	6	7	8	9	10	11	12
A								0.071	0.0835	0.0963	0.0949	0.1221
B								0.119	0.1095	0.0866	0.1372	0.1259
C								0.1794	0.18	0.2106	0.2209	0.1859
D								0.1913	0.2103	0.2115	0.2056	0.2012
E								0.1379	0.1419	0.169	0.1617	0.1169
F								0.1886	0.1922	0.204	0.1986	0.1999
G								0.1026	0.1191	0.1196	0.1436	0.0995
H								0.1581	0.1416	0.1971	0.1639	0.1149

Cell Index at: 14:30:26

	1	2	3	4	5	6	7	8	9	10	11	12
A								0.0701	0.086	0.0913	0.0932	0.1185
B								0.1136	0.1096	0.0891	0.1394	0.1334
C								0.1775	0.1712	0.216	0.2279	0.1859
D								0.1925	0.2094	0.2153	0.2037	0.1991
E								0.1351	0.1495	0.1667	0.1572	0.1227
F								0.1842	0.1943	0.2099	0.1917	0.2033
G								0.0995	0.1135	0.119	0.1403	0.0988
H								0.1572	0.139	0.1954	0.1658	0.1061

Cell Index at: 14:45:27

	1	2	3	4	5	6	7	8	9	10	11	12
A								0.0677	0.0814	0.0962	0.0817	0.1195
B								0.1107	0.1057	0.086	0.1307	0.1313
C								0.1894	0.184	0.2157	0.2171	0.1912
D								0.1875	0.2015	0.214	0.2018	0.197
E								0.1473	0.1544	0.1696	0.1574	0.116
F								0.1843	0.1946	0.2163	0.2005	0.2106
G								0.095	0.1139	0.1174	0.1418	0.0994
H								0.1565	0.1422	0.1999	0.1637	0.107

Cell Index at: 15:00:27

	1	2	3	4	5	6	7	8	9	10	11	12
A								0.0638	0.0836	0.0972	0.0817	0.1177
B								0.1159	0.1009	0.0913	0.1242	0.1313
C								0.1806	0.1788	0.2213	0.2218	0.1887
D								0.1854	0.2154	0.2111	0.2031	0.2075
E								0.1393	0.1544	0.1694	0.1561	0.1152
F								0.1841	0.1947	0.2123	0.1976	0.2109
G								0.1006	0.1147	0.119	0.1458	0.1044
H								0.1571	0.1366	0.1964	0.1643	0.1085

Cell Index at: 15:15:27

	1	2	3	4	5	6	7	8	9	10	11	12
A								0.0622	0.083	0.095	0.0814	0.1188
B								0.1115	0.1	0.0904	0.1279	0.1302
C								0.1929	0.1787	0.2183	0.22	0.1908
D								0.1877	0.2085	0.2149	0.1986	0.2053
E								0.1446	0.1574	0.1759	0.1549	0.1079
F								0.1838	0.1886	0.2128	0.2059	0.205
G								0.0949	0.1185	0.1178	0.1368	0.1073
H								0.1569	0.1412	0.2006	0.1618	0.1099

Cell Index at: 15:30:26

	1	2	3	4	5	6	7	8	9	10	11	12
A								0.0628	0.0864	0.0944	0.0803	0.12
B								0.114	0.1021	0.0862	0.1249	0.1259
C								0.1801	0.1741	0.2172	0.2202	0.188
D								0.1981	0.2087	0.2245	0.2106	0.204
E								0.1456	0.1559	0.1705	0.1582	0.1186
F								0.189	0.1915	0.2157	0.2048	0.2106
G								0.0973	0.1144	0.1227	0.1407	0.1015
H								0.1611	0.1363	0.2007	0.1611	0.1081

Cell Index at: 15:45:26

	1	2	3	4	5	6	7	8	9	10	11	12
A								0.0666	0.0885	0.0961	0.0805	0.1155
B								0.1162	0.1045	0.0909	0.1253	0.1308
C								0.1815	0.1767	0.2156	0.2221	0.1924
D								0.1945	0.2039	0.22	0.1995	0.2026
E								0.1312	0.1472	0.1669	0.1555	0.1143
F								0.1889	0.1909	0.2112	0.2006	0.2063
G								0.0953	0.1158	0.1198	0.1385	0.1012
H								0.1633	0.1368	0.1995	0.1493	0.1113

Cell Index at: 16:00:27

	1	2	3	4	5	6	7	8	9	10	11	12
A								0.0598	0.0839	0.0926	0.0793	0.1178
B								0.1077	0.1001	0.0949	0.1228	0.1336
C								0.1825	0.1827	0.222	0.2228	0.1904
D								0.1889	0.2115	0.2223	0.206	0.2144
E								0.1409	0.1424	0.1751	0.1538	0.1208
F								0.1977	0.1905	0.2088	0.2042	0.2096
G								0.0919	0.1152	0.1132	0.1412	0.0998
H								0.1582	0.1405	0.2017	0.1528	0.1095

Cell Index at: 16:15:28

	1	2	3	4	5	6	7	8	9	10	11	12
A								0.0626	0.082	0.096	0.0786	0.1206
B								0.1055	0.1004	0.091	0.1193	0.1347
C								0.1831	0.1861	0.2201	0.2265	0.1905

D								0.1868	0.2065	0.2123	0.2149	0.2093
E								0.141	0.1481	0.1822	0.1486	0.1241
F								0.1918	0.1949	0.2176	0.2025	0.2148
G								0.0916	0.1174	0.1186	0.1247	0.0996
H								0.1624	0.1395	0.1985	0.1499	0.1118

Cell Index at: 16:30:29

	1	2	3	4	5	6	7	8	9	10	11	12
A								0.0607	0.082	0.0933	0.0776	0.1212
B								0.1051	0.1028	0.0864	0.1191	0.1353
C								0.1869	0.1797	0.2205	0.2254	0.1959
D								0.1944	0.2079	0.2184	0.2084	0.213
E								0.1369	0.1457	0.1689	0.1544	0.1097
F								0.1886	0.1958	0.2205	0.2039	0.2066
G								0.0911	0.1128	0.1187	0.1286	0.0979
H								0.1587	0.1412	0.2024	0.1515	0.1114

Cell Index at: 16:45:20

	1	2	3	4	5	6	7	8	9	10	11	12
A								0.0659	0.0771	0.0963	0.0752	0.1223
B								0.1055	0.1035	0.0877	0.1194	0.1402
C								0.19	0.183	0.2178	0.2276	0.1938
D								0.1937	0.2007	0.2207	0.21	0.2113
E								0.1427	0.1487	0.1699	0.1482	0.1185
F								0.1941	0.1923	0.2146	0.203	0.2102
G								0.0892	0.1177	0.1197	0.1251	0.1014
H								0.1606	0.1427	0.202	0.1533	0.1118

Cell Index at: 17:00:18

	1	2	3	4	5	6	7	8	9	10	11	12
A								0.064	0.0845	0.0961	0.0766	0.1186
B								0.1018	0.105	0.0899	0.1174	0.1262
C								0.1791	0.1752	0.22	0.2339	0.1968
D								0.2047	0.2038	0.2209	0.2041	0.212
E								0.1541	0.1472	0.1721	0.1511	0.1195
F								0.2047	0.1954	0.217	0.2001	0.2094
G								0.0917	0.1107	0.1162	0.1244	0.1051
H								0.1609	0.1384	0.1983	0.1512	0.1129

Cell Index at: 17:15:16

	1	2	3	4	5	6	7	8	9	10	11	12
A								0.0611	0.0791	0.0933	0.0731	0.1233
B								0.1054	0.1023	0.0933	0.1223	0.1265
C								0.1762	0.1821	0.2208	0.229	0.1893
D								0.1905	0.2006	0.215	0.2132	0.2091
E								0.1382	0.1418	0.1671	0.1489	0.1162
F								0.1953	0.1889	0.2106	0.203	0.2149
G								0.0903	0.1134	0.1105	0.1297	0.1048
H								0.159	0.1401	0.2018	0.1501	0.1125

Cell Index at: 17:30:16

	1	2	3	4	5	6	7	8	9	10	11	12
A								0.0628	0.079	0.0893	0.0723	0.1184
B								0.103	0.1073	0.0874	0.1185	0.1287
C								0.1875	0.1855	0.2238	0.229	0.1985
D								0.1945	0.1981	0.2246	0.2099	0.2072
E								0.1459	0.1429	0.1746	0.1449	0.1109
F								0.1978	0.1922	0.2192	0.2061	0.2145
G								0.0903	0.1078	0.1182	0.1289	0.1024
H								0.159	0.1414	0.1975	0.1513	0.1153

Cell Index at: 17:45:17

	1	2	3	4	5	6	7	8	9	10	11	12
A								0.0622	0.0748	0.0791	0.0756	0.1169
B								0.1084	0.1036	0.0889	0.1199	0.1288
C								0.1815	0.1824	0.2258	0.2304	0.1956
D								0.1964	0.1976	0.218	0.2093	0.2079
E								0.1469	0.1384	0.1696	0.1522	0.1141
F								0.1894	0.1923	0.216	0.2076	0.2091
G								0.087	0.1109	0.114	0.1302	0.0964
H								0.1562	0.1376	0.2012	0.15	0.114

Cell Index at: 18:00:17

	1	2	3	4	5	6	7	8	9	10	11	12
A								0.0614	0.0762	0.0807	0.0745	0.1152
B								0.1054	0.1027	0.0897	0.1182	0.1266
C								0.1756	0.1849	0.2108	0.2243	0.1897
D								0.1886	0.2038	0.2164	0.2103	0.2148
E								0.1468	0.1387	0.1771	0.1463	0.1118
F								0.1909	0.1916	0.2066	0.2073	0.2145
G								0.0853	0.1078	0.1179	0.1269	0.0992
H								0.1632	0.1407	0.2014	0.1485	0.1167

Cell Index at: 18:15:18

	1	2	3	4	5	6	7	8	9	10	11	12
A								0.0617	0.0794	0.0782	0.079	0.1161
B								0.105	0.0934	0.0915	0.1245	0.1272
C								0.1917	0.1856	0.2241	0.2298	0.1942
D								0.1957	0.205	0.2186	0.2132	0.2098
E								0.1406	0.1381	0.1774	0.1461	0.1142
F								0.1939	0.1923	0.2125	0.2047	0.2135
G								0.0895	0.1049	0.1142	0.1293	0.0983
H								0.1594	0.1395	0.2	0.1497	0.1163

Cell Index at: 18:30:19

	1	2	3	4	5	6	7	8	9	10	11	12
A								0.0677	0.0757	0.0835	0.0721	0.11
B								0.1056	0.0948	0.094	0.12	0.1265
C								0.1869	0.1802	0.2228	0.2232	0.1936
D								0.1936	0.218	0.2191	0.2171	0.215
E								0.1446	0.1387	0.1788	0.141	0.112
F								0.19	0.2	0.2121	0.2027	0.2109
G								0.0843	0.1057	0.1167	0.1233	0.0972
H								0.1598	0.1406	0.1973	0.1483	0.1128

Cell Index at: 18:45:20

	1	2	3	4	5	6	7	8	9	10	11	12
A								0.062	0.0724	0.0852	0.0718	0.1095
B								0.1056	0.0917	0.0909	0.1198	0.1272
C								0.1843	0.1839	0.2202	0.2282	0.1946
D								0.1825	0.2054	0.2209	0.206	0.212
E								0.1472	0.1363	0.185	0.135	0.1047
F								0.199	0.1944	0.2154	0.2062	0.2102
G								0.0922	0.1116	0.1122	0.1312	0.1005
H								0.1597	0.1393	0.2002	0.1478	0.1153

Cell Index at: 19:00:20

	1	2	3	4	5	6	7	8	9	10	11	12
A								0.0577	0.0747	0.0814	0.0709	0.1097
B								0.0999	0.0932	0.0881	0.1203	0.1185
C								0.1811	0.1808	0.2221	0.2243	0.1909
D								0.181	0.2058	0.2258	0.2093	0.2151
E								0.1367	0.1338	0.1748	0.1484	0.1104
F								0.1967	0.1876	0.2158	0.2086	0.2174
G								0.0863	0.1031	0.1134	0.1266	0.0938
H								0.1586	0.1409	0.2006	0.1527	0.1145

Cell Index at: 19:15:20

	1	2	3	4	5	6	7	8	9	10	11	12
A								0.0591	0.0713	0.0779	0.0705	0.1137
B								0.102	0.0994	0.0885	0.1171	0.1244

C								0.1872	0.1914	0.2293	0.2299	0.1877
D								0.1795	0.2044	0.2219	0.2137	0.2197
E								0.143	0.1405	0.1703	0.1481	0.1086
F								0.1969	0.1924	0.2158	0.2071	0.2195
G								0.0902	0.1048	0.1156	0.1267	0.0962
H								0.1608	0.1413	0.2009	0.1499	0.1165

Cell Index at: 19:30:20

	1	2	3	4	5	6	7	8	9	10	11	12
A								0.0561	0.0709	0.0806	0.0646	0.1134
B								0.1003	0.0952	0.0873	0.1183	0.1264
C								0.1896	0.1873	0.2254	0.2282	0.1958
D								0.1956	0.2041	0.2139	0.203	0.2175
E								0.1482	0.1258	0.168	0.1473	0.1169
F								0.1921	0.1899	0.2147	0.2045	0.2164
G								0.086	0.1008	0.1162	0.1199	0.095
H								0.1642	0.1397	0.1983	0.1491	0.1169

Cell Index at: 19:45:20

	1	2	3	4	5	6	7	8	9	10	11	12
A								0.0555	0.0752	0.0784	0.0629	0.1119
B								0.0985	0.0927	0.09	0.113	0.1287
C								0.1863	0.1903	0.2179	0.2322	0.1926
D								0.1811	0.2091	0.2133	0.2067	0.2148
E								0.1475	0.1296	0.1722	0.1395	0.1103
F								0.1996	0.1936	0.2122	0.211	0.2163
G								0.0883	0.1022	0.1113	0.1239	0.0908
H								0.163	0.1423	0.1974	0.1494	0.1185

Cell Index at: 20:00:21

	1	2	3	4	5	6	7	8	9	10	11	12
A								0.0555	0.0707	0.0865	0.0655	0.113
B								0.1027	0.0884	0.0918	0.1176	0.1257
C								0.1848	0.1879	0.2211	0.2337	0.1997
D								0.191	0.2102	0.214	0.2103	0.2129
E								0.1444	0.1291	0.1721	0.1439	0.1129
F								0.1943	0.1908	0.2189	0.2077	0.2137
G								0.0874	0.0984	0.1147	0.1256	0.096
H								0.1656	0.1399	0.1994	0.1524	0.1186

Cell Index at: 20:15:21

	1	2	3	4	5	6	7	8	9	10	11	12
A								0.0547	0.0733	0.0871	0.0668	0.1165
B								0.1008	0.0945	0.0901	0.1151	0.1243
C								0.1989	0.1871	0.2238	0.2319	0.1931
D								0.1782	0.2109	0.2113	0.2086	0.2143
E								0.1446	0.1358	0.1692	0.1421	0.116
F								0.1983	0.1969	0.2178	0.1891	0.2162
G								0.0869	0.0976	0.1125	0.1225	0.0949
H								0.1624	0.1454	0.192	0.1515	0.1179

Cell Index at: 20:30:22

	1	2	3	4	5	6	7	8	9	10	11	12
A								0.0544	0.0698	0.0809	0.0656	0.1103
B								0.0956	0.0915	0.0891	0.115	0.1292
C								0.1791	0.1878	0.2205	0.2329	0.1953
D								0.1861	0.2097	0.2138	0.2058	0.2107
E								0.1495	0.1284	0.176	0.136	0.1066
F								0.1951	0.1967	0.2229	0.1927	0.2138
G								0.0857	0.0959	0.0988	0.1262	0.0985
H								0.167	0.141	0.1895	0.1547	0.1155

Cell Index at: 20:45:22

	1	2	3	4	5	6	7	8	9	10	11	12
A								0.0525	0.069	0.0857	0.0684	0.11
B								0.1042	0.0855	0.0857	0.1147	0.1271
C								0.1773	0.1901	0.2273	0.2364	0.1983
D								0.1853	0.214	0.2103	0.2136	0.2105
E								0.1419	0.1291	0.1735	0.1476	0.1096
F								0.1933	0.1957	0.2192	0.1921	0.2186
G								0.0863	0.1027	0.0983	0.1226	0.0919
H								0.1677	0.1407	0.1903	0.1505	0.1165

Cell Index at: 21:00:22

	1	2	3	4	5	6	7	8	9	10	11	12
A								0.0504	0.0695	0.082	0.0652	0.1097
B								0.0967	0.0915	0.0808	0.1088	0.1286
C								0.1857	0.1891	0.228	0.2369	0.1975
D								0.1868	0.2114	0.22	0.2091	0.2188
E								0.1419	0.1358	0.1614	0.1507	0.1111
F								0.1984	0.196	0.221	0.1957	0.2156
G								0.0917	0.1017	0.1008	0.1251	0.0972
H								0.1679	0.1469	0.1925	0.1542	0.1136

Cell Index at: 21:15:23

	1	2	3	4	5	6	7	8	9	10	11	12
A								0.0541	0.0649	0.0798	0.0662	0.1095
B								0.0971	0.0893	0.0813	0.1089	0.1218
C								0.1855	0.1858	0.2228	0.2411	0.1938
D								0.1776	0.2077	0.2113	0.212	0.2167
E								0.1406	0.1249	0.1678	0.1374	0.1152
F								0.2007	0.1944	0.2209	0.1951	0.2204
G								0.0887	0.1017	0.1005	0.1248	0.0918
H								0.1682	0.145	0.1929	0.1532	0.1152

Cell Index at: 21:30:23

	1	2	3	4	5	6	7	8	9	10	11	12
A								0.0498	0.0628	0.0707	0.0675	0.1046
B								0.0929	0.0894	0.0829	0.1032	0.1216
C								0.1921	0.1822	0.2304	0.2313	0.1925
D								0.1773	0.216	0.2116	0.2095	0.2106
E								0.1362	0.1397	0.1663	0.1408	0.1124
F								0.2004	0.1933	0.2234	0.1971	0.217
G								0.0877	0.0998	0.0981	0.1247	0.089
H								0.1651	0.1457	0.1974	0.1538	0.1152

Cell Index at: 21:45:24

	1	2	3	4	5	6	7	8	9	10	11	12
A								0.05	0.0643	0.073	0.0689	0.1043
B								0.0963	0.0918	0.087	0.109	0.1193
C								0.188	0.1858	0.2296	0.233	0.1959
D								0.1869	0.2111	0.211	0.2114	0.2149
E								0.1439	0.1313	0.1661	0.1484	0.1027
F								0.197	0.192	0.2177	0.1888	0.2198
G								0.0854	0.1008	0.0989	0.1203	0.0923
H								0.169	0.1443	0.1995	0.1562	0.1127

Cell Index at: 22:00:24

	1	2	3	4	5	6	7	8	9	10	11	12
A								0.0471	0.0652	0.0709	0.0637	0.1061
B								0.0912	0.0861	0.0796	0.1081	0.1235
C								0.1836	0.1853	0.2312	0.2318	0.1975
D								0.184	0.2142	0.2113	0.2101	0.2166
E								0.1395	0.1313	0.1653	0.1421	0.1134
F								0.1981	0.1951	0.2172	0.1963	0.2224
G								0.0879	0.1005	0.0965	0.1211	0.0883
H								0.1669	0.1426	0.1934	0.1554	0.1167

Cell Index at: 22:15:25

	1	2	3	4	5	6	7	8	9	10	11	12
A								0.0481	0.0628	0.0723	0.0629	0.1021

	1	2	3	4	5	6	7	8	9	10	11	12
B								0.0935	0.085	0.0802	0.1106	0.1251
C								0.1899	0.1844	0.2321	0.2361	0.1944
D								0.1898	0.2095	0.218	0.2098	0.2175
E								0.1399	0.1365	0.1621	0.1369	0.1156
F								0.2042	0.1893	0.2145	0.1951	0.2218
G								0.0878	0.0968	0.0968	0.1209	0.0866
H								0.1663	0.139	0.198	0.1529	0.1155

Cell Index at: 22:30:26

	1	2	3	4	5	6	7	8	9	10	11	12
A								0.0467	0.0618	0.0734	0.0578	0.1042
B								0.0938	0.0837	0.0768	0.1001	0.1168
C								0.1905	0.1829	0.2305	0.2418	0.1881
D								0.1838	0.2252	0.2161	0.2145	0.2134
E								0.1293	0.1334	0.1729	0.1405	0.1166
F								0.2006	0.1947	0.2168	0.195	0.2162
G								0.0832	0.0967	0.0957	0.12	0.0843
H								0.1646	0.1379	0.1933	0.1505	0.1137

Cell Index at: 22:45:27

	1	2	3	4	5	6	7	8	9	10	11	12
A								0.0451	0.0606	0.0748	0.0583	0.1011
B								0.0962	0.0785	0.0805	0.1043	0.1188
C								0.1874	0.1861	0.2277	0.2429	0.1977
D								0.1854	0.2159	0.2163	0.2098	0.212
E								0.1253	0.128	0.1658	0.1325	0.1121
F								0.1998	0.1938	0.2148	0.1941	0.2175
G								0.0835	0.0976	0.0952	0.1187	0.0846
H								0.1705	0.1396	0.1938	0.1555	0.1164

Cell Index at: 23:00:27

	1	2	3	4	5	6	7	8	9	10	11	12
A								0.0486	0.0595	0.0737	0.0592	0.1023
B								0.0941	0.0847	0.0793	0.1061	0.1154
C								0.1894	0.1875	0.2256	0.234	0.1967
D								0.1887	0.2198	0.2158	0.2045	0.2109
E								0.1331	0.1281	0.1664	0.1265	0.1096
F								0.1995	0.202	0.2218	0.1993	0.2118
G								0.084	0.0973	0.0951	0.1151	0.086
H								0.1662	0.138	0.1943	0.1535	0.1138

Cell Index at: 23:15:27

	1	2	3	4	5	6	7	8	9	10	11	12
A								0.0471	0.058	0.0725	0.0588	0.1004
B								0.0888	0.086	0.083	0.106	0.1197
C								0.1937	0.1866	0.2314	0.2436	0.1973
D								0.182	0.2063	0.2138	0.2088	0.2112
E								0.146	0.1304	0.166	0.1329	0.1088
F								0.1993	0.1966	0.2198	0.1934	0.2142
G								0.0849	0.0968	0.097	0.1162	0.0856
H								0.168	0.1291	0.1927	0.1492	0.1103

Cell Index at: 23:30:28

	1	2	3	4	5	6	7	8	9	10	11	12
A								0.0468	0.0527	0.0747	0.058	0.0988
B								0.0945	0.0836	0.0767	0.1082	0.1164
C								0.1893	0.1877	0.2297	0.2452	0.1943
D								0.1834	0.2146	0.2103	0.2191	0.2158
E								0.1378	0.1311	0.1592	0.1386	0.1061
F								0.2014	0.192	0.2197	0.1894	0.218
G								0.0828	0.0944	0.0939	0.1136	0.0837
H								0.1642	0.1345	0.1911	0.151	0.1141

Cell Index at: 23:45:28

	1	2	3	4	5	6	7	8	9	10	11	12
A								0.0482	0.0542	0.0719	0.0567	0.0923
B								0.0885	0.0857	0.0787	0.1027	0.1193
C								0.1935	0.1864	0.2355	0.2428	0.1931
D								0.1849	0.2104	0.2171	0.214	0.2101
E								0.1318	0.1189	0.1629	0.137	0.1042
F								0.2097	0.1954	0.2212	0.1946	0.2149
G								0.0791	0.0927	0.0924	0.1156	0.0818
H								0.1667	0.1339	0.1942	0.1513	0.1137

Cell Index at: 24:00:29

	1	2	3	4	5	6	7	8	9	10	11	12
A								0.0464	0.0515	0.0743	0.0528	0.0953
B								0.0942	0.0813	0.0787	0.1042	0.1139
C								0.1942	0.1857	0.2289	0.2477	0.1976
D								0.1815	0.2159	0.2095	0.2135	0.2046
E								0.1361	0.1239	0.1637	0.1388	0.1131
F								0.1986	0.1955	0.216	0.1933	0.2128
G								0.0756	0.0918	0.0966	0.1138	0.0788
H								0.1687	0.1352	0.1916	0.1512	0.1146

A vs B	0.030214	B vs C	0.000101	C vs D	0.676655	D vs E	0.00024	E vs F	0.000355	F vs G	2.68E-06	G vs H	0.006549
A vs C	1.43E-05	B vs D	2.07E-06	C vs E	0.001088	D vs F	0.826246	E vs G	0.004231	F vs H	0.01535		
A vs D	4.13E-06	B vs E	0.006095	C vs F	0.574245	D vs G	1.91E-06	E vs H	0.313948				
A vs E	0.000469	B vs F	2.61E-06	C vs G	8.22E-05	D vs H	0.012582						
A vs F	1.08E-05	B vs G	0.751259	C vs H	0.011078								
A vs G	0.046671	B vs H	0.008362										
A vs H	0.000894												

Cell Index at: 24:15:28

	1	2	3	4	5	6	7	8	9	10	11	12
A								0.0449	0.0542	0.0723	0.0556	0.0925
B								0.0887	0.0831	0.0814	0.1071	0.1099
C								0.1853	0.184	0.2309	0.2494	0.1963
D								0.1858	0.208	0.2201	0.2136	0.213
E								0.1344	0.1201	0.1527	0.1326	0.1058
F								0.1946	0.1987	0.2212	0.1933	0.2163
G								0.0783	0.0922	0.0908	0.1138	0.0849
H								0.1634	0.1349	0.1908	0.1524	0.1145

Count	*Sum*	*Average*	*Variance*
5	0.3203	0.06406	0.000419
5	0.4723	0.09446	0.000224
5	1.0541	0.21082	0.000692
5	1.025	0.205	0.000191
5	0.6756	0.13512	0.000361
5	1.0162	0.20324	0.000109
5	0.4566	0.09132	0.000234
5	0.7613	0.15226	0.000882

Cell Index at: 24:30:29

	1	2	3	4	5	6	7	8	9	10	11	12
A								0.0473	0.0524	0.0736	0.0567	0.0917
B								0.0873	0.0798	0.0803	0.1082	0.1085
C								0.1891	0.1923	0.225	0.2449	0.1957
D								0.1783	0.2138	0.2195	0.2161	0.2161
E								0.1288	0.1274	0.1534	0.1391	0.1065
F								0.1968	0.1931	0.2157	0.1955	0.2141
G								0.0751	0.093	0.0904	0.1114	0.077
H								0.1633	0.1363	0.1923	0.1532	0.1152

SS	*df*	*MS*	*F*	*P-value*	*F crit*
0.117299	7	0.016757	43.08214	1.62E-14	2.312741
0.012447	32	0.000389			
0.129746	39				

Cell Index at: 24:45:30

	1	2	3	4	5	6	7	8	9	10	11	12
A								0.0448	0.0543	0.0736	0.0552	0.0896
B								0.0839	0.0767	0.0827	0.1064	0.1059
C								0.1896	0.1918	0.2316	0.2402	0.1942
D								0.1774	0.2144	0.2242	0.2091	0.2176
E								0.1349	0.1231	0.1471	0.1331	0.1108
F								0.2067	0.188	0.219	0.1897	0.2174
G								0.0701	0.0948	0.0936	0.1129	0.0775
H								0.1642	0.1277	0.1901	0.151	0.1123

Cell Index at: 25:00:30

	1	2	3	4	5	6	7	8	9	10	11	12
A								0.0412	0.0535	0.0697	0.0538	0.0921
B								0.0889	0.0775	0.0821	0.1017	0.1141
C								0.1853	0.1905	0.2312	0.2392	0.1945
D								0.1765	0.2156	0.2168	0.2137	0.2156
E								0.1377	0.1274	0.1442	0.1277	0.1087
F								0.1999	0.1879	0.2166	0.1962	0.2087
G								0.0737	0.0933	0.0918	0.1135	0.0777
H								0.1653	0.123	0.1925	0.1562	0.1124

Cell Index at: 25:15:31

	1	2	3	4	5	6	7	8	9	10	11	12
A								0.0439	0.0565	0.0678	0.0515	0.0889
B								0.0876	0.0774	0.0779	0.1004	0.1081
C								0.1887	0.1892	0.2309	0.2464	0.1989
D								0.1855	0.2097	0.2192	0.2096	0.2146
E								0.1327	0.1338	0.1435	0.1353	0.1068
F								0.2083	0.1924	0.2213	0.1955	0.2146
G								0.07	0.0941	0.0867	0.1007	0.0714
H								0.1653	0.1228	0.1903	0.1553	0.1088

Cell Index at: 25:30:31

	1	2	3	4	5	6	7	8	9	10	11	12
A								0.0414	0.0568	0.0673	0.0556	0.0899
B								0.0881	0.0771	0.0841	0.101	0.1085
C								0.1867	0.1841	0.2388	0.2439	0.1944
D								0.1808	0.2164	0.2142	0.2184	0.216
E								0.1376	0.1276	0.1474	0.1339	0.1086
F								0.1985	0.1907	0.2188	0.1952	0.2117
G								0.0688	0.0882	0.0886	0.1029	0.0782
H								0.164	0.1259	0.1944	0.1544	0.1073

Cell Index at: 25:45:32

	1	2	3	4	5	6	7	8	9	10	11	12
A								0.0434	0.0562	0.0665	0.0537	0.085
B								0.0905	0.0765	0.0864	0.0961	0.1082
C								0.1881	0.1881	0.2352	0.2386	0.1916
D								0.1813	0.2163	0.222	0.2143	0.2114
E								0.132	0.1325	0.1453	0.1325	0.1032
F								0.1945	0.1982	0.2208	0.1977	0.2152
G								0.0705	0.094	0.0878	0.0959	0.0731
H								0.1625	0.1218	0.1893	0.1553	0.1077

Cell Index at: 26:00:31

	1	2	3	4	5	6	7	8	9	10	11	12
A								0.0399	0.0595	0.0687	0.0528	0.0845
B								0.0898	0.0806	0.0808	0.1021	0.1114
C								0.1895	0.1868	0.2317	0.2442	0.1944
D								0.191	0.2149	0.2118	0.2152	0.2195
E								0.1312	0.1277	0.1453	0.1332	0.1037
F								0.2035	0.1961	0.2242	0.1962	0.2109
G								0.0713	0.0938	0.0908	0.0976	0.0743
H								0.1648	0.1228	0.1951	0.149	0.1061

Cell Index at: 26:15:32

	1	2	3	4	5	6	7	8	9	10	11	12
A								0.0377	0.0579	0.0695	0.0531	0.0838
B								0.0881	0.0795	0.0791	0.0971	0.1101
C								0.1933	0.1884	0.2295	0.2397	0.1974
D								0.177	0.2169	0.2168	0.2169	0.2213
E								0.1311	0.1349	0.1431	0.1269	0.1107
F								0.2067	0.2023	0.2147	0.1941	0.2137
G								0.0761	0.0898	0.0888	0.1006	0.0759
H								0.1618	0.1178	0.1938	0.1488	0.1061

Cell Index at: 26:30:32

	1	2	3	4	5	6	7	8	9	10	11	12
A								0.0336	0.0574	0.0662	0.0555	0.0816
B								0.0901	0.0833	0.0797	0.0953	0.1021
C								0.1921	0.1904	0.2299	0.2356	0.1929
D								0.1742	0.2121	0.2195	0.2116	0.211
E								0.134	0.1314	0.1446	0.1312	0.0995
F								0.2019	0.1907	0.2232	0.1974	0.2224
G								0.075	0.0921	0.0853	0.0968	0.0696
H								0.1647	0.1226	0.1922	0.1517	0.1026

Cell Index at: 26:45:33

	1	2	3	4	5	6	7	8	9	10	11	12
A								0.0302	0.0556	0.0647	0.0559	0.0832
B								0.0881	0.0807	0.0797	0.0949	0.1056
C								0.1952	0.1882	0.2288	0.2379	0.1914
D								0.1783	0.216	0.217	0.2168	0.2125
E								0.1329	0.1159	0.1359	0.1319	0.1054
F								0.1998	0.1867	0.2188	0.1944	0.2139
G								0.0707	0.0861	0.0871	0.1008	0.0742
H								0.1603	0.1179	0.1883	0.1516	0.1061

Cell Index at: 27:00:34

	1	2	3	4	5	6	7	8	9	10	11	12
A								0.0315	0.0543	0.0662	0.0523	0.0837
B								0.092	0.0765	0.0823	0.0908	0.1006
C								0.1967	0.1874	0.232	0.2404	0.197
D								0.1759	0.2101	0.2299	0.2243	0.209
E								0.1324	0.1261	0.1347	0.135	0.0988
F								0.1992	0.1908	0.2285	0.1952	0.2202
G								0.0661	0.0902	0.0884	0.1031	0.0715
H								0.1624	0.1181	0.1912	0.1525	0.1035

Cell Index at: 27:15:34

	1	2	3	4	5	6	7	8	9	10	11	12
A								0.0304	0.0501	0.0629	0.054	0.0802
B								0.0865	0.0794	0.079	0.0904	0.1006
C								0.1895	0.1913	0.2293	0.2393	0.1901
D								0.1825	0.2132	0.2194	0.2158	0.2146
E								0.1236	0.1234	0.1399	0.1254	0.104
F								0.1913	0.1902	0.2244	0.1921	0.2216
G								0.0713	0.0886	0.0855	0.1026	0.0698
H								0.1627	0.1146	0.1918	0.1551	0.1051

Cell Index at: 27:30:35

	1	2	3	4	5	6	7	8	9	10	11	12
A								0.0262	0.0511	0.0606	0.0526	0.0775
B								0.0853	0.0766	0.0784	0.0908	0.0977
C								0.1935	0.1915	0.231	0.2424	0.193
D								0.1758	0.2182	0.2195	0.2206	0.2147
E								0.1193	0.1167	0.136	0.1291	0.1018
F								0.1975	0.187	0.226	0.1973	0.2158
G								0.0698	0.0862	0.0869	0.1004	0.0716
H								0.1631	0.1191	0.1918	0.1517	0.1055

Cell Index at: 27:45:34

	1	2	3	4	5	6	7	8	9	10	11	12
A								0.0287	0.0461	0.0579	0.0512	0.0803
B								0.0756	0.0743	0.0802	0.0901	0.1014
C								0.1985	0.1888	0.2242	0.2425	0.1937
D								0.1775	0.2182	0.2228	0.2209	0.2007
E								0.1257	0.1175	0.1413	0.1271	0.1064
F								0.1995	0.1904	0.2219	0.1963	0.2137
G								0.0758	0.0875	0.0888	0.102	0.0701
H								0.1605	0.1179	0.1919	0.1538	0.1015

Cell Index at: 28:00:35

	1	2	3	4	5	6	7	8	9	10	11	12
A								0.0274	0.0486	0.0606	0.048	0.0769
B								0.0744	0.0771	0.0809	0.0889	0.1031
C								0.2041	0.1884	0.2234	0.246	0.1929
D								0.1738	0.2226	0.221	0.2133	0.2071
E								0.1213	0.1127	0.1443	0.1267	0.1026
F								0.2	0.1935	0.2231	0.1924	0.217
G								0.0682	0.0863	0.0898	0.1061	0.0701
H								0.1643	0.1187	0.1931	0.1563	0.1034

Cell Index at: 28:15:35

	1	2	3	4	5	6	7	8	9	10	11	12
A								0.0313	0.0464	0.0618	0.0519	0.0804
B								0.0796	0.0757	0.0804	0.0861	0.1031
C								0.1953	0.1903	0.2214	0.243	0.1952
D								0.179	0.2091	0.2239	0.2153	0.2088
E								0.1288	0.1158	0.1358	0.1215	0.0898
F								0.1957	0.1891	0.2275	0.1935	0.2128
G								0.0636	0.0844	0.0858	0.1036	0.0676
H								0.1639	0.1209	0.1906	0.1528	0.1027

Cell Index at: 28:30:36

	1	2	3	4	5	6	7	8	9	10	11	12
A								0.031	0.0411	0.0588	0.0483	0.0783
B								0.0795	0.0707	0.079	0.0844	0.1046
C								0.1978	0.1912	0.222	0.2443	0.1981
D								0.179	0.2026	0.227	0.2143	0.2094
E								0.1189	0.126	0.1377	0.1217	0.0993
F								0.1991	0.1916	0.2275	0.1923	0.2147
G								0.071	0.0825	0.0864	0.1032	0.0711
H								0.1617	0.1182	0.1917	0.1513	0.1018

Cell Index at: 28:45:36

	1	2	3	4	5	6	7	8	9	10	11	12
A								0.0256	0.0438	0.0546	0.0498	0.0782
B								0.0805	0.0716	0.0792	0.088	0.1035
C								0.1926	0.1986	0.2217	0.2475	0.1991
D								0.1768	0.2186	0.2124	0.2162	0.2071
E								0.1178	0.1213	0.134	0.1308	0.0963
F								0.1976	0.1899	0.2233	0.1935	0.2123
G								0.0664	0.0822	0.0818	0.1028	0.0671
H								0.1631	0.1179	0.1887	0.154	0.0987

Cell Index at: 29:00:36

	1	2	3	4	5	6	7	8	9	10	11	12
A								0.0265	0.0454	0.0582	0.0461	0.0743
B								0.0843	0.0761	0.079	0.0842	0.1035
C								0.1981	0.1945	0.2131	0.245	0.1965
D								0.1853	0.2142	0.2199	0.2226	0.2043
E								0.1097	0.1195	0.1272	0.1217	0.091
F								0.1938	0.189	0.2266	0.1908	0.2145
G								0.0644	0.0835	0.0805	0.1018	0.0716
H								0.164	0.1196	0.1935	0.1534	0.1024

Cell Index at: 29:15:36

	1	2	3	4	5	6	7	8	9	10	11	12
A								0.0278	0.0451	0.055	0.0473	0.0704
B								0.0767	0.0697	0.0739	0.0835	0.1039
C								0.1873	0.1883	0.2112	0.2491	0.1969
D								0.1828	0.2162	0.2232	0.2163	0.2081
E								0.1167	0.1241	0.1355	0.1305	0.0947
F								0.1993	0.1899	0.2315	0.1928	0.2134
G								0.0636	0.0831	0.0826	0.0962	0.0706
H								0.1657	0.1183	0.1899	0.1535	0.0995

Cell Index at: 29:30:36

	1	2	3	4	5	6	7	8	9	10	11	12
A								0.0283	0.042	0.0557	0.0454	0.0727
B								0.0768	0.0738	0.0754	0.0859	0.1038
C								0.1932	0.1897	0.2111	0.2365	0.1881
D								0.1852	0.2177	0.221	0.2147	0.1939
E								0.114	0.1192	0.1375	0.1259	0.0898
F								0.1957	0.1885	0.234	0.1928	0.2167
G								0.0665	0.0845	0.0851	0.1044	0.071
H								0.162	0.1189	0.1921	0.1557	0.1007

Cell Index at: 29:45:37

	1	2	3	4	5	6	7	8	9	10	11	12
A								0.0256	0.0408	0.0538	0.0458	0.0713
B								0.0782	0.0704	0.0779	0.0895	0.0997
C								0.1902	0.1947	0.2078	0.244	0.1974
D								0.1859	0.2208	0.2326	0.2146	0.2068
E								0.1235	0.1188	0.136	0.1217	0.0894
F								0.2065	0.1866	0.2227	0.1955	0.2121
G								0.0628	0.0846	0.0814	0.1	0.0692
H								0.1633	0.115	0.1889	0.1497	0.103

Cell Index at: 30:00:36

	1	2	3	4	5	6	7	8	9	10	11	12
A								0.022	0.0402	0.0538	0.0435	0.0692
B								0.0813	0.0682	0.0785	0.0816	0.0977
C								0.1948	0.1961	0.2163	0.2442	0.1956
D								0.1827	0.2252	0.2218	0.2122	0.2116
E								0.1195	0.1217	0.1417	0.1186	0.099
F								0.1993	0.1878	0.2301	0.1855	0.2181
G								0.0595	0.0868	0.0762	0.0976	0.0737
H								0.1638	0.1171	0.1922	0.1539	0.1047

Cell Index at: 30:15:37

	1	2	3	4	5	6	7	8	9	10	11	12
A								0.0221	0.0413	0.0567	0.0461	0.0704
B								0.0756	0.0686	0.0776	0.0825	0.0977
C								0.1973	0.1985	0.2089	0.2524	0.201
D								0.1799	0.2163	0.2235	0.2168	0.2024
E								0.1173	0.1215	0.128	0.118	0.0897
F								0.1954	0.1904	0.231	0.1959	0.218
G								0.0644	0.0816	0.0825	0.0943	0.0695
H								0.1629	0.1167	0.1878	0.1495	0.1071

Cell Index at: 30:30:36

	1	2	3	4	5	6	7	8	9	10	11	12
A								0.017	0.0404	0.0554	0.0482	0.0716
B								0.0743	0.071	0.0736	0.0811	0.1005
C								0.1917	0.1943	0.2133	0.2481	0.1991
D								0.1722	0.2184	0.2155	0.2139	0.1976
E								0.1225	0.1162	0.139	0.1168	0.0976
F								0.198	0.1843	0.2248	0.1922	0.2114
G								0.0625	0.0833	0.082	0.0961	0.0699
H								0.1608	0.1148	0.1771	0.1526	0.1048

Cell Index at: 30:45:36

	1	2	3	4	5	6	7	8	9	10	11	12
A								0.0156	0.0374	0.0507	0.0437	0.0695
B								0.0693	0.0669	0.0727	0.0826	0.0954
C								0.197	0.195	0.2101	0.2476	0.1965
D								0.1736	0.2267	0.2231	0.2095	0.2095
E								0.115	0.1112	0.1384	0.1259	0.0887
F								0.1866	0.1894	0.2231	0.1949	0.2114
G								0.0589	0.0851	0.0796	0.1002	0.0656
H								0.1645	0.1121	0.1783	0.1528	0.1029

Cell Index at: 31:00:37

	1	2	3	4	5	6	7	8	9	10	11	12
A								0.0182	0.0353	0.0556	0.0434	0.0625
B								0.0793	0.0674	0.0769	0.0788	0.0917
C								0.189	0.1915	0.2149	0.2509	0.193
D								0.1757	0.22	0.2326	0.2122	0.1904
E								0.1177	0.1184	0.1384	0.1224	0.0949
F								0.1921	0.1908	0.2335	0.1988	0.2078
G								0.0573	0.0833	0.0802	0.0973	0.063
H								0.1631	0.1163	0.1757	0.1557	0.0997

Cell Index at: 31:15:37

	1	2	3	4	5	6	7	8	9	10	11	12
A								0.0178	0.0396	0.0511	0.043	0.0598
B								0.07	0.063	0.0779	0.0763	0.0879
C								0.1959	0.1893	0.2122	0.2485	0.193
D								0.1783	0.2123	0.2276	0.2076	0.2012
E								0.115	0.1078	0.1324	0.1217	0.096
F								0.1954	0.1897	0.2271	0.2003	0.207
G								0.0589	0.0796	0.0834	0.0909	0.0631
H								0.1575	0.1143	0.1761	0.1528	0.0968

Cell Index at: 31:30:38

	1	2	3	4	5	6	7	8	9	10	11	12
A								0.0159	0.0378	0.0464	0.0426	0.0631
B								0.0721	0.0652	0.0729	0.0792	0.0898
C								0.1903	0.1923	0.2137	0.249	0.1956
D								0.1755	0.2133	0.2223	0.2158	0.2031
E								0.1112	0.1131	0.1337	0.1228	0.0911
F								0.1971	0.1873	0.2311	0.2012	0.2122
G								0.0581	0.0817	0.0808	0.0924	0.0574
H								0.1589	0.1143	0.1756	0.1541	0.098

Cell Index at: 31:45:38

	1	2	3	4	5	6	7	8	9	10	11	12
A								0.0172	0.0374	0.0469	0.0407	0.0618
B								0.073	0.0583	0.0711	0.0783	0.0841
C								0.1951	0.1977	0.2173	0.2536	0.1961
D								0.1749	0.2217	0.2279	0.2097	0.2104
E								0.1116	0.1208	0.1352	0.1291	0.0953
F								0.1962	0.1973	0.2225	0.1985	0.2054
G								0.055	0.0771	0.081	0.0942	0.0604
H								0.161	0.1162	0.1746	0.1526	0.0982

Cell Index at: 32:00:39

	1	2	3	4	5	6	7	8	9	10	11	12
A								0.0138	0.0377	0.0485	0.0392	0.0582
B								0.068	0.0657	0.071	0.0758	0.0907
C								0.1929	0.1935	0.2111	0.2491	0.1981
D								0.1743	0.2207	0.2232	0.2171	0.2016
E								0.1099	0.111	0.1383	0.1226	0.092
F								0.1996	0.1883	0.2269	0.196	0.2097
G								0.0526	0.0798	0.0796	0.0956	0.0594
H								0.1577	0.1199	0.1746	0.1526	0.0981

Cell Index at: 32:15:38

	1	2	3	4	5	6	7	8	9	10	11	12
A								0.0137	0.0322	0.0463	0.0407	0.0619
B								0.0735	0.0647	0.0689	0.0786	0.0874
C								0.1972	0.1936	0.2135	0.2583	0.1947
D								0.1735	0.2137	0.2316	0.2173	0.2068
E								0.106	0.1142	0.1334	0.1217	0.0867
F								0.193	0.187	0.2271	0.1973	0.2093
G								0.0535	0.076	0.081	0.0904	0.0584
H								0.1616	0.1153	0.1787	0.1535	0.0989

Cell Index at: 32:30:39

	1	2	3	4	5	6	7	8	9	10	11	12
A								0.0116	0.033	0.0499	0.0375	0.0613
B								0.0704	0.0683	0.0668	0.0772	0.0842
C								0.1907	0.1993	0.2182	0.2498	0.1919
D								0.1751	0.2169	0.224	0.2186	0.2131
E								0.1146	0.1134	0.1268	0.1163	0.0951
F								0.1962	0.1911	0.2289	0.1988	0.2086
G								0.0493	0.0772	0.0814	0.0895	0.063
H								0.1627	0.1151	0.1779	0.1525	0.1005

Cell Index at: 32:45:38

	1	2	3	4	5	6	7	8	9	10	11	12
A								0.0174	0.0325	0.0473	0.0384	0.0561
B								0.0661	0.0642	0.0692	0.079	0.0863
C								0.1985	0.2008	0.2208	0.2501	0.1981
D								0.1782	0.2139	0.2267	0.2181	0.1933
E								0.1154	0.1197	0.1295	0.123	0.0845
F								0.2007	0.1879	0.2248	0.2018	0.2027
G								0.0529	0.0796	0.0739	0.0925	0.0647
H								0.1597	0.1158	0.179	0.1528	0.0988

Cell Index at: 33:00:38

	1	2	3	4	5	6	7	8	9	10	11	12
A								0.015	0.0324	0.0452	0.0384	0.0576
B								0.062	0.067	0.0661	0.0795	0.0811
C								0.1945	0.202	0.2143	0.2534	0.1942
D								0.1811	0.2159	0.231	0.2138	0.2017
E								0.1078	0.1157	0.1288	0.1142	0.0828
F								0.1933	0.1914	0.2237	0.1975	0.2075
G								0.0505	0.0714	0.0788	0.0938	0.059
H								0.1593	0.1159	0.1787	0.1559	0.0983

Cell Index at: 33:15:39

	1	2	3	4	5	6	7	8	9	10	11	12
A								0.0164	0.0353	0.0443	0.0368	0.059
B								0.0588	0.0642	0.0675	0.0758	0.081
C								0.1949	0.1938	0.2187	0.2464	0.1989
D								0.1756	0.2236	0.2325	0.2081	0.2038
E								0.1023	0.1127	0.1302	0.1215	0.0761
F								0.1919	0.1933	0.2265	0.1963	0.2042
G								0.0525	0.0707	0.0738	0.0944	0.0545
H								0.1576	0.1126	0.1782	0.1535	0.1003

Cell Index at: 33:30:39

	1	2	3	4	5	6	7	8	9	10	11	12
A								0.0153	0.0321	0.0442	0.0358	0.0565
B								0.0661	0.0644	0.0608	0.0777	0.0819
C								0.1995	0.1949	0.2149	0.2518	0.2005
D								0.1763	0.215	0.2341	0.2125	0.2082
E								0.0966	0.1112	0.1253	0.1146	0.0718
F								0.1942	0.1871	0.2326	0.204	0.2019
G								0.0489	0.072	0.0765	0.0908	0.0561
H								0.1599	0.1144	0.1771	0.1522	0.1002

Cell Index at: 33:45:40

	1	2	3	4	5	6	7	8	9	10	11	12
A								0.0113	0.0308	0.0389	0.0361	0.0567
B								0.0667	0.0641	0.0675	0.0714	0.0843
C								0.2	0.1987	0.2129	0.2474	0.1919
D								0.1816	0.2216	0.2306	0.2189	0.2057
E								0.1003	0.1087	0.1275	0.1158	0.0828
F								0.1983	0.1909	0.2307	0.1998	0.2116
G								0.0503	0.0707	0.0698	0.09	0.0581
H								0.159	0.1174	0.1789	0.1548	0.1008

Cell Index at: 34:00:39

	1	2	3	4	5	6	7	8	9	10	11	12
A								0.0112	0.0332	0.0403	0.0355	0.0557
B								0.0625	0.063	0.0627	0.0705	0.082
C								0.1994	0.2012	0.2181	0.2489	0.1923
D								0.1927	0.2179	0.226	0.2171	0.2104
E								0.1068	0.1088	0.1242	0.1086	0.08
F								0.1963	0.1926	0.2267	0.1974	0.2085
G								0.0494	0.0708	0.0694	0.0901	0.0543

	1	2	3	4	5	6	7	8	9	10	11	12
H								0.1564	0.1159	0.1809	0.1501	0.0999

Cell Index at: 34:15:40

	1	2	3	4	5	6	7	8	9	10	11	12
A								0.0119	0.0329	0.0406	0.036	0.0528
B								0.0626	0.0629	0.0637	0.071	0.0803
C								0.1919	0.1999	0.2197	0.2562	0.1896
D								0.1898	0.2194	0.2408	0.2134	0.2061
E								0.1023	0.1123	0.1193	0.1158	0.0844
F								0.1981	0.1911	0.2306	0.2033	0.2032
G								0.051	0.0704	0.0717	0.0906	0.0531
H								0.1545	0.1139	0.1807	0.1525	0.097

Cell Index at: 34:30:40

	1	2	3	4	5	6	7	8	9	10	11	12
A								0.0098	0.0344	0.0361	0.0359	0.0533
B								0.0643	0.0596	0.0671	0.0681	0.0785
C								0.1971	0.2032	0.2158	0.2585	0.1948
D								0.1793	0.2193	0.2317	0.2108	0.2104
E								0.094	0.1114	0.1302	0.1129	0.0871
F								0.1996	0.1896	0.2373	0.1963	0.2092
G								0.0484	0.0713	0.0747	0.0894	0.0555
H								0.1609	0.1133	0.1791	0.1495	0.0965

Cell Index at: 34:45:40

	1	2	3	4	5	6	7	8	9	10	11	12
A								0.0097	0.0291	0.0347	0.0347	0.0523
B								0.0604	0.0606	0.0646	0.0699	0.0809
C								0.1923	0.2	0.219	0.2561	0.1909
D								0.1832	0.2129	0.2316	0.2134	0.2137
E								0.1016	0.11	0.123	0.1102	0.0772
F								0.1986	0.1962	0.2336	0.2012	0.2093
G								0.0476	0.069	0.0729	0.0863	0.0529
H								0.1595	0.1109	0.1813	0.1495	0.0978

Cell Index at: 35:00:41

	1	2	3	4	5	6	7	8	9	10	11	12
A								0.007	0.0294	0.0369	0.0371	0.0505
B								0.0617	0.0619	0.0652	0.0645	0.0773
C								0.1817	0.1942	0.2177	0.2517	0.193
D								0.1794	0.2155	0.2289	0.2094	0.2171
E								0.0968	0.1173	0.1271	0.116	0.0849
F								0.1983	0.1919	0.23	0.1993	0.2044
G								0.0534	0.069	0.0676	0.0847	0.0501
H								0.157	0.1108	0.1787	0.1474	0.0954

Cell Index at: 35:15:41

	1	2	3	4	5	6	7	8	9	10	11	12
A								0.0071	0.032	0.0367	0.0373	0.0555
B								0.0619	0.0574	0.0627	0.0682	0.0775
C								0.19	0.1926	0.2244	0.2498	0.1928
D								0.1804	0.2159	0.2357	0.2178	0.2057
E								0.091	0.1185	0.1288	0.1131	0.0751
F								0.2034	0.1942	0.2321	0.1962	0.207
G								0.0496	0.0648	0.0679	0.0849	0.0528
H								0.1559	0.1132	0.1798	0.1545	0.0987

Cell Index at: 35:30:42

	1	2	3	4	5	6	7	8	9	10	11	12
A								0.0049	0.0295	0.0343	0.0332	0.0525
B								0.0656	0.0541	0.0648	0.0669	0.0822
C								0.1898	0.1932	0.2221	0.2525	0.1899
D								0.1798	0.2221	0.2354	0.2167	0.2046
E								0.1086	0.1145	0.1275	0.1102	0.08
F								0.201	0.1914	0.2338	0.1985	0.2023
G								0.0508	0.0725	0.0665	0.0864	0.0512
H								0.1618	0.1094	0.1804	0.152	0.1001

Cell Index at: 35:45:42

	1	2	3	4	5	6	7	8	9	10	11	12
A								0.0068	0.0278	0.0424	0.033	0.0496
B								0.063	0.0524	0.0584	0.0675	0.0786
C								0.1916	0.1891	0.2177	0.2505	0.1912
D								0.179	0.225	0.2259	0.2172	0.2107
E								0.1022	0.12	0.1266	0.1108	0.082
F								0.2017	0.1925	0.2372	0.2057	0.2104
G								0.0472	0.0671	0.0686	0.0777	0.0522
H								0.1562	0.1013	0.1817	0.1516	0.101

Cell Index at: 36:00:43

	1	2	3	4	5	6	7	8	9	10	11	12
A								0.0046	0.0305	0.0385	0.0328	0.0484
B								0.0576	0.0559	0.0561	0.0661	0.0811
C								0.1924	0.195	0.2183	0.2577	0.1935
D								0.1865	0.2233	0.2312	0.2181	0.2058
E								0.1032	0.1141	0.1244	0.1083	0.0808
F								0.2056	0.1998	0.2327	0.202	0.2103
G								0.0411	0.0677	0.0675	0.0773	0.0499
H								0.1582	0.1031	0.1827	0.1539	0.102

Cell Index at: 36:15:42

	1	2	3	4	5	6	7	8	9	10	11	12
A								0.0059	0.0303	0.0406	0.0316	0.0507
B								0.0575	0.056	0.0548	0.0622	0.0799
C								0.1931	0.1902	0.2248	0.2582	0.1973
D								0.1829	0.221	0.2304	0.2145	0.208
E								0.1016	0.1225	0.1217	0.1035	0.0769
F								0.207	0.1963	0.2265	0.2044	0.2165
G								0.0507	0.0648	0.067	0.077	0.0487
H								0.1548	0.102	0.1824	0.1539	0.1014

Cell Index at: 36:30:42

	1	2	3	4	5	6	7	8	9	10	11	12
A								0.0053	0.0267	0.041	0.0322	0.0486
B								0.0601	0.0541	0.0551	0.0667	0.0823
C								0.1937	0.1851	0.2223	0.2599	0.1922
D								0.1835	0.2284	0.2248	0.2229	0.2054
E								0.1139	0.1123	0.1217	0.1034	0.0829
F								0.2067	0.1949	0.2344	0.2058	0.2163
G								0.0406	0.0634	0.0656	0.074	0.0539
H								0.1568	0.1033	0.1826	0.1552	0.1011

Cell Index at: 36:45:42

	1	2	3	4	5	6	7	8	9	10	11	12
A								0.0053	0.0264	0.036	0.0313	0.0466
B								0.0539	0.0544	0.0521	0.068	0.0821
C								0.1871	0.1983	0.2201	0.2542	0.1924
D								0.1869	0.2323	0.2344	0.215	0.2108
E								0.1029	0.1097	0.1207	0.1027	0.0803
F								0.2069	0.2005	0.2326	0.2024	0.2076
G								0.0506	0.0668	0.0682	0.0764	0.0477
H								0.1515	0.103	0.1812	0.1499	0.0977

Cell Index at: 37:00:42

	1	2	3	4	5	6	7	8	9	10	11	12
A								0.0049	0.0261	0.0389	0.0262	0.0476
B								0.0592	0.0533	0.0578	0.0658	0.0832
C								0.1898	0.1935	0.2242	0.2564	0.1954
D								0.1844	0.2283	0.2348	0.2221	0.206
E								0.1043	0.1033	0.1209	0.1114	0.0773
F								0.2049	0.1956	0.2319	0.2036	0.2161

G								0.0411	0.0671	0.0703	0.0695	0.0481
H								0.1555	0.1012	0.1839	0.1494	0.1012

Cell Index at: 37:15:43

	1	2	3	4	5	6	7	8	9	10	11	12
A								0.0092	0.0257	0.0396	0.0293	0.0465
B								0.0561	0.0529	0.0575	0.0621	0.081
C								0.1908	0.1918	0.2191	0.2546	0.1946
D								0.1825	0.2323	0.2291	0.2221	0.2019
E								0.1013	0.1116	0.1194	0.1124	0.074
F								0.2084	0.1922	0.2322	0.2029	0.2133
G								0.0444	0.0635	0.0656	0.0714	0.0491
H								0.1562	0.1026	0.1802	0.1521	0.1018

Cell Index at: 37:30:43

	1	2	3	4	5	6	7	8	9	10	11	12
A								0.0064	0.0285	0.0416	0.0313	0.0461
B								0.0553	0.055	0.0555	0.0617	0.0781
C								0.1877	0.1946	0.2329	0.256	0.1876
D								0.1791	0.2274	0.2276	0.2226	0.2056
E								0.1007	0.1131	0.1122	0.1061	0.077
F								0.2064	0.202	0.2327	0.2	0.2113
G								0.0397	0.0613	0.063	0.0705	0.0521
H								0.1589	0.1038	0.1784	0.1527	0.0991

Cell Index at: 37:45:44

	1	2	3	4	5	6	7	8	9	10	11	12
A								0.0062	0.0279	0.0368	0.0284	0.0483
B								0.0519	0.05	0.0544	0.065	0.0818
C								0.1905	0.1876	0.2265	0.2584	0.1841
D								0.185	0.2256	0.2341	0.225	0.2129
E								0.099	0.1137	0.1126	0.1003	0.0712
F								0.2083	0.1974	0.2297	0.208	0.2132
G								0.0412	0.0645	0.0675	0.0733	0.049
H								0.1575	0.1059	0.1825	0.1497	0.1031

Cell Index at: 38:00:44

	1	2	3	4	5	6	7	8	9	10	11	12
A								0.0043	0.0266	0.0388	0.0308	0.0479
B								0.0542	0.0529	0.0583	0.0646	0.0772
C								0.1853	0.1863	0.2224	0.2606	0.1893
D								0.184	0.2245	0.2305	0.2234	0.2062
E								0.1101	0.1134	0.1161	0.1017	0.0684
F								0.2057	0.198	0.2333	0.2009	0.2132
G								0.0381	0.0642	0.0659	0.072	0.0481
H								0.1594	0.1065	0.1864	0.1522	0.1004

Cell Index at: 38:15:45

	1	2	3	4	5	6	7	8	9	10	11	12
A								0.0035	0.0259	0.0407	0.0265	0.0453
B								0.0518	0.0497	0.0548	0.065	0.075
C								0.1835	0.1914	0.2258	0.2592	0.1904
D								0.1782	0.2182	0.2265	0.2298	0.2141
E								0.1001	0.1115	0.1125	0.111	0.0751
F								0.2163	0.1983	0.2265	0.2023	0.2108
G								0.0413	0.0642	0.0672	0.0707	0.0469
H								0.1592	0.1066	0.1809	0.1543	0.0985

Cell Index at: 38:30:44

	1	2	3	4	5	6	7	8	9	10	11	12
A								0.002	0.0257	0.0356	0.0277	0.0451
B								0.0503	0.052	0.0507	0.0653	0.0771
C								0.1862	0.1868	0.2228	0.2581	0.1843
D								0.1815	0.2212	0.2246	0.2234	0.2085
E								0.1069	0.113	0.1097	0.104	0.077
F								0.2085	0.1964	0.2391	0.1949	0.2097
G								0.0434	0.0604	0.0693	0.0676	0.0493
H								0.1595	0.1117	0.1805	0.1536	0.0991

Cell Index at: 38:45:44

	1	2	3	4	5	6	7	8	9	10	11	12
A								-0.0009	0.0212	0.0395	0.0264	0.0442
B								0.0486	0.0505	0.0558	0.0651	0.0775
C								0.187	0.1892	0.2216	0.2657	0.1865
D								0.1821	0.2186	0.2331	0.2334	0.2094
E								0.1001	0.1044	0.1101	0.1077	0.0721
F								0.2159	0.2011	0.236	0.2041	0.2061
G								0.0398	0.0617	0.063	0.0706	0.0515
H								0.1541	0.1095	0.1829	0.1543	0.1011

Cell Index at: 39:00:45

	1	2	3	4	5	6	7	8	9	10	11	12
A								0.001	0.0232	0.0369	0.0268	0.0486
B								0.0493	0.0447	0.0546	0.0692	0.0763
C								0.1814	0.1915	0.2199	0.2573	0.1907
D								0.1861	0.2166	0.2312	0.2291	0.2095
E								0.1053	0.109	0.1035	0.1106	0.0775
F								0.2126	0.1968	0.2334	0.2066	0.2095
G								0.0357	0.0637	0.0617	0.0737	0.05
H								0.1618	0.1094	0.185	0.153	0.0979

Cell Index at: 39:15:45

	1	2	3	4	5	6	7	8	9	10	11	12
A								-0.0019	0.0236	0.0363	0.0221	0.0458
B								0.0501	0.0452	0.0556	0.0646	0.0785
C								0.1791	0.1847	0.227	0.2654	0.192
D								0.1858	0.2241	0.2332	0.2266	0.2099
E								0.1011	0.1031	0.1066	0.1089	0.0757
F								0.2068	0.1986	0.2393	0.202	0.2145
G								0.0398	0.0584	0.0572	0.071	0.0473
H								0.1588	0.1082	0.1824	0.1532	0.0973

Cell Index at: 39:30:46

	1	2	3	4	5	6	7	8	9	10	11	12
A								-0.0023	0.0201	0.0384	0.0219	0.0451
B								0.0466	0.0467	0.0579	0.0648	0.0775
C								0.1846	0.1844	0.225	0.2615	0.1875
D								0.1859	0.2252	0.2294	0.2273	0.2067
E								0.1002	0.11	0.1144	0.1106	0.0801
F								0.2063	0.1982	0.2419	0.2039	0.2121
G								0.0386	0.0584	0.0659	0.0743	0.0469
H								0.1617	0.1079	0.1884	0.1549	0.0961

Cell Index at: 39:45:47

	1	2	3	4	5	6	7	8	9	10	11	12
A								0.0004	0.0189	0.0369	0.0233	0.045
B								0.0496	0.0444	0.055	0.0708	0.084
C								0.182	0.1881	0.2281	0.2606	0.1896
D								0.1871	0.2305	0.23	0.2285	0.2089
E								0.0955	0.1039	0.112	0.111	0.0772
F								0.2049	0.2043	0.2337	0.203	0.2064
G								0.0381	0.0611	0.0618	0.0693	0.0489
H								0.1586	0.1077	0.185	0.1548	0.1008

Cell Index at: 40:00:48

	1	2	3	4	5	6	7	8	9	10	11	12
A								-0.0002	0.0194	0.0395	0.0214	0.0415
B								0.0516	0.0441	0.0519	0.066	0.0798
C								0.182	0.1761	0.2264	0.2604	0.1943
D								0.1889	0.2301	0.2332	0.2317	0.2056
E								0.1049	0.106	0.1037	0.1085	0.0776

F								0.2107	0.2	0.2367	0.2012	0.2099
G								0.0373	0.0591	0.0713	0.074	0.0496
H								0.1601	0.109	0.1869	0.1569	0.099

Cell Index at: 40:15:48

	1	2	3	4	5	6	7	8	9	10	11	12
A								0.0003	0.0243	0.0381	0.0209	0.0401
B								0.0493	0.0492	0.0484	0.0678	0.0824
C								0.1778	0.1829	0.2254	0.2606	0.191
D								0.1882	0.2271	0.2364	0.229	0.2078
E								0.1017	0.1034	0.0994	0.1055	0.0768
F								0.2122	0.2006	0.235	0.2019	0.2122
G								0.0388	0.0624	0.062	0.0702	0.0481
H								0.1608	0.1113	0.1846	0.1567	0.1028

Cell Index at: 40:30:48

	1	2	3	4	5	6	7	8	9	10	11	12
A								-0.0003	0.024	0.0395	0.0199	0.0404
B								0.0484	0.0446	0.0494	0.0689	0.0782
C								0.1845	0.189	0.2283	0.2647	0.1952
D								0.1916	0.2297	0.2335	0.2308	0.2042
E								0.1023	0.1028	0.1062	0.117	0.0836
F								0.2105	0.205	0.2316	0.2041	0.2114
G								0.0384	0.0615	0.0665	0.0689	0.0472
H								0.1663	0.1115	0.1845	0.1595	0.0999

Cell Index at: 40:45:49

	1	2	3	4	5	6	7	8	9	10	11	12
A								-0.0006	0.0233	0.0413	0.0186	0.0396
B								0.0467	0.0475	0.0541	0.0695	0.0762
C								0.1829	0.1897	0.2276	0.263	0.1974
D								0.1862	0.2259	0.2409	0.2354	0.2054
E								0.0952	0.0955	0.1027	0.1062	0.0753
F								0.2095	0.1994	0.2341	0.2024	0.2143
G								0.0382	0.0624	0.0595	0.0685	0.0485
H								0.1654	0.1111	0.1847	0.1484	0.1003

Cell Index at: 41:00:48

	1	2	3	4	5	6	7	8	9	10	11	12
A								-0.0031	0.0244	0.0358	0.0199	0.0377
B								0.0479	0.0461	0.0546	0.0664	0.0781
C								0.1873	0.1893	0.231	0.2673	0.1909
D								0.1835	0.2255	0.2386	0.2326	0.2119
E								0.0938	0.1027	0.107	0.1091	0.081
F								0.2145	0.1966	0.2372	0.2009	0.2139
G								0.0392	0.0608	0.0644	0.0692	0.0442
H								0.1637	0.112	0.184	0.1503	0.1034

Cell Index at: 41:15:47

	1	2	3	4	5	6	7	8	9	10	11	12
A								-0.0031	0.0243	0.0376	0.0169	0.036
B								0.0489	0.0488	0.0517	0.0693	0.0771
C								0.1837	0.1867	0.2291	0.2676	0.1887
D								0.1926	0.232	0.2268	0.2291	0.2106
E								0.0973	0.1064	0.1094	0.1102	0.0783
F								0.2064	0.2044	0.2374	0.2126	0.2168
G								0.0391	0.0608	0.0676	0.066	0.0434
H								0.1653	0.1129	0.1846	0.1468	0.1018

Cell Index at: 41:30:47

	1	2	3	4	5	6	7	8	9	10	11	12
A								-0.0006	0.0246	0.0367	0.0195	0.0357
B								0.0438	0.0477	0.0492	0.0665	0.081
C								0.1799	0.1866	0.2236	0.2648	0.1973
D								0.1838	0.2302	0.2274	0.2294	0.2087
E								0.0974	0.1068	0.1061	0.1076	0.0796
F								0.2069	0.1995	0.2325	0.2048	0.2134
G								0.0359	0.059	0.0597	0.0632	0.0429
H								0.1644	0.1134	0.1852	0.1484	0.1043

Cell Index at: 41:45:48

	1	2	3	4	5	6	7	8	9	10	11	12
A								-0.0046	0.0244	0.037	0.0207	0.0339
B								0.0466	0.0455	0.0494	0.0654	0.0792
C								0.1772	0.1856	0.2261	0.2636	0.2017
D								0.1822	0.2284	0.2304	0.227	0.2088
E								0.0924	0.0941	0.106	0.1062	0.0715
F								0.2092	0.2028	0.2318	0.2073	0.221
G								0.0374	0.0598	0.0626	0.0624	0.0461
H								0.1649	0.1117	0.1834	0.148	0.1019

Cell Index at: 42:00:48

	1	2	3	4	5	6	7	8	9	10	11	12
A								-0.0046	0.0217	0.0367	0.0237	0.0366
B								0.0478	0.0454	0.0507	0.0672	0.0736
C								0.1865	0.1803	0.227	0.2654	0.2021
D								0.1934	0.2278	0.2275	0.2269	0.2087
E								0.1001	0.0977	0.1105	0.1077	0.0804
F								0.2105	0.2005	0.2312	0.2038	0.218
G								0.0357	0.0612	0.0628	0.0627	0.0468
H								0.166	0.1134	0.187	0.1504	0.1038

Cell Index at: 42:15:48

	1	2	3	4	5	6	7	8	9	10	11	12
A								-0.0043	0.0246	0.0399	0.0219	0.037
B								0.0435	0.0475	0.049	0.0643	0.0796
C								0.1866	0.1817	0.2299	0.267	0.203
D								0.1897	0.2338	0.2306	0.2292	0.212
E								0.0968	0.1	0.1082	0.1074	0.0822
F								0.2093	0.1949	0.2334	0.2066	0.2192
G								0.0343	0.0573	0.0586	0.0629	0.0456
H								0.1656	0.1137	0.1863	0.1481	0.1024

Cell Index at: 42:30:47

	1	2	3	4	5	6	7	8	9	10	11	12
A								-0.006	0.0257	0.0353	0.0199	0.04
B								0.0452	0.0413	0.0484	0.0696	0.078
C								0.183	0.1845	0.2268	0.2654	0.2011
D								0.1859	0.2367	0.237	0.2329	0.208
E								0.1027	0.0947	0.1046	0.1107	0.0806
F								0.2055	0.2037	0.2316	0.202	0.2209
G								0.0394	0.0617	0.0587	0.0677	0.0471
H								0.1653	0.115	0.1893	0.1488	0.1

Cell Index at: 42:45:47

	1	2	3	4	5	6	7	8	9	10	11	12
A								-0.0041	0.0228	0.0356	0.0155	0.038
B								0.0427	0.046	0.0489	0.0656	0.0773
C								0.1816	0.1804	0.2287	0.2662	0.1988
D								0.1865	0.236	0.2346	0.228	0.2101
E								0.0951	0.0989	0.1134	0.1099	0.0768
F								0.2022	0.2004	0.2292	0.1974	0.2173
G								0.0381	0.0613	0.0582	0.0655	0.043
H								0.1624	0.1155	0.1883	0.1518	0.0988

Cell Index at: 43:00:48

	1	2	3	4	5	6	7	8	9	10	11	12
A								-0.0051	0.0261	0.0358	0.0203	0.0405
B								0.0455	0.0499	0.0498	0.0684	0.0779
C								0.1889	0.1874	0.2275	0.2671	0.2001
D								0.1828	0.2325	0.2374	0.2331	0.2097

E								0.1008	0.0965	0.1153	0.1096	0.0807
F								0.2097	0.2043	0.2342	0.202	0.2186
G								0.0346	0.058	0.0547	0.0698	0.049
H								0.1633	0.1144	0.1861	0.1532	0.1008

Cell Index at: 43:15:48

	1	2	3	4	5	6	7	8	9	10	11	12
A								-0.0053	0.0257	0.0345	0.0184	0.0389
B								0.0454	0.0516	0.0514	0.0664	0.0834
C								0.1887	0.1894	0.2255	0.2645	0.2007
D								0.1839	0.2231	0.2339	0.2276	0.2124
E								0.0991	0.0985	0.1179	0.1095	0.0834
F								0.2125	0.1982	0.2322	0.2014	0.221
G								0.0395	0.0589	0.0609	0.0685	0.0466
H								0.1642	0.113	0.1833	0.1517	0.1

Cell Index at: 43:30:49

	1	2	3	4	5	6	7	8	9	10	11	12
A								-0.0049	0.023	0.034	0.0217	0.0399
B								0.0453	0.0522	0.0446	0.0657	0.0792
C								0.1899	0.178	0.2315	0.2614	0.206
D								0.1797	0.228	0.2407	0.2382	0.2033
E								0.0957	0.0947	0.1171	0.1016	0.0744
F								0.2164	0.1944	0.2348	0.2012	0.2204
G								0.0432	0.0603	0.0594	0.0638	0.0463
H								0.1637	0.1169	0.1848	0.1505	0.0989

Cell Index at: 43:45:49

	1	2	3	4	5	6	7	8	9	10	11	12
A								-0.0056	0.0288	0.0302	0.0236	0.0376
B								0.0496	0.044	0.0551	0.0642	0.0787
C								0.1885	0.1873	0.2301	0.2668	0.2034
D								0.1715	0.2292	0.2286	0.2362	0.2021
E								0.0982	0.0964	0.1157	0.1099	0.086
F								0.2113	0.1996	0.2334	0.2066	0.218
G								0.0395	0.0607	0.0597	0.0676	0.0458
H								0.1604	0.1173	0.1868	0.1487	0.0999

Cell Index at: 44:00:50

	1	2	3	4	5	6	7	8	9	10	11	12
A								-0.0059	0.0254	0.0345	0.0216	0.0385
B								0.0447	0.0479	0.052	0.068	0.0837
C								0.19	0.18	0.2279	0.2708	0.2069
D								0.1818	0.2343	0.2365	0.2255	0.2088
E								0.0969	0.0981	0.1174	0.1132	0.0817
F								0.2195	0.1995	0.2328	0.2055	0.2184
G								0.0389	0.0623	0.0612	0.0687	0.0477
H								0.1605	0.1146	0.1872	0.1473	0.0993

Cell Index at: 44:15:50

	1	2	3	4	5	6	7	8	9	10	11	12
A								-0.0031	0.0268	0.0363	0.0178	0.0393
B								0.0449	0.0445	0.0506	0.0703	0.0835
C								0.1871	0.1789	0.2302	0.2725	0.2008
D								0.1879	0.2307	0.2276	0.228	0.2048
E								0.0997	0.0976	0.1125	0.11	0.0777
F								0.2167	0.1996	0.234	0.2007	0.2182
G								0.0407	0.0586	0.0623	0.0654	0.0451
H								0.165	0.1179	0.1886	0.1478	0.0982

Cell Index at: 44:30:50

	1	2	3	4	5	6	7	8	9	10	11	12
A								-0.0035	0.0271	0.0347	0.0219	0.0397
B								0.0464	0.045	0.0519	0.0677	0.0787
C								0.1912	0.1826	0.2328	0.2685	0.2029
D								0.1838	0.23	0.232	0.2292	0.2069
E								0.0994	0.0977	0.1116	0.1173	0.078
F								0.219	0.2071	0.2345	0.2045	0.2169
G								0.0377	0.0585	0.0627	0.0693	0.0549
H								0.1643	0.1181	0.1883	0.1465	0.1004

Cell Index at: 44:45:51

	1	2	3	4	5	6	7	8	9	10	11	12
A								-0.0032	0.0276	0.0415	0.0209	0.0409
B								0.0423	0.0472	0.0477	0.0641	0.0808
C								0.1953	0.1859	0.2346	0.2646	0.2047
D								0.1869	0.2312	0.2304	0.2299	0.2068
E								0.0947	0.103	0.1208	0.1219	0.0772
F								0.221	0.1946	0.2269	0.2071	0.2144
G								0.0389	0.0618	0.0601	0.0683	0.0494
H								0.1608	0.1231	0.1865	0.1477	0.0993

Cell Index at: 45:00:51

	1	2	3	4	5	6	7	8	9	10	11	12
A								-0.0038	0.0258	0.0377	0.0186	0.0389
B								0.0436	0.0457	0.0478	0.0644	0.0803
C								0.194	0.1802	0.2302	0.2686	0.2032
D								0.179	0.232	0.2288	0.2257	0.2093
E								0.1084	0.0993	0.1174	0.1129	0.073
F								0.2172	0.2015	0.2366	0.2063	0.2198
G								0.0401	0.0542	0.0643	0.0697	0.0512
H								0.1611	0.1218	0.1871	0.1479	0.0988

Cell Index at: 45:15:52

	1	2	3	4	5	6	7	8	9	10	11	12
A								-0.0047	0.0252	0.0414	0.0181	0.0363
B								0.0411	0.0445	0.0485	0.0646	0.0798
C								0.1945	0.1881	0.2339	0.2665	0.1988
D								0.1779	0.2365	0.2265	0.2267	0.2112
E								0.1067	0.0931	0.1152	0.115	0.0776
F								0.2219	0.2029	0.2382	0.206	0.2185
G								0.0345	0.0603	0.062	0.0749	0.0502
H								0.164	0.1187	0.1859	0.1472	0.0974

Cell Index at: 45:30:53

	1	2	3	4	5	6	7	8	9	10	11	12
A								-0.0052	0.0233	0.0382	0.0207	0.0361
B								0.0395	0.0483	0.0489	0.0674	0.0817
C								0.1885	0.1892	0.2316	0.2662	0.2025
D								0.1856	0.2412	0.2295	0.2278	0.2142
E								0.1009	0.0979	0.1062	0.1125	0.0777
F								0.2187	0.1994	0.2416	0.2042	0.217
G								0.0405	0.0625	0.0635	0.0687	0.0487
H								0.1646	0.1216	0.1885	0.1488	0.0972

Cell Index at: 45:45:54

	1	2	3	4	5	6	7	8	9	10	11	12
A								-0.0061	0.0249	0.0396	0.0178	0.0412
B								0.0398	0.0439	0.047	0.0649	0.0767
C								0.1922	0.1825	0.229	0.264	0.2015
D								0.1802	0.2389	0.2299	0.2188	0.2127
E								0.1005	0.0936	0.1153	0.1155	0.0757
F								0.2179	0.2044	0.2383	0.206	0.2168
G								0.0382	0.0633	0.0663	0.0746	0.0511
H								0.1644	0.1215	0.1869	0.1455	0.0982

Cell Index at: 46:00:54

	1	2	3	4	5	6	7	8	9	10	11	12
A								-0.0091	0.0233	0.042	0.0073	0.0363
B								0.0393	0.0422	0.0486	0.0667	0.0813
C								0.189	0.1822	0.2261	0.266	0.1967

D								0.1834	0.2394	0.2311	0.2244	0.2103
E								0.102	0.0963	0.1165	0.1103	0.0829
F								0.2242	0.1986	0.2417	0.2001	0.213
G								0.0359	0.0651	0.0685	0.0701	0.0512
H								0.1624	0.1212	0.1885	0.1458	0.093

Cell Index at: 46:15:51

	1	2	3	4	5	6	7	8	9	10	11	12
A								-0.0073	0.0231	0.0396	0.0087	0.0388
B								0.0372	0.0432	0.048	0.0649	0.0784
C								0.1968	0.1855	0.226	0.2621	0.2017
D								0.1912	0.2369	0.2309	0.228	0.2109
E								0.1055	0.0966	0.1127	0.1114	0.0822
F								0.2191	0.1991	0.2432	0.2002	0.2159
G								0.0338	0.0617	0.0606	0.0714	0.0524
H								0.1656	0.122	0.1894	0.1444	0.0942

Cell Index at: 46:30:51

	1	2	3	4	5	6	7	8	9	10	11	12
A								-0.0077	0.0219	0.0453	0.0097	0.0386
B								0.0385	0.045	0.0476	0.0683	0.0791
C								0.1933	0.1916	0.232	0.2698	0.1996
D								0.1836	0.2322	0.2209	0.2291	0.204
E								0.0976	0.0963	0.1122	0.1075	0.0779
F								0.2102	0.2031	0.2411	0.2013	0.2117
G								0.0368	0.0579	0.065	0.0754	0.0488
H								0.1653	0.1215	0.1879	0.1449	0.0971

Cell Index at: 46:45:52

	1	2	3	4	5	6	7	8	9	10	11	12
A								-0.0075	0.0217	0.0429	0.0122	0.0394
B								0.0375	0.0426	0.0471	0.0682	0.0826
C								0.192	0.1867	0.2356	0.2601	0.2003
D								0.189	0.2356	0.2282	0.2277	0.2066
E								0.1048	0.0969	0.1093	0.1091	0.0856
F								0.2113	0.2026	0.2419	0.2025	0.2089
G								0.0337	0.0653	0.0642	0.0733	0.0477
H								0.1658	0.1209	0.1886	0.1438	0.0968

Cell Index at: 47:00:53

	1	2	3	4	5	6	7	8	9	10	11	12
A								-0.0072	0.0148	0.0419	0.0092	0.0353
B								0.0375	0.0409	0.0477	0.068	0.0788
C								0.1957	0.1907	0.2309	0.2665	0.1952
D								0.187	0.2397	0.2261	0.2221	0.2094
E								0.0996	0.1014	0.113	0.1072	0.0717
F								0.2136	0.2011	0.243	0.1989	0.2121
G								0.0358	0.0641	0.07	0.0753	0.0489
H								0.1673	0.1196	0.1888	0.1435	0.0974

Cell Index at: 47:15:53

	1	2	3	4	5	6	7	8	9	10	11	12
A								-0.006	0.0173	0.0425	0.0097	0.0381
B								0.0406	0.0438	0.0449	0.068	0.0815
C								0.1914	0.185	0.2353	0.264	0.1944
D								0.1892	0.241	0.2344	0.2298	0.2086
E								0.0986	0.0954	0.1059	0.1099	0.0774
F								0.2197	0.2009	0.24	0.1992	0.2119
G								0.0376	0.06	0.062	0.0764	0.0513
H								0.1656	0.1227	0.1889	0.1466	0.0978

Cell Index at: 47:30:53

	1	2	3	4	5	6	7	8	9	10	11	12
A								-0.0085	0.0173	0.0406	0.0087	0.0364
B								0.0408	0.0461	0.0436	0.07	0.0802
C								0.1902	0.1825	0.2321	0.2617	0.2019
D								0.1926	0.2299	0.2259	0.2308	0.2056
E								0.1016	0.0882	0.1133	0.1097	0.0727
F								0.2215	0.199	0.2405	0.2008	0.212
G								0.0362	0.0585	0.065	0.0736	0.0464
H								0.1651	0.1233	0.1822	0.1423	0.1021

Cell Index at: 47:45:53

	1	2	3	4	5	6	7	8	9	10	11	12
A								-0.0037	0.0184	0.043	0.0095	0.0362
B								0.0361	0.0471	0.0452	0.0696	0.0803
C								0.1938	0.1829	0.2352	0.264	0.1976
D								0.1938	0.2392	0.2265	0.2325	0.213
E								0.0979	0.0869	0.1167	0.1132	0.0832
F								0.2176	0.2037	0.2364	0.2016	0.2133
G								0.0388	0.0671	0.0617	0.0747	0.0445
H								0.1677	0.1231	0.1812	0.1469	0.1013

Cell Index at: 48:00:53

	1	2	3	4	5	6	7	8	9	10	11	12
A								-0.0066	0.0185	0.0418	0.0074	0.0352
B								0.0397	0.0478	0.0475	0.0676	0.0793
C								0.2023	0.1823	0.2397	0.2657	0.2007
D								0.186	0.2404	0.2249	0.2268	0.2142
E								0.1071	0.0883	0.1125	0.1127	0.0806
F								0.2205	0.2016	0.2429	0.1975	0.2131
G								0.0399	0.0685	0.0659	0.0727	0.0458
H								0.167	0.125	0.1838	0.1444	0.1006

A vs B	0.012755	B vs C	8.86E-05	C vs D	0.986073	D vs E	1.16E-05	E vs F	5.46E-06	F vs G	5.55E-07	G vs H	0.002364
A vs C	1.66E-05	B vs D	1.09E-06	C vs E	0.000562	D vs F	0.790801	E vs G	0.002104	F vs H	0.005191		
A vs D	2.78E-07	B vs E	0.002262	C vs F	0.865714	D vs G	1.43E-06	E vs H	0.037767				
A vs E	0.000122	B vs F	5.28E-07	C vs G	0.000121	D vs H	0.00408						
A vs F	2.17E-07	B vs G	0.830674	C vs.H	0.008059								
A vs G	0.008442	B vs H	0.001917										
A vs H	0.000232												

Count	*Sum*	*Average*	*Variance*
5	0.0963	0.01926	0.000393
5	0.2819	0.05638	0.00027
5	1.0907	0.21814	0.001142
5	1.0923	0.21846	0.000416
5	0.5012	0.10024	0.00022
5	1.0756	0.21512	0.000325
5	0.2928	0.05856	0.000216
5	0.7208	0.14416	0.001089

SS	*df*	*MS*	*F*	*P-value*	*F crit*
0.233503	7	0.033358	65.54862	3.62E-17	2.312741
0.016285	32	0.000509			
0.249788	39				

Cell Index at: 48:15:53

	1	2	3	4	5	6	7	8	9	10	11	12
A								-0.0062	0.019	0.0408	0.0087	0.0343
B								0.0414	0.0438	0.0456	0.0646	0.0811
C								0.1915	0.1841	0.2333	0.2658	0.2015
D								0.1924	0.2378	0.2313	0.236	0.2117
E								0.1006	0.0852	0.113	0.1108	0.0821
F								0.2241	0.1986	0.2472	0.2096	0.2211
G								0.0371	0.0651	0.0655	0.0738	0.0458
H								0.1693	0.1196	0.1852	0.1434	0.103

Cell Index at: 48:30:53

	1	2	3	4	5	6	7	8	9	10	11	12
A								-0.0048	0.0218	0.0417	0.0097	0.0329
B								0.0445	0.0459	0.0432	0.0687	0.0827
C								0.1937	0.1842	0.2339	0.2647	0.1968
D								0.1935	0.2452	0.221	0.2299	0.2073
E								0.0937	0.0908	0.1178	0.1098	0.084
F								0.2174	0.1961	0.2391	0.2016	0.2193
G								0.041	0.0666	0.0595	0.0744	0.0458
H								0.1659	0.1216	0.1839	0.1442	0.1014

Cell Index at: 48:45:54

	1	2	3	4	5	6	7	8	9	10	11	12
A								-0.0066	0.018	0.0416	0.0091	0.0346
B								0.0431	0.0438	0.0468	0.0672	0.0773
C								0.1906	0.188	0.2403	0.2691	0.1992
D								0.1875	0.2374	0.2168	0.2331	0.214
E								0.101	0.09	0.1129	0.1066	0.0791
F								0.2199	0.1981	0.2308	0.2016	0.2211
G								0.0396	0.0628	0.0643	0.0726	0.0504
H								0.1705	0.1232	0.1824	0.1474	0.1035

Cell Index at: 49:00:55

	1	2	3	4	5	6	7	8	9	10	11	12
A								-0.0056	0.0215	0.0405	0.0095	0.0343

B								0.0426	0.0413	0.0436	0.0696	0.0811
C								0.1864	0.1853	0.2397	0.2644	0.1926
D								0.1859	0.2371	0.2223	0.2333	0.2114
E								0.0973	0.0869	0.115	0.1071	0.0809
F								0.2183	0.2058	0.2246	0.1979	0.2207
G								0.0418	0.0666	0.0644	0.0738	0.0461
H								0.1722	0.122	0.1829	0.1431	0.1019

Cell Index at: 49:15:56

	1	2	3	4	5	6	7	8	9	10	11	12
A								-0.0074	0.0189	0.0404	0.0079	0.0342
B								0.0411	0.0458	0.0451	0.0677	0.0755
C								0.185	0.1829	0.2403	0.2655	0.1954
D								0.1883	0.2431	0.2107	0.2256	0.2183
E								0.0971	0.0963	0.1196	0.1077	0.0756
F								0.2194	0.1982	0.2333	0.2005	0.2163
G								0.0382	0.065	0.0604	0.0688	0.0473
H								0.1669	0.1212	0.1831	0.1469	0.1018

Cell Index at: 49:30:56

	1	2	3	4	5	6	7	8	9	10	11	12
A								-0.0067	0.0191	0.039	0.0065	0.0353
B								0.0417	0.0422	0.0485	0.0732	0.0711
C								0.1866	0.1833	0.2384	0.262	0.198
D								0.1925	0.2379	0.2202	0.228	0.2133
E								0.1055	0.0888	0.117	0.1121	0.0844
F								0.2165	0.2049	0.2287	0.2015	0.2216
G								0.044	0.0651	0.0595	0.0693	0.0449
H								0.17	0.1236	0.1829	0.1501	0.102

Cell Index at: 49:45:57

	1	2	3	4	5	6	7	8	9	10	11	12
A								-0.009	0.0185	0.0403	0.0081	0.0352
B								0.0381	0.0347	0.0471	0.0696	0.0798
C								0.1881	0.1848	0.2361	0.2616	0.1982
D								0.187	0.2422	0.2134	0.2264	0.2069
E								0.0941	0.0923	0.1173	0.1071	0.0821
F								0.2171	0.2053	0.2291	0.2002	0.2158
G								0.0446	0.0661	0.0618	0.0639	0.0456
H								0.1688	0.1258	0.1816	0.1481	0.1022

Cell Index at: 50:00:57

	1	2	3	4	5	6	7	8	9	10	11	12
A								-0.001	0.0184	0.041	0.008	0.0315
B								0.0454	0.0433	0.0475	0.069	0.0742
C								0.1871	0.1858	0.2407	0.2627	0.1952
D								0.1873	0.2434	0.2226	0.2328	0.2097
E								0.1028	0.0883	0.1188	0.1018	0.0799
F								0.2178	0.2016	0.2351	0.1991	0.2143
G								0.0459	0.0602	0.0602	0.0664	0.0465
H								0.1688	0.1185	0.1818	0.1498	0.1031

Cell Index at: 50:15:57

	1	2	3	4	5	6	7	8	9	10	11	12
A								-0.0032	0.018	0.0361	0.0057	0.0356
B								0.0417	0.0409	0.0518	0.0689	0.0778
C								0.1826	0.1879	0.2405	0.2593	0.2
D								0.1865	0.2429	0.2211	0.2322	0.2117
E								0.095	0.0914	0.118	0.1078	0.0741
F								0.214	0.2022	0.2328	0.2085	0.2113
G								0.0454	0.0628	0.0627	0.0649	0.0437
H								0.1701	0.1182	0.1807	0.1494	0.1026

Cell Index at: 50:30:58

	1	2	3	4	5	6	7	8	9	10	11	12
A								-0.0034	0.017	0.0394	0.0097	0.0364
B								0.0445	0.0419	0.0548	0.0694	0.0738
C								0.1815	0.177	0.2326	0.2626	0.2046
D								0.1844	0.2362	0.2172	0.2342	0.2119
E								0.1017	0.0947	0.1189	0.1026	0.0868
F								0.2154	0.2027	0.227	0.2003	0.2119
G								0.0396	0.0627	0.0621	0.0651	0.0427
H								0.1726	0.1219	0.182	0.1474	0.1071

Cell Index at: 50:45:58

	1	2	3	4	5	6	7	8	9	10	11	12
A								-0.0031	0.0204	0.0367	0.0054	0.0346
B								0.0369	0.0427	0.0538	0.0706	0.0731
C								0.1744	0.1759	0.2427	0.2626	0.2067
D								0.1839	0.2402	0.2256	0.2298	0.2057
E								0.1005	0.0953	0.1175	0.1027	0.0769
F								0.2199	0.2046	0.234	0.2028	0.2052
G								0.0412	0.0645	0.0624	0.0655	0.0435
H								0.1706	0.1206	0.1824	0.1482	0.1038

Cell Index at: 51:00:59

	1	2	3	4	5	6	7	8	9	10	11	12
A								-0.0033	0.0186	0.0358	0.0106	0.0337
B								0.0407	0.0434	0.0537	0.0675	0.0694
C								0.1807	0.18	0.2405	0.2601	0.203
D								0.1866	0.2387	0.2241	0.2268	0.2101
E								0.0988	0.0863	0.1157	0.1031	0.0819
F								0.2096	0.2011	0.2338	0.2128	0.2053
G								0.042	0.0641	0.0644	0.0632	0.0455
H								0.168	0.1225	0.1808	0.1473	0.1046

Cell Index at: 51:16:00

	1	2	3	4	5	6	7	8	9	10	11	12
A								-0.0003	0.0167	0.0364	0.01	0.0345
B								0.0404	0.0405	0.0492	0.0643	0.0727
C								0.1777	0.1811	0.2465	0.2659	0.2055
D								0.1866	0.236	0.2165	0.2237	0.2161
E								0.1006	0.0958	0.1172	0.1008	0.0826
F								0.2169	0.2103	0.2363	0.2089	0.2042
G								0.0412	0.0647	0.07	0.0623	0.0446
H								0.1686	0.1229	0.1797	0.1474	0.1063

Cell Index at: 51:31:01

	1	2	3	4	5	6	7	8	9	10	11	12
A								-0.0033	0.0201	0.0369	0.0094	0.0355
B								0.0389	0.0387	0.0481	0.0694	0.0685
C								0.1815	0.1809	0.2444	0.2657	0.2003
D								0.1972	0.2301	0.2207	0.2261	0.2186
E								0.0996	0.0903	0.1091	0.1082	0.0819
F								0.2165	0.2029	0.2302	0.2103	0.2035
G								0.0417	0.0615	0.0667	0.0638	0.0468
H								0.1746	0.1205	0.1768	0.1489	0.1068

Cell Index at: 51:46:00

	1	2	3	4	5	6	7	8	9	10	11	12
A								-0.0005	0.0184	0.0397	0.0049	0.0334
B								0.0392	0.039	0.0458	0.0674	0.0677
C								0.1804	0.1734	0.2451	0.2641	0.2039
D								0.1881	0.2266	0.2195	0.2329	0.2121
E								0.1016	0.0914	0.1134	0.1097	0.0808
F								0.2138	0.2077	0.2342	0.2104	0.2005
G								0.038	0.0637	0.0686	0.061	0.0437
H								0.1725	0.1234	0.1809	0.1505	0.1095

Cell Index at: 52:01:01

	1	2	3	4	5	6	7	8	9	10	11	12

A								-0.0014	0.0175	0.0417	0.0094	0.0334
B								0.0399	0.0358	0.043	0.0641	0.0663
C								0.1745	0.1727	0.246	0.2659	0.2035
D								0.1867	0.2304	0.2216	0.2253	0.2115
E								0.1064	0.0892	0.116	0.1049	0.0745
F								0.2151	0.2085	0.2371	0.2159	0.2059
G								0.0412	0.0685	0.0676	0.0647	0.0389
H								0.1741	0.1233	0.1815	0.1474	0.1102

Cell Index at: 52:16:01

	1	2	3	4	5	6	7	8	9	10	11	12
A								-0.0064	0.015	0.0397	0.009	0.0336
B								0.0383	0.0348	0.0496	0.0652	0.0735
C								0.1744	0.1756	0.2525	0.2701	0.2039
D								0.1813	0.2324	0.2226	0.2355	0.2148
E								0.1045	0.0879	0.1094	0.111	0.0817
F								0.2166	0.2064	0.2403	0.2177	0.2053
G								0.0433	0.0638	0.0669	0.0689	0.0473
H								0.1665	0.1254	0.1813	0.1517	0.1092

Cell Index at: 52:31:01

	1	2	3	4	5	6	7	8	9	10	11	12
A								-0.0043	0.0132	0.0362	0.0086	0.0344
B								0.0372	0.0389	0.0457	0.0661	0.0734
C								0.1788	0.1784	0.2536	0.2676	0.2009
D								0.1935	0.2344	0.2234	0.2338	0.2172
E								0.1014	0.0847	0.1141	0.1062	0.0805
F								0.2177	0.2051	0.2395	0.2134	0.2122
G								0.0372	0.0652	0.0718	0.0673	0.0449
H								0.171	0.1248	0.1822	0.1519	0.1096

Cell Index at: 52:46:02

	1	2	3	4	5	6	7	8	9	10	11	12
A								-0.0033	0.0143	0.0386	0.0114	0.0375
B								0.0405	0.0359	0.0447	0.0658	0.0708
C								0.1783	0.1733	0.248	0.2686	0.2052
D								0.1869	0.2288	0.2215	0.232	0.2195
E								0.095	0.0882	0.1152	0.1074	0.0823
F								0.216	0.2036	0.2398	0.2212	0.2079
G								0.0403	0.0666	0.0662	0.0677	0.0391
H								0.1712	0.1216	0.1817	0.1501	0.1102

Cell Index at: 53:01:02

	1	2	3	4	5	6	7	8	9	10	11	12
A								-0.0053	0.0143	0.0392	0.0093	0.0413
B								0.0416	0.036	0.0419	0.0654	0.0685
C								0.1829	0.1705	0.2497	0.2694	0.2039
D								0.1923	0.2289	0.2248	0.2293	0.2205
E								0.1028	0.0918	0.1102	0.1087	0.0799
F								0.2185	0.2083	0.2374	0.2262	0.211
G								0.0355	0.0635	0.0657	0.0659	0.047
H								0.1708	0.1228	0.1847	0.1477	0.1077

Cell Index at: 53:16:03

	1	2	3	4	5	6	7	8	9	10	11	12
A								-0.0044	0.0153	0.0395	0.009	0.0387
B								0.0363	0.0365	0.0471	0.0641	0.0666
C								0.1733	0.179	0.2507	0.2667	0.2078
D								0.1868	0.2294	0.2261	0.2368	0.2167
E								0.0981	0.0931	0.1075	0.1109	0.0805
F								0.2211	0.2088	0.2419	0.2269	0.2084
G								0.0423	0.0633	0.0645	0.0676	0.0454
H								0.1711	0.1234	0.1806	0.1511	0.1096

Cell Index at: 53:31:04

	1	2	3	4	5	6	7	8	9	10	11	12
A								-0.0057	0.0158	0.0386	0.0102	0.0365
B								0.0407	0.0352	0.0468	0.0672	0.0689
C								0.1749	0.1777	0.2515	0.2669	0.2098
D								0.1884	0.2324	0.2301	0.2414	0.2157
E								0.0999	0.0872	0.117	0.1113	0.0769
F								0.2232	0.2047	0.2463	0.2209	0.2111
G								0.0429	0.058	0.0613	0.0682	0.0448
H								0.1727	0.1212	0.1815	0.1501	0.1088

Cell Index at: 53:46:05

	1	2	3	4	5	6	7	8	9	10	11	12
A								-0.0062	0.016	0.0389	0.0105	0.0394
B								0.0381	0.0297	0.046	0.0669	0.0697
C								0.1791	0.1796	0.2527	0.2683	0.2136
D								0.1905	0.2302	0.2343	0.2337	0.2142
E								0.1052	0.0961	0.1129	0.1084	0.0783
F								0.2245	0.207	0.2453	0.219	0.2134
G								0.0408	0.0593	0.0647	0.0636	0.0472
H								0.1713	0.1253	0.1825	0.1507	0.1094

Cell Index at: 54:01:05

	1	2	3	4	5	6	7	8	9	10	11	12
A								-0.0061	0.0146	0.037	0.0107	0.0403
B								0.0347	0.0372	0.0524	0.063	0.068
C								0.1793	0.1853	0.2562	0.2602	0.2122
D								0.1875	0.2356	0.2262	0.2391	0.2119
E								0.0981	0.09	0.1056	0.1126	0.0651
F								0.2219	0.2135	0.2495	0.2245	0.212
G								0.0388	0.0602	0.065	0.0664	0.0442
H								0.1737	0.1255	0.184	0.1488	0.1108

Cell Index at: 54:16:05

	1	2	3	4	5	6	7	8	9	10	11	12
A								-0.0045	0.0166	0.0408	0.0114	0.0399
B								0.0342	0.0345	0.0488	0.0653	0.0662
C								0.1738	0.1811	0.2588	0.271	0.2136
D								0.1828	0.2375	0.2282	0.2392	0.2178
E								0.0978	0.0941	0.1076	0.1114	0.0748
F								0.2262	0.2157	0.2505	0.2249	0.215
G								0.0425	0.0615	0.0634	0.0607	0.0445
H								0.1739	0.1246	0.1847	0.1508	0.1136

Cell Index at: 54:31:05

	1	2	3	4	5	6	7	8	9	10	11	12
A								-0.0036	0.0166	0.0354	0.0128	0.0419
B								0.0405	0.037	0.0468	0.0654	0.0637
C								0.1786	0.1776	0.2604	0.2674	0.2115
D								0.1877	0.2407	0.2311	0.2373	0.2225
E								0.0969	0.0896	0.1119	0.1124	0.0789
F								0.2265	0.2172	0.249	0.2235	0.2193
G								0.04	0.0585	0.0608	0.062	0.0449
H								0.1715	0.1225	0.1857	0.1503	0.1136

Cell Index at: 54:46:05

	1	2	3	4	5	6	7	8	9	10	11	12
A								-0.0066	0.015	0.0375	0.0123	0.0386
B								0.0409	0.0337	0.0504	0.071	0.065
C								0.1798	0.1863	0.2619	0.2717	0.2096
D								0.1903	0.2384	0.2299	0.242	0.2181
E								0.1059	0.0891	0.1097	0.12	0.0787
F								0.2277	0.2186	0.2446	0.227	0.2152
G								0.0428	0.0549	0.0629	0.0642	0.0458
H								0.1693	0.1206	0.1849	0.1529	0.1135

Cell Index at: 55:01:05

	1	2	3	4	5	6	7	8	9	10	11	12
A								-0.0044	0.0143	0.0367	0.0126	0.0386
B								0.0352	0.0291	0.0501	0.0641	0.068
C								0.1777	0.1852	0.2602	0.2728	0.2106
D								0.1941	0.2342	0.2273	0.2359	0.2248
E								0.1006	0.094	0.1114	0.1125	0.0764
F								0.2271	0.2118	0.246	0.2233	0.221
G								0.0395	0.056	0.0641	0.0622	0.0423
H								0.1716	0.1222	0.1832	0.1537	0.1144

Cell Index at: 55:16:06

	1	2	3	4	5	6	7	8	9	10	11	12
A								-0.0012	0.0144	0.0373	0.0107	0.0376
B								0.0372	0.0307	0.0462	0.0669	0.0685
C								0.1732	0.1874	0.2606	0.2688	0.2106
D								0.1865	0.2357	0.2307	0.238	0.222
E								0.1014	0.0959	0.1159	0.1141	0.0783
F								0.2283	0.2187	0.2422	0.2235	0.2249
G								0.0416	0.0537	0.0591	0.0616	0.0434
H								0.1711	0.1213	0.1845	0.1497	0.1145

Cell Index at: 55:31:07

	1	2	3	4	5	6	7	8	9	10	11	12
A								-0.0057	0.0131	0.0354	0.0103	0.0377
B								0.0405	0.0287	0.0487	0.0684	0.0636
C								0.1748	0.1836	0.2638	0.2757	0.2131
D								0.1863	0.239	0.2255	0.2389	0.2255
E								0.0981	0.0975	0.1154	0.1088	0.0738
F								0.223	0.2175	0.2528	0.2317	0.2227
G								0.0425	0.0546	0.0632	0.0566	0.0408
H								0.1756	0.1222	0.1848	0.1521	0.114

Cell Index at: 55:46:07

	1	2	3	4	5	6	7	8	9	10	11	12
A								-0.0041	0.0169	0.0369	0.0121	0.0376
B								0.0381	0.0329	0.0487	0.0679	0.0632
C								0.1737	0.1876	0.2684	0.2749	0.2135
D								0.1942	0.2269	0.2268	0.2435	0.2211
E								0.1007	0.0895	0.1074	0.1167	0.0776
F								0.2251	0.2171	0.2487	0.2302	0.2242
G								0.0392	0.0562	0.0621	0.0592	0.0416
H								0.1756	0.1164	0.187	0.1531	0.1127

Cell Index at: 56:01:07

	1	2	3	4	5	6	7	8	9	10	11	12
A								-0.0033	0.0154	0.0374	0.0073	0.0386
B								0.0345	0.0302	0.0504	0.0681	0.063
C								0.175	0.186	0.2599	0.2778	0.2124
D								0.1897	0.2335	0.2289	0.2454	0.2321
E								0.1035	0.0933	0.1098	0.1095	0.0769
F								0.228	0.2179	0.2528	0.2294	0.2276
G								0.0404	0.0565	0.0614	0.0629	0.0457
H								0.1733	0.1218	0.1801	0.1508	0.1162

Cell Index at: 56:16:06

	1	2	3	4	5	6	7	8	9	10	11	12
A								-0.0044	0.0161	0.0358	0.0102	0.0383
B								0.034	0.0353	0.0507	0.0655	0.0648
C								0.175	0.1887	0.2685	0.2783	0.2095
D								0.1887	0.2356	0.2281	0.2443	0.2299
E								0.0959	0.0889	0.1118	0.1124	0.0827
F								0.2299	0.2188	0.2581	0.2277	0.2211
G								0.0416	0.0544	0.0623	0.0565	0.0427
H								0.1725	0.1216	0.1791	0.1556	0.1159

Cell Index at: 56:31:07

	1	2	3	4	5	6	7	8	9	10	11	12
A								-0.0018	0.0119	0.0372	0.0085	0.0388
B								0.0361	0.037	0.045	0.0688	0.0669
C								0.1764	0.1901	0.2668	0.2672	0.2094
D								0.1896	0.2376	0.2313	0.2469	0.2267
E								0.0977	0.0948	0.1121	0.1158	0.0784
F								0.2286	0.2245	0.2585	0.2305	0.2307
G								0.0441	0.0511	0.0629	0.0581	0.045
H								0.1725	0.1193	0.1759	0.1533	0.1189

Cell Index at: 56:46:08

	1	2	3	4	5	6	7	8	9	10	11	12
A								-0.0027	0.016	0.0364	0.0091	0.0386
B								0.0346	0.0341	0.0399	0.0691	0.0693
C								0.1791	0.1852	0.2715	0.2725	0.2118
D								0.184	0.2461	0.2305	0.2482	0.2201
E								0.0968	0.0925	0.1091	0.1116	0.0765
F								0.232	0.2229	0.2545	0.2256	0.227
G								0.0441	0.0544	0.0589	0.0617	0.0415
H								0.1736	0.1214	0.1742	0.1537	0.1169

Cell Index at: 57:01:09

	1	2	3	4	5	6	7	8	9	10	11	12
A								-0.0099	0.0147	0.0373	0.0066	0.035
B								0.0371	0.0339	0.0439	0.0682	0.0673
C								0.1771	0.1859	0.2731	0.2701	0.2134
D								0.186	0.2473	0.2364	0.2429	0.2199
E								0.0923	0.0913	0.105	0.1141	0.0784
F								0.2332	0.2181	0.2624	0.2253	0.2225
G								0.0395	0.0558	0.058	0.0605	0.0427
H								0.1755	0.1208	0.1763	0.1591	0.1152

Cell Index at: 57:16:10

	1	2	3	4	5	6	7	8	9	10	11	12
A								-0.004	0.0121	0.0346	0.0067	0.0393
B								0.0376	0.0348	0.0402	0.0713	0.063
C								0.1818	0.1787	0.268	0.2686	0.2106
D								0.1827	0.2431	0.2329	0.2477	0.227
E								0.0962	0.0969	0.1064	0.1091	0.0826
F								0.2294	0.2157	0.2572	0.2288	0.2248
G								0.0445	0.0519	0.0549	0.0569	0.0442
H								0.1787	0.1189	0.1795	0.16	0.1178

Cell Index at: 57:31:10

	1	2	3	4	5	6	7	8	9	10	11	12
A								-0.0032	0.0137	0.0392	0.0081	0.0364
B								0.0361	0.0325	0.0446	0.0658	0.0599
C								0.1867	0.1843	0.2702	0.272	0.2158
D								0.186	0.248	0.2295	0.2483	0.2165
E								0.096	0.0912	0.1124	0.1148	0.0792
F								0.2335	0.2205	0.2551	0.2242	0.2277
G								0.0383	0.0522	0.0564	0.0577	0.0414
H								0.1778	0.1218	0.1799	0.1582	0.1173

Cell Index at: 57:46:10

	1	2	3	4	5	6	7	8	9	10	11	12
A								-0.0024	0.0135	0.0374	0.0043	0.0387
B								0.0381	0.0345	0.0415	0.0655	0.0603
C								0.1793	0.181	0.2675	0.2774	0.2154
D								0.1885	0.2405	0.2322	0.2522	0.2208
E								0.0958	0.0925	0.1109	0.1189	0.0849
F								0.2247	0.2243	0.2564	0.235	0.226
G								0.0412	0.0516	0.0562	0.0612	0.04
H								0.1767	0.1193	0.1822	0.1645	0.1176

Cell Index at: 58:01:10

	1	2	3	4	5	6	7	8	9	10	11	12
A								-0.0049	0.0133	0.0376	0.0037	0.0399
B								0.041	0.0311	0.0427	0.0711	0.0601
C								0.1827	0.1895	0.2707	0.2715	0.2167
D								0.1828	0.2486	0.2302	0.2576	0.2219
E								0.0909	0.0925	0.1126	0.1149	0.0838
F								0.2269	0.2217	0.2583	0.2312	0.2262
G								0.0436	0.0479	0.0589	0.0571	0.0409
H								0.176	0.1231	0.1816	0.1657	0.1197

Cell Index at: 58:16:09

	1	2	3	4	5	6	7	8	9	10	11	12
A								-0.0042	0.0128	0.0354	0.005	0.0399
B								0.036	0.0286	0.0451	0.0684	0.0637
C								0.1851	0.1873	0.2751	0.2717	0.2213
D								0.1831	0.249	0.2394	0.2517	0.2352
E								0.0931	0.0883	0.1115	0.1158	0.0779
F								0.2364	0.2224	0.254	0.2317	0.2319
G								0.0428	0.0511	0.0576	0.0599	0.0407
H								0.1732	0.1216	0.1854	0.1666	0.1193

Cell Index at: 58:31:09

	1	2	3	4	5	6	7	8	9	10	11	12
A								-0.0027	0.0119	0.0351	0.0081	0.0418
B								0.0358	0.0335	0.0415	0.0688	0.0641
C								0.1812	0.1917	0.2722	0.2762	0.2234
D								0.1982	0.2459	0.2336	0.2485	0.2295
E								0.0967	0.0873	0.1192	0.1155	0.077
F								0.2364	0.2273	0.2547	0.2294	0.2308
G								0.0426	0.0502	0.056	0.0599	0.0413
H								0.1761	0.1208	0.1845	0.1637	0.1187

Cell Index at: 58:46:09

	1	2	3	4	5	6	7	8	9	10	11	12
A								-0.0057	0.0125	0.0318	0.0059	0.0389
B								0.0312	0.0295	0.0431	0.0691	0.0654
C								0.1835	0.1932	0.2792	0.2762	0.2221
D								0.1972	0.2497	0.2331	0.2551	0.229
E								0.0966	0.0894	0.1191	0.1053	0.0779
F								0.2293	0.2253	0.2639	0.2269	0.23
G								0.0415	0.0555	0.0548	0.0617	0.039
H								0.1743	0.1199	0.1844	0.1641	0.1142

Cell Index at: 59:01:09

	1	2	3	4	5	6	7	8	9	10	11	12
A								-0.0041	0.0129	0.0324	0.006	0.0405
B								0.0353	0.0318	0.0431	0.0613	0.0677
C								0.1804	0.1889	0.2736	0.2767	0.2184
D								0.1947	0.2438	0.2392	0.2534	0.2317
E								0.0916	0.0814	0.1088	0.1057	0.0734
F								0.2332	0.2225	0.2587	0.2285	0.231
G								0.0421	0.0547	0.0557	0.0609	0.0393
H								0.181	0.12	0.1825	0.1644	0.1162

Cell Index at: 59:16:09

	1	2	3	4	5	6	7	8	9	10	11	12
A								-0.0014	0.0099	0.0316	0.0057	0.0411
B								0.0357	0.0346	0.039	0.0673	0.0619
C								0.1861	0.1924	0.2788	0.279	0.2251
D								0.1918	0.2427	0.2327	0.2401	0.2295
E								0.1011	0.0899	0.1184	0.114	0.078
F								0.2394	0.2236	0.2566	0.2271	0.234
G								0.0407	0.0531	0.053	0.0613	0.0375
H								0.1807	0.1192	0.185	0.1666	0.1179

Cell Index at: 59:31:10

	1	2	3	4	5	6	7	8	9	10	11	12
A								-0.0031	0.0082	0.0298	0.0054	0.0402
B								0.037	0.0276	0.0418	0.0681	0.066
C								0.185	0.185	0.2791	0.2778	0.2205
D								0.1843	0.2508	0.2379	0.2523	0.2355
E								0.098	0.0904	0.1151	0.108	0.0783
F								0.2463	0.2311	0.2619	0.2353	0.2314
G								0.0465	0.0519	0.0592	0.0609	0.0358
H								0.1746	0.1175	0.1847	0.1676	0.116

Cell Index at: 59:46:10

	1	2	3	4	5	6	7	8	9	10	11	12
A								-0.0035	0.0038	0.0275	0.0021	0.0386
B								0.0312	0.0333	0.0429	0.065	0.0655
C								0.1819	0.1902	0.2797	0.2807	0.2205
D								0.1904	0.2452	0.2381	0.2458	0.2339
E								0.0965	0.0878	0.1118	0.1069	0.0873
F								0.2439	0.2232	0.2618	0.2291	0.2323
G								0.0405	0.053	0.054	0.0586	0.0337
H								0.1758	0.119	0.1828	0.1669	0.1186

Cell Index at: 60:01:10

	1	2	3	4	5	6	7	8	9	10	11	12
A								-0.0005	0.0055	0.0261	0.0033	0.0387
B								0.038	0.0267	0.0415	0.0647	0.0635
C								0.1874	0.1813	0.2727	0.2895	0.2185
D								0.2074	0.2463	0.2347	0.2409	0.2323
E								0.0895	0.0993	0.1184	0.1127	0.0763
F								0.2397	0.2238	0.2597	0.2318	0.2316
G								0.0371	0.0512	0.0545	0.0599	0.0348
H								0.1776	0.1199	0.1804	0.17	0.118

Cell Index at: 60:16:10

	1	2	3	4	5	6	7	8	9	10	11	12
A								-0.0021	-0.0004	0.0245	0.0041	0.0354
B								0.0336	0.0303	0.0434	0.069	0.0645
C								0.1871	0.1871	0.2826	0.2839	0.2277
D								0.204	0.257	0.2315	0.244	0.2301
E								0.1014	0.0935	0.1117	0.1092	0.0833
F								0.2383	0.2235	0.2661	0.2339	0.232
G								0.0453	0.0504	0.0568	0.0586	0.0391
H								0.1763	0.1174	0.1826	0.1683	0.1213

Cell Index at: 60:31:10

	1	2	3	4	5	6	7	8	9	10	11	12
A								0	0.0027	0.0293	0.0044	0.0371
B								0.0311	0.032	0.0434	0.069	0.0652
C								0.1833	0.1882	0.2812	0.2861	0.2228
D								0.1977	0.2483	0.2356	0.2516	0.2411
E								0.0993	0.0824	0.1143	0.109	0.0798
F								0.2442	0.2256	0.2647	0.2354	0.2312
G								0.0405	0.0479	0.0506	0.0602	0.0406
H								0.1822	0.1177	0.1825	0.168	0.1207

Cell Index at: 60:46:11

	1	2	3	4	5	6	7	8	9	10	11	12
A								0.002	-0.0005	0.0285	0.0027	0.037
B								0.0328	0.0327	0.0443	0.064	0.0624
C								0.1829	0.1866	0.2775	0.2835	0.2277
D								0.1971	0.2533	0.2383	0.258	0.2374
E								0.1043	0.087	0.1091	0.1139	0.09
F								0.2458	0.2268	0.2671	0.2366	0.2318
G								0.0412	0.0483	0.056	0.0612	0.0409
H								0.1785	0.1203	0.185	0.1721	0.1229

Cell Index at: 61:01:11

	1	2	3	4	5	6	7	8	9	10	11	12
A								-0.0008	0.0002	0.0278	0.0038	0.0366
B								0.0333	0.0336	0.0425	0.0637	0.0661
C								0.1824	0.1839	0.2819	0.2849	0.2278
D								0.2037	0.2471	0.2343	0.2571	0.2378
E								0.1031	0.0972	0.1138	0.1134	0.0794
F								0.2449	0.2246	0.2689	0.2403	0.2295
G								0.0394	0.0469	0.0513	0.0598	0.0416
H								0.1772	0.1217	0.1826	0.1741	0.125

Cell Index at: 61:16:12

	1	2	3	4	5	6	7	8	9	10	11	12
A								0.0017	0.0018	0.0318	-0.0002	0.0394
B								0.0352	0.0282	0.0387	0.0645	0.0632
C								0.1884	0.186	0.2785	0.2794	0.2242
D								0.2078	0.2442	0.2412	0.2532	0.2356
E								0.1019	0.0931	0.1145	0.1167	0.0896
F								0.2459	0.2268	0.2683	0.2359	0.224
G								0.0397	0.0479	0.0542	0.0628	0.0399
H								0.18	0.1225	0.1828	0.1735	0.1254

Cell Index at: 61:31:13

	1	2	3	4	5	6	7	8	9	10	11	12
A								-0.001	0.0024	0.0311	0.0012	0.0366
B								0.0367	0.0348	0.0387	0.0649	0.0643
C								0.1871	0.1866	0.2797	0.2859	0.224
D								0.1967	0.24	0.2377	0.2588	0.2455
E								0.0923	0.0914	0.1166	0.1083	0.0822
F								0.2449	0.2332	0.2619	0.2398	0.2287
G								0.0402	0.0488	0.0525	0.0611	0.0433
H								0.1832	0.1238	0.1818	0.17	0.1296

Cell Index at: 61:46:14

	1	2	3	4	5	6	7	8	9	10	11	12
A								0.0001	-0.002	0.0321	0.0009	0.0351
B								0.0391	0.0323	0.0391	0.0601	0.0628
C								0.1905	0.1848	0.2786	0.2888	0.2253
D								0.1981	0.2441	0.2398	0.2527	0.2467
E								0.0979	0.099	0.1119	0.1141	0.0902
F								0.2436	0.2235	0.2625	0.2433	0.2316
G								0.0406	0.0453	0.0559	0.0615	0.0451
H								0.1807	0.125	0.1879	0.1689	0.1274

Cell Index at: 62:01:14

	1	2	3	4	5	6	7	8	9	10	11	12
A								-0.0001	0.0017	0.0299	0.0041	0.035
B								0.0342	0.0317	0.0403	0.0638	0.0672
C								0.1902	0.1854	0.2817	0.2857	0.2309
D								0.2024	0.2538	0.2398	0.2613	0.2361
E								0.0944	0.0929	0.113	0.1112	0.078
F								0.2534	0.2224	0.2668	0.2424	0.2276
G								0.04	0.047	0.0573	0.0619	0.0446
H								0.1814	0.1233	0.1899	0.1711	0.1299

Cell Index at: 62:16:14

	1	2	3	4	5	6	7	8	9	10	11	12
A								0.0018	0.0027	0.0314	0.0045	0.0329
B								0.0361	0.0327	0.0346	0.0662	0.0647
C								0.1959	0.1873	0.2792	0.2926	0.2211
D								0.2057	0.2481	0.2427	0.2594	0.2406
E								0.1008	0.0908	0.1136	0.1072	0.0826
F								0.2533	0.2176	0.265	0.2421	0.232
G								0.0429	0.0523	0.0549	0.0615	0.0466
H								0.1822	0.126	0.1911	0.1721	0.1284

Cell Index at: 62:31:14

	1	2	3	4	5	6	7	8	9	10	11	12
A								-0.0003	0.0038	0.0295	0.0023	0.0265
B								0.0351	0.0337	0.0426	0.0666	0.0602
C								0.1932	0.189	0.283	0.2928	0.2274
D								0.2032	0.253	0.246	0.2571	0.2394
E								0.0962	0.0918	0.1171	0.107	0.0824
F								0.2447	0.2244	0.2693	0.2469	0.225
G								0.0371	0.0454	0.0546	0.0597	0.0475
H								0.1788	0.1266	0.189	0.1732	0.1286

Cell Index at: 62:46:14

	1	2	3	4	5	6	7	8	9	10	11	12
A								-0.0006	0.0042	0.0283	0.0038	0.0236
B								0.0349	0.03	0.0415	0.0683	0.0614
C								0.1935	0.1918	0.2861	0.2911	0.2274
D								0.1979	0.2531	0.2341	0.2611	0.2449
E								0.0987	0.089	0.1188	0.1092	0.0793
F								0.2507	0.2261	0.2672	0.2456	0.2317
G								0.035	0.0447	0.0564	0.0617	0.0465
H								0.1852	0.1298	0.1871	0.1754	0.13

Cell Index at: 63:01:14

	1	2	3	4	5	6	7	8	9	10	11	12
A								0.0008	0.0044	0.0334	0.0044	0.0262
B								0.0383	0.0304	0.0379	0.0686	0.0618
C								0.1988	0.1905	0.2821	0.2925	0.2296
D								0.198	0.2484	0.2471	0.2585	0.2387
E								0.0935	0.0918	0.1108	0.1158	0.0771
F								0.2524	0.2182	0.2665	0.2498	0.2273
G								0.0356	0.0465	0.0552	0.0592	0.0477
H								0.1863	0.128	0.1913	0.1762	0.1301

Cell Index at: 63:16:15

	1	2	3	4	5	6	7	8	9	10	11	12
A								0.004	0.0069	0.0311	0.0032	0.0272
B								0.0286	0.0307	0.0372	0.0712	0.0629
C								0.1995	0.189	0.289	0.2902	0.2324
D								0.1912	0.2469	0.2467	0.2608	0.2424
E								0.0976	0.0869	0.1088	0.1201	0.0766
F								0.2518	0.2226	0.2694	0.251	0.2268
G								0.0297	0.0449	0.0519	0.0587	0.0443
H								0.1865	0.1328	0.1932	0.1725	0.1324

Cell Index at: 63:31:16

	1	2	3	4	5	6	7	8	9	10	11	12
A								0.0018	0.0016	0.0337	0.0027	0.0269
B								0.0302	0.0338	0.037	0.072	0.0619
C								0.1999	0.1925	0.2863	0.2972	0.226
D								0.1965	0.2477	0.24	0.2598	0.2413
E								0.1005	0.0886	0.1148	0.1119	0.0773
F								0.248	0.2232	0.2726	0.2453	0.2266
G								0.0319	0.0472	0.0515	0.0575	0.047
H								0.1878	0.13	0.1915	0.1777	0.1315

Cell Index at: 63:46:16

	1	2	3	4	5	6	7	8	9	10	11	12
A								0.0055	0.0019	0.0345	0.0031	0.0296
B								0.0345	0.0291	0.0378	0.0713	0.0634
C								0.1955	0.1962	0.2863	0.2991	0.2275
D								0.1995	0.2587	0.249	0.2716	0.2417
E								0.0965	0.0875	0.1111	0.1044	0.0782
F								0.2542	0.224	0.2682	0.2454	0.2301
G								0.035	0.0458	0.0537	0.0598	0.0434

	1	2	3	4	5	6	7	8	9	10	11	12
H								0.1853	0.1292	0.1924	0.1803	0.1334

Cell Index at: 64:01:16

	1	2	3	4	5	6	7	8	9	10	11	12
A								0.0024	0.0019	0.0347	0.0037	0.0309
B								0.0317	0.0293	0.0401	0.068	0.0641
C								0.1934	0.1932	0.2887	0.2939	0.2291
D								0.2032	0.2456	0.2467	0.2612	0.2461
E								0.1006	0.0928	0.1137	0.1096	0.0815
F								0.2557	0.2206	0.2642	0.247	0.2314
G								0.0324	0.0433	0.0537	0.0525	0.0474
H								0.1873	0.1296	0.1948	0.1811	0.1334

Cell Index at: 64:16:16

	1	2	3	4	5	6	7	8	9	10	11	12
A								0.0038	-0.0003	0.0336	0.0024	0.0294
B								0.0285	0.0309	0.0389	0.0667	0.0625
C								0.1981	0.1925	0.2871	0.2943	0.2286
D								0.1963	0.2484	0.2383	0.2687	0.2408
E								0.1003	0.0924	0.1124	0.1101	0.0783
F								0.2492	0.2157	0.2665	0.2472	0.2329
G								0.0355	0.0465	0.0508	0.0505	0.045
H								0.1895	0.1302	0.1955	0.1808	0.1359

Cell Index at: 64:31:16

	1	2	3	4	5	6	7	8	9	10	11	12
A								0.0032	-0.0018	0.0334	0.0038	0.0309
B								0.0312	0.0258	0.0394	0.0688	0.0631
C								0.1951	0.1922	0.2908	0.293	0.2237
D								0.1988	0.2579	0.2529	0.2681	0.2432
E								0.0918	0.0836	0.1096	0.1029	0.0788
F								0.2483	0.2107	0.2732	0.252	0.2331
G								0.0337	0.0492	0.0512	0.0525	0.0446
H								0.1899	0.1287	0.1961	0.1838	0.1362

Cell Index at: 64:46:16

	1	2	3	4	5	6	7	8	9	10	11	12
A								0.0072	-0.0024	0.033	0.0035	0.0296
B								0.0321	0.0281	0.0386	0.0658	0.0646
C								0.1925	0.1967	0.2994	0.2943	0.2257
D								0.1952	0.2542	0.2513	0.261	0.2417
E								0.0942	0.087	0.1199	0.1095	0.0742
F								0.2511	0.2222	0.2694	0.2488	0.2383
G								0.033	0.0486	0.0555	0.0549	0.042
H								0.192	0.1246	0.1947	0.1844	0.1366

Cell Index at: 65:01:16

	1	2	3	4	5	6	7	8	9	10	11	12
A								0.0064	-0.0011	0.0323	0.0058	0.0256
B								0.0308	0.0252	0.0389	0.068	0.0602
C								0.1953	0.197	0.2912	0.2972	0.2245
D								0.1957	0.2579	0.2594	0.2673	0.2458
E								0.0874	0.0931	0.1136	0.1046	0.08
F								0.2563	0.2215	0.2702	0.2494	0.2399
G								0.037	0.0454	0.0571	0.0567	0.0466
H								0.1928	0.1272	0.1987	0.1836	0.139

Cell Index at: 65:16:17

	1	2	3	4	5	6	7	8	9	10	11	12
A								0.0076	-0.0005	0.0342	0.0047	0.023
B								0.0327	0.0275	0.0356	0.0703	0.0604
C								0.1964	0.1949	0.2919	0.2954	0.227
D								0.1946	0.2597	0.2485	0.2646	0.2354
E								0.092	0.0929	0.1149	0.114	0.0732
F								0.2446	0.2256	0.2678	0.2481	0.2355
G								0.0345	0.0473	0.0567	0.0554	0.0454
H								0.1942	0.1267	0.1995	0.1856	0.1366

Cell Index at: 65:31:18

	1	2	3	4	5	6	7	8	9	10	11	12
A								0.008	-0.0011	0.0344	0.0071	0.0261
B								0.0337	0.0286	0.0425	0.0667	0.0647
C								0.1915	0.1972	0.2908	0.2943	0.2281
D								0.1919	0.2607	0.2545	0.269	0.2454
E								0.0953	0.0839	0.1084	0.1149	0.0755
F								0.2447	0.2228	0.266	0.2549	0.2372
G								0.0355	0.0481	0.0571	0.0572	0.045
H								0.1942	0.1233	0.1977	0.1871	0.141

Cell Index at: 65:46:17

	1	2	3	4	5	6	7	8	9	10	11	12
A								0.0066	-0.0021	0.0344	0.0044	0.0277
B								0.0338	0.0268	0.041	0.0653	0.0616
C								0.2003	0.194	0.2895	0.2994	0.2268
D								0.1949	0.2702	0.2478	0.2711	0.241
E								0.0972	0.091	0.0987	0.1188	0.0785
F								0.2507	0.2234	0.2707	0.256	0.2402
G								0.0389	0.0449	0.0585	0.0548	0.0442
H								0.1937	0.1219	0.199	0.1874	0.1391

Cell Index at: 66:01:17

	1	2	3	4	5	6	7	8	9	10	11	12
A								0.008	-0.001	0.0344	0.0101	0.027
B								0.037	0.027	0.0379	0.067	0.059
C								0.2029	0.1967	0.2929	0.2927	0.2293
D								0.2034	0.2562	0.2582	0.2724	0.2455
E								0.1005	0.0881	0.1031	0.1195	0.0766
F								0.2536	0.2185	0.2685	0.2528	0.2433
G								0.04	0.0497	0.0589	0.0525	0.0454
H								0.1961	0.122	0.2002	0.1889	0.1428

Cell Index at: 66:16:17

	1	2	3	4	5	6	7	8	9	10	11	12
A								0.0075	-0.0037	0.0322	0.0039	0.0309
B								0.0353	0.0219	0.0368	0.0648	0.062
C								0.2009	0.1952	0.2941	0.2915	0.2292
D								0.1989	0.2697	0.2625	0.2681	0.2439
E								0.0931	0.0824	0.1046	0.1144	0.071
F								0.2488	0.2283	0.2694	0.2628	0.2488
G								0.0423	0.0453	0.0569	0.055	0.0451
H								0.1963	0.124	0.2	0.1866	0.1401

Cell Index at: 66:31:16

	1	2	3	4	5	6	7	8	9	10	11	12
A								0.0043	-0.0007	0.0336	0.0039	0.0297
B								0.0388	0.0258	0.0376	0.0698	0.0623
C								0.2054	0.1948	0.2906	0.2984	0.2234
D								0.1991	0.2575	0.2641	0.2645	0.2511
E								0.0867	0.0884	0.1046	0.1197	0.0781
F								0.2537	0.2297	0.2658	0.2581	0.2434
G								0.0359	0.0488	0.0574	0.051	0.0385
H								0.1981	0.1241	0.2005	0.1911	0.1448

Cell Index at: 66:46:17

	1	2	3	4	5	6	7	8	9	10	11	12
A								0.0066	-0.0038	0.0335	0.0025	0.0332
B								0.038	0.0279	0.0405	0.0665	0.0603
C								0.2067	0.1984	0.2938	0.2983	0.2278
D								0.204	0.2686	0.2607	0.2653	0.244
E								0.0995	0.0953	0.1043	0.1135	0.0731
F								0.2476	0.2283	0.2658	0.2541	0.2421

	1	2	3	4	5	6	7	8	9	10	11	12
G								0.0363	0.0444	0.0592	0.056	0.0394
H								0.1977	0.1292	0.2042	0.1894	0.1437

Cell Index at: 67:01:18

	1	2	3	4	5	6	7	8	9	10	11	12
A								0.0055	-0.0026	0.0325	0.0055	0.0274
B								0.0366	0.0248	0.0402	0.0682	0.0629
C								0.201	0.2005	0.2944	0.2979	0.2261
D								0.2004	0.2695	0.2646	0.2656	0.2472
E								0.094	0.0893	0.1093	0.1214	0.0757
F								0.2508	0.2287	0.2661	0.2543	0.2431
G								0.0352	0.0501	0.0579	0.0551	0.0339
H								0.1982	0.1266	0.2025	0.1919	0.1414

Cell Index at: 67:16:19

	1	2	3	4	5	6	7	8	9	10	11	12
A								0.0115	-0.0011	0.0323	0.0046	0.0303
B								0.0398	0.03	0.0427	0.065	0.0614
C								0.2001	0.1972	0.2955	0.2927	0.2273
D								0.2057	0.2631	0.2655	0.2659	0.2439
E								0.098	0.0917	0.1144	0.117	0.0775
F								0.2582	0.2275	0.2683	0.2521	0.2428
G								0.032	0.0447	0.0624	0.0525	0.0339
H								0.2025	0.1301	0.2047	0.1939	0.1428

Cell Index at: 67:31:17

	1	2	3	4	5	6	7	8	9	10	11	12
A								0.004	-0.0014	0.0328	0.0054	0.0262
B								0.0392	0.0277	0.0401	0.0661	0.0616
C								0.2014	0.2021	0.2941	0.2984	0.2217
D								0.2038	0.2643	0.2704	0.2676	0.25
E								0.0977	0.0919	0.1098	0.115	0.0798
F								0.2535	0.2316	0.2663	0.2583	0.2418
G								0.0395	0.0496	0.0618	0.0535	0.0399
H								0.2047	0.1298	0.2011	0.1895	0.1438

Cell Index at: 67:46:17

	1	2	3	4	5	6	7	8	9	10	11	12
A								0.0052	-0.0018	0.0323	0.0048	0.0306
B								0.0419	0.0261	0.0429	0.0653	0.0672
C								0.1966	0.1965	0.2941	0.2951	0.228
D								0.2044	0.2673	0.2683	0.2673	0.2545
E								0.0965	0.0905	0.1128	0.118	0.0767
F								0.2512	0.2335	0.2685	0.2508	0.246
G								0.0343	0.0544	0.0628	0.0551	0.0375
H								0.2073	0.126	0.2062	0.1895	0.1445

Cell Index at: 68:01:18

	1	2	3	4	5	6	7	8	9	10	11	12
A								0.0045	-0.0012	0.0345	0.0052	0.0273
B								0.0372	0.0256	0.0379	0.0684	0.0608
C								0.2056	0.1971	0.297	0.3004	0.2209
D								0.1959	0.2695	0.2641	0.2671	0.2473
E								0.0983	0.0948	0.112	0.1177	0.0773
F								0.2455	0.2346	0.2666	0.258	0.2404
G								0.0333	0.0491	0.0605	0.0505	0.0362
H								0.2069	0.1254	0.2043	0.1895	0.147

Cell Index at: 68:16:19

	1	2	3	4	5	6	7	8	9	10	11	12
A								0.0049	-0.0018	0.036	0.0071	0.029
B								0.0372	0.028	0.0421	0.0666	0.0638
C								0.2	0.2008	0.2982	0.2965	0.2233
D								0.1945	0.2679	0.2626	0.275	0.2471
E								0.094	0.0908	0.1045	0.1111	0.0752
F								0.2528	0.2359	0.2744	0.2586	0.2458
G								0.0387	0.0514	0.0619	0.0519	0.0344
H								0.2101	0.1283	0.2014	0.1935	0.1484

Cell Index at: 68:31:20

	1	2	3	4	5	6	7	8	9	10	11	12
A								0.0055	-0.0001	0.0336	0.0047	0.0287
B								0.0396	0.0259	0.0399	0.0639	0.065
C								0.2043	0.2007	0.2954	0.2979	0.2251
D								0.2021	0.2713	0.2674	0.2746	0.2491
E								0.1009	0.0885	0.1087	0.1143	0.0777
F								0.2558	0.2372	0.2717	0.2576	0.2453
G								0.0383	0.0488	0.063	0.0522	0.0373
H								0.2083	0.1282	0.195	0.1925	0.1483

Cell Index at: 68:46:21

	1	2	3	4	5	6	7	8	9	10	11	12
A								0.0079	0.0003	0.0331	0.0044	0.0266
B								0.0403	0.0241	0.0396	0.0638	0.0634
C								0.2063	0.1991	0.2969	0.2955	0.2208
D								0.199	0.2633	0.2652	0.2737	0.245
E								0.1071	0.0841	0.1148	0.1087	0.0773
F								0.2421	0.239	0.2687	0.2628	0.2446
G								0.0391	0.0499	0.063	0.0537	0.0348
H								0.209	0.1255	0.1923	0.1955	0.1459

Cell Index at: 69:01:22

	1	2	3	4	5	6	7	8	9	10	11	12
A								0.0063	-0.0033	0.0338	0.0073	0.028
B								0.0416	0.0273	0.0408	0.0645	0.066
C								0.2025	0.2011	0.304	0.3007	0.226
D								0.1969	0.2736	0.2725	0.2746	0.254
E								0.1036	0.0839	0.1113	0.1076	0.0773
F								0.2463	0.2367	0.2679	0.258	0.2478
G								0.0364	0.0459	0.0631	0.0527	0.0341
H								0.2115	0.127	0.1977	0.1924	0.1492

Cell Index at: 69:16:23

	1	2	3	4	5	6	7	8	9	10	11	12
A								0.0031	-0.0028	0.0332	0.0047	0.0268
B								0.0393	0.0254	0.038	0.067	0.0597
C								0.2078	0.1981	0.3021	0.3078	0.2282
D								0.1954	0.2683	0.2711	0.2723	0.2458
E								0.0961	0.0886	0.1141	0.1195	0.0744
F								0.2494	0.2391	0.2656	0.2618	0.2519
G								0.0354	0.0447	0.0616	0.0561	0.0351
H								0.2082	0.1278	0.1998	0.1929	0.1513

Cell Index at: 69:31:24

	1	2	3	4	5	6	7	8	9	10	11	12
A								0.0045	-0.0047	0.0321	0.0048	0.0289
B								0.0425	0.0268	0.0422	0.0673	0.0612
C								0.2081	0.2014	0.3065	0.305	0.2244
D								0.1999	0.2779	0.2691	0.273	0.2532
E								0.1063	0.0896	0.1056	0.1182	0.0717
F								0.2483	0.2325	0.2766	0.2603	0.2479
G								0.0318	0.045	0.0619	0.053	0.0335
H								0.2111	0.1284	0.1953	0.1937	0.1538

Cell Index at: 69:46:23

	1	2	3	4	5	6	7	8	9	10	11	12
A								0.0024	-0.0034	0.0332	0.0085	0.0246
B								0.0394	0.0206	0.041	0.0658	0.0603
C								0.2072	0.2072	0.3056	0.3095	0.2254
D								0.1978	0.2731	0.2685	0.2694	0.2518
E								0.1087	0.0824	0.1116	0.1203	0.0752

F								0.2473	0.2319	0.2791	0.265	0.2461
G								0.033	0.0459	0.0608	0.0559	0.0309
H								0.2113	0.1288	0.2003	0.1943	0.1516

Cell Index at: 70:01:24

	1	2	3	4	5	6	7	8	9	10	11	12
A								0.0026	-0.0037	0.0341	0.0039	0.0272
B								0.0393	0.0264	0.044	0.0688	0.0595
C								0.2051	0.2044	0.3012	0.3051	0.2294
D								0.1932	0.2686	0.2727	0.2807	0.2552
E								0.1002	0.0801	0.1152	0.1112	0.0753
F								0.2507	0.2357	0.2761	0.2593	0.2489
G								0.0326	0.0434	0.0587	0.0554	0.0309
H								0.2138	0.1291	0.1967	0.1942	0.1507

Cell Index at: 70:16:24

	1	2	3	4	5	6	7	8	9	10	11	12
A								0.0038	-0.0055	0.0301	0.0076	0.0259
B								0.0431	0.0278	0.0398	0.0724	0.0605
C								0.2092	0.2028	0.3117	0.3026	0.2306
D								0.1997	0.2734	0.2769	0.2764	0.2478
E								0.0991	0.0839	0.1133	0.1167	0.0772
F								0.2561	0.2326	0.2801	0.2655	0.2512
G								0.033	0.0432	0.0585	0.0533	0.0318
H								0.2125	0.1276	0.1958	0.1966	0.1511

Cell Index at: 70:31:25

	1	2	3	4	5	6	7	8	9	10	11	12
A								0.0037	-0.0028	0.0338	0.0062	0.0292
B								0.0434	0.0293	0.0418	0.0707	0.0584
C								0.211	0.2078	0.3077	0.3062	0.2248
D								0.1978	0.2823	0.2772	0.2746	0.2469
E								0.1072	0.0938	0.1118	0.1097	0.0779
F								0.2546	0.2325	0.2786	0.2694	0.2552
G								0.0253	0.0441	0.0591	0.0563	0.0304
H								0.2128	0.1257	0.196	0.1979	0.1521

Cell Index at: 70:46:25

	1	2	3	4	5	6	7	8	9	10	11	12
A								-0.0028	-0.0007	0.031	0.005	0.0231
B								0.0433	0.0215	0.0441	0.0706	0.0608
C								0.2094	0.1999	0.3042	0.3101	0.2254
D								0.1958	0.277	0.2775	0.283	0.2507
E								0.1091	0.0868	0.1065	0.1164	0.073
F								0.253	0.2381	0.2797	0.2666	0.247
G								0.0275	0.0434	0.0623	0.054	0.0314
H								0.2118	0.1269	0.1945	0.1976	0.1514

Cell Index at: 71:01:26

	1	2	3	4	5	6	7	8	9	10	11	12
A								0.0026	-0.0047	0.0332	0.005	0.0275
B								0.0415	0.0272	0.0409	0.0697	0.0655
C								0.2136	0.2071	0.3057	0.308	0.2257
D								0.1954	0.2723	0.2792	0.2798	0.2528
E								0.1119	0.0844	0.1154	0.11	0.0732
F								0.2522	0.2379	0.2834	0.2694	0.2526
G								0.0286	0.0477	0.0589	0.0555	0.0308
H								0.2092	0.129	0.1952	0.1982	0.1525

Cell Index at: 71:16:27

	1	2	3	4	5	6	7	8	9	10	11	12
A								0.0013	-0.0023	0.0311	0.0051	0.0266
B								0.0442	0.0263	0.0402	0.0718	0.0573
C								0.2056	0.2046	0.3059	0.3067	0.223
D								0.2029	0.2729	0.2845	0.2768	0.2481
E								0.1099	0.0855	0.1227	0.1082	0.0746
F								0.2593	0.2323	0.2796	0.2675	0.2558
G								0.0271	0.0419	0.0583	0.0537	0.0338
H								0.2118	0.1311	0.1962	0.1987	0.1534

Cell Index at: 71:31:28

	1	2	3	4	5	6	7	8	9	10	11	12
A								0.0033	-0.0045	0.0344	0.0052	0.0235
B								0.0478	0.0257	0.0439	0.0762	0.058
C								0.2134	0.2085	0.3043	0.305	0.2307
D								0.1919	0.2772	0.2741	0.287	0.2522
E								0.1099	0.0921	0.1179	0.109	0.0814
F								0.2514	0.2316	0.2786	0.267	0.2548
G								0.0323	0.0376	0.0549	0.0509	0.0315
H								0.2119	0.1296	0.1923	0.1979	0.1508

Cell Index at: 71:46:29

	1	2	3	4	5	6	7	8	9	10	11	12
A								-0.0023	-0.0028	0.0306	0.0056	0.0277
B								0.0519	0.0251	0.0462	0.0764	0.0557
C								0.2135	0.1991	0.2927	0.3069	0.2274
D								0.196	0.2823	0.2848	0.2814	0.2524
E								0.1082	0.0876	0.1268	0.1129	0.0762
F								0.2537	0.2246	0.2804	0.265	0.2549
G								0.0325	0.0419	0.0604	0.0474	0.0338
H								0.2097	0.1337	0.1988	0.198	0.1534

Cell Index at: 72:01:29

	1	2	3	4	5	6	7	8	9	10	11	12
A								0.0012	-0.0055	0.0327	0.0066	0.0261
B								0.0477	0.028	0.0433	0.0754	0.0556
C								0.2119	0.208	0.3049	0.3067	0.2272
D								0.1928	0.2821	0.2827	0.2841	0.249
E								0.1158	0.0797	0.1161	0.1097	0.0774
F								0.2541	0.2281	0.2761	0.2624	0.2568
G								0.0343	0.0313	0.0624	0.0528	0.0325
H								0.2096	0.1325	0.198	0.1985	0.1597

A vs B	0.007764	B vs C	0.000377	C vs D	0.827768	D vs E	0.000221	E vs F	1.1E-06	F vs G	4.55E-08	G vs H	0.000217
A vs C	0.000183	B vs D	6.49E-05	C vs E	0.001242	D vs F	0.895914	E vs G	0.000991	F vs H	0.00347		
A vs D	3.12E-05	B vs E	0.002874	C vs F	0.87988	D vs G	8.56E-05	E vs H	0.002568				
A vs E	6.9E-05	B vs F	7.15E-08	C vs G	0.000411	D vs H	0.009333						
A vs F	1.61E-08	B vs G	0.485343	C vs H	0.030785								
A vs G	0.014106	B vs H	0.0002										
A vs H	5.32E-05												

Cell Index at: 72:16:30

	1	2	3	4	5	6	7	8	9	10	11	12
A								0.0008	-0.0014	0.0306	0.0042	0.0245
B								0.0478	0.0138	0.0458	0.0754	0.0572
C								0.2022	0.2089	0.2953	0.3101	0.2305
D								0.2056	0.2755	0.2826	0.2785	0.2548
E								0.1004	0.0833	0.1155	0.1133	0.0807
F								0.2553	0.2357	0.2796	0.2666	0.2597
G								0.0331	0.0373	0.0598	0.0525	0.031
H								0.2119	0.1324	0.2022	0.2031	0.155

Count	*Sum*	*Average*	*Variance*
5	0.0611	0.01222	0.00027
5	0.25	0.05	0.000303
5	1.2587	0.25174	0.002487
5	1.2907	0.25814	0.001551
5	0.4987	0.09974	0.000381
5	1.2775	0.2555	0.000307
5	0.2133	0.04266	0.000199
5	0.8983	0.17966	0.001053

Cell Index at: 72:31:30

	1	2	3	4	5	6	7	8	9	10	11	12
A								-0.0005	-0.0043	0.0351	0.003	0.0273
B								0.0481	0.0202	0.0422	0.0748	0.06
C								0.2077	0.2063	0.2963	0.3142	0.2293
D								0.1982	0.2749	0.2814	0.2669	0.2547
E								0.1048	0.0865	0.1084	0.1117	0.0816
F								0.2587	0.2375	0.2765	0.2665	0.2574
G								0.0343	0.037	0.0646	0.0539	0.0343
H								0.2128	0.1373	0.2043	0.2012	0.1574

SS	*df*	*MS*	*F*	*P-value*	*F crit*
0.383849	7	0.054836	66.9754	2.63E-17	2.312741
0.0262	32	0.000819			
0.410049	39				

Cell Index at: 72:46:31

	1	2	3	4	5	6	7	8	9	10	11	12
A								-0.0029	-0.0047	0.0321	0.0034	0.0243
B								0.0441	0.0152	0.0402	0.0763	0.0586
C								0.2111	0.2045	0.3025	0.3166	0.2286

D								0.1953	0.2775	0.2782	0.285	0.2516
E								0.1039	0.0908	0.1176	0.1099	0.0796
F								0.256	0.2382	0.2747	0.2726	0.2554
G								0.0369	0.0409	0.0648	0.0514	0.0368
H								0.2129	0.1349	0.2053	0.2045	0.1549

Cell Index at: 73:01:32

	1	2	3	4	5	6	7	8	9	10	11	12
A								-0.0032	-0.0011	0.0327	0.0023	0.0242
B								0.0487	0.02	0.0398	0.0761	0.0596
C								0.2128	0.2111	0.3005	0.3155	0.2261
D								0.199	0.2742	0.2854	0.2774	0.2608
E								0.1055	0.0887	0.1125	0.1033	0.0837
F								0.2641	0.2451	0.2767	0.2703	0.2542
G								0.037	0.0359	0.0632	0.0509	0.0347
H								0.2143	0.138	0.2052	0.2036	0.1575

Cell Index at: 73:16:33

	1	2	3	4	5	6	7	8	9	10	11	12
A								0.0033	-0.0031	0.0365	0.0073	0.0221
B								0.0473	0.0165	0.0375	0.0752	0.0576
C								0.2035	0.2043	0.3025	0.3169	0.2251
D								0.1995	0.279	0.2841	0.2911	0.2535
E								0.112	0.0925	0.1141	0.1079	0.0858
F								0.2618	0.2462	0.2774	0.2696	0.2544
G								0.036	0.0383	0.0655	0.049	0.0378
H								0.2135	0.1353	0.2026	0.1997	0.1547

Cell Index at: 73:31:33

	1	2	3	4	5	6	7	8	9	10	11	12
A								0.0028	-0.0027	0.0333	0.0044	0.0221
B								0.0463	0.0233	0.0395	0.0757	0.0585
C								0.2097	0.2073	0.3011	0.32	0.2318
D								0.2033	0.2759	0.2887	0.2801	0.2585
E								0.1125	0.0814	0.1146	0.1104	0.076
F								0.2586	0.2487	0.2812	0.2704	0.256
G								0.0348	0.0383	0.0613	0.0531	0.0345
H								0.2133	0.1369	0.2046	0.2042	0.1577

Cell Index at: 73:46:34

	1	2	3	4	5	6	7	8	9	10	11	12
A								0.0027	-0.0034	0.0319	0.0023	0.0226
B								0.0456	0.0207	0.0403	0.0789	0.054
C								0.2079	0.2035	0.3056	0.3205	0.2291
D								0.1992	0.2779	0.2778	0.2843	0.2607
E								0.1026	0.0879	0.1133	0.1058	0.0809
F								0.2585	0.2428	0.2786	0.2695	0.2573
G								0.0342	0.0371	0.0643	0.0502	0.0354
H								0.2168	0.1349	0.2026	0.2034	0.1555

Cell Index at: 74:01:35

	1	2	3	4	5	6	7	8	9	10	11	12
A								0.0049	-0.0011	0.0349	0.0066	0.0233
B								0.0484	0.0206	0.0391	0.0774	0.0556
C								0.214	0.2081	0.3073	0.3202	0.2318
D								0.1995	0.2819	0.2835	0.2892	0.2525
E								0.1122	0.0861	0.1238	0.1119	0.0783
F								0.2602	0.245	0.2804	0.2688	0.2654
G								0.0309	0.037	0.0624	0.0492	0.0361
H								0.2147	0.1344	0.2091	0.2025	0.1575

Cell Index at: 74:16:35

	1	2	3	4	5	6	7	8	9	10	11	12
A								0.0079	-0.0062	0.0299	0.0079	0.022
B								0.0487	0.0203	0.0412	0.0748	0.052
C								0.2111	0.207	0.3072	0.3254	0.2301
D								0.1968	0.2839	0.2808	0.291	0.2559
E								0.1072	0.0819	0.1198	0.1139	0.0798
F								0.2606	0.2445	0.2768	0.2689	0.2638
G								0.0305	0.0371	0.0653	0.0533	0.0345
H								0.2178	0.1324	0.2106	0.2017	0.159

Cell Index at: 74:31:35

	1	2	3	4	5	6	7	8	9	10	11	12
A								0.0081	-0.006	0.0334	0.0031	0.0253
B								0.0457	0.0249	0.0402	0.0777	0.0553
C								0.2138	0.2025	0.3004	0.3177	0.2298
D								0.1999	0.2757	0.286	0.2938	0.2475
E								0.106	0.091	0.1163	0.1123	0.0803
F								0.2639	0.2444	0.2862	0.269	0.2653
G								0.0293	0.0381	0.0687	0.049	0.0352
H								0.221	0.1359	0.2129	0.2051	0.1617

Cell Index at: 74:46:35

	1	2	3	4	5	6	7	8	9	10	11	12
A								0.0052	-0.0057	0.0321	0.0055	0.0238
B								0.0457	0.0189	0.0363	0.0737	0.0549
C								0.2153	0.2078	0.3106	0.3227	0.2357
D								0.1925	0.2682	0.2856	0.2881	0.254
E								0.1127	0.0916	0.1179	0.112	0.0842
F								0.2648	0.2428	0.2811	0.2731	0.2574
G								0.0288	0.0354	0.0659	0.0501	0.0347
H								0.2225	0.1318	0.2116	0.2041	0.1583

Cell Index at: 75:01:35

	1	2	3	4	5	6	7	8	9	10	11	12
A								0.0074	-0.0029	0.0356	0.0063	0.0253
B								0.0489	0.0147	0.0361	0.0737	0.0554
C								0.2105	0.2115	0.3063	0.3197	0.2348
D								0.1918	0.2741	0.2852	0.2984	0.2507
E								0.1057	0.0898	0.1163	0.1126	0.0801
F								0.2722	0.2397	0.2904	0.271	0.2666
G								0.0196	0.034	0.0634	0.049	0.035
H								0.2209	0.1339	0.2164	0.208	0.16

Cell Index at: 75:16:36

	1	2	3	4	5	6	7	8	9	10	11	12
A								0.0074	-0.0041	0.0327	0.0046	0.0211
B								0.0484	0.0159	0.0342	0.0772	0.0542
C								0.215	0.2074	0.3022	0.3218	0.232
D								0.2034	0.275	0.2885	0.2954	0.2591
E								0.1114	0.1	0.1133	0.1103	0.0775
F								0.2646	0.2419	0.2887	0.2699	0.267
G								0.0203	0.0351	0.0628	0.0504	0.0338
H								0.2284	0.1317	0.2181	0.2051	0.1579

Cell Index at: 75:31:37

	1	2	3	4	5	6	7	8	9	10	11	12
A								0.0025	-0.0064	0.0353	0.0075	0.0185
B								0.0441	0.0214	0.0352	0.0765	0.059
C								0.2141	0.2054	0.3062	0.3208	0.2349
D								0.1965	0.279	0.2785	0.2934	0.2613
E								0.1143	0.0938	0.115	0.1137	0.0757
F								0.2653	0.2413	0.2861	0.2741	0.2649
G								0.0194	0.0352	0.0653	0.0496	0.0345
H								0.224	0.134	0.2171	0.2055	0.1566

Cell Index at: 75:46:38

	1	2	3	4	5	6	7	8	9	10	11	12
A								0.0032	-0.0031	0.0331	0.0061	0.0187
B								0.0436	0.0263	0.0349	0.0745	0.0583

	1	2	3	4	5	6	7	8	9	10	11	12
C								0.2154	0.2038	0.3047	0.3189	0.2375
D								0.1996	0.2738	0.2828	0.299	0.2552
E								0.1123	0.095	0.1215	0.1063	0.0748
F								0.267	0.2474	0.287	0.2711	0.268
G								0.023	0.0382	0.062	0.0482	0.0342
H								0.2256	0.1363	0.2207	0.2056	0.1579

Cell Index at: 76:01:38

	1	2	3	4	5	6	7	8	9	10	11	12
A								0.0039	-0.0042	0.033	0.0062	0.019
B								0.0435	0.0242	0.0363	0.0726	0.0608
C								0.2054	0.2053	0.3067	0.3143	0.2366
D								0.1946	0.2796	0.2895	0.3013	0.2577
E								0.1132	0.0894	0.1064	0.1186	0.0763
F								0.2697	0.2468	0.2889	0.2757	0.2633
G								0.0199	0.0368	0.0614	0.0511	0.028
H								0.2275	0.1355	0.2192	0.2052	0.1583

Cell Index at: 76:16:39

	1	2	3	4	5	6	7	8	9	10	11	12
A								0.0045	-0.0053	0.0316	0.0051	0.0176
B								0.0421	0.023	0.0342	0.0716	0.0539
C								0.2111	0.2045	0.3025	0.3204	0.2364
D								0.2036	0.2753	0.2914	0.303	0.2537
E								0.1125	0.0911	0.1159	0.1086	0.0699
F								0.2636	0.2384	0.2891	0.2794	0.2632
G								0.0184	0.0375	0.0667	0.045	0.0268
H								0.2258	0.136	0.222	0.2095	0.1621

Cell Index at: 76:31:39

	1	2	3	4	5	6	7	8	9	10	11	12
A								0.0022	-0.0043	0.0279	0.0068	0.0158
B								0.0423	0.0252	0.0327	0.0776	0.0545
C								0.202	0.2041	0.3053	0.3265	0.2366
D								0.2008	0.2795	0.2928	0.3031	0.2604
E								0.1025	0.0892	0.1114	0.1123	0.0696
F								0.267	0.2345	0.2877	0.277	0.2673
G								0.0216	0.0346	0.0627	0.0441	0.0262
H								0.2294	0.1373	0.22	0.2087	0.1645

Cell Index at: 76:46:40

	1	2	3	4	5	6	7	8	9	10	11	12
A								0.0017	-0.0036	0.0305	0.0056	0.0152
B								0.0452	0.0211	0.034	0.0744	0.0533
C								0.2085	0.2014	0.3051	0.3235	0.2375
D								0.196	0.2763	0.2842	0.2999	0.2556
E								0.1096	0.0832	0.1134	0.1176	0.0701
F								0.2727	0.2394	0.2916	0.2813	0.2661
G								0.0185	0.0394	0.0609	0.0465	0.0275
H								0.2306	0.1373	0.2221	0.2104	0.1634

Cell Index at: 77:01:40

	1	2	3	4	5	6	7	8	9	10	11	12
A								0.0016	-0.0049	0.0333	0.0081	0.0081
B								0.0431	0.0241	0.0318	0.074	0.0525
C								0.2093	0.1999	0.3127	0.3253	0.2427
D								0.1951	0.2758	0.29	0.2979	0.2645
E								0.1001	0.0921	0.1147	0.1074	0.0699
F								0.2735	0.2431	0.2897	0.2815	0.2656
G								0.0191	0.0394	0.0601	0.0488	0.0277
H								0.2317	0.1351	0.2231	0.2069	0.1656

Cell Index at: 77:16:41

	1	2	3	4	5	6	7	8	9	10	11	12
A								-0.0017	-0.0036	0.0313	0.008	0.0094
B								0.0457	0.0235	0.0316	0.0775	0.0506
C								0.213	0.2037	0.3078	0.3236	0.247
D								0.2048	0.2816	0.2827	0.2999	0.2589
E								0.1068	0.0927	0.1088	0.1091	0.0669
F								0.2733	0.241	0.2843	0.2844	0.2686
G								0.0191	0.0403	0.0629	0.0494	0.0291
H								0.2304	0.1382	0.2236	0.2139	0.1624

Cell Index at: 77:31:41

	1	2	3	4	5	6	7	8	9	10	11	12
A								0.0003	-0.0046	0.0305	0.0092	0.01
B								0.0445	0.0253	0.0329	0.0782	0.0539
C								0.2126	0.2033	0.307	0.3242	0.2418
D								0.1975	0.2792	0.2896	0.3002	0.2555
E								0.1067	0.0893	0.108	0.1108	0.0655
F								0.2686	0.2422	0.2939	0.2781	0.2724
G								0.018	0.0396	0.0613	0.0479	0.0261
H								0.2287	0.1383	0.2204	0.2133	0.164

Cell Index at: 77:46:42

	1	2	3	4	5	6	7	8	9	10	11	12
A								-0.0006	-0.0055	0.0323	0.0062	0.0104
B								0.0468	0.0217	0.031	0.072	0.0542
C								0.2186	0.2067	0.3089	0.3288	0.2425
D								0.1958	0.2744	0.2932	0.2897	0.2569
E								0.1027	0.0905	0.1142	0.1139	0.0659
F								0.2811	0.2379	0.2931	0.2856	0.2699
G								0.0209	0.0362	0.0609	0.0478	0.0283
H								0.2329	0.1355	0.2174	0.2096	0.1658

Cell Index at: 78:01:43

	1	2	3	4	5	6	7	8	9	10	11	12
A								-0.002	-0.0066	0.0332	0.0047	0.0149
B								0.0455	0.0276	0.0324	0.0755	0.0515
C								0.2085	0.2036	0.3114	0.3274	0.2474
D								0.198	0.2875	0.2963	0.299	0.2541
E								0.1005	0.0874	0.1129	0.1132	0.0719
F								0.2685	0.2374	0.2965	0.2851	0.2722
G								0.0256	0.0404	0.056	0.0488	0.0237
H								0.2335	0.1326	0.2189	0.2119	0.1689

Cell Index at: 78:16:43

	1	2	3	4	5	6	7	8	9	10	11	12
A								-0.0029	-0.0066	0.0321	0.0061	0.0115
B								0.0467	0.0231	0.028	0.0755	0.0491
C								0.2085	0.2052	0.3154	0.3295	0.2449
D								0.195	0.2818	0.2957	0.2945	0.2605
E								0.1072	0.0877	0.1149	0.1089	0.0682
F								0.2734	0.2472	0.3044	0.2801	0.2792
G								0.0207	0.0423	0.0618	0.0468	0.0302
H								0.2343	0.1333	0.2189	0.2129	0.1662

Cell Index at: 78:31:44

	1	2	3	4	5	6	7	8	9	10	11	12
A								-0.0013	-0.0035	0.0352	0.0066	0.013
B								0.0489	0.0256	0.034	0.0717	0.0488
C								0.2163	0.1982	0.3145	0.3304	0.2446
D								0.192	0.2784	0.2892	0.2908	0.2532
E								0.1	0.0922	0.1117	0.1162	0.0638
F								0.2694	0.2392	0.2999	0.2858	0.276
G								0.0231	0.0397	0.0636	0.0478	0.0293
H								0.236	0.1344	0.2225	0.2115	0.1676

Cell Index at: 78:46:44

	1	2	3	4	5	6	7	8	9	10	11	12
A								-0.0036	-0.0036	0.035	0.0076	0.0124

B								0.0444	0.0265	0.0362	0.0726	0.0513
C								0.2096	0.2043	0.3179	0.3338	0.2421
D								0.1979	0.2818	0.3004	0.3	0.2538
E								0.1055	0.0856	0.1116	0.1134	0.071
F								0.268	0.2451	0.302	0.2975	0.2756
G								0.0235	0.0439	0.059	0.0505	0.0281
H								0.2366	0.1336	0.2198	0.2134	0.17

Cell Index at: 79:01:45

	1	2	3	4	5	6	7	8	9	10	11	12
A								-0.0035	-0.007	0.0319	0.0122	0.0119
B								0.0423	0.0216	0.0317	0.0709	0.0563
C								0.2128	0.2069	0.3134	0.3348	0.2476
D								0.1929	0.2832	0.3081	0.2918	0.2576
E								0.1079	0.0862	0.1196	0.1151	0.0676
F								0.2703	0.2456	0.3038	0.2956	0.2775
G								0.0181	0.0431	0.0617	0.0509	0.0267
H								0.2399	0.1347	0.2237	0.214	0.1686

Cell Index at: 79:16:45

	1	2	3	4	5	6	7	8	9	10	11	12
A								-0.0064	-0.0037	0.035	0.0092	0.0149
B								0.0474	0.0231	0.0279	0.0722	0.0489
C								0.2092	0.2053	0.3144	0.3362	0.2467
D								0.1995	0.2841	0.2982	0.3019	0.2563
E								0.0998	0.09	0.1182	0.1131	0.0655
F								0.2743	0.2399	0.3037	0.2904	0.2785
G								0.0202	0.0375	0.0604	0.0491	0.0311
H								0.2366	0.1381	0.2236	0.2147	0.169

Cell Index at: 79:31:46

	1	2	3	4	5	6	7	8	9	10	11	12
A								-0.0069	-0.0033	0.0353	0.0076	0.0142
B								0.0457	0.0265	0.0303	0.0678	0.0495
C								0.2079	0.2082	0.3124	0.3331	0.2463
D								0.2017	0.2812	0.2936	0.3021	0.2522
E								0.1066	0.0937	0.1181	0.1124	0.0664
F								0.2782	0.2506	0.3019	0.2926	0.2745
G								0.0224	0.038	0.0598	0.0469	0.031
H								0.2378	0.1359	0.2229	0.2123	0.1723

Cell Index at: 79:46:47

	1	2	3	4	5	6	7	8	9	10	11	12
A								-0.0058	-0.003	0.0311	0.009	0.0136
B								0.0444	0.0238	0.0289	0.0689	0.0495
C								0.2137	0.1999	0.3166	0.3347	0.2495
D								0.1989	0.2877	0.301	0.3054	0.2504
E								0.1032	0.0859	0.1124	0.1133	0.0696
F								0.2817	0.2543	0.3102	0.2959	0.2753
G								0.0229	0.0391	0.0612	0.0464	0.032
H								0.2379	0.1405	0.225	0.2098	0.1698

Cell Index at: 80:01:47

	1	2	3	4	5	6	7	8	9	10	11	12
A								-0.0006	-0.0023	0.0314	0.0093	0.0139
B								0.0481	0.0276	0.027	0.07	0.0472
C								0.2136	0.1987	0.3209	0.3382	0.2477
D								0.1991	0.284	0.3002	0.2981	0.263
E								0.1028	0.0939	0.118	0.1085	0.0707
F								0.2796	0.2522	0.3096	0.2928	0.278
G								0.0201	0.038	0.0607	0.0495	0.0308
H								0.2368	0.1362	0.2279	0.213	0.1717

Cell Index at: 80:16:48

	1	2	3	4	5	6	7	8	9	10	11	12
A								-0.0037	-0.0016	0.032	0.0093	0.0107
B								0.0465	0.0284	0.0311	0.0683	0.0478
C								0.2064	0.2032	0.3127	0.3332	0.2509
D								0.1953	0.2868	0.297	0.3129	0.2595
E								0.1009	0.0975	0.1128	0.1019	0.0693
F								0.2752	0.2532	0.3111	0.2965	0.2797
G								0.0237	0.0373	0.0609	0.0478	0.0293
H								0.2394	0.1406	0.2274	0.2109	0.1724

Cell Index at: 80:31:48

	1	2	3	4	5	6	7	8	9	10	11	12
A								-0.0005	-0.0029	0.0308	0.0087	0.013
B								0.0426	0.0291	0.0252	0.0696	0.0504
C								0.2163	0.1994	0.3178	0.3375	0.2514
D								0.1928	0.2913	0.2968	0.3051	0.25
E								0.0981	0.0864	0.1131	0.1088	0.0747
F								0.2812	0.2569	0.3144	0.2949	0.2816
G								0.0199	0.0411	0.057	0.0466	0.0291
H								0.2388	0.1395	0.2264	0.2147	0.1732

Cell Index at: 80:46:49

	1	2	3	4	5	6	7	8	9	10	11	12
A								0.0008	-0.0048	0.032	0.0089	0.0146
B								0.0437	0.0245	0.0303	0.0683	0.0487
C								0.2161	0.1981	0.321	0.3447	0.2491
D								0.1964	0.2861	0.299	0.3068	0.2574
E								0.0987	0.085	0.1127	0.1044	0.0666
F								0.2824	0.254	0.3104	0.294	0.2774
G								0.0182	0.0387	0.0582	0.0417	0.0279
H								0.237	0.142	0.2273	0.217	0.1726

Cell Index at: 81:01:50

	1	2	3	4	5	6	7	8	9	10	11	12
A								-0.0017	-0.0041	0.0336	0.009	0.0098
B								0.0355	0.022	0.0311	0.0682	0.0514
C								0.2126	0.2045	0.3194	0.3377	0.2549
D								0.1977	0.2874	0.3052	0.3077	0.262
E								0.0907	0.0899	0.1154	0.1074	0.0674
F								0.2794	0.2565	0.3101	0.2918	0.2752
G								0.0189	0.0395	0.0613	0.0466	0.0317
H								0.2391	0.1442	0.229	0.216	0.1708

Cell Index at: 81:16:50

	1	2	3	4	5	6	7	8	9	10	11	12
A								0.0036	-0.0066	0.033	0.0102	0.0142
B								0.0422	0.0194	0.0299	0.0632	0.0455
C								0.2122	0.2057	0.3222	0.3351	0.2559
D								0.199	0.2875	0.3032	0.3077	0.2629
E								0.0976	0.0923	0.1167	0.1094	0.0701
F								0.2773	0.2483	0.3214	0.2992	0.2742
G								0.019	0.0359	0.0643	0.0476	0.0328
H								0.2362	0.1405	0.2264	0.2194	0.1724

Cell Index at: 81:31:51

	1	2	3	4	5	6	7	8	9	10	11	12
A								0.0027	-0.0024	0.032	0.0117	0.0121
B								0.0412	0.0217	0.0268	0.0613	0.0497
C								0.2179	0.2006	0.3223	0.332	0.2483
D								0.2046	0.2892	0.3025	0.3074	0.2566
E								0.0988	0.0956	0.1099	0.106	0.0702
F								0.2835	0.2547	0.3168	0.2999	0.277
G								0.0191	0.038	0.0626	0.0465	0.0316
H								0.239	0.1418	0.2295	0.2176	0.1726

Cell Index at: 81:46:52

	1	2	3	4	5	6	7	8	9	10	11	12

A								-0.0006	-0.0029	0.0314	0.0077	0.0159
B								0.0412	0.0231	0.0284	0.0647	0.049
C								0.2203	0.2047	0.3271	0.3353	0.2481
D								0.2014	0.2877	0.3087	0.3167	0.2617
E								0.1003	0.0931	0.1127	0.1072	0.0697
F								0.284	0.2559	0.3177	0.2999	0.2755
G								0.0205	0.036	0.0627	0.0459	0.035
H								0.2381	0.1438	0.2284	0.2165	0.1744

Cell Index at: 82:01:52

	1	2	3	4	5	6	7	8	9	10	11	12
A								0.0019	-0.0047	0.0342	0.0084	0.0133
B								0.0446	0.0163	0.0322	0.0667	0.0513
C								0.2243	0.203	0.3273	0.3383	0.2555
D								0.199	0.2863	0.3063	0.3175	0.264
E								0.1033	0.0897	0.1124	0.1093	0.0661
F								0.2755	0.256	0.3228	0.2996	0.2802
G								0.0222	0.0412	0.0657	0.0473	0.0316
H								0.2403	0.1464	0.2285	0.2132	0.1736

Cell Index at: 82:16:52

	1	2	3	4	5	6	7	8	9	10	11	12
A								0.0024	-0.0042	0.0321	0.0083	0.0137
B								0.0438	0.0203	0.026	0.0633	0.0532
C								0.2166	0.2089	0.3238	0.346	0.2554
D								0.1975	0.2913	0.3085	0.3193	0.2672
E								0.102	0.0931	0.1112	0.1105	0.0741
F								0.2864	0.2576	0.3079	0.3	0.2787
G								0.0191	0.0376	0.0637	0.0475	0.0305
H								0.239	0.1442	0.2303	0.2175	0.1759

Cell Index at: 82:31:52

	1	2	3	4	5	6	7	8	9	10	11	12
A								0.0024	-0.0019	0.0377	0.0082	0.0137
B								0.0377	0.0162	0.0245	0.0616	0.0497
C								0.2204	0.2076	0.3348	0.3436	0.2602
D								0.2021	0.2971	0.3125	0.3186	0.273
E								0.1023	0.0957	0.1073	0.1104	0.0632
F								0.2854	0.2546	0.3199	0.2964	0.2793
G								0.0216	0.0408	0.0597	0.0449	0.0272
H								0.2393	0.1449	0.231	0.2175	0.1763

Cell Index at: 82:46:53

	1	2	3	4	5	6	7	8	9	10	11	12
A								0.005	-0.0032	0.0377	0.0122	0.0144
B								0.0371	0.0155	0.0288	0.0606	0.0512
C								0.2221	0.2001	0.3356	0.3413	0.2588
D								0.2033	0.2865	0.3095	0.3138	0.2654
E								0.1133	0.0991	0.1057	0.1119	0.0661
F								0.2855	0.2587	0.3162	0.3005	0.2787
G								0.0221	0.0399	0.0671	0.0444	0.0255
H								0.2424	0.142	0.2284	0.2177	0.1725

Cell Index at: 83:01:53

	1	2	3	4	5	6	7	8	9	10	11	12
A								0.007	-0.0061	0.0372	0.0081	0.0108
B								0.0374	0.0153	0.0282	0.0614	0.05
C								0.2234	0.2086	0.3379	0.349	0.2605
D								0.1969	0.2893	0.3088	0.3096	0.2709
E								0.0979	0.0913	0.1131	0.1133	0.0688
F								0.2844	0.2562	0.3159	0.3036	0.282
G								0.0224	0.0428	0.0609	0.0466	0.0292
H								0.2451	0.1423	0.2318	0.2143	0.1739

Cell Index at: 83:16:53

	1	2	3	4	5	6	7	8	9	10	11	12
A								0.0052	-0.0068	0.0394	0.0123	0.0126
B								0.0346	0.018	0.0307	0.0617	0.0505
C								0.2256	0.2092	0.338	0.3514	0.2568
D								0.201	0.2927	0.3145	0.3166	0.2707
E								0.1035	0.0915	0.1122	0.1152	0.0652
F								0.2867	0.2607	0.3157	0.3108	0.2763
G								0.0224	0.0402	0.0602	0.0455	0.0233
H								0.2457	0.1462	0.2258	0.2159	0.1755

Cell Index at: 83:31:53

	1	2	3	4	5	6	7	8	9	10	11	12
A								0.0039	-0.0094	0.0381	0.0109	0.0113
B								0.0352	0.0194	0.031	0.0604	0.0557
C								0.2321	0.2027	0.3437	0.3552	0.2595
D								0.2082	0.292	0.3159	0.3153	0.2667
E								0.1066	0.0885	0.113	0.1124	0.0671
F								0.2858	0.2599	0.3324	0.3061	0.2746
G								0.0188	0.0422	0.0565	0.0456	0.0281
H								0.2448	0.151	0.2315	0.2138	0.1742

Cell Index at: 83:46:54

	1	2	3	4	5	6	7	8	9	10	11	12
A								0.0075	-0.0076	0.0404	0.0124	0.0116
B								0.0332	0.0202	0.0304	0.0605	0.0524
C								0.227	0.2071	0.3389	0.3495	0.261
D								0.2079	0.2915	0.3123	0.3216	0.2754
E								0.1079	0.0893	0.1111	0.1117	0.0643
F								0.2876	0.2639	0.3261	0.3084	0.2788
G								0.023	0.0368	0.0567	0.045	0.0228
H								0.2394	0.1498	0.2296	0.216	0.1742

Cell Index at: 84:01:54

	1	2	3	4	5	6	7	8	9	10	11	12
A								0.0048	-0.0098	0.0388	0.0114	0.0094
B								0.0355	0.019	0.0328	0.0604	0.0536
C								0.2271	0.2087	0.339	0.3508	0.265
D								0.2195	0.2973	0.3133	0.3132	0.2691
E								0.1065	0.0843	0.1003	0.1208	0.0641
F								0.289	0.2604	0.3316	0.3105	0.2722
G								0.0178	0.0431	0.0542	0.0485	0.0244
H								0.2437	0.148	0.2317	0.2179	0.1732

Cell Index at: 84:16:54

	1	2	3	4	5	6	7	8	9	10	11	12
A								0.009	-0.0077	0.0376	0.0132	0.0093
B								0.0415	0.0184	0.0304	0.0616	0.0605
C								0.232	0.2113	0.3385	0.3548	0.2647
D								0.2166	0.2891	0.3153	0.3139	0.2732
E								0.095	0.0869	0.1045	0.1143	0.064
F								0.285	0.2639	0.3247	0.3097	0.2759
G								0.0146	0.0409	0.0522	0.0465	0.0273
H								0.2438	0.1493	0.2288	0.218	0.1743

Cell Index at: 84:31:55

	1	2	3	4	5	6	7	8	9	10	11	12
A								0.0073	-0.0077	0.0412	0.0106	0.0091
B								0.0423	0.0132	0.0329	0.0567	0.0614
C								0.2294	0.2101	0.3448	0.351	0.2623
D								0.2125	0.2875	0.3207	0.3216	0.2782
E								0.1023	0.0932	0.1157	0.1081	0.067
F								0.2863	0.2626	0.3323	0.3044	0.2789
G								0.0206	0.0378	0.0485	0.0461	0.0252
H								0.247	0.1527	0.2328	0.2181	0.1768

Cell Index at: 84:46:56

	1	2	3	4	5	6	7	8	9	10	11	12
A								0.0041	-0.0075	0.0427	0.0128	0.0105
B								0.0377	0.0194	0.0314	0.0581	0.0623
C								0.2315	0.2141	0.3402	0.3489	0.2604
D								0.2125	0.2828	0.3135	0.3141	0.279
E								0.1034	0.0915	0.1198	0.1175	0.0684
F								0.2904	0.2599	0.333	0.3096	0.2765
G								0.016	0.044	0.0468	0.0498	0.0287
H								0.2494	0.1493	0.2328	0.213	0.1795

Cell Index at: 85:01:56

	1	2	3	4	5	6	7	8	9	10	11	12
A								0.009	-0.0094	0.0405	0.0127	0.0113
B								0.0394	0.0205	0.0318	0.0586	0.0594
C								0.2303	0.2099	0.3389	0.3551	0.2638
D								0.2159	0.2906	0.3144	0.3172	0.2726
E								0.1059	0.0816	0.1087	0.1122	0.0702
F								0.2889	0.2624	0.3317	0.3177	0.2778
G								0.02	0.0414	0.0483	0.0414	0.0231
H								0.2515	0.1539	0.2332	0.215	0.1797

Cell Index at: 85:16:57

	1	2	3	4	5	6	7	8	9	10	11	12
A								0.0068	-0.0094	0.0414	0.0102	0.0099
B								0.0403	0.0176	0.035	0.0625	0.0595
C								0.2275	0.2146	0.3433	0.3514	0.2632
D								0.214	0.288	0.3098	0.3143	0.2809
E								0.1017	0.087	0.1046	0.0996	0.0653
F								0.287	0.2652	0.331	0.3088	0.2743
G								0.0212	0.0404	0.0538	0.0444	0.0258
H								0.2489	0.1568	0.2315	0.2141	0.1781

Cell Index at: 85:31:58

	1	2	3	4	5	6	7	8	9	10	11	12
A								0.0056	-0.008	0.0416	0.0097	0.0093
B								0.0405	0.0206	0.0333	0.0651	0.0592
C								0.2232	0.2105	0.3443	0.3495	0.2684
D								0.2201	0.2811	0.3087	0.3178	0.2704
E								0.1035	0.0902	0.1093	0.1061	0.0727
F								0.287	0.2622	0.3279	0.314	0.2723
G								0.0222	0.0411	0.0527	0.0503	0.0299
H								0.2546	0.1584	0.2345	0.2134	0.1799

Cell Index at: 85:46:59

	1	2	3	4	5	6	7	8	9	10	11	12
A								0.0072	-0.0079	0.0419	0.0111	0.013
B								0.0387	0.0225	0.0311	0.0617	0.0583
C								0.2264	0.2125	0.3385	0.3491	0.2673
D								0.2201	0.2882	0.3118	0.3196	0.2733
E								0.0985	0.0941	0.1115	0.1083	0.0696
F								0.2882	0.264	0.3333	0.3153	0.2804
G								0.0204	0.0405	0.0532	0.0523	0.0295
H								0.255	0.1605	0.2384	0.2114	0.1801

Cell Index at: 86:02:00

	1	2	3	4	5	6	7	8	9	10	11	12
A								0.008	-0.0039	0.0384	0.007	0.0093
B								0.0386	0.0237	0.0307	0.0639	0.0587
C								0.2269	0.2145	0.3412	0.3546	0.2655
D								0.2183	0.2865	0.3174	0.3174	0.2798
E								0.1011	0.0947	0.1089	0.1037	0.0693
F								0.2894	0.2633	0.3227	0.3114	0.2769
G								0.0203	0.0374	0.0495	0.0476	0.0192
H								0.2521	0.1594	0.2373	0.2138	0.1796

Cell Index at: 86:17:00

	1	2	3	4	5	6	7	8	9	10	11	12
A								0.0048	-0.0062	0.0368	0.0096	0.0099
B								0.0371	0.0215	0.0333	0.0613	0.0609
C								0.2201	0.216	0.3455	0.3528	0.2643
D								0.2164	0.2864	0.3199	0.3176	0.2791
E								0.1044	0.0956	0.1066	0.1073	0.0698
F								0.2966	0.2642	0.326	0.3173	0.2789
G								0.0208	0.0397	0.0529	0.0442	0.0307
H								0.2531	0.161	0.2396	0.2185	0.1798

Cell Index at: 86:32:01

	1	2	3	4	5	6	7	8	9	10	11	12
A								0.0034	-0.0075	0.0394	0.0094	0.0074
B								0.0423	0.0238	0.0316	0.0619	0.0604
C								0.2243	0.2165	0.3507	0.3514	0.2662
D								0.2171	0.2894	0.3194	0.3272	0.2747
E								0.1009	0.0954	0.1074	0.1031	0.0768
F								0.2925	0.2648	0.3241	0.315	0.2841
G								0.019	0.039	0.0566	0.048	0.0274
H								0.253	0.1603	0.2391	0.2205	0.1782

Cell Index at: 86:47:01

	1	2	3	4	5	6	7	8	9	10	11	12
A								0.0044	-0.005	0.0407	0.011	0.0123
B								0.0416	0.0216	0.0315	0.0602	0.059
C								0.22	0.2168	0.3529	0.3475	0.2668
D								0.2148	0.2799	0.3184	0.3188	0.2764
E								0.098	0.0854	0.1083	0.1027	0.0633
F								0.3016	0.2632	0.3262	0.3191	0.2812
G								0.0217	0.0403	0.053	0.0482	0.0324
H								0.2558	0.1606	0.2395	0.2196	0.1828

Cell Index at: 87:02:02

	1	2	3	4	5	6	7	8	9	10	11	12
A								0.0012	-0.005	0.041	0.0107	0.0064
B								0.0433	0.0238	0.0325	0.0584	0.0618
C								0.2218	0.2188	0.3534	0.3632	0.2675
D								0.2227	0.2906	0.324	0.3223	0.2758
E								0.0949	0.0936	0.1118	0.1011	0.062
F								0.2948	0.2618	0.3241	0.3215	0.2796
G								0.0194	0.0402	0.0499	0.0505	0.0302
H								0.2547	0.162	0.2366	0.2195	0.1806

Cell Index at: 87:17:02

	1	2	3	4	5	6	7	8	9	10	11	12
A								0.0002	-0.005	0.0418	0.012	0.009
B								0.0428	0.0224	0.0328	0.0605	0.0595
C								0.2275	0.2214	0.3503	0.3613	0.2695
D								0.217	0.2902	0.3203	0.3191	0.2853
E								0.0988	0.0842	0.1113	0.1013	0.0637
F								0.2987	0.2611	0.3316	0.3269	0.283
G								0.0237	0.0371	0.053	0.049	0.0269
H								0.2517	0.1635	0.2399	0.2217	0.1831

Cell Index at: 87:32:03

	1	2	3	4	5	6	7	8	9	10	11	12
A								0.0046	-0.0033	0.0385	0.0112	0.0113
B								0.0395	0.0242	0.0344	0.062	0.0604
C								0.2289	0.2185	0.353	0.3659	0.272
D								0.2204	0.295	0.3213	0.3253	0.2889
E								0.0918	0.0882	0.1152	0.0963	0.066
F								0.2908	0.2651	0.325	0.3222	0.2865
G								0.0224	0.0429	0.0529	0.0485	0.0299
H								0.2488	0.166	0.2401	0.2224	0.183

Cell Index at: 87:47:03

	1	2	3	4	5	6	7	8	9	10	11	12
A								0.005	-0.0049	0.038	0.0126	0.0145
B								0.0427	0.0229	0.034	0.0616	0.0628
C								0.2274	0.2172	0.3606	0.3624	0.277
D								0.2139	0.2946	0.3283	0.3207	0.2787
E								0.099	0.0862	0.1098	0.1034	0.0674
F								0.2947	0.2736	0.3313	0.3239	0.2856
G								0.0222	0.0427	0.0539	0.0482	0.0307
H								0.2486	0.1638	0.2411	0.2261	0.1797

Cell Index at: 88:02:04

	1	2	3	4	5	6	7	8	9	10	11	12
A								0.0057	-0.0011	0.0393	0.0107	0.0146
B								0.0413	0.0234	0.0376	0.0686	0.0575
C								0.2308	0.223	0.3587	0.3667	0.2701
D								0.2138	0.3006	0.3239	0.3201	0.2841
E								0.104	0.0922	0.1136	0.0987	0.062
F								0.2976	0.27	0.3315	0.3283	0.2876
G								0.0246	0.047	0.0538	0.0504	0.0302
H								0.2535	0.1648	0.2422	0.2264	0.1767

Cell Index at: 88:17:05

	1	2	3	4	5	6	7	8	9	10	11	12
A								0.0036	-0.0017	0.0415	0.0109	0.0136
B								0.0401	0.025	0.0344	0.0721	0.0592
C								0.2315	0.2181	0.3542	0.3717	0.2819
D								0.2191	0.2937	0.3306	0.3204	0.2845
E								0.1026	0.0882	0.1228	0.1054	0.0632
F								0.2982	0.2722	0.3247	0.3265	0.2854
G								0.0221	0.0406	0.0558	0.0502	0.0259
H								0.2534	0.1614	0.246	0.2243	0.1806

Cell Index at: 88:32:05

	1	2	3	4	5	6	7	8	9	10	11	12
A								0.0063	-0.0037	0.0385	0.0103	0.014
B								0.0414	0.0288	0.0353	0.0718	0.06
C								0.2305	0.2185	0.3548	0.3674	0.2783
D								0.2189	0.2979	0.3243	0.3275	0.2872
E								0.0977	0.0961	0.1158	0.1062	0.0678
F								0.3016	0.2773	0.3255	0.3281	0.2894
G								0.0272	0.0456	0.0522	0.0537	0.0309
H								0.2537	0.1635	0.2497	0.2249	0.1791

Cell Index at: 88:47:05

	1	2	3	4	5	6	7	8	9	10	11	12
A								0.0064	-0.0023	0.0387	0.0098	0.0173
B								0.0451	0.0258	0.0357	0.0643	0.0609
C								0.2333	0.2197	0.3578	0.3695	0.278
D								0.2195	0.3115	0.3274	0.3296	0.2833
E								0.098	0.0899	0.1182	0.1113	0.0666
F								0.3038	0.2758	0.3301	0.3235	0.2852
G								0.025	0.0461	0.0542	0.0515	0.0302
H								0.257	0.1643	0.2464	0.226	0.1802

Cell Index at: 89:02:05

	1	2	3	4	5	6	7	8	9	10	11	12
A								0.0072	-0.0058	0.0409	0.0081	0.0155
B								0.0378	0.023	0.0387	0.0701	0.063
C								0.2331	0.2226	0.3576	0.3703	0.2815
D								0.2241	0.2988	0.3248	0.3283	0.2841
E								0.0966	0.093	0.1183	0.1107	0.0682
F								0.2992	0.2782	0.3319	0.3275	0.2875
G								0.0215	0.0435	0.059	0.0508	0.0318
H								0.2578	0.1622	0.2476	0.2266	0.1821

Cell Index at: 89:17:06

	1	2	3	4	5	6	7	8	9	10	11	12
A								0.0052	-0.0013	0.0417	0.0077	0.0152
B								0.04	0.0209	0.0394	0.0674	0.0613
C								0.23	0.2206	0.3621	0.3703	0.2842
D								0.22	0.3006	0.3235	0.329	0.2837
E								0.0991	0.0867	0.1206	0.1071	0.0683
F								0.3001	0.2798	0.3341	0.3245	0.2949
G								0.0216	0.0428	0.0519	0.0511	0.0347
H								0.2623	0.1652	0.2482	0.2265	0.1831

Cell Index at: 89:32:06

	1	2	3	4	5	6	7	8	9	10	11	12
A								0.0098	-0.0041	0.0387	0.011	0.0167
B								0.0363	0.0248	0.0417	0.0669	0.0624
C								0.2308	0.2187	0.3595	0.3702	0.283
D								0.2233	0.2993	0.325	0.3311	0.2881
E								0.097	0.0874	0.1181	0.1133	0.0656
F								0.3043	0.2755	0.327	0.3275	0.2892
G								0.0245	0.0405	0.057	0.0526	0.0278
H								0.2622	0.1655	0.2468	0.2265	0.1822

Cell Index at: 89:47:06

	1	2	3	4	5	6	7	8	9	10	11	12
A								0.0102	-0.0048	0.0336	0.0091	0.0177
B								0.0336	0.0282	0.0414	0.0737	0.0602
C								0.2335	0.2223	0.3616	0.376	0.2779
D								0.2176	0.3074	0.3347	0.3309	0.2912
E								0.1014	0.0897	0.1191	0.109	0.0643
F								0.3078	0.2753	0.3329	0.3302	0.2894
G								0.022	0.0456	0.0569	0.0519	0.0295
H								0.2625	0.1677	0.2485	0.2262	0.1883

Cell Index at: 90:02:07

	1	2	3	4	5	6	7	8	9	10	11	12
A								0.0077	-0.0043	0.0366	0.0076	0.0174
B								0.0354	0.0261	0.0403	0.0688	0.0595
C								0.2338	0.2215	0.3618	0.3712	0.2845
D								0.222	0.3026	0.3314	0.3313	0.2887
E								0.096	0.087	0.1172	0.1126	0.0707
F								0.3083	0.283	0.3351	0.3297	0.2951
G								0.0229	0.0456	0.0573	0.0516	0.033
H								0.2651	0.1671	0.2508	0.2269	0.1851

Cell Index at: 90:17:05

	1	2	3	4	5	6	7	8	9	10	11	12
A								0.0098	-0.0054	0.038	0.0116	0.0169
B								0.0374	0.0296	0.0404	0.073	0.0619
C								0.2411	0.2256	0.3577	0.3654	0.2882
D								0.218	0.3069	0.3359	0.3272	0.2912
E								0.0976	0.0852	0.1232	0.1175	0.0685
F								0.3025	0.2788	0.337	0.3299	0.297
G								0.0242	0.0427	0.0583	0.053	0.0334
H								0.2635	0.1691	0.2497	0.2292	0.1876

Cell Index at: 90:32:06

	1	2	3	4	5	6	7	8	9	10	11	12
A								0.0113	-0.0032	0.0393	0.0051	0.0168
B								0.0358	0.0285	0.0461	0.0715	0.0631
C								0.2423	0.2251	0.3632	0.3706	0.2887
D								0.2193	0.307	0.3295	0.3393	0.2898
E								0.1005	0.0956	0.1205	0.1148	0.0728
F								0.3086	0.2841	0.3309	0.333	0.2904
G								0.0237	0.0441	0.0558	0.0504	0.0316
H								0.2705	0.1683	0.2479	0.2292	0.1893

Cell Index at: 90:47:07

	1	2	3	4	5	6	7	8	9	10	11	12
A								0.0147	-0.0012	0.0408	0.0069	0.0185
B								0.0374	0.0314	0.0444	0.0678	0.0574
C								0.2432	0.2285	0.3715	0.3737	0.2869
D								0.2142	0.2973	0.328	0.3419	0.2881
E								0.1014	0.089	0.1245	0.1176	0.068
F								0.3049	0.2842	0.3337	0.3326	0.2945
G								0.0243	0.0425	0.0578	0.0473	0.0329
H								0.271	0.1687	0.2505	0.2307	0.1891

Cell Index at: 91:02:07

	1	2	3	4	5	6	7	8	9	10	11	12
A								0.0131	-0.0006	0.0383	0.0093	0.0207
B								0.037	0.0298	0.0444	0.0668	0.0569
C								0.2373	0.2262	0.3682	0.3738	0.2884
D								0.2159	0.303	0.3303	0.3354	0.2869
E								0.0935	0.0897	0.1233	0.1141	0.0677
F								0.3078	0.2882	0.3318	0.3418	0.2963
G								0.0234	0.0488	0.0569	0.0448	0.0309
H								0.2722	0.1673	0.2506	0.2339	0.1904

Cell Index at: 91:17:07

	1	2	3	4	5	6	7	8	9	10	11	12
A								0.0151	-0.0006	0.0408	0.0093	0.0208
B								0.0407	0.0288	0.0421	0.0685	0.0585
C								0.234	0.2348	0.3679	0.3724	0.2916
D								0.2248	0.3133	0.334	0.3333	0.2885
E								0.1033	0.104	0.12	0.1103	0.075
F								0.3107	0.2829	0.3413	0.3407	0.2923
G								0.0269	0.0449	0.0608	0.0502	0.0276
H								0.2721	0.1676	0.2529	0.2321	0.1877

Cell Index at: 91:32:08

	1	2	3	4	5	6	7	8	9	10	11	12
A								0.0129	0.0002	0.0394	0.0087	0.0219
B								0.0399	0.0334	0.0444	0.069	0.0581
C								0.2306	0.232	0.3621	0.3733	0.296
D								0.2238	0.3102	0.3331	0.3293	0.2924
E								0.0919	0.0926	0.125	0.113	0.0661
F								0.3079	0.2855	0.343	0.3378	0.2924
G								0.0277	0.0467	0.0563	0.0498	0.03
H								0.2712	0.1683	0.252	0.2352	0.1882

Cell Index at: 91:47:09

	1	2	3	4	5	6	7	8	9	10	11	12
A								0.0107	-0.0021	0.043	0.0109	0.0201
B								0.0379	0.0331	0.0449	0.0681	0.0521
C								0.2333	0.2353	0.3679	0.3775	0.2961
D								0.2271	0.3072	0.3417	0.3372	0.2952
E								0.1022	0.0923	0.1252	0.1133	0.0697
F								0.3134	0.2869	0.3372	0.339	0.2982
G								0.0297	0.0478	0.0557	0.0508	0.0253
H								0.2738	0.1722	0.2527	0.2356	0.191

Cell Index at: 92:02:10

	1	2	3	4	5	6	7	8	9	10	11	12
A								0.0131	-0.0004	0.0415	0.0081	0.0209
B								0.0433	0.0347	0.0447	0.069	0.0586
C								0.2344	0.2373	0.3594	0.3773	0.2997
D								0.2244	0.3006	0.3359	0.3355	0.2883
E								0.1063	0.0919	0.1279	0.1222	0.0732
F								0.3101	0.2912	0.3384	0.3413	0.2947
G								0.0252	0.0446	0.0581	0.0503	0.0282
H								0.2713	0.1686	0.2552	0.2363	0.1872

Cell Index at: 92:17:11

	1	2	3	4	5	6	7	8	9	10	11	12
A								0.0162	0.0015	0.0425	0.0072	0.0221
B								0.0394	0.0359	0.048	0.0742	0.0557
C								0.2345	0.235	0.3625	0.3774	0.2985
D								0.2207	0.3133	0.3283	0.3333	0.2987
E								0.1047	0.0906	0.1258	0.1176	0.0718
F								0.316	0.288	0.3393	0.3443	0.2943
G								0.0298	0.048	0.0592	0.0492	0.0337
H								0.2732	0.1737	0.2531	0.2346	0.1886

Cell Index at: 92:32:11

	1	2	3	4	5	6	7	8	9	10	11	12
A								0.0145	0.0012	0.0426	0.0068	0.0199
B								0.0467	0.0361	0.0456	0.0704	0.0575
C								0.2323	0.2363	0.3574	0.3765	0.3006
D								0.2257	0.3135	0.3294	0.338	0.2948
E								0.104	0.0897	0.1259	0.12	0.0749
F								0.3197	0.2877	0.3372	0.3461	0.3011
G								0.0294	0.0498	0.0557	0.0494	0.0324
H								0.2758	0.1719	0.2544	0.2372	0.1917

Cell Index at: 92:47:12

	1	2	3	4	5	6	7	8	9	10	11	12
A								0.0169	0.0034	0.0416	0.0056	0.0221
B								0.0477	0.0319	0.0439	0.0714	0.0549
C								0.2375	0.2386	0.3638	0.3798	0.2968
D								0.2325	0.3114	0.3251	0.3388	0.2905
E								0.1	0.0909	0.1219	0.125	0.0702
F								0.3241	0.2939	0.3463	0.3494	0.3059
G								0.0306	0.0483	0.058	0.0481	0.0325
H								0.2781	0.1736	0.2551	0.2356	0.1937

Cell Index at: 93:02:12

	1	2	3	4	5	6	7	8	9	10	11	12
A								0.0152	0.0024	0.0432	0.0052	0.0221
B								0.0422	0.0344	0.0467	0.0712	0.0544
C								0.2377	0.2383	0.356	0.3792	0.3064
D								0.236	0.315	0.3345	0.3365	0.3006
E								0.1019	0.0925	0.1262	0.1217	0.0711
F								0.3186	0.2984	0.3437	0.3453	0.305
G								0.0277	0.049	0.0605	0.0544	0.0326
H								0.2762	0.1724	0.2546	0.2359	0.1925

Cell Index at: 93:17:13

	1	2	3	4	5	6	7	8	9	10	11	12
A								0.0143	0.0052	0.044	0.0059	0.0192
B								0.0436	0.0334	0.045	0.0696	0.0556
C								0.2505	0.2342	0.3616	0.3792	0.3059
D								0.2311	0.3102	0.3252	0.3325	0.3017
E								0.103	0.0993	0.1274	0.1227	0.0665
F								0.326	0.2952	0.3464	0.3472	0.3024
G								0.0315	0.0503	0.0606	0.0495	0.0324
H								0.2779	0.1753	0.2579	0.235	0.1936

Cell Index at: 93:32:13

	1	2	3	4	5	6	7	8	9	10	11	12
A								0.0138	0.0079	0.0407	0.0065	0.0213
B								0.0426	0.033	0.0486	0.0753	0.0516
C								0.2451	0.2355	0.3612	0.3809	0.311
D								0.2312	0.317	0.3351	0.3411	0.2957
E								0.0998	0.0951	0.1305	0.1208	0.0681
F								0.329	0.2997	0.3458	0.3392	0.3052
G								0.0319	0.0534	0.0578	0.0538	0.0332

H								0.2788	0.1713	0.2599	0.2362	0.1929

Cell Index at: 93:47:14

	1	2	3	4	5	6	7	8	9	10	11	12
A								0.0134	0.0051	0.0437	0.0073	0.0225
B								0.0479	0.0329	0.0476	0.0736	0.055
C								0.2449	0.2336	0.3563	0.378	0.3047
D								0.234	0.3127	0.3326	0.3353	0.3054
E								0.0964	0.0977	0.1286	0.1196	0.069
F								0.3247	0.297	0.3492	0.35	0.3104
G								0.0317	0.0547	0.0582	0.0533	0.0339
H								0.2791	0.1757	0.2601	0.2377	0.1944

Cell Index at: 94:02:15

	1	2	3	4	5	6	7	8	9	10	11	12
A								0.0167	0.0065	0.0446	0.007	0.0237
B								0.0419	0.0312	0.0467	0.0783	0.0554
C								0.2485	0.2415	0.3551	0.3777	0.307
D								0.237	0.3101	0.3357	0.3337	0.3035
E								0.0951	0.0929	0.1243	0.1246	0.0712
F								0.3211	0.2983	0.3466	0.3481	0.3116
G								0.0298	0.0546	0.0548	0.052	0.0338
H								0.2807	0.1785	0.259	0.2387	0.1961

Cell Index at: 94:17:15

	1	2	3	4	5	6	7	8	9	10	11	12
A								0.0154	0.005	0.0426	0.0066	0.0247
B								0.046	0.033	0.0455	0.0775	0.0562
C								0.2481	0.2388	0.3563	0.3874	0.3047
D								0.2312	0.306	0.3421	0.34	0.302
E								0.101	0.097	0.1276	0.121	0.0779
F								0.3282	0.2982	0.3456	0.3523	0.3057
G								0.0293	0.0527	0.0573	0.0578	0.0344
H								0.2812	0.1771	0.2598	0.239	0.1977

Cell Index at: 94:32:16

	1	2	3	4	5	6	7	8	9	10	11	12
A								0.017	0.0037	0.0395	0.0078	0.026
B								0.0445	0.0313	0.0458	0.0751	0.0576
C								0.2445	0.2399	0.3591	0.3837	0.3109
D								0.2395	0.3067	0.3429	0.333	0.302
E								0.0938	0.0933	0.1254	0.1167	0.0768
F								0.3273	0.2974	0.3466	0.3519	0.3047
G								0.0315	0.054	0.0582	0.056	0.0352
H								0.2823	0.1818	0.2605	0.2382	0.1997

Cell Index at: 94:47:16

	1	2	3	4	5	6	7	8	9	10	11	12
A								0.0125	0.0029	0.0427	0.0053	0.0238
B								0.0405	0.029	0.0474	0.0745	0.0573
C								0.2466	0.2387	0.3601	0.3865	0.3077
D								0.2401	0.3144	0.3445	0.3338	0.2989
E								0.0953	0.0993	0.129	0.118	0.0761
F								0.3279	0.2968	0.3542	0.3567	0.3062
G								0.0379	0.0551	0.0596	0.0532	0.0397
H								0.2829	0.1822	0.2636	0.2408	0.2017

Cell Index at: 95:02:16

	1	2	3	4	5	6	7	8	9	10	11	12
A								0.0161	0.0039	0.0412	0.0078	0.0247
B								0.0462	0.0316	0.0465	0.0757	0.0546
C								0.2421	0.2421	0.3643	0.3839	0.3084
D								0.2437	0.3146	0.3436	0.3333	0.3065
E								0.0962	0.1028	0.1292	0.1236	0.0731
F								0.3267	0.3002	0.3534	0.352	0.3023
G								0.0338	0.053	0.0621	0.0588	0.0351
H								0.2832	0.1787	0.2622	0.2418	0.2036

Cell Index at: 95:17:17

	1	2	3	4	5	6	7	8	9	10	11	12
A								0.0185	0.0026	0.0425	0.006	0.0242
B								0.0425	0.0298	0.0454	0.076	0.0551
C								0.2461	0.2432	0.36	0.3934	0.3106
D								0.2401	0.3099	0.3394	0.337	0.3082
E								0.0959	0.0911	0.1271	0.1174	0.0781
F								0.3333	0.2974	0.3565	0.3535	0.3041
G								0.0304	0.0541	0.0603	0.0548	0.034
H								0.2824	0.1833	0.2654	0.2388	0.2007

Cell Index at: 95:32:18

	1	2	3	4	5	6	7	8	9	10	11	12
A								0.019	0.001	0.04	0.0072	0.0279
B								0.0513	0.034	0.0492	0.0732	0.0525
C								0.2471	0.2432	0.3636	0.3827	0.3088
D								0.247	0.3077	0.3387	0.3406	0.3061
E								0.106	0.0943	0.1309	0.118	0.0687
F								0.3352	0.2959	0.3542	0.3534	0.3018
G								0.0314	0.0549	0.0624	0.0628	0.037
H								0.2875	0.1814	0.2687	0.2416	0.2032

Cell Index at: 95:47:19

	1	2	3	4	5	6	7	8	9	10	11	12
A								0.0159	0.0002	0.0417	0.0089	0.0223
B								0.0442	0.0342	0.0487	0.0747	0.0531
C								0.2427	0.2493	0.368	0.3879	0.3103
D								0.2477	0.3179	0.3465	0.3338	0.3079
E								0.0941	0.0936	0.1372	0.1224	0.0691
F								0.3388	0.2991	0.3492	0.3558	0.2953
G								0.0323	0.0559	0.0573	0.0598	0.0378
H								0.2845	0.1825	0.2663	0.2398	0.2033

Cell Index at: 96:02:20

	1	2	3	4	5	6	7	8	9	10	11	12
A								0.0195	0.0008	0.041	0.0082	0.0244
B								0.0456	0.0304	0.0455	0.073	0.0533
C								0.2475	0.2423	0.368	0.3903	0.3124
D								0.2479	0.3211	0.3482	0.3395	0.3086
E								0.1039	0.0957	0.1283	0.1191	0.0687
F								0.3329	0.3025	0.3583	0.3531	0.3019
G								0.0378	0.0516	0.06	0.0547	0.0388
H								0.2853	0.1834	0.2665	0.2383	0.2045

A vs B	0.013838	B vs C	0.000681	C vs D	0.978968	D vs E	3.2E-05	E vs F	6.92E-07	F vs G	3.26E-06	G vs H	0.000384
A vs C	0.000427	B vs D	2.63E-05	C vs E	0.001331	D vs F	0.460955	E vs G	0.003735	F vs H	0.004287		
A vs D	1.5E-05	B vs E	0.003525	C vs F	0.610003	D vs G	6.04E-05	E vs H	0.000745				
A vs E	0.000257	B vs F	4.9E-07	C vs G	0.000824	D vs H	0.017314						
A vs F	2.54E-07	B vs G	0.908562	C vs H	0.070701								
A vs G	0.008901	B vs H	0.000232										
A vs H	0.000111												

Cell Index at: 96:17:21

	1	2	3	4	5	6	7	8	9	10	11	12
A								0.0181	0.0003	0.0414	0.0067	0.0223
B								0.0442	0.0325	0.0447	0.0772	0.0525
C								0.2536	0.2407	0.3676	0.3976	0.3152
D								0.2458	0.3169	0.3404	0.3393	0.31
E								0.1074	0.0974	0.1306	0.1217	0.0715
F								0.338	0.2962	0.3572	0.3541	0.3134
G								0.0348	0.0565	0.0609	0.0595	0.0377
H								0.2863	0.1832	0.2688	0.2391	0.2041

Count	*Sum*	*Average*	*Variance*
5	0.0939	0.01878	0.00024
5	0.2478	0.04956	0.000241
5	1.5605	0.3121	0.004571
5	1.5653	0.31306	0.001566
5	0.5157	0.10314	0.000532
5	1.6487	0.32974	0.000722
5	0.2429	0.04858	9.72E-05
5	1.178	0.2356	0.001781

Cell Index at: 96:32:21

	1	2	3	4	5	6	7	8	9	10	11	12
A								0.023	0.003	0.0383	0.0075	0.0238
B								0.0387	0.0336	0.0506	0.0759	0.0498
C								0.2511	0.2426	0.3691	0.39	0.3147
D								0.2471	0.3226	0.3415	0.33	0.3079
E								0.0981	0.0994	0.1269	0.1316	0.0681

SS	*df*	*MS*	*F*	*P-value*	*F crit*
0.633728	7	0.090533	74.2743	5.65E-18	2.312741
0.039005	32	0.001219			

F								0.3369	0.3047	0.3541	0.3638	0.3065
G								0.0341	0.0564	0.0591	0.0604	0.0365
H								0.2822	0.1815	0.2691	0.2409	0.2042

0.672733 39

Cell Index at: 96:47:21

	1	2	3	4	5	6	7	8	9	10	11	12
A								0.023	0.0024	0.0405	0.0087	0.0247
B								0.0457	0.0317	0.0497	0.0765	0.0549
C								0.2518	0.2421	0.3715	0.3947	0.3126
D								0.2417	0.3263	0.3413	0.3344	0.3105
E								0.1067	0.0912	0.1297	0.131	0.0657
F								0.342	0.3108	0.3509	0.3597	0.3062
G								0.0361	0.0527	0.0655	0.0591	0.034
H								0.2831	0.1828	0.2726	0.2433	0.2056

Cell Index at: 97:02:21

	1	2	3	4	5	6	7	8	9	10	11	12
A								0.0243	0.0015	0.0408	0.0067	0.0252
B								0.0483	0.0298	0.0558	0.0781	0.0497
C								0.2538	0.2404	0.3712	0.3948	0.3137
D								0.2435	0.3219	0.3499	0.3346	0.3032
E								0.1063	0.0911	0.1364	0.1211	0.0708
F								0.3439	0.3058	0.3541	0.3612	0.304
G								0.0381	0.0517	0.0641	0.0574	0.0355
H								0.2838	0.1864	0.272	0.2437	0.2064

Cell Index at: 97:17:22

	1	2	3	4	5	6	7	8	9	10	11	12
A								0.0223	-0.0006	0.0429	0.0087	0.0256
B								0.0472	0.0351	0.0536	0.0768	0.0553
C								0.2583	0.2436	0.3711	0.3977	0.3173
D								0.2519	0.3191	0.3443	0.3359	0.3098
E								0.1051	0.097	0.1401	0.1171	0.0706
F								0.3429	0.3122	0.3479	0.3554	0.3076
G								0.0401	0.0555	0.0608	0.0574	0.0364
H								0.2871	0.1808	0.2728	0.2442	0.2066

Cell Index at: 97:32:22

	1	2	3	4	5	6	7	8	9	10	11	12
A								0.0246	0.0021	0.0459	0.0079	0.0252
B								0.045	0.0321	0.0553	0.0803	0.0528
C								0.2538	0.2386	0.3679	0.3876	0.3065
D								0.2531	0.3201	0.3479	0.3334	0.3063
E								0.1005	0.0966	0.1292	0.1169	0.0714
F								0.3441	0.3116	0.3558	0.3595	0.3119
G								0.0402	0.0539	0.0618	0.0584	0.0368
H								0.2873	0.1821	0.2709	0.2417	0.2035

Cell Index at: 97:47:23

	1	2	3	4	5	6	7	8	9	10	11	12
A								0.0232	0.0003	0.0451	0.0094	0.0268
B								0.0452	0.0308	0.0585	0.0754	0.0545
C								0.2546	0.2402	0.3703	0.3893	0.3119
D								0.25	0.3209	0.3481	0.3354	0.315
E								0.1139	0.1007	0.1339	0.1204	0.0652
F								0.344	0.3098	0.3556	0.3511	0.3067
G								0.0373	0.0543	0.0658	0.0548	0.0388
H								0.2847	0.1861	0.2732	0.2428	0.2104

Cell Index at: 98:02:24

	1	2	3	4	5	6	7	8	9	10	11	12
A								0.0221	0.0004	0.047	0.0073	0.0247
B								0.0451	0.0323	0.0598	0.0727	0.0546
C								0.2556	0.246	0.3728	0.3905	0.3097
D								0.257	0.319	0.3517	0.3338	0.3169
E								0.1047	0.0995	0.1387	0.1206	0.0726
F								0.349	0.3118	0.347	0.3572	0.3167
G								0.0413	0.0542	0.0635	0.0614	0.041
H								0.2834	0.185	0.2764	0.2427	0.209

Cell Index at: 98:17:25

	1	2	3	4	5	6	7	8	9	10	11	12
A								0.0235	0.0004	0.0491	0.0081	0.0239
B								0.0434	0.0328	0.0552	0.0761	0.0522
C								0.2534	0.2463	0.3697	0.3928	0.3139
D								0.2507	0.323	0.3443	0.329	0.3057
E								0.0962	0.1019	0.1303	0.1143	0.0707
F								0.3501	0.3111	0.3508	0.361	0.3116
G								0.0447	0.0529	0.0644	0.0561	0.0374
H								0.2858	0.1864	0.2745	0.2445	0.2103

Cell Index at: 98:32:26

	1	2	3	4	5	6	7	8	9	10	11	12
A								0.0229	-0.0008	0.0523	0.0085	0.0247
B								0.0411	0.0317	0.0573	0.0737	0.054
C								0.2577	0.2394	0.3725	0.3928	0.3161
D								0.2527	0.3201	0.3412	0.3401	0.3092
E								0.1147	0.0998	0.1325	0.1249	0.0716
F								0.3456	0.3206	0.355	0.3585	0.3082
G								0.0383	0.0524	0.0626	0.0584	0.0375
H								0.2834	0.1873	0.2776	0.2465	0.2138

Cell Index at: 98:47:26

	1	2	3	4	5	6	7	8	9	10	11	12
A								0.021	-0.0004	0.0471	0.0079	0.0251
B								0.0485	0.0287	0.0545	0.0724	0.0523
C								0.2565	0.2449	0.3703	0.3945	0.3155
D								0.252	0.3231	0.3472	0.3382	0.3083
E								0.1025	0.0959	0.1375	0.1229	0.0712
F								0.3528	0.3202	0.3617	0.3603	0.3035
G								0.042	0.0525	0.0614	0.0592	0.037
H								0.2848	0.1862	0.2755	0.2466	0.2116

Cell Index at: 99:02:27

	1	2	3	4	5	6	7	8	9	10	11	12
A								0.0262	0.0034	0.0473	0.0118	0.0269
B								0.0491	0.0293	0.058	0.0799	0.0544
C								0.2595	0.2416	0.3685	0.3998	0.315
D								0.2497	0.3265	0.3456	0.3369	0.312
E								0.1123	0.1004	0.1423	0.1196	0.0729
F								0.347	0.3173	0.3567	0.3629	0.3083
G								0.0408	0.0546	0.0645	0.0586	0.0382
H								0.2879	0.186	0.2747	0.2488	0.2157

Cell Index at: 99:17:28

	1	2	3	4	5	6	7	8	9	10	11	12
A								0.0259	0.0035	0.0495	0.0115	0.0246
B								0.0493	0.0323	0.0585	0.0771	0.0521
C								0.2606	0.2451	0.3783	0.3961	0.3116
D								0.2565	0.3215	0.3442	0.3451	0.3065
E								0.1054	0.1067	0.1381	0.1192	0.0779
F								0.3509	0.3122	0.3534	0.3674	0.3042
G								0.0453	0.0575	0.0618	0.0579	0.0367
H								0.2891	0.1843	0.2757	0.2486	0.2141

Cell Index at: 99:32:28

	1	2	3	4	5	6	7	8	9	10	11	12
A								0.0241	0.0046	0.0453	0.0065	0.0249
B								0.0495	0.0307	0.0545	0.0773	0.0543
C								0.2612	0.2438	0.3821	0.3995	0.3169
D								0.2549	0.32	0.3469	0.3353	0.3056

	1	2	3	4	5	6	7	8	9	10	11	12
E								0.1052	0.0996	0.1391	0.117	0.0722
F								0.3479	0.3174	0.3622	0.3655	0.3105
G								0.0399	0.0573	0.0635	0.0566	0.0367
H								0.2889	0.1853	0.2797	0.2501	0.2148

Cell Index at: 99:47:29

	1	2	3	4	5	6	7	8	9	10	11	12
A								0.0257	0.0046	0.0492	0.0094	0.0274
B								0.0484	0.0337	0.0586	0.0789	0.0481
C								0.2571	0.2476	0.3758	0.3998	0.319
D								0.2562	0.3241	0.347	0.3457	0.3073
E								0.1021	0.0996	0.1379	0.1153	0.0725
F								0.3547	0.3149	0.3655	0.3647	0.3096
G								0.0444	0.0573	0.0703	0.056	0.0363
H								0.284	0.1863	0.2793	0.2486	0.2169

Cell Index at: 100:02:29

	1	2	3	4	5	6	7	8	9	10	11	12
A								0.0257	0.0074	0.047	0.0092	0.0247
B								0.0512	0.0277	0.0593	0.0806	0.0528
C								0.259	0.2507	0.3808	0.3982	0.317
D								0.2621	0.3287	0.3522	0.3465	0.3099
E								0.1099	0.1037	0.1367	0.1153	0.0709
F								0.3453	0.3221	0.3645	0.3704	0.3071
G								0.0431	0.0568	0.0685	0.0565	0.0363
H								0.283	0.1851	0.2778	0.2481	0.218

Cell Index at: 100:17:29

	1	2	3	4	5	6	7	8	9	10	11	12
A								0.0275	0.0052	0.0447	0.0101	0.0247
B								0.0493	0.0303	0.0563	0.0813	0.0507
C								0.2557	0.2445	0.3777	0.4021	0.3184
D								0.2619	0.3273	0.3541	0.3483	0.3087
E								0.1064	0.0984	0.1317	0.1211	0.068
F								0.3512	0.3211	0.3646	0.3686	0.3128
G								0.0464	0.0513	0.0636	0.0576	0.0343
H								0.2875	0.1882	0.279	0.244	0.2161

Cell Index at: 100:32:27

	1	2	3	4	5	6	7	8	9	10	11	12
A								0.0256	0.0092	0.048	0.0078	0.0255
B								0.0501	0.031	0.0571	0.0831	0.0451
C								0.2639	0.2471	0.3784	0.3975	0.3243
D								0.2565	0.3238	0.3514	0.3464	0.3102
E								0.1062	0.1013	0.133	0.1187	0.073
F								0.3533	0.3214	0.3594	0.3717	0.3103
G								0.0438	0.0508	0.0639	0.0487	0.0382
H								0.2874	0.1886	0.2814	0.2457	0.2154

Cell Index at: 100:47:28

	1	2	3	4	5	6	7	8	9	10	11	12
A								0.0255	0.0107	0.0466	0.0094	0.025
B								0.0478	0.0357	0.0588	0.0793	0.0515
C								0.2565	0.2587	0.3846	0.3993	0.3206
D								0.2625	0.3275	0.3545	0.3515	0.3105
E								0.1124	0.098	0.1394	0.1186	0.0718
F								0.355	0.3263	0.3637	0.3657	0.3148
G								0.0422	0.05	0.0617	0.0551	0.0391
H								0.287	0.1908	0.2797	0.2493	0.2212

Cell Index at: 101:02:28

	1	2	3	4	5	6	7	8	9	10	11	12
A								0.0271	0.013	0.0488	0.0099	0.0254
B								0.0474	0.0348	0.0548	0.0802	0.0466
C								0.2605	0.2536	0.3825	0.3961	0.3195
D								0.2664	0.3393	0.3588	0.3552	0.3102
E								0.1104	0.1012	0.1352	0.1197	0.0673
F								0.3615	0.3182	0.3628	0.364	0.3187
G								0.0451	0.0472	0.064	0.0505	0.0355
H								0.2863	0.1906	0.2824	0.2519	0.2216

Cell Index at: 101:17:29

	1	2	3	4	5	6	7	8	9	10	11	12
A								0.0294	0.0101	0.0494	0.0125	0.0258
B								0.0497	0.0335	0.0554	0.0809	0.048
C								0.2583	0.2535	0.3728	0.3954	0.3188
D								0.2583	0.3242	0.3521	0.3504	0.3095
E								0.1145	0.089	0.1349	0.117	0.0742
F								0.3587	0.3256	0.3675	0.3722	0.3126
G								0.0436	0.0478	0.062	0.0537	0.0357
H								0.2858	0.193	0.28	0.2493	0.2203

Cell Index at: 101:32:29

	1	2	3	4	5	6	7	8	9	10	11	12
A								0.0299	0.0108	0.0532	0.0123	0.0277
B								0.049	0.0351	0.0587	0.0802	0.0481
C								0.2573	0.2537	0.3795	0.3988	0.3187
D								0.2635	0.329	0.35	0.3487	0.3113
E								0.1184	0.0985	0.1351	0.1255	0.073
F								0.3593	0.3181	0.3587	0.3729	0.3131
G								0.0439	0.0488	0.0595	0.0486	0.0396
H								0.2872	0.196	0.2831	0.2463	0.2244

Cell Index at: 101:47:30

	1	2	3	4	5	6	7	8	9	10	11	12
A								0.0315	0.0094	0.0489	0.0115	0.0295
B								0.0499	0.0341	0.0554	0.0772	0.0478
C								0.2626	0.257	0.3786	0.4078	0.3203
D								0.266	0.3273	0.3486	0.3535	0.3159
E								0.1152	0.0976	0.1414	0.1239	0.0682
F								0.3612	0.3178	0.3607	0.3693	0.3172
G								0.0419	0.0521	0.0596	0.0461	0.0385
H								0.2902	0.1925	0.2817	0.25	0.2236

Cell Index at: 102:02:31

	1	2	3	4	5	6	7	8	9	10	11	12
A								0.0312	0.0088	0.0532	0.0124	0.0299
B								0.0466	0.0406	0.057	0.0776	0.0505
C								0.2635	0.2584	0.3821	0.4016	0.323
D								0.2692	0.3312	0.3562	0.3602	0.3149
E								0.1123	0.0958	0.1423	0.1191	0.076
F								0.3613	0.3275	0.3639	0.3749	0.3171
G								0.042	0.0504	0.0591	0.0531	0.0386
H								0.2888	0.1949	0.2849	0.2499	0.2247

Cell Index at: 102:17:31

	1	2	3	4	5	6	7	8	9	10	11	12
A								0.0318	0.0136	0.0539	0.0146	0.0297
B								0.0438	0.0348	0.0579	0.071	0.048
C								0.2626	0.2546	0.3745	0.4077	0.327
D								0.2713	0.3266	0.3572	0.3533	0.3077
E								0.113	0.1009	0.1427	0.1205	0.074
F								0.3658	0.3228	0.3647	0.3741	0.3185
G								0.0491	0.0526	0.0592	0.0499	0.0403
H								0.2893	0.1892	0.2851	0.2515	0.2279

Cell Index at: 102:32:31

	1	2	3	4	5	6	7	8	9	10	11	12
A								0.0349	0.0138	0.0536	0.0154	0.0285
B								0.047	0.0388	0.057	0.0741	0.0492
C								0.2585	0.2631	0.3779	0.4053	0.3182

D								0.2672	0.3275	0.3566	0.363	0.3085
E								0.1073	0.0973	0.1346	0.1202	0.0791
F								0.3699	0.3193	0.3613	0.3772	0.3141
G								0.0435	0.0532	0.0609	0.0508	0.0394
H								0.2888	0.1936	0.285	0.2523	0.2298

Cell Index at: 102:47:31

	1	2	3	4	5	6	7	8	9	10	11	12
A								0.0333	0.0132	0.0558	0.0153	0.0278
B								0.0501	0.0383	0.0542	0.0753	0.0476
C								0.2634	0.2617	0.377	0.4058	0.3214
D								0.2636	0.331	0.3522	0.3547	0.3144
E								0.1137	0.1009	0.1372	0.1211	0.0729
F								0.3674	0.3205	0.3635	0.3797	0.3093
G								0.0412	0.0539	0.0621	0.0516	0.0398
H								0.2877	0.1947	0.2843	0.2534	0.229

Cell Index at: 103:02:31

	1	2	3	4	5	6	7	8	9	10	11	12
A								0.0369	0.0143	0.0559	0.0171	0.0275
B								0.0461	0.0384	0.0568	0.0787	0.048
C								0.2588	0.2546	0.3789	0.4179	0.3197
D								0.2683	0.3328	0.3548	0.3552	0.3213
E								0.1193	0.0983	0.1351	0.1194	0.0712
F								0.367	0.3153	0.3635	0.3757	0.3187
G								0.0437	0.0553	0.0638	0.0519	0.042
H								0.2896	0.1953	0.2846	0.2537	0.2314

Cell Index at: 103:17:31

	1	2	3	4	5	6	7	8	9	10	11	12
A								0.035	0.0159	0.059	0.0158	0.0257
B								0.049	0.0382	0.0571	0.0745	0.0492
C								0.2621	0.2587	0.3771	0.4127	0.3182
D								0.2712	0.3297	0.3562	0.3653	0.3279
E								0.1111	0.098	0.1354	0.115	0.0738
F								0.3607	0.3239	0.3647	0.3764	0.3177
G								0.044	0.0525	0.0644	0.0534	0.0416
H								0.2904	0.1922	0.2822	0.2558	0.234

Cell Index at: 103:32:32

	1	2	3	4	5	6	7	8	9	10	11	12
A								0.0374	0.0153	0.0572	0.0161	0.0268
B								0.0472	0.0407	0.0512	0.0766	0.05
C								0.2607	0.2615	0.3841	0.4078	0.3208
D								0.2631	0.3339	0.3567	0.3533	0.3248
E								0.1167	0.0944	0.1358	0.1236	0.0732
F								0.3688	0.3282	0.3725	0.3776	0.3139
G								0.0391	0.0579	0.0619	0.0536	0.0449
H								0.2926	0.1941	0.2851	0.2539	0.232

Cell Index at: 103:47:32

	1	2	3	4	5	6	7	8	9	10	11	12
A								0.0414	0.0189	0.0596	0.0148	0.0257
B								0.0458	0.0413	0.0573	0.0787	0.0484
C								0.2608	0.2631	0.3844	0.4103	0.3226
D								0.2685	0.3347	0.3572	0.3538	0.3221
E								0.1157	0.1011	0.1395	0.1195	0.0751
F								0.3657	0.3209	0.3714	0.3828	0.3197
G								0.0457	0.0595	0.0595	0.0513	0.0453
H								0.2934	0.1954	0.2865	0.2561	0.2351

Cell Index at: 104:02:33

	1	2	3	4	5	6	7	8	9	10	11	12
A								0.0417	0.0189	0.0561	0.013	0.0279
B								0.0408	0.0423	0.0512	0.0763	0.0513
C								0.2615	0.2621	0.3877	0.4031	0.3298
D								0.2651	0.3361	0.3673	0.3629	0.319
E								0.1121	0.0968	0.1345	0.1206	0.0719
F								0.3672	0.3266	0.3753	0.376	0.3137
G								0.0388	0.0588	0.0641	0.0551	0.0449
H								0.2944	0.1977	0.2831	0.2588	0.239

Cell Index at: 104:17:33

	1	2	3	4	5	6	7	8	9	10	11	12
A								0.0405	0.0192	0.054	0.0159	0.032
B								0.0452	0.0424	0.0594	0.0784	0.0493
C								0.2619	0.2614	0.3827	0.4068	0.3258
D								0.2667	0.3369	0.3642	0.3588	0.3243
E								0.112	0.095	0.1446	0.1223	0.0715
F								0.3665	0.3297	0.3778	0.3809	0.3157
G								0.0391	0.0561	0.0615	0.0495	0.0464
H								0.2963	0.1984	0.2849	0.2559	0.2402

Cell Index at: 104:32:33

	1	2	3	4	5	6	7	8	9	10	11	12
A								0.0405	0.0203	0.0565	0.016	0.0292
B								0.0418	0.0429	0.057	0.0785	0.0495
C								0.2619	0.2611	0.3851	0.4073	0.3223
D								0.2626	0.3299	0.3533	0.3547	0.3179
E								0.1123	0.0943	0.1359	0.1272	0.0733
F								0.3696	0.3311	0.3724	0.3822	0.3143
G								0.0429	0.0558	0.0613	0.0517	0.0497
H								0.3005	0.198	0.2826	0.2587	0.2387

Cell Index at: 104:47:33

	1	2	3	4	5	6	7	8	9	10	11	12
A								0.0395	0.02	0.0576	0.0171	0.0302
B								0.0396	0.0433	0.0593	0.0795	0.0487
C								0.261	0.2671	0.3898	0.4046	0.321
D								0.2555	0.3286	0.357	0.3665	0.3115
E								0.1203	0.0977	0.1445	0.1295	0.0701
F								0.3763	0.3346	0.3714	0.3916	0.3192
G								0.0457	0.0571	0.0605	0.0493	0.0482
H								0.3003	0.1968	0.2811	0.2624	0.2404

Cell Index at: 105:02:33

	1	2	3	4	5	6	7	8	9	10	11	12
A								0.0397	0.0206	0.0547	0.0165	0.0324
B								0.0425	0.0414	0.0619	0.0775	0.048
C								0.2604	0.2575	0.3914	0.4074	0.3269
D								0.2642	0.3267	0.3612	0.3637	0.3151
E								0.1216	0.1055	0.1363	0.1223	0.0738
F								0.367	0.3299	0.3792	0.3882	0.3181
G								0.0401	0.0589	0.0586	0.0492	0.0473
H								0.3016	0.1984	0.2839	0.2621	0.2387

Cell Index at: 105:17:34

	1	2	3	4	5	6	7	8	9	10	11	12
A								0.0396	0.0222	0.0524	0.018	0.0322
B								0.0427	0.0403	0.0609	0.0743	0.0456
C								0.2611	0.2657	0.3868	0.4085	0.3218
D								0.2704	0.3343	0.3624	0.3664	0.3219
E								0.1101	0.0994	0.1421	0.1191	0.0681
F								0.3644	0.3271	0.3755	0.3892	0.3185
G								0.0427	0.0576	0.0644	0.0494	0.0469
H								0.2973	0.1998	0.2854	0.2653	0.2345

Cell Index at: 105:32:34

	1	2	3	4	5	6	7	8	9	10	11	12
A								0.0395	0.026	0.0497	0.0176	0.031
B								0.0407	0.0458	0.0601	0.0745	0.0489

C								0.2593	0.2615	0.3881	0.404	0.3248
D								0.2602	0.3234	0.3543	0.3621	0.3176
E								0.1086	0.106	0.1448	0.1218	0.0756
F								0.3719	0.3325	0.3794	0.3886	0.3236
G								0.0414	0.0562	0.0656	0.0513	0.0449
H								0.2997	0.1969	0.2853	0.2646	0.2383

Cell Index at: 105:47:35

	1	2	3	4	5	6	7	8	9	10	11	12
A								0.0359	0.0251	0.0504	0.0174	0.0329
B								0.0401	0.044	0.0618	0.0738	0.0495
C								0.2591	0.2648	0.3917	0.4096	0.3286
D								0.2588	0.321	0.3588	0.358	0.3229
E								0.1176	0.1079	0.1451	0.1221	0.0651
F								0.3763	0.3323	0.3785	0.3894	0.3213
G								0.0412	0.058	0.0632	0.0525	0.0441
H								0.2985	0.1978	0.2854	0.2663	0.2385

Cell Index at: 106:02:35

	1	2	3	4	5	6	7	8	9	10	11	12
A								0.0368	0.0249	0.0516	0.0219	0.0306
B								0.0448	0.0433	0.067	0.0744	0.0501
C								0.2648	0.2704	0.4071	0.4117	0.3239
D								0.2627	0.3299	0.3641	0.3588	0.3231
E								0.1119	0.1037	0.1443	0.1249	0.0697
F								0.3728	0.334	0.3823	0.3913	0.3189
G								0.0435	0.0636	0.0611	0.0523	0.0494
H								0.3008	0.2003	0.284	0.2645	0.2398

Cell Index at: 106:17:35

	1	2	3	4	5	6	7	8	9	10	11	12
A								0.0372	0.0255	0.0519	0.0229	0.0294
B								0.0417	0.0441	0.0643	0.0764	0.0502
C								0.2602	0.2702	0.4002	0.4144	0.323
D								0.2634	0.325	0.3636	0.3672	0.3214
E								0.1184	0.1064	0.1417	0.1228	0.0747
F								0.3718	0.3348	0.3798	0.3949	0.3263
G								0.0435	0.0519	0.0638	0.0524	0.0482
H								0.3078	0.2005	0.2834	0.2642	0.2379

Cell Index at: 106:32:36

	1	2	3	4	5	6	7	8	9	10	11	12
A								0.0326	0.0251	0.0487	0.02	0.0325
B								0.0389	0.0463	0.0646	0.0774	0.0473
C								0.2602	0.2691	0.4031	0.4131	0.3268
D								0.265	0.3174	0.3615	0.3705	0.3264
E								0.1185	0.1076	0.1438	0.1252	0.076
F								0.375	0.3364	0.3759	0.3936	0.3185
G								0.0476	0.0594	0.0646	0.05	0.0492
H								0.3068	0.2002	0.2832	0.2673	0.2425

Cell Index at: 106:47:37

	1	2	3	4	5	6	7	8	9	10	11	12
A								0.0334	0.0287	0.0501	0.0185	0.0316
B								0.0368	0.0508	0.0654	0.0823	0.0506
C								0.2621	0.2631	0.3999	0.4167	0.3225
D								0.2635	0.3173	0.3725	0.3734	0.3237
E								0.1146	0.1057	0.1376	0.1264	0.0746
F								0.3736	0.3385	0.3794	0.398	0.3241
G								0.0435	0.0533	0.0609	0.0554	0.0504
H								0.3071	0.2024	0.2842	0.2674	0.2418

Cell Index at: 107:02:37

	1	2	3	4	5	6	7	8	9	10	11	12
A								0.033	0.0283	0.0482	0.0179	0.0333
B								0.0424	0.0466	0.0645	0.0731	0.0519
C								0.2668	0.2664	0.4026	0.4195	0.3285
D								0.2726	0.3201	0.3635	0.3683	0.3246
E								0.127	0.104	0.141	0.1269	0.073
F								0.383	0.3376	0.3888	0.395	0.3262
G								0.0465	0.0582	0.0582	0.0559	0.0488
H								0.305	0.2058	0.2865	0.2699	0.2417

Cell Index at: 107:17:37

	1	2	3	4	5	6	7	8	9	10	11	12
A								0.0335	0.0279	0.0505	0.0198	0.0329
B								0.0362	0.0453	0.0637	0.0775	0.0501
C								0.2627	0.2708	0.3981	0.4246	0.3309
D								0.2699	0.3302	0.3658	0.3695	0.3278
E								0.127	0.1068	0.1439	0.1238	0.073
F								0.3864	0.3328	0.3818	0.396	0.3229
G								0.0466	0.0573	0.0618	0.0536	0.0486
H								0.3046	0.2064	0.2878	0.2721	0.2437

Cell Index at: 107:32:37

	1	2	3	4	5	6	7	8	9	10	11	12
A								0.032	0.0242	0.0505	0.0181	0.0318
B								0.0386	0.0474	0.0604	0.0751	0.052
C								0.2687	0.2683	0.4001	0.4211	0.3278
D								0.2688	0.3273	0.363	0.3747	0.3291
E								0.125	0.0957	0.1421	0.1256	0.0713
F								0.3866	0.3423	0.3841	0.4011	0.331
G								0.0505	0.0586	0.0682	0.0524	0.052
H								0.3043	0.2061	0.2907	0.2675	0.2412

Cell Index at: 107:47:37

	1	2	3	4	5	6	7	8	9	10	11	12
A								0.0287	0.0233	0.0529	0.0215	0.035
B								0.04	0.046	0.0644	0.0795	0.0524
C								0.2707	0.2738	0.4008	0.4251	0.3263
D								0.2787	0.3291	0.3701	0.3749	0.3287
E								0.1328	0.102	0.145	0.1243	0.0762
F								0.3847	0.3492	0.3849	0.4027	0.3277
G								0.0492	0.0556	0.0617	0.0575	0.0515
H								0.3061	0.209	0.2943	0.2715	0.2441

Cell Index at: 108:02:38

	1	2	3	4	5	6	7	8	9	10	11	12
A								0.0334	0.0225	0.0488	0.0223	0.036
B								0.0411	0.0471	0.0658	0.0797	0.05
C								0.2721	0.2672	0.4024	0.4293	0.3304
D								0.2774	0.3251	0.3685	0.3772	0.3251
E								0.124	0.1008	0.1378	0.1318	0.0771
F								0.3892	0.3455	0.3827	0.4001	0.3323
G								0.0454	0.0633	0.0664	0.0544	0.0533
H								0.307	0.2084	0.2935	0.2683	0.2488

Cell Index at: 108:17:39

	1	2	3	4	5	6	7	8	9	10	11	12
A								0.0309	0.0196	0.0519	0.0227	0.0342
B								0.0405	0.0418	0.0609	0.0803	0.0523
C								0.2706	0.2744	0.4052	0.424	0.3246
D								0.2738	0.3314	0.3641	0.3835	0.3233
E								0.126	0.101	0.1414	0.1249	0.0702
F								0.3899	0.3475	0.3829	0.4022	0.3317
G								0.0443	0.0566	0.0636	0.0562	0.0513
H								0.3083	0.2128	0.2947	0.2706	0.2474

Cell Index at: 108:32:39

	1	2	3	4	5	6	7	8	9	10	11	12
A								0.0294	0.0253	0.0503	0.0207	0.0391

	1	2	3	4	5	6	7	8	9	10	11	12
B								0.0347	0.0456	0.0554	0.0799	0.0565
C								0.2765	0.2755	0.4082	0.4229	0.3261
D								0.2811	0.3367	0.3701	0.3791	0.3257
E								0.1234	0.1058	0.1417	0.1333	0.0738
F								0.3876	0.3495	0.3886	0.4059	0.3321
G								0.0452	0.0588	0.0617	0.0553	0.05
H								0.3102	0.2112	0.2971	0.2726	0.2485

Cell Index at: 108:47:40

	1	2	3	4	5	6	7	8	9	10	11	12
A								0.0303	0.0225	0.0515	0.0183	0.0373
B								0.035	0.0438	0.0557	0.08	0.0546
C								0.2684	0.2759	0.4063	0.4284	0.3262
D								0.2806	0.3352	0.3699	0.379	0.3257
E								0.1201	0.0985	0.1384	0.1366	0.0742
F								0.3909	0.3528	0.3876	0.4045	0.3278
G								0.0452	0.0564	0.0648	0.0577	0.0483
H								0.3115	0.213	0.2937	0.2763	0.2489

Cell Index at: 109:02:40

	1	2	3	4	5	6	7	8	9	10	11	12
A								0.0314	0.0222	0.0556	0.0206	0.0391
B								0.0372	0.0469	0.0536	0.0782	0.0572
C								0.2778	0.2705	0.4105	0.4276	0.3234
D								0.2829	0.3398	0.3801	0.3787	0.3287
E								0.132	0.1009	0.1424	0.1332	0.081
F								0.3878	0.3603	0.3944	0.402	0.3266
G								0.0469	0.0602	0.0662	0.053	0.0431
H								0.3104	0.2092	0.293	0.2721	0.2512

Cell Index at: 109:17:41

	1	2	3	4	5	6	7	8	9	10	11	12
A								0.0283	0.0266	0.0569	0.0231	0.0368
B								0.0379	0.0444	0.0565	0.0819	0.0551
C								0.2709	0.2706	0.4093	0.4268	0.3286
D								0.2885	0.341	0.3776	0.379	0.3334
E								0.1229	0.0971	0.1371	0.1325	0.0754
F								0.3845	0.3545	0.3894	0.4036	0.3261
G								0.0417	0.0579	0.067	0.0586	0.0457
H								0.3096	0.2101	0.2937	0.2761	0.2488

Cell Index at: 109:32:41

	1	2	3	4	5	6	7	8	9	10	11	12
A								0.03	0.0295	0.0518	0.0234	0.0389
B								0.034	0.0482	0.0551	0.0807	0.0592
C								0.2779	0.2697	0.4132	0.427	0.3298
D								0.2852	0.3349	0.3761	0.3773	0.333
E								0.1253	0.1008	0.1364	0.1305	0.0748
F								0.3894	0.3577	0.3917	0.4063	0.3268
G								0.0486	0.0559	0.0671	0.0593	0.0491
H								0.312	0.2118	0.2978	0.2763	0.2505

Cell Index at: 109:47:41

	1	2	3	4	5	6	7	8	9	10	11	12
A								0.0326	0.0279	0.0528	0.0175	0.0405
B								0.0333	0.0493	0.0534	0.0817	0.0536
C								0.2805	0.2708	0.4053	0.4318	0.3302
D								0.2827	0.3283	0.3786	0.3891	0.3273
E								0.123	0.1034	0.1373	0.1324	0.0778
F								0.384	0.3508	0.3902	0.401	0.3257
G								0.0465	0.0621	0.0706	0.056	0.046
H								0.3135	0.2091	0.2962	0.2777	0.2514

Cell Index at: 110:02:41

	1	2	3	4	5	6	7	8	9	10	11	12
A								0.0305	0.0257	0.0544	0.017	0.0381
B								0.0339	0.0453	0.0549	0.0819	0.0561
C								0.2847	0.2655	0.4077	0.4343	0.3327
D								0.282	0.332	0.3828	0.3793	0.3294
E								0.129	0.1091	0.1339	0.1354	0.0764
F								0.3897	0.3572	0.3945	0.4014	0.3278
G								0.0447	0.0651	0.0681	0.053	0.0522
H								0.3108	0.2099	0.2963	0.2747	0.2531

Cell Index at: 110:17:40

	1	2	3	4	5	6	7	8	9	10	11	12
A								0.0315	0.0276	0.0561	0.0195	0.0393
B								0.0373	0.0485	0.0559	0.083	0.0592
C								0.2791	0.2715	0.4077	0.43	0.3312
D								0.2907	0.3277	0.3824	0.3843	0.3296
E								0.1296	0.1053	0.1398	0.129	0.0728
F								0.3965	0.3512	0.3876	0.4094	0.328
G								0.0475	0.0636	0.0698	0.0548	0.0488
H								0.3049	0.2144	0.2972	0.2715	0.2536

Cell Index at: 110:32:40

	1	2	3	4	5	6	7	8	9	10	11	12
A								0.028	0.0291	0.0566	0.0187	0.0402
B								0.0354	0.0448	0.0536	0.0805	0.0569
C								0.2774	0.2711	0.4163	0.4271	0.3355
D								0.2925	0.3377	0.3815	0.3916	0.3365
E								0.1199	0.1036	0.1302	0.134	0.0789
F								0.3909	0.3607	0.3919	0.4117	0.3274
G								0.0401	0.0636	0.067	0.0583	0.0482
H								0.3041	0.2185	0.299	0.2718	0.2583

Cell Index at: 110:47:40

	1	2	3	4	5	6	7	8	9	10	11	12
A								0.0278	0.0302	0.0578	0.0184	0.0384
B								0.0334	0.0483	0.0588	0.0847	0.056
C								0.2827	0.2772	0.4113	0.428	0.3355
D								0.292	0.339	0.3825	0.3853	0.3396
E								0.1273	0.1039	0.1441	0.1337	0.0794
F								0.3936	0.3561	0.3874	0.413	0.3283
G								0.0491	0.0673	0.0695	0.0578	0.0516
H								0.3062	0.2139	0.2968	0.2729	0.258

Cell Index at: 111:02:41

	1	2	3	4	5	6	7	8	9	10	11	12
A								0.0297	0.0324	0.0573	0.0183	0.0398
B								0.032	0.0465	0.062	0.0807	0.057
C								0.2757	0.273	0.4112	0.4319	0.3381
D								0.2953	0.3346	0.3762	0.3907	0.3332
E								0.1243	0.1063	0.1387	0.1294	0.0771
F								0.4002	0.3559	0.3906	0.4077	0.3338
G								0.0455	0.0656	0.0695	0.0553	0.0508
H								0.3032	0.2169	0.2984	0.2715	0.2584

Cell Index at: 111:17:42

	1	2	3	4	5	6	7	8	9	10	11	12
A								0.0329	0.0321	0.0572	0.0203	0.0402
B								0.0339	0.0451	0.0552	0.0802	0.0567
C								0.2818	0.2716	0.4021	0.4352	0.3374
D								0.296	0.3375	0.3877	0.388	0.3416
E								0.1288	0.1074	0.1374	0.1326	0.079
F								0.4027	0.3549	0.3941	0.4079	0.3317
G								0.0461	0.0664	0.0685	0.0608	0.0495
H								0.3088	0.2173	0.3026	0.272	0.2596

Cell Index at: 111:32:43

	1	2	3	4	5	6	7	8	9	10	11	12

A								0.0296	0.0346	0.0606	0.0221	0.0385
B								0.0356	0.0464	0.0563	0.0837	0.0537
C								0.2852	0.2816	0.4119	0.43	0.3329
D								0.2938	0.3436	0.3809	0.3979	0.337
E								0.1306	0.1057	0.1342	0.1308	0.0733
F								0.3995	0.3629	0.3948	0.4133	0.3332
G								0.0523	0.0653	0.0726	0.0591	0.052
H								0.3071	0.2158	0.3006	0.2771	0.2621

Cell Index at: 111:47:44

	1	2	3	4	5	6	7	8	9	10	11	12
A								0.0317	0.0346	0.0583	0.0214	0.0381
B								0.0362	0.0457	0.0573	0.0828	0.0567
C								0.2881	0.2715	0.4065	0.438	0.3362
D								0.2883	0.3308	0.3876	0.3863	0.3342
E								0.1326	0.1081	0.1454	0.1403	0.0766
F								0.4057	0.3574	0.3997	0.4121	0.3374
G								0.0467	0.0649	0.0688	0.0585	0.0494
H								0.3109	0.2197	0.2993	0.2716	0.2617

Cell Index at: 112:02:45

	1	2	3	4	5	6	7	8	9	10	11	12
A								0.0279	0.0305	0.0596	0.021	0.0398
B								0.0371	0.0453	0.0583	0.0842	0.0587
C								0.2829	0.2703	0.4092	0.4424	0.3375
D								0.2958	0.3396	0.3868	0.3894	0.3342
E								0.1369	0.1026	0.1374	0.1388	0.0763
F								0.4066	0.3674	0.4002	0.4194	0.333
G								0.045	0.0657	0.0678	0.0584	0.0507
H								0.3172	0.2171	0.2988	0.2759	0.2615

Cell Index at: 112:17:46

	1	2	3	4	5	6	7	8	9	10	11	12
A								0.0319	0.0312	0.0561	0.0214	0.0397
B								0.0342	0.0424	0.0567	0.0862	0.0572
C								0.2894	0.2747	0.4091	0.4408	0.3378
D								0.2873	0.3366	0.3836	0.3958	0.3399
E								0.1354	0.1058	0.1356	0.1296	0.0756
F								0.4077	0.3738	0.397	0.4152	0.3294
G								0.0462	0.0635	0.0661	0.0565	0.0521
H								0.314	0.2188	0.2991	0.2752	0.262

Cell Index at: 112:32:46

	1	2	3	4	5	6	7	8	9	10	11	12
A								0.0329	0.0316	0.0589	0.0213	0.0438
B								0.0334	0.0423	0.0583	0.0851	0.0596
C								0.2907	0.2765	0.4164	0.4478	0.3355
D								0.2949	0.3334	0.3882	0.3948	0.3428
E								0.1247	0.1084	0.1366	0.1353	0.0815
F								0.4045	0.3667	0.403	0.412	0.3319
G								0.0448	0.0629	0.0697	0.0608	0.0583
H								0.3222	0.2172	0.3002	0.2773	0.2607

Cell Index at: 112:47:47

	1	2	3	4	5	6	7	8	9	10	11	12
A								0.0333	0.0319	0.0577	0.0235	0.0415
B								0.0351	0.0452	0.0578	0.0843	0.0605
C								0.2882	0.2702	0.4126	0.4447	0.3393
D								0.2902	0.3318	0.3887	0.3926	0.3397
E								0.13	0.1049	0.1448	0.1327	0.079
F								0.4012	0.371	0.4017	0.4146	0.3292
G								0.0465	0.0648	0.07	0.0622	0.0539
H								0.3188	0.2174	0.3025	0.274	0.2613

Cell Index at: 113:02:48

	1	2	3	4	5	6	7	8	9	10	11	12
A								0.0332	0.03	0.0592	0.0236	0.0438
B								0.0401	0.0453	0.0582	0.0871	0.0584
C								0.2898	0.271	0.4094	0.4472	0.3383
D								0.2898	0.3365	0.3899	0.3846	0.3435
E								0.1268	0.1028	0.1453	0.1407	0.0846
F								0.4086	0.3702	0.4034	0.4203	0.3346
G								0.0422	0.0647	0.0666	0.0623	0.0584
H								0.3186	0.218	0.3025	0.2763	0.2642

Cell Index at: 113:17:49

	1	2	3	4	5	6	7	8	9	10	11	12
A								0.0342	0.0302	0.0565	0.0243	0.0431
B								0.0394	0.047	0.0584	0.0904	0.0611
C								0.2899	0.2691	0.4124	0.4437	0.3323
D								0.2993	0.3431	0.3867	0.3881	0.3375
E								0.1362	0.1043	0.1367	0.1356	0.084
F								0.4077	0.367	0.3985	0.4144	0.3405
G								0.0451	0.064	0.07	0.0515	0.0526
H								0.3231	0.2161	0.3063	0.2782	0.2629

Cell Index at: 113:32:49

	1	2	3	4	5	6	7	8	9	10	11	12
A								0.0367	0.0317	0.0603	0.0264	0.0421
B								0.0397	0.0441	0.0602	0.086	0.0591
C								0.2915	0.2783	0.4131	0.4468	0.3331
D								0.2982	0.3466	0.3931	0.3903	0.3373
E								0.1323	0.097	0.1385	0.1391	0.0785
F								0.4123	0.3673	0.4029	0.416	0.3429
G								0.0438	0.0645	0.0679	0.0562	0.0547
H								0.3232	0.2156	0.306	0.2762	0.2629

Cell Index at: 113:47:50

	1	2	3	4	5	6	7	8	9	10	11	12
A								0.0372	0.0334	0.0571	0.0253	0.0409
B								0.0434	0.0489	0.0574	0.0902	0.0629
C								0.2966	0.2813	0.4099	0.45	0.3395
D								0.2938	0.3388	0.3864	0.3932	0.3398
E								0.1314	0.1063	0.1403	0.1348	0.0792
F								0.4119	0.3701	0.4034	0.4122	0.3411
G								0.0457	0.0636	0.067	0.0527	0.0567
H								0.3204	0.2154	0.3065	0.2761	0.2644

Cell Index at: 114:02:50

	1	2	3	4	5	6	7	8	9	10	11	12
A								0.0353	0.0337	0.0595	0.0262	0.0437
B								0.0418	0.0453	0.0605	0.0902	0.0621
C								0.2947	0.279	0.4085	0.4518	0.3388
D								0.2981	0.3472	0.3825	0.3935	0.3453
E								0.1319	0.1054	0.1445	0.1412	0.0812
F								0.4118	0.3763	0.4037	0.4172	0.3407
G								0.047	0.063	0.0691	0.0542	0.0551
H								0.32	0.2152	0.3124	0.2795	0.266

Cell Index at: 114:17:51

	1	2	3	4	5	6	7	8	9	10	11	12
A								0.0375	0.034	0.0575	0.0253	0.0429
B								0.0409	0.0454	0.0577	0.0907	0.0599
C								0.2986	0.2865	0.4113	0.447	0.3335
D								0.2942	0.345	0.3953	0.3974	0.3392
E								0.1337	0.1054	0.1449	0.1354	0.0785
F								0.4106	0.3725	0.4013	0.4162	0.3458
G								0.0459	0.0657	0.072	0.0526	0.0588
H								0.3167	0.2129	0.3114	0.2779	0.2637

Cell Index at: 114:32:51

	1	2	3	4	5	6	7	8	9	10	11	12
A								0.0383	0.0307	0.0592	0.0273	0.0429
B								0.0406	0.0456	0.0555	0.0892	0.0595
C								0.2911	0.2793	0.4087	0.4454	0.3395
D								0.3003	0.3469	0.3931	0.398	0.348
E								0.13	0.1058	0.1461	0.1417	0.0806
F								0.4128	0.374	0.4023	0.4142	0.3391
G								0.042	0.0683	0.0689	0.0579	0.0529
H								0.3209	0.2163	0.3136	0.2806	0.264

Cell Index at: 114:47:51

	1	2	3	4	5	6	7	8	9	10	11	12
A								0.0323	0.0346	0.0599	0.0242	0.0423
B								0.0409	0.0445	0.0552	0.0882	0.0662
C								0.2983	0.2811	0.4137	0.4545	0.3346
D								0.3027	0.3449	0.3853	0.3928	0.3485
E								0.1333	0.0985	0.1497	0.1411	0.0796
F								0.4095	0.3738	0.3969	0.4175	0.3351
G								0.0442	0.0675	0.0669	0.0557	0.0574
H								0.3195	0.2144	0.3151	0.2784	0.2671

Cell Index at: 115:02:51

	1	2	3	4	5	6	7	8	9	10	11	12
A								0.0339	0.0348	0.0588	0.0238	0.0434
B								0.0396	0.0448	0.0545	0.09	0.0627
C								0.2969	0.2832	0.4135	0.4616	0.3359
D								0.2957	0.3402	0.3925	0.3948	0.3441
E								0.1304	0.1048	0.1484	0.1385	0.0783
F								0.4118	0.3742	0.4005	0.4186	0.336
G								0.0454	0.068	0.0673	0.0574	0.0539
H								0.3217	0.2161	0.3143	0.2789	0.2631

Cell Index at: 115:17:51

	1	2	3	4	5	6	7	8	9	10	11	12
A								0.0342	0.0345	0.0625	0.0229	0.0424
B								0.0427	0.0428	0.0594	0.0915	0.0656
C								0.2917	0.2776	0.4087	0.4598	0.3427
D								0.2891	0.3433	0.3872	0.3918	0.3431
E								0.1399	0.104	0.1402	0.1308	0.0864
F								0.4183	0.3755	0.4031	0.4223	0.3371
G								0.047	0.0682	0.0711	0.056	0.0589
H								0.3183	0.215	0.3162	0.2772	0.2695

Cell Index at: 115:32:51

	1	2	3	4	5	6	7	8	9	10	11	12
A								0.0338	0.0347	0.0617	0.0262	0.0436
B								0.0447	0.0447	0.0568	0.0937	0.0668
C								0.2949	0.283	0.4167	0.4583	0.3414
D								0.3004	0.3432	0.396	0.3978	0.3457
E								0.138	0.1028	0.1448	0.1372	0.0861
F								0.4142	0.3695	0.4071	0.4215	0.3408
G								0.0453	0.0651	0.0686	0.0575	0.0584
H								0.3174	0.2185	0.3161	0.2822	0.2661

Cell Index at: 115:47:51

	1	2	3	4	5	6	7	8	9	10	11	12
A								0.0361	0.0351	0.0629	0.0205	0.0434
B								0.0465	0.0461	0.0574	0.0896	0.0676
C								0.2943	0.292	0.4164	0.4571	0.3384
D								0.2901	0.3448	0.3988	0.4019	0.3448
E								0.1361	0.1067	0.1478	0.1305	0.084
F								0.4131	0.3713	0.4021	0.4215	0.3411
G								0.0504	0.0657	0.0717	0.0604	0.0542
H								0.3191	0.2186	0.3171	0.275	0.2662

Cell Index at: 116:02:52

	1	2	3	4	5	6	7	8	9	10	11	12
A								0.0349	0.0385	0.0591	0.0225	0.0405
B								0.0449	0.0499	0.0619	0.0929	0.0663
C								0.2978	0.2873	0.4204	0.4615	0.3416
D								0.3003	0.3404	0.3954	0.404	0.3514
E								0.1408	0.1055	0.1442	0.1318	0.0844
F								0.4097	0.3777	0.4094	0.4213	0.3409
G								0.0471	0.0634	0.0667	0.054	0.0583
H								0.319	0.2171	0.3127	0.2762	0.2679

Cell Index at: 116:17:52

	1	2	3	4	5	6	7	8	9	10	11	12
A								0.0357	0.0387	0.0609	0.0234	0.0426
B								0.0477	0.044	0.062	0.0872	0.0688
C								0.2962	0.2901	0.4168	0.4655	0.3403
D								0.3008	0.3461	0.3973	0.4094	0.3478
E								0.1355	0.1098	0.1487	0.1334	0.0813
F								0.4207	0.3706	0.4035	0.423	0.3441
G								0.0472	0.0703	0.0707	0.0618	0.0563
H								0.3193	0.2149	0.3114	0.2764	0.2692

Cell Index at: 116:32:52

	1	2	3	4	5	6	7	8	9	10	11	12
A								0.0359	0.0352	0.0619	0.0207	0.0453
B								0.0479	0.0431	0.0617	0.0924	0.0681
C								0.2999	0.2824	0.4151	0.4614	0.3403
D								0.2973	0.3515	0.3963	0.4066	0.3525
E								0.1319	0.1096	0.1421	0.1347	0.0795
F								0.4149	0.3758	0.4034	0.4232	0.3454
G								0.0494	0.065	0.0676	0.0569	0.0557
H								0.3231	0.2154	0.3173	0.2755	0.267

Cell Index at: 116:47:53

	1	2	3	4	5	6	7	8	9	10	11	12
A								0.0353	0.0336	0.0582	0.0229	0.0404
B								0.0464	0.0378	0.0631	0.0913	0.067
C								0.3052	0.2889	0.4185	0.4621	0.3378
D								0.299	0.3432	0.4032	0.4037	0.353
E								0.1406	0.1074	0.1459	0.1397	0.0832
F								0.4243	0.3749	0.4012	0.4282	0.3428
G								0.048	0.0675	0.0697	0.0574	0.0538
H								0.3232	0.2156	0.3145	0.2771	0.2691

Cell Index at: 117:02:54

	1	2	3	4	5	6	7	8	9	10	11	12
A								0.0405	0.0331	0.0582	0.0207	0.0442
B								0.0485	0.0405	0.0634	0.0916	0.0671
C								0.3034	0.2864	0.4165	0.458	0.3413
D								0.2995	0.3435	0.3954	0.4075	0.3533
E								0.1314	0.1086	0.1455	0.1403	0.0818
F								0.4204	0.3777	0.4071	0.424	0.3513
G								0.0469	0.0677	0.0707	0.0572	0.0543
H								0.3241	0.2139	0.3169	0.2803	0.2681

Cell Index at: 117:17:55

	1	2	3	4	5	6	7	8	9	10	11	12
A								0.0398	0.0326	0.0631	0.0208	0.0431
B								0.0483	0.0422	0.061	0.0891	0.0687
C								0.3059	0.2905	0.4169	0.4617	0.3419
D								0.3009	0.3491	0.3982	0.4012	0.3473
E								0.1321	0.1109	0.1563	0.1375	0.0809
F								0.4239	0.3825	0.4037	0.4248	0.346
G								0.0473	0.0684	0.0707	0.0576	0.0554
H								0.3255	0.2162	0.3186	0.2798	0.2704

Cell Index at: 117:32:55

	1	2	3	4	5	6	7	8	9	10	11	12
A								0.0392	0.0314	0.0603	0.0203	0.0411
B								0.0451	0.0442	0.0637	0.0921	0.0693
C								0.3056	0.2904	0.4181	0.4651	0.3414
D								0.2965	0.3504	0.4037	0.4035	0.3544
E								0.1291	0.1101	0.1418	0.1387	0.081
F								0.4257	0.3817	0.4104	0.4294	0.3526
G								0.0504	0.0665	0.0697	0.0562	0.0576
H								0.3267	0.2188	0.3185	0.2817	0.2692

Cell Index at: 117:47:55

	1	2	3	4	5	6	7	8	9	10	11	12
A								0.0442	0.0324	0.0608	0.0222	0.044
B								0.0459	0.0422	0.0651	0.0932	0.0685
C								0.3106	0.2898	0.4225	0.4606	0.3427
D								0.292	0.3568	0.3964	0.4036	0.3503
E								0.1361	0.1112	0.1514	0.1352	0.0785
F								0.4182	0.3798	0.406	0.4281	0.3502
G								0.0466	0.0674	0.072	0.0597	0.0605
H								0.3282	0.2203	0.3186	0.28	0.2694

Cell Index at: 118:02:56

	1	2	3	4	5	6	7	8	9	10	11	12
A								0.0446	0.0333	0.0577	0.0234	0.045
B								0.0422	0.0401	0.0606	0.09	0.0665
C								0.3074	0.2915	0.4292	0.4653	0.3453
D								0.3001	0.3467	0.4079	0.4056	0.3511
E								0.1395	0.1076	0.15	0.1366	0.0805
F								0.4225	0.3781	0.4076	0.4292	0.3502
G								0.0504	0.0715	0.0738	0.0556	0.0582
H								0.3264	0.222	0.3245	0.2802	0.2716

Cell Index at: 118:17:57

	1	2	3	4	5	6	7	8	9	10	11	12
A								0.0439	0.032	0.06	0.0229	0.0452
B								0.0472	0.0428	0.0629	0.0945	0.0696
C								0.3055	0.2874	0.4354	0.4684	0.3447
D								0.2975	0.3516	0.4013	0.4074	0.3496
E								0.1386	0.1169	0.157	0.1383	0.079
F								0.4253	0.3774	0.4094	0.4276	0.352
G								0.0525	0.067	0.0749	0.0569	0.0589
H								0.3294	0.2227	0.3242	0.2811	0.2708

Cell Index at: 118:32:58

	1	2	3	4	5	6	7	8	9	10	11	12
A								0.0477	0.0379	0.0566	0.0236	0.0445
B								0.0456	0.0405	0.0644	0.0906	0.0724
C								0.3149	0.2903	0.4296	0.4686	0.3465
D								0.2912	0.3478	0.3969	0.4119	0.3591
E								0.1345	0.1087	0.1509	0.1314	0.0808
F								0.4298	0.382	0.4123	0.4301	0.3527
G								0.0515	0.0697	0.0746	0.0558	0.0524
H								0.3284	0.2263	0.3239	0.2799	0.2733

Cell Index at: 118:47:58

	1	2	3	4	5	6	7	8	9	10	11	12
A								0.0489	0.0371	0.0566	0.0242	0.0434
B								0.0451	0.0394	0.0629	0.0882	0.0694
C								0.3078	0.2872	0.4343	0.4664	0.3472
D								0.2994	0.3457	0.4041	0.4065	0.3566
E								0.1386	0.1157	0.1524	0.1368	0.0832
F								0.4323	0.3839	0.4099	0.4303	0.3503
G								0.0504	0.0658	0.0719	0.057	0.0553
H								0.3285	0.2294	0.3231	0.278	0.2726

Cell Index at: 119:02:58

	1	2	3	4	5	6	7	8	9	10	11	12
A								0.0458	0.0373	0.0566	0.0243	0.0418
B								0.0393	0.0417	0.062	0.088	0.0697
C								0.3141	0.2932	0.4295	0.4629	0.3439
D								0.3105	0.3501	0.4032	0.4212	0.3573
E								0.1358	0.118	0.1431	0.1376	0.0839
F								0.4293	0.3777	0.4053	0.4333	0.349
G								0.0487	0.0739	0.073	0.0554	0.0605
H								0.3345	0.2262	0.3251	0.283	0.2724

Cell Index at: 119:17:59

	1	2	3	4	5	6	7	8	9	10	11	12
A								0.0456	0.0367	0.0595	0.0287	0.0413
B								0.0419	0.0423	0.0602	0.0912	0.0723
C								0.3101	0.2908	0.4304	0.4611	0.3539
D								0.2977	0.354	0.4118	0.4149	0.3563
E								0.1415	0.1073	0.1507	0.1439	0.0811
F								0.4258	0.384	0.4078	0.4274	0.3532
G								0.0429	0.0686	0.0741	0.0554	0.0608
H								0.3313	0.2229	0.3259	0.2799	0.273

Cell Index at: 119:32:59

	1	2	3	4	5	6	7	8	9	10	11	12
A								0.0503	0.0357	0.0582	0.0279	0.04
B								0.0445	0.0425	0.0633	0.0926	0.0754
C								0.3106	0.2954	0.431	0.4645	0.3471
D								0.302	0.3432	0.4094	0.4144	0.3563
E								0.1436	0.1101	0.1527	0.1332	0.0793
F								0.4286	0.381	0.4059	0.4397	0.3501
G								0.0526	0.0719	0.0756	0.0509	0.0611
H								0.3371	0.2229	0.3264	0.2811	0.274

Cell Index at: 119:48:00

	1	2	3	4	5	6	7	8	9	10	11	12
A								0.0467	0.0367	0.0536	0.0273	0.0438
B								0.0456	0.0425	0.0669	0.0891	0.066
C								0.309	0.297	0.4316	0.4729	0.3537
D								0.3081	0.3484	0.4082	0.4211	0.3679
E								0.1447	0.1179	0.1529	0.1349	0.0764
F								0.4331	0.3833	0.4149	0.4304	0.3566
G								0.0448	0.0735	0.0721	0.0547	0.0622
H								0.3363	0.2234	0.3296	0.286	0.2713

Cell Index at: 120:03:01

	1	2	3	4	5	6	7	8	9	10	11	12
A								0.0449	0.037	0.056	0.0284	0.0374
B								0.044	0.0401	0.0672	0.09	0.0707
C								0.3165	0.2946	0.4377	0.4645	0.3562
D								0.3037	0.3474	0.4074	0.414	0.3641
E								0.1419	0.1128	0.1534	0.1464	0.0786
F								0.4299	0.382	0.4159	0.4312	0.3525
G								0.0439	0.0725	0.0722	0.0536	0.0664
H								0.3333	0.2276	0.3266	0.2853	0.2737

A vs B	0.08066	B vs C	0.000425	C vs D	0.871053	D vs E	2.31E-05	E vs F	1.03E-06	F vs G	4.14E-06	G vs H	0.000139
A vs C	0.000481	B vs D	1.68E-05	C vs E	0.00077	D vs F	0.208677	E vs G	0.006546	F vs H	0.001997		
A vs D	4.86E-05	B vs E	0.006277	C vs F	0.469646	D vs G	5.05E-05	E vs H	0.000201				
A vs E	0.002192	B vs F	5.48E-07	C vs G	0.000584	D vs H	0.023605						
A vs F	5.31E-06	B vs G	0.951531	C vs H	0.067283								
A vs G	0.02121	B vs H	5.41E-05										
A vs H	0.000119												

Cell Index at: 120:18:01

	1	2	3	4	5	6	7	8	9	10	11	12
A								0.0449	0.0363	0.0561	0.0278	0.0434
B								0.0459	0.0427	0.0663	0.0907	0.0655
C								0.3103	0.2932	0.4396	0.4638	0.3548
D								0.2953	0.3521	0.4094	0.4155	0.3677
E								0.1393	0.1188	0.1494	0.1367	0.0762
F								0.4313	0.3809	0.4135	0.4315	0.3524
G								0.0448	0.0731	0.0748	0.0523	0.0654

Count	*Sum*	*Average*	*Variance*
5	0.2037	0.04074	0.000107
5	0.312	0.0624	0.000422
5	1.8695	0.3739	0.005544
5	1.8366	0.36732	0.00206
5	0.6331	0.12662	0.00096

H								0.3389	0.2292	0.3289	0.2872	0.2711

Cell Index at: 120:33:01

	1	2	3	4	5	6	7	8	9	10	11	12
A								0.0461	0.0382	0.061	0.0305	0.0456
B								0.0445	0.0402	0.0675	0.0947	0.0673
C								0.3114	0.2978	0.4348	0.4705	0.3488
D								0.3086	0.3467	0.414	0.4131	0.3626
E								0.1405	0.1141	0.1541	0.1417	0.0858
F								0.4386	0.3839	0.4149	0.4311	0.3613
G								0.0476	0.0775	0.0751	0.052	0.0662
H								0.3382	0.2316	0.3302	0.2885	0.2717

5	2.0115	0.4023	0.001169
5	0.3086	0.06172	0.000158
5	1.4465	0.2893	0.001848

SS	*df*	*MS*	*F*	*P-value*	*F crit*
0.870046	7	0.124292	81.05397	1.53E-18	2.312741
0.04907	32	0.001533			
0.919116	39				

Cell Index at: 120:48:02

	1	2	3	4	5	6	7	8	9	10	11	12
A								0.0496	0.0335	0.0628	0.0285	0.0475
B								0.0457	0.0454	0.0651	0.0891	0.0683
C								0.3154	0.2985	0.4342	0.4677	0.3507
D								0.3053	0.3547	0.4116	0.4204	0.3687
E								0.146	0.112	0.1583	0.1435	0.0783
F								0.4386	0.3907	0.4155	0.4325	0.3572
G								0.0495	0.0733	0.0777	0.0508	0.0633
H								0.344	0.2303	0.3286	0.291	0.2716

Cell Index at: 121:03:02

	1	2	3	4	5	6	7	8	9	10	11	12
A								0.0481	0.0361	0.063	0.0296	0.0492
B								0.0427	0.046	0.0695	0.0919	0.0678
C								0.3115	0.296	0.4347	0.4644	0.354
D								0.3088	0.3585	0.4186	0.4151	0.3688
E								0.1373	0.1231	0.1579	0.1451	0.0777
F								0.4444	0.3853	0.4173	0.4351	0.3641
G								0.0461	0.0775	0.0777	0.0526	0.0661
H								0.3392	0.2294	0.336	0.2905	0.2755

Cell Index at: 121:18:02

	1	2	3	4	5	6	7	8	9	10	11	12
A								0.0501	0.0337	0.0602	0.0301	0.0485
B								0.0422	0.0463	0.0632	0.089	0.0674
C								0.3216	0.3033	0.44	0.4719	0.357
D								0.3061	0.3567	0.4157	0.423	0.366
E								0.1329	0.1138	0.1583	0.1411	0.0814
F								0.4324	0.395	0.4184	0.4358	0.361
G								0.0483	0.0679	0.0813	0.0501	0.0666
H								0.3393	0.2277	0.336	0.2912	0.2733

Cell Index at: 121:33:03

	1	2	3	4	5	6	7	8	9	10	11	12
A								0.0509	0.0372	0.0625	0.031	0.0489
B								0.0492	0.0461	0.0667	0.0912	0.0702
C								0.3214	0.308	0.4346	0.4715	0.3633
D								0.3048	0.3663	0.4146	0.4202	0.3723
E								0.1404	0.1131	0.149	0.1352	0.0869
F								0.4415	0.3903	0.4136	0.4273	0.3523
G								0.0451	0.0747	0.0749	0.05	0.0671
H								0.3393	0.2304	0.3388	0.2923	0.2765

Cell Index at: 121:48:04

	1	2	3	4	5	6	7	8	9	10	11	12
A								0.0536	0.0394	0.0636	0.0336	0.049
B								0.0443	0.0466	0.0667	0.0903	0.0718
C								0.329	0.3074	0.4381	0.4789	0.36
D								0.3024	0.3585	0.4184	0.4273	0.372
E								0.1376	0.114	0.1602	0.1433	0.083
F								0.4328	0.3918	0.4086	0.4355	0.3573
G								0.0455	0.0732	0.0692	0.0535	0.0659
H								0.3343	0.2296	0.3429	0.2882	0.2803

Cell Index at: 122:03:05

	1	2	3	4	5	6	7	8	9	10	11	12
A								0.0527	0.0422	0.0628	0.0343	0.0496
B								0.0499	0.0453	0.0663	0.0896	0.0702
C								0.3294	0.305	0.4411	0.4762	0.3689
D								0.3035	0.3578	0.4144	0.4196	0.3732
E								0.142	0.1203	0.1596	0.1374	0.0779
F								0.4377	0.3904	0.4099	0.4344	0.3592
G								0.0479	0.0754	0.0725	0.0519	0.0675
H								0.3416	0.2336	0.3412	0.2885	0.2777

Cell Index at: 122:18:06

	1	2	3	4	5	6	7	8	9	10	11	12
A								0.0543	0.045	0.0667	0.0315	0.0484
B								0.0506	0.0467	0.0673	0.0904	0.0699
C								0.3298	0.3067	0.4456	0.481	0.3687
D								0.3059	0.3529	0.4201	0.4238	0.3747
E								0.1333	0.1119	0.1522	0.1476	0.0816
F								0.4387	0.3862	0.4103	0.4374	0.3579
G								0.0467	0.0748	0.0745	0.0523	0.0628
H								0.3353	0.2313	0.3413	0.2926	0.2797

Cell Index at: 122:33:06

	1	2	3	4	5	6	7	8	9	10	11	12
A								0.0541	0.0489	0.0693	0.0322	0.0501
B								0.0516	0.049	0.0643	0.09	0.0742
C								0.3273	0.3058	0.4468	0.4803	0.3647
D								0.3029	0.3664	0.4217	0.4294	0.3795
E								0.1385	0.1168	0.1531	0.1451	0.081
F								0.4384	0.3939	0.4127	0.4354	0.355
G								0.0477	0.0731	0.0756	0.0588	0.0658
H								0.3401	0.2327	0.3416	0.291	0.28

Cell Index at: 122:48:06

	1	2	3	4	5	6	7	8	9	10	11	12
A								0.0573	0.0474	0.0709	0.0305	0.0476
B								0.0493	0.0468	0.0677	0.0948	0.0684
C								0.3228	0.3051	0.4472	0.4742	0.3642
D								0.3076	0.3696	0.421	0.4281	0.3796
E								0.1395	0.1195	0.1589	0.1378	0.0867
F								0.4413	0.3882	0.4126	0.4337	0.358
G								0.0465	0.0753	0.0752	0.0574	0.0677
H								0.3397	0.2319	0.343	0.2938	0.2805

Cell Index at: 123:03:07

	1	2	3	4	5	6	7	8	9	10	11	12
A								0.0531	0.0488	0.0687	0.0293	0.0483
B								0.0482	0.0482	0.0676	0.0937	0.0728
C								0.329	0.3066	0.446	0.487	0.3656
D								0.3091	0.3624	0.4169	0.43	0.3798
E								0.1477	0.114	0.1494	0.1428	0.0824
F								0.4483	0.3834	0.4176	0.4382	0.3643
G								0.0451	0.0788	0.0727	0.0533	0.0671
H								0.3427	0.2319	0.3462	0.2931	0.2807

Cell Index at: 123:18:07

	1	2	3	4	5	6	7	8	9	10	11	12
A								0.0597	0.0464	0.0708	0.0315	0.05
B								0.0485	0.0475	0.0646	0.0955	0.0761
C								0.3229	0.3018	0.4434	0.4867	0.3689
D								0.3134	0.3598	0.412	0.4338	0.3824
E								0.1411	0.1157	0.1459	0.1406	0.0765

	1	2	3	4	5	6	7	8	9	10	11	12
F								0.4437	0.3908	0.4163	0.4399	0.3624
G								0.0482	0.0753	0.0774	0.0529	0.0662
H								0.3477	0.2338	0.3443	0.2903	0.2817

Cell Index at: 123:33:08

	1	2	3	4	5	6	7	8	9	10	11	12
A								0.0601	0.0507	0.0709	0.0305	0.0519
B								0.0486	0.0457	0.0661	0.097	0.0759
C								0.3265	0.3057	0.4385	0.4827	0.3675
D								0.3116	0.3605	0.419	0.4336	0.3752
E								0.1351	0.1191	0.1503	0.1396	0.0838
F								0.4468	0.3814	0.4247	0.4413	0.3646
G								0.0548	0.0777	0.0787	0.052	0.0677
H								0.3496	0.2409	0.3478	0.2888	0.281

Cell Index at: 123:48:08

	1	2	3	4	5	6	7	8	9	10	11	12
A								0.0583	0.0497	0.0712	0.0301	0.0528
B								0.0464	0.0511	0.0668	0.0922	0.0787
C								0.3313	0.2987	0.4451	0.4751	0.3703
D								0.3061	0.369	0.4191	0.4323	0.3736
E								0.1347	0.1211	0.151	0.1443	0.0839
F								0.4371	0.3887	0.4243	0.4444	0.364
G								0.0495	0.0802	0.0758	0.0549	0.0701
H								0.3482	0.2374	0.3488	0.2913	0.2802

Cell Index at: 124:03:09

	1	2	3	4	5	6	7	8	9	10	11	12
A								0.0623	0.0472	0.0725	0.0309	0.0522
B								0.0457	0.0474	0.0686	0.0945	0.0805
C								0.3316	0.305	0.4482	0.4938	0.3678
D								0.3138	0.3663	0.4296	0.4281	0.3756
E								0.1403	0.1066	0.1431	0.1415	0.0845
F								0.4443	0.3903	0.4201	0.4481	0.3697
G								0.052	0.0789	0.0763	0.0538	0.0727
H								0.3511	0.2398	0.3482	0.2935	0.2818

Cell Index at: 124:18:09

	1	2	3	4	5	6	7	8	9	10	11	12
A								0.0635	0.0503	0.0749	0.0333	0.0532
B								0.0489	0.0511	0.0674	0.0922	0.0744
C								0.3345	0.302	0.449	0.4898	0.3738
D								0.3116	0.3672	0.4266	0.4258	0.378
E								0.1413	0.1199	0.1516	0.1433	0.0805
F								0.4425	0.3828	0.4183	0.4485	0.3688
G								0.0485	0.0786	0.0764	0.0569	0.0702
H								0.3483	0.2393	0.3513	0.2912	0.2848

Cell Index at: 124:33:08

	1	2	3	4	5	6	7	8	9	10	11	12
A								0.0664	0.0475	0.075	0.0314	0.0549
B								0.0504	0.0535	0.0658	0.09	0.0774
C								0.332	0.2909	0.4513	0.4873	0.3749
D								0.3125	0.3669	0.4303	0.4297	0.3793
E								0.1363	0.1188	0.1459	0.1329	0.0783
F								0.4481	0.3909	0.4237	0.4466	0.3679
G								0.0529	0.077	0.0732	0.0599	0.0714
H								0.351	0.2394	0.3477	0.2907	0.285

Cell Index at: 124:48:08

	1	2	3	4	5	6	7	8	9	10	11	12
A								0.0696	0.0476	0.0749	0.0292	0.055
B								0.047	0.053	0.0699	0.0909	0.0779
C								0.3324	0.2966	0.4489	0.4864	0.3729
D								0.3141	0.3715	0.4233	0.421	0.3817
E								0.1375	0.1201	0.1547	0.1315	0.0824
F								0.4446	0.3867	0.4301	0.4458	0.3698
G								0.0509	0.0741	0.0756	0.0636	0.0768
H								0.3503	0.2419	0.3487	0.2924	0.2853

Cell Index at: 125:03:08

	1	2	3	4	5	6	7	8	9	10	11	12
A								0.0712	0.0477	0.0789	0.0295	0.056
B								0.0466	0.0553	0.0697	0.089	0.0807
C								0.3354	0.3008	0.455	0.4903	0.3779
D								0.3159	0.3766	0.4252	0.4241	0.3787
E								0.1415	0.1255	0.1513	0.1337	0.0852
F								0.4491	0.3905	0.4318	0.4547	0.3713
G								0.05	0.0809	0.0769	0.0603	0.0732
H								0.3512	0.242	0.3515	0.2905	0.2852

Cell Index at: 125:18:09

	1	2	3	4	5	6	7	8	9	10	11	12
A								0.0731	0.0494	0.0795	0.0273	0.0549
B								0.0477	0.0567	0.069	0.0947	0.0798
C								0.3429	0.2991	0.4647	0.491	0.375
D								0.3198	0.3764	0.4352	0.4302	0.377
E								0.1356	0.1238	0.1539	0.1369	0.0839
F								0.4481	0.3918	0.4338	0.4487	0.3714
G								0.0483	0.0767	0.0765	0.0589	0.0758
H								0.3533	0.2438	0.3525	0.294	0.2875

Cell Index at: 125:33:10

	1	2	3	4	5	6	7	8	9	10	11	12
A								0.0719	0.0511	0.0782	0.028	0.058
B								0.051	0.0517	0.0702	0.0965	0.0776
C								0.3324	0.2926	0.4593	0.4895	0.373
D								0.3088	0.3769	0.4305	0.4242	0.3731
E								0.1411	0.1259	0.15	0.1334	0.0826
F								0.4513	0.4028	0.4333	0.4419	0.3715
G								0.0531	0.0766	0.0791	0.0551	0.072
H								0.3541	0.2397	0.353	0.2923	0.29

Cell Index at: 125:48:11

	1	2	3	4	5	6	7	8	9	10	11	12
A								0.0726	0.05	0.0784	0.0276	0.0581
B								0.0519	0.0506	0.0628	0.0934	0.0796
C								0.3328	0.296	0.4551	0.4888	0.3768
D								0.3228	0.3699	0.4364	0.4293	0.3837
E								0.1415	0.1251	0.1533	0.1279	0.0863
F								0.447	0.4003	0.4336	0.4431	0.3716
G								0.0522	0.0834	0.074	0.0597	0.0744
H								0.3551	0.2423	0.3542	0.2914	0.2956

Cell Index at: 126:03:12

	1	2	3	4	5	6	7	8	9	10	11	12
A								0.0725	0.0481	0.076	0.0297	0.0564
B								0.0496	0.0508	0.0668	0.0923	0.0827
C								0.3368	0.296	0.4587	0.4919	0.3812
D								0.3192	0.3757	0.4403	0.427	0.3818
E								0.1386	0.1312	0.1559	0.1428	0.0782
F								0.447	0.3999	0.4282	0.4505	0.3702
G								0.0529	0.082	0.0759	0.0611	0.0747
H								0.3543	0.2412	0.3524	0.2942	0.2947

Cell Index at: 126:18:13

	1	2	3	4	5	6	7	8	9	10	11	12
A								0.0741	0.0527	0.076	0.0311	0.0597
B								0.0581	0.0514	0.0676	0.0931	0.0849
C								0.3443	0.2981	0.4604	0.4909	0.3868
D								0.3249	0.3745	0.4345	0.4188	0.3775

	1	2	3	4	5	6	7	8	9	10	11	12
E								0.1461	0.1244	0.1499	0.1419	0.0803
F								0.4568	0.3996	0.4362	0.45	0.3727
G								0.0539	0.0822	0.0778	0.0594	0.0737
H								0.3574	0.2394	0.3549	0.2938	0.2939

Cell Index at: 126:33:14

	1	2	3	4	5	6	7	8	9	10	11	12
A								0.0727	0.0531	0.0758	0.0321	0.0582
B								0.0522	0.0531	0.0658	0.0918	0.081
C								0.3368	0.2944	0.4607	0.4896	0.3751
D								0.3244	0.3619	0.4365	0.4299	0.385
E								0.1381	0.1252	0.1596	0.1473	0.0814
F								0.4454	0.3985	0.4335	0.4459	0.3775
G								0.0547	0.0834	0.0763	0.0631	0.0759
H								0.3574	0.2429	0.3592	0.2937	0.2972

Cell Index at: 126:48:14

	1	2	3	4	5	6	7	8	9	10	11	12
A								0.0711	0.0504	0.081	0.033	0.0582
B								0.0536	0.0543	0.0684	0.0909	0.0801
C								0.3374	0.3025	0.459	0.4897	0.3842
D								0.3346	0.3786	0.4395	0.4305	0.3837
E								0.144	0.1234	0.1523	0.1393	0.0788
F								0.4531	0.3972	0.4311	0.4459	0.3807
G								0.0566	0.0874	0.0749	0.0631	0.0753
H								0.3593	0.2418	0.3591	0.2947	0.2992

Cell Index at: 127:03:15

	1	2	3	4	5	6	7	8	9	10	11	12
A								0.0727	0.053	0.0843	0.0337	0.0563
B								0.0535	0.0537	0.067	0.0867	0.0837
C								0.3402	0.2985	0.4635	0.4901	0.3845
D								0.3357	0.3737	0.4381	0.4343	0.3847
E								0.1437	0.116	0.1558	0.1417	0.0892
F								0.4499	0.3987	0.4335	0.444	0.3796
G								0.0566	0.0888	0.0738	0.0665	0.0734
H								0.3626	0.2462	0.3611	0.2943	0.3006

Cell Index at: 127:18:15

	1	2	3	4	5	6	7	8	9	10	11	12
A								0.0726	0.0537	0.0825	0.0332	0.0531
B								0.0514	0.0512	0.0667	0.0957	0.0803
C								0.3372	0.2965	0.4617	0.4883	0.389
D								0.3254	0.3783	0.4302	0.4278	0.3779
E								0.1362	0.1195	0.158	0.1406	0.0833
F								0.452	0.3988	0.4279	0.4436	0.3785
G								0.0542	0.0882	0.078	0.0635	0.0744
H								0.357	0.246	0.363	0.2964	0.3017

Cell Index at: 127:33:16

	1	2	3	4	5	6	7	8	9	10	11	12
A								0.0762	0.053	0.0853	0.0372	0.0531
B								0.0506	0.0563	0.068	0.0938	0.087
C								0.3437	0.3048	0.4609	0.4896	0.3843
D								0.327	0.378	0.4369	0.4285	0.3834
E								0.1478	0.1122	0.1542	0.1351	0.0858
F								0.4557	0.4024	0.4283	0.4425	0.3833
G								0.0531	0.0862	0.0777	0.0612	0.0738
H								0.3564	0.2459	0.3648	0.2975	0.2995

Cell Index at: 127:48:16

	1	2	3	4	5	6	7	8	9	10	11	12
A								0.0742	0.0518	0.0852	0.0308	0.0548
B								0.052	0.0559	0.0665	0.0926	0.0833
C								0.3403	0.2974	0.4581	0.487	0.3799
D								0.3234	0.3756	0.4318	0.4339	0.3769
E								0.1516	0.105	0.1599	0.1316	0.0877
F								0.4483	0.4024	0.4354	0.4447	0.381
G								0.0547	0.0881	0.0832	0.0648	0.0774
H								0.3603	0.2472	0.3639	0.2994	0.2994

Cell Index at: 128:03:17

	1	2	3	4	5	6	7	8	9	10	11	12
A								0.0732	0.0507	0.0837	0.0332	0.0539
B								0.0532	0.0587	0.0682	0.0963	0.0786
C								0.3433	0.2986	0.4641	0.4854	0.3852
D								0.332	0.3841	0.4382	0.4313	0.3735
E								0.1401	0.1024	0.163	0.1411	0.0834
F								0.4494	0.4049	0.436	0.4438	0.3898
G								0.0553	0.0843	0.0809	0.0679	0.0723
H								0.3605	0.2454	0.3632	0.3	0.2976

Cell Index at: 128:18:17

	1	2	3	4	5	6	7	8	9	10	11	12
A								0.0726	0.0511	0.0836	0.0354	0.0566
B								0.0504	0.0573	0.0697	0.094	0.0801
C								0.3382	0.2982	0.4642	0.4908	0.3851
D								0.326	0.3788	0.4303	0.429	0.3888
E								0.1441	0.1123	0.1613	0.1406	0.0865
F								0.4535	0.403	0.4327	0.4499	0.3783
G								0.052	0.0818	0.082	0.0678	0.0726
H								0.363	0.2463	0.3612	0.2988	0.3002

Cell Index at: 128:33:17

	1	2	3	4	5	6	7	8	9	10	11	12
A								0.0746	0.0526	0.0812	0.0344	0.0569
B								0.0525	0.0575	0.0739	0.0938	0.0783
C								0.3531	0.2999	0.4693	0.4938	0.3936
D								0.3235	0.3818	0.4332	0.4229	0.3852
E								0.1438	0.1107	0.1645	0.1413	0.0883
F								0.4549	0.403	0.4329	0.4542	0.3836
G								0.054	0.0889	0.0856	0.0716	0.0791
H								0.3612	0.2473	0.3589	0.3018	0.2992

Cell Index at: 128:48:18

	1	2	3	4	5	6	7	8	9	10	11	12
A								0.0749	0.0523	0.0836	0.0349	0.0555
B								0.0494	0.0579	0.0744	0.0948	0.08
C								0.3533	0.2997	0.4642	0.4945	0.3846
D								0.3169	0.3687	0.4388	0.4247	0.3838
E								0.1438	0.1118	0.1632	0.1419	0.0846
F								0.4576	0.4098	0.4357	0.4465	0.3842
G								0.0548	0.0873	0.0822	0.0691	0.0802
H								0.3624	0.2496	0.3658	0.3035	0.2998

Cell Index at: 129:03:19

	1	2	3	4	5	6	7	8	9	10	11	12
A								0.0744	0.0519	0.0863	0.0381	0.0584
B								0.0471	0.0558	0.0709	0.0982	0.0844
C								0.3542	0.2971	0.4647	0.4878	0.3877
D								0.3318	0.3768	0.443	0.4296	0.3936
E								0.1432	0.1167	0.1683	0.1423	0.089
F								0.4533	0.396	0.4344	0.4609	0.3917
G								0.0492	0.0915	0.0892	0.0697	0.0791
H								0.3629	0.2481	0.3599	0.296	0.3043

Cell Index at: 129:18:20

	1	2	3	4	5	6	7	8	9	10	11	12
A								0.0722	0.0494	0.0855	0.0301	0.0575
B								0.0492	0.0542	0.0698	0.096	0.0819
C								0.3505	0.3135	0.4727	0.4801	0.3915

D								0.3224	0.3812	0.4385	0.4258	0.392
E								0.1481	0.1121	0.1608	0.1365	0.0829
F								0.4521	0.4024	0.4309	0.4513	0.3829
G								0.0544	0.0891	0.0844	0.072	0.0807
H								0.3607	0.2469	0.3662	0.3016	0.3017

Cell Index at: 129:33:20

	1	2	3	4	5	6	7	8	9	10	11	12
A								0.0737	0.0552	0.0831	0.0393	0.0601
B								0.0524	0.0579	0.0706	0.0957	0.0859
C								0.3616	0.2995	0.4666	0.4961	0.3885
D								0.3244	0.378	0.4397	0.4337	0.3807
E								0.1497	0.1107	0.1664	0.1385	0.0826
F								0.4559	0.4079	0.4417	0.4556	0.389
G								0.0533	0.0866	0.0854	0.0701	0.0798
H								0.3651	0.2515	0.3659	0.3022	0.3068

Cell Index at: 129:48:21

	1	2	3	4	5	6	7	8	9	10	11	12
A								0.0721	0.0533	0.0818	0.0344	0.0615
B								0.0485	0.0592	0.0748	0.0958	0.0813
C								0.3547	0.3093	0.475	0.499	0.3865
D								0.331	0.3842	0.4394	0.4222	0.3823
E								0.137	0.1086	0.1657	0.135	0.0879
F								0.4538	0.413	0.4432	0.4572	0.3969
G								0.0531	0.0892	0.0848	0.0689	0.0796
H								0.3659	0.2491	0.3682	0.2997	0.3029

Cell Index at: 130:03:21

	1	2	3	4	5	6	7	8	9	10	11	12
A								0.0739	0.0538	0.0846	0.0352	0.0614
B								0.053	0.058	0.0754	0.0964	0.0842
C								0.3531	0.3059	0.4786	0.509	0.3932
D								0.3311	0.3897	0.4452	0.4308	0.3867
E								0.138	0.1132	0.1656	0.1369	0.0816
F								0.4563	0.4092	0.4393	0.4578	0.3857
G								0.0535	0.0925	0.0858	0.0672	0.0802
H								0.367	0.2532	0.3686	0.3032	0.3106

Cell Index at: 130:18:22

	1	2	3	4	5	6	7	8	9	10	11	12
A								0.0759	0.0558	0.0886	0.0368	0.0663
B								0.0542	0.0575	0.0764	0.0998	0.0835
C								0.3551	0.303	0.4736	0.5063	0.3974
D								0.3345	0.3835	0.4388	0.4324	0.3854
E								0.137	0.1146	0.1609	0.1369	0.0876
F								0.4566	0.4081	0.4405	0.4563	0.3879
G								0.0548	0.0914	0.087	0.0678	0.0776
H								0.371	0.2528	0.3669	0.3006	0.3096

Cell Index at: 130:33:22

	1	2	3	4	5	6	7	8	9	10	11	12
A								0.0764	0.0573	0.0894	0.035	0.0634
B								0.0508	0.0558	0.0766	0.0994	0.0873
C								0.3511	0.3046	0.4713	0.506	0.3978
D								0.3368	0.3855	0.4431	0.4368	0.3867
E								0.1444	0.1088	0.1693	0.1351	0.0901
F								0.4578	0.4088	0.4419	0.4589	0.3857
G								0.0576	0.0918	0.0843	0.0665	0.08
H								0.3695	0.2523	0.3676	0.3022	0.3105

Cell Index at: 130:48:22

	1	2	3	4	5	6	7	8	9	10	11	12
A								0.0765	0.0563	0.088	0.0357	0.0621
B								0.0562	0.0568	0.0752	0.0984	0.0825
C								0.357	0.3051	0.4749	0.5039	0.3903
D								0.3395	0.3818	0.4425	0.4302	0.3928
E								0.1401	0.1088	0.1652	0.137	0.0853
F								0.4548	0.4137	0.4399	0.4643	0.3888
G								0.0508	0.0876	0.0844	0.0676	0.0767
H								0.3685	0.2541	0.3672	0.3041	0.3145

Cell Index at: 131:03:23

	1	2	3	4	5	6	7	8	9	10	11	12
A								0.08	0.0608	0.0853	0.0372	0.0618
B								0.0543	0.0605	0.0762	0.0988	0.0841
C								0.3589	0.301	0.4673	0.5058	0.399
D								0.3371	0.3891	0.4437	0.4359	0.3902
E								0.1448	0.108	0.1681	0.137	0.0836
F								0.4543	0.419	0.4397	0.4616	0.3944
G								0.0562	0.0872	0.0869	0.0684	0.0804
H								0.3668	0.2591	0.3679	0.3064	0.3146

Cell Index at: 131:18:23

	1	2	3	4	5	6	7	8	9	10	11	12
A								0.0776	0.0607	0.088	0.0403	0.0616
B								0.0561	0.056	0.0734	0.0995	0.0876
C								0.3624	0.3033	0.4741	0.5068	0.4022
D								0.3299	0.3925	0.4449	0.4408	0.3922
E								0.1365	0.1093	0.1698	0.1393	0.0805
F								0.4557	0.4252	0.4483	0.4664	0.3993
G								0.0535	0.0895	0.0909	0.0729	0.0804
H								0.3705	0.2581	0.3654	0.3059	0.3132

Cell Index at: 131:33:23

	1	2	3	4	5	6	7	8	9	10	11	12
A								0.0726	0.062	0.0861	0.0385	0.0636
B								0.0602	0.0646	0.0742	0.0987	0.0828
C								0.3607	0.3067	0.4818	0.5111	0.3981
D								0.3344	0.3865	0.4459	0.4311	0.3851
E								0.1392	0.1132	0.1624	0.1498	0.0865
F								0.4582	0.4223	0.4469	0.4696	0.3961
G								0.0514	0.0917	0.0818	0.066	0.0809
H								0.3708	0.2612	0.3629	0.3076	0.317

Cell Index at: 131:48:23

	1	2	3	4	5	6	7	8	9	10	11	12
A								0.078	0.0615	0.0845	0.0393	0.0594
B								0.0594	0.06	0.08	0.1018	0.0852
C								0.3545	0.305	0.4847	0.509	0.3968
D								0.3381	0.3886	0.4392	0.4389	0.3841
E								0.1422	0.1112	0.1606	0.1437	0.0857
F								0.4554	0.4176	0.4523	0.4762	0.4007
G								0.0541	0.0951	0.0843	0.0687	0.0833
H								0.3735	0.264	0.3622	0.3094	0.3189

Cell Index at: 132:03:24

	1	2	3	4	5	6	7	8	9	10	11	12
A								0.0747	0.0651	0.0853	0.0378	0.0623
B								0.0583	0.0635	0.0782	0.0965	0.0849
C								0.3527	0.3055	0.484	0.5158	0.4057
D								0.3371	0.3956	0.4391	0.4387	0.3942
E								0.1491	0.1203	0.1718	0.1451	0.0796
F								0.4571	0.4154	0.454	0.4702	0.4012
G								0.053	0.0889	0.0808	0.068	0.0838
H								0.3729	0.2651	0.3635	0.3086	0.3203

Cell Index at: 132:18:24

	1	2	3	4	5	6	7	8	9	10	11	12
A								0.078	0.065	0.089	0.0356	0.0625
B								0.0601	0.0634	0.0793	0.0981	0.0849

	1	2	3	4	5	6	7	8	9	10	11	12
C								0.358	0.308	0.4896	0.511	0.4004
D								0.3308	0.3805	0.4439	0.4395	0.4042
E								0.153	0.1213	0.1666	0.1432	0.0895
F								0.4591	0.4216	0.4547	0.4757	0.3989
G								0.0485	0.0874	0.0833	0.0694	0.0827
H								0.3708	0.2631	0.3626	0.3128	0.3202

Cell Index at: 132:33:24

	1	2	3	4	5	6	7	8	9	10	11	12
A								0.0739	0.0644	0.09	0.034	0.0657
B								0.0648	0.0605	0.077	0.1004	0.0868
C								0.3625	0.3078	0.4936	0.5168	0.4069
D								0.3429	0.3818	0.4501	0.4472	0.395
E								0.1398	0.1167	0.1689	0.1518	0.0864
F								0.4641	0.4288	0.4478	0.4729	0.4032
G								0.0543	0.0963	0.0816	0.0719	0.0781
H								0.3747	0.2636	0.3615	0.3125	0.324

Cell Index at: 132:48:24

	1	2	3	4	5	6	7	8	9	10	11	12
A								0.0751	0.0652	0.0876	0.0394	0.0662
B								0.0639	0.057	0.0739	0.103	0.0855
C								0.3607	0.3079	0.4922	0.5039	0.4017
D								0.3451	0.3849	0.4475	0.4479	0.3905
E								0.1491	0.1117	0.1696	0.1457	0.0775
F								0.4624	0.4239	0.4515	0.4788	0.4023
G								0.053	0.0942	0.0819	0.068	0.0814
H								0.372	0.2657	0.3616	0.315	0.3244

Cell Index at: 133:03:25

	1	2	3	4	5	6	7	8	9	10	11	12
A								0.0772	0.064	0.0844	0.0409	0.0657
B								0.0608	0.0605	0.0793	0.0985	0.089
C								0.3575	0.3116	0.4887	0.5103	0.4031
D								0.3404	0.3853	0.4492	0.4455	0.392
E								0.1463	0.1102	0.172	0.1512	0.0873
F								0.46	0.4226	0.4535	0.4791	0.4019
G								0.0543	0.095	0.0876	0.0693	0.0812
H								0.3751	0.2673	0.3597	0.3123	0.3248

Cell Index at: 133:18:25

	1	2	3	4	5	6	7	8	9	10	11	12
A								0.0742	0.0645	0.0872	0.0404	0.0703
B								0.0613	0.0597	0.0713	0.1023	0.0866
C								0.3672	0.3182	0.4969	0.5144	0.4075
D								0.3498	0.3861	0.442	0.4566	0.3985
E								0.1535	0.112	0.1749	0.1474	0.0846
F								0.4673	0.4262	0.4565	0.4825	0.4036
G								0.0542	0.0913	0.0842	0.0675	0.078
H								0.3754	0.2653	0.358	0.3088	0.3269

Cell Index at: 133:33:26

	1	2	3	4	5	6	7	8	9	10	11	12
A								0.0742	0.0672	0.0893	0.0403	0.068
B								0.061	0.0559	0.0752	0.0966	0.0885
C								0.3639	0.3141	0.4943	0.5212	0.4036
D								0.341	0.3869	0.4402	0.4378	0.3985
E								0.1451	0.1155	0.1739	0.1494	0.0782
F								0.4578	0.4301	0.4579	0.4883	0.4013
G								0.0566	0.0929	0.0848	0.0667	0.082
H								0.3761	0.2682	0.3588	0.3097	0.3284

Cell Index at: 133:48:27

	1	2	3	4	5	6	7	8	9	10	11	12
A								0.0738	0.0659	0.0882	0.0401	0.0703
B								0.0654	0.0613	0.0739	0.0995	0.0925
C								0.3709	0.3093	0.491	0.5159	0.4068
D								0.3504	0.3826	0.4488	0.4534	0.4058
E								0.154	0.1215	0.1742	0.145	0.0887
F								0.4678	0.4273	0.4514	0.4866	0.4011
G								0.054	0.0968	0.0879	0.0661	0.0825
H								0.3763	0.2695	0.3602	0.3101	0.3294

Cell Index at: 134:03:27

	1	2	3	4	5	6	7	8	9	10	11	12
A								0.0794	0.0647	0.0925	0.0409	0.0729
B								0.0644	0.0599	0.08	0.0994	0.0878
C								0.3667	0.3141	0.4978	0.5142	0.4081
D								0.346	0.3915	0.4474	0.4476	0.4061
E								0.143	0.1127	0.1735	0.1403	0.0781
F								0.4681	0.4367	0.4619	0.485	0.4008
G								0.054	0.0962	0.0885	0.0645	0.0809
H								0.3805	0.2678	0.3617	0.3131	0.3267

Cell Index at: 134:18:27

	1	2	3	4	5	6	7	8	9	10	11	12
A								0.0825	0.063	0.0947	0.0432	0.071
B								0.0647	0.059	0.0799	0.1033	0.0863
C								0.3653	0.3153	0.5024	0.519	0.4107
D								0.3555	0.3908	0.46	0.4504	0.4059
E								0.1528	0.1159	0.1741	0.15	0.0797
F								0.4688	0.4265	0.4628	0.4887	0.3941
G								0.0496	0.0968	0.0889	0.0635	0.0828
H								0.3786	0.2739	0.3648	0.3125	0.3263

Cell Index at: 134:33:27

	1	2	3	4	5	6	7	8	9	10	11	12
A								0.0787	0.0619	0.0915	0.0374	0.0746
B								0.0623	0.0645	0.0781	0.0999	0.0872
C								0.3623	0.3174	0.503	0.5153	0.4126
D								0.3535	0.3871	0.4598	0.4537	0.4082
E								0.1585	0.1088	0.1767	0.1476	0.0815
F								0.4728	0.4366	0.4625	0.4843	0.3986
G								0.0539	0.096	0.0898	0.0629	0.0843
H								0.3754	0.2727	0.3669	0.3133	0.3255

Cell Index at: 134:48:27

	1	2	3	4	5	6	7	8	9	10	11	12
A								0.0809	0.0658	0.0949	0.0416	0.0738
B								0.0601	0.0649	0.0763	0.1039	0.0856
C								0.3805	0.309	0.5043	0.5205	0.4123
D								0.3556	0.3828	0.4502	0.4481	0.4093
E								0.1497	0.1075	0.1731	0.149	0.0906
F								0.4667	0.4333	0.4597	0.4855	0.4034
G								0.0547	0.0953	0.0926	0.0662	0.0845
H								0.3737	0.2716	0.3686	0.3127	0.3254

Cell Index at: 135:03:28

	1	2	3	4	5	6	7	8	9	10	11	12
A								0.084	0.0657	0.0913	0.0362	0.0725
B								0.0656	0.0631	0.0762	0.1022	0.0909
C								0.3706	0.3157	0.5021	0.5125	0.4059
D								0.3549	0.3914	0.458	0.4485	0.4116
E								0.1527	0.117	0.1649	0.1534	0.0897
F								0.4672	0.4351	0.4598	0.4938	0.4015
G								0.0552	0.0978	0.0893	0.0642	0.0853
H								0.378	0.2728	0.3716	0.3143	0.3225

Cell Index at: 135:18:28

	1	2	3	4	5	6	7	8	9	10	11	12
A								0.082	0.069	0.0955	0.0369	0.0722

B								0.0629	0.0636	0.082	0.1003	0.0934
C								0.3811	0.3211	0.4959	0.5175	0.4079
D								0.3572	0.3966	0.4563	0.4486	0.4053
E								0.152	0.1188	0.1688	0.15	0.0861
F								0.4746	0.434	0.464	0.4926	0.3977
G								0.0537	0.0972	0.0905	0.0717	0.0813
H								0.3782	0.269	0.3745	0.3104	0.3235

Cell Index at: 135:33:29

	1	2	3	4	5	6	7	8	9	10	11	12
A								0.079	0.0664	0.0986	0.0366	0.0733
B								0.0635	0.0636	0.0775	0.1035	0.0895
C								0.3743	0.32	0.4989	0.5211	0.4192
D								0.3495	0.3929	0.4605	0.4416	0.4171
E								0.1481	0.1169	0.164	0.1502	0.0799
F								0.4679	0.435	0.4662	0.4939	0.4022
G								0.0613	0.0956	0.0889	0.068	0.0814
H								0.3868	0.2714	0.3702	0.3163	0.3293

Cell Index at: 135:48:29

	1	2	3	4	5	6	7	8	9	10	11	12
A								0.0769	0.0712	0.0961	0.0395	0.0749
B								0.0682	0.0658	0.0791	0.0986	0.0863
C								0.3768	0.3113	0.4966	0.523	0.4132
D								0.361	0.3907	0.4635	0.4425	0.4045
E								0.1489	0.11	0.1726	0.1463	0.0782
F								0.4746	0.4357	0.4748	0.4972	0.4017
G								0.06	0.0977	0.0906	0.0669	0.084
H								0.3857	0.2747	0.3717	0.3172	0.3279

Cell Index at: 136:03:30

	1	2	3	4	5	6	7	8	9	10	11	12
A								0.0785	0.0726	0.0945	0.0424	0.0762
B								0.0699	0.0667	0.0771	0.1004	0.0949
C								0.3821	0.3197	0.4975	0.5253	0.4273
D								0.3568	0.3996	0.4619	0.4439	0.4113
E								0.1603	0.1099	0.1691	0.1481	0.0824
F								0.472	0.4388	0.4681	0.4951	0.4077
G								0.0646	0.0971	0.0927	0.0687	0.0778
H								0.3844	0.2723	0.372	0.3156	0.3333

Cell Index at: 136:18:30

	1	2	3	4	5	6	7	8	9	10	11	12
A								0.0826	0.0732	0.0969	0.0405	0.0764
B								0.0678	0.0646	0.0803	0.0991	0.086
C								0.3705	0.3211	0.4901	0.5276	0.4246
D								0.3661	0.3972	0.4586	0.4412	0.4067
E								0.1479	0.1176	0.1697	0.1525	0.0859
F								0.4698	0.4385	0.4739	0.4987	0.4044
G								0.0577	0.0979	0.0949	0.0703	0.0779
H								0.3851	0.2712	0.371	0.3194	0.3314

Cell Index at: 136:33:29

	1	2	3	4	5	6	7	8	9	10	11	12
A								0.0816	0.0757	0.0953	0.042	0.077
B								0.0682	0.069	0.084	0.1042	0.0878
C								0.3778	0.3227	0.496	0.53	0.4224
D								0.3575	0.4	0.4704	0.4413	0.401
E								0.1626	0.1127	0.1763	0.1482	0.0802
F								0.4827	0.4398	0.4708	0.4911	0.4048
G								0.0625	0.0996	0.0894	0.0632	0.0781
H								0.3858	0.2652	0.3728	0.3185	0.3282

Cell Index at: 136:48:30

	1	2	3	4	5	6	7	8	9	10	11	12
A								0.08	0.0789	0.095	0.0414	0.0729
B								0.0692	0.0639	0.0837	0.1045	0.086
C								0.3712	0.3184	0.4978	0.518	0.4197
D								0.3531	0.4006	0.4614	0.4497	0.4082
E								0.1554	0.1172	0.1684	0.1511	0.0863
F								0.4745	0.435	0.4666	0.4939	0.4093
G								0.0563	0.1012	0.0938	0.0681	0.0792
H								0.3842	0.273	0.3735	0.3185	0.3302

Cell Index at: 137:03:31

	1	2	3	4	5	6	7	8	9	10	11	12
A								0.0826	0.0776	0.0955	0.0427	0.0765
B								0.0727	0.0638	0.0843	0.1029	0.0897
C								0.373	0.321	0.4938	0.5223	0.4237
D								0.3565	0.3929	0.4705	0.4491	0.4089
E								0.1573	0.1189	0.1716	0.1578	0.0852
F								0.4758	0.4375	0.4771	0.4942	0.4109
G								0.0572	0.1013	0.0906	0.0707	0.0837
H								0.3877	0.2723	0.3757	0.3212	0.328

Cell Index at: 137:18:32

	1	2	3	4	5	6	7	8	9	10	11	12
A								0.0798	0.0785	0.0992	0.0433	0.0802
B								0.072	0.0665	0.0855	0.1036	0.0906
C								0.374	0.3175	0.5072	0.5237	0.4211
D								0.3629	0.3998	0.4753	0.4594	0.4123
E								0.1557	0.1202	0.1735	0.15	0.0815
F								0.4775	0.4334	0.4745	0.4875	0.417
G								0.0586	0.1021	0.0885	0.0707	0.083
H								0.3858	0.273	0.3768	0.3195	0.3311

Cell Index at: 137:33:32

	1	2	3	4	5	6	7	8	9	10	11	12
A								0.0804	0.0801	0.1008	0.0415	0.0784
B								0.0683	0.0659	0.0803	0.0998	0.0897
C								0.3786	0.3234	0.5049	0.5301	0.4203
D								0.3543	0.3991	0.4721	0.4511	0.4167
E								0.1579	0.1156	0.174	0.1503	0.084
F								0.4714	0.4394	0.4761	0.4956	0.415
G								0.0589	0.1065	0.0864	0.0723	0.0818
H								0.3848	0.2735	0.3762	0.3217	0.3318

Cell Index at: 137:48:33

	1	2	3	4	5	6	7	8	9	10	11	12
A								0.0833	0.0752	0.103	0.0399	0.0793
B								0.0753	0.0674	0.0816	0.0991	0.0863
C								0.3758	0.3248	0.5063	0.5269	0.4177
D								0.36	0.3968	0.4791	0.4607	0.418
E								0.1588	0.1194	0.1727	0.1577	0.08
F								0.4814	0.4412	0.4809	0.498	0.4183
G								0.056	0.1059	0.0895	0.0745	0.0821
H								0.3869	0.2771	0.3801	0.3177	0.3354

Cell Index at: 138:03:33

	1	2	3	4	5	6	7	8	9	10	11	12
A								0.0823	0.0764	0.0977	0.0405	0.0788
B								0.0735	0.0713	0.0817	0.102	0.0866
C								0.3779	0.3253	0.4914	0.5257	0.4216
D								0.3675	0.399	0.4758	0.4498	0.4212
E								0.153	0.1175	0.1653	0.1595	0.076
F								0.4766	0.4413	0.4801	0.493	0.416
G								0.0589	0.1062	0.0885	0.0693	0.0843
H								0.3865	0.2767	0.3777	0.3213	0.3321

Cell Index at: 138:18:34

	1	2	3	4	5	6	7	8	9	10	11	12

A								0.0819	0.0824	0.0985	0.0377	0.0823
B								0.0708	0.0679	0.085	0.1034	0.0881
C								0.3747	0.3212	0.5007	0.5331	0.4227
D								0.3726	0.4059	0.4748	0.4602	0.4141
E								0.1524	0.1142	0.1651	0.1551	0.0778
F								0.4832	0.4435	0.4764	0.4993	0.4132
G								0.0541	0.1093	0.0875	0.0715	0.0824
H								0.3884	0.2775	0.3777	0.3258	0.3364

Cell Index at: 138:33:35

	1	2	3	4	5	6	7	8	9	10	11	12
A								0.0847	0.078	0.1022	0.0398	0.086
B								0.0736	0.0705	0.0864	0.1011	0.0955
C								0.3799	0.3296	0.5015	0.5268	0.4218
D								0.3587	0.4101	0.4769	0.4525	0.4231
E								0.1544	0.1218	0.1753	0.1654	0.0848
F								0.4831	0.439	0.4842	0.4983	0.4108
G								0.0591	0.1094	0.0892	0.0689	0.0811
H								0.3868	0.2776	0.3812	0.3241	0.3365

Cell Index at: 138:48:35

	1	2	3	4	5	6	7	8	9	10	11	12
A								0.0859	0.078	0.0992	0.0402	0.0849
B								0.076	0.071	0.087	0.1	0.0919
C								0.3781	0.3248	0.4969	0.5299	0.4291
D								0.3749	0.4065	0.4745	0.4591	0.43
E								0.16	0.1184	0.1723	0.1584	0.0862
F								0.4817	0.44	0.4809	0.4923	0.4222
G								0.0592	0.1106	0.0851	0.0715	0.0835
H								0.3882	0.2816	0.3833	0.322	0.3358

Cell Index at: 139:03:36

	1	2	3	4	5	6	7	8	9	10	11	12
A								0.0899	0.0787	0.1006	0.0406	0.0872
B								0.0717	0.0661	0.0838	0.1037	0.0907
C								0.373	0.3232	0.5021	0.5308	0.4245
D								0.3768	0.4094	0.4797	0.4583	0.4279
E								0.1549	0.1277	0.1659	0.1573	0.0922
F								0.4807	0.441	0.4804	0.5023	0.4211
G								0.0559	0.1059	0.0832	0.078	0.0845
H								0.3894	0.2816	0.3855	0.3277	0.3358
