# Supplementary material for: Single-cell RNA sequencing and spatial transcriptomics reveal cancer-associated fibroblasts in glioblastoma with protumoral effects
Source: J Clin Invest. 2023 Mar 1;133(5):e147087. doi: 10.1172/JCI147087 (PMC9974099; doi:10.1172/JCI147087)
Supplement: Supplemental tables 5-8 [file jci-133-147087-s015.pdf]

ID	Age at Diagnosis	Gender	Days of last follow up after diagnosis	Deceased at last follow up (1=yes; 0=no)	Total cells	NPEE Cells	ACTA+ cells	% ACTA+	FAP	% FAP+	PDGFRA	% PDGFRA+	PDPN	% PDPN+	PDGFRB	%PDGFRB+	# of NPEE cells that express at least one CAF marker	% of total cells that are NPEE expressing at least one CAF marker
SF11956	63	M	457	1	3359	2748	0	0	0	0	132	3.92974099	401	11.9380768	115	3.42363799	907	27.06%
SF11977	61	F	1031	0	952	0	0	0	0	0	0	0	0	0	0	0	0	too few cells
SF11644	57	M	1270	0	1287	591	14	1.08780109	0	0	444	34.4988345	137	10.6449106	13	1.01010101	508	39.47%
SF11979	76	F	1030	0	669	529	3	0.44843049	2	0.29895366	20	2.98953662	34	5.08221226	7	1.04633782	54	10.16%
SF10022	65	M	496	1	432	331	2	0.46296296	1	0.23148148	16	3.7037037	16	3.7037037	5	1.15740741	30	9.03%
SF10127	44	F	560	1	835	766	4	0.47904192	3	0.35928144	37	4.43113772	36	4.31137725	13	1.55688623	92	11.02%
SF12090	61	M	947	4	55	51	1	1.81818182	1	1.81818182	2	3.63636364	6	10.9090909	0	0	0	too few cells
SF4400	51	F	1063	1	413	394	0	0	5	1.21065375	0	0	12	2.90556901	9	2.17917676	18	4.27%
SF4297	39	M	7277	0	2498	1504	15	0.60048038	40	1.60128102	94	3.76301041	92	3.68294636	68	2.72217774	174	6.98%
SF6996	40	F	167	1	402	350	4	0.99502488	3	0.74626866	8	1.99004975	8	1.99004975	3	0.74626866	8	1.87%
SF9259	73	M	470	1	673	505	0	0	8	1.18870728	36	5.34918276	10	1.4858841	2	0.29717682	3	0.41%

Supplemental Table 5. Survival of individual GBM patients did not correlate with CAF levels. Shown are survival from time of diagnsosis, age at diagnosis, gender, and % of cells positive for CAF markers but negative for markers of other stromal cells in 11 IDH wildtype glioblastomas. Multivariate analysis revealed that in this cohort, neither age (P=0.9), gender (P=0.9), nor CAF levels (P=0.4) impacted survival. Abbreviations used: NPEE=negative for pericyte, epithelial, endothelial, and immune cell markers.

	Age	Gender
GBMpt1CAFs	58	M
GBMpt2CAFs	62	F
GBMpt3CAFs	61	M
GBMpt4CAFs	57	M
GBMpt5CAFs	59	F

Supplemental Table 6. Information on patients from which GBMptCAFs were obtained. Shown are age and gender of newly diagnosed GBM patients from whom GBMptCAFs were derived.

Supplemental Table 7: Primers used in this manuscript for qPCR

Name	Species	Experimental Role	Forward	Reverse
FN	Human	Measure total fibronectin	CCACCCCCATAAGGCATAGG	GTAGGGGTCAAAGCACGAGTCATC
EDA-FN	Human	Measure EDA fibronectin	CCCAAGCTTAACATTGATCGCCCTAAAGGA	CCCGGTACCTGTGGACT GGGTTCCAATCAGG
Arg1	Human	M2 macrophage gene expression	CAGAAGAATGGAAGAGTCAG	CAGATATGCAGGGAGTCACC
iNOS	Human	M1 macrophage gene expression	TGCATGGACCAGTATAAGGCAAGC	GCTTCTGGTCGATGTCATGAGCAA
MMP9	Human	M2 macrophage gene expression	GATGCGTGGAGAGTCGAAAT	CACCAAACTGGATGACGATG
TGFB1	Human	M2 macrophage gene expression	CCCAGCATCTGCAAAGCTC	GTCAATGTACAGCTGCCGCA
CXCL10	Human	M1 macrophage gene expression	AGAACGGTGCGCTGCAC	CCTATGGCCCTGGGTCTA
Il1b	Human	M1 macrophage gene expression	CCACAGACCTTCCAGGAGAATG	GTGCAGTTCAGTGATCGTACAGG
GAPDH	Human	Housekeeping gene	CATGACAACTTTGGTATCGTGG	CCTGCTTCACCACCTTCTTG
ACTB	Human	Housekeeping gene	GAG CAC AGA GCC TCG CCT TT	ACA TGC CGG AGC CGT TGT C
NANOG	Human	Assess GSC gene expression	AGT CCC AAA GGC AAA CAA CCC ACT TC	TGC TGG AGG CTG AGG TAT TTC TGT CTC
Oct	Human	Assess GSC gene expression	GAC AGG GGG AGG GGA GGA GCT AGG	CTT CCC TCC AAC CAG TTG CCC CAA AC
SOX2	Human	Assess GSC gene expression	GGG AAA TGG GAG GGG TGC AAA AGA GG	TTG CGT GAG TGT GGA TGG GAT TGG TG
Nestin	Human	Assess GSC gene expression	GCCCTGACCACTCCAGTTTA	GGAGTCCTGGATTTCCTTCC
CD44	Human	Assess GSC gene expression	TGCCGCTTTGCAGGTGTATT	CCGATGCTCAGAGCTTTCTCC
LIF	Human	Assess mesenchymal gene expression	TGA ACC AGA TCA GGA GCC AA	AAG GTA CAC GAC TAT GCG GT
CHI3L1	Human	Assess mesenchymal gene expression	CAG CAG CTA TGA CAT TGC CA	ATG CCC ATC ACC AGC TTA CT
COL4A2	Human	Assess mesenchymal gene expression	TGT GGG CAT GAA AGG TCT CT	AAA ATC CAG CCT CGC CTT TG
FOSL2	Human	Assess mesenchymal gene expression	AAG ACC TGG CGT GAT CAA GA	GCT CAG CAA TCT CCT TCT GC
TIMP1	Human	Assess mesenchymal gene expression	TAC TTC CAC AGG TCC CAC AA	GCA GGG GAT GGA TAA ACA GG
SPOCD1	Human	Assess mesenchymal gene expression	ATG GAG TGA AGC TTG TGT GC	TGG AAA ACC TGG CAC CCA

Supplemental Table 8: Antibodies used in this manuscript

Antigen	Species Source	Vendor	Catalog/Clone Number	Dilution Used	Fluorochrome Conjugation	Application	Notes
Mouse/Human CD11b	Rat	BioLegend	101212	.25 ug/test	APC	FACS/flow	
Mouse MHC Class II	Rat	eBioscience	M5/114.15.2	.02 ug/test	PE	FACS/flow	
Mouse CD206	Rat	BioLegend	C068C2	.125 ug/test	FITC	FACS/flow	
Human CD140a/PDGFR-a	Mouse	Invitrogen	16A1	5 ul/test	APC	FACS/flow	
Human CD140b/PDGFR-b	Mouse	BioLegend	18A2	5 ul/test	PE	FACS/flow	
Human a-SMA	Mouse	R&D Systems	IC1420A/1A4	10 ul/test	APC	FACS/flow	
Human PDGF	Rabbit	R&D Systems	AB20NA	5 µg/mL	None	Blocking	10% cross-reactivity with PDGF-AA, 60% cross-reactivity with PDGF-AB, and nearly 100% cross-reactivity with PDGF-BB
Human TGF-b	Mouse	R&D Systems	1D11	1 ug/mL	None	Blocking	
Human TLR4	Goat	R&D Systems	AF1478	5 ug/mL	None	Blocking	
Human Osteopontin	Goat	R&D Systems	AF1433	4 ug/1ml	None	Blocking	
Human HGF	Mouse	R&D Systems	24612	1 ug/1mL	None	Blocking	
Human Her2	Humanized	Genentech	Trastuzumab	5 ug/mL	None	Blocking	
Human and Mouse Fibronectin	Rabbit	Abcam	Ab2413	1:100 or 1:200	None	IF	
Human Nestin	Mouse	Abcam	Ab22035	1:100 or 1:200	None	IF	
Human and Mouse EDA	Mouse	Abcam	Ab6328	1:200	None	IF	
Human and Mouse CD31	Rabbit	Abcam	Ab28364	1:20 or 1:50	None	IF	
Human PDGFR-B	Mouse	Santa Cruz Biotech	18A2	1:50 or 1:500	None	IF	
Human PDGFR-a	Rabbit	Abcam	Ab203491	1:500	None	IF	
